# Supplementary material for: Elucidation of Japanese pepper (Zanthoxylum piperitum De Candolle) domestication using RAD-Seq
Source: Sci Rep. 2021 Mar 19;11:6464. doi: 10.1038/s41598-021-85909-9 (PMC7979906; doi:10.1038/s41598-021-85909-9)
Supplement: Supplementary file 1 — Supplementary Information [file 41598_2021_85909_MOESM1_ESM.pdf]

Elucidation of Japanese pepper (*Zanthoxylum piperitum* De Candolle) domestication using RAD-Seq

Maddumage Dona Ginushika Priyadarshani Premarathne[1,2], Nami Fukutome[3, #], Kazuaki Yamasaki[4], Fumiyo Hayakawa[5], Atsushi J. Nagano[6], Hisataka Mizuno[7], Nobuo Ibaragi[8], Yukio Nagano[1, 2, #, *]

[1] Analytical Research Center for Experimental Sciences, Saga University

[2] Graduate School of Advanced Health Science, Saga University

[3] Department of Food Sciences, Tokyo Seiei College

[4] Faculty of Health Science, Hyogo University

[5] Food Research Institute, National Agriculture and Food Research Organization

[6] Faculty of Agriculture, Ryukoku University

[7] Gifu Agriculture and Forestry Office

[8] Association for Conservation of Asakura Sanshō

[#]These authors contributed equally to this work

*Correspondence to Y. N. (nagano@cc.saga-u.ac.jp)

Supplementary Table S1. Number of reads for each sample.

Plant Number	Number of reads
1	492,671
2	1,020,522
3	2,415,554
4	2,355,924
5	3,693,650
6	4,164,827
7	3,422,496
8	2,772,637
9	3,171,379
10	2,440,405
11	1,943,956
12	1,807,098
13	2,128,142
14	2,635,067
15	2,802,228
16	3,229,276
17	2,564,887
18	3,563,144
19	3,372,967
20	2,710,040
21	3,862,417
22	3,422,944
23	3,453,792
24	3,312,854
25	2,829,563
26	3,107,223
27	1,449,052
28	2,211,696
29	4,053,452
30	3,324,127
31	3,878,846
32	1,599,156
33	4,035,473
34	2,114,298
35	3,053,713
36	2,889,502
37	1,724,458
38	3,824,334
39	1,555,847
40	3,627,469
41	2,364,229
42	1,962,361
43	1,161,984
44	2,956,102
45	3,184,775
46	3,517,124
47	2,917,710
48	1,960,905
49	2,128,278
50	1,500,949

51	1,827,290
52	2,368,973
53	1,858,291
54	3,405,094
55	3,524,157
56	2,544,278
57	2,611,042
58	2,726,379
59	3,071,209
60	4,002,421
61	3,209,430
62	2,592,742
63	3,827,753
64	3,010,996
65	3,226,056
66	4,378,973
67	2,407,660
68	2,256,885
69	2,081,604
70	1,104,256
71	3,356,050
72	2,439,638
73	2,376,232
74	2,534,822
75	1,135,946
76	1,304,899
77	2,427,326
78	2,043,761
79	2,032,929
80	1,001,683
81	2,411,373
82	831,665
83	1,568,828
84	703,535
85	1,855,774
86	530,151
87	1,336,823
88	1,837,433
89	2,661,439
90	1,815,876
91	1,268,454
92	3,136,579
93	2,870,434
Total	235,208,642
Average reads	2,529,125

Supplementary Table S2. Depths of coverage for processed samples.

Plant Number	Depth of coverage
1	24.34x
2	24.73x
3	25.88x
4	26.25x
5	28.62x
6	28.64x
7	29.41x
8	26.09x
9	28.60x
10	26.44x
11	27.96x
12	26.76x
13	28.99x
14	28.71x
15	28.45x
16	28.58x
17	28.09x
18	29.55x
19	30.17x
20	27.86x
21	30.28x
22	28.57x
23	29.14x
24	29.35x
25	28.79x
26	28.90x
27	28.31x
28	28.51x
29	28.03x
30	27.77x
31	28.05x
32	27.73x
33	29.46x
34	28.60x
35	26.25x
36	27.75x
37	27.40x
38	28.30x
39	23.34x
40	28.92x
41	28.94x
42	28.17x
43	22.95x
44	26.68x
45	28.05x
46	29.57x
47	29.69x
48	27.16x
49	26.13x
50	27.08x
51	24.57x
52	26.14x
53	25.39x

54	27.14x
55	26.86x
56	24.35x
57	24.24x
58	29.66x
59	28.69x
60	27.61x
61	26.99x
62	26.76x
63	28.86x
64	27.45x
65	28.11x
66	30.15x
67	28.28x
68	28.11x
69	28.80x
70	26.70x
71	29.54x
72	26.98x
73	29.29x
74	23.22x
75	20.60x
76	26.06x
77	27.74x
78	20.97x
79	24.68x
80	28.15x
81	29.73x
82	27.13x
83	26.95x
84	25.55x
85	22.23x
86	24.57x
87	27.43x
88	27.23x
89	29.49x
90	25.79x
91	27.84x
92	24.64x
93	27.77x
Average	27.32x

Supplementary Table S3. The degrees of conservation of heterozygosity between each pair of the plants 35, 38, 40, 42, 43, 44, 45, 46, 47, 48, 49, 50, and 51.

	42	43	44	45	46	47	48	49	50	51
35	35.8	29.5	30.9	32.3	32.0	34.4	32.3	31.6	33.0	32.3
38	35.6	30.5	32.5	32.1	34.1	36.5	32.9	33.0	34.5	33.5
40	34.7	29.5	33.1	31.9	34.3	33.9	33.4	33.2	34.7	34.9
42		50.4	50.9	50.4	52.6	55.9	50.6	52.9	55.0	53.0
43			52.9	51.0	51.9	56.9	52.6	54.9	61.9	59.3
44				55.5	59.5	62.9	57.1	56.2	60.4	57.8
45					62.1	63.9	60.9	59.0	59.2	55.5
46						68.9	62.3	62.3	58.8	58.6
47							66.4	66.1	66.7	62.9
48								60.1	61.7	58.9
49									63.9	61.5
50										67.6

Supplementary Table S4. Summary statistics for each group for all positions (variant and fixed).

Population	Sites	Variant Sites	Poly Sites	%Poly Loci	Obs Het	Obs Homo	Exp Hete	Exp Homo	Nucleotide diversity	Inbreeding coefficient (Fis)
H	242383	2287	751	0.310	0.00265	0.99735	0.00147	0.99853	0.00256	-0.000130
B	402530	4085	1378	0.342	0.00312	0.99688	0.00165	0.99835	0.00258	-0.000810
F	463469	4967	3598	0.776	0.00322	0.99678	0.00289	0.99711	0.00323	0.000200
E	464236	4973	4095	0.882	0.00319	0.99681	0.00303	0.99697	0.00322	0.000540
C	460079	4923	2973	0.646	0.00320	0.99680	0.00253	0.99747	0.00298	-0.000330
A1	463672	4964	3433	0.740	0.00347	0.99653	0.00277	0.99723	0.00312	-0.000540
A2	450414	4802	2142	0.476	0.00362	0.99638	0.00209	0.99791	0.00235	-0.00232
D	464287	4973	4593	0.989	0.00324	0.99676	0.00321	0.99679	0.00331	0.000840

Poly – Polymorphic, Obs Hete – Observed Heterozygosity, Obs Homo – Observed Homozygosity, Exp Hete – Expected Heterozygosity, Exp Homo – Expected Homozygosity.

Supplementary Table S5. F3-statistics. The table was sorted by the values of the f3-statistic.

Outgroup; X, Y	F3-statistic	Standard error	Z-score
A1;C,A2	-0.00942	0.00118	-7.97
A1;E,A2	-0.00931	0.00133	-6.99
A1;A2,D	-0.00877	0.00133	-6.61
A1;H,A2	-0.00782	0.00184	-4.25
A1;B,A2	-0.00738	0.00169	-4.37
A1;F,A2	-0.00682	0.00134	-5.09
D;B,E	0.00452	0.00121	3.75
D;C,A2	0.00467	0.00120	3.88
D;C,A1	0.00532	0.00140	3.79
D;F,E	0.00552	0.000834	6.62
D;E,A2	0.00553	0.00106	5.20
F;C,A2	0.00572	0.00127	4.50
D;B,C	0.00576	0.00182	3.16
D;F,A1	0.00595	0.00152	3.92
D;E,A1	0.00606	0.00119	5.10
D;B,A1	0.00647	0.000929	6.97
D;B,F	0.00654	0.00108	6.08
D;F,C	0.00683	0.00105	6.54
D;E,C	0.00717	0.00112	6.40
F;H,D	0.00728	0.000853	8.53
D;B,A2	0.00786	0.00128	6.14
F;A2,D	0.00788	0.000818	9.63
F;E,A2	0.00790	0.000926	8.53
D;F,A2	0.00790	0.00109	7.27
F;B,C	0.00817	0.00200	4.08
F;B,E	0.00825	0.00186	4.43
F;C,A1	0.00832	0.000860	9.68
D;H,A1	0.00838	0.00213	3.93
D;H,E	0.00848	0.00113	7.52
D;H,F	0.00850	0.000411	20.7
F;H,A2	0.00871	0.00145	6.00
D;H,C	0.00881	0.000746	11.8
F;C,D	0.00895	0.000650	13.8
E;H,D	0.00903	0.00104	8.68
F;B,A2	0.00921	0.00163	5.66
F;B,D	0.00924	0.00152	6.09
F;H,C	0.00926	0.000822	11.3
D;H,A2	0.00933	0.00157	5.95
E;C,A2	0.00949	0.00105	9.00
E;C,A1	0.00960	0.00139	6.89
F;H,A1	0.00971	0.00152	6.40
D;H,B	0.00977	0.00208	4.70
F;B,A1	0.00977	0.00158	6.17
F;A1,D	0.00984	0.00106	9.31
F;H,E	0.0102	0.00107	9.59
F;E,D	0.0103	0.00137	7.50
E;C,D	0.0103	0.00105	9.83
F;E,A1	0.0104	0.00106	9.80
F;H,B	0.0105	0.00295	3.56
F;E,C	0.0106	0.000824	12.9

E;H,C	0.0107	0.00180	5.95
E;H,A1	0.0113	0.00171	6.63
E;A1,D	0.0115	0.000938	12.2
E;B,C	0.0116	0.00193	6.00
E;F,C	0.0117	0.00178	6.57
E;F,A1	0.0119	0.00133	8.93
E;A2,D	0.0120	0.000528	22.7
E;F,D	0.0120	0.000434	27.6
E;H,F	0.0120	0.00141	8.53
E;H,A2	0.0128	0.00136	9.44
E;B,D	0.0130	0.00120	10.8
E;B,A1	0.0134	0.00129	10.4
E;B,F	0.0140	0.000979	14.3
E;H,B	0.0143	0.00293	4.86
E;F,A2	0.0144	0.00120	12.0
E;B,A2	0.0153	0.00141	10.9
A1;H,D	0.0177	0.00184	9.64
H;B,E	0.0178	0.00297	6.01
H;C,A2	0.0181	0.00201	8.99
A1;B,E	0.0181	0.00139	13.0
H;B,C	0.0187	0.00345	5.42
H;E,A2	0.0193	0.00348	5.53
A1;F,E	0.0196	0.00111	17.6
A1;B,D	0.0196	0.00190	10.4
H;C,A1	0.0197	0.00294	6.68
H;B,F	0.0198	0.00380	5.21
H;B,A1	0.0199	0.00350	5.68
A1;E,D	0.0200	0.00139	14.4
A1;B,C	0.0201	0.00245	8.19
H;F,E	0.0201	0.00264	7.61
A1;H,E	0.0201	0.00148	13.6
A1;F,D	0.0201	0.00141	14.2
A1;B,F	0.0202	0.00173	11.7
A1;H,F	0.0203	0.00245	8.28
H;B,A2	0.0203	0.00316	6.44
H;F,A1	0.0206	0.00337	6.11
H;E,A1	0.0207	0.00360	5.77
A1;C,D	0.0208	0.00130	16.0
A1;H,B	0.0210	0.00351	5.99
H;F,C	0.0211	0.00202	10.4
A1;H,C	0.0212	0.00227	9.34
H;E,C	0.0214	0.00224	9.57
H;F,A2	0.0216	0.00298	7.26
A1;F,C	0.0217	0.00146	14.8
H;B,D	0.0218	0.00333	6.53
A1;E,C	0.0219	0.00112	19.6
H;A2,D	0.0222	0.00337	6.58
H;C,D	0.0227	0.00241	9.44
H;F,D	0.0230	0.00265	8.69
H;E,D	0.0231	0.00240	9.62
H;A1,D	0.0232	0.00415	5.58
C;H,D	0.0232	0.00202	11.5
C;F,E	0.0236	0.00160	14.7
C;B,E	0.0236	0.000759	31.2

C;H,E	0.0246	0.00143	17.2
C;E,D	0.0249	0.00136	18.3
C;H,F	0.0249	0.00158	15.8
C;F,D	0.0252	0.00172	14.7
C;E,A1	0.0256	0.00154	16.6
C;E,A2	0.0257	0.00211	12.2
C;F,A1	0.0259	0.00210	12.3
C;B,F	0.0260	0.00226	11.5
C;B,D	0.0263	0.00118	22.2
C;H,A1	0.0263	0.00220	11.9
C;A1,D	0.0267	0.00184	14.5
C;H,B	0.0272	0.00276	9.89
C;A2,D	0.0274	0.00171	16.1
C;B,A1	0.0275	0.00124	22.2
C;H,A2	0.0279	0.00277	10.1
C;F,A2	0.0285	0.00210	13.5
C;B,A2	0.0295	0.00213	13.9
A2;F,A1	0.0325	0.00145	22.4
A2;B,A1	0.0331	0.00151	21.8
A2;H,A1	0.0335	0.00144	23.2
A2;A1,D	0.0345	0.00139	24.8
D;A1,A2	0.0349	0.00193	18.1
A2;E,A1	0.0350	0.00153	22.9
A2;C,A1	0.0351	0.00113	31.1
B;H,D	0.0364	0.00273	13.3
F;A1,A2	0.0368	0.000855	43.1
B;C,A2	0.0372	0.00242	15.3
B;H,A2	0.0378	0.00365	10.4
B;H,A1	0.0383	0.00371	10.3
B;A2,D	0.0383	0.00247	15.5
B;H,F	0.0383	0.00301	12.7
B;F,A1	0.0391	0.00245	15.9
B;C,A1	0.0392	0.00174	22.6
B;E,A2	0.0393	0.00310	12.7
B;H,C	0.0394	0.00322	12.2
B;F,D	0.0396	0.00223	17.8
B;F,A2	0.0396	0.00362	10.9
B;A1,D	0.0397	0.00120	33.2
B;H,E	0.0403	0.00290	13.9
B;C,D	0.0404	0.00190	21.2
B;F,E	0.0406	0.00236	17.2
B;F,C	0.0407	0.00270	15.1
E;A1,A2	0.0408	0.00139	29.4
B;E,A1	0.0412	0.00174	23.7
B;E,D	0.0416	0.00201	20.7
B;E,C	0.0430	0.00219	19.7
H;A1,A2	0.0487	0.00423	11.5
C;A1,A2	0.0569	0.00284	20.0
A2;H,D	0.0600	0.00166	36.1
A2;B,F	0.0601	0.00239	25.1
A2;B,E	0.0605	0.00295	20.5
A2;H,F	0.0606	0.00112	54.2
A2;F,E	0.0614	0.00219	27.9
A2;F,D	0.0614	0.00216	28.4

A2;B,D	0.0615	0.00270	22.8
A2;H,B	0.0619	0.00221	27.9
A2;B,C	0.0626	0.00389	16.1
A2;H,E	0.0630	0.00144	43.8
A2;F,C	0.0636	0.00203	31.4
A2;E,D	0.0638	0.00226	28.2
A2;H,C	0.0641	0.00192	33.4
A2;C,D	0.0647	0.00225	28.7
A2;E,C	0.0663	0.00241	27.5
B;A1,A2	0.0667	0.00288	23.1

Supplementary Table S6. F4-statistics. The table was sorted by the values of the Z-score.

Outgroup, X; Y, Z	F4-statistic	Standard error	Z-score
F,A2;A1,D	-0.0289	0.000912	-31.7
F,A1;A2,D	-0.0270	0.00108	-25.0
C,A2;A1,D	-0.0296	0.00128	-23.2
E,A2;A1,D	-0.0288	0.00125	-23.0
B,A2;A1,D	-0.0284	0.00145	-19.6
E,A1;A2,D	-0.0293	0.00153	-19.2
C,A1;A2,D	-0.0302	0.00164	-18.4
H,A2;A1,D	-0.0265	0.00165	-16.1
H,A1;A2,D	-0.0255	0.00161	-15.9
B,A1;A2,D	-0.0270	0.00244	-11.1
F,C;E,A2	-0.00488	0.000675	-7.24
F,E;A1,A2	-0.00249	0.000408	-6.09
B,C;E,A1	-0.00380	0.00100	-3.81
F,D;A1,A2	-0.00195	0.000557	-3.51
F,C;A1,A2	-0.00260	0.000781	-3.32
B,C;E,A2	-0.00584	0.00182	-3.21
F,C;E,A1	-0.00228	0.000718	-3.18
F,A1;E,C	-0.00206	0.000756	-2.73
B,D;E,A2	-0.00334	0.00129	-2.59
B,C;E,D	-0.00265	0.00104	-2.55
F,C;E,D	-0.00165	0.000656	-2.52
H,A2;F,C	-0.00354	0.00148	-2.39
B,C;F,A2	-0.00348	0.00146	-2.38
H,C;E,A2	-0.00334	0.00148	-2.25
F,E;C,A2	-0.00271	0.00123	-2.20
F,D;E,A2	-0.00238	0.00110	-2.17
H,C;F,A2	-0.00299	0.00146	-2.04
B,C;A1,A2	-0.00203	0.00107	-1.90
F,D;C,A2	-0.00107	0.00062	-1.73
H,A2;F,E	-0.00235	0.00137	-1.72
B,D;E,A1	-0.00195	0.00120	-1.62
B,A1;E,C	-0.00198	0.00122	-1.62
H,C;E,A1	-0.00175	0.00115	-1.52
F,A2;E,C	-0.00217	0.00144	-1.51
B,E;C,A2	-0.00375	0.00290	-1.29
H,B;F,E	-0.00200	0.00161	-1.24
H,A2;B,C	-0.00224	0.00191	-1.18
B,A1;E,D	-0.00154	0.00133	-1.16
H,C;A1,A2	-0.00160	0.00141	-1.13
H,A1;E,C	-0.00108	0.000978	-1.10
B,E;A1,A2	-0.00192	0.00178	-1.08
B,A2;E,C	-0.00209	0.00196	-1.07
F,D;E,C	-0.00131	0.00124	-1.06
B,E;C,A1	-0.00182	0.00176	-1.03
B,F;E,A1	-0.00152	0.00147	-1.03
B,D;F,A2	-0.00132	0.00129	-1.03
B,C;F,A1	-0.00145	0.00144	-1.01
B,D;C,A2	-0.00210	0.00222	-0.950
B,D;E,C	-0.00123	0.00131	-0.945
B,F;E,D	-0.000993	0.00108	-0.923

B,A2;F,D	-0.00136	0.00149	-0.911
H,E;A1,A2	-0.00149	0.00169	-0.878
B,A2;F,C	-0.00245	0.00284	-0.862
H,E;C,A2	-0.00215	0.00253	-0.851
H,C;F,A1	-0.00139	0.00168	-0.825
H,A1;F,C	-0.000941	0.00116	-0.809
B,E;F,A2	-0.00131	0.00164	-0.801
B,F;C,A1	-0.00160	0.00208	-0.769
B,E;C,D	-0.00141	0.00188	-0.750
B,D;A1,A2	-0.00139	0.00187	-0.745
B,A2;E,D	-0.00101	0.00136	-0.742
B,F;C,D	-0.00107	0.00155	-0.692
H,A2;E,C	-0.00119	0.00183	-0.649
F,A1;E,D	-0.000548	0.000892	-0.615
H,D;E,A2	-0.000845	0.00148	-0.572
H,D;A1,A2	-0.000952	0.00194	-0.491
H,B;F,C	-0.00109	0.00227	-0.480
H,D;F,C	-0.000311	0.000657	-0.474
B,F;E,A2	-0.000957	0.00210	-0.457
B,D;C,A1	-0.000715	0.00157	-0.455
H,D;F,A2	-0.000825	0.00187	-0.441
B,F;C,A2	-0.00104	0.00244	-0.425
H,A2;B,E	-0.00105	0.00251	-0.417
H,E;F,A2	-0.000811	0.00208	-0.390
H,D;E,C	-0.000331	0.000912	-0.363
H,E;C,A1	-0.000668	0.00187	-0.357
H,D;C,A2	-0.000514	0.00178	-0.289
F,D;E,A1	-0.000429	0.00152	-0.282
H,F;C,A1	-0.000447	0.00175	-0.256
F,E;C,D	-0.000339	0.00148	-0.230
H,C;B,A2	-0.000648	0.00322	-0.201
B,A2;F,E	-0.000352	0.00178	-0.198
B,C;F,D	-0.000292	0.00159	-0.184
F,E;C,A1	-0.000221	0.00122	-0.181
F,E;A1,D	-0.000119	0.00105	-0.114
E,C;A1,A2	-0.000110	0.00104	-0.106
H,A1;B,C	-0.000205	0.00237	-0.0864
F,A2;E,D	-0.0000137	0.000565	-0.0243
B,F;A2,D	-0.0000360	0.00191	-0.0189
H,D;F,E	0.0000199	0.00121	0.0164
H,B;F,A1	0.0000609	0.00218	0.0279
H,D;E,A1	0.000107	0.00224	0.0478
B,D;F,A1	0.0000663	0.00129	0.0514
H,D;F,A1	0.000127	0.00216	0.0588
B,A1;F,C	0.000151	0.00250	0.0604
B,F;E,C	0.0000805	0.00119	0.0677
H,F;B,E	0.000260	0.00323	0.0805
H,A1;F,E	0.000139	0.00146	0.0950
H,D;B,A2	0.000438	0.00286	0.153
H,D;C,A1	0.000438	0.00210	0.208
H,F;B,A1	0.000797	0.00369	0.216
H,B;F,A2	0.000498	0.00210	0.238
H,C;B,A1	0.000948	0.00362	0.262
B,A1;C,D	0.000442	0.00160	0.276

H,F;C,A2	0.000553	0.00179	0.309
H,A1;B,F	0.000736	0.00229	0.321
H,A1;B,E	0.000875	0.00260	0.336
B,A1;F,D	0.000593	0.00173	0.343
H,D;B,A1	0.00139	0.00392	0.355
B,F;A1,A2	0.000563	0.00154	0.367
H,D;B,C	0.000952	0.00256	0.372
B,D;F,C	0.000781	0.00206	0.379
H,C;F,E	0.000359	0.000930	0.387
H,F;B,C	0.00124	0.00316	0.394
H,E;F,A1	0.000675	0.00169	0.400
H,B;C,A1	0.00115	0.00282	0.408
B,E;A1,D	0.000408	0.00100	0.409
H,E;B,A2	0.00145	0.00320	0.452
H,D;B,E	0.00128	0.00274	0.469
H,A2;F,D	0.000605	0.00124	0.486
H,B;A1,A2	0.000437	0.00087	0.504
H,F;B,A2	0.00180	0.00342	0.526
H,F;E,A1	0.000537	0.00102	0.528
H,B;C,A2	0.00159	0.00301	0.528
H,D;B,F	0.00126	0.00232	0.544
B,E;F,A1	0.000615	0.00106	0.578
B,A2;C,D	0.00109	0.00177	0.615
H,A2;B,F	0.00130	0.00207	0.628
H,F;A1,A2	0.00100	0.00158	0.633
E,C;A2,D	0.000859	0.00136	0.633
B,F;A1,D	0.000527	0.000780	0.676
C,D;A1,A2	0.000644	0.000921	0.700
H,E;B,A1	0.00293	0.00366	0.801
H,B;E,C	0.000904	0.00112	0.807
E,D;A1,A2	0.000535	0.000602	0.888
F,C;A1,D	0.000630	0.000690	0.913
E,C;A1,D	0.000749	0.000809	0.926
H,C;B,E	0.00270	0.00289	0.933
H,F;E,C	0.000984	0.00105	0.935
H,E;B,F	0.00226	0.00236	0.956
H,F;A2,D	0.00143	0.00144	0.992
H,C;B,F	0.00234	0.00234	1.00
H,A2;B,D	0.00190	0.00191	1.00
H,F;E,A2	0.00154	0.00147	1.05
H,B;E,A1	0.00206	0.00196	1.05
F,D;C,A1	0.000886	0.000842	1.05
E,D;C,A1	0.00111	0.00104	1.06
B,E;F,C	0.00244	0.00228	1.07
H,B;A2,D	0.00147	0.00133	1.10
H,F;B,D	0.00323	0.00282	1.14
B,E;F,D	0.00102	0.000890	1.15
B,A1;F,E	0.00213	0.00184	1.16
E,A1;C,D	0.00186	0.00159	1.17
B,C;F,E	0.00236	0.00201	1.17
H,A1;B,D	0.00329	0.00277	1.19
H,B;E,A2	0.00249	0.00208	1.20
B,E;A2,D	0.00233	0.00185	1.26
H,E;F,C	0.00134	0.00103	1.30

H,E;C,D	0.00164	0.00123	1.33
H,C;E,D	0.00131	0.000979	1.34
H,C;B,D	0.00401	0.00290	1.38
H,E;B,C	0.00360	0.00259	1.39
H,B;C,D	0.00306	0.00212	1.44
H,B;A1,D	0.00190	0.00131	1.45
H,E;A1,D	0.00231	0.00157	1.47
B,C;A1,D	0.00116	0.00077	1.51
H,C;F,D	0.00167	0.00110	1.52
H,F;C,D	0.00198	0.00129	1.54
H,A1;F,D	0.00256	0.00162	1.58
E,D;C,A2	0.00164	0.00101	1.62
H,B;F,D	0.00196	0.00119	1.65
H,A1;E,D	0.00242	0.00146	1.66
H,E;B,D	0.00524	0.00258	2.03
B,D;F,E	0.00202	0.00098	2.05
F,A2;C,D	0.00216	0.00100	2.16
B,C;A2,D	0.00319	0.00147	2.17
H,F;A1,D	0.00243	0.00107	2.26
H,A1;C,D	0.00350	0.00153	2.29
H,A2;E,D	0.00295	0.00128	2.31
F,E;A2,D	0.00237	0.00100	2.36
E,A2;C,D	0.00250	0.00105	2.39
H,E;A2,D	0.00380	0.00149	2.55
F,A1;C,D	0.00152	0.00059	2.57
H,A2;C,D	0.00414	0.00149	2.77
F,C;A2,D	0.00323	0.00114	2.83
H,C;A1,D	0.00306	0.00103	2.98
H,E;F,D	0.00299	0.000992	3.01
H,F;E,D	0.00297	0.000885	3.35
H,B;E,D	0.00396	0.00113	3.51
H,C;A2,D	0.00466	0.00101	4.60
B,A1;C,A2	0.0274	0.00303	9.04
H,A2;B,A1	0.0284	0.00296	9.61
B,A2;C,A1	0.0295	0.00288	10.2
B,A1;E,A2	0.0255	0.00227	11.2
H,A1;B,A2	0.0288	0.00253	11.4
H,A2;C,A1	0.0306	0.00238	12.9
B,A2;E,A1	0.0274	0.00202	13.5
H,A2;F,A1	0.0271	0.00186	14.5
B,A2;F,A1	0.0270	0.00160	16.9
H,A1;C,A2	0.0290	0.00169	17.2
H,A1;F,A2	0.0281	0.00161	17.5
H,A2;E,A1	0.0294	0.00162	18.2
B,A1;F,A2	0.0276	0.00142	19.4
E,A1;C,A2	0.0312	0.00155	20.1
H,A1;E,A2	0.0280	0.00135	20.7
E,A2;C,A1	0.0313	0.00144	21.8
F,A2;C,A1	0.0311	0.00138	22.5
F,A1;C,A2	0.0285	0.00106	27.0
F,A1;E,A2	0.0264	0.000953	27.7
F,A2;E,A1	0.0289	0.000919	31.5

Supplementary Fig. S1. Cross-validation (cv) error against the number of ancestors (*K*) from *K*=1-12 and *K*=1,8,9,10,11,12 admixture plots.

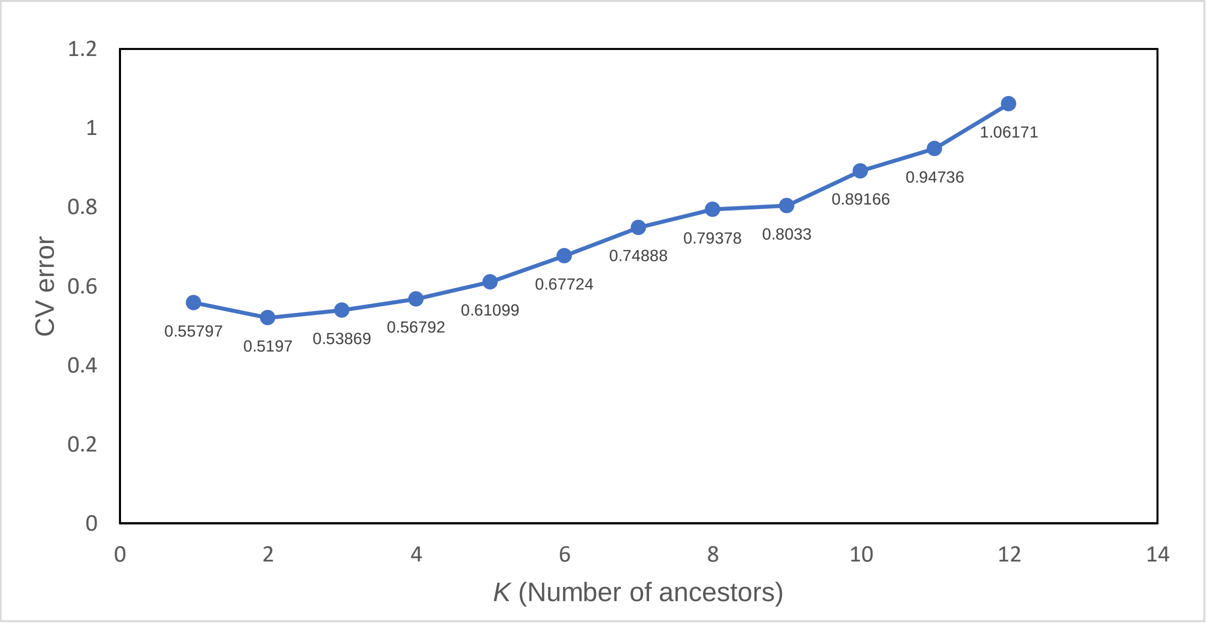

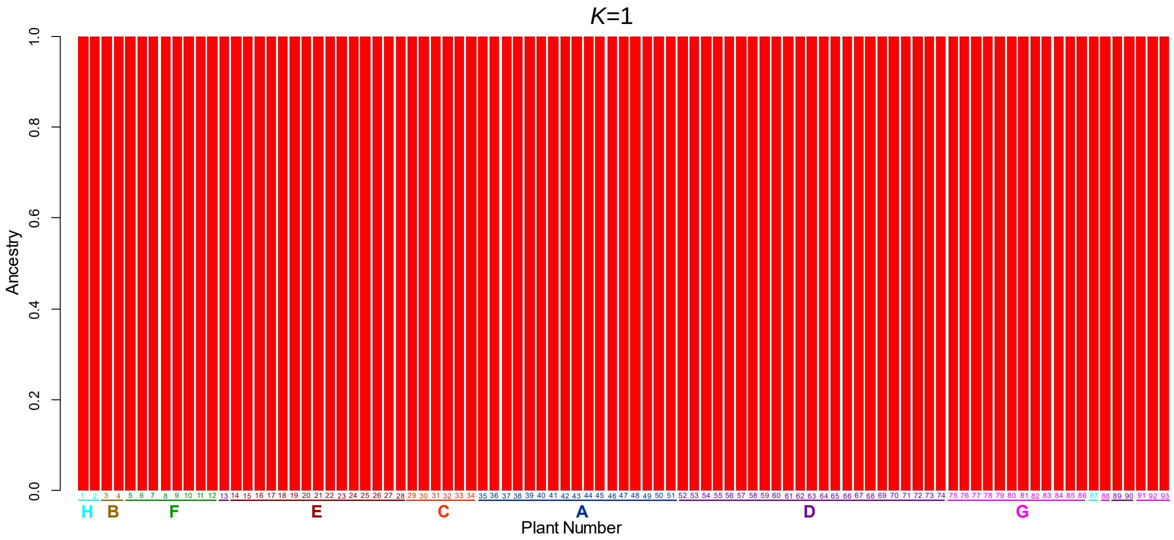

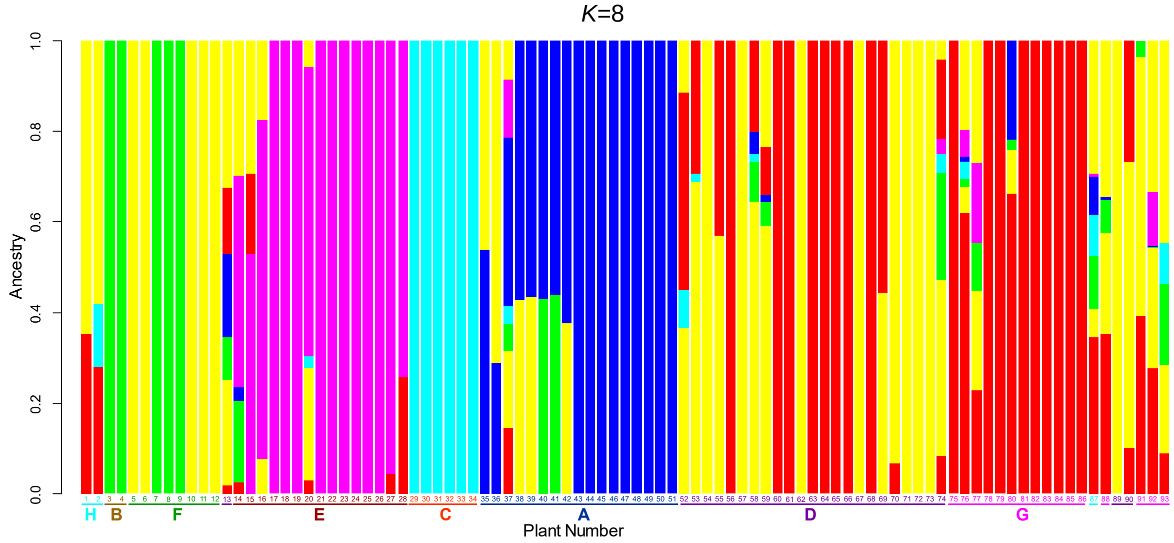

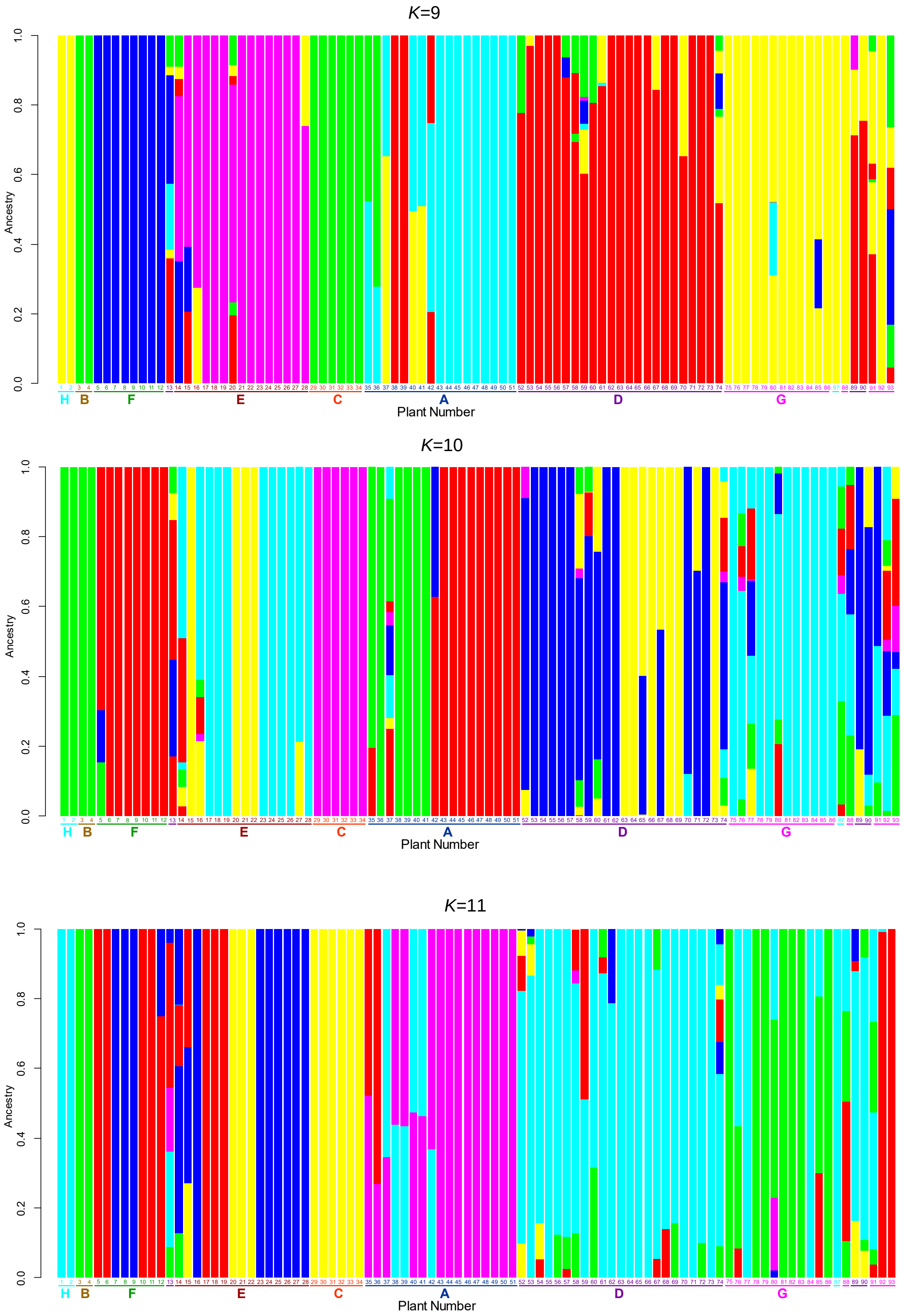
K=9
K=10
K=11
Ancestry
0.0
0.2
0.4
0.6
0.8
1.0
1 2 3 4 5 6 7 8 9 10 11 12 13 14 15 16 17 18 19 20 21 22 23 24 25 26 27 28 29 30 31 32 33 34 35 36 37 38 39 40 41 42 43 44 45 46 47 48 49 50 51 52 53 54 55 56 57 58 59 60 61 62 63 64 65 66 67 68 69 70 71 72 73 74 75 76 77 78 79 80 81 82 83 84 85 86 87 88 89 90 91 92 93
H B F E C A D G
Plant Number

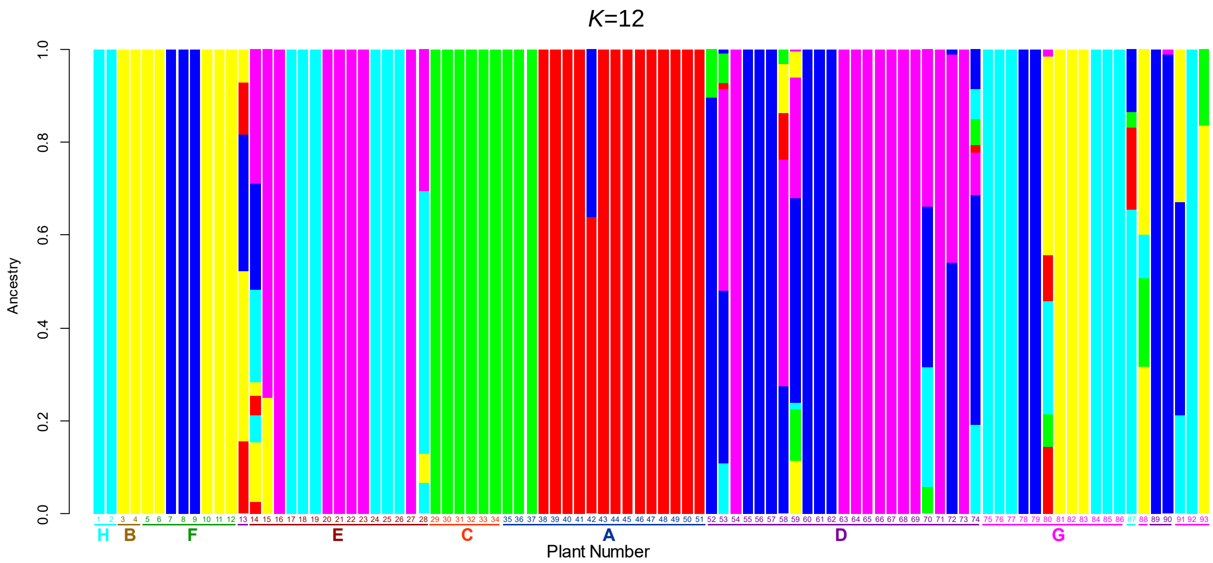

K=12
Ancestry
1.0
0.8
0.6
0.4
0.2
0.0
H
B
F
E
C
A
D
G
Plant Number

Supplementary Fig. S2. The pairwise alignments between plants 8 and 9.

```
CLUSTAL 2.1 multiple sequence alignment (clustal format)

8                YGGCKGTWTMCYWAMYKYKSRWYCRKWKCARRRYKYRYYRMKMKSAMWRRMYYKRWKYYY
9                YCRSKRYWKMTYWRMCKYKSRWYTRKWKGGRRRTGCAYYRMKMKSGMWRRMYYKRWKYYY
                 *  .*  *.* ** * ******* **** .***    ********.**************

8                RRRKSMWTGTRYYYKMRKKTTARYKYWYYKYYYWYYMRYWRWMKKKRRRMYRYRKSRRKM
9                RRRTSMWYRWRYYYKMRGKAGGRYKYWYTKYYYWYYMRYWRWMKKKRGGATRYRKSRRKM
                 ***.***   ******* *: .****** ******************    *********

8                MYMWRMSYKSRRYKRYYYYYYRRYYSYWWCRYSYRYAMWRYWYRYYMYRSRWKSWTWRRM
9                MYMWRMGTKSRRYKRYYYYYYRRYCGTATYGTGYRYCMAACWYRYYMYRSRWKSAWWRRM
                 ******. **************** .      .***.*   *************  ****

8                WYRYMMWRYWMRYRWRRYYRKWRYKTRYRRWMRYMSRAYYMTCRWYCTGYYYRCTRWCYR
9                WYRYMMWRYWMRYRWRRYYRKWRYTCAYRRWMRYMSGGYYMAYRWYMWKCYYGYGRAGYR
                 ************************.  ********* .***: ***    **   *  **

8                WSKYWKKRTMKMYRYMRMYMRKYYGWRKMMRGKKRYYYMKYKMYYRYYYRGAYYTRGYRR
9                TCKYWKKGYMKMYRYARMYMRKTYAWRKMMRRKKRCTCCKYKAYYRYTYRTGYYGGACRR
                  .*****  ****** ****** *.****** ***    *** **** ** .**  . **

8                YRYYKYWRRYWMCGYGCYRYYMKKWWMWMYKSWYCWRTWRWRYCMYYYYKRRMMYTWRRR
9                YRYYKYWRRYWCYKCRYYRYTMKKWWMAMYKSWYMWGYWRTACYMYYYYKRRMMYYWRRR
                 ***********      *** ****** ****** *  **    *********** ****

8                RMYRWRMRYKYKYRRYCRKARRWWYYMRKRMMSSYRMWKMKRRKMWYYMYRRWSRYCSWK
9                RMYRWRMRYKYKYRATARKCARWWCTARKRMMSSYRMWKMKRRKCAYCMCARWSRTYSAT
                 **************  .**. ***   *****************  * *  ****  * .

8                YWWRYMRRSWRMTTTYYYRYYRWWRYYKYRCRGYTAAGWYWYCMYATSKRTYYRRWMSYC
9                YWWRYMRRSWRCCCCYYYRYYRWWRYYKYGTGRTWWTATCAYYAYMKSKRAYYRRWMSTY
                 ***********    **************       :.   *  * .***:*******

8                GGGATWWYAYMYYWMMKRCAKYRYRMTYRTTKKYYRTTCKKGCCTCRRYYRYRTGMWYGK
9                SRACGWWYGYMYYWMMGGYRKYAYRMKCGGCGKYYRYCAKKKYTCTRRYYRYRCTMAYRK
                 . .. ***.*******    ** ***.     ****  .**     *******  * * *

8                RSMWMYYKRKKTKRMWMCCTMYYGKYRYRYRYRRTCTYRWAYTKRWKGRWMYKRMYKKYR
9                RSMWMYYKRKKYTRMACGTCMYYRKYRYRCRCRRYSYYRATTAKRWKRAWMYKRMYKKYR
                 *********** .**     *** ***** * ** . ** : :****  ***********

8                CYAKMKWRATCGRCKCYRYYYKKRKWMYRWKKCKWCGMYKKTWWGKRKKYKATTKAAMKW
9                YCWKMKWRCCYTRTKTCGYYYKGRKWMYRWKKTKWYTMCKKGATRKRKTYKMYYKGGMKW
                    *****.   * *   **** ********* **  * **    ***.**   *..***

8                YKRRRYYYTRTTYGGGRKSRYTGCWTYMKKKRKKYMKKRSMSMWKKRKRGAAKWRKYAYT
9                TKRRRYYYYRYKYRRRRKSRCGRAAKYMKKKRKGYMKKRSMCAWKKGKRATTKWRKYRCC
                  ******* * .*   ****   . .******* *******. *** **.::*****

8                CGRRSYYKWYYRKYKCRSRRGRKRAYKKRKCKKGGMRSWYRKKMMYKKKMRYTKMKAAGY
```

```
9               TAGRSYYKWYYGGYTSRSRRKRKRMYGKRKMGKARMRSWYRKKMMYKKGMRYCKMKCRKY
                 . ********  *..**** *** * ***  *. ************* *** ***.  *

8               SKTTTYYYRRKMKYRMKYMKAMRRYYYKRRKAGACCYGRTYYKRGMWKKRRKARYRYWKY
9               SKCCCYYYRRKMKYAAGYMKCMARTYYKRRKRRWYYTRRYYYKRKAWTKRRGWRYRYWKY
                **   *********   ***.* * ******       * ****  *.***  *******

8               TKKWRWGYSKKKYKRRRKYMYRSYYRKRMMMRYRYRSWWYRWKMWKMYCKRKAKYKYMWY
9               KKKWRWKYSGKKYKRRGKYMYRSYYRKRMMMRYRYRSWAYRWKMWKMYSKRKRKYKYMWY
                .***** ** ****** ********************* *********.*** *******

8               YASKRRGYYWSYYKMKWMKYKYGKRAAKGMYCGTMRYYYYCRRGAGAKYAYKGGCWGMWY
9               YRSKGRKYYWSYYKMKWMKTKYAKRWTKRMYYKWMRCTTYYAATGRTKYGYKRRMWKMWY
                * ** * ************ **.** :* **   **   *    . :**.**   * ***

8               AGACGKYYKKKKKYRKWRMMKCYAYYRSRKRRYYRRWKKTAAYMWYKYAKKKTYRWRMYK
9               GSWYKGYYKGKGKYRKWRMMKMCWYYRSRKRGYYRRWKGYMRYMWYKYCKKKKYRWRMYK
                ..    *** * *********   ******* ******    ******.***.*******

8               KYRYKYYWKRMKRKRRYYKWRKAYYYRKYKTGCYRYCRYRRGKRKGRYGWYKTYWYMYKK
9               KYRYKTTAKRCKRKGGTCKWRKMYYYRKYKYRYYRYYRTRRRKRKKACKWTKYCTYMYKK
                *****   ** ***    **** *******   *** * ** ***    * *   *****

8               TRYKWTAKKGYGTCRYKKCAKRKMMMYRYTKKKYKKKWKYGRYSWKCRCYYWMMYAGKRR
9               ARYKWCWKKKYAAYRYKKYRKRKMMMYGCCKGGYGKKWKYAACSWKYRMYYWMMYMRKRR
                :****  ** *.: ****  *******   *  * *****.  *** * ******  ***

8               KYRKRKTKKKCTTYRRYCCATKAYKYRWKGTCKMAAMWCKYKMWYYSRMAKKSYRYRKCT
9               KYRKRKCTTKTGGYRRYGAGGKWYKYRWKSCTKCWCMWTKYKMWYYGRMMKKSYRYRTMY
                ****** ..*   **** .. * ******.  *  .** *******.** *******.

8               RKRMYRGRKYYWKRRMYKKWYYKKSKYRTCMKKYRRKKKYKRKKMRKKGGRRYKRYWYYK
9               RKRMCRKRKYYWKRACYKKWYYKKSKYRCTMKKYRRKKKYKRKKMRKKRRRRYKRYWYYK
                **** * *******  ************  ******************  **********

8               KWRWYRRCGGARKYKKAYTGKKKRKKAAACMRKGKRRRMKWKKMRKYSKYYKCTGYCGSR
9               KWRWYRRYKKRRKYKGCCCCKKKRKKGGTMMRKKGRAAMKWKKMRKYSKYYKYKRYSRSR
                *******    **** .   ******..: ***  *  ************** . *. **

8               GAATAWWMKGKKKAATYTCAKMKGKGKTRYAYCTARGCAMGGYYGAGWRRKGYAKTTGYC
9               ARWWCWWAKKKKKCCGTKMCKAKKKKKYRYGCYCCRAYGMATTYRRRTGRKRTRKWYKYY
                .   .** * ***..  . .* * * * **.   .*. .*.  *     **   *   *

8               RRRCTGTWMWTCRYRAAKRMWYYKWYTKKYYGWMKRMSKCYRRRKYWKMYMARRKYMMKM
9               RRRMYKCACWCTRYRRRKGAWYYKAYYKKTYTAMKRMSKTCAAGTYTKMYMTRRKYMMKM
                ***      *  ***  *  **** * ** *  ******     .* ****:********

8               KYMGRMRKYRKACKYTSTMMYRRRMRWARKKKATGKMRRKYWYRRGGCKMYKKYMKYKYM
9               KYMKRMRKYRKGGGCYSYMMYRRRMRTMRKKKTWATARRKYWYRRTKYKCTKKYMKYKYC
                *** *******.    * ********  ****: .. ********   *  ********

8               YYRRCTYRKWYCCKYWRYTYYMKYCMKYYKGGWKKRKYTGGAAAKKWKRWMWWWMMYYWR
9               YYRRTYCGKWYAYKTARYKCCMKCTMKYCGACTKKRKYCRKRRRKKWKRWMWWWMMYYWR
```

```
     ****    ***. *  **.  **  ***  .  *****      ****************

8    RRKRYKKRMCKRCWYRGRARKMKTMYKTYKTWKRKKKYKTTRRRYAKSRSYYKAMKMKYR
9    RRKRYKKGMAKGYWYRKGGRTMKKMYKYYKAWKRKKKYKYYRRGYRKSRSYYKTMKMKYR
     ******* *.*   ***  .*.**.*** **:********  ** * *******:******

8    RRWYTKCTKGSMWKRKRYKYKTTTAWRRKKYMWYKWKAYTRTWRRYRYTTKYRKRTKCRW
9    RRWCAKGAKRSMTKRKRYKYKAACGWRRKKYMWYKWKRYAACARRYGCYYKYRKRYGYRT
     *** :* :* ** ********:: .************ *:   ***    *****   *

8    SRYRRGCKYYMKGKKKTTGMSKYRGKTWMYKGGTKWGAGTWKTAKTGAAMACGTAATMGT
9    SRCGRKTGYYMKKKKKKYKMSGCGAKYWMYKKRYKAAGCCWKYRKGSGWMCMAAMRYMKK
     **  *   **** ***.  **   .* ****   * ..  **  * .. *. .:   * .

8    YYYYYWAKWKKRKYKMYGCKKKMMKRCCCTRYYWRRKYRKMKKMYYKTMTYYGYTAKRYK
9    YYYYYWGKWTKRKYKMYKMKKKMMKRTTMGRYYWRRGYRKMKKMYCTAAKYYKCYGKRYK
     ******.**.*******  *******    ****** ******** .: .**   .****

8    KKWYMKKRRMMYARKRWSGKKMKACCKTAGGMKRRYRRCYMYMRYKCKKKKKKCWWRSYY
9    KKWYMKKRRMMYMAKRWSRKKCGCTTGYMRKMKRRYRRYYMYMRYKYKKTKKKYWWRSYY
     ************  **** **  .       ******* ******* **.*** ******

8    YCCTTCYYKKGGRCRYYMAWYYRYKWKKKKSMKYWKMGAGYMMSKRKTKGTAYKKWYYMG
9    YYYCCTYYKKKKRMRYYMMWYYRYKWKKKKSMKYWKMKWRYACGKRKYKKGGYKKATTCR
     *     ****  * **** ******************   *  .*** *  .***

8    CTYMYKRKKKKRWWRRRYRKYAMKYCCYRKYMWRKWWWRRKMSKGATAKYYYAGRRRKRY
9    AATMYKRKKKKRWWRRRYRKYWMKYTTYRKYMWRKWWWRRKMSKRRCCKYYYRKARRKRY
     .: ****************** ***  *****************   .****   *****

8    WWKYYYCCYYRKKKKRRRTRMKKWRKCCRKYKKKRRKRKCCKYMKRSRKYTWWKMKYRRA
9    WWGYYYTTCCAKKKKRRRYGMKKWRGTTGKYGKKRRKRKYYKYCKRSRKCAWWKMKYRGM
     ** ***     *******  *****    ** *******  ** ***** :*******

8    GGAWKGRKKARMKRRRYGGAKARKYRKCWKKAYCTGKYYYYWTKYGYAGMRYYMAKMMSM
9    SRRWKKRKKRRMKRRRYRRMKCRKYGGYWKKWYTAKKYYYYWYKYAYMTAATTAMKMMSM
     .  ** *** *******   *.***   *** * : ****** **.*        *****

8    YYCCRWRMMRRYMRWRKRKYKYGCKMKCKRYRRKRYMKKCCGMKKMMCGRYKKKKMYKKR
9    CTYTRWGMMRRYMRWRKRKYKTAYKMTTKRYRRKRYMTKMARMKKMMMRRYKKKKMYKKR
         ** ************** . **. *********.* . *****  ***********

8    MKCKMKCKKYRYKSRYYMYWKACRTKRRTGWRRRRYWRYMAGYCGATCWKWRGGTYKKYG
9    MGYKMKMKKYGYKSRYTMYWKGMRCKRRARARRRAYWRYMTRCGTRGYWKTRAAYYKKYR
     *  *** *** ***** ****. * ***:  *** *****:       ** *.. ****

8    TCYWCRMRTKATYWKYMKYRGKRRRKYKGRKGRMKYYTTTAAKSRGTGCKYMCKRRGTGW
9    WYYWYAMAAGCCTWKYMGTGKKRAGTCTKRKRRATYYACCWWTSRKCAYKYMMKRGKGAA
       **  * : .  ****    **  . . ** * .**:    .**  . *** **   .

8    YWCMMYMYYRKAMRYKYASKYGRWRCTKWKKACGWYRRGTARRKKMCYATYMRKSRRRKM
9    YAYMMYMYYRKGMRYTYWSKYRAWRTCKWKGMYRWYRGTAGRAKGMYTRKYMRKSRRRKM
     * ********.***.* ***  **  ***    ***  :.* * *   .**********
```

```
8          MYYRKATKGGTTKKWWARKKMRRRAKSKWRYYRKYRRYYRRYWMRRMGAKAGKMRTTTGT
9          MYYRKRYKKAGGKKWACRKKCGGGWKSKARYYRKYAAYYRRYWCGGARGKWTKAGCGYTY
           *****  * .  *** .***     *** ******  ******     .*  *

8          MAGARACGMYYYYKGRRYGWYYYCAAYGASKRTCCRMRRTYWGACKWYYGRYARMAAACK
9          ARATRWYKMYYYYKKRRCAAYYYMTGYTWSKGCAMRMRRACTATMGWYYKAYWRMWRWYK
             .:*   ****** ** . *** :.*  **  . ****:  .:  ***  * **    *

8          GYTYKRRYWTCTTAKWKYAGKTKATMRAKMKYYKRRRKKKRKAYKYRCKYKYCAKYKRYM
9          RYWYKRRYWGTAWWKWKCTTKCTWAMRGGMKYYKRRRKKTRKMYKYRTTTKCYTGTKRYM
            * ******  :  *** : * . :**. **********.** **** . *  :  ****

8          YRRWKGRMRKKYAMRTCWTGWKRKRRKYMRCAAKMYWKYRRSRRYRRMRTCKWRKYYGAA
9          YRATKAGCRKKYWMRCSWCAWTGKRRKYMRMRRKMYWKYRASRRYRRMRYMKWRKCCRRR
           **  *.  **** ** .* .*. *******   ******* ********  ****

8          MCTCTYKAGTCKKRTKKYKAACCTTYRWWRRYRYYYYKMYMKWYACCYCKKYRRGYKTWK
9          MMYTYYKGTAYKKRYKKYKMRSMYKCATWRRYGCCCYGMYMKWYTYMCYKKYRRKYKCWK
           *    **. : *** ****  .  .   ****    * ******:    ***** ** **

8          RMRRKKKGAGAKRWYKKYKMGKRRRGGWRYMYKRMKYWTAWRYKRKWSKGYRCRRYYKKG
9          RMRRKKKRRRGKRATGKYKMRKRRRRRWAYMYKRMKYWCCWRYKRKWSKKCGMRRYCKTR
           *******   .**   **** ****  * ********* .*********    *** *.

8          YWKRTGCAYRSMYWKMKAKGCGMRYYKRKKWMYRKRRKWKYTYRYGRYSARKRKKYGKKY
9          YWKRWRYWYRSMYWKMKWKAARMRYYKRKKWMYRKGAKWKYYYRYKGYSMRKRKKYKKKY
           ****    ********* *.. *************  **** ***  ** ****** ***

8          YYMKKRCKYWRTGKWKMYKCRWKYGYTAYKYGRYKTKTTYGCRKKMYKCTKKKKTCTCCT
9          YYMGKRYKYWRCAGWKMYGYRWKYATCRCKYKGTGKKYYYRMRKKMYKYYKKKKKSYYMY
           *** ** **** . ****  ****.    **    .*  *  ******  ****..

8          CTRWCAGKKYRAATYRMAMKKYMYTTCYCKRKKYAKRKKWSMYCATTGGKYYKCTGYATW
9          TYRWTGKKKYRWRYYRCRAKTTCYKKYCYTRKKYWKGKKWSMYTCKYRKKYYKMYRTGCT
             ** . ****   **   *.  *..   .**** * ****** ..   ****    .

8          YYKRKACAMRKMKRWCAKKAAGGCYGGSRARYKGYYCKRRKMASCYGTGKWRCTRTARSY
9          YYTGGMTGMRKMKRTYCGKGTTAACAACGRGYKKYTYKRGGCRSMYKWRKWRSAGCTRSY
           **.    .******  . *.: .. ...   ** *  **    * *   ***.:  :***

8          RMYYTRRYRYMTTWWRCCYCTKTYMMYRYRYKCTGACCCTTAACYRGKTYARYYRWTMTA
9          RCYYKGRYRYCKWWWGTATTKKCCMMYRYRYKYGKCYGYCAWWYYRKTCTCGCCGWWMYR
           * **. **** . **  .  .*  ********   .    :   ** .  .    * *

8          TCKYYYCSRTKKKGTTSYAYAYWRTMCKYYWRYRWKKYTGCGRYKYRWCRKRKWSMYKWR
9          YYKYCYYCAYTKKKYKSYTTGYWRCATGCTARTRWKKYWRMAGTKYRWYGGAKWSAYKWR
             ** * .  .**  .**: .***       * *****   .  ****    *** ****

8          CMRAGKYKKKWGRKYRYAAKRMACWKRWYGARCMYWYCGKKRKYASRMMKKRAAAKYYKR
9          MMGGAKYKKKWRGGTAYCGKRMTYTKRWYRRRMMYWYMSKKRKCGSRMMKKRGTGKYCKR
            * ..******     *..***:  ****  * **** .**** .*******.:.** **
```

```
8              KGARKGAMKKWCGWYKAYKKYSMKYMMRKCCGRRKRCCCYWYWSMWRKCATKMCMRRYMY
9              KTRRKAGCKKWSRWCKMCKKTSCTYMMRKYYKRRKRYYYCWYWSMWRKYRYKAMMRRYMY
               *  **.. ***. * *  ** * .*****   ****    ********   *  ******

8              GYMKKMYYYRKTTKWRRRWTYYRAKARRWKSRYYKKKYYRTTKYKRKSKRCRYKSKYRKK
9              RYMKKMYYYRKWYKWAGAWYYCGRKTRGWKSAYYKKKYYRYKKYKRKSKRMRYKSKYRKK
                **********  **   * *   *:* *** ******** .******** *********

8              YRRSYKRYYKYKSSWRKKYSTKKYWKKYGCSGKWMRRRMGCCRRKKKYMRTAKRMMGCWY
9              YRRSYKGYYKYKSSWRKKYSYTKYWKKCTYGAKWMRRRMKYYRRKKKYMRAGKRMAKYWC
               ****** ************* .*****   ..*******   ********:.***   *

8              MKRKKYYYKWSSAMRSCGGAKKYTAYMSRRRTMRMKYKYRKKWWWYYMYKYRKWYGCKKY
9              MKGKKYYCKWSSTAGGTRKWKKYYRYMSRRGYMRMKYKYRKKWWWYYCCKYRKWYAYKKY
               ** **** ****:  .    ***  *****  ***************  ******. ***

8              KYRRYRKRKTTSWMMWRTGATCGKKKRRGYGWYYMKTKMRWRGYYRKYKCGYMKMYRYKK
9              TYRRYRKRKYKSWMMWRGTRWMRKKKRGRCSTYYMKWKMRWRKYYRKYGAATAKMYRYGK
               .******** .******      ****   . **** ***** *****  ..  ***** *

8              KMWARYMYRRMRRMMRRYWKMSRRWKYWKKTKRRTKTKRWKYYKTYAMACAKSWYYYWWK
9              KMWRRYMYRRMRRMMRGYWKMSRRWKYWKKKKRRAKYKRWKYYKKYWMRYRKSWYYYWWK
               *** ************ *************.***:* *******.* *   *********

8              KYYTRYRTWYYYAKYRCMMRWYKYGYKRRYKKGYKYRYKRKRKRYMYKRKRYYKKYWCAT
9              KYCYGTRYWYTYRKCRYACGWYKYRYKRRYKKKYTYGYKRGRKRYCCKRKRYYGKYWSGC
               **    * ** * * *    **** ******* *.* *** ****  ****** ***..

8              GKTCTYMKKKWKGTRAMARYYWYKYKYRKMMKRYTKWYYYRGMKTKYMTYGRYKRYWYCA
9              CKGYCTAKKKWKRKGMCMRYYWYKYKYRKMAKRCGKWYYYRKMTGKCMCYKRYKRYWYTG
                *     ***** .    ************ **  ****** *. * * * ******* .

8              CWTYYKTYSYWYSYYYRGYMYYGYMARKRCRKYMRYTTTGKKYYRRWWRYKAKYRCCAAY
9              AWGCYKKYSYWYSYYYAKCCCYRYMWGGRMRKYMRYCCCAKKTCGRWWACKWKCGTTGRY
               .*  **.*********     * **   * ******   .**   ***  * *    . *

8              WRRMCSRKMKYAKYRMRKYYMSWKRYYRKYR
9              WRRCYGAKMKYMKYRCRKYYMSWTRYYRKCG
               ***  . **** *** *******.*****
```

Supplementary Fig. S3. The pairwise alignments between plants 33 and 34.

```
CLUSTAL 2.1 multiple sequence alignment (clustal format)

33              RRYYWYMCACKKGARTWRKYRYYYMMYWWATYYKMWRYWRWSRMKYCSYRKKKYTMMSWY
34              RRYYWYMTGYKKKCGYWRKYRTYYMMYWWWWYCGATRYWRWSRMKYTCTATKKYGMMSWY
                ******* . ** .  ***** *******  *    ********** .  .*** *****

33              ARYGRYYRYRRWYYWRWYRRMCAGYGMSCKRRRTSMRMYRRKRYYRWWMRGCRMMRRMYA
34              TRYRRYYRYRRWYYWRWYRRCGGKCCMSTKRRRYSMAMYRRKGYYRWWMRATRMMAACYG
                :** ****************  .   ** **** ** ***** *******. ***   *.

33              YYSWTKYMYYTGWKSYYRRKYMWYKGCWSTMKYRWYYYYYYRRMYRYTRTMYYMWYKRRR
34              YYSAWKYMYYAAWKSYYRRKYMWYKAAASYMKYRWYYYYYYRRMYRYCGYMYTCTYKRRR
                ***  *****:.*************.. * *****************   **   *****

33              KRKKRYTCKMRAMRYRWWYYMMMTCYTCWKRYCTGGSARRKMMYKYSYYYYYYSMYRTAR
34              TRKKRYCMTMRRMRYRWWYTMMMAAYWTWKRYTKRKSWRRKMMYKYSYYYYYYSCYGGTR
                .*****  .** ******* ***:.*  **** .  * **************** *  :*

33              YYRAATYMRTKYCMGYWKRMKYSRYMYTRRYWRKGMCMMYSCYAMKWYYYWRYWRWTKYK
34              TTGRGCTARCKYYAKYWKRMKYSRYMYWRRYWRKKMYMCCCMYCMKWYYTAGCWRTKKYK
                    .   * **   ************ ****** * *   . *.*****    ** .***

33              MTGYYKGARYRCRCSYYWKYYWRYYCMYWWYSRRYRMRRMRMYWMGGGRTYKWWRYKKRK
34              MAAYYKTRAYRAAASYYWKYYWRYYYMTATYSRRYRMRRMRMYWMRRRACYKWWRYKKRK
                *:.***   **. .*********** *   ***************     **********

33              MCCAWMMYYMRSKRYKTYKYRMYKRYMKKKMTYRKMYMWMCAAGTYTACGMSYKRRRMTK
34              MMMRAMMYYMRSKRTKAYKYRMTTRYMKKKMCYRKMYMWMYWRRYCYGAKASYKGRRMGG
                *    ********* *:***** .******* ********       ..  *** ***

33              MWAGARWTYYRYMMRRCCGYRTRRACTTAMWWSYMYWATYTAYGYKKCYGCTTYCGTGGG
34              MWRRMRAKYYRYMMRRYYKTACRRGYKWMMWWSYMYTGCTKRTRYKKTCSTYKYMRWTAA
                **   * .********      **. .  ******* .  .   ***  .  .*    ..

33              CAGCKYKRKSYYMKMKWYSYYKYMCAATTAWTTYYKARRRRSYSKRSRYKMAKYWKGGAA
34              MMRSKYKRGSTTAKMKWYSYYKYMYCCAATTACCTKRRRRRSYSKRSATKMRKYWTTARW
                   .**** *   *********** ..::: :   * **********  ** ***. .

33              YYRRMKKRYYRKYSRKWMCYCGYCARMKYACGRCAAAATYYSWWKRATGRWRYRRRWTCK
34              YYRRMKKRYYRKYSRKWMTCGACYRRMKYGTAATTTTCKYYSWWKRGGRRWRYRRRWYYK
                ******************   .   ****. .  :::..*******.  ********  *

33              MMYRWKYYKWYKKYRKKKMYYMYWGMAMYKSYYKMKGTAKATRRKYKRKWYRTTRKYYWK
34              MMYRWKYYKWYKTYRKKKMYYMYWRMMCCKSYCKMKTWGKGCRRKYKRKACAYCRKYYWK
                ************.*********** *   *** ***  .*. *******     ******

33              KMKTRWKMYGSMKWRAKGCMKKWWYKKYYWKRKKWWYMMYRYMRKKKKAYRMSKYWYKRK
34              KMKKRWKMTTGMKWRRGAYCKKWWYKKCYWKRKGWWYMMYRYMRGTTGCYRMSKYWYKRK
                ***.****  .****  .  ******* ***** ********** .. .***********
```

```
33          KKMMMKRKKMMYKKRTRYMRWTRKYYYYKKMWKKKYYYYKKGCCCAKCRRKRKTWTATGC
34          KKMMMKRGKMMYKKACRYMRWAGKYYYYKKMWKKKYYYCGKRMYYMKMGRKRKGWYRYAT
            ******* ******  *****: ***************  *     *  **** *   .

33          KRTMRSKCCCKKMYYAAAKMMKMYMKSWKKKKYKRKRWKRKYMKYKKSKMTTRRMYKRWC
34          KRWCRSKTYTKKMCYCGGKMMKMYCKSWKKKGYKRKRWKRKYMKYKKSKMGCRRMYKRWY
            **  ***   *** *...****** ****** ******************  *******

33          RRYKKGKYRYRRYRKKGRKRRAKCTYRYKKKYRRYYKRWYRWRTSYYRGGGTRWMYRWYT
34          RAYKKRGYRYRRYRKGARKRRGKGCCACKKKYRRYYGRWYRWRWSYYRRKKKRWMYRWTC
            * ***  ******** .****.*     ******** ****** ****   .******

33          YKYYYGARSYYYSYRWRRYYKKWYKRTRKGGGKAATKRKYYKAGGMKRYCKKYMMYWRRK
34          YKYYYRCRSYYYSYRTAGYYKKWYKRKRKTKAKWRYKRKYYKCCKCGRYAKKCACCARRK
            ***** .********   ********.**  .*   ******.    **.**     ***

33          RYSKYKSKYAYGWYATTGTYYYTWKCCACAYGRKWWWRKYGCTTRYYYYKWSWRMYKRWM
34          RYSKYTSKYWYRWYGGCTYTTTATGGYGAGYRRKWWWRKYRYWKRYYYYKWSWRMYKRWM
            *****.*** * **.       :    ...* ********   .****************

33          WRTYYAWYTKMYYAAYYYKKTMKRAYRKRKYYKMYMRRKAKYRYYRMYYRYKKKMYWKYR
34          WRKYYMTYCKMYYRRYYYKKKMKRTYRKRKYYKMYMRRKGGYRYYRMYYRYKTKAYWKYR
            **.**  * ****  *****.***:**************. ***********.* *****

33          YKYWKMYCTTGKKMRGCGKAAAKRAAYMRKRSAWWKKYMRKYRRACAWTCYSWYKATATC
34          YKYWKMYACCAKKARRMRKGGTKRRMCMRKRSWWWGKYMRGYRRTTMTCTYSWYKTCGCY
            *******.  .** *   *..:**   ***** ** **** ***:     *****: .

33          YSCCYRKMCACYKYYWYSYYRMKYKWYKAGGYTYKMWKKKCKGCTTAYGARRAACKMCGT
34          YSGMYRGMMMYYTYYWYSYYRMKYKWYKGAACCYKMWKKKTKAACYGYKWARGGTGMMKA
            **  ** *   *.***************...  ******* *..  .*   *..  *  :

33          CCKWKKRWRWYRKSTRKGYRCCGCKYKYMTAGRKKKYKKYRWCTATCTTCRYYRYKKKKR
34          TTKWKKRWGWYRKSCRKAYRYYRYKYGYCCMKRKKKYKKYRWGGGYYYYMRYYGYKKKKR
              ****** ***** **.**    ** *    **********  .     *** ******

33          MWRYYKWRWKTKACTWRYRKKMRKKYMYCKKKCKKYWKKRRKKKMKMYKYCTTTRTYWCK
34          MWRYYKWRWKKKWYKWRYRKKMRKTTACYKTKTKKYWKKRRKKKAKMYKYACACGATTTK
            **********.*  .*********.    *.* *********** *****. :  :   *

33          KKWWKYSMYRRRKTATTWGKRKKRYKMSRSYMMYWAGRYKMCAAGKKRAGYGAWWKMKKY
34          KKWWKYSMYRRRKAGCAWCKAKKRYKMSRSYMMYWMKRYKAYRMRKKRGACARWWGMGKY
            *************:. :* * **************  ***     ***.. . ** * **

33          YYKKWKKRKKRRWRRRCSRAATAKYTTGYWYGKAGGWYKYWYMYMRKCAARKRKKKKKYY
34          YYKTWKKRGTRRWGGGTCGTGKWKYWCRYWYATGRAWYKYWYMYMRGYRRGTGKKKKKYY
            ***.**** .***    .  :.. **   ***... .**********     . *******

33          WKMKWRWWWYWKKRRRYYRYYTKRKKTRWMKGTMKKSYKRTWAKYKMRKMKYKKMRRWKY
34          WKMKWRWWACWKKRRRYYRYYYKRKKKRWCTRWMKKSYKRGTRGYKMRKMKYKKMRRWKY
            ********  *********** ****.**  .  *******    ****************

33          RKKAGTKRKGTRYYRYMCMKWWYKKCYYYYWYYKYKKAGTKRKYYRWKYKKMRKKKRKTK
```

```
34              RKKGAGGRKAYRYYGCCYMKWWYKKTCYYTACYKYKKMTYKRKYYRWGCKKMRKKKRKAK
                ***..  **. ***    *******  **   *****   *******  *********:*

33              KKKSYYRRGYRYGTTYMYWRSRYAYWTAGKKTMKKAWWRMMKYKKRYYYYMGMRYRKMKK
34              KGKSYYRRRYRYAGCYMYWRSRYRYWWWSTKKMKKMTWGMMKYKKRYYYYMRMRYRKMKK
                * ****** ***.  ******** **  ..*.***  * ************ ********

33              KKMMKMMKKSYRWGWRRYYRWKKKKRTMTMKKKKCRYYRWSRRKRWWTRYYYKRRYYKRR
34              KKMMKMMKKSYRWSWRRYYRWKTKGGCMGMKKKKTGTYGWSRATAWTGRYYYKRRTTKRR
                *************.********.*   * *****   * *** . *  *******  ***

33              RKKGKRYKRCAYYKKAKCCAYRKTGGCTAKKSYKMYGAKKYCTTGKYYGARRYRRGGCCT
34              RKKRGRYKRTMCYKKWKYYWYRKGTTYCRKKCYKMYKTKKYMYWKKYYATGGTAGRAYYK
                ***  ****   *** *   ***      **.**** :***    ***.:      .  .

33              YKRRKRKCKRRKRKKYTKRYTTTTTTYYTWTMKYTWYRWGWTGACTMCGMYRKYSYYKKK
34              YKAAKRKMKRRKRKKYAKRYYKYWWYTYKAACKYWWCGARWCTCMKCTTMYRKYSYYKKG
                **  *** ********:*** .     *. : ** *    *  . .   **********

33              WMRYRWRKGGRYYKGTYSYKKSRYMRYRRMCRYKWRRYMKKKTRTCTTAKYRKKMRRTGA
34              AMRYRWRGAAATCKRKYSYKKSRYMRYRRMYRTKWRRYMGKKKRYMYWWKYRKKMRRYSR
                 ****** ..   * .************** * ****** **.*     ******** .

33              RKMRKGRYWKKYWSMRYSTTCCRYKYMCGCGWAGTMSRCRKKYYKRYSGCCCGKRYRCAK
34              RKMRGARCAKKYWSMRYSYYYYRYKYMAAAAWRAKMSRAGKKYYKRYSAATTAKRYRARK
                **** .*  *********    *****....* ..***. ********..  .****. *

33              KYKRKMWYYSKCSTKMTRMGRGCGRRKKKRYKKYYGCTATMTYWYKKYYYKCAGRRAYWM
34              KYKRKMWYYSKTSKKMARMRGRYARRKKKRYKKYYKTWWYCYYWYKKYYYKYRRRRRYWM
                *********** *.**:**    .***********       *********   ** ***

33              RMWKRTRKWATAGYYRKRKRSKKKKKGYAWKKTWYRRRRKYWRRYYYYMGKKKKWRSCYK
34              RMWKRKRKATGTKYYRKGKRSKKKKKKYGTKKCTCARRRKYWRRYYYYMRKKKKWRSMYK
                *****.** : : **** ******** *. **    ************* ******* **

33              AYCYRYWYMRGAYKKWTYYRYYWWYGATACYRRYYKMKRKMRRWMAMGRASMMARMMWGA
34              GCYYRCWYMRRRYKKWKYYRYYWWYTCWGMTGRYYKMKAKMRGACMMAAMSMMMRMMWRW
                .  ** ****  ****.******** . .   ****** ***    *.  *** ****

33              WKRKMYYRYKKRYYTACCYKKAYRKYTCKYYRRWMCCKRAAKMKRWRMKRRYKWWYMWWG
34              WKRKMYYATTKRYYCCTAYKKWYRKCCAKYYRRWCTTKARGKMTGWAMKRRYKWWYMWWS
                *******  .**** . .*** ***  .******   *  .**. * ************.

33              RTKKCYYARKCTGCSYRKKTACKRWRRRMYMYRYYRRRGRWCRYMYMRYWGGAGYKYKYC
34              AAKGYYCRATYYRSSYRKKWMMKRWRRRMYMYRYYRRRKRWMRYMYMRYWAATAYKYKYA
                 :*  *   .   .*****   **************** ** ********..:.*****.

33              ARKYMYSGAACAAWYYKKKKCGWKMSKMRRYYRKCYKYRKWKKKRSSKWKKKSRKRKYAW
34              RRKYMYSRRMYWGTYYKKGKGKWKCSKMRRYYRKSYKYRKWKKKRSSKWKTKSRKRKYTW
                 ******     . **** *  ** *********.***************.*******:*

33              AGGTTRCGCTKYKSCWKCCYYMWYRRRCAGMWKKKKWTTTCGCRCYCRMMKKGKMYRMRK
34              RRRYYRYATAGYKSTWKYYYYMWYRGRSRRMWGKKKWYWYMRMRYYYRMMKGAKMYGMRK
```

```
                         * . : *** **  ****** *.  ** ****      * * **** .*** ***

33                  GYKWWKWAYYKKMYWYCCGMKKSWSYMKYMMKKTAMGRYKKRKKATTTYWTKKACGRGGK
34                  AYKWWKWRYCTKMYWYTMRMKKSWSYMKYMMKKWMCRRYKKRKKCCCCCACKKGTARKSK
                    .****** * .*****   **************    *******.      **. .* .*

33                  RRRKCYGMYWYMYKMCAAGTYCGMSYWYYCRTMYYSRKRGCGMYKMRKYYRKMWRYKRAG
34                  RRRKGCKMYWYMYKMARCRYCTAASYTTCYRYAYYSRKGATAMYGMGTYYRKMARTKRCA
                    ****   ********. .    . **    *  ***** . .** * .***** * **..

33                  TAWRSTCAGYAATKKGTAWRRKTGTYGKYKGCYMRGRYKWMYCTRWCYKYKKKTCMRKMR
34                  AGWRSWMMRCGGAKTKAWWAGGCACYTKCKKTYMRTRYKWMYTAGTTYKYKKKKMARKMR
                    :.***     ..:*. : *    . * * *  *** ****** :   ******.  ****

33                  KGGGTWYKSMWRWKMWKWWKRKAGYYKRKRYTRMKYMYKYRWYRYYRYKRAKCGTKTKKY
34                  KKTRCWYKCMWRWGCAKWWKRKGTYCKRKRYKRAKYMYKYRWYRYYRYKACKYRYKYKKY
                    *    ***.****   ******. * *****.* *************** .*   * ***

33                  MMKYRSRKKKKKKRRRRRYMWRYRKTTMAKYYYACTKYKKKGTCKKWSKKTGYKKRYKYRW
34                  MAKYRSRKKKKKKRRRRRYMWRYRGACAGGCTYRAYKYKKKACGKKWSKKKRYKKGCTYRW
                    * ********************* :  .   * . *****.  ******. ***  .***

33                  RRYKMKTTTKYKGWRATTRYTAAYTYWWRYRYMWRTMTWYTCKRKYTATYRMKYKKTRSM
34                  RRYKMKWWYKYKKWRRYYACWWWYYYWWRYRYMWRYCGWYYYKRKYYMKYRMTYKKKRSM
                    ******   *** **        * **********   **  ****  .***.***.***

33                  YAKTWATGGARMMCKYYYMYAGRARKKKRKTTTTCAKWATKKRMRGWYMWMMYGGYYKKY
34                  YGTGTRYSKRGAAMKYYYMYGRACRKKKRKGGACTGKWRYTKRCGKWCMWMMCAAYYKKY
                    *..    .      ******.  .******  :  .**  .**  * ****  ..*****

33                  YYWYRKRRGYATTCKKYMAAKRRKYMATTMKKKKKMKWKKYYMKGRKMYYWRKWRRGWCK
34                  YCAYRGRRACCCYMKKYCGWKRGKYMRACMKKKKKMKWKKYYMKKRKMYCWRKWRARAYK
                    *  ** **. .   *** . ** *** : *************** **** *****    *

33                  YKKYKGRKTGTTGATCCGGAKGTAYTKKGKYYWMKKRMMRYKKGYKRKKWKKRYMWRRYK
34                  YTKYTAGKARCYARGYYATGGAGGCKKGKKYYWMKTRMMRTTGRYKAKKWKKRYMWRGCK
                    *.**.. *:   .    . . . . .*  ******.**** .  **  **********  *

33                  YWYYMYTTCYRWKYGMRRRRKMKTGCRKYYRWMYMRSTKRSRAAWYRTTRKYYYKKYMKS
34                  YWYYMYWKYYRWKYRCRRRRKMKCRYGKYYRWMYMRSCKRCGGTTCGACRKYYYKKTAKS
                    ****** .*****  *******    ********** **. .:   :*******  **

33                  MYKTTRCYYYYWWKMRYYSYCMGCWKTRKGAWAKRCMYKMAAKKAKKSKWKAYRKYYKYSY
34                  MYKKKGYYYYYWWKMRYYSYMMKYWKKGGAGTTKRTCTKMGCKKTTKSGWKMYRKYYKYST
                    ***..  ************ *  **.  .. :**   **..**:.** ** ********

33                  RWRMAAWMCKYWAGRYKRKRAKRKYRYRTRCCKKRKRRRYYYYYYKYKRKKRYCKKRYKK
34                  GTACCCWMYTYWCARYKRKRRKRKYRYGKRGYKKRKRRRYYYYYYKYKRKKRYAKKRYKK
                        ..** .**..****** ****** .*  *********************.******

33                  MYKMASYWRTYCCATYAGGKSRGTMSMAKTRMKKKKR
34                  MYKMWGTTAWYTTGCCGTAGCRRCMSMRKYRMKKKKR
                    **** .    *  .  . . .*  *** * *******
```

Supplementary Fig. S4. The pairwise alignments between plants 3 and 4.

```
CLUSTAL 2.1 multiple sequence alignment (clustal format)

3               KRYMGAYACYGTAGATMGGTTGTTMRGMYMWCGYGYWYWWKYRRKRGWMSMARYYYMGKR
4               KGTCTCCTYYRWMACCAATCAACAARTMYMWSRYKYWYWWKTARKRAWMGARRCYYMAKR
                *    . : *   ..  .  :. : * ****. * ******  ***.**.  * ***.**

3               WRRKWTKRRYAARYYYMMYSMYSYYYGRRAMSWTGKCRRRMTCCYGAYWCWAWYWRWYYR
4               AAGKWKKRRYMRRYCYMMYSCYSYYYRRRGMSWWRTYRAAAYYMTTGYWYWMWCAGACTG
                   **.****  ** ***** ***** **.***  . *        .** * *

3               WKMWKRYKKYSWRYWYRKYRYMYWRMMMYRCMWWRYMTCWYRRWAYGKSWSCRRYYRRCM
4               WKMAKRYKKYSWRCWYRKYRCAYWRMMMYRYMWWRYMWTTYRRWGCAKSWSYRRYYRRAM
                *** ********* ******  ******** ******   ****. .**** ******.*

3               KSMMYRRCSKMMARWYTGKRTYMRYRKYYYRCMRYRWWYYGYKAAWWKKYYYRGWWRMWY
4               KSMMYRRYSKMCTRWYYRKAKTCRYRKYYTRYMRYRWWYYRTKGRTWKKYCYRKATACWY
                ******* *** :***  * .  ****** * ********  *.  **** **     **

3               YKKWKMAGYMKKWWWMYRRYWYYSMWMWRWRMYYTSYWRRKMYWKGCRYSRMRMMKYGSY
4               YKKWKCGAYMKKWWWMYGRYWYYSMWMWRWRMYYKSYWRAKMYWKCTAYSRMRMMKYRSY
                ***** ..********* ****************.**** *****   ********* **

3               KRRRKYRRYYYYGWYARRRYMYSMRYYRYGWYSWMRMYRYYMKMKWRCKKRMGCMWYRKY
4               KRRRKYGRYYYCTAYRARRYMYSMRCYRYRWYSWMRMYRYTCGCKTGMKKRMTYAAYRKY
                ****** ****   *  ******** *** **********    *   ****    ****

3               WYMRYYKKWCKAYYYRGAKGRRGAMRWGRRSRYKRMWATWWYYYMYWRYWRMMYTYCKWS
4               WYMRYTKKWMKRYYYRSGGRARRRMRWAARCRYKRMWWWWWYYYMYWRYWRCCTCYMKWG
                ***** *** * ****..   *  ***. *.******  ************    * **.

3               RKRRRWRYWRYYRKRKAKGAKKMYGAMWKYRWKSRWYKYWRCCCRTAYRTTTYMYRYRWY
4               AKRRRTATARCTGKRKTTRGGGCCARCTKYRWKSRWYKYTGTYSRCGYGGCGYMYRYRWY
                 ****    *   ***:. .    .   ***********    .* .*    ********

3               KYRRKGWRGKAGKTTCTGASWMYCACGGYWMRKTMTTTWRRAYGCYTTCATRGMMKRCYR
4               KYRRKAWARKMKKKYSKRMSWMYSRTATYWMRKCCGWGTGRRTKMCACYWWRACMKRYYR
                *****.*  *  *. ..  ****.  . *****       *     :    *. *** **

3               ARGKRRRYYTRRTSKCAAAYYGGWYYKTWKMWMMWSMYWYWMKAGGCTCATYWRRGTTRR
4               RRTGRRRYYKRRKSKTGGGYCRRACYKKWKMWMMWSMCACTMKTATYWMRKYWRRRYYRA
                 *  *****.**.** ...*     **.*********    **:.     .****   *

3               YRWMMYSSKGTGYRYAWAGTGYYMMAGKRKRYCYYYYCAKWKAKKRAYKAGYYATYTTAA
4               YATMMYSSKSWKCGTGWGKWRCYCMRRKRKRCAYYYYTRKWGGKKRGCKGAYYGCYACCR
                *  ******.     .*.    * *  **** .****  ** .***. *..**. *: .

3               AYTWCASGTYKWRRYSRGGGARKRKKGKKYKWMKSCCGKKTYRGACTKWTATCCGYRAGK
4               GTYTYTCSATKWGAYSRARACRKRKGKKKYKTMKSATATKWYGRMYCKWGTAAGKTRGTK
                .    :..: **  ***. ..****  **** ***. ..* *     ** ::.   *. *

3               TWMRYATTKYRKWYYRMMRGGTKRRTYYKKWYGTGTARMRRYCAGYTYTWCSRKYTSYAK
```

```
4  YACGCRKCKYRKWYYRMMRSKKGRRWYYKKWYTATCGGMAACYWKCWCYAMSRKYWGCWT
         . ***********. . ** ****** :  . *            **** .  .

3  TGRKRYRATCKRYMKKKTKKAYGCACTYRRYRWRGKKRCAMWAATTKKACTWGKATTACT
4  GARKRYRWWMKRYMKTKYKKRCTTGTAYRRYRWRAKKRAGAWGGYCKKWMWWATCCCGAK
    .*****   *****.* **    . :*******.***.. *..  **   *...  ...

3  MTATRRKTRKYTAGCKRMKGTARGGGCMMMRMRRYGGARMRTGYWKMYRRRMMSTAGKGC
4  MKRYRRKCRKYCWRYKRMKACCRKCAYMMMRMRRYAAGAMRYSTWKMYRRRMMSWWCKRM
   *.  *** ***    ****. .*  . ********... ** . **********   *

3  YWMMRKCGATWAGKACGCWKRKRRTTCWWCYRYKARGTAWTGAYKARKMTAAKYYMYKRY
4  CTMMRKTTTYWWAKMYRYWGRKRGCWYWWMYRYKGGACWWCKMCKRRKMKMMKCCCYKRY
     ****  : * .*    * ***    ** ****. .  *    * ***.  *   ****

3  KRKKTAYKYWKCYYGATYKMGKRMGKMYYCACMYRYWMYRGTACAAKRKACCCKKKMGKW
4  TGKKCTCKYWKTTYCRCYKAAKRMKKMYYTCTMYRTWMYRAYMGGWKGKGTTTKGKMKKW
   . ** : ****  *   ** .*** **** . *** ****.   . * *.   * ** **

3  YRRMYRGYWSTCAGKAGKYAAKGTGYYRSYMYYGTCTCCTTRMRYMCGTTKYYACCGCGK
4  YRRCYAKCWSAATAKMAKYWMKKAKTYRSYMYTRWYCGTCCGCRYMATGGKYYTTTRMKK
   *** *   **:.:.* .**  * :  ******           ***.   ***:     *

3  KYRRTYTTSYRRKRKRRYCTGKYRWRGRYCAWCATCRKTRCCACAYAKMRMRMRYRRYCG
4  KYRRYTYKSYRRKRKRRYYYRGCRAATRYSMWTRYTRKCRTTGMGCRKMRMRMRYRRYYT
   ****   .**********    *    **. *    ** *   . .  ***********

3  MRKKYRYKTTWYKYKYRACYYKYYYYSAYWAKKSMKMYWWRYRGCKYKYRMKKYYKRKRC
4  MGKKYRYKKCWYKTKYARAYYKYYYYSGYWWKKSMKCYWWRYRKYGYKYRMKKYYKRKRY
   * ******. *** **  .********.** ***** ******   *************

3  TTKYWYSYCYCCTCAAGYYTTWYKKKKARYCTTMCRWKMKCTCAARAWGGCASKYKSRTS
4  CWKYWYSYYYMYCTGGKTYKKWYKKKKTGCAYWMYRWKCKYWTRCRMWRRMWGGYKSRKS
     ****** *    ..  *..******:  .  * *** *    .* *    . ****.*

3  RSMGTYWKWTTWGMWKGTGAGCRATRWRGACTTRYWRYYTGRRKKRYYGCYTKYMGYRKK
4  AGMCKYWKWYYWRMWKKKTCAGGRYRWRRGYKGATTRYYGARRKKRCTAYCKKYMCYRKK
    .* .****  * *** . ..    *** . .    *** .*****  .  .*** ****

3  WYKMMRWCMMKYRRRYMRWRMYYTKRRATTAKYCYWYKYKKTWWSMKKKGRYTAATKRKR
4  WYKMMRWGMMKYRRRYMATAMYYWKRGWYWRKYGYWYKYKKCWWSATKKCRYCTTWKRKG
   ******* *********   *** **     ** ******* *** .** ** :: ***

3  RKAKKWYKKRYKKTYKKRWAKGTYYCCCCYRWMWCATSMYYMMKMWWKKKRRMKRSCKRK
4  RKMKKAYKKRYKKYYKGGTMKRKYYTTYSCRWMWMRYSMYYMMKMWAGKKRRMKASYKRK
   ** ** ******* **    * .**   . ****   *********  ****** * ***

3  YKYWWRSKKKWMYRGYTKWKRKGYKRYGKGAYRKACAYSRMRRYRRKWTGGWYYRSRWKR
4  TKYWWRSGKKWMYRKYWKWKRKKYKRCTTRMCRKMMMYSRMRRYRRKWYKRWYYRSRTKA
    ****** ****** * ***** ***  .   **   ***********   ****** *

3  TCCRYGCKGKYWKYKYRGACCRYWTAARRTRRCGWMRSRTAKRGAGYRCTARWAAKKKTR
4  GTYRYTAKKKYWKYKYRAMYSRTACGGRRARRMRWMRSRCGGGKWAYRYYGRWRCKKKYR
```

```
                         ** .* ********.  .*   ..**:**  ***** .    .**  .** .*** *

3                     YYRMKSYRSYRMYACGYGCKYSRCTGTSRKTCKMRYKRKRGAGTGYMRYCRKRKCTAGSA
4                     YYRCKSYRSYRMTTYATKYKTSRYASYCRKYMKMRYKRKRRRRYKYMRYMGKRGYWGSSR
                      *** ******** : .   * ** :. .**  ********     ****  **   ..*

3                     YMYYYWMRYTGGMYYYKTYRTYRCGYWRRKYKWSGKRACAAATARRCTWARGTTGCYYMC
4                     CMYYYWMAYYTKCTTYKYYRCTRTAYWGRKYKWSKKRWMWRRCGGRTCTCRRWKATYTMY
                       ****** *      ** **  * .** ****** **      . *   .*  .. * *

3                     CTYWWYRRKKKKMASGTTYGAGKYCYYGCYKKRKRYAYTGKYRWGYTSTAGWRCTMYKRT
4                     TWYWWYRRKKKKMTGACCYKWAKYYCCAYYKKRKRYGCKATYRTRCAGKRRAAMKMYKRW
                        ***********:..  *  .**   . *******. ...**   :..     .****

3                     KYKYRWYYYMMMRKRWMMKRKKSMWRMKRKMCGAAGTCYRYTTTKKYWCYYMRWKCYKKY
4                     KYKYRWYYYMMMRKRWMMKRKKSMWRMKRKCTTGCTGYCRYCACKKYWYYYMRWKTYKKY
                      ******************************   ..    ** : **** ****** ****

3                     GTSYWRKMKKRMYKRKWYKRMYKYCGKRSRYMRYTMTCTAAACCAKYRYMYRRWWSYKMS
4                     ACSTWRKMKKRMYKAGTCKRMYKYMRKRSRCMGYCACTWRWRMMWKYRYMYRRWWSYKMS
                      . * **********    ******  **** * *            **************

3                     YYKRKKCCKKCYRYRKKKRKSKKKKMKRKYKKACMRKYGKKWYWWKRRTRKYYRRKSYRS
4                     YYKRKKTTKKYYRYRKTKRKSTKKKMTAKYKKWMARTCAGGWTTTKRRKRKTYRRKSCGS
                      ******  ** *****.****.****. ****   *. .  *   ***.** *****  *

3                     KGKKKRCYKYYYRYKYMTYKKTCACKMAAAYMGGACYKSKYKGYKWMTRCMTRYCKKYMRY
4                     KTKKKRYYKYYYRYKYCAYKTKYRYKAGRTYMATGTYKSKYKKCKWMCRTMCRYTGTYARY
                      * **** ******** :**..    * . :**. . ******  *** * * **   .* **

3                     YSYCMTTKMYRKMAMCRRMKKTTRWYRKYRYYRCATCMGCKARYYYRMRGRWKWMRYTTG
4                     YSYYCCCKMYRKACMYRRMKKGCGWYRKTRYYRYCAAMAAKGRYYYRMRKAWKWMRYYYR
                      ***    ***** .* *****   **** **** .:.*..*.*******  ******

3                     GAYTYGKKYTAWMTGAMRYKYKRRGWSYMYMWMGTACKTRRMMRYWAGYCMKSWWKYMKK
4                     RGCKYKKGTARWMCAGMGCKYKRGTWGYAYMWCSWRMGAGRMMRYWMRTYMGSWWKYMKK
                       . .* *  : ** ..*  ****  *.* *** .    : ******    * ********

3                     RCGRTKKRKMRYRKRTKKYYRKMTYCAAWRSTCTYYKKGKRYYYYRMMKYRMYTKKYRKY
4                     RYKGGKTRGMAYRKRYKKYYRKMGYSRMWRSAACYYKTRKRYYCYRMMKYRMCYGGCAKY
                      *    *.* * **** ******* *.  ***:. ***. **** ********      **

3                     YMWGKKYRRYRMYMRYKKRKYCTAWKRYSWMRRYYWTYKSYAAMKYRKCCKRRAKKGAYT
4                     YMWKKKYRRYRCTAGTKKRKYYCWWKRYSWMRRYYWYYKSYRGMKYRKTTKRRMKKKTCC
                      *** *******     *****   ************ **** .*****  *** ** :

3                     TGKYWRYWYRYYRMWKYWMMYRRWSYYWCAYRYYWYCTKMRRAKRRKGARKWWRKKYKKY
4                     CKGCWRYWYRYYRMWKYWMCYAAACYYWARYGCCWYYYKMRRWKRRKRMGKWWRKKYKKC
                          *************** *   .***. *   **  **** ****   *********

3                     KYKRKKYGKMTTTTMYMMKYACYKYRMKWGGTGMKYYRKTGRYRWGKRCATTARKWKRKC
4                     KYKRKKYKKMYYYYMYMMKCCSYKYRMKWRACAMKYYRKKKRYGWAKGSWCKMGGATRKA
                      ******* **    ***** ..******* . .******. ** *.* .   .   .**.
```

```
3          GGYYYRKTCGCAGGGAKKMKTYGATCWTRYGAMTTGMSWKYRYYATRMWMASMTRWYMMR
4          AATTTAKYMRMRRRRWKKMKAYAMCYWYGYKRMWKRMSWKYRCCTCRMWMRGMWRWYMAR
           ..    *         ****:*.   *  *  * . ******  : **** .* **** *

3          MTYAYCYYTTYKTTYSAWKYYYKRRRRWYYSATGWKKRYKWKRWRKKYMRYKYRYYYGKY
4          MCYGYMCYGACKAGYSMWKYYYKRRRRWYYSRYKWTKRYKAKRWRKKCMRYGYRYYYKKY
           * *.*  * : *: ** **************   *.**** ****** *** ***** **

3          RRSRKYMYRCAGCAYAGMKYYYRYMMYRRKKRYYYKSRRWRRWKYYRMYMMYYTMYKRKY
4          RRSGGTCYAYMTTMYRRMKCCYRYMMCRRKGRTCTKSRRWRGWKYYRMYMCYYKMYKRKY
           ***    *      *  **  ***** *** *   ****** ******** **.******

3          WYRTKRCTCGTYGATGKRKYYKYWWKAAKYYRYRRWKYKKKSRRKKKMMKKKYYKARKMY
4          WYRKKRACGAYYKCCAKRKYYKTTTKMWKCCAYRRWKYKKKSRRKKKMMKKKYYKGAKMY
           ***.**.  . * . .******   *  *   ***********************. ***

3          YYKKYKKWTYKRKYKRKGAKKYGKMMRKMYYRSRTARYWKKKMCWTCARKWWSYKTCCKY
4          YYKKYKKAWYKRKYKRKACTKYATCAAKMYYGSGKRRYWKKKMYTWMMGKWWSYKCTTKY
           *******  ********...**..   **** * . *******      ******   **

3          YKYMKWWWKGKGRWARTCGYMKKKKRWWKKRMKKYMGRKRGMMCMYYYWGYMRRRGCCCM
4          YGYMKWWTKRKRRWWAYYSYMKKKKRATGGRAKKYMKRKRKMMYCTYYWKYMRRAKTTTM
           * ***** * * **    .*******    * **** *** **   *** ****     *

3          WRYKKTKYRYMYYKKKTSCKTKYYCCARRYKYYSKGYWRKGYMKTKKYKYYKRKMAGCMR
4          WRYKGKKTGTCYYKKKKCAKYGYCYAWRRYKYYSKTCARGRYMKKKKYKYYKGKMTAMMA
           **** .*    *****...*  *  . ********   *  ***.******* **:. *

3          KYTKYYKAYKWRCAKKKYWKYYYWRKKTARARYMYYSRRKSRKMRTMRYRGCYWSTCTKK
4          KYWKYYKWYKWGTGKKKYAKTYCTAKGYWRMRYCYCGRRKSRKCRYMRYRCGYWSCTCKT
           ** **** ***  .**** * *   *   * ** * .****** * ****  ***   *.

3          TTGRMMYYAGGKKYRKWKKKYYTYKWRCCAASWWKYYRKWWKYYGCACTAGRWKWRCTGC
4          YYKAAMYYGRKKKYRKWKKKTCKYKWRGYMGCAWKYYRGWWKCTRTMTCGARWKWRYKRS
                ***.  *********  .****   .. ***** ***       ..***** . .

3          KYYKYSRTATAMCKYYKRATCGYWWYRKYAYRYKARRGYRWMTAWYCAKRKKCAYARRSY
4          KYYKYSRYWYRMYKYYKRMKMRCAWYRKYWYRYKGGRAYRWMWRTYTTKRKKGCCTRRSY
           *******    * *****  .    ***** ****. *.****   * :**** . :****

3          MKTMRGRYGGRKGYYKMYRYKCWRRCMYWTSKSSAKKKTYRRYRWMRGTGAYRARKGTTT
4          AKYMRCRYRAGKRCCKMYRYGMTRRGMYWWSKSSWKKKYTRRTGWMRKYSMYGGAGKYYC
            * ** ** . *   *****   ** *** **** ***  **  ***  . * .

3          TCRSRARYSWWKKRSMYYWRTKRGRCCYYMGYGKKRYRYYKKATCCGRWYYYKKKRKTKT
4          GMGCAWRYSWWKKGGMYYWRYTRKRTTYTMKYKKKRYRYYKKWWYYRRWYCYKKKAKGKG
              .  ******* .***** .* *  * * * *********     *** **** * *

3          KYWRGGMRKRYRWRSYRYRRTTKWYWRYWKYCACYWMMWTKRYMSKKYTWYYKYRYKRYY
4          KYWRATMGGRCRWRSYRTGAWKKWYWRYWGYMRMCWMMAYKRYCSKKYKWYYKYRYKRYY
           ****. *  * ******    .******* *    ***  *** ****.***********
```

```
3          KYKYCMKRYYYCYKRYAGSMTRKYSYKMMKARKYKYMKKKKYWKWKTKRMWSRYMRKMAY
4          KYKYYAKRCTTTCKRYRRSMAGKYSYKMCKGRKYKYMKKKKYWKWKCGAMWSRYMRKMCT
           ****  **     ***  **: ****** *.***************   *********.

3          CAGTKRKSRKWKCYTAATGRYYKRMKKRRTCGYACYRYGGAAGGYMGGRGCKCTTCKYYR
4          MRRAKRKSGKWTYYYMRYRGYYTGAKKRRCYACGTTRYARCGCTYMATAATKTYYTGCCR
              :**** **. *      **.  ****  . .  **. ..  **.  . *       *

3          ATKKRKYRYRMKKKAKRGGATYWRSMKMRKRCCYAKYRYRRWRMYCYRYKMMWYYYGMGR
4          CGKTRKYRYRCKKKWKRCKCKTTRSMKMRKRYSYTTYRYRRWRMYTYRYKMMWYYYCMKR
           . *.****** *** **  ..  ******** .*:.********* ********** * *

3          YRYWWWYRTCTGWRYWKGKKTWWYKKKYCCAKKKGWYRRCRRMCACKTTRWAYKKYWKKY
4          YRYWWWYRWYWRWRYWKKKKYATCKKKYTYGKKGRWYRRMAACYRSKGCRWGYKKYWKKY
           ********    ***** **    ****  .**  ****      .*  **.********

3          RSKMGYYKWWKWYCRYKYYGRKKWKYKKYKKCTTTKYKYKYKTYKYTWKYWKTGKRKMGK
4          RSKMRYYKWWGWCTGCKYYKRKKWKYKKYKKMYWYKYKYKYKKCTTKWKYWTWRKRKMKK
           **** ***** *    *** ***********    *******. . .****.  **** *

3          KRRKYTKYRAGAYKMSRRMKRRYKKGRYAKKKRAKTKRWTAYKKWYYWYYKKCCWKKTMM
4          KRRKYKKYRTRGYKMSRRMKRRYKKARYMKGGRGKCGAAGGYKKWYYWYYKKYMWTGYCC
           *****.***: .*************.** *  *.*     .***********  *.

3          TRYKKKWRCRYMWYTTMRYKKYTYYSGGAYKGKYKARRKKKWKMKGMKKRMKWWKYSRWR
4          WRYKKKWGMRYMTCYAMRYKKTWYYSRRRYKRKYKGRAKKKWKAKKMKKRMKWWKYSRAG
            ******  ***   :*****  ***   ** ***.* ***** * ************

3          TYKYKYYRKRKKMRGKRYYRWSAGRAYKGARWYTCCTRKMRRGAAGWRGWWKATYTYRCA
4          YYKYKYYRKGKKMRAKRYYRWSWRRWYGRRRWYKMSYRKCGARGGAWRRWAKWGCCYRYR
            ******** ****.*******  * *   ***. . **     ...** * *    **

3          TAATYYKCGGCATTCYWGRTRMMWSKKKRRKKTTGTTWYYMRKCRSYKGAGCSKKMRKMY
4          YWRYYYKMRRYTAYTYTARWAMMWSKKGAGKKKWKCCWYYMRKTRSYKATATCKKCAKMY
               ***    ::  * .*  ******   **.    ****** ****.:. .**  ***

3          KYWMYYWMKMYKYRRRKGCTYKSKYKAGTCAYTKRKKTKRRKYAYWKRRKRWRYSWGACG
4          KYWMCYWMKMYKYRAGKSYYYKSKYKRRWMGCCGRKKKKRRKYRYWKRRKRWGYSWKGYK
           **** *********  *.  ******    .   ***.***** ******** *** .

3          GGSRCCMRTSTCRSARGAYMYTAAACWKKSYWYRYGGCAMYRMYYYRRSGRYKKCGSYYY
4          KRSGYMCRKSCYRSMGTCYMYYRTCTWKKSYWYRYAATGMYRMYYYRRSRGYGKYKGCTY
             *    *.*  **   .***  :. *********.. .**********  * *  .  *

3          YKSRRYRRRGGGYMRRYRWYYGGTKKWMRRMSKMRKCYYYRWKKMCYAYGYRTKKYRKSY
4          YKSRRYRRAACRTMRRCGWYYKRKKKAMGGCSKMRKYYYYRWKKMACGYCYRKKKTGKSY
           ******** .    ***  ***  .** *   ***** ********. .* **.**  ***

3          CTGCWYMWRMMRWYCGTAKWGMYMWKARMRR
4          YCCSWYMTRMMRWYYCCTKWKMYMWKGRMRR
              .*** ******   :** *****.****
```

Supplementary Fig. S5. The pairwise alignments between plants 40 and 41.

```
CLUSTAL 2.1 multiple sequence alignment (clustal format)

40              YRYYYYYSCYWKWMKRYMRYWCTKRRCYYRYGAGYRKKYRYMKYRYMRMRYRRGWSRKYM
41              YATCYYYSYYWKWMKRYMRCTTWKRAYYYRYRCTCRKKYRCCGYRYCRMRYRRAWSRKYM
                *   **** **********    **  **** .  *****   *** ******.******

40              RSWMKWYTWGYMMKWYYGGTRRTYRYRWRRYMMKWYRKYYGGTTYWRYRRYYRRYWMRYY
41              ASWMKTTCATCMMKATTTACRRKYRYRWAGTMMKWYRTYYTTAYYWRYRGYYRAYWMRYY
                 ****      ***    . **.*****   ******.**  : ***** *** ******

40              RRYMYSKMWYRTCWWTGCYMYGYWWWYWAWRKYYTWMYSKGARKYYMYRRMCWKYMMRYM
41              RRYAYSKMAYRWMWWYKMTMYTYWWWYWRWRKYYWWMYSKKRRKYYMYRRMMTKYCARYM
                *** **** **  **    ** ****** ***** *****  *********  **  ***

40              KKRWMYWRRRKYMCRKMRYSYYYKRMMRSMYRCMYKCCWSYMYWYRSKYYKRYSYYAWRK
41              KKRWMYWRRRKYMMRGMGYSYYYKRMMRSMYRYMTKYTWSYMYWYRSKCYKRYSYYCWRK
                ************* * * ************** * *  ********** *******.***

40              MRRRYWMYYRRKKWSWKTTYYRYKRYWKWKRYYKRKYMRYGWYKYSWYWKGTKWMRKYWR
41              MRRGCWMYYRRKKWCWGWWYYRYKRTTKTTRYYKRKYMRYKAYKYSWYWKAWKWMRKYTA
                ***  *********.*   ******  * .**********  ********. ******

40              YKKYMWRKRYRGKYMRWRCYMYKRRKATWASMRRSWMMKYMWKMWRARARRYKMGMYRKR
41              TKKCCTRKRYRRTYMRWGYYMYGRRKTYTWSMRRCWMMKYMWKMWRWRTRRYKMKMYRKA
                 **   ***** .****  *** ***:   ****.*********** *:***** ****

40              YYYMMRRAAKACRCMYYRRTTRGMRRRGCRRRYRYMYYMKMMKWWYMYYYYRRCTTRYRK
41              CCCMMRRTGKMYRMMYYRRKWRKMRRRTAGGACGYMYYMKMMKWWTMYYYYRRYCYRYRK
                   ****:.*  * *****. * **** .     *********** *******   ****

40              YRRMRAGAGKTAAKRGWGGRRRKYKRKAKYYRWWYRKYRYCGTYWKKAGYYSATAYKGMR
41              YRRMRTAGAGYMRKRTAKAGAGKTKATGKYYRWWYRKYRYTAGYWKKGATYSGGWCGRMR
                *****:...    **   .   * * ..************ . ****.. **.     **

40              YYKYYKWRRSKKYTYRGMTYKRKKKRYYRYTTRGMYKMKRMKKCGAGRMRKRYTTTKMKK
41              YYGYYKTRRSKKYKYRKMCTTAKKKACYRYAWRRMCKMKRMKKSATKRARGRCCAGKAKK
                ** *** ******.** *  . ***  ***: * * *******..: * * *  : * **

40              KTTCGAYYSRYKYKTTTYYMMRYRRKGGCKCCMRYKCTAWMYAYSAKTGGRRGACARRMK
41              KYCTRCTTGGYKYKWYYYYMMGTGGKRAYKYTMRYKGAWWMCTYSCKKRRRARRMMARMK
                *    .  . ****   ****    * . *  **** : ** :**.*.  *      ***

40              AWCTCWGYAYTKYATRYMYCRTGRYWKAAAMRYMKRCCTWCRYKARYTMRWWAYSRMSYK
41              GWYCATKYMYYKYTGGYAYMRWRRYWKGRWMRYMKRSMYWMRYKMRYCMRWACYGRMSYK
                .*  .  * * **:  * * *  ****.  ******.  * *** ** *** .*.*****

40              RRYKYKRTTWAKKKKKMCKTCKMRRRKYARGGTKARTKRKKYWMRWACACKYYKKYYMYY
41              RRYGTKGGYWGKKKKKMYKCTKMAAGTYRRKTCGCGWGGKKTTAGWGYRYKYYKKYYMYC
                ***  *   *.****** *  **   .* *    .    **    *.   *********

40              SRYYARTAYKKKMYRRRKSMKGTRRRRRAGYATKKKYKYYTAYYKKMWKYKRTKRYKMAR
```

```
41          SRYYTRGMYKKTMYARRKSMKKGRRRRRGAYGCKKKYKTYYRYYKKCTKYKACKATKMRR
            ****:*  ***.** ******  *****..*. ***** *  ****  ***  *  ** *

40          MSYKMYSKRYWRKYWKRYMTKGTKKKKRTKKYRKMRKRTGRKCSKTAYKKKRYKCCMKYM
41          MSYKMYSKRYWRKYAKRYMKKKCKGGKRWTKYRKMRKRAARGYSKYWYKKKRYKSSMKYM
            ************** ****.*  *  ** .********:.*  **  *******..****

40          KYKRMRRKRRYKGAAKRYKYYRKWKKKRMKRCTTGGKTYRCKCCCCKKKKTRSKKSRKTR
41          KYKRMRRKRRYKKRWKRCGYTAKWKGKRAKATCYKTGCTRYKYSTMKKKKYRSKKSAGYR
            ************   **  *  *** ** *         * * .  **** *****   *

40          AGKMKTTTKSRYKSYWYKWSMMMYYKYYGYMYKRYYTTRKWRRCGCMWWKWWYMKRYKKY
41          CAKMKCAATSRCGSYWYKWSMMMYYKYYKYMYKRYYYCRKWRRTKTMWWKWAYMKRYKKC
            ..*** ::.**  *************** *******  *****   ***** *******

40          YYCGTCGTCGWRKWSRWSYKRMKYYYRYSKKRYKRYRRKWYRMKKKYRGCRRYKKKKMTK
41          TCGTGTAWMKWRKWSRTSYKRMKCTYRYSTKRYKRYRRKWCAATKTYRSMRRYKKKKMAG
                  .   ****** ******  ****.**********   .*.**. ********:

40          GTMMYCAAAKKMMKRATCCWGTRMKRKRCCGRSRYKMWTMWWKGTTYKRWKCCTKKWRKY
41          AWAMYYRMWKKMMTGMWYYWTWAMKRKRYYRRSRYKMACCWWKRWYYKRWKMYYKKWRKY
            .  **    ****.     *   *****   ******   ***   *****   ******

40          MKACYRKWWRKMRRYKRKYKRKKYKWTKCTTCTKYCKRYKKMAMCKGYYYYRKKYRKRKK
41          MKCTYGKTTRKMRRYKRKYKRKKYKWYKYAAMWGCSKRYKKAGCYKKYYYYAKKYRKRKK
            **. * *  ***************** * ::    .*****  .  * **** ********

40          SWYKRYYRSYYYRYYKWKWWYMYYRKMMWCKMWWYSRKKKKKWRYRKSRMKWSKYSKMKK
41          CWYKRYYRSTYYRYYKAGWWYMYYRKMMWYKMWWYSRKKKKKWRYRKSRMKWSKYSKAKK
            .******** ******  *********** *************************** **

40          MMMSKKKRWYRKMMRRRAATKKYKYACYCYRKYTKYYMYYWAKCKWRKRKWRMKKCMKYW
41          MMCSKKKRWYRKMMRRRMWYKKYKYGYYYYRKTCKYYMYYTGTYKWRKRKWRMTGMCTCW
            ** **************   *****. * ***  ****** ..  *********.   . *

40          MWKMYKKYYMKKRWAACTYMCSWAYYCWMRRGGYSKMMWSYKRYTCAYKKMMCYYTAWMR
41          CWKMYKGYYMKKRTRCTYYMYSWMYYYWMRRKACGGACASYKACYTGYKKMAMYCYRWMG
             ***** ******  .  ** ** ** **** . .    ***    .****  *   **

40          KYRCKAKYRMKTGRACMTAGGWYRCKYRWRYKKKMRYKKKWYRTYRRRAYYKWTGGARTA
41          GYRTTGKYRMKYRGRACWWRRWYRYKYRWRYKGKMRCKKKWYRWYRRRGYYKWKRRRACG
             **  ..*****    .     *** ******* *** ****** ****.****.     .

40          TYYYKRWSWRRGMGWYMKCRKKMMRGKYGKMRYMGRRYYMYMYKKYRYWMYYYYRYYGRR
41          AYYYKRWSWRRKMAWCCKYAKKACAKKYRKMRTMKRACYAYMYKGYRYWATTYYRYTKRG
            :********** *.*  *  **    ** *** * *  * **** ****   ****  *

40          YYSYRTCAARAAMAGCRYMACAYGWWMKKYKWMRRWKKWWYGGCYGKKKKRYYYRAAKKK
41          TCCYRWAGGACTMRSYRYMTMCYTWWAKKYKWMRRWKKWWCARTTAKKKKRYYYRCCKKK
              .** ... .:* . ***: .* ** *************  .   .*********..***

40          RCTMRKCCYYRYMRRRYRKKWTAWYRGKTRYKKKMAWWRKMKKRKAGCWMYYYRSRRKKK
41          RYWCRKMYYYRYMRAATAKKWCGWYGRKKRYKKKMTWWRKMKKRKMKAWMYYYRSRRKKK
```

```
                  *   **  ******    *** .**  *.******:*********  .************

40                YWWARYKRSMWYRMRRWKMWYKRYYMKKKRKKKKYKSRYWKRRRMYWRKRYKRYKRKWAT
41                YWWMRYKRSMWYRMRRWKMWYKGYYMKKKRKKKKYKSRYWKRRRMYWRGGYKRYKAKWTA
                  *** ****************** *************************  ***** **::

40                AYWRKKRKKYCRYSCWWGKKAKYKKMYKWYMYRRKMKYMYCKTTCTYYKWRYKKRWMRRK
41                GYWRKKRKKTTAYSATTTKTMTYKKMYKWYMYRRKAKCMYSKAAGAYYKWRYTKRWMRGK
                  .********   **.   *. .************* * **.*:: :******.***** *

40                TTKKRGGSMRYYYKKKWRGWKRYKWCGCGTYKKSKMMSRKRKAGAAGKKYKKKKKYRSMR
41                YWKKRRRSMACTCGKGWRKAKGCKWTKMKKYKKSKMMSRKRKRRMRAKGYKKKKKYRSMR
                    *** **     * **  *  **    .************    .* ***********

40                KMYYKRSYRRKKKSKRYKRRKYRKMMKMYKGRKYRMMRYKKKMSKYYKCYCCRSKYYRYG
41                KMYCKGSYRRKKKSKRYKRRKYRKMMKMYKKRKYRMMAYKKKMSKCYKMCYSRSKYCGYA
                  *** * ************************ ****** ******* **   .****  *.

40                MKCCCWMYRKKKYRYKKKYTTTGWKYWYYRGCTGTMGSKMYYKWRYMWGRKMRYYMTAGT
41                MKTMMAMYRKKKYRYKTGTGWCTWKYTYYRRYCAYARSKMYYKWRYMWTGGCGYYMAGTC
                  **    **********.      *** ***   .   ***********     ***:.

40                AKARRWRGKWYRTSCKMKRRMGRCWGCMRWRYWWGTRKTRWKKKWGGGKMKYKRKYYMYY
41                GKRRRWRKKWTRGSYGMKRRMRRGTTAMRWRYWWRKRKGRWKKKWRKTKMKYKRKYYCCC
                  .* **** ** * *  ***** *   .******* .** ******   *********

40                CTYKYMRWRYRRWRKTCGCAKKWYRYRKRRYRRTTCTCKGSGRYRKRAMKYKKWWKCGYR
41                TKCKYMRWRYRRWRKKMKSMKKWYRYRKRRYRRYYYYYKACAAYRKRRMKYKKWWKTTYR
                   . ************.  . *************     *... **** ********  **

40                MYSYCGTGMRRKKCKTYWYKWKKYKKWCRWKSWGWYYWRKGGCGTATYMMRRTMKYWRMY
41                MYSYYRYRMRAKKYKGYWYKWKKYKKWTAWKSWKWYYARKRKMTAGCYMMGRKMKYWRMY
                  ****    ** ** * ***********  **** *** **    :. *** *.*******

40                RYRGGKKRKYKGAKYYAKKRKYYRWARATWYMTYYMYRMTCCRRMMCTWRKKSGYKRACG
41                RYRRRGKRKYKACKYYMKKRKTYRWRRWWWYMCYCMYRMCTTRRMMTCWRKGCACKRTTA
                  ***   *****..*** **** *** *  *** * ****   ****  *** .. **: .

40                RSMKYKTCRYYAAKMRRYYKKYWRYSYRKSGWMKYYRRTTMTRYTTGTGACWYKYKRRRR
41                RSMKYKCYAYCRGKMRGYYKKYWRYSYRTSSAMKYYAGACCGRYCCAKKCTWYKYKRRRR
                  ******   *  .*** ***********.*. ****  :   **  .. . *********

40                YWKKYYRYKSRKRWMRRYKTCAYYKKAYMKYYRRYWRGYKMYYMMTKKRTTCCCMYCKYR
41                CTTGCCAYKSRKRWMRRYKWYGCCKKGYMKYYRRYWRKCKCTYCCAKKRCAMASMYYGYA
                    .    ************  .  **.**********  *  *  :*** : ..**  *

40                YRAWYYAGCGKKKKWRRMKMGSGKMMKGGCKCCKGCCMCRKWRWRYRWKWTTTSYWRKKM
41                YRGTCCGSYRKKKKWRRMKMRCKKMMGSKYKTYKATTCTRKWRWRYRWGWKWWSYWRKKC
                  **.   ..  ********** . *** .  *  *.    ********* *.  ******

40                RRMRTKGCMYMKCTKWWSKKYWYRGYKYMCWYYMCYCYGKRSSSRRYMMMYRYYKKWGCG
41                RRMRYKTTMYMKSYKWWSKKYWYGSYKYMYWYYMYTACAGRSSSRRYMMMYAYYKKWATA
                  **** *  ****. ********* .**** ****  . . *********** *****. .
```

```
40          GGTTAGRYRKKTACWKGMAYKRWYRGKYMMRRTRGCGKKMKKMGWATKWRRWRYYMTYWR
41          TKAGGARYRKKYGMTKRCRYKRTYRSKYMMRRCATTKKKMKKCTAGKKWRRWRYYMKYWR
              : ..***** .  *   *** **.******     *****   ..*********.***

40          KTAGCYTCMYWYWKKRKKYKRKKMKYSYRKKYRRWMYYYKMYRRGKKGGKYGKYYRWMMY
41          TAWAMYYMAYACWKKRKKYKRKKMKYSYRKKYRRWMYYYKMYRRRKKRRKYRKYYRWMMY
            .: . *   *  ******************************** **  ** ********

40          TKKWCKRRWKTYTMMTKRGKYYYKTACCCMWYTGWYSRRYYKYKYKWKWATCYYYYCYRY
41          YKKWYKAAWGCCCMMAKRTGCCCKKGTAACACYRTYSRRCYKYKYKWKARCACYYYMYRY
             *** *  *    **:**     *.. ..      **** ********   . *** ***

40          CSWRRYYGKMRYRGRYYCYAWGAMTTTAKYMYYMYMSRSRMMYMYKWTAARYRCCTKRMR
41          YSWRRYTAKMRYRTGYYYYGTTGMGACGKCACCCYMSRSRCATACKWACGRYATTGKRMR
             ***** .*****  ** *.  .* : .*     ******     **:..**    ****

40          YKMTAMYSRRSCKKRRYKKWMRRMRYGTCWCYYMYMKKSYKYWCTYKCTRRWTAAKWWRR
41          YKMWTMYSRRSMKKRRYKKWCRRAACCCTWMYYMYMKKGTTYAMCYKTCGRWKTWKWWRR
            *** :****** ******** **        *******. .*   **   **.: *****

40          YRWMAAATYYYGTKMRWYRRWGATSRRTKCCKRKYYYYTGYRYYKMTGAWYKMYRYMRYM
41          YRWMRWRYYYYRCKARWYRRTCGWSRRYGYYKRKYYYYARYRYYKMATGWYKMYRYMRYM
            ****    ***  * *****  . ***    *******: ******: .***********

40          WKKRYYYKWCRMYKKYKMGKYYYCYRKRRYGCMMMKSMYMYRMYCARWRKKKYKKRGYRW
41          WKKRYYYGWMRMYKKYKCRGYYYYYRTRRCKYMMAKSMYMYRMYAWGTRKKKYKKRRYRW
            ******* * *******   *** **.**   ** *********.   ******** ***

40          WKRMRKWYRRKMRRMMKWYYTKGCGGMMMKCKKKKRRACAMRSRWKYKRMKSCRYYWKCC
41          WGRMRTWYRRGMRRMMKWYYKKAAAAMMMKYKKKKRRWMRMRSRWKYKRMKCSRCCWKTY
            * ***.**** *********.*....**** ******   ***********..*  **

40          GKYKKKKYTYSRYYTAGTKYRRYSCYTACAMGRRTYRSRRYKAKYGTAYWWRYRGGRMKY
41          AKTGKKGYATSRYYKRAWKYRRYGYTYRYWCRRRGYRSRRYKGKYRKRCWWRYRRTGMKY
            .*  ** *: ****. . *****.        ** *******.** .  *****   ***

40          KYYYWMAGMKRYMYKYKRYRCKTWYYYRYMKGCKKRMGRKKKYCKRRMYTYCCKRWGTMR
41          KYYYWAMAMKRYMYKYKRYRMKCWYYYRYCGRYKKGCRRKKKCGKRGMYACTTKRWTAMR
            *****  .************ * ******    **   ****  ** **:   *** :**

40          RRRYGYGAATMCATAKRAGACRGKMYYYYRYYMRCTYAGWCGTRWRRGMYCTYCTRWMGK
41          RRGYKYTWMYCAGCTKRWRCYAAKMYYYYRYYMRTAYGAWTKKRWRRSMYYWCYWGTMRK
            ** * *     .. :**  .  .*********** :*..*  .****.**       * *

40          KAAAKYAWKWMYWYRATCMGCRKKMWGYKCGYYWKKCTWTYRAKKMAAYRWMKKRKRRYR
41          KWGTKYTWKWMYWYATGTMATATGCTTYKSKYYWKKGCWKYRMKKMRWYRWMKKRKRRYR
            * .:**:******* :  *.  .    **. *****  *.** ***  ************

40          YRYKYGKYKRYKYRTRRYWMKRMWMKMMAKYMGGGYACKWSRRKRCRRRWKRRKKKYKWT
41          YRYKTRKYKRYKYRKRRYWMKRMWCKMMMKCMKRRYMYKWSRAKRTRRRWKARKKGYTTC
            ****  ********.********* *** * *   *  **** ** ***** *** *.
```

```
40      YWRRWKYKKYTCWRKMRGGKMKSYRWAYGTYRTRYKRRRMSRYYYYWYCCACYKARYTGT
41      YWRRWKYKKYYMWRKMRRKKMKCYRWMYAYTGYRYKRRRMSRYYYYWYSMGTYKGRYYTW
        **********  *****  ***.*** *.    ***************. . **.**

40      RWYWRWKMSYGSSKKWWKWSAGCARMMYRRAYYYYWRWRGMTGCKYMYYKKRRKYKKATT
41      RWYWRWKMSCRSSKKTATACTTTRRAATGATYYYYWRWRACCATKYMYYKKRRKYKKWKY
        *********  ****  . .:   *     :********.  . ************* .

40      TAWRYYYYYYKRKSTKKKRSKKKYYKKKCKWKYRRWYRRAKRCRMCCKKYSMKRRRYKYK
41      KWWRYCTCTYKRTCKKKTRSGKKTYKKKMKWKYRRWYAARKAMRMATKKYSMKRGAYKYK
        . ***    ***...**.** ** **** ********   *  **. *******  ****

40      KWYKYYYCKKWMYYMWYGYKKYRKWWAGGRMWTMRYGKCCKTKAKKGGAMKYMWCKRGKR
41      GWYKYYYSKKWMYYMWCRCGKYRKWWTAAACAWAGYAGGYKYKWKTRAWMKYATYGRAKR
         ******.********    ******:..      *.   * * *. . ***    *.**

40      RKSRYYYWWTCTCCWAWKKKRYMMKAYCKTWYWKKKKKWYKKWAGCK
41      RKSRYYYTTAACYMWTWKKKGTMMKTYYKYWYAGKKKKWYKKAGATK
        *******   :.  *:****  ***:* * **  ******** .. *
```

Supplementary Fig. S6. The pairwise alignments between plants 38 and 39.

```
CLUSTAL 2.1 multiple sequence alignment (clustal format)

38              YKYYRRKRKYRMAMWKMYRMYYAGTWSRKRGTAAGKMRYRYYRCCGGCCMYYKYRWRKAA
39              YKYYRRKRKYGMRAWTMYRCYYGACASAGGRACMKTCRYRYYRMTRKMTATCKYRWRKMR
                ********** *  *.*** **..  *    :.  . ******         ******

38              RRRRKRWWSRMKMYKKYYRTKYMRYTTGGGYYMKYKYWMWRCGMGCYMRYAMYRSYRKWR
39              AGGRKRAWGRMKMYKGTCRYKYMRYAYRRKYYMKYKYWMWRYRMRYYMRYGMYRCTRKTA
                   *** *.******   * *****:    ***********  *  ****.***. **

38              MRYWRKMGRYAGARYWKYMRYSYMGTTARRCMAWMATKRRRRYMYMWCCSKYRRMTTRRY
39              MRYWRGMTRYTATRCWGYMRYSYMRCCGRRMMRACWYKRRRRYMYMWYYGKYRAMAGRRY
                ***** * **:.:* * *******   .** *     **********  .*** *: ***

38              YCAGGKYWRYWWCCYTMMTCGTKMRKRKGMMYYRKMYTTCRCWYMWRATYMAAGYYWMRW
39              YACKRTTARYWAYYYYMMCGTAKMRKRGRMCTCGKMCYKTGTTCATAWWYMWGAYYWMGA
                *..  .  ***   * **   :*****  *    **  .          ** ..****

38              WYRKWRYRKWRAYKCMGWYWRRYRYRKCWMRYYRWCMRRWWMRRRSYYYRRKKRRRRTCY
39              WYRTWRYRKWRRYKMMSWYWRRYRYRKAWMRYYRWAMRRWWMRRRSTTTRRKGAAGGKTY
                ***.******* ** *.**********.*******.**********   ***     . *

38              RYYWATWRWTWKTTAYMMMMMRKTYRRRYMGKRCRMKYKYRWMYRYGRMMYWWSRRWSRK
39              GTYWWYAGTKWKWWGYMMMMMRKYYRRRYMRKRMRMKYKYRTMYRYAGCMYWTSRRWSAG
                  **     .**  .******** ****** ** ******* ****.  *** *****

38              WMMYAGYRRTCYRWRYRWYYYWTWWMYMRMRKKRTTCWGRSTRYRKSYAYTGGGTCAWAW
39              WMMCRKYRRCTYRTGTGWYYYWWTWACAAARKKGYAAARRSYRTGKSCGTCATRYMWTRW
                ***   ***  **    *****  *     ***  :.  ** *  ** .  .       *

38              KRWTRWCYWYRKYSWYTGGRYWWYYARYRWWMKKRYYKKKGGYKYMTYRKSWKKYYYKRR
39              KRAYRWYYWYRKCGTYAACRYAATYRAYRWWCKKRYYKKKKKYKYMKCRKCAKKYYYKRG
                **  ** ***** . *:. **   *  **** ********  ****. **. *******

38              GYMYYRTTWRYKYKMCMKSSKMYRWRGYRYKYWYRWKMYRYKYTARKKCAGKYKRWRYKR
39              KYMYYRYCWRYKTTCYMKSSKMYRWARYRYGYWYRAKMYRCGYKMRKKSCKKYKRWRYKR
                 *****  **** .  *********  *** **** ****  *. ***.. *********

38              YKRWMYYRKKAGGTYKKRYYMRRKSKARGGAGRAYRTYMYWAYKRYMRYYKRKKAWTWKS
39              YKRACYYRKKRRRWYTKRCYCGRGSKGRAKTKRRYRKYMYWGYGRYMRYYKRKKWWKWKS
                ***  *****    *.** *  * **.*. : * **.****.* ********** *.***

38              CKTSYKKTWCGSYTKTYRRSRWYKYKYRMSRWWSRRWKMYKSRWYYGYCKYSYMKMCATC
39              YKGGCKKKWYRSYGKGYRRSRWYTTGCAMSAWWSRRWKMYKSRWYCTTYKYSYAKCARYM
                 * . **.*  ** * *******.    ** **************    **** * .

38              ARYATRKRRTCTRWRYGRWYGACTMMWKKTTKKKKKYGWGRKRTYRRYRYYWGCTGGCYR
39              RRYRYRKRRKSKAAGCKRWCACAGMCTKTACKTGTGCKTRAKRGYRGTRYYATTGTKMYR
                 **  ****...     ** ... *  *.: *. .      ** **  ***       **

38              SYGYGTKAMCWMYKKMYYKAYKKAKYRRYKRKMWKCGKTTYYWYTACCTYKYKKGTCRYK
```

```
39      SYKCKGGTCYWCYGKAYYKMYKKMKYRRYGRTMTKTTGYYCYWYWRTMKYKYKKTCYRYK
        **     :  * * * *** *** ***** *.* *      ***    .*****   ***

38      RYKYRKKMYTKGRRRKYMTAARWKRKKYRYYRCRWSRYCCKYYRGRMGRKYKYRYWKKKS
39      RYKYRTKCTKGSRRRKYCCCGRWKGGKCGYYRYRWSRYTTKTCRARMTRKYTYRYWKGKS
        *****.*  . .*****  ..***  *  *** *****  *  *.** ***.***** **

38      MYYRRWGARTARGTAGGCACRKAGKTYKKKGKCTRAKYMRMKGKKKYWTCKRCKMYMKTG
39      MTCGRARMACCGACGAATTYRKWRKWYKKKCTTKRGKYMRMKKKKKCTATGRYKMCMKCR
        *   *     . . ... : **  * **** . .*.****** ***  :  * ** **

38      GTAKKYWKYGGSTRRYYTGWARAGTTTWMSKAMKAWYCTACKYRCMCTKRARYCGYCRKR
39      ACRKKCAKCRACARRYYGRTWGRRWYYAMSTGCGMWCMCGYKYRTMYWGRGRCTRTYRKR
        .  **  *  ..:****           **..   *   . *** *   *.*     ***

38      YKGKTCARMRKTCYRRGCTKRWGGWATGKYWYYYSYYKCGTGYRMMWRRWYYRRKWKWKA
39      YKKKGTTRMGGKYYRGAACGRWTRWCGRTYWYYYCYYKMRYAYRMMTGGTCCRRKWTWKM
        ** *  :**  . ** ..  **  *.  .*****.***   .****      ****.**

38      YGYTWRRCRRKRKYYMKTYGGGGTAYMWYRMKRRRYRWCCCGRRMRRSKYYYSKRKGGSR
39      YCTAARRTAAKRKYYMKYTRSAKYGYMWYGMTRRRYRATTTTRRMRRSKCYYSKAKTAGG
        *  : **   *******   ..  .**** *.*****     ******* **** * ..

38      YRYYCGWKYMRCCTGSYWKCYKGTGGKCRKGYCKKSRKMRWWKGCGGYAWKYMWKWKRYK
39      YRTYYAWKTAAYMKKSYWKMYKRWRRKTATTCTKTSRKMRWWKATARTRWKYMWKWKRYK
        ** *.**     . **** **    *  .   *.********. .   ***********

38      AACCYTGKYGYTTCGRMACYTTTKYGYRMRCTGAATRYRRAGAARGWKTGAGKYKKYYGT
39      RRAAYYRKTAYWGTARMGTYCWCGYRYRMRAATMGCGTGAGTTGRKTGWKRRKCKKTTAG
          ..*  * .*   .**. *    * ****.:  .     . :.*       * **  .

38      AKMCYKCGCMKAAGGAYCYYGYKRAMWCKYWRYKKARRRKCACRCCTCRRKRCKAYTGAK
39      GKMYYKMAACTRGRTRCYCCRCKRGMAYKYWGCKKTGGRKYGTGGTCTRRGRYKTTGATK
        .** ** .. . .         **.*  ***  **:  ** .      ** * *:  .:*

38      RYTTACYCKSTKRKRTWYCRGWGYKRTWKYRSKYKKWYMYYKRMWKKRRWRKRKRRGGCT
39      GTWWRTTTKSGKRKRKWYTRSAAYKAYTKCACKYKKWTAYCKRMWKKRGARKRKRGRATC
                ** ****.** *. .**   *  .*****  * *******  *****  .

38      RRYTGAGYRMRGWAGGSKWSKYRCYYYMMRKRMACGCAKKKCCWCYACTKKYRRMKKTAA
39      RRCARTCYRMRATCTASKWSKCAMYYYCMATAAWYATGKKKYYWMYRSWKKYRRMKKCCG
        ** : : ****. . .*****   *** * .    . .***  * * . ******** ..

38      ATYCAAWCGCCRRACCTGGGYTGCGYTGGWRATYRTWTGKWKRMKKGGYSTWYRYRRRYA
39      RYYATGTATTTGGTTSYSARCYATRCYATTGCKYGCWGKKTGRMKKCRYCWTYRTGGRYR
          *.:. .     : . ..   .    .   ..*  *  *  ****  *.  **   **

38      RWRMMRGRRKKGTWRKYRRACYGYTKYMRRRYRRRYKKYKRGRYKWRWMYYKTRTACTMT
39      RWRMMRTGRGKRKWRKYRRTACATWGYMRRRYRAACTKYGGARYKAGAMYYKKRGRYAMW
        ******  * * .******:. .   *******   .**  .***   ****.*   :*

38      TRKRWKRRSYGRKGYTGTYYCGTACGGAGAARKATAKYWSSYMKYWKKGYWTWWMGKWKT
39      KRKRWGRASYCGKKTYAKYYYRYCTAAGTCWRTRYWKTASGTCKYWKKCYWWWWMKKWKK
```

```
                    .**** * **  *    ..**    . ... . *.    *  *.  ***** ** *** ***.

38                  CCKYRYYWWKYYRMRWKSGYKKTCKYYKYRYKYRYKMYKMRRWWMSSYGRMTKRYASRYY
39                  TTTYRYYWWKYTRMRWTCKTKTWYGYYKCAYKYRTGCCGCRRWWMCCTRGMCTAYRSRYY
                      .******** ****..  *.    ***  ****      *****..    * . * ****

38                  WKYRKMKKYCKMCCWMGTWTKWGYKKRGRKMYYGRYTTKYKRYRATAAWKMKKTTYRKKW
39                  WKYRKMKTYYGCAMWCKCTKTAKYKKGKAGMYYKACACKYTRYAGCGGWGAKKWWYRGGT
                    *******.*    . *     ..   ***     ***    : **.** . ..*   **  **

38                  WAMYKRRYKRYKTCCMKYYKRRKGMGKYKKKWRSYYYMYYKKKKYSKRYKGWTAMKKKYS
39                  AWMYTRRYKRYKWYYMKYYKRRKCARKYKKKWRSYYYMYTKKKKYSKRYKRWYRMKKKCC
                      **.*******    ********    ************* ********** *  ****  .

38                  KGKYYRKKWRGTKYKCAKRYKKRKTWCWYWKRCRKAAYRMKWRWYAKRYRKYGMMWTKKY
39                  GKKYYRKKWRACKYKMWTAYKKRKKWYWYWKGMGKRWYRMKWGTCTGRYRKYRMMWCKKY
                      ********. ***   . *****.* ****    *   *****    : ***** *** ***

38                  RWRKCYAGYMAYRRWKKTKTKMAGYRYMRWYWKMTATRWYMRYRKCGRCRKSYKKWYYTT
39                  GARGMCCTYMMYRRWKKYKCTCRACRYMRWYWKCAGCRTYMRYRKYRRTRKSYKTWYYWW
                      *    . ** ****** *  .   . ******** :. * ******   * *****.***

38                  RWAWYKTYRGGYRRKKYKKYRRKRCTCCCCKCKWKTGMKYWWRKARKRRMKKYMRWWCRW
39                  RWWTYKWYRRRCARKKYTKYARTRYYYMYYKYTAKYRMKYWWRKCAKRRCKKYMRWWTRW
                    **   ** **     ****.** *.*      * . *  *******. *** ******* **

38                  KRYKRMCRSCTMKGCTTTRMCTTKSKCACGKYYGGMCYTRRMKKYYYMRWRRMYRKCTRK
39                  KRYKRCYRSYWMKTTAAWRMTCYKSKYRYRKYYRRMTCGRACKTYCTARWRRMCAKTGGK
                    *****   **  **   :: **    ***      ***  *    *  *.*   *****  *   *

38                  YRYMKKRRCCCTWWTKAAKTRCWCRTYTARKWAYGGKRWKWWRMAWCCSYYYGRYTTTAK
39                  TRYMKKRRATTGTTYGTGGCGTTMRCTCGRKTWYKKKAAGTARAGWTYCYCCAGTCGATG
                     *******.        :.       *    .**  *  *      * .*  .*  .     ::

38                  RWGKATYKWYTSKRTMCTCAGMYRCYTTGMKYYYSRKMKYGCAAWGYMKKMWYYMTCCCY
39                  GARKTCCKTCCCKGWCYYMMRMYRYCYWRMKYTCGGGCKYRTTGTRYCTTMWYTCKMMYY
                       *:  *   .*          ***      ***   .    **  :.  * ..***   .   *

38                  MYRRRTMYGMMKMKMSMKKYKKMRKMKCTKCAAGACAYCKKKCKKYYTMACTKYRSYCYK
39                  MYRRRKMYRMCKAKCCAKTCKKMRKMKMYKYWMRRYRYTKTKMKKYTWAGYYKYGSYYCG
                    *****.** * * *  . *. *******  *          * *.* ***     .   ** **

38                  YTCYRCYKCRTAGTYYYRMKKKAAAGRKYCGKKWRRKKYMRTAKWTAKCSYRRKWMKWWM
39                  TYYCGMTKTAGTACYTTRMKKKTTTTRKCYKKKWRRKKYCAYRKWKWKYSYRGGTAKTWM
                           *    :. *  *****::: **    ********     **. * ***     * **

38                  GKTMYCTCRMKAYRYCKSRWKRKKAKARYYRKSKKKRRKKYYKKKKGTRMRGCCGTCAKY
39                  RKKMYMYYRMKTCGCYKSRWKRKKMKRRYYRKSKKTRRKKYYKKKKCCRMRRYYRYGGKT
                     *.**   ***:     ******** * ********.**********  ***       .*

38                  YRKYRMKWTAKKKTTSYMYYKKWYRAGTCAAGKKRYSCAYMGTYTTGTAMYTCTSRKKYT
39                  CRTYRMKWCTKKGCCGCMYYKGWYRWAAAGGTKKRYSYMYCKWCCWRYTCCAYCCAKKYC
                     *.***** :**    . **** *** .:... *****  *         :  :  . ***
```

```
38          RAASWWCMMACGYSMWYRKTRTRYKRSKTRRRYRCKWKKGMRYGRRYMKYWKYKGTGKWR
39          RTGSWWYMMRTAYSMWYRGCGCRYGRSKCGRRTGYGWKKCMRYKRRCCKYWKTKKKKKWR
            *:.*** **  .******    ** ***  **    *** *** **  **** * . ***

38          KRGTYGGYYYYKRARYYRRRCGCTACTCYAGCTTYGCWKGGAAGWRKKKSMYWTKRTTAY
39          KRKGYKKCYYYKRTGTYRRRTTAGGTYTYMRTWCYRYTKAATGRWRKKKCMCWKTRYCTY
            **  *   *****:  ****  . .   *     *   *..:. *****.* *..*  :*

38          CTTRMYWWKYKRKYMMRCTRRATRWKWMYYYWMWSWGYCTCYRSCCSSYRYWKYWWSKAK
39          YWYRCCTTKYKRGYMMRYWRGGCGWKWMYYYWMWSWRYMCTYRSAASSYRYWKYWWSKRK
               *    **** ****  * .  ************ *   ***..************ *

38          KKYGTATYRCCYRRKRWYRARYKGYMMASRKMAGTCYSYKKTTYMYKGKRAGMYKTTAAC
39          KKCRYTCCATAYGRGRWYRGGTTKYMMRSRKMGACTYSCGTACTMYKKGRMRMYTCCGGT
            **   :    .* * ****.  . *** ****..  **  .:  ***  *  **.  ..

38          KKYYKMKKCGTAARKKKKKGCGCRSYWRKGKKKTCAKKKYRRMKRYWKKRSCARTCYYYY
39          KKYTKCKKSRYMRRKKKKGTTTTRGTTAKRKKKAYRKKKYGGMGRYTKKGSYTRGTYYYY
            *** * **.    *****     *.   * ***:  ****  * ** ** * :*  ****

38          CRGGGCCYCYCTMARWYGACARWKKKKRMYYYWKKKKKRYRRRRAARWKRWCKMMKRYYM
39          YRKKAYYCYCYWMGATTKGYGRWKKKTRMYYYWKKKKKRCGGRRMWRWGRWAKMMKRTCM
             *  .       *.    . .*****.************   **  ** **.*****  *

38          KKAGYAKRMKYKMRKYKYAKRTGTKRRKSTGTATRTKGSKYYCCMKRWMSGCMKWKSACT
39          KKMRYMKRMKYKMAKYKCRGGYRWGGAKCYRWRYGYKRGKYYYMATAACSKYMKWKSGTA
            **  * ******* ***          *.       * .***   .   *  *****. :

38          TGYKYKGGYYYYKKKCKTWSKYKRKYSSKYWYGAGYRYKYYRKYCKKWRRKWRWCRTCRR
39          GAYKYKKRYYTTGKKTGCTGKYKAKYSSKTWYRRKTGYKYYRKYSKKWRRKWRATGATGG
             .****  **   **    .*** ***** **     *******.********   :

38          ATTKRRMYRKCRKWKCGWYRCKKKKAGAWYRYYGYYRYGCYRKYKKSWGRCKGKMRKKTG
39          TGCKGRMYRKTRKWTTAWYRTTTKGWSRTCGCCAYYRYTTTRKYKKSTCATTAKMGKKWR
            :  * ***** ***. .*** ..*  .      .****   ******    ..** **

38          RKMRRSYKRYRRTYYWYRYGTKTYATAYSYAATKYRKGRYKYKRYRRGKKYKMRKKCTWM
39          RKCARSYGRCARAYCWYRYRYKCTCYGCGYWWWGYAKARYGYGGCRGAKKYKMGKKTAAM
            **  *** *  *:* ****  *  . . .*    * *.** *   * .***** ** : *

38          RRYRKTYRYWKMRKYYKMTRYWYKYKYRGRKRYMARRATYGYTGAKTCGCRGCKKRWKYY
39          GACATGCGCAGMRKCTGACRYWYKTGYRARKRYMGAGTYYRYKRWKCTAARAYGKRWTYC
                .      ***     *****  **.*****.  : * *.  *  ..*.  ***.*

38          MWYYAATRWCAMSGRTCYMMKTYSRWAARWCYYMGRKGMCCYKYRMYWKGTTWRKKTAGA
39          CTCCRWYGTTRCSARYACACKCYGGTWMAAYTYMRAKACTGYKTRACAGRAAWRKKAGAC
                        *.* .   * *.        **  *.   ** *     ::****:...

38          KMKKAAAAAKRKCTAYKMKKKKWARRKCYRGRRRATKKCTYRYYWRKKRKKWGGTWKRRY
39          KCKKCWCRRKAKYYWTKMKKTKARGRKYYRSRRRCCKKTATAYYWRGKRKKWRTCAKGRT
            * **. .  * *    ****.*   ** **.***. ** :  **** *****    * *
```

```
38          RKKKRKYRRWKYTRRYWYSYKKMRKKCARYYKKKARRYMRCGGYKKKMYKGGCKTGCMYT
39          GKKKRKYRRWKYCRRYWYSCGKCRKTTRACTKKKGRRYMRMRRYKKGMYKKAYGYKYMYY
             *********** ******  * **.     ***.*****   *** *** .     **

38          GYKRRAKTGTKYCCYCAMWYYKCRGGYMRWKSSKKWYRAKRWRTMMWRYRKAYGYTKMRK
39          RYKRRMGARWKCYYCTCCWYYKMRKRYMRAKSSKKWYRCKRWRGACTRYGGRCATWKMRK
             ****  :  *     . **** *  *** ********.****    **    .  ****

38          KKGRGGYYYRCMMKAMKKKYWKKTGWKWKYRKRKRCTYYYRYSYYYKRGCKKKRTARKAA
39          KKRRKRYYTAAAMKMMTTKYTTKYAAKWGTAKRKRYKTTCGYSYYYGRRYKKKRYRRKMG
            ** *  **  . ** *..** .* . **   **** .    ***** *  ****  ** .

38          RMYYKYYKRRKCKRRCKTYYWKCTCCYKYYRKKYKYKKRKTKYTKRMYRYMKMKGKKKWT
39          RMYYKTCKRRKAKRGMKACCTKTCMMCKYYRKKYKYKKAGKKYKKAACACMKMKAKKKWW
            *****  ****.**  *:   *     ***********  .**.*     ****.****

38          AGKKMRRARMKMKRKMKGCGGKRRYMKATTRYKKRKKGGKKRRRAMSKAWSWMKKWRKKY
39          WRKKMGGRRMKCGGTAKASAKKRRYCKGCYRYGKRKGKRKKRRRTMSKWAGTATKWRKGT
              ***   ***   . *... **** *.  ** ***   *****:***  .  .****

38          YYRKWYAAYWRYGRYYYKSRYCWTTGTYSYKWAWATGKTTTRKMYRMTRYGRCCYYKYYR
39          TTAKWTRWCWRTAGYYYKCGYATYKSKTCCKWRWTWRKYWYGKMYGCGRYRRMYYYGYYR
               **    ** . ****. *.  ... . ** *:  *    ***   ** *  ** ***

38          RYWTTCCSARCGYYK
39          RYAYCYSGMATACCK
            **    ..   .  *
```

Supplementary Fig. S7. The pairwise alignments between each pair of the plants 35, 38, 40, 42, 43, 44, 45, 46, 47, 48, 49, 50, and 51.

```
CLUSTAL 2.1 multiple sequence alignment (clustal format)

The pairwise alignments of two accessions 35 and 42

35              KYYYRYYRMYSWGTGTKWYTMWYYMKRGYGGRKKYYGCMKWYKMRMRYRGKMKYYRAYMA
42              GCYYRTYRMYSWRGTGTWTWAWYYMKRRTRRATKYYATMKWTKARMRCRRKMKYYRGCMG
                  *** ******    .*   ******     .***. *** * *** * ******. *.

35              CRKRRSYWMRATRKCWRYYCMYAGMWKMTYATMRRWWWRKGTRMTCCTAACRKRYYRRCA
42              GRKRRSCWCGMGGKMWRYYMACWKMWKMKYTAMRRWWTRGRYAAKAYWGGYGKRYYRRTW
                 ***** *     * ****     ****.*::***** *     ..  ..  ******

35              TSRRCTAAGKWMWYWYYYKKMYRRRRRWRRRYMYWTMWYCAAKSRKSCCTAYWYRYRYMR
42              KCAATWCWATWMWYACCCKKACRRRRAWRRRYMCWKMTCYRWKSRKSAMYRYWYGCGYMR
                ..    . ..****    **  **** ****** *.*     *****.   ***   ***

35              KGGWKAYTCCMSCAAMYYYYYYYCRWKRMRRRWCMKRRYRGTRYKSYRYCYYMRSGGWYM
42              TTRWKRYYYYCGYRWMYYYYCCCARAKRMARRWYMKRRTRRWRYKSYRYAYTMRSRAWYM
                .  ** *    .   *****   .* *** *** **** *  *******.* *** .***

35              YKYKKRRTTRRAASGKRYWRKYRYKMYTRWGCWRKRYTTTGYMRYRRAYKRYGMRWYYYY
42              YKYKTRAYYGRGGGTKRYWGKTATKCCYRWAYWRTRYCACKYMRYRRWYKRYAAAWYYTY
                ****.*    *... **** *   *   **. **.** :  ****** ****.  *** *

35              KKTRCGRYGRYMACGYTYTYYKRYRSMCMRYMCCYCCKRWWWRWMWYYYRSRGATSAYYT
42              TKYGMSRYTRYMRMTTCTCYYKRYRGCSMRYMTYCSSKRWAAAWMTCCYRSRKRWSGCCG
                .*   .** ***       ******. .****   ..***   **   ****   *.

35              GMYKRYMYKRYRMKARYMRRYRRRGKCYYWTYMRAGYGMRKMMWMRYYKGWRWYRWRYYT
42              RMCKRYMYKRCRCKRGYMRRCRRGRGTYCWYCCGGKYKMRKMMTCACTGAARWYRWRTYY
                 * ******* * *  **** **    * *    . * *****      . ****** *

35              TCCATYGKWYSRRCRCMRKYGTYMYKYRCCKAKCGAAATGGCCKKCYSRAAMGMYAGGAR
42              YATWCCSGACSRRSRAMRKYSKYCYKYRTSKMKYKRRCWKCTTKKAYCGCTMAMYRRRCR
                 .    .   ***.*.****..* **** .* *    .     **.*. .:*.**   .*

35              TCCAYKYKYRYKWWRWTGKKKTRGMYCAGCKTMTACCGAKSKYGKKTKCYMRWRKYMRKR
42              YYYRYGYKYRYGWAATAAKTKWRRMYYGCMTYCYWATKCGSKYAKTYKYYMAWGTYMRGR
                    * ***** *   :.*.* * **  .  .    .  . ***.*. * ** * .*** *

35              MRRRTAKCTCGKRYAAWGAYYRRRKGCGWKKMRKKWTGAMKWACYRRRTTWKKCCGYMCK
42              MRRAYRKYWMKKRYRMWRMTYRRRGRATWGGAAKKWYRGAKWGTYGRGWCTKKTTRYCMK
                ***   *    ***  *   ****  . *    ***  . **. * *    **   *  *

35              YRYTATTAMKRWRYKRTTMRWKRWCAKAGCKCMYGRKGTGGYYRTCTCYWTKARTYKMYY
42              YRYCMWWMMGAARYKRWCMRWKRWYRKGATKSMYKRTAYKRYYRWYCMYWGGRGCYKMYY
                ***     *   ****  ****** *.. *.** *..   ***    **     *****

35              RMWYRCCCTRTKGACRMWMYKGWARYKRTCWCCWRYMYTRSRTGCMMAGWTYWMTYTCSC
42              RCATASYMYRCTTRMAMWMYKSWRRYKRYYAYMWRYMYWRGGKRYCAWAAATAMCTKYGT
```

```
        *    .   * .    *****.* ****     ***** *. .     . :  *  . .

35      CTTYMCRTTYGYWGTGATKCRTYKWKYRRAWAACTAKCCGYYYYRKYYMYWMRYGTTGKK
42      TCCYMMAAAYRYWAAKRYKSAGTKWGYRRGAWWMKMKSYKYYCCRTCYCCAMRYRWWCKK
           **  ::* **.:   *.   ** ***.    . *.  **  *. *   ***    **

35      KACCKWRTKGCAKWKRWWCWMCGCACYWYGCRYMKKKRKKGCYKKKRKYKRCMRYGGAKK
42      KRYGKARYGATTKTGRWWYTCYAMMTCTTCTRCCTKKRKKRYYKGKAGTKRYCGYKKMKK
        *   * *  . :*  ***    .        *  .***** ** *   **   *   **

35      KTCGTTMKYWGCYYMTYYCKMKAAYTGATCAAAGRRTTCYCKGSCKTGGGKRKWYWWSTG
42      GKTTGYMKTTKYTCCYYYYTAGRGTYKRYYRRWRRRKYYCMKKSYKWKRSKRKTTWASCR
         .    **        **  .   .          **.    * * *   .***  * *

35      WRGGAGRGCCCCCTCKKWRSYGTGGYGYMYYYATGKRMYCCTATKKMRKYWWWYCYTACK
42      WRKKWRRRMMYYSYMKKTGGTTGKRCKYMYCYMKRTRMYTAYRYKKCRKYWWWYGYWGTK
        **    *     .  **  .       *** * . .*** .   ** ******* * . *

35      CTTGKGTKASYCKCKYKYKRYGCCGSTARKKRRCTYKWATAAGMKKKTTTMYAKGWRYRM
42      MYYKKRKKWCTMTTKYKYGRYATYASWMRKKRRTWYKWWKMRRMKKKACYMTWKRWRYRC
            * .* .  . **** **.  .*  *****  *** .   ****:  *  * ****

35      KYRGCAYTRGTKTTSWTATMGCGGCCAAACCTTYARTTGWYRMTMYRWRWKGRSKRATCG
42      KCAKMGCYRAWKWGGTKGAATTACAYRMMYYYYCRRYCKTTACGCCRTRWKRACGRRWTA
        *    .  *. *  . ..:   . .          *          * ***  . *   .

35      ASYGWYTTRTYTYMGMATATKMAKAAMKATGYRMRCTGRCKSMGGAAGGMYRYTWTYTTS
42      WGYAWYYYGKTCCAACTCGCGCCKRGAKTAAYRCASYRRMKGCATCRTRMYRCKWYYKKS
         .*.**   .    . : .   .* . *::.**  .  * *. . .   *** .* *..*

35      YWTTTAKYYKYACAYGGRWYKYMYMYRRCCGTCCGRWRRGTKRMARWTTMGTCCGGKKRR
42      YWWWYMKYYTCGYWTKTRWYKYMYMYAGYSRWYARRWGAACTRMRRTWCCRCTSRRKKRR
        **    ***. .     *********   .   . **  . .** *       .  ****

35      RYATWAGKRWCKGRRRYKCYWKSAKGAKKGRCRYARKKRCAWRRRTMYGGTGKRSKGWKR
42      GCRGTGAKRWMKKRRRYKYYWKCTKATKTRAYRYCRKKGMMARRGACTKRYTKRSKRWGR
             ..*** * ***** ***.:*.:*.   **.***    ** :      **** * *

35      GSYGTRKATGYRKAARKRYMRKGKKCWCCTKMYYRYYWCCAKSYMAAGGYYTCTRYAKKK
42      ASCAWGKTCKYRKGCRKRCCAGKKKYAYTKKMYTGYYWYSRKGCMRRRKCTKYYACCKKK
        .* .  *:  ***..***     **    .***  *** . *. *      .    .***

35      KKCGWKWKCGGRYKKYTYYRTTAAKAGCRCYTGRRKCKTMKTAYGKCATTKTMCKRKTAC
42      TGAATGWKTAARYKGCCYYRCCTTKMSYRYYWKGRKAKWMKYMYRKTRACKYMMGAGYRS
        . ..  ** ..***   ***  ::* . *   **.* **  * *  : * *       .

35      TYACAYRMYKKCYRMTGYKYYMATAWMAGWWGYRRKRCKCKGKKKWTMRYYTCKCRRKMG
42      CYGTCTAMCGKMYRMYRYTYCCTATWCWSWTRYGATRYKYKAKKKWWMRYYYYKYRRKMA
         *. .  *  * ***  *.*  :::*  .*  *  .* * *.**** ****  * ****.

35      RCGCKRGGCCKKKKRKACGRMTWKTTGTAMYKMKAATTACMRMRTMRTRTAKRARYTAAK
42      RTAGKRRTTGKKKKAKRYKGCKTKKYRYWMYGMKMTYKRYMRMRYMRWRCGKGRRYYWTK
        * . **    **** *     . *.    ** ** : .  **** ** * .*  **  :*
```

```
35    WRRWKGTWMTWRRGAKRYYYWCTWTGMKCGGMYYTTCGMWMWTYGGCYCKTTTAGRGCCA
42    AAAWKRYTCCWRRRCKRYYYWMYAWRMKYAKACCYYMRAWMWCTAKYCSKGAWMRGAATG
         **     *** .******     ** .         ***  .   .* :    .. .

35    YRAATRTGMWTKTKWSKRTGRATGRRMTRKMMYAKSKGKYKGKYYMWTCAACAACGGGAR
42    YRWGYGKAATWKYKAGKRYKGRWKGAACRKACCGKCKRKTGRKYYMWYMGTMCWMRRRRR
      ** .  ..   * * .**          **   .*.* *   *****  .: .      *

35    TMWGCWGAGAKRRCCKYWYACKCTRYKYGGRSRSKAYRRGRKKMCGTKRRMRAKCTAYCA
42    CMTRMWRMKMKRAGYKYWYRYKSYRYKYRRGSRSKWCAARRKKCTACKRRMRRKSCRCMR
       *   *    **   ****  *. ****   ****     ***  . ***** *.

35    TGAGACKRYRYYKYRTGGTMKGTTYCGRTRYKKYRRWKAGGRKTTKWYAYYKTWRRMGCW
42    YRRKMYKRYRYTKCAWRCCCKSWWTYRRCAYTKYRRWKRCRRKYYKATRYYGYAGACRYW
            ***** *       *.     *  *.******   **  *   **        *

35    KYYWYYGKKRKWYATCAAYMSYAGCTTKRTCRTYAKWAYTARMWWYMSKTGTCWATATKT
42    KYYWCYKKKAKWYWKYRRYMSYWSYWKTGWTRKYRTARYWMGCATCAGKCAYSWWKMWKW
      **** * ** *** .   **** .  ..   *.* .  *        .* . .* .  *

35    CGGKKKTCWCYKYKYYTKYKKTYTKCCYGTGCACKKMRAAGYTTWTWGRKCCTTRACGGK
42    MRRKGKYATTYKYKYYYKCKKCYKKMMCKCRYRMTGAACGACYKWWTRAGTYWWRRMKRK
         * * .  ****** * ** *.*         .   ...  .*         *    *

35    RKAAGCKKKYKWYTAYMGTYGYAKCKRYCTYRYRTCGYCRYAKYKCRGWKWYRAAKYAWK
42    RKWRKYKKKYKAYWRCCCCTKYCKYKGCAGCRYRCYKYYATGTCTMARWKWYRGGKYRWG
      **    ***** *        *.* *  .  ***   *   .. .   *****..** *

35    MTAKWYMAYYYTWTCCYCTKCYCYRATYRYGKRATATCGCRKSTATTRTGRCCMYKKKTK
42    MCRTTTAGYYYCWGTTTYCKYYYYARGYRYRTRRWMAYCMGGGCCGKRCKGAMMTKGKKK
      *  .   .*** *      * * *   *** .*   :     . . .*   . * * *.*

35    KCSYKWRCTTATAKGTTTGMKKWCCWRARYWSTYKRMGGWKKRSGKKCCWATCTCKYGYK
42    GTSCKWRYYCRCGKCCCYRMKKAMTTGWRTASAYTGCRATKKRSAKKMYWMWTGTKYRYK
        * ***     .*     ***      *  *:*.   . ****.**  *     ** **

35    AACSTRKAKTAYKRKKTKGAWMYKGTATKCYSSCAKTKRMKKTMYRRGAYATYTYRKWKY
42    WMMSKAKGKYWYKRKKCKTRWCTKACGATTYSSYGKYKRMKKKMYAGRWCGCCYYRGWKY
         *. *.*  ***** *  *  *. .:. *** .* *****.**     .   ** ***

35    WYRWAKGMGGKAKKKTYYMYAAGTKAYYAGWGTAYGYATTAYRWYYAYRKYRKKGATAYR
42    WCRWTGRMKKKGKTKGYCMYMWRWKWYYRKWRYGYRYRYWRTRWCCRYRKYRKKRRYWYR
      * **:  *  *.*.* * **    * **  *  .* *     **   *******    **

35    GRCKYWWKWYWCGYYYRTRRARWKYYMYTMARGTCAAGWCTACGAYACKKYYKKKCGRKY
42    AGYKYWWKTCTTACCCRWRRRAATYYMYKMRRKWSRRKWYCGYAGYWYKKYYKKGTKRKY
      .  *****    .   * **   .****.* *  .   *  . ..*  ******   ***

35    KRKYAYRCYRRYCGGCGMAAWYSRCTYYMGRGYRYMATASWWKKGAAYGAGGGKWKYRCG
42    KAKYRCRYYRRYYRRYKCMWTYSRYAYYCRARYGCMRWRGTWKKRRRTRMRKRKWKYRMR
      * **  * ****         *** :**    *  *    .***         *****
```

```
35          TARKRTARMYCKTYCYRYMCCTRKAYSKMYYGATCRATKMMKYRGCTCTTSMKCYKKATR
42          YMRKRKRRCTMKYYYYRYAMYKRKMTSKMYYRWATARYGMMGYRATATWWSMKYCGKGCG
              ***. *   * * ***   .**  *****  :     ** **. :   ***   *.

35          GTAACAKKAATCCGTCYRAWGTGRRTYYKRGTGYGAGTMWRWYATGTYGWTACGTYGACA
42          AYRGMRKKGRYYYAKYYRTTCARRRKCCKGKCTYAGRYAWRWYGCKGYRTYTMRYYAWSR
            .  .  **.    .. **:  : **.  *    *..   ****.   *   :   *. .

35          MWASRKGGKTRRKWWATASRRSKYWKKMGYYKGCGTTTGMMYRRRAKKKCGYYCRRKYMC
42          CTRSAKRKKWRRKTTWYRSRGSKTTKKMTYYKRSRWYKCMMYRRRCTKKTKCTMRRKYMM
               * *  * ***     ** **  *** *** .   . ******..**     *****

35          YRRKMYGAMCWKRTTMGTTMYWCTTATMGKCKWTKWWSKKTATTRRGAKKGCASMGRKMT
42          YGGKCTRGMMWKRYYCSWKAYWYAYRYMRGYKWKKTWSTKAMWKRAKCKKTTGGAAGGMG
            *  *   .* ***   . . ** :   *   **.* **.*:  .*  .**  .. .  *

35          RRKRRACCACAACKYCMCYYSYYYAATTTCGTYCCCYTMMYYYRTGKMSRSWKSKARRWK
42          RRKRRCTYCTCRSGYYMYYYSYYYMWGWKYRCCYYYCYCCTCYRYAKMSRCTGSGRAATK
            *****.  . . . * * ******    .             ** .****.  *     *

35          CCATKTCRGYYRRRWSACCAWTAKCAMYRYKTMCYYYCAGYRRMATATRTKAGTYYGGAM
42          TYWGKYYGRCYRRRWSCYYWWGGKTTMCGTKYMYYYYYRTYGRAMCTYGWKGTAYYTKMC
                *     ******.   * .* :*   * * ***   * *   :   *. :**

35          GTATYWGAYKKYRAYWKKKYYKYYRTYGGKRMTYSSGKRKWRGRCMAMRAGTKKGWKMMC
42          RKRACATWYKTYRRYWKKKYYGYYRWYKRKRCYYSSKGGKWRKRMCMMRWRKKKATTMMT
             . :    **.** ******* *** *  **  ***   *** *   **  .**. .**

35          RRKGRRKKYCTRCATCCKKYGAAWAKYYWRARKTRMRGRTRTTAAGGYGYRAGTGYYRKM
42          RRKRRRKKYTGRYWYTTKKCRRMWRKYYWGRRKYRMARRKGWWMCAACKYRWRKKYYRGA
            *** *****  *     **    * ****  ** **  *.    ...  **  . ***

35          RCGKTKYKRATAAKTWRSYWYYRTKYTARYAYYWTRRTMMSRTAKTCCTGGAWKGCGCKK
42          RYRGWKTGRMYRRGYWRSYWYCGCGYCCRYTCCWKRRYMMSAKMKCTYWKRGWKRTATTT
            *    *  *      ******    * .**:  *.** *** . *      .**  . ..

35          GAMCYKCTTGKYRKCKGMKGGTGYCWTRTGRYKWRWKYACGATGTCKRRCRATRTGMARG
42          TWMTYKMYKKKYRKMKTAKCTYRTTAAAYARCGTGTGCGTRWCKYMKRRSGWKAWKCMGK
              * **  . **** *  *       :  .*       .       ***.  .

35          KAGCTCTCGWTCGTTKKKGARRCACATTTATTTCYCAACCGKGCRKAGGCKWRYKYCAWT
42          TRATAAYMRWKMRYCKKGRCAATTTGCYWMKWAYCTRRYYRKRAGTWTATKWRYKYYWWC
            . . :.   *.    **  .   : .    . :        * . .  . ******  *

35          AYYATCYKYMRGYYKCYTKKRCMYRKCKCATRGCRCGTCKYKRRKACATGKCKWSAKRYK
42          WYCWCYTTYMRKYYGMYCKKRMMYRTMGSMCRRYRATGAKYKRGKMYMWRKMKTCWTGTK
             *     .*** **  * *** ***.  .  *  *.  .**** *     * * . .  *

35          MAAYMKCCCKGTSRYAKGTAAYGKACTATYRRRWYRTYRRAWYYCARRCRSYARRTKGTG
42          MRMCAKYYMKKGGRYRTKWMRYRKWMWGCYARRWYRYCARWTCCTTGGMRSYRRGYKAYR
            *    *   *  .** .    * *   . * *****   *     :   *** *  *.

35          TRACAWGGGKMKWMKKTGGAACMRWRSMRSRRKATTTCCMRMYKMKRMRTTGYRYGARMY
```

```
42              YGCTGTATAKCGWMKKYRRGCAARWRSMRSRRKGWWKYGMRMCKAKAAGYKKYRCACGCY
                  . . . .*  ****   ... **********.  .  *** * *    . ** ..  *

35              RGKAAGAAGYWYYSKCTGTAKTAAGMWMRYCTKCARKRYRTMRKTMGGSRKWKTRKGCCK
42              RTKGWARMKYWYCGKYYSWGKCCCAMWMRYMYGTGAKRCRKMRKAMCRGRGTKARKAYYK
                * *. .   *** .*  . .* ...*****    . ** *.***:*  .*  *:**.  *

35              YCCCRRYKAKRARYYRTRKYRMRYKKMYATCATGYCCRKYGATYKMMKKCTTAKKKKYTM
42              CYSMGGCKCKRRRYYRCRKYRMRYKKMCWYYRARTYYAKYRWKYKMMKKYYWMTKKKYKM
                  .    *.** **** **********     :     **  .******    .****.*

35              YMKRSTCYRAGYKYCMMRGTMRYSTGRGWWAACCGRGACCARTRAGATCCAYATYYCKCT
42              YMKRSATYGGACGCYMMARKMGCCWRARWTRCTMRGRMTYRRCGGRMYTTGTGCYYYKMG
                *****: * ..    **  .*  .    *  .         *  .     . . ** *

35              WCYGKCTTATTYTGCGCGMAYTGACGYMKTKCGRRACTTGKTCKAYAGYCCGAAYCMRMM
42              WMCRGAYYWKWCWRYRGKMRTKKWMKYMKAKMKRRWYCCKKYSKWYTTYAGAGGTTAGAC
                *    .   .        *  .    ***:*  **     * .* *: *. ...

35              YRCCTCSKRTGTRRRYYCYRGYRTKRWTKTTTYTKARGYYSTYYRTTAGATTATTRTCCT
42              YAYYCASKRYRCAGRCYTYRRCRCGRTGKCAACYKWARYCSGYYGYKRRMCCMYWRYYYY
                *    .***     * * **  *  *  * ::  *   * * **  .        *

35              TCYCGYWAKYWYRWKAAGRWRRYMTACACAARTKYTTTKRRKYKTTYMKGCYRKWTTCRT
42              KYCMSCWMKYWYRTTRRAGWGRYMYRMMMRMRGKYYWYKAGGYKACYAKAYTAGTYYTGC
                .   . * ***** .  . * ***       * **   *   **: * *.

35              SRMWRGRAKMAAGTRTWAYRMYWYCCWGMKKGCRGWYASYAGGGGCATAKMRRTWYRGMK
42              GAMTGRRMKMMCSYRKWMYRMCWYMYWAAGTAYRRTCCCCTACATTGCGKARRCACRKAT
                . *   * ** .. *.* *** **  *.  .. *   .. :. .  . .* **   *  .

35              ARKRCMRCMSTTTAKWTTACYRWARCRTGTTCCKYRGGAKWGTAGMCWMAATTGTGRKRC
42              GRTGYMGGMSCCKMTTYWCSYGAGGTGYKWYYYKCRKKMKWKGGAATTCRRYGACCRKGA
                .*.  *  **  . .   ..*  .         * *   **  ..        .  ** .

35              AYSYKKKAWGCRRGMGCTCYMGKKAKYKYGTARGTCKRKYRWRRGACYGWYGSKAAATTA
42              TCSYKKKMWTTRRRCKACYCCKKKGGTKYRYWGAWYKRKYRTAATRYCRWCRSKRGTYKM
                : ***** *  **   .     **.  **    .  *****        *  ** .: .

35              ATAYMKKSWKAKKGRCCWTRYGKGKAGRTWGWRTAATKCRCRTAKGGCYYCCGTGGRYGR
42              MKMCMTKSTKGGKARSMAYGTKKKKGKAKTATRYMMWGARMGCGTTATTCTMRYRKACKG
                 .  *.** *. *.*.      * *.  . . *     .*   .. .

35              MKYACCAYTSAAKKARAKYKCRTMWTCTYKTTGGKRKMGGATTTGGGGGKKYWCYGCKKY
42              AKTTYYGYYCRMKKRAGKYKTRCMWWYWYKKKACKAGCKKGWGYRKSRKKTYAYCRYKKY
                 * :  .* .  **  .*** * **   **... *     .     .  *.*     ***

35              YYMWKKYMKMCGCWRYWTMWKTRYAATTKSYKMCGCSGAKRKRRGACGTGKKTAKYTGCT
42              YYCTGKYMGASSTAGCWWCWKWACGGACKSYGMYRYCAGKRKRAKWYKYKKKYMKTCAAY
                **   ***  ..    *  **   ..: *** *   ...****       **  *  ..

35              TRMKTKCYRTAYYYMKTGYCGRYTWWGTTRKKKCWKRRKYSKGRYYAGMYRKRGGCKAWA
42              YGCKKKTYGWMYTTCKAAYMKRCYTWTGYGKKKTWGRGGTCGRACYWRAYRKARRYKGWG
```

```
           *.* *   *   *:.*  *   *    *** * *   .    *    ***    *.*.

35      CTTRCGYMAYKGTKRCYYAGYCCRGGAYKKGTRYKRWTAGTGTKRYYYAYYWATCACCAG
42      YCKGYKCMRCGRWKRMYYWATMTGATGYGKKCRYGRTKRKAKYKRCTYMYYWRYTTTTRK
          .    *     ** ** .    . .* *  ** * . :  **  * ***   :

35      CKRKRYTGGATYRYRKYKKMTMRRRWYRCGGWKTGAWCKCKSKAKWAYYAAGGTATMAAT
42      YKRKRCYARGCCGYGKYKKCGMRRRAYGYRRATARRWYKYKSKRTWRYYWRKRKRYCTRY
         ****  . .   * ****  **** *     .:  * * *** .* **    .   :

35      GATGCRRSKKWYWRTGTARYRWGTAGCYYKSKTSKACCMRMATGYCSGTMYYYKCAMRTT
42      KRKSYGGSKKWTWGCRAGGCRWAKWATYYGCKCCGCGMMRARCAYMCCWMYYYKYMCACY
          ..   **** *   :.  **.. . ** .* . .  **   .* .  *****

35      CKGTAKYYACCAYWYTRMYWTYCGKKKT
42      TKSKRGYYTTAMYACWAMCWWTYRKKKW
         *..  **: . *    * *    ***
```

The pairwise alignments of two accessions 35 and 43

```
35                WYGYMYSYWYKYKRKRMWRYYWRTGRAMRGGCTTMTAMYACRYRKCRKMYMRAAKSRKSY
43                WYTYMYSCTCKYGGKRMWAYYWRKRGRCGKATCCMCRACRYRTATTGKMYMRWWKSRKSY
                  ** ****   **  **** ****.      .   *      *  .  *****  ******

35                YRYYRKCGTRYYYRWRKYYYYYYGCARGYRCTTRWARCYKRSMGARCGWWMRRYYYYRGT
43                YRYYRKSRKGYYCRWRKYYYYYYRMGRRTGYYYRWRGYYKRGCRGGYRTWMRRYYYYGAY
                  ******. . ** **********  .*      **   ***.  .    ******** .

35                TCCATTRWWYYRRATMWTAMWCYKWMYYYMRRYKWYYAWTRWKKCRWYCKCCYGCRMKTC
43                GYYRAWGWWYYRRWKCAWGMAAYTWMCYYMGGCGTTYWTGGAGKYAWCTGYYYRTGMGYY
                      :  ****** .   .* .*.** ***      *      *  *     *   *

35                WKGAYCRTTTWKYKMWYWWMKKYKRMRRAGKCRYRCAKKGGYMACCKKMKRKYTCTTYWR
43                WKAGYMAGYYWKTGCWYWWAKKTKRMRRCSKMRYRATGKKRCCGTTKKMKGGCCMAATWR
                  **..*     **   **** ** *****..* ***.: *    .  ****     :: **

35                CCCTTYTTMCYTRRYYTYWMRSARAKWRGYCCAYYRYWTTYYRYYRTTKYYRYYKTTWYC
43                TTYGGTGYCMCGRRYYWYTCGGGGTKTRKCAGGTYATTWKYCAYYRGKGYYRTYKGKAYY
                              **** *   .. :* *  . . *    .*  *** . *** ** . *

35                RYWACCCKACMYYGRCGRKMSYRYCMSRWSYMKYYMYKKRMYKKGKYRWCYMYMCRYKMR
43                ACWRYTAKWMMYYSATARKMSCGTMMSRTCCCTTCMCKKRMYKKRKTRWTTMYAYRYKCG
                    *   .*  ***.  .****    ***  .  .  * ******* * **  **  ***

35                SRGCCRYKGWMRWAYRYTYSRKAWWYTMAAGKRYYYAKCTCGYACTTGGACTGRGRYCRA
43                CGKYTRYGKTCRWTYGTYCSRKWAACWMRWRTGCTCMKYYTATRTGYCKRTYCGKRYTRC
                  .    **    **:*    ***     *   .     *   .             ** *.

35                GTWYATRRGTGAACCRRYACCCGKAAAKYGTAGKCACRTRCGGWGTCGRKGYRSKTYRYT
43                AYATMCRGRYRWMYYGRYCTATAKWMMKYAGGRKTGYGYGTAAAAYAKRKTCASKKYRYC
                  .     *         **. . .*   **. . * .     .. . . **   **.***

35                MYYAAGGMATGATKMYYYRACAYWAYKGRWGGRMWCYYRGYGGGCASKMRKMRGYAMYYY
43                MYYTGTACRYKRKTMYYYGGGGTWGYKKATARACWTCTASYATRMGCKCAKMRAYRMYYY
                  ***:. .     ..**** . . *.**   .   *    .*.   ..*  ***.* ****

35                YGAGMTCRMTAKYTYKYCRRRKKKAYRYRTAGAARKACGACGRTCKKCACRWRYCYGRMA
43                YCRRMYYAMKRKCGCKCTGGAKKTTCATACTRRMRKMMTRYKAGYKKYGYRTGCYCRGMG
                  *   *   *. *   *     **.:     :   **         ** . *       *.

35                CCKATGCCYCMCKYKAYRTRARYMCRAACAWYTGGGCAAYGKSKRTAATWAACRAATCTK
43                TAKMYKYMYMMMGCKGYRWGRACCMARRMWWYYRTTYGCTKTGKRKTCYATGARGGGTYK
                   .*     * *   *.**            **     ..  ..**.:.  :..*..   *

35                GSYRCYCGATWRRCWRYTCCYWYYGATTAAMYYCKCAKKTGAKKCGAKCAYTACRCRGGY
43                AGCRYCTTTAWGGTAGCWYMYWYCRRKYRRAYYMKMCKKCAGGTMRRGSMYYCTRMGRSY
                  .. *    ::*         ***   .    ** * .** .. .    . * . *   .*

35                GCRKTYCRSKGGGTWAYWSKMYKGMKYMCCKGMSATYTRRYTYAACAYARYCGCAGRYYS
43                RYRKCYTRGGKRRYAWCTCKMCKRCKYMYYKRMGRWYWRRYYCRGTTYTACARACRGCTS
```

```
                  ** * *.         .** *  ***  * *.  * ***   . :*:  . ..    *

35              YRRKGRTGTWGAAYKRTKKGGMRSMRSRRKRMYKGTAMKRYGRRWKYGCWCWRYSYKMRK
43              YGGKARYRYTARRYKGYKKRRARSMGCGGKRMCKRYRMKGTAGGTKCATWYWGCCTKMRK
                *  *.*    .  **  **   *** .  *** *   **  .   * . * *  . ****

35              GTRWCCTKYTGCCKRGRSRYMCMMYKMYTRKATAKCTCMGCCRRTRRAYGAWCRKKYYCT
43              ACRWYMKKTCAAAKGRRGGYCMAATKMCYRKGCCGYYMMRYYGGCGGGYKTAYRKGTYTC
                . **  .*  ...*  *. *     **  **. .    *        .* :  **  *

35              KGRCRGCTTTKRCTGTRKTRWCGMYGTTKYRGCMWCKRYYYASKKAGRKTMYAATWWGAK
43              KAAMRRSKYKKGYYSYGKKGATTCCRWWKYRRMMTTKRYYTRSGKGTRKCACRGYWTRRK
                *.  *  .. .*   .  *.         ***  *  ****  * *. **    . *   *

35              RYCKKYSAKGYCKYRKKKRRTTGAAYWKCTSAWYWTMYKGCWRYRATCMKRCCGTCTAGG
43              RYTKKCCGKTTTKYGKKKRRAATGGYWKMWSRACAYMTTAGWRYRWYYCKRYMKCYYRKC
                ** **  ..*   ** *****:: ..***  *     * .. ****    **

35              AAKYKKAYATAYRRKWYKTWTTCCGGRYAGCCRRCKRWRRMARMRYRKCKCRYRWKYMYR
43              CMKYKKWTRWRYRRKWYKGTKYYYRAGYGAATAGYKRWGAMRRMRYRTYKMGCAAKCATA
                . ****     *******  .    . *...    ***  * *****. *     *

35              CGGMKKKYWTKYAAGMWCGWCYMCGKRRAGTMYACCYRARYMGGKGKCKGKRSTRMCYWR
43              TRTAKTTTWAKTRMRATYKTYCCMKKGRMRAMYGMSYRRGCMRKGTKMKRGRSCGMTYWR
                    *.. *:*              * *  :**. .**   *    * *  **  * ***

35              YRRKMYKGRYYWKASYYYYRTKRMACKYSWWSYYKGCKACKMGCKYKKYGTARGCCYRGY
43              TRAGMCKAGYYWKMGCCCCRGKGCMYKYSWWGTYKTTKWTKCTYGYKKYRYWGAYYYATC
                 *  * *. **** .    * *    *****. **  *  *    ****    .  *

35              GYTYTGGKMKAMSGCWKAKCCWSGKGGWRTTYTATYKTRCATAAKCWGRWKGGCKGGGGY
43              ACKCAARTAKMMSCTWKGKSMACKTRRWAWKYWGCCGARYWYMMGMTRATKTATKRKKKY
                . . :. . * **  **.*.  . .  *  .* .   :*           * . *   *

35              RYKWKRMMKKYACATCATKTKAYMYKRRWMWRGTCAYAATAYKRKMYKWKCTYYKAYRAG
43              ACKAKGMMKTYTYGWYWWKYKGTCYKGRACAARYMRYGTGGTKAGCYKAKYATTKGTGGA
                  * * ***.*: .    * *.  ** *        *.: . *   ** * : *.   ..

35              CTKRWTAATCCGYYYRRTKKRGTAACAMMATWYCYGKWRTAAKKMYMRYKYCWAMGCTTW
43              YYKRTCRTGYTACCCRRCTKRKWTRTGMMRYAYTYRKTRCWRKKATAGYKYYTCCRMYYT
                  **    :   .   ** .**  :  .**   * * * *   **    ***  .

35              YYCCGCYRRYKRYYWYSKYAYRMYGTAYYRATCASYKWTWCGKTYTCRKKRYKYYGRMKK
43              CCYMATCGRYGRYYTCSKYRYAMCRAGTCAGAMRGTKTATYAKKYKYGGKGTKTCTRATK
                    .   ** ***  *** * *  :.   .:  . * :  .*.*.   *  *   * .*

35              ACYACTGSYRGTTACTCSMCTKRCCWRRAKATRCATGTGTTTMKAGKTSTWMKATTRCRY
43              CMCRAYRGCAAYYMTATCCYAGGYYTAARKGCGTCCACRYYWMKRAKYGCAMKMYWRMRC
                .   .  .  .    : .  :        *.   . .     ** .* .  **   * *

35              GKYGAWYRWKYRYMSACACAGARRKYYCKWARWTCCACRTSACGMYCTWCYRMRKKTYSC
43              RKYRRWYRWKYATCSCAGTGARGATCYTGTMGTYAYRTGCGTAKACAKTTTGAGKKWCCY
                 **  ******   *... ..   . *       .     .:.  ..       **  .
```

```
35      YYCTTTKGATATCMAWKRRTWYCCAAARKRKGCRCTGCCCGCGAKKYKGMACRWAKTCWK
43      CCACAGTAGCWWTACAKRGCACATTGRATGKRYRYACYYMTATCKKYKAMMMRWGKKTAG
          . : ...     . **   . :.  . *  * :     . .****.*  **.*.

35      YKYRMWCAAAMYRGRCGAYGSSTKYYTRCTKWKKKWMYKMKTKAATGCMRYAKRACKYGR
43      YKYACTMRRRCYGRGTTGYASSGKTTGAYKGWKKKWMYKMTGGMWGCACRCRGACGKYKG
        ***        *     .*.** *    . *********.      . *    . **

35      WAAKCATTRYAYYRRKYYSRYYYKCCWCTYGYMTCMYGYRKSATCWRWKAKKYKYRYMYR
43      WWRKSRYWGTRCCGGKYYSRYCCGYMTTYYKYCKACTKYRKSCCYTRTKMKKYKYGYMCG
        *  *.          ******        * * ..   ****.   * * ***** **

35      YKTCKTCCCGGAYAARAACCCGAYKKAWKAKKKRCTYRRGAMAMYYKKAKKWKYYCKMCT
43      TKCMKCAYYRRMCTGGGWYSYATCGKGWKTTKKRTGCRRKMCRMYCKTRTKWKYTSKCMY
         *  * .      :. .  . .:  *.**:.***   **    ** *. .**** .*

35      ATKTRRTCCMACMKMYKTCAGATKKKRYAAWKGTTTYCRYRTCYACCYTRTKTAMCTTAC
43      RKGYRRCYACMTMKMCKCTTAGCKKKRCGCWKRCACTMRYRYYTMYMCWGWKCCCTKCCT
         .  **  .   *** *  :.. **** ..**  :   ***          * .  . .

35      GMRRYCKKGYWYRRWKYGRCKTCTTCKYKKYYYWYAMCCCGYRTKKRGKRRYMRRSYRKR
43      KMGGTMTKKYTYRRWKYCRYKYYCCTKCKKYYYWCCCATTKCGKKKASKGACARRSYRKR
         *    .* * ****** * *     * ****** . .    .** .*     *******

35      CKRYRAGCATTRAAYRYTAMWWYMSGWTKRMKKGTAWWYRKYKRYKRGGCCCWKTTKWKT
43      MTGCGWRMCKYGGRCRYWMCATCAGRWGKRMKKRGCWWTRKCKRYKARRYATWKCCGTKW
         .      ..  .  **       . * *****  .** ** ****    . **    *

35      TRKRKKKCGKKRCRAYCCKRCWRCYATGAKTYCRYCRMAGWYMWYCTSYMYCAGCTGCCY
43      WGKRKKKTRKKRTRRYATKAGWRAYGCAGGYYTGCMACCCTYMTYYYSCMYTGATCATMT
          *****  *** * *. *  **.*. ..  *      .  ** *  * ** ..  .

35      MMTAWSTTCCAKGAYWKYWACAGRRYTMYAGRACGTTKRGMGYCYAATATTARGACCCKT
43      MAWWTCAYTTGKAGYWKTWGTRRRATCCCGCRRTTYWGRRAKYACRWKGYGGGAWSYSKK
        *    .:   .*..*** *.   *     . *      *   *.  .. . . . . .*.

35      AAGKATAKRGCCGGTGTACTGRGYRKAYCAGKRYRACGTMYRKYAYYWYYTGARSGRAKY
43      RTTKTGTGRRYTTRWRYRTYAATYRGRCTGAKGYRCGAYAYRKYWYCWCCCRRGSRGWGY
         : *: : *           .  **    ..* **. .  **** * *      *    *

35      RMRKCCRRKWWASKYKMKGMMYYTMRMYMKKTTYTYRRKGRWKRYWTKYRKTATGKTYYY
43      RMRKTMRRKTTWSKTKMKRMMYCKMRMYMKGCYCWTGAKRRWGAYWAGYGGCRYRKWYYY
        ****  ***   ** *** *** .******        * **  **: *      * ***

35      ACCYYWMKMRKMACYWRKKRAGRKCRTGAGGYMRKTKKTMRCTGGRSYWYCKATWTKYRK
43      CYYYTACGCAKMWTCARGKGCAGKTRYRRTAYMGGAKGYMRTGAARSYWYTTRWWWGCGK
        .  *      **    * * .. * *    .**  :*  **  ..***** .  *    *

35      WGYYKKATRMRMGWWYCCTKWKKTTATCKYYWGKWTYGTGYKYWWSTGYYMTMATRTRCA
43      WRYCKKGKRMRMAATYSYWTWTTYKMKMKTCAKKACTRAAYGCAAGGKTTCCCWKRCRMG
        * * **..****.  *.  .*.. . . *    *    :.*    .        .* * .
```

```
35              ATKWRCKYGGTTRGCCKTGCRMRTTKKAGTTYAYGCRTCTTTKRMGTKTRCATACGYYYC
43              RYKWRYTYTAAYGRYYGGAAGMRWCKKRRYKYWYAYGYYKKKKGCRCKCRTGATGAYYYA
                  *** .* .:       .. **  **   .* *.    ...*    * * .:: .***.

35              CGMYYMAMTGKRWMWTYRTATYKTKWSCCRTCAKTKCATGRRMTRAKKKYKKKGCTCGGT
43              YKMCCMCAYAGRTCAWCGGTKCKYTAGYMGCYGKYKTRWKGAACAGKKGTGKKRSYAAAY
                  *  *.  . *       :. * . .     .* *         .**   ** . ...

35              RRYRGKCYRGGCYYKRWGCGYWKRRMRAKCCGRYKYRTTCCATCCYRCGKKMRKWAGCCG
43              RRYGRKTYGTKGTYTRWKYAYAKRRMRRGTMRRYKYRWCATCCYAYGATGGAAKWGATTT
                ***  * *     *.**  .* *****     *****  . .  .* .     **..

35              GAYMYGTGGRKAGGMARATTYRARRTTTCGTAGTTCMWCAMYTAKYYTCKYRYGACRRRT
43              KGCCCAGCCGKMKACRGWYCYGWRGCGCTCKGRACYCWYRCTYRTYYWYKCGCRCAGRGC
                 .   .    *  .      *  *      .. :   *      .**  *    .. *

35              GAAKRRARTWTGATAKRYKCYTTWCKRMYRYACMRYRAMGCMAKWRSWSCCTTYMTTYKK
43              RCGKGGGRCWGAGATGRTTTYCCTYKRCTACWYMRYGGMATCWKAGSAGTTCCTAAAYTG
                 ..*  .* * ..:: * . *    **      *** .*.   *  * .      ::*.

35              GGCGYRATAGGACTACGYKCAKYCKYYRWWARCTRGGTRTCTMYRYTTTKKGRWTGWWWM
43              KASRYRRKMARWAGCGRTTMMTYYKCCAAAWRTWRRKKRYYYMYRYAWYGKCGAWRAAAM
                 .. ** . .  . .   .  .* *      *  *  .*   ****:   *        *

35              KKMWGCRKKKRMGAYRYMKYCGGKKARCRMRCGKTYYKWYAYGKATCAAAGKGKGATGCCR
43              KKMACTGKKACTGYRCCKTTATKKTRYGCGMKKKTYTWYGTKKRYYRRWRKAKKTATAYA
                ***    **   .**  *  . **:*      *. *.**.  *       *.* :: .

35              YGTCACGARCYRYWYWWSWRTTTTYGGCCGGYRYMMYCKRYKMYCYRYCYTTAAGTCATT
43              CKAYGTAGRMCRYWTWWSWRYKKKYARMMTAYRYMCYYKACKMYTCACTCCCTTRWYGWC
                  : . ..*  *** ***** ...*.    .**** * *  ***        ::   .

35              YCRKATCRYCKCGAGKTRMYAACYYKYRMYYGYTGAKRCRWAGWCGYRTKKKWGMAAYGW
43              CYRKGGTRYMTYKRRKGRMTWRMYYKTAMCTAYCACKGYRWRRTSRYRYKTKARMWWCKW
                  **.  ** .    * **    ***  *  .* ..*  **   . ** *.*  *    *

35              YKMCYSCGSKCGTRRKKKCGKACARMWWGATCGMYKYRRWAKKRRCATGYATGACKGKKC
43              CGMTCCYRGKMRKRGKKKTAKRYRGCTTAMYTRATGCGGTMKGGAYWCACGCRRYKRKKY
                  *  .  .*  .* *** .*       .            *      . .    * **

35              TGACKYAKAGGTTAYYSRRTCTGACWYWGKWRYGRGGYCYRMRTCARRCTGKCMKYRRTA
43              WSMYKTWTWRACYRTCGRRKGYRWMWYARKTGTKRARTTCRAACYWRRMYTKAAKTRGCT
                 .  *  .  .     .**.     **  *    *.    *     **  *. *  *  :

35              YCTGGGAYCRTTCARYKCKKAYTTKYYWTAKKTTCRRRRRYRKYGKYCGCCCTTTTTAAA
43              YTAASRCCYGWGAWRCTMKGRCCWKCYWWRKGYKYRGARRYGTCAKCYAATYGKCCAWRR
                * :.. .     . * . *     * **  *  . *  *** . .*  ..   .  :

35              AKCKYMKTCTCGKMKKRKGTCAGRGMSSYRAGYTAGTGYKWTYCCTAAWTACTCTAMYSK
43              MGMKCAGYTWMKKMKKGKRYYCRRRAGSTRRTCCTAGACGTCCAACRTWCMAAAAGCCGK
                   *        **** *   . *  .* *    :. .     ..  :*  .:.:.  .*

35              KYAKMWWRYTRKRRMSKRMKKSWMRRRRYKAASKKYKYCKKYARYYRRYRKKTYARAAYS
```

```
43              KYGKATARYCRKAGMGKGMKGGAAAAGRTKTTGKKYKCSKKYRATTARYRKKKCTATGYS
                **.*   ** **  *.* ** .     * *::.**** .***     *****. : :.**

35              GYRRAATKGGCYCYKKGAKAAGCTYWTCKRKTCYGGKYGCGYMYKYRRRCGAATMRYSGT
43              KCGRRRAKRKMYMTKKRCTMWRYWYTAMKAKYTYRSKCRMRCAYGCGRAMRCTKMGCCRY
                   *  :*   *  ** ..     * : * *  * .*      *   *   .:.*  .

35              RCCACGWWC
43              RTTTMRWTT
                *  :  *
```

The pairwise alignments of two accessions 35 and 44

```
35              YYWGARKWKYRSKKRYYYMRWWRYTMTYACTYYYRRATYAAYYYYKYRTTYRYMCMGRAK
44              YYWRGATWKYRCGKRYYCCGTWRYWMATRAWTYYRRRCYTGYYYYKYGYWYAYCGARAMK
                *** . .****. ****    *** *:  .  ****  *:.******   * *      *

35              RRKWARMCTSMKRWATMSRRRKYWMKYMTARACCYWWRRYYGTWWMRMYKKYYYYAYYRW
44              RRKWRGAYGGCKRWWKMSRRRTYWCTCMAWGGYYCAAGGYYTCWWMRMYGKYYYYWCYGW
                ****     . *** .*****.** . *:  .       **  ****** *****  * *

35              RKSRRYMAAGMYRRRKWRWWYYACYYMRKKKSYYRYYKRTRYKYKYRAYRKCYTCCMCRY
44              RKSRGYMMCTACARAGTGWWCCGYYYMRTGKSYYRYYGGKGYKCKYRWYRKYYGTTCYRY
                **** ** .    *    **  . ****. *******  . ** *** *** *     **

35              YYMMKYMRKMKTTCYRCYWRMYYMKRYRMCTTTTMRGAKWYKMKTRAMRYMTCTCSYRKM
44              TCMMTYMRTMKYYMCGTCWRMYYMKRYRMYYWKWMRRRGTTTAKYRGCRCMATCMSYGKC
                  **.***.**       ***********   . **     . * *. * *:   ** *

35              RYRRRKWWRYCKRWYRAMYWYCCTACARRKTCTYRATWRSRGCTCAMRTTMRWKKYRYCR
44              GTAGAKTWRYYGRWYGGMCWCTYWGTTGGKGMAYARWWRGRAYYTRMAYYMAWKKYGTYA
                     * ***  *** .* *    . :  *  :*   **.*.    *   * ****

35              YCRYSKKWCYYKRMRWMSGWCYMRKAMGTCKKMATKWRTAMYYTYGYMRYRYGGTRRYRK
44              TAGYSKKAYYTKRMRTCGKWYTMRGRARWTGGCRYTTRCMATTWTAYMRYAYKRWRRCAK
                 . ****  * ****  . *  **           . *       .**** *   **  *

35              TYRYCCGCWYWYRKRRAGMYKWCGGMTTKRKCRWYYGCAYYRRTWYYRYRSRYMSYYYYR
44              YCACYYRTWCWCGKRRRCMYTAMRKMGKGRKYRWTCRMTCTGRATTCATRSRYCGTCYYR
                        * *  ***  **.    * . ** **    :   *:     **** .  ***

35              YRYRWYWYWCYRYRWRWKCTGRGCKYMWYYRMYKYYRAGATTYTYYRAYCATTTYRGYYA
44              YGTGWYWTAYCGCRWRWKYYSRAMKTATCCRCCKTTGWRMGCYCCTGTTMRKYYTRRYCW
                *   ***      *****  .*. *     *  *        *    :   .   * *

35              KCGGGYTTAMRRMYYYRWGYAKTAAGAGTACAGAATACAAAKCATCCTCACATGACGYCW
44              KAARACCCCCARCCYYGTRYRKYWGCCCYRATCTTAMYMRMKGWYYMWAMMWYRWMKYYW
                *.. .   .  *  **   * *  . .   .: :::     *      .        * *

35              YCAMWYRRRMKCKYAWRATYGKAAGWRYYWWMRMYRRYYCAGCTCCCCYTAYRYCMTGWT
44              YYRMWYRGACTAKYCTGTAYSKWRTWGYYWWAACTAGYYYGRYCTATSYYMCRYTCAATK
                *  ****   ..**.  ::*.*   * ****      ** .    . .*   **  :. .

35              ARYMMRMATKCTGWKCATTRTGRTRGTAYAGTRYGGGKAWMTAGGCRKKWRYKKGWTKAY
44              RGCACGCTYKAKRAGYRYYRGRRKRRCGYGRCRYKRAKTAMCTTATRKKARYKKSWYKWY
                       : *..       *  *.*  .*.  **  .*: * : . *** ****.* * *

35              CYKYMGYTWKYYCCYKTYYYKKCTCTRTKKCTKRRKKAATYTRYTYRMYRRWYATACKCT
44              YTTCAKCKTKYYYMCKCCCTTKSYYGRAKTMCKRRKKTTCYWRYWYRMYRRWYTCGTTYW
                  .    . ***   *    .*.   *:*.  *****:: * ** ********: . .

35              SMYRRCGWYSWKGTCYGCRWWSAWAYMKYTYAKCYKMRSAGTYKTAAAYWYARYSRRTTR
44              GACGRYRAYSTKKWYTAGAWTCWARCAKTYCMKYYKMRGRKWCKAGCCYWYGRYSRRAAR
```

```
            .   *   ** *    .  * .     *    * ****.    *:...***.*****::*

35          RYTGAWYARRTGGCTCRRRYCSARRKSRWYWTGGYWKMRRRTMGGKCGRSMTTRSRRKAG
44          RYYKWTCGARKRKMGAGRGYMSRGGKCGTYAYAATTKCRRGYCRRGYRRSMACRSRRKGS
            **     . *.    . * * *   *.  *  ..  * **        ***: *****..

35          GMYKGYRMRKRRTYAGRGYARMRYYYGARKMATWWGGYKYTGYKRGRMYCCTRRYRCKCG
44          TMCGRYAAGGAGYTWKRRTCGMRYYYKWATAGYATARCKYKRYKRRRMYTACGGCGAKYA
             *   *          *  . *****   . .   .  **. *** *** .     .* .

35          YTCMCCKTCCATRSKGAARRMMRKYCACKKCYWKGYKYTAMYGCGGCKRKRRTKAARRMY
44          YYGMTYKKMTTGRSKKRWAACMRGTMGTGTMTAKTYKYWCCYRYRRYGRKRRKKGGGACY
            *  *  *.  : ***      **   .  .   * *** . *      ****.*..   *

35          KKSYAKKGWYTWMYCYYGMKKKYWRYRKKRRKAATCKGKKCACCCGRRKKGYCRYWCGYY
44          KKSTCKKKTTCAATMYTKCKTGCWRYRKTRRGCTATKKKGGGMMYCGGKKRCTGCWYKYY
            *** .**        *   *.  *****.** .:: * *  .      **     *  **

35          CKMRTMRYMAATTYSTACYMKAKRGTKAKGRKTGTCKAYCAGTRMWRKATKRRYKKCMYT
44          TKAGWAACMGGACCCYWTTMKRKRRYGMKKRKYKATKMTMGRKGCWRKWKKGGYGKYMCY
             *      *..:  .    ** **    * **  : *   . .  *** .*  * * *

35          RRRGTCAAKATCYKRRRRRRKYKGYTCKKRKRCTMYSSCKWYTRCRTRRWKMTYMGTTAC
44          RRRSYYMGKGGYYKGRRARGGTGRYCYTKAKRYKATGGYKACCRYGCRGAKCYCMRWWWA
            ***.   .*.  ** ** *     *  .* **. .. *   *   *  *   *    .

35          AGKYYCARAYKGYYKTCRYYAAAKYMMYTATMGRYYKACSYTTYKYRWYCYRTARGKTTG
44          GRKYYTGARYGACTKCAGTTRWRKYCCCARYCKRYYGMTGCACCKCAATGCGWWRTKGCK
            . *** .  * . * .      **   :    ***   . :  *         * *

35          YGCTAGACCCRRKTTAWRMTMRYKYYARWKTCAAGTMGTGTTTCGSGGGAGATYKRMRYG
44          YCAAGRCMYYRRKYAGAACCCGTTCCGRWKCTGGRCCRYCCGGMRGAKKRRMATTGARTK
            * .:. .   *** :.       .  .***  ..           ..     :  .  *

35          TMTCWYYYYTGAGKAGCTCASRMKRMMYRWKAAYMTRAYMYTGGATACTYAGTTGAGYST
44          ACCYTCYYCKKCTGCKMYYWGAMGAAMTGWKMGTMWACYATYRTWWMTWTMTCCATKYGC
            :     ** . .  .     . *   *  ** . *  .*               .: *.

35          ATGMGCAWGTSKAATCMTGTTYRGMMATTCRGATASGCAATCTKTATTRGYTCCTYCGCC
44          GKACRMRARWSKGGGTMWTGGCGRCATGATRRMYWSRYGGGTCKCMYWRTYYYYYTYRTT
            ...       **..  *         : : *    *  ..   *    * *

35          KRYTKWRKYYYGYTGWYRGRYMKRARGTYMGKWKTTYKMAGKRRKYYTYCGMKTACWYWR
44          KACYTWRKYYYRYYRWYRKRTCKRRAAYTMRKWGYWTKMWAGGATCYYCYKATYRTWYAG
            *   .****** *  *** *  **  .  * **    ** .   . *     .   **

35          WCYRATCAMWRMRMGGTTMCCWWKGCAYRMWWRKKYRYYCTGTWRYMCAAYMKCYYYSCY
44          WYYRMYYRCTGAGMKSYYAYTTTKATGYRMWWRKKYRCCMYSKWRYMTGGCMTAYYCCYC
            * **         * .       *. .**********    ..**** ..*..** .

35          KYAYKTTKCATAMAAMKRRTWYCTGGCRRTSWMYKYYRKRGCRAGKWYCGATCAAYSYWR
44          KCRYKWKTTGCGMCMAKGRCACTWTATRRKSWAYKYYATGRYRWRTTYTTCYSCGCSYWR
            *  ** .. . .*.  * *      . **.** **** .   *  . *  . ... ****
```

```
35      TRYRWYYRTTKYGCAGRKARRMWTGRRYRGTTGYRKACGKKGTMYRCATYKYYMKYYMAA
44      CRYGWYTAWYKYRYRRGKWAACTAARRCGTCCTYRTCGRKKRYAYRMWWYGTCAKYYMGW
         ** **    **     *     :.**      **..  **   **   *    ****.

35      YTATARSYCTAKKTKYKWYKKWWYAWSKYKYGGAWKMYMCRGCKYWWYYMMKGKYRTMYR
44      YYCWRAGYYWRKGCKYKWYKKTTCWWSKYKTAARWKATMYRRYKTTTCTAMKRGTACAYR
        * .   .*   *  *******    ***** .. **  * *  *      **      **

35      RAGWKKRAYKMMYRRYAKGKKKYYRRKMGTCYGGRKKKRMKKAARRKGGTATWMMGACKG
44      RRCWKKGCYGMMYRRYMTRKKGCTGAGMRAMYTAGTKGACKKGMRRKAAYRYACCRMTTR
        *  *** .* ****** . **      * : * . .*   **. ***..         .

35      AYRGTRWRYWKYYYWSATCYRYYWMRAKRRMKKWRARCGGAAACRKYRRRRRCTWKCKKM
44      GTRTWRWRYTKYYYWSCCYYRCTAMACKGGATKWRGGYKRCGRTGKYRRRRRYKWKYGGA
        . *  **** ******.  **   * .*   .***.    ..   ******* .**

35      RAGCCGACAATMRKKAYYMYGYRRRRTGRGKAGGYMTKYTTWTGATKTARRYTCATMTTC
44      AGATTTWMRRKCAKKWYCCCRTGRGRKRRKKMKRYCAKCYYTCSMWKWMRRYKAGCMWKY
         ..       .  ** *      * *. * *   * :*     .  *  ***... * .

35      ATWCCKCMYTAKGYYCYRTKKRKYYRYGKMYYRGRRCRWSGGTWGCCKRKGYYWWCATRC
44      RYWYYKSCYGRTAYYYYRKTKRKTCGYRKMYYRRRGAGTGATCWRYYKRKSYYWAMWGRY
          *  *. *  ..** **..***   * ***** * .  ..  *   ***.***    *

35      MTACGKGGTAYYKSMGAMTGWTAWTCYYYGSCCTTTYMCRRSCGGATTGTRTWAYSRARS
44      CYRMRKKTGRCYKSMKMCWTTKTACTYTTKGTTCCYTAMAACYRKGKCAGRCAWCSRWRS
             *     ****      .:   *   .          .   .. . *    ** **

35      GRMTKRYYKYAKTCTAMGGGKKRTGATSYYGRRCGKKTTGGAAAKMATRKCTCACRWKCY
44      RRMWTGCTTCRKAYCRMASKKGRYKGCSYTRRGYRKKCGRRWMMKCTCRKACYRYRAKYC
         ** .   .  *:   *.. * *  . **  *   **       * : **.    * *

35      GCRCGGWCMWGTAGCTKSRRYYKYYAAGGTAGCCACCCRKRGATARAMSKKWGKRRYKRW
44      RSGTAAATCWRWTTYKKSGRYYTTTTGTACWCTARYYAGKRACAGGGMSKKWTKRGCKAT
         .  ..   *  :  .** ***.  :. .    .   . **..:. .***** **  *

35      TAGGRMGGCRWYKAAKKMRCKSGCMYRCYMYGCMYYYAWKMKYAGAAMTCAKTGMTAKRY
44      WTRAACAATGTTKGRKKMRMGSAMMYGMCCCSYMYYYTAKAGCRRCGAWYMTCKMYRKAC
         : .  ..    *. ****  *. **     . ****: *     ..    .  *  *

35      YYCCKKCTGTAKAKTRTYKRMYGCWAYYTKCCKTGTTKAKATWCKGATRRCRAGCKMGWR
44      CCYMKKTCSKRKTKCGCYKAACRTAWYYWKTTTWRYKTRKWYTSKAGGARMGRRYKMRTG
            **  .. *:*   **       ** *  .   .. *   .*..  *     **

35      TTYKRYMYYSMMRGTYCTGTTWTYAGGARCATTTGAKCYCGRGSKGGCCRYYKKYYRWMA
44      CGCKRTMYYSAARKWYYCRYYAKCMRTGGYGAGCACKTYMKRRCGKKSTGYYKKYYRTCR
           ** ****  *  *      .    .  .:  ..* *  * .   .  *******

35      WYRYRRYKYTTMMWAWTATYYMYGRGCMGCRATGYYCGGKGGAGTAKGGYSGKACTGYYA
44      TCRCGRYKYKYMCTMTYRCYYMCRARTARYAGATCCYRKKKSRKAMKRRYSAKWYYRTCG
          *  ****. *       ***         .:      * .  : *  **.*      .
```

```
35          TCTTAYRKKKYTRYKYYGRMYYYGYGAMYACGCYYKGATCAAGTCCKAKKCSCMTGKYRG
44          ATCYGYGGKKTYGTKTCTRACYYSYRGMYCMRMCYKRWATRRAYAMKTKKMGYMKKKCAA
            :   .*  **    *   *  **.* .**.    **  :   . . *:** . *. *  .

35          CTCTTYSMKRCKATGTCTAGKTCGCKMRKYCGAAGTCKRKKGRCAAGAWKYGAGTTCKCA
44          TATWWYCCKGYKCCACYAGRKATAAKMRKYTAMRAKYKRKGRRAGMACWKYCCRGGYKGT
             :   *. *  *. .  :. *: ..***** .  .. ***  *.. ..*** .    * :

35          GCYMYRCKYYYTCGKTGCRMYRYSTTTGKKCACAKWWWYRYATATGKWRTKYCACGWMWW
44          RMCCTRYTTYYWMRKKRYRMYRYGAWYTKGYRMTKAAAYRYMCTYCKAAWGYYMYKTMWW
                 * . **   *.  *****.:   *    :*   ***  :  *    *     ***

35          GCTGKCCACMKCKWWGCRCRKYRRMRKKTRARKYSKRYAMMRCCTGKYWRKKGWMKCRCC
44          KTGTKYACTMGSKAACTRYGKYRACRKKKGMAGTSKRCCCCGYACKKYWRKKKAAKYGSM
                * .. * .*    *  ***  ***.     *** .    .  ******   *  .

35          CTYYRKKMWKAMAKRAAAAGTGTCKAYATGTSCSGRCTRYGKAGYRKYWKWWSTGCCAGR
44          YATYGTTAWGRMGKRRRRWRYAYYKGCWGTYGYSRRYGRYKKGRCRKYWKWWSCRYTRAR
             : * .. *  *.**      .  *.     . * *  ** *.  ********     .*

35          KGKRCRCCKWRSTGCGYGGYGRYMYTCWYMYAKKYWYYSMRRTYGTKYYMMKCMWKWWWM
44          KRGGMGMYKTGGCAATYTAYKRYMYYYACCCWTKYWYCGAGGYYCAKYYCMKMATKWTAM
            *       *  . .. * .* ****       .**** .    * :*** **   **  *

35          RYRKWCGMKTRKYRYRKKMASYATGCTCATSWMRRMYSWKKAASWYCKTTRACTKTKGRR
44          RYGKAYAMGYAGCACGKKMRGYGWAYYYMWGWMRRMTSWKKTTGTTTKKYACTCKGKAAA
            ** *  .*        *** .*. .     .***** ****::.   *.  .  * *.

35          YYYYKAGTATKYGACYYGYKYYARYYRTKYRACMRKKMYTCAKRAGAYRAAWKGKMGCMT
44          TCTYKRRYTAKCKRSYYKCKYYRATTACKYRTAMRKKMCYTGKRGACCRRRWKRKCRYCC
               **   ::*   .**  ***      ***:.*****   .**... *  ** *

35          YTKARRYKAYKMYRYTKCTCMKKTCGKARKYKYRMAAAGMTKGTGKCMGGRGTCTGACCR
44          CAKGAGCKMYKMYRYKGTAAMKKATSKRGKCGCGMGWMRCWKRWRKSAKRRRYMYKRYYR
             :*.   * ******.  :.***: .*  *    *.     *   *.   *        *

35          YGWWKCCGCGRRGCYYCGMYAAGTWMGGYWRYKRYCACRTTAAAWYTCCTKRKCWKMWST
44          CRWTKTTKYRAGRYCTMKMYCGAYWMKRYTRYKACYMYGCCTTMACYYYKKRKMWTCWSY
              * *             **... **  * ***        ::      .*** *. **

35          AYCGKGSTGTRTMCYYKAATCTKRAKYRMYYAAMTKCKCYWRRTWRGCYKCCYMMMCTGA
44          MCMRKKSARCRYMMCCGGTCMYGAMKTAMCTGGAAGSGMYWRRCWRRMCGYSCCMMYCAC
                * *:  * *    .:      *  *  .. : .  **** **     .  **  ..

35          WMKGRKWWRGGTCGRKTKKKTMRYYTCKCWRKKGYGMMTYCAKWYYKMYYSGTAGKYRCG
44          WCKAGTWWRRKWYRRKYKTKKMRYYYYKYTAKKRYRAAKCMRKATTKMYYSRWWRKCATA
            * *. .***     ** *.*.****  *   ** *   .   *   *****    *   .

35          CWKTTKCKKKKAKTAGTAACAKKAKRMWTYATMKWKKRKGRYRGTGKYTRYSCYTKYYWR
44          GWTKKKTGKKKRKACAKTRYRKKMKGCWKYGKMKWGKGGRACGRKRKCGGTCMTYGYCWR
             *...*  *** *:...:   ** *  *.*..*** *       . *    .    * **

35          AGRCACGKMRAKKWGTAYYMARSYKSSTTCAAYKCGKGGWCAYKKYYYKKTWTYGCYRKY
```

```
44      RRRMRTRGAGRKKWAWRCTAGRSYKCGYKYWWYKTAKRTAMWTKGYYYKKGWATKTCRKY
          *        ***.     .****.. .   ** .*      * ***** *:    ***

35      GAGGGGCATGTCCSTACWAAAARKSYRKGTRRKYCMKKKCSTCYAKARTCYKTGTAKKAA
44      RMRKRAMMKKGAMSCWATWRRMGGGYRKRKGRKCYAGKGTSAMTTTWAYTCKCSCGKKMR
             .  .  . *   .      .*** . **    *  *:  :.     * . .**

35      GTTRRGMGAKWYYKATKMSSTMGCCCCYMGKRMTTKCGTGGAYGTGTRRKMRSGGGGTKY
44      RKYAGRCARGWCCGCGTCCGACRMYTTYMRTGCCWKTKGCTWCRKRGRRKMRSRARKKKY
         .     .  *   . . ..:      ** .    *        .  ******  . .**

35      KAACSWCCRWAKMYARWKTRMWMCWMMKYKMYSSCKMCRKKKAMRRYRCTWCKCYACWGK
44      KWMMSWYYAWGGMYWRAKCACTMMWCAKYKCYSSYKMAGKKKGMAGYAYKWYKYCWYWKK
        *   **   *. ** * *    * *  *** *** **. ***.* *  .* *     * *

35      YKTWGAKKTGTGCKKYYAAGGTAGTCYARCAGYTATTAAYTAATTAYYRYRKMRTCKTKT
44      CKYWARKKGKKSMKGCYRRRKKRKWTYCGSWRCCRYWGMTCGCAGRYYRYRKMRWYGCKW
         * *. **  .. *  *    .    *. .       .   ..:  ********    *

35      TCAKRKRKYKTKYKKKGRRWTTGGTAAWRCTRKCRKTAGTATATACGKYYYYCWRCGMMA
44      YYMKGKRKYKCKYKKKRAAWYYRCYGTWRYCRKYGKCCKYRYCATSAKCYYYAARYAMMM
           * ***** *****   *     .:**  **  * .   .::..* ***. * .**

35      MTKRKTTWMWKTCTAYKTATSAWRYRARWTAGKTTTCKWSTATYRTARRYRRRATGYRTA
44      ACKGGAYTCAKWTWGTTWMYGGTRCRGGWKMAKYGCTTAGCWYYGCGGRYRAARWKYAGT
          *  :    *   . .   .. * *. *. .*    . .   *  . ***     *  :

35      KTTTKGKYKKTCRKCTRKGAKGCRGCMRYKGCTKGTGCCRYRCAGYACKRWWGGYYRRYK
44      GGGKKRKTGKYMRKYYRKSWGAAARACRYKKYWKRGCGARYRYGAYGTKRWWTKTYGRTK
           .* *  *  **  **.  ..  . ***   *    .*** ..*. ****   * * *

35      GAWRWGKYYMKMKYYGGATWKRCGGYTYRAYGTGTAYSRTCCRCKWYRCRMGYMYKTTKY
44      RWTGARTYCCKCKCCKRRKAGRTTTTATRWYAATCRCSRWYTRGGTTRAGCKYCCKCAGC
              .*  * *     .  *    : * *.:    **   *    *.   *  * :

35      GKYKGCCGGRYYRKGCMYGAGGTGACTCTCTATTCMRCCYSTYWRRRCYMWMRKGGCATT
44      KKYKTTAAAATCAKKYMYRRRCCKRYYACTKWAWTCAGYYCWYWGRRSTAWMRKRRGCKC
         ***  ...    *  **         .  . :      *. ** **.  ****   ..

35      CCAGCGGTYKYRYWACATKGATRGYWTCRWCACKCRRTRYTKAAATMRMWCAYACTAAAT
44      TTGSYKKYYKYRTWRYRWGSGYGAYAYTATAGTTMGGWATCKGTGYCRCWYGYMSWWRWK
          ..    **** *     ..  .*     .. .       *.:.  * * .* .    .

35      RGSKYTTGWRTYKRGTGTTTRYMKKWGMKWYRYRTTAATAKCCACTGGTACWTTCTTTYY
44      RSSKYGKTTAYYTGRYACGGRYAKTWKMKWTGTGKCGTGTGAYRYYKKYRTWCATGKKYY
        *.*** .    *.   .   ** *.* ***    . .: : .         * :  ..**

35      CKMCAAYSAAGGGKGTGTYYKYMWTYRCCYTMRTGKMYYRTMYRGKCCKMWSKKGCGGCS
44      SGAMWRYSCGCATGTWAKCCGYMTCCGYYCYCAAAKCTYRCMYRAKGYKMTSKKAMKKYC
        .     **.. .    ..   **          :.*  ** ***.* ** ***.     .

35      KKRSKCGSKYRRRRWKKKKWWCARYKTACACKCAAGRKCRMAMMGSCGAAGTCKGGARKK
44      KKACGMRGGYRRAATKKGGAWTTGYKYRMRMKMRRRGTYGAMMMTSYAGGAYMKSKRRGK
```

```
        ** .   . ***   **   * : **     *     .    ** * ....  *.  * *

35      KKKRYGYRMMMACACYWGAKKWRAKCGAKKYYKYAGYGGKRTGAGKKARKRRAKMCRAKK
44      KKKRYRCRAACRTRYCATWKGWRRTAKGKKCCKYGRYTAKRYTTAKGRRGRRRKCYRWKK
        *****  *           * ** .. .**  **. * .**  :.*  * ** *  * **

35      MKTRRKKKYYKTCAGAYKKYYWGYAYCCACRRYRTCGGYKYCRGKMYWYRGATMGTRKCT
44      MKYRRKKKCTKCTTAGYGGCYWKCRCMMMYRRYRYYRRTKCTRRTATATGCTCCCAAKTC
        ** *****  *  :..*   **        ****     *  * .      :   : *

35      ACMTGWGGGTRTTRYACGCGYYGARCKRTTCKYYKKMAKGGGGYYWYAMCCCGYRKACRG
44      CTMCKWCTTCAYYRYWMRTKYYSGRYKGYYYKYCGKMRKRRKRYYWCCCATTKYRKRSAS
        . *  *       **     **..* *    **  ** *    *** . .   *** . .

35      KRYKKARRRTMRRYRSYSGCTRKKRYTRAGGTWGYYYATGCCYKYTARGKYMKAYMKWTC
44      KATKKWGGGKMRRYASYSSYWRKTGYAGWARYAATCYRCRMMCKYWMGKTCAGWCMKWGA
        *  **    .**** ***.  **. *:  .   .  *      **    .     *** .

35      TTAGTTKGKAGYKYYKYGGTAACYYRGCKKMRTMTGCYARCCYTKKRWTTKWRAKRTTYM
44      WGMRYYTRKRKYKTCKYRKYGGMCCARYKKMRYMKKTTWRMYCYKKRWCCKTAMKRWWCM
              . *  **  **   ..      **** *.    *    ****  *   **   *

35      KRKKYAMKKCTGGTAYMTGYCGCKKGATRMYYRRRGGKWTTRRWYWTGWGKYGRSYKGCG
44      KRKKYWMKKYKRRWRCCCCTGCAKKTRKRMYCGARKKKWGKGGTYWGTWAKYKGGTKTTA
        ***** *** .           .**  .***   *  ** .   **  *.**  . *  .

35      KMACAGCYKKKKRTYTMRCGTCGCGYYWWYCMKWAYGAMSGCWCRCCWYRMWMKGTTGKG
44      KMRYTAMYKKKGGGCGMGYKWYAGKCYWWCMAKWMCAGMSCTWYRSMACGMTAKKCATKR
        **  :. ****     *     .   ***   **  ..**  * *.    *  *  : *

35      GYGCCGTARTAKRTAAGRRKCWRWAGCTAAWGKGGCYYRYGKWRKRSTTMMYRCCAKTCC
44      RYRTMRYRAKMKRYMMRGGGAWRWMTTGTWWCKTATTCACTKARKGCYWMMYAYYGKYMM
         *       . **       .***    : * * .      * ** .  ***   .*

35      ATTYTRCTYRKKKRAYMYYYKRGAAYKKGYKRKMCRRTYTGYCTRWAKATGYKGKKAGTG
44      RYYYKGMCCGKKKRGYCYYTKRKGTYKKATKAGCYRRACGACYCRAGKGGRYKKKKGRKK
           *.     ****.* ** ** .:***. *    **:  .   * .*.  ** **. .

35      GRGWKGTYACGMRRYWKRRRMRARRTAGWMRATKTGYAATARACWKTTSYYRSYWYRWKG
44      SRRAKKKYCYACGGCAKRRAMACGRYWTWAGRYGAKYTMYRRRSWGYGGCCRSYWYRTKT
        .*  * .*. .     *** * . *   *     : *:   * .*   .  ****** *

35      TCAYGGGTCTYWKTATRCGSRGCYTACCTKWKGCTTYCAGGYGGGGYGTGRKCYKYWGTA
44      GYGYSRKCACYAKKRKRYTSAKYYCGYYWKWGRYYYYMTARTTRATYRGKRKMYKYWKYW
          .*.   . * *. .*  *   * .   **     * :.    . *   ** ****

35      GSWYKRGRGGCRWTAKYGWWSKYKGKGGRTCCATGSRKATGCMCRTTRYRCATGKCKKAY
44      KSWTKRAGRCARWKGGCRAAGKYKRKKRGAATWKKSRKRYRTCMGYYRCRMRYKKTKKWC
         ** **.   .**..     .*** *   :.  . ***         * *    * **

35      GCAKWMKGGTMCRKACGRGTWGAKYKKAYKYRYTCRWYKRAYRRCAYYGCKRWRYMARMR
44      RMRKTCTTAAMYGKRARGCYWCTKYKKWCTCRTWYRWYKGMYAGYWCCRAKRWGTMRRMR
           *  . .:*  * .    * :****  . *   ****  *       .***  * ***
```

```
35                  KRYKRCRRWKYMYRKKCARRRYYYTGKYTTGWWKGAKGRCRYACRGKRAGRRGWYWGKKK
44                  TGCKRYRRWKCATAKKYMRRRYYYYRTTCCAWWKATKRRYRCMYRKKGTTAGRACTRKKK
                    .  ** ****    **  ******   .  .***.:* * *   * * :        ***

35                  AAAGMKKCKTRYSWRKGRYGKKKRKRYMKRKGKCWKCMYYRGRYYWKSYMAC
44                  WGTAMKKMKWRTCWGKRATRKKKATACCTAGKKYAKTMYYRAGYYWKGCMRM
                     .:.*** * * .* *    ***  .   .   *  * ****. ****. *
```

The pairwise alignments of two accessions 35 and 45

```
35              YRYRWMSWYRMGCYYMRKRRSATKYMYGYYYYKYWKYRYMGMRATKRRRWAATGRYGGGA
45              TAYRWMSTTGCKAYTMRKGRSWKKYMYRYYYYKCTKYGCCTAAMCKRRRWCRYKRYAAAR
                  *****     .* *** ** .**** *****  **        *****.   **...

35              RYKRSMYMRYYYRKTKYRYRYSYRYKRCWRKCCYCAGYYKKYTRKGARAWMRYYYYYWWR
45              GYKRGCYMRYCTGGGGYGCACSYRYGGAWRKTTCMTCCYKKTWRKKMRRWMRYYYYYAAR
                 ***. ****      *    ****  .***    :  ***  **  * ********  *

35              RRWRKSRKYRKYMMCTCTMCAYTATARTCWKCTGTGCRAYKWMMRSTKRYMMTTTCKRWY
45              RRWRKSRKYRKYMMTYYACTCTYTCGRWYWKYYSYTYGTYKTMMRGWKRYMMYWKYGRWY
                **************   :  .  : .*  **  .    :** ***. *****  .  ***

35              MACYYCCKGGWRRWRKMYMMRMMYRRRYKYMYRRYAYMTKYRKMGRYRYRCKRRYMRRTR
45              MTYCCTTGRKARRWRKMYCARMMTAGATGYCCRRYMYMGGYGTMRRTGTRAKRRYMRRYR
                *:         *******  ***      *  *** **  * .* *   *.******* *

35              WRYYKRYRYTYWYWWKMYTCWYKRMAARWAGRYYWGGTRWYRRMWYYRWRRRCYMYKYRT
45              WRYYTRYGTYYATAAKAYCTAYKRMWWRWRKRYCWKRWRWYAAATCCRWRRRTCCYTYRK
                ****.**   *    * *   ****  **  ** *   ***      *****   *.**.

35              TARWMYRRYYYAYARKKWYMYYYYACWRYWYMCKWWRKWYRYKKYRWGTGCGTCGRRYYY
45              ARRWMCRRYTTRYWRKKWCMCCYYRYWRCWYMMGWWATWCAYKKCGWSWKTAATARRTCC
                : *** ***   * **** *  **  ** ***  ** .*  ***  *.   .: .**

35              RRRYCRYRSYYYRYKRMWMWYRMKRGYRRKGYYMKMGMMKRSRRWTKAGKWYKWRTTGTG
45              AARYYACAGTYYRYKRAAAWTRMKRKTARTRYYMKMRCMKRSRRWAKWKGTTTTRCGTGR
                  **    . ******   * ****   *. *****  *******:*     . *

35              GTYYKRYRRSGCYWGWMAKYTYRWMTCRCTCKYMWRMYRRTRMYATACRYYYKRSMRTTW
45              KCYYKRYRGCATCAAWMRKCYYRTCGTGTAYKCCTRCYRGKGACGGTAGYYCKGCMRWCW
                  ******  ..   .** *  **      : *   * **  .   . :. ** * .**  *

35              ACACRRKYAYKAKKCGCRCGKRMTGGRGWGRWRWTRCATACGKGYYYYWCWCCGGAGKTA
45              WYMTGRKYWTKGKKYRTAYAKGCCAAARWRRWRWCRYCATGAKRCYYYWAAYYRRMAKYW
                     ***  *.**     .*   ..  * **** * .:: .*  ****.      .*

35              MTTGKARKKRWMWYTCTTRYKKACTTAGRKTATYRRAWTTGTTTTCKWSTTGRWRGCRWT
45              ACYAKWGGGRTCAYWTCCRTTKWYGWMTGKYRWCRGGWKYAAYGCTTAGACAAARKTGAC
                   .*    *   *    * .*       *    * .*. .:    . .: .  *

35              MKRCYKYRRMRTKYKKKKCWTGTCGCTAACATYAYGGCTRGAMWYYCTKKKCWTYYSKCC
45              CKGTYGYGRMAGKTGKKKGTGAYMAGCGTMTACMYAKYYRSWCTCYYWKKKGAKTCGKTM
                 *  * * **  *  ***   .  .  .: ::  *.   *.    *  ***  .  .*

35              YGGTACKKGYGAKTGCWKRRSKCYRRKKMACATYYACCKRCGTGCCGAGGCRAKMRKYKW
45              YRTKMYKGKTRWKCATATGGGGMCAAKKCRYRWCCRMYGRTTWRYYRTTATRWGCRKYKW
                *  .  *     * .  .  .     **           *       : . *   *****

35              YARRCTKAYMRYWSMGRYRMWMYYKCGGYKTYYYKKKGKCTGGKRKAAACWYSARKCYTA
45              YWRRTATWYMRYTCCRGYACTAYYGYAACKCCCTTKGKTMCARKRKTTCTATCTRGTYCG
```

```
        * ** :. **** .   *    **  .. *    .*  .  . ***::.   .:*  * .

35      RKYWYATAGKCKSMKKGCWSWKACCYGTCTGRGAWSTTARRYWKKAYKYAKRKCAACKCS
45      RKYATTCGAKTTGAKKRYAGTKRSYYTKTAAGTGTCYKWAATAKKRCKYTGRKYWGTKTG
        ***  : ..* .. **   . * . * . :.  . . .     **  **: **  . * .

35      AKWWTKSWYWRASRYSRCATWCTRRWRTATYRGAYRWYAAYSMMGRKGGCTCYAYGWRSY
45      RTATKKGTCWGWGRYSRMTGTAARRAACGWCAKWTATCRGYSMMKRKRKMGATTCRWRSY
         .  .*.  *  .**** :  .:**   .          .**** **    . :  ****

35      RRSKGYRYKWSYGAAATGKKRYYRKTMMAAMTRSMRRRKMGKGWYRMRRMYYRYYYTKYY
45      GGCKAYGYKTSYAWRMWRKTRCYRKGCMCTAKRSMRRRKMRKTWYRMGAMYYRTYYYKTY
          .*.* ** **.     *.* ***  *.: .******** * ****  **** ** * *

35      YARTWGGRKMRSGGKKWAKYRKRKYGAAACSATCRKRMWAKRYRARYAGCKKRMWWMKKK
45      YCGGAACAKMRSRKKKWMKYGTGKYRMWMMSTCAAKRAWWKRYRWRYGTTKKACTWCKGK
        *.   .  ****  *** ** . **     *: . ** * **** **.  **   * * *

35      MYYSSAKYWKTKKMKRRRGRRKWKCCCGKKYWCYRCKYCTTTWMYYCKAKKTGGTGASKK
45      CYYCGGKYWKGKKCKAAGKAAGTKYMMRKKTTYYGYGYTCCAWMTYMKRKKGTRWCCCKG
         **...**** ** *        *    **   *   *   :** * * **     ..*

35      YYGKTKYRMRGTKCYYGGTTATTARYWYAGAYRYRKCMRMRWKKMCWYYKMKGCCRRTRY
45      YYKKCGYACAACKSCCKRYWRYWGGTWCRRRYRYRKMCGMGTKGAYAYYKMKSYMRGWAC
        ** *  *   . *.         .  *    *****   *  *    *****.  *

35      YSYWKKMAKRCCRTMKYTAGGCCTYMRAGCMWRSKATKRRYKKACGYMRKMRGWYRTAAK
45      CCTAGKMRKRYMRYCKTCGARMSWYMGTKYCWRGGWKKGGYGKGMKTCRKMRSTTRCTGK
         .   ** **  *  *  ..  . ** :   **.  .*  * *.    ****.  * :.*

35      CGKATCCGTYYWKCRKRRRTRRRCYKCGKSTRYTWTMTKGCCGYGRTCTCGCWYKRCTTT
45      GRKWGYTTCYYTKTAGRGGWARGATKYAGCARYCTKCGGTTTTTKAYYGYRGWTKGSKYC
          *      ** *   *    * . * . .:**  .                * * ..

35      WYTTATRRAGTRWKGGMYCMKYMTAKTYCGRCACMRAYRYAAAKKATYAARTTKGKRKYC
45      ACCCGGRGTTKGAKRKCTYCKCMYTTYYMKRMTMARRTGTGTGKKRYCGWRKCGRKAKYM
            . * : .  *      * * :. *  * :  *    .:.**   . *.   * **

35      TKRYYRTCCYYKYCGKCTTMGTSYTYGRTRYKYWGWGAGRKTKWYYYYMYWRRMGCACYY
45      KKRYYRYYTTTGCTAKAGCCSWSTCTKAYRYKYWKACRRRKYKWYTCCMYAGACATRTYY
        .*****        .*.   . *      *****     ** ***   **    .   **

35      WYYYRKRGKRKGGGAKRRRMMKMYRRMSYSAGCTGKRRACWWYARRTYCYKACYKRMWWY
45      WCYYRKASKRGRRRGKRRGMMKMYRACSYSWSYWRTGGWMWWYRRRCYMCKWYCKGCATC
        * **** .**    .*** ******  *** .   .    *** ** *  *   *

35      MSKYRMTCWGTGATTGYATTAGTGTRMKAYYKACKYKYKGYKYKKMRYMRTTKWKTTKKR
45      AGKCAMYSWCGKRYAACCWYRACACRMKRTYKRMKTCKKYKCKKMRYMRYKKWKCCKKA
         .*  * .*     :. .   . . ***  **  * * * ** ******* .***  **

35      RTRTTTTKRKYGCTCKGACCKCGGKYTATCAKGRYRRKARSRYCAGMAAKRTYYKYAYYT
45      GWRWWCYKRKYKYKMKCGTTKYRRKYWRWYRKTRYRGKWRSAYTWAMGTTGKCTKCMTTA
          *    ****  . * .  *   **     * *** * ** *  .*.:. .  *    :
```

```
35    CTYGTCTATCGGKAKRRGKRYAGKRRGGGTCCKYRMKRWYRYKCAAAKRGGKYKKKWRCG
45    YCTRYYGCGMSKKRKRAATRYCCKRGRRRYYYGTAATGWYRYKYWMMKRCCKYKKKAGTA
             .  . * ** ..**. **           . *****    **  *****   .

35    GWKYGAKKKGYYRYYKYYSTAAAGGKCRTMCRKKGATATGYRACSKKWKRTRYRWTRGGR
45    AAKTARKKKTCYRYYTYYCCGTGTAGMGCCTGKKRMWRYRCGMYSKKWTAYRYATCRRRA
      . * . ***  ****.**. .:. .       **          ****.  **   *

35    MAGMGGCCYYKMKMCTGCYGCMWYCGTYSYTWWKMTGRKRWMKCTGAKYGGCCKYWYKRM
45    CRKCAATYYYTMKAYAATYAMMWYSKYYCTWWWKAWRRKAWMKTCCRKYKRYAKYAYKAA
          ..  **.**  :. *. ***.  *.  ***   ** ***    **   .** **

35    YMGCTYYRCWGKCTRGYCTKYCCGTTKKKYARGRTGGGYGGCYTRYTKCRKRYKGCTYCY
45    CCRYATTGAWKKYYRSYYWTYMYRYKTKKCGGRRKRKAYSTTCGRYYKYRKRCTKTCCTC
          :   .* *  *.*  .*    ..** .  *.  .*.    ** * *** .

35    WRCAKRYCAGKTAAAWGYYTATRRYKCGYMAWRRWKKYAGTMATTGTTCAAATSAGKARY
45    TGYRKACMRTKYWRRWAYYWRKRGTTTRTAGTARWGKYWKYCRYYRYKYWWCWGWRGRRY
          *     *    *.**  .*  .    .  ** **         .   . .    **

35    KKTKGKGKTYCKTAAYYTYGCKTTCTCKARGTKAAAARKSRTAYMKKSTAYKTTCARCGT
45    KGKKKKKKYCMTACWTCYCAYGYYYGAKWRRYGWMRMGGGRYRCAGKSARCKKWYWATRC
      * .* * *   .:.     .      .* *         .*     **:  *.

35    ACGTAWKKKKRTRGMGGAWRWSWWRACCAASRTCGGKRMGKSKMTGGTGYWAYRKYYCKM
45    MYSCGAGGKKRYGRCACGWRWSWTGTMMGCGRYMRATGCKGGKCGTRKRCTGTGKYTYKC
        . .   ***    . .*****  :  ...*   ..    .*    .   .  **  *

35    CAWRTYKCGYYYKRTRWKKYMYSYMYSMRGTTWRYRKCKRTRGWCSTYKKCRYYKGYYKW
45    YRTGCYKTACCCKRCAATKTATSCAYSMRKWYARYRKTKRYRRWMCYYKKSGYYKKTCGT
           ** .   **   .*   *  ****    **** ** * * . ***. ***

35    MAWYYCYRRYKMCYKTMCWAWKMTTYYGAARGYTGCCYMGCRATCTYYTWGKGKAYSGAC
45    MRTCTTCGRYKCGYKCCYTMTGCYCYYTGTARTKRMTCARYAGAMGYYCTKKKKTYSAWY
      *       ***  **          ** .:   .        .:  **   * *:**.

35    TGYGTGACRKKRKKMYKRAGTGCTRYKCYYGCMYRYYGYKKKMYCYYRTCRCCYYYKKCC
45    YRTRWRRTGGKRKKCTTGTAACMYGTKTTCTYACGYYSYKKKMYMCCGATAAACTCKKAM
                ****  . :.:     *        **.******    :  ..   **.

35    MTGKYRGCTCSMTGAGAGKRTRATTRCAACMRAKRTTAGAGTCYYYCWGRCGGGKYWWTK
45    MKKKCAATATCCARCRGCTGCRGCCRARGTMRRKRAACAGAKYCCCYWKATTKKKYWWGK
      *. *  . : . : . . .  *.  *. . ** **::.....     *      **** *

35    TAGKMWMRRWGATRRKRKYRKCCSAGYSWWSYYKAWGCGRRTRYRYCCTTACGMGGACYM
45    KGAKATCGGTSRKRRKRKYRKAACGTTSWWGTYKMWTTARRCRYRYYYYWWTRCTTRYCC
      ...*      . .********....  ***. ** *  .** ****

35    CYKMGTARTCTYWRRKYGSWMKWKYRMGCWCYRASGKGTCGKTACMYCYTWKKRCTRTCW
45    MYKMRYWGWYYTTAAKCRSWAKWKCRMCTWYCGWCRKRCYAKKRYMYYYKAKKRTCAKYT
       ***           *  ** *** **  *    . *   .*.  ** *. ***   .
```

```
35               RATAAKYCYKGKGGCYYAGRYGKTAWCGKRSGYMMKKKRCCTYTKYYYYYYYWRAKTTTR
45               RWYMMGYAYKCKTATTCRKACKGKWAYRKGCKCMMKTKAYYATCTCTCTCCTTATKCGKR
                 *     *.** * .          .    * .  ***.*    :  .         :*  .*

35               KRAKYRWRMWTKKGTGAGKTRMYYARKGACGGAYTTGGYKKGKAYWKWAMGGTRYYYCRY
45               KRGKYRAAMWWKKAACWKKWRMYYGAGRWTTAGCCAKRYKKKKGYAKACCKKKRYYCYGC
                 **.***  ** **.:    * ****.        ..  :  *** *.* * .    .***

35               WCRRTWSKYMGTRKRTTGAGGKSMRYRGATKTYKCAKKYAKTWTRSYWYMAAKYRAGGGT
45               AMRRYWSKCMCAGKRYWKWRTGGAGTAAGCGAYKYRGGYGGYWGRSYWYMWMKCGCKRKC
                   ** *** * : **         .     ..  :**     *.  * ******  *  .

35               KYCWKCYYKYTRGYTWMMSRTKYCCTCTTCTAATGWKCGCKGCTKAYGYWMWACKCTGTR
45               GCAWKYYYKCKRRYWWMMSAGKYTAKMWWSWGMYRWKYRYTRYYKMYKTTCTRMTTKKKG
                   .** *** .* * ****  ** ..    . .    **    .    * *        . . .

35               AYARYKYWGCCKKKKRGYWRWKMGTRCGRAWAKYGWWSTMCKRRAATKTGACGTYYYRCG
45               RTWAYGCWTATKKKKRRTWRWKCAAGTRGCTGGCRAAGCMYKRRRWKKWKRYKCCCCRYA
                     *  * . *****  **** .:     . .      . * ***   .*          * .

35               AYYCKKWRCRKGYKTGAACATMRGCCKKGKKCGRGCMWYYTCGTTTYYYAKKWMTGWATR
45               RTCSKKWRMGKKCKAACGTGCMGRYYKKRKTARGRYMTCTKYACGAYYYCKKWMCATCCR
                    .****  *  *:... . *     ** *..     *    . .  :***.**** . . *

35               YKCYTTAAKTRRCCKRCTKYTGRRKRCKMKGTAGKKGSMKKRKAYRMYYKWRASCKCWRY
45               YKTCCCTTKYGAYYKRYKKYWKRRKRMKCKKYMRKKKSMKGAKMTAMCTKTRWGAGMWRY
                 **     ::*      ** .**  **** * *    ** ***   *    *   * * ..  ***

35               MMCTGAWMKRYWWRYKCGWRKTKYRKKKWMRYYTCKCRRKGAAMRYCTSKYTTGWWYYYY
45               AMYCACWCKGYWWRCKMRTGKYKYRTKKAMRYYYYKYARKRWWMRYMYCTCACTWWYYYY
                  *   ..* * **** *     * ***.** ****   *   **    ***    .. :  ******

35               SGTAGCCYCCMCATKCKKKTAGSRAKACARMWKWGTYAKATMAMYKMKGTYYYYGWKMRR
45               SRWWRYTCMTCTTKGTKKGATCGRRKRYRGCWKWRKYWKGKCRATGMKRYCYTYKWKAAG
                 *            :.   ** :: .* *      *** .* *..       **    * * **

35               AGAACYYCAATGCKACGGGKACGAMKCKCMTKMYCRWACCMRKTAYTAYTGTTACSRYRY
45               GRMCACTMRRYRYGRSRTCKMYRCCTMKYMWKCTMRWGAGMAKYWTYRTCAAACTSRYRY
                 .   ..            .     *     . . * * *    **.. * *        .::. *****

35               CCKKCTATKACGYYWGCRMKCKRMKMRGCYTGTYTAACMTGARWCCGMRMYRYTCCCCKC
45               TTGKGCGCKWMKYYWAMRCTMKRMKAGAYYWTGTKRTTCAACRTTTKAACYATCTATMKY
                    *   . *   ***. * . ****   . *     . :  :..*          *     .  *

35               RTAARRYYKTGWTARKCRWYKWKYATCCMYGWGTGSGGKGGCCCMWYRCCYCMYRCYYKC
45               RYGMRRYYKAATKRGKARWYKWKCTGSYCTAATYKSKRKATYYMMWTAATYTMYRYCYKS
                 * . *****:. .   *.****** : .     .      *  *.      **   . * ***  **.

35               AYKYWASRTCTYCRCTKCKWYWTGGTRCWMYGWYYRMYAYCYAGGCCGCACKAATCTTGM
45               MCGCTTSRWYYTMRGYTGKWYWAAACRAACCRTTYRCCCYTYMRTYAATRYKMMYMYWKM
                      :**      *   . ****:.. *.        **   .* *      ..     *        *

35               GCMKGTTTGACTYCASRMTKYMMCCMMRCTCCAYWGYYWCGCAYKGYKYTGGKYCCTTTG
```

```
45    SYATCCCYKRYYYAMCRMGGYMMSYMARGCTTGYWKYTWAATGYGSYKCGAATYYYCCYT
      .  .        *. .**  ***. * *    .** * *.. .* .**  ...*

35    TGWTMKRRYCAGCYRKATATGGCTTGMYYTAAATGACCRTWRAKRGTKCRYMRTYWMRRW
45    WSTCATRAYTGCMYGTRYGWRRMYYKAYYACRWKCTATRGTARTGRYKYGYMRYCWMAGA
       .   .* *  .   *  .   .         **:.   .  :. *      .     *   ***   **

35    CCATAAKTMRCCCACGCSTTRTTMRRYCTRMRTKTMKRYTGTYGKRMYCCACTRKKKMRK
45    MYTGTWGACGTAYRMKTGYGRKKCAGCYYGARGKWMKRCKRYYKTRMYTAMYKRKKKCRK
        : :  :    .       .  *..         * * *** .   * .*** .  .**** **

35    GKCCKTTCTTMRYRYKARRMRSWKGYWGRYKYMCGYTKAMYRTGCKKGRKRRRAAYWKKG
45    RKTYKYKMCCARCGYKWAACRCWKKTATGYKYASACCGTCYRCRYKGRGKRRRGGTTTTK
       *  * .     *  **     *.**      *** ..    : **    *    ****..  ..

35    KMMYAYGMKGCKWRYRMKGTKRKGRWSYWKWGAKYKYWYKRYAGCCRRYYCKRWRRWMCA
45    KMATWTTCTATKWRYRCGRWKGGCRWSYWKWRMKTKTWYKGTGAATAGYCAKRWGAWMYC
      **       .. *****     *    *******  * * ***   ...    * .***  ** .

35    GGCGGRMKKTGCRCRYCTKGCYTTWRRWKKYWWKKKYTAYYTGKCYKKKGAKYMWAGRCR
45    ASMRRAMTKYRTRMGCTCTATCCGWRRWKKYWWKKGTCCYYYRKTTKKKATKTATWAATG
      ..     *.*    *      ..     ***********    .**  *  ***.:*     .

35    CYCMRKRGRCGTGRAYKYGSKYRMRGWKKWTTRYKKYMGARKGTCAACGGCWMCMYYGRY
45    YCYCRKGTGTTCRRGYKYRGKYRMRKWKKWWCGTTKCCRRAGRYYTGTCKYACYMYYAGY
          **        *.*** .***** ****     .*          :.        ***. *

35    TAKSYMAACYRTKAMTRYKACKGTMCYKKRYYYKKKYAYTGWTAYRYMWARSRTGWKKAG
45    CTKGCMMGAYRYKWCWRCKCGKRYAGTKGAYYYGKKYGYYRWYCYRYCTRGSAWRWKGTT
       :*. * ..** *   * *. *      *  *** ***.*  * .***     *   ** :

35    YWYRRAWWYACASCKKKWKTKMYMTCCKACYWWYYMCKKYRAGMMYRRYTCAKGKKWKYK
45    CWYRRCTTCWGRSYKKKWGCKATMWTTGCTTTTYYASKTCRRCMMYRRYATGTRKKWGYK
       ****.      * ****  *  *     .    ** .*. *  ******: .. *** **

35    KYWYRKMACMGTGYWGYGGGKKRGKCATGKGKAKKYTATMGCKGAYYKAKKATCGYYYWS
45    KCWTGKMGYMRASYWRYTAAKGARGACAAKKKMKGYYRYARTTAGYTKGKKRYMRYYYWS
      * *  **. * :.** * ..*     ..:.* * * *       ...* *.**     *****

35    YKAAKYRMKMGWRTKYMYACRRYMAACGCGMMKWCYAKAYYSKYYKKGTKKYYMCCKCAT
45    CKCMKYRCKMKTRYTYMYCYGGCCTCTTAAMMGTYTRKMYYGKTCKTCAKTTYCGTKMCA
       *. *** **  * .***.      :.   ..**      * **.*  *. :*. *    * .:

35    TTMMWWMRYMMAKGRAGKCGCRYRCKMASRKMAMYSYRCACAKKAATTKTGTCATAYRRY
45    KCAATAMAYMCGKTGRKGATTACGAKMRGGKMRMTSCAYRYMKKTTCWKKKWYGCTYAAT
      .     * ** .*      .      .** . ** * *       **::  *.    . :*

35    YKKYCRRYYYAKCGAKAGRYYRTYTAATKYRATMRCMYWCAKKKKYRKRAATKKYCCTMG
45    YKKCSGGYYCGKMRWKRKATTACCCGTCKYRGGMRMMCWTGKTGKCGKRRRAKKCTTYMK
      *** .  ** .*    *         .: ***. ** * * .*. *  **  :**     *

35    CYCYGCCYWAKKRGRYCTCMYWKKATMTYKKKCTCTAAGKCKTATYYGCGYMYKYRACCC
45    MYMYRYMCWMTKRRRCYWMMYTKKMYMKYKKKYYSYGGSTGKYRGCCRMACACGCRGYTA
```

```
       * *    * .** *    ** **  *.****  . .... *       .     *.  .

35    KMCWATACMWRYRYSRGCTGAYGWWKAKCCCCGCCCGARRYRRMGTCTACTCKACYKCAK
45    KCTWCCCTMWRCGCCRRMYKRYRWTGCKTMYYATMYSRRRYRRARAAGCGCGKTMTTACT
      *  *. . ***   .*     * *  .*    .   . *****  :. .   *:  ....

35    YCMKCYYWWKTKYYKRKMMYWTAGCYTTTKTKWWWCCGRYRYYKKGWRTWKYTYRWKCCA
45    CTCKYTCAAKKTYYKRKMCCAKCRYYAWYGWKAAAYSSRYRYYKKKAAWAGYATGTKYAC
         *     *..******   ..  *:    *    ..*******      *:   * ..

35    CMYWYKWWGCRCRKYKKKRMYTTKKYAYRWYKKRMRMYGYAYWWCTKGWMCRCMMGGTRT
45    TMCTTKAACTRYGTYKKKACCAGKKYMYATYGKRCGMYKYWCTTGYKKAAYGYACAACRA
       *   *    *  .****   : *** *  * **  ** *      *        .. *:

35    YYRKKMKKKAKKTATCTAGTCGYASKTGKARCYAAAGAGKYRYKYWWWSTGWRYYRGTAA
45    TYGTTAKKKGKGGRYYYGAYYKYRGKYKKGATCTGCKGRKYACKYWWWSCTWRCTGAYMG
       * .. ***.*      ..   * .*  *.   :.. . **  ******  **   .  .

35    GKCYYCYGTTCKRKWRSYYMYCWKKWTKKYYKRCGCTGGGWKKKMRKKWGCAMTGCCGKM
45    AKMYYMTRYWYKGKTGGYTAYYAKKWCGGYYKRYRYKTTTWKGGAAKKWKYGAKATTTGC
      .* **      * *  .*  *  ***   ****   .   **    ***  . ..

35    RYMYGYRRRTYGGKWWRGKKYMTKTTAGTCYRKCRKTAKRRRYTTTMGTATWCCAKKYMY
45    ACCCRYRRGCTRTGTWRKKKYCAKYYGAYTYRKYGGWMKRRRYKACCRGCGWYYRKKYCY
           ***       ** *** :*  ..  ***     *****.:    . *   *** *

35    TTCKGTAYYTCTGKWKACMWYRAACTARGKTAGAWSMCWMKGTAAKTAGAKRGRWAWTRK
45    GCAKKYRYYWYCAKWKCTCATAGCACGRATCCARWSMAAMKKCWMKCCRGGRSRWTWGRT
        .*   **   .***.     ... .*.. .. ***. **    * . . *.**:* *.

35    YAACCATACGTGTKGKRWRYMYKGMAGCMKTYMGWYMWGYRCSGCTTGGYMCRYRSCGYK
45    YGRYYWYRMRCACKKKGWACCCKTMMATCKAYATTYMARTGYGRACCRRTAMACACYAYK
      *.         . * * *    * * .  *:*   **     . .          . .**

35    ACWCCGTTMGYTTRATKYRTTCACRYKYTTAAKTCTCCATACTAGGCTCCTAYWYGCARK
45    CTWTYACACKTAWGRCKCAWATGTGTKYYYRGGGYCTAWKGYYRACAAYYAGCWTATCAK
      . *  . :   :    *   : .   **   .     . ..   . .:  :. * . . *

35    RKMMCCAYGCAKRWKAATMCGGATTTMYWMYGTAGTSGMYYKGTCYYTCTGKAYYSKKYM
45    AKCCMYWCSSGKRWKRRGMTSKCGCCMYWMYRCTTCSACYTTKAYYCKYGKTGCCGGKCC
       *      ...****   * . .   *****  :  *. * . : * .   ..  . *

35    GGRMMAMRRAACTGYTGGKWRGTTTGAGCGGAKSKRCACTTCMKTWAKWCWAGAMRATGC
45    KRGCCGMRARRMYRCYRTGARKWCCATTSKKMTGKRMMYYYYMKAAGGTTTWKRCRTGAS
           .**            *    .: .   ..**      **: .        *: ..

35    TCYMYSKSMRTRYKYYKMGYYGGRYRGKCCKCAASATKSYRTCTTGCKKRSKCGSKRRWK
45    CYYAYSKSMRKRYKCCGMRCYKRRYRRKGYKTCCSCCKCYRAMKWTTKKRSKMRGKAATK
        * ******.***   *  *  *** *  * ..*. *.**: .   *****  .*   *

35    RKCKKYYCYRYTWCKYYYYRMRRMMMRGMYKTAAYTTKKRGYTAMMMAMYAYWYKMYRKY
45    ATYGGYYTCGTYWMKYYYYAMGGAAMARMCGKRRYKWKKRRCKTMMCRMYRCAYKMYRKY
       .   **     * ***** *    *  *  .  *. ***  .:**  **   *******
```

```
35                RGKTAKCGMWWYCGTAKKYGCGWRMRWYSSGKMRGKKRRKMACYMYAKMARRKYTKRCAT
45                RKKWRTYAMWWYYRWRKKYKSRWRCGTYSSKKMAAGGRRKCMYYMYWKMWGRKYGKRYWY
                  * *  . .****    *** . **   *** ** .  ***   *** **  *** **

35                TWKRRKKYWGATMTGKSTCWATTRTKKCGAGTCCKTWRKKYARYGYWRWRRYMRTMCRCR
45                YTKRAKGCWRMCCYKGSCYAMYWRCGGTAGRWYTKYWRKGYGRYRYWRTRRTCRKMYGMR
                    ** *  *       *      *    ..    * *** *.** *** **  *.*   *

35                YKKYYCTTTTRRKYYCMKRWMSRACCTTTCCACKTCKKGMWRCARAGCTAGTWKYRMAWW
45                TKKTYTYWCKRRKCYYATRWMSRRYYWGGTTRMGYMKKKCTGSTGMRMWWCCTKYRMRWW
                   ** *    .*** *  .*****             **    .:         **** **

35                RKKYTTCGAMMRYYKKYTYSYKYTKTTKCGTTAKRCGRTCKSWKAMYGCYYRKRMTAGTK
45                RKKCWYYTTMMRTCKTCKCCCKCCKWKTMKKGCKRMTAKTTSWKGACTYYYATGMYTAAK
                  ***     :***  *. . . *  * ..  . .**   . .***.    ** . * :.:*

35                GCRACMSGKWYAKCYKRTTT
45                RYRGAMSRTTYCKSCKRGCY
                    *..** . *.*. **
```

The pairwise alignments of two accessions 35 and 46

```
35              YYYYRMWRYYATWAYYTYMKMRRWYYKGCCGWYWWRRMAYMMRCKWYCTKRYCRYRYRRR
46              CCYYRMWRYYRWTGCCGYMKMRRAYCGKSYRWYWWRRMWYMMATKWCTCGRYYATRYRRR
                  ********   .   ****** *   .  ******* ***  **    **   *****

35              RRTTCKKKTARYYYRRKWWTTKMYYSCGTKKKGTRMWYMKRMRWMYYRCWKCGTTRMYYG
46              RRYYTKKKCGATTYRRKWWAGKAYYSYCYKKKAKRMWTMKRMRTCYTRYAGMRGGACYYA
                **   *** .   ******: * ***   ***..*** *****  * *         **.

35              RTAAAYGGWMTRRMTCGCCYMYRMKYYMRYMSWACYKCCRCAGARMRTTYRRRRYKWTRW
46              RCGRRTATTMGRRAYAKMMYMYRMKYYCRCMSWGGTKTAGTWAMACGCKTAGAGCKTKGW
                * .   .  * **  .   ******** * ***.  * .   .     .      * . *

35              RYYAMMTTAWTGAYMRYRKYYRKRRRYRRWYYYKKKTYTCCTGTWWTCRRTCCGKYRKGY
46              RYYRCMWYCWCATYMRYAKCTAKRRRCARWCTYKKKAYYSTCAYWWAAGRYTTRKYRKRY
                ***  *  .* .:**** *   ****  **  ****:* .  . **:. *    **** *

35              YYYKYYKKRMTARYKRSMCMRWAYWYYRYGMWAMMCRYKYYMTTTTGTKRWYYRRCGATM
46              YYYKCTKKGCYRGYKRGCMCATMTACCRYRMTWMMMRYKYYMYWKWRWKRWYYRRTAWKA
                ****  **     ***.          ** *  ** ******    . ******* . .

35              WMGSRRGGGARMWYCKWCKGARWYKMMRRKTKKWYASWRWMMRCGYWCWGWYWMRMMRRR
46              AARSRRTAAGAMACYKWYGRMRWYKMMRGKCKKWYMGWRWMMAYKCWMTRWYWMRMMRRR
                   *** ... *   **    ******* * **** .*****    *   **********

35              WCRCRRRRGCMSYTTKTYGRWYYGRMKRRMCYYYKCCKYKTGMMCKTMYYMYARYRRRWA
46              WTATARARCACGTGKGAYRGWCTKGMKGGAMTTYKTTTYGYACMSGYACCATTGYRRRAW
                *    * * . .  . :*  *    **      **  .*  . *.       : ****

35              ATCAKRKRKSKTKWYKGTRYRGKYYYYRAMRRRKYRYAWYTMWYYYWCCYSRWRKRKWKR
46              RWARKGGGKSKYKWCGRYAYGCKYYYYGRARRRTYRYWWYKCWCCCWYYTSRWRKRKTKA
                  . *   *** **     *  *****   ***.*** **. *   *   ******* *

35              MKMRYTCMKWKMRKSKRSRKKMYRSTCAYKWGYYWWMYYTYMRCMRYWKTRRTRMRKMCA
46              CGCRYGYCKWKMRKSKGCRTKMTRSYAWYGTTTTTTMYYYCCGYMGCAKKRRYRMRKMAT
                   **   ******** .*.** ** . *       ***     *   *.** *****.:

35              YMRMACGACRKKARCTAGAGYGKSMRKGAYTYRACTGWKTMWCTAACCTRSYWYKRKGTG
46              CMRMCATGTGKKWRYYWRRAYTKSAGGRMYAYRRYGTWGYMWYGMWYYKRSYWYKRKACA
                 ***.. .  ** *     .* **     *:**    *  **      .********. .

35              GKYWRYGYYKYKAATRWRTYMRGTCYCTWKKTYYGKMWTGKRKCGRACGAMGGATCYYMM
46              KGYWRYAYYKCGMRKRWAGTMAKYYYYWWGKYYYAKCTKKKRKMKAMMRRAAATATYYMM
                  ****.***    .**   *    *  * * **.*  . ***        ..:: ****

35              KKCKYRKKWGGGCTTCAARAKWTGKYWWSTGMYYYYRRATGGGTCSRCCTGACGAYYRCA
46              KKYKTRKGWKARTKYYWWGCKWKKGCAAGCKMTCTTRRWKKATGGSRYMWKRYKTCCRMR
                ** * ** * .  .     .**.     .  *    **  . .   **       :  *

35              TGYACYCYGCACATKYRKCGRWARCRYYAGCTKAGAAWYWAWYYTKTRCWKYSSMRGMAG
46              YRCRSYMCRMRTGCKYGKARGWRRMGYTRRYYKMKRRTCWTWCCCGCGTTGTSSARKMRK
```

```
                .*      . ** *.  * *  *     *      *:*          ** * *

35          SGCTCRYGRCRYGCRKCYKWRTACYKKMSKKKMMGCRYYYWMKWYYRGYRRKRYMYKTMM
46          CTTYYRYRGYGCRMRKTYKTRCCYYKKCCKKKMMKSGYYYTCKTCCRRCGRKRYMYKCMC
            .    **       ** ** * . *** .***** . ***  *   *   ******* *

35          CAWAWSYKATYYMGRGCAYMGCCWAAYMWGKKAYSGYKKGYTGKYMRCRKTTARWMYKRK
46          YWTMTSYGRAYYMRARTGTARYMTCRYMWKKKTYSACKKRTWRKYMRYGGGWRATCTTGK
                 **  :***    .      . *** **:**. **    ****          . *

35          TRYCYKTYYTMGCARYRTKKKMYCYYKTCRKKGGCCMYRGCTCTTSMTYAAGCYTYKRAT
46          YGTMYKCTCAAATCGYRWKKKMYMCYKWTAKKRRMMMCAATATWWCCAYCGCYTCCGGCC
                **   : . . ** *****  **   **    *  . :   . :*..       .

35          GTRARCAMRAKRYGTCTYRRKACGACKKRKYRYKYYRTCGMYWGCRMYRYSTTTGGGKKW
46          ACRRRAWMRRKGYAKYCYRRKRYKRGGKRKTRTTYYRWMRCCARYRMYRYSAWYTRKKKA
            . * *. ** * *..  ****      *** * .***        ******:     **

35          WWGRKWRTWKYGCYRCCACAGGKMYKKWWGCCRKKRRMGTTKTGRKYARWKAYKRACAYG
46          AASRKAAWAGYATYRYACTWAAGMYKGAACTYGTKRACKGGKATRKYMATKGYKGMARYT
              .**     *. ** ..  .. ***       .**     *: ***   *.**  . *

35          RKSKYGTAMMRTMMTATGYSKGWMKCRTTYKKMKKAAKKRATKYCTGTCGYATAGATGGA
46          AKSKYRWRCCGCMMKMWKYSKKAAKYRKATTTAKGRGKKRRYKCGYAYYKYWGCAGACRG
             ****       **.   ***   * *.: .. *  .***  *   .   *  ...:  .

35          CYRYSYMRRKTKTCTAGYKCKWYYWYWWSWRGGGGTMTTAAGKKRCTASYYRYTTCKAWR
46          YCRCGYMRRTYGYYWGRCKAKTTYWTWWSWRKRRRCMYWMRAKGGMYRSYYRTYWYKGTG
              * .****.     .  *.*  ** *****     *    .*     ****    *.

35          SKTYYMMYGTCCWKGYKYWTTKYYCTGAWKCKKMRKWTGRARGCCGKMRGKTMAGYMYRY
46          GKCYYMCYRYYYAKKYKYWWCKYCYKACWGYGGAAKWYRRGRATTTGCAAGKCTKCCCGY
            .* *** *     * ****  **  ...*      **  *.*.      . . :     *

35          RRRWRTCKWTWRGKKAGTKMKTTCGCTKTTCCRKRWRTRYTTTCTTTMTRRWCMAKCKCM
46          RRGWRCAGTCWRKKKMKYKCKYYMRYWKCGTTGKRWRKRYYKYYKACMWGGWYARKGKSC
            ** ** .   ** **   * *      *     ****.** .  .: *   *   * *.

35          YTTGRTAKGAGGYTCTTGKTAYYRYCKMYYRYATRRSCTKKGCWSMAKCGTGYRKRRKRG
46          YGCKRYRTKCARYWYKCAKGGTCGTYKMYYRYGWRRSMGKTAGWSMGKYKCRYRKRRKRS
            *   *  . .. *  . .* .     ******. ***  *.. ***.*    *******.

35          GTACRTAKTKRYKMCATACGTGTTCCKRCYTCWCCWYYKGCMYMGAWTCYYTGSCCCTTC
46          RYRYRWTKGKRTTMYWYRMRYRYCTTKRYCCTATAWYYGATCYAKMACTYTWAGYTTCCM
                * :* ** .*            **      .*** .  *      *  ..

35          TTYMCRRSGYTGCTAGCGRYYWTATRGTTKKCCRKAWKYWYYWRRGTSKTYKKGTGKYYM
46          YYTAMAACKYYKSYWRTKRTYWCRYAACWKKTTRKMATYWYCWRRKYGKYTKTSWRTTYC
                   . *  .     * **    .  **  **  .*** ***  .*  *..  . *

35          YKCMMWKWTWMRYCKACMKAACGCRYRKAMYMWASYKSRYMGYKAAWKCGTGCYRRYYYY
46          YKMAATKWYAMRYMGTYMKWMATTACGKMMTMWRGYKGRYCRTGTTTKTKWATYAATCTY
            **    **  ***  : **  .     * * ** .**.**    :: *   . *     *
```

```
35          KTAGTATYGAAACGKYYYRWKYKRSSGTCCCAYARYYRTKYRWYYACMRYKKMYRYWWWC
46          KGRRYTACKRGCTCKYYCRTTCKRSCAAMSMWYRATTACKYRWCYTAMRYKKMCRYWWWT
            *    ::   ..  *** * . ***..: .  *      **** *:.****** *****

35          AAYRKRAACRGYCCRKKYMGCYCYMGCMCACKRGKTCTTWAKKGTCYKYKTMYWYTRRAK
46          GRCGKRRRMARTYYAKKYMKMYMYCRYCMGYKRCKCYYWWMTKSYYYKCKWMYWCAAGGK
            .   **         ****  * *     . ** *    * .*.  ** * *** :  .*

35          MYRKKKRSCCGYYTARKYKYMMATMYRGCTGACRRCYGWWAYYKAKCRATCRGCGAGGCR
46          MYRKKGRSMMSYYYRGKCGCMCGWMYRRMYKRYRATCRWTRTTGCKTRWYTRRYRTTATR
            ***** **  .**   *   * . ***      *    *     .* *   *   : . *

35          RKWAGRYYKWTKYCYCTKAYMGGRYRMWMYYKGGCTYKTKARRRRKKKCTTCKAATYWWY
46          RKARKRYYKWYKYYTYATWYMKRGYACTAYYKRKTYCKCKWRRRAGKTTGCAKTTCYAAT
            **   ***** **   :. **   *    ***     * * ***  *.   .*:: *

35          SKWAGACRTGCRCKRTTGGAGSMKGSWKYYCGWSRRYCAGCAGGKRYGMTAAAYKYKRKC
46          CGAGCTGRAKARTTRWYSRGAGAKRGTKTCTATCAATMRKYRKTTGTACCGTRCGYTRGY
            .  . : *: .* .*  . ... * . *   . .          .  .  .:   *.*

35          AKMSKWATGKTAGTAAYTKWSRRYRWYGRRTCCTRSYRWRRTSGWGTKMYYKGGMRWRSM
46          WKMGKACCRGCRKWMRYYKWGRRCATCKRRGAACRSYRWGGCCKTAGTCCYKRRARWRSM
             **.* .         * **.**     ** .. *****   .  . .  **   *****

35          RSRRKTARMYKGKYRMRKRMYYRRYKYKRYTAYRTGRWRRYKAGTGKACGTMTKKYRYKK
46          RSRRTYCGMCGRKYAMGGAMYYRGTKTKRYKCYGCRRWRRCKCTCTKCGRYAYGGYRYGK
            ****. . *   ** *   ****  * ***..*   **** *.   *.       *** *

35          YAYTWGSYYRYYKYTAYRYMWTARSRSYRAKTYRMRYKWYKKYASKKYRWAYYMMYWWYY
46          YWYWARSYTGCCTCYCYRYCTCRGSACYRGGCCAAGYKWYKKCTSKKTRWGCYMMTTTYY
            * *   **    .  .***     * .**.      ****** :*** **. ***   **

35          MCAGYRAMKWGRAGKWKCYKKYYRKMGYKMGGAAWYGYMKAGRKKCWKRAGTTKYKMKKA
46          AMRRYRMCTARRRRGWGYCKKCTAKMRYKMTKRGWTRYAKRTGKTMWGAWSYYGCTCKKM
                **  .  *   *   **   ** ***   .*  * *   *. *   .    . **

35          RMKKTATWMGRGKTAKYYYWSKATMRWCCRKSKGCRSMCGAAGGWRCTATTACRRAMMAT
46          RAKGYRYACRGKKCGGYYYWSKCCMATYYRKGTTTRGAAARGCAWRYGGCYCTGGMAMMC
            * *         * . ******. *    **..  *. .. . .**  .  .     *

35          RKKKTYYRMYCCGTGACAKKKMRAAAYYYCTCCMGGYRYKYGARRMMRSKAKKYWGKYKW
46          ATKKKYYRMYTAKCAGTCKKKCRWWMYYYGKTYAKSCGYKYKWAACMRCKGKKTATGCTA
             .**.***** .  .. .*** *   *** .    .  ***     **.*.**     .

35          MYRYSCGCRAAKYKSMKKGKMMYAGGCKAYGMRYKKMTTWRYRMGRAAKKKARSGRRRRW
46          CYRYSYRYRGGKTTSMKKKKMATGRAMKWTKCRTTTCGCWRYRCRRWWKKKWGGRARRRW
             ****   *..* .**** **  . . *    * ..   ****  *  ***  .  ****

35          STACGAAAGKRTYYYYYYCTCCSTTACAACKGAGGKKAKYYTTGRYRGGGTKYRRRGYYY
46          SWGMATGTATGKCTCTTCMAYYSKCWYRGMKSRRKGGRGYCYYTRYRRRRYGTAGRTTYY
            * . .:.:.. .       :  *.    . *.       *    ***        *  **
```

```
35              YMAAAKMRKKKMARKRCGGWGRKKGKYRYGRRYYKYRYYYAAGGKCRTTAKGGCTRKATA
46              YMWMMKCRKKKMRGKGTAAAKRKKTKYRYRRRYYTYRYYCTGTAGAGCYWGKCTKGKMWR
                **   * *****  *  ..  *** **** ****.**** :. . .        . *

35              AYGKYRMMKGKGYTARRTYKRWGGRMAGGGAGGCRYGYTAGRRKMRTATKCCMGGCMYYS
46              GCRKTGMAKKGTYAGRRGYKATRRACRKRRRAATACSYKRKGRGCAAGAKMAATAAMYYC
                .  *  * *   *:.** **           ..   .*.   *   :.:* .  ..***.

35              YKMYYYKKKACCCMKRKRKKCTGGTAACCKAKTAYKRMYKGRTYYRCTKYTKGTTTYKKK
46              YKMYYYKGKCYYYMKRKAKKTCSCKRTTAGGKGWYKAACKRAATTGAYKYWTAYKKYTKG
                ******* *.   **** **  . . : . .*  **   *  :   . ** .. ..*.*

35              TRAWRCWATTGWTRRCKATRKKRWYWRCKRCASYSYKAWTGCGRRCCATAMAGAGKKMGT
46              WAMWAYWWYWKWGGGYKWKRKKGTYWRYKGATSYGTKMWKTTARRGAWCWMRKWRKKCKG
                   *  *    *    * .***  *** * .:**. * *.  .** .   *    **

35              YWYGTARWMTCYYWRRKYYTYTAARMMAYMTGCKRTKCKMWKKGKWRRATAAKWAGGCYY
46              CWTRYWGWMWYYYWAAKCCCCYGTRMAMCMKCTGAKKTKMAKKRKWARWYMMGWWTATTC
                 *     **  ***  *     .:**   *.    .* ** ** ** *     *  .

35              KGRYGGAWRKRRMMKAKRCCGTATTSKKKTTYRARCYCMGGWYYKAKRTTAKKKYMRKMC
46              KKACTKWARKGRMMTTKAYMRYGWYCKKKCCCRGRYCSARRAYYKRKRGCTKKKTMAGCM
                *       ** ***.:*     .  .***   *.*  .    *** **  :*** *

35              CGGACWAAYKGYKAATWKKKYWTGTKYTTYGTYCCRYGWGRTCYYKWGYGKCAYKYSRKC
46              TTAGYAGRYKRYKGTAAKKKYAYKKKYAWTRAYATRYRTCAYYCYGATYKKSMCGCSRKT
                  ..  . ** **.:: ****   .**:   :*. **       *   * *.    ***

35              GRGCCKWYWRCWSYYMYKYTCYKCAGAATCGAAGCCGCCYCRRGMMTGGCYTGACTCCRG
46              RRKGGKWYWRAWGCYCCGCYTYKMRRMRWYKRRTYAATTCYAGKCCWKSYYYKRYYYTGA
                 *   *****.*. *      **            ..           . *        .

35              YKCCYTSMYAGKCYCCYMMAKGGCARCGTCCAAYYGYMCMWKTYWCYKGAAYWTRWCTGT
46              YKAYYYCCYMAKGYYMTMARKRRGCRSKCTTGRCYTCCGMWKCTWMYGSGMCAYATMCAW
                **. * . * .* *   *  *   .*.    .  *    ***  * * ..        .

35              RKCCRRRTRMMCWMTRRKMGRKRAATTTCTRWRRTKRGTSCAMKCGTGGRRKGMTTTRRR
46              GGYYRRRWGCCMWMYRRTAKRKRRWKCAMKRTAGYTGRYSYWMTTAWTKRGKRMWYYGAG
                    ***     ** **.  ***  . : .*    .   *  *. .   * * *

35              WTATAKKCAKTACTGTTKMRRRRYRWWYAARCKTTCMKACCTTTCRMRTMRYWRKCCYGC
46              AATGTKGYRKYRTGRKKKCAGGGCATAYGCRTKGCTMKRYTAYKYACRACRYWRKYYCCG
                 :: :*   *     ..*         *..* *   **   :  .   *: *****

35              RACRWRYYTYATTCTATGGTRWACGGCTYKRMYYKGTCMCCYYYRYYTYRTYAMRRRKYT
46              RGYRTRYYWYRGWSCGKKRYGAGTACTCTTGCYTTKATCTYYYYRYYKTGYYTAAGRKYY
                *. * *** *   .  ..     . .    .  * . :    ******.   *:   ***

35              YTAGCGAMARYRGKKYMMMCATTTRCGAKYTGAKAACCTAYYAMRSTKTTCRKAACWSKA
46              YYGATCGAGRTRSKKCCCCMMYYCASKWKCYRTTMMGMWWCCMCRGGTACARKCRYASKG
                * ..  . .* *.**          .  *   :.          *. .: .**.   **.

35              ATCMCCYMATGYGYMTTKRRTKRTSTGCGRKYGCRYKCRTGCTAWCWYAYRRCACYAKGK
```

```
46              GGTMYYYMTGACRYCGCKRRKKRGCGAMRRKCAYGCTMRYRTGCWMWCCTRGMRTCGKTK
                .  *  **: .  *   ***.** . .  ** .   . *    .* * . *     .* *

35              YYWWAWGTTGTAKCYGRYMCMYYAYYTGTYCTKSYKGMKATTGKMRACGRYKRKGRYYKT
46              YCTWRWRYYAWRKYYRRTAYCYCGYYCKCTTCGSYKKCGRYYRKMRRTKRYKGTRRCYTA
                *  * *   .  * * *    * .**       ***       ***   *** . * *.:

35              AKATKGRGTTCSGMGRAAAAARKSATGAGMMMTARATAKYCYMKCMKKKCSTCCYKKCWW
46              CKRYKKRAKGASKMRAWWRRMGGGWYRMRMMMYRGTKTKCYCAGMCGKGTSKYMTTGYWA
                .*  * *.. .* *         .     ***   :.:*        *  *.   .  *

35              KKGTTTRRCGMRTCWRWYTGTTMSSATTCMGYRKGTGKCCKMYSKASMRKAYCCGRYYYK
46              GKSYYYAGYRMRKTTGWYYRYYCCGWCCAMRTGKKKKKYSGAYSKRSMRGWYTMRRYCCG
                 *.       **.   **     ..   .*   * . * .  *** ***  *   **

35              MWGTYYTRTTCCTGYMYGAKTMYRGKCCKMKCTTWSATKKRYRRSKSKRRWKKMKKKAWK
46              MTRCYYCGYYYYWRCCTAGKYMYRRTGYKMKYKKTSCCKKRYRRSKGGRRTKKAKGGGWK
                *   **           ..* *** .  *** .. *. ********. ** ** *  .**

35              CAYRYKTCCCYAGRRYAMMKGAGRRCYRKKKRKYRYCMMMYAKAYAKKMYRKYRACKCGT
46              TTYGYKYMMYYRRARYMAMKAGAGGMYRKKKRKCRYYMMCTRGRCRKKMYRKYRRMTYAY
                 :* **    *   **  **...   ******* ** **       ********  . .

35              CTKKYKRYCGGGWRRWTRKRKTWRAAKMACMAMMARTYCTKRAAGTTTGGRRYAYCCCAR
46              SYKKCGRYMKRRWRGTYRGRGYWRWRKCMYMWMMWRGCYKTRTWRWYCTTGACTCTTTGG
                . **  **    **   * *  **  *   * ** *   ..*:          :    .

35              RKKYGCMWYYCTGTCGTTTRYACCWMGCRGWRYKTTAAAAGTCATTYKAGYCRCTKKYTG
46              AKGCRYMTCTMKRYYACGAACCTTWMKAASTRYKCCTGTMRWYGWCCKMSTYRYKKKYWK
                 *    *    .   .  :  .  ** . . ***  :.:    .   *  .  * .***

35              CRKKCMGAGKACGGSRCKTTTKRYKAYRRMYYKKCYRMWRKKYMMMMCTGAKWMRRRWYC
46              YRKKMCKMRKGTAKSRMKCCWGAYKMTRAMYTKGMYRMWRTGCCMAMYCACKWCGRRWCM
                 ***     *. . ** *     ** * ** *  *****.   * *  ..**  ***

35              KCWGRARRYKTKKWMRYYTCKCWRGTTTCMGACCMMYWCGATMTYYTSTGCCKCCGCWRC
46              KYTRRGGATKYKTAMRYYYYKYTARYCWYACGTAAATWYKWGMATCKCAAYTGYTAGWRM
                *   *.   * *. ****  *          . .   *    *:  ..:.     . **

35              CSYGKCTGARTKCKKKAKACKAARMWKTYAKKATTYTMYKMKKRKRMRMRRTTRGWGTSY
46              TSYRKGCRGGKKTKKGRKRYKTTRMWKKYRKKGKYCAATGMKKRGGMRMRACYACWRWSY
                 ** *   . .* **  *  *::****.* **..  :   ****  ****     *  **

35              WYKCYRCGAYKYTACRYRRYYGCKRWRRKMARWTMTCKGCGGMKKCRYRWYMYRCKKGTT
46              WYGYTRYRRYKYWRYGYAGCCTAKRWGAKMRRTYCYYKSMRRMTKMGCRWCATATKTTAY
                **   *   ***    *     .***  ** *     *.   *.*   **     *. :

35              MMWMKCKARARRYYYTGKYKKKKGAKGRCRYCYGKRRCWYWGRAKAATCAKYSKKKAGCK
46              MMWCKYGMRCRAYYYYRTTKKKKATKRRYRCYTKKGGYACTRRGGCCCMMKYSKKTCKTG
                *** *   *.* ***  . ****.:* * *    *       *. ..   *****..

35              CWSTRCGWRYKKRKRYMKKATCWAGMYGCRYCCAKSYMTCCTSTCRATTKKTCAKTKATC
46              MWSCGYATATGKRKRCCKKRYYAGAMYATGYYSRKGCMWYAYSCYGMYCGGCATKYTWCS
```

```
       **   .    ****  **    ..**.  * . *. *  . *         .:* .  .

35    WGRYTGWYRCGGGARRYMRSCKRYRGTTKAYYYTTMGYCTTCCTARATCCTACATCCCKR
46    WKGYYRWYRYAKRMRRTCGSMKATRKGAKMYTYYWMRCYCYSTYGRMGTYARMMYYSMKR
      *  *  *** .   **   * *  *  :* * *  *     .  .*    :     . **

35    RACCRGCMWRCARAKWRRYYGGGTWKCAKWWRKKCYCRMYMKKCYYCSCYYTKATAMGYA
46    RGYMRRMCTGYMGMKWRRYYRRSYTKMRKWWRKKYYYRMYAGTAYYSCYCYCTGCCARTG
      *.  *         ******  .  *  ****** * ***  ..**..  * .. .   .

35    KRCWKTAARRTSWYGRKRMGRGCRMSGKWYAWTCRYSYWRKCARYCCKYRSSWTARTKYC
46    KRMWKARRRRKSWCRATGMAARYRMSRTTYCWYSRCSYWRKTTGCTTGYGSSACGGYGYA
      ** **:  **.**   . *.   *** . *.* .* ***** :     * **   .   *.

35    MCGCSMATGRSAKMMWTYYAYGAACSRRTWAAYGCARYKKKRMWGMWKMYAATAMYKKKK
46    AYRYCMRARRSMKMAWWYYWYRMMMSARGWGGYSYWRYKKKACTAMWKMCGGCGCYKKKK
          .* : ** ** * ** *    * * *..*.  *****   .**** .. . *****

35    MYSSCKMTKAGMKGRRGARWGCATARTAKCCWYCTTTRWAYKAAKKTAYAAKKTACCCYA
46    CYSSYKAKKGAMGTAGRWAWRYRYWGKGKYSWYYCCARWWYGRRTKGRCMWKGGRMTAYR
       *** * .*..*       *      ..* .**   :** *   .*     *     .*

35    RAKRMWYGTAAAYKYYYGACTWYYCAAYRRYRKTKYCCAACRRTCCAGGYYKYCRGTKMY
46    GTGACWYRYWRMTKTCCKGYYWCCGCRCRGYRKWGCMMWMYRRYYTCAATYKCTRAWTAT
       :   **      *    .  *   .  * ***        **   ... **  *. .

35    GATMGTRMTAGARGCCGTGTRYKKGYWYGRKCRTTCWYRMMYGYTYKYRGCAYYWYYYRK
46    CTCCSWRMCCRWAKMMCYRYRYTKKYAYRRKYGYYYAYGACTRCCCKYGATRYYWCYYRK
       :  . ** .          **.* * * **      *        **  .  *** ****

35    SKRGKRCKRYKWKAGWYAMMKARMMYMRRRYRMSYSAGCTRKTKRYRATRGYAKRKCTTK
46    SKASKRYKGCGAKGATTWMMKGGAMYMRRGYACSYSWSYWAGCTGYGWKRRYRTGKYCWK
      ** .** *    *..   ***.  ***** *  *** .     . *  .* * . *   *

35    KYYAKTRMWWYMSYRKKWGTTTTGATTYAYCMCGGRTCATTAGKYYCYCCGACRSKAGCK
46    KCCWKYGCATCAGCAKKWCGGGCRRYKCMYYMYRRRYYRKWRKKTYMTYTAGYASGGATG
      *   *       .  ***        .  * *   *   .   * *    ..  * ..

35    YGCAKKMRCTYRRTCCCYAMRCMYTWKTTKWKRKRTTKRKSWATACTCCCGGKTATAGRM
46    CRYGKKMRAATGACTATTWARMCCYWKCCKTKAKRWWKRKSWCGWYKMYYRRKWRYRTRM
         .****.:     .    *    **  * * **  *****.   .     *     **

35    RTTTCAACACCCTTAGTTKYAKGKKRMSGACTTATRCRGCAAKYYYGCGCKCGGGAGMYY
46    RWWYYMWYMTYYYWRRCYKTGKCKKGCGRGAYYCCRYGKYGCGYYYRARYGYRRRMAACC
      *                 * .* **  . ..  . *    .. *** .        .

35    TMAMTKTWMWRTTYCKYTGTARKRRAKGKTTTCTKWSKTTGCRWRYRGCTCTYTTRYRTY
46    YMMACKYTCARWWCYKCGAWMGKRGGKAKYGCTKTAGKACAMAARYGRTCYYYYCGYACC
       *   *    *    *  .   ** .*.*    .. .*: .   **      *   *

35    AATGRTTRKKYKKKCGCTAACAARGYCGGYYGCCRGAKMWAYGYYTTYATATTCCTAKRG
46    RRWKAGKAKKTGKKMRSYGTMTWGRCMRRYYKTTRSWGCTGCKYYYYTGCTWCYMCMKRR
            . **  **  . .: :       **   *.    .  **   . :      **
```

```
35      KCMYKCTGKGYWWCYGGACTKGYKWWCSTRRSYRRMCYMYYKMCGTCYYGCKGGACKCGC
46      KGCYKGCRGKYWWMYATGTYKKTKWAYSKRRSCAAMYCCYYKCTACTCCRAKKRRYKTKY
        *  **     *** *. .  *  **  *.***   *   ***  .     .*    *

35      YMWKGGAGRACTAYMRWKRYCTGYWRYTMRYAATTCYRCKSYKMGAKRGTYMYRWCRSTY
46      YCTGKKWRGRYYRYMGTKGCYCKYWGCWAACGGACYCAYKCTKMKRKRAYTATRWMRCYY
        *            **  *     **      ..:     *. **  **.    ** *. *

35      MAKYAGKTTRTYMWRSKKRYYMGCCKKKKYCRMKYTRRRGWKKKCGYCYWKRRTRTRCCW
46      CMKTWKTYYGATCWRGTGGYTCATGGKKKYYRMKCYRRRSTGKKYRCYYWKGGCGYGYST
          *   .   :  **..  *  .   **** ***  ***.  **    ***       .

35      YKRKKYTRYGTKCGTCTGKKTTKAWYYTRTGTRTRTRWKGGMYCMTTGGCAAMTCYRRYK
46      TKGKGTARYRWKAAGTCRKKKKGWACYCRYSYGCRKGAKRKCTYCCKTATGWMYMYRRTK
         * *  :**  *..    **..    * * .   *.  *       . . . *  *** *

35      TCYTMAMYCTKKGATACAKACSYTYTTKRTCRAGAACYWWYKATGACGKATTCTCCGWAG
46      YMYCCWCCYAKKARYGMTGCYGCCYACKAATGGRMCACWWTKRYRRYAKGCKTCGATWCR
          *      :**. . : . .  *: * :  .  .. ** *     .*. .   . *.

35      AGCKRMCMCCYTAYTGWRMCGMCGCCYYKCMTGGYCWYKMRRTYRWMMKGRMKGKACYAC
46      MKYGAAMMSYYYRTCATRCYKMMRTTYYGGMYRKYYWYKAGGACRACCKKGMKAKCATRG
               *. *    . *   *    **  *   * ***   : *   *  **.*..

35      ACATRMRMYRTCCCSTCAKRATYTRRCYGWTRCAGYRTKCWTTGWTRWYYMTSTYATMYC
46      TTMAGAACTACTATSAATKRMYCYRRYCKTCRTCRCRCKATYKRACGWTCCCGACWKCTM
        :  :        . *:.:**    **     * .  * *.  .    *    .:  .

35      KTAGCMSGGGWTTKGGGCMW
46      GCGATMGKRRAWAKSRKACA
          .. *.     :*.  .
```

The pairwise alignments of two accessions 35 and 47

```
35          TGAAWMRRMYKYYYYYRKWRKSRKRGYYYRRATWMMMTSRRKWMKYMRYKSYKCRRKYRW
47          YRRRWMRRMYKYYYYYRGWRKSRKRRYYYRRWKAAMCWSRRTWMKYMRCKSYKYRRGTAW
                ************* ******* ***** .  *  ***.****** **** **   *

35          KKSGAYYRYAAGMYKKYRKRSMGTYRYKWYCCCYCGRCMWRYKYATTKKCCWRMTKCAMY
47          KKSTMTCRTRRAAYKKYRKRSMAWYRYKWYYYYYAARYCWAYKTCAYTKYYTAAYGYRCY
            ***    *   . *********. ******   *..*  * ** .: .*          *

35          RYRCGSWGRYWWYTWKCYKCMRWYYWYKKYRKRKRYSGRWSKMYMMRRKYMCCMYRRKYR
47          RYGYAGWAGCATYGWKYYGACRWYCTCKKYGKRKRYSKAWGKMYMMAGKYMYYACARKYG
            **  ..*.    * ** * . ***   *** ******  *.*****  ***     ***

35          WMMWYRKRWMAYYTCRAGYCTYYRYYYWRTRYYYMAMTYRRKYGSYWRYRYTKARGRMMR
47          WCATYRKGAMGTYGTGWRYMWYYRYYCARKGYYCMGCCTGAGYCCYARYRYYGMRKRMCG
            *   ***  *. *     *  *****  *. ** *.      * .* ****   * **

35          TKRKMACCWKTTAGCRRRYRKTYYKMKRTRWRMKMGMRRYYKTGYYMKKYYKRMRYMRYY
47          GGRTMTAMWKWYRRYGRRCRKYTTKCKRYGTACGCTMARYYKYKTYAKKYTKRMRYMRYY
              *.*:. **      ** **   * **        * ****   * *** *********

35          YWASYYYWRKCCMCACGRGGYTYACMMRKCYTTTSMKGYGGTRWYACYKRRTMWGYYRKY
47          YWWSCCTWRKTTMMRYARRKYCCCYMMRKYTYWKGCKKCKRWRWYGYTKRRWATRCCRKY
            ** *   ***  *   .*  *  . ****    .. *     ***.  ***      ***

35          YYRSYCTCMYYRYRGTMYSTGYKKWYYMKACCARRMCYTGAKGTYWWCKYYMRWKTYYRG
47          CCAGTMCYCYYRYGRCMTGCATGKWCTMKCYYTGGAMTCATTRYYWWAGYCCGTKATYRA
               .     ****   * . .  **  **.  :      .:.  ***. *    *: **.

35          YYYYYMRYRWAARRSYMRRKRKAYYTYKYKAMCRWKKYMRWKKRYWTGCRKWKTRYMYSY
47          YYYYCMRYRACRGGCYMGRKRKGCCGYKCGWCTGTGKYMGTKGGCWCTYGKWKWACMCCT
            **** **** .   .** ****.   **        ***  *   *    ***   * .

35          MKRKGTKKRKKTMAYYGKGCATTRMWRKKATKKKRKKMRGKYTRRRGWKTKKMKAATCYC
47          MKRKRYKKRKKYCMTYRKRTGYYGCWRKKWKKKTGGKMRRKCYRRRSTGWGTMKGTGYCY
            ****  *****    * *  .    **** .**.  *** *  ***.    .**.:

35          YRTRTRCWKRRKYGGSTRYMKYTAAKGRTCGRRCTCGYMYSSWYTCRTRTGTRWCGGGMY
47          CGCGYGYWKRGGTKRCARYATTWWWKKAYYRGRSKYRCMYSSACCYGCRCTKGAYKRKCT
                   ***     .:** .    *      *..   ****      *  .

35          CKTYGTCCWKYRAYYYSRTCYAKYTMAMYTATAARKRYTKYTTKMMATWWTYYAAMRYYR
47          YKYCRWMTAKYRRYYTSGYMTRKYCCWCCARYGGRKRCCGYACKMMGCATKCCTTARYYR
             *       *** ** *     **     :  ..***   *: ***.   .  :: ****

35          RMYTCAKKKMRKGAAMATTAYCWKTGTCYMAKTCKRCCGAARRMCAGCSAKKYWGKGCKG
47          RMYYGCKKKCRKAWWMTYCTTTTKYRYGYMGKKMKGMMKRTAACYWRYCRKKTAATTGKT
            ***  .*** **.  *:  :   *    **.*. *     :       . **  ..  *

35          YKYKSYKYKRWWGCKKKRRKAAKYWKWSKGKMMYCAYGMKMWRYRMTRGAATCGYGKTCG
47          YKYKSYKYKRAARYGKKRRKGGKYWKWSKKKMATMWTKCTCWRYRCYGRMWWYAYRKCTA
```

```
                **********     *****..******* **       . ****        .* *  .

35              MRTTGTTCACKCRATAAKGRTMKKRMRRWSTGCYATWRGCTRCCGYTCAATACGKYYYYC
47              MRWCKWYYMYKTRMCWWKCGKMKKRCAAWGYRTYTYWRRACRMTAYATGCATGAKCYYYA
                **        * *    *  .****   *.   *: ** . *  .*: ..:: .* ***.

35              KCGYMTTGKARKKTWMWYTKYTMTCYKTATRAWGYARARWGKCYRKYKTTCWKWSTTGTC
47              KYAYMCYAKWGGGYTCACCTCWMWMCKWMYGGTTCRRGGWKKMCGKYKGCTWTAGACAAM
                * .**  .*          .  *   *    .    *. * *   ***   *. .: .:

35              GWGSSRMRRCRMWATGRGGCACCTGKKMKKKARYYWCTRGAKKGCKTTYYYYAGCYKCAG
47              AAACCGMRRTGMWRWKATATTYYGRKTCKKGWGYYWYYRSWGKAAGKWCYCYMRGYKGGK
                . ... ***  **     . :    *. **   ***  *.  *.. .  * *   ** .

35              YWCKCRWGYGARWRRSYRRTYCYMKMRRYYATYAGGACCKKRGRYRRAMRACGAACTAKC
47              YWMKMATKTRWGAGGGCAACCGCCKCGGCCCCYMKRRMYTGRKRYRRGMRRYKRWAGCKG
                ** *              .        *      . *       . * ****.**      . .*

35              GAAACTTCYKKYMYKRCKYYRTKYYRKMYWMGCRMYRYSTTTGKWWWCCGRKWRTWKYYR
47              KTRMMKCYYKKYCTKRYKTCRGTYYRKCCAMRYRMYRYSAWYRGAAAYSSRKAAWAGCYR
                 :    .  ****   ** *   * .****    *  ******:           ..**       **

35              SMCATGKCACTKKYWYKAWWGCCRMGRYKKKKKKRYRKYKSKAYRYGKGYYYWYCYWTKG
47              SMTTGTKACTKKKCTTKRAACTYACKRYKKKKKKGYAGTGSKTYGYKKTCCCTYGYWYKK
                ** :  *.. .**    *         ******** *     **:* * *      * ** *

35              AWMCTRTYRKKMKMAKRATKCWATGTCGTAYSKCCAAGCYTCTGRKAGKKTATKKYWYWW
47              WWAYKRATGTTAGMGKRRYKTWGAAYYKGCCGTATTRAGYYYWKRKGRKKCCCKKYWTWW
                 *   .*:   ..   *.**   * *.:.     . ... : . *     **. ** . **** **

35              STMCWRTAGRKGKRCYRYCRCKWRSTGCGGGYGYMMYGTCGKGSYWAKKYKKYAYMTACC
47              SCCYWRCMARKRGGMYRYMRYKTGGCAATTAYKYMCYACTKGTSYWMKKYTGCWCMYRYY
                *   **  .**     *** * *    . ..  .* ** *.      *** ***.     *

35              YKARYKACTWYYRYRRCKKCKRWRRKGCGGRRKKRCRYWGCYTTGWRGRWKYMYRCGTTK
47              TKRRYKGYCATYGYAGYKKAKRWGAKSMRRAATKRMGCTATCCGATAKRWKCATATKWYK
                 * ***.     * *   **.***  *.      .**     .    .   ***          *

35              GYTTAYYTGKYKWKKGAKYGGRCRYACRGKRRRRRCTTATYAKYGRAYAGKKYKKRSTTT
47              RTKCCYYYRKTKWKKATKCRRRYRCMYRKKGGRRRSKAWYTRKYRRRCWRKKYKKRSCCK
                  . .**   * ****.:*    * *    * *   ***..:     ** *     *******   .

35              GRKAAGYTGGWRYKKCCYMKRKGKCWCCAGMYGRYCCASYMWKMTGKRYYYYAYKTCTCK
47              KGKWWRYCARWATKKYMCCKAGKKYAYAGAMYKGYYSRGCMTKMWATGCTCTRYKAYCYT
                  *    * . *  **     *    *    ...**  *  . . * **  ..        **:    .

35              GGYARGGKKRYYTGTYRKKYRRMKRRTCYGKMKAAAKMTKCTKCARWRGTKRCGGWAGCG
47              TRYRASKKGRYYYKKYGKGTAGMKRRWYCRTMKWMMKCYKACKYRRAGACKGTAAATAYT
                  *   . * ***   .* *     ****     .**    *   *. *   *   . *   .. :.

35              GSGRRYYKCCYRYYYAAGGKRMAATGATTGCKRGRCAACTWTKRYWGYRRTYKRWGGRMA
47              CCRGRYYTMYYRYYYTGTAGGCTRYKRKYCTKRRGTGMYCAAGGTWTYRRGYKATRAACR
                 .  ***.  *****:. .    :     .    **    .     :    * *** **     .
```

```
35    GWYYRRTYKCGGGACKKMATGCMYRYMYMYYYKRKRMKKGTAYTACCKYWYKRMYGTCWK
47    KWYYRRCYKYACRGTKKARCAMMCGYCCMYYYKRKAMKKKKRTYTTAGCAYKAACRAAWK
       ***** ** .  . **   . *  *  ******* *** .   :.    **    :.**

35    TKKKKTYRYTYGRKTTAMCTCCTATTYWYAGYTATMATTWYARWKYRYYTYAGTAAAMTA
47    WTTKKWCAYYYKGKGCTMAAYYAGAATACWATARGCRWGWCGRWKYRYYWYGAARRRMCG
       ..**   * *  *  :*.:  :.::    . :      * .******* *..:   * .

35    GTTTMGTMYKGKCYYYTMKMCGKSRMAMCAYRGCCGGRMMRATGGKTTTTYMYRKWARTS
47    RCAYCAACTTKKMYYYKATAYTTGAMGATCTGSYMKRGCCAWYRTGWCAWCCYRKWRACG
        :  .:  . * ***. .   .. *.  .  .               :   ****   .

35    ATTAKCACTTKMAWYYMKYWAKWWAYAMGYRYKRGTWKGRYGCTRYKKCTAWYRMAACYT
47    RGARKMMYYYKCRWCYMKYAGGTTWCWMACACKRRWATRRCAYWGCTKMGCWYRCRRTYY
        : *     *  * **** .      *.   **   . * .    .*  .***    *

35    CGRYCAKKWWTAWTACYYSRYRYMARRKRRMCKGATTGTTCKRGKGKKTKTTCCSAAARK
47    YRGCMRKKWWCRWWRACYSRYRTAGRRKGACSGKRYYRYKYKRKKRKKYGYGAMSWWRGG
            ****  *  . *****  .***   .       . ** * **  .   *

35    SKTGAGKTAWRAYMYACKKSTYAKKWRATCTGTAWKKRCGWCCWAWYKMRYCKATKMSSA
47    GKYRMAKYRTGTCMYMYKGSKCTTGWRWYTCSCGAGGRYRAMTTWWYKCGCTGTGTCCGW
      .*   .*    : **  * *. :. **    . .   *       ***     : . ..

35    TCGYKRMKCGSKYWGCKCMSAGGSMRKRMWRYYYKYGCCYYMATYTGGCTMKYRGKCCKM
47    GYRYTGCKYRGKYWTSGSASCCASMRGRMWRYCCGCSYYYYMGCCWKRYYCKYRRKGTKM
         *.  *  .*** . . *. .*** *****    .  ***.        *** *  **

35    SYKKCSYYKRSKKRRWKKKKKKKATWRYRYKTAACYRMGRKCRMAMCGSRRYYKKKKKRK
47    SYKKMCTCKRSKKAATKKKKGGKGKWRCGTKYTTMTAMRGTYGAMMMKSGGTYKKKKKRK
      **** .  *****   ****  *..**   * ::   *  .    *  *   ********

35    GYGAYMYAATYWYKKMYRGGKRAKCKCCAAKWKYYYKYGCGCGWRACWMYSSGAGKRRTA
47    RCKMYMYRRACAYKKMYRATKRRKYTYGGRKWKYYCGYKSRYRWRTTTMYSSTTAKRRYG
          ***  :  ******. ** * .  . *****  * .   **:  **** :.*** .

35    KRRKTMACYYMRARRAKKRMARRRKKYKTCAGATTKKRYYRRYAAWTAYRCRKCTKKMGW
47    GGRKCCMYCYMGWGRWKKRMWRRRKKCKCTTAGYKKKRYYRACGCTGRTRMGKYYKKCRT
        **     **   * **** ***** *  :.. .******  ..    *  *  **

35    TTYWYCGYYYTRTRKKWYMYSYMTYSGTACMTGTAGTWMYRKCYACKRWMYKKGCRGGYK
47    CCCTYTACCCWRCATKAYMYSYMKYSKWGMAKRWRKYAMYRKTYTTKRWCYKGKSGAAYK
          * .    *  .* ******.**  .  .      **** *: *** **  . ..**

35    GGRYYRWMGWMKWYYRGYKYRYATTCTAMMKWWGKSRKMATRGTGAYMTMAKKYSGKGYT
47    RRAYYATCKTMKTCCRRTKCGYGYWMYWMCGWTAKSRGCRCARCAGTAGCCKKYSAKRTW
         **     **   *  *  *.     *  * .***       ..    .****.*

35    GKRKRKMYKRTRYKRYMRTYGMYCCYKKCTRKMACTGACSCMTGYRGTTCTCSMTTRYAT
47    RKGGRKCTTGYGTKGCARWYSMYYYYKKTWAKCRAYACMGMMKKCAAYYTATCCAWGCCC
       *  **  .    *   * *.**  ***   *  . .. . *.   .   : . :   .
```

```
35          GTARMRAKKGTCKATGMCYARSGKWKGYGCYAKYGAACSRWAAKYGMCCAYARKKRMWTM
47          ACRRMRRKKAKYKTCAMAYGRSRKWKKYRYYWKYRWMMSRWGWKCAMYYGYWRKKACTAM
            .  *** **.. *: .*.*.** ***  *  * **    ***. * .*  .* ***   :*

35          WMWAAMYKMGTYYAASSCYWAKTTCYTACGRKRRRRKWWCSTKYWCWTYKKAATYMYGKK
47          WMWRRCYKCKYYTGGSSYYWMKKGMYKRYRGKAGAAGWAYCGKYWYWCYKKRRGYCCCKG
            ***   **   * ..** ** *.  *.    *     *  . *** * ***   *   *

35          TACACYAAAAKRWGTKWWTYYKYYYGRRGATTTWYYAYRYRGARTYYACGTYTACYKYYM
47          GRMRAYRTGTGRWRYGTTCCTKTCCKAARGACGWCCRYRYRCMRKCCCGRYYGGMYGTCA
                .* :.: **        *       .:  *   ****  *.  .   * . *

35          KYARYYKAGTARRSYRKTYRMRYRRRKWWTCYASTCKYGTGKWKMYYWWACMCAKAGAAY
47          KYWRYCTCRCRGASYRGCCAAGCRRRKTTKMCWSATKTAGAKWKMYTTTCTASGKTRCCC
            ** ** ..     ***       ****  .   *: * . .*****   .  ..*: ..

35          TTTACYCYMMYYKGGKKYKGYTYRGRKMTYKWMGYGGGRYWKRAGTTYKMKKAAWYATCG
47          WYKRYYYYMMYYTTRKGCKKCWTGSAKMAYKWARYKAAGYWGAWSYYCTCKKGMWCRYAK
              .  * *****.  *  *     . **:***  * .. **   .   . **. *   .

35          WTKRWYYYKTAYKTYYGGTWMARRSMGYRKRTACYRKRRGTKWRKYWWTKKTRRWYWWRK
47          TYGRTYYYKACYGKCTACAAMCGGGARYRKGCCYYGKGGRCKWAKYWWGKGKGGTYWWRK
               * ****:.* .  . : *.  .  ***  . * *    ** **** * .   *****

35          RCACYSYYKKGCRRMMGACCYKKMWGTAGCTCGYYRYWRRAYGYGSAAMKWAMSGCWWKA
47          GATMYGTYKKTTRRCMKRYMYKKMWRYWRYWYAYYRYWAARCRCRSGTAKWMMSCTWWKG
             .: *. ***  ** *    *****       .*****       *.: ** **  ***.

35          YRAKGTACCCWYRAACSGRCKWYTRKCYCMGTTATGGCGARYGKWRKRSRMMYKCTATTS
47          CGWKRKRYSMACGCTYCRAYKTYCRGACMARYWWKTATKRACKKARKGCRMMYKYYGWYC
               * .  .    .: .   * * * .       . .      * ** .*****  .  .

35          KKKKKYYKRGAAMWYKTYKRMMRKMKGACAYYMCAGYGKGKAKCTKWCRTGKYTYYYYAA
47          KKKKKYYKRTGTMWYKYTKRMMAGCKRWMGYYMYRRYCKKKGKYKTAYGCAKYWYYYTCR
            ********* .:****  ****   *   .***   * * *.* ..    .** *** .

35          GTGTTCYRTCYKAYKCYTRRRCTCRCSAACAGYYTKYWYCCMYKTTRRSRYKYKACGAAK
47          SYRWYATACYCGMCGMCKRRRGYGRASTGGRRTCGGCWYTTCCKYWRACGYKYKMYKRRK
            .    .           .***   *.*:.        **    *  * . ****     *

35          MCMTTGRAGWYMMCYGACTCCCMRCYTSMYGGRMKCYMMCTGCMTGMKGGATCCATCKTY
47          CACWWKRMSWYMAYYKRYYYMAMRYYYCCCRRGMKTYMMTCASMKAAKRRCCTTGKAKCT
             .    * .***  *      .** * .     ** ***  ..*.. *  .   ...*

35          WTYKGATGKYTTTRTRRYRMWTAAATYCRCTTGMYCGAAAATRGSKYRKWRATTKRGTKY
47          WWYGSGCKTTCCYRWGATRMWCMWRYCARYYYKAYYRWRRWKRSSKYRKTAGCYTGRYKY
            * * ..  .    *    ***      .*     *      .*.*****  .  .   **

35          MKRYRRYRKGGTRMYTGACATAAKMRKCKTACKRRAGAATGYYATKYYKAGAGCKRRMRK
47          MTRYRRYRTKAAAMYKSGMTGTWGMRKAKYRTKAGGRMCCACTRYKYYKCRCTTGARMAK
            *.******. .: **... : :  ***.*   *  .  . .    ****. .    ** *

35          TWAYTAMCCAGAATKCCYKKCCTRGMACGYCMRRCRMMKRKMWRKKCAKMWARMRMYRCY
```

```
47    WARYYRMMMWSWWWGTTYGKGYYAKMWMKYYAGGMRCCKGKMTGTKGTKCAGRAACYAYT
         *  *   .      * *     *   *     *  * **  .* :*  .*   *

35    WTCCCGKTCRARATTRCAKYCRYRRTYGGCRWTWRRWKYMTSTYACKTGTGMYSGGGGGA
47    WCTATRKCAGRRMYYRYRKYTGYGRYCRRARWAARGTKCCCGACWMGKTCTMCSKRASRC
      *  .  * .  *   *  **  * *    .**: *  *   .:    .   * *  .. .

35    YWYCKCRYRTCMTAGGYAAAKYRGTKGCATCGGATMKGTRYGRTTTGWRATRCYKYRMWY
47    CWCMGYRYRYYMYCAATGGCKCGRWTRAGGMRCTCCKSWRTKAYKYRWRWCGMYTYRMTY
       *    ***  * ... ...*    . ..    :  *. *    .  **    *.*** *

35    RRWKYAGRTCRWYYYKRGYAYYWYYAMCCCGYRKAKRGKRTGATKWYAKTMYMRRYMSYS
47    RRWKYRRRYYGWYYCKRSYRYYWCYCCATTKYRKRKASKRARGCGTTWKKMYMRRYCSYS
      *****  *   *** **.* *** *. .   *** * .**: .     *.****** ***

35    AGCTRKKRTRAYRRAYAARKCTGYRYKKKTRWYRMWWYMSKYRMTCKWGCTTGYAATTMY
47    WSYWRKTGCGWCGRGYRRRKMYSCRYKKKYGWYRCATCAGKCAMYSKWCYGAACCMKWMY
       .  **.      *.*  **  . *****  ***     .*  * .**   :. . . **

35    KRMGKYACYKWWCKYYCCGGCKKMRYMRCMMTWKTTKWTRAKRKRTTRKRKSWYATACAG
47    KRMKKYRAYKWWMKTYMARRYKKMRTARMMCYWKCCGTYAMKGTRWWGKRKCAYCGWYRR
      *** ** .**** * * .   ****  * *  **       * .*   ***. *.

35    ATCCCGGKYTAYRCKKGYYYKGCWYYCTCTGYYYKAGKATTTRACMCGGTWRRYKTTAMY
47    WKMYYRRKYWRCRTKKTYCCKRYTCTMKYCRYYYKRRKWYCARGYMARRYWRRYKCCTMY
       .     **   * ** *  *      .   ****  *   :*. *.   *****  :**

35    AKAGYWYTCRCYTGRKTCMKGTAGGSTGATRTMKCYCKKRKAATCKYCRMYYWRSCKCYW
47    TKMSYACYYRYYWKRKYMCKKYMRKSAARCRYMTYCMGGRKMTCAKTTAMCTTGGAGMYW
      :* .*    * *  **   *     *:.  * *.     ** : .*   *    ..  **

35    RRWRYRYKYWYYMMMMCTGAKWMWKYRWWRRYGRRKTKGCTKWMRYYTCKCWRRKGMRYK
47    RRWRYRYTYWYCCMAMYCACKWCWKYGWWARCRRRKYKRYKTAMRYYYYKYTARKRMRYK
      *******.***  * *  ..** *** ** *  *** *  .. ****  *   ** ****

35    MYYSRYRRCCSYGTTKCKTSRKACAKKRMWMMRYCWTYKATTYCKRWCMMYKMKGTYKKR
47    MYYSRCARMTSYRKKKTKKGRKRYRKGGCTMMRYMWKYKGKYCYKRAYCATGMKRYCKKR
      *****  *  ** ..* *..**   *    **** *.**..   **      **   ***

35    KMRMRRATGKSTATTRCRYKTRYTGCCAGRYMKMSGCAACYKGTRKKYTTMGTGRRKYRC
47    GMRMRAMYKGSCMYWRTRCTCGYYRTATARTCKMSKMCGTTKKGGKKTYWMACRRRKCAY
       ****     *    * * .  *   .:.*  ***  ..  *   **   *.  ***

35    MKRCMYTCKMACGGAGMWRYGARAMGTTMCWWGCAYRMWWRKRYYMMYWYSCYYGTTKCG
47    ATRYMYGTKMMMKKMKCTGYKMGMMSYYATTTAAGYRMWWRKRYYCAYTCCYCYKWKTTA
       .* **  **         *    *.      ...**********  *  .  *  .. .

35    GAMYAKRCAATKSWAYTAYRKRAMAATGCRATACSGKWYRACCYSYWRYAGGTGTKYRYR
47    CCMYGKRMGGKTSWMCKTCATGRMRRWRYRWWTGSRTTYRCSMCSYWRYRRRAACKYRYR
       .**.** ....**  .:  .  *     *  : * . **..  *****   :. *****

35    RRRWTKYRCTKRWWRRGGGWGKKRKKTGAMKKMRMTTAGAYMYYRRRRGKAGMTKTAKRW
47    RRRACKYRYKKRWWRRTTTWRGGAKTYRGAKGCACYWWKGCCCYRRGRKKMKCAKWMKRW
```

```
            ***  *** .******   *    *.  . *        .   *** * *   :*  ***

35          RGCRYTTTCTTTRWCKKGRTRTAKGGGCYRTCRACTKWKMYYRTGMYRAATTCRCGRWSA
47          RKYRYYKYYKACGWYKKTGGRYRTAKRYYRWYRRYYKWKMYYRCTCTAGMGAAGYRRWSG
            *  ** .  .:  * **   *  ..   **  *   *******     .  :.   ***.

35          KARWKTAGAAKRGYTAWTKRYKGTYGGTAKYATKWYRYMYGMGCMMAGWGCTTCYSCCTT
47          KMRWKCCRCGKRSTWRWGKRTTRYYRKYRKTGCKWYRYMYAMATCAMKARYWCTTGTTCC
            * *** . ..**.   * ** .  *    * . *******.*.            .

35          YMCRYRSGAGYTGCTKTCGTATTKWKAYYSMRRTYGTMYTTCMWWMRYCTAMKCARRYRK
47          TAMAYACACAYYKSYKKTKCRYYGTKMYYGAGGYYCACYWWMATAMRYMYRMKTGAACGT
                * ....*  . *.        * **.    * : *      ***  **  .    .

35          MRASRKYRSSKAASKWKYKCCKTACKKYRRYKKAYGAYYYTRCKARYYRTAATKYRTWAC
47          MRRGGKTAGSKTTGGTKYKGAKYGTKKYAATKKMCKRYYCKGTKGATTACGTCKYRGTTA
            ** . *  .**::.  *** .* . ***   **    ** .  *.      .: ***  :.

35          MRKKYCAAARAACTKGGCYCYMGCMKYYSYAKWKKCYGAKYKKWTACAKKMSGCTYGGYC
47          MRKKCTGRRRRRYAKKKMYMYCRYCTCYSYGKWTKMYRWKCKKTAGMWKKMGRAYCSSCY
            ****  .  *   :*   * *    . ***.**.* *  * ** :.  ***. .  ..

35          ATCARTTYKYRRMRTCMGGGCTGARAAKWWYYCGGCRRGGGGCCGAGGCRKKWRYKYKTK
47          MYYRGKYCGCGGCAWYMTKRMYKRRTWKWTYYTRRTAGRRKRYYRTTATRGKARYGYKCG
                 .          *       *: ** **             : . * * ** **

35          GAYRRCYKGAYTGGSMKGMYYCYKTKKKKKKCTKTCYTGRRTRKTTRTCKWTCTSMKMGW
47          KWCRRYTTATYACKCCKRAYYMCKCKKKTKTMCKCTCAARGARKKYRWTTTCYWGATARA
               **  ..:*:  . *  **  * ***.*.  *   :.* :**. *  .    . .

35          SWKTCYWSRRYYCMYCAKMAYKKRRKCAKMSAKGTAAKYKSYWMRRWGTTAKWYYACAGR
47          GTKWYTTCAATYTCTACKMRCKKRRGYWKMGRKKWMRGCKGYTARRWACYWKTCCRTTKR
            . *    .   *   ..**  ****   **. *      *.*  ***.   *     : *

35          AKARRSKGWSKGKKMYRMWMRWKRSMGTGCCMWMYGYCRMRRYWYRMYYRRYYTRYARYW
47          WKRGGCKATSKAKTCCGCWARWKRSMRWRYSMWMCRYMRMRRYWYAMYYRGTTWRYCGCA
             *   .*. **.*.    * ******    .***  * ******* ****    **.

35          RRTYKYRMRKRCARARKYRTGGKSYATMRYRTTTYKTGATCATARACTWGKTWRSYWYWA
47          RRYYKYAARKAATRCGKCRYRTKSYWYAGYRCKGCGAKRKMMYRRGTWAKGYWRSYWYWW
            ** ***  ** .:*. * *   ***    ** .   :  .    *.      *******

35          YRKGGTYGCYYWYKYKTRRTKKGSTYCTYKWKKGCTTTAAYKMWATGRKYWMKKKSWYWK
47          CGKRRAYRYYCAYKCKKRAGKKRGYYYWYKWKKRYYKKMMYKCTRKKRKYWAKKKSWTWG
              *  :*  *  ** *.*  ** . *  *****   ..  **   . **** ***** *

35          KGRGRWAKYGAWWSMYMTRRATRKGSRMTATMRCCRMATGKKYKCRAGGTCRGKKYKCGR
47          KARRRWGGCRRAAGACCCRRWKRGKSRMATCMRMYRMRYRKKCGYRCTAAYGRKGCKARG
            *.* **.      .    ** .*  ***:: **  **   **   *. .:   *  *.
```

The pairwise alignments of two accessions 35 and 48

```
35              AYYYRCYYRMYSYRYCCKACARYGWYARMKRYYAYWRKGKYMAAATMGARKCCCYYYYKY
48              TCYYRMTYGATSYRCTTTWTRRTKWYMRMKRTCRCARTAGYCRRMKAAGRKATTYYYYKY
                : ***  *   ***   .   *  ** ****     *.. *    . ..**.  ******

35              YRGMCRMGKGARYKRCKWWWKRWYCCCAAYSACYWYYYCCKMRRKKYGCTMWRKRATWRS
48              CGRCGRARKARGYKRYGTWWGRWYYYYGTYSWYCTCYYTTGMRGGKYRYWMWRKAGAWRG
                     *  *.  ***   ** ***   .:**     **   **  **   **** .:**.

35              WRMWYMMRMRKTRGYCKWWWGRYKYYTYMRATKTAYAYYYYRRYGWKKTGSRRKWMKYMW
48              WRMAYMMAMRGYRKYMGWWAKRYKCYYCCGWWKATTRTYYYRRYKWKKWRSRRTWMKYMW
                *** *** **  * *  **  *** *      *::   ****** ***  ***.******

35              ARYRYMYYYRRGRGGAGWTMWYGKTMYAKTTKSRKSYCRTRYRYRGYRRYYRRYTKMRWR
48              TRCGTACCTARKGRTGTTGMWYRKKMTWGCGKSRKSYYRKGYAYAKYRRTTRRYYKAAWR
                :*        *    .   *** *.*     ****** *. * *  ***  *** *  **

35              YYKRTYGYRTATMGCYSCGWMATYYKRMCKRYYGGTRWYAWMRYKRRWYYRWRRCMRKKS
48              YYGGATATAWRKAAAYSTCWCTKYTKRMYKRYYKRWRTYGAAGTKRRTCCRWRRYCGKKS
                **  : .    . ..**  * :.* *** ****   * *.    ***   ****   ***

35              CGCGARWYRMYRYYYRYCCRSMRWMYMYWMTCYCYMKWKMGKKTKWRGAATRGTARACKG
48              MRYKWATTGCCGTTCRTMYRSMRWCYMYTCCYTYYMKTTCAKKYTWRRGGGAAYCRRYGK
                               *   ***** ***      *** . .** .** ..  . .*

35              TCMRMRYRMTTTRMRKKTMCKYTATYRYKYAYYMMCKRRYYYRTMMCRMRYRRGYTCKWW
48              YMMAARYRMYWKRMRKKCAMKYWRYYRCTYMCYMMYKRRTYYRACMMACGTGARYWMKTW
                  *  ****  .*****   **   ** .*  *** *** ***: *        *  * *

35              RRSRYKACWWCGCRRMKKGWKCRGKCCYMRYWYTRTRYRYRRRKWKRWRKMYKGTTGYYY
48              RRSRCKWYWTYRMARMKKRWGYAKKYYYMRYACYGYACACRRGKWKRWAGCYKCCAATYY
                **** *  *     **** *    *  ****         ** *****   **  :. **

35              YYWARYRYRCYKWKSRTCTCYRWSCKWYRCGMRWYYMYKGGATTCYTGCCCGAGCGCRYS
48              YYWMRTRTAYCGAKSRATCTYRTSGGTTRAKCAWYYCCKTKTGATYAAGTYKRTAATGCS
                *** * *      ***:   ** *    *.   ***  *  : : *:.      ..   *

35              AKGYTGACTMTCSMYGMMGCTGRRCTCYMGGTCCATGCYYWGTTGGMYKGYGCATKKRAR
48              TKTYYKRYYCWYCCCRMMATCARRGCYTARRCTTGKTGYTWRYWRKCYGSYRYGCTTGWA
                :* *        .   **.  .**          ..  * *      * .*  . ..

35              WCTAGCYRAATMGRRCKKACACTRAGKSKYCTRGWRTYKRCYATCTKRCGMAWYRKMWYA
48              TYGGCMYGWRYMRGRTKKTTMSWRWSKSKYAGRATAYTTGYYTCYGTRTRCGAYRKMWYR
                   .  *    *  * **:  . * .****. *.    .  *:   .*   . ******

35              RYATAGCKMRCARKKTAACACCRAGGCRRWYRCGKWRKKGAWYTKCYCTKKTWGRMWMYY
48              GYTGTRSGMRYRRKKYRGTTATATTATRRWYRYKKARKKTTWYYKYTTATGATRACTAYY
                 *: : . ** ***   . :.  : . *****  * *** :** *   :. :      **

35              CKYKTAYAKKKKCKRKAATYYTACRTCRKCTTRTYCKRCTSMKTTTGGGGSWKKWSAWWY
48              YGCKCCCCGKKTMKATTTCYTACTRAARKTGCGATTTAYWGAKYCCRRRKSTKKTCWAAT
```

```
       * . . **. * .:: * :. *:.**    :  .   . *       * ** .

35    CMAYKYMRRCCAKCMSKAGKTAAYSWYWRSGRRWTYRAWYYAYRAKMKSGGCTCARRRRS
48    YMRCKYMRRYYWKTAGKRKKWMRYSWYWRGRRRWYCAWTCCRCRTGCTGRKMGACGRGRS
       *  *****   *  .*  *   ******. ***         *:  ..    .. * **

35    YARRKACTKTTSKGWKGTYRRRRAYMKKGGMRWKRSMRSRRCAKKRRMKMTKRMRRGYCK
48    YRGGKTTGGWACKATKAKTGRRRRCCKKRRARWKRSMRSRRMGKKRRMKAYKRMAGKTMK
      *   *:    :.*. *..  ***   **   ********** .******  ***     *

35    TYMWAWWKRWMWKRTYYKYYYTCWKCAYGAGGKRYRATGKWRYGRGGGTCCRGGACKAAA
48    YTCATWWKGTMWTGKCTKCTTAYWKYRYSRRKKRYRCYKTWGYRGAAACYYGAARYKWMM
          :***  **. .  *   : **  *.   ****.  .* *  ...    ..  *

35    KTATSRKKCTCACTTRWRRCGGWCGKTKCKKKGYRRKYRYAAGGMAGCYYYRCATYWYYY
48    KYTCSRKTACYRMCCRARGTAAAAATYKYGKKTCRGKYRYTGTACWCTYYCGGGATWCYY
      * : ***..      * *  .. ... *  **  * ****:. .    **  .:  * **

35    TARYKRWGGRMAGMKGGCYYRTGMATRYGWYWYGGCKMKYRYYTYGCTAASATGTCMRCM
48    AGRYKATTAACRKCKAATYYRWKCRGACSWYWYACTTAKCRTCCYTYGWRSGAACTMRMA
      :.***   .     *.. ***       .****.  . * *   *     *.:.  **

35    RMTGGGGCMWCCMYSGCMYYYWWKAGMKRTTCKYYGYATCKATTAACCYRMYGAGTKMYT
48    GACACAKMMWYYMYCCTMYYYWWKRRMKRYYYKCTACMWYGGCKRTTAYAACRRRKKMYY
         . .  **  **.  *******  ***   *  .     . . : .*      .***

35    TYKKKRATCACYYWWYATTYYWCKATYCCYGGCSKRMGTRRKTATAYYGRCCCKKCMGAY
48    KTTKKGGKTGGTCWWTRYKCCTYKGCYMTYRRMGGAARYGAKYWYRTCARATTGKGMAGT
      . .** .. .   **   .    *. *  *   .       *      .*.   * *..

35    CCCTGCTCTYCACYCWYKRMWTKTCGRRTATMKGCTTMWRGGTRGYAACGTAMTGAMRRM
48    TATARTCTCTAWMYYWYKRMWATWYRRRYRWMKRAKYMTGAAYRKTRTTRYGCAACARAC
       . :      .  * ******:.   **   ** .. *  .. *   :   . :.. *

35    RYYGATRKTRRRYKYTAAATRTKCTGMCATCYCTGKATACGGKYYYAWCYWGGGWMGGGK
48    AYYKRYRKYRRRYKCCGTWWGWKAGACTTGAYYKKKTGWTTAKYYCGWYCWKRRACATRG
       **   ** *****  .:    *. .  : .* . *:    .*** .*  *     .

35    GCATRYAATWWRYGTAGGTKRRMYTKKKKMRACYYCCTTCGTTMKGARRMCAGCRSKAYC
48    ATCCRCCGCATRYKKGAKGTRRMYYKKKKCRRYYCTYYKMKCCAKKRAACYWRYRCKGTA
      . . * ..    ** ...  .**** **** *  *    .     *         *.*. .

35    AGCYWKYKYACGRGGCMGRGAKYKAGGGARKRAKKRTMYRAMKKMWRYRMKGAATCKKKY
48    GTTTAKYKYGMRRRRYARARGTYKTAAAWGKRWTKGYATRWCTGCWRYRCKRMWWYKKGC
      .    ****.  *       ..**:...  ** .*    *  .  **** *     **

35    RWWKKTCTMCACKTAYWRGMTWYCAGTRGAAATMTATGTTTRYARRTGGSWRYWAGKRRR
48    AAWKKGYCMMGYTAGCWAACAACSMRYRKRRRCMCGYCCGGRYWGRYRASWGYAGAKRGA
        ***   * . .:. * . :  .   *     * .     ** *  .** * ..**

35    YYAGACTTKACKATCGGKMKMCMAYCRYAASGGKRAKGGRMRCGRWTWARTTGGKWRTYM
48    CYGKRYYKKTYGWWMRKKATAYCWTTGTGGGRSKGMKKRGCRYRATYWGGCYRTGARWCC
       *.    .*:       * .        ... .*  *    *     *.       *
```

```
35          STAACACTTGMCCRKYACRAAYMAATWAWWYTCCATGYGTKTCRKYAGCKWWWTTYWCGY
48          GGCGMMYYYACATRKYRYGWWYMGTGAGTTYYYGTGAYRYKGAGTCRRYKTWWYYCTTAC
            . ..     . . ***     **.:  .  *   : .*  * . .    * **     .

35          YYRKTGYMYSYSGTMAGTWAGGRCYKRTAYTKATCRGKCTCKYGRYKGKRRWYMAKMWYY
48          CCGKYRTMYSYSKWMGTYAWKKRTYKRCTYWKCWYGATYYMKTTGYKRGRATCCTGCTCC
               *   ***** *.      * *** :* *.   ..   *   **  *    :

35          RAAYYRKRYCGRMYTGKMMAAWSRKTGYYYRMGRGYMRCWGKKAAYSTGTGSKWYYGTRT
48          RRWTCGKAYYKRMYCRKMCWRTSRGAKYYYAMRARTAAMWKKKRTTGYRWRSKWYYRYGG
            *     * *  ***  **    ** : *** *       * ** : .    *****

35          RMGTTCTKYKRTYYGCMYWWRKKKMYCYYKGTTYRTCRCCYYYMKYRGACTCGATCTCTK
48          RCACYMYKTKGYTCTMACWWRKKKMYMCYKRYKTAATAAAYTCMKCAAWYWTARCTATAK
            * .    * *        ********  **  .  :  ..*  **  .    .   : :*

35          ASCKGAATRAARTCGKMRACGGTCATGGYRKMYWWTTMWMRGTGKRCSYKKAWACGCRRR
48          GCYKKGGCGTRRATAKCGRTAAKYRYKKYRKMYWWGKATCGACCKGMGTKKMWGYTTRRG
            .. * ..  : *: .*    ...     ******* .    .  *  . ** *.   **

35          YRYKKMAMGKCKCYKKTWMWKYGRKYRGYGAYGYMAKWAMCGKKRWKACGTACCCWGKKG
48          CGYKKMWMKGYGMYKKGAMWKYARKYRAYTRCRCMRKWMMYSKGAWKGYRKRYSMARKKK
              **** *     ***  ****.****.*     * ** * .*  **.  .  .   **

35          TWRTCKWYRTAACTAAKGGCYYTCTWYYRYGATGGKWRMSGRMMYKCCCTATYYKRTSTY
48          YWAKYGTYRYMMAKGWKTATTCKSKTCTACRRWTKKAGMCRGMMTTYYMYGWCTKGYCCC
             * .   **   ... * .   ...          *  *.  ** .    .   *  .

35          ATRCGAYMMGYKARGRMWYKTKTYTRMMRKGACGGARWYCACTGGYKKGGTKTYMRYYST
48          TGGYKRTCMKYKRGRRMWYKKKYTWRMMAGRWTTAGRAYYRYGKRYKKAKKKWCMRCYCC
            :       * **   *****.*   ***      ..* *      ***. .*  ** *.

35          TTCAKCRKRYTTAYTKGCKYKKGTRTCGCTCRKGCATAAACGKKWCWCGGARGMYYTMAM
48          ACTCKTGKRYCYWTYKATKCGGRCRYMKAWYGKKYGCCCTGAKKWAAYRRMAKMYYYMMA
            :  .*  ***     *. *     *   .   *  . ..: .***.       *** *

35          TTGKARKTKRTWMWTTTARTKYRRRATGWATAKTKWSTTGWCRWRTAGRYTYRRMCYTRT
48          CYAKWGGAGRYTCAWGWMGGKCRGGWKATRKWKYTAGACAAMAAGYRRATCCAGMYYCAK
              .*   : *          * *   ..  . * . .: .              * *  .

35          RGKYKKTGWGCTAAARRTATRYYMRATTTTTKTTAKGCYYYKKTAYYWRCYTKGRWKKGT
48          RAKTGKKSWAGCGTTRRYRKRYCCRGACYYWKCCMKRGYTYKKATYYWRMYYKKATKTTG
            *.*  *..*.  .::**  .**  *.:    *   *  * ***::**** * *   *.

35          GTRGYGAGWCKRYCACKGCKMCGTGGGCRRMRGGAKTGYTRYRKMRACTAKCGRKCKKCK
48          TCGKTATAAMKRYYRYKRYKCTAYKARTRRMRKRRKCKYARYRKMRWAGCKGKGGYKKYK
                 .:.  ***   *  *  .  .  ****   *  *:****** . .*     ** *

35          YRTKYYKMYWTAAGCRMYRYTTTGGKKKWWWCCGRWRKWKYYGCWWWCTGKCGCACKMYW
48          TRYTYYKCCAKMMRYRMYRYAWYTRKKKAAATGSRAAKAGCYATTWWYKKKYAACTKMCT
             * .***   .    *****:    ***     .*  *   *.  ** . * ... **
```

```
35      YWWGCYAAGYRMGKRKYKYTKRGYRKYSKAYMMRGYYYWRKKGWKGGTRYKYWKKMKAAA
48      TAACTYCGAYACKKRKYKYKKRRYAGTSKTYMCGTYTYWRKKKAKAACRTKYWTTAGRRG
             *...*   ******.** *   **:**   * *****  *.. * ***..    .

35      MATKKGGGTCGAASCYMGTCYCCGGRKARYKKYWYWWSWRGGGGGYTGCRTAAGKGKRCY
48      MRYTGRAAYYKRWGAYCAGYYGAAKRKGGCKKYWTWWSWRKRRRKCCRYRYWRAKAGGMY
        *  .  ..     ..* .  * .. **.  **** *****         *   .*.   *

35      YSYYCCKKAWRSTGCGKGGYGRYMMYGTCWYRTCGKMTGRAGYKAGYRACTKKTMKYGRY
48      YSYYMYKKGTGGCAATKTAYKRYMCYRYYAYRCYRKCWRRGRCKCRYRCGYKKYAGYRGY
        ****  **.  . .. * .* *** *    **   *   *.  *. **.  **   *  *

35      GGYKYTGWAACCTTARSRYKKTYRMRKKYASKYRAKKMYWWYYMCCAGTKYKYRTMAKGG
48      KRTGYYRWGGYYWCRGSAYKGCCAAGKKCWSGTRGKKMTTTYYASMRRYTCGTACARKCA
            *  *..      * **      **  *  *.***   ** .    .       * .

35      CMMYRRAKGKWKYKKYYRRKMGTYKRRRGYCKRGTTGCCGKKWKRATWMGKRKGKKYATK
48      AMMYRRMTRKWGCKKYTGAKMRAYGRRRRCMGASYYKMAKKKWGARYACRKGKKKKCWKG
        .***** . **  ***   ** :* ***     .    . ***       * * **  .

35      WYYWSKKTAGYTKKKMRASKYRKYCRKAATRGSTMCRCWATRYAYRYTKATYTGWYRCTG
48      WCYWSKKACAYYKKKMACGKYRKYTGGGTCRKSCCYGYAMYRCTYRCYTCAYYRWYRTCA
        * *****:..* **** ..*****   .: * *        * :**  ..:*  ***  .

35      TATARRYMGTACACRGWKKYTTMKRRKYMKYRCKWAKRMRCARMWRYACMTAGTATCWKR
48      AGKWRRTCKKCAGTAAWKKYYWMKRRKCATCGYGTMKRMRYRRCTGYWTMWWSYTCTTKR
        :.. **   ....  .****  *****  .      ****  *   *  *  . :   **

35      YRMAWKWRMTTMYGYTTKYGYYYKRYAARRKSWAGYKAGTAYYYRKRMGCRCSGKWYTGT
48      YRMRAKWRMAAAYKYWKTCRCTTKRYGGRRTSWRRCKRRWGCYYATGCRYRGSRTTYCAC
        ***  ****:: * * ..     ***..**.**   *   . ** .    * * . * .

35      RATCAWAYYWRATGRYYTSSTTTCAAKYTKYRMTGYYCRCTGGGAWKKMRKKWTGARKMR
48      RCWSGAGCYWRRYARYCWSSYKYTGRKYKKTAMCTYYMRYKTTTCWGGAAKKWYRGRGCA
        *. .. . ***  .**  ** .  . **.*  *  ** * .   .*    ***  .*

35      WCGARTGRGTACTKKTTTMWGTKMTKKGCAKRWTWTTTCTCTTMGWCAWMYTGTAAKCTG
48      TYKWGYRRRYGMAKKCACCTRYKCAKKRYMGAAKWYKYYKYACCRWYRACYGKYRGTMYK
               *  . :** :     * :**      .* .  . :   *    *    ..

35      TGAYCYRTCGKGAYACTCKARKMYYRYMWYRGSCWMRWKRCWKRTRYKMCKKRWKKSMGC
48      YRGYYYRWYATKRYGTCAGCAKMYYRYCATARSMWMRWKRYWKAGGTTMYKKRWKKSMAT
          .* **  ..  *.  . . ******     * ****** **    .* ********.

35      MKYAKCYYYSYMCRYRSGTGCTTAKTGGCAACCRYYATRWWKMRYWKTACRCTRMRKMCA
48      CKYWKTTYTGTAMAYACAYKSYWMTKRATGCTTATTRYGATKMGCAKAGYRYYAARKMAT
         ** *  * .    * ..  .   .. . ..          **   *:. *    ***.:

35      RRACACKRATTAGMRYYRGATKTGATARWKTWRSYWYMKTGTWMRAGGKTCAAGTWGYWC
48      GGCGCYKRMYKWTAGCYGAGCGAKMYRRWGYWRSYWYMKYACACGTRKKYYMWRCWACAY
          . . **  .     * ..  :    **  ******** .    :  *      *.

35      YKYKAATTRCMCMSGSCRTTKWKTRKKYKAGGGKGGMAGMRKYYKGRGYKWRAKYGWWST
```

```
48                  YKCGMRKKRMCYASKGYRWWKWKGATKYKRTRAKRKMWKMRKTCKAARYGTRGGCRAAGC
                    **    ..*    * . *  ***  .***   .*  *  ***  *.  *  *.     .

35                  GCARRMATCGKGMRCRKTYRRMRATGGTKARRTTYCRCRKGGTKYRGKCGRMYYCGGGAK
48                  KYMRRCWKAAGKCRYGKCCRRMRRYRRYKRRGCCCYRGAKTAAKCGRKARGMCTYTTACK
                       **  ...   *  *  ****     * *     *  * .:*   *.  *     ..*

35                  AGAWCMTTRWRYYYTTAMGRAAKWYCRKYTGRAACCTMRYGGKACGGSTGTCMYKYKRKA
48                  RRGWYAWKRTRYYYCCTMRRTCKACYRKYWKRRRYMYCRYKRKGTAKSARCYMYTCGAKM
                      .*   .* ****  :* *:.*   ***  *      **  *. . *:   **.   *

35                  KYCRMYYWRSCKYWRYYMMCTGACYACWMRWWRRWCCSYRWGGCRRYRKTKKWMRYYWRR
48                  KTTAMCTWRSSGYWRYCCAYCACMYGYWCGWWARWMAGTGTARTARYRKYTKAMRYYTAR
                    *   *  ***. ****     .. *. *  ** ** ..   .   **** .* ****  *

35                  KGYMMKTKWYYGYTYYSKKTCACCGSKCMGTTCKKKAACAKRMWCKYATKGTTKRTTMYK
48                  KRCAAGCKWYYRYGYYSKKYTTTYRGKTCRKKTKKGRRYGKGCTMKYGKKAAWGGCCATG
                    *      **** * *****  :   .*   .. **    .*    **..*.:

35                  GYWKKMMRMRKMTCGGGTKKKAYWYSKTYYKGTYKTKCMMWWWWMRYCTWKWCGMGKRRK
48                  KCWGKAMRMRKAWMRRAGKTKMTAYGKYTYKCAYKAKMAATTAAARYAYWKATACSKARK
                      * * *****     . *.*   *.*  ** :**:*        **. **  . .* **

35                  RYRKAMSYYTCRCGSWMRRYKKAASAWKKKYRRYYKRTKYGACYYGYKCYYARYYRAATK
48                  ACGKCCGYYATRYAGWMRRTKKTTGRTKKKYAATYGRAKCKRSYYKCKMCYRATTAGTCK
                       *. .**: * ..**** **::.  ****   * *:*   .**  *  *     .: *

35                  YRRTCMRKKCMYWCASYRTAACRAATGYCCRKGMYYMGCMGKRYSYKWKYGRKYKCTCMY
48                  YRGCAMRKKMACWTGSCGCCGTRRRAATTTAKRMTCCRYCRKGTGCKWKYRRKCKYWMMY
                    **  .****   * .*   .. *  :.    * *       *  . **** ** *   **

35                  WCAKMYYSYCRCGGYTAYKYMMACCACATTACKGTCRYKGWWYYAKCGCGWYAKYYRWYK
48                  WMWGMYYGCTRYKSYYRCGCMCCYMWYTYWGTKRKYRCTRWTYYCKTRTSWTWKTYRWYK
                    *   ***.  *  .*     * .    :  . * . * . * **.*   .*  * *****

35                  GTYMYRRRWRRGTKYMARYWMRRGKRKRMCGCYTTRWKYMYRACCKRRYYYTGCYWKAGS
48                  ACYMYAGRWGAACGYMRRYWMRRKTRKRMTATCCGRWKCATARYYKRRYYYYRTTWKTAC
                    . ***  **  .  ** ****** .**** .    ***       ******    **:..

35                  KGRCRYACGKRARCMCRGRRRRKCKSAGWRGRSKCGWRSYGCTRKATCCTGKKRKRYMKK
48                  TAATGCMYKKGMGACTGRRRRRKYKSCKWRKRSKMRWRSYRMCGKTCTTGCKKATACCKG
                    ..       *   .    ***** **. ** ***  ****    *:     ** .   *

35                  KKCWCCTCAGMGRYYKSYCTKYYYCTYCGTYRRYRTCYKTAYCRKYGGCCTTGATMGTGC
48                  KKYAYTGAGAMAGYYKGCMWGCCCACYMRYYRRYRYYTKKMCTRTCARGTCWCTCCSWRT
                    **     ...*. ***.       . *   *****   *.   *. .      :  .

35                  TACYCCAWKYTRYCKKGYRMAACCYRRWKGARKAARTTCKWYRMMYGKMYWRRMGCAKYY
48                  CCTTYYMAKYYRYTKTKYAAWGMYYRRWKCRRGRWGYYYKWYRMMCKGCYAGACRYRKYY
                     .      ** ** *. *   .  *****  *       ******    *       ***

35                  WYYAKKRGCRYKWGAGTKARRRMMMGKMYRGTRMSYSAGCTRKTKRYRACAYKWWRYYKR
48                  WCYRKKASYRYKWAGACKWRRGMCCRGMYGRYACSYSWSYWRKKTGYGWYRCTAARCYKG
```

```
        * * ** . ****... * ** *    **     *** .  **.. *     .  * **

35      MWWYMSWGTATTKRMYAGYKCYKYKCYKCYCGCKKMRRAYRGYYYRYCYAMRCMYMTWTT
48      CATCAGWCGMAGTRMYRKTKMTKCKMYKMCYRYKKMRRGTATCCTATTYWMGMACMYWCC
             .*   : .***   *  * * **     *****.         * *    * *

35      KTAWKRGRTTRACTGKRKATKKYGKKCGKTAGTRKMRATGAKMRSRGKKYYAACYRMWKA
48      KCGTKARRWWGTTATKRKCGKKYRKKYRKWRKGRKMRTCARKMRSAAKKYYWMMYAAWKG
        * . *  *   : : ***. *** **  *    ****: . **** .****   *  **.

35      RGAGRYKKTRMWRWTAGARAATAMYKKTCMYASSCAACGGKYWRRTWTKAMKKRRGATRR
48      RKWRAYKKCACTAWATTCGGGCGCYKKWYCYRSSYMRYRRKCTRRAWYKGMKKAGRWKAA
        *    ***     *:: . .. . ***   * **      *  **:* *.***    .

35      CTKKWCYWYTTTWMTAAKKTYYYMYAGTGAGCRTKKAAKWYGTYYATTARTGAAWAYRKC
48      YKGKTYYWYCCAWMWRRKKGYCYMYWRWAGTTAWKGRCGWYRYYYRYWGGWRRGWRYRKA
         . *  ***  :**   ** * ***   ..    *  . **  **   .    .* ***.

35      KYCCRKYMRWKKCCRYWKCRWRATRYAATTSTCAGCYMTRGTKYCKTGGKAYKYRRKRKC
48      GYYTGKYMGTGGTAGCWKYRWGMWACGGACCCMTRTTMYRRYKYTKAAAKMTKYRRKGTY
         *   ***     .  ** **     ..: .  :   * *  ** *:..*  ***** .

35      YGCMRSATKRYKGYRCYYMYTRRRGWRGAATKATCYKRKRKCKYKAGARYMYYTKGRKYR
48      TTTCRGWKKGTKRYRYYYMCYRAGSTRTGGCKWGYYKGKRKYGTKGATRYATYKKKAKCR
            *. .*  * ** ***  *  . * .. *   ** ***   *..:**  *.*  * *

35      KYYWYKRCTWYYGKWYTCRKATGTRCGGGMYCKYMGTYRCAYYTCYCGCYGTKRKYTKCK
48      KYCWYKGSKATTRKACCYGKCCTKGYKRKCTYKCMRWCGMRTTYMTYATYKCKRKTCKSG
        ** *** ..    *     *.  .        * *            . *  ***  *.

35      YMYYSYYYAAGGSMRKWGRYYKYWGCTYYYRTRRYYRYMYGCCKSATMRKKKRSKSKWGC
48      YAYYSYYYCGCASMRKWKRYCGYTRTYCYYGYGRCYRYMYRGYKSCCMRKKKRSKGGAAT
        * ******.. .***** **  *     **   * *****   **. ********.  .

35      KKYKKKWKCARYRYTRRAGCCCYYRYRRMAKTCAKTKGTKKRYTMYCMMGAMMCMCAAYW
48      KKYKGGWGTTRCGYYRRGAGAMTYACGGAMKYMWGAKRWKKRCKMYYMMKMMMMCYGRCA
        ****  *  :*  * **.. .  *      *    :*  *** .** **  **   .

35      YKKYRTGGKAKCGKAKKACCATYAGCGWTRGMGRSRGKTRKTMCMACMRKKTGRYRCATA
48      YKGTGGAAKRTYAKRKKRSMGYCCKSRAYGRCKGSGRKYRTCCYMWYMRKKGAGCRYWYT
        **    ..* . .* ** . .  . .        *  * *.   *  **** .  *   :

35      ATTTCKRCKYAAYWATAGYRGYMRTKSTGGGACGYTCYKCGTKGTCWATAATYWRRGRYK
48      TYGCTKAYGCRMTWRCRSCRRCCRKKCKRAARTRCGTCTMRAGAAMWGCGGKYWRGRRCK
        :    *       *   . *   *.*.. ..       .  : .: *. ...***  * *

35      YAWGMKKKCCYMATWGTTYKRCYMYRKYRYKGKKGGRKTRGTCRKYAARKSATGACTARW
48      YRWAMKKKTMTAGCWKCCYKGTCCTRKYRYKRTKAKGKKGAGAAGYWRGGGWCRCMYRRW
        * *.****    . *   **     ****** .*.  *. . .  *    .   .   **

35      YGWKKTGGRCSGTYACARYKYTRRGMWYYKWKWGTSCSCCKRYKMYW
48      CRWKGCAARMSRKCCATAYKCCARRMWCCGAKWCYGYSMYGGYTCCT
          **  ..* * . ..: **   * **    **  . *    *.
```

The pairwise alignments of two accessions 35 and 49

```
35      RRGWWMMWWMYYYYMKKRKRKSRKSYYRYYGYWRRTRTRYSYRMAKCYWYYRYWAGGGMA
49      RAKTWCAWAMCYYYCGKRGRKSRKSYYRYYRYARGKGKAYSTGCTKMTACCRYWWRKRMW
        *   *  * * ***  ** *********** * * . . **   :*     ***    *

35      RGYRYMRYYKMSCASYTTTTSMYRYRCCCARCRYSYYYMRGWSGCTRYRKRSTRWKMYYY
49      ARYAYMRYYKMSYRCTYWKWGCCGTRTYYRRMGYSCCTMAKWSRAGRTRKRSYGWKMYYY
          * ********  .   . .    *    *  **   *  ** . * ****  ******

35      YYYYYRMATCMRKTRRACKRRAAKRRKMATMMAKGGYRATWKTYKWWAYSWWKAACRCAM
49      YYYCTGCRYMARKWRRWYGRGTWKRRKMTACCMKRRTRRGWKYYKTTTYGWWKCMGAYGM
        ***        ** **   * : *****::   *   *  ** **  :*.***.    .*

35      GYKYKYYMRYCYKYKCTRAGTCYYCKKYKWRWYTGGYACYWYYYKYRTKGWATRARTYCY
49      RYKTKYYMRYYYGYKYCRTRWYCYYKKTGARWYYTATGMCTCYYKYGYGRAGGRMAWYSY
         ** ****** * **  *:    * **   ***  . .    ****     . *   *.*

35      WWRGCCMMMYRSYRTTKYYYRGTMYSWYKCYWWYMMKRAWWYWRWKCGGYMTCMMYMRRY
49      WWRAYTMMMCAGTRGKGCTYGRCMTGWCTYTWWYMMKRGWWTWRWTTKRCCCTCCYMRRY
        ***.  ***  . * .   *   * .* .  *******.** ***.         *****

35      RGKWYKSRRYWKYYRRKWMKYGAYRRRKGYGCKRTKRWYRGMMRGAGKWAYYTYMRGGTR
49      GRGTTTSRRYTKYYRRTTMKYRRTAGAGKYSYGRYKRAYRKMCGRWKKWGCCGYMRKRWR
             .**** *****. ***        *.  * ** ** *     **.   ***   *

35      KYYRRTWGTTRTGTKWRWYAWRGKRYTYMRTCCCCAYRRMWYYRWRMTACRYGYRYYKMR
49      KYTRRATRKAGWRYKWRWYRWRAKRYYCCGGYTTTRTRRMTCCRWRMYRYGTTCRCCGCR
        ** **:  .:    ***** **.***            ***   ****       *    *

35      WRMRYYKCCATKGAMMRKAYRWRYYYMKMYKWYMMRGGKYYCWRKRMGTYGTRWRWKYRW
49      WRMATTGYYRAKRRAMAKMYRWRYYCCTMCKAYMMASRKYYYWAKRMRYTAARWRWKCAA
        ***        :*   * * ******  .* * *** . *** * ***   .:*****

35      GRAATCTCTAYMAGACGCRCCAACCYACCCCYCMYWYGWYRMMCCCWGYRAKRWKARAAC
49      RGGGGYCYGWYMRRWMKMAYTGRYTCGTYTACSMCTCKTTACCYYYWKYRGKGAKMRRRY
          ..      **          .    .   . .*             * **.*  * *

35      SYWAMWGTGTWMRYRMYYKCGYYTCACTYCRKAKATTAMYRRRKRAGKKRMMMGRAKTGC
49      SYWGMWRYACTARTRCCTTYKYYKMRYYYYRGTGGYCGATGGATRWSGGGCCMKGWKYRY
        ***.**  .   * *   .  **.    * * : .  .      .* .      *   *

35      TAGTGGRGTCGAAAARRSTAMGTAAACSMKYWMGYRTGGKRRMWAWAACRYKKKTKMKGA
49      WMTWKRRKWTTMRRRARGGGCAWTCRMSMKCAMAYRCCAKAACTTWGTTRCTKKYKAKRR
              *         *. . . :.  ***  *.**  .*     :*.: * .** * *

35      YRGKYAKCYWGAYMWYRSYRTTAKMRTYRMRYRKKGYKRRKKWWYASKYAWKKMYWWMCK
49      YRRTCTKTYWKRYMWYASYRYWRGMRCYRMRCRKKKCKRRKKTTCWSKTGWGKMTTTASK
        ** . :* **  **** ***    ** **** ***  *****    ** .* **    .*

35      KAMKYKYRTMAYMMYRKGRMYMRKKKWKCYKKYYRTRKMAGYKYRCWKRGTTMTKKMKWG
```

```
49              KTATCKTACARYMMYRTRGCTCRKKKTGYCKKCTGAAKMWRYKTGMWGASYYCGKKAKWR
                *: . *     *****.     ****    **    : **  **    *   .     ** **

35              KCTTATRWMMMGKGKYYYYKTAYKRTYRMRWCCATTMKWRRTAARYKCKYRTGCKCTAWKK
49              GTWYRYRAMCMRKKKYYYYKACYKRKYRCATTTCYYMKWRGCGCGCKYKYGGAYKYCGAKK
                      * * * * *****:.***.**      .  ****  ..  * **  . *  . **

35              ACACKRRYTTGAYAKGTACKRTCKTGCTCGTTWTRTKCGAAYCTACGKYYYYGWCWGCMA
49              WYMTKGGYCCRWTGKCKGYKGGTKYRTYARYYWCRAKYTGCTYATGAKCYYYKAAARYCC
                    *  *      .* .. *    *     .    * *:*   ..   :: .* ***   .    .

35              MTTKWMWGTRTGAYAKYTATGRYAWTATAGWRTKWSTRWGCRTATATRRYYKWRATGRGY
49              ACCGTCAKWRWTGTGTYWMYKGCGTKWKMAARYTAGAAAAYGYRACCRRYYKTGRWKARC
                         *  . ..*       . . . . * . .:  .     :. *****

35              CCCGYTTKRYKKGKAAACGGCTRKGAACGCMWMGGCTTGCATYYYYAKGTCYAGKGACTK
49              YAYRCGKKATGKKKGTTMRRYYRKCTWYAYCTCRKYYYATGWCYCYMKAKGYRKKAGTCT
                 .    .*   * *.::     ** :  .          . .  * * *.. *  *..  .

35              YYWWTTCKTRWRYKKCAAGATCAKKKMCGTYYTATGGGAKKTRYGCCGAGGCRRKWRYKK
49              YYWTYCAGYRWGTKKTGGAMATGTKKCTACCCKCCAKRRTGARYATTATTATRRKARYKK
                ***    .  **  ** ... : ..**   .    .. .    . :**.   .: . *** ****

35              RRCYKAYMRWGRMWMYYCKTTYYKAKKCGCTCKCTGGARCAYWYSKWRKRKRAAATKWYA
49              RRYTTWYMRTRACTAYYMKCCCYKCKKARYGTTMCRRWRSMYATCGARKRKAWMMYKATT
                **   . ***      ** *   **.**.     .      *. *   .   ****     *  :

35              TAGCKCSMKGSWKRCKKCARCCYWWSARRYMTKYCMAYKRRRKCCTCGAKGTWMSTGKAW
49              CGATTYGAKRSTKRGKKTRAYYTTTCWAATMKGTTMRCGRRRKYTCAAWKRGAMGYKGRA
                 .. . . * * ** **       .     *.    *    ****    .. *    *.

35              WGTYSWWRRYSRTCYRRTYRRAWYYARGGCTCYSYCACGARRSRWGAKMWMKKGGTRWRS
49              TKWYCWTAGTCGGGTRRYCARWTCCRRRKMGATSYAMMARGGCGTATKCTCKKRRKRWRS
                   *.*     .     **    *     *     . **.  .   .  .:*    **   .****

35              MGKAGGMYKYRMRRRKGCKGCGKGCMWRWKRCCAYTATRYRTCAMKYYRKMYGATMKGTC
49              MKKGTRMCGYRMGAGKRMKAMKKRYCAGWKGMMWYCCCRYRYYMAKTCGTATCTCCGSWT
                * *.  *  ***    *  *.  *      **     * . ***      *     .    :    .

35              TACYCAGRKTWKRYGTTYTCKCCCAYKWYRRWKGAGRCKTCTATTCKYYKKYWRRMCYYW
49              CCTTYMKAKYWKGYRAYCWATAYSRTKWYRRWKSRRRYKYTWRYYYKYCKGTAGACTYYW
                 .      * ** * :    ... .  *******.  * *        ** *         ***

35              YYTKCRKRKAMYMRRRYRMSYGCTSRKRTRACYAAMKKCYRYKARMMMWWTGGYMSKTCT
49              CYGKSAKRKWMYMRRGYACSYSYWSRTGCGWTYRGMKKMCRYKWGMMCATATACAGKWYY
                 * *. *** ***** *  **.  **.      * .***  ***  **    : .   .*

35              YRMTWTTGYAAWYKRMAGKYKYKYAKYRKYKKTYYGCAKKMRGYYRYCYAMRCTTWTTWG
49              CACYTGAACCMWYKRMRKKYGCKYRKTRKCKKCYCRYGKKMRKCTATTTWARMYKWCCTR
                      :. . *****  **  ** * ** ** *    .****          *  .*

35              ARYKRTTTSRGYAAAKKCTGGTAYMTGTYKKKGGRYRYKYGRSMRGKAYTAYYKACSTTRR
49              MACKRWWCSRACTCWKKTGRRWRCCCCTKKKTKRYRYKYCRSMRRGWYGGYYKMMSAARR
```

```
        **   **. :. **           ***  ****** ****   * .***  *::**

35    MWAGGCRYKKCTRMWKMKWMTRKRKMGKKKYWAKKAMKRGYRRCTKRCWMMWKCYYTTWM
49    AWGTCTAYKKYCACTTMKWCKRKRKCSKKKYWGKKGMKRKYAAYKGAYACCTKYYCCAWM
       *.    ***     .*** .**** .*****.**.*** *   .       * *  :**

35    CKAKRKKTGAACAKKWWYAATKKYYYATTYGTKRWYAYRKYYRKYSRAMGKRYYYAYYKR
49    MKRKRTKGKMWSRKGWWYTGCTGTCCRYWTAATRWCRYRKYYRKCSRWCATGCTCMTTKA
       * **.*    . * ***:. .        .:.**  ******* **  ..       *

35    TCTTYGARYRGTTCCTGSYYTYGKRRGGGTKYRRKKGYAAAKMTAATRTAACARRCGGWG
49    AYAWYKRRYRAGKTTYKGTYYYRKRGAAACGTAGKTAYWMMKCGRTCRYGGYRRGTAAAA
      : : *  ***. .    . * * ** ...     *..*   *   : * ..  *  .. .

35    TCKSYYAAGGKGGCCAARKCRKGAGTGRMKRGRKYKGAGCGYMGCYYRYKKRRAAGKAGM
49    ATKSTTTGTAGKCTAGMGGAGKRGAYRGMTRTRKYKRRKYAYCATTCGYKKRRWMKGRAM
      : **  :. .    ..   . * ..   *.* ****    .* .    *****     .*

35    CMYSRGCMYYYWWKYGCAMRKYYKTKMTACCAKYYKRKMYKTYRCTYTTKKCYGCYYYYK
49    YMYCACTMYYYWWKCCYMMRKYYKKKMYTTAGKYYKAKACKATGAAYYKTKATTTYYYYG
       **.   *******    ******.** : ..**** *  *:  .:* ..*.   ****

35    YKAGTSRTCTRCTGGTRRYMYTYACGAAGCCGYACATTTYTGMGAGGACTYTYKCYYGYG
49    CGGAWSRATCRGAAACRGCCCYYMYKRRTTAAYGYGCYCYWKMAGAKRYYYYYKAYTRCR
        .. **:  * :.. *     *       ..*. .   *  *...    * **.*

35    SMAGATYMMYMAGGCKKCCAGTYWKGRWRWCCTCTRKCWTRATTMAGMGAACTRAAAATR
49    CMTTGAYMMTARRRSKKTTGKCTWGSRAATAYKYWAKMACGRYCMRKAKCMSWRWRRWKG
      .*: .:***     .**  .   * .*   . .   *       *    . . *    .

35    KWRTKRMKATKGTYKYTTYRATAKTCARTGGTTATCAGCCRGCRWKYKCKYRWYKYYMGG
49    TTAYTGCTWKKACYKCYYCGTGTGCYRAKRRYYRCTTAATACYRWKYKMKYRWYKYYMRR
      .   .  . .*. **     : :     .       :..    ***** *********

35    GRYAGCCRRYGRWRRKYMARGCGGMKKRYWCGKAGCAYTTRWKYMYRKKWWRRRRYYYTG
49    RGYGAATAGYKRWGAKYMRRSMRRMTKGCWYKTMRARYYKRWKCATAKGTAARRRYYYYR
        *...   * **  *** *.   *.*  *  .  . * .***    *    ******

35    YWWKGAKKGRCRYCYGKRRRGRCTTAKYSYKGRSKKCGTRSRKYTCRTWRGMRGGYKAYC
49    TWWGATKKAATGCYYKKGGGRRGGKGKYSYKKRSKKMRWRSGKTWMAYTACMRATTGTTG
       ** .:**.     * *    *  ..***** ****   ** *        **.   :

35    TTGARKRYMKKGCWKAWMYGRYKSYMYYKATWWYTRMYCTRYKACAKKMRCGKATGCCKT
49    CGTCATACCKKKYAKGAMYAGYKGCMCYKGYATCKRMYYKACGRYMKKCRYTKGCATTKY
         . .   **   *. **. **. * **.    .*** .      ** *  *. .  *

35    TCRKAARRMRSYKKYWKGACCMGYKYCYAWAGCKAAKMYWKAWKCGCKGKMMYYRMKKKY
49    KMRKRTAACGCYKKTATTCTGATYKYTCMWCRYGGGKATTTGAKYSYKKKMATYAMKKTY
      . ** :    .***  . .    ***   *.   ..*   .. * . * **  * ***.*

35    MYRCTKAATYRKRGRYTKRYKGAWGCAGWGYATYKYAKYGKYWTAKKKYCGYSRGAYYMM
49    CTGAWGMWWCRKRRRCCKRCTKMARYRRARYCAYKCRKYTKYWWRKKKCTRCCGKRYTAC
         .      *** *  ** .         *.:**  ** ***  ***    .   *
```

```
35      YYAYCYTGSYKTTCTCKAARYKKRKKTRCTATCCAARKSRTTGATRTARWRYCMTRRRWR
49      YCGTYYCKSYKYKYGMKWWRYKKGKKARTWMGAMRMGGGRACACGRYRRWRYYCYRRRWR
        * .  *  *** .   *  **** **:*    .     .*: .. *  ****   *****

35      KKSTAACWRKAKKGRRCMKYWCWAWKTTSRTCYAGGRMGATGCGSKMYKAWRYYGWTYSM
49      KGSKTGMWRKWKKRAGYMGYAMTWWKKYGRYAYRKRRMRTAKYRGKMTKMARYYAAKYGA
        * *.:. *** **    * *    **. .* .*   ** ::   .** *  ***. .*.

35      RRTKKGTRMKCMMWWMRYRKGCARAAKYKKCGCRYGRAATAMASRYKAMYSAASKWKYKY
49      GGYKTCAGMGMAATAMRYGKAYRAWMGCKKATTACKGGGCMMRGGYKRMTSTTGKTKTGY
           *. : *      *** *.       **.      ..  * . ** * *::.* *  *

35      RRYYAKCYGACYKKKYARYYRTWRKYRMRKKCYWCAKSYRRAATRGRGYCYMMKYTRKYS
49      AATYRKYCKRSCKKKYRATTACWRKYRMRKKMCTTGKSCRRRRARRRKYMYCCKYCGKYS
           * *    . ****      *********    .** **  :* * * *  **  ***

35      YGKKKYRKYKWYTRACCAMKKKGGCTCCTGTGKARYYKYKKMRCMGACYRGGTCTGARGW
49      YRKTKTRKCKTCAAGYMWMKKTRSYGYSYSAAKRGYCGCKGCRSAKTYYAKRYMYKRRRW
        * *.* ** *  : .   ***. .   . .:.*  *   *  *.  : *        * *

35      WRCKYWYCATGCTSCYTCYKCAASRACMKRCGYRKTATWAYYGYWTATAWRMMCCCYMAC
49      TRTKYWYTCCAYWCMCGTTKAGRGAMYCTAMATAKYWWAWTYKYWYRGCTRCMMTTYMWM
         * **** . .  .     *.. .    .  .  *      * **   . * *   **

35      GYCRSAKGKCAWTTGTKGKMKTRGAAKYCAYGCGYMGCGYRRMYRCAGKRTYRTRKGTAT
49      KYYGGRKKKMRACCACKAKMKKGAGGTTGTCKSTTARYRCRACYAYRSKRYCGYRKAYRC
         *  . * *     . *.***. ....  :  .       *  *   .**    **.

35      GYRTKCRKTTGGGWKGGGCAGGKCGGCACGGYGGYWWGTTKRRWYWMRYKYRRCASYYKR
49      ACGCKARKKATAAAKATAYRKRKYRRTWTTTYKKYWWAGGKGGTYWCRYKYRGATGTYKR
        .   *.**.: .. *. .    *        *  ***.  *   ** ***** .:. ***

35      TGCGRRTKAACGYMCYKKWTATYGTAYRMTCYYWRRYWYGSKTMSGCRWKCYRASKYAKC
49      YTTARRCKWRYKCCMYKKWCGCCRYWYGMWYYCWAACWCRSKACSCTAWKYCGWCKCMKS
           .** *       **** .     * *  * *   *  **: *   **    .*  *.

35      CWRYKAGTWRTTCWKGWRKTAAKCACKAGGGCYYTCTWYGRYKGKWRKRGTATCARYMMK
49      MARYKTKYWAWKTTKATRKYMMGTWTGGCTATTCKSKTTKACKTKAGKGAGGCATRYMMT
          ***:  *  .  *. **        .  .   ...     * *  * . . .:****.

35      RCCAYKKRCKGAAKYCRRKYKKGACTYKRMRKMKGRAACMYMKCGGAAAYWCMCAGYWKW
49      AYYGCKKRMGKRGKYTGRKYKGACMGTKRMAGCKAGGGMCTCGTTAGGRYAYCTGRYWKA
           . ***    .**  **** ..   ***   *. ..       ... *    . ***

35      ATYAMCCKMKTAGMMYYSKRAAGGWCYYKYGAYMWTRMGYCGGYRCTTGTKCSKKWRSKS
49      WKYCAYSGAKYWRCACYSKRCGCAAGCCGCRMYMTCRCRTYRKYRSYYKGKGSKKTRSKG
         .*.  .  *      ****.. .        **  *      **.    * *** ***.

35      RRWKYKKKWYRYKTCTGAGRRGMGAGAYKKGAAGYKTCGYRKKRCTYRACMMGAMYGKAM
49      AATKYKGGWYGYKYMYRWRARRMSRSMCKKAGGACGKMRYRKKRYKCRCYMMKMCTKGRM
           ***  ** **       * *. .  **....  .  ***** . *. **       *
```

```
35          YKMYRKTARYKCWCAKWTCAAYGAYGCGRMWWRWRCRTMMRRRKMTTTRAYYKKTCAGAT
49          YKMYRKWRATKYAGGKWKTGRCRTYKSRRCTWRWGMRCCMGGRKMCCYRMYCKKCTTAGY
            ******    *   .**. .   :* . *  ***  *  *  ***   * * **  :..

35          KRYAYCCCARCYRWKTCTTKTCTGTGRRGGGGWKKMRKCTGARGCCGKMRYMYTGGRRRG
49          KACTCTTTGGYCRTKCGCCKWTWKYTRRATTTWGGAAKMCAGRATTTGCACCCGSSGRRK
            *  :    .   * *    *      **.   *    *  ..*.          .. **

35          KTYAGGYCMTTAMRYTTTCTTTCMRWCAGAKKTKCGTAKGGYYCYRTCKATTWKYKMYYR
49          KWYMKRYMCAWMMRYYKYYKACYCGWYRRRKKYKSKYRTARYYYYRWYKMYCWKCKMYYR
            * *   *  :  *** .  .:    *    ** *.   .. ** **  *   ** *****

35          CRRTWSMKRWKRGGWATKRYKYTAKMYTRYTYYKRAMGCMTCTAAGWAWTCKAGGYYSCC
49          AGAWWSMKGWKASKWMGKRTTYYRKCTCACKTTKRGMATCWYWWRRTTACTKRKKYTGTT
            .   **** ** . *  ** .*  *     .  **.*.         :   *   * .

35          TTCTYMCTARRSGKYKTKGCAACWRKRKCCTGTMRYWRYKYKRRRKAKARKRTAGGYRGY
49          CCSYTAMWRAACKKYKKKATGCTWRKGGYYAKCMGCARCGTKAGGKGKCGKRYWRATATY
              .        . ***.*. .. ***    :  *   *   *   *.*. **   .   *

35          TYKMATKTKATARWKWGGRSYWYMKYYWMKTRTGTCCSRTKYKTYKAWKKCTKYWCTGYR
49          YYKAGCGAKMYRRWGTAARSYWYCKYCAAKKRWAWYSSAGKYGKTTGWKKYYKCTMKKYA
             ** .  :*   **  ..***** **   *.* .  .*  ** . ..***  *   . *

35          KGGGAGAGCYYMMKASWYRWKYRYGRGRCKGCKYGWWSKTGKKCTCATTKGRRTCTYATK
49          TKAATRRAYCTACKMSWTRTKCRTARRGYGAAGCRAAGKCKKKTATWKAGTARCYYYRYK
            . ..:  .     * ** * * * .*    ..     .*  ** :  .:   *   *  *

35          GCKWYRKCRKTCGRKGTGKTCCTTATTRCWCAYTCYTGCCATWAKTYAYRKCCAYYRRRY
49          SSKWCAKYGKAARGKKYRKCTTYCMYWRYTATTKGCYRTATCWMKCYRTAKTATYYRRRT
            ..**  *  *:.  *   *        *  .: .     .: * * *   * .:*****

35          MAMSYRTKKYYCGTTKTYYYCMKGYRCATCCCCAMWRARMAATCWYWCAYRMWWRKKCCR
49          CRMSTGWGKTYTACAKKCYYYATATATMYYSMYRCTGMGACCCTWYTMRYRMWWRKKYYR
              **    * * . :*. **  ..      .         ..  **   ********  *

35          CGMAYKYKKCATACYRYKRKRRSWYAGTTTYYMRMSGKWAWRYRGYTGTTSSKMYTTMKW
49          YCATCKYKTTGCGMTRTTGKRRSWCGAAYKYYMAMSRTTCWRCGAYGACCSSGCCCYCKT
               : ***. . .  * . ***** ..: .*** ** . .**  .* .  **      *

35          KYKMRYCKMCWCACRYWYMRWRTRYSAAAYRAKRGTKTTYTGAGTTCCRKAKTYGTCKAY
49          GTTCGYMKAYATGMGCWYCGWGWACCTGMTARGRRYKCCTYTGAWGTARKMKYYKATKMT
              .  * *    .   **  *    .:.     *  *     ..   .** * * : *

35          GTCKRKMWRSKYGRYYYKMRRRYTRGKKGAATGCRKATCYYWRMTTRRRKYKSTRYTYYA
49          RKTKGTCWRGKYKGYTYGCARRCYRSGKTGGCAAAKTGYYYWRMCGARGKTKCARYCYTR
             . * . **.**  * *   **  *. * .. .. *:  *****   * * *.:** *

35          CAKRTCGCTCTYWYTKCTGTRTRGMYCMTTRYAYKTKYCAAKYYYSKAAAGMGYKCAYKG
49          MRKAYYRSKYKYACCKYYSYGKGKCTYCYGGCWYKCKYAGGKYYTSTGTGRAAYKYRYKR
              *    .. .*   *  .  .           ** **...*** *..:.  .**  **

35          GGAATRTKTKGCCGAGGKKAGCTGKCKTMGYWKYRYYTATATTAACAYGRRYKAGTCRYT
```

```
49      SRCRYRKKCKRYYRGTRKKRRTCATYTYMRYTKCACYKTAGCCTTMRYSRRYKMSYYRCA
        . .  *.* *    .  **    .. . * * *   *.::.  ::  *.**** .  * :

35      GRKAACCKMRYGGKACGTGTRMCKKTCCTRKTYAKYRMYYWRSCTAATAAKKCKMMCKWM
49      TRKRRYMTCRYKRKGTAAACRMMKGCTYWAKCYMKTAMCTWRSMATGYRWKGMGAMYKWC
         **    . **  *. .:. ** *      * * *  *  *** ::.   *    * **

35      YRWWGRGRRKTKTKWMRYYTCKCWRGCCCYMMTGWTMRYYSKYYGTRRCKKGKMACKWKT
49      YGWWSRRRRKYGKTAMRYYYYKYTARYMSCAAATWGMRYYSKCYRKRRTKKKKMRYGWKK
        * **.* ***  .. ****  *      .   : * ****** * .** ** **   **.

35      YAATCAGYTTMKAACKKMMRMRAYRRYCRGCTAGAAWCGYYYRKRTRWKGYYMYMMGTCA
49      YRGCTTATKYMKTCTKKAAAAGGTRGTYRATCMKRRTTACCCRKACRWTRTYMYMAKWYG
        * .  :. . **:. **     . *   *.        .   **  **.  ****    .

35      GATMTWTATAAYMKKWMCWTYCRKCYKRTGWSMSKYKGCRTGACCYKKYYYWAKWYYRYR
49      ACAMYAYWYRRYCGGTCAACTYRKTYKRCRASCCKYGKSGGAGTTYKTYYYTRKTCCRCG
        ..:*       *     .    ** ***   * .**  .  ..  **.***  *   *

35      KCGKRYMAWAWSYSRTKTCYYMGCTARGTYKMGCCASTCCWKGKKWTYAYSGGYAYMRCR
49      GYKKAYCWTMTSYSRKGYYYYMRAGTARATKAKMYRGCYYTKKKKTATMYSARTRYMRYG
           * *     ****.   *** . :  : *     .    * ** :  **.   ***

35      KTKKYKRTRYKYYAMRKKKCACYYKGTTCTTRMACTCYKKSCMTTGYRGCTCWSMTAGCK
49      GGKKTTGYGTKYCRARTKKYCMCYKRYWTCWACRAYMTKKGMMYKKCAATATWCCARSYK
          ** .    **   *.** .  **         .   **. * .   . : *. : . *

35      TKCATGTAKTKGGCKAAKMRAKGCTYKAWGGYRCAKKACTAKCGAAACMKRCKYTTYTAT
49      KKYMCACGGCGATTGRWTMRRKAYKTKCAAAYRYGKKWAGCKGKTRMYCKRYTTWYCATC
        .*   . .   .     .** *. . *. ..** .** . .*  :    ** .    ::

35      RRKCCKYYKRMYWRMYRYYTGKKAGGGRKWRTWYRWWWCGCACKWKMGCYWYKWGCCYYM
49      ARKAATYYKRCCARMYRYYARKKCAACRKAAWAYRTWWYAACTKTKMKTCTTKACTYTCM
         **...****   ******: **... **    ** ** ... * **     *      *

35      KYRRMKRRKKRSKAYTAMRMGGTTGRKTKGMAYYGRKKWKAAKRATCMRYRAGKGYASCK
49      KYRACKRRGKASKTCYMCGMTTGCARKYKKACTYKGTKWGRGKRRYYCACGGAKKYWGAK
        ***  *** * **:     *    .** *  . *  .**  .**       ..* * ..*

35      CRYGGCAKKKYYYYWKWWSTACCAWRGGGGATTATAGGRKGKRGCCCCKKKRSGGYGYCW
49      YGYKKYGKKKYCYWKWWSWWMYRWRKRRRMKKMWGAARKRGGKMMYYKKKGGTAYKYYA
          *   .**** ******     **     ..  ...**        *** . .* *
```

The pairwise alignments of two accessions 35 and 50

```
35              WRYYKGAYYKTTTMYKKKMWYYMKRMAACRYKGRGYRRMYYRCYYYTRRYYKWCRRCKRW
50              WRYYKAGTYKAGCAYKKKMWYTMTACWWYRYKKGRCGGCCCGGCCCYRRTTKWYGRYKRW
                *****.. **:   ******* *.     ***               **  **  * ***

35              YWMCMRMKMKYSKSACYYRRYCYRYWCYRYRKCRSCCMARWYMYMTGYKKWSYWAYYYYK
50              YTCYMACGCKYSKSGGCTARYTCGTWTYRTRKYRSAMCGRWYMYMCATGGACYAWYYYYK
                *   *    *****.    **    * ** ** **.  .****** .    .*  *****

35              GTRMAMTTKYYCWRWCTAGTGYKKYYRRTAKWMKYRTMMWYAYRSYRCYYYRYRGTMYSK
50              KWGATMGKGYYYAAWYCWTCKYKKYYRRAGTWMKYRCCMWYMCAGTGYCYYRYGRYATSK
                    :* . **   *      *******:..*****  ***   .    ****     **

35              RWYTGTRYWYMKWRRMYCWKGYMKYKRYYGGTRYAWMRYRRKWRWYMWYYRRRTTMCMCC
50              RWCGAGRTWYMKWGGATAWTACCGCKRYYKRWRYGWMRTRRGARWYMTCCRARCCCYMMT
                **  . * *****    .*..    ****   **.*** **  ****   * *    *

35              CCGYTYTMYYMGRCTMCYMTTSMAMRMRKYRMKCARWKMMRRAMKAAAGYWYYTKYKWRK
50              AGTTCTCMYYMRGGCCYTAWKGCRMRMRKYRMKYRGAGMMGRTCKGGGACTCCYKYKWRK
                .      ****         ..  *********     ** *: *....     ******

35              RWSRKRSRTTSAAYYTMKWTYKKRYAGWTWWRAGTTKRMRWKTKWWRYMYYYYYYYWKWR
50              AWGAKRSRKACRGCCGAGWYCKKRYRTTKWWRWRGGGRCRWKYKTWRYMYYYYYCYWKWR
                 *. ****.:. .     *  ****   .***     * *** * ********* *****

35              MMGAMWKWYKRRYKTCCTKWYKRTRWRYMRYMRYYYAGYRYRKSYMRRYYMCWMYCMYRR
50              MMRRCTGTTTRRYKGTTAGWTKGKGWRYMRYMRCTYTATGYRKSCCGGTYMYTATYCCAG
                **       .****   : * * . ********  *:.  ****     **

35              KRRYKSRMYWRKAYRKYAGCTKMWYWRCCGYYYRKRGTCYKYYMYSCTARGTYAGTWKMA
50              KRATKSRACTGKRTGKYGAYCKMTCWGYTACCCRGRACTTKTYMYSYKMRKWYGTYATAW
                **  ***    *   **..  **  *   .   * *.   * **** . *  *.   .

35              RCYACKYRGKYGTKCCKMYMGCRYKWKMAKGCTTYYAYTYRRYCGARYMTAGMWSYKAMG
50              RTYWYKYRRKYRWGMMGATAKSGYKWGMRKRMYYCCWTCCGRYYKCRYMCRRCTSYGRMR
                * *  *** **          . *** * *           **  .***     **  *

35              YCYMCASYWYKKYSGYCRGCMRRKTKYKRCTRYCYKRYYGMRTKCYYKTCACTCKCMYYR
50              TTCAMRGYTCKKYSATMRRYMRGGGKTTGMYGTMYKRTCTARWKMCYKATRAYAKMMCCA
                      .*  ****.  *  **   * .      ***    * *  **:  . .* *

35              TACGTTCTCTTSMTKKCYKATGRATGTKKCAGTCATGGMKMSRASMRRYYYKMWKYRTCT
50              WRYAYYTATWWCCAKKYYKGCRGCCACKGARAKYRYKKMGASRWSMRRYCCGMWKYGYYW
                   .   :   . :** **.   . . * . ..     *  ** *****   ****

35              GGYMRYRYMYKGCCKKGCTGCSRRWKKYRRRWSKKKWKCARYRYKGRTACYYYCAGGYCT
50              KRCCRYRTMYKRGYKKAMWKYCAATKKYRRRWSKGGWKTTRYGYKTRKRMYYYAGACCTC
                    *** ***   **.    .   *********  ** :** ** *.  ***...

35              RMGGCGAMMAAMYKAAMKKKKRYSWWKKYTWGAMAKWGMYTAAWGAKKTACKKGWCAWAR
50              ACRRMRMMMCMCCKWWCKKKKRYSWWKKCGWKMCGGWAMYKRRATWKTWRMTKRAGGWCR
```

```
                       **.   *   ***********  *   . *.**.     *.  .*     .*.*

35              YYKWKRKRTRRRTMAMGAKMRYAGTTKTKAYYCCARCYAAWMRTKTTCAKACACRYTKYR
50              CCGWKRGRYRRRCCMMKWGMRCGRWYKYKWCCYYRATCRMWCGCKWYYMKTTCTRYCKTR
                   *** * ***   *   ** .   * *           *   *    *: . ** * *

35              TMKWTRYKTAAKYWCCCGGMAMTGKKRTWMWTYYTTYKKTATGGRSAWGAYKRATATKTK
50              KAKWCRCKCCCKYWAYYRAMMACAGGRYTCACTCWWYKKWMYARGGGTTWYGRGKWKKYK
                . ** * * ..***.   .*   .  *         ***   .  ..    * *.. .* *

35              RKTCKWSTTGTCRWWYATGYRMKACTTKYKMTCRCGGYCTGYKMYTTAKCTYTTGYWKCR
50              RKAMTAGACAAMAAWYRWKYRMKWSGKKTGCYMGMRRYYYRYKATCAMKGATACKYWKMR
                **: . .: .:   **   **** . .*         *   **   : * : :  *** *

35              WRGAYCCRYWTYYWCTWRRSKGACKGACGTKKGAKAAWRTTYGRTTCAGATCTTTRYTRY
50              TGKRYYYRTAGTCWYKARRSGCTGKRRTACKKRRTTGTRCCCRRKGAWAGCYGYCRCCGT
                    *  *     * . ***  : *   . **  .:. *    *. . ..     *

35              SYKCGCTAWAARATCCGKWGRTAYYSRTMYYATRKTTCGRYKKKTGAKCTAARKSKTARA
50              CCTMRAYRWRWGCGTYRKWARWRTCCGCCYCTYGKYKYKRYGKKYTRKYCWRGGGKYRGT
                . .  .  *   .    **.*    .   * :  * .  ** **   *      .*   :

35              WYKKGSYTYTAAKYKKCMKCGCGKTGTAKKKCGWWCWAWMRWSYRKCGSKGTGMRGGCAY
50              TCKGSSYKCCGWKYTGYCGAKYRKCSCGKKKTRWAMTWWMRAGTGKYRGKRKRMRRTYWC
                  * .**.  . **.    .   * . .***  *    *** .  *  .* . **

35              ATWWTYYYRMYTYMCKMYGAARRMMRSTKAKYWGYGCAKKRRMYKKCYMMYTWKMCATGC
50              GYATKCCYRMYWYMTKAYKRWAACMGCKGGKTATYRYCTKRRMYKKYYCATYAKMYGCRM
                .   .  **** ** * *      * .. .*   *  ..******* *     ** .

35              CCMYAKMWRYRARMKCKYCYYYCSRRCCTRATTCAYWYYMYMCGARAGRGGGGCTGACTY
50              YSATWTCWRYRTRCKYKTTYCYASRRGYYRRYYGCCWYYCCAYKRRGAGARKSYYKRYYY
                 .   . ****:* * *  * *.***   *    . ***      *.. .  .      *

35              YGCGGYYYASYCCCCRTRMMYMMAGGATCCAGAWKYWKKKKAYAATCAGRAACCCRWAMA
50              YTAACCYCTCCATATGGAMMTMARRRCYTTGCTWKTWGKKTTTRTWYGCRWRATGRWMCM
                * ..  * :. . .    ** *    .   . :** * **.:  :  . *  .  **

35              CTRAAATRAGTTCMYRMWTATAKCCACTCGCGAWTTGTGKYRRYSYKGCRRKKTMGCKTA
50              SWRWRWKRGCCYYMYRMWKTGTGAYRMATATKMAGGACCKYAGCGTKTTRRTKYMKYKWR
                . *   .*.    *****.: : .   : .      .  **   . *  **.* *  *

35              MTYKKWKMTYCCCWRMTCWRRKYWRGACYGWYGSKWTAKSKACYRGRKMGCCWKGKGGWR
50              MYYKKAGMKTYYSWGMWYWRGTCWAKRYCRWCRSKWYMTSKGYCGARKASSMAKKTRRWA
                * ***  *.   .* *  ** . *      *  ***  .**.   .** ..  * .  *

35              MCTTACTKWRCGAKKWRWGTGAGGGCYYKRYGWASGTTAMMYKKCCATTKTCAKCAGYYK
50              MYAYRYKKTRTCTKGWRWCYRGTTATTCGACKACCKCYWMMYTKYYGAKKATTKYGKYYK
                * :   .* *  :* ***   .  .        ..    ***.* .:.*: :* . ***

35              ARMWTYKKGTGYKRKMGACGGAAMCAGYGKKYKGTATCGTYYGYYRCGRTGRKYKKKTMY
50              RRMWWYKKAGCTKAGCATTTAGGMYRRYKKKYKKKTAYAKCCKTYRTAAWRRKCTKKYAY
                 *** ***.   *   .:   ...*   * ****  .:: ..    ** .   ** .**  *
```

```
35          RYCACGKYYMKKYRGTARKYMCTYRMRKRKWWYAKKYTWKMYWWKGKYAYKGMMYKMKKK
50          RYYRYRGTCAKTYRRCRAGCMYCYRMRKRKTTCWKKTGWKMTTTKRTCRYKRMMYTMKKK
            **        *.**      *  *******    **  ***   * .  ** ***.****

35          KKCYYRRKKCYKYWYKMKKCAKAWKTATRGRCRRKTRWRYTTAYYYWSKTACKCTGRCKA
50          KGTCTGAKKMYKTWCKCKKYGKMWKYRYRRGAARKYAAGTAWMYYYWSKACYKYKKAYKW
            *      ** ** * * ** .* **   *  . **     :  ******:. * .   *

35          TAAKARGTKTTCGATCATTRACAYTKGKWYRCWRRYMCTTTYGGCYRCGCTATKCRTSYR
50          YWMKGRKYKCCMTMYYWWWRWMWCYTRKWYRYTRRTCMGCACAAYTATSTGMYKTGCGYR
               *.*  *          *     . ****  **     : ..    .    *   .**

35          CGGMGTMWKYRMMRRGATAMYKWYRRMYGKKCCATAMAKTWKAMYKAGTCAAACWRKRMG
50          TRKMSYATKYRMMRRRTCGATKTCRRMYKKTSTGCGMCKCAKRAYKRRWMWRRMAATGAR
               *.   ******* : .  *  **** *.. . .*.*  *  **          .

35          KWACYSYWRYYTRSSKGTGTTTTWYSRAAAATCKRYYYATCTGGAYAGAGKKRWRYTGAW
50          TTCYCSYWRYTWASSKACAYYWYTCSRWWWRWMTGCTCRAYCKARTRSMKKKRARYYKTA
            . .  *****   ***. .      **      .     :   .   .  *** **  :

35          GCKRGCKYRRRKAAAKTRKYKATGACARWKMWAAGGRKYAAGGKRTMCAGCRRMSKKWCA
50          ATKGRYGTAGRKWMMKGRKYKRYRRYRRAKMWTWCRGTYTGTAKGCCTWCTRGASKKWAT
            . *       **   * ****      * ***:    .*:. .*       *  ****.:

35          CKGRRYKGMWGGRRYKWYTWCYKMCGGGSRRAASCARMGTAYCMMWRYMYMYYYTAYKYG
50          TTTRRYKACWAARGYKWYGTYYTAYRRRCRRTTCTWGAACGCMCMWRYMYMYYYGTYKCC
             . ****. *..* ****   *.     .**::.    . .   ********** :**

35          CAGMRKYYMKKAWKTCTATKYRYYACCKTTAKRGCKTTTYWKRKTRMKRKGRRCGRMRTT
50          GRRMRKCCMKKCAGWYCRKGYRCYTYAKCGTKARSKWYKCAKRKYRMKAKAGGTAGMGWW
               ***  ***.      . ** *: .*  :*  .*  .  *** *** *.   . *

35          ATACARKRTAGGKSRMMRKTAAKRKTWRSYWYKRWKTGTWMRACYRTKYCTKTRRAGCMC
50          MWWMCGKRYWRTGGRMAGGATGKRGYWRSYWYKRWKYACACGTYCGCGCAKKKRRRRMCY
                . **     .**   ::.**  ********** .    :      ..*.**

35          MSRGACTTKWKKGCTTTAKMWCTGRKCKRGKKYWWKGRGGCYCKWGWGGTCKYWWSKTGA
50          ASAKGYWWTWKKRYYKKMKCTMKKRKMKAKKKTWWGARRRTCMGTKWKSGYGCAAGKCKG
             *  .   .***   .. *   . ** *  ** ** .*        * .      .*  .

35          ATRTKYTGACGTRCTAYACRAAAKYCATGGTRGKKKCGRTTYRKACAYTRYRTCGGKKYC
50          WKAAGCWKRYKCRMYCCRYRRRMGCTGCTAAARKTKARGWCTGGWMRCWRYRYYAAGKCY
             . :        *  .   *      .  .:  *.*.             ***  .. *

35          RTKYGTTTGATMKGTRKCTACTCGTYYYKGYRWYGRKTTTKYRMMYKKTGGYAKYYWYAM
50          GWTCSYACCTCCKSWRKTCCTCMTCCCYGKYRAYSRGYYYKTRMMCKGCARYRKYYWCCC
              . . :  :  *. **  .       *  ** *.*    * *** *  . * **** .

35          CCCGYRTKRGKKRKWCGRRMSYRKRKRYRAGTRYYKTATRCAMWWKYMSKRYKTAYRYKR
50          ATTKYRKKASKKRKTMRGACSYRKRTGYGWRKRCYKWMYGMRCATTCAGGACKGMTRYTR
            .   **.* .****      *****. *   .* **         .  .   *   **.*
```

```
35    MTCGYGAKCCYYKMYYKYYKYKKYYGGCKKMRCYRYYYRYCTTWKKTTKWGTYRTTAYCK
50    MWMRYRGKMYTCKAYYKYTKCKKYCRRYKKMRATAYCTATTYKWKKCCGTRACRWWGYYK
      *   * .*    * **** * ***    ****.  *      .***     : *  .* *

35    TARRKGYRMYAKCKKKRAWMWAAWRRYYYKYRTTARYAGYTCRMRWCKTMGGKGSTTRCM
50    WRARGTCRMYCKYTGTAGTMTCRWRRYCCKCGCCTRYTSYWYRMRWMTWCKRKKSACRTM
         *   ***.* . . . * . ****  *    :**:.*  **** .    * *: * *

35    CYKRKTYCTAYAAKYCRYYYWRRWYKAAMMCTGAWMKRCGRWGKTKYKKMRYYTCCWRRK
50    MYGAKCCTGGTCMKTTACTYWRRWYGRWMMYCACWCKGYSRTRKYKYTKMRYYYYYTARG
       *  *    . . *     ******   **  ..* *  .*  * **.*****     *

35    YGTMYYSKYGCKKCKKKACRCKATTTMYKMKTTRTTCTWASWKRKWAKYRRCWWKKKMRK
50    YKGMYYSKYRGKKTKKKRYGMKGKYCATGMKCGRYWMYTWCWKAGWMKYRAYWWGGGAAK
      *  ******  ** ***    *..     **  *      .**  * ***  **     *

35    WAMTTCCTARRKAGMTKYRAACCKTAKRWRRYTTATWCAKKCMCAATYTGTAKGTCTTGY
50    WGAKAYYCGGRKMKCAKYRGCMYKWMKRWRRYKGCGWYGKKSCSMMWYGKYRTRYYYYRY
      *. .:   . **   :***..  *  ******. . * .**. .   *    .      *

35    AAGYYGMSCTCGCKGAYKMYYRAKAWSKKRYWKRYKRYKCGWKRWKKMGCMKRSWTWTCY
50    RGCYTKMCYWYATKRGCKMYYRGKWWSGKRYWGGTGRTTMRTKRWKGMATCKGSTKACTT
       . *  *.   . * . *****.* ** ****    * .   **** *.  * * .

35    STYMCRYRSKKCGTTGTATKAMKAYYTCTAMWYSKTYKGTKYYMCYKTMMWMRYRAWCMK
50    GYTAMAYACTKYACAKCRYKRMKMYYAAAGCACGKYYTCAKYYCYTKAAAAMRYRRAYAK
      .     * ..* . :    * ** **:.:.   .* *. :***   *:   ****    *

35    RGGCGRKKRYRKAMRASYAAYMYYMKKSMMCYSKAASAWKGKGGGRRYYYKCYKYCTTCR
50    ATRYTRKKACGTMMRRGYWRYMYTMKKGMCMTSKTTGWTKTKARRAATYYGYCKCMWKMR
           ***   . ** .*  *** ***.*   **::.  * *.     **   *   . *

35    YKYYARYYRTRKMRKKYGMTKACWTARAATAGYYKKKGRRMAKKCYGYTGMYWTACAMKY
50    TKYYRATTACRKMRKKCRACGGMWKRRRRAMRTCKKKRRRMMTKMYRCYRCTTAGMWMKY
       ***      ******     . *. *  :    *** *** .* *       :.  ***

35    KSGGCGYAYKYTMRGGWTTGCCRYKWWCRCRGAATGYYASYYRWKRGGKKCACKCACRAM
50    KGRSYSYRCGCYCAARACGRYYRYKWTTRTGRMCCACTRGCCGTGRAATGTMYKYRMRGM
      *. . .*       .       ****  *    . .   .     *...    *   *.*

35    GACYRKWYYGTTARCCYCCKAGYCACYCWYATACAGKWTRKKMKRWRKAAAYMWCRYMRM
50    SMYYAKWYYACYRRMTYTTGWRTTWMYYWYGWRYGRKWARKKCKGTGKGGTTCATRYAAC
      .  * ****.   *  *         * **.   . **:*** *   *..:    **

35    YYTCCCRRTRYCGCKCWTRWKTMYKSCTGWMGYRRACTAKCTRACACCMCMKRCTYYYRW
50    TYCTATRRCRCMKYKAAYGWKYCTKSAGKAMRYRGWAGCKGCGMSTAYCYCKRYKTCCAA
       *  . ** *    *.   **   **.   * **  . .*    .:.    ** .

35    WRTYYYTRTCGMYWMGCYTKWRWRTWYRWWWKCGCACKCKCCGCMKRMKKKKCCTKKKYK
50    ARGYYYWRAAACCAMRYYAKARAAWAYRTWWKYAACTKSKYYCTCTACKKKKYYKGKKYK
       * *** *:..   *  *:* *    ** *** ... *.*     .  ****  . ****

35    RKYKRYMMRYRKKGCGTRYKRKMWMAKKRATCAAAGKGTCKGYCASKKKGGYARYWYWWS
```

```
50          AGTKRYMCGCAKKKYRCRTKGTAWMGKKRRYYRRWRKAYYKKYYWGKGKKKTGRYWTWWS
               ****    **    * * . **.***       *.  * *  .* *   .*** ***

35          MWRKTMWGCAGRCWCYRYCYCCTCKKSGGYYMYCWTAGKKACGGYYARKMWAGTAGARMT
50          MWRKCCARYMARGWMCGTMTYSYMKKGTAYYMYYAGTAKKWYRKYYWRKAWGKCWRGGMK
            ****      .* *       .  **. .****   :.**    ** ** *.    . *.

35          YAYYKKGCCGCTTRMWACWCMYYKKMKYCKYWAMKAMKYRRCTKWCKYWCYWMYKMAAKK
50          YWCYKKRMYATCWACTGMWACYYKTCKYYKYWGMKGMKYAAYKGTYKYWYCWMYGMRRKT
            *  ***   .      . *. ***. ** ***.**.***   .   ***  *** *  *.

35          TAAYAAKTGCGTKKYKYYYAGYAYGTGYYWYYAGYYRRGYRKGAKYTGYKKYYYYRWYKA
50          GRRYMWKGCARWKGTKTCCWRCGTAGACTWCCRKCCGGRYRKSMKTGAYTKCYTYRWYKR
               *  *  .  *  *      . . .  *         ***. *  .*.* * *****

35          GWYMRRCKRWRRKKMARGMKKGARCRYTKYRWYMYRCKWRRRYYYTGKYKAAATAKKGAK
50          RTYMAGAGRWGAKKMRRKMTKRRAMGCGTYAACATATGWRRRYYYYRTTKRRMYWKKATT
              **  . **  *** * *.*       .*        *******  .  *     **.:.

35          GRCRYACRGKRCAGCTKRKYSKYTCAWRKGRSCKKRSRKKYMKKRKGKCWMMTYKGRYCC
50          RRYRCMYGKKGMMRGGGRKYSKYCTRTRKKRSMKGRSGKKCCKKAGKKYACMWCTAGYYS
             * *     *       ******    ** ** * ** **  **   *   *  .. * .

35          ASYMMKMKKYWWTRYCYRYSYMRKGTKKRTGTCKYGTKTTRKKMWRKATRYYYTRKATCY
50          RGCMCKMKGYWWWACYCACCTMRKRYKKRYKATKTACTYYGKTCWRKWKRYYCYRKGGYY
             . * *** ***       . ***  ***  : * . .   *. *** .***  **.  *

35          WKKTRYYKKGRTCRGCTCTYGGGAGMYSSWYTRTRTRCGMYMCAWAGARRYKKYKTATYT
50          WKGARYYKKKAYYRRSKYKTARAGAMYSSWYCGCRKGYKCCMMTTWRGRRYKKYGYRYTC
            ** :*****    * .. . . ...******   *.     * :   .******

35          KTTRYRAARCTCTCWRMGGCARAARGWRMMKMMRGGTACASYTCGCGSKGRRGCCMYYKA
50          GACACARWGTGYCMAAMACATGTMAKTACCKMCRSRYGYGSYCTRTASGARRSYYCCTTT
             :              *. .: :       ** *.  . .**   .* .**.      .:

35          GCTYTCYAGAKGGWTTCACKAAMGTGTMMMRATGCAMCGRKAATCMCYMGKAGGCKKWYY
50          KMKTCACCTCKATAGKYCAGGGAKWRWCCMRWYRARCSKRKGGGTMTCCKKTTATKKATT
              .  . . .*.   . .. ..       **   .  . **..  *    *: . **

35          RYKKCCYKRGKYYCCGCATRKCKKYACKRACKKGAYCKYWSKKAMAYYCAKMSAKGAAKT
50          RYKTMYTTRRKYYYTKMCCGTMKKYCTKRTTTKRRYSKTTCKKRMRCYYWKMGRKKMRKC
            ***.   .* ***    .  . ***. **: .*  *.*  .** *  *  **. *   *

35          ATARRWAWYYCAYYCARRSKGRTWWGYKGMKRRYWMKKGGMRSMRRKMTACAKWRMRRMY
50          TCTRRWWTYCTTCTMRGGCKAGKATKCKACKRRCWCKKRRARSMRRKMYRMRGWAAGAMY
            : :***  *  :      .*. .    *. *** * **   *******     *    **

35          YRYYYAR
50          YRYTCCG
            ***  .
```

The pairwise alignments of two accessions 35 and 51

```
35          KMYAGYYRWMRTTKKCCCCRWYYACYYWRAMYYMCKKMYTTTSMYRYMGASGRGTTTCYR
51          KMYTRYYGTCGGKGKYYMYAWYYRYYYWRWMCYMTKKMTWKWGCCGTMKRCKGRWGCMCG
            ***: **     . *     ***  **** * ** ***  . .    *  .

35          RCWYCKTYYMKRRKTYMCTRWWYTMTKCYMRRRKYACTKMRWRYYRYRGYYKGMCYKKRY
51          RGWCMKYTTCKGRKGYMYYGTWYKMAKYYMRRRKYWYYKAATATTGCGATYGKAYYKKRY
            * *  *    * ** **    **.*:* *******   *         . *    *****

35          YMKRWYYRRMSRRKWMKYTWAYRWMRMYYTAGGYRWRYRGSYYYRYTYKRWYGKWYMKRR
51          TMKRWYYRRMSRRTTMKYCWWCGWMRMYCYMKAYGWRCAAGTYTRCACKRWCRKWYMKGG
             ************. *** *   *****    .* **  .. * * : ***  *****

35          MCYYYAGGMYWTRYKCCCWMMMATCMAAMRATAKCKARTRCKKCMYYMYRRRWWYRKYKG
51          AMTYYMKRCCAGRCKTTAWCCCCGMAGCMRWYWKYKRGCGMGKTACCATARATWYRKYGK
               **       * *  .*   .   ..**   * *      *       *  *****

35          KYKSYKRTRTTTYYAKTYGCMKWYKWWKMCCMTMRMWRYRATYRAMWKRGKWWRYMRWYY
51          TYKSYKRGGYWKTCGGATRYCGTTTTWKMYYCCMRAWRYRTGYGGMWKRRKTWRYCRWYC
            .******    . .  :       . ***    ** ****: * .**** * *** ***

35          WYYYKRTSWKWTYMRYRYGGTRKKYYKRRATYYWTWMYYTTTMRYCRTYWYYYWGYRKYY
51          WCYYGAGGWKWWYMRYAYKRWRKKTYKRRTCYYWCTMCYKAKCRTYAYCWCTYWRYRKYY
            * **   .*** **** *   *** ****: ***  * *.:. *     *  ** *****

35          RKYYYYYYATKRTYWTGRYTYKCGCWGRMYYRKGRYCACYAGTTGAGGTARRYMAAGSST
51          RKTTTCTYCGGRKYACARCGCTMKYWRRMYYRTARCMRYCRTYCARAAWRRRTAGTKCGY
            **     *.  *.*  .*   .   * *****..*         . ..  **  .: ..

35          TCAATKYYMRYGYKKRKGYACTCTCRKAARKSTMGTARAKYYCYKKKCSWYYWRKMKWKK
51          KYWWCKYYMRYRCTKATACRYCYGAAGWRGGGGCKYRGTKCYYYGKKTSWYYWRKCGAGK
            .    ******  .* ..      .      .      :* * * ** *******    *

35          ARAWTCRRGKRTGTTTGTTSKGGYRACCRACATCYCCGKKKYRACTMKRYRKYYYTAMRG
51          MRTWCYAGRKRYRYYKCCAGGAKYRWYMGTTGGYYTYKTKKYRCGYAKAYRKYCTCRCGR
             *:*     **    .  :. . **    : .  *   .****.   * ****

35          SAYCRKTYRMRYGGKKWYRRWWAWSKYAKKWKKCCCYMYYWWMRKKGAGGCCMMYRRCKG
51          GRCMGGCYRMRCRRKKWYRRTTWWSKTGTKAGKTAYYMYTTTAAGKRRCATAMMYRRMTR
            .      ****   ******   *** ..*  * . ***      *   . .***** .

35          KKWKTYTYRRRTMYGKGCYKCTYAAWTTYKMTAMKWKYTATGKWTGYYYARGKAWKGYRM
51          KKWGCCWTRRGAMYRKCMYKMWYRTWYYYTCAGAKWKYYRYRKTATYYYCAKKCWKAYGM
            ***     ** :** *  **  * :*  *. :. ****    * : ***.  *.**.* *

35          ACGACGRTCKMTCAYSRAAAGTARYYYKGYWARYKMWKAYRGASATKCGWCCCKRRWCKY
51          WTAMYRGCYGAWYRYSRWCMACRRYCCGKYTRGTKMWKRYRKMSCCKMTTATMKAATTKY
              .           *** . .  **    *    **** **  *. *   .  *    **

35          KRYYKYWYRYKYCYCCYAGGRGTMSYGRKKRKRYCMMGAMMMYRWTYKKAKCGAKRCATY
51          TRYYKYWYGYKYMCMYYRRAGKCASYRRKKRKRYYMMKMMMCTRAGYKKRKYKGKRSGYC
```

```
                .******* ***    *  .    ** ******* **  **  *  *** *  .**..

35              AGKACMWRTRRTMACMACARRRRRKKYYCGAYCACCWCYRYYRRKKYAAWYWCTTRKARY
51              CRKTTCTRCRRCCMYMWTGARRRRRKKYCARMCMMMMWYCACYGAKGCRMWYTSGYGKRGY
                . *:   * **    *  . ******* .       *    *  *    ** .   *  *

35              CKTCWCGYYYRTYYMYYSMRGTMAGTWYKCYKYRACKYGCRYKWYYRWYMAKMWYYAAYY
51              YKATTTACCCRCTYMYCCARKWMRKYACKTYKYRGGKYKSGYKWYYATCCTGMTCCRWTC
                 *:   .   *  *** . *  *     * ****. ** . *****    : *

35              RYKRMMCWGCYMGRGATAYMRATTCASMAKKAYSGGKWTRRYKTYYRKMYRTYSWKMYWR
51              GYKGCCYTRYYMRARGAGTAAGAKMRGCCGKTTGARKWKRGTKWTCRTACRWYSWKCYWR
                 **       **   .:.   .:.  . . *: .. **.*  *   *.  * **** ***

35              MMCYYKTCYACTYYYKTGGTTCTCWSMGATKCYATRACAYAKATCCCACGGGYAWWTYRC
51              CMMCYKATYRAYYTCGKKAYYTATWCCAGAKYYGCGWTRYGGRCYTGRTAASCCWTKYAT
                 *  **: * . *   . .   : *. ..:* *.     *.        ... .* .*

35              KCRRTTATKAAKAYWWWGCKTCMCKTCKRRCKYKSKAWKKYWWGYKYSYKATGAKKRRTT
51              KYGGWYRYKWWTRYTWTKYKWYMTKKMKRAYKYKCGGWKTTATTYKYSCGCAAGKKRRAA
                *       *  . * *   *  * *. **  ***. .**.    ****  .:..****::

35              AAYAWSTCTGYAYGMKCWRYRMKMRRATCCTKTMKWKRMWYRYWYRTRYMATTSYMRYCK
51              GGYRWSCACKTWTKCTYARYRCTMGGTWYAWKCCGTGGAACGCWYGAACMGWCCTMRYMK
                ..* ** .       .  *** .*  :  . *           ** :  *.  . *** *

35              KTGGYTCGYYRRKRWRKGGKRYKKKKYRTMWMAMYTRGWKKATCYYWKAATYWWKGKRKK
51              KYRKTCMRTYRRKGWRKRSKGYKKKKYRWAAAWMCYRSTKKTGYYYWKGGCCAAGRKRKG
                *        **** *** .* *******     *  *. **:  ****..      ***

35              YRYYYWMYKRKRCTTWTKCRKATTRMTRYGCCGGCAMGTMAACYKGYYSYYKKRGRTGGK
51              TRYYYAMTKAKRYWKWCKYGKTCKGCGGCRYMTATGMRWMGRYYKAYTSYCKKAKRCKRK
                 **** * * **  .* *  *: .         . .*  *.  **.* ** **  *   *

35              YYYRCKYWSRRKCCRYMYKCYKCGAATCAMWGACTYYGYYGYCSMCYGGCCCATWTYYWK
51              TYYRAKCWSRRTGGRTCCGTYKYKRRKYMMTKRYYYYATTATTCMATRRSTTGKWCCTWG
                 ***.* ****.  *     **    .  *     **.  .  .*.   .  ..*   *

35              GAYGKAAATTKRYCAGCYTYKMGAAMTGRKRACTRAAATRRWRTKRGTYTTTKKYRMKAA
51              SRCRTTRTWWKATYGCMYWYGCKCCAYRRKRMGAATRWKRRTACTGRYYCCCGTCGMKWT
                .   .: :  *   .  * *   ..   ***  : :  .**   .   *    .  ** :

35              TAKACGTCTACTAKGGCCGGCARTTGTSATTRMAYRYKGRKKWYRCACCARYMCATYGKG
51              GTGWTACTYRTYGGAATTARTRRYCACSMYWRMTTRCTRATKWYRYCTATRTCYRKTRKA
                 :   .      . ..  .   *  . *   **: * .  .**** . .:*    .  *.

35              AKTGRRKCACRCTAWTCCAKCAAAGGWKYRMAAYYGCYKAYKYMRYKMYAMRRTWYSWKM
51              GKYARRKTTTRTAMTYMYRKTTGMRKTKYRMGTCCRYYKTCKCMGYTMYRARGCACSWKA
                .* .*** : * :      * :.    ****.:    **: * * *.**  *    ***

35              GCRMYCACKCGYTGRYKRYRSSKYRWCTRYTTRTTKGSAYTGYAAGGCGAGTCCCGGYGW
51              RYRMYACSKTKCCAGCGRYGSSKCAAACRYYGGYAKKGWTKKYWWACAACKWMYYRKYKT
                  ***...*    .   ** ***   . **    :* .  . *  . ...       *
```

```
35              RMKTCKKCYGCRACKSARCAMWCGCGRTGTYRMYTKGKWCRYTCTRGKAKGAMWYRATAC
51              ACKGYKKYCCGGGTGSRRYGMWTRTARYAAYRCTYTKKWYRYKMCRTGCKAWAATRWAWM
                  *  **      .  * * .**    .* .:**    . ** **.   *   .*.     * :

35              GGRMMRRAWTATCYTGWATGRKSTAYCAGAWMWAKWATTAGMATCCARYATGAGACCCCY
51              KRGCMRRWWYRYYYYKAWCTRTGGCTMRRRWMAGGTWYYMRATGTMGGTRKCMCTTSYYT
                    *** *     *      *.. .      ** .         :   .   .   : .

35              RWYKYRYTRRCRWRRKYMARGCGMYRYKKTCTRYKTGYMYRTTRWKKARRYYTGKKKGRC
51              RWYKYGYKAGYRWGAKYMRRSMRMYRYTKYTGGCTGCCATAWARWGKCRRYYYRKKTRRY
                ***** *.    **  *** *.  ****.*      .        :** *.****  **. *

35              RYCGKRGGCTYKKAGCKCGWKRSTRKKYMRKGKKCWKMYKGRYKSYMAGKRTTCTAKCAY
51              RCYKKGRKGGYKKCTTGMRWGRSCGKKCCAGKKKYAKMCTAGYKGCMRRGRCGAGCKGTY
                *   *     ***.     * **  **      **  ** .. **. *    *  . .* :*

35              KKYKYRCYYYWWYYTRMYWTAAACCRMYRYSTTTKGRYRYYKWRKMYYYYWWKYWTWYRW
51              KKYKTRYTCCAAYYWRCCAKCWMYYRMYRYSAWYKCRYRYYKAAKMYYYYWAGYWWWCAT
                **** *      ** *    ..    ******:  * ******  *******  ** *

35              CATGKKCMWRMGMGKYRWKAGYKKAYMMRMGTCYWYCYWKGMCRYGTTYKKMKTWAAAYT
51              TTGTKKTMAACKMTKYATKGTYTKGYMCGMKYTYTYGYWKKAYGYTCATTTAGWAGRGTY
                 :  ** *     * **  *. *.*.**  *    * * ***      *  : ..     . .

35              GKATCAAAGGTCKGYSCCRCARYGAARTTKKYWWSMWRGGGGTGGRKGKRKGYCSYYCRS
51              KKRYYRRWRAYYKKYGAYGYTRCKRGRCCKKTWWSCWRKRRRCRARKAGGKACMGCCMGG
                 *        .  * *..   :*   .*  ** *** **        .**.  *.  .    .

35              GGYMTCWKYRRRTWTKRCTWCKKMRKTGAMCCAKTGMRKGYMYGTATYRRCTACRRRKYA
51              TAYMKYAKYRRRYACGRYKWYGGAATYRGAYMRGKSMAKKCCCTAGKYRRTCGTGRRKYM
                 .**.  *****     * .*      .  .      ..* *      :..***  .  ****

35              GYTMGCCTCAMGRATAKRWRTTTTWCMKCCMYTAGYTGYCYRTCTTYAGAYRCRWSKWMR
51              KYYCATGCYGCRAGWMKRWRKKACWYMKGSCYYMAYKRYYYRWYKCYGSCYRAGWSKWMR
                 *   .    .   .   ****..: * ** . *   .*. * **   . *...**. ******

35              KKRWRYKCYTGWYYMGCMYKWRSAWYSTYMRRSMGGKACGTGKCGYSRAATKRTYYYACY
51              KGRWRTTYTCRWYYMRYCYTAGCMTTGYTAAACMKRKCTAAKGSRCSRWWWTGKCTCMTT
                * *** .    ****    *.  .   .     .*  *. .:  .   **    . .

35              TYGTCKGYATCGKKRMYYTATGRCCWYRYMKKAAAKTMRYRWAAAWGSKRYYYGYAAGGG
51              ATKAYKTYRGTKKKRMYTGGCRRYYWYRYMKKWMMKGMRYRAMCGWTSKRYYCRCTGTAA
                :  : * *    *****  .  *  *******    * ****   ..* *****    :. ..

35              KCRTAGCYYTCRCGRCASKKWYWKKCTGKRRATYKKRWATGRMAAYTACYKRRKGGCMRM
51              GMGCWCTYYYMRTRGYGSKKWTWKKYCTKRRRAYKKATRYRACGGCCMTYKRGKAAMMGC
                       **  *    .**** ***   *** :***        ..     *** *.. *

35              YTMYYYKYGAGTMKRYYYKAACGATKAAKRMYARYYKTKKKKMYGGMGAGKKWYYWRRSK
51              CWMYYYKCSRRWMKACCCGRRTACKGTGGAACWRYYKWTTKGCCKKCKRKTKAYCAAGGK
                  ***** .   **         ... :.     **** ..*            .* *     .*
```

```
35          TKKGTYCKCMMWWMRYTAWGGGKRYRCTATGYCATSYRYTCAKCSYSYGRCACAAASKKW
51          YKTCAYTKMAATAMRYYRAATTKACGSYMYSTMTWGYGYYYMGYGTSCAAYRYMTTGKKW
             *. :* *     ***   .  *   .   .  : .* *     . * .     ::.***

35          KGGTRRYYKYAACKYSAYTAAGRYYRTRKYRMRYKKMYCAAKGYRTAARAATGGCYCYMM
51          GSKWAATYKCGCTKCGGYYRRKATTACRKYRMRYKKCCTGCGACGACGRRRARTACATCC
             .     ** .. * ..*         *********   .. .  :..*  :  . .

35          KYGKCGTGKKCTCYWCKMKKKYKRRRYGTGTTARKAGGCYKYRARTMTGRYSTGCTGACC
51          TCRKTAATKGTAAYWMKAKGKYGRAGTSWRYWRGKGAAYCGCGGRKCGRGCCWRMYKRYY
            .  * .: *  :.** * * ** *   .      *...     .*.     .

35          RRKWWCCAYWKRKTCTRTMRKRARMARKRTAGGGYMRKTKYYTRTKTMWRSYWYAGKAAY
51          RRKWTTYRCAKRKYGYACARKARRACGKRYWRRKYAGGAKYTCRKGYMWRSYWYWKKTWC
            ****      ***      **  * . **     *   :**  *.  *******  *:

35          CGKWRYGYYWKKATRKSRCKKWKCTKTATMKCRKCGKKWTYRRTYRWKGRWGTCKYGTGR
51          ATKWRYAYYAKKGKRKSAYTKWKYYKKMKMKMRKMKKTACTRRCTRWKARWSGYGCRCKA
            . ****.** **..***  .***  *. .** **  *.   **  ***.**.

35          CMATTTKYYATRCKWRRYKGTCATCYCAARKKCGRRKTRAAGTCYYAKGTWAGGMGCKCT
51          YCWKYAGCYTYRMKWRGCKRKTGCYCTRRGKKARGGGWGGCAWYCTRKRKWCARCKSTYY
               . :  *: * ***  * . .       **.      ...     * .*..   ..

35          AYYRTGACTAGYAKAGYCTYMKKTRKWRGRCGRGCRMRMYRYTCCCKMRMYWKCRWTGWT
51          RYYGYRWMGTKYRKRTTGATCKKCGKTGAAYKARTGMACTATCTATKARCCTKARWKRAY
             **      : * *    :  **  *  .       *       . * *   *.**.

35          RGCGCTYYTASGGYAYCMWTMRRTKTGTATKCRRTTYTAYGRGTTGTGRYKKTWRTRYGC
51          GRMRYWYYGGSCRTCCMCAYMRRCKCACGKKTGRYGYCWTAGAYYRYRACGGYWRCRCKY
                  ** .*   .     *** * . ..*  *  *   . .          ** *

35          AAWCCCGMMTKKRTWMWTYKYKTTCGRRRWATGKTKWSTYYRYATGRMGTKGKKMTKAAA
51          GCAAYYKMACKGRYTCAWYKCKCATAGGGWWKAKYTAGAYTGYRWKRMRGKAKKCYKGTT
            ..  .   *  * *     ** * : .   * ..* . .:*  *   **  *.**  *.::

35          RCGGRCTRGCTATKMRYTKCKKKRWGYGAWSRRSKCYRRTYAGGTYYRRYRGGACKKTAM
51          RMRRRTCARACGAKCGYWKGKKKATTTRWASGGGKACAAWYWKAYCYRRYRKRRAGGGCA
            *   *    . .:*  * * ***       *  .*.    *  .  *****   .   .

35          GTTTTYCCWCYKKRYRGGYKYCRTGATMTKACGRRCMRYYCKRKYKWYRRWKYARKTCTT
51          TYYCWYAMWMYKKRYRRRTKCTRWCTCCYKRMKRAMMRYYTTRTYKTCGGTGYGRKYYAA
                 *. * ******   *  *  :   *   *  **** .*.**      *.**  ::

35          KWYGYYKKTGGKYYWYYAMCCCGYRTKGRGKRCKRRRMCMRYRMSYRKKRGKAMYRYKTA
51          KTYAYCKKCARKYYWCYCCATTKYRKKKASGGYKRRGMMMRYACSYRKTGRKRMCRYTWM
            * *.* ** . **** *. .   **.*  .   *** * ***  ****.  * * **.

35          TRCAMWWYMWGTAAKRMKKKYYKYKYKKYGCKKMRYYRYCYMRTWTTKWRTYTTGAYRKR
51          YGMRCATCATCGMMKRMKKKYYKTKCKKYRYKKMRYYRYTCCGYWCCKTGAYWWRGCGKR
                          ********* * ***  ********     *  *  :*   .  **

35          KATCKCKCGGTAYRGYMGTKKGATKCRCTWRCKKYACRKATMGAWKKKMRAYRMWAYWMK
```

```
51      KCGYKYKYRRWRCGTCCCCKKTGGKTAYGWRYGKYMMAKGYMRWAKKGAGRYACTGYWCK
        *.  * *            ** . *    **  **   *. *   **    *   .** *

35      KRSSCACCTATYRGYYMKCRRRRKSCYTTTWYCMAKKKTYMYKYAAAWYGTKCAGATTAY
51      KRSSYMMYKGGYGRYCMKYAGAAGSYCCCAWYMMRKTKGYCCGYGGCWYRYKSWRRYWGT
        ****    .. *  * **      *    :** * *.* *   *...**  *.     .

35      TGTGAYGGACYYARYYKWRWTKTKRRWGTGKWARGCAKSYGCKRRRYRYKCCATAGCKCY
51      YAGAMTRRCYCCRGCCKWAWGGKKGGTACCKWRGAATGGTTTKRRGCGCKGAWCWKYGMY
         . .    .       ** *  .*   .  **  ..: .   ***    * .       *

35      KRKMWTYRMCYRKYRACYGWYAWTAYRMGCKGRKAYASKGCCWGGGGRTYTCGACWRKKY
51      KRKMWKTGMYYRKYRRYCRWCRWYMCRMCTGRAKGCWCKASMARKRRAKYCYRRYWRKGY
        *****.  * *****    *  *   **     *.  .*..       .*     *** *

35      TYARKGGCKRYGGWKRMMYKCAKCKKCKKAKMKCRWMWTCTYKKGTGYKTRMRKMGACTC
51      CYGGKTATGACTKAKGMMTTSTKYKGYKKGKMKYRAMWWYWYKGAGCTKWRMAGCRWAYM
         *. * .       * ** ..:* *  **.*** * **   ** .   * **     .

35      TAGTTTTYCAGYKKATKYCMYYYYCYGCCGAGCKWYYYRYYKWYTKCYKGWTGKMYYCKTY
51      YRRCCAYYYRRYKKGKTYYMYYYYTCATTATATKATTRYYKWYCGYTTRTYSKAYYMTCC
             : *   ***...* *****  .  .:. *   ******    .   .* ** .

35      YYKTKCTKYYWWACYGCTCRCKRSMRKCWGGTTWTCRCTRCAGMAKYKMGTGRRCCCYKM
51      CTTCTMCKYCAACTYRYAARTTAGAGKYATRCCAWYRYKRMWAMTKCGCAAARRYYMTKM
          . .  **   . *  :.* . .  *         * .*  .*:*   .:.**    **

35      SAKTYGRRWRAWYYACAYTCCCGTSYRRKTSKGWWYKGKMYWMRMKRSMRSRRCKMYKYR
51      GRTWTRRRWRWTCCGTTYGATATCSYGGKYCKAWTYKAKCYWMGCKRSMRSRRAKMCKYA
        . .   ****    . :* . .  **  * .*.* **.* ***  ********.** **

35      MRRYRRYYCYCGTCRYAKAGCCMYWACTRYKYRYYRTTAAKAGWYCRRCKMKRTMCYKKR
51      AGATRGCTMYYACTACCKRRTTMYTTYWRYKCACYRCCTTKMSACYRRMTCKRYMMYKGA
            *    * .    .*    ** :  ***   **  ::* .   ** . ** * **

35      KAKYYKCKYGYMMCRYWCWAACRWKGCGCAGARKKMRYYWRGMMRWMGTAGCACGCCGTK
51      KMKTTKSGYRYMMYGYAATMWYATKRYKTCAGRKTMRYYTARAAGWMRWWRYRTAGMRKK
        * *  *. * ***  * .      *    ...**.****      **       .   .*

35      CKAKACKRMWKKTYATTMKK
51      TKRKRYGGCTKKWTGKYMKK
         * *      **  .. ***
```

The pairwise alignments of two accessions 38 and 42

```
38              KYYYKYSRTTGCRMYMYSYRAMSRRGTRWGGRKWMRRYMYRMWYRWRYYAMWRRKCTGTA
42              KCYTKYGRCAATGMYMTSYRWMCAATYRWRRAGWMRRYMYGMWTRTAYYCCWRRKTGAWG
                * * **.* :.  *** *** *.    **    ******* ** *  **. ****  . .

38              AYGWWGYWYRKTRRWKWYKWCGRYMSRMMYRWRKTGYMARWTRATYRRAKKYYYKYYWWR
42              RCRWARYWYGKARRWKWYKWARGCCSGMMCATGKYKYMWRWWGWWYRRGKKYYYKYYWWR
                   *  *** *:********.    * **    *  ** **    ***.***********

38              SRWYAMMGRRWTTGCTMYYRMYAGTGYAAWYMKRMWYARYYSYTCAKKCTCCTGMRYKGR
42              SRWYMMMKRAAYAATCMYYRMYWTCATRWWYMGACWCWGYYSYAATGKYCMYCACATKRG
                **** ** *   :.  ******   .   ***   *   ****:.: *     .   *

38              WTAYRKTGCWRMWRWWMRSSWCYKWYYRRGCTAYYKTRSKWYGMCYRYYTRSRMKMYKTM
42              WWMTGKAAAWGMWRWTCASSWTTTWYYRRATWMYYKGASKWYRMTYGYYGGCRMTCYKCM
                *    *:..* ****   ***  .*****.   ***  **** * * **  .**. ** *

38              AATKGTCRGGYRYWYYWKGGTCWYCYRAKMKCGRKYWTRRKGYMKMYRRRYRYRRRRTTC
42              MWKTRKMAKRYRTATCWKRKYTWYMYRTKMKMARTTACRRKRYMKCTGARCAYAGGAWWY
                  .. .    **    **    ** **:*** .*.   *** ***    *  *

38              TKAYMKCGTYAWAATGARYYMKTTGTGRKYMCTGYWYMYARWRRTCCGRRSTRMGCKGAT
42              CKGYMKMKYYWTMRCATGCCMKYCKWRRKYATGAYACMTRGWRAWYYRRGCCRMATKRRY
                 *.***   *     .:   **     ***   .*  *   **     * . **. *

38              YRKWMYGTYMGSYRRYGKRRSKGRTCCASMYWGTGMGTAYYMRYCRYYRSKCAARRKKSR
42              CRGWMTKYYMACYRRCRGGRSKAGYYYMSACTRYRMTAGTYMRYTAYYRCTSRTARTKSR
                 * **   **..***    ***.     *      * :. ****  ***... : *.***

38              RKRGMKYRAWRYRYAKYRWYYSRWWMKYMRCTCYARCTTKMRRARGTKRATTGWMCGCGT
42              RKRRMGYRWAACATMGTATYYSRWWATYMGYWTCGRTAYKMRGRGRYGGWKWKWMYRTTG
                *** * **           ****** .**     .* : ***        .  **

38              TKAKCGYWTYYYTCYYYAGGAYRTAAYKATAGTMMRAYYCMKWKWKRRYMWYACWRTCAG
42              CKMTYRTWKYYYGYCYYRAAGCGARWCKTKRSAMMACCCYMKWKTKRRCAATWMTGKACK
                 * .   *.***   ** ...  :   *:. .:** .   **** ***        ...

38              GCYYKRYRYSTGTATTRRMRGSYSGKGAMYRAYCCTYTTSCRKYMRATGGSYWMYMKKTG
42              KSCTKGCRYSGRYWCYRRMRRCYSSKACMYGRCTTATCACAAKYMGWGAASYWMCCGKAA
                 .  *  ***      **** .**.*..**     :  :.. ***   ..****   *:.

38              CRKKKRRKKKYTCTTSRKRMRRWKYARRGYWYMKAAGYGKAGRCKCTGAKTKYYYKMYRY
42              AAKKKRRKGKCWTCCSRKAMRRWKYMRRRYAYMKRRRYKKGRAYKGCAGKYKTYYKMCAY
                . ****** *     *** ****** ** * ***   * *.   *   ..* * ****  *

38              GGCSGYKKKKAAYGTKTMKTKKKCGCAGKGARTTTRRRSKTYKYKRKYRARYKKRGAATC
42              KRSGRYKKKKMRCRYKKCKKKKKSRYRRKRMGWCCAGASKYYKYKRTYRRRYKKGRMCWY
                  .. *****     *. *.***.    *         ** *****.** ****   .

38              AYACCAKGTRYKRKKRKAKGTKCKGTYRTSKKRKCCCAGTAKYTCCCRYMTYRCTCARKM
42              GYCTYGTRKRYKRKKRKRKRYGMKCGCRYGKGGKYMYGAWWKYCYSSGCMWYRYYYWRGC
```

```
                  .*.  .. .******** *    *   *.*   *   ..  **  ..  * **    *

38                AGRKAYKYKKKYRKKCARTRTTATAGTTYKKKMYCGTRKACGMTYYAYKYGTWAMYYKAC
42                WRRGRYGCKKTYRKKYGGWAGAMKRAKWYKKKAYYCKGKTYCAKYYRYKYKGTCAYCKTT
                    *  *  **.**** .    : . .. **** *  . *:   .** ***   . * *:

38                CSTCGRYRYSWTRAKWRTSWTAYRRATAAYWRKKWRTTCRGTCRKTTYKKTGKRTMYCRM
42                GGCTAAYRYGTKRCKWRYSAWMYGGGARGYWRKKWRYKMRRGGATYYYKGYRKRWMCTRM
                   .  .  ***. .*.*** *   *  .: .******* . *    .  **  **  *  **

38                RRWSYKKRMYKTKWTWYGWACCRGGAKTAYRMKYWWARKKKYMCAATKAKYRCATCYKAM
42                RRWSYKGRMYKYKTKWYKWRYYGKAGKYWTAMKYTWRRGKKYMYWRKKKRKYRMMYYYKCM
                  ****** **** * .** *     ..*    *** * * ****   .* ***    **.*

38                KRGSCRKYRRWKKCGTACTCAYCMRWCRGRGAKYWAKCGGGKMRCAACTRGWRAKTRTCK
42                KASGYRKYRRWKKTRYGYWYRYSMRWSRRARMTYWGKSTAKKMRYGTYWGRAGWGCAKAT
                  * .. ********   .    *.***.*    .**.*. . *** .:          ...

38                CRKMARMRACWRSWYYTACRWTGTYKTGRRRTCYKCYTKRKYYARRKKMWRMYMKACCCG
42                YRGMWRMRRYWRGWYYWMYAAWRYYKWKRRRWGCGTCGKAKYYGAGKKCAGMCMTRTTAA
                   * * ***  **.***        **   ***         * ***.  **    * *.   ..

38                TTACCACYCGGCCKCTYKRGTACAGGACCGRYYMGRWMSACRSGCCAAGGWGRMYTGRKR
42                CCCATWAYMKATYKACCGGRYWYTRRWYYRRYCCRRTCSMMRSTTTRGTRWKRACKAGGR
                    ..  .*  .  *.        :      **   *  *  **    .  * *  ..  *

38                WKMSCKCCRRYRAAKCYSYAMKARRKGMRTMYCTCAWTGRRRMRCMGKACKTKGYCGYAT
42                AGCSYTYAAGYRRTGTTSYWAGWRAKTCGCACMGMMTWARRRCRYARKRYKKKACYRCRW
                     * . .  ** :   **    * *            .*** *   *  *.*.

38                CYKKMMGCTYGWWYRYTKGMGCGGCKKRTTCAKMGTCCRRCRYARCGAKRYYAGCCTCMA
42                SYKGMCATAYATWYACYKRMKSRRMKKRWKTWKAKYMYAGTGCCRYRRKGYCTAYMCMMR
                  .** * . :*. **   * * .   *** .  *          .*   * * :.    *

38                GTATKRSGAWKRKRKCGKSKWYGTKAKGKKTKYTWYTKTYYKYWKKGTGGACRWMKWKTR
42                RWCKGGGACWTGKRKYRKCKWTRWKMGATKKKYCWYCTKYTKYWKKRCRRWYRWMGAGCG
                    ..  ...*. ***  *.**   *  ..*.** ** ..* *****      ***

38                RCACRCWTYRCRCCTCTSWYKYWWYGTAGAACCTGMCCRMGYCCTWCYKRKAYCRKKKKK
42                RMMMGYWWYRYAMYYYCSTYKYWWCKKTKCGTMACMMYRMRCTTATGCKRKCYYGKTGKK
                  *     * **       * *****  .: ..  : *  **    :   ***.* *.  **

38                RWKYCYKYMACCTMTCYGRSWGAGGGCTKGAYYYKRKRRYCTKWYGCGAGCRKGRKMCYA
42                RWGTYYKTMGMTYMWYCSGSWRRKRRYYKKTCTYKRKRGCSYKWYRYRRRYGKTRKCYTR
                  **   ** *.   *   . **       * :  *****  . ***       * **

38                ARMCKGTGRYWYTRCKKYRRKGTRYTKRTKAGGKAKYYCRWRTAYKAYAYRCGRYYYRGR
42                MAMMKRWKRTWYKRTKGYRRKAYRYWKAWGWRRKRKYYGGTRCTYKRYRTGYARYYYRRG
                    * *   * **.* * ****. ** *      * ***   * :** *    .*****

38                KRYKRYYRKGYKCKGAACKAYAACMGRYAACRCRWAAGGCTKTRRAYRMRYYKKCCCGAK
42                KRYKRYYRKKYKYKRMGYKWYRRTMSRYRWYRMRWWRKRMYGCGARYRMRYYKKTTMRRG
                  ********* ** *  . * *   *.**   * **           *******
```

```
38    TCKRAWTGKSGKKRRKYKCGYRRYYYKYYMMYKKAWYAKRYAGGSTWRRAKKSKRAWYKY
42    KYKRWWKRKSRKKRRKYGYRYRRYYYGYYCCTKKRWYRGAYRKASWTGGMGKSKAWACTT
      . ** *. ** ******   ****** **   ** **   *  .*      ***    .

38    YMKCTKYTTTGAGCKAKKATTKTATTRACKKMWATAGYRKMYKKGGACKTCGAKMWATRC
42    YCKYCKYYGCCRAYKRKKRYYKYCYKGRYKGCWTACKYRGAYTKACGTKCTAWKMWRWRY
      * *  **     . * **   * . .   *  *::. **  *.*. . *  . ***  *

38    GTKAKRYMCATAAWCKTAYKYRKMYWWYAGGTYRGACYMMKGRKCMKYYKRRTCYGAKKS
42    KCKMGGCATGCGGTYKGRYKYRKMYWWYRAAYYRTRYCCCGRGKMMGYYKRRKMCATKKG
        *      . ..  *  ********** .. **         * * *****.  .:**.

38    WYRCTMCYYRKYCTTTTKGCTTWWRRKRKMTTCCTRKRAAYYARYYYTAAAMAGTYKTAA
42    AYRMWCYYYRKCMYCYGGATCCWAARKRKMGGTYWRKRWRCYRGCCCWGTGAWRWYKGRR
       **    ****       .   *  *****     ***   *      .:.    **

38    RRCCTAYRYRARGAMCCAKTGYAAGARGYKSCMMRWRAKYTRGCYGCRGWMCGRTTKRKY
42    GRYTYGYGTAMAKGCATTKATYRMRGARYKSTMMRWRGKCGAAYCKMGKTMMARYKKAKY
       *   .*      . . :*: *   .  *** *****.*   .       * .* .* **

38    RYKRMTAWKYYRTGAAATTGMGCRTTGAKRKTCRKGARTKWMKASGCCSTYTGAGTSRAC
42    RCKGCARTKYYRYRMRCCAKMRYRWWKGTGKCTAGRRRYKWATGGRMMGKCARTCYGGCY
      * *  :  ****    . : *  *   .. *      * ** ...   .. : :  . .

38    CTTTYACGKSTACYRMAATMKMRMRKRTRYYCCRCTWYRGRYGWKTTTSYGTTKKKKTAW
42    SYGCTMTRKCWWYCACCTAMKCGCRKRKRYYYARAAWYRRACRWKKYWCYRCGGKKKCGW
      .       *.      .::**   ***.*** .*.:***    **.  .*    *** .*

38    CYMWTCCAAGYKMCGATCGMTRTYCYYTCYRTGGTTTCMGGTACGWGGTTYRTRRRKRKT
42    YYATYYYGRRCKMYKMWYRCAACTMYYYYYGCRACCKYMRRCWTAATAYKYRKRRRKRKA
       *     .   **       :    **  *   .  . *     .  . .**.******:

38    TKKACTCKGCATRRAGCAYKYKYKTRCAKGGKAKRKTYARCWCWSAGTAKSYTCAKKTCR
42    AKKWYCTKSYWWRRCKYWYKYKTGCGTRKTAGWGAKWTGGYTMWCGKCGGSYYYTKKYYG
      :**    *.   **.   ****      * .    *  .    *..  . **  :**

38    CWAKGTACKKYWKWCCCAKCAAGTCTCYYGAATGACAGKYRYYSRKMCKKMAMWYKGRGY
42    YAGGTCGTKKYTGAYTGCKAGRAGYYYCCKWRGAGTMRGYRTCGGGCMKKMRMTYKCARC
        .   . ***      .*.. .          ..    **  .    *** * **

38    KMARKCCTACWMTTGTATGAYGGCYATTCTTATMYYRCGKKTKMWWWTKRGMGKRKYGRW
42    KCRGTTTGTYTMYWKAWYRMYAAMYGWWTKYTACTCGAKKKWKMWWWWKRRMKKRKCRRT
      *   .   :  *   :    *.. *.   . ::    . ** ***** ** * ***  *

38    ACGYYGYYRMKCAARTYKTCCAGAYYATCTCYYKWYKWMMACCTRCTKKTYGRRCAAATR
42    RMKYTRYYRCGYRRRYYKCTTRSMYYRYYKYYYKWYKWMMWMYKGMGKKCYRGAYMWMYR
         *  ***     * **    . **   . *********   .   ** *        *

38    KCATGTRYTGKAAYCGGKGYKGSKCCRKRYCTYKAGAWAWWRYCGGMMSCACCGYKAKRW
42    GMRYKKRYYRKRRYYRRKRTKKGKTYRTGCYGTKMRWWRWWRYSRRMMSMRMYRYKGKRT
           .**  *  *   *  * .*  *.     *   * ****.  ***     **.**
```

```
38    YYKRWRYGYKCTACRGRRARMKMRSTYGYRRCACWKYGYYKGSMGKKRCMYRRRYGGRYR
42    TYGRWRYRYKYCCMGARAGGMGCRSAYRCGAYRYTKYRTYGRSMRTKRTATARGTARGYR
       * **** **  .  .* . *  **:*        **  *  ** .**    *  .  **

38    GRYWKKYYYATRARAWRMGAAYGTTKSRAMMYKACKGGGGRAKKTRYTRCARRMMMKAYW
42    ARYWKKYTCMCRCRMWRCKGTTRWWKGARMMYKCMTKKKRRRKKCRCCGYMRAAMCKCTW
      .******    *.* **  .:    *.  ****. .    * ** *     *  * *. *

38    RYKKKCCTCMKYKYGYTCGMKKKSYCCWMTCRCTRGCKMRRAATYYGTYWARCAGCRKTA
42    GTKGKTTCACKYKYKTYMRMKTTGTATAMCTAYKRRSKMRRWWYYYRKCTWGYMRYRKYR
        * *   . ****     **... .  *    .* .****   ** .        **

38    GTGATARGGGCAKTGTYKGGKCCYKYGTKTSGTTTWCYTTCGGYKKTTACKMSKAWWSTT
42    AYKRKRRKKRTMKYSKYKKSKYMTKYACKKSKKKWTYTGYTCRYTKYWWMKMSTMWWSYK
      .   . *     * ..** .*   **. *.* ..         *.*    ***. *** .

38    SYRCGYCMYKATAATAATYMRWTWYTGAATTYCWYWWTTATATGAGGMTAMKTTTTGCGG
42    SYRYRYYMYKRWRWGCCYCARAATTGAGRYACATTTWWGCCCKRRRKMCGMGCYYYKYRR
      ***  * ***     ..   * :   ..  : .   *  . ..    * .*

38    CATTKGGARYRYTGACCKKKMYWGKTTMKGCMKMCRRYRYGGTRTAKGGYTAGYKRKACR
42    MTACGKRRGCRCKRMYMGKKCCTRKYWMKATCGCYRRYRTRTWRAWKAAYWRRCKRKRSR
       ::       * .     **    *  **.     ****    *: *..*    *** .*

38    AGTGYGAAMKKCGGATCAYRSRKWYATACGKTGCYRTRRAKMCTWRYYCMCAATGCYWGA
42    MKGRYKRRMKKYRRGWMWYACRKTCTYWMKKAAYCGWGRRKMACWRYYTCYRRKTYYWKW
          *   ***   .   * .**  :    *:.     * **. ****     .  **

38    TCCCYKTRGCYYGKKYCKTYGKTGGCTARTKKMRRYKWKKCTCTGGAAMCCGACTTKKAA
42    WYMYYKGRRMCYRKKYMKYYKKCARYYTRYKKMRGYGAKKTCTYSSRRMATATAACGKGC
          ** *   * *** * * * .   :* **** *  **    ..  *. .:.:  *..

38    KCGMACGRGTTATMKTKCTGTYYYYRKTAYSKRAGGWTKKTTCRTSYWYYYYGYYSRRYK
42    KAKAWYARRKCRCAKKKMYAYYYYCGTYRTGGRGRRTAKKWCMAASYWYYYYRYYSRRTT
      *.    .* .    *.*  . ***  .   . *.   :**    :******* ***** .

38    KYRRAMYTARWYGRWKKAYMGGYKCTCKWRMTWKKRTCWCGTRMCCGAAAKYKWCYCTKC
42    KTGGMMCKGATTKGTKKGCAARCKTWYTTRCYWKKRYYTMAYRAMYATMWGYKTTTTCKY
      *    * ..      **.  .  *   . *  ****    . *   .:   **     *

38    YKWKGYKKGGMTTAYRKCMKKRWRMKYYMYCKYKTKRCTTTKGYTTTTKRRAGCAWCYYG
42    YKWKATGKKKMYYRYRGMMGKGAACGYTMCYKCGYKRYKKYKKYAAAAKRRTTYRTMTCK
      ****.  *  *   **  * *     * *  *   ** .. * *::::***:

38    KTTASKKARWAGTTMRKMKTWTKGCCTGTTKCGTYCKTCRYYKAKAYAGKTCAAGKATTT
42    GKYRSKKWAACAGGCRKMKKWCKKATCAYYKYKCTYKYYGCYKRKCCCAKYSMGAKCAYC
       .  ***   ..   ****.* * .  .  *     *    ** *. ..* . ..*.:

38    RYYACCTATYWYCCGKRYYRRYAYYTTCGTRWTGMWKMSKKCRRWAWWKRYYCTKGYCRR
42    RYCGAYYWWYWYYTKKRYYRRYGCCCYYKYRAAACWKMSKKTRRWRWTKGCCAGKRYSRR
      ** ..    ***   *******.       * :. ****** *** * *   . * *.**

38    GGGMCAATRKAARKMMKTKACAAKRCYKCCCYMRMKKRGAYTKRYCAAKMKARWKWYWRW
```

```
42          RRRMTGMWAGRWGKCMKAKTYGGKRYYKYYYYMACKKRAMYWKRYMMRKMGRRAGTYTAW
               * .       * **:*: ..** **   **  ***. * ***   **  *   *  *

38          KRRKGAKATTTGGWYMCKKAAAAATKMATSYKGCKACKTMAWAYWRYYYKSRYTGKGCTA
42          KARKTRKGGKYTRACMYKKMWWRRKKMMKSTKRTKRSKYMRWWCWGYYYKGACYRKRSYW
            * **  *. .     * **     .** .* *  * .* * *  * ****.    * .

38          AGKKGTMYKYGAYTGCWMMYGYYKGTCWARCGTCAGTCCSMYRGGKRKYAMGTTMTARCC
42          WRKKKYCTKYRMYYRYWMMYRYYGRWYWWRYRYYWRKYMSATGSAKRKYGMKAAMKRRYY
              **    **  *   **** **    * *      .  *   ..****.* ::*. *

38          ACCARTGYYRMYSCACCARTAKKKAWCRKYRYRTTYRKGTYKRKSYRARKKKARKCRYKG
42          CTTTRYTTCRMYCMRYSRRKRKKKCWYAKCRCRYYYAGKKYKRKSCGRRGKKRRKTGCGT
            .  :*    ***.   . *. ***.*  * * *  *   .*****   * ** **

38          CYWRCRARYRRRCYYCRYKKSYKKAKYKWGARKGCTYRYTYCKAAWYTMRYKTYCMWMRT
42          ACWRYRWRYGRAYCYMRYKKGYGKWGCKAKRRKKSKYRYWYMKMGTYAARCKWYYMWMRW
            . ** * ** *   * ****.* *   *   ** ..*** * * . *: * * * ****

38          KMGTGCYRATKTYRYWKRTGYGGMKRCATGRWRRKCAAKTTGTGYTCACYYKKCARSTTT
42          KCRYATCACCTGCGCAGAYKYRRMKRMRKRRWRRKYWWGAWAAKYGMGMCYKKYMGSGAG
            *   .   . .         *  ***  . *****    : .: *  .  ***   * :

38          GAYTARYMTRKKSKKWKRRYAATTYWKRTTGKKRWKMCTGGMMWACTCKYMKKKAMRKYC
42          ACTYMRYMYRKKSKKWKGGTRMYYYWKRYWRGKATTMMWRSACTTYAMKYMKKKCARKYA
            ..   *** ********       ****    *  .*   .   : : ******. ***.

38          ACKYYMCKKKYKWKTACKKCATGGCMYAWKYRKWRKYCRWTSYGYYTKKAAAGWRWKGAW
42          GYKYYMMKKKCKWGYRSKKTTCTATAYRTKYGKWGKYTRWKSCKCTKKKRRWRTRWGRTT
            . **** *** **   .** :  .  *  ** ** ** **.*    .**     **  :

38          CGRRGATARMYYKYYKAYGAGKYGGYAMYYMTKRKTTKATTCGYRYYWYKSYKTSCTKRT
42          YRRRRWYRRMYYKTCKMCTGRTCRRYMMYYAKKRKGGKMYYYRYRYYWCKSYTYGYYKGK
              **    *****  *   . .   * *** .***  *     ***** ***. .  * .

38          MGCARTMYYRAAACSYTTTYKGTTKYYYMYWTACCACRKGKCTCRACMRTYRTYKKKGKA
42          CKYRRYCTTAMTGTSCYYYYTSWAKYYYMYWKWYYMYAGRKTATRTYMRKYGWTKKKKGW
                *      :. *    *.. :*******.        * : *: **.*   ***

38          ARRKGKTAKTWKKGRRKKKYTYTTKMRSWAKCRRKKRWAGARRRYYRAGGRYRWRCTTYK
42          RRRKSKAGKGWTKRRRKKKCWYYWKCASWWTYRRKKAWWRTGGGTTGRRSRYRARYWYYK
             ***.*:.* *.* *****  *  *  ** . **** *  :        .*** *   **

38          YTCCTYKGYYYCYWGKYKRYYKATTARRKKKKKMATGGYWRWYWACCCAAKRYKYMACKY
42          YYYAGTGSYCCTYWAKCGAYYKRYWMRRKKGGGMGATKYWRTTWRTMMCGKGYKYMMMKT
            *  .   .*   **.*   ***    ****   *.:  ***  *    ..* ****  *

38          KRKTKYAYRYTKAKMYAYAGAKRRYCRAYYYCMYATKKTRCKCTAAGGKGMMRRRCAGTT
42          KAGCKYGYRTKKGKCCGYWKRKRRCYGRYYCMMYGWKKWGYGYYGGTATAMCARGYGSKC
            *   **.** .*.*  .*   ***    **  **. **      .. ...*  *  ...

38          YYCGARKRRCGTCKSYMAMAYSAGKYYYYAGGKKKACCCYWTGGGKKYYYTTCMKRKGTT
42          CCMKMRKGGATGAKCCMTMWYSWAGYYYYMRRGKGRYMYCWYKRKKKTYCYKMMKGKKYY
```

```
                        **  .  .*. *:* ** . ****    *      *    ** *  . ** *

38                 RYRRRGWMAYGKRKYYCTWSWTRARYYAAWRTYRSSRMCRYKKMRYWYMKKYTTWYRSRW
42                 RYRRRTWMGCTKRKYYYYTSWGRRRTCMWTGAYRSSAMMRYKKMRYWYMKGCYWTYGGRW
                   ***** **.  *****   ** * *      :**** * ***********     * .**

38                 YYKMRKKCKKCACTSTGRWCTTCKYKGKYYMMYKTGKCRCRYTRTRCRWYKMGGGAAASR
42                 YYKCRKKMKKTGTWCGRRTTCYMTYKRKCTMMYTYKKTGTRYWRKRYGAYKMACATMCGA
                   *** *** ** .  .  *     .** *  ***.  *   ** *.*   ***. .: ..

38                 MKYYKKRRGYMRTTYYRKGKRRGYTTGRYYATGGWCKGYRACWKRKRSAARGATTMGGCK
42                 MKTTKKRARYMGGCYYAKKKRRRYYCRRYYTCKRWYTACGTYWKRGRSMGARWYYMRATK
                   **  ***  **   ** * *** *   ***:   * ..  : *** ** .     * . *

38                 RKKARTRMCKTKSACCGYYYATWTKAGGCKTKTAAGCKKTATCRWRKWYAKRGAKKKYKY
42                 RKKWRYRMMGCGCWYMKYCCWYTKKWKRMKYTWMGATKKYRYMRWGKTTGKGRRGKKYKY
                   *** * **    .    *     .*    * .  .. **    ** *  .*    *****

38                 RY
42                 RY
                   **
```

The pairwise alignments of two accessions 38 and 43

```
38          YCCGRWRRTCCYAACYKAGYWRYARKWWWYTGKWMRTRCCKKKWRYRRRRRKYYAYRKKW
43          CYYRRWRRCYMTTCAYKMRYWRYRGKAAWYGTGWMRCRYYKGKAACAGRRRKCCMTATKA
                ****    :..**  ****  *  **   *** *  * *     ****     .*

38          KYYYTRRTYMMMWRCTYRWRMYRMKWTRMWACCTKYKRAYYRYRTATTSRWTRKWKGKTR
43          KYYYYAGGCCCMWGAYYRWRAYAMKTYRAWWTTYGTTRTYYRYRAGCAGGWWRKTGKKKG
            ****       ** . **** * **  * *      .*:*****:. :. * **   *.

38          YCYYRRCKGWAYYAWYATWKRYKCTAYYYYYWYKRKRYSCSTRYRSMMRWYRYGYMKTKC
43          CAYYRRSKRTWCCWTYRAWKRYGYCRYCYYYWYKRGRCCYCWRCGGCCAWTGYRYMKYGA
             .****.*       * :****    * ******* * . . *  .   *  * ***  .

38          GKSRWWYARYRMTAGGYCYMMYAMWKKMYCWWMYWWSMRKKGCGRKRYRWCRYWKAKRYY
43          TKCGTTYRAYRCYRRSCTCCCYRMWKTCYMWAAYTTCCRGKCTRGKRYRWAAYWKGGGCT
             *.   *  **    .     * ***. * *  *  . * *    *****. ***.

38          YSMGRMAWMATCATWYKRGTKWMTKRWARSWRRKGTWYTKRKYYYRRMKKAWGKCMKYMK
43          CSMARMRACWYTGYWTGGRYKWAKGRWGAGTRRKAATTYGRKYYTRRMKGGTRTYATCCT
             **.**      . *     ** . **. . ***.:    **** **** .  .  .  .

38          SYYYRWMKSRKKMYCTWMYKMYYYYKGGYAMRRYMRGAMGGGGMYRTGWCTYTRRWWCYR
43          STYYRWAKSRGKMYMAWMYKMYYYYGRKYRCRATCGRMCRKKKMCRYRWSYCKGRWWTYG
            * **** *** *** :*********   *  *           * *  *.  . *** *

38          YYYCGTRYWKWWSYRCTGTRCCTCMACYAGGCYKWAGATMCGWMRTRKMTTYKGCRYYAT
43          YYCMAKGCWKAAGCGYCAYRMAAAMGTCMRRMYKWTKGGCARACGKRKMAGYGAARTYMW
            **  ..  **  .    . * .:.*.      ***: .  .    .***: * ..* *

38          KGMKMRWMCAACGCYYSCYYKGWRCAGCCWRTCTATCKRCYKYTGAKGKCCKGMCTKTTR
43          GTMKMRAMAMWTRSYTSMTCGRTGYMRYMTRWTKRYTKGYYKYCAGGSKTAKKMYYGKYR
              **** *.    .* *             *  .   *  *** .. .* .* *   . *

38          YYWGATCTCTGAAKAMTKKCRTTGAGARYTKKYMCCARCCASRYTTTKKRTCTKWTKKCR
43          TCWRRYYYMYRRTKMAKKKARYYTGCMGYYKKYCTTGAMYMGGYYYYKKRWYYKWKKKTR
              *         :*  .**.*   .   * ***   .    . *   ***   **.** *

38          TTKYRKGYKAKCGTMCKKGKRKKKACSGTKKWCRRKKAYCGRKWCCTGKYWGCATTRMYA
43          KWKCAGRYKGGAKGATKGAGRKKKGTSSKKKWYRRKKGCMRRKWYTWKKCATTGCYRMYM
            . *    **. .    * . ****. *..*** ****.   ***    *    .  ***

38          KYMRTCAYKYTCKYYCGCCTYKCYTRYYRCARKKKYKTCTTAAYSRYGTTRKMGGCTCYW
43          KYMGWTTYKCCTKYCYTASYCKTYYRYYGYGGKGKYKGMKYRRTCRYKYKRKAKKTCACA
            ***   :**   **   ..  * * ***  . * ***  .    .**  .**     .

38          TYYTYTTTKYKWRCRYKKKCRTGCYKTYACYYGGCWYWACCKRRRKTAATAYRMRWRKTG
43          YCTATCCYKYKWAMACKKTYAWRMCKYYWYYYRATWYWRYYKGGRKYWMYWTAMGTRKAT
               :    ****    **.      * *  ** . ***   *  **       *  **:

38          RYKRKKKYCRWMMYKYMKRWAGMKYYTATGAYRRWKKYATCAAMTTCMRWCACTCMMKYA
43          RYKRKKKTMGTCMYKTAKGARRAGCCCCAACYRRWKKYGWTRRMCCGMRWSRYKYCCKCR
```

```
                *******     ***  *         .:..*******.    *   ***.  .   *

38              AATGMTYGKRAAKTTRARAAMKAATTCWSKKKSACCCWGCRMMWRWYRTTKRTYWGKYYK
43              MGGRCWCAKAGTKCCGRGGGAGWTCGSAGKKTSRTTMTRTRMMWRACGCWKRACARGTCG
                 .     .* .:*     ..   :  . .**.*       *****     **:

38              TCYKATWYTTATGTTYAAAMKYTAKYATGYKRYTTARTYKYAAYWCGGSTRCKTYKGKKG
43              WYYKGATTYKMKAGWYTTTAKYCTKYMWKTGGCCYMRCYTCRRTTMACCKGYKKYKKTTK
                  **.:   . ..  *::: ** :**          * *.      . ..  *.** ..

38              GGCAGTYSCCTCGKGYKTCMWWGGACKGRRWRKKTYYAACCYKGTARKKGGGGAWAWKKT
43              RAYGRYYSMYCYKTRYTCAAATTAWYTKRRWRKKCYCRMYYCKRYCAKKAARKRWRWKKG
                 . .  **     . *. .    .  . ****** *      *  . **..   * ***

38              TGATGAWMCRMKKGYYYYRKAKKRYKRSGCTAAAATRYKTRWKKTYCAAKCCKWRMAATW
43              GACYKWWAYRAKKRYYYYRKRKGRYKRCRAATCTRKRTTWGTKKYCMRCKTTKTRCCGCW
                 ..   *  * ** ****** * ****. .::.: .* .   **    .*  * * .. *

38              KRYKKKRTWCGRWRGATKYYGAKRRKSAMMMRYWAAGKKYYYKRAAKTRTMTCGKKSWGR
43              KRTKKGRYTMCRAGTTWKYCKMKGGKCWMMCGTWWMAKKYYCKATGGYRCCKYSKKSWTR
                ** ** *    *   : **   *  *. **   *  .**** * :.  *  . .**** *

38              RGMYKWTCRTTCCTWGTYAAYRTGKARTTSSRTYYRWMYYKWRTRWRAWKRYWYRAGTTM
43              GTMYTWATRCAAAAWTCYMWYRCKTRRYYSSAYYCRWMYYGTRYGWRMWKRYWYGGTCCC
                  **.*: * :..:*  *  **  . *  **  * *****  *  ** ****** .

38              YYYKMCMKYYGKYWCRCGKGCMGRWKKKKMRCTMAKRMKKCTATCRKRRRGKKRTTRWMC
43              CCYKCGMTTYRGCAYRASKKSCRGWGTTKARACATKGCGKACTAAGKRRGATKGAGRWMT
                  **  *. *     *..* .   * ..* *.  :*   *. ::. *** ..* : ***

38              STGWKTATCYKYKKTCAACMYGCTAMYWWYCWKCRKRYAYYYYRATKKGYKKMWWKGMCK
43              GCRWKCCYYCGYKKYSGGTCCAYYWMYWWYYTKYGGGYGCCCCRWKKKACKKMWWGRMYG
                .  ** .    *** ...   .   *****  *    *.    * .**. *****  *

38              RGWATGACYKRMCWMTKWRTAGASYAMYYKKWYCKKGAACGGRCGCTTKKKARCATYTYY
43              RAWGARRMTKRCSWAYGWGCRSMSYRCTTTKWCTKKKTGGATGMRYCGGKKMGMRGCGYY
                *.*.:    ** .*   *   . **    .** ** :. .        **       **

38              KAACCCRAGAACGKGCTGTKTGKAGATRWCMMRAYRWRYRYGYKTRTRRWYKMMRTRKSR
43              KRMYAGGGRRRYRKKMCKWTGAKMRWYAWAMMRRTAAGCRYRYKCRYRRWYGMMGWRGGR
                *   .  .     *     . .*     *.***      ** ** * **** **  * .*

38              RMRRYGRRCKAGCCMAWKKYKCSRAGCKMMACGRATKCRYAGYYYRYWMRRTGCGRCCKT
43              RMRGCRGAYKGAYYCWTKKYKTCRRSYTMMRMRGWYKTGYRRYYYAYACRRAATAGTAKW
                ***      *..     **** .* . .**      *  *  *** *  **:. .  .*

38              ACCAMGACAMCYTWCSCTRMGAATAAGACRYAATCGCWYRMRRYWYSTACTACKYKRGAA
43              WMYRMTWYTAACYTTGYGAAARTYRGTWTACRGGYRMACRCGACTTGWGTCGYKYKRTGW
                    *   : .    .    . :  .      .      *      . .  . **** .

38              TGCYGWCGTMRTYGCTCKRKACRTTGGCGTCRKGGTKTKMMKTGGMGKYRAARCRTACAK
43              ARMYRAMSYCGCCTTCTKRGRMRYYRRYTCTRGRRYKYGCATCTAARGCRRRGTRCRYMK
                :  *   .         **   *        *    *    .  .    *    *    *
```

```
38    YRMGCMRMMAKCAKCYGCAWKGRWWMKRKMASTRWCYYTTCCRCGKTYKCTCAATTYRRG
43    CACTTCGCCCKTTKAYRYCWTRATTCKRKARGGRWYYYCCATAYRKGYKTYYGGGATAGR
               .* :*.*  .*.     ***  . ** **  .    * **   .. :

38    TTTTRRKRAAKRYCGTTAACKCGYYYYRKCCCYTSTYTGCAYRMMKCWYKWKRCTARRMR
43    KACGRRKRRRKRYTTGCRGTGYRTYYYRTGTGCCSYYCTTGTAMMKTWTTTKATCGRRMR
      .:  ****  ***     .     ****.     * *   .  *** * . *   .****

38    KRYMWYACKWMCGCRYWYTCCSGARKGAKCRYYRCRKATRSYWARCRRRCMYMCGKKKKT
43    TRYMWYWMKTMTKSGCWYWMMCSGAKACKTRTYGMAKGCRSYWWRMARGMACCYKKKKKG
      .*****  * *  .  **   ... *..* * *   *. **** *  *       ****

38    KRKCRSRKRWGGYTWKCRGAKCTYWWSKKGKAGKRTKYACYRMGSGCTRYCCKKAWTKMC
43    GGKYGSRGAWRRCKWKTGAGTTCTATCGGRKGAGGYKYWYTRMRSRTARCYMTGRTWKAS
        *  **  *   .**  ...     .   *..   **   ** *  :*   .    * .

38    TMRWKWWWGYCKWGTAGRTKGCTCGATCTMYATTYACMARMCKKTYRATGAGYTWYGCTW
43    YAAAGTATAYYKAKCGKRWKRTWTTRYMYAYCYKCTYMGGAYGTYYRTCARTCWWYKYYT
              .* *   . * *          *. . : *.    . **: .    **

38    KSWKMAMYRTYTACGTTMTWRMKCCGGGYWRCGTTYKTAYRKGKYGRRRSYKYCAYRARR
43    GGTKMGMTRWCYRMTAYMGARMKMTRRRTWRTACKCKYGYAKRKYAAGAGYKYAGYRRRR
       . **.* *      : *  ***      ** . . * .* * **.   .***..** **

38    CYRYYYTGTKYTKRAATGGRAACAMMYCKKWTMRYRRCCWTTTCRRYAYYSGRAYKAKGK
43    YTGYCCCRWGYKKRRWWRTGRWYCCACMKKWCMRYAGATWYWCYRGCGCYGRRRYGTGRK
         *      *.**         .    *** ***  . *    *  . *. * * :  *

38    GGRRYMRYYYKKKYKMYGMTWRRCMTGMKKKGYCCWRTGGRTAYCTTTCTYMMYYKGWMG
43    RRGRYMRTYYKGGCGAYCMKWRRYAYRMKKKRYTGTAAARRYRYTAAAYYYMMYYKKTMR
         **** ***     * *.***    **** *    :. *  * :::  ******  *

38    TTWAYKWRGRSRTACYWAATCKRYKKCAGGGYKYACYWWKCTACCGTCAGGWWWGGAWTT
43    AGARCKARSRGAKRMYWTTGMKGCGKYGRKRTKTGMCATKYKRYSKYTWKKTTWRATWYY
      :    * *.*. .  **::  *   * .    * .    * .  .        * .:*

38    TGATYKMGATTTRKCKKCRYYYKSCMGGTACCACTKTATCWCYKKSRRYYYGTWSYTGTT
43    CAGACKMRRCCCRGMKKMRCCTKCAMKRYRYMGMCKCGCATACKKGAATCCKWTCYGACG
       ..: **     *  ** *   *..*      .  * . . . **.        .* .

38    AKCYCCTCWWSCTRRWKMYATCYYMRAWMYWYAYRTSATRKWTCTRKGAWARCRGCRTMA
43    GGYYTTAMWWSMCRRWKMYGAMYCAGGAMTTYMCAKSRYRKWWAWGGAGTTGTARMGKMM
      .  *  : ***  ******.: *   . *  *   .*  *** .   .. :      .*

38    RWYGACGAGCKRAACTTKCCWKTTCAMTTGAKACMKKYMRRCRYKTAKGGGGRGTAGYKK
43    RWCACAKMTMGARRAWYKYSTGYWMWMGGTTKGTCGTTCAAYRTKWWGRSRSRRWRRCKK
      ** ...        .  * .      *   :*.   .     * *    . .*     **

38    AGGACACRTACKGTCKTKCCTATTKTTAAAGKCTYRMYCCRACTKTCWRKRKKKKTGKTT
43    GATTGGTAKRYKTGAKKKMMYRCAKYWGRRSKACTGCCATGGYYTYTARKRKKKKKKKAC
      .. : .  .  *  .*.*     :*  .  .*.     .  .  .   *******. *:
```

```
38             TTCTCYGYAKAYTCAGATGTTWYGCYAAGTGRKACARACAWYRWMYCRRTRMACCTCAKK
43             CCTGTYRYGGCCCTGACCACWWYRMCWWKGAGKRYTGMYTWTGAATGAAGGMWYMCYGKK
                    * *. .   ... .  **       . *  :   :*          *     .**

38             CTTRAKYYTRWAGGCWMYRCTRAATWKYRSKYYYYYKATCTMTARGCKRARKWCRGATTC
43             ACCGGTYCCGWGACTTATATWRGGGWKYRSGYCTCCKRKYWMKRGKYGGRRKWYARWYCM
               .   ..*   *..         *.. ***** *     *  .  *.        ***

38             WYRYRYCRYRYAAYKSGKYRKRSWYRKGRKKKTTGRGSCCYGRCRYRRCCGGACRYYWRT
43             TCGCRYMRYAYGGTKSKKCGGRSWCGKAGTGGYAARKGMYTRRYRYGRYTTRGMRYTWGK
                   ** ** *.. ** *   ***  *. .   :.* .     * ** *     . ** * .

38             GTYYRYRRCKKTTKKMWRKTMTYRYWKRMKAYYKAACACAGGMGRYCATGMRATYKKKYR
43             AKYCRYRGYKTCWKKMTGKCCGCGCAGAMKTCTKGGGGTGRRAKRYAGGAARCYYKKKYR
               ..* ***  *.  ***  *          **:  *.. . .    **.. . *. ******

38             TCTAYMMKKWYARCKWWWGGMSWAKRYKCTRMYKSRYYYRATTARCCKMMKRYYKYYTCG
43             WYYRCMMKKWYRRYKWWWTACSTTKACKYCRMYKSRYYYGGCCWAYMGCCKRYCKCYCTA
                    ****** * ****  . * :*  *  ********* .         *** * *  .

38             GTTRKKKCCGCGKCAYKYRWGGYRAMTKWWCKKKGRTAGMYKRRTGYKKKATKAWGKMGA
43             KYYAKKKMYTYAKYRYTYRWKRTGRACGTAYKKKRRYWRCCGAAKCCKKKGAKMTAKART
                   ***    .*  *.***           *** *         .  ***.:*  .*  :

38             CCTYYCRGCTWKATGARMCACRSYRRYRRTKMYTRGGYRYYCCWWMYTCTGRKRKRKGRS
43             TYKYYTAATATTGCRGGCGMYGSTRRYRRKKATYRARTGTCMTATCTGYATRTGKAKRRG
                 .**   . : ..   .      * *****.*    *.               : *. * * *.

38             YSKWYRYRRATGCYTCTAYAGTTSCYMWKCAKSYCRCCTRATAARKYRCCTCSWYKYWWM
43             YCTTYRYRRRWKSCCMAGCGTYYGMTCTKTGGSYTRTTWGCKCMGTYRMYYYSTYGYWWA
               *.. *****    .   :. .    .     * . ** *     ...   .**     * * ***

38             ARTMKTMTKRRKRGCTCACATATGKYAAYKTARAYGGRRTGCRKRWCTGGKKACKYYYKM
43             GGCCKYAGKRRKGKMGMGMRYMYRKTWMTTYMARYRRRRKRTGKAWAAASKKCATYYCKM
               .   *   ****      .      *     .     *   **.    * *.:..**...** **

38             YKATGGCGAWKRYTGMAKSYTKACKKTKKRYRRRMYYKKRRKRWKAKRWWKKKRRTRKYT
43             CGTCTATRRTKGYAAMMKSCKKMMKKKKKRCGRRMYYKKGAGATKRKGAAKKGGGGAGYC
                 :  .    * *:.* ** .*  **.***  *******      * *    **       *

38             GCTCAACKKKKAAAKRKKRKGTGKGKGARKWCKGMRAKAYCCCWWRWWCCGTATKRTTGC
43             ATATRWYKKTKWRMGAKKATAGAKTKAGRKAMKKMGRKRCYMMTTAWWAYTKGGKAAGCA
               . :     **.*      ** .. .* *..**  * *  *          **.   .. * :  .

38             KKMWKAWRKKGRYYYKSRYTGWYSYAGKRKKYCAGTGYGCRMYKYYAWRACYWRTYTTAT
43             KTAWKRAAKKRGTCTKGACYRWYCCWRKGKKYGRRYRYRYRACKTYGWGTYCWGCCYYRW
               *. **   **      *.      **.   * ***       *   *   * *.* :  *

38             GTKYYMGGYRRKWSAYYCAKRRYKGAAYTKGGKTTMAGTKMYRMCKGTRKKKKWKKCCGT
43             RWKYTASRTRGKWSMCTYTKRRGCGTCGYKKATGCCMMKKKCTAMMKRWGKGKKWKKTTAY
                 **   .  * ***     :**     ..*.*.      *   .*     * *    * *****  .

38             RKKKKRYYGRYYRRRKYKKAYCKATRCCAYRAACRKYMYAAGGKTRRGTRMRYYCGARKS
```

```
43                RTKTKRYYARCTARRKYKKWCTKCKGMYRCRRWYGGCMYTGTAGCRACCRMRYYMRRRKS
                  *.*.****.*   ******   *..     *      **:. .  *   *****   ***

38                KKRR
43                GGAG
```

The pairwise alignments of two accessions 38 and 44

```
38              YWKKYYTKYCKSMYCMACYYSYRGWWMSRRTRRRGRGRRYTMYGMSYKRRGCRCYAAMWM
44              YWKGCTCKTYKGMYYMRYTYSYGRATMSRRYRRAKARRRYWCTRASTGGAATATTCCCWM
                ***    *  *.** *   ***    **** **    ***     *    .     ..**

38              MMRKYWMMRKKRRMYYRYKMYRAGTATGYMKYRACTAWRWTCYRWRYRYGWSWRWWRKAY
44              MCRKYWMMRGKRACTCGYKMCATKYWWRYMKYRTTGTTRTGYYRAACAYSWSAAAWRKWT
                * ******* **     ***  :     *****:  : *   **    *.**   ***

38              RWYAACYWATCTYYTTRAYWRWWWGYKWRSTRWYYMKWRRWRRKAAKCRKGAGYKGYYRK
44              RTCRMACTMYYYCTCARWCWRWTTKYGWRCWRWCYMKWGGARAKGGGTAGRTATKSCYRK
                *    .         :*  ***   * **. ** ****   * *..     :. *. ***

38              KMYYYRKMKTRKWMCYWWWSMMWYMWRCGYGKCRCGCGWTRYMRTYTKGGGWYCTGRYMW
44              TCCCTGKCTCRKAAYYWWTCCMTYMWRYRYRKYRTTMKWKATCGACWTTRAWYYCTRTCW
                .     * . **   *** . * ****  * * *    *.    :  .  .**   *  *

38              WRRWYWRRKYKYYRRAAGWYMYMYYTRSRRMAAWKKKMRYRMTYCGWGKYRAYTCWKRCM
44              WRRWTWRRKTTYYRRWRRWCMYMYYKGCRRMMWAKKKCRCGCKYMTTCTCGCYAAAKRMC
                **** **** .****   * *****. .***   *** *   .*     . .*:. **

38              RYYWWMYKTYYWMRRKRRRYYRYRGAYMSRMMYKYGGYWTGGGTAYTKTKYYMCYGCYCY
44              RYYWWACKWCYWMRAGRRRCCRYGAGCCSGMACKYAAYTATRRWRYYKYKYYAMYTMCTY
                *****  *  ****  ***  ** ..  * *  **..* :     * * ***  *    *

38              MMRYRWMRYGWRCRRYYRTAGKYCWKYAKSRMTSYMYWRRYGTRYRRKRYWYMRYYYWSM
44              ACATGWCRYKAGMGAYYRATATTGAKCGGCRMWCYCYWRRYAYRYRRKRYWYMRYYCACM
                     * **      ***::..   * . .** .* *****. *************  .*

38              YMRMRWRSGYYRKTYRYKKMRWWMSMWYYWWKSGCRRATYGKTAARKYYACGYWRYYYRS
44              YMRMAARSRYTGKYCAYKKMRWWASMTYYTWGSRYRGWKYRKWWMAGYYCYRTWGYYYAG
                ****  ** *  *   ******* ** ** * *  *  .* *     **.   * *** .

38              TYKWGCAYKACCATYRRRCKGCTKKYYAARRRKKRMYYMKKAYMWYACWKCCCGCYYYRR
44              YYGTATRCKTYSWWYGRRAKATWKKTCGMRGGKKRMTTCKKTYMWYWMTKYYYKSCCTGG
                 *  .   *: .  * **.*.  **  . *  ****   **:****   *    .

38              RRYRWGKTATYGCATCCASRTYYSKYGARKGACMYRWYYCCTCRGRGSRCRKYMRGCRYS
44              RRYRWTKYWYYAYRWMMWCAACCCKYSRAKACYMYRWYCATGTRKGTCGAAKTAGAMACS
                ***** *   *.      . :  .**.  *.. ***** .   *   . . *   .   *

38              YWRRRYKKARWKKTKARRYGRGYYWKRMRWSYYGAGAAWKGCYAYMWYMKWSKKRYKAGR
44              YWRAGYKGTRWKKGGGRRYRGTCCTGAMRWSYCRGAGGWKRTCWTCACCKTCGKRYKGAG
                ***  ** :****  .***        *****  ....**         * . ****..

38              CCKGWYCTKTKYKGRTKACKKAACRYYYYMKKWGRMTCTTYGCRKRKKRKGMRCTCYYMY
44              YTTKACYCKYKYKARWTRSKGWWSRCYYTMKKWRACGMWACAARKRKKGKRCGTGTTCCY
                  .     * ***.* . .*   .* ** ****      : ..***** *         *

38              TGCTMAAKAAWAAYRCYYGYRKTAKKCARGATGTYTTGTYTCTTKYGCKTCCMTAWRTAR
44              WTMYMWRKMRTGRCGYCCRCGKKRGGMGGACCACYYKRWCCTCCTYRMKWMMAKRTRCTA
```

```
                          *   *    .            *.     . .. . * .        .*   *     .  * :

38                    CGTTAAWYACRAWKYYRRMKGTCATRMATAYCYRGGAAGCACAKWRGRYKSKYGKTYYTR
44                    TRACGTWYWYRTWKCCAACGTGYGGGMWCGTMYGATTRCTMTTKWGRGYKSKYKKYCCCR
                         : .:**  *:**            .  *  .  *  . :      :**   ***** *     *

38                    YYWMATYCGWYRYATKAYYYGYRKYWGAGTTYWRGAYAGCRKTTCACRRCTYCGYGMKYR
44                    YYTARYTMCWYGYRCTGYTCACRTYWRTACGYWRAMCCAYRKWYYMTGGTCYTRCRMKYA
                      **       ** *   ..*   . *.** :.  ***.   .. **          *    ***

38                    KKMRKYCKGCKKCTWASYWRCKRATYKYTAKMCAKSKTKTGTKYARRMYRKSKRYAWYRR
44                    KKCAKCAKKMKKYWTMSYWRYKGGKCGTYWKAYGTCKKKYACKYGRRMTAGGGRYRTYRR
                      **  *  .*  **     **** *  ..     *   ...*.*  . **.***    . **  ***

38                    RRCAACTGTKTRTRSYYYYYKRCYRRGMCGWGRYMWKKKKCKWTWRTAWKKWCYKKRTWC
44                    RRMRRSKRKKCRARCYYYYTKRMTGGRMTTTRGYCTKTKGYKTYWRYMWKKTTCKKRYTM
                      **    .. .* *:*.**** **      *      *  *.*  *  **  ***   ***

38                    GWRYKKWRKTCGTKWKYACTATRKKACTYKCGCAGYRGCTAYGKYWKRGRRSMAACAYKY
44                    CWRCKKWRGYYRYTWKYMSGCAGGKCMYYKGATGRYRRYYWYAKYWKAKGAGMGGTGYKY
                       ** ****      .*** . .:  *.   ** . . **    *.****     .*.. .***

38                    ARKGYRARRRTTWKYKMRYYKWMRYKTCRTGKKYRTAAAYCTCWKKKMSGCYRSKGAMMK
44                    RRTRYRRRRGAAWKTKMRCYKWMRYKGTRKKKKTRCTMTCMKYWGKKMGAYTGGKKGAAK
                       *. ** ** ::** *** *******  *. ** * : :  . * ***..   .*  .  *

38                    RKMKYRCSWYKKCCAKTTYTCRRRYTWTACMYYYSACWAMCRCRKRYTGKCATKCKKYKK
44                    GKMKTAYCTTTTATMKYWYCYRRGCKWWRSACYCGGTWRMYAMAKRYWRKTTGKTKKYKK
                       ***    .  ...  *  *  **  .*  .  * ..  * *    ***  * : * *****

38                    TKGAARMWYGTCTAGMKKGYMMARMAWRYKCTCKRYGRTRKCKAKMYSRCWMKKYAYKTR
44                    YKRMGGATYCKYYRKMTTACMCRRACARTTTAATRYRRWRKATWKMYSRATCGKCGCGYG
                       *  .   *  .    *... *  *  .  *  . :..** * **.. *****.   * .

38                    AKTYWTAATACAKCTCGCCMYRKYCTRSWCAWGKRRYTRGWSAGARRRKYRACGATTGYC
44                    RKKYTYRRCCTCKTATTTTCYRKYTCGGTYCWKKARYWAKWSWKRGGAKYAGGACYKAYY
                       *.*     .  .* :     ****    .  .* * **   **       ** . .. ..*

38                    CTCMCKRYYATAYRYTMRAATTGKYCGKYGTYSRKKAGGWTTTCCKKAMSRTTGGCCMGY
44                    SGYMTTATTGGGCRCGCGTGCYRGCMRKTTCCGRGGCTCWKKWMYKKTMGGKCRKYYMRT
                      .  *  .   . . *    :.       *     .*  .  *..   **:*.  .      *

38                    RYAYCYYAAMTGCTGCTCWWTMCGAAYGCGCYYYCAKAYGKMGMCKTTGRMTTKKGCTAC
44                    RYTYMCCGGCYRYWRTGTAWYMTRMRYRYSYCYCMRGCCKTCAMAKCKAGCAYKGSTGTT
                      **:*    ..            * *     *   .   *      .  .  .*.* ..  : *  .  :

38                    ACRGTGARGKATGYMRKRRKRKTCRKGMTWTMMGKYYATRKTGTTRKYMCTKTKACACAS
44                    WMRKYRMRRKGKRTCGKRRTGKCTAGRMYWCCARGCYWKRKWSKGRKYCTCTCTRYCTMC
                        *     * *..    ***. *      * *       * .** .. ***    .  .  .  .

38                    KYRARKRKYYAKYCMCSKCGRTTGKWYYRAKKGARAKKKAGTGGARTACARYYMYYTCCT
44                    KYGGATAKCYRGYYMAGGYRRKAKKWYYRRKKSWRGKTKGTWRRGRCCTTGCTMYYYYYY
                      ** .  . * *  * *..   *.: ***** **. *.*.*.     .* .  :   ***
```

```
38          AYRYARMCGAKCMCMKMMRYWYRYTCAATTYKTGAGRKYRTGTTTAAKKKYRKTAKYRRY
44          GYRTMRMYRGGMCMAKCCATWYGYYYRRKWTTKKMRATCGARCGKRRKKKYRKYRGCRRY
            .**  **  .     *    ** *    .  ..    .  :   .  ******    ***

38          MMKKWYRGGACKMWWWGRGRRSCATKTSKRYAMSRGTYMKCMTATKRMYASRYYWYRKRA
44          MMKGWTRTRRTGMTTTRATGGSTMAKGSKACMCSGRACCKYMCTKKRMYGSRYYWYGKAG
            *** * *     *        *  :* **    *  :  * * :.****.****** * .

38          TTKATYRMATAKKCCGCTKMTGGYTCTMRCAYGAKKKCRYTCATKYKTCGAAAMYKMWKR
44          CCKMGYRCTWTKKYMSGGKCACRYGTCCRMWYAGKKKMRYKYRYKYKCTARWRMYTMWKA
              *  ** : :**  .  * :  *    *  *..*** **.   ***  .   **.***

38          KTCTKGCRMKRTGKTGMRWKTTRYWCKYRYWAKYRKKGWWCYMMTMYRMCCGCRCTRRYK
44          TWTCKTMGCGRYRKCACATKKKATTAKYRTWRKTGGGTTAYCMMKMYRCMTRYGTAAATK
            .   *     *  * .   *..   .*** * *         **.***       :   *

38          TRYKGGWAACCTMKYCKTTYKYYKSWKYYSWGYAKKRTYYGYGRRAYKKKAKKRWYCKYY
44          YGCKRRAWTMSKCGYYKKWYKCYKGAKYYSWKCWKKRYYCRCKAACYGTGGKKGATYGCY
               *    : ..  * *. ** **. *****   *** *      .* . .**      *

38          KYMYGYGRKSWGKWSTCCRYKYYCKRTKRYGGCRWRCGAGYCCKKMYRMCKKGTCGRYKY
44          KTACRYCGGCARKWSYYTGTKCTYKRYGACAATRTRYRRRYYYKKCTRMMKKRWYKGTKY
            *    *   .  ***     *   **    .. * *    *  **  ** **      **

38          KKKCKKTWKCCGTTAGRRRYRAKSKAKCRAACTTAMRKAAAYRCRAYYRKRKRRRTRYSY
44          GKKMKKKWKYYAWYRSRARYRWKSKRTSRGCMWWWCAKGGRTGYRWYYRKRKRRGCGCGC
             ** **.**  .   .* ** * *** ..*..      *..    * ********    .

38          MGTTYKYWKGCYYAAKGYSTKYKTKCRCYMYAACRKYYCTTGAAGGCKTTRMRYYKCCCG
44          AKYCYKYWKATCCGTKSCCCKYKKTTGMCCCRWYRKYTYCACTGTAMKWWRMRYYKTYMR
                *****.   .:*. . ***..         ***   : :. . *  ******

38          ARKTAYSKKRCTTKKCAGGRAMYWRMYYYCRSYRYAMYAAMGKGARGKKKACCYYYTYYC
44          RRKWRYSKKRYCCKKYCCRRMAYWAACYCMRCTATRMCWRAAKRMARKKGRYMTCTYYCA
             **  *****   ** .  *  **   *  *.    *    .*    **        * .

38          TCYRKTRAGTRWAGGWMAYYKWGRWYAWAGCYRKYAYRWSSSCRRYRMCCRYRGRGWCCC
44          KYYRGYRGRCGTWRRWMGYYKWKRWCGWMATTGKYMYRWGSSYRRYRMYMRCRKRATYYT
            . **  *.       **.**** ** .* .   ** ***.** *****  * * *.

38          AMATGGTGSKKYAYTRGCWYTTTRWRAWKTKRWGCCGTAKMMRKMACGGAGYAGKWKCCT
44          TMRYRSCKSKGCTCCASTTYWCYGWRMWKKKRARTMRCCKCMRKMGMSAWKTWKGAKYTC
            :*   .  **  :   .  *    ** **.**      .* ****. ..       *

38          TTKRWAYKSMKKKYRKMMRWAMRGKRTYRGTTTTKRRTCGAYMCYYGKKKRRWATTMKYR
44          CCKGWGTKSAKGKTGKCCRTCCAKGRCYGKAAAAKRRCATTYMMTCKKKKRAAMKKCKTG
              * *. ** * *  *  * .    * *  ::::*** . :**    ****   .. *

38          ARMWGKSTTKWYCGTYKRKWAAAWYRTTCYKKYAYKAKYAGKKRRYGTCTACCCTRMTTS
44          GRMWRKSCCKWCTTCTKRTAGMRTTGGYYCKKCGYKMKYGAGKRRCAKYYWYYYYACYWS
            .*** **  **     **. .         ** .** **.. *** ..          *
```

```
38          GGAATATYCCTGCTKATAYYRYAYYKGTYRRTCYTYATWKMSKKGYKCKCCKAGTCYYGK
44          RAWRKRYYYMWAYWKWWMTYRYGCCKCCCRGCTCYCWKWKMSKKACKYKTAKGTCYTCRG
             .  .  *   .  *    ***.  *   *       .******. * * .*.

38          KKMGRYMTKKGKTKKTMKKRMCTRRKWCATGAKAAYTCSTTTCKKYYRKATTYTAYWKKG
44          KKMSRYAKKKKKKKKYMKKRCAGRRKWYRCTCKWRYATGYYYATKCCGKTAYCCCYWTKR
            ***.** .** *.** **** . ****    .*  *: .   ..*   *::   .**.*

38          TYAACRTYKYMCWWGGGTKCTTGKKAGCAAGCKTCWCAGRMYKCGCSKGYRKKKTACCAY
44          WYRRYGYTKTCYTATSKKKTCGTKKTATCCRYGGYWTRARMTKYKMCKKYRKKKYGTMRC
             *      *      . .*    **:. ..     *  .** *   .* ***** .

38          GTAGKWKKWCKRRKKYKYMRKKTTKMKWACCGGGRKGYKTRWKTGATRYRWGTARRTYYR
44          CAGKKWKKWYTRRKKCKCMRKKYYKAKWWTTAASRKRYKWGWGCATCRYRAKWRRGWYYR
             :. ***** .**** * ****  * **   ...** **  *  .: ***    *  ***

38          KYTCCRKATGCACTYKKYTSRRRKMWKRKCWAKAWKTRSARYYTGCRKKKYKYGCGCGCG
44          KYYYMRKRACTRMWCKKCWSRRRKMWKRKYTRKTWKKGCRRYYKRTGKKKTKYKYTMAMR
            **   ** :      **  **********   *:**. . ***.   *** **    .

38          KAGRRYKGRTRKWKWSWRGACGGAYMGGSWTARRYTTYWRRRYTKCRAATYKKRRGYTKT
44          KGRARYKARKRKAKWSWRAGYRRGYMKKSAWMGRCYYYWRAACYTYAMCAYKGARRTCKY
            *.  ***.*.** *****..   .**  *    *   ***    .   .:**  *   *

38          TCWTWYRWACRRKTATAYRTYWCWCCWTYGMKCGRRWMMAATKAKKYRYCAMKYYYKYTA
44          CGTKWYRWRYGRKGGGWTACCTYTMMWAYKCKMKRGTCMRRKKRKKYRCACAGCCCGCAC
               .****   ** .           *:*  *  *   *  .* **** ..       :.

38          MKGTCRGATGYGAYRYCMRWKCCAGYWMMYKGTRYRRYAMRYRCYWKSRKKAKAAKTTAT
44          MKSWTGAMAAYRRYRYGMRWKSMWRYWMCYKKWATGACTMAYGMYAKSAGTTGWMGGAGC
            **.   . :.*  *** ****.   *** **       :* *  * **  .:     :.

38          MWRSWYYSRWGAATYKMWWYRRRRRRKCYTKARKYYMKRKTKMKRGCCGAATRKTTGYYK
44          MWRGWYYSAATGCCYKMTWYRRRRRRGYCGKRAGTCCKGKCKMKGKYTAGRGRKYYRTCK
            ***.****   .. *** ********    *      * * ***     ..  **     *

38          RYRMYKCRCTMCRMKRKYMKKSRCRKCMTRRKSKGKKYRKRKCYYSGTYRGKRCKMKKTY
44          GCRMYGTGYKCYRAKRKYMGKCRYAKACYAAKSKKKTCGGRGTCCSRYCGKGAYTMGGYY
              ***    .  * ***** *.*  *.    *** *.   *    *        .*   *

38          RCKRGTGKGAAWYWRRRYRRCRCKTTYCRYKKYCKWRRTAGGTGCAYYRYRTYTYKKRRY
44          GMGRKCTKKMMWCWRARYGRYAYGYWCMRYKGTGKWRGKGKRKAYRYYRYRWYCYKKGGC
               *   *   * ** ** *        ***   ***  ..  ..  ***** * ***

38          TATATTAKTGKMWRKTWGCYRATATYRYWKRMRTCGGTGGMGCRRKWTAKKCARWYMRKT
44          CGCWAWTKWAKMWAKCWATCACCMKCGCAGAMGCTRKYRRMTYRRKTKWKKTGAACARKY
             .  : :* .*** * *.   .  .      *        *  *** . ** .    **

38          TYYMRTSTTTWCYTTTAKKWYCKMMWATTWKKATYYRWWSCWSSYWTTTARRWKRRCMYK
44          WYYMRKSKKWTYYGYWGGKWYYGCATWKCTKTGACYGWWSMWGSYTCYKWRRWKGRTMYK
             ****.*..   *   . ***      .  *..: * *** *.**   . **** * ***

38          ATRWACGTGYTKGAGTYYSTKCYATTGTYRKSCYATTKKWWTRKYRKGAWATATMYACMK
```

```
44        GAGTWTCCAYAKSGTCCTGYKYYCACKYCAKSMTRYWKKTWWATTGGAGTTWRKMCGTCK
          .:       .*:*..     . * *.:      **      ** *   .    .. :  .* .  *

38        TRRCAGCWGTYGMAYKTKKMYWRMTCATCTTGAKACMKMYRYTYCACAYAGRTKRYYKCR
44        YRRYRRMAKGCRMMYGKKKCCTAMYMMYSKKKWGGTCGMYRCGYYGYTTGKRYGGTYGYR
           **          * * .**     *     ...    .   ***  * . : . *     *  *

38        GRYRAGYYKSAGGCYRACAAARYCTCGAACCMYKACCARYGYCKWYKGKKYARRARRAAT
44        RGYRRRCTGCGATYYRTMWRTAYMCTKRRYYCCGGYYGATRTYKTCKTKKCWGRGRGRWC
            **      ...  **:   : *            .  .      *  * **   *.*

38        GCAKKSRRMYRMTGTYWWKKWWSYCYYRARKTRAWASGTAACYRYARTTTAYKRRAGCGT
44        RTWKKGGACTGCKKYYAWKKAAGCTTCGMRKYRMWWGACGGTCGYTAGWAGTGAARRMTC
             **.       .  * ***   .       ** * * .. ..   *:    :.

38        MYTKGYKKCKKGGGRTCMKGMGCKKRYYRYGGYTWTYCTKMYMKKKARCAAYRRAGAYMW
44        CCYGKCKKYGTKKRGCMAGRMSYKKRTYRYRACYAWTAKKMTMKKKGATTRCAGTCGCMW
                 **  .          *. *** *** .      ..** ****.  :     : . **

38        STYKYYTGRCYYYRCAMSKTYCRTAKKGCTARWACYGCTTTRTYWAGWKGKGYKYYKGYG
44        CWCKYTKRGMCTCGYMAGKKYTAWRKKKAGRGTGYCKYGWWRGYWWKTTRKKYKYYKKYS
          .   ** .            .*.*     ** .     .        * **    . * ***** *.

38        TTTCCTACATWAGTATKRMATGACYRWKKTGGMWWKRCTGKRRGGGGCYRWGTTCMYKKT
44        CYYTAYMYWAWMKKCGGACGGATTYRWKKYRRMWWGRYCRKRARRRKMTGTAGGSCYKKY
               .     :*  ..     . .: *****   *** *    **           .  . ***

38        GGCWAGAYAGAMTCTCYYKKWWYKCKCTRKGATGWMCCTAACAAWSRWAYKGTYRYKTGK
44        RRYWRSMYWKRCYYKYTTTKWACGTGTKRKATCRTASMYTMMWMWSRWRYKTGYRYGYRK
              * . *        .   .**       .**.:     .  :    **** **  ***    *

38        CGAACGGAGKGCSRRKRYKCRYTGAWWTRYMMKMSCCGAKYSMTWRRYYKTRWGWAAGRY
44        YRRRYRRRRKKSGRRKRYKYRYGAWWACGCMMKMSMYRRKYCCCAAATTGYRWCTWGARY
                      * ..****** ** . *     *****     **.            **    ..**

38        GKMRMRRRKWTKCCCCYAGMTTSSRGSARYTTTAKRCYRRCYWACKKYATYCSMTYAKMM
44        RKMRMGRRKWYKYYYMTMKMKWSSRRSCRYAGARKGMCGAYYTRYKKYMAYYCCGYRKMM
           **** **** *        *. *** *.**: : *       *    *** :* .   * ***

38        CRATCRYYYYYCRKRRKGTYATGTYKAYKYAKYKTARCYMATGCWGAGSKACSKKWKRRY
44        YGWYTGYCYYYYAKRRGRYCRKKKYKCCKTMGTKCCAMYMRYRYARWRGTGYSKKWKRRY
                 * ***  ***       . .**. *     * .  **          ... ********

38        WRGACCKKRWCGMACTCKRYYMKKAAMMRKAKRCMRMKGKKKTYRMKCYKWMMKKATTKA
44        TGATATKKATYSCTTAMKAYCCKKWRMMRKCTGAARMTKKKKAYRMKMCGWMMKGRKYGT
             .:. **    . : : * *  **  ****.. . **. ***:****    ****   .  :

38        GCATGGCTKWKYRAKRTTTCMYWRKYRTYSYYYTKKYGKAAAGARRWYKRKKYKYYKRRK
44        ATRCTATYKWKYGCKGCACTMYWRGYRGYGCCTKKGCKTRRWRGRRWCKRKKYKYYKRRG
          .     .  **** .*   :  **** ** *.    .*     .      .*** ***********

38        RTKGAMYATGGYRWYTTYAAKMRRYYGCMKAAAMATCAKRYYACMYYKMAYRKMKTATGY
44        RWTSMAYGATKYRTTYWYCGGMGGYYRYMKRWWAGGTMGGCYRSMYYKMGYRKMKYMWKY
```

```
            * ..  *.:  **    *.. *  **  **    .      * .*****.*****    *

38          MYRRWMRKAKTTAYYRRCAAAACSYAGKATYCKTRYTTCKAAAGGTGKKAKRWKTGAAYY
44          ACRRWCAKRKCGRCTRGMCCRGMCYWKKGCTTKKGYCAAKGGTTACAKGMKATKWAGGCC
              ***  * *     *  .. . .*  *.   *. * :.*..: . .*  *  * ...

38          WKGWWYKTYYKGYCRKKKTCRRTKYGATMTCAARRRYKMMKTGRAAKKAARWKRKAYMAR
44          TGTAACGYYCKRYSGTGKWSRAKTCRGWCWYRWGRRYKCMKWTRTTTKRMRTGAKWYMRG
                    * * *. . * .* ..  .       **** **  *::.*  *   * **

38          YMRATKGTGTTGRGTGCCRCRKGWCCMCYKMCTAKARSKYCCCWRWRGKACATRKKTGCT
44          YMAWYTRKRWKKRAAAYSRYRKKAMAASCKMYWRKRRCKCYMMTAWATKGYGGAKTKSMG
            **   . .  . *.:. .* **   . . **   * *.*      *  *. .  *...

38          GGRRRKMCGACWRKKARYAWGYWKKSRYTGGCYSYCAGKKRKRKKYTGGCKYKGCWMYYY
44          CKRGGTAYKRYAAKKGRYRWRCWKKGACYRRYYSYYWRKKGKRKKYWRRSKYKRYWMCYT
              *  .       **.** *  ***.      ***   ** *****   .***  ** *

38          YKYYRRACYKCAMYKRGTWACCMCGKRCRCAGRTGCGRTTCAMTACCAKRGTCYATTTGM
44          YKYCAGTYCGYCATKGSYWMTTAATKRYRYRRRAATTRCYYMMAWMYRGGRYYYRYGCCM
            ***   :    .  * . *    . ** *   *:.  *    *:         *     *

38          YYGKKRWGYATRWMGCATCTTGAAGGTAAAGCYMMKTYYRCSRACAGRWMYKRRMCRMKW
44          YYAKKGTRCWYGTCATCGMACARRRKKCRGAMYCAKCYYGYGGRYGTRWACKRGCAAMGA
            **.**         . .  : .    .. .. *  * **  .   . **  **  . *

38          ATCGYCCWKCRYYYWCCKAACMAKACTGCAMAYAMYCCKGAATGMGMTRTMYCKTCAAWK
44          TWMRCMYWKYRYYTWMMKWGGMGKRMYSMRCRYGAYSYKRRMATMRCCRCACYKGMWWAG
            :      ** *** *  * . *.*   .    *. *. *   : *   *    *

38          KCACTSTWGGYRWYGYYYMMYGRTTKGKRWMYKAACKGTTRCKWWYRRWKWKGCSRTACC
44          KTGTACGWRACRTCRYCTMMYKGAGGRKRWMCKTTAKRAWRYKATCAGAKAKRYGAKRMM
            * . :. * . *   *  ***  :   **** *::.* : * *      * *  . .

38          KAAKMKYGTRGYGCCGKKRRWKCRMKKAAAGKKCGAMATAGTAWAWRCTTKKYCAGYRAC
44          KRCKMKYKWARYAYYRKKAAAKYRMKKCGCAKKYRWARYWAKWTRTGMYYGTCYRRCGTY
            * .****    *.   **   * ****....**       ..         .      :

38          WTGTKWYRKGATTMACKRMKGRKYTRTTRRRMKKKSCYMCACGSGKWKYYCAKMGGGKRT
44          WCAGKACRTAWCCMMTGGAGRRKCYGYYGGRMKKKSMTMYMYKSKKTKYTYWKMKKRTRW
            * . *  *..   *       **       ******  *    * * **   **   .*

38          AKKTATCRYRTWRKYKGCRTKSRRYRKATAAYRYRAWGGGGKKRCMCTGCTKYKKMGCTC
44          MKKCGCAGYRKTGKCKATGCGSRRYRKGCRRCRYAMTAATAKGAYASKRYKKYKGCATAT
             ** . . **.  * *.    ******.    **   .. .*    ..  .***  . :

38          TRKWYRAYCWGWRYYMKCKRKYMYMYRRYAARRYKGYMYMRYYKKWAAMKRYWAATAKYA
44          CGKWYGRYMWRTAYYCKGKRKYATAYAATTGRGTGATCCAGTCKKAGCCKGYWRRARKYC
              ***  * *   ** * ****   *   :.*   .       **  .. * **  : **.

38          RRTMWGRKGCGGRWKRKGTYSKYSKGCGCGAYKRKKCKATRMKCYYKSACWYSRYKYAWA
44          GRWCTTATRSRRGTGRKRKYCKTCGKYRYSRYKRKKSKRYGAKYYCTSMTACGGTKYGWM
             *     . .     ** .*.* .     . *****.*    * * .*    .  **.*
```

```
38                KKCSKWRKKCRTYKWKRCAKKWKCCTCCATGKWCYWTAWKYARKMG
44                KKYSKWRKGMGCCGAGRMMKKTKMYYYTWCTKATCTGRWKCGGGCK
                  ** *****        *  ** *        *      ** .
```

The pairwise alignments of two accessions 38 and 45

```
38              YRMKTRKYWMYGRWCTKYYWYRKWRYRRYCGRYMKCTWACKCRGYRCCAKRCYMRYYAYR
45              YRMKYRKYAAYKGATGKYYWYAGWRYGRYTRRYMKYWWCYGGRTYRTTRKRAYMRYYRYA
                **** ***  *     *****  *** **  ****  *.   * **   **.***** *

38              YCKKMWKMMRGAKWKYWRCYGAYRWMMYMKKRKYRYKMTTTTYRRRRRGAKRTYGCKYWR
45              YYGKMWTMMRRCKWKTARMTTGYRWMMYMGKAKYACKMYCCWCAAGGAKRGACCAGTYWR
                *  ***.*** .***  *   .******* * **  **                . .***

38              SRWWRWYMMRWRRYWYRYYKSYKKCYGSSCATYRSYKYYYRWATAMKSYSYRKAYYSYGK
45              CGTTRTYMMRWAAYTYRYTKSYKKYCRGSMMYYGGTKYYTGWRCMATGTSYRGGTYCYCT
                .   * ***** * *** *****   .*   * . ***  *    .. *** . *.* .

38              YRAYRRRCKRRRSGAYMYYYRMKTRYYRRYWRKTKGYSGSYWRRRGGSTRGKGTCWRSYA
45              CGCTGARYTRAASARYMYYCGCKWGYYRRYWAGYKTTCKCYWRRRRTCWRAGRAAARSCG
                  .   * .*  *. ****   *  ******   *  . .*****  . *.  :. ** .

38              CAKSYCAAYKWYTGRWSRGRRGATSWYKRYAKTTGYGTARYCRMYSKRRYKYYYMRWYSY
45              ATKSCTTGYKWCKRRWSGARAAGYGTTGGTRGWKTYRCGRCYGMTCKRRYKYYYCRWYGY
                .:**  :.*** . *** .* .. .        . *  .*   * .******** ***.*

38              WWRWRCAKMRMMKSRKAYWWYCGGTWSMKCCARCTTGKYYYYMYYRRCACWMAYMRRYMM
45              WWRWGAGKMRMMKSRKRYWWYTAACTCMGTMWAMYWTKYYYYCYYRRTGGWCCCCRRYAA
                **** ..********* **** ..  .*         ***** **** . * .  ***

38              RWRWTCKWMRRRYYYYWRKRKWYTYCKAYWGYWTAGRYRWKYRYYYWSYYWKYYYCKMYY
45              GAAWCTKWMRAGYYYYWRKRKTCYTYKTCARYWYGKATGTKYRTTTTGCYWKCTTYKMYT
                   *  ****  *********     *:   ** .     ***    . ***    ***

38              GMTMYMMYGYYKYYWYMCCMTMGYYRKYMRYGAYRRYRWYYWTCAARARCCCRYYRRCKK
45              RMWCTMATRTTTCTTCCYYMYCRCYRKTMGYRGCRRYRWYYWYYRRRMRAGYRCCAASKK
                 *   *     .       *    *** * * . ********    * *.  *    .**

38              YYRTRAWMYTKMRKWRYRRRRKYYRAYRYRWKYMCMAMRYRKGYYTTYRWWKGKTTKKGT
45              YYGKGWAACWKMRGAACARRRGCCRGYACGAKCAAMTCRCRGCYYYYCRWWKTKKYKGAC
                ** .      ***     ***   *.*    *  .*: * *  **   **** *. * .

38              GTACTRTRKYRTGAKKRTTGCTRTTGGACGYYMRRYKCCCKRGAKTCRKGTGGYCMGGGR
45              AKWYYRWRKYAYRMKKRYYKMGRWWKAGYKYTCGRCGMYYTGSRKCTAGRYAKYAMTTTR
                ..   * ***    ***     *   ..  *   *     . . *      . *.*   *

38              TRYYMMATAATGRKTRYACCKRKYYTCKAGCTCSKWSGGCYRTGGYCYMCTWMSTRCCMK
45              CATYMMWCRGKSRGGRYGYMGAKYCCTKTTTATCGAGAATYRKRKCYYMAWAMSAGTYMK
                   ***   ...*  **.    **   *:  : .  ... **.    **.  **:   **

38              CAYGAKKTCGKYRRSYWTMYWMSTCMWYACYRYAAMCGMYRRRWYGTGCTGGATCAWGGG
45              TGYSWTTKYRTYRRCYACMTTMSCYATYMTYRYWWMYRMYRGGWTRWSYGCTCYYGTKKR
                 .*. ...  .***.*  *  **    *  ***  *  ***  *   .    .  .

38              ARTYCTAACTWWGGCTTTKRRRKKMGYWRWYCCACAAKYCRYWATTCKMYYKTTGGMKTC
45              MGCYTYWRTWWWRRACGKKAAATGMKYWRTTAAGACGKYAGYWGACTGMYYKGCTCMKCY
```

```
                   *      **  .  .*   . * ***  ......**. **.:   ****    **

38                CKARKRYGKYKYRYKTGATAGMYACRKAKRYCRTTTAYYCKRTARAYTTGCRSMKTCKYY
45                TTMRGGCTGYKYRYKARCYRKACMMRGRKRCYGYCARCTYKRYRGGCWYTMACMKCTKYY
                   . *     ******: .       *  **     :    **   .      .**  ***

38                RRGKKWRYMGRCAYKKCAYKYACYCTYGKWGAGKRGCCCATKRKAGCTAKKCYATTCKRT
45                GARGKWGYCKAATCKKTTCKYRSYTYTRKATRRKRKTTMTCKRKCCMYRKKYYMWWYKRC
                      ** *   .: ** : ** .*    *    **    : ***.    ** *    **

38                TTMGAMRCTCWKAGCSKYTGCTKRKYYCRWRGCATKKMSTSKKMAGGTCGYTTMYCTYCT
45                YYAKGCGTGTWKTAACKYWRMYKRKYYTRTRKAGKKGCCCGKKAWAKCYAYYKCCTWYMC
                      .     **:...**    ***** * * ...*  . .**  .   .* .    *

38                GCAAACKTTAATATCRRCKAAAGCGGGGGCCGTCAWYCKGRYGTRRGAAMGAAKACCGCC
45                RMCWWMGWWGRCMCTRATGTGWTTACAKTYYTGTCTCYGRGTAAAAKWRCKGCKWSYRYS
                    .      .     *   :.   . .       .       .:       ..* .   .

38                GRYTATTAATMWKRKYKSWMRRCRGYYYRGKAWMYMGWTKGYKKRYGRGCGGYRKGRTTR
45                ARTKGKWGRYMWKGTYKSWMRRMRRCCYRTGWTATMAAYGRCTKRYARCSAACRKTAYYG
                  .* ... .  *** .******* *   **      *.     .***.* ... **

38                YKAACCCCMRCGTGCGWYAGGCATTTACCATATKRGTCTCCCAYACAGCTCCCATCYMRT
45                CTTTTATTAGMRAATTACMAAAWKCWTATGAGAGGRYAKYTYGCTTTRAYYYMRYMCCGG
                   .:: .      :.     ... .  :. .:.:    ..   . : : .

38                WCYYTTCARWMKTTGTSTGYKCGRKYMAYRCATTACWKMYRGTYYGATCYRYWSGAWKMR
45                TACCYWMWRTCKACACGGATGMRRKYCGYGYRYYGYWKACRKKCCRGYYYACTGATAKCA
                   .      *  *: . .    *** .*     . **  * .   .  *   ..: *

38                YTACGGCWKCRTRKYGTYKKYGRMAKACACTGGGACYRKKYAAGTRKCACTWWWRKKGGY
45                YCGTTTYWKAAARKYKYTKKTRRMWKGYRMYSRRGTTRKKCRMTGGGTWMWATAAKKRAC
                  * .    **. :***   **  ** *.    .  .  ***               ** .

38                CCWSRKYWCCYYMMYYGYRTTCTRWMYKTGKCAGTTKTRRWWRWKWRCSRTACRKCGCKG
45                YGTGAKYWMYCTMMYYKCGWYYGRTCCKAAKYWAAAGGRGATGAKARYGAKRMGGTTGKR
                     . ***    ****       *   *:.*  .::  *     * * . .       *

38                CKKMRYGTGRGCMCGRRWCAKRMKRWMYKCMKKAAAGCCGCGARMWKRYKARWWCTTKGG
45                AKTARYKWRARMCARGRWYGKAAKRWCYKYMKKMRMRYSKYRWRMWKRYKWGTTMYYTRA
                  .*. **       .  **  .*  *** ** ***     .    *******       . .

38                YGTTRYRACCWKRKMAYYWAATGTTAGGKARRRMGTCKAMRYGYYWKKTCATMRACGTAR
45                CCCCRCGTYAWKRKCWTCARMWAWWMCRTWGGRMKYMKMMRYTCCWKKKTWWMRGMRWMG
                      *  : .****        .     .   **   * ***   ***.   **.

38                YRTATCYRYRWCYRSRRYSWKYRYAAKMMWWRKKRGMAGRCKTAATGKRYRKAKCWARKK
45                TACGCACAYRTACRSRRYGAKCRYCGKMMWWAGKRRMWRRTKATTGRKGTRKCGSWWRKK
                     . .  ** . *****. * **..*****  ** *  * *:::  *  **. .* ***

38                RTGCWCAGACYTAGTAMYYKWTWWCYYRRKKAYYMRCGAAGCTKKYRAACRCTWSRACAG
45                RYRYWTRSMGYCGCARCTTTWWWAYCYRRKKRCCMRYKTRKTWKKTAMMTGYYACGWYTT
                  *   *  .  * . :    .* *   *****   **  :    **         .   :
```

```
38      TKTGKCRGAATKYYTAWCGKYKGASTYKKTGTRAYAWYRTCAWGTRYMKKYSWYWWRKTR
45      KKYRKYRRRRCGYCYMAMRKYKKWSCYKTGAYARYWWYRWYRAACGCMKTYCTTAAAGYR
        .*  * *     *      ***  * **. .   * ***     .  **.*.       *

38      WWRYKWGWAACARYGKTMRRYRATSCKKSRWATRRTKYGCAYCGTSCASWRCYTYGMRRY
45      WAGTGTCTGTGGRYRKCCGRYRRKCYKKSRWMGAGYKYCAGTTKWSYWSWRYYCCTCGAT
        *       .: .** *   *** .. *****     ** ..    *  *** *

38      CKACWKKTCGYKCGSMTTCKGKGMMCACRATKCYRYAYYYKARCRYCTRTAKRGRTCKKY
45      YKRYTKKATAYGYRCCCGSGKTKMMTRYGWYKTYRCRYYYKCGMRTAYRKWTRRRGAKKY
         *   **: .*   .   .  . **      * **  ****.  * . *. .* * .***

38      MCWWKAWKCKYCACGTMGAGMAYRKYACTKTARSCWCACKRGMGAGGCCACSAGKAGRWK
45      AMTTGGTKTGYTGAAACTTTCTYAKTGYKKCWGGYTTGYKRRCKGAAGYWYSWRKTTGAK
             . *  * ...:  :  :* * . .*   .   . **   ...    *  *:   *

38      SGTACGGWATTCCYTMTMKCCCYRMYTTGGARYYGCCYRRKCYTAAKYWMCYSRKMGGKK
45      GTCGTRAWCYKYSYCMYCTTATTGCYCGRRGRYYRAACRRKTCYTGTTWMMCGRGCAAKK
        .  .  .*. . .* *  . .    *    .*** .. ***   :.. **  .*  ..**

38      MWRAGCTKMGYRYCYTRYKMGRRKKGTGYCAGTTACWKGGAGCGCWATTKCCAKARCMCG
45      MWRGRYCKMRTRYMCGACKCSRGTTRKKCTGRCYRMWKTRMRYSYWGCGGAMCKGRTMAA
        ***.   **  **     * .* .. .   .     **     . *.   . .*.* *..

38      CMCTRTGTYGYGGTMRKYWGYAYKKKKYRTCMKYKRYRWRMKYMRYYKRKRYRTTTTKRR
45      YAMKGCTAYRCKRKMRKYWACGTKKKKYRKMMKTKGTGWRCGTCACYKRGAYGAAAAKRR
           .   :*    .*****. .******. ** *   **      ***  * ::::***

38      WMKKWTKKCAKSKYYYRTTAGTTRKMRMKAGKSYTKAYCGYAAKTCKYYTKKYAKAGKSC
45      TMKKTAGTYTKSKYYYGWKMRKKGGMRAKRRKSCCGRYYKTMRKYYGYCKKGYMKMRKCY
         *** : . :******  .  ..  ** *  **    *     *   * .* * *  *.

38      YAGKWCRTTCTASGKGYCWTKATAMRYYRYKYRRTCTAGYTYRMKMKKYKGCTTCKGGYS
45      YGAKWMRCAYYWSRKRYYWWKGGGMGTYRYKYGGYYWWKCYCRCKAKKYKAYKYMKRRCS
        *..** * :   * * * * *. .*  *****          * * ****. .  *   *

38      TTWSAYTCTGTGCKWCYTRRKTTCCTYGCKMWMRWWSTCWRCCTTMRWKCGATRMWKYGT
45      KWTCMTGMGRAAGKWYYKRRKACTAGCAYGCTMGWWSGMWRAACCARWKTAGARMWKYCC
        .  .      :. ** *.***:  .  .    * ***  **..   *** ..:*****

38      AGTCYMRAWKCTCYATTAYRGCATSATRWWWRKYRCARCYCMMMTRRKRWTCTCRRYKAC
45      GTCYCARRAKTAYYCACMCAKYMWSRYRWTWRKYRYWRYCYAMCCGAKRWKSCMAGCKGY
        .     *  * : *.:        *  ** *****  *    *    ***..     *.

38      CCACTWTCATKWRKKMYWRKACMKCGCMGWSRRTTYRRACKKGGCRGAAATAGYWWRKRW
45      SYMMKWAATCGAGKKCCTAKGTCGAKTMATCRRKWTRGCTGGSSYRRGTCWRRCAWRKGW
        .   .*:.:    **    *.   .  *. .**.  * .   .. * .:.     *** *

38      AGGCYRAKGGKMTTMYCKWGYKKYRYTARRAGYWRRKTACKKTTTCGCYRKKCASRRYCT
45      RRKYYRRGKRKMKCCTYKTKCKKCATWRGRGKYWGAGCRMKKCCAYRMYGKTTMGGAYAC
            **    **.    *   **      *. **      **  :   * *.  .  *.
```

```
38    RWYAWGKKRRCMGSACCATGTKYYATKAAMGCTRGGCAACTRWYYRYYRGWRARRGGKMC
45    RWYMTAKKRAYARSTTYCAAKKYYRWGWRCATARKKSGGYYGWYCRYYARTAGGATATCG
      ***  .***    *:  .:..***      . :*  ...   ** ***    .   ..

38    ATTRCKTYYWRKGMYMTRRRYAKCAMYKMARYYYKWRWTMCCMAYKRATYTARYMWAGRW
45    RKKGYKACCTRKKATAYRRRYGTACCCGARGTCYGAGAKCMTMMYKGCCYKCGYCTRKAT
       ..  *:   **     ****....        *    .   * ** . *.. *

38    CAWKTKRKARWRKGRSKSKCTAACYRCKGAKTRGKGGTMRKMYATRKYAWKYCATKTTTG
45    TTATATGKWRTAKRRGGCKYYRRYYRYGSRKYGKKRCACRGMYTCGKYGWKYATWKCCCC
       : .:. * *  * *. .*     **  . *   *  : * **:  **.***.: *

38    YKKRMSTCAYARRMGATKKYRATTTGSWYKWYWWYGTMWCKRCTMMAKRRMGTGWYKCYT
45    YKGGCCWYRCMRRMRCATKYRRYCCKSTYKAYWWCKKATATGTGCCRKRRMRARTCKMYG
      **   .     *** .:.***     * ** ***  .  ..      **** :   * *

38    ATTRKGKAAKKRGGRYKTCTAKSMWYKYAWRKKGAGAGRRRKKYGCMRYYTRGTRYWGMC
45    MYYRTKKCCKKRRRGYKKYYMKGMWYKYGWRTKRGRTARRRKKTKMMRCYYRRKRYWKCT
         *. *..***   **.   *.*****.**.* . :.*****   ** * * .***

38    KTYRKTGTYKAMMKTCWKTCMRYMTTTTCGYRSKRKTRRCMKYRCSGYKKCCATTYCWRR
45    KAYRKYACTKCCAGKYWKYYMACMGCGYYRYRGKGGYAGSMKTAYCAYKKATMYWYYARG
      *:*** .  *.   . **  *  *      **.*     .**   ..***.    *  *

38    YMTAYTCYAYYRYYSRYMCRRKKMRTRKKGCCKCGGCATCWKKGTTWTCRTTKTYYKGYY
45    CAWRYWGCTCYRCCGATMYAAKKCAWRGKRYMKTTRTTGTAKKACCAWMAGAKCTYKKCY
          *   : **  .  *   **   * *   *    :   **.       :*  **  *

38    GTKMMWMMYCCTACGCTMKMTTACCCTYKTRRKRATWTAYCTCGTTAKYYYCTAGTCGYW
45    ACKAATAAYTTWTYCYYMKAKKMYYYYTTARRKGWKWCCYMYTTGCRGYYYYYTAAYRTW
      . *     *   :    ** ..      .:***  .* .*        ***  :.:   *

38    YMTCYYYWTTRYAATGGYTGKKYKKWGTWGYRYYYKCYCATAMCACKWYCWCCTARRWRR
45    YAKYYYYAGCGYRMYSRYWKKKYKKWRGTTYRYYCKYTSWWRCSMTKWTYTTTCGRRARR
      * . ***    *   . *  ******    **** *  .    .  **      .** **

38    MRWKKMRAATYMWYACWKKYCGCYYRRRYRCGYKYTCCRTMYSTMCCMCKGTACARKMYY
45    MGWKGARGTAYMWYWMTKKYYKSCTGAATGYTYKYWMMAAATCYAMYATKSKRYRAKATY
      * **  *.::****   ***  .         ***    :  .      *..    *  *

38    CCTRRCCWRCRKYMRGCRCYSYWRACKRYGGRTAKRGCGCAWYMYGWKGRCCTMAKATWG
45    MTGAATGAGMAKTAGKMAYYSYWGRYKRYRRGACGAAATTGWCMYKWKRRYYYAWTGAAR
                 *       ****   ***   :.  ..  .* ** ** *     ..:

38    RRRMYMACTAWTRRYWSKKRYKAGRCTCTARYGYYTTCKKRCYYGCKRYMKACKAYYRKA
45    AAGCTCWSGGAYRRYTCGKRYKGAAYATCGGYACYACSKKRSYYATKRTMTRSKRYYRGW
             . .  *** . ****..  :  . *. *: .***.**. ** *. .* ***

38    ASYRMRTTCGYKRCCMAARGACTTYMYCCARMYTCRKRGWKSWYTCCYRRGKCYRYRTTR
45    WSYRMRKAYRCGRMYMGGGTGMGYYCYTGCRMYKSGKRKWGCWYCTTCGATGYCRCRYGA
       *****.:    *  *..  .   * *  .***.. ** * .**          * *

38    YKKGYRRYYSCGMKGTRGCRAWGCYARKKRRKRWGMRRYKCAGCYARCRYACACRYKKKY
```

```
45              YKKKCGRYCSTKMKRYGATRTAATTCAGGRRKRTKMRRYKTGTACWAMRYWTWMRYGGKT
                ***   ** *  **   . *: .  .   ****  ***** . .    **    **  *

38              TTSKKKYYYRGARTAAYYRYCTYWRYTATKGYGKYMWGKTKWYRYWKRTMTCTCACAGGM
45              WWGGKGCCCRKRGKGRYTRYGCYTRCCGCKATTKYMWRKCKACGCAGAYMCTCGGTGRRM
                  . *    *   .. * **  * *  . *.  **** * *        *    . .  *

38              KGKKRYWYCATGMKYGRKKYRYKCTCCKTAKYYCTMMCKKWYARAGRKKYRYGGATSKSR
45              GRKKRYACAGGAAGYRGKKYRTKMAYTGYRGCYSYMMMKKWYRGWCRKKTAYTATKSKSA
                  ****  .. .  *  **** * :       *. ** ****    ***  * .:.***

38              TYYMSMKCCKAKRCYKSRYKCRAWKKATRMTKCTMKGWCMRMRYMRKAWGKKCMKYKTCG
45              YCCCSCKYTGTKRGYGGGTKYRRTKKMKRMGKGGCKKAYARMRYMRKGARKKMMKYKCTA
                    * *   :** * .  * *  ** .** *   *    *******.  ** ****  .

38              AAAKAMYKMCWATGGKTTTRKTRRYGKRYCGKTGCGRWRYKACTCMKYRRYYWARKYRGG
45              GTGTWMYTMYWRWKTGCCCAKCRRYTGGCYTKATYTATGYKCYKYATYRRYTWRRGTGKT
                .:.. **.* *         * ***      *:      **. .  .**** * *

38              TYMMCYYRCCTKRRYKRAAKCCMKYACGGAKKTMYYKGAYWSYYSRKWMTYKKRKYAYAG
45              YCCCYTCRATAKAATKRRMKMSCGYWMRRRKKKMCYTRWTWGYYSRKWAYCKKRKYCYWK
                       *. :*   **  * .  *     **.* *.   *.******   *****.*

38              KKKSCRWRYYCKYYYGKYMMTGRSWTYKTGWSYCGYYCKRTKRTGTCTYTWYGARRCGAG
45              GTGCSRARYTYGYCYKKTAMKCGCAKYKKRWSTSACTYKRYKRWRCTGCYWYKMARYRRR
                 . ..* **   * * *  *. . .**. ** ..   ** **       **   *

38              KKCRKGGKTTCKKYMRKYGRYKGKKKWGKMYRTYRRRAKTKAKRAGATKMRTCCCCGRYY
45              KKYGKATKCCYKKTMRKTKGTKAGKKAATCYRYTAAAWKWKRTRGKCGKCAWYGYTARYY
                **  *. *   ** ***    *. ** .. **      * * .*. . *       .***

38              RKRRRRYSYMCAKKTMRAAGGAYSAATGKYTRKRTATRCTYACTGGAACRMYRWKAAGGK
45              RKGRRGYSYMYGKKYMRMGSKWCCWRWRKYCRGGKCAGMWCCYCRRRWYRMYGAGTGTAG
                ** ** **** .** ** ..   .    ** *  ..:    .       ***   :. .

38              RGARTYTGRMRYYKRCTKTAYCTKSYYKRRCATCGKMYCSMKTCGKRRTGKRKCGMYYKA
45              RRRAWYYRRMRYYKRYYKWRYYWKSCYKAGACWTKGCYYSMKKYRKARCAKRKTAATYKC
                *    *  *******  *  *  ** **  ..     * ***.  * * .*** .  **.

38              ACGTTCGARYKSKTKMRRRKRCAAKCGTSYTTTKRGGTKAAKYCTAYYRYYMCWWTYKCG
45              GTAYYYKWRCKGKYKCRRRKRYRMKSRAGYYYYGGTRKKWMKYYYRYYGTTCYWWYYGMR
                . .     * *.* * *****   *. :.*       .*  **   **     ** *

38              ACTKCKYCCTGCGRACKTTWAMYRMTYKAKKRARACGKWKKGRRKKRYYMRMRKMAKMGC
45              WTWKYTYTACAAAGTYGGGTGMYRMWTKRKGRTGGTSKWKTRRRKKRCCMRCAKAWTAST
                   * .* . ... :     .****  * * *: . .***. *****  **  *  . .

38              GGSRKTWMTCGGYKKRWRTTRTAARYRWRTGRYTCYRKYTCCCATKYSRRKMWWRKCAWK
45              AASGGKTAYMRRYKKGWGWWRWGCRYRAGWKRYGATRKYYYYMWGKYSRRKMTTRKTGTK
                ..*  .      *** *   * ..***    ** . ***      *******  ** . *

38              AKACTACTKWRKAKRKYKKYKYGKGKATRYSCRKKCTCATCTRKKTRGSTYWTAKGGTYR
45              TKCACGMKKWGGRKGKYGKTKYKKAKGKATCYRKKYCYCYYKRKKGRKSACAWMGATACG
```

```
        :*.. . .**   * ** * ** *.*..  . ***   .  .*** * *:     . :

38      KRAYRKWRTTGGRYTKCRATGCMYKKRGTGYGTTTTCWGKTWYWACCKRRRKAYRMWYYY
45      KRGYRKWAYYRKRCCTYACARMACKGARKRYKCYYKMTKKKWYWRYYKGGRKWTAMTYYY
        **.****     *  .  .:    *   . *    .   *.***   *  **   * ***

38      KYCGTRWMYMAKAYYYCAAGYKYTAMKRGKYRARAWCKCCRKTRAYRYCMRWRTGYWYYM
45      KTMKYGTCYMRKRTYCACRRCGCACMKASKTARATTTTTTGKWRGYRYGMRWGYRYWYCC
        *       ** *  * ..     :.** .*    :  .   * *.*** ***   ***

38      MKYTYKWRWYRRYAMTRRGCGRWGCRKRMKAKTTATATWRSYKWYYGWCRWKMWYCTYRG
45      CKCWCGTATTGACTMWRGRTRGARTATGAGWKGAGCGAARGYTWYYKWTAAKMTYYYYRR
         *           :* *         .    * :. .: *.*.*** *   ** *  **

38      RTYGRACTKYYCRRWRKAAYWGYKTMKAGKRGYCYAGGYTTGKCAKKAAAKTYCGYYMTT
45      RATKARYYGTCYARWRTTTYWKYKCCKCTKRACMYCARCAKRKMTKTWGCKCTMRCCMGA
        *:           ***.::** **  *. **.  *..  :. * :*. ..*      * :

38      CKMWKKSYRKKKKYTGGKCMCGGGSYWRCRMYTTKAAMKTKTAACRRMYYYYRKYSAAWM
45      TKMWKKCCAKKKKCYSSKAMYRKTSYWRTGACKYKWGAKKKYRWMRRMYYYYRKYGMRTM
         *****.  ****  ..*.*    ****    . * . *.*    **********.   *

38      SRCTRYYYYYYYSRKRKRMARWYRYWWKKKCCAAGAWKMTWRTWKKYGWTTYKKCTAARK
45      CAYAGTYYYYYYSRKRKRMWATTGCATKKKTTMRRMTKCYWRYWKKYRTWKCKKTKGCRK
        .  :  *************        ***       *  ** ****   . ** ...**

38      CWCGRCKKGCCYYYRKSCAYAAMGKYYYAMYKKKYYKYTCMTCKGRGKYCYGTTRGWMAY
45      TTMARYKKATMYYCRKCTWTWGMAKYYYMMYKKKCCKTYAMKYKKRRGTTTRYWGTWMGT
           .* **.  ** **.    .*.**** *****  *  .*. * *          **.

38      YKRYMWAATGYWWGCYKRYCAYGYRYCSGYTRCMCWYMRSYWKKYATYTRWRRKYCCCTC
45      YKRYAWGCCTCWWATCGGYMTCAYRYSGAYYRYMYWCMRSYWKGCTCCCATRRKYTATCT
        **** *..   **.    * : .***...* * * * ******  :     **** .

38      YKRKMMCGKMRYWWKKWWSYKYYRSMTGMTTAGYCGATMMCGKCTRCGTTTYRKKKGGGW
45      CTGKCMYRKMRYAWKKAAGTKYYRSMWRMWAGRYYKMCMMTAKMYRTTCCCCRKKTKKRA
         . * *  **** ***  . ******  * :. *    ** .*  *      ***.

38      GMTRKTGGTAACGMRRGTYRGRKKWRYKYCRWMWYMTGTARARAYYWCKTAGRSYGCYYA
45      RCYGKCTACWRTAMRRACCRKAKKARYKYTATMWTCCACGRRRTCTASGWRAACCRSYTW
            *  .    .***.  *  ** ****   **   . .* *:   .   . .  .*

38      GRWKKMYCYYYWKRGTGSCTKGAKGGCCYRKCGCWGAARGRGWYMAYKYKGGKTTGCCYC
45      RRTTKMYMCTCAKGKYSGMWGRMGKSATYRKAKYTRWGAKRKWYMGYKYKKSKYYKTATA
         * .***     *   ..       .. ***.     .  * ***.**** .*    . .

38      TCTGGGCRYRRCGTTTGYKGCATCKGRYATYGWGTYRYACKCACACAAYTARCYMTRYWS
45      CTARRRYGCGAMKKYYKYKTTTWMKAGCRKYKWKKYRYWMKYGMRYMRTYMAMYMCGYAC
          :          .   **  :  *.   .* * .***  * .          **  * .

38      KSKKWSKYYYWRGWKCCRWMGGTTGMMRWKRYKGAGGKKAKRTCCCCTRCRAAKYMRYCK
45      KSKKWSKYTYWGKTKYYATCATAYSACRTKAYKTGARKKCKRGTSTTYRMGWRKYMGYMK
        ******** **   *     . : .  * * ** .. **.**  .   *    *** * *
```

```
38              KKYKKWMMKKACRKCRAYCKAGAACRTGGCMKWKYRKRCGCKCYTGYKKTYYSYYYTARM
45              KKCGGWACGKRMGKYGCYYKTAWGYRCTATMKWKYGKGYRYGMYAAYTKGTTGCCYKMGC
                **   *   *   *  .* *:. . *  . ***** *      *:.*.*   .  *.

38              KKKKRYGRKKTRMYYKGKWGGWWGRRWKCACGTMWWTTATYYYRKGYCMTCRYKKKMRRK
45              KKKTRCRRKKKRMYYKAKASAWWAGATKYRYTACAAYCWYCCYGKRYSMWSRYGKKCRGT
                ***.*  ***.*****.* ..**.   *    :         * * *.* .** ** * .

38              YGRTCAARKKMMKGAAKYYWKRYCKRYWWGAAKCCGYMRKRATMGTGRMKGKRGYRKRCA
45              CRRWYRWGKKCMKTTTTYYWKGYYGACTTTWWKTTTYMRGAWYCRKRGAKTKRAYRKRYG
                  *     ** ** ::.**** *         *   ***      .   * **.**** .

38              ATGWCCMCMMRGMKGCTYAARAKYGCCCWRKRWAYRYYATKKKKKAAAKMACCKCGWRKR
45              GCKAMMASMMRKMKATGYRRRMKCKYMMTAKAWGYAYYRAKGTKKWRRTARMYKYKWRKR
                .      .*** **.  *  * *       * *.* ** :* .**   .    *  ****

38              KAYACYTAKMWGYKSRGYGCYKKWWGTACAGTRKYKKCGGAYYYAYGWTRTAAYKAMYWR
45              KGCTTCATKMWRCKGARYRYYKGAAACCYWRCGKTKTSSRMYTYRTTTWGWRTCKCATWG
                *. :  ::***  *.  *  **   . .     * *...  * *        : *.  *

38              CCK
45              YYK
                  *
```

The pairwise alignments of two accessions 38 and 46

```
38    YMKTTRKWMYRKWWWSMWYMRRKYWRYRYGTKRGYRCRCCGYRKYYYYYRRGGAYMWSRM
46    YMKYYRKAAYRKWWTCCWYAAGGYWRTGYCCKGRYRYRTTTYRKYYYYYAGTAGCCWSGM
      ***  **  ***** . **    ***  *  *  ** *   ********   ..  ** *

38    MRMWRKYRRRYMKWRRAYTRMKKYYSRRMAMYMYYMKRKKRSGSYYRKWWRMYMRMRRST
46    CRAAAGYRRRYMKTRGRYYRMKGTCSRRAWCTMYYMKAGKRCKCYTGKTWRACAAAARCW
       *    ******* *  * ***   ***    *****  **. .*  * **      *.

38    RKRRACARSMMRRGCYRKSYCTRYKTRYMRSYTKGGGKAAMYMYWYRRYRWYYWYRAYCK
46    RGGGRTTRSMMRGRYTGKSYMWGYKKATCGGCWKTRAKGMMYCCAYRRYRWYYWYRTTYK
      *     :*****     ***   **.    .  *  .*. **   ***********:  *

38    MYCCTAACRMYTKWMRRRWRYRCMAAYYRMKSYMKSYRKRYYSYTCYGAKYRAYGRRKGR
46    MCMMYMRGRACWKWMRRRAACAMMRRCCRATGTMKSYRGGYYSTCAYCRTCGCTKGATCR
      *       *   ******     *    * .. *****  ***  .*  .  .    . *

38    RRSYWAYKTGYRMRTRSRWWMMRWTRRTGTYMCAYYRMYCGTTATCTTCYYSRYYYWTYR
46    RRSYACYKCAYGCGWRCGTTMMRWWRAAACYMYWYYRMTMKAGRWMWAACYGRYYYWCYR
      **** .** .*    *.   **** * :. **  ****   :     :. *.***** **

38    RKRSYYWKYYYYMYCMACYKRMRGCRCYAARRSYKRTWMWTRYRWWAMWTCRCGSYWWGR
46    RKRSYYWKCTCTMYYMRYTTRMRKMRTCGMARSTGGYACWKGYRWWGATWSRMRSYWWTA
      ********    ** *   .***  *  .  **      *. ****.   .*  ****

38    YRWKYRCWRRWYARWSRKYYTWRYYYMKRRYGYRKCGMRYMMRCRYSMSWTCRYYRMRGK
46    TGTKYGYWGAWCCRWSRKYTAWRYYYMGARYKYRKAACATCCRSRYCMSWCMRCTRMRKG
         **  *  * .****** :******  ** ***..     *.**.***  *  ***

38    ARRRYWGYCGAYKTYRCTMRRYCTCYCGRKYCRAWTYAAGYWYMRYMKWRRRWYYYRCSY
46    RAGGCWKCYRMYGWYRSYMRRYACTYYKRKCYRWWCTTCRYWYMRYMKWRRRTCCYGTSY
           *     *  **. ****.  *  **  * *  :. ************   *  **

38    RTCMRYRYKMYRYMSCYYRRCYKYGYASATYTKYKKKTYGGYYKYTGRKRKTGTTAKTAC
46    RKYAGYACKMCAYMSYYYRRSCKYKCGGCCYGGTTKKYCRKYYKYGTGGAGCAWYTGKWY
      *.   *  **  *** ****. **  ... *   .**    ****       .  : .

38    TCARKKKTTAGMCRTTGAYMRKRAKTCRKGATRTMGYSGKYMATRRTKYTARYSRTAGCT
46    GTTGKKGGCGAAMRWWKGTCGKRRKCTAGRRYRCCTYSRGCMWKRRWKYGWRYGGKTTTW
        : **   ..  *   .   ** *       *   **   * .** **  **. .:

38    CCCYCTACACSWSGKYRKRATGWAYGRMRKRMGYYGGYCYRTATMKCTGGCGSTTATYGC
46    YYATMCCTMYCAGAKYATGRAATRTRACRKRMTYYRKCYCAATCMKAWAAYASWGCYYRY
        .   .   . ..** .  :.      **** **      :: **. .. .*  . *

38    RGYRKKGRAKKTKTKACWTTTMWCRTTWCYYTACTAAAMWCTAYCKMCCGYRKRRCKCGT
46    GRYRKTRGTKTKKWTMYAYYCMWYRKKWGYTWGYCRRRATTAGYAGMYYRYRKGGYKTTC
        ***.  :*..* .      ** *..* *  .        :.*. *   ***   *

38    RMRWAYYMYRTCAATTTCMYYGARYRCTWWGYTRRRKGGGAAYKYGMGAMYYRKAWKRYK
46    AAATRYYMYRYYRRKWCTMCTRMRYRTWWWRYKAAATTAAGRYKYKAACAYCRKGWKRYK
```

```
            *****    .   *    ***  ** *.   . ... ***  .. * **.*****

38          YSRRYRKKCTRRKWWYMKWYTGKSWYTGRKRYYWRRTRRGKYGAKTKCRAATKRTTYYWY
46          TCGRYRKKYKRRKAWTMKWYCKKSAYAKGKRYYWRAYARKKCRRKYTYAMCAGACYYYTC
             . ***** .*** * ****  ** *:  ******   * *   * .   .:    **

38          GTKWYGGGACAWACCATARRAYRMWGRWYKYCRWMMAATRKAYAKYCARRAGYKKYTAMK
46          RKGWYKTTWMWWRYYGCWGRWTAMTKGTCKTMGTCMRRKAKRTWKCACRRRRCKGCACMK
             . **      *   .   *   *     *     *  . *   * ..**   *  :.**

38          RGSRRWKCAGTCTKKTARYRYCMRWCCYWYYTRMMKGAWATRRRWYRRYAKAMKRRGSRR
46          ASGAAWTYWRYTYKKWRGTRYGMRWSYYWYCAGCCKKGWCWRRATTGACTTWCKAGRSRR
             ..  *.      **    ** ***. *** :   * .*. **      :.  *   ***

38          GRTTRTKRMGKMKTTATRRSYRWGAATYRKMWTAWYRCCAYKYRTRRGCYKCYKARKYYC
46          RACKRCTGAAGMGGAGCRRGYAATGCCYRKMTCGTTRYYRTTYRARRKSYGYCKRAGTCT
               .* .  . *  :. **.*   .. ****  .  *    .**:** .*   *

38          AKKKMCKKRGMAGKRWMRYGAARCGKRCKYRWYAGTTRMMTARYKRTATGGAGAAMTCTC
46          TKKKMYGKRRMWRKRTAACSWRRYRKGTGTRATGAGGRCAYTRYKRCWYRRRSMRCYYKY
            :****  ** *  **    .  *  *    *  ..  *   :****      .     .

38          YYKTYKKTRKWMKMAGRAAGRKWSRYRKGGGGTGYRYYYKTGCRGAACCYTAWCKYWRYK
46          TTTWCGGKRKWMKMTRRMMAGTWSRCGGTRRRGAYRYYYKYRYRRRRSMYYMAMKTAAYK
              .    .******: *  . .***        .******   *   . *    *   **

38          YTGKAAGWGAASWAWTCAWWTRYMMWAYMYSMWYYKTRWRYGYYMRTCAACTTSWKWTRT
46          YGAKGWKARTGSWRWATGWACRCMMWRYMYCCTTTGYRWRYRYYARCYTGYYKCWGWGAA
            * .*.    :.** *: .*  * *** ***.      **** ** *  :.  ..* *  :

38          RKRWTKGMAATKWKSMMYGARCYSGRYRCYRRRCKACWAKKWYKSMCWKMMRATCYRYYY
46          RKRTYKCMWRWTTTSCAYRRRYYSKRYGMCGARYKRYTRKKWYGCCSWKMMGWYTYRCYY
            ***  * *   . .*  *  * ** **     * *    **** . .****    ** **

38          YAYTCCKYKKKTAYYKYCYMMKKWYAYRKRYRYAGCGGRGRRSAKKKSKRCRYMSAGTMK
46          YTTYYYKTKKGYRYYGCYYMMKKWYRYRKRTAYRKTARATGGSMKKKSKAYACCSRRACK
            *:    * **   **   ******* ****  *   .     * *****     *  : *

38          CMACKATKRYMYSRYYKAYCRAWTAKKKGGGTTRTRTTCTMWRRMKRMRYMRKAKCGYTC
46          YMWTGTKKRCMYSRYYKCCYGRTKRKKKRRRKYAKRCAGGCWRRMKRMRYMRKCKMKYCT
             *   :.** *******.     . ***   .  .* :   ************.*  *

38          GMYKYKRKTCGTRRYKAGMRKRKKATAARKAGMKYRRYMKKCYRCYCMMRKCTRRYMGRM
46          AMYTYGRTWYKCRRYGGRAAKAKKGCGGGGRRATYRRTAGKYTGYYYMMRKTAAATMRRC
            .**.* *.    *** .   * **. ..     .***   *    * **** :   * *

38          KCYMYKRRCYKMATTYYKKKSWYSRKWGCKYYGCTGKKMGRRWYMCKGMYYKATTTMWKY
46          KSYCYKRRYTKMCCCCYKKKGAYSRKWRYKTCRMYRKGMSRRWYATKAATTKCYYKMWKY
            *.* ****  **.   ****. *****  *      * *.****  *.   *.  .****

38          WKSTRMGRRYMAAAAYCGSYTTTYCKTCKTYWGGCTTCTYKYMTGYYMYWWWTKCKRCKR
46          WKGYGCKRRYCRMWRYSRGYYYYCATYYKKYWSSYKWYYCKTCCACYMYWWWKKTKRYTA
            **.    ***     *. .*    ..  *.**.. .    *   . ******.* ** .
```

```
38          KGAGCARCACMRMYKKRKTACAYAGRGKTAKKWKTTWKAYKYMRKKKMACYMRGKAARYR
46          GRTATCGATYMRMTTKGKYGTRCMSRRKAGKKWGKKAKGCKCMRKKKAWTYMGRGRMRYR
              :. . .: *** .* * .    .* *:.*** .. *. * *****   **     ***

38          WTRTCRTCYTCRMKYYGGGCKWKWWRCACWKRAKAKAKRSCWTCACKARKKKRKCGCCAC
46          AGGWYGGAYYYRMKYCKAAAKWKTTRTGMTKRRKWKGGGCMWYYMTTMGKKKAKMKTTGT
                   .*  ****  ...***  * .  ** * *.  . *    .  *** *    .

38          TSTWGGYWSTCKKYYYWCYKTRTTWRRCTGCYTYRCRCYKWKCSRYWCGCMTKKYCCCGC
46          ACGWRACTGYMKKYCTTMCTYGWYTRRYCAYYWCRYGTCKAKYGAYWTTGMYKKCYYYRA
            :. * .  .  ***     .     **  . *  *    * * . **   * **     .

38          RGYMTCCCCRGGRKKKRMKRWGGRYKKTMKTARCGTGGYCKKKKMAARGGWWCTTKCAGG
46          ARYMGTTTARATRKKKAAKRWKKRYKKWAKCGGYKCRTTYKTGGMWWGKKTTMYYTYRRA
              **    .*. ****  ***  ****  * .        *.  *          .   .

38          TRACWAKKRMKWYRKMAGRASGKYYGKRCTTTCARRTKKSMYCMSGMYRYYWYKACTMMA
46          WGTYWCKKRMKACAKMMKRGSTKCCRGGYYCCTGGGYKKSMYMMSTMYRCCWYKTYWMMG
              : *.*****   **  *.* *          .   ***** ** ***  ***:  **.

38          CGTAMRYRYKKTATCWYCRGYYMYRWRCYRKGGAKSRRYKSKYRYMKAAKAAATRYKRYC
46          MRWMMRYRYGKCGCATCAAACTAYRTGACRKKKWGSRRYKSKCRYCGGGKGGGYGCTRYT
                ***** * . .  . .   **  . **    ******* **  ..*...   .**

38          CATCTMTACTYYRMTGCGARSTKMTTYKTATKRGTYACTCCAKGCKTCCCCAGTTTYRWM
46          TGCTCCKMMCYYRMAATRMRCCKCKAYKAGAGGRYCRYYTARKRATCAMTMRRYWGCGTC
             .    .   ****:.   *. * .:**:.:         . * .. .

38          GTGSGYKCCMCYRYRSRACGWKGRMYAYCAKRCMRRYWYSRKKTCGRCAARKSTTCWKCY
46          KYRGATGMMCTYRYGGGRYTWTKRACGCYGKRSCGACTYGGGGWMRRTGMAGCCGYWKAC
               ..      *** .    *. *  .  .**.     *.      *  .   .   **.

38          YAWYTRAYWARMMYAKKYYCYWRAGAACGAGGAAGKYTRMTCRWYATWCRGTRTYGGCGT
46          TGTYWGRYWWRMMCWKGCCYYWRRKRGTAGATRMTGCKRMGYRACTWWTRRYGKTCCTTG
             . *   ** ***  *    ***   .  ...     .**  *  : * *   .

38          TGKKACKYCGYTGAGCYYYYTGTYTKWCTMGAGCTAYYARCTYMMCARCKWYWYGRCTAR
46          CKKKRYGYYRTCRCAYYYYYWKYYKKWYKCRWKYYRYYTRYYTMMSMRTKWTTTAATCGR
              **   *     .. ****   *.** .       **:*   **. * **   .   .*

38          RRRRKRMYMRKMASYMWYWTCCKCGCYYYRRYRRYRWGRRYKGATAATCCRATSGKMKGR
46          RRGGKRMTCRGAGSYMWYTAYYKYKSCCTGGCRRYRATRRYKTGYWWWMMRWACAKCKSA
            **  ***  *  .***** :  *  .      ****  **** .      * :..* *.

38          KMYGCCTCRTCCGRCRKAYMTRTTRATGGTRMCASYWRRRAACGKKGKRWAGTYKGRYGG
46          KMYKMTGTRCTGKGMAKWTAGGCAGWGAAGAMTTSYWRAGTGAAKKSKRWTSYYKRRYRK
            ***     *       *      :   ..  * :****  :...**.***:. ** **

38          KRGCGCWSYWKGCGGYMAYGRRRMYYKYRWRWSKRMKRYGCAGRCTAYKTTKCYYRKATA
46          GAAATTWSYWKATARYATYRAAGCYTGCGARTCGRMKRYAYGAAYATYGYYKSYYRKMGR
              ..  *****. . * :*     *     * . *****. ..  ::*   *.****
```

```
38        CKYGCYWGWWCTGKRCCTRWACACMGYYWKKWWKGYCRKKKGTCAGAKKMRRRKTKYTCK
46        SKYTMYAAWWYWKKGTTAATWYRYMKYCTGKAAKRYSGTGKRWSRKRGKCGGAGKTCWYK
          .**  * .**   *   :      * *   *  * *. . *  .    *       ..  *

38        AAYRGKMKCCAAMKAAKKYYWRRWYCAGYAYMCCTRRATYKGGAGATYGRCGGWGCMMRG
46        RWTGKKCKTTCWMKTTTKYYWRRWYYMRYWYMYYYARWYTTKTGACCCARMAAATSMMRK
               * *  . **::.********   * **    *   .  ...  .* ..  .***

38        MAKKAARKYCCCWWRKRWYGAKATGGKRGKMACGRMWRCCKCCKAWCTMGCAWYYGYWCK
46        MRKKRRRKCYMMTTAKAWYTGKGGTRTRKTAMYKGMWRYTKATKGWGYMRMRWYTRCWYK
          * **  **        * ** .*.   .* .     ***  *. *.*  *   **   * *

38        SRYTGGCGYSYCACKGAYRKMYKYTGGCKAAGAGCWRYYRAWGWRKACYSRYYYYMYRGG
46        GACYRRYKYGTYMYKRGTGKCTKYWRRSGWRRWRYWGTYRWWKWGKTYCSRYYYYATGSS
          .       *.    * .  *  **    .       *  ** * * *:  ******   ..

38        AKRKTTCAKTCKWRRKMYSRKCWMKYAKTRCAWACMRCWKWRSAACWCTRRGGCMWAYAA
46        WKRKKYYWKWMTWRRKCCCAGTTCGTGGCGTRTWTMAAAGAGGTGYTYKRRRATCWGYGG
           ***.   *  .****   .       .        * .    .:.   .** .  *.*..

38        GCCACSAGGACCYYGGGGCMKRARKGTACKCCGYCCMRMTKMKYRCTGCYKYGAARRWGA
46        ATYWYSWKKRYSYYTKKRYMGGGGGTCGTGSMAYYSCRMCKMTTGYYRTCKCKTGRAWAG
          .    *     .**     *  .     .  . .* . ** **.       *  :.* *..

38        TGGAKYACKYTMYRYRYWKWRRYWYACAAGAGCCMGWYRTYKYCGYTCRKMGTRRKCCTA
46        ACRGKYRYKTKMTRYRYWKTAGCWTTTGCTGKYYMRTTRWCKYMTTAAGKCSYRGTTTGT
          :  .**  * .* ******    * : .. .   *   *  **   :. * . * .   :

38        KGGGGGYCCTWAMKGACCCMYAAYCYATATGAMCAWGRKAKGGCTKCATAYTCKKCWRAY
46        KRRRRKCMYKWRMKRMSTACYGRCMCWWCCRCMAMTARKMTKRYKKYRWWYYMKGYAGCC
          *        .* **  . . *.      .  .*.  .** .    .*    *  *    .

38        WAAGCTRGMKWYATYRYYRCWGWRARGGKYRMCTCKGAYRRYKTYGAGCTRAMRRCSKRY
46        TWRATAAAMKWYRWCRYYAMWRTAGRKRTYRCGWYKTRCRRTKYTAGKYKRWMGGYCKGT
             . : .****   ***  *   .*  .**     *   ** *   ..  .* *   .*

38        YTAAKWRWGAAMYAYARMATATWAGKGGRWKRKKCGTRSYSKWKCYRKKRTTTTGCKTCA
46        CCGTGAGASMRCYMYCGCMYRYTRKTRRGTKRKKTRKRGYCKWKYYRGGRWKKGTMKYYR
            .:    .   * *.         .    ****  .*.*.*** **  * ..   *

38        TRMYYMWTKASAWTGTRYSMTRTATCKKYWKKCYACCMKRGTTTTGYWWSGCATTYRCRC
46        YRMYYMTCTGSMWYSYGTGCKAKCGTKKYWKKYYRTTCKRACCYCCYWWSKMMWWYRTAM
           *****  ..* * .   . . ..  ****** *    **.     ****     **

38        CTCATGWYRRRCYWYWWGGACCGKYGTGKKARRMTMTTSTGTTTWCGSYTCTKTKKCYKM
46        YYYRYKTCGGGTYAYWWAKGTYSKCKKRKKRRRMYAAKSWKKKWTYRCYGMYKGTKYYKM
                      * ***. .  .*  . ** ***  :.*  ..    .*   * .* ***

38        SYKGATGTAWWSWTGTTRRWKCMYKRMWKCTGCYSRWYTKYCAKYMWKYCRKTCCTMYWY
46        SCGRMCAATWWSWWKCYRRWKTMYKRMWKTCAMYSRWYAGYTTKCMAKTYGKAYYGATTY
          *      .::****    **** *******   . *****: * :* * *   *:      *

38        ATTKCKSATRTKGWWTRKYTAAARKWMKMTRCRRSSGGCTRCTYTGTCYTWMAWRKRAGK
```

```
46              CACKTKSRYRWKRTWWRKYYWRRGGTMGCCGSARSSRRMCGTGCWRKYCYWMMWRGRGRK
                .: * **  * *  * ***       *    . ***          .   ** ** *. *

38              KRMKACMKRYMYRWRRYRYCAGGCGCCYWSRTTYCTCCMRKGTYGACKTKKCRCRYTAGY
46              KAMGGTCGRYMYRWRRYRYTTRATATGTWSRKWTAAGYCRKRYYRRYGCKGYRYGYWRRC
                * * .   *********** : . .   ***.  .:   **  *     *  *  *

38              YKRRKYRAAARKYCGTCGKMKAMYRCWYRGKKKRRRRAAACTTTKYTSKSRRAMCTWTGT
46              TGRRKYRMTCRKYMTGAKKMKGCTRYTCGRKKKARGRRGRMCCAKCGGTGGAMCACWKKY
                  ***** :.***   . ***.  *     *** * * .    :*  ...    . *.

38              RYYYSWYMYYYYKGARYGKYWYKAGTWCRTGAGCYKCAWKAAGTTCATTYCYYAMTATTG
46              RYTYSWYMYYYCGTGATKKCWYTCKGWGRKACAYYKMTWKWGCWYYMCCTMCCWAAGCCK
                ** ********   .   * **..  * *.... ** :** .             :.

38              MKMYWKRKKRKWKKKCTGTTTGGGKCGACTGTRCTSYWRMCYTGGCKTKTKCYKYGACKT
46              MKMYWGAKKGGAKKKAWRYYWRKKTTATACRYGYWSYWRAYCCTTGKKKKKACGYAWTGY
                *****  **   ***.        . .:.      ****       *.*.*.  *.

38              KKMRAAWMGGRKKTYGTCCTTYRRMAKRYWCMKTRSCTYYSRKYKRRKYRRMTRRMTCAW
46              KKMRWGWARARKKYTACYYYWCRRMRGRYTYMKGACYWYYSRKTTAAKTGGCAARCCTWT
                **** .*  .***  .      ***  **  **  .  ***** .  *    : *

38              CAGRWKKKCCGRTGCTTKAWKMYWRTWKKKKCCRTWKCGGRRGTRYWKKWWSYRYKWMAY
46              TWKGTKGKTTKAATGCGTMTKCCWRCWKGKKTGRCTKMACRRSWGCWKKAAGYGTKAAWT
                     * *    :    .  *  ** ** **  *  * . **.   ***  .*  *

38              YRTATTGATAWTCGTYSMAYKGMRTASTYKRGCGTMTRMMMYGRGAYCTGTGYKKKKGGA
46              CGYMCAAGCMWATCATGMRCKKMRWMGAYKRRYTCMWRMMCCRGRGYTYRCKCKKGTTTR
                     :..  *:  : .*  * **  .:***    * ***     .*      ** .

38              GRYGTKCGRCAYRWKRSWKYYWYRRYKYRKRWYMKTGTAKRMKCTGCCRRGGSTGGCGKA
46              RGTACKYRRYMCRWKASWKTYAYRRYKYRKAWTMKYRYGKRMGYKSYYRARRCWASYKKW
                   . *  *   *** *** * ******** * **   .***  ..  *   . ..  *

38              SRMCTAWKKMAAMTGCYYYWKGRCAMGSTTGWKCKWGYYKCTKTKARWTGTACTRAGGMK
46              CAAMKWTTKMRGCATMCTCAGRGYMAKSGATWKTGACYTKAGKKKRRWYRYMSCRWKRMK
                .   .  .** . :             * : **    * *. *.* **    . *   **

38              WYMRTAAGAYKYKGCCCAGYRGTCAYTTTGGGYGWYKRYRYRSYMYTCRRCGRMKGYATG
46              WYMACGRKGYKYKKMTARTCGKKMWYAAATAKYKTTKGYRYRSYMYAMGRTAGMKTTMAT
                ***  .  .****   .     .  *::: . *   * ********:  * . **   :

38              KGCKRRMYYYKYRKYWRCTTTMGRRKYWMRKAKAYYTRTARCSMGCAYYAKYKYYKGAAA
46              GTSKGRMYYYTYRKYWRTCKWCKRRKYWCAGRKRCTYGYRGMCMSYGTYWKYKYYKATRR
                  .* *****.******  .   *****    *         .*. . * ******.:

38              AGGRTGMRACYWWYCYAAWRKYAATTKTAAKATARYKGCGCAGYYGTRKRRRWGYSKMWT
46              WKRGWAMAWYCWWCMYMRWRGYWMYYTACCKCGGAYKKGATGRYYRCGKRRRAAYGKMWY
                     .*    **  *  ** *    .:..*. . **  . . **   **** .*.***

38              GAAACTRKCGYRRRRTTTYRMRYYCATGSAGARGTRYWYTKCYRGKYCATCAGMMKWSKM
46              KWRWYYRTYRYRRRGACATRMRCYMRYKGGKTRRKRYWYCGTYRRKTYMYYCACAKWSKC
```

```
                  *.  **** : : *** *    .. :* .****   ** *     ..  ****

38    CKTCTCACKMTCAMSKKMMKRRCMTYRRKCSGWYKKCCWATTYYCRRRYMTKYRKYYTYS
46    TGYYAAGYKMCTGMGKKAAKAGSMKTARKYCAWYKKATWMYWYYYRRGCAWKYRKCYKCG
          :.. **  .*.**  *  .*.  ** ..****. *   ** **   **** *. .

38    RSRMYYKRMCRCRKRYKKCATCYKKTRTTAGCACGTTAKKAMMWMMTYATGCTAAMAMRY
46    GGRCYYKRMYAMAKRYGTATGTTGGGAGAGAYGAAYYWKKMAMTMMYYRKCYYRRMWAGC
       .* *****    *** ..:         :.. ...   **  * ** * .     *

38    CCTYKGGTYYYGAKWYGATYTWGGCCKYCACCKAKMRCTCYRRTRCMMYYAYRYYYRKCT
46    YYYTTARWCYTRRKACKGCYWWRKYMGTYWMYKTKCGTGTCRRWRMMMYYGCGCCCRKYY
          ..   *   *   . * *         *:*      ** * ****.     **

38    TTAKMAGATGTGTTTKWACKTTRMATGTRMWARRCAGTCAGYKYARRGAAWAMMTRMTTA
46    GGGKAWACCACKYKWGACMGWWRMWKRKRCARRATTRWTCRCKCCAAKGGARMCGGAKKM
        .*  .. .   .   .    ** . .*   *  :   .  * .   ..  *    ..

38    AYGYKRAKWKKAKWMMRWRKCCGKTCGYYCYGAAAWMYWAYKKYYKYCGKCYCYRRRWRS
46    RYAYKRMGWKTRKWMMRWRKMYRGCTAYCGCTRTWTATAMCTKYCKYYRKYYYCGRRWRC
       *.***  **. ********       .*     :       .** **  * *   ****.

38    CRGMTAYSAGRGAMYKMYGKYKYTWCTCGKYGGYGTTRKGWMAYKTTGAAWKKRKYKYKA
46    TRAAKWYSRARRMMYKCCRKCTTYWAKYSKYKRTRYWGKTWMGYKYYSRRWKKRKCGYKM
       *. . ** .*  ***   *  .  *.. .**       * **.**  .  *****  **

38    ASGKRTTCSSACMCCYRCMYMRSYWYSCKYWRYRWSRAKKYTAYKMAKCMTAKRGTRTTY
46    WSKKRYAYSSGYMAYCRTMYARSYWYSYGCTRYGWSRMKKYCCYKCRKMMKRKGTKGCYY
       * ** : **. *.  * ** ******    ** *** *** .**   * *. *   .   *

38    KRYRMYKCYRRCRKGKTATKWYCRKKATTCTYARYGYMWKTKYRCKAGTMKKGYYRCACR
46    KGYRMYKTCGRYRKRGCGAGWYYAKKWYYMCCGACRTCAKYKYAAKGTCAKKKCCGGGTG
      * *****   * **   .: **  **       .      * ** .*.   **     .

38    KRKYSCWKYRRAAKKKTKRTTRRGGMRRYKYWKRMCAYCCRYKYCKKKYYYKGAGCGRMT
46    GRKCSTAKCGGGGGGGYKRCCRARKMRRYKCWKAMYWYYMRYGTGGKGCCCKKRATAGMK
       ** *  *    ..    **  *   ***** ** *  *  **     *    *  . . *.

38    GAYRYRRCYWMTAKTKWRWRKKTMTGCYRATYWKRMRTCCTYAAAMGCKRRWYCWYCATG
46    ARYRYRRSYTCWWKWKWRTGKKCCYATCACCYWKAMGCTACTGRGMKYKRRWYYACAGGA
      . *****.*    * ***  **    .   . *** *    .  . .*  *****   .. .

38    MCCCYYYGACMRKRRMKKCKATMYWTCACYGTYKGKYTTAAKSKYKTARYMTAGSKRRSK
46    AYTTTYYYRRSMRKRRMKKSKRKMYWKAGACKKYKKKTKYRWKSKTKYMAYMYWRSKRRSK
          ***  .********.* .***....  .** * .    *** *   **   ******

38    YAYWKRYKRWSMCRGMWAKRYGRKKAAMRKATKGGRYRCACYRCCKYCKYKCMKWKGCAT
46    YMYWKGYKATSMYRSCTTKAYRRKKWRMRKCCTSCRYRMRYYGYMKYMKCGTAKWKATRC
      * *** **  ** *.  :* * ***  ***. .. ***    *    ** *    ***.

38    GGCKWKYRKRTTCGTGCKTKAYKGGTRSYYYRYYTTGTKAMKKKWYRYRYKRKKGSCRTR
46    TATKWKYGKGYWYRKRYKCGWYTKRGRSCYYRYTCCAKKCCKKKTYRYRCKRKKRSMAKR
       . **** *     .  *   *.    ** ****    ..*. *** **** **** *  .*
```

```
38    MYYKYYKRRMCRTWYGAGAGYGTKCKRKKRWKGAYKKKRRKKYYYMCMKKYRYKRWRTKM
46    MYYKTCKRRAMGCWYATTTRCRKKYKRKKGWKAGTKKKRRGKTCYCMMKKYGTKGAAYGC
      ****  ***    **.: :   .* **** **.. ***** *  *  ****  *

38    RWMRRGYCKYKGAAKRWYWWTKCGRGWMCYYGKKCSKASKYYYRTTAGTTMRRWMMAKAA
46    RTCAGKCYKCKTTRGRWYWWAKMAGKTMMTCKKKMSKRSKYYYGWKMRKKCGRWCMRKRG
      *       * * :  *****:* .   *    ** ** *****  .  ..  ** * * .

38    AGKSYKYCGTYTYRAAAGKRYGYAKAGKTCYKGRYYGKRKCTASGKWYCGTATYCCTKGT
46    CTKSYKCTKCTKTGRMRSGRCRYMKMRKAGYKARCTATRKYYWSRKTYTRCCAYYMWKKK
      . ****     .     . *  * *  *: **.*  ..**   * * *   .:*   * .

38    KYYRRYKKTCGYTYGTMCKKYKGYCKGGYYCWTYMKKRTKYTGTATYACKKKGCKWCKYY
46    KTYRRYKGYYKCYCKGCAKKYGAYMKRRCCTTYCMKKRYKYYRYCYYWYGTGAMKAYGCY
      * *****          .*** .* *        **** **   . *   . . *    *

38    KYMMKGGRKRWSWGKKWGSGKYYCGYYCYMRTKRRGYCKTACGGAACGAGYCKKGGKTTY
46    KTAMGRCGKRWSWRKKWKSKKTTGACTYCCRYKRGKCAKYRMKKMRYRRRYMKKATKCCY
      *  *    ***** *** * *   .     * **   .*           * **. *  *

38    GGGGCKMYRCKARKMRKKKAKKACWWGKMKRKKRATKAKTACRAGATCCRWTGRCYYRKG
46    TARSYKCTRMKGGKMRGKGRKKRYAWATCKRKKAWWKRTWRYRGKCGYGGTKARYYYRGR
       . . *  * *. *** *  **   *.. ****   * .   *. .     ..* ***

38    RKRCRRYSYMYYRKYKGGKYTMGWWTGCKKTCTKAYSKCAMCTKTTGKTRCATYGAAACR
46    GKRYRGCGCAYYRKYKRKGTGAAATATGKKYSYKWCCKTGATCGGCRKKGMGWCKMRWYR
       ** *  .  ******      .  :  ** . *  .* .       *.  .       *

38    TMYWGKYAAGGTKRARRMRYYKCGARKTAYCTYKRRG
46    KMYWAGCTGTAWGGRARMRYYKMRRRKWRYYWYKARK
      .***.  :. .     ******   **  *  ** *
```

The pairwise alignments of two accessions 38 and 47

```
38              RWYMWRMMRKKTYRRRYMYRWRYMYMKTKYYSRRMKYYWWMYMRYYRRKAKYSKKRRMMW
47              GWCCWGMCRGKYYRRRYMYRTGYMTMKYKYYSRRAKYYWAMYMRYYRRKGGTCKKRRMMW
                 *  * * * * ********  ** ** ****** **** *********.  .*******

38              KYRRCCTAKYRYWKAKYYTYCKYSRMYMGYGKSYKMRRWMKRRRRYTGYKRYRTMWTTMY
47              TYRRMYWGKYRYWKRGCTCTYKYGRMYMRTKKSYKMRRWMKRRRRYWRTGGCAKCWYKAC
                .***   .******       **.****   ***************       . * .

38              KWTYWMRYMCATCYGRWRYRGTTRAAAAWWRYYRKSYKYRKRCKWKWRAKGYRWYTYWWG
47              KWWYWMRYMAGWYCRRAACATCARRWRRWWRCCRKSYKYRKGSGATWRGKKTATCYTWAR
                ** ******..    *      :*    ***  ******** .  .**.*       *

38              YWRTYCRYATCYTRWWWYKYRYACYWWYGKYRAMMTMYGGRRRYAGGGYGYRYKRYYYWW
47              YWRCYTRYTGTCYGTTTYGYRYRYYWTYCTCGCACWMYKKRRRYRATTCRCAYGGYYYWW
                *** * **:        * ***  ** * .  .   **  **** .      *  *****

38              AWARMYYRWTYRCTATSYWRWKRWYWYMYMRMRWTRMYWWCRKKTMCATYRYCYGYYRWY
47              WTMRMYYRTGYRYAGASCARWKGTYTYMYMRMRWWAMCWWTRKKKCMMYYGTYYKYTGWY
                   *****  ** :.:*  ***  * ********  * ** ***.    *   * *  **

38              RAKKMRYYYRRCKRMKCCYRYMRRRGYMKRWARWRGCCCTKGYMYRGYRYYRYKMWWYRR
47              RWKKCRCYYRRSKGCKAGYATCGRRKYCKGWRGWRAYATCKAYMYRRYATCGCKMWWYAG
                * ** * ****.*  *. *    ** * * *  **. .  *.**** *     *****

38              RAWKRKGCYCKMMRYYKMKRWRMRYWSMWMYRGACKKRRMSCCYYYYRGYRKYYYRMKTS
47              GRWGAKTMYYKMCATTTMKRWRCRCTCCAATRRRYGTRGMSTSYCYYRTCRKCYTGMKYS
                  *  *  * **    .***** *  .    *    .* ** .* ***  ** *  ** *

38              MYRRYMCWGRYCCWGTRYRWWMGGGRACCRRYMWRYRRRYCGSWCMKMSRKSSTRGRSRW
47              MTGRYMYWTGTYATKKGYRWWMATKGRYYGAYCWRTRRRYYRGTSMKMCAGCCWRARSRW
                *  *** *    .  . *****.        * ** ****  . .***.  .. *.****

38              YTGGRTTYYRWYMMKSRYYGYCTTAMTYYYSRKKCGTRKMKWYGYCMWTAYWGGGRYTTY
47              CATTGKYCYRWCMAKSRYYKYYYKCAKYCTCAKGYAKRKMKWYACMCAWMCWRRKGCYYY
                 :   .  *** * ***** *  .. .*  . *  ..******.       *       *

38              WKKKWRTCTGGRTCRKKYTKCRAGCMYTKRMGRYYKWTCWYWACCRKKAYRRMGRKKKYC
47              WKKKWAYMYRKACGRTKCYTYACRMACKGAMRAYCTTKTWYWRYYRKKWTAGMKRKKKTM
                *****         *.*  .  .    .  *  *  . . ***   ***    * ****

38              GYRWMMAMWATKAYMYCARMKYYYTMKRGYRRWKCRAYYTAKMTYRYCMRWCCAGCYWYK
47              KYRTCMRATRKGRTACACRAGCCCAMKASYRRWKTGGTTWRGMYYRYGMRWSMWKYYWCK
                 **  *    .     ..*     :** .*****  .     * *** ***.    ** *

38              MMTAKYGTRWYRRYAMTTKRWKGSRMKMYRKTTATWRKSYKCWYYRWYRKMWKTYRKTRC
47              CCKMGCKWATTGACTMAAKRWKRSRAGMCRKGAGCWRKGYKAWYYAAYRKMTKAYRKYRY
                  .           :*::**** **  * ** :. ***.**.***  **** *:*** *

38              TRRCYKCYTKARKYYKATGGGYWRWYKCTTATAAKATARKMYKMGACYAKRYAKYKYRKG
47              ARRGCGTCGKWAGTCGGATAKYWRTTKGYKMKCGKGCGGKMYKMRMMTMGGCRKYTYRKK
```

```
                   :**      *      .: . ***  *  . ...*. . *****         **.***

38                 MYYYKWKAKAYYTTARRGGSMMTCYAKKYKYYAGRRRMMGGKTRCRAGRKRATWGTCGTT
47                 MTCCTWKRKRCTCYRGAACCMMWYYWKKYKYYCRRRGMMKRKWRMRGCRKGWKWCWTTGC
                   *   .** *        . .**  * ******. ** **  * * *. **  .*

38                 RCTAKAKCGYWRYYYRSYKAYKACTYYCACKWKWKTTYRCTARRTRRKMYAYMWYACWTC
47                 RTCTGRGYRTWGYYYGSYTRCKTYYTYSMTKWKTKWYTATCGRRKGRGAYGYMWYWMTKY
                   *  :      * *** **.  *:    *.  *** *      .**. *  *.****    .

38                 GCKYYRRYRWGRKTATATCCATKGTACARKCMYSRYRMYCCTCRGCCRCRKRATGGRGCY
47                 KSKYYRRYRWTRKYWYWWMMWCKSKRYRAKYMYGRCRMYATGTRKTGGMAKGTKRRATTY
                    .******** **          *..     * **.* ***.    *       * :.      *

38                 SYWRRRYKGAKGTKRYGKRMRGCGCWSYYMYAWKMYCAGRRRMYWRYARMKWSKKRYGKA
47                 SYWRAGYKRWKSYKRYRGAMRAATTWSYCMYCWKMYSWRAAGCTAGCRACKTCGKRYRKG
                   ****  **  *. ***    **..  *** **.****.               * . *** *.

38                 GCTAYYRTKYYYYYKYTTGMGYRKCYYYKMGTTMTKKKCKTTMGGCWCRYSYKSKRYKTK
47                 AYATYYRYKTYYYYKYWWRMRYRKYTCYKMSYYAKKKKTKYYMSRYWYRCSYTGKGTKYG
                   . ::*** * ******    * ***    ***.     .*** *   *.   * * **..*  *

38                 KTACCMKMRSRRCAKCYCGTSYTTTYCRWKYYYWKKYTCTKYRYMCTKKCTKCAGCAKCA
47                 KYWYYCKCASRRYRKTYSRAGYYYYCYAAKYYYWTKYWYYKYRTCYKKKTKKYTATCKMT
                   *     *  ***  * *. :.*        *****.**    ***     .** .* :. .* :

38                 CMRKTRMYKGAARTACCAYCGRKKWKGRRKKYYTKTTMYAKCGRWCWGGKRYRWARTRWW
47                 YMRTKRMTGRWRGYGTMRCYSRKKATRRRKKCCYKYYACWKTSRTYAKAKRYRAGRAGWW
                    **..**        .      .*** . ****    *      * .*     .**** .*: **

38                 TYKCKRYRYCYKWWRKCWKGYMKCTRSKGARKKYKRWYKKAAWRTTRKAYKKAAYKKGGR
47                 WYKMKRCGCTYKTTRKYTKRCAKMKGSKRGGKKYKRTCKTMRWRAYRTCCKKCCYKKRRG
                    ** **     **   **   *    * . ** . *****  *.   **: *.. **..***

38                 YTWTRSMYKYARKYRKRRRRYYRYMRYYWGGGRGTRYGKKKYATCAGMMWKATCKCTYMA
47                 YCAARGMYKYGRTYRKRRRGTYGYMRCYARTRRRKRYAGKKTMYYCACAWKTYYKTYCAM
                   *   :*.****.*.******  * *** *     * .**. **     ..   **:  *

38                 CSKKMRYGRCRTCKMKYRRCSYKKCCATTYTMCRRYKMAYMCCYYWSGGMCRCRKKRYKY
47                 YGKKMRYKRYGKSKMKTARYCTTTATMYWYAMYRGCKARYMMTCYWGRTMYAMAKKRYGY
                    .***** *   ..***   *  . ...     *:* *   *   **    **.   *     **** *

38                 KKYYRMYKKCATCRRTTYKKCKYKMYGTMCMTTACCCTYKMCGRKYGTCRAKYKKRWAYK
47                 GKTCRACKKTTGTGAGAYKKYKYKAYCKMYAKKMMYYYTTAMSRGCRKTGGKYKKGWGTK
                    *  *   ** :       :*** *** * .*   ..        .  .*    .   .**** *. *

38                 KKTTKYKRYRWRYKMRKKRTWTYCKTATRKRRYGRKKSKYYAGCTTCRCCGRWMRMRCTG
47                 KGGGKTKGTGWRTGCAKGRCACYMKCCCGKRRYKGKKSKYTCTTWKAGAAAAACGMGYWT
                   *    * *    **      * *    * * .  ****  *****  .     .. ...      *

38                 AYMKWKSYMCCTGTKGTYTRTAAATCAYMKGKKAGYKCAYYCRCKCTATCSGYYKWAYAC
47                 CYMKWKSCCATCACKKCTYRCGMRYYGCMKRGKMRYKGCTCARAKYYWYYSRYYKTMYMY
                   .******   .  . *     * .      . **  *   **  .   .*.*      * ***  *
```

```
38    YCTKGYYRRYYWTAMTWKMSKKYKKYWRKGYTKCRRKGYCYAGCYTKKTTCATKCYYKGY
47    YAAKKTYRRYCWGWCKWKMSKKYKKCWRKKYGKSRGKACSYCAYCWKKWYYMCGMCCKRY
      *.:*  **** *   .********* *** * *.* *. .*..   **         * *

38    KTWCSYRKKRKKKYTGGACKGGCTSYWRTYYATKTTMAGRCGKMCYCKTYYWCAARRMAK
47    KYWYCCAKKGGKKCYSSRAKRKYWSYWRYYCGKKGYMWTRMSKAYCYKKYYWYCCRRMRG
      * * .  **  **  .. .*    **** * ..*  *  * .*    *.*** ..***

38    RYAWKKRRSYYYYYYSRRRWRMWKRRARWKCKWGCAKKTKWRTWKKYCTKCARTWWCGGR
47    RYRTTKARCYYYYYYSRRGWRMWKGGGGTKYKTKYWKKYKWRYWKKYTAKYCRYATMACR
      **  .* *.********* *****  .  * *    ** *** **** :* .*    . *

38    YMRTYARGACCMMTACYTGCGYWWKSAKMCMTCKRGTGCTKMKACACYYYMGTTYATSTG
47    YCAYCCRSRTTACYMMYAATKYWWKCWKAAMAYGGAYATCTMTTTTAYTCCRYWCWYGCT
      *    .*.        *:.  ****. * .*:   . .  .*.: :.*         .

38    CGCAGTCRCATCAYYRCSRACWKYGRTKRYWYSWKCCCWKASRYYMAKCWRMKSAYKCAC
47    TAMCTATRMGCYRYTGYGGRYWKCRRYGACTYGAGYTYWKRSAYYARKYTRMGSWTKYRM
       . . : * .   *   .   **  *     *.     ** * **  *  ** *  *

38    TGYACAATTTKGAGGTCMGGWTKAYYCGRKKTTYRKGARRKSMAYSGTRGAKYGGKTKWK
47    YSYTTWTCACKKMTTGYMRATAGCCCTAGKKWYYRKKMRGKCMWYSAGRAMKYARGYKWK
       .*:  : : *      * . : .   . **  ***  * *.* **. *. **.   ***

38    CTTGYRTYYKYYGYCTCGMTYGGKTRKWMTGYGRYKWKYYWKRRYYYYRSTTCSSAACRY
47    YCYTTGGCTKYCAYAKYSCWYKRGYGGWMYRYRRYKWKCYWKGRTCYYRSYWYSSMGAAY
               ** .*.. .  *      **  * ***** *** *  ****   ** .. *

38    YMCCRYCCCGWCAKTMGTGWSTKKMWYRGRAKKYCGTRAGACCTTTAKMMGAKMYRYKTC
47    YMYMRCYMTATYTKWCRYRWSKKGMTYGKRMKKYGAAGTATATYYCCKCMRRKMYRTKAY
      **  *    .  :*     **.* * *  * *** .: :.:.    .* *  **** *:

38    CTAYYYYGKGMMKKAGAGRGRRSMYKKKSRYMSMKCTMAKRMRRWKYRRAKYKATYRCTK
47    TYRYYCYSGKMMKKRKGRATGGSATKKKSACCSCKYAMMGRMRRWKCGRRKCKMGYRGGG
         ** *.  ****  .     *  ****   * * :*  ******  * * *  **

38    MRMKKKRKKTCGAYMYKMWYKKRKKRRKCRRYMRMRTKKKYRACKRYWAGMKKTYRKKGW
47    CRMKKKAKKCTAWYMYTMWYKGRKKRRGMAGCAAMRGKKKYRRSKRYWRRAGGKTGGGTT
       ***** **  . ***.**** *****       ** ***** .****     .

38    WCKTYMMRKACGACKKRSWAKGMKYYKRRKMKYAYKCSWYRYGKWTYYKTTYKCCKWRRM
47    AYGYYMMRKGYTTGKKAGACKKCGCYKRRKAKCWTKYGTCAYRKWWCTKYGCKMMKWAAC
          *****.  : ** . .*    ***** *   * .   * **   *   *  **

38    ACTKKRKRGAKACTGYGKRTTKGGAGTGTACGTATAAYYTAKRTTKMGATGTTTRMYTYG
47    TTGTKRKRARKWMKKYTKRAATTTGAGCCGTKWCKCGYYKGKGCCKAACCACYKRCCWYR
      :  .****. *  . * **::.  ..   .   ....**..*   * .. .  .*   *

38    CTAAGYTRCAWAWRATTCTGTGYAATAMCAWWYKKRRKGAKKMRMAMGGTTGAKAATKWK
47    MCGRCTCATWATWGRKWMWKGACGGWWMTCWWYKKAAKKRKKMGMWAAACCTTGGMYKWK
        .        :*  .     . ..  * .*****  *  *** *  ..   : .  ***
```

```
38          YKSWYGAMWKCKKWASYGWMCRYTTGWKYKKAGTATYGRYRTTWRMKGARTACGATTGKR
47          YKSWYATMWKMKKWGGCRTAMRCKYSWKCTGTKWTCYARCGAAWRMKKRGCCTAGGCAKR
            *****.:*** ***..     * . .** . :  : *.*  ::****    . ..  .**

38          MYCRAMWKAYCRKKKAGAMKKYRYYKKGKYGACRKKYYCKKGTYRKGGCRGKMKKMRKRG
47          MYTGRAWGMYYAKKKWSRCKKYAYYKKKTCAGTGKKYCTKKRYCGKARYGKTMGGMRKRK
            **    *  *  *** .  *** **** . ..  ***  **    *.    .*  ****

38          MRKYGGAATYGCRRYRRKWRGCRYKKYKTKAKWRGARRTAYRYRYRYYKTKMMRKTTYRY
47          MRKYRKMMWCRYARYGRKWGAMRYKGTGCKWKWRKRGGKRYRYRYRYCKWKMARKCGCGC
            ****         ** *** . ***    * ***    . ******* * ** **

38          WKRTMTCYAMYWYKRMTRWYMKYAAYRKGWGGYWCTGYMMYYRWCYKGYRGTKTRWMKKT
47          AGAYMCTCGMCWTGGACRACAGYWTYAKTWRACTSWRYMMYYRTMCKKCGKYKWRTMKKW
                *   .* *     *    *: * * * .  .  ******   *     * * ***

38          TCCKWYRRWKSRTACMCRYYRYTKGTCRGYMCGGCRRKCWTCYKARMKRWMRYKKCRMKR
47          GMYKTCAGAKGAKRMMMRYYGCYKKWYARYMYTTARRKAWKMYKCAAKRWCATKKYRMKR
               *     *. .  * ***   *     **   .***.*. **.  ***   ** ****

38          AAAGTCWCTGAKRKAGAYGGWRWKCAGGTRACCWTAYKRMAAGYCYGTGGAMCKKSCCMK
47          MRMRCYWTARWKRKWAWYKKTRTTYRRAWGTYYWYCYKRMMWKCTCAAKTRMMKKSMMMK
                  * :  *** . *   * .   .  :  * .****      .:   * ***  **

38          GMYRYYWYKKCATMGGRACGTARYRYKWGYRYYYMYRWRCYRKSRRYWTGKYRYRKKKKK
47          TMYRCCWYKKYWWMKRRGMSWMRYRYKTRCATCTAYRTGACRKGAATAYKKCRYAKGKKK
             ***  ****   *  *. .  *****        **  . **.      * ** * ***

38          KGCGKKKTRTTGRARAKRTCMKRKYTGGGGTKGKMSAKTGGARYSMRYAACASAAATGYR
47          GCARKKKGRWWKRGRRTGCTAGAGCYRTTTCKAKMGWKARCWRYGCGYMCTMCRWRYSYG
              . *** *   *.* .              *.**. *:   **.  * .  .    .*

38          MMTRKRRYGYCRTACMCTCYYRWTAKYYRGARTGRAKTTYRSYAKKKTKGMWGTAAKAYA
47          CMKRKRRYKCYAATSMAWYYYRWGCKYYRSWGYAGTTKWTASCGKKKCKKATRKGGKWYW
             *.*****    ::.*.  **** .****.   . :..   * .*** *    ...* *

38          MCGCMYRKAYTCAATTTGMCYASYYMMGWKCTGTTTTRGRRWCACKGWWAWYKYYKKGTC
47          MYRSMYRKRYYYRRKWCRCYCWSYTMMSWKTWRKYKKRAGATYRYKTAAMWYKCCTGRWS
            *  .**** *    .       ** **.**   . ..*.      *    ***  .   .

38          RKKRRKRKTYGRTCAARGTAGKMATAKKGAAAMKKAARWCYKAGCCRKMKKKKGRKATCC
47          RGKGGKAGKCRRWYRWGKYCKKCGGRKTTTTMCKTRMRTYCKWSYYAKCTKKKARKGCMM
            * *  *  .  *       . * .  *. ::  *.  *   * .   * .***.**.

38          MCKGMAKKRKYWRKRCWGAATWYRKAKYKKKKMSKKCGCWRKCCKATKAWRWRCWYKCSR
47          ASKKMRKKRKCTAKAYWTGGGTYAGGGCTGKTASKKYKYWRKATKGCKRWRWRTACTYGA
             .* * ****   *  * ..  *  .  . *. ***   ***. *. * ****   . .

38          YTGGCYKSYAAAGACKGGGKRKMYRMKRYCYCGCMYYKKAAGRATYWSRMYKGRCTAKRG
47          CYRRYYKSYGGWRMYKRAAKGKCTACKRYSYMRYMTYGTGWKGTYCASRATKKGYYRKRC
                 ****..    * ..* *    ***.*   * * ..   :   **  *     **

38          TTWWGMKKWWSYTAATYTARTMKGARTRTTKGGASTAGCKGGTYRKMMAMCYYAAKAKKM
```

```
47          AYAWRAKKAAGYAGGATCMRCMKRWRKRWKKTAGGAGRMKRKCYRKCAGCYCCRWKCGTC
            :  *  **  .*:..:   * **  *.* .* ...:.  *   ***  .      *. .

38          GRYGAAKGMYRAMRYRCKKYYWKYKRWMWYMKKKKSYARCYYRKWMMGGSTKGCASRACK
47          RGTAWRKRMYRMMRCRMKKTYAGYKATMWTMKKTTGTGATYTAKAMARRCWKRSWCAGMK
               .  * *** ** * ** *  **  ** ***... .  *  * *   . * . . . *

38          MYGYYRCAMKYAGTAMKCMAMAATRRTCGCTKTWGARKAGATGGYCKATARGYKYATCKW
47          MCKTCGYMAKYRRWRMKTMRMRRWRRGAKAGKKTRWRKWKRYRACTKCGGGRYKYWWMKW
            *        **    ** * *   ** . . *.   **     .  *. .  ***   **

38          TTTCCCRGGGCTGTCTTGYYCCTWTTKTTKRYYAKKKACCASCCRARGRYCKYYKYMMKK
47          GYYSTAAAARYWKKMYYKCCYYATCWKYYKRYYWGTGTGAGCYMGWGRRTYGCYKTAMKG
               . . ..    .        :   *  ****  . : ...      *    **  **

38          AYGRSWKKTGKTTCTKYCRYYKCGYYCRRTRGYTTATGAARYCGAGKCKTTKKMYRMRAK
47          MYCGCAKKKRKKCMYKTTGTTKGACTYARYGKCACCKKMRAYYRRRTMKCCKKCTAMRWK
             *  . **. *.   *     * .    *    : ..    *    . *  **   ** *

38          KGTRCGRYWYRKYMATAKWWKGGYTRRRYRAKKAKKRAAKMRRWAYRCGRAYYRKRRKRY
47          GRWRYKGTTTGKYMTYWKAWKAKYYRARYRWKKRTKRGMKCAGTRTGYARTYYRKGRKRY
               *       ***:  * **. * * *** ** .**. *        .*:**** ****

38          KRRYSYMYYRKMKYWWKKYMRRAAAAGYSACKGMRAAYGAAACRMYWYWAAGGGTTARMR
47          KRGCGCAYYRKMKYWWKKYCGRMGWWSCCRTKRMGRGCKMRWYRMYWYWTGTASYYRRMR
            **  .  ************  * .  . .  * *  .      ******:. ..   ***

38          YYCCRKTAYSCTKSYKKRRGKKMGCTKKRGMKRMRYWGGRYYKYWTTAATYMRMRYKKCA
47          YYTTRKWRYSYWKSCKKAGKKKMRTWGKRRMKRAACTTRGTYKTAGGGRWCCRCRYKKYM
            **  **  **  ** **   ***    ** ***        **    .    * ****

38          TRTYGAGAYAAMTCTCYYKTWWCYYRRCKKMKARKTTAWSRWKGGTGGGTGYRYSTGCTK
47          YGCYRRSMYWRCYYKYTTTWWAYCYRRTKKMKTGGGGMACGWKATYRRRGAYRYSYRSWK
               *  . *     .   . *   *** ****:      . **.      .****  . *

38          CAAYWCGTSRSYKGTCCTTRTKYKKRRYTGKAGATRAWYRTCWWTRYATWCCGYAKYSWY
47          YRRCAYRYSRSYKKCYYCARCKYKTRRYGAKMRWYAWWYRWYWACGCTAWMYRYRKYCTT
                    *****     :* ***.*** .*      ***  *    ::*   * **.

38          TRWRYGYRSKTRRYRRATTCAGACTCYGTKSRWTGWMGGATRGYGKSRCYRKRCYRRYCK
47          CRWRYRYRSKCRGTGRRKCYTRGYYYYSKKSRTYCTCATWWRRYRKSRYYRGGMCGATYK
             **** **** *   * .  : .   *..***     .   * * *** **        *

38          ACWKKMMYKSMAWATTCKYGGMRATCCGCAYYYKRWYRRRRWTAKKKGKRRKCTMGAATK
47          RYTKKMMYGCCRWTAAAKYKKMGWYTTATGYYYKRWCAGRRAYWKTKRGRRKYKMRRWYK
               ***** .  *:::.**  *     . .******   **   *.*  *** .*    *

38          YCCAKWSRAKKYKWCTRSCWCRGMWGACYKACCSAGGRGAKYYCWGGYYYWKWRMCTKMK
47          YMARKTSRWKKYKWYKGGYTYRRCWKMMYKWYYSWRRRKRKTYGWRAYYTTGARMYCKMT
            * . * ** ***** . .   *  *   **   *   *  * * * .**    **  **.

38          TTRYYGCKYACRTYKYATWGCWGWAKMWYYYATAKCGYMTRRKGACTGGCRAMCGACCCG
47          YKRYYRAKCTTRACKTWYWAYTTWGKMWYTYTCTKMKCCCRGTRRYWRRYRRMYRMTAAA
```

```
       .*** .* : *: *   *.    *.**** *: :*     * .       * *    ...

38     CGYWWKCAYMKMMCKKGMYAYATTCACYYATYKYARSKYKKRCYMTTAGSSKKWRRSKYY
47     YATWWKMCCMTCMAKKSMCRCRKGMGMCYCCCKTMRSGTKKAMYMGCWRSSKKWRRSKYT
        . *** .*. *.**.*     .  .  *.  *  **  **  **    **********

38     YWRYKRWSGMMCWKRYRMMRKKAKRMRKRGKYRKYMRYCKCYWMKWKCRKGCCAKCTGGC
47     YWGYKATSSACYTKAYRMMRKKCTGARTRKKYRKYMGCMKMCWMKWKYGGRYYTKYCTAT
       ** **  *.    * *******..  *.* ******   *  *****      :*   .

38     TMYWKYRRAYYAYGCMGWRKTTKTKTRRSYTAKKTKKTARGKRWYKRKTRGSRMYYKYYK
47     KAYWKYGGCTCGCASCTTGKYCKKTGRRSCKMKKKKATAATRWCKRKWGRSRMYYKTCK
       . ****  .  . ..    *  *.. *** . **.**:: ..** ***   ******  *

38     RRKAWGSYRRKRCMKKGACKACCATGTAATYKRAWMGCTRCTRWYARYGYRCKGWRMCRK
47     AGGMTACCRRKRYATKARATTTYCAAKRRWYKGTTCATAAYYGWYRRYTYAMKRTACGRK
            .. ****  .*. ..:  .:..   ** :  . :    ** ** *  *     **

38     KYGRTKTRRRYRAMGYRYCMAAAARTTMWAGRKRKGRRYRYSYYKGAYKATRMYYTKMYK
47     KCRRKKYRRRYRGMRYGTMCMRRCGGACTRTAKRKRAAYAYCTYKSRYGRYRMYCCKMYT
       *  *.* *****.* *       .  :     ***   * *. **. *   ***  ***.

38     GRYCRATCYARYWCKKKAYCCCKGTTTGGCACWYRKCCTCATGSWYRRRGKCYWTAWKYG
47     CGTMACGTYGGCTYGKGRTMTTGTCYCCKMMYWYRKMYYYRYKSTCGGGAKTCTGGWKCK
            .  *.     *                ****       *     .*    .**

38     TMRMTGMTGTSTGTTWCCGTSCCCTCKYRRKRYSKMTYAKTTKCTYAWWSCCCCTTGTGR
47     KCRMYRAARKSWKKWTYYRYSYMYKYKYRRKRYSKMYTWGYYKAGCMWWSMAAACYACRR
       . **   : .*  .      *   . **********      *.   *** ...  .  *

38     MRWKCKMKYGGCTYCKCYATTSATYWTKKWTRKYRKAWATATAAATRTYGCTRTYTMAYG
47     ARWKTKMKYSAYYCTKYYCACSRYYWWKKWWRKYGGRTWGCCCTGCRYYRMAGACCMMCR
        *** ****..    * *.: *  ** *** ***      . .:. * *  : :  *

38     ACMKKKMYWRMTTGAKACKMKCSMYCGYWSYYRRTAKGGYGRYAGYGAGGCYRMAAGGTC
47     MAMGKKCCTAMGKTTKGTKCGAGCYTAYWSYTRRWWGRRTRGYRRCARRKYYRMWRKTGA
        .* **    * . :*. *  .. * .**** **        *   .    ***     .

38     GAMTTGRAKTYRSRCKYAKKYRRRARCKTAAAGAKKAARCSRRKMTM
47     KWAKAKGGKYCGCRYKCTKKCAGRGAMGCGCRSRKKCCGTGGAKCCM
          .:  .*   .* * :**   *.    .. . **..  .  *  *
```

The pairwise alignments of two accessions 38 and 48

```
38                MYWAMKKWMAGRKYWWSMWYMRKWRYRCGYCSWGCRYMYGTYRCWRMYRYRWYRMGMYWR
48                CCATMKKAAMRRKYTTCCTYMAGWRTGYAYTSWCTRYMYTCYRYWRMYRYRTYRMTMYAA
                     :***    ***  .  **  **   .* **  ****  ** ******* *** **

38                YRKCARGYTYTGCGGKRKKWTGAKCYSSYYRTWRCCGYRCRSKCKRRRSMMRRWYRKYTA
48                CAKGRARYACWRTKAKAGKWKKRTYTCCYTRAWRTATCATRCGYGGRRSMMRGWTGKYWG
                    *    *:     .*  **.  .  ..* *:** .    *.    ****** *  ** .

38                YKRGCRRYYRAMTRKYSRKYMCWRTKAKSRRRRYRYSRWKWWRYCYWRGRKGGRAARTAC
48                YKGRYGAYYRRAWRGTCRTTAGARKGRKSRAGGYRCCGTGAAGCYYTRAAKRRRWWGYGM
                  **     ***   *  .*.    *.  ***   **  .        * *. *  *    .

38                AYRWRYWKYMTYYRYATWYYWKYMRYKYYYRRMKKMAKCKSYKWRYYMMYWGRKYKRSCA
48                GYRWRYWKYAYCYAYWAACCWTYMRYKYYYRRMGKARTYTGCKWRCYACYTKRKTKGSMM
                  .********   * * :   *.*********** *  . .. *** *  *  ** * *

38                TYRYSRYGYRYWWSYKRYYSYGKYRAWRKCCRYMKRRSGAWGTYKRMRAAGMYATMYAYC
48                YYGYGRTRYRYWWSYGGYYSYCTCGCTRGYYGYMKRRSARAAATKGCRGCAACCWCYGTM
                   * *.*  *******  **** .  . *    ******.  .: *  *...  .  *.

38                MRYMYGAKCCGYWRYYCMACYAGGGSWYYTYYKYYRRTYRGYTCKMYRYMRKYYRYTRGW
48                CRCMYTGKYYRYWRYCYMRYTGATTGTCTYTTTYYRRAYARCWSKMYRTCGKYYGYKGKW
                   * ** .*   ****  *   ..  .      .****:*    .****   *** *.  *

38                YMKMWYGMRYGYMRYCGMRCYACRKWMRRRYYYYMRWWAMWGARMCSYWWRRKMRAKYCK
48                YMKCWCAAAYKYCATYRCRTYGAGKAAGAGYYYYMRWWGATRGRMYSYWWRRKARRGTYK
                  *** * .  * *      * *.. *     ********.   .** ******* *    *

38                SRRATRCATMYWYKRRMMYMKMRKYMRCMCGYTTCKMYYYMKRRRYRKYTAYSYRYRCCY
48                SRRGCRYGWCTWTGGGCCCCGCRGYMGYMTRCCCTGATTYMKGRRYRKCAGYSCAYRAGY
                  ***. * .   *          * **  *          *** ***** :.**  **. *

38                ATAWYWRWSKYWRRRCMKRYCYAYWWYWYGKCTTKGRGTACRGTGAKKTASKTTTTGAGY
48                TCGTCWRWSKYWGAGYMKRTTCWCAWYWCRKTCAKAAAGTTRAYRMKKATCKGCWWKGAT
                  : .  *******    ***      ***  *  :*. . : *.   **::.*     ..

38                MRRRKTCGRKAKGGGMGYMGKYATSRKYRYSKGTCAKWSGGCRKRGYCYWKCTAYYRWTY
48                CGRRTCTCAGWGTTTCTCAAGCWKSRKYRYGTRYTMGAGAATATAKCYTWKAWGYYRWYY
                    **.              .   .******..      ...  .     **. .**** *

38                TAYMKGRGAAYTRYRWMKKTSCWGKKKAWCRTCMWARYMCGCKGYRRCYWYYYTCAATTY
48                KMYAKRGRMCYYRCATCKTASYAKKKKGWYAGTATWAYMYRSKACAGYTTCTYYYRRKWT
                  . * *    .* *    *.:*   ***.*        **  .*.        *    .

38                TRGRTCAAYKYKKYYWKYYTKGTGYAGCYAKYGRCATTCAGCRTCYYMKAMKRKCKKKGG
48                YRRRKYTCYKCGKYYWKCYCKCWACCAYCGKTTGYWACTCCTGCYCCMKMMKAKMKKKSR
                   * *. :.**  ***** * *  . ..  .*     :  .       ** ** * ***.

38                GCTRCAKAGACKTMRTTGCGMCRTKTKTYAYCCTTRRSTAGRRMYYYYRKAAKRYWMKTT
48                KYWGMGKMAWYGYMRACTARAYRKKKKYCTCYYYWRRSKGSAAAYYYYRKRRGRYTMKGC
```

```
                          .* .    **:  .   *.*.*  :     ***...   ******   ** **

38                  RCRSCYYYYYYSRRKKYRRWRCYRWKKCCACKRWRMCTTYWKKKAACTYKYKKRKWCGGR
48                  AYRCAYYYYYYSRGKKTGGWRMYGTKKTTRYTRTRCTCCYWKKGGGMAYKTKKRKTMACR
                      *..******** **   ** *  **    .* *    **** .. :** ****  . *

38                  KKKMCKKRGMAGKRKGGGGGKYRMKYYRKGTTTTKWAATYYRMARRKAGGKGCTGGATRK
48                  KKKMAGKRRMWRKRGRKAARKYGAGTYRKAGGKCGTGRWCYRCTRRKGRTGAMYRKWKRK
                    ****. ** *  **    .. **    ***.  .   .   ** :***.   .     .**

38                  AGAYAMYYKKWAWWYKKTKMKGAGKKYRRAYMRRMRGACATGGGTGYRYYYKYKTGCTKC
48                  RSMYRCTTTKWRWACGGGKMKKTRKKYRAMYMRRMRTCTRYRRRGAYRYYYKCGYRSWKY
                     . *    .** *     *** : ****  ****** .        .******    . *

38                  GAATAWCCGTSRGGGSKYKKGCTGYAGATRSWAWARGGWWGTRCYAMKGTYTYSTWRRWY
48                  RRRYMAMYRASRSAKSKYKTAYGAYMRWYASWRWCRAKWARAGYCRMKRYYAYCCAAATY
                             :**.. ****..   .*     ** *.*. *  :    **  *:*.     *

38                  YYWKTRWYGYKYTRGRRATTCAAWTRTKAAATRRTCGTRRRMRRGYRRYCKYWKKYKGWR
48                  YTAKCRWYRCKYCGKRRRKCYTGWARYKCWWGARYMKKRRRMRRACGATYKYTKKYGRAR
                    *  * ***  **   ** .  :.*:* *.    *   .******.     ** ***   *

38                  MMTCRATCYRYYYYRKGWTYRRRAGSTTWSYCKTKKGWYYTKKYKYRTKTYYCWWSCCAA
48                  MMGTGWYTYRYCYYRKAAKYRRRTTSKKTSYMKGKKKWYTKKTTGCAYKACCYWWSMYRR
                    **       *** ****. .****: *.. ** * ** ** .*.     *:   ***

38                  WGARAMCCKTRMWKACGTGSRWYKTKAGYMAWKGGCYATTAYRSATCTKKWWTRKYRKGA
48                  TKRRGATTKCGATGGTCCASRWYKYKRSCARAKRAYYCACMCASRYMWKKWWWRKYGGAR
                       *.   *     .   .***** * .    * . *.:    *    **** ***  .

38                  WARCARTAMACRYMKTRGTCTCKYMKTTCAWRRCMAYGACKKKMYWRACKACMKRYYKGY
48                  TWRYWRYRAGTRYCKKRRKSKMKYMKCCYWWRRMMMCACAGKKCCTAMMKGTCGRYTKKY
                      *  *   . ** *.* ... ****    *** *   ... **      *.   ** * *

38                  MYGYAGCYGWYRGARKGAAKCRGRYKRTAGYKRCGRRWTAGGCYRAAWARKKGCCYKTGA
48                  CYRTRRYYAWTRTGRKSMRTYRRGYTRWRRCGRYRRGTYRRKYYRMRATRKKKMYCKKKR
                     *     *.* * .**.  . *  *.*     *  *       **   :***    *.

38                  GTATTTTTAYRTYRSRCKKKYRRAAAAATTYKTTTTYYRTSGKSRRMCTYRMKRRKAGAR
48                  KYWKKCCWTTAYCGCRYKKKCGRGMRWRACCGCCAYTCAGGSKGGACACTGCGRRKGAMA
                       ..   :     .* ***  *.    :     :     ..*.   .     ***..

38                  MWCGTKCCYATTGTYKYKYCARSGCTGYKWKGYAGAACGRRSKYYMYRMWWTCTGRWKCT
48                  CAMAAKSMCRKKKKYKCKTTMRSATAKTKWKKYWRGRMTRRSKYTMYGCTTCAAAATKMW
                       .:*.   .. .** *   **. :  *** *   .    ***** **     .:.  *

38                  GAGMMWKRYYMKGCKKKAKRCRKRYYRGCTRKGYCCYYMRYSKWWKKCRTKACMCRTGGC
48                  RMSACTKAYCCKRYKKKCTGMRTRYYRRYYRKKYMYCCMGYCGWTTKYGKKTYCYRCTAT
                      .   * *  *  ***..  *.****   ** *     * *. * .*  .*:   *  .

38                  KWKYRKRGCKYKYKRYCKTWKRYKYTRMKKWKRWYRKKGASRMYYYYKRRKRKGYWGWYAAK
48                  KWKYGKGRYGYTRYYGGWKRYKCKGCKKTKRWCRKKRGSACTYTCKRRGRTKYWRTTCGK
                    **** *    *.**   ***** .  ** *** *** .*   *  *** *. **    ..*
```

```
38    RCKYRRATGAGTMYYYAKRYCYYCTGKTGGKYRAGAYRRYMCAKRRWMRKAKKAKAYTAR
48    RTGYGRGCAMRYMYYTMGGCTYYMWKGYKRKYRRKGYRRCCTMKRRWCRKTKKRKRCYRG
      *  * *. .   ***      **       ***  .***    **** **:** *

38    CSMACCYKYRYKYYTTAKYWTKRWRMGGRGMYGATTKTKCCMTMKACGCCGYRYTGCYMT
48    MCMMYYYKYATKYYKKCKYWAKRWRCKRAKCYAWCAKYTTTAKCTCAAYGATRYAATYMW
       .*   ***  ***...***:****      *.  :* .   . ....  . **:. **

38    KRGTAKGCCYTCYMGTTTTRWMGTGSGCRMYMMACYYRATTKWYRAWYTCGWRTGCCYGK
48    GGAYWKRYYTYMCCRYWGYGTCTCAGTTGCYCARTCYGRYYKWYRRACWTTWRCRYSCRG
        .  *                  ..    *     *    ****      **   .

38    TCARRMRGTAKRMRKCAKSAGCWKCCCTRYGYTRYWGAWYRMTGYATKYYTCCCYKWGAY
48    CTGRGCGRWRGAMGGTGGCGRYWKAYTWRTKYWGTTRWWYRMYSYWKGCCGTTYYKWKTT
        .*        *   . ..  **.   *  *      ****.* .       ***  :

38    KTAGKGRTCKGGKTTKMMYGCRGRTWKGAGGGTTKGTTKYRAKYTTTTWYRRKWRSRTAC
48    KKRTKTRGYKKRTYYKMMYKYAKGWTKRRRRRKCKRWWKYRGKYCCGKTCAGKARGAKRM
      *.  * *  *  .  ****       *     . *   ***.**   .    * *. .

38    AGCKKKGRGTTAACCCKYRMRYKMCRMKGTYGAAKGGWWKKKWGGYRCCWMKAGCRKYYT
48    WRAKKGKARCCGGYYMKYACATKCYRMGKYCATWKKKTTKTKTRACGYYWCKGTMRKCCC
        .**      ..   **    *  **    .: *    *.*  .    * *.  **

38    KRRRTKKSCMKSGKYYWKYKAMGGRTATATCYRKCTRYYMTRWCYKYRKSRRYKYRYYRY
48    GGGGYKKSYMKSKKCCWKYKWAKRRWMCGCACRKYYAYTAYRTACKTGGSRRYKCRYYRY
           *** *** *  ****    *   . . **   *   * . *   ***** *****

38    WWKKWWSYGGCRGCTTCGCYYRTATRAWGYSTCTTAYCYRKTGGMMMTYYCKGACGWRYG
48    AWKKAAGYAAMGRMACMSYTCGYMYRMWSTGWYWAGTYYRKKKSMMCKCCMKSRYRWGTR
       ***  .*..    :  .       * *. .   :.  ***. .** .   *.   *

38    AAKCCCRYRARWCAKGTATAARCCTKYWWGRYTRRRKRYMAKKSYTGWCYRRGGSTYGCA
48    WRKYMYGCAMRWSRKRKRYWRAAMCKYAWKRYAAGRKGTMGTTGTCTAMCAGRRCWCASG
        *       **. * .     .  ** * **:  **  *....          .  ...

38    GCYYAMYACYKYYWRCAMGKRCTAGCRCGCTKYTACAAGATGGKWRAGKTTCCCATGWGT
48    RSYTWACGMCKTCTGYMAKGGGCTKTRAKAGKYKRMRWKRYRTKWGGKKYYSTAGAAWKK
       .*    .  *            :  *. .  **.         ** . *  . ..:.* .

38    YTYCKRYAYAKKKGTGCTCKRWCKYYYKYMMYGKGRSWKKWGSYRKYYCGYYCRTKRGYK
48    CYCYKRYCYWGTGKCAYGSGRAYGYCCKTAMYRKCGCAKKTKCTRKTTSACTYRYKRKCG
          ***.*  .   .  . *   *  *  ** *  . **  . **  ..   * **

38    GKTGYYKGGKTTAAYKMYRCRAKKGTYKRYKMKKWWGTRCGCKKRRYRTKAKRATMRCYG
48    KKARYTKATKCCMWYKCTAMRWKGRWYKGTKMGKAWKYRYSYKKARYRAKRTRGKCAGYK
       *: * *. *    **    * *   **  ** * *  * . ** ***:* .*..    *

38    GARYYYRYACRRKRYKRYYRKKWYWWKKMRTAAAGYSKCMGCAAKTAGGRCAYATAACKM
48    RTRYYYRYGYGRKRYKRYYRKKWTWWKKCGYMGWSCCKTAAMMMKKWRAGMGCMCRWYKM
       :******.  ************ ****    . . .*  .   *.  .  .      **
```

```
38                  YAAGGKTMKRMRYYKKYCGARTACTGKSYYTRRAWKGRAKRCMGKACCATGTKKYSRTWW
48                  YTGTAKWMKRMRYYKKTMRRRARYWAGSCYYAGMTKARMKRYARKTTYCAAKKKYSRWWW
                    *:. .* *********    *:   . * *     *.* **   *:  .:..***** **

38                  ATAKYWGCTRWWYRKRYARGWRKARCRRYAKMATRRCRYAGGCAATAAKARRYKCATWMT
48                  RWTKCWATAAWTTRKRYRARTAKGRGGRYTKAWYRRYRYMKAYRGCGGKGGGTGMMAACC
                      :* *. : *  ****     *.*  **:*   ** **  .  . ..*.      :

38                  RYYAGCCGMYYKRGSCGRAYARTTMGSGGKRKMRSKWSCCTTCMKKYYGCCRRCRTTKKM
48                  GTCTAYMKCYYKGRGTAGGYCGGACRSRRKRKMRGKWSYTGCYAKGYYAYARRARWWKKM
                       :.    ***  . . .*.  :  *  *****.***      * **. .**.*  ***

38                  GWTYRKMRYKKYARYWWTATKKCCSCAWCCKRSKKKTTGCYTTTARRMCATTCTWTGWYR
48                  ATKYRKMGTKKYGGCTWGGKKGYTSATWTTKRCGGTGCAYCYYWMRRMMMCGTGWAAAYR
                    . .****  ***.   * ..*   *.:*  **.  .  .      ***      *:. **

38                  CTAWKCGCCTCATGSWYKYWWKYYGTMWWYRGKMRRMGMTGRWKCACMGYTYCWKRACTT
48                  TWTTKTAMYYYRYKSTYKYWWKCCKKATWYRKKCRRMRMWRATKYRYMKYYCYTGRGACY
                      : * .       * ******   .  *** * *** *    *   * *     *..

38                  TWYKARKGYCGWTCYKKMRRRKKYARTCAARAGKTRTYKMKGRAAKAYRRAAARTGKRYK
48                  YWCKWGKRYSRTWSYGKCGGAGTCGRWYRWGWAKCGWCKCKTRTTTMYRRGGGRAKGACK
                     * *  * *.   .* *     . .*      .*    * * *::. ***...*:    *

38                  KYYCCCRATMKKTTGRGRRAGGTTCCMCAYMRMAKARKYCCCWWRWKRYMAKTATRAAGR
48                  KCYYYSAWYCTKWKKRARRTRRYWMMAYGCMRMRGRRKCYMMWTAWKAYMGKKGGRRWTR
                    * *  .    .* . *.**:        . ***   **    *  ** **.*.. *   *

38                  YKKWKRRRGCACTKMWKCGYKAYCTAWRYGRCKSRYTGGCYKKWWMACAGKTTTTGGKRK
48                  CKTAKRGGKYTYKTAWKYKYKGTSYRWRYAGYKGACYRRYYKGAACRYWRKCYCCRRKGK
                     *. **    : .. **  **. .  ***.  *.      **        *      * *

38                  MMKYTGGCKAGGKAAGAGCTMYYYYYRCYGTTATAWRACYKKGACTMYKAGCGRGKGAGC
48                  MMKYWRRSKRRRGWRRWRYYMYTYYCATTTWWRWWTGTYCKKRCYCATKRRYRGSKAGAT
                    ****   .*           ** **            :  ** .    *     .*...

38                  AARATWWTYCGTTTRGTAGKAKYYAAACGYAWYMRCYWYTGSTYTKWYGARAYRAYRYKA
48                  RGGWKWWKYTTGCKRCCGAGRGYYRCCYRTRWYAGYYTYWKCYYGGTYATGGYRRCGCKT
                     .  .**.*    .*  ..   **  ..    **   * *  . *   *.: .**    *:

38                  KRCYMMGCRRCCYKWKTTYCRRMRRRAKAACAAYMWYACWRKKCGCYYWGRYKATATCCA
48                  KRYTCCATRRTTTKTKWYTTRRCRGGMKRGMMTYMWYWMTRKKYKSCTWTRYKGYWWMMW
                    **    . **   * *    ** *   * .  :****   ***  .  * ***.

38                  GSYCGGTACARKMYCCTCKWGCKYMRTGGCMSYWARRRGRYKKAGTKTKRGYSRKRGMRW
48                  KCCMSSKRYRAKMYATGTKWKAKTAGKRRYMSYWRRRAKGYKKWSYKGKGKYSRGAKMRW
                     .  ...    ***.   ** .*   .   **** **   *** . * *  ***   ***

38                  GTWKGAGCYKRRRMYWRYACRMCCKGRKAWSKGAGKTGTRCCKWTAKRMRYMYTTKTKCY
48                  RYWKARCYCKAAGCTAGCGTACATKKRKTTCGAGAKGACAYYKWATKGAGCCYYYKYKST
                      **.    *        .   . * **: . ...* .    **::*     *  * *.

38                  ARTTGGMACKKYYYKYGYGAMYYKAYSRYCGTRKKGKCGWYGWTCGTTRKYCCTGTTYWY
```

```
48             GRWWRKCRSKGYYYKYKYACAYCKGTCRYYAKRKKKKTTACKTCTTCCGKTMMCAYKYWY
               .*      .* ***** *.. * *. .** ..*** *            *    . .***

38             RKKRTTRRKYRRGAKCRAATGCKKRAYGGTTCWGKYSGCTWYWACKRRAYRMKWRYYKYC
48             RKKAYYRRKCRRRRTYAMCARMKGAGYRKYKMTKKCCRTKWYWRYKGRWTAMTTRCYKTM
               ***   *** **  .   .:  *  .*   .   * .  .***  * *  *. * **

38             GRRWMMKKAKYCARTAGYASAYRRWKCTCGTCCRAGYRYCMRWCMRMKCACYWMMKYGKM
48             KAGTCMKKRKCACRCCACMGRYRRWKTYYRYYYGGRYRYGMRWSAACTMWYYWCCGCKTC
                    *** * ..* ..  . *****        . *** ***.   .  **      .

38             TRWYRRYARRCYWGKKMAYCCTKTTATCCRKSYKTWGAATYTAWYRYKKTYGRTTTRRYKA
48             WATTGACTRRMYWRTGMWCMTAGGAGCATRKGYKYATGCCYCGTTRYKKWCRRKARRCKR
                      :** ** . *    :  :. . **.**   .. * .  ****   *.:** *

38             RKYYTRMTATRGYGGYYAKRYRMYSKCRCKRKYMWKYTSKYCRKWYRYYKYRATMKKGKY
48             AGTCARAKRKGTCKRTCGKGYRMYCKTGYKRKYMWGCCCKYYAKTCRCYKYAGYAGKAKC
                   :* . .       .* ****.*   ******   .**  *  * *** .   *.*

38             RKAACTGAYYSCKGYRKRKRKRKKTAKRKRGMRYWGRCYGGRMRRMYRRWARCRYKSYKK
48             GGWGTCTWCCSTKKCGGRGAKAGGYGKRKRKMRCWRRYCARAMARMYGRWRRMRYKGTGK
                  .      * *    *  *    .**** ** * *  .  * *** ** * ***.  *

38             KKCTARSWTMYRYRCYGWAYTATTAKRKKYWRCCTCKTMTYRYWKRTMKTCAAAMKKCRK
48             KKAWMGGWKMYRYGSYKWGYCGCWWKRKKYTGTTGTKCMGCGCAGACMKCTGRGMKKARK
               **.   .*.**** .* *.* .   *****      * *        **  . .***.**

38             WYCKTCAYRTGWYCRGAGAKGYGTRWWYKCAMKYKWGAYKMRKKMAYYYMTCMKKRYRWR
48             ACTGKYWYGCTWYTAATTTGRCRKAWWCKYRMKYKWAGTKARKGCMTCCCGMMKTGTGAA
                   .  *   **  .: :    . ** *  *****.. * **         **.

38             KMRWTMRRYKYKCKRKYATCCRTTTTCYYGKKKKSKACRKRWMRKKMRKAWKSAYCCTGT
48             GCATYCGACKCKAGRKYTAAMGAAAAMYYKKKKKSKWMGGRWMGKKMGKRWKSWCATCAC
                        * *. ***::.  :::: ** ******    *** *** * ***  .  .

38             KAGGTYRAAKMKRCTGRTYKAKGTKTYYKKTCYKCWYACCTASKYCTKRCWYYRRKGGYT
48             KGRTYTRCGKMGGTCAGYCKMKAYGCTYKKAGYKYACGAYYWSKYYKKGATTYRRKRKCY
               *.    *..**    .   * *.    ***: **   ..   *** .* . ****

38             YRWMTKYKGCKGGYCGCACRACKKAAAYKAARKTYTCGYWYYYGAGKSAKYAAAGAMGGR
48             CRACKKYGAAGAACTTAGYRTTTKWRWYGRCRTYYWYRCWYCYKMRKCTTTWRMAGMARR
                *  .** .. ..   .. *: .*   *  .*. *    ** *   *.:.   ..*.  *

38             RRWKYYTSYYYMWCATMTCSWMRGRRGGCCKGWMAYYWGRWYGTTTRRYYYYAYRTTCSS
48             RRWKCCYCYYYMWARCCKYSWAGKRRRRMMKTWMGYYWKRWCKAWKGRTCYCMYRYAYSS
               ****   .*****.   . **   **    * **.*** **  : . *  *  ** : **

38             CCCCRYCYTCCKGRGWAMRSYWWSCYWRYKRYRAKCKRTAYKMMYGGTKGYMAAATAAYM
48             YAYMRCTTKYAGRGATTMRSYWWSYCTRYKGYRMKMKRCCYKCCYAKGKKCARWTATTCC
                .  *   . .   . :*******   *** ** * ** .**  *.  *     ::::

38             AMMYKYRKCGCAYKTRCAKTGWKKSRATCCGCTMRKYCTARSWRTRGMKGAAGCCAATKC
48             GCCTKYAKAAYGTGYGYRKGRTKKSRWWYTASCCAKTTCGGGTRGRRCGKGGATGGGAKY
```

```
                   .   ** *.. .      *   ****    ..   *   . . * *    ...  ..:*

38                 RWKGSAKCYGACCKYGKRWAKKGGYGYCCMCMMKRTTRYYGRKAGMCRAAKYCRGGKYCC
48                 RWKASWKTYKRYSKTAGGWGTKRRYRCTGMYAMTRYKRYYRRKMKCYGTGTTYGRAKTGT
                   ***.* * *   .* .  *..*  *     *  *.* .*** **      :..     .*

38                 AMWRYYKKAMWYYGRKRRCYKMGTRKGYWTGKAWTTGKMGCTAMTMCGGTGCCMMGCYYK
48                 CMTGCYKKGMWTYRGGARMCKCSCGTRYTAAKGTGYTKMCAACCGMTRAWRMMCMATYTK
                   .*   ***.** *     *  *  .  . * :.*.     ** .:.  *  .      *. * *

38                 KTTTYKKWGWKGKRKMMKKACCTGKRTTKMRCTCSTTTMCRKKCGRWRTARYYYKYRGTT
48                 KCYGCGKWRWKRKRKAAKKWMYKKKRAATCGTGTCYKWMYAKGAARTRKGGCCCKYRAYK
                   *     ** ** ***  **    . **::.      . . *  *  ..* *..    ***. .

38                 TYKYRRARCTMACGGTYGYARYRRKGTAYGTTKWKKYKSWMRKTCGYYTYAYWMRWATAC
48                 WCKCAGRATACGARRWCRYRGTAAKTYRTACCTWKTYKSWAAKCTAYCCYWYTARWMCGT
                     *      : ..      *      *     .  .**.****  *   .*  * *  **  .

38                 GATYKKTACCTYKYRTMMMWKAAWRKTTCGTKKGTMYRKAAYKCGCAGGAARTRGRCYST
48                 TGCCTKYRATCYKCRAAACTKMRWRGYYYRYTKKAAYRKCCYKSRYRRRWTGCAKGACGY
                    .  .*  .  ** *:     *  **       .* : ***..**.        :     . .

38                 KGAAAYKYARKGYRRWRKRYRRMRYATWKSYAATCKTYYCKTCTAMTTGTSKMMRKRCRK
48                 GTTGTYKYRRTRYRAWGKGTRGMRCRYWKCYRWWYKYTCYGYYCGMGCRGGKAARKRYGK
                     :.:*** *. ** * *  * **   **.*     *          .*    .*  ***  *

38                 MYRRCSGAYCCACATTYTGCTYCRRMTARCYWRYYGYYSMCRCRKKYKKKCKRGCATCKK
48                 MTARYCAMCATMAMYWYYKAWYYRRAWRGMCWRCYKCCGCYAAAKKYGKTAKRRTTGTKK
                   *  * ..  .  .   *  . * **       ** *    .   . *** *..**  :  **

38                 RTTAGGAYYYKKKMMWMMYTATGCGTCTMGYMTGTMTTACCTYKKCMKKKRACTGYYMTR
48                 AGARASRTYYKKKAMTMMYKGGKYCKYYMRYAYRYCKKMYYYTTGMCKKKARMYRYYAKG
                     : ..  ***** * ***..    .  * *      ..     .   ***     ** .

38                 KRGMYYKTTYRGAGTKASKRYKTKKCGAMMGGGRRCAAATWGYGRKTCRSTTTYKKYCTY
48                 KRAATYKYYTRKRSKKRGKGTTYKKYKRCCAAAARYRRRYWKTKGKAYRGYYYCKKCYYY
                   **.  **    *  ..* .*  . **      ... *      *     *: *.     **   *

38                 RYKYMCWWTKCAKYRKGYAGCAKACKTTARYTTTTRMYKKTACCSGCRRYKTAKWYKRYK
48                 GTKTCYWWKKTTKYAGRYTATCKTYGWGRRTCCACRMTTKYGTMSSYGAYKAGKWCKRCK
                     *    **.* :**    *:. .*:      *    : ** .* .  *.    **:.** ** *

38                 YMRMAKACKCGGKKYRKWTCRYRWRTYKKKYRCYTCGYCYKSRRKMWKRKAWKAGYMKKG
48                 CMRARKWYKTAAKKYAKWYYRYRAGWYKKKTATYYYSYTCGSRRKMWKRKGTKRRCAKGK
                    **  *  *  ..*** **  ***   ****    *  .*    *********. *    *

38                 AKRCWAKCACRKAGYTRAAKYKYRYKKTCCTATAGYYKYYTYMMCKKKWYAGCCRGGGKG
48                 RKGMWGKTCTGKTTTGGGGKYKTATKKAYTYRGMCYYGCYAYCCYKKKWYRTYYRRCRGK
                    *  *.* .  *:     ..***    **:        **  *:*    *****     *

38                 MRYAGWWWSMYKKSRKYMSMKCMTGCKARMRYKYRKAKKATAGCTMMWGRMMYKKKKKTC
48                 MAYGTWWWSATKKSAKCCSCKYMCRTGTRARYKYGKRKKMGMRGGCMWRRMMYKKKKKCT
                   * *. ****  *** *  * * *     :* **** * **        ** *********
```

```
38                  GMYKMWKRKTKRKCRRYSRTKKCAGAMKYRRYWAKKCYRGCYCMMGKCCTAGAKRRYKKC
48                  AMYTMWKRTCKRGMAGCSGGKKYRRCAKYRRYWRKKYTGKYYYMMRGATAGTTKAATKKT
                    .**.****. **     *  **   . ****** **     * **  . :. :*   **

38                  GKCMKYYKRRKGTYYKGKCKSWKWT
48                  CKMCGYYKRRKRKCYTRKMKGAKWY
                     *   ****** . *. * *. **
```

The pairwise alignments of two accessions 38 and 49

```
38              MYMTKGWKCYGRWKRTGCRKSWCRKYWYKRRKWMTGYAKYYCAMWKYRWSMGYWMYYSYR
49              MYMWTRAKGYRRWKAWSMAKGTYATTATGGAGWMAATRGYYGGAWGYRTCCRYAAYTSYR
                *** .  * * ***  .  *.   .       **:.   ** . * ** .  *  * ***

38              KGYYSYYMMYAKRYRTRMYMKRRSGGCRYGCRMWRKYMRYGRYAYYRYYKKYGRRMMKTT
49              GRTCCYYMMYTTRYRWGCCCTRRSSAMRYRYRMWRKYMRYRRYRYCRTYKKYRAACMTWY
                    .*****:.***     .***.. **  ********* ** * * ****    *.

38              YYSRRWWRWRMYMRMRYKSSRSTYCYGYYRWWTTRSYRWWMRKMYRRYWWMCCCKMCTMR
49              TCCGGTTRTRACAAAAYKSGRSYTYYKYTGWAYKGSYRWWMRKMYRRYWWMYYTKAMKMR
                  .    * *      ***.**   * *  *  . ****************   *  .**

38              RRRGRMMRYSMRRCAATMRKMYYRYRRYKWMARYKRRRYMMRMMRMKKYGRRRRYRWWRK
49              GARARMMRYSAGGMCCCMAKMYTRCARYKWMTRYKRAGCCMGMCRAGKYKRRRACGATGK
                  *.******    .. * *** *  *****:****    * * *  ** ***      *

38              YRKYYSRRMYKYYWYGCYAYSKYTWRWRRYMGKRRSGRSYYMCSWYTMKTKYTRCCCGRY
49              TRKYYSRRCTKYYWYRYYMYCKYWWRAGACARGGRSRGSYYMYSWYCMKYKYWRMMYRRY
                 *******  *****  * *.** **        **  **** *** ** ** *    **

38              GAYYRRYRYYWTWYCKMCGGCGGRWRYWYGYWRKYTRMKYRRKYMWYMRYAWRGTCCCYG
49              ACYYAATATCTYWTYKMYRRYKSRWRYTCRYWGKCARCGYRRKCMTYMATRWRRKYTTYC
                ..**        *  **     .****   ** * :*  **** * **   ** .   *

38              TYKMYYRRYCMRYMRMRCYAMWRWKRYYYKYYRRCKMKYMYKAAMTKSRYWWTMYAMRYG
49              AYKAYYRRYTCATCRCRSYGMWRWKGCTTTYYRRSKMKYMYGTRAWKSRCWACACRCRCK
                :** *****     * *.*.*****    .****.****** :   *** *      *

38              MYYCCMYARRCCKWRYRYGGYGRYWKTTYYYTGCGCARRTYYCTYSAYYYTATCTCTKAY
49              MYYTTMTWRRTMKAACATSRCRGYWKAYCCCAAATYGRRGYCYYTCWYYYGTYTGGAKWY
                ***  *  **  *     .    ***:    :..  .** *    . *** :    :* *

38              KMKKRAGYGWRCTKKCTWKWWWRCRRSKGCMCAWGAGACGACAARAAGYGCCSATYGAKM
49              KMKGGRATTTGTAKKYCAGTATRYRRSKATCTRWKGKRMRGTCTACGAYRAYSWWYKRKC
                ***   .     :**       * ****.    * .    . .: ...* . *  *  *

38              CKGGYYCCCCRMCKKRTTYTGCYYCGGTYKYRYRKYCAGCYSRKMCGTWAGCKYWRYKAM
49              TKRRYCYSYMRMYKTRYKYKKMCCACTACTTRYRKTGGRTCGGGCMCYAMRYKTTGYTGM
                 *  *  .  ** *.* .*.    .  :  . ****  .   .          *   *..*

38              WYYKRKCTACYYTCRTGCRWKTMATCCYYAYCMMCGMCKWGYYCATMMYCKGRTTKRWYK
49              WYTKRKMYRMCCYMRYRMRTGYMCGTMYTCCMMCTAMAKWRCYSWYMMYMKKGWYKRTCK
                ** ***        *   *   *.   * .  *  .*.**  *.  *** *    **  *

38              KWWGTTTYCGWWYRRKKGSRTACYWAAARYYKKYTAGKKGTCYRGGCCCRKGRGGRYMAA
49              KWWRWWKCYRWTCAGKKKGAKRMYWTTCRYYKKYYRRKKKWYYARRTTARKRATTGCCRT
                ***   .   *    **  . .  **::.******   **   *     .**        :

38              RRTCTTAAWKTRMAGAGGWWKYCAGYGTRGACWMKAGARGATTMCTGGTYYGTRAYKCAR
49              RRCTYKRMWKAAAWKTKKTTTYYRRTAWGKTYWMGRATAATCCMGCKRWCCAARWTGMGG
```

```
        **   .  **:    :    .*    .   : **  .: .:  *       .:*    .

38      TTTTGGRRKKSMTGAKYYWYTKTTTGGTAMKKTATCKRWRKRCGTAATAAKYGRKKRGKA
49      CCCCAAGGKKSMCKWTCCWYWKKWCKRWMMKTCGCAGRTGGRMCCGGCGGKCARKGRRTM
            ..  ****   .  ** *.      **. . *   *   .. ..* .** * .

38      ATGGYWYKTCCKKRRCTAAAYYKAGTAKKRYCYYRKYYYKGKMAGWMRMWSWWAKAYYRT
49      GATKYTTGYYTKKGRYCTTTYYKRACMKGGCSYYRKYCYKKKAMAWCACAGAATTRCTRY
        .:  *      **  *  :::*** .  *   .***** ** *  .*    .  :.   *

38      ARCSRMMAKRKRRKTTAYMKTGKKRRYKGKTRYKTGCCMTRTGCGRYTCTACCATATACW
49      RGMCRMMTKGKGRKYYWYMKCKKKARYKAGYGCTASTTAGRAATTGYCTWWMYRAGAWYA
           .***:* * **   ***  ** ***.    .:.    *:.   *       :.:

38      MACATTYTCAYMSRMCRRYMCYRCTATTAGARMYRMRRWMKYYWRCAARKSCTTATTTWW
49      MWYTYYYYATCMGRMMRRYCTYGYWRYYRTWRACGCGAAMGCCTGTGCAGCTWCGAAAAW
        *  :  * .: *.** ***  *         *       *      ..  .   .::: *

38      KKGACRMGAKATAKMYYAKMKTGCMCSYKWAYGACCRRKMRTYCRGCARTGGCTCGAYKA
49      KKRGTRMKRKRYGKACYWKMKATTCTCYKWRYKRGTGAGARGCMGRYGRYACTCTRTYKG
        ** . **  *  .*   * ***:    .*** *        *      .* .     :**.

38      RACCACGYWKAGATRWWTYKYKKKMRRKYGAARRKTCRGKMTAAKAAAKWYCAKRKKCCY
49      GGAYRYTCTKGRRGGAAYCKCTGKCGGTCTRWGAKCTRKKCATTTGRMKTYYGGAKGYYY
         ..      *.        * . *   .      *  * * :::..  * * .  *   *

38      MKRTMYKKKAGATTRRTAGGKCTYMMRMKKARSKCCCWRWARWYCTAKGAATRTRYYATA
49      MKAYCTTKKRACCARRATAAKMGYAAGMKKRRCKYMMTAWMRWYYCCGTGGGRCAYYGCR
        **    .** .. :**::..*  *   *** *.*     * ***  . ..  *  **.

38      TATASAAMKRGGRYAAKCGKYYKTAWRWTAWCAGRKSRYTGGCKSYKMATAGACKGKRKM
49      CRCWGRWMTGRKGYRRTYKKYYKYRWRWWMWTGRGKGACYRRYKGTGCRKWRMYKRKGKC
            .  *.    *  .  ****  ***  * .  *.      *.    .    * * *

38      YRMKKYYYTAAGCMKRYYYKYYRAWACYKASYYYYKGRGRKYCRTYYTCTTKMATWTYWT
49      TGAKKYTYYRMRYMGRCTYGYCATWTYCKCSYYYYKKGSRKYTRCYTCTYGKCGYAYTWY
           *** *     * *  * *  :*:  *.******  .*** * *     * .    *

38      RWKAGWGGTATGARRKKACCTGYKRKYMRTCYAYTYMTARRGWTGARWAYRYYYRTCTMG
49      AWKRRWTRGGYCMRRKKWMTKKYKRTYAGWTYGTWCCWWGARAAATRTGCGCCCGYTCMK
         **  *   .   ****   . ***.*    *.          :.:* .         *

38      CMGATGTTATTTYTYTCKTTRRAKRCRARAGCWRTCTGTMRWWCCTWMTTSTRRMMATMT
49      YAAMCACGGGYKYWYYMGWWRRRGATRGGTAAWGWTCAACRWWSYYAAYCSAAACAWYAY
          .  .  . .* *     **    *. :..*    .: ***.      *:

38      ATCGCGTTARGATWKKKCYKSYKRTATRAKYTCGWYRYGAAWMCAGRSYGKRYGTTKYKA
49      RCTAYACCTRKMKWTKKAYKSYTACGCRGKYCTAWYRCTMWTAMCRASYKTGYAYAKYKC
           . .  :*  .*.**.****.  . *.**  .***       .  ** . *. :***.

38      MYYKAGCRYRYCTRKYRKWKWASWMKWYGCGWTAYRKRCYWRKYKWRCRKYKCRAGCYKR
49      AYCKGTMRYRYYKRKYRKTTWTSWCKATAMTAWMYGKRYYWRKYKWAMATCTYAMRMYGA
         * *. **** .***** .*:** *  .     * ** *******   . .     *
```

```
38      YTTYTTCWGTWYWACCRRAYRMWKCRKKYYRWMMYCCRKAYMKTYCAYYKAMKGKKCRKR
49      YCYTYKMTKKWYWRYYGRWTAMTKYRKKYYGTCMYYYAKRTAGKCACYCKCMKSKKTGKA
        *    .   .***     *    * * *****    **    *     . ..* *.**.**  *

38      RSTKTTTTGTKYCCCACYWYYAMMMGKYGTTTTGRTARAKTTTRWRRATTCRTKGGCKMA
49      GGGGYCCGRWKTGSMWTCAYCRMCCKKCKWWWGRRWGRTTAAYRTGGTCYMRWTSKSGMW
         .        *  .      *  *    *       * .*:.:: *    :    * .. . *

38      YWRKSKCCWCTWGAATKTWTRRTTRKARKYYTKKKMKRGMTKRMGRYGTAAGYYRKRMMT
49      CWRKGKTATTCATGCCKWTARRWCATRAGTCKKKKMGRRMYKRMRRYSATTRYCGKRCAY
         ***.* .      .. *   :**    .      .**** * * *** **.::: *  **

38      TYKTYYYTTTTAKATTTKACCGAYYMTCTCYYKWGGWYKKAAGTKKTRRAAWYKTTGTGK
49      ATGAYTYWYKYMGGKKCGGTTSMYYCYYKYTTTTRKWCGKGTRGKKCGAMWWCGCGTGAK
        :  :* *  .    ...  .  . **    .    .    *   *.:   **      *      .*

38      KKACRGAAYCWCGAGCKYKKRYTGKCAGAGWASWAWMCCGAKWYYWRWTACMRRYGYKYM
49      KKGYRRRRCSAYRTKSKYKTRYGAKYMRWKATSWRWMMYRRTTTYARWAGTMGRYRYKYA
        **. *     .    : .***.** .*       :** **      .  * **:. * ** ***

38      RYRRRYKYWYRRKKTYMTSRMSCSGSAYGYRRYCACKKYGYCSMGTTWKKYMKMMRATCC
49      RTGGRYKYWYRRKKYYCWSRMSMSRSMYACGATYRYKKYRYYCCRCGWKKYMKMMGWYTY
        *    ********** *  **** * * *.        *** *  .     *******

38      CGGTAGGMYYYYRRRKKYRKTAYMMKWYRRYARSAKKSWKYYMCMTTKRMKYGSRYYWYR
49      ATTCTCACYYYYRRRKKYRKYRCMMKWYAAYTGSMKKSTGYCCYCCKKRMTYRGGCTWYG
        .    : . ***********    *****  *: * ***  *      .***.* .    **

38      AKATYARCKCTMKKRYKKTCGAAAAYMYKMWYRKCRTMRATAATRTKACAKYRWGKKTYR
49      RKMGTWAAKGGCKKRCKKCTAGTGWCMYTMWCRGMAKAAGCGGYGKKRYGKYRWRKGGTG
         *     .*   *** **   ..:.  **.** *    .  . ..   .*  .**** *

38      KKWWYCMMYRRYKRYKYKCMYKRRKTYYYKCAKKSAYRMYSKWRWYKKWRKAATCGTTGM
49      GGTAYYMMYAATKGCKYKMCYKRRKKYCYKTGKKGGCACTCKWRTCKTWRGWMYYRYGTC
            * ***   *  ***  *****.* ** .**..     .***   *.**

38      KTYTKKYKGAGTGGRKACSMTYKYMRKGYRATATGKRYKYKMRYGWGGTAGCKKGTYWTM
49      TKCKKKYKRRKWRKGKCGGMYYKYMRTAYRGYTAAKGYKTKMRYRWAACGATKKACTTGC
        .. .****       *. .* *****..**. ::.* ** **** *.. .. **.

38      CATCTCAKYTCACCAGSKMMKRCGRCKMKYRSWCCATTYCRRYTCRYWCTGGYYYYSGYT
49      TMYYAAGKCCTGATTRGKAAKRYKGSKMKTACAATMYWYYRRYGTGCWSWTRCYCCGRYA
            :..*   .. : .*  **   .***  . .     * ***     *.     *  . *:

38      MCKKAYKKATAATTCTTKKKTTYKGGKYACTKMYGTCTACKYYMTTARCCTYKCWYMTYA
49      MYKKRYGKTCGTGKTCAKKKGAYKRKKYMTCKAYCKYYRYGYYAKKMGYYYTTYATAWCW
        * ** * *: .: .   :*** :**  **   * *  .     ** ..       .

38      RMCACKKTGMRYCCTWRGTTGGAYMRRKAKTCRKYCRRYYWSMMYTTGGGMGKYAYSRTT
49      GCTCTKGYKMRYMMGWRRWWKAGTCGRKRKCTAGYTGRYYWSMMYYATTTARGCTYSRWG
           . *   ***   **     ..   ** *     *  ******** :       :***
```

```
38          AGRYYRTRKAGCTSKRKRTAWMMKRRKRYCYKCTCCCTCTTTAACKGAKRGRAYARWWMW
49          WRRYYRYGKTTTWCKRKRYRTMMKRATAYYCKAWYYMKMACKCGAKCTKGRGWCRATTCT
              ****  *:   .****   **** . *  *.    . : ....* :*

38          KTKYRRKKWTGWCTYYMWACMTCMYRYCRCRGTGAARYTCAATTWRWGACYGTGTCTATA
49          TGTYRRKKWCAWYCYYATMYMWSMYRYMGYGRWSGTGYYYRRKWTGWRMMTKCACTKTCG
            . .****** .*  **    * .****      ..: *    .   *      .  .: .

38          CGTTTRAAAYGKRMGRCMGCTKARATYSWWRKYYAAMGCTGAACTRKCRYGAYRCGWRRR
49          TRYKKRRRMCAKRCKAYARYKKRRRWYCTTRKYCWRCATAKGTYYGKARYKCYAMRTARR
               ..*    .**       .* *  *.  ***    . : .:   *.** .*     **

38          MCAAACRKRTRTKMYRMYTCGKMYKMRYYAGCCKWARWAMKCRKKTARAAKRCWCRKGRS
49          CGTMRYRKRKRAKATRAYYYRKCCGAGTCTATMGARRWMCTTGKGYRGCRKRTTMAKRRG
              :   ***.*:*  * *   *       :.     **  .  *    . **    * *.

38          KGYSKYGCCKYRAAGKYTKMGKRMKYYATKYCTGTRAKYGARYWWKGGCCCTCCCGAGWC
49          GKYCKTRMMKYRRMRGYKGMKTGAKYCWCTCAYSYAMKYKGGCTWKAAYMYKTYTRWKAT
              *.*    ***    *. * .  **   . . .   ** .   **..   .

38          CMKTGTTGKYYCARCGTTGWWYRKCCTCGSWGKWYWWKGTTYGTKMRWYRRMATYCGWYM
49          TCGCRYCCKYYMMGMKAAAATYRKMYYYKSTRKAYWWGAAACKKGCRATRRMGGCTRTCM
                    ***     ::.  ***     *  * *** .::  .  *  ***.      *

38          TWKRTKYYKKKKKKRRRGYCKYYKKYMGGKMGRSWKTGYRYYCGYYCKRTKRYKKWYTTG
49          KAKRYKTYKGTGKGGGRCTYGYCKKTASRGMKGCAKKRTGTTGACTYKRYKRCGKWYYKK
            . ** * ** . *   *    * **  .  *  . *.      .   ** **  *** .

38          AARCGAGYMMKKMYRMCKKYGRYKKKKCWGGRYKRKAKAKRAAYKGTGRAYYRKRKRRRY
49          MRRYRRRYMMKKCTAMMKKYKGTKGKKTWATRYTAKWKRTRGMYGAKARWYYRKRKRRGC
              *    *****   *  ***   * ** *. **. * * .*. * ...* ********

38          SYMYYRKGRKWWKKAAAGCYSACTTRCAYAAACRKAAGGKRKTRTATTAGTYRMRYYKCC
49          GCAYYRKRGKWWKKMGRATCCRTCKGMGCMRWYRGTGTAKRGCRWWYYGCCYRMRYYKAM
            .  ****  ***** . .  .   .  .     * :. .**  *    .  *******.

38          GARKKTASCTKTKSTTWSGCAYTTTKKWCYRYSMSCTYYCKAWWSCCCCTMRWKCGGCTK
49          RRRKKWRSYWKYKSKKTCKYMYGYGGKWYYATCMSYGCCYTMWWSMAAAYARWKTRRTCK
              ***  *  * **.. .   *    ** *  .**     . *** ...  ***     *

38          CGTGYCAGYMRAWCKCGCYATTYRSATTKWTRKYRKWTTATACYKMTARRGTRAGYGTCT
49          TCCRYYRRCAGGAMKTTYYCACCASRYWKWWRKYGGTYWRCGGYGCCRGARYRMRYRKSK
                *      .  *   *.:   *   ** ***       . *        *  * ...

38          CWRYKMAYKKTKKMYWRKACKMKKYAGWSYYRRYMKKGGCRGRRTAGYAARKAGYCYRGA
49          MAGCKCCYGKCKKCCTAKGTTCGGCWAWSYTRGTCGGSSYRRGRWRRCRRAKRKYYYRKT
                * .* * **    *. .     .*** *     .. *  *       *  * ** :

38          TCCYGTCRTTTCYRKCKAKYRRRATKMACSRRMCTYRYRKRYWMKKWWSYRCKATRWGRT
49          KYTYTGARCGGTTAKAGTKCRGRGAKACTGGACACTGYRKRYWAKKAAGTGYTMYRWKRK
            .  *  .*      *. :* * *.:* . .   .   ****** **  .   .  ** *.

38          GGRMRTAATYRKTKAYYKACKKGGGRYACAAKRGTMYWMWRCMAKARKYWRYKCGRYKGR
```

```
49      KRRMRWRRKYRKWKTYCKWYGTKKRGTRAWRKRACMCWMWRYMGKGRKYARYKYRRCKAG
          ***    .*** *:* *    .       .  **. * **** *.*.*** ***   * *.

38      AWRARWYKYKYAMWYMKKKSYCYRRYASGGCYYASRCKYCRCYYRCARGKACTCGGWKYY
49      GTARATYGCKYRMWTMKTTGTTCARYRSKRSYTWCAMKYMAYTCGYMRRGMTKYATAGTT
        .     *  ** ** **...     ** *  .*  .  **         *     . .

38      YCCTKGGTTAGAGATYMWYMYAYKYKGGKCCTGKTTATWRWRMYSKGYWTYGTGYAYWAY
49      TAMGKRRYYRKWKRYYAWYMYGYKYKKSKTAATGACGAWRWRMYSKKCWKYKCACMTTTC
         .  *           * ****.**** .* .:  :  .:********  *.*   .    :

38      TTTCTKGKCGRTAYKYAACCKYWKSYRTCKKAGGRRCTRKTKTAMCRTATKTRRSARRMY
49      ACWAKTRKYSGCRTKCGCTTKYWKCCAWYKKRRKRRYWRKGKYWAYRCGCKKRRSTRRMC
        :  ... * .     *  ..  ****.     **    **   ** *     *  . *.***:***

38      CRKTKRYGWMSCMTGTKTCRSRCRKRARWRWKAKKCCRKKGWRTRTWKKWCYKKYRTTWK
49      TRTCGRYRTMGSCKRKKCYRCRYAKGGATGTGGKKTTRTKKTRYRYWKKTTCKKYRYCTK
         *.  **  *.. . .*  *.*  * .      .**  *.*  * * ***   ****   *

38      RRRGTAGKKTGTKKCYATWCAAATTYKKSAYAYRRARSKACATAYSKKWRRKRKKRWMGM
49      RRRSCGAKGAAAKKMCRKWYWMMYKYKKSMTRYRAGASKGTMWWCSKKWRRKGKKATMSA
        ***. ..* :.:**    .*      .****    ** . **.      ******* **  *.

38      RWRYKAAKMRKAKRKKRKYYYRCTKTMRYKKYKWWKCRYWGCCRKTGGCKWKYRKRWGCW
49      RTAYGWRKMRKCTGKKRKYYYRYYKAMGTKKCGTWKYGYWRYYGKCTATKWKYGKGWRYW
        *  *   ****.. ********  *:*  **    **   **     *   . **** * *  *

38      TYKARTRCYCCCSYYYATKAKATKKAYRRKGKRMYKYYKKCRTCYYGAKSMYAAARKKYC
49      CYTWRGAYYYYYSCCTMKKMKGAKKGYRRKRKRMYKYCKKMGWYYCKMKCMCWRMAKKYS
         *. *   *   *     .* *.:**.**** ****** **     *    *.*      ***.

38      YYKYYTYWCCCTCCYGRGKTRGWMAYSYWWYGYKGWMWKAARTTCTWTGYYRYAAYRSAK
49      YTKCTYYWAYAGTYYKRAGYGKWMGYSYWWYKYKKTAWKGCRCAAAWCRYYGYMWYRSWK
        * *   **. .    * *.    **.****** **    **..* :.:*  ** *  *** *

38      YACMCRKYKRMYRTATGGYWSCKATATRWRYRTRACRRWYTTAYKMMRYCTTGMRRRGCG
49      YRYMMRGCKGMYRWRYRSYWSYGTCMCATRYGCRMMRGWYKCCYKCCGCTGGAMRRGRTT
        *  * *  * ***     .***  :      **  *   * **. .**        .***

38      TTTATGRKAYKCGYWCYYRGWGWTCYTGSKKWAYYCMMYRWRCCKWTAKWAKCTARRTRK
49      GCYRAAGKRYKYRTWYYYAAAAAGYCATGKKWRYYYMMTAARATKWCTTTTKTCGRRKRG
            :. * **    * ** . .     : .*** ** **    *. ** :. :*  .**.*

38      RKRMKKMGGTARRACYMWYACWKKYGCGYTTCCAGYRRSGRGACMYSRTGYCCTCRYTGC
49      RGRMKGARKYRGAGMYMWYWMTKKYKSKYYWMMWKCARCSAACYMYGGKRCATGTRTCKA
        * ***          . ****    *** . *          *.. .. **. .   .    *    .

38      KYMRATKSYWAGRGRGYMKRYGKKKTAGGGSCRKRWRAACCMSKRAGRRRMCYWAARRYW
49      KTAGWYTSYWWRGKARYMKGYSKKKCWRRRGYAGAWRRCTYASKRWRAAGCYTAGGGGCT
        *       .***        *** *.***       .     ** .    ***          ..

38      SKRYAGCCTGCGGWCKKWYCRAMTGACYRCTGKAASYRWYKGTGMRKKCMKGYKRWGAAY
49      CGRYGAYGCRYRKWMKKTYTRGMWRRSYRYCTGWWSYRWYGRKKAAKKYMKKCKGWAMGT
```

```
                    . **..       * ** * *.*   .**      *****  .   ** **  * *. .

38                  KKKSKKKTMKKYKRRWRKMRWKCMRYKYKARTWYWWTYATGCYRAGCMYKRCTKASKYKT
49                  GKKSKKGGMKKTKGGWRGCATGACATKCGMGKTCTTCYGARMYGRRYMYGATGKRSKYTW
                     *****  *** *  **     .   *    .     *.:  *    **    * ***.

38                  TRGRTMRKRKYMTAGKWGKTWGCYYKAAWYRYTKKCGKCGTYMKAGTCAGYKTCCTACSR
49                  KRAAKCGKGKYMATKKWAKCTRMYTKMRTTGCKKKSRGSTWTAKMRAGGAYKKAYYWYSG
                    .*. .  * ***:: **.*    * *      .**.  .    *  : ..**..    *

38                  TAWKGACGCCYCKKAWYRRYAYYKGKTGYTYRAMGGKGYTKRRWTRRGACGWGKYATKRY
49                  YRTKRWMKMMYYKKTWYRRYRCYKRKWKCYCRWCRKKACAKRRWKGGTCTATAKTRYKGC
                       *      * **:*****  ** *     *    *. :****.  . .  .*   *

38                  RMYCRCKCTKRKYMKAYCRCGKYKTCAGKAYRTYKYRGTATWSAGKYRACGCCTYYSYRA
49                  RMYTGYKYWKRKYMGCYYAYRTYKAATAKGCRYTKYAAGTAWSCKKCGGYCYSCCCSCGM
                    ***   *  ***** .*    .**:.:.*. *  ** . ::**. *  .   .   *

38                  KSKMKKTGRKCRGKTTWGRGYRRYRRCRKYWRCRYKKTYKKKYYWGARTKYRYYKRGGKT
49                  GCTAGGYKAGYRKKYYWRRKCAAYGRYRKYWGMRYKGCTKKGCYWKRGKKYRYYKATTKW
                     ..        * *  * *    * * ****  ***   **  **   .******   *

38                  YRTRKTRKMWKTGWGCYRATTYRYWKRTMGYAAACACAAAWYTAGGKRKWYMCKGYCAMG
49                  CGAGKWRKMWGCRWATCACCGCGCAGAYMACGRGGGTGWRWYWGRRKRKACAYTAYYRCK
                      : * ****   *.   .         *. . . . .  ** .  ***    ..*

38                  GCYGGGRAMYMTKCKGMYYKTTMWYAAGAGTKSKRYKTCTTKKYMYWCAYTRYTAAAAYS
49                  RYCRRRRRMYAKKTKAATTKYYMWTRWKRSKKGKGTTWYYWGKYCYWYRCCACCMTWRYG
                          * ** .* *.   *  **     ..*.*  .     ** **        :  *.

38                  YTTTYYYWCTAAACWWTKCRKRKGRAKCMKYRMACCGKGGKRKACCGRKGKKWKGRRKKT
49                  YYYYCYYWYYMRRYWWKKTRKAGRARKYMKYRMTGTAKKTKRKGTYSRKRKKWTRRRKKC
                    *    ***      **.* **     * *****:  .*  ***.  .** ***. ****

38                  YMAGSTWACCGRWKKWKRYRWCRARTCCTTYRRYTCMKGCYKAGKSRRKMKWWAKSRYYR
49                  CAGAGAWWGTARWKKAKRYRAARGGYYYWYYRRYYYMKCYYGWAKGRRKMKTTRKSRYYG
                      ...:*   .**** **** .*.      ****  **  *  .*.*****   *****

38                  SGAAASCARKKYKK
49                  CTTCTCMGGKKYKK
                    . :.:. . *****
```

The pairwise alignments of two accessions 38 and 50

```
38              AKYWWTAWMGYMRKKRGCMRYKMGCYRYYAGKRCKATYGCWWYWCTGKKRWSYKWRKTGW
50              RKTAWYRWMRTMRGKRAYMACKMTMCACYRTKRYTWYTKAAATAYCCKKRWSYGWRGWSA
                 *  *  **  ** **. *  **     *  ** .    .       ****** **  .

38              RSRWWYMRWMGWYYRMKSYKYWTASGTTRRYSRYCKAWAKGYYRYTGSMKYRYMYWRYCR
50              GCGTTYMRWCATTCRMKSYKCAYCSAGCARYGRTYKRWWKKYTGTCAGMGYRYACWRTMR
                 .   **** .   ******   .*.   **.*  * * * *    ..* ***  **  *

38              MRYMYKRRYRRMRTGGKAAARYAGGTYGGYYCACYYMRYYYYRRYMMMWRMRYRWRKKYR
50              CRCMYKRRYRRMRYRRGGCGRYRARCTRRYYYRYTYMRYYYYAGCCCMWGCRCATGGKYR
                 * **********    ...** .     **    *******     **  *     ***

38              RRYKWTYRRKYYSRRMMYCYYWMYSTRRYYKCYKRYYKSYWWRYCTRKMYYRYMKTTMRY
50              RRYGTKTRRKYYSRRACTAYYWMYCARRYYKAYGGYYKSYWWRTTGRTCCYRYMKYYARC
                ***  . ********   .*****.:*****.*  ********   *.  *****   *

38              GWWWSMMRARWRYRGYYGGCCGTCAGAYRCWKAGGCCYCGGCRKCWRYRRKSGAWYWRRR
50              RWWTCCMARGWRTGAYYRCTYRYMWATYRYWKRKRTAYTAATRKYAACAGKGKWWCARRA
                 ** . *   **  .**        .:** **    .* .. **      *.  *  **

38              YMRKWRRGWYRYKCTTYKRYRKSSKRRCSTRRYRSTACKARYWYSWYRKKSWRYWKTTYR
50              TCGTAGGKWYRTKYWYTGGYAGCCTRATCWRRYRSAGTKMRYWCGTTGGKSWGYWKWYCG
                   .    *** *      *  ...*  . *****:. * *** .    *** ***

38              MYYYRCWWCWMRRKRKTARKRRMYMRYYKMRYGGYRRTRYYYYRRRCKYMYTRGTCMKRS
50              MYCCGTWWYWMRRGRTGCRKAACYCATTTCRTTKTGACGCTYYRRRSKYMYKGACTMGAS
                **    ** **** *. .**   *    . *          *****.****. .  *  *

38              YMKTRRRAGGRACCMSTTKYYRRKKSRYRKRWWKRMYYYACAKTAACCYTYGATRWGKRG
50              YMTCRRRWTKGRYYMSYCKYYRRTKSRYRKRWWGGCCYTRYRKYWRYMYYTRWYAARGRR
                **. ***       **  *****.*********    *    *     *         *

38              CTGKRATCMTGKCYCGMCGRKWACKYTAMAGATGTRAATGCTARRCTGCGCTGTCAYRYR
50              YKKKRGGTMWTKTCMSCMKGGARTGTWGAWACCACRWWWRMKRRATARSRYCAATCCRYG
                 . **.  *  *   .           .  .. . *     . *  : .   .: . **

38              YSAGRYRTAKRMAYYKARGAYKSRKGGYTRCYGWMTTAAKKYARTYACRYRWKAAWRKTT
50              TCTARYRCGKGMWTYKTRARYKSRKRRCCGSCTTAYCGGKTYTGCYRTRCRTKMRWRGYY
                 .:.*** .* *  **:*. *****     .      ..*.*:  *  * * *  **

38              CGTTGMCWKYTARRGKKGCCKKYKYGYGYSYGWRGRRYSMTACRKAARRRRYRMRYYKGT
50              YRYGTCTWKYKCRRKKKAYYKKYKCRTRYCYAWAKGACGCYWYRTMRRRGGTGMRYYGSW
                       ***..** **.  ****    *.*.*     .    *.  **    **** .

38              GTRTRMKTMRCATTCKAKMTAKRCRMYRCSCCWYTTYCRCARYMTRYYSRYSWWMCKAYK
50              ACRKRMGGCGTRKYYKGTMCMKRYGMTAYCATWYYWYYRTGGCAWRCYGRYSWWMYKRYG
                . *.**      .  *..*  **  *   .. **  * * .    * *.****** * *

38              KYAGRCATCYKARRMRRTTCGYGYKRKKMYRGTCTGMTTACCTYKMRCTATAACKMWACC
50              KCTCRTTGTYKGGRARAGAAATAYKRKKAYRCKYYCAKKMTTCTTAAMGGYTCGKCARTT
```

```
        * : * :  **. * *  :.. .***** ** .    ..     .    . :. *

38      MWMYCRGTGCTCMAKAYTTYTRMSGGGTGCAAGGMYRWSWKTCGWKGCKYKAMAGCWCAC
50      AWMYMRAAATYYMWKRTYWYYRMGRAAYTYRGTAACAACAKAAAWKRYKYKRMGRMAYRM
         *** *.:.   * *    * **. ..    . .    . *:..**  *** *.

38      TGATRYRAYGRTYRYWWKKKWWSRYRWCATGWAKTRYTYKRAGGYRKMTRGCYKRCTYKG
50      YSRKRYRMCTRGYRYAWKKKAAGGYRTYMCAWRKYRYWYKRRRRYRKMWRKYYTRTCCKR
         . .***   * *** ****  . **    .* * ** ***   **** *  *.*   *

38      RCCGCTAAKATTMRYRWGCMCACKYWRYKRWYYKYMWYKRCCCCATAWCKGGCYYWWYTK
50      GTAAYCWRKGCCAGCRWAGCACYKYARYKATYYKYMWTKRATTGTYTASKKRSYTATCCK
          ..    *.     **.  .. ** ***  ****** **.   : : .*  .*     *

38      CYCKRYYKWYRCARYCMGCTTYATGAGATYRCMKWYMGYKYYKGGKTAKCCWCATGKKYG
50      GCMKRTCGTYGYMRCTMKAGCCRCAWKRYYRMAKTTCKYKYYKKSKGWKTAAAWGCKKYK
           **    *   *  * .     .    **  *    ***** .*  * . .   ***

38      YGCCAYKWKRYKCGTRRKGYGMGSWTATKAGGGRWRKKWRTTTRKAYRKCRAGCRMRRWS
50      YRYYCYKWKRYKYAKRRKACRCKSAWMYTWATAGWRKKWACCARTRCRTYAMRMRMRRWS
        *   .******* ..***.    *    . . . *****   :*.  *.     ******

38      YKKTYWTWYRWACKRRRKKTAYRMTYGWCWCKKYCGATMGKAYMYCARTYKKCAMMCKTC
50      YKKYYTKTTGWRYKGGRKKYWTAMCCRTYTMKKTMKWCMAKRTACACRACKTYCMAYKCT
        *** * .   *  *  ***    *       **     *.*    ..*: *. .*  *

38      CRTTRCMRWCCAYWCMMTKYKWRRWYRRYAGTARRKYKAKTTATWRSKWYYCWCWYYKTG
50      TGYWRGCGTSMWCAMCCWGCGTRATTGACTRWWRGKYTWGKCTCARGTWCYMAMAYYKWK
            *    .            *      :   * **.  . :  *..* *    ***

38      KYRGYTRRTKYTRKKACMKYAACKKKKRWKKKYRKKTKMRYRWRKMRYCKRWKTWYCYRT
50      KYRRYARRWGCCATKRYMTYGGYKKKGGWKKKYRGKKKMGTGWRGCACYKGWGCACMYGW
        *** *:**     .*  *.*.. ***  ****** *.**   **     * *     *

38      SKRKSKGCYRYRMKTWTKYGTYRGATCKRATYKYKAGKTCAGKYYCYGCGGCTAASRCSW
50      SKRKSKKSYGTGMKYWCKCTCTGRGYYGGTCCKYKMRKYSGRKTCACAMAAYYWWSRYSW
        ****** .*   ** * *      .    :  ***  * .. *  . . ..    ** **

38      KWYCYKYRRYAYYKAGYTYRAMWKCCKGYRMRAGKKAACRAWMTAGKTCGATCGRTRYMG
50      KTYYYKYRRYGCCKWKCYCRWCWKTSKACRMRMAGKCGMRRWMWWKKCTRRYTRRCATAR
        * * ******.  *     *  ** .*. *** . *.. * **   *       *

38      KYATSRKYTGAGRYMRTCYTACCSYCGYMMCRKRGCACMGYCYCTRKTAMYYRWMKYTTA
50      GCWGSRKCARTCRYMGCATCCTYCYTSYMMMGKRTTGTMTCYCAWRKYRMYCATCTYCCG
            *** : : ***  .  .  .* .***  **  . *    . **  **    .*  .

38      WCRYYMWTARAGAAMCGGCGTTTCAYKGCRKYRGGGTTRRRKAAWRWRTMTKGKYTMWWG
50      WYRYYATKGGRKWWMYRRMKKKYYGTKRMATCGRTTCGAAATRRWRAGWAYKKTYGAWWA
        * ***  ..     *     ..  . *   .          .  **     * .*  **.

38      TYKKTTCATCYAACCKSYRKKKKCWGGYWRKTKMKKATKTKAMCRKKKKYYRRMTTCYRK
50      KCKTWYYMCTCRMYYKCCAKKKKAWRKYWRKYGAKTGKKYKWAYRKKKKYCRRMYKMCRT
        . *.           *.  ****.*  ****   *...* *   ****** *** .  *.
```

```
38      TAKTTKTKWMKTTRCTKRYYYYRKYRRRRRGRWKGYKKCCCGRGKKWMRTWKKKKKRTKW
50      CRKWKGYKTMKGCAYAKRYYYYRKTGGRGGKGTKRYKKTTYKRRTGTCRYWKKKKKRYKT
          * .  * **    :********   *     * ***    * .   * ******* *

38      RKTAKYRYKKYRKGRYAGGRGRRSAGCWTKKCTSRYYKMCARMKKYSRYYSCGKGAKKTR
50      RKYRGCRYKKCRGKAYGTRATGGSMSATYKKYYSAYCKCYRRMKTYSRYYGATKRGKKGR
        **    **** *   *.      * ..  **  * * *   ***.*****.. * .** *

38      CTMMTRMRYMRKKKCKTCGAKMYKKRKKRKGAKCRRYMRWRGGTKTYKKWKGMGKRYRCT
50      GGCMCRMRYMRKKTAKCTAWKMYTKRKKRKKMGMAGCAATGTTKKGYTKWKRAKGGTGYK
           * ********..*  . ***.******             .* *.***        .

38      AYMYGTWKKAGGARMYKRRKTKYYKSGKKKKMGGMWWTKRGMRRWGACYYKGTTRMRYYY
50      MCCCAKTKKRAACRCYKRRKKKCYKGRKKKKMRRMWWWGRRMRRWRRMYTGAGGRCRYYY
            .. ** ...* *****.* **. *****  ***  * ****   *  .  * ****

38      KWKAAYAMTCTCYYKWWYKCKKWMMGTAGRTTKKTYGRRCARGRTTGKGGGTTGKCYTAW
50      KWKRMYRCYYKYTTTTWCGTGKWMMKCTRRCGKKCCRGAYMRTRGYRKRRRYWSKYCYMA
        ***  *    .   . *    ****  : *  **       * *   *     .*

38      CAGCSKYKKCTATATRWTRYMMRAYAWYYCYTRWRARTRYGKYMTACTYRRYWRKWGKGC
50      MWKSSKYKTYWRWWCAACGCMMRMYRTTYYTKAAGGGCRYRKYACCYYTRGYTGKWRKCA
           .****.           *** *   *  .   .  ** **  .   * *  ** * .

38      WMGMTSMTCSRYRRCKACWKTGGYYKGSMAAMMRATRMCAYYYYYCRTAKYYTWTGCKKC
50      TCKMWSMYTSRCGAYKRYWGYSRYYGRCCMRMMGWYRMYGCCYYYYRYMTTCCTKRMTKA
           * **  **    *  *  . **  .   **   ** .  *** *  .    .  .*.

38      WAYWTGMRWKWWWTGGKGGYTCGKWSAKKCYYKKYCCCRRMKYCKYYGGTRKYRKMKKAM
50      WRYTKRCAAGTATKRKKKRYGYKKWSWKKYYYKGYYSYRACTYAKCCCTKRGCRKMKKGM
        * * .        .  *  *   *** ** *** * . *  .*.*    .*  *****.*

38      WYRCGCYCTACKMGTCTGGKWGGTGAYWWRCYCMCCAACKRRGWKTGYATTCACGTYAYT
50      WTRMRMTMYRMKCSYYWRRKWKCWRMYWWRMCTMTYTTYKRRTWGYKCRKGMGMKKYCCA
        * *        * .     **     ****   *  :: *** *     .  .  .*. :

38      YACAKYYRGRYSYSKKWRRYRKRWMMAWKRYMKKKACRCAKCKYMRYCKCYKCRGCCATG
50      TWTMGTYAAGYGCSKKWRRYGKATACRTGAYCKKKCARMRGMKCMGYMKACKYGAYYTCT
              * . *. ******* *        * ***..*    * * * *. *  .  :

38      GCKWKYRKRGMYWRTKGYRYSYTTGACTTAKTKKAAAGTRCCAGRWCYKGSYKKRRKKMK
50      ATKWKYGKGKMYWRKGAYRYSCCGRCAAKMKKKKRRWRGRMMTKGWYCTRCYKKGAKKMK
        . **** *  ****. .****    ..:. *.**     *  :  *  . .***  ****

38      ARTTGCTGGYKYYMTMYYKTTMWYRYGAGSKRYKTYMGCAKKGYRSYKTGTGTAACRAYK
50      RRYYRMYRRYGYYAKATTKYYMWTRTKRSGKGTTYYCKYRKKAYRGCKWAAAARRYGRTK
         *       * ** .   *  ** *   ..*  . *    **.**. * .:.:      *

38      WWTKCAKKCRKGTGCAGCKATTCAKRYKGYKKRRGCTRSAGRGKWCYGRRKKATARYTWA
50      WWKKTTKKYAGRKTAMSYGGGYYRTRTTRCKKARAACGSMSRRKWYCRRRKKTCGRCYWW
        **.* :**    . . .  .    .* .  ** *..  * .* **   ****: .*  *
```

```
38         CGGCRTWKRYKSRTYMRMYTCKGCYWKRKYKCKRYYRSGTACACRKKYKKMYYWYYMAAK
50         TAAMAYAKRYKSGWYMRMYYYKCTYWKRKCKMKRYYGSCCGYGYGKKYGGAYYTTYMCGT
            ..    *****  *****  *  ***** * **** *  . .  ***   **  **...

38         MYRKGTTMYKGTAKYYRKMYYCRYWRKCCAGKWAYTAWCSAAKTRKAAAGGRTMTWYKWR
50         MYRGAYAMTKAYMKYYRKCTTARYWRGYTGKKWRTYRTMCMTKKGKGGTTARWCYWCKWR
           *** . :* *.  *****    .****    . **       . :*. *..: .*    * ***

38         MKARGCTCCAKRMYTGRCRRKYMWKKWCRKGKRTYRGTATWSKKYRKARGMGARKMKTRK
50         MKRGKYCTGCGRMYGRGYRRKYMWGKWYATAKRYYAAGTAWSKKCGGRGKMATRTMGYAG
           **         . ***    ****** **   ..** * . ::****       *.:*.*

38         RAGSYGRCGYRRCYCRYTKKYKCRYTTRYKKRCTYKTCARYWCATCTAKKMTTTKTMTYR
50         RRKSYARYKYGRTYMRYGKGTKYGYKKRYKKGSWYKCSGRCAMGCMWWKKMWAYKCMGCG
           *  **.*  * * * ** *  *  *..**** . **  ..*    .     *** : * *

38         YWKRMGGGKACCCCAWCATGMCKGKTTRGYAKKKCGTSKKCAYCKYYKYMGGYKRKSWKK
50         CAGAMKRRKTTYYYRAAGGAAYGKKYYRRYWGTGYRKCKKYMTYGYCKTASRTKGKCAKK
               *   *:       .. .    *  * *   .   ..**     * *   .  * *. **

38         WGSYAYWYYYCRTYKYGARCGAGMKCRCMYRMCKGRYWYKKMKTKKMGCATRYRAKAKKA
50         TKCTMTACCTYRYCKYKMAYRRRMGTGYCTAMMKKGTWYKKMKKTTCRMRYRYAWKRTKC
             .         *  **        *        * *   ******...      **  * .*.

38         TYAYCGGRYAYYRGKRYKRKYKKTYYRCCAYSACRTGRATYAAACKTMYYAAGGRKTRRY
50         GYTTTKARCWYYRRKRYKRKYKKYCYRATWCCRTRKRGGWCMRWYKKMYTTGTARGCRAY
            *:    .*  *** *********  **.    .  *.  .       *.** :. .*  * *

38         ARMRYYCCCGAKTASKKKGKTRCCRRATYCCGTTGTAKYCGYWRKYYYSKTKKWTACATA
50         RRMRYYTTMRRKWRSKKKRKWRMTRGWKCYTTGCCCRGYYRTWGKYCYGKYKKWCGYWYW
            *****      *  **** * *   *   .          *    * ** *.* ***  .

38         AYKCMMCMCCRGKTTRCTARRRKRKATTACGKMACWMKKGCRRYRGGTATCCSMKGRGAK
50         RCKYMMAMTYRRKWYATCGRRRKRKGAGGYKGAWMTMKKKSRRYRRTCTWMMCAKSAACK
             * **.*  * *     .******.: .       *** .****    :    .  *.  ..*

38         RKGYCCRGKCKYMATGGCTSYWGKGGTKKARGSRKRMRWGCRGYYWTGAGWSKKWTGWKT
50         GKRYMTRTKMKTAWKRKMKSYWSKKRGGGGGRSRGAMRWKTGAYTACRMRTCGKWYRWKY
            * *  * * *     .   .***.*       .  **  ***    .*         .  **  **

38         TKKYYYGYYRMRAWGKCCTTRCMAAGGKACTKKMRSGYWKGCTWMCTRWTKAWYARYRGA
50         CKGYYYRYYRMRMTAKYTGCRYARMRRTTTKKGMGCACTGATAAAYYGTGTGWYRRYARG
            * *** *****   .*     *       .: .* * ..    .  :        ..** **   .

38         RMCAACRKTRYKYKYMGYKGKARTRRRYRYRYYACCMSGATTMATATWKRWKRGSKKYKR
50         RCGMRYGGKRYKCKTARTGAKTGYRRRYRYGTCTYMCGACGACMYRYTTGTKRRCKKCGR
           *        .*** *      .*:  ******     :    ... :        .  ** .**  *

38         RGAGAAKGTTSYSARYGCRATCKWRKCKYCCKWATTTYYCAGTKYRCCTCYKKAYWWYYG
50         GAGTCWTRWYSCGRGTSMACGYKWRKYKYTTGWMGCCYYMMKAGYRMYYYYKKGYWWYCT
            .. . .    * .    .  .  **** **    *     **     : **     ***.****

38         AAACAMRRMTTTMTWSYTKTWCYTKMKKWWSTCWTWYRRWKCMYKRMWKASRWYCYATTT
```

```
50                  RCGYCCRRMCKCCWTSYGKYAYYKGMGKWWSGMWYWYRRWKTMYKGATGGSRWYYYCACY
                     .. . *** .    ** *   *. * ****  * ****** ***    .**** *.:

38                  SYATWTKWRKGAWAGTSRCARAMMKRYRGTGCCTTKWMWYMTKKKMYWRMKACKMKYKYM
50                  STRYWWKWRKRRWWAASRYWRWMCKAYRRCTATAAKAMWYMAKGKCCTAMGGTKCGTTTC
                    *   * ****  * .:**  * * * **   . ::* ****:* *    * . *   .

38                  CGWSYRRTRYYYCRGYKTRTAGYSKRWGRCTCRTTYKGMKYTYKAKYYYWTRRGYRAACK
50                  YAWSTRRYGYYYYRRYTAAWRRTGGAWARAAAGYGYKKCKYKTKGGYCCTCGATCAGRYK
                     .** **  *** * *.:     .  *.*.:.   **  **. *. *         .  *

38                  KSRRMCTKCACKGWWWYKGGRRRKTKYGAAKAGGAAMMKAAKAKRWKKAGGWMRKGTKKY
50                  TGGACACKYRYKTAATCKRAGGAGGTCRRWKCRRWRCMKTTTMKRTKKCATTCATRKKKC
                    ..   . *   *     * .     .    *.     **::. ** **..    . .**

38                  YKGTRWACKMMRGMAKSKYCCCKWRGAKRGYKKTGCKMMKKTCKKAYYTKAGYRYYYKSR
50                  YKACRAWMKMMRTMRKCKCYMMKTATGKAKCTGGCATAATKKTKGGYYKKWRCGTCTKGA
                    **. *   **** * *.*    *   .*   .   ..  .*. * .**.*       *.

38                  YTGTGCWYSYKWAGKRKKYCKYGCYYYYRARATGGTTCYRGAGCKRKCKWCYKMMYYRCK
50                  CYRYRYWYSYKAWRKGKKYSKYRYYTYCAWGTWRAWAYCGSGATGAKMKWSYKMMYYAMK
                          *****   * ***.**  * *    :  . :   ...   * **.******  *

38                  GRTMKKCCRTGGWYRRWGYWMKKGTRCRRYRRYKCGRMKYTATTCCGTCAGGWWRKTRAW
50                  KGAMKKTYGGATTCAGACYWMKKKWRARGCRRCKYRRMKYKRCYYSKYYWKKTTGTWGTW
                      :***    .       *****  *.*  ** *  ****.    .         .  :*

38                  TCCKMRRSGARKGATTGWRAYYTRCKGKSRCGMYRYYCTGMMTACCACGGATTAKKTATC
50                  YMMKCRRSAGATRWYYKWRWCCYGMKAKSRMKMYRCCYWRCMYRYMGMRACWWMGTCGCA
                       * ***.. .     **      *.***  ***      *    .  ..    . . .

38                  TGAYRWKKKKTATTCKYRYYKGAYCGAGKRKSTAYSYYYAYYYRRRRKYYYKYTGRAAKY
50                  CATYRTKKGKYGWCTKCRYYKKCCMKMTKGKCGWYSYYYMYCYARRRGTCCTTYRATGGY
                     .:** ** * .   * **** .     * *.  ***** * * ***    .    :. *

38                  CRTCYGRTYRGMAYSYWWYWGWGSWWACRRYYYAAYRSCATRMCAKKYCWYRAWWYYMKK
50                  ARKYYKGYTGTMGCSYWWYWATKGWTWTGRTCTMWYRGYTKRMYTKGCYTYRMWWYTCKK
                    .*. *      *. ******.  .*    *     **. :.** :*    ** ***  **

38                  M
50                  M
                    *
```

The pairwise alignments of two accessions 38 and 51

```
38                  CYWYMGRRAYYWWYYRKYWCSCTYSRWKYYRYRAMRKMRKGAYKARAAAMCKRWYCTGWR
51                  MCTYMSAARYYWWCTRKCAYGTGYGRTKYTGYGRARTMRKTRYGGACGGMYKRWCYYTTR
                       **.   ****  **   .  *.* **  *   *.***  * . ...* ***     *

38                  YKKTYRYRKYTTYGAAYMYWKRWYCKMTAGCRKCAMYYCWACTGMYMRYMYRRGYSTCRT
51                  CGKYYRYRKCWWYRTCYCYTKRWTYKACTCTRKYWACYYWMYYRMTCRCMYRRCCSYYGC
                      * *****   * :.* * ***  *  :  **    * *    *  * ****  *

38                  CCYAACAYGRKKMYWRTCRYWMCACYYMRYKYKYRMGWRKCATRAYRMYGWRTAGTRGMC
51                  YMTTCARYTATKMTAAATRYWMYRYTYMRYKYKYACRWRKTTWRRCRMYKWRCGRYRCAT
                       :.. *  .**   : ****    ********   *** : *  *** ** .  *

38                  YKTKTRWKCAYMKWYWTRYAGAYRYGYRRWTWYMRCARCWRWRRSYMCRKACKSSRRCRS
51                  YKKKYGTKGRYMKWCTWACRKRYRYAYRRWWWCCRTGGAARWAGSYMYAGRYKCCRAYRC
                    **.*   *  ****        ***.**** *  * . . **  ***     *..*  *.

38                  TRKCKRMMRGRKKSYMKTYYMWMKYRRRYKRAYRSRRMMYKYYWCYSGCRYYYRRATASW
51                  WRGYGGMMRRGKKSTMKYYCCWMGYRRRCGGWYRSRRACTKYYWAYSRYRCYYRRTCGGT
                     *    ***  *** ** *  ** ****    *****   ****.**  * ****: ..

38                  RYYYKYGCTYCKCYRYYTRKMYWSSYYWKAKRACARRSYKRRCTTYKWRYRKWRYRRWWA
51                  GCTTTYAGGYAKTYGYYGGTCYWSSYYWKRGRGGGRRSTGAGYCWYKAACAKWRCRRWWT
                        .*.  *.* * **  . ********  *. .***       **    *** ****:

38                  YGSYWWMKYRTGGRTTRYYKGRRYWYKMYRYAAGRYWMYYSRKRWRCTCWMWSMYMTRTT
51                  YRGTATATYRCAARCARTCKARRYWYKMCAYCTARYWMYYCRKGARYYMAATCCYMKGCY
                    * .    .** ..* :*  *.*******  *.:.******.**  *      . **.

38                  YCRTGCKTCTAYMGYRTGCMTATMMYMARWAAMSGRCTGGACAGTGKMYWAYAAKRKKAT
51                  CSAYAYGYTCRYCTTRAATMAGAMMCCMGTWRCGKRMCKRWTGTAAGACARCGGKAGGGC
                     .  .      *   *:. *:.:**        . *      . :.      ..*   .

38                  KSCCRCAGKKTRGTAAKAGCCCCAKMRMYACTRMCCYATSGTTWSYTTCGKWCYTKMSYW
51                  GCYAAYRRKGKRKARGKRTYYYMMKCGACTMWAMYYCCKSKKWTCYGATRKAYTKKMSYT
                     . .    * .* : .*       *    :   *   ..* .  .* :  *   .****

38                  WWSGCCGTYRMRWKGMYAACATRWKCGTGCTYYGTTTTTCYYRAGSRMTWTGTYMRRKRY
51                  WWSKYYKYYRARWKTMYTTTGAGTGTCCAAKYCRYWWWKYYCAMTSRMWWWRCYCRAKRY
                    ***     ** *** **:: .:      ...*      . *    *** *   * * ***

38                  RCKMCKATAMARYGACKTTKMYWKMTCATCKACKGMGWSRTCGTYAAARYRYYGRTAGYY
51                  RSKMMKTATMMRCACAGWYKCCTKMYMMYSKGTKRCAWSRKMRGCGRRRCRYYRGWRRCT
                    *.** *:::* * ...   *   **    .*. *  .***.    .  * ***

38                  KWWGGAGGCYRAAYKGKTTTACYCACKTYGRATTTYGYATKMCTRWYRMYGRYYMKYKTA
51                  GWWAKRRKYYRWRYKKKCYWTMTYRYKYCRGGCCAYRYGCTCACGTTGCYKGTCAKTKYR
                     **.     **  ** *   :     *    .  :* *. . .      *     * *

38                  KYYYMMKWYAAGCCCGCRAGRGRRCASAKSRAWCYMCTGTKRMTSRYYWKYRRAYRKACT
51                  GCYYMMKWYRMCTTTTMGRKATGGYGSMKSATTYCCYCRKKRMCSRYYWKYGRRYRGGGG
```

```
          *******                .* ** :       .*** ******* * ** .

38      KMTMMKYMGCRKTKKKMYKRKCGMGCRYSGATAAWKTMKYRKWAGYRRKKGWWCYCMMMY
51      KCWCCKYMKARGKKKKMYTRKYRMTMGCSTGCGGTKGATYRKWRRTGAGGTTAYYYMMMY
        *    *** .* .*****.**  *    * . .. *  .****           * ****

38      KKRRYKKGGWAKTAGYMYKRRKTAGTTYYTKCTCYWRYWKKSATGTRCRTCRGSARTYTR
51      KKAATKKRRATKYWRYCYKRRKCTTKCCYGKYATYWRYWKKGMCAYRARAARRCTAGYAA
        **   **   :*   * ***** : .  * * : *******.  . *.*:.* .:  *:

38      YKRGCMCYTKYSYKKMCKRAAKKGGTYRKGRYKMKRWYWRYGTKKMKKKKSYWWMKTYTG
51      YKRRMCYYYKYSCKKCYGGTGKGRACCAGKRYKAKRWYARYKCKKMKKTTGTWTATWCAT
        ***    * *** **    :.*  .     *** **** **  *****... *  .  :

38      CCTGCYGGCYYSAKCCMCYRYYRCAGCRYCYGTAWRMRKGCAGCACYCKTAAAYKYYKGG
51      TTGCTYARSYTCGKGYCMCGTCGYMRYRYTTKKRWAMRKSMWKYWYYTKCGTGYKYYKKS
             *. .* ..*             **   . * ***.      * * .:.***** .

38      YTCCCKCKMGYWWYTATKKGTYRCYAKKYMKKTYCGAYYKYKKRTMMYATAGTCKKYKGR
51      YGTTAKAGMKYWTTKMKKKAGYGYYGGGYMGGCTTTCTTKYKKGCMMCMYRAYYKKYKTR
        *   .*. * **  . .**. *  *.  **      .  ****  **    .  **** *

38      YCRTAYYTARYWCSMAKTRKRKKAGGGCWKKWRTCGTRKKAAGGYAKTWTRGRRCGCCSY
51      CYGARCTYRGYTMCMTKKGKGGGGARKYTKTWRYYRYRTKCWRRYWKYWAAKGAYRYYGY
           :      *  .*:*. *   ..    *.**    *.*.   * * *:        .*

38      KYYRMATCRGARKCTGYYRMRYYWCCYKWRKYRTKTTCWKKRYKGTTGCYRTGGAMMKRM
51      KYYRMRAAATCRTYYKYTGCGCTAYYCKWRGYRKKKKYWKKRCTCGCATTGCRRMAAGGM
        ***** :.  .*.   *          *** **.*.. **** .   .           *

38      KYRCCCWGATCYTRRYTYCCRYGYKRMCRRKKRYRAYKGCATCYKKGGRCCAYYYKKKAK
51      KTAYATWCGAMYYRGCWCSSRCRYKRMYAAKKATRRYKRTTGTYKKATGYTGTYYKTKMK
        *   . * .: * *    ..*  ****   **  * **  :  ***.    . ***.* *

38      MYGYCTGMTWGKCMAGGCTKYKKRSTCWMRWYCRYAGWRKAYRMCTCTAACRYRCKTRRR
51      AYCYYYRACTAKYAMRRYKKYKKGCAMTCAWYMRYRRTAKGYRCGWYWMRYGYRYKYAAR
         * *      .*      .**** .:    ** **    *.**         ** *   *

38      ATTRYAGKMYAMATATWYAGRKRWKRKKGTRATCGRRSYSYRYCWCCGACGTKRMWCMKY
51      RYCGTTRTCTCCMYRYTYRTRTGTKRKKRYAGYMKGRGYCTACSWATRMSCATRMTTAKT
             : .  .      *  *.  ****   .    *.*.   .*.   . :.**   *

38      TCKRYWACWACGYCCTTTGWKCWYRKCCTCSWKYWWYGTKGGMGKKKMWWTATKKYRGMW
51      GAKGCTGYWMYAYTTCCCCWKYTYRKMYYYSTGCTWCTGKRRARKKKMWWYRWGKYRRMW
         .*   . *  .*      **  ***    *    *   *    ******    *** **

38      YRWRWKRTAAACWACCACYKGTTRMCCCGCYKTKTTRTTGAGAYTAMYYKKWWYKCKCTK
51      YRWRWGRATTMTACATRMYKAGGRCSMTTTYKCKCCRCCARSMYGRCTTTGWWCGYGTKK
        ***** *:::   ..   **.  * .    ** *  *  . . *     . **     .*

38      KKAAGRKKARWSRTGTYRYTGCTKRGAATCKCTAMYWCGGAKGCYKGYTAAGAGWAWRRW
51      KKTRKRKKMRACGATGYRYYRSWKGRRRWYTAWCACAYRRTKKSCTTCYMCAMRAWARRA
        **:  *** * . :  ***  . *      .. .      :* . .    ..     **
```

```
38              TRMMRYAKGCKSMYWRWRYGKTTRGCKGTWKCMRSGKASCRGYRRCKWKKCKYSMWRMKM
51              CGMMAYRKKYKCCTAAARYRKCKAKMKSKWKMMGSRGGSYRACGAYKTKKYKYCCWRMKM
                  ** * *  *.     ** * .   *..** * *  .* *.    * ** **. *****

38              MWRATCRYYYRYWMYKYTTTAMTYYKARYCTRKWAMTKWASWWTATTAYWTYKRTCTRAY
51              MWGWYTRYYYRYACYKYGYKCAKYCKGRYYKRKACCCGWTSWAWMGCGYWKYKAYMGRGC
                **    ******  ***  .. .* *.** .** .   *:**     .**.**    *.

38              RKKCRAGCKYKYGTCGAGTYTTCWGRTTWYWACCKRKKAYRMWRYKYCGGGRWMTMATRK
51              RKTYAMRMKYKTKYYARKYYYKMTKRCKWYWRYYKRKGWTAMTRYKTMKKKGTCWMRKAK
                **.     ***    .   * .   * .***   ***    * ***         * . *

38              AKYKYKYYKAYYRRWKKCRTGMTYAYRYCMRWCAMMTGYWAATTRRACRAKRMKATTRSK
51              RKCKYGCCTCYCGGTGKTGWKCGYRYRYGMRWMWCCWRYTGTAAGRTTAGTGAGWKCGGK
                 * **   ..*     *      * *** ***      * .::: *:  ..    .  .*

38              KRMMRTARWYTARYKYRGTRRKKYYYYRTGRAWYCYGARKRRKSMWAGRRWWKACCWYKA
51              KRMMRYRAAYCGRYKYRRARRKGTCYYRCRGRWYYCKMRKGGKCMWWRAATWGGTAWCKT
                *****    * .***** :***   ***    **    **  *.**     * . .* *:

38              AKYGTRCATMTCGYYGTRGWAYMGTTSCYKGWMWYRMRGSWYRRYYYYRSRGKYSSYMCR
51              GGYRYRAGCCKYCCYKYGTWGCMKCAGTYKKTAWYRMRKGWTGRTCYYRSRKKYSSYMAG
                . *  *..  .   *    *. *  :. **   ***** .*  *  ***** ******.

38              KTMYYTWSKKYATRCTRRATTAWYYTAYKMGKMMRTYGTTTKRTKTGTATYAKKRYYKYM
51              GWMYYAWSKGCTCAMYGRMYCGWYYCCYKCAKAAGCCRKYYKRYKYRYCYYGKKGTYKTA
                  ***:***  :     *   .*** .** .*      .  ** *   . *.**  **

38              AGRKCTWSWKWSYAYYRTRGYTYTACGGRCGCKGGAMKMYMCKGTAARRRYWKTAGRRAK
51              GCGKTCACAKAGTCCTRYGKCKYKRMKRAYRYKATCMKCTMMKRWRRGRRYATYRSRAWK
                .  *   . * . .  *    .*.        *. .**  * *     *** .  .*  *

38              KRAAYGCRTAYYRYGRRKRYKRRYSYMYYRKYKYWWAAKAAGSACTCARKRTTRCYAAAC
51              KRGCYATRYTYYRYAGRKRYKRGYSYMYYRKYKYATMWKMGSCRTCYCRGGKKGMCMRWY
                **..*. * :****. ****** ***********    * ...    .*  ..

38              GMAKTTTTAAGGGRTYRMRYYCCGAKAYKKTRRYYCGTTAKAWTCAYRRSKAAKRCCMRC
51              KARGCCAGTGTARRWYRMRYYTMRRKRYKKARGTCTTGCRGRWKYMTGGCKRRKRYYCRT
                      : :. . * ******    * ***:*          *.     .*  **   *

38              TCYKKTCTARRRKKWKMWMRAYMWYACWMYCGCRRYRRRYTTTCCAGSCAMMKGKGAMYY
51              YYTKKCTCGRRRKKWGAAAGRYMWYWMTMYYKSRRYRRRCCYWMMWKCTCACKSKACMYY
                   **   .******      ****   **  .******        . .  *.*..***

38              CCTCRYCACYRRKRTGGRRRRYKGGTGGGTKTKARGRYGSRKRMRKATCGYSKRRRRMYW
51              ATGTRTTRMYGAKGGAAARAGYKSAAAASYKGGGGRRYRSRGAAAKCCTACSKRAAGCTA
                .   *    *  *  .. *  **..:... *  .  ** **    *.  . ***

38              RYRMRRCTTYGKYGKRAACKKYYYKYGMRCWTWAKAASYKMKMKGCKACTGYKYARWYYM
51              GCACGGYWWCAKYAKAMWYKTYYTKTACGTACTTKWWSYKMKMKSYKRMYRYKCMRWYYA
                          .**.*    *.** * .      :*  *******. *    **  ****
```

```
38          TKKCKTTGYYCYWKRYKTKTYMGRRCAYYGRAKYRSWYTTTYCAGKTCTAKTCKYMYWWW
51          KKKYKYYKTYYYWKGTKYKWYCTRGYRTCKGMKYRGAYYYYCACRTCTWWKWYKYMYWWW
            .** *    * ***  * * *  *        ***. *    .. .    *  *******

38          TKCYYRTKCRKGAGCAAACMKYTRMRCAGYARRSGAGTGKWCKRRKKWRYMRMKWAKCGS
51          KKTCCRKKYAGRTATCGTYMTTARMAMWKTWRGSRMRYSKWYKRRKKAACCAAKWWKTRS
            .*   *.*    :. ..: *. :**      * *    .** *****      ** *  *

38          CATTYRYRSRTAMMKGYCYSRWKWWRKCRYYRGTACARKKKTKCCTRWKCACTTCGAYRY
51          MTCYCRYRSGWCMMKCYTYGAWKTTRKARYYGCCRYMGKKKKKTTAATKYRYWYYAGYGY
             :   ****  .*** * *. **  **.***       ***.*  :  *      ..* *

38          WWYGTCRRRTAARYRGYMKAAMKKKRRRRKYKKYYAWGGWYATACCKWRMKKKRGAGYRR
51          WTCRWSRGGGRWGCRRYMKTTCKTKRRAGKCKKCYMTAKTCTGWYYGTACTKKRATATRR
            *    .*       * ***:: *.***  * ** *  .   :        .***.:. **

38          CKGMTTARSYWRWKYKCATKRTAYKKAKMYCCMKKKGKAWRWYTCAYGRKYYYKSRYTGW
51          MKKMCCRRCCTAWKYKYGGTAAGCTGWTMYMYATKKKKRWAACWTWCKGTTCTKGACYRA
             * *   *.   **** . . :. .  .**   .** * *         .   *.

38          CAGCKGKRKMMYGYRMKYYCKAYKRARACTTCYTTCWTTGASMYRGGCWRCYWYYSGYGW
51          YWRYKRKGKCCTKYACKYTSKMTKAWGTSKYYCYYYAACAGGATGSSTWRYTWYYSKYKT
                * * *    *  ** .*  *   :..       : ...   .. **  **** *

38          CGAGYTYGGRKKGKATKCYYKASKYARKRYKWKARGGCGAAGKAKTACYCKYKYCCTSGA
51          SACACWCARGKKKTWCKYCCKCSKYMAKGYGAKRGRKYRTWRKGKYWYCYKTKYYAGGAG
            ....   .  ** .  *   *.***  * *  *      :  *.*     * ** . ...

38          RRMYYYYAKKKKYWKRSAGTGTTCRWRYRWKKYYKCCACATCKGGWRCGMWKKWCYKKCC
51          RRMYYYYRKKGKYTKRCCAGAKCYGWRYGTKKYYKTTMYRCYTKRTRAACWKKWTCKKYA
            ******* ** ** **... ..   ***  *****       .   *.. ****  ** .

38          GARTWCGYACWAATKTTYRKRKRKRTGYKRGAGKTCKSWKTWCCWCMGYYKYARGRTYMG
51          KCRYTMCCRTATGYKKYTAKRKRKRKKYKRKTAKGTGCWKWWYTTTMKYYTYGGARWYMR
             .*        :. *.   ******. *** :.*   .** *    * **.*. .* **

38          CATGKAACARRCTRTGKCAAGYTGAGTCCAWRYRRGATRMAYYKKAGWKRYACCCTATYA
51          MRWAGGRYTGGTAGGRKYGRRYCAMRAMTCWGTAAKRGGMWTYKKTAWKRYWYYTCRYTM
               . .  :   :   * .  * .  :  .*        *  ***:.****

38          YAWKYRSKGGTYGRYRKKGRKKKYKRGAYKKGRKKYKMRRYKMKMRYKKKRTCRKRRYGM
51          YWTKCACKTCCYARCRKGARKTKYKGAGTGKKRGKTKMRGTKCGCACKKGRCMGKRRYRM
            *  *  .*   *.* ** .**.*** ..  * * * ***  *     ** *   **** *

38          CYYGKKKYAGCYRWMKKRWGGKSTKGCGYYMWWYRTKTCYRKTYKKAGKTTCAGKKRYCT
51          MTCKKKKYCTTYAWCKKGWTCKGCKAYKCTMWTTGCKYYYGKCCKKMRKYAGGAKKRCYY
                ****.  * * ** *  *. *.    **    *  * *  **  * : ..***

38          ASGAWYCCTWCYMYWYYRYAYYTAKYYRAMKCCYGYYYKKTRYKKAYKTRCAKWTARSGY
51          WSTGTYYMWAMYMYWTYRYGCCWWKCCRTCKTGYACCCKKARYKKGTGCGYRKTKGGGRT
             * .*     **** ***.    *  *: *  *.   **:****.      * .. .

38          KYCTRMWSYWAAWGATMGMGKWYCSTKYRRMYTTCTAWYGAGYCCRMCCKCYGCCAAYCT
```

```
51                  TCYKRCWCCAWWWKGWCAMKKWYYSWKTGGCYKGAYGTCTTAYTGRMYTTACRYMTGYTC
                    .  .* *.    * .  .* *** * *    *. . .   :.*  **  ..    :.*

38                  WMKYACYYGSCKMAWKKCSAMYRCTGCRRTACKMGKGYMKYTCGAATMCKYCYRGKTARR
51                  WMTTRYTCRGYGCCWKKYGGATRYYRYARYRMKCSTKYMKCWTRRWYMAKCMYRRKKRGG
                    **.      .   .*** ..  *     *   * .. ***       *.* ** *.

38                  GKCRKCAACRKCRKYATTCAKKWCRAKRYRGWSGMKKKGCTTKRRSKKCGCSCKRKKTTS
51                  RGTGKYRMYRKTGTCTCGTTGKWYAMKRYAAWSCAKKGKMKYTGRSKKASMSTKRGGYYS
                        *    **  . :   : **   *** .**  **   . . ****.. * **    *

38                  GYRGMRGGRYKRYYSYYWGRMRYRRTRTACCRYKCTKRYKKWRYYRYKRYKRATAKTKMY
51                  RYRKMRRRATTRYYSYCWRAMRYGRCACGTMRYGYKGACGKWRTYRYKRYKRGWWKWKMT
                     ** **    .***** *  *** *   .  **  .    *** ********.  * **

38                  GCCCTTKTYYWKRMKAACACAAGGMACRWYCATGMCCTYKRMAGWKKARRKKKTTWTCRK
51                  KMTMAGKCCCAGAMKGGGGTGGRRMTTRACAGGAMYTCCGGCGAWKGGGRKKKGCACTAG
                        : *      **.. . ..  *: *  ..  .*       ..** . ****

38                  TWTMGKYYTKSAASRRYSRCSAYATKYGCTAGAGTRRCYYRARTYRWWMKYRKKKYYMWA
51                  YWCCRGCCCKGWTSRRYGGTCTYCCKYKAWTSGTCRGYYYRRGKCATTCTTAKKKYYATW
                     *       *. :****.  .:*. ** . :..  *  ***  .     .  *****

38                  TCRCATCGKRGCTRCKTCAATTYRKYRGTTTYCKAARKCKWGGTWTCYYYMMYRCKRKRR
51                  KMRYMWTAKGKTCGYKYYRRKWTATCGRCGKTYGRRAKMKARAWTCSCTYMMYAMKGKRR
                    . *    .*      *    .   .     .      * *  .   .  ****  * ***

38                  GGKCTATTYCKCTYTGWWWGRSRCKRKGTCRGGACMCRCCCCRRKRRRKRKTMTATCGAA
51                  ATKYWWKGCYKAGCKAWTASRGAMKRKKWYARRGACMRTGTARRKRRRKAKAMKRYYRWW
                    . *   .   *.  ..*  .*.  ***      ..  *   .******* *:*.

38                  GGWWKCAGTRACWAKTKRMKKTMKGRRRCKKKSGTYYCMKYYWYACMGGTCCTKTATCYR
51                  KKWTKYRRWATTWCKWKRMKKKMKTRGGMGKKSAYCCMMKCCWYWMMKRYYMYKCGCACR
                      * *     : *.* *****.** *    ***.    **  **  *      * . . *

38                  WRKCYRKKSRRYKYRYMKATCGKGTKTGTKATKTAATAYKCCTSATRCSCAYKCYMSKKW
51                  TGKACRKGSRRYKCRYMGGAYKKRKGAAAKRKKGGRYMYKATCGMYAYGYRTKMYMSKKW
                      *. ** ***** *** .:  * . :..:* .* .  **.  .    .   * ******

38                  RTKCARWWRAWKMGTACGMWRGGKKKKCGRGGRKYYCKMKKKRKAGGCYTWKRYYGRYTK
51                  GGKTRGTTGWAKCTAMYSCTARRKKKKARRKRRKYYYKMKKKGKTTATCKWKGYYKRCKG
                      *        *  :  .     ****. *  **** ***** *: .  .** ** * .

38                  KCTKKAAAGRWCYCRKMKGATCGRMYKYYKRRK
51                  KMKKKRRWRRWYCYRTMKRWGTTRMYKTCKRRG
                    * .**    **   *.**     ****  ***
```

The pairwise alignments of two accessions 40 and 42

```
40      MWRMKKGCSRRATGYYWRTYWGGSYKMYRRKKGARTYKWKTAKYWGYYWYWKYKRRASWK
42      CWRMGKRTCAAGGACTWGWCWAKSYTMYGRGKKCRWYKAKGGKYWKYYAYWKYKRRGSWT
         *** *  .  . .  *   *. **.** * * .* ** * .*** ** *******.**.

40      YRYAYGSMMKYYRRYKACCRAWMRRMMAGWKWRRRRYWMMTRYRWRRRYYWKYAYMMCSW
42      TRYRCTSMMTYYRRYKGTTRRTMRRMMMKAKWGRRRYWMCYRYRWRGAYYWKYGCAMMSW
         **   ***.******.  *  *****   ** ******  *****  *****.  * **

40      MKYRSCMMKWRTRKYYRWWRTYYCMYRYYWRYKMYMYGSYGCRKAAGYYYKTWYYYTKYY
42      MKCRGMMAKWGYRKYYRWWAYYYMCCRTTARYTAYCYRGTRMRKRWRYYYKCWYYTKKTY
        ** *. * **  *******  **   *   **. * * .   **   **** *** .* *

40      YKRKAKMMSGAGMCCRATRCWYKGTCYMWRYYRRYYKWWKKWMRWKATKYKRKYYRSRRR
42      YGGKWKAAGAGAATTRWGGAWYGRYMYMWACYRRTCTWWKKTCAWKWKKCGRKYYRSRRR
        *  * *  ....   *   .**    ***  ***  .****   ** .*  *********

40      GWRRCKWMYRCRKYRAGMYMRCSCYYCMWRKYMRRTYRKWWRYYRAGGCAYRSWYAYRGY
42      AWRAYTWCYRYRKYACTMYMRYSYYYMMTGGYMRAAYRKWARYYRCATMCYRSWYWTRKY
        .**  .* ** *** . **** * ** *   *** :**** ****..  .*****  * *

40      GYRYCRGATTRTTKCYWMKRYKKYRATYYRYYWRYYGRYYRYMWKYMRYCKKWTMWWYYK
42      AYRYYRAGCWAACGYTWAGGYKKYRGYYYRYYWRYYTRYYRYMWKYMRYYKKTAMWWCTT
        .*** *..   :    *   *****. ********* ************ ** :***  .

40      WKACGGWMMYWMYKKYCACKARWTTAYRYGWYRTYKKYCATTCATAAATAGCGKCYGTGR
42      AKMATTWMMCWCYKKTYMTGMGAKCGCAYRTYACYGTTYWCGYRCGCTGGRYRGTYAAAG
         * .  *** * ***        . .  *  *  * .        ..: .     *.:.

40      ACGCTSYGATGGCCGTYWTAACRYRWAAAAYAACAYKAACGYYKACMKAWSTCCGMGCCA
42      MYKTYSYKRWTKMMKYYTGGRTRYRWTRTGCGWSRYGMRMKTCKWMMGRWSGGTRMKMYR
             **         *  .  ****: :. . . *       *  *  **    *

40      GATTKGCMTGKYRMKRCTCTAGGKAATGRGWRGKMKCCRMKKWYTWKSYMRTAATGATTA
42      RWYYKRSAASTYGMKGYATAWAAKTTGCRRTGRTATTYRCGKTCCTKCYMRKCGYRRKKR
            * . :..* **  : : ..*::  *    . .  *  *    *.***...   ..

40      RSYKATAAKYGTTGMARTKCYGCRKYCWYMYGAAYWYKCYCKGGKAYWRYATAKKATARK
42      GGYKTATCKCKAYSMGGKKTCATRKYMTTATRWRYWYKTCAKRRKWYWRYRYMKKRYMGK
         .**:::.*  : .*. .*  . ***        ****  .*  * ****   **    *

40      WMRKTTAKAASAMCCTRKAKKWGRKCYSKMMTTTGTKCWWWRWGRWTRAWKAGAAKYAGC
42      AMRKCARGGGSRAYYYRGRKKWARKYCSGCCYYYRYKAAATRTARWGRMAKRRMRKYRRT
         *** :  ..*     *  ***.**  *        *.   * .** *  *    **

40      SAAGWGAKRRMYKTAAAWAARTTACMMKRRRCAMCKWMKCGTGRTMKATRRGSKWYYYWK
42      GCCKWRRKGRMYKGMMCTTCRWCCYMMGGGRYRMYKTAKTTYRRCMKRYRRRSKWYCCWK
        ... *  * ****   . :.*  . **   *  * *  *    * **  ** ****  **

40      GTTCTRKKYKMMAWKGTKAAYCKCRRWYYTTCTTGAGKYRRYRYMYKWAGTTACGARRYT
42      KGATARKKYKAMWWTKCGRGTYKYRRTYYWWTKYRMRGTGACRYMYKWGKACCYSWRRYY
```

```
        : :***** * *.    .  * ** **   .         ******. : . . ***

40    CKTKYKGKARCTCWWGYMKKCGGGCTKYRKSTAKRTTYYGRYYTCRRRATKYYRMGKTKR
42    MKWKYKRGGRYYYWWAYMKKTAAKAAKCAGSYWTAYWYYAACTATRRRWYKCTRMRKYGA
       * ***  .*   **.**** .. .:*   *  .   **.   : ***  *  ** *

40    TYRKGMCTTTGARWYWYYGTATTCRGRTYYKSYRTRAKYKGCKCTKATTCKTKRCTCAGG
42    YYRGTMYYKCRMAWYTCTKYWKAAAKRWTCKGCRYACKTKSYGTGKCCCTGKKRGCMMKS
       **  *  .    **      .:.  *   *. *  .* *.    *.    .**     .

40    TKRRMAYWKMTGAGKCGTATGYCYAGTGTYGGCMCTTGGYATCKTGGTRCRYWKKTTWTC
42    CKAGMRTWKCYRRKKYKKWYAYYYGRWKWCRRYCYGWRACRKYKCRRAATGYWKKKWWYS
       *  *  **     *  .  .* *.             .  . *   :   ****. * .

40    YAKYCTCCYRYTACRATAWCCCGSYYKCKYYRATKARCRTGTRGAAAAGTAMYAGYMMYT
42    YRKYYWMSYRYCGGRGAGAMYTKCYYKTKYYRRGGTGYAAAAGATGWGAWWMYWKTMMYW
      * **   .*** . *.:.     .*** ****   :   :.: .:. ..  **   ***

40    AGCYCCRYKRKCMARYMRWAGCCTTAAGGCRTYRCCMMCWTATYCCCCCGRGGCCGACRY
42    WKMYTYRYKRKYCWGCMRWRATGAACGTTAAYYRYMMAMTCGATYYMTGARAATAKCARY
         *  *****     ***  .  ::..  .  **  *    .:      .*.. . ..**

40    YTCCACKAYYCWMSRYRAYWMRCKRCTTTTRYSWKMSWKKKKKKYRAAMKYWRCGKMKKY
42    YYYYRTKRYYMWMSRTGGCWMRYKRYKKYGRCSWKMSWKKKKTKCGRGCGYWRMRGAKKY
      *     * ** ****  . *** ** ..  * **********.*   .  ***    ***

40    TYRCATATTKRRTKAYRKTYYYTYKACRWKKKYTKKRTKRYGCAKRACRCTATTTRCTCT
42    YYRTGYGACKRRYKWYRKCYCTWYKGTRWKKKYWKTRWKRYAYRKRGARYWRYYYGMYAC
       **  . .: *** * *** *   **. ****** *.* ***.  **..*         .

40    RYYKKRKGGKCSWARYYGYYYRYRKWCWWRKMWTGKRKKGCATTMSKKKKKGGKYSRTAA
42    RYYKKRKRRKYCWRRYTRYYYGCGKWYWWRKATWRKGKKAAWYWAGGKKKKKRKYSACWR
      *******  * .* **  ***   ** ****    * **..    . ****  ***

40    KGKWRTMMYTASTKRWYRWKMMRKYRRTKTGRGAYKCTACKYRKYYRMMMTACYYMCCCC
42    KKKTAGMMCYRCYTGTYRTKMMAKYRRCKYRRTGYKTCRMKYRKYYRCCMWRYCYAMYMY
      * *   **   . .  ** *** **** *  * .**    *******  *    *

40    GWRGYRYGCACMCYWTKTYCCGTCYGWYKKGATCYMMYRARCCCTCTKCRGCGTYKTGKG
42    RWGKCRCRYWMCSYWYKCYMMAAAYRWCKKRMYYCMMYAMRSYMYYAKYAKYACYKYRKA
       *   *      .** * *  .:.* * **     ***  *.    :*   . **   *.

40    KTTTACGTSSACRKKARWYAAYARWCKKGTTTAASYWCCKMTKMGMYYYCGMATAAWCTK
42    KGCKMMRKCGCYAKKGRTTCGTRAAMKGRKYWWRSYWSSGCYKMRMYCTATMCYGCWSYK
      *  .   ....  **.*  ..     *  .    ***..   ** **  . *.  ..*. *

40    KAATSKWWSACTGKKWYRMWRYYYKKMMKCACAGAKRCTCKYTRTGYRTYAYTCTRAWYY
42    KRRKGTWWSTTAKGTAYRMWRYYYKKMMKTTAWRGKGYYMKCWGYRYRYTGYCTYAGWYY
      *  ...***: :  . ************* :.  .*    *   **   .*     .***

40    CCRSACGGYYTCGWRCCTAAAKTGGYAYAAKRYMKWMTAATRRKMKYRTTKTCCGTYMTG
42    TTGGWMSKYYGTTAGATCWMTTAAACCYCTGGCCKWCYWMYRGGAKTRCAKWYMTWYMCA
         .  . **     .    :.:.. .*.:    **     *   * * :*     ** .
```

```
40              GWAKYWMYRKWTCKGGCGYYKMKKWKAYYATGGATGYYYATTCKYCCTWCTTAWSKRACG
42              AWGKTWMYAGTWYKKATAYYKCKKWKWYYWYARGCRCTCGCYMKYSMWWAYAGTGTRGSR
                .*.* ***     * . .*** **** **   . .      .    **.   *. :. ..*..

40              CATYAWRWKTWKTSKAGYGTAGKAKWYRKCTGAATRCKKSAYKRAGATRGCCCMYYYKCA
42              TRYCRWGWKKWKKSKMKYRYRAGMKWTGKAWKTCCRYKKSRTKRRKMYAKTTMCYYYKAC
                     * **.**.**  *    .  **   *.   :. * ***   **          ****..

40              AYSRSWGWYYTTAGCAYRMYWYSYRCGTGATMTCMARATWMGATAYYRKGKCGCKCRACT
42              GYSRSTRWTTKYRTMMTRMYTCSYRYACARWACYCWGTYWMKGYRCCGGKKYKYKMRCAG
                .****  *   .       ***  ***  . .         : ** .          *    * *..

40              GRKYMACMKTCAAKSCCYAWGRRKAWGKKCCWKAKGTKRYGWYMRAKCGRRCGMMTWYRR
42              AATCAWYCKCYTTGGYSYMWTRAKRWRKTYYWKTKRKGACRAYMRRTYRGRYRMMCWCRG
                . .      *   :: . .* * * * * * *.  **:* .      ***  .    *   ** * *

40              KTAGCKKAWRMYRRGAKTTGWKCATAKYYYMYYMSCCGGCGARYMMYRCCTWKATCGRRY
42              KKWKGKKCWRMYRATGKCYAAKYRWMKYYYCCYCCYMATTRWGCMACRYYKWKWYYRRRY
                *.    **.*****   .*   . *     ****   *  .  .         *   *   .**     ***

40              CRMWRYKYRYRMGGYRTCCRWGMCRRGACACTCGYTCGTYAYRRGYYAYAWTKAYWYTMR
42              YRMWAYKYGYRMSRTAKSMAWRMYAGAGMGTCTTCCTKWYRTRRRYYGYWWYKGTTCGMR
                 *** *** ***.    ..   * *    .. .           *   ** **.* * *.      **

40              YYGKCAAAYYGYTAAAACARMWMRYRSWYRRYTMGYKGCKAKRYTAGKCTCCRYSKGKRK
42              YYKKYRMMCCRTGTCMTSRAMWMGYAGACGGTKARYKRYKWTRCYRKKMCYYRTGTATRK
                ** *         :. :.  *** * .     .  **   * .*     *     *  ....**

40              CTCKKTRKMKYRRKRTGCSYYSRGYKKAGTGTTTTGRAKWRMYSGWRCATCRKKMTTYYK
42              MYYGKYRKMKYGAKRWKYSYYSRTTGKRRYKYYGCCRRKWRMYSRWRYWGMRKTMCYYYK
                    * *****  **    *****    *         * ****** **     **.*  ***

40              MYRMAKCCCRRRRGMWMCCCTCAKTWWAGWCAMRKTCCGCGMKYYATWRRTARACAAKAT
42              CTAATKATSRRRGRCWMTGYYYWKGTAWRTAMMRTATYRAKMKTYWYAAAYWRGYRGTWK
                    :*. .***   **      *       . **.:    . ** *         *.   .. .

40              WCTYTGKMMYMRAYYGKYKTAYYYRTCRCTSCKATACGGAWTAMTRRRTTMTAKRCGTGG
42              WMCYYRKAACMGGYYRKCKKGYYCGCYRTCCTKCYWYCARAAWMYRAGWYCKTKGYRKKR
                *  *  *    * .** * *..**     *  . *.     .  : * *      .:*    .

40              WGCYKAYKRRCRRTYATCGGYMRTGAGAYRGAGGTMMCWGCYGYYTYTCTCYKAMGRKKG
42              WRYYKTYKRRYRRGCTCTRKCMRCACRMCAKGASKMMTAAACRYCYTWYYTCGGCAAKGR
                *  **:**** **  :      **  ..     ....**  ..  *           . . *

40              GTGTRMGRCTCYTGTGCCKYRCKKWYYATGAGMRKYYRTGCRYGMKRRKKKRYMKGGMWY
42              SKKWRMKRGKSYKACATYKYRYKKWYYRCRGKCRGCCGCATGCKMKRRKKTGYCTRACTT
                ..  ** *  ..*.. .  *** *****    .  *       .     ******. * . .

40              MYYMYKKKTACTRMRRRKYMYWCWAGCRATWRAMCTYYRKKKMYCWKGCCRACKGCGKKT
42              ATYCYKKKYWMKGMRRGGYMYWTTTAAGGWTGRMTATYRKKKAYTWKRYTGGYTTMRKGA
                  * ****    . ***  ****   :.. .     * : ***** * **     . .    * :
```

```
40        ATTTGTCKKGKRSKYYCWKYTTARTGKCKMGACRCTYSYWYKAYKAMCTAYKAYYKAYMG
42        GCGWRATGGTTRSKTYYWKYCYTRGRGYTCCGTATKYSYWYKGYKMCMWCCGMYYKGYMK
          .    :    .*** * ***  :*    .  .   .******.**    .   ***.**

40        AKKYRYGAYWRRWWKWGYMCGSTCACKTGRYTGMMCAATCAAKAAAKTCYWYRTYCMRAW
42        MKKYRYKWYTGAWWKTKYMMRGCMGAKACRYYRMMGTGCYRRKGRMKGGYWCRWTTARWW
           *****  *   ***  **  .  ..*: **  ** :.    *.  *  ** *    * *

40        GGTRKRRRYMATYMYGTARYACTMAWKCCRRKASRRYCRCTCRCWTGTAWGGTKWYYGTK
42        KRGGGGRRCCMGTACRKCGYMTGMRAKMYAAKWGGRTTGSGAGAACACCTKRYKWCCRKK
                **        .. *   *  *    * . *   . . . .  .    **   .*

40        GCYCAMGYMYYATATMRAMTTYYKKATCWAKTTWYMYYYRGWWCTMRGTYRRRRACRRKA
42        KMTYRARTCYCRYCYMGGAWYYCKKWYYWTTAATYATYYRRTAMCARAGYGRRARYRRKR
                   *   . * .   * **   *:.:: *  ***      *. * **   ***

40        MAWRYMKCGCCCACCYRWYTTTKKYYCGAYYKKARRRKGKRYYCCKTAYKKTAAKGTRAR
42        ARARYMKTRAMYGMYYAATGCGKKYYMRRYTKKWGRGGAKRYYYMKWCCGKCCGKKYRMG
             ****  .  .  *      ****   * **  *  .****  * .  * ..*  *

40        RYRWYRYMKWGWYGGGKGKKYRYKAKWCGRYKTWCKAKWTAAAACTKGWYYYCTTTGKKK
42        ACGTYRYMKAKWYKRRKKKKYRYTRGWGARYTCWMKCKTYWMRRYYGRTCYYACCYRKKK
              *****  **   * *****.  * .**. * *.*            **.    ***

40        AMYRGWKRGTGSWTYYRMRMYSWRWKAMWYAAGKRGYTYAMKKKRKTYSRGGYWCRKRTG
42        WCTGKTKRRWKSWYYYRMRATGTAWTRMTTWRRKRRYCYCATGGRKYYSRRACAAGKRCA
                **   ** *****  .  *. *     ** * *. .  ** *** .  . ** .

40        RRRMCAYWYRRCYKYRYKWATACCKTWRGAGGGKKKKMACGCTTCCATSCRGAGYRCRSK
42        RRGMMRYTYAAMTKYRYGTTAGTGGGWRRRKRRKGKKMWYRYKYMYGCGTRRRACRYRCK
          ** *  * *    ****  ::.    **     * ***    .  .  . *  . * *.*

40        GCKYKKKAKARGRKWKYTYGTGKTCCAKTRTMYKKYSMKKAYTGGKGWYYKYWWGTYMKR
42        AAKYKKKRKWRRGKTGYYYRARKWSTTKYRCCTKKTGCKKGCYRKKAWYYKYWWRYYMKR
          ..***** * *  *  * * : * . :* *   ** . **.    *.*******  ****

40        YKGGGYCTMGGCYRKWAGTTRMRRGYKSACGGYKKGTMYTTKKCMKCGAAGTAKYMRMWK
42        YKRRKYYWMKRGYRTTGRYKACRRRYTCRYRKCKKRWCCCGKTYMGMTCWACMKTMGATG
          **   *  *   **. .  .  ** *..     **      *. *   . .  * *

40        RKKCKSRCGRYYYKGTTCRGTCTWRCWYWKYRYMCYGWKTGACMTKKWYKKWKKCTCKKT
42        RGKTGGAYKGYTTTACAARTCTATRYWCTKYRYMMTSTKWRRYAYKKWYKKWGKTATKKG
          * *  .    *  .. :.*   : * * *****  . *      ******* * : **

40        CTGYRKCARCKRKTKRCYRGGMYYYRRYKKYTGAWAYMTRCTKKCAAACGYTRGCTRKMC
42        TCKYRTGTGMGAKYGAGYRAAMYCTRRYKTYYRWTRCAYRMYKTACGCTKYKGRTWGGCY
             **. :    *    **..**  ****.*        *  *.....  *.

40        TKCAMKYRRRTGRYSKACAKKYCACYTCTYYWGTTCTCAACAAGCYCCKKGCGGMTGGYR
42        WGYWCTYRRRWRGCGKCYMKKYSRYTYYWCTWRYYYYTCWTGGATYYMKGSMRKMCTTCG
               .****    .*.  ***.        *      .  ... *  * .   *

40        ASYKGGYSTTCTKAYYCAATATMMMCATKCCWYTRYYKTGGGKGAKGATCTCTMYYYAMY
```

```
42              GSYKRRYSGGMCGCTYYWWGRKAMAYMKKYAATCRTYKYKSRKSWKRRYYKYYMYCCGCC
                .***  **     . *     . *   .* .   * **  . *. *    .  **  .

40              GKKAMRSSSRRYMMMYRTKTKYATGGTAAAKYYTTKKTAMCGCYARKKYYARRRGMATMR
42              TKGGMRGGGAGYCMMYRKKYGYRYRRKTRWKTYCCKKYMATAYYMAKKTYWAAGKARYMR
                 * .**...  * ****.*  *    .:  * *  **    . *  ** *        **

40              RRGAKCKMCKCCCTRCATKWGARARWTMTYGCTKCKAYWYRKRRRRKYRCSMRYMYTRKT
42              RRKWKMKAYGTYMARTGYKWRTGGGTKAKCRSWGYKMTACRKRGGRKTRMSMGTCCGRKK
                **  * *      :* . ** : .  . .  .   *    ***  ** * **     **.

40              KRYWTTKYKAYSYRKTYYRRWMYYYRYKKTGGRKTKYCATKYGCYAYRWKKWKWKYYAKM
42              KRYWKYKTKWTGTRKYYYRRWMYYYGCKKWRRGTYKYAMYKYAYYMYRWKKWKTGCYMKC
                ****. * *  . ** *********  **    . **.  **. * *******   * *

40              RCCKAKYYYAKWGYRATYWTCAWTWRCGKKCAGAWRATAARKKTCGGTKYAATKRYMSWG
42              RYMKRKYYYMTTKYRGCCACYGTCTAYRKKTWRRAGGGTGAGKAMAAAKYGWWKRYMCTR
                *  * **** .  **.     .      **      . :.  *: ..:**.  ****.

40              RRWYCTRWGGYRKGGAAKYGRGRCRGCTYGKCAGCCGKKCKWMRRYYSCTGTAYYRWTTG
42              RRWCYGATRCTRKCACRKTKGAGTRTAATKTTTCTTKKGTKTCRRYTCYCACWCYRACYR
                ***        ** .. *   .  * .:  . :    *  *  *** .  .   **

40              RYKYYMRYRWYGYCRRRYMARGACKGMRRCCKAKRYMTCRRAGYCATCSARYMATKYRCR
42              AYKYTMRYAWCRCARRRYMWRRWYKACGGMMKWKRYMGTAARRYYRKTGCRCCRYKCRMG
                 *** *** *   .***** *   *.     * ****      *  . ..*    * *

40              MKRYTKAGKKYRYGAWKTKCAYRAAACYWYCTRYGKKAATTRGKKGGMMKRCCRYAWRYR
42              CKRTCTWATKYRYRMAGCKYRTRGGTSYWYTAGCRKKCTCYRSKKRAMMTGYTGYWWRYR
                 **  . ..****     *   *..:.*** :   **.:  *.** .**.    * ****

40              YMMGTAKRYRKSRSRKKAKKYKCAATTGGGWKYRTCKGTKGGWRRKRCYMKYRYKRRKMR
42              YMMKWMGRCRKCGSRKKMKKTGYRRWWRKRWKCGYSGKKKRRWGGGGYYMKYRCKARTCR
                ***    * **. **** **          **   .  .*  *     ***** * *. *

40              KGTAYRKGRKKCYCGKRYSGSWKTTRYKRYMYTKCCARYKKRGYCYYKCKMSRYRMRYKY
42              KRKMCRKKRKKYYYAKRYSASWKAARYKGYMYCKTATRYKKRKYMYYKYKMSRTGMRYKY
                * .  ** *** * .****.***::*** *** * .:***** * *** ****  *****

40              TSGRSKYGTGWGCTKCGRMCTATKKMYRYYYRWGGKGAWRSKRGGGRRGTGAYYWGGGCM
42              GGRAGKCKCTAATCKSRGCMWWWKKMTGCYYRWRRKARWRSKRRRRRRKYRRCYWRKRAM
                 .  .*     .  *.       ***   ****  *. *****   **     **   .*

40              RYTMWMATAKYKYMKACCWCAGRRASKTGTRGRRGKMTTMWKRYYRKRKKYTATTTCRMM
42              ACKCWCTGGKCKCMKGATTTCARGGGKKSWRRGRTGMYYCTGRYYATRGKYYWKAKGGMA
                  . * : .* * **..   ..* ..*.. *  *  *     *** .* ** .:.   *

40              CKTGCASYKYWTRGKGAGGCWWWWRRTYRWMACGCYYTTKWYRTSRRACTRGTRSMRTKY
42              MKCTAWGTTTAGGAKAGKAYWWWWRRGYRWMWMRYTYCCKWYRWSRRGYGRRWACAAKGY
                 *  . . .    .*.. . ****** ****     *  **** ***.  *   .  . *

40              YTRYRKKYRSCCAKMYYMAAWKTAKRYMMYCYTYRKKRTRYCMGKTMYAACCGCAYWKKC
42              YYRYGKKYRCYMRKMCYMRRWGCTKRCMMYYCAYRKKAKRCYMRGWMCGTYTRYTYWGKM
```

```
        * ** ****.   ** **  *  :** ***  :**** .*  *   * .:    :** *

40      KAKAGTRGCCCYCGGKTRKARACRCAGWATRRKCMTAYKCTGMYYWKRRKGKGRKGAYKT
42      KWKTRYRAYTGYYRSKKGGRRRTRMRRTMKGGKTMGGYGYKTMCYWKGRGRKKAKKGTKK
        * *:  *.   *  .*.   *  *     .  * * .*  . * *** *  *  * . *.

40      CMKRKARGGTASRTGRTCYWAARTARMGAYKRYYRYAKTCKWRYGTGRRYAYCCCWMCGA
42      TCKRGWRRRWRSRYRRCYTTRRRYWRMKRYTATTRCWKYYGTACRKRRRCWCAYMWMYRR
          **  *    **  *      *  **  *.   *  *       . **   .  **

40      GKAGMCWRAYKYAATMGARKCAATGGGAACWMSCTRKYRRACRYCMRKKYAGAKKAACTG
42      RKWRCYWARYKCRGGMRWGKMCGKRKRCGMWCCYYGKTRRMYACSAGKKCTRWTGRGYGA
         *    *  **  . *   * ...   .. * .   * **    .  ** :  .  .  .

40      RYWGAKTSYKGTCKCAKAYATKKGTTRYKWYYGARTACGCCMAGRYAKYYY
42      RYWRRKCSYKRYMKMMKWCRYKKTGYGCGWCYRWRYWYRYYCRKRCMKYYY
        ***  * ***   *  *    **      * *  *         *  ****
```

The pairwise alignments of two accessions 40 and 43

```
40                  RYTGRTGCMKYYRRWYACCKYAMWRWRYCCCKAGWWMKACCTTCAGTRYYTGRYKTAKRG
43                  GCCAAYAMMTYYRRWYGTTKTRMWGWRTATTKMSTTCGTYMCCYGRKGCCWRAYTGWKRT
                       .  . *.******.  *  ** ** .  * .    :     . .      *.  **

40                  TATWMRGCRARCTRCYWYYKWMCGATYGMGGTKYRRKRKCGCYRGAKMYKAYWGAWYRAG
43                  GWKWMRTTRWATAATYWYCTWCSATYCTMKRKGYRRGRTAAYTRATKACKRCWKCTTRTR
                      .***  *   :  *** .* ..:   *  . *** *...  *.:*  *  *  .  *:

40                  RRYRSKWWRYMKKCWKKGKGCYYGAKWYKWRYYGTAGGTYWCRTGYTAARKYKRKMAWYC
43                  RRYGGGTWRYMKKYWKKAGRTCYKWKWYKWRCTAATATGCWYACACKTTGGYKRTCWAYY
                    *** .  ****** ***.    *  ******  .::.   *   . .::  ***.   *

40                  WRYKWASTATRMYYYWKYWRRCGWKCRYRYCYKYMYKRYYMYWRYCGRYYRWWYCAGGKY
43                  WGTKATSWWKRMYYCAKYWRRTTWTTACATATGTMTGGYYMYWGYMCRYYRATYMRRRTT
                    *  * :*  .****  *****  *.     .   *   ***** *  ****  *    .

40                  MRYTCYSYAWSYMMKMKKYTTARCRRYKRRKAKCGTMYYRYKRMRGGYYWMYTYAYGTTG
43                  CGCYMCSYMWSCAMKAKKCYWWRYGRYTGRGWKMRGAYYRYKRMRRTYTAMYGCRYACCR
                          ** **  ** **    *  **. *  *    ********  *  **   *.

40                  RWRRGSYMWYMYYKRRRRMCYWRRCTGTYGSGRGAYGMMMWKRYRYGCYGGKRAGCTGTG
43                  GWRRKCYMWCATCTGGAACYYWRRAARAYRGKGKGCAMCAAGRYRYKMCRKKGTATKSKR
                     *** .***    .      ****.: :* .   . .*    ****     * :. ...

40                  CMRAMCARKRYWTATYYYKYKTTTATKGYTCCAWWYYGTKATGTTRTRTAWCSYARRRYK
43                  TCRMMYWRGGCAYRKYYYKYGWYKMKKKYWYYRTATTKYKGYSKCGARWWWYGYRAGRCK
                      * *  *      .*****   . .* *          *. ..  :*  * .*   * *

40                  TTWGGKYKAMMAGGYKYRWYCKGGMTRCTGGTYYCCTGRYYKCCCRTATCACTMRSAKYT
43                  GKTKRGTGGMMWRAYGYRWYYKRRCCRYCRRYYYTAARACCGYTTGKTAYRYWARGRKCG
                     .      .**  .* **** *    *     ** .:         .::     *. *

40                  AYTTCAGWAWRYKAGYTTAMWGYKYKAMWGAWYYGTYRKGCTKYTAYKRRRMTRKATKRC
43                  RTCCTRSAMAAYKGRTWYRMTAYKYGRMWACTCTKYTAKRTWKYKMYTRGRCYAKWYGAM
                          .    **.     * .***  **..       *   **. *.* *   *

40                  TGRYMTKTCYCTWTCWGMRKCCTWYMYCYTCGTSRWRCRYRMTMTGKAGYRRACCRGKTS
43                  CARYCCGCTYAAACTACMRGTTGWYCTYCWYRKGATGTRYRMCMCAGRKTAGRSMAAKGG
                     .**     *.:     **    **       ..    **** * .       .  .* .

40                  RACAKRTTYWKATTCYWCGTRYRCYARRKYWYGCRYYWATKYYRYKRKTAWYKYCYTTYK
43                  RRYWGAYYYWKRWAATWYRKRCGTTWRRKYTCRMRYYWRKKCCRYKRKWRAYKYTYCCCG
                    *       ***  :. *  .*     ****    ****  .*  *****   *** *

40                  GAKATWYATAGCTTMCGKGGRKMKKRTGRKKACTGATKSCGRYRCRSKCWAWAAKATTYT
43                  RCTMKATRYRKMCCMYTKKCRKMKKRCAGTKRAARRYKSTARYGYRCGMAGWTGKRCYTY
                     .. .         *  *  ****** . .* .:   ** .**  *.   .*:.*

40                  CKKACTRATAKTAKYMKYKWRMTKYSMTTGKGTAGKWYAWWTCCTWKYACGAMGGYMYMC
43                  SKKGSKRGCTKYTGYMTYKTRMCKTGCKKAKAKMKKWYGTWYMAGTTYCTAGCKRCCCMT
```

```
              .**...*. :* : **.** ** * . ...*..  ***. *  .  .*. ..      *

40            KRWTKRKYRKWYRMWRTGTTTTKCGGKRKMYSKKTRCCAKTGACKCAKSRGGAYYGKTCT
43            TATCKRKYATTCACARKRWKYCKYRKKRKCCSKKAGYMMKWRRTKTTKGATTGTTRKCTA
              .   **** .     *.  .  *   ***  ***:    *    * :*.   .   *  :

40            CKKKKWACCKKGYKWWKYCGTYSYKYWYKYAYRWKWRGWWRRWMACYGCKAGKWYAWAWY
43            YKKTKTRYYGKKYKWAKCYKCYGTTTATKYWCGATTRRWWRRAMWMYRYGGRGTCWTGWY
               **.*     * *** *    *. .    **    . * **** *  *   .      .**

40            RARRRAACGTRSMRACCTGYKTWYCRYRAKKYRTKKKRYYCGCGTACRTRKGMYTYCWKR
43            RMRRGGGTRWACAATTYCTTGKTYYRCGTKKYRWKKTACYYKYRWRYAKRTRATWCMTKG
              * **  ..    .  :      . * *  :**** **.   *        .*.       *

40            TACKCKTYRKRTRWKWKKMKWYYCKKTTCRCMRYGTYCMYCRGWRTCTMYRAGCRRATYY
43            GGMKAKYCAGRGGTKWKKAGACCYKKKGGRYMRYRWTTATMRAWRYYYMYRGTTGRMGYY
               . *.*    *   ****      **.  * ***        *.**   ***.    *  **

40            RYCCCGGCTMTCWRYYRGRWSRWGYCTAATKYRMTTCKGCTAGYRKGGMTYGYKWMRTYY
43            RYYMMRRYYMGYTRYYRRRWGGTRYTKRRWKYAACYYTKMKGAYRKRSAWTKTKTARGCT
              **        *    **** **.    *  .    **      .  ...*** .      *  *

40            RGRRYRAGCKKRYCAKCGCCGTRYCAYRMYKKAYMRTMRYRKKRRKKGTKRYKCAKYMKA
43            ASARYRWRMKGRYYRKTAAAKCGCATYRCYKTRCCGYMGYRKKRRKGKWTGYKSMKCCKM
               . ***    * **   *  ...     .:** **.      * *******    . **. *   *

40            GCAGGGRKGRMRRKGCKTGTGKKKKGMYCGAATAGGYYTGKKMYKRSKACYAYCWCYRTT
43            TYRACTAKRRMAGKRYGYRYRKKKKRAYTRRWWRTRYYAAGKMYTRSGCMTMYYWYYRAY
                 .    * **  *       ****  *        **:. ***.** .    * * **:

40            GGGRKTAGARGKSRSYWYCCASYWACAMTKYYKYYRRKGTTMGAGCGMRGAAACCAGGKR
43            KARRKCMTRRTKCASYWYAYGSCACMRMYTYYGCTRRTKGYCKMRYKMGRRRMYYGATKA
               . **     * *. ****. .*   .   * .**    **.         *        .. *

40            WWKCTYGGAGRYMCGYAYYWTKWRYYWKWTTWGWRMCYCAAAWSCTTGCKYTYTGAGAGG
43            AAKMWYCACARYMMRYGCYWYKTGCYWKACYTSWRMYCAGTGTCAAATAKTYYCKWAGAC
                *  * ...***   *. ** *    ***    .***   ..:. ..:: .*   *   ...

40            CKRTYTTRAYSWTYGACTYYWCMTTKYRTTGYKGAGYCTRGARWKTKKKTCMGTTKRMMA
43            TKGGYYYGWCGWATATYKCTWAMYCKYRGGRCGRMKCYWARGRWKAKKKCYCKYWKRMMG
               *  *     .*: .: .   *.*  ***              .***:***       ****.

40            TKKTMCKTCTWRCGYYARWCKWGCCKWYKSYWTKMRRMCATGCTMKTMYRKRKYWGGCCK
43            AKKYMYKGAAWRYRTTRAAMKWAMTGTCKSYWYGCRRMYRWKSYCKCCCAGAKTAKRMAK
              :** * * .:**          **.      ****   ***     .  *       *     .*

40            WWSRKKTTYMYCAWYKCKRYYKMYAYCYWRRKKMKKRKARKAAGCKTAKWRYRYMRTCRG
43            WWSRGTKYTCCSGTTKAGRYYGMYRYMCAGGKGMKKGGRGKRMAYKCMKTGYRYMAWTGA
              **** ..     ..  *. *** ** *      * ***     *   . *   *  ****     .

40            RGCAKKMYYWWYGGKYAKSGRYKKTKGYRARRWTTGCAGAATCTGAKWKMTTRGKSRATG
43            GRMRKKCCYAWYKKKYRKSRRYTTKKRYGWRGWYYAATACGAYYARKTKACCATKGGWKK
                  **  * **  ** ** **...* *  * *  ..:...:  . * *     *.  .
```

```
40        WRYRRRWKWYAAAKAAGGACCYMKKKKCKKAWTKYKGGGRKRTTWYYRRWTCYTCKCSYR
43        TRCARAWTTTGGTGTGTAGTYYATGGKSKKCWCKYKRRARGRYGTTCRGTCATKYKYSYR
           *  * *.  ..: :. ..  * .  *.**.* ***  .* *     *   . . * ***

40        YKRYKKRKTTAAATAGCYYTTWCWKKSRSSSCRYMMMYTYYTGGTKYAAKAYYCKAYTRC
43        YGRTTGGGCCGGRKMCMTCYYTYAKTSGGGGYGTCMMYYYYCAAKKYCWTRCYYKRYCGT
          * * .     .. .          *.* ...    *** ** ...**. .  * * *

40        TCGYRRRRGMGAKYMMCKCATGKRKWYWRKRCGARAKCCTRYWKMYSYTYRMYYYTTYGK
43        WMSTAAAGKAKWKYMAYGTGCRGRKWYWRKAMRMGRKMYKRYWKMTGTYCGMYYYAGYSK
            .         ***    .   *******      *  .***** .    ****: *.*

40        CTYCKGYTRYRKTKGWYYYRWWKSGRGATCGAKWWYCTTAKMKTKARKGTGCCATYYAGG
43        TACAKAYKRCGGKKRTCCTGTTTCRRRWWMKRKWWCMKWCKCGAKGAKKAATTRYYYTAR
           : .*.*.*   .*        .. *      ***  . .*  :*. * :.    **:.

40        TRGTCRCYRTTRYRYCAGGCAYRKKKGRKGTAAMRYYGAYRKTTGYMRWTTYSGYARTKK
43        CRRCYGMTRYYAYGYMRRRMRCRTTKSRKTCTRMRCCRCYRGGGAYMRWYAYSTYMAKKK
           *      *   * *       *..*.**  : **   .**   .**** :** *  .**

40        MCCCSTAKATARYYYGCWWCCCGTRKGYYYRKACAYYRRTKKAGSTTRYKKCCMMYKRKC
43        MTTTCYGKWGTATTYRTTTMTGAGGKKYYYRKRTRTTAAYGKGASYCRYKKMYCMYGRTM
          *   . .*  :   *       .  * *****         *..*  ****   ** *.

40        TKKMYKGYMATYYYCKTKRYRMRRWCKGGMGGRTTAKWKRSAMYKKRCAYRKMYCAYKKK
43        WKKMYGRTCRYYCYYKCKRYRMGGATKRKCKKAKWGGWTASRATTKAYMYRGACYWCKKK
           ****      * * * *****    *      . . *. *   .*   **      ***

40        MMKGTTCTTGSWKAGAAKYGKGWYRYYYYYYMWSYRRYYKKTATYYAGYAKYRYMKYGYY
43        MMTRYGAGYRSAKRRMRKTRKRAYRCYYCCCMWCCGATYKKGRCYYGAYGKYRYMTYRCC
          **.   .   * *    *  *  ** **   **.    ***   **..*.*****.*

40        CTYYTGRYAARGRTAAGMYYKWGTATGARRRYTCCGTTYYGCWKGGKYKYRYGTGKSMRW
43        AATTKCATMRGAGCGTTCYCTTCCCCCTAGGCWMTAGAYTKTAGRAGYKYRYACRKSMRW
          .:  .      .  .:  * .   .  :       . :*      . *****.  *****

40        MRARRCYMGYSMKCAWYRTRKMRTRGGKKTRYMWRTWSRTKKKKMKRYRSRGCTCCWCKK
43        MGRGGTTCRYSCKYWACGCAKCGYGKKTGGRYCWRKWSGCKKKTAKRYRGGRYWYSAMKK
          *        ** *       *      .  ** **.**  ***. ****.     .  **

40        KWRYKTRRYRAYKKMCMKYCRYRYMATTTAKMYYKSRTKGTWAWWKGTTKCGAATTKCKA
43        TTGCGYRGCRRYTKMTMTYYRYRYMMYKWMGMYCKSGCGKGAMWWKRWYKYARRWWTSGR
          .     *  * *.** *.* *****  .   ** **       ***   * .    ..

40        WKCACCAACGGKRTAYKMRRKMGRKTRKYRWCATGMYKRGGRKWRYYMKYATAKCYYGCY
43        AKYCTYGGTTAGACGYTCRRKMARGYRKTGAMWWRMYKGARRKARYYMKCGYWTMYCTAY
           * .  ..  .   .*. ****.*  **       *** . ** ***** .  . *  .*

40        YAGTTTGGTTYTYKKYAWGKWTRYRAKTRCTSCTTGWCYKRRAATRRTGTGAMYGYGCGY
43        YMRYWCRRCAYKYGKCCWCKAWACGWKYGYACTACRWYCGAARWWGGGKCRCACKCRYSY
          *        :*.* *  .* *      *   :. :  *              .      .*
```

```
40      RYRRGKRMGRCTWKCTKRYRYCKYYYYTKGRACCCCKKRKKRGYYYYYYYRYKWRKWMTY
43      RTAGRKAMKRSGWKTAKRYRCMKYYYYYKRGRMMYMKKRKTAKYCCTYYYGCKARGAMWY
        *    * * *. ** :****  ***** *       ****.  *   ***  * *  * *

40      KRWGMKMWWYKRSKKKWRYRKSRGRAKTTKWRAACCSTKRCTKYRWKAMMRRCRKTKTKY
43      KRWRMKMATTKAGGKKWRYRKSRKAWKYAKTGRWYTCYTGMKKYRTKWMMRRYAKCKKKY
        *** ***   * . *********   * :*      . .  .*** * ****  * *.**

40      KCTGCRMRGCCYWKYRWYGTYCYWCYTCATTRKRWKGSYWTTACRKGTTRMKYRYKKTWY
43      KYYTYAMGRYYYTGYRTYKCCACTMCCTRWWGGGAKASYWACGAGKRYYAMKCRCKKYWY
        *     *    *  ** *   .             *.***: .. *    ** * ** **

40      GMYKYTSMSKYTGCACCTKCKMRYRYAYAWKRMGMRYKSWRYMMKYMYARWGGGRCGTSY
43      RMTTYGGCGKCCATCGSKKMGCATGCGCGWKAMACRTKGAACMCKCCYGGTAKKRTAWGY
         * .* . .*  . . ..*       . .** *. * *.   * *  *.  .  * . .*

40      MKKRRGRRTGKRYWRRKTTTGYTGRCCATCYRKWAYKCKRCGTRCMTYKYYGGTAKTACA
43      AKKGAAGAWTKRYWARGGGAAYGTGMYRWYTGKTTTKTGRTTCGTMYTKYYAACTKKRYT
         **  .    **** *   :.*          * : *  *     *  ***.. :*.  :

40      ATAGAATYGCAAAAGRTRAMCGTKMTTWTRCTRWMCKWARMMTGRTYKGCCKGRRRCRMY
43      CYRSGWYYRYTTGTARKRWAMKCGMCKTAGAYAACTGWGACMWKGKYKRTMTKAAGTRCC
        .  ..  *  ::.:.*.*      * . : .      *.  *   .**   .     *

40      GKRSTKYWCCRWATACACCCMATYGKARMYYYSRRTAKCGGYMRRCKCTKMCKYRRGTAK
43      KGRSYKCAATGAGYGARTMYMWKYAKTRMYCTSRRWTTASKYAAAYKMYTMTKCGRSYGG
          ** *  .   . ..    * .*.*:***  *** :... *    *  .* *  *. .

40      ARRRRRACKRACGKYTTCTYYRCYCCRKKRTRSYMSWACKKWYTGTTTTWYRRKKCATMA
43      RRRRGGGYKRMYRKYCYYWCTRTTTARKGGGASYMGWRTKKWYAKCYGCWYGGKKTGCCT
         ***  . **   **      *   .**    ***.*  ****:     **  ** .  :

40      AAYCKKKKRGGCTCTCKKYCCRYKCATSGTKWYRYRRGCAKYMWYKGKRARMRRWGYYCA
43      WTCYKGKKGAKTGSCYTGCSMGCTYMWSRYGATGYRRTTGKYMWYKKKRMAMRRTRTTAC
         :  * ** .   .  .  .   .   *      ***  .****** **  ***    ..

40      YCGTGMTGRATRWTKKGCCWRKAWKKCCWGRKATGTKWARAATTAAMMKCATAAYCCRRG
43      TTACAAKKARYAAYKKRTSTRGRWGKTYWRRKTCAKKARRGGGAGGMMKTGYWCYYYART
          . . .       **  . *  * *  * **: ..*  *.. :..*** .  .*   *

40      AGWKWRCTYTYAMYGAAAAAYCKGGCGGGCAGCTMRRTARATCCAWCACAAGAACTCACM
43      GKAKTRYCYYTMMTAGRMRWYTTATTTTATMKYWCARYRGRYAMMTYGTWGKRMMYTWMM
        .  * *  *   * ..    * ..    .       *     .    .  .        *

40      KRCAGTTYRAWTTRYRAGTMTAGGGGMWGWRKGTAWKCGTARCGYYKGTWACCCTRTRRK
43      GRYTAGCCGGTYYRYGWRGCWWRSRSCTATGTAGGTTTAKRGYSCYKRYTRAYMYGYGRK
         * :.    .   **        . .  .  .. . . ..   . **    .      **

40      KKTTCKKAYTKRACRRRKWRRYSRATCRYKAACKCTAGAYKRYYTGAKWTAGRRYRCKTG
43      TKCATKKGTWGRWYRRRTWRACSRTCTRTKRRYKTAGARTTATTYRMGTYRRAGCRYKAR
        .* : **.   *  ***.**  **:  * *   * :..  .              * *:

40      CKTAACWYYRGWRTTYAWCRCRKACTTRYRYYMRKKGYAAGWCARTGKKGGCKYKKGATC
```

```
43          TKWRGYTYYGSAGYKYCWYGAAKRAWYGTRYCAGKKRYWRATTRGCSKKRRMKTTKKRKS
             *  .  ** .   .*.*  . * .    **   ** *  .     .**   * .*  ..

40          AAAYCKWYTTTTAACGMYRYSKGCATRMAYGAKYYYAAMA
43          RTTCYGACYKCAGWYSCCGCGKRYGGAMWCACKYYYTWMW
             ::      . :.  .    .*  .  *  ..****: *
```

The pairwise alignments of two accessions 40 and 44

```
40              YMRRASMMKYYRRWYRGCARYKCGGYWMRMMKYKWRRRRYCCCKCCCCCCRRRYCACWKK
44              TMGAMCCMTYYRRWYGRTTRYKYKKTWMRMMKCKWGAGRTATTKTTYYYMRAGTYRTWKT
                 *   . *.******   :***    ****** **   * .  *      *      **.

40              YYMTGYKKYYCWKMMKAYTYKYRTWKRKKYMWYRYMKRYRKKRRKAKCCGYMYYKAGMRR
44              YYMCTYKKYYYWKAAKGYCCTYAWAKGGKYMWYGYMGGTGTKGRGWKYYRCATCTWRAGR
                ***  ***** **  *.*  .*   *  ***** **    .* *  *       .    *

40              ARGRYYWRYYWWCYRMYMKRYYRYMWRTYYKKAKYCRAWAMKYKRYYSYKKKWCTTYMYY
44              GACATYWRYYWWYYRMYMKRYYRYMWRWCYKKRGYYAMAMCKTGGYTCTTKKTMWWCMYT
                .    ******* **************  ***  *      *   * . .**     **

40              YWMCTCCTKCYKYYRWKAGGGYYGSYYRRRAATGRAKTYRYYYYKKMGGKMKGTTRYKYY
44              TAMMYYYWGYCKTTAAKRRRTTYRGYYGRGGTCAGRGGYGYCCYKKMKRKMGAKAGCKCC
                  *        *    *     * .** * .: .    * *  ****  **  ..:  *

40              YKYYRKYYRGGTWYRRMYYYKRRRKMYMRYTCMWCSSYWWYYYWRTCYYYTAYRSRTRKK
44              CKYCGKTYRRRGTYARMCTTGGRRKMYMRYWSMWYSSYWWYYYARGYTYYYWYRSRKGKK
                 **  * **    * **     ******** .** ******** *   ** ****.  **

40              YTGYYRRYKGWATWMRTRCAYYMKYKYGYKYWCYWWYKAYKRKYYGRYKMWRRWRSTAMT
44              YYSYCRRYKCWWKWMRCATCYCCGYGTKYKCASCATYKRCGRGYYKRCTCWGGAASWWMY
                * .* **** * .***   .*   *   **  .   **   * ** * . *    *  *

40              CAYRWRYMYSTGRCAKGGRWWSTGGWGSYGGMYCYAGYMCYKYMMRCMYYRGYKCCWRRW
44              YMYRWRCAYSWSRYGKRKAWWSWAAAKSCRRMYYTWATCYYKYCAGYCYYRRYKTGTART
                  ****  ** .* .*   *** ..  *   **   .   ***     *** **    *

40              RAYKRKKYAWYWRCRYKRYYWWGGGTARSRASAYYMKYCWRGYYRTMMYSTARTATAGGC
44              RCTGGTKTMWYWRTRYGACTAACTTCTRSGGGWTYCKTMTRKYYAACCCCYRRWTCCKAA
                *.   .*  **** **          :** ..  * *   * ** :   .  * : . ..

40              GGTCCTCCRKCYCRRCAWWYYAGGCTAGKARKTTKRRRMRSAGGCWKYRGSCYCRYWKCC
44              AAYYYYYMGKYTTRRYRTATTWKATYWKKGRKKCKGRRMGGRAAYWKYGRGTYSRCAKYY
                ..       *   **        .    *.**. * *** . .. ***  . *.*  *

40              CTCGKWMCGCACTTACCYMWTCRRMRYYGKKYRRYSRYKCKGKCTGCWTCCCGCTSRRYT
44              YYTAKWCYAAWYCCRTTTCACYGRMRYYAGGTGACGACGYTRKTCRYWYATTKYYSRRYY
                   .**  ..             *****.      .    . *    * .    ****

40              CCTTTMGKATKGAYCGTTCRAGWAWRYRGAYMKKYCTKYRGKRTCTYGRRACCAMTYYKG
44              YMYYWMRKTYKTTYTTYYYGRSAMAATARTTMKKTAAKCAKTAWMCYARRWMYRMACTKR
                     * *: * :*       .       : *** .:*   .    *.**    *:  *

40              MRYGYKYCAKRYRGGCTKTRKTGAWYYGTYCRKTKAAKTRYKYRRMRAATKKKKYYYTKR
44              MRYRYKYSMKAYRRRTCTYGKCACTCTKYYAAKWKWRGWGCKYRRCAGGAKGKKCCYWGA
                *** ***. * **    .  * ..     *. * *      ****  ..:* **  *

40              CAGGTGYKRYWAYCRSYKYWKYKGGTAYKRWKWSAAGWRRYRWMACYYYWWCGCKGCCTA
44              MMKSCAYKGCARCTAGTTYAKYGAACWCKGATTSTWRWRRYRWMWMYYTWWTRYKKTGAW
```

```
                   . .**       . .* ** ..   *  . *:  ******* ** **   *   :

40              TYYATCTYCRRRWYRSRRRMYRYGTRSMRKRYYYYRYRTKKYRKMCKKGCGSTCGSKYYR
44              CTYWCACYYGAAWYRCRRGMYRYRWACAAGRYYYYRYGCKKYRGMYKGAYACGMRCKYYG
                  *  . *    ***.** ****   .   *******  **** * * . ..   .***

40              KRYAGKMYKRYGRATRYCMMSYMKMCYMGYTGGTAAKKRAKCAKRGTTTYCGGTTARWRK
44              KRCGAKMYKRYSGTKRCYMMGCAKMYYCRCKKKWWTKKGRKMWKRRWCYCTACWKWRWRG
                ** ..******. :.*  **.  ** *   .    :**  *  **      .  . ***

40              ARAMMRTGYTCRWWKWKCTYWTKCGAWYYTGRTGTGRWKYKGYRWCWGCRCMYWCTTTRY
44              RRRMMRGRCGTGTTKWKYGYWYGARRACCKARYRYAGTKTGRYGAYWKMRMACWTYYYGY
                 * ***        ***  **  .     ..*   .  *   *   *  *   *     *

40              KRRMKCGRKAWAWCGWGWAYKKTYYRAGAAKATWRCTTYAMTYYTTKKCGYRWRKTAYAA
44              GAGCKAAGKRTWATATRAWCKTGYCGGRGGTWKWGMYYCRAWYYYWKGARCRWRKKGCGR
                    *.. *     .     *. *  . ... .*        **  * .  ****.. .

40              GRAAYGYTAAMKYRTYYAGGAWMARYCRAYTRCCTGATTGRCACGTYKARRTGYATCYYY
44              KRGGYRCCGCCKYGYCYCCKWACRRYYGWCGGYYYRWKKRAMWYTAYKWRRGKYWYYCTC
                 *..*   .. **   *.      **           ..      :** **  *

40              RYAYKSCMCTRRKGMGTYGMTRKRTATYMKAMGGTKYRRGTTCAYCRTYKACYTATKYYK
44              ACMYKSMAGCGAKRMAWCRCKRGRCGYCMGGCAAKGYRRRAAMWCYAYCKTSYKWAKCYK
                   ***      * *.    .* * .  * . ... *** ::       *:.*. :* **

40              GGWAKRRCTGAKKWKYRSTRKKGAYTTTGKKRMGGTARYGRYWTKAACCKAYKRYYCAWK
44              AKTGKGGYCRGKKWKYRSCAKKTGTACCAKKRCRRWRRYRRTTKKRRYYKRTTATTMGTK
                .  .*     .*******  ** . :  .***     ** *  .*    *  .    . *

40              YKWTAACGRRYYWCGAGWATRWWCYYMYMSKAAYYWYMYYGARWTYYTCRAAAASCWSCY
44              YGTYRGTRAGCYWYRRRATGRWWYYYCTASTGMYCWYCYYRWRACCTYYRCGCGSAWCYT
                *    .     **     : *** **   *.. * ** **  *      *....*.*.

40              TRAYKRCYTYYACAGATRYGCMRKKKAGYAACKWCYRCWARTCYKKARYCKYTCKWWRCA
44              CGTYKRTTCCYMMRRRKACRYAGKKKWKYRGYGWYYRTWRGCSYKKRRTMKTYMKAAGMM
                  :***    *     .      ***  * .  * ** *   .*** *  *   *

40              TKYYMAAAKMYKRSYYRAKWWYYGYRACRCMKKTCYRARRRRYSSATTTTGACGAYYYYK
44              WKYCCCGGKACGGSCYRMGAACCATRWYAYCKKYYYRTRRRAYCGTCACCAGTACYYYYK
                 **  ...*    *  **      . *     **  **:*** *..: :  .. ..*****

40              WTYKKCYTTCTKYTCKACCATCTATTYYYYTAKKYCRYYKRKRKYKRYKAYCYYYTRYRK
44              WYYKKSYWAAAKCKTKGASMYYYMYYYYYYYTKKYMRYYKGKRKYKRTKRYMYYYYGCGG
                * ***.* :.:* . *...       **** :*** **** ****** * * ***

40              WKWWKRYKWGRTTYWCMTKRWGMKYWCKGYRSGKTKKWRYRKSRGKSRKACAKKTKTAKT
44              AKAATRYKARGWCYWMMCKRWRMKTTGKSCAGAKYKKWRYRKSRKKSAKWMRKKYGGTKW
                 *  .***     ** * *** **   *.  ..* ********* ** *   **   :*

40              KWAGAYAMAYCYRYASTKRWYRWKGKYARGAMMRRRYRKCYKCTYRKKRMMMTACWKKRK
44              KTTTRYWCWYMTACRCYTGWYRTKRGCGGAWMMRRAYRKMYKYYYAKKGCCMWRYTGKRK
                * :  *   *     . . *** *   . . **** *** **  * **   *     ***
```

```
40          WAACGWARRAGRWRKKCAWGTAMYYYYYCTMWGYYSRRYRACWTYTCYGYRRRCARCTYY
44          TGRYRWGGARKAWRGKYWTRCGMYYYYYTAMWRYYSRRYRGYAYTCYTRYRRRSGRACYC
             .   *.     ** *     .****** :** *******.        ****..*. *

40          RTAYYMRWGYKYRYMAYSWRRYYYWGTGSYYCGYYTKYMSRSRTTAKWRRRKRYYCYCCS
44          GGTYYAGTAYKYRYMRYSWRRTYYWKYASYCTRCTKKYMCRSRYCRTTRRRKRYCYYYYC
              :**   .****** ***** ***  .**     .***.***   . ******  *  .

40          TYCCARMKTGRAGKTYYKYMRYSMRRTMYGKRRRYMKRGAGCWTRTRTCGCGMRRYKKKK
44          WTTTCRMKCAARRGYCYKYMRYSMRGAMCRTRGGTCKGATATAKGYRKMSMRMRRYKKKK
                .*** .      ********* :*  .*    * .:.  .  *. .  ********

40          MYTMRYACYCWYGACWGTYGYYRRKYMYYRCGGRYKTKCTCYRGKSYYWKKACATGYSGR
44          MTCCGYRMCYWTACMTRWYSTTGGGCCYTGYRRGYGYGYKYCARKSYYAKKGMRYRYSKG
            *    *    * ..    *.       *      *    .    **** **.    **

40          SSSCRYMKKAMMYKTGRYMGTKYYRTGGYGAYTCTAGTTAGTGRYRRAAACTTAYMYCAM
44          GGGYGTCKKRMMYKYKRTAKYGYYRCAACTCYYYCGCWKRRCRACAAGWTTYYRYMYYMM
            ...    ** ****  *     *** ..  .*   .  .        . :    ***  *

40          YRGYYARRRRATMMRMRKACAKCKMKKRKWARARWRYYRMMTGYCTCATCAKCTCKYWKR
44          YASTYWAAAGMYAMRMAKTTCKMKAKKRKWTGRGTRYYAMMKRYSWAMWMWKYYMKTWKA
            * . *        *** *: .* * *****:    *** **. *. .    *   * **

40          RRTKYTCGYYTYWTYKMRKYYSYRKRGAYRKCAARGRWKMYYYYRCTRYTWWKTMCGKTK
44          RGCKTWMRYYKYWCYKMRKYTGTRKGKTCGKMRCGRRAKMYYYCGYGGYAWWKKAAKKYT
            *  *    **.** ****** . **  :  *  .  * *****     *:***. . * .

40          KGYRMCYRTCGGKWKRRWGCAWKKWWKSRKKRYGKAYYMKYYYGMKYCCMGGCGKCCTWY
44          KAYRMSCGAYRKKTTAAWAMWWKGTTTCGKKRYKKRYYCKCCTRCKYTTMATTKGYYKWT
            *.***.  :   * .  *.  **   .. **** * ** *     **  *.      .*

40          KKMYRMYYRRCGRYCRGTYYGKYRYRMGGTGKCTYRAKGCCRWGWRTSMGRRSYRTGGAC
44          KKCYACTYGRYRGYTRKCYCKKYGYRMCACAKTKTARKSSMAWKARGGAKRRSYRWRRRY
            ** *   * *   * *  *  ** *** . .* .   *..  *  * .  *****

40          AKAWWRKRRYKAKKGTTCACGYGYRKATGTYRYWGKYWRYGCMRYYKYCGYYGTAMWMTA
44          WGGTTAKRRYKRKTTAAYMYKYRTRKCCRKCGTWRKYTACRMMRYYGYTCCCAAWATCWR
              .   ***** *. ::    *  **.  .   * **     **** *    .:

40          WRRTRSKKYGWRYCGAKYKCRGGAAKAKRKWSTAGAKCTRYKGGRRKKRKCKTCRKMKKT
44          ARGKAGKKYAAGYARRKCGMGRAGGTTTAKWSYRKRKMKRTTKCRRKKAKSTWYGGAKTW
             * . .***.  *.  *     ....:. ***    * .* .  **** *..     *.

40          AGCCCCGYYRYKTYKKTKRYYGYKCKYCWCYAAYKSRCRYRCGMKWSKRCGWWKWAKSCA
44          RRYYMYKYYRTGYCTKWKGYYACKTKCYWSYWGTGCAMRYGTACKTGTGGATTTACKCAC
                   ***    .* * **. * *  *.* .  .  **  . * ..  .  . .*...

40          TGGTKTCKAWYRYRTKSMCMGARAYYKAACCTCYCKGYRGKRKAAYYCSWGWTYYTTARM
44          CKRYKWSGMATGYRWKSMMMRMRRYYKGGMAWYTYKRYRKGAKRMYYSSTRTYTTKYRRM
                * .     ** *** *  * ***.. .    * **   *  **.*      .  **
```

```
40          YAWYYSYTRMTRYRACKKYYMKKGRCCAWKKWKAWKCKYKKWMMTWCRWWRRWYATMGTA
44          YTACTSYKRAYACGRYGKYTCKKRRYSWTTTTKRWKYKYTKWMMKTTAWWRRWTTCMAKG
            *:   **.*        **  ** * .  .. * ** **.****.   ***** : *...

40          ASWAYGWACRACTGCTRMAATTRKTATTTRKMCTGRCCWMYRKRRGATGCKWRKGYGAKG
44          RSWMYRARYRCYYRYYGMRRKWRKYCCWAGKCGCTRYYTCCATARTGKTTKWRKRCRRKK
             ** *    *.      *  . ** .  : *    *      . * ..  ****    *

40          AATTGTAYYYYYTAYAYMRMRRCKGCYSYRKRRKKRYMKAKYKKKYMMMMKYWRKGTCRR
44          CTGYAWMCYTTYWRYGYCGMGRAGATCSCGKRRKTGYCKMKYKKKCCMMMKYAAKKWYRR
            .:  .   *  *  *.*  * *. .  *  ****. * * *****  *****  *   **

40          RMRRAARGCGAKMMKYMYWKGAAWAMTTWRAKAAKYKKKGGKCKGAATARGWYKKTCKGA
44          RMAGTGGRYRRGAAGYMYWTKRWTGMGATRMKWWGCTKKRKTTKRRWWRGTWYKKATKTC
            **  :.         ****.    .* : * *    .**  . *       ****: * .

40          AAGAATTCGTAKRSTAAKYKTATSTRKGKMRSRCYKYSYWYKCASKTAYYWAGCTYATYY
44          GCKMMWCTKAGGRSCCTKYKWMYGCRKTKMRCATYKYSYWYKTGSGCGYYWRSMAYCKYY
            ..       :. ** .:***   . ** ***.  ******** .*  .*** . :*..**

40          TTTAKYRCGKYKMKGRYGYAGGKGRKWGCRAGGMTTGWWKWYMCTKTGGGAGRYTGCAAT
44          CCCTGCAMKKYGMKKRYKYCKATARGTRYGGATACGAWWKTCAAYKWKCACARYYRSWGC
               :     ** ** ** *. ...*     ..    .***   . *   ...**  . .

40          KATARRMKCATRCCGYWMTRRTGWKYGACKSWAMRGTRGKCTTAYMRKKYTAYKMMYYSC
44          KCYWRRCGAGAGYYRYWCGRRYAWKTTTTKGAWCRACATTMWWWYARKKYKRCKMAYYSY
            *.  **  ..:    **  ** .**  : *.   *.   .    * ****.  ** ***

40          CSKCACTYACTRCTCKKRTYCYCGATCWGTTGYGYCAGCCRWRSTAACAKCCMWKTYYCY
44          SGKYTAGYMYYRTAATKRCCYTMRGYYTAKCATACYGRYTATAGWMRYMKMTMWKYTYTY
            ..* :. *   * :..**      .   .. . .  .      .     *  ***  * *

40          ACCCRGWCMCTAKMKCCCCKYCCTCYCRRYRGATTWRYWGCRTYWGATTKRTRTYGMKTT
44          WMYTRRWSMMGWGCGYAATGYTTGACYGRYRAWCCAATTAGACCAKTGCKRGRGCRMKCY
                * *.*       ..  *   .   ***.       .      :  ** *   **

40          MTCWKAGTAWCYGMGGATTCYKGGTSYTYKWTKTAATYTYRGKARTKKKRRMKMGGGCGM
44          MATTKRCCGTMYAMRRGAGAYKKACSYAYKWYKYMGGCYYAAKCRAKKKRRMKCARKTRA
            *:  *   .  *.*  .: .** . **:*** *  .   * .*.*:******* .

40          MMYKCTAKKGTTAGCCCKWRYARWCKKKGGYTRKYSYWRTKKMCKTGWGTYRMKTMYYAM
44          MMYKYKTKKKYYCATTYKTGTRAAMGKKKRCYRKYSYWRYKGCYKARTAACRCKWCCYGC
            **** .:**   ..   *        **    ******* *   *:  .: * *   *.

40          MYTRKRCGCAKKYWWSRAKKRKTTYWYRWCYKWYARRKKTTAMYRTTRGYTYGRYGMYTT
44          CCARKRYRMTKTTWWSRGGTRKKYTATGTYTKWCTGRKGYYGCCGYYGRYKCAGTKMYYY
              :***   :*. ****. .**.        ** : **    .       *. .   **

40          AYKACAKRCTKGCAKYTWYWATTTKRGAACRRSYTGYAGRMGCATAMRAAKGGATTKRGA
44          GTKGTGKRTCGRTGKYAATTTKYGKRRTCYRGGYYKYCRRMKMMCGMRCTKRRRCCKRKT
            . *. .**     .**:   :.  ** :. * .*  *. **    .**.:*     ** :

40          TGCCTYGGTAAWTMCARWACYGTTKYGATWARWTYWAMTAAARKKMWTAAWMTTRYRYMK
```

```
44        CRYMCCATYRMWAAYGATCTCRYYKYRRWWRRWKYWGMYTRWATGCTWMRWMCWRCRYCK
                .    *:  .  .     **   * **.**.* :   .      **  * ** *

40        KKTGYRASRRRGMRTRYWMRWTTTTCKAKRTAGKAGTGTGGAGMYRYRYSRRTKCAATAY
44        KKCKYGRGGARACGKATTAGTYYCWTKCKRAGAKWSYKYRRCKMYRYRYSRRWKMTCCGY
          **  *  .  *.  .           *.**:..* .     . ********* * :. .*

40        CRAYCGMRCRKKCCTGKMCCGYYRRGRKSMKKTKCGARGYATGARRKRYSWTMMKAAKGA
44        MRWYMKAAGAKKMACRTMTYKYCGGRGGGCKKWGYATATTRCTGAGKGCGTAMCKWCKRM
           * *      ** .  .*   *      . **   .:      .  *  . :* * .*

40        YGAYCTCTYYKKYCCRKKGYMYRGCRRYKSYAMKKKYRWAMYMACKWYTKCAKKCAWKAC
44        TAGYSYYWCTKKYTARGKKCMYGRYGATKSYRMKGKYRWRMYAMTKWYCKYRKKTRWKGM
           ..*.     *** .* *  **      *** ** **** **   *** *  **  **.

40        CTTCRKTYGAAAYTYKGYKKRMAGRTTWKKGGRWAAWRRMYRKSAYYYRKKCCCMATCWT
44        MKGGGKYCRGGTCWCKAYGGRCGARYYWKTRARWMRTARMYRKSCYYYGKGYMMMGYYAG
           .   *   ..:   *.*  * ..*  **. .**    ******.*** *    *.

40        GAKRCRMYKSWWGSWTCATKMMRWYTRYWWCAGCGGKGCAMYGMARTRGAMYATMGGRTR
44        RWKAMRMYKGWWKSAAYWAKACAWTYATWWMRRTRRTTMWATKMCRWGAGMCMAMKAGCG
            *  ****.** * :  :*   *    **      .      *.*  ..*  :* .

40        RYYRATCCYKGYCTRKWGRYKYYRATGAWWGAGCATWYCTCCGTMRTRCACARAAKACCY
44        ATTGGCTTYKTTTKGKTARTKYYRCAATTAATTYRYACACTTRCAAYRYRTWACTKGYYY
              .   **   . * .* ****.:.:  .:      .        *     .:*.  *

40        YTRKWMRAGCYTCYKRYACAKRTWWGGTKKRYCAGRKCTWTARKKGMKWKMGKKTCWKYK
44        YCRGWMRRSYYYYTKRYRYRKRAWARKCKKGCATAAKTYWGRRKKRMKWKCRKKYMAKYK
          * * *** . *   ***   **:*    **  .:. *  *  *** ****  **   ***

40        YTYATKTKAKGCGWYWSKCYYTKWMMSWTRYRRRCCMCKAAGTCRATAAGRWWGRRYRAC
44        CWYRKKWKMKKYAWYWGKYCYKKWMASWCATRARTYATGGTRYGRGAGRAGATAGRTRMT
            * .* * *  .***.*  *.*** **   * *     .:   *.:. .   . * *

40        CRAAACWTGRTYWRKRYWYTCCGGMTCTGACGKKATTGTGMMYYRTGATRTCAAATRYYY
44        GGGGGYWCTRKYWRKATTYWYYRRMATYRRSKGKTCGRYRMMYYAYKMYAWYCWGCRCCT
            ... *  *.****   *     *:    .  *:     ****        . . *

40        SAGKCAKTRATMGGCRTKKGMMCATACAAATTYYYTCCCCGRKGCCKKTCCARAAKCYAY
44        CGKKTMKCRWKMKKMAYKKKMAYRKMYMRMYKTTYCTATGAGTKTTGKYYYWRRRKTTGT
          .. *  * * .*     ** *   .      .  *  .  . .    *    *  *  .

40        RRATKKTYMCCAGGRRAKTTTCRYKRYMRKYKKGRWKYYYMYKKCMCCGAAYRMGYGACT
44        AAGYKKCCATAGARGATKCYATRYGGCMGTYKKCRWTYYYMYKKAAYTAWRYAMRCKRYY
            . **    ...   :*  : **   * .*** **.*******.   .  * *

40        TKSYRYKAYTCATARYCCWMKRTTCAKWKRACCYSAMTGGARKKRCWKGRYCYGGRTKCA
44        WKGYRYKGYCMRYMGYYYACKRKCTGGWTAWTSTSRAGTRRGGKAYWGRRYMCTRGCKMR
           *.****.*      *    **.  . *.   . *        *  *  **      *

40        YCRGAYKMCMTCKYTYWYKTCTRKWAGAAKKGRYKKYRRMYWRSRAYYKKYRYYYKCTGG
44        CYGTWCKMYMYMKYWYACKGTCAKARRMRKKRGYKKTGRCCARSGRCYGKYRYCTKYWRR
```

```
                  ** *  ** *  *    *     **  ***  *   **   * ****  *

40          MKRATRYYRYRACCKYKTMCKYACRKTKCTRWRSKTWCRKKKWGKMWYARTAAKCYGGAK
44          MKRCAGYYGYGRYMKTGCMAKCGTGKCKTAAWGGKYWTAKKKAKTMWYTGWMGKTYTRRT
            ***.: ** *    *   *.* .  * * : * .* *  ***  .***:   .* *   .

40          GKTKAYRYKKCKKRSKAWAGYKTWRKAARGTGCTWMGGMRSRRRWSCGRCKAYWTYYGWY
44          AKAKCYRYTKYKKRGTMWRKYTCTRKCGGRAAYYTMARARSRRAAGMTGMKWYWKYYKTY
            .*:*.***.* ***.. *  *.  **..  :.   *.  ****  .    * **.**  *

40          KRKGGKCCWYMRMRRWKWYMAAAKAAGGMKAKRKACYRKYTAATRKGRCTCRYRCYYRAM
44          KRKRRGYMACMAMRAWTTTMGGTGTGTAMGTGAKGTYRKTCTGARGARYYTRCRMYYRRM
            ***       * ** *.  *..: :. .* :  *. ***  :.:* .*   * * *** *

40          WGGRCYYSYGRYKRKWYYYCCKTWRKRKKYTCCGTGKYRWMRRYSYGSGRYYYRMATMYM
44          TARAAYYSYKRYGRTTTCTGYGGWGKGKKYCTTAYSKCGAAAATSCASKRYYYGCRKMYM
             .  .**** ** *.        * * ***   . .*       * .* ****   .***

40          YKGGTRYYTYCYRKSRKMCYMRYKATCCCRSKYYAGTTYKGTGWAMMCARYRKMYRYKYY
44          YKTACAYYACAYAKSGKMMCMRTTMYTMGAGKCYMKWCYKTCTAWMMYWRYAGCTGCKYY
            ** .  **: .* ** **  ** .      .* *    **     **  **      ***

40          WKYRGMRRYCCATGYKSAKKCCGGKCRYTMAWWWSRKTTATTAYSRYRYKYMRYSAMRWK
44          WKYARCRRCTTCCAYKGRKKYYKRTYACKCGWATCGKCCGCCTYGGCRCKCCGTCGCGTG
            ***   **   . .**. **    .   . .*  . *  .  :*.  * *    ..

40          CMTMTAKWYARRYASKKGTRRRTAGKGRRRKRRYWGKKMWRYWGWYWCTAGYRKRKYWKK
44          MCWAYTKWTGRRYGGKGCAAGAWGRGRAAAKGRCWTKKCTRYWKWTAAGGRYATRGTWKK
                 :** .***..*  :    .      * * * **  *** *  . . * .*  ***

40          WYYMGGGGAYRGMCKKKKYWYMGKKYYYYWWRSMYKAYGYGTKYGYTGGKRTYKRYRKAA
44          WYYMRRKSRTARAMTKKKTAYMKKKCCYYWWRSMYKCTATAWKYAYCARKGGYKRYRKMT
            ****   .      .***  ** **  *********. . . **.* . *  ****** :

40          WYCAYYYTYARYRKKCGAATCRRARSKYYCKKMRAYKCWCATWKKAMSCRWYWGRSWGCG
44          WTYTYCTWYGRCAKKTARWYYRGRRCKYCGKKMRGYKMWMRYWGKWMSYRWYAKRGAATA
            *  :*   *.*  ** .    *  *.**  ****.** *   * * ** ***  *. . .

40          RMTATCATWCTCTKYSMGRRRKMCKGTKKTRYYYYAYKTCMGGTGGYGGYKRAGMKTKGC
44          RMCTCTGCTACMCKTGMRGGAKMYTAKKKYRYCYTGTKWYCKRCRRCRRCGACAMKYKRY
            ** :  .  .   * .*    ** ...** ** * . *              ..** *

40          KYRKWWTCRRMWMRYYGTTTSCGGTKMYSMKTTTKTTGAKCMCAYKMWRGCTGGKKCAKT
44          TTRTTWCYAACACRYYRWKYCYRKKGCCCATGCGKCCTTGYMMMYKATGKAYRRGKTTGY
            . *. *       ***  . .   .   . .   *   :  *  **    .    * :

40          SRYYYKCCYCYATYYWKKKRRRMWKYAKCMKGKYWWKKWRWCTWRKMTSCGYGYYRYRSW
44          GAYTTKMYCYTRYYCWKKTRRRCTKCRKYAKTKYWWKGWRWYGWRGMCGTKYRCTGYRSW
            . *  *       * ***.***  *  *  * ***** ***  ** * .  *    ****

40          KARKKYMKAAARCYWYRWCYKKYWYYWRYYGTKGWRGTTAATTAATRARTMGMKKKMCMA
44          KRGTKTMKGGTRGYWYRAMCKKCWCTWGCCRCTCAGRKYTWCYTWARRGWASCKKKMTMT
            *  .* **..:* ****   ** *  *     .    . :   : :*    . **** *:
```

```
40    GRYKGCCKYRYMKKWRTAKRYKGTAACAKKRSRYRKGYGTTYKTCAATTGKWKKRMCKAY
44    TGYKAYTTYRYMKKWRWMGRCKSCGTMCKKGCGYRKKYRWYTKYYWRWWRKWKKGCSGRC
        **.  .********   * *. .: .** . *** *    *       ****  .

40    WYGAATYMKYACKKTCGGYRYYKRKRAKMRYKGTGGCYAKKYKKRKKAAC
44    ATTWMYYMKYCYKKKYKRCATCGAKRWTCGTKSGAATCRKTCKKRKKWCY
            ****. **.         ** .   *. ..   *. ***** .
```

The pairwise alignments of two accessions 40 and 45

```
40        WRRKAKTYMYYKRRRRRCCYWRKWRRYCYRCMMMYMKRWRKYRWTRRYKTACAYKMRTTY
45        WGRGWKYCATCTRRGRRYYYWRGWRRYTTAMMMMYMKRTGGYGTKAGYTWWYWYTMRWYT
          * *  *     .** **  *** ****    *******   *  .  *.    *.**

40        KWKWSKMYYRYYWKYKRYRYMYRYKKWRYKKGYKMGGRKCMGCTMMYAWKGYTMRRCRKR
45        GAKTSKAYYRYYWKYKRCGYAYRYKKTGYKKKYKMKRAKMMAGKCMYCWGACAMAAMAKG
            * ** **********  * *****  *** ***   * *. . **.* . :*    *

40        YYYYKYGMMYYTTTTKYYWYWMRKYMGKGMCYYRSWYYWAYRCYGKWKACAGGGCKKCAC
45        CCCCKYRAAYYWKCKKYYWTWARKYMKTRCMTTRSWYYWTTAYYAKWKWYWAARYKGATY
              **   ** . .**** * **** .     ******:   *.***   ..  * .:

40        WKYGWRCWMRKKKRMMMRSWRMMRCYWMYTRRAGKWATTRGRRWRYYMMMRRYMYCAKAY
45        WKTAARYWMAKKKRMMMRSWRMAGYYWCYKGRMAKWGYCGAARTRYYMMMRRYMYYWKMY
          ** . * ** ************   ** *. * .**.   . * ***********  * *

40        YGCYRWRGCAGTRRRRKGATTRTYKYWKYMRRYMWYKRSMMGCTGRRCMRYGYWYGKMRY
45        YRMYRWRSMRKCGRGRKAGCAAYCKYWGTMRACAATGGSMAAAKARAMCGCTCAYRTCRY
          *  ****.     * **.. :   ***  **       ** ....*        * . **

40        MRKWYWTYWGSKCYYKRYYRYWCCRMYRTRWRYRYYCRSWKYKWYYRWMRYWKGKYATKW
45        MAGTYWWCAKSGYCCGGYCRYTTYAAYRKRWRYACCMAGTKYKWCYRWMRYTKRKCWKKT
          *   **    *      * **     **.****     . **** ****** * *  .*

40        RYKRKKYRYMRKYYRRWYCAATGWYRMMRSRKRYYRRKMGMRGASYWWYWTWYRWAGGYM
45        ATGRGTTGTMGTCCAAWYYWRYSWYGMMGSRKACTAAKMKCRRWGYWWYWAWYRARRRYM
             * .   * .    **    .** ** ***     **  *  .*****:***    **

40        RYYWYRWYGGCYGYTMGYRAKRTWAMYKRYRRSWTWMYCRTGTTYYSACWWRCAAKGRTR
45        RYYWYRAYKRYTTYYCKCGRKRGAMCCKGTRASTWWACTRKAGCTYCCYWTRSTRKAGGG
          ****** *     *      **     *  * *  *   *..   *.. * *.: *.

40        WRWCGCKYRSTATGKWCTAKCCYMYRMAGTKYCTCARMKGMATTKAWAMKCKMKYYYKAT
45        AGAYKMKCRSWMYRKWYYRKTYYCTAATAAKYSWTTGCKKCGGCKRTWMKTKMKTYCTWK
                * **     **   *  *    :.:**.  :  *  .  *   ** *** * . .

40        WCTTTYCMRTYTGTRMRAYYAGGTTACTKATCCGYTAGYWTYCSGRTYYRYTTCAARTKS
45        WYWWWTYAGYYCTYRMGGYYCASYWWYYKWGAARCWTRCWCTYGCRAYCGTCWYRGRCKS
          *         *   ** .**...     *  ..   :  *   . *:*       .* **

40        CWYACAYTYRCYAAARGGKRCTYAGCAAGRYARTTGGRRGYTYARATTTYRYYATKSYRA
45        YWYMACCYCATCTGCGRRKGYKYRRMWMRRYWAWARARRTCKTWACCGGCATCMYKSCAG
           ** ..      :..   *  .*      **   : .**  .   .         **  .

40        GKKMYKGCMCCGTRAYMKCGMGKYRYGWMKYRRMRRGKCATYRMGTGTTKRCMTKWCCCW
45        AKKACKAYMYARWRCCMGSRCAGCGYATMGYRRCRAKGYRYYAMSKKWKKAYCYGTGATT
          .**  *. * .  *. * .  .   *. * *** *      * *..  .*       .

40        GKCKGSGYMRTAAGYATGKRYRYTCCCGAYARGTCYKYYCKKGMYRRWKGGYAYYMYKKG
45        TKMKRCRYMRKCGATWCKKRYRYAGYMTGYMRRCTCGCCMKKCACARWTRRYWYYMYKKR
```

```
         * * . ***....    *****:    .* *        **    **.  * ******

40       CGYYKCYMGYRRGMGGGAYGGWYYCWRYKAKKCYATARRTKGGGWMRTAKSAMRYKKCCT
45       TAYYKTCMRCGRACTKRRYRRWCYYWRYKCKKYYRYMGGATRCCACRCGGSRARYKKYYY
          .***  *   *.     *  * * ****.** *     :.     * . *  ****

40       YRCWKKYGCGGGYKCYAYSKKMCMTTCKKKSRKCKMAKKKGAGWKYRGYYSGRKMYYKCG
45       CAYWGGYRYRRRYKYCWCCKKMYMYWMKKKCRKMGCTKKKRMKAKTGRCYSRRTMYTKTK
            *  *     **    .*** *   ***.**   :***    *    ** *.** *

40       KYYYYGAACKTGMWGYWYSYTWTYTCRAATAYKWRRCTTSRTYACCAYRYYGTYYCCYWK
45       KYYYYRRMYKWACARYWYSYCWYTCYRTRCCYKWRRYKWSRYCMYSGYRYYAWYYYAYWK
         *****    *  .   ***** *    *:  .***** . **    ..****. ** .***

40       KYRYGMAMSWWYATGYCYYYWGRYYYSYGRWRCAGTMMATWKKGAGCCTYMMYKWSSRCM
45       KYRYCCMMSWATGYRYMTYCWTGYYYSYKRWATRSWCCTGWKKRWKATGYMMYTWCGAMC
         ****   ***  .  *  * *  ***** **   .   : ***   .  ****.*..

40       MYMYTTASKWYYCYCCAATTCACYCTACMCRRKYKYAATTCMRYTKCYKYYAYRMAAKRY
45       ATACYCGCTTYCYYYYWRGWYGMTYCGGMAAGGYKYMWWWYARYCKSCKYYRYRACGKRY
               ... *  *       .    . *.   ***      ** *. *** ** ..***

40       RYKTKKYKACACTTKRYCATWCAAGYYRRRKYYTGGKTCTKKMRCRTTTTRKYAAGKWRR
45       RYKWKKYKWMMYYYKGCYGGATTGRYYRRRKYTYSAGGGCGGMRARWWCGGTYMWATTAG
         *** ****      *   .   :. *******  ..      **.*     .*  ..

40       RRRAYTTYMCYTTCCAGGCCGCCCMKWGAAKCCCRRWSACWGRAGWYTGTMKKMMTRATT
45       RRRMTGGTAYYACTARRKYYKYMMMKWRWMTMYTARWGRYTRGGAAYCACCKKACAAMWW
         ***       *:  .         ***   .    **.     .. * . **   :

40       CRMMAYYGGCCTYTAYTTATYGMRRAGGTTWARRRYMYYRAGRRKTCTYTATGGACYKGC
45       YAMMMCCTRYYKYKGTACTAYKARRGATWKWCGRRTAYTAMKGRKYYWTCGCTTGTTKRY
           **       .*.. : ::*  **..  .*. **  *     **     .   .  *

40       CGYRYWMTGRYKYYRWWYACAWYTMKRGRAYYTGYYKCAAKRYGAKKAYTWWMGKKCYTR
45       MRYGTTAGTRTKYYRTAYCGWACCAKRRAGYCCRCCGYRRKRYKGKKRTAWAMRKKYYCG
           *      * ****  *.      **  .*         *** .**  :* * ** *

40       WRMWTRYRMCTCYKTYSYKYWYSCSCAATYYAYCGRKGGACGYAYCCRWGKYSTWRRMAC
45       AACWGRYRAMWYYKYYGTTTATGTGTTGWTTRYYRRGAAWTCCCTYTGARGTCAWRRMWM
            * ***    ** *. .   . . :.    *  * ..    .        .:****

40       CTMKYRRCRYWYRTGGASRATRTRSMRCCCKAWRKYYYYATCTYYRRWRRKKMRAKKYRT
45       TGAKTRAYRYWYRCACGCRTAGKASMRYYYGGTGKYYYYMYYYYYRGAGAKTMRRKKYRC
            * *  *****  . ..*:: . ***    .  *****    ***    *.** ****

40       CCCMYYYYTSCGSMYCKRYTKCMMRTGKCGCYYGKYGRKATRRRMGMKMYWKMGYKRRKYC
45       TTAMCTCYSMRCMCTKRCCGMCMRCTGTTTCYRKYSAKTKRRRMKAKMYWKCRCKAAKCT
           .*    *  .*  **     **       * **. *:.****  *****   *  *

40       KRTAKKRKRGMYKCGGYGTRCRWRKRAMRGTWYCTMACCYCKWYYTCTGKTAAWAMRYTT
45       KGGGKKAKRRCCKTACTAKATGAAGRRMRTGTYYGMRYYTMGACCKMCAKYMRTTMRYYY
         *  .** **   * .  ..      * **   *  *         .  .*    :***
```

```
40              GRRKCARCRTTRWRMGKTYCATAWYTYATRCGAWKATTTKYYKGARYCCGATGMYYYYCA
45              KRRTMRGYGYAATRMRGKYYRWCTYYTGWRYAGWKTKYGKYYKATRCMYSRYKCTYYYAM
                 **.       :  **   .*    . *   . *  ..**:.   ****.:*    .      ***.

40              RTWAYAGKGTWTRGTACTGRYMGGWCCRWTCTCTTTRTTTGAKTTYATMCAYCTKYAYKM
45              GCWMYRRTKCWKRRATMYSGCCATWMYGACMKYWCCGYYGAGGCGTRKCMGYTWKYMYGM
                  * *   .   *.* ::   .     . *         .         ..       .  .*  ** * *

40              TTYMTRYCTGGWARTMTTRYCYTWCTKTKAGRYGMCATTGAKRYMATARTYWWYRYRAYY
45              YGCCAGTTYRRWGGYMKYGYTTCWAAKWKCTRYRMACAGATKRCMCACGCYWWYGYGRCY
                    :      *.  *.  *    *.:* *. ** *..: .:** *.:.  **** *    *

40              CKKKKCKRKRKCRTAYCTRYRWCCKTCARRGRGGCCAKKWGWYKRTAGGGTKGGKKRKAR
45              YKKKKAKRKGKMGCMYTAACGTATKYYRRATAKRMYRKKAKWYTGWMKKRYKRRKKRKCR
                 ****.*** *     * :      . *    *         **   **.          *   ****.*

40              YKRMKKKAAGAAWTRKRAATAGCRWCTGYAGYCTCCGKKYRYTGTSYKRKGGSWYRRWKM
45              YTRAGKTMWAWWWYATRRGACAGGATCTCGTYMAYTTKKTACKKYSYKRKRRSACAAWTA
                *.*  *.   .   *   .* .:..         . * :    **     .  *****   *      *.

40              WYMKAAAKAAGGKACKMGCRYTYTGGKYYKKGTTASRRKGRRGTCRMYWTRCYCYYAGGR
45              TTAKGGTGTGTAKGTKCAGRYGCCRTKYCKKKKYRSRRGARRAATGMYTYAACGTYCTAG
                   *..: :. .*. * . **      ** **  .   *** .**.:   **     .     *. .

40              CRYKRGATCWRTCCWRKRGKAGGYYTGRGTYWYRRSYYYWKKYKKGAAKGTRYRRAARGR
45              YRYGGKCGTWRAYGWRKGRKGRRYYYTRACYWTGASYYCWKKYGKRMRGKGRCRARMRRR
                 **    .  **:  ***   *.   **   *. **    *** **** *        * *    * *

40              RKRYTKTYMKKRRMTTTKTKGMWWTCGTCGKMMCKYGYMYRYASGTRKYTCTYARWGCKR
45              RKRYAKCCCKKRRMYKWKWKRMWWCTACYAKMMYKYAYCCATCCRWAKYKMATRAAKMKR
                ****:*    ***** . * * ***    .   .*** **.*      ..     **. :       **

40              KWMTTTYTKRSYWGKMMCKRRMMTYRKRKKYWGCGCAGCYWWSACRKWYWYKKWMRYCGW
45              GTMGCCCYKASYWKGMCMKRRMCWCRKRTKYWKYRMTSMTWWSGYGKATTYKKACATMKW
                  *      * ***  *  ****    ***.***     :.  ***.  *    ***        *

40              TTYYMTRCAWKGYTKKACARCRYYRKWMWWRMYKKTKYGTTYTGTKRRTAGRTKWWYAYG
45              KYYCMARTGWKKYKKKTTWGMRYYRKWCWWRMYKKWKYACYYYRWKGGGWRRYKWACTTS
                . * *:* .** *.**:     ****** ******* **.  *    *      * **   : .

40              GCTAAMGATYCKKRGWYCRGAKCTGAATYAGWATWKYYRKRMKSTGMAGCKYMKWAAGGC
45              RTGCTARCYCSKKGRATYRAGKYYSWTGCRRWTYWKTYRKRMKGCKMCKYKYAKWWRRKY
                   .:  .  .**     *..*  . :     *: ** ******.  *.  ** **

40              CCGMGTCTCTCRKWCGRKMMYYRYYGKGCATACGSAYKWWCACTTARSRKKKWTACMWMM
45              YSKMCCAGYWMRGTAAAGMMYYGCTRKKMRWWMKSRCKWTYGTCAWAGGKKKAYWMMWCA
                 . *  .    *  ..  ****     *        *  **   .  :  . ***     **

40              RTYAAYRWSCCTCKGAGAGCGKGCKKKAGGYRWATRYCAYKRMKGGGTCTTKYWWYKMWA
45              GCTWWCAWSTTGAKAGTTTTTTAYKTKTTACGTCGRCAWYGRMKRRAATKKKCTWCKMWR
                       **    .*.. :    .. *.*: .    . * . * ***   .: ..*  * ***
```

```
40          YRAYKCTKRKMKYTKKKMRTGRRRCCRCTGTYCGRYASRRWYWWRTKCKGCAYRKMYGCT
45          TGMYKTGKRGCKCYKTGCRCKAAGTGGAAKCTYKGYTGGAATAARCKMKRYTYAKMYRYY
               **  **  *  *.  *        .:      *:.      * * *  :* ***

40          TKGACYTGRTAGGAGTGTCCYRGKMYRYRYSRRCMAKYKCCCRTAYCAAMRKCRCKGCKA
45          CGSTMYKARAGAKWSYKYMYCGKKMYATATSRRYAWKTKYMSGCWYGGTAATGAYKRMKT
              .: *..*:..  .        ***    ***   * *  .   * .:  .   *  *:

40          KGYKYGGMRRWAKRGRRKSMGKTCTKCMYKYMAWTKRGCGCYSWKAWTKGAGATGGAYCC
45          TKYKCTRARRWCGGRAGGGCKKCTWGYATKYMRTWKGRTCTCSAKCWWGRMTCAAAGYST
            . **    ***.      .  *       ***   *      * *.*     .:...*.

40          YATTCTYYMKMWTTCTCRKCCTAKGCYYRCGYTCMSYAYMTYCGSCKTKKRATRCMCKGY
45          YTCYYWCTMKMWYYYYYRKTAYTKKYYYGTAYKTCSYGYMYTYKGMKCTKRRCGYATKRC
            *:      ****     **  . :*  **  .*.  **.**    . * .**      *

40          CAACYKRYCTSWTRRGTCGTKKCGGGRYYKATGAWKGCAKGKYRGWAGKGKTKKRKAKAY
45          GMTTYKRYYCCACGGACAAATKMAACGTCKGCAGWKKTGKKKYRRACAKRKAKKGKWGTT
              : ****  .    . ..:.* ..    *. ..**  .* ***  ..* *:** *  :

40          TTWYYGKGACKYCWGCMAKMRCKGWSKCYRCGMWTRWYKWKKTATGGTATCGKTTTATWY
45          CCAYYKTKGYKCYWTSAGKMGATKTGTYYGGAMTKRTYTAKKYCYKRYRAGRGACCGGAT
               ** . . *  * . .** ..  .. *  .* .* *. ** .     :   :  .

40          RYRCGGRARYYACYCGYRYRMRGKRAKAMYKCKYSCGRSWGWYYYYKCARMYSYRTRMTT
45          GYRGTTRGRYYRYTYRYRYRMRKKRRKGCTKSTCGMKGSTRTCCTTKTCRMYCCRKRAWK
             **   *.***     ****** ** *.  *.. .   *       * .***. *.*  .

40          TYGRATRTCCKTKCTRYRRKYMTCRKCCWYTGKGTYATCRAWKKYKKCWWRSWWAATYYS
45          YYKARYAYMYKKKTGGYARKYMWYRKYSTCCTTAYCCCGRRWKKYKTYTTRSWWMTCYYS
             *        *.*   * ****  **  .    ..  .  * *****.   **** : ***

40          WKGWACGWRTGAGTCYWCTGAAKRCKTGACTYRWWYAKKGATRRYYWCYCCWRCMYRKTG
45          WKRARYKWRCACRWMYWYYRCGTRYGCRCMKYRWWYWKKKGCGRYYWAYYYWRMMYRKYT
            **     ** ..   **   ...*    . .***** ** .  ****.*  ** ****

40          AAWKRTCGWATKYGKAATAKYYRTRGCGKACAWWYYKAKGYCCCKRRKMWYYGRKSGYCR
45          GRWKRCYKWRKKYKTMGWMKYYRWRTTAKRYRTATTKGKTYATTKGRKMWYYRGKGAYSR
            . ***   * .** . .  **** *  .*       *.* *.  * ******  *..*.*

40          YYKCTAGKAMMKATCGTKCYKKKCCRCGCGSRYYRKKYRRYRYMYTRCCSRRYCTTTTWW
45          CCKSWRAKRAMKWGARKKTTKGKYYRTAYAGRYYRGGTGACACCCYGMTSRRYMYYGWWW
              *.  .*  **  . .*  * *  * . ..****              ****     **

40          AKYCAATKAGWCAGWAWRYRATTMGGGAATKTYYCTKCKRTTGAAKAGMTAACCAATMGY
45          TKYTGGAKTRAYRSAMAATATCCMRAATGWKKTTAATMTAYWKTRKRKACGTATGRYMRY
            :** ..:*:    .      :  * ..:. *.  .:. .    : *    .:. .  * *

40          YKAACYYRYMRKYKYKRYRKMTMCTKRKGAWYYGTYCKKKTYTAYMKKTKKAYYKTRAAC
45          TKRWSYYRYCGKYKYGAYRGMYMTCTGGACTCTKYYAKKKCYGMCCGKAKTWYYTYAGGT
             *  .****  ****  **  * *  .  ..     *.*** *     *:*. **.  ..

40          TKAKGCKCTKTTGATTCKYYRTYKRCAGGWTGKYYMSAGGAGGCWYKMYRMCCYRGGRYC
```

```
45     AGGGCTGTGKWWKCCCTKCCGCYGAMMKSWCAKCCCSRSKWRKYWYKCYACYTTGRRGYT
       : .      *   .   *    *     .* .*   * .     *** *         *

40     RRYYAWCGKYRARKYRATMCCAAGGAGGKGRAGTAKRCTCWGTSMRRSYRKKKRKRYWYA
45     RAYYTATTKYGGRKCRGWCMTGGRRRKRGRATKYRKAACGAKGGARACTAKKGAKRYWTR
       * **:   ** .** *.    ..        :   * .     . * .  **  ****

40     RKYRKRTRTYRCKGRKTYRYWRRGGCCKMYYYMRYYWKKYYWGYTATAKWRYRSKAGWRY
45     GKTAGAWRCTRYKRRGACGTWRRRKYGKMYYCMRYYWKTCCTRTGTWRKAGYAGKMAAGY
        *     *  * * * :   ***    **** ******.      :  *  * .* .  *

40     YRGCGGTKYKCRGMGACCAAKYRCTTARYKGGMATCAKKCMTKKTTAYKRRRKMKAKTAY
45     YRKYRRYKCGMRRMAWTATTTCRMYYRRTTKCMWWTGKKYMWKTACGYKGRAKAKTTWRY
       **     *   * *.  .::. *    * .  *   .** * *.: .** * * *:.  *

40     YSRYKKYKATCTCGTTRKYMKAGRRSSSCRYMMYCKRRRKTYMGTKYYRTGGSYKWKAYT
45     YSRTGKCTGYYKYKYYRKCMKGKAAGGGYGTMMYSGRGAKGTAKYGYTACAACYKWKCYY
       ***  * ..  .    ** **.   ...   ***. *  *      *   ...****.*

40     CTTTAGTKRYRRWKAYMRKCGTTYCRKYTRCTRGAGYKYRRRRMRRMKMRRGAKKCMKWK
45     YCAKGRCKACAATKRCCAGTAACYYRKYCGTWASRKTKYAAAGARGATMRGKWKKYAKWK
         :..  *     *      .: * ***     .   **     *  .**   **  ***

40     ARRWRYYYRKMMSYCWRKTTGYCAGCTCMMAKARRRRYKYAATYKMRRMTTCCRRTYYRW
45     TGGTRYYCRKMMSYYTGKAKRYSWKYYMMMMKWARGGTKTMRKCKMRACCCTYRGWYCGW
       :   *** ******   *:. *.     ** *  *   *   . ***      *  *  *

40     CTCYRCAYTYRKKCTGKKGYYRCAYKKWGGKRRWKWKKYGGRWWKSMTTTGKRACAYYYW
45     YWYYGYMYWYRKKAYRTKAYYRSRTKKTRKTAAWGWKGYKAGTTTCCAWKSKRGTRTCTT
          *   * ****.  .*.***.  **   .  * ** * .   .. : ..**.

40     ASCMCGRRATSCRKRYSGCKYACCGCTCATCYACYKRRACTAACWWKYCWGKRKRTCKTW
45     MSTCTARGGYGTRKRYCTAKCTAYTAAATCYCGSYKGGRSKGWSWTGYYARKGGRKTKYW
        *   .* . . ****. .* :.  .:.:   ..**   ... .*  *   *  *. * *

40     RMTMYATTCAMCKTKYSMMWKCGAGKRYTYMKRYYKWYKACGGCTCMKATTKRAGKKTCK
45     RMCCTTKCTGMMTCKTGCCWKMRWAKRYYTMTRYCKWYKCTRKYWYCKGCKGACAKKCGK
       **   :.  .* . * .  **   .***  *.** ****.       *. .  ..**  *

40     KRMRKWTCMRYGTTTTSSCGGRMAAGKGCKKMYMKTTKCMACAYKYMRMMWGKCKTRYKT
45     KGMRTTCTCRYAAAGYCCYRKAMRWRGRMKKCCATCGKYMWMMYKTMGMATKKTGARYGY
       * **.    **.::  ..    *      **   .  * *   ** * *   *  :**

40     SRCGGRTTYYYMWKKYAYKWYYKCTACKGWYWWAWRYKCKYYKRWKACGRARRRRMKKWR
45     GAYKKRYKYTTCWKTYRCGATCTYWTMKTWYWWMWATGYKCYKGTKTTAGGGRRRMKKWR
       .    * .*   **.*      .  : * **** *    * **  *: . . ********

40     WTYTRKAGAYRKTTATATACKARKRACTCTKKRRYRKCYWAAGRCKAACAYKRYYWYKWC
45     WCYKAKMTGTAKWARCRGRMKWRKRMYKAGKKRRYRKYTTRRKAYKWGARTTATYTYGTY
       * *. *  .  * :      * ***  .. *******        * ..  .  * *

40     TGRRYTCCGAGAAMCKYTMYMKRSKKCKKRKRGYWYYTGAMYYRCGKRAAAATAGWKMSC
45     KRAGCATYRRRWWCYKYYCTAKRSGKYKGGGGRCWYCYRWCYYGYRKRCGCGCGAWKCCY
```

```
        .    :         **    *** * *      **     **   **.... ..** .

40      KTKRRTYGAARYGCMRKRKKTYKGGYAKRCTACAATGGGRTAYRCCWARTCRTCYGTTWK
45      KCKRGCYKMCACRYAGKRKKKCKCRYWGAGAGTRMYSRKGYRTRYTWGGWYRCMYSYYAK
        * **  *  .      ****. *  *    :.    .      *  *.   *  *.   *

40      KKACCCTTCTKKKSARKAKGGGTKAGGYTGWYYYRCACYRTGTYRRTRTSCTCYGAKYYY
45      KKGMYYWYMCTKKGRRKRKATAYKTRRYYRACCTGMWYCRWKKCAGCAKCYCSCACKYYY
        **.       .**. ** *. . *:  *           *  .     ..  .  ..****

40      AYACAGAKCYYWKYMMRKMCRKKYYTTAAYKRWRRKKGYACSYRTGCRYGMKTAKRYMKY
45      WYGYWTWKMYYWTYAARKAYRKKYYYYRRTKGTGRKGRCMYSCGCATGCKMGWWTGYCKY
         *.    * ***.*  **  *****     *   **     *   .    *   . * **

40      WYMYKRRMYMYWRKRRMRRGGCYGRGCKGAAGRCCAYYRKKKTTCTKGKRMYCKTGRAAT
45      TTATKGGMCCYWRKRRMAGAATYKRRYGKRWRGATMTYRKKKCCTWKRTRAYTKCRARWW
            *  *  *******  .. * *        .   *****    * .* * *

40      AYYGGGTACKGGGACKKGKRSKYCKYACTKSGMMRKCRGKMYRSRCYKKSYWYKCAKSMC
45      RYCKSRYWYKRRRRMKTAGRSKYTKYMAYKCRMCAGYRTKMTGCATYKKSYWYKTGKSCM
         *  .    *     *.. **** ** . *. *    * **  .  ******** .**

40      TGAYKYYARRAGCYKMKGGYGRMRKKWGRYAGGRMKSYMWKYMCTKACTGAGTRKCTARY
45      ARRYKYYGRRGTTYKMKACCKAARGKTRGCRRKAAKGTCTKCAMYKTTACMAWRKTGCAT
        :  ****.**.  ****.     * *         *.   *    *: :  . **  .

40      CAATYCACTGKGRMWRKARKGYKKYYKCRGYRSKAYKYRGCRCWCYCGACKKKTYYYWRY
45      GTGCYMMYKRGRAMTGKTRKACTKYYGMGRYRSKRYKTGRYRGAMCYTWAKTKCCCTTGC
         :. *   .    *  *:**. .***    **** **    *       .*.*

40      KYYGTCTKKGAATYTTRATGGTTTKKMMKRWAGRACTCKWCRCTTYRYMMKMYYKRKRSR
45      KCYRCYKTGRCGCCYARRWRTWWKTGMMTGWWKGMYWYKTYRAYWYRYMCGMYCTRKGCG
        * *   ..  ..   :*      .. **. *       *  *.  ****  ** .** .

40      CYKAMKGTTYKTCRATTGWKGTAWYYMKACRCRGACYYRKRATGTCYRKMGKYMRKCYGA
45      AYKMCKRWYTKYYRRWWRWKKKRACYMKCYRAGACGCTAGATGTCACATCRKYMRGYCRG
        .**  *    *  *    ** .   ***. *. ..      :   .  .  ****    .

40      TGKYKKKAATCKWGKYTRTCTGGKGATTRKYMAACGTTYYYTCGKCKKCYCCCKRKAAAS
45      KKTCKKKWMYYKWTKYWRYMWKKGATCCRKCAMGARYYYYYYTAKYKKMYYMMKRKWRRS
        . . ***    ** ** *      .:  **    ..  ***  .* ** *   ***   *

40      YYYYTRYKRWKWWRAMYYRKKRWGCYRKSKKKWRYRKKCGCTKYSRKTAKKATTARYKMA
45      TYYYYGCKGAKAARRMYYRKKRWRYCAKGKKKWRYRKKARYYKTSAKAWKGCYGTGCKAR
         ***   *  *  * ********    *.*********.   * * *: * .  :  *

40      TWAGTKMWMMYRASTKRKWYRWYRAMMRRCGCMKKCYKCTGYGRKKRYMWYRYTACCYYK
45      GTRCGKCAMMTARCYTGKTYRTYGWMMARTAACGKMYKYYSYRAKKGCCWYRYWRYYYTG
             *  **   . . * ** *  ** * ..  * **  .*  **   ****    *

40      RWKMMMTWTTAWRAGRWRCACWKRKGTGAGMRCTRYCTWKMKAARGAWYKYACGMRYCKT
45      RTGAMMCTYCRWGRKAWRYWTWKRKKYRMAMGYYGCMKWKCKRMGTWWYKYGYKCRCTGW
        *   **     *    **   ****    .*      .** *     ****.   *
```

```
40          WGAGSKKTGMTGMSWGAARKKTATYTGYWWRAAGCTWKRRMYKCKRGMCRKAYMGTAATY
45          WTTTGKKKRMKKASWKGCGKKCGCCAATTWRGTRYCWTRRMTKTKRTMMRKGCCRCGGCY
            * : .**. *.  ** .. ** .  :.  **.:   *.*** * ** * **.    .. *

40          RTCTGACWKKYCATCCTWYCCTRTTCTATMRWTAYAKGAACTTAAWYGAMYGGCRTTKTA
45          RATYRRSATKTMTCGAWWYYYYAKWYATCMRWGRTRKAGCGAACGTCARCCKKMAYYKYW
            *:    . .*  :  . **    .  :: ***    *... ::..  .         *

40          RRKCKGMTKTTCYAWCTCCCCYRGGGCGCRGKGCGACYCGKTGGKMRRYYGKGSYWKRYK
45          RRGYKTMAKGYYYGWYCTATMTGKSRYRYRRKCGTWYYYAKYSAKAAAYCKKASYWKRYK
            **  * *:*   *.*   .     .    * *     * .* ..*   *  *.*******

40          RMATMMYKGTTRKYKCMRYCCYYKYKMSRCGGTYMRYCCAYRYGTTKGTGWCYCRCTKKM
45          GCRKMATKRYCAKYKYARCYAYCKYTMSGMKKWCMRTTGRCGYKWCKKYKAYYTAMYKKC
               .*  *    ***  *  .* **.**      **      *   *     *    **

40          CRTYRYYYRWYKYYTWTYKRWYYARMCRYSYKYYKYCRRRAKSWGCTGATCCGGCCRYKT
45          YAGTGCCTGAYTYYAWKYKAWYYTRCSRYSCKCCTYYRRRMKGWKYGATCMTTATMACKK
                      *.**:*.** ***:* .*** *  .* *** *.*   .:    .    *.

40          MMKRRGTSAMATAGTCYKRRMCYYMYKRYSKAKKGRWKGGKWKYGKMYRCRCRYKSKGTM
45          CMKGATYGMCWKRCATCKRRMTCCCYKGTCKGKKAGTKRKKWKTAKMYRARYRYKGGSWM
             **    .   .  :  ****    **  .*.**.  *  *** .****.* ***. . *

40          KRRTAGRRKAGGRTGKMWRYWYWMRKRKTTYTTGCKWKKGKAGACMCCCKKCYAMRR
45          GGAWGRGRKRRSAWTKCTRYWTAAATAGKKYCGRGKWKKRKMSRYAMAYTKATMMRR
                .  **  .   *  ***    .  ..*    **** * .    . .*.  ***
```

The pairwise alignments of two accessions 40 and 46

```
40                  KYMWCCRGYSCGYMRWRRKAKGRGYMGSRRRYRWYWRRRYWCCMMYYMKRWRKGRRRYRR
46                  KCCTYYGTYSARCARWGRGWKARTCAKSGRATRTYARRRTWTMMMYYMKRTGGKRAGYRR
                    *       **.   ** *  *.*    * *  * * *** *  *******    *  ***

40                  MTWRYWGSRKSRRYYWSWAGRCYTGMKWWMRCYKYRSRTRKKYYTGRYYRRYGATYWKCW
46                  CYAGYWKSRKSRRCTWSWRARMYYKAKTTMGYYKYRSRKGKKYCWRACTRRYAWKYWKYW
                        ** ******  *** .* *   *  *  ******. ***      ***. .*** *

40                  MRYRKRYYSYWWYGKYRYRYMKAKTGYYRRMYYCYYMYKAYWGGGRRSRRRSWYRKRRYA
46                  MRYRGACCSYWWCRTYRYRYMKRKWKYYRRCCTYTYACKRCWKKRRGSRRRSTYRGGGCR
                    ****    ****  .******* *  ****     *  *  *   * ***** **

40                  TYKGTGKYRAYYYYWRTRCTGGCRYYYRYWMRYKTKKRRYGWWMMRCGATKRAGRARAGR
46                  CYTCYRKYRGYYYYWRCAYCATTRYCYAYWCACKCKKRRYTATMMRTRRWGRCRRCGTKR
                     *.   ***.******    .  ** * **   * *****   ***     *. *. : *

40                  RMCRWCTTYYYKYGYYYYYYCKTWAYYMGCAWAYCCKKWRYKRKYGRYKYCYRGGATWKY
46                  RMARWMYKCYCGTKYYTYYYYKYWTCYMRAGTTCTTKKWRYGRGYKGTTYYYRAAGCWKY
                    **.**  . *    ** *** * *: ** .. :   ***** * *   .* **... ***

40                  MMRCMYKYKYYRAYTYAGKRAYATCAWGYMYKRAYAYYMKMWYRRTCKWGWYMKMKRGKG
46                  MMRYCTKCKCTRTCKYRTGRTYGCATWTCMTGGGYTYYATCWTRRWMKARTYMKMGATKR
                    ***   * *  *: .*   *:*. .:*  *   .*:** . * **  *   ****   *

40                  TMKRRTTCCTAKAYWYMRTMMWYRYYWGSYMRYYRWYWYMMMAGGKYAGAAWCWKWSRRR
46                  WMGAACYTYYGKRYWCAGWAAWYRYCWKSYMRYYRTYWYMMMRRRTTTTGWAYWKWSGRR
                     *        .* **      **** * ******* ******   . : .   **** **

40                  SRWTRGYYRWCWGGWWMKYMTAACYKWYWRYMYMYYSYYYWYMSKWKTYKYRRAMYKRRK
46                  SGWKRTYYRWYAKCTTCGTMWCWYYKWTWRYMYMYYSYYYAYASGTKWCGCRRMCYKRAT
                    * *.* ****         * .  *** ************ * *  *    **  *** .

40                  YYRWKKCAAYKCGGRKYRRMYSKTCGRYRWYMYGCKRKAACCYYMYRMWRMRKSRGMRCM
46                  CYRWKKSWGCKMRAGKTRRMYSKYSRGYGACMCKMKRKRRTTYYCTAAAGAAKSGACRYC
                     *****. . *  . * ****** .  *   *   ***    **        ** . *

40                  YRRKAWCTAWYMACYKYCAATAYSRRAMTMKYTCYRCKKATRCYCCGTCRYTMRAYYTTK
46                  YRRKRTTGWTYAMSCKCTTTGCYCGGGCGMKTASCGYKTWKGMTYYKYYRCYMGGYYYWK
                    ****      *  . *  :: .*.  .  ** :.   *. .        *  * .**  *

40                  KTYCGYRWAASCYYTGCTKYCMCTCGTMAYTTTCAAYKSCAAKTYAGCTATGGWTCRAYK
46                  KYYARCRWWCCYYYYKSYKYYMYCYKYMRYCWYYRGCTSYCGKCYCCYYWYCAAWTRWCT
                    * *.  ** .. **  . ** *     * *     . .* ..* *.      .   *  .

40                  YKKRCKTGRGYKYKRRRATYGRRTKTAGYATCYACTRYRYYKYACYAGCMTCACGGYCWR
46                  CKKGYKKRGRYKYKRRAWAYARRYKYGTCTCTCRMYRCATCKYMMCGAMACTGYRKCTAG
                     **  *.   ******  :*.** * .  :      *    **   ..    .

40                  KCACRCYTAYKAMGKYYYAYRRGKKCCRYGSTKTGATMARRRKWKMAKKTAAKGCKMMRY
46                  KYMTAAYYRYGGCAGCCYRYRRRKGYYACKSKKYRMWMRGGGKWKCMKKYRMKRYKMCRT
```

```
            *    .*  * . .   * *** *      *.*    *    ***  **   *  ** *

40          YATRMRRCGKRYCTCAMCTWAKTGCCCCGCRCRTSSKYGATAAMAMSWRKTRRTGCGAGK
46          CRKRMRRTRKRTAYMMMTCWTKWTMYYMATRGRGGSKYRRYGWMMASWRKCRRGAAATTG
              .****  ** .   *  *:*      . * * .***   . *  **** ** ...:

40          RAAGTAACGRYRGKRRTAYRCGSAGMMGGMRGTCGASYGCCTGACCGKKKTGKYCTGMMY
46          GGTRKGTGRGTRTKRRCRCRTACGCCMRSMRAWYKWSYKGTYRRSYRGKTYRKTMKRMAC
             .: ..:    * ***   * ...  * .**.    **      .   *.  *  . *

40          WYMRKGTTCMRWAYGAGKCTTAARTCTKSYCRTMYYATGYRWTYRTCTTCCCYYRKGACC
46          WYMRKRYAYMRWMTCRKKGAACGAYYYKSYYRCMTYGAKTRACTGYYWCTATYCGTKMMM
            *****  : ***     * ::..    *** * * *.:  *         . *  .

40          RGWWMKRCCGCTTTAYMKYMYYMMSTKTCMGGCRKCCTWKMYGRYYTSRWRARYKCRYRR
46          RRTAAKRYYTTKGYRYCKCCYYMMCYKYYMRKYGGYYKWKCYRRYYWGATGRGTKTRCRA
            *    **    .   * *  ****. *  *       .** * *** .      * * *

40          YYYRMGKYAYRAAARAAGGAARWKRTSMKRRRCRSYRTGGGRGKKRYWYAYRKRTRRTKR
46          YYYGMAGYRTAMRRGRGATCGAWKRGGAKRRRYRSYRWRRKRSGKRYWTRTAGAWRGCKR
            *** *. *        .. .. *** . **** ****    *. ****       *  **

40          RYRCKATGKTGCCCGCCGWYRYAWRRGGCKYMYYWRYACTMRYYKWKKYYGGYTAAYWKR
46          RTRYKCCRGAATTYRYYRWCGTWWRRRKYKYCCCTRCGYGMRYYKWKTCCKRTGTWTAKG
            * * *.   :.       *    ***   **    * .  *******.      :   *

40          MGRSTGWYRYYRMACRMGCACTTAKACKRWYCAWYWSTAGKKCTATARYKYGGKMKRMKC
46          MRAGYAACGYYRCRYAAAYTYWYMKTTTAATYGACWSYRKKKYKRYRRTTCKCKMGAAGY
            *  . .   ***     . :    *: .    .  **   ** .   * .   **

40          KACKRTRKMMKYKTATATCTTCCRCYYSRYKTYGKRMCGCRRYRTSKKRYGWCTCATCKY
46          TWMKGCAKMAKTTWRYCWYYCYMRYYYSRTGYCAKRCTAYRRYGKCGKRCTTTCTTYSKT
            .  *   ** * .   .      * ****    .**  . *** .. **      : .*

40          KAKRCKYKMSACCWARRKRGRTMYATTCACRTKAGYTKKTRYYKGKWKATCMTYYCAKTC
46          KWKRSKYKAGTTYARAGGGKRCCTTKCTGMRCKTACKKKYRYCKKKWKGWYCCCCTGTYG
            * **.*** .:         *   :.  . * *:. .** ** * ***.       ..

40          KYGRGKWWKTCMRYYGTTTYTSCGGRCGCMYSMKTTGCATCMKTGAAGCGYAYKYMRKKK
46          KTRRATTWKCTCRYYRWWKYYSYRKATKSCCCATGCRYRCYMKAAGTCATYMYKTMGKGK
            *  *.. **   ***   .* *      .  . .       **:..: . * ** * * *

40          CAKTSRRATAYYMKWWWYAKAAKYCRRCYWATYYCTWKGKKRYMKKWKYTGACAKGKYWW
46          TTGYGAGGAGTTCKTWWYCGGGTYTRGYYWTWTTTYWKRTGRCCKKWKCWRRYRKKKYWW
             :  .  .:.   * ***. ...* *  **:     ** . *  ****      * ****

40          WKKKCKKKMKYKAATTGYRRRYRMASRRRMGARGAGWMRWTTAGMKGGMYRRRRTGMYCC
46          WGKKYKKGCKCKMMYCKYAAGYRMRGGARMRTRARRTAGWYCRRCKRRMYRRRRWRAYYM
            * ** **  * *     *   *** .  ** :*.     *     *  ******   *

40          CAYKMRCRCKGCCWTGGAMWCGYCRYKCYRGYWRRKSMKGATKCKAKATRRRARRRYRYK
46          SWYKAAGAYKRMYWCRSWMWTKYYRCGTCGRTARGGGCKKMWGYKTKRKRRRMGAACGCK
            .  **     *   * .  **  * *        *  . *     *:* .***       *
```

```
40    KAGACKAYCYCGKYCTTCTYYKTTCTKCCAGGTTTAGRMGTYTRGTWGSYGAMTYCSCTT
46    KWRMYTGTTYGRKYSYYYWCTGYYYYKTAMKRYYCWKRMKYCWGRGWSSYARMYTYGMCK
      *    ..  *  **.           * .        **       *.**. *   .  .

40    AKKKYRWACTYMWWKWGGCKWYTKRYYKWYWKWATRACYAARKGYCARCYRCTRGYMYYA
46    WTKKYRWRMWYATTGTKRTKWYCKGTCKWYWGTRWGTMYRWRGRCMRRYCGYYGRYCCTG
       .*****   *        *** *   ****     : *  *     *       *   .

40    GCKYTAKKYRGCCKCWACTCKMYCTGCGAWGAAKCAYYAAKTTGKRYMGGKWKGRMRRCR
46    RYKYGMKKYRKATGTTCTYYKCTYKRYRRARMMKMMYYCTKCWAKGCCATKWKKRARRYG
        **  **** .    .   *   .        *  **.:*  .*   . *** * **

40    RWGTCRGTTTGAKYCRARWTRRYSCTYYYRTRCWRWAKKRCCATCYTAKAAKTAAMKKTK
46    GASKTRCYYGAGKCTRRRWKGGYSTWYTYRKRATRWGKKRYYWKYTGGKMRKWWTCTKAK
        .. *    ..*  * **.  **  * **.*. **.***   .   .*  *  : .*:*

40    RAYYSKCYGAYKSRCRYKGSKRCGWRWYKSGTKTCGKAWYRYRSCKARYRKCTKCKACYM
46    GWYYSKYCTGTGCAMRTKAGTGGATRTYTSRYKWSRGMATGYRSAKGRYRKMKKYKRYYM
        ****   .  .  * *...  . * *.*  * .      ***.*.**** .* *  **

40    WYKYGYRGKRTAGARGYATYYCKRRGWGWTYYGGYAYRMYAWYYSYTRCGTGTKGRKKAW
46    WYKTRYRKKRYRKMATYMYYYSKRRKTRTYTTRKYCYRMYWACTSYKRTACAYKKAKKWW
      ***  ** **      *  **.***         *.****    **.* . . *   ** *

40    KAGCTTRYCCYYWYYWRAWKKYKKGAACKYKKCWRWRSWWKATTSWKTGWACWTCTGRAA
46    GWRTKGGTYSYTTCTTRRWTTCGKRCGYKYTKYTRTACTWKTCKSWTWRARYWGYYRRRR
          .    .*     * *..  * .. **.*  *  . **: .**.     *    *

40    TTGTARCKTTGTYRMMMTYRKMCAACYKCCWMYRKKRRGATTKRWYTATGGATCKRTMWK
46    KWKGCRYKYWKWYRMMMCYGGCTGCYYKYYTCCAKKARTGKWGRWYWMCTAMGGGGGCWK
      .    .* *     ***** *     .. **        ** * ..  ***      .       **

40    KMTRYCMGRTTGGTTKCYTRAAGRTCTGGCAWRRMYRARRRAYYMTGYCCCMKWGAAGKC
46    KMWRCTCAGYCAKCGTMCWRMGAGYYYKTAGTARMYRCGRRMTCMKKCYMMMKWRWMRKM
      ** *    .    .    .    * ..        ..  ****. **    *.       ***     *

40    WCRYKRGTRSCRSTARWTGTMKKMMRGMYYGMCCGKYYKRRRATMAYAYTRRYRMTYTTK
46    AYRYKRRYRGYGSCCGAYRYCKKACAKACCKMYTRTYTKRRRGCMMTWYWRRYGAWTYYK
        ****  *.  * .       **         *    .* ****. *    * ***       *

40    AATGYRYWMYRYYYRGGWWCWYTATAAKKCARRKAYRYYYKWMRYAWRYAKCYATAWWRG
46    CCCRCGTTAYRTYYRTRTAMACCGAGTTKYRRRKGYRTCCGWMRYRARYRKYCTGCWAGR
      ..       ** ***         .:.:.*  ***.**     ****  ** *   : .*

40    CYTGRYCAYRMYTRMWCTCKGGMTTKKGKGCCSYGTYKMYKKMKRYYRCCRTKCKKCKRR
46    YYCRGCATYRCCGRMWMWYKARMWYKKKKAMMSYAAYKACKGCGRCYAYMRAKYKKYKRR
       *     .:**   ***    *. *  ** *.  **.:**  *    * *    *:* ** ***

40    MTTKTMGKGMMWGYTAYKKRGCACSRCAWGYYYARWKWTTTGYTKRSYWKKMKRRMTCTY
46    MKWKWCRKRMMAKCWTYKKRRYCYSRAGTRTYTRAAKTGCCRCYKASYWKGCKRRMWMYY
      *. *    * **     :****  . **..    *     *        * ****  ****    *
```

```
40          TMTYYAYRKRYWGKGAARSYWWSRACTAYWYMRKRWYCCYYKTTAYKACRKKMCTYRKKY
46          YCWCYGYRKRYWTKAGGRGTWWSRWTAGYATMRKGTCGYTTKKYWCKCYATKAYCTRKKY
                *.****** *...*. ****  :.*  ***       *.   *.  .*    ****

40          YMRTMRRWRRYYKTRYGKRRKKKRYKKRAKKMGKYMRYWRKTGRRMTRRKRYKMMRKGYM
46          YCGYMGGTGRYYKCGCKKRRKKTGYKKRMKKCRKCCRYWRKKRRRMKAGKRYGAAGGKYM
            *   *    ****    *****. **** **  *  *****. ***.  ***      **

40          YWTWGAARAKYKYYRGKKACACGKKMKAYCKGWGAKKTYTGRKSYKGAAAKSYMKRSKYC
46          YWYTKRWRMKYTTYRTKKRYGYKKKAKRYTKRWAGKGCYAARKSYKTCGCTSYAKRSKYT
            **     * **. ** **  .  ** * * * *..*  *:.***** ....** *****

40          WYKTSTTGMCRKTCCACGKMYRKSRCYKYSYWYKSMRCTGAYYKMKGKYYYARRTYKRYG
46          WYKYSGARMGAGCTYCATKMYRKCATYKYSYWYKSMGMARRYCKCKKGCYYGRRKYKRYK
            *** * : *      .. *****.  **********  :  * * *   **.**.****

40          MRKWRRCKWYMCCTACTYKGTRYTGYCAATKCACTGGMYKYYYTCCKTMWTTYTSTGCCR
46          MRGTGATKTCAMMWGMAYKCWRYYRCGTGCKMMYKRRMYKCCCKMYKWCAYACKCAAYTR
            **     *      . :**  **    :. *   .  ***   .  *    : ..:.  *

40          RAYAWTGMTYCATYGWWATRRAWTCRYGAKYYRKRRYTWYRYTYRRWYYTAKCCYYYYRW
46          RRYGACRCYTYGATRWTMGRRGTWSRYRRKCYRTRGYAWYRYKTRRWTYYGTMTYYYCRW
            * *.       .:  *   **.  .**  * **.* *:****. *** *  ..  *** **

40          RACRMATKWRYTRGRGARGGGGYKTAAKWMSRGSRMMYYKWARRRYYCYCCCYGWRCKCT
46          AWYRMTGKWRTKGAGRMGARTTTKCGTTTMCRKSRCMTCTTWRRRYYTTMTTTRWRGGMC
               **: *** . .    .    * .:. *.* ** *  .   *****       **

40          GRAMRYGSMRACWTTYGMYTMAMMYRRCCGYRYKCKMYARGTCKATWTYGTGKCGKRWMR
46          AAMARYASMRCGWCKCRMCYCWMCCGRYMRYRYKTKMTRGSYYKRKACYSYSKTAKRAAA
            .   **.***. * .  *    *   *   **** **   .  * .  *. .* .**

40          RYGKGSYWKCTCCRYKKKMATMMYKGTKKRTRWKMGYCRYRKYCTTWMSKMCYMRYKCCC
46          ATKKASYWKTYTYRYKKKCRKMMYKRYTKRCRWKAKCARYGGYTCCTMSKMMCMRTTTMG
               *.****    *****  .****  .** ***   .**  *    ****  ** .

40          YSKYYRYTYRGMCAYGGGCRCKKMRYRYYYRWGYWKCYRWCYYRTCSKRMRGKGATYYKY
46          YGKCCGYCYRKMYMYRRRYAMKGCRTGCYYRWAYWKYYATMTCGYSSKRCRKTRGWCCKY
            *.*   * ** *  *      *  *   ****.*** *       .*** * . .   **

40          WGKYCGGCCMRYRTMMWWSTRSMKYKYMCKRYSGYRGYRWKMMGGKWGTTTCRYSCKCGT
46          WKTTYKRYAAACRKCMATCAGGCKCKCCYKGTCRYRRCGTKMMRKKWAGWKTRYGYGMSW
            * .     .   *. *  .: . * *   *  . **    ***  **.  . **.   .

40          RMRMRTAGYYGRRKRTWGGGKMRWMKWYWWRKRRCKWYYMKKTCGGTWMMCCTACYCTKK
46          RMGAAWGRTYRGRKAWARKTKARWCKWTAWATRAYGWYYMKKGATTKAAAMAATTYYKTG
            **    .  *  **      * ** **  * .*   ****** .  .    .:: * ..

40          CYMRKTCTCAACYRTKMYMAAGSSSRCRYMKACKRRTKTGTTYYTGGTWYTTKRYRKCWA
46          ATMRKYYKYTGGCRYKMYMGGKGGGGYGTCKRSGRGKKYKYGYYCAAKATCCKACAKYTR
            . ***  . :.  * ****.. ...     * . * .*    ** ...    *   *

40          MYCAMYRGKYYGRRRRMRTTMRMTARGAKCMKCKCTRCAKKWRWRYYMATYGCCTCCMAG
```

```
46          MYYMMYASGTYKAAAGAAYYMRMYRAKWKMAKYGGARTGGKWRWRYYMRKCRSYYMMMRR
            **  ** .  *         ***     *  *   :* . ******** .  .    *

40          CKAYWWRYARKGACKCACKKYRKRGSWTRYWYKMGKATRKYYRARMYYYKKYRCKYTGGK
46          MKMTWWRTWRGACAKYMTKKTRTAAGTKRYWCKMRKWYRKCCGTRMYYYKKYGYKYARRK
             *  ***  * ...*   ** *. .. .*** ** *  **   :********  **:  *

40          MYCTKKGYMCKYRKWGGKKRRWKWKYYAYGSTMWWKSMKKTRTKKAYKCGWYCRWMWYWK
46          CCAYTKAYMSKCGKTRKKTAAWGWKCCRTKGGMTTTCCKKKRYKKRYKMKWTYGWMWYWK
              . .*.**.*  *   *.  * **     . *  .. **.* ** **  *   ******

40          WYYWRYRKWRMRYWRGRGTAWRMYTCCTGMYTKTKYYGYKRTKRRTYRKWCCYTYACCYY
46          WYYTAYGKTGARYARARRCMWRMYAATCRAYWKGGTYAYKRCKGGGCRKWGGCYTTATTY
            ***  * *   ** *.*   ****:.    * *   *.*** *    ***     :.  *

40          RTWYGAKKKRRTKGCAWWYYAACYGGKAKAKSSMTTGRTGKMWGGAYKRMKRATGGSYSR
46          GCATKMKKKGRWKKYRTATTMRACKKKGKGKSSMYYSRAAKMWKRWYKRMKGGKKRGYSR
                  *** * *         .   *.*.****  .*:.***   ***** ..  .***

40          YRYKWGKYKTATAACRCCYWYCASRKGGCMTCAGMRAWTATKKYRRYSRRYMYKTCRCCC
46          CRCKWAKYKYRYRWYRTTTWYYGGRKRATCCAGCARTWGGAGGTGACGARYMYKYYGMTT
             * **.***      *   ** ..** .   ..  *:* .:      . *****

40          ASRRYTCCCTTTTMSWTTYCTGYKTTRYACRTTCRYCAGWAWTAARYRGAYMKKYCTTYR
46          RSRRYYMYMYYGWMSWGGYMYRCKAGGYRMGYYYGCTRSAMAATCATARTYMKKYAAYCA
             ****        ***  *    *:  *          .   ::.    :*****.:

40          KGKRCCAKACTGTTAAMYYGAACMRYKYKRYRTTACKMKRKTGAWYYGTYCRTACTKRTY
46          GKTAYYRKTTGCCCGRMCTRRWSMRYKYGAYRCWWAGMTGKKACTCTKYYAAWRYWGACT
              .    *:     . *     .*****  **   . *. *...     *.

40          KRYYTSRYRCKCTRCCCTTGAAACYKGGGKTTGTAAYYRYKRRRRGCACAGGTGKRYAWK
46          GGTCAGAYRYTMYAYYYYYSRGGTCKATAKWWKYRWCCGYGAGGARYRMMKSCAKRYMWK
                :. ** .        . ..  *. .*         *           . .*** **

40          CAGCACTKKRYATRTYGRRMKRYYYCCKKYKRYCKKRKTGGCTRRARWKCGGCCARAWYG
46          ARAACYAGKRCCAGCYRGGCGRYYYYMKKYKRYAGKGKCAATAGAWGTKYAAATWARWYK
            . ... : ** .:  *     ****  ******. * * .. :     * ...    **

40          RKGRKTTGGKYRYKMCCTTKAGWRWMKACGAKYKKTWCKAKAGATAGAAACGWTWYGGCG
46          AKRRTCGTRKYRYTAYYYWKMAWGWMKRYKRKYTTYTMKCGGRGACRMRRYRAYTYAATR
             * *.    ****.     * .* ***    **..   *. . .:.         *..

40          MCTTTRGKKCYWGWTARSYKRKCCSMWYRMRRWKMWYMAAAKAAGGTACKGCYGCMWRRK
46          AACCYRTKKMYWKTAGGSYKRKYMSMACAMRAWTATTMGGTGTGTACGTGTTYRSATART
             .   * ** **  :. *****  **   ** *.   *..: :. . .    * .   *.

40          GKGGCYRRRSRGYWKGRRRRRYWGRCYRYKWATARTGGKRGKKGKKYGCTMTYMCGATTG
46          RKAAATAGASRRCAGARRRRGCTRAAYRYTWTAGRCTTKGRKKKKKTTTGMCCCYRRYGA
             *...    **    .****     .***.*::.*   *  ** **    *        .

40          WYCCASRWTTGKCGYMTTAYYGGCAATACCTWYRCCACACTYYKGGRCACGTTMRRGCGS
46          WTYMMGGAWYTTYTYAGGGYYRKYGTGGYSCTTRYYCYWAGYYKARRYMYKYWMRRKMAG
```

```
                 *    .     .  *   .**   .: . .    *  .  . ***. *      ***  ..

40               KMATATKKCGTCWACATYTRTWRYRSRKRKGTKCMWTYGKKYRRCMWTACKTWSYCWAGR
46               GARWRWGKMKYYTMYGGYAACTRYAGGKAKRKTTMWYYRKKTRGSMWYRTKCWSYYTTAR
                       *       . *:   ** . * * .. ** * ** * .**   * ***  :.*

40               WRYWGGCKKRMWRATKCTGTTRMKCTTTAGGKYRKMWATAGTGYCGYYKACGRARMCRWK
46               AATTAASKKRMTRCGKMYSYGRMKMGAATAAKCRKMWRCMTGAYTACYKTYRGRGMYRWK
                     ...**** *. *   .  ***  ::::..* ****     .* . **:     * ***

40               TKGAYKTWMKYYKRAYYRCRYWTAARKRKRMYRAYKRYAGYGGRYYKWCGTGRRYGYWCG
46               CGTRTKAWCKCCKRWYYRYGTTKRRRKAKRMYRRTTATGRTRKRCYGTYRKRAGCATWYR
                      *:* *  ** ***    .  ** *****  .  .    * *    .    . *

40               AGKTCGAGCRKKMCWRRSKTCCCCYAMYYGWYRYCSKAACSCMSRWTATGRRTKAGAAKY
46               RRKCYKRAYRKKCYWRASTATYYTCGCYYSAYGYYSKCGMSACCGTATGTRRCKMAGGKC
                   *     . ***  ** *.:      . **. * * **.. *. .  ::  ** * ...*

40               CGGCMRKTYKGAGGYARTYWAYCCAGKTGAACKGRGCWARTSYWMKAGCGCKYKWWRCAY
46               GKRYAGKKCTSWKRYWACCTCCYTRRGYTWGYGKGKTWRGCSYAMKRRYRMKTKWWRMMY
                       *. ..   *      .          .      *   ** **     * ****  *

40               KMYARCCAAKMKYRWYYTTGYRCACTYARRKAYGGAYYYYKYCRKTWWGCKGSGTYMTAG
46               KACRRMGCGKAKCRACCYKATGSWYYYWRAKWCRACYYYYKTYAKYWWKMKRCRYYMKGA
                 *    *   ..* * *     ..  .     * * *    ..*****    * **   *  .   **...

40               TTARATKRKYYMKCYKAYKRYMRKYKGAWYRKYAYYMYKYRRYKCYMGYGACTKGTYKRG
46               KKRGWCKRKYYMKYCGGCGGTMGTYKCRTCRKYWYYMYKYRRYKTCMRCKRYYKKWYKGR
                 ..     *******    .     * .**     *** **********  *       *   **

40               GAYMGSKYWRKTGAATAKGARRYCMKMRTTKYAKKRAMRYYMCCTYKRCWKRRGWAAKKC
46               RGCMKSKCWRKGRGGGGTRRGGYMCTCRCCKCGGTARARYYCYYYCKAYWGRRRWTGGKM
                   . * ** ***   .. ..    *  . *  *  . .   ***      *  * ** *:. *

40               AACAYKMCMTGTKKWYKRYRRKWAKKKGAKWCTCCTMGKYRRYSMYWRYKKTRYWGYRCY
46               RMYWCKMYMYRKKKACKRYRAKATKKKRMKAYKMYWCTKTGRYSMCWRYKKYGCATCATT
                      ** *  .**  **** * :***  *   .      *  **** *****

40               GGWYRCAAGKTRRMYRGCKYYYYGGGWYRRWSRRRSMRCCCKRYYYYATCRYYGYRTCCA
46               RKWCGATTRKWRRMYRRYKTYCYRKKWYRGWCRGACAAYAYGGYYYYMYYRYYRYGAYSR
                   *  .:: * *****  * * *   *** *.*  .    .    ****   *** * :  .

40               AWKYCRYRGCSTCCCMWMYWYYKSSMRMATTTKRRKKKRGYAGTKMMYYCKYYCGKGKRA
46               WWKYMRYRRYCCTTAMWMCWTCKSCMRCGCCKKGGGKKRKCGAAGMAYYYKTYTAKSTAW
                  *** ***   .    .*** *  **.**  .  .*    ***   ..: * ** * *  .*..

40               TRRYCMMGKKSMYCYMGYTWKRRWRAKKKAKRYKCGGYTRKRATMRGWKWCTYWGGAMRR
46               KRRCYMARKKSMYYYCRCKWTAAWGGGKKWKRCKTACYKAGRRCMRRTKWYGYWRSMARR
                 .**  *  ***** *    .*.  * . ** ** * . *.  *  **   **  ** .  **

40               MKWYYKWRGTGGRYACGTSYCKAYKYYCYRGSKACGAYYRKAAARCYWWCYKTKWRYKTK
46               MGACCKARTARARYWTSCGCMTTYTYYMYRRSKRYRRYTGKGGTRSYWAMCTGKTGCKCG
                 *     * * : .**   . .   .:*.** ** **     *  *..:*.**    . *   *
```

```
40      RGKYRGTTTRAGMTKKMTYRGWRRYKRWATCTGYMGWRMKAGAGKAGAAARSRRKATGMM
46      ARTCRRKYARRSCGKKMKTGKWGRYKRTCGYCSYMKWRCGRKRSGRCRWRGCGAKCCACC
          . * . :* .  ***.   * **** .   .** **     .       .  *. .

40      WCKGTTYMKCRRAACMATTGKWKKCKGTAYWKRMWYYMKACYTRKRRRKMRKAATGKWKK
46      TYKRWYTCKYRRRRTCRWWRKWKKSGKKRCAKRCAYYMKCTYKRGARATCRKRRYRGTKT
          *     * **        ****.  .   **  ****. *.*  * . **      *.

40      YYRRKAKCWKKCTTCTKGTCYKKRYWMKGACCTATTKYYYWRYYTAKKKYCCCKRKTGGC
46      CYRRKWTYWKKSWAAAKRYMCKTRCWMKAGATWMYYKYYYWRYYYTKKKYMMMKRKWSSY
         **** . ***. :.:*    *.* ***...     ******** :****   *** ..

40      YSYRYYYYTRYKKWCMGRKTTKYRKSKTKKWRYRKMKSKRKKYAKTKTKRYKYKWAKCKA
46      YSYRTYYYYGCKKAMMRGGKWKCAKGGYKKWRYRKMKSGAKKYWKYKGKGCGTKTRKSKW
        **** ***   **  *   . *  *.  **********  *** * * *    *  *.*

40      MMMYASTKRCAACWYRWYYRAMMRTRYRRRMKKCGGCTGRKMRCCCGGMMMTAYKMKYWK
46      MCMCRCYTGYWWSWYRTCYGWMMRYAYRRRMKKMRRYYRAKMGMYYSKCCMWRTGMKYTG
        * *  . .    .***  *  ***  *******       **    .   *    ***

40      MYMYRWKTTACGWRAGYYRKCACRY
46      AYMYRTTYCRYRWGRKYYRKYWTAY
         **** .     *   ****    *
```

The pairwise alignments of two accessions 40 and 47

```
40                  KYMYRRRKAKYMCGRRRRRYRWCCYRRMGWSCMMTMYMKRWRKRWYYGTTRAYWMGYYAK
47                  KCAYRGRGWKCATAGGRRATATYYYRRMKTSACMYMYMKRTGGRTYCSYWGRCTAKYYMT
                    *  ** *  *   .  **      ****  *. * *****   * * .        ** .

40                  MRTCTRYMTRKACRGYYYYMTCKWGGRTYGSKYYYKMYRWKYRWKYWYTGKYRRSYRYKT
47                  CRCACRYMKAGMTATYYCTMYYKWAARWCKSGCTYGMYRWKYAWKYWTYAKYRRSYRTKY
                     * . ***.      **  *  **..*   *   * ****** ****  .******* *

40                  RAYYMWRRTYKARRWAMYKYKRGYYKCRRRKKKWRGGYRKRAYKARMRRYYRTKCYWRYT
47                  RWTYMTRGYYKRRRAMCYTTGGAYYKMRRATKKTRAAYRGGRYTMRAAGTYRYGYYWRYC
                    *  ** *  ** **   *.   .*** ** .** *..**   *. *    **   ****

40                  RCARYYYRYWMRYMYYYRWKCYGCMGYYCAYGKWRRRRYYGCKYGSWYRKWMRWKKWAYY
47                  AYRRYYYGYWCACMYTTAAKMYASMKYYYRYRGARRRRTYRMGTKGTYRKWMRTKKAWCY
                       **** **   **    * *..* **  *   **** *     . ****** **   *

40                  RATACMYKAYKRKRGGTRRMMGWYYCSYTWKMSRRTCWCKRYMRYRWMRRRGYKSWGYKS
47                  RWKTTCYKRCGRGRAAYGGMMCATCACTWTKAGRACYWAKRYMRYRAMRRGAYKCARYKG
                    * .:  **   * *..   **    ..   * .*   *.******* *** .**.  **.

40                  MYTMRCYKYRSRGTRTGYRYGYTYRRYGWYWKRYYWWWTKTTCTMKAAKCGCGWYKRAWC
47                  MTWMGYYKYRCGRKGWRTACRTKYRRYAWYWKRCYWWWATCCMYMKGCKYRARATKRMWY
                    *  *  ****.  .        .****.***** ****:.    **..*  .   ** *

40                  ARRMYRRKGKGGAYMKKRCWKMKMKGCYYRRYTMGYCYMYRWWGRRYYYKYRTWYKRMMA
47                  RRRMYRRKKTAATYMKKRYWKAKMKTTYYRRYYCKCYTMYRWWRRGCCCTYRGWTGGMMR
                     ******* ...:***** ** ***  *****      ***** *    .** *   **

40                  GGKYWSRWYMYCYYRWCSRRRRRRCGWRKKYKMYYMYKYRYYCGCYRTTAKGRWYTRYAR
47                  RRTTWSGAYMYYTYRWYGGRGRAATAWRGKYKMYTMTKYRTYYRSCRGCGGAGTTGGYTG
                      . **  ***  *** . *   .** ***** * *** *  . *  . .     *:

40                  WTGGTCCRYTMTTYMYRMWRMRKRGSTMTAGCWYRRKAWAWARSMYACTYCRMAMTYKKT
47                  ACARCYMRYAMCYYCTAAAGAAKRACYCKRKMAYGRKRTWAWRSMYTTAYYRMGCGCKTK
                      .    **:*  *        **..  .    * **     ****: :* **.   *..

40                  ATRKYCTYTMYGKAYYTTKTCGYTYWRSYTGCTTMAAKCRAAAYYCRYTWKCWAWKAGTG
47                  WKGKTYYYYACKKGYYYWKCARCWYTRCYYKSYKMRMGYARGGYCYGCCWKTACWKCCCC
                     . *   *    *.**  * .   * *.*  . .*      ..*     **  .**.

40                  TAYWTRAKKYYRRTTMTRCTGSWGYKAYKRRRRKGYYYYAGMRKTYTTTMAGGTYKWKYR
47                  YWYAWGWKTCYAGWYCKGYKRSWRYKWYKRRRRGTYCCCGAAGKKYWKYMMAAKYGTGYR
                      *    *. *     .  . ** ** ******  *   ..  *.* . * ...*   **

40                  RGKRGKCAKYKRCRTTGRRRMGAMKAGAYCCAKSRYMRTTKKYCCGCCKYCCACTYYTGR
47                  RRKRRKMWKYKRSRKYKAGAMAGAGGATTYMMKGRYARCGGTTYMKTYKYYSWAGYYAAG
                    * ** *  ****.*.     *..  ..:    *.** *   .      ** . . **:.

40                  AGRRMRRKTYGTATTWWRRCATAKAACMWKTCACACCMWTAWCCMRYWSGYWRYWKCWSK
47                  MKRAAGRGYTKKRWYTWRRYGGWKRMMMWGYTWMWMSMWGWTATAGTWSAYAATTKSWSK
```

```
                  *   *    .    *** .  *   **       .**   .    **.*    *.***

40              RRRWATRTTYGRMMKTAGGWGYRYRWRYTCKYCMCRCRRRTTTTMMRKYYAAKYRWTYCW
47              GGRTCGRWWYCRAMKWWRRTAYGYRWRYGGKYYCYGMRAAWCGKACGGCCMWKTRWATGT
                  * . *  * * **     .* *****  **     *     .        * **:

40              RRMYRKCTYYKKAAATTGMWGAYCTATCGRWSKCRWKKGWTRAGKSGKGYKMYCACMRYM
47              ARMYRKTCYCGKCCCAATMARWCMCGAYRRWGKYGAKKRWCARAGCRTTTKACTGGMRTC
                 *****  *  *...:: *      .:  **.*   ** *   . . .  *   . **

40              AAGRKYMTYTGTYKTKGWWMGSYYKYYYRWWWYTMATAAACKMTTARKRTARYYYKAWRY
47              MRKRKYAWTYRCTKYKRTTAAGTCKTYYRTAACCAGAGTWAGCYWRAKRKGRTCCGRARY
                   ***       * *    ..  * ***      .:.: .      **..*      **

40              AYGKKGRKGGTWGMWKGYYKWAKRRCTKTYRGAYRKTRKAYYYCKKRYMKRRYRTGCYWK
47              RYRKKAAKAACARAAKKCYKTRKGRTCKCYATGTRKARKWCYCGKTRYMTRRYRGRYTTK
                 * **. *..     *  **  * *  * *  . **:**  *  *.***.****     *

40              AARCKTYYRAYKRYAAKYWKGYKWCGGRRYCWCGAGAMCTTWKSKKCYMAMCYTGAMRCC
47              RRRYKYYYRRTTATGRKCTKRTGTYRRAGCAWYRRRWCYKKWKSKTTYCRAYCYRWCGYY
                  * * ***  .  . *  *          .*       ..****. *

40              RAAGAAWMSCACKAATRTCTKAGAAKYAMRKKTYGAGGYAYRRKAWMKYRWYRCWAKGTK
47              RCGKCGWCCYTTKGTCRCSYKMAGGKCRAGKKKCSWKRYWYRRGGWMGCGWYRTWRKRCK
                *.. ..* . : *.: * . * ...*    **. .   * *** .**   *** * *  *

40              WKKGYGCGCKYWWRCAKGYMGCGYTMCKCRKKGGGTMYAYRAKWYYYGTYRTACRYKTAC
47              AKKACAYRMKTWWRMMKRYMKTTCKCMKSRKKATACACTCRMGACCTAYTGYWYRYKGWT
                 **. .   * ***  * **    .  *.***. .   : *      .      ***

40              KTRRRGTTWYGAKYYYTGKKKTGYCWMYRCYRSRRTCGCARKRKGTKTKKYCMYRYRRRG
47              KARRASKWTCACKYYYCCKKGCKCMTCYGMYRGGACMRYTGGAKACTAKKYMMYRYRRRR
                *:** ..   ..****  **       *  **.      :   *. .:*** *******

40              CMKYAYMRTRCKCKGKWYGYKYRGCTTYRKSMTTKTKCMYRYATRRYGRYKRAGGACGKG
47              YATYWYAAYAYKMKCTTYKYGCGRYWCCGGGCYYKWGYATRYRCGGCRRYKRWARMYAKA
                  .* *     * * . * *          .   *     **      **** .   .*.

40              AATGYGGCYCTTTTCTYYMKKTTCTCYRYCCKGMMTTRGAACAGRMSYKTYCGATKCKRW
47              MCAATAATYSWYYYYWCTMKKYYYYYYRTTAKKMAYWGRTGGRRAMSYKYTYKWWKYKRW
                 .:. .. *.        ***     **  .* *     :.    ****      * ***

40              KACMCATCYWYTKKRYYKRWCCAAAAKKGYKWKYTCMRYRTSACTAGGAWKMSTWCKKKW
47              KRYAYMKTCATCKKGTCKRWGYRGGWKKRTGWGYGYMGCGCCGTYTAAGWKMSKAGKKKT
                *     .     **   ***   .. **   * *  *    ..  :...**** .  ***

40              RYYCTYACGRMSWWKKSWRYTCCTCYKYYKACTGRKWRYAYACCTCCTAGTCGAGTTYYR
47              RYYTYCCATRCSWWTGSWRYAATCTYKYYKCYCTRKWRCWCTATCTTCCAWTARRAGCTA
                ***   .. * ***. ****:.   *****.   ****   :.     ..  .  :

40              WRKGMAYTKYRRTKCSTGYKGCGAYKCYRYGASWKTAGGAYKYRAAARGCWCYKKKWWRY
47              ARTAMWYCKYARCTYGYRYKAAARYKYYRYKMSWKCTTARYKTGGGTRRSAMCTKKWTGC
                 *..* * ** * . .  **... ** ***  *** : . **  ..:* .   .***
```

```
40          TKKKGAATTTTTRYACTMMKKMKKGRWMRKTKCWRYKKMYRYKRAARSRSRKAKKGTTYM
47          KKTGRCGAGWYYRCTYAMMKKMTGTGWMRKYKSWRYKGMYRCGRRWGCGSRKMKKRWYTC
            .*.  ..:    * : :*****.   **** *.**** ***  *   . *** **

40          CGRKKMRAATTGKCKGTYWACGRCTCYYMKYYRRACYRRCGRCSKRRGYRKMRAATGKWK
47          YRRKKMRRRWWRKSGKKCAMYRRYYYYYMKYYRRCTCRGTAAAGGARTYATCRRRYRGTK
              *****     *.  .     *   ********.  *  . ..  * * . *      *

40          KYTAAKGRKKAKYCKKWRGKACGGYCKYGARKRWSKCCGSYRGTWTRWYWKKSKCGTATC
47          TCCGCKARKKWGYYTKWGAKWAKKYYKCTGGKRTGTYGACCRACTKRTYAKKSKARYRWS
            .  ..*.***  * .** .*  . * *  . ** ..  .. *.  .* * ****.    .

40          KAWYRYRRWAYYRYKGACYMWYYCYRGKRTAGACCACYYKGSSCWGTYYYYCGRMYAWYY
47          GMATGYRRTGYYRYKRRYYMWYTYYRKKRYRKMMYRTYYKKGCGTRYCCTTTARMYTACT
                 *** .*****   ****  ** **        *** ..         .***:

40          ASYRRMKGRATRWTKKKYMWKYYCCWYTGKYYTAWRKAWKGKKKKACKWRWRKATGTCGS
47          MSYRRAKKARYAAYGKKYMWKYTYSTCCTTCCCCARKRWGRKKTKGTKTAWRKTCAKATS
             **** *        *******  .    .   . ** *  **.*. *  ***: ... *

40          WKGWACGGWRTACTGATARAAATTTARCKTWCTACTYRMTYRKKCKCCCWRCMYRRTGAT
47          WKRARYKKWRCCYYRGATACRRKWGCRYKYAYGGYCYRMCCRKKYKMYYWRMMYRAYTGW
            **      ** .   .:: .  .  .* *    .  ***  *** *   ** ***   .

40          RKRYKRYGAATTTTAKYYYCGTWTTKYMRRYKGSYWRYRMATMYYMYKGTRAKKTKMRGY
47          RKRYKAYACCGYYWMKYYYYAGTGAGCAAATKASYWRYGCRKMYYMYKRYRMKKCKMRKC
            ***** *...     ****  .   :      *.*****   .******  * ** ***

40          CCMMYYKKMSRYRRKCYMTRYKYATCCCRSKYYYGTTYKWGCCGMCKYGGGCKKKMYRYY
47          AACCYCKKMSRTGGKMCCYGTTYMYTMGAGKCYYKWCYKWAATAMYKYRRRMKGGCTGCY
            ..  * *****   *      .*      .* **   ***.. .* **    *      *

40          YRWAWAKWTWKYKRKRKCCYAYKYTGKSWGCGCCMRYTMMWKSMRKYKGYMYRKRYSAKR
47          YRWGWRKTAWKYKAKRKTTCGCKYYRGGWKYKYMAACKCMWKGCRKCKKCCYRKGTCGKR
            ***.* * :**** ***   . **   .*         . ***. ** *   ***  ..**

40          WKMMGGTYGAGMYRTRYYSKGTMKKRRAGKGRAATRRTGKYKYRRWYMWRYSYWYWMRKR
47          TKMMRKCTRGKMYRWRYYGGSWMKKGAGKGRGRMKGAWTKYKYRGWYCTRYSYWTAAATG
             ***     . *** ***. . ***  .      .    ***** **  *****    .

40          KYGGAWKKWYYMGKGACCKKYAAGCRWMTGAGTAGMARRWWWKMKGAAKWKTKMYKYTAT
47          GTRSRWKKWYYMRKSRMATKTCCCYRWMYRMAWRAMRRRWWWKCKKRMGAKAKMYKCWRK
               . ******* *.  ..* ..  ***   .  .* ****** *     *:****   .

40          CATTCKMTATAGKYTGYGKMSWTATTACRRAKRYRAAGTGMRRYMRYKRGAAMTTRKSKG
47          TGYWTGAAGWTKKYKRCCKMSWCMWKMYRRWGGCGGTRCKCGGTMGYKGTGGACGRKSKA
             .     :. : **.   ****   .  **     .:       * **  ..   ****.

40          GCKAMCCYRTGRYWGYCTCTGTGKAKKRWMWAYGKTYTCMRWKTYYSCACTTAARTCTCG
47          SYKRMYTYRARRCAKTAWTYRCTGCKKAWMTGTKKYYWYMRWGGYTSAGGAACGAYYYYK
            . * *  **: *    .       .** ** .  * *  ***  * *.. ::..
```

```
40        RMRMWKAYYRSRYRYYCWTCCCWYRKRCCGYYGYACGTKRGGAAAAAYMTKTCCCMMGGC
47        RMRATKGYTRSAYRYYYWCTATWYRKRTGAYYKCMMRYKGSKWRRGGYCYKYYTTCMRKY
          ***  *.* ** **** *  . *****  .**      * .    ..*  *     *

40        WRMMYRCCTKRAYRYYCGSRWRYAAAGCKARCTTYYKRYRMGATATRAKYAYRAAAKKRW
47        WGMACRYYKKATTRYYYRGATGYRRWRYGRRMCKYYKGYRMRMKRCGGGYRTARRRKKAW
          * *  *  .* : ***  .   *       *  .*** ***  .   . *      ** *

40        GMTSMKRYGKGKAWRWGRYYWAKRRTWYRCATGTCGWYRYYACATCAKWRYGCMRYYKYY
47        RMGGAKRYKKSGGTGTRRYYWRKRGCWTRYCCRATRTCGYTWYGCTGKTRCRMMRYYKCC
           * . *** *. .    **** **  * * .  :     *   .  .* *   *****

40        GYTAAAAWRYGRSGWYRRYYRMGAYYKCACTGAARAKAATCATKRRYTAGKCKACCAATT
47        RTGTCMTAGTKAGAACGGYYRCRRYYKTTTAKRMAMTMTKYMYTARCYRKKMKMYYGTKY
             :. :     ..    ***   *** : :     . :.   . *    * *   .:.

40        ATATRYGGTTCGMKRKAKRRYKKYKTAGTACGKTGYYSRYKAGTYWAYKRYRTCGKMMTT
47        RYMYRTKKCCTAMGAKGTAGYKKTTWRKWRYKGYAYYSRTGGTYCTTYKRYRYMTGMMYY
              *      .*  *..  *** .         .****  .    :*****    **

40        GARRCARRRRGKRATTATYKRTGCKCKAGAACRYATTAKMYYYSGARWRKKMGYCAYCWA
47        KTRRMRRGRGRKRGACGCYKRGRYKYGTRTCYAYWYCRKCTYYSRMRAGGTCSCMMYMTC
           :**  * *  **.: . ***   *  : :.  *    *  ***  *   . .   *  .

40        AGGKRRYMGGWRRRRGTTTRAKTTYRTGTYSCTYYKRMWAYKMGATAAGAMKCTRCRSYK
47        TKAKRGCCATWRRRGCYYCRRKCKTRCRKYSTWYTKGAWGTKMRMWMRKTCKYYATAGTT
          : .**   . ****     * * . * .**  * *  *. **      : *     . .

40        YWYTKYYRRGAGGCAAYKRWKWGWYYYSTWRRRRMACCMGCTTAGCKTKYKWYAWTYGAA
47        TATWKYYGRAGKAYTWCGGATTKWYTTCWWRRAAMWMTARYYGGCAGGKTGTCWTYYTTG
              *** *.. . :     .  **  . ***  *        . .  *       * :.

40        TCTYSRRGTGRSMRCKYYYATCTRTYYKRRYRCCAAKYRKSWMWTTKMYRYGSTSKCCRK
47        CACCCRGRWRACAATGYYYMYYAGYYYGRRYGYSRWKYRGSWMWGKKMCRYRSKCKTTGG
           .  .*     .    ***   :  ** ***  .  *** **** .** ** *..*

40        KRYMRTGKCGYYGATGYYRKRGGTRMCMSYKMYWRYMGYTRRWKKCKGKRMYKCGGTRRR
47        KRCMRCTGTKCYAGATYYRKAACKRMYMGCGMYWRCCRCKAAWKKMKRKRMCKTACKAGA
          ** **      *..: **** . .** *.  ****    .  *** * *** * . .

40        KMRAWMRGTGWKTYWYKWYYKRCGCGACYYRRSWWKWAKKRKKRACTARRTCKCTGGYCW
47        GARRWMRTGRTKGYWTGACCKRTAYKRYYYRRCWAKWGKKGKKRMSKRRGCAKMYRTYYA
            * ***    * **     ** .    ****.* **.** *** .. *  .*    *

40        GRMTMYATCKAATYMTGGAMCKGKKCAGCCTGTRYYYKCCMGGTMYCCMMKCCGKRMRGK
47        KRMCCTTKMKTGGTMKRRWMYTAGKTCRTGARYRYCTKYYCKRCCYYYMMKGSKKRMRAT
           **   :. *:.  *.   * .. * .   :  **  *       *  *** . ****..

40        WWTCARGTTTAYCTTGATKCGGWMRRKKMYSKTKCMKCTAGAKYAACRWMRMKTKCAKTS
47        TWCTWRRWWKTTGCGTCYKYRKWMRRKKCCSKGKYMKMYRRMKTTGTRWMGMGAKTTGCG
           *   *  .:      . *   ******  ** * **     * :. *** * :* :  .

40        RTYYYYAKKCRCWKKRYYGWWYAKKGWYCKWYCYRATCTCYKSYYKSGRRSSSMKAMMYR
```

```
47          ACYTTCCGTYGYWKTRYCRATCRKGKWYSGWYYYRWYYKYCTSYCGSKRGGGGCGRMMYR
              *   . .   **.**      *  **. ** **   .  .**  * * ...   ****

40          RTTYGTYYWATCTTTAGTTGTRYRRYGAYMKYCATACGYRRRRMYAAMRMTARGAKKMYC
47          GKYYKYYYACYYCAGGACCACACAACRRYMKYYMCGMSTAAAGAYGGMRMYRAKWKKAYT
             . *  ** .   : ..  .        ****   . .      *..***     ** *

40          AKKWYARRWRYYTRTMAGYKMMYKCAAYYKKRKYRYGSWTTYKMRGGKYSYAAATGRCRA
47          GGKWYTGGWRYYYRYMRACKMMYGTCMYTKKRKTGYSGTKCYKMRRKKTGTRWRYSRYGT
            . ***:  **** * * . ****  . * ****  *.. . ****  * .     .*  :

40          YRYWTTCRWMYYYGYCTKKRYKMCYYCYTKKGYYRWMCKWKRRGWKGGKYYYKYYCMWWK
47          CGYWAGGRWMYYYRCYAGGGCKCACYAYYTKAYYRWMMKTTAARWGRSKCCTGCTYMTTT
              **:  ******   :    * . *.* .*.***** * .   *  .*       *  .

40          SRKCRRKAYTKCTGGKRKGGRMATRYRRAYCCKCKKRTRAYRYYRWCTTYMCCCWRRGCA
47          CRKMRRGRYYKYWRRKGKAKRMCWGYGGRYYMKAKKGCRMYACCGTTYCTAYATWRARMR
            .** **  * *    * *. **.  *   *  *.**  * *           .**

40          KKMWYKRTAKKYGMRYGGTCYRYKKKKKAWTACGARKWKAARTYGCCCTTTWMRSRRKKW
47          KTMWYKGWMKKYRAGTRRKMYRYTGKTTMWYRYKRRGTKCGRACRYAYYAYTARSRRTKA
            *.****   ***      . ***. *.. *     *  *..*:   .  :   ****.*

40          SRAYWGWYKRTGGYCCTASWGYRMRRWKMWYMKAAGGACCYGCKKMAKYKGGCSRRATKG
47          GGWYWKTYKRWKRCTACGSAKCAMRAWTATTMKTGTAGTYYTSKKMMKYKAAASRRRYGA
            .  **  ***     . .*    ** *.   **:. ..  * .*** ***...***   .

40          TGYRYKWTYRRCRSYARYRAATWATAKWRKRGACAYGKTGTCCYKMYKGKCATTGYYRRW
47          CACGCKTYYRAARSYRRYARCAWTAGKWRKGRWSWYAKCAAMAYKMCKAKYGCYCCYARW
             .   *  ** .*** **  .:*::.****   . *.* .: .*** *.* .    * **

40          KTKTKTCKRRMTKMGGKGGMMKYYKKCGGMYRYAKWRTCTWRWYYYARWKKYTTTGAARS
47          KGKATCYKRRMYKCARKRRMMKYCKKTTACCATCKWRKMAWRTTYTRAAKKCKWWRWRRS
            * *:.  **** * . *  **** **  .    .***. :**  *    ** .     **

40          YWRTWKMRAACTMTAYTMAMYTAGMKYWGCGCKKYWWSTTYWYKKRCKTKKTAAGTTRGC
47          YWRCTGCRGGTACWRCCCWCCATTCKYWRMSMKKTWWSKYTATKKAYKYKKKCGACYRAT
            ***    *.. :         ::  ***  . ** ***.    **  * **....  *.

40          YRRYMYKGWYMYRRKYACGAAYCYMGYGKGGYYWRYKAKTRRWMATKAKTSAMCCTYRCW
47          YAGTMYKCTCACARTYWMRWRYTCMRCKKRRCCWRYKGTCGGACWKKGGKSRAYYYCAYW
            *   ***      *.*     *  *   *    ****..      .*. .*        *

40          KKMGAGGGCCACYCAYGSKMCMTTYKWYRYRKWAKGARKWKYRRGYSMYWRYKKWGTKCT
47          GKMRWKKAYMGYCYWCTCKMYMWWYKACRYAKATKKRGKATTGRKYSMYWRYKKWTCKSW
             **    .  .      .** *  **  ** * :*   * .  * **********  *.

40          KKYYKRYKWAKGACYYYTTGYCKKKCKKCKRKKKRSYYYYYTYAAGGKWKWWRAYWCMCK
47          GKYYKRCKWRKKGAYYYYKTTGKTKMKKMKRKKKRSYTYYYYCWGTTKAKAARCYWMMYK
             ***** ** * ..*** .   *.* ** ******** ***   .  * *  *.** * *

40          RWGRTMYCTTKTKGYRKSTKKWRYRKGTCTCKSKTARKKACAKRTARYKMACTWAKKWGT
47          RWRGCMCYWATGKSCAKGYKKWRYRKKYMWYKSGWWAKKWMRKGGTGCKARYGTRKKTSG
```

```
        **   *   :. *.  *. *******     **    **    *  :  *      ** .

40      AMMMYRCASKTGATCCTKRCTCAACWYWAMMMRYRMKKYKCTGYGRKMRMGMMTATKWKK
47      WCMCTAYRCKCAGWYTYTGMKYWWSWYTWMMMAYRMGKYKYYRYKAKMGCRCMWRYKTGK
          *     .* ..    .  .   .**  *** *** ***   *  **    *   *  *

40      MYRKACGWAAGRWYRAYGMAGAYCRCYRCAGKRAAGWRTATAGCGCGKGYCAWWYYGGRK
47      MYRKRYRWRWARWYGCCTCRKWCYGYCRYWRKRMMRTRWTCCAAAMAKKYYRTATTKKRK
        ****   *  .*** .           *   **    * : .... .* *        **

40      AKAKTSKRRTWWKGYGTRGSYYCWKGTRWGYRGGKKATCYMTTKACCYKYCTCSKYYTAK
47      GKGKKSKGRATWKRYRCGRGCCYTKKGRTRCGAKKKWGAYMCCKGTTTKYYCTGGYYGGG
        .*.*.** *: ** *    .    *  *    . **  .**  *.   **   . ** .

40      KYRRYSRRYKKSTCRCCCTSRRYTCCTTKWKYKTGGCAGWAWRYRGAYKCTKYRKSYKRW
47      GTGACGAACKKSYYGATTYGAATYMYCWKWGYKACTTRSAMAATARTTKAAKCAGSCTAW
             .   ***   .   .        ** **:    .       : *.:*   * . *

40      TTCTYYYRYMTATYKYKRYRRAAGMKKWKTCAKKRCTGAWYYGTYCCGTKTYYKRTRCAA
47      YWYWCTYRYMCWYYKYGAYRRRWRMTTWGGAGKTGMCACTCTKYYAMAWGCTCKRYATRM
              ****   ***  ***   *..*  ..*.   ..     *. .     **

40      KKCCTTTGATTCKYYRKRGCACAGGCTGMYYYYYYACKRGYYSYARTCYCRYGYKWCTCG
47      KKTAGWWKCCCTKCCRGARYRMMKSYCAMYYYYYYMTTRRYYSYMRCTTYRTRYTAAYYA
        ** .    .   *  *        .  .*******  .* **** *    *  *. .  .

40      TCRKRATYYRYYCMRWYAAKRYRYGYSWWRRYTAYYYWMYYYGAMMAKYMMYTTAAKWSK
47      WSGKARGCYRYYAMRWYTGKRYRYTCSWARRTYMTYYWMYYCRGCCRKYMMTKCGTTWCK
         . *    ****.****:.*****  ** **    ******  .   **** . .:.*.*

40      SRMMYMYTKWMRRRGGTRYYSGYYCCATMKATGRYKYMRYMMYMRRRYMGAMCYRYRYKR
47      SRCATACKTTMRRGTTCRYTCKTTTTCCMGRCAAYKYARYMMCMGGGTCKRAMYRYRYKR
        **     .. ***    ** .     . *   . *** **** *         *******

40      CACTTARGTGYCCAGATWATGTTCTTGYCGKKYYYRMARRYYKCYSYRRYGMKRRKYKRY
47      MMYYYRGSYRCYYTAGGWCATYWMKYSCYKKTTYYGMMGRYYGTCSCGGCKMKRRKYTGY
               .     :.. *.:    . .   *.  ** * ***   *     ******. *

40      AKKWYMYYMKWRKRRRMRRKGRGCGAKMMAKTWGAARTATYYRATCMKKKKGKCGWGKCC
47      MKKTTATCCKWRKRRGMAGKKRRYRRGAAGGCTKRWRCTATYRGCTCKKKKRTTRWRKYT
         **      ****** *  * *       .      * :: **.   **** .  * *

40      TWCRGTTCSYGTTCKGAAKRSKYCWKAGTGSGMRKTAYRSRCYKYSYWYKKSCWMKMCTA
47      CTYGCAATSYRWWYKKMMTRSKYTWKMRYRSRMRKYMYRCATYKYSYWYKKSTWMKCMAR
             :: **    *   .**** **    * ***  **.  ********** ***  :

40      YMYYKYAKRKYGAKGGCGAGKGGRKGWRYCAGGKRAMKWYMCTTGTRYACAATCACTG
47      YCYYGCGKAKYKMKKRYKCATAKRGKTGCARRKKAMAKTCAMYWCWRYRGTGCMMYKR
        * **  .* **  *    .... *     .   *   *        **  :.    .
```

The pairwise alignments of two accessions 40 and 48

```
40          CYRYMRWRYMYYKCRYRRMWRWACCMMYMKWRKGRYGTYYKYRGYSWKKKGKYRWMRWGK
48          AYGCARWRCATCTYGYRRMWRARYACMYMKTGGKRYAWYTGTGKTGTKKKKKYRAAGTAK
            .*   ***    .  ******   . ****    **. *      . *** ***    .*

40          AYRMMTKAYKWRYKRYATKYYRGRYTAKSRWCAACWRACCCSWWYRYYWGSRYKYMRYGC
48          WYRMCGKRCTTATGRYRYGYYRKRCATTGRAMRRAWAGATTSWWYRYCWKSACKYMRYCT
             ***  *  .    **   *** * ::..*    .* ..  ****** * *  *****

40          YMYRYYYWYKAARRRRRKTGRRKCMKKCMRYMYSRKMRYMKARMGCKRGSRRGAATRGYY
48          YMYRTTYWYKGCGRGGRKAAAAKYMKKACGCAYSRTCRYMGRAMKATATSRRRGGGAACT
            ****  ****.. *  **:.  * ***.    ***. ***   * ..  *** ..  .

40          WWRMRTYWCGTAKCYAYMWRYWGSRKARRAMKYKRYKRRWRKYYYCCMYRYMWMKKAGGY
48          WWGMRWCAYKWGGYCTYMWRYWRSAKRGRMCKYKRYKRRTATYCYYYMYGTMWMKGRRRT
            ** **      .   :****** * *  *  ********  .* *  **  ****

40          YYGYKRKTKTCTYYCTCWSWYRCYWMKTRRARYKWYRMRRWRYRYYRRKMYGYCYKWYTT
48          YYRYKGGAKCGAYYTAAASWYGYYWCGKGRMRTKWYRMRRTRYRYYRRKCCRTYTTWYYY
            ** **  :*  :** :. ***  **  . * * ******* ********      .**

40          AKTRYCARSKKKRMRKTTSWYYYYRYMTTRRYRGRKMYKMMYKSRTGYRYYATTRTRRYW
48          RKWGYSCRSKTTRARKKGSWCYYYRTCGYGAYRRRKMTKMAYKCGWRTACTGCAAARRYW
             *  *..***..* **. ** ****      ** *** ** **.       . : :****

40          GCRRCYYSYWWKYRMTYYAGAGCKWATTTSYTCRYYKYGYWYWRWCGSKYRRYYMKKMCW
48          TTRAYCCSYWWTYRMKYYRAWKTGARWWGSYAGGCYKYAYWYWRAMKCKYAGTYMKKMYW
              *    ****.***.** .         **:   ***.*****   .**   ***** *

40          KYGGWMRGKYRYMAYWKYRWYMMYRCYKRKRGTGTGRTGGCMCAAYKMGRKYYWRWYGCA
48          KTKRWMRTTYAYMTYATTGWYMMYRYTGGGGRYACCGWKRSMSTGCGMAGKTCWGACAYR
            *   *** .* **:* .  ******        .      .*.:.  *. *  *   .

40          TCGRKWYMYRMWRAGKTCYCMKMYTWATTWGTCYWAACYCKRGKATYMKCGCYWRYRMRA
48          CTKRKWYCTAAWRTAKWYCMCKCYGAGGCTTATTAWMSYTTGRGGGYMKTAACWRTGMRW
               ****    **:.*     * *  .    :     .* .   . *** .. **  **

40          TWCYTTTGTTGAKRRMRRYYKTTKYMYWARYYYRGTGCTCTMARAATYYYRTRCAMYCYK
48          KWMTYCCTYWRWTARAGRYYKYWKCACWWRYYYGTYKSYTKMRGGGCYYCGCRTCCYYYK
            .*          . *  ****  *   * ****    .  .*  .. **   * . * **

40          KKTWYRYAGTAGGAMTRRYKYKKAGRCGAAWGCYYKRRAATRRYRCYACAACTKRYGMAY
48          KGWTCGCCCYWCAWCAGACTCKKTRGYRTGWRYYYKRRRWWRRTATCGTRGAAKRYKMRT
            *      .    .  :   . **:    :.*  *****   **    .  ..:*** *

40          RYRYYYKSKYRAGYRKMKRRMTCKCRRAMKAMGKRWYRRGCAGKCARMRTKKKTKRAYYG
48          RCATCYKSTCAGAYRKMKRRACGKGRRWCGGCAGRTTAGRYMRKMWAMRKKKKYKGWYYR
            *    ***.  ..*******   * **   . . *        *   **.*** *  **

40          GCKYCWCCCTYAKSCARYRGAWSKRCGSYGATATWTRYKTCGKSATAGKAWYTRYRMRYT
48          KYKCYWSAMCYGGCMRAYGKCTGTGGACCARCGYTKRYTKMKKSGGRRGMATKGYRCRTW
```

```
                       *  *..  *. .   *  . ..  .. .  .  .**..  **.      . ** *

40                    GCKACYMWYKYGYRGKRAGKAGAYAYYAKGYSTCRASWGWYYYYGYRMYSYTCGTGMWWG
48                    SYKRYYMWYKTRYRKKRGTKMTGTRYYTKKYGATGTGTRTCCTTKYRMYSYGTACAAWWA
                      . *  *****  ** **. *   .  **:* *.:  :.        ******  . . **.

40                    TCCATGGRGYKCAARKCCWKRGRKAWKGCKKCWWATTGSWKGWAKTCTTGAGTCYWCTGA
48                    ATTTYKKAKCKAGGRKYSTKRTRKRWGRYGGYTWTCKASWGRARKWYYCACRWMYWYYRC
                      :  :      *...** . ** ** *        *: ..**    *     ..   **   .

40                    MAATTKCGCTACKTACMMWWYAMKCRGAKKCCWRCMYRGACGRKWGKYTAYTCCGTGGGA
48                    MRRKWKMSYKMYKYCMCCWWYRMKTGRCGKYYWRMMYRTGMKGGWRKYWMYCTYAYSRRW
                      *   . * . .  * .   *** **   . *  ** *** .     * **  *   . .

40                    RKYRWRWYGSWRYTKRCTWAMMMYKTRMAKRMAMGYCYRYKYKMSRYRKCCYMRYKCCCS
48                    RKCRARWCASWRYKKGTGTGCMMYKCRCCKRMRAKCMYACKYTMSRTGKYMCMRTTTAGG
                      ** * **  .****.*    . **** * .***      *  **.***  *   ** . . .

40                    KYYYRYYGTTKWKCAYGGGCRCCKMYRYYYRWKYAGTTTWKKRWYYCTGKRRYGAYAYTG
48                    KCYCGYYKWCKAKYMYRRRYAMYGCTRCYYRWKYCKAGCWKKATYYSWRKRRCRGCGYYR
                      * *  **   * *  *          * ******. :  ***  **.  ***   . .*

40                    SWGKYCGGCKRYYRMWRTMGCYMCYKKYMKRYSRWGGKWGTAKKRSKGTRKCRTAGKRRG
48                    GWKKYYKRYKACYRMWRKCRTCCTCKKCCKGTCGTRKKWACGGKRGKSWRGYGWGRKGRT
                      .* **    *  *****.       **  *   .     **. . **.*. *     . * *

40                    KKRWKMKGWYWRKRKAWKKYYMTKTCCCMCCTAYKKKWYRRYYTKKRYMATTTYYRYRAR
48                    KKRWKCKKWTAATRGMWKKCTCAKGAYYAMAATTTKKWTRRYCYKKRCMCAKCYYGYGRR
                      ***** * *   .*  ***   :* .     .:: .*** ***   *** *.:. ** *  *

40                    YYCCYWRRKKAACKRYRTAKRTGRYCTYRKTSTKTWCRRRYRGCKKWGWYKRGGRRKYGG
48                    YYYACAGGKKCCATGCGKMKRCRRYTACGKWGCKYWTAARTRRMKKAKWYKGKKARKYKR
                      **  .    **....   . **  ** :  * . * *    * *  **  ***     ***

40                    KGTCRYKKCCTTSAKWRTRCGRAGRYKWKATTGGCTCTATWKYMCTTTRSRRKKKKWSRA
48                    KRKMRYTKYYYWGGTWGYRYKRGARYTTKCACAAGYYYTYTKCAMYYYRSRRTKKKAGGW
                      * . **.*    ...*  *  *..**. *.: ..    :  *      ****.*** .

40                    GWSYKRGCYSMWYRMRRWKWYKAAGGACKCGYRTGRRMWRAAGTRYKARKARGGATGRRR
48                    KTSYKRRYYSMACAMRAWTTTGTGTAGTKAATGYTRGATAWRRAGYKTGGTAATRCARRG
                        ****  ***   ** *.   :. .. *..     *        : **:  : .   .**

40                    TMYWTCKRYYYRYRWTWKRGKKYKYRMWKYRYRSYSKYKWWWGCRMARYYWGRWGWRYGCT
48                    GMYTYAGRYYRYRWKWKGRKKYKYRMWKCRCRSYSKTKTATCTGAGRCCTARTRWRYAYA
                       **   . *******.**  ********* * ***** *       .*   .*  ***. :

40                    CCTKYCGTGYYTGRYRKGTGRTKGKKWCCRYACYGTCGYYYYKTCTCAACAGYWMTTKWY
48                    ATCKYMTGAYYCARYRKRARRYGRKKWGTRCTMYAWTACYCYKYTAYRTYGACWCKYKWT
                      .   **    .** .**** : *    ***   * : *.   . * **   :  : .. * . **

40                    CAYKSTRWGMAACYMKKKYWYCCCGGYCCGYYCKCTRGYSKMYKATGCWYYATCCRSRAA
48                    YMYKGYRWKCWTTYAKKKYTCTGYRKYTTACYYKYWRAYGGAYKRWKYTYYGYTYAGGRM
                        **. **    : * ****        *   . * *   *.*.  **      **.    .
```

```
40          MAACGRCYTMWTKYYTYCMWGMKKWSWCRWRYWGGRTWGCKGRTCTTTYRGTYMWTMWRY
48          ATGAAAATCMWYKTYYCSMWACGKWSWYRAATTAAACATTTAGGMGCCCRRGYMTKCTRT
             :... .  ** * *  .**.  **** *    ..     ..       *  ** .  *

40          RATCATCKYRWRYAKYYRKRRKWKMWTRKKGAYRKTWYKRRMRYSRRTGYYRRYRTRYWA
48          GMGYGYMTTGWGTGKCYGKGRKWKCACAKKTGTAKWWYKRRCRYSRRYRYYRRYRKGTTR
                .  .  *  .* * * ****    ** .  * ***** *****  ******.

40          ARCKYWRAYKRYAACRTYWYKWRYGTATACGRRYGYWKTYGRAAKCWYMRGRTKKCKYCY
48          RRYKYWRGTTATGGARKCWYGTACRYRKGTRAGCATWKWTRGWWKYWTARSGWKTYKTYC
             * ****. .  ...*. **       ..     . **      * *  *.  *. *

40          TCWGTTGAMYYGRKSAAGAAWMSCRCGRKCYRARYCGMRYKTKAGGAAGCKGRYRCWART
48          GGTAYYRWCYYSGKSCGRCGWCCYGAKRKTTGMACSKAGYKKTWKRWGRSGKGYATWRGC
               .     **. **.. ..* .  . **      .   **..    . .   *  *

40          TWMKARTCWCAKKARCKKGGGTMRKKAAWYYRAKACWWRYTARRRRAYGACWYWKTGKGK
48          YAMKRRYMWMMKKRRSKKATACCRKKTMACYRTTWYTAAYGWRAGAWCACYWYWKWRKKK
              ** *  *  ** *.**. .  ***:   **:.     *  *     .. ****  * *

40          GRTGYYRRGWKYWKTTYYYYTCACKAAYCKRRCKKCCTKRRSYYKYYYTRYKRKWKWWRK
48          RAWRYYGGRAKCWKYYCYYYYTGYGGGYMKGRMKKTAAKRRSYTKYYYYGCKGKAKAARK
                **    * **   ***  .  ..* * * ** .:***** ****   * * *  **

40          MWAGMYWCMTKRWGRGGTMSGAKKKTWRYRKGKSWRACCAGCGCCKTRTAKRYKWAYKKM
48          ATTCMYWMMYKRWRGAACMSKRTKKYWRYRKKKSWAWMAGTTATMKYGGTKGCKTGYKKC
              : *** * ***  .. **  .** ***** ***   ..  .  *   :*  * .***

40          CMMYRRASTWYRWKAMMARRGKKWYKGGCTYGYRKMRYRYMMMTACYYKMYWKMMCGWMR
48          YMMTAGRCYAYRTKWMMWARKKKWYKRRTCYRYAKMGYRYCCMWRYYTGAYTGAMYRWMG
             **    .  ** * **  * *****    * * ** ***  *   *   *   *  **

40          WYRRAGRYYYCAKRCWKRYGYTTSRRTARTRRMGKTYMGGYTAYRGTTAYKAGCRCTKYY
48          WCGARKAYYYYWKRMTKGYRYYYSRRYWGYRRMRGKYAKTYKGCATYCGTKGRTRTCKCY
            *      ***  **  * * *  ***    ***  .*   *..     . *.  *  * *

40          WYWGKGYYCGCWRSTMYTCRGAAAAARMCWSAYAAKAKKRYWRMGRWCGGCRYTRGKTAK
48          ATTRKAYYTRTWGSYCTCTATTGTMMRAAWSMYCTKRGKRCWRATRWYKSMAYCRCKGGK
                *.**   * *       :.:  * .** *.:*  ** **  **  .  * * * .*

40          RTGGRWTATTGAYASCTTARGWMACTAATTGGACTARYMSKMCGARSKGGTYKRTTYAKK
48          ACACRWKGYCKCYGCAKKGRKWMGTWMRCWRRRTAGRYCSKCTATGCKAAATKGGYYRKK
              . **..   .*......* **.          :.** **  .: .*..: *   * **

40          KCTAGTTARKWKCWGKRATKRCTGRTAATCCKAGTYSMMWGYYWYKGACKMCGKGMKCAC
48          KSKGACCWRKWTYARKRRYGRMKKRCTTCYMKTRGTGMCTAYYWYKRWYKMYAKTMKYCT
            *....   ***.   **   * . * ::   *:   .*  .*****   ** .* ** .

40          RMWGTYGYKCACYGGCTMGGTTACCCGTTGYGGYKRAGKCWKRAYRGKWTCAMRYGTTTK
48          RCWRYCKYKYCTTRKYWCKRCCCYYYRCGRCRRCGACAKGWKRRTRATTCTTCRYRWWKK
            * *    ** .           .             ..* ***  *..   : **   .*
```

```
40    KATASSCGGRMYSMKTTTKMCCTYAKYKRWMRMKKKCAKTSRYYYMTYTAYTYYWKKARY
48    KRYGSCYRKACCSAKGCGGMYMYCMKTKRWMGCGKKTTGCGAYTTCWYWWCYYYWKTMRY
      *  .*.      * *    *     * ****   ** :  . *    *    ****. **

40    YYAWYTGAKKKKKKACKKCRTKWKCKGSGTAAAGCCTCTGYRCARKGGTCYRKKCCYRAK
48    YCMTCWRRKKKGKGRYKGYAYGWKAGACAKCWGATGATCTYRTCGKAAGGCGGKYYYRTG
      *       *** *   *     **. ..... ..  :   ** . *..     *  **:

40    RMYKGWYYRRCKTYAYYMYKCGYKCGYGACTGAGRCKKGGWYYWRYRRYKMKARAAKRSA
48    GMYKCTCCARYTKYWYYMYKYRYKTRCKRYYKGTATKKRRWCYWRYGGYKCKWRGMTASR
       ***     * ..* *****  **        .   **  * ****  ** * *. . *

40    MRYARKCCTKRCWKWRAAGCGTKYACTYKYKMMCKAGWYCKSRKWKAGAAKYKGGKYWKK
48    ARYGGGYYYKAYWGWRTGAYAAKCRYKCKYGCCMKRRACTKSAKAKRRMRKTKRKKTAKK
       **.     *  * **:.. .:*   . **    *     ** * *    * *  *  **

40    YRRYSWRKKKCMAWWWKMKTCKYYMAYCGYMRRTTCKGMCATCTTTTCTGWGAYTTGGTM
48    TGRYSWRGKKAMRWWWKCKYMKYYCTCAACMRRWCTKKAATATGAAAAYKWTCCCKRACM
        ***** **.* **** *  *** : .. ***   *  .::  :::.  * .  . . *

40    MSWTRARTYRRRRGGARKYRGTACCCCGRMCGYKKCRKSKCRWAMRYRGYRYKWTMMMYM
48    ASWCATGCTRRRRTAGGGTGRYTAATTTRMYKTKKTRKSKGRARMGYRKCRYKWACMMYM
       **  :   **** ..      :..   **   ** **** *  * **  ****: ****

40    CTGACCKKMYMGCATKYYMRAGYTTCAAATCMRWYAAAGKARASGGCRCTKCRKWMKRTM
48    TYRRSYGTCCCRTGCKCYMRRAYKWYCWGCAMRWTRWRRKGRTCKKMAYYKYATACKRAM
          .  .     . * *** .*.  . . .***     *.*:.      *  .  **:*

40    AAMMWGAYRCCGYYKRKGACGGTRKAARAYCKAAAMKYYMYYMMTKTCATCTMKGGCWYK
48    GTMCTRMTRTGAYYKGTKCARRYRKWRGGYYTRGGCKYCCYYMMYKYYTCYYMKRKYWYK
      .:*     *  .*** . ..   **   .* . .. **  **** *  :   **   ***

40    CMYYRYYCGSRWRYCRTYYCCRYAWRKMYAYRTTAKRWKGTSMRRTRKRSYRTGGTGTAG
48    YCYYRYYYRGATGYTRKYTYMRYWAGGMYRTAYARKAWKAGGAAGGAKRSYRWRRARAGS
        *****  .   * *.*   **    **    : * **. .     *****   : :..

40    MKRYWYTAAYRCKATKGCGYRYGGGCKYYGACTAYKGMRYYGRGKKYYCAWMTWRRSYYR
48    AGRYWTYRWTRYKCCKAGRCGTKRKYKYCKGYGWCGRMRYYAATKTCCMWTCWAGAGTYR
        ***     * *. *.         **  .      ****.  *.          . **

40    KRMKAAKYMGRYKWSTAGKKCTWATTCTAKRWRKKRYKCCYGKKRMKGTCGGTTRMKTCT
48    GRMKMTTCMRRYKWSYRKKGMAWRGKYYRKGWRGKRTTMTCSKGAAKSWMATCCGAKATC
       *** :. * *****   *  :*  .   * ** ** .   .*   *.  .     *:

40    RCAATAKKTKTAGSCCRCYYSRKAGMYYYYCKTMWGYYSRRARATYKACRGTCTTGGKAG
48    GATRYRKKGTWRSSYMRYYYSRKGTMYYYYYKWMWRYYSRRMRGCTKCSAAKYKGCTKRR
       .:   ** .  .*  * *****. ***** * ** ***** *.  *.. .. .   *

40    ARYYAGCYAYRYTYTGMRRWYYCTYCWYSYYGAMATAWYYMMTTAAGCTKWGCTAACRTR
48    RAYYGAMYGYRYACWTARRWTYMKYTTTSYCRGCTGRATYMMKCGTKMYTTKMCCGTRYR
        **.. *.***:    *** * .*   **  . :    ***. .:   .    .. * *

40    YGTYGYRTGMKCRYKYRYSMRCWMYMGARGACAKKRRYKKKAGTCACTTARGTGGYCCWT
```

```
48          YRYYRTGKKMGAAYKYRYSMRSWMCMKRRRWYCTKRRYKKKWRYMMYYYRGSYARCTYWC
            *  *   . * . ********.** *  *   ..*******          . .    *

40          TCAGGYGAWRKYRTKSMYKKYRYASKKTAGGARYAYRCRWCYKKWRYKGKKKRYRMMGKA
48          YTTRAYSMAGKCGCKGMYTKYRYMSKKCTTARATMYGYRAACKTTGCGKKKTRCAMCKKG
              : .*.   *   *.**.**** *** : .    *  * . *.     **.*  *  *.

40          MMRACTTGTWYMCRYWRKWMRKYKRSRSRAACKAMMMYKAGTTYKTCKAACMATTKCKGT
48          MMGTYGYAAWYMSGCWRKWCRGCKGCGSRRRYGMCCCTKTRWYTGYYKRGYCRWWKSGKK
            ** :   .:***.  **** *  * . **         *:       * .     *.  .

40          AYGCWWYKCYCGGCYTRKKCYRKRRKMRKKYMRKACCTCRSYKYWYAYYTTCCGRGRYYW
48          GCRYAWYKYCARATCWRKKYYRGARTCRGTCMRKWYYYTAGTTTATRYYAATYAACRYCW
            .    ***  . .   *** **  *. * . ***      . .    **::  .  ** *

40          YRYCCCRRWGKWKRRRRAYRWMACCGCKYKKWWYGTTWGGGWYRSMTRGTRSMRARYKRK
48          YRCTATGGAAGWKRRAAGTGAMWMTRYKTTGTTYACKAATTWYRCMWGRWACAACRYGGT
            **  .    . ****  .   *     * .   *. . .  ***.*     .  .**  .

40          YYYYKGRYRWRRKAKYGKYRTCCCGMYCRWCGMTYCSSKRKKRGYMTYGCGRKYSKKKRG
48          CYYYGRRYGWRRKWKYKKYRYYYMRMCSRWYAACYMSCKGGKRKCMYTSYKRKYCGKTAA
             ***  ** **** ** ***     * .** .  * *.*  **  *  .  ***. *. .

40          GRTRYMCMSYGKMCYGTARMGYGAKWKAYTTKKKKKKRYCGGTRRWRKMRAMRWACACTY
48          CRKRCMYMGCRKMYYRGGRCRCRMKTKMCAGGKKKKKRCTACKARWRGMRRMRTRYWYGY
             *.* * *.  ** *  .*     * *  :  ******  . . *** ** **      *

40          WGGAMCCYCKACAKMTTCWWGKMRMYTYCMYCTAGKKKGKWRRMYRTKCRAYTTKCCCTC
48          WRSMAYYTMGGAMKMKGGWARGMRMCWTTATGYMSGTTAKTARMYRCKYAMCGKGYMMGT
            * .       .. **.  *   ***         . ...*  **** *     .

40          MKGAKARCKSTGRRSWTKTGTYGKKMMTRWGWGTTGMYYYGKGCKGRMCGYYCKACKGTK
48          MKATKCAMKGCRRGSAYKYRYYAGKACWAAKWRYWRMTCCRTRTKKRMTACYTGGGKRGG
            **.:*.  *.  * *  *   *. *      *    *    .  * ** . *  . *

40          YATYRAGRKTCYYTKGTYRYWKMRRTYYKRYYRWWTWYWTTRRYCYAAATGAKYYYRTMA
48          CMYTGGTRKYYTTCKACTGTTGARRYTTKRYYRTACACWCGRACATGGTCSGKYTCRKAR
                 . **     *.       **   *****     *  *  . ..: ..**  *.

40          CAWRYGCAWKGACAARYATWTTSSCYCYKRKMCKYKKTYAMRRWGACRYYKRYGMKRRKK
48          YRARYKYRWKACATGGTCGWGCGGYTGYTGKASKYKKCCGMGGTRRYRYYKGCKMGRRKG
               **   **...:.  . *  ..   *. * .****  .*      ****   * ***

40          TKRYMKGGAKGCYAMGKYMMGMGRMKYKKTRRMRRKGRGCRYKTTGTACCWATTWAYKYY
48          WTGYCTTAMKAYCRCRKCCCRCCACKYKKKRRMAGKKRRYRYGYYSYRYTTGGATWCGTY
             . * . *.      *        ****.***  * *  **   .     . :     *

40          RKKKKGMAYCKKCYAATAATGWYRKAKYKATCKGGTAKKGMAYKRSKAAKYCYRKTSYGM
48          RKKKKRARYTKKMYGWWRGGTTTRGWKYGGATKAACGKTRAGTTRSKRMKYTYRKYSCRM
            *****   * ** *.   .    *  ** .: *.. .*.  . .***  ** *** *  *

40          RKCSRCYKKKSYWYKCAKTWSMCTGAYYTKSRTTATARKYGAKGGCCARYGARKWRAGGY
48          RKYCATYKKKSYWYKYRKGWSMMARRYCKKSRGCTCGAKYKMKARYMWRYKTRGTGRRKY
```

```
            ** .  ********* * *** :  * .***  : . **  *.    ** :*      *

40          KRMWWKWYMCCGTRYKCAATCACTGGTATAGCGGMMMGRRRCAAWWYYATTTGGAATGTR
48          KAAWWKTCAMACWRYKGTGCMMYKRKWTCCRMRRMMMKRRRYRRTATTRGCGKKGGYSKR
            *  ***    .  *** :.    .   : .    *** ***             .. ..*

40          SKRRACTTGWYGGSYCYKTRWYGKKKMMGCATGTGRYKCTKRKMYARKGKYRTCCCKCKS
48          SKGRRYYAAWYRRGCSCKGGTYAKKKMMAAWWRGSRTKYYKRGMYCRGRGTGYATTGYKS
            ** *   :.**  . . *   *.*****..    .* *  ** **.*      .    **

40          RRYTCCTAKYYKTTCAGWAWTAARYRGAGRYKCTYRSKRTTGGAGMYRRRMYYKGRYMTT
48          RRYYMYWTKYCKYYYRSAMAATCATARTTGTKAACASTAYWRRRKACRRRMYTKARYMAC
            ***    :** *    .   ::.    :   *.:  *.         ***** *.***:

40          ATGKYKYKATKRYRMKKTGAWYYGTYRTRTYKRKYCYMKGCWRKTRYTTGCTKYYRKRGC
48          TCRGYKYKMYGAYRMTTWACTCTKYYAWACTGGKTMCCGRTTRTYATWWKTCKCCGGAAT
            :   ****    ***.. ..     *       *        *.        *     .

40          AMCTGYRYCWYRSYYYYKASGYCRYMKMMYKRRRAATYMGTKYYTGGTWYAYTCGCGGTT
48          GAMCAYRTYWCRSYCCCKGGTYYGTCKMMYGRGARMYTAKYGYYCAAKATCYYYATATCW
            .   .**  * ***   *.. *    **** *          ** ...  .*  . .

40          TGTKRYRRYTTAYYCKAWRGAAACGYARRRRMMRMRAKKWKKCAKWRAKKTRRWMKTYGC
48          KRCKACAAYCYRYYYKMWARRMRMSTWAAAGAMRMACKKWKGTGKWGWGGWRRWMTKCRS
            .  *    *   ** * *      .       *** .****  .**     ****..  .

40          KWRYGKCTCMMKYKRGGRRRKKYTAKRTRYWGGRKAYSYRRYYRAGRKMYYYRCKWWYKK
48          KARCAKYYMMMKTKAAAGGRKKTWRTAKRYWRRRKWTGTRRCCGRRRKMYYYGYKWWYKG
            * * .*   *** * ..  ***   . .***  **  . **     ******  *****

40          YMCGTGKKGYYRMYYRATTKGTWKRRGWGCAKYYYTTTGRTTGGWWKSMRRKKAKYCCTK
48          YAAKYRTKACYRMTCGMCWKRYTTAARWAMGGCCTKCCAGCAAATTTCCRRKGRKCTYKK
            * .   .*. ***      *   .   *. .    .  .  :..  .. ***  *   .*

40          KTGRRRCCWRCKCYASRRRMYRTMTGAMYKRARKATAGMTAYYMYRYRRCMAKAYRCKCA
48          TCKARGTGAGMTMYRGGARCTRCMKRTCCGACRKWKRACCCYYMYRYRRYAWTWYAYKMT
            .   *      . * .  *  * *. :    .** . .  .********   . *  * :

40          TKMGYYKYRKMKCTKCKARATRRRRRKKRRRYRAYSKAACWGMATGGAYGCTTTTCTYYM
48          YKMKYCGCGGCKYWGYKTRRKGAAGRKKGAACGGCSKWCTWTMCAAAGYKSWYYYYWCTC
             ** *      *    *:* .    ***     . ** . * *.:...* .

40          KTTCTYKKCCRKRYKGCCAGMTGYGYAGRTYMRTASYKAYTYCSKKRWKATRCMAGCWYT
48          KYYYYYKKTARKGCKAYYTKMCTYKCTCGYCAGKMSYKRYYTYGKKRWKRGGYARRTATC
            *    *** .**  *.  : *  *  :      . *** *   .*****

40          GCRRKCGGGRYYCAAAYCKGGKTCKGTRRCKRKGAYYRWRCKKTKKTTGTYMSRRMKMGM
48          SAGGKMAACGTCYRGWCYKRRKCYKACRGTGGKRMCYAWRTKKAKKCGTCCCGRRMKCRM
            ..  * ..      .   *  *  *. *    *   * ** **:**      .****  *

40          KGAKMMYMYRYACKTRWRYYYARWKKKGYTSYWTYKKMRRKRKTTMYCCGTKYWGCKWYW
48          KAGKMMYCCATCYKYRWRTYTRAAKKKRCYSYWCYKGCRRKRKWCCCTTAAKYWRMKATW
            *..****    . * *** *    ***   *** **  *****      .:***  *  *
```

```
40              WSKKGTWYGCGWYKKMM
48              WSGTRCATSAATTKKMM
                ** .    ...  ****
```

The pairwise alignments of two accessions 40 and 49

```
40              KYGYSWYYKYWKYKKAMYKAYKWRYKRTYRRYYARGTRTAWTKYKRRYYRRYSGYWKCRY
49              GTKTGTYYKYTKYKKMMYKRCTTATGRYYRRYYWRRKGWRTAKYKRRCTRRYGCCATTRC
                    . **** **** ***  .    * ***** * .    :*****  ***.   . *

40              YSYWWKYRYMKMRYAAGRRRYCWKWYMKKRWGCWKKAMGAYYKGGRYCKCKSRWTTCGMS
49              CSYWWTCACCGMRYGTTRAGTYWTWYMKKAWTYWKKMMSWCTTRRAYAGYKSRAAATAMS
                 ****.     ***.: *    *.***** *  *** *.   .   *.  *** :: .**

40              WYRCYWMRAWRYMWCRWRGMRWYRYWMYCYYRKMWRYMMRWMKRKKRKWSRRYYTYWAGS
49              WYGYYWCRMTRYMWTRTRKMRWCGCTAYYCCRTCWACAMAWMGGGTGTTSRRCTWCAGKS
                **  ** *  **** * * ***     *   *. *   * **   . . ***     . *

40              KCYAATYCGYTRGYKKYKKKGRWMCAYATTWMYKAKYWCYGYYYMRYYYRGGTGYKYYRR
49              GYCTWYTMRYYRKCKKYKKGTAAMYRCRYWAMYKRKYWMYRCCCMRYYYRRRYRYKYYRR
                   :     * *  *****    *       *** *** *    ******    ******

40              CCMYYKCYKYRWWRWMRRSKRWMMYMKYMRRKAKYMMRRWKRMYMKRWRKYRRRMMRRWY
49              YYCCTKYTKYRTWRWMGRSKRACAYCTCAGRGWKCAAGAWKRMYMKRTGGYAGRAARRWY
                     *  *** **** ****   * .   *  *     ********   *  *  ****

40              KRCCRWAMYKRYWYRCYSYRRWKYGYCYMYYMTYRKAGGYYRRRKMGRGTWCRTYWYRRG
49              KRYYAAMCCKRYTTAMTCTRRATYRCTCMYTMWYRKRRRTYGGGKMARKYWAGYYWYRRK
                **       ***     . ** .*    ** * ***    *   **.*  *.  *****

40              YYGTTGGYRYMGSCKMKYKCWKTCASKYMRWAKRYAMCGKYRGYYGTTRRYRKRRYYRWY
49              YYRAARKYATMKCYKMGCTTAGGYGSKYMRAWGGCTATAGYRRCYACAAGTAGRACYRWT
                ** ::  *  * . **  .     .*****     :  . **  *. :     *  ***

40              KRMMKYMYMRYYKWWAAACAACYYMWMWYTWTYCWYMAGWYCWYRYRRWGTAGGTYRAKK
49              GGMMTTMYMRYCKAWCGCARRYTTCWMWYCTKYYTYAGAATTWYGYRATAATATGCRTKK
                  **. ***** * *....      ****  .*  * ..   ** **  .::.   *:**

40              TKRTATGTTTTKKYCCMARWSKCRCGSMRYRGTAWKKSKCGTTCKAWYRYRKYSMCGCGG
49              AKGCGGTGWYKKKCYSAGGTGTYGGACMRCGACRTTKSKARYWSGMATGYRGCCMARSTK
                :*  .     .**  . .  ..   ..**  .   .***.   .     **  .*. .

40              ARCKACYMWYKYGRYRMRKRKAKKRYYTTYYCKGWGWKTYYRMYAWYYMTTAGACGRYAC
49              GRYKRYYMWYKTRRYRMRKRKRKGAYYYYYYSKKTRTGYTTRMYTACTAKYMRRYKACRY
                .* *  *****  ******** *  **  **.*        ***:    .

40              KCCKYYMKYTARGGGYCCTTGTWYGKTTRKAWKKKWWKRWTATSWGWAKRTATTCTGRAA
49              GMYKYYMKTYRAKRRTYSAGTGTCTTYYRKRWKKKTTTAWKTCCTRARKRCGGWYYRRMR
                   *****         .:      .  ** ****  . *.: .    ** .     *

40              ATTRKTAYRMMSRSRKMTACACGCCGATYKGRKRKCAGTAKYYYSGTKTCTWMGCGMYKM
49              RKWRKYCYRMMSRSRKCKWTGYKYYTGWTTKGKRKYRAWMKYYYCKYKYYGAMSYRMYKM
                 . ** .********* .  .     .  .  ***  .  ****.  *    *.  ****

40              KCCTWYKKRYCGRRYKCARRYAKYRYMMYAYRTTAGACACCRKTSMRRRKKGAAWCRKRK
49              KYYKWYKKAYYRRGTGTRRAYGKYGYMMYRTAYYRRRYRSMAKGGAGAGKGTRWTYATAK
```

```
            *  .**** *  *     * *.** ****          .  * .    *       . *

40          YTAGKWCGKYRKATCRRGYRYTAYAWRRGGCKYYYWRYAMRYYAAATYYTAKRMRSAGWY
49          TYRRGWTSTTRKCCTARRCGCAGTWWRRRKYKYYYTRCRMRYYWMMYCCWRGGMAGMAAC
                 * .. **.   *    :.  ***   **** *  ****          * . .

40          RYYRYKGCGCCTRGAAAAAKYCTRAKYWSTAGAKKCTCTATARYGKGGATKRKCKKTCTR
49          GYYRYKRMSYYWRKRMMMTTCTWGCKYWSYRKGGKMKYKRYRRTRTKCMKKAKSTGATCG
             *****  .   *     :.    .****   . * . .   *  .   .* *.. :

40          TTKKYRCKTACGSYYKTYKTATGSYKYWYTRCAGTAYACRWRKYSWRRMACYCWYGYRTT
49          KGKKCGTTWRYSSYTGYCKYRYRGTTTATCGYGCCWCMTGARKTCWRRMWMTYTYRCGAC
            . **   .   .**    *    . .      .        ** .****     *   :

40          ARYMRKKYYYTYYYRACKAAWYKKYRATKGSTSRKAAARRKKRYCAAAMYGKYKKGKRKT
49          RRYMGKGYYYYYYYGTTKWRATKKYRRYKRSKCRKWRWRGGKRCTGGCMTKKTKKSKAKK
             *** * *** *** : *    ****  * *..**   *  **  ...*  * **.* *.

40          RYCMSYATGKMKYGMGYTCGWKKKCYCAKAKRYKCGGGKRKTGTGAWRAMRGWTYCTYWK
49          RCYMGCCARKAKYTCRCKYAWKKKMCMCKWKRCKTACAGGGCKKAWWGRMRRTWYYGYWK
            *  *. .: * **    . .****   .* ** * . .     .. *  **   *  ***

40          MCGAWYYKWCRCGAKYRRMGKKGRACAAGRCTYCRTRRGGTGYCWYGCKATCKCTKRTRT
49          AARRACCKTYGMKTKYRGMTKGRRWMRMRAYWTYGYGGACCRYTWCTTGGYYKTCGRGRC
             .     *     :*** * *  *              .   * *    .  *   * *

40          GAACRRRGGCCWATATYARMGKCTYAAACTYKCTGKWRYMGGTRACARWAAGCCKKMKTG
49          RTCYAGGSKSYWRCGYYMRMTKMWYCTRYYYKMCAKWGCCATCRRTRRWMGAAAKGAKGA
             :.    . . *  . * ** *  *.:   **  .**   .  *   ** ....*  * .

40          AKKTCMRWTCYRKWGMRKKMWTATMGTAAAGACRYMTYGTCGAYYMKGATGSGTKKYCCA
49          GKGYYMRWKTTAKWAMATGCTCCCMRWMRCTGSRYCYYKAMTWYYCKKMCSSRYKKTYMM
            .*   ***.   **.* .    . *    . ..**  * :   ** *   .*  **

40          AKKSAWTYCATACCSKKRARTMRARGWKYGMATYTGTSYKYACCTRSRACRKTYYMCMKC
49          GKKGWWYYTTGGYSGKGGMRYMRMGTAKYKMRWCYAYSYKYWTTCAGGRYAGYTYMACGY
            .**. * * : . ..*   * **    ** *    . ****     .       **.

40          CYKAGAACGARGATTRKWRYWGCACARTWKYRRWKATTATYYGTKTTMMKCAYRMKTCGG
49          ACKRKGGYKGRATCCRKAATTASTTTACAKYGRTKCWYRGYCAWKYYAMKATYGMKATSS
            . *  ..  .*.:  **    ..: :   ** * *.    * . *   **.:* **: ..

40          GYWATCGGTGTGTAWYRCYRWRYTSYYYMWSAYARMYTAYYKGTTGTRYYYRYYCCKYRA
49          KCTRATTRYRATCMTYGTYRWRYYSCCCCASRYMRCTCCTYKSACCCGTCYRYYMAKYGM
                :     :    *  ***** *     * * *   . **.:      **** .**

40          YRYMSWRRYGTYSYCAGCMMAKWYAYYMMTCTAAKWMSRSRMMYCAKWCRYYWCYCCKYC
49          YRYMSWRRTAGYSYMWRYMCRKWTRYYMMKYCGTTAMCRSRCMCGGTTYRYCTTTTTKTY
            ******** . ***    *  **  ****.  .:. *.*** *  ..  **      *

40          CAKTRARKTCRYKYRYWSMRGTYAGTRWARRYMCSAMRGACKCCKGCRYRYKKRKAMMYA
49          YMKCRWRKCAAYKYRYWSMRRCCTCAGACRGTCTSCMRRWYKYMTRYRYRYKGRKWMCTG
              * * ** . *********   : :  .*    *.**   *  .  ***** ** *  .
```

```
40          YAKATWYAYYRCRASKKCGCYRRSKKTCKKKCTRRKCWRKGCAKCATGYTAMYTTCACKK
49          YRKGGWTCYCGSAMSKKTAYYGRCKKYSTKKSKRRKYAAKKTTKYRYRYCMCTKCTGMKK
            * *. * .*  .  *** . * *.** ..**..***   *  :*    *    .  . **

40          RYKYRGKKRMWYYKYWWTRYYYKGWMCYTCMGGTATTTACKKCGCYSMAARKWRMRGTTT
49          RYKYRAKKRMWYYKYWWYRYCYTKACAYWYCKRACKYYRYKKYKMTGCTGRTTACRRWWK
            *****.*********** ** *.    .*      :..     **     . :.*.    *   .

40          YYCGGRYCKRKMYTKGCTCAYKYMRCCTKKATKCASRGGRCCYYYRWWAGRCYTYKKKRR
49          YTYRKACYGGKCCGKRYYMMYKTAGYSWKGMAKTTGAKKGMYYTTGAARTRYYKCKKTRR
            *         *   *     **     . *  :* :.       *        * *. **.**

40          AAAYRYKWRRGAWKCTAYCAKGGAWYKWAWKWCRRKYRCCGTCGTRWGCRAMKTTYYKMY
49          WRWYRCTAGGKWTKYWWYMRKTKWWYKWGAKWYRGKTRASTCGRGGATMGMMKCYYYKCC
               ** .       *   *  *   ****. ** * * *..            **  ***

40          RAMCKRGRMRMATTRCMRKTAWWWYKKCCKRACAACTYMKYKKYCTTKMRCGYYTTKYTC
49          GTCTKRTRCACGGYRYMRKKRTATYTGTMKRMAGCTGYMKCKTTYCCKAGYAYYYWKCKS
             :  ** *   .  * ***.    *.   ** ...  *** *.     *    .**  * ..

40          TCGYARYKTYGYRTGRAWKGYGYAWTAGGMGGRYRACTKTKYKRCKMCTTATRGGAAWCY
49          KARCWGYKKCRYGCKAGWKSTAYCWYWSRCRKGYGWACKCTCKGYKCYWKMKRRCCWWYY
            ..     **.  *     .**. .*.*   .      *   . * . *  *    . .*   . * *

40          AYKRRRRGYMYRAYAGGAAYCMTCAARYKRCTMKAMGTKYRAYRRGGRGKCARKSRMGCT
49          TYKRRRRTCMCAMYGATTWYMACTGWRCGRMYMGRCRYGYRGYRRRKRRKMWAKSGMKSK
            :******  *    *.. : *     . *  *  *      **.***   * *    ** * ..

40          YYTCRRRTWKKWYRSTRCKGAYTTRAKRRYTKKYRTTCTYWAATTATRKWCRCAYKRYAK
49          CYGAGGRCWKKWYRSCAYGTGTWCRWKRRYAKKYRKWYWTTRRACGARKWARARTTATGK
             *  .  * *******       .   * ****:****.         :  .:***.*.   .   .*

40          WKCAGGGTAKGRRYWCGAGKYKMCWYMSKKYYKYGTTGAMRWSSKAACWMSCYCRCTRAC
49          TKTCATRYRKRAGCWYRRRKYKCYWTASKTYCKYAYYRWCGASGKCGAWCCYYMRYCGMM
             * ..     *    *    ***  *   **.* **.         *.*...* . * *

40          KARMRKKTKKGAGGYAKKAKYCWKRYGTTWKKKAWGCKYTCKCAYTAYAMCKYRAKWYYK
49          TMAAGKKGKTSWKRYWTGGGYTWKRYSYYAKKKRARMKTYMKMMCWWCRCMKCRMGACYK
            .     ** *..    *  .  . * ****.    ***     *    *          * *     **

40          RGTRTACTTKKRKGTTAYGAKYYYKWAGCKYTTGGGRYGGCKACGKYYYYMCYTCKKCCC
49          RAYGYWYYKKKAKSKWWCACKYYYKWTTTKYWARKKGCKRYKGASGYYYCMTYYYTKMTA
            *.       .** *..     ..******:  ** :          *... *** * *   .*   .

40          RCKRGCTYAAASYAAATAYYYYRYRWKWAWRAKMTGGTMKMWWYKRSKKKKSRYKSARKT
49          GMGGKTCCTGGSYGCGCCTYYYGCGAKAMARRKCGAACMKAATTKAGKKKKSRYKSWAKC
                    :..**... . ***     *    * *   .. **      * .*********   *

40          YYACARKTRYKWGYKCKMMMYRASKTKRYRWKAMMRRRKKKCGCYKMGYRKRCAMRMMTA
49          TCWMRGKYGCKTKYKTKCMMTARCGYKGYRTKWMMRRAKGKTAMYKCRYAKGMMCRCMWR
                  *   *  ** * **    .  * ** * **** * * . **   * *     * *
```

```
40      YKMYWKMMACGWRWYGRRAGRCGRKRYKATGCRMRCKKKKWWRYYWGRWKWSWRKMYKYG
49      TGAYTGAMRYRWRTYRGARKATRRGRYKGKKYGMRTKKKGTARYYTARTKWGWRKAYKYA
           *   *   ** *        * ***..   ** ***   *** .* **.*** ***.

40      TGAAKRTYYRWWYAGAYYYYKYYKRCCARKKAYTRRRACYRTKCSGKKGKYRSKTCTWAG
49      CRCGKGGYYRWACTKMCYCTKYYKRYYRRTKWCCAAGMMTGCGMGKKKAKYRSKYMYARK
          ..*  ****  :   *  *****   *.*             . **.*****

40      KYRYCYYRMATWRYYKKKRCAYRAMTGYKCKKKMCMKTAGRCRYKCMRKMYYGTRAARYS
49      KTGYSCYRARYTGCYKTKRTGCRRMGKYKTKKKMTMTWWKGYATKSCGGMYCSYRMMGYC
        *  *. **      **.** . * *  ** **** *.       *.   ** . *   *.

40      RKYAACKAYYGYYWWKKGTWKGTTYMKYRCACGKKMRATTGTWACYGTCKGTRWWYYRYW
49      GGTRRYKMYYTTYWWKTKATKRWYTCKYAYWYKKKMRRWWRYWCGCKCSGKKRWWTCRCA
              * **  ****. : *     **     ****     *.    .  .***  *

40      YYYYWYGGYKYRRKRRSKRKRGYRKMRRYKGCRKYKYRMRKKCKKTKKKSAAGGAAGCGY
49      YYYYWYRKTKCRGKGAGGAKRKYRTCRGTGRARTCGYRMRKKYKKYKKKCCTKCMGATAY
        ******   * * *  .  ** **. *    .*.  ****** ** ***..:   .. .*

40      CYYCKRKYCKCGGYKMGYYAGRGGYWRYTKRRWKKMARAKKRSMCCTGRCRWKKGKKWKA
49      YTCMKRTYMKTARYKMRCTWKRRRCTRYCKGGAKKCWRGGTASAYYYAATRWGKRKKWKG
            **.* * . ***     *    ** *   **  *. . *    .  ** * ****.

40      WYKMAGCAYKMCMTTCKKKRYCTTACSRKCCCRWKAGAAKYGKGWAGGCYYRRSMYKKKK
49      WYKMMKYWCKMYMYWMTKKRYSCCGTSAKGTTAAKRRMRKTRKRARARTCTGRSCCKGTG
        ****     ** *   .****.  . * *     *    *  *   .     **  * .

40      GMCCKCYCGASRRRKMGKRKRTAKCTATGACGCMATCCGTAGGAAYYGTMYYYSRRRYTA
49      KMYSTYYYKGGGARKMRGAGGGGGTYMYRWMKMMWATYKAGAKMRYYRGMYYYSRRRYKW
         * .. *  ..  ***      .          * :   :..   **  *********.

40      YCWAYRCRCKCTKGAMGYRYRYARKSMKCCTKCARRRRRRRYRYTYYTKRAGGACRTKYC
49      YYWWYAGAYKMYTSWMKYACRYRGGGCKYTWGYRAGGGGAACGCKCTCKRWCRMYAAKYS
        * * *    *  .. * *  **   . *                .   **      :**.

40      TTCTYYMTTCTCCRCRKTGCGMRAMSYAYMATYCGSTKKRAMCYMKKCYWYACCGTYGTT
49      YYYWCTMYYYYTARMGKYSMKMGRMSYRYMTYTYKGCKKRRMTYAKKTYWYGYTACYTWC
              *     .*  * .  *  *** **:    . *** * * ** ***.  . *

40      RRKRYYRWCCAKGAYKGTCTCYYRTKYMGKMCRSSSRYKKAMMYRRTTRGTKYYYRTGGC
49      GGKGTCRWGYRKKWTKCYYKYTCRYKCMKKMYGGGGGTKKGMMYRGKYRKYGYYYRCAAM
          *   **   *   *   .   * * * **  ...  **.**** . *   **** ..

40      SYTWKATCGYTTRMYMCGYCAYRCGYYARRRRRMRMRKCKMCKTAGTKKKWRRWMAMGSC
49      CCKAKCYYRYCCACCMYAYYMYAMSTYWAAAGRMRMAKMKAYGATTAGKKWRRWMRACSM
        . . *.   *     * .*  *  . *     **** * *   :: : *******   *

40      RWYRYGACACCCKMMYKGAYYWKRRKKYRCKRGGCTYTRYWYKMRRKYSYRAAKYRRWMY
49      RACRCACMMTTTKMMYGAMYTWKRGKKTGMTAAATCTKRYWYKMRRTTGTRGGKCGRWMY
        *  * ..     **** . * *** **   . ..   .********. . *..*  ****

40      YYRCGKKKCKKTGKGYYRKRKMCMYYYRGCCTWKRRGWKWKKYMTTGGWWKSMYAKRRKK
```

```
49          YYGYRKGKAKKYRTAYYRKRKCYATTCGATTATTAARWGWKGCMCAAATTTCCYRKRRKK
            **   * *.**  ..******       .  : .   * **  * :..  .. * *****

40          GGTCGKCTGYRKTKCKKKTTTMMKGAMMMGMYRMCGCAKKCAKCRSRKKGWRWCGCYYCK
49          AAAARKYGRCAKAKYKKKYWKCMKRRMMMKMYGMTTTTKKTCKYRSRKKAWRTYRYTTMK
            ..:. *     *:* ***  . **  *** ** *   :** .* *****.**       *

40          WAGAGYTSYWRTKTTMRRCKRMTCRCGTMYARCKWWSRKKKTTYWYRWWMRMMKKMAGGW
49          TGAGACYSYWRYGWACRRYKACWSRSTWCCGRYKWWSRGTKKYTATGTWMRMMKKAWTSW
             ....  ****   : ** *   .*.    .* ***** .*.      *******   .*

40          TKMTKKKGAYKGKMYKYCRMRRTCCTGMMCYYSKYTGYGMSWCTRRRGKAAGKCRAKKKC
49          WKCKKKGTTYKRKAYKCMRMRRWTYATACGTYSKYKRCRMSWMCARRKGGTRTTRRKTKT
             * .**  :** * **  ****   :     ****.   ***   **  .: . * *.*

40          KAGGRAYCTKSKKKYKKAGARYKWSCMGTCASYCTGACRKYAGMCWTTCYCTCTTMRWYY
49          KGTARCYAAKSKKKYKKGRRRYKWCYMAWYWSYTYRRSRKTTAMATGGTCTYAAYCGTCY
            *. .*.*.:********.  ****. *.   **    .** :.*.       .:     *

40          YSAKKGGCTRTSYCTAMMSTGKTGATTKTTYCCCCGYKTTTTCGAGYWKAGGATAAYAAA
49          TSGKKKKMKAYSYYAWMACAAKGAGYWKACTYMTGAYGWCWWTRGKYWKGTTWWMRYMCM
             *.**   .  ** : * .:.* ..  *:      .*       . ***.      * .

40          TKGCGCGMGMMKKCAWWYYGGTKATRRASWGYYGRTSACTACKTTTKGTATYKCKACAGA
49          WKAAAMAMRMMGKYRTATTKKYKGKRGRGWRYYRGCGGMWGYKCACKAGTAYKGKWSWAG
             *... .* ** *         *..*  .* **   ..  . * : *. ::** * . ..

40          YWAKTCTTKCCAGSKYRMYAYWTAGKGKYRRYYWTCCCCMGCGGGTCGTTTKYYKAGTTT
49          TTTGWYWKKYTCAGGYRMYRYTGGAGRGTGACYWYYATTARARAKYMCGGWKYCKMAAYY
              :    .*  ... **** *  ..       **  .    . .       ** * .:

40          CAGWAWRYRGTTYKKYCTYRKSGKRWTAMYACCATYYKGAACRYYKYKAKRTYRWMKGGA
49          YRSAMAATARCCTKKTAACAGSKTAWWTAYWMYRACTGRRWSRYYKYKMGAYYRTMKRAC
              .          ** .:   * . * : *    :      .******    ** ** ..

40          YWYYGTCRKTKKWWYKSWWRRRKCTAKYYTTGKYYRTKRGCACAGGRTGYRKKWRRYTYR
49          TTCTKYAAKWKKWWCKGTTAARTAYCKYYWWKKCCGAGAATGMMKSRCAYRKKTGRCCYG
                  . * **** *.    *.. .***   *   :  . .   .* .****  *  *

40          RACKMCGCAGKCTKTRRTKTRAKGYRRWCCKCKRGCAGACKWGKKMWYTGGMCAGGKKGG
49          GRYKMMAYCSKACKKGGCKCGGGKYAGTATKTKARMMRWMKAKKTAWYKRKMYRKKKKKR
               ** . ..*. *.   *  .  *   . * *       *  *. **.  *    **

40          GKRGKTYRYKKKSCWRAWMKCATRKKRAATYAAACATWMMYYYRTKKWSGRAYWMYRGWR
49          RKGRKAYRYTKKGYWGGWMGYRWRGKRCGATMRRYMYTCACYYAWKKAGTGWYWMYRKTR
             *  *:***.**. * .**    * **..:           **  **  .   *****  *

40          TGGCCSMWYYRMRRWKWYAAGGTKACKGYGCKKAKKYAGMYRRRKRRYYRCRKWKRCRCA
49          WKRYMSMACYAMRAWTTTTGTACKGTKKYTSKKWKKYTKCTAGAGRRYYRTRKTKATAAC
                 **  * ** *.  :. . *. * * .** ***:       ***** ** *   ..

40          TTCGSYTGRYKRWGCTAGWRKRGCATCKTYGTAKKYCYYYWKCRWKYYKMRTMRRYYKRY
49          AATRSYYTRYGRWTTARTWRGGRTGAAKWYTAGGKTGYTTWKYRAKYYKCGYMGRYYKGC
```

```
            ::  **  ** **  :  **    .:.* * :. *  *  ** * ****   * ****

40          GKTAKKRYMKCAYKRRMYMKKCAGGGKRMRRRGCRYCKMMGKWCGRGRYKKYYRKKKGWK
49          KKYWGTGYCKYMTKGGMCCKKYRACTKRMAGRRYRYYGAASGTYRGKRCGKTYRKKKRWT
             *   . * *   *  *  **  .  ***  *  **    .      *  * ***** *.

40          CKKGGCYKAKCCTCKKGKRSKYCWKATGAKGMRKGRSRCYKYSYWYKCWAKSYKTTKYAK
49          TKKRTYCKTKYTATKKTTRSKYTWKMYAMTRMRKTRCATYKYSYWYGYARKSCKCCGYGK
             **    *:*  : ** .**** **  . . *** *.  *******    ** *   *.*

40          YKGAKWWGCRYGRKGWRRWWKSYMWYMCTATKTCACAATGGAGTRYTGYCAATACTGKRT
49          TTKMKWWRYRYKRGKTGAWWKGTCTCAMYRYGWAWTGRKCACAWRYYRCGTGCMYKRKGW
             .  ***  ** *     ***.           .  . . ... **    :.   . *

40          TTAGGKAKKRWRMYRCRRTGAARAKAATGCCCAWKRCWRYYCGWKSTCRYTGTKGMMRGK
49          CGMTRGRGKRWRMYRYRRYATCGMTCGYRYMMGAKAMTRYYTKAKGKYGYWKWGKACAKT
                    ******* ** .:.  ...     . *   ***   *..  *         .

40          ATCWGKGYAKMGAGTRTKRYAARGRGAARYTAYCTYYKKWMYYYRWWWYTMRRGTAYRRK
49          CCATRTKTRKMTGAARYKRCRMGKRAGGRYWWTTYTYGKTATYYRTAACCARGAGGYGRK
            . .  .   ** ..:* **     *...**      * *   ***      * . .* **

40          AYYYKTCWRYCAWWAAACKKCYTRWGYCTCTCYCGTGGKYRWRMRRYKYKGSKKRAMATM
49          GTCCGYYARYYRTATTGTKKYYGAARTTAAATCYAYSRKCRAGAAATKCKASKKGMCRKM
            .       **    ::. ** *      :.:   . . * *      * *.***    .*

40          GYMYKGTKYKKCYCYYYKKMSACYKYATRSKYGTTGCGCMCCTKKMYRYYYRWYTYWRWA
49          AYMYKRYKYKKYCAYYCKTMSRYTTYRYAGKCKWCSYRYMYTCKGCTGCYYRWTGYTATT
            .****  ****  .** *.**   .*   .*    .   *   *     ****  *   :

40          SKRGMRRYKYCRRRAKSWMRYRTMKRRGTYSMRKMRCYKTRYMKRYSAKRWKWGYAMYRG
49          SKRRCRRCKYYRRRMKGWAACRKCKGAKWYGMGGCGSCKCRCCKGTCGKGTKWKTGMYRA
            ***  ** ** *** *.*   *. *    *.*    . * *  *  ..*  **  .***.

40          GCCGTSRAGMRRTAGKKRTRAATTSMRTGWGKKKWYWMRKRKYGKTACKKAMCKKKKYR
49          TAMTWGRMSMGAWGRKKRCGRMKWSMAWRWTGKKWTAAATRGYRKCGTKKMAMTKKKTR
             .   .* .*   . ***    . **   *  ***    .* * * . **   .*** *
```

The pairwise alignments of two accessions 40 and 50

```
40        RAYTTGGRTMKYYRRWCGGKACCKKGGRTMRMMWKWRRRYCCCKWGWWMKKCCKCGWRRK
50        GRCYKRTGAMTYYRRWTRRKGTTKGKAAKMRMMWKWGAGYATTKWCTTCGKAAKYTWRRK
              .   :*.*****   *.  *  . .*******   *.  **     *..*  ****

40        MYACAKGAYYYYRRSRYYWCCCKTTAYRTGYMYKRYCYRWYYRCARRRGCCKACRWRCMY
50        MYRYRKACYCTYRRSGCTTYSYTCCTYRYAYMYKRYYTGTTYRARRAACTAKRMRWRMCT
          **   *..*  ****     . .  :** .******     **. *    .*  ***

40        TGAYTRCAKRYRWYKYYMYKRTTYTYTRKKYCYTGYRARRKKWWMRMRWSYWWMYKWMTT
50        GAMYYRMWKRYRWCGTTCTGGWWCYYKGKKTMCWRTARRRTKWWMRMRTSYWWMYTACCC
           . * *  *****            *. **        **.******* ******.

40        CTYKRRRWYYMRRRAGGKCWKYMRYWKSWYWRRGGARYRYMKKMWYAGKKWRCYMYTGTY
50        ACCGRRRTYYMRRRRRRKYWKYMRYWKSWYARRKRMRTRTAGTCWTWKKKAATTMYWRWY
          .   *** ******   * *********** **   * *   . *   **    **   *

40        KYRWCCYRAYWCGYRYKRWYRYYGSKRTAGAMACYSCWRCMCYYRRKRMYYKRRRYKKCT
50        KYRWYYYRRCAARYRYGRWYRYCKSKRACTCCCYTCYAGYCYYYRRKRMTCTRAGTTKYY
          ****  **   . *** *****  ***:. . .  .      *******  .*   .*

40        CARKMYRRKAKMRYGYYKRYRMRYYMYKRMYKCTGTKRAATTGRKAYRCMKWTMGYWGRS
50        YRATMYGRGWKMRYKCYKRYRMRCCAYGACYKAARAKRGTGCKGKRCGYMGTKCKYWKRS
             .** *  ****  *******   *   **.: :**.:    *    *  .  ** **

40        RRYMGMKSMCMKYKGMRMRRKCYMYGSGCAYGYWWRKGAGYYYKRWKWRYRYGSTATAKC
50        RRCAAMKSMYAKYKRCRMGRGYCCYAGRYGCAYWTGGTRKYYYGGAKWRYAYKCWTCCKY
          **  .****  ***  ** *    *..  . .**      ***   **** * . : .*

40        YRRRCAWWYYGGKKWMKSKRGRKMYKWYCARKKKWAGGGTATYKCAAYKWYTKCSRRWAA
50        TRRRYRTATTKKKKTAKSKGKGKMYKWYSRAGKKWRRRAGTAYKSWGTKWYCGYGRRTTC
           ***        **  ***   ******.   ***   . ::**. . ***   .** :.

40        GKRTAKKYRRYCCTRTKYARYTTTCWSAGWAWRYRAYKKCTKRWTATMRRRCYYKYKYKR
50        KKGGGGGTGACMTCRWKYRGCYYYYWSRSAMAATATTKKAATAWWGCCRRRACTKYKYGA
           *  .         * **       ** .      : **.:. * .  ***.  ****

40        YRMKGARWYYGTCYRKTYKRYKWSTGYRATRAACKAKKCKMTRRTTMRKRAKWRRMYRKC
50        YRMKACRTCTKYACAKCTGGYKWGYRYRWYAGGTKWGKMKMWRAWWCGTRMGWAGACAKY
          ****..*     .  *    ***.  **   .. *  * ** *     .*  *     *

40        TTRCCCCMCGCWARCKARRCMWRAGCCMMRWGTYCTRACGKGCYKCACTCKACGRRACRT
50        GKGYYMMMMAYTWRYTGGRYCARTAYMAMAWRKTYYACYRTKMTKYMYKYGGGRRRMGRW
           .     * .   * .. *   *:.   * * .    .  .   *   .  .  **  *

40        YTGSYYTKWGMRYYYRGWGRKWKTMKGYRKGRCAYYYKWMRWRYKGMKRKTGGKRMAYYR
50        TYRSTTCKTRARTYYRTTSRKAKCAKRYRKSAYTTCCGAAAARYKAAKRKWRRKRMCYYG
             *   *   * ***  .** *  * ***.  :        ***. ***   ***.**

40        RAKKYKRKCKTAATRMRKRKATARTYRYMRGCAKWGKWYRGGKTRKKGKRYYKKKWKAAA
50        GRGKYKRKAGKRWKRARKGKCCMGYYRYMARMRKAKTWYGKKKGRKTRKRYYTKGWKMGG
```

```
         *****. .  .* ** *.    ****    *  .**   * **. ****.* ** ..

40      GAWTRKWRMKATKGTAAAACGKMYYYRACATAKKYRRRYAGWTRRSRGCCMWYRRWKWYM
50      ATWCRTTRMKCAKKCTMRRYRKACYYAGYWYWKKTGARCWKTGGGSRAYMMACAAWTTTM
        .:* *. ***.:*  :     *  ** .    **   *       **.  *    *.  *

40      AAGGACKYYRAYKWCGYWCKRGRYWRCMCRYRYRKRKRYWRKTRYWWKWRWKAAAKYRYK
50      TGTAGTKTYRWYKWTACAAGRAGYTAAMTRYRYRGRTRYWRKCGYWWKWRWKCCTKYGYK
        :. .. * ** *** .  . *. *  .* ***** *.*****  ********..:** **

40      WWRYYWRWKAGGCWRRYCAAAMKYTCAGTCKRYTRTCKYMSKWCCYAGTCGWWYYTYKWR
50      TARYYWRTKGRRMWRRYAGTGAKYCYWAYYKRYYRYYGCCGGWGGCTAWTAWACTWYKWG
          ***** *.   ****..:. **   .  *** *     . *   :.  .*    ***

40      GTGTAYGTCCYKTRTYRWRRWYYTTGCTKCTCMKTKYKMGKMMGKSGATCKTWRYCKKKY
50      RCACGYAAMAYKGAKYAWRRWYYYKRYAKMCYCGYKYKCRKMMKKCRCKYKKWRYMKKKC
          . .*.: .**  .* ****** .  :*      ***  *** *. .. *.*** ***

40      TTSYWTWRKMRRMACTKTGGTRMTMTAMYGRKRATAAKYWGCAKGSAKWWSWYRMWCCYK
50      YKSYWYWRGMRRMGTAKAAAARCWMCRCCTRKRCCGCKYWRMTKKGCKWWSATAMTYTTK
         .*** ** ****. :*:..:*  *     ***. ..***  :* ..****   *    *

40      MKKYYMGGCGWYMCAMKYYCRRSRWRYCRTYWKYRMTYRAGGAWAGCCRGRTRRKGRYWY
50      CKTYYMAAMTWYCSMCKYTYRGGATGYTRCYAKYGMYTATAAGARSSMATRGAGKCAYWT
         *.***..  ** .  **  * .   * * * ** *   :...  ..   *   *  **

40      ATCRCTTWYRCYRYYAWGGCKYWAGYGGCRCGMRYYKYYTATAKWRRSKKYYYKTGAAKR
50      RWTGAACWTRYCGYTWWRKYKYTTRCTRMRATMRYYKCCAWWRKAGAGKKYYCGKAMMKR
            .: * *   *  *   ** :     *. *****  :   *   .****  ..  **

40      MYWSTAGKCCTTTATYGGKKCKCKRKKRRRMTKTAAAGGSCCRYYSRYTCCYYRGGKSYW
50      MCWSYRKKMACAYRKTKSKKGGSTGKKGGGCWTWGGCASSYMRYYSRCYYYTCATAKSYT
        * **   * . :  .  .**  .. **     . .....*  *****        .***

40      WYYMKRGSSSKMMYTGTKYATGGTTCTAGRYRRACACAYRGAMMRRGAGKTYMCKMWKKG
50      AYYMKARGGGKMMYYKYGYMCAAKYYCRSACARTYGYMYASWAMRAKWKKGCAMKAWKGA
         ****  ...****    *  ...    .   *: .  * .  **    *    * ** .

40      MWCAACATKTMGMCKRYKMTAMMMCYWRKRRRKYRTKCKRRYMYRSWTKRYWGMYSYTRR
50      CAYCCTGCGCACCAKRYTAAGAMMYTWRKARGKTRWKMTAGTCCRSWKKRYWKMTGTWRR
           .. .      .***. :. **  *** * * * * .     ***.**** * .  **

40      YRCAARMYYYMRRRCTTTGMKCKMYMCTKGYRCKYKWKRRGWWKYYYWWKSRKRCTTKMK
50      CGMRCRMYYYMRGRYYAGKCKTKMCAAYTAYRYKTKTTAAAWTGCCTTTTCRGRMWKKMK
            .******* *  :   * **  . ..** * * .  .*       ..* *  .***

40      AMCKYMRTMRRYSYRKRRKYKRYKAKYMKYKKRMRRGKKRGCAKRAAAKKGMRAYKCKGG
50      RAYKYCGYMGRCSCGKRRKYTGYTMGCCKYGKRMAGKKKRRYGGRWWWKKRARRYTTKRT
           **   * * *  *****. *.    ** ***   ***  . *   **   * *. *

40      GYCRCCTTCSYKSYKTGTTRSKTYCWCRAGTSKMTGRKCRYRKSRCYSYWYKAKTMTRMC
50      RYTGYYAATSYKSYKCRAARSGKTTWYRMRYSKMCKRKYRYRTCATYSYWYKGKYAARCA
         *    :: ******  ::** .  * *   ***  ** ***..  ******.*  :* .
```

```
40          TYKAAKYYKKYGAYRMYGAKGCCRYGGGRKKWRACGAMWWKWYMCCTACAATGGAGTKYC
50          AYGGCKYYGGYKGYRACKMKRYMRYKKKRGKTGTTAGAWWKTCAMMWTTGRKCACAWKCG
            :* ..***  * .**    *   **   * *  : .. ***      : . . ... *

40          AATWTCGTGGKRMRRYKGCGGKYRRKKGWRYRAGTGGGYMYKKKGKMTAAGYYYKMSRYR
50          TGCWCTACCAKGAAATKKATKGYRRKKAWRYGGACAARYMYGKKAKCCTRKCYCKMSRTG
            :. *  .  .*     * .   *****.*** .. .. *** **.*  :   * ****

40          KYKYATRRSKYYGTTTAGRAKGGWKTGCYRCKMYRYGATGYYRWYYAMKRGGTTTGAYYK
50          KTTYRWRAGKCYKWCCTRRRKKTAGKKYYAMGCTGCAGWKYYATTCTMKRKTYKCARCYK
            * .*  * .* *    : * *    .  *       ..  **    :***   . .  **

40          SRYRRMWKGSMMKCKCCYYMKRYSAKKGARTACARYSKCGTRRAATTRMYAGKKKWKWYW
50          GACRRMWKSGCMKTGMTCCCKGTCGKKKGRYRMRRYGGASWRGRMKWRMCRTKGGTGWTA
            .  *****.. **       *  ..** .*    **. .. *   . **   *    *

40          WGTRRKWGWKWYYMGGRRCCATCKKYKGKTAAAWTARRYKGYRRRWYATRYKWWAKYYKM
50          TTCARGWKTGWYYMATRGMYRWYTKTKKKCCGGTCTRGYGRYRRGTYWYRYGATTKYYKM
                * *   ****. *      .* * * ...  :* *  ***  *  **   :*****

40          GMGAACRYACYAAGAYGCAAAWYCRWWTRGTGARYARRWYKMAGYYRKKMWKKTCTKGGM
50          SMRTCYRYCAYTTRMYAMMCTWCTRWTWGCGAGGCWGGTYGMMRYYATGCTKKWMYKRKM
            .* :. **..*::  *.  .:*  **     ..      * *  ** .   **   *  *

40          TKSYKYWYKCGYRAWYKACCTRTRAAAGKKTWWRRYRWMACRGCKGYYGAAMATTWYRKR
50          YKGTTYATKARCRWWCKCYTYGWGTWWRKKYWWRRYRWMWMRRYGATYSRRMRCCWYRKR
             *. .*  *.  * * *.      :   ** ********  *   . *.  *   *****

40          TRKRYYYTGAYYRKKMRAYRSWKMCTRKKYMYKCCYYRGRTRRMSYGMKCYTYYYTTWRR
50          AGGGYYYARRYYGKKMRRYRSWKMTCRKKCMTKYMYYRSAKRRMGCRAGYYCYYCKCTAA
            :   ***:  ** **** ******  *** * *  ***. .***.     * ** .

40          AAKCKAKKRGCCTGYKCGGRKCMAWTRAWWTKYKWYYTTKKTTGCRTCSCATRYCCCTAA
50          WWKMKWKKRRSYCCCKTACAGMARWGRGTTWKTGACCKYKKKCATAYTGTTARYSMTGCT
              * * *** .    * .      * *.   *     . **. .    . ::**.   .:

40          YMMRKYKRKYACKYKMGYRTCRGGACTYGKGGWYWRYTKATARRWKMRAKWTSAMRYCCT
50          TMMGTYKRTYWMKTKMRCGKYGRKRYYYKKRRWCWRYCKRYMGGAKCRGGWYSRARYTTC
             ** .***.*  * **   .       * *  * *** *      * *. * *  **

40          GKRCKKCAYKMCMTKGWYKAYSRKWCKMMKKYGKGAGAYRSGYYGWKRRMCWKKTTYTCC
50          AKAYGGYWCGCTCCKAACKGTSAKAMGCMKKTAKRCKMCRSACYATKGGMYWKKCACAMM
            .*            *.  *. * *    *** .* .   **. *. *  * *** : :

40          TRCGAYATAATTKRYYYRMGGTARYSRKTKYRTYWKKCKRMGAYKRYYGGKWGTGTATGR
50          CAYTGTMWWGAGKRCYCRCRRWRRYSRKWKYRKTTKKYKRMARTTATCATGTRCKYRKRA
                .    .: ** * *     ***** ***.  ** ***.  .   .        .

40          RYYWCGAGKYRAACYYSKKCYCTMYYRRAAKWAASWMSCYKYRYGKKTKYAYRMRKYKRY
50          GCYWYRRRKYRWWYYYSTKYCMGCTYRGCGGWCGSWCCYYKTRCKGKGTTRYRMRGCGRC
              **    ***   ***.*      ** .. *..** . ** *   * .  ****   *
```

```
40          GRRATCTRYWKKGYCKYTCKWCAYARMGRACTCAYYAKWYACKYYKKRRMRTCSYGAYRK
50          KGARWYCRYAKKRYMKTYMKWMMCGRCCRGACYRCYMGACTTKYCKKRAMACTCCACYAK
                   ** ** * *   **   .*  *..    *    : ** *** *   . ..* *

40          CGRYRRSWKGKTTKKRMCTAKRKKCTGMRCWGYRKTRTMYTTCACKKYSMKGACKGTTKW
50          TARYGRCAKTTCYKKRMSKWKRKGMYRCRYARYRKKRCCTKCTGMKKTGCGATYTAKCKW
             .** *. * .  ****.. ***     *   ***.*   .  . ** .  .: ... **

40          YCKWGTYMKRYYGGKCACTMYMYCCTGCCYTCKRKWRMWTAGTTRTCGGRAKTKKCKCTA
50          YYKWAYTATRYCKRGYCTWCCCCYYKATAYYGKRTTACAWGAGCRYYRKAWKGKTYKMYT
            * **.   .**     .        .. .*  **.     ..  *      * *. *  :

40          AKKKCSRGGRYYYTCGTCCTKKRYGKKYWTGAKAGCGAAKGAYKKKCAMAAGTKMKWKMC
50          MKGKTGAKKGYTTAARCYYCKTRCKKKYTWRRKGRYSWWKTWYKKGYRMRWSWKCKAKMT
             * * .    *  :.     *.*  ***    *.  .  *  ***   *  . * * **

40          KGYRMRRCGKKTRCACAGTCAARRWTSKSYGMSWTTARYATYRRRKGCACRATAWRRCTA
50          TACRMRRTAKKWRYCACACTGRAGAAGKSYRASWACTACWCYRRRGRTGGRGAGARGYYW
            .. **** .** * ....  .    :.***  **: :    ****   . *.:. *

40          CGKCKAGGGCCCKSKKGCMAGMTSYTGRKKKYYMYYCCMRTMRWAKKYCRTAACWMMKCG
50          TKKTKRRKRTAMTCTKSYMMAMASYYRRGKKTYMCCYTMAGCGTGKKYGAYRRYACMTAR
              * *     . ...*. * .*:**  * ** **    *     .***        *..

40          KATCCCCCGYGKYYKAYAAGCGYWYMKKTKGGKRYWWKCAYYYYTGKCCCYYCGSYYYRY
50          KWCTATTGAYKKYYKCTRTRMAYWYCKKWKRKKRCWAGYMYYYYYTKMMMYYMASYYYGC
            *   .   .* ****.  :  .*** ** *  ** *    ****  *   ** .****

40          KWKWWAMYYRKCMAKRWGRGGTTAKRSTKKWRYRKSRGMKGCSKTACRACAKTRKWARGW
50          KAKAARMYYRKMMWKRWRGAACYCKAGYKKWRYRKSRKMKTYSGWWTAWAGKYGKTTGTW
            * *   ***** * ***  ..  .* . ********* **  *      ..*  * :  *

40          GRTAMYRYRSWYWKAMMRRRKKYKATYRKRMMMTACCYAWGGMRRKGWCTTGCGRKYGRW
50          SRKWCTACRCWYTKWMMRRAKKYKGAYAKGCCMWRTYYRWAKMGRKRTMGCCSAGGTRGA
            .*.     *.** * **** ****.:* *   *    * *. * **      ..

40          CGCRGWTGTATRYKCMYRCRWKCKAMKYWRKATWYTYAYYTTKKCGYGCRTSTACYYAYS
50          YKMRKWWRYMYGYGYCYRTRTKTTGMKCWGTWKWTCYGYYYWKGARCATRWSKRTYYGCS
               * *      *   ** * * ..** * . .*  *.**  * .  . * *.  **. *

40          CMYRYATATAGGWTRYKYKKRCTWWCYKYKRRCRRYYCKYTCYTRWWYWMRYRAAGMYTM
50          YMYGTCKRYWSRAWGCTCKKGYWWWYYKYKRRYGGYYAKYYYCYRWTTTCAYRMGAACYM
             **  ..   .     . **   ** ******   **.**    **     ** ..   *

40          KAGKCAKCRYGTGTCMYYYYMWGYSRRYTGRYRSRGAMYYYYKKYRYYGMMRRWYCYSYC
50          GWRTMWGYACSKKWSMYYYYCARYSGATCARYRSRRRMYYYYKKYRYCAMMRRWTMTGTA
               .      ..  .*****   **    .*****  ********** .*****   . .

40          WWWMGYTTYWGKKYRGRMMTAASRCMMYYKMRCTTKWCKATTYKMKCRYMRYSMRWMKAR
50          TTTAACKGTTAKKTGAGMMCGTSRMCMTCGARTYCTTAKRYGTKMGAAYMRYSMRWMKWG
                . .   .**  . ** .:**  *    *   . .*    ** . **********

40          GARCTCTRTGCGYKRRYKKKYAYCCTAMRYCGCAAAGCACAAYYRRKTGKKGTCCKYASR
```

```
50          RWRYCSCGCRMRYKARYKKKYTTTYCTMRYAAMGGTRTCTTGTTGGGYSKTKCTSGYGGG
              *  .      ** ******:    :***.. ..:  . :.      .*.   . *..

40          RRTCKSTKTTYYMYSRTTAKCCAAYMRRKTWKKMYKRGCTACRKSMGTCYTRRRARYSKA
50          AGYYKSYKYCYYMYSRCKWKTAMWYAAAKYTTTMYKGRYWWMGGGCKWYYARRRMGCSKC
                ** *  ****** . * .  *   *  ..***        .    *:***   **.

40          ARKYCTTTCTYYTTTCTCYCCTKGMTGGCRRKKSYAYMTYCKRYWRAMYMWWKKCWYTTR
50          WAKYSWYYYWCTYYYYYAYTAWKKMCTTYRGKGSYRYMYTYKRYWRRMYAWWGKTWYCCG
              **.            .* . * *    * * ** **   ***** ** ** * **

40          KRYYCAYGKRGMASTTAKYKCYYKCWYRCGWKSKTGTWYRYRRCACKYRGAGAKATTKGW
50          GGTCYRTRKGAMWGWCGKCKSYYGMTYGGATKSGYRYATGYRRYRYKYRTGTMKMYYKKT
                    * .* .  .* *.**   *  . **       ***   *** .  *   *

40          GWTYYYYRMYAWYYSYCGTGMRTTTGRKKCTGYRKYMKCCWYRKAWKKKRWKAATGTAKG
50          RTYCCTTRMYTACTSYTACAARWKYKAKKGGAYATCAKYSTYRKRWKKTRWGMTCAKWKR
                   ***:   ** . . * .   **  .* .  * . *** ***.**  : .. *

40          WACGWWGAAACKMMWWTKKAYTCCRCGGATTKRWKCATAYKMYAATMTGKYMKAWMYMYR
50          ARYRAWRGGGYKMMWWGKKCYYYYAYKTGKWGRWKYRWMYKMCRMYAGAKTMKMWMYMYR
                 * ... ***** **.*       ..  ***    ***      .* ** ******

40          YCKCCYYYCTRAATGCGCWCAAACTTYTACCMWSCGATTWRYWGGRMKARMKTYTAGGWG
50          YTKYSYYTGYRWMYKMKYTTRRMTYCTCWMSMWSYRWYYAATTAARCTGAMKCYAWAATR
            * * .**   *                   .***         ..* .. ** *: ..

40          AMTGTACYRWRYATCCARKATATTTYRTATTKKYSWCAYKYRKAAACKWCYKKWRYCTKK
50          MAATCMYTGTGTGKTTGRTTATCCKCGCMWKTKYSWYRYKTGKGGTSKAMCKKTGCTGTK
              :         ..  .*.:::  .     ..****  **  *..:.*   **     .*

40          MAYRGRCAGGKKKMYMKYRWWCYRTAKMYTYTKRSRYRKKMMTWTGGTTYTCAATTKKCK
50          MCCRKGTGASTKKMYMKTGWWYYRWMGMYKCKKGCGYRGKCCATASRWYTYYRRWWKTSG
            *. *   ....******  ** **   **. .* . ** *  : :.          *..

40          KAYGCWWKYYKRRRKMRKKRKKYKRKKA
50          KGCRYAWKYYGARATCRKKRKTCKRKKW
            *.    ****  * . *****. ****
```

The pairwise alignments of two accessions 40 and 51

```
40              KGYYWSYWRWCARSMYCRRGRYRKWRGTTRRACGWMKRCYAMYRYCRAMKAGGRYYRWRW
51              GKYYWSYWRWTTRCMTAGGRACGKWRRACAGTTCTCKRMTRMYRTMRGCKRRRRTYRWRW
                  ******** :*.* .      *** :   :    **   ***  *. *   * *****

40              AMYGSYCGGWRRRGKKYYRRRRMCYKWMRKAYWCCTYKARWMRWYWKWGSWKYYRKWACC
51              CCYAGYTTATGRGTKGYYRRRRMYTKTMGKRCWYTCYTMRWMGWYWKTKGTTTYGTWGTT
                . *..*  .  *  * *******  * * *  *   *. *** ****  . . * .*.

40              KRCRAWMMKWTARCCCKMWTYRYMGKKTAYKRCCGYWYYYSRGTRYYKKYYYYRYYYKRG
51              KRTRRWMMKWCTRATTKMWWCRCAKKGYWYKRYMRYTYYYCGRGGYCKKYYCYRCTYKAK
                ** * ***** :*.  ***  *   *   ***   * ***.    * **** **  **

40              CYRYYSYWWYRCRRCRCCYWAMMARRKRYRYGSRYKYMRYTGCCCWRKKRRRRKAKRYMY
51              YYRCCGTATYRYRRTAYYYTTCAMAAGRCGCKSACKYMRYYKMMAWGGKGRGRGWKRCAY
                 **  .   ** **    * :      *    *  *****    .*  * * *  **  *

40              YKRYRWWWYWSMMYMKRAMMGMCYYKYMYKYRGGTYCCYMKGCWWKCMCRSMYWYARRGY
51              YKRYRWAWYWSMMYMKARAARMYTCGTMTGYRRRKYMMYMTKAAWKYMMRSMYAYMARRT
                ****** *********     *     *  **  .*  **. . ** * **** *  *

40              MWYTWKMSTGCRMMWKYRWGYCTRRYWGCTRKKKKKYAGCKYYRARCKYYCTACRYRGGG
51              ATTCATCSCRTRCCWKYRTRCMWAGTWAMWGKKKKKYRRTKCTGTRYKCYYYWYGCRRAA
                     . *   *  ****        *.   ******   *   :* * *      * ..

40              RCRYYKMKAWCMYKYKKYWYYARGTKRMWKTSRRTYKCWRKATGRTKSCKKMKGTYATYR
51              GYRYYKMGRTAMYKYKKTTYYWARAGGAATKSRRCYGSARGTCAGCKGTKTMKGTTTYYR
                  *****   .******  **   :    ..*** * . * : .  *. *.**   : **

40              YSWKAYKYRKWCKKWCTYARYKKKARYRAMMKKMMYRATACTYRYMWKWRTAYKKSRKYY
51              YSWGRYKTGKAMTTTYCCTGCGTKCRCRRMCGKMMYGMWWYYYRYMWKWRWMCTKCGGTY
                ***  **  *  ..    :   .*.* * *  ****      ********   .*.   *

40              RKAGWYGTTYKKCAATTGWKKKGTKGYWWYMKACYCATYKRRRRMRKAYRAYKYKWRKKY
51              RKMKTCRWYTKGYRRWWRWKTGTGKTCAWYMKCTYACGYGARRRCRKWTGGCKYKWRKKY
                **        *       **.   *   ****. *.. *  *** **   . ********

40              RWKKMYRRYCRWKYWRYAAYCTYCTCATTWCKKRRTAYMSMAGCGYMYYYAGCARWKYTK
51              RATKAYGACTRTKCWRYRRYTCYYCYWCCWYKGRRYWCCGMTATAYMCTYGTTGRWKYAK
                * .* *    * * ***  *  *      * * **    .*:. .**  *.  .****:*

40              CYGCGKRGRYGKACCTYYYYTAAKKYACMYYYWKKRKWCTGKRYYYYRYARKWKWWYKWY
51              YCRMKKARRCRKGATWYYYYYTTKTYMMMYYYWKKRKATCCKRCYYYGCGGKAKAACKAY
                     *  *  *..  **** ::*.*  *********    ** ***  . * *   * *

40              WCMYKRWGRGAYKMWWYSKSKKWRYRKSRGCYYSKRRACAKTKYSRYKYKWARGAKAMRT
51              WMMYKRWRGRCYKAATTSKGKKWRYRKSRKMCTSGRAWMRKYKYSGCGTKTWGKGKWCGY
                * *****   .**    **.*********    * *    * ***    *    .*

40              CASTWYRWKAMMGGARKYRRTGGYTKYKRKYYRRYRYWYRYTACYTACCYYKMRMYWTGC
51              TRCYWYRTGWMMAAGAKYRRCRKYYKYKAKYYGRYRYWYRYWRYCYWYYYTGMRAYWCAA
```

```
        . ***   **... ****   * *** *** ********        *  ** ** ..

40      RWYYMKRKRAWSKTTTKCGRSWWYCGTTCKKRAAAKWKTYGCWRKTWRMTMYTTCAKTWC
51      ATYYAKRKRMTSKCCCGAARCAACGRWYSKKRGWMKWKAYKYAAKKWRMCCTKCTGGYWM
          ** ****  **    ..*.       .***.  ***:*    *.***   .  .  *

40      TCKKYSMMGGACKGYTKRWYAGGYTYACGTCMGGYYKTCWKTWTAYMRKWWKRAARKGYT
51      YMKKTGCMRRWYTACKKRWYGRRCYYCTCWYCKRCCKYGTKCACTYMRTTWTGGRRKRCK
          ** . *    .. .****.    *.         *   *   :***. *. . **  .

40      TKTAGAATCMCATGKYRWMRKKCAKTSRTGCYYYYCYAKKCKKYKKRYKRYTKWTGAYCC
51      GKCRRGRCYMMTATKTRWMGGKTTGYGAAATYYTTTCCGTYKTYGKRYKRCKKTAARCTA
         *   .   * :: * ***  * :  . :. **    . . *.* ***** .* :.   .

40      KKGAYKWGKCAATWRTATAGCGCGTATKRRRWWYYGGGTAAYARATCAWMKTKRMRRGGR
51      TKTWYKARKYMMWTRWTCCAAAMAKMKKGGATATTKKRCTTTGGRYMWACKKTRCRGTRG
        .*  **  *     * : .... .. .*           :: .       *..* *

40      TCGRKMCTKRMKSGTTARYYYAKKRMGATRWKGYKACKKWTKCRYCMYRMMAGCMYAYWT
51      CYRRKMTAKRMKGRYYRACYCWKKRMTGGGTKAYKWYKKACKTRCTMYRMMCKTMYRYTG
           *** :****.      *  **** .   *.**  **  * *  *****.  ** *

40      ATKGKYRKRYWTYCCCRATAACSRRYTWKAYYGTTTKTAGWAWTAAYRGAYMKYATYCTY
51      GWGRGTGKACWYYATTRTCRTYSRRYWWKTYCTAKKKARSAMAATCTARTTMKYCCYAAC
        .      *  * *.  *:  : **** **:*  :..*: .   ::.   : ***. *.:

40      RSKRTTTAMKRYYKYKRCKKTGAWYYGTYCRRTCTAGYKRYMKCTTRRYRRRMTRTTKCK
51      ASTAYWYRMKRYYKYGAMKTCACTCTKYYAARWYCRRYGGCCGYYARRYRRRMYAGAKTK
         *.     *******   *. ..     *. *     *       :*******   :* *

40      YKRRKTKWKGYRSTYTKAYRKTRKYYRMGGTAWRRYRCYKWTKCTTYCAYKRYWKWCGTA
51      YKGGKCKWKSYRSCYAKGTAKWRKCCRCRRWRARRYRYTKTKKGYYYARTTATWGTYRYR
        **  * ***.*** *:*.  * **  *      ****  * .*   *.  .  *

40      TGRRYGYWCRCKKAYTCYYMYYCYYAACWSCYTAGRKTYTACATGCYGKKYKYATAYRMR
51      KRAGCRYWYRYTKRCGAYCCYYYYYCGAWCYYCWARKYCKMMRKSMCTKTTKYRKGYRMR
        .     ** * .*   .*  ** **...*. *  .**  .   ..   *. ** ..****

40      RYRCWRTYKKRGCKYYKAYRYRYSYCYACCKAATAGGTRKWYGYGYRTAKACKYKGCYRM
51      RTATWGCYKKRRMKTYKRTRCGYSYMCRMSGCGWGAACGGACKTATGAGKWYKCKRYCAA
        *   *  ****  * **  *  ***    . .. ...       .  :.*  * *

40      YGAKAAAKYYYCTAMYYMMYSMYKMKWYKMYRTKCCTTTYCGSRWRYKCRATTTYYGTCG
51      CACKTWWKYYCYYRCYYMMYCMYKMKWYKCYAKKMYYYWYYRGATGYGTRMKYWYTKYYA
         ..*:  ***     *****.******** * .*     *  .   *  *  .  *    .

40      AGKYRMGATMYRAARATTAAACCGGAACCRGSMRRTRKACAKYWYAYRKATTYRTYGAWG
51      RKKYGMRMKMTARRGCKYRGMSYATCGSMAAGAAGGRKRYWKYWTRTRKCCKCGWTTWWR
          ** *  .*     .. . . . . ...  ..   **   ***   **. .      *

40      GCKYWCYGCKAMRYYKYYCATAKWYRSWYRYMTYKGGAMAGTKAKYWSTAGAKKMCGCAC
51      KYKYTYCRMKGMRYYKYCCAWWRKAYAGACGYCKCGRSWMTKYKMKYWSYRKRKKMMRTGY
          **     *.*****  .   * * .   * .   . *:  * ****    ***   .
```

```
40          TATAYKGKCCGYKCKRMKCKRKKTTKGTGSCCCYYSRYKYTCCGTKRWRMRRYCYKGSWK
51          KRYRTTATTTCCKGGAAGSTGKKYWTRWSSYMYTTGGTGCCTTAGKRARAAATYYKASWK
            .    ...    *     .. **  .  .*     .       . ** *     **.***

40          RYKGCGTRMYRKKYCRYRYKKMSRYRKMGGTYKCCRSKYCTYRYCGTTKKKCYKKYRYKA
51          RYKTTKGGMYRKKCAGYACKKMSRTGKMSKWTTTGAGKCYYCGCMKWCKKKYTKKTGCKG
            ***     ***** . *  *****  **.   .   .*          ***  **   *.

40          TGKYYWWTGYKRYYMSKRMYRYSKTKRYYRMWRTAKTTAMRACACYKYMTYRACKAKYYR
51          YRKYYWTWKYKAYYMSKRCYRCGKWKACYRMWRCCKCCGCRWYMYCKCCWYRRYKGKCYG
              ****   ** ****** ** .* *  ***** .*  . *     *   **  *.* *

40          WMMGGRKTARRSCAMRTAGKGRATTSMKRRWGKKAMWWYWMRKYRTWRKWKKWGCCKKKK
51          TMMRKRKCGAGGYRMGWGRKRGMKWSMKAGWTKKCCTWTAAATTGATRGWKKWKYMTKKK
             **  ** .  .  *  . *   .  ***  * **.  *    .  : * ****   .***

40          CMRGRMYGTCTRWGCATGRAGKWAYYKMYRMKRMKMRKCRSWKACGTCKCKRTMKYKKWC
51          AMRCGMTRYSGGAAYRCRGWRKWWYYKCTAAKRCKMRKYGCWKCGSGMKTKRGMKCKTWM
            .**  *   .   .       ** ***    ** ****  .**. .  * ** ** *.*

40          YTMMYWYYCGYAGRTAYCRAGRATGATGAAGGWTAKYKCRCTGRGATATWCAGWCYYKRR
51          TYAACAYYARCRARKRCYGGSRTCCRCSRGCAAAWTCKAGYATGATCGYWYWKWYYYKRR
                  **.   .*.    ..*:    . . . : . *.  :  .: . *   * *****

40          RRTCAYTGYGYRYRYYAGGCMKRKMGKYRRGCACATKRGTGTSYKYTCGGKYTMTYRYKK
51          RRGMMCGTTKCRCATCGAAMMKRGCRGYRRRMWYRWKASKKWSCGYKYRRKYGCGCRYKK
            **         *    ... ***    ***      * ..  *  *.   **    ****

40          YYRWRYRRRWAGTGCTKKKRRMCCTKMCGMKGRKMMWYGMYRYACKRRYYRWCRKTGYKT
51          CYAWRYRRAWRRKRYAKKKRRMMTYKCMRMKARKMMAYACYRYCYKRAYTAAMRKCRCKY
             * ***** *  .  :******   *   **.**** *. ***. ** *    **   *

40          KAGKMTMRTMYACGCKCWWSKKRKWYWCKGWCCYMYYAGTWWGKKAYCRSSSCRYMMYTR
51          GWRKCWMRCCCGYRMKYWWSGTRKATTTGSTYYYMCCGRCTAKKGGYGGGGGYGTMMYKA
               *  **   .   * *** .**     .   **  .     * .*  ...   ***.

40          TYMAKTYYYCYTAMWTGGKYTAGTRYRKAMATTAYMRMATRRCGYYARRRRMMRMTARKC
51          YTAWGGYYYSCWWCTCAAKTCRSCACAKGCTCYRCCAMMYRAMSTYWAAAGAMRMYRAKM
                  ***.      ..*   .    *. :      *  *  . *      ***   *

40          KMWKTCATWCKKWRYYMKMMMYRWKKRRRKTKYYTYTKRYWYKMRYSYRYRRWMYYYSGR
51          KAWKKTGKWAKKWRYYMKAMMTRWKKAGGKCKTYCCKKRYWYKMRTGTRCGRWMYYYSKG
            * **. ..*.******** ** ****   * * *  .******** . *  *******

40          YTKKKKCRKKYCTGKGYRAYYRYAKGGKRRGWKWKKYYMTRKAYRTWMKYYRTTMRRKGK
51          YAGKKKTRKKYAYRTAYRMTCGYGKATTAARWGWKGCTMYGGRYRGAMKYYGYCMGRKRG
            *: *** ****.  ..**    *.*. .   * **   *    **  ****   * **

40          YKTAKRYKAKYKWYMYYMKRMRRKGRGCRYGKYMYWGAARYRACKTGKCKGAATAGKATT
51          YGWWTGYTMTYKTTATCCKRMAGKKRRYRYAGYMYWKRWRYRRMKARKTKRRWWRTKWCA
            *   . *. .**      ***  * *  **. ****   ***  *: * *      *  :
```

```
40              CKGSYKRSKTKCCSRKSRCKCYSYWYCAAKSRWMAGCTYKYYYAYKYGAKGRKWRAGGKT
51              TKTSYKRSKKKTYSRKCATKTCSYWYYRMKSRWMRSMAYKYYYGYKYKMKKRGTGRRKKY
                 * ******.*  ***.  *  ****   ***** . :*****.***  * *      *

40              CKWKYMGCATCAKTGGAGKCAATCCTGRCARSYKYWYYAYRWKCKWWRRMACKRYKTCTY
51              TKTKCARGRCMGKWCACAKGTGCMYKRRYRAGTTTATCWCGAGYKWWRRMWMKRYKCACC
                 * *       .*  ...* :.   . *   . .          ******  **** .

40              WYRRTWTRRGTWKYYYRYRKKYRKSTSRKRRRRKRYYTKCYMKRTRMKMMYKTMGYTKTR
51              WYRGCAWRGRWWGYYYGYGKKTAKCGCRKGAGGKRCYYKYYMGAKRMKAMYKYCRCKKCA
                ***    *   * *** * **  *. .**    ** * * **  .*** ***    .*

40              WKCAKGGTYCGGTRRWRMCGTTTWKTKACYRCYCMGRCTCAGATYCMYKWYRMYRKRRT
51              WGMWKRAYCTACKAGWRCGMKGYGTKAKMAYGTCTCAGAAMMRGWTTATGWYRMYRKRRC
                *   * .   . .  **        *:* .*     . .:   .      *********

40              AYMRYKATCTGMGGCWAATTGAACRCRRWKSRRCCWGGRWKYWTGTTTGAAKTTATKMGG
51              MTAGCTCATATMKAYTWWGGRCTARYARWKGGRMYWRRRAGYWYRYAARWRKCCRYGARK
                     ..: : * .       .:.*  ***. *  *  *  **   ::   *

40              CGKGAGYKRKYTRRATCCYMTYTKTAYGWMTGRYYRWWGWYTAMGRRKKACWRYCAWWGR
51              MTKKGAYKRGCYGRRYYYYAWTYTAGTRTAGTGYYRTAKACCRACARKGRYARYYRWAKG
                  * ..***    *    *    .:.       ***          **    **  *

40              KKCYWRKCASGTKRYYGRCARGTMTATTATYAKCGGAACWWRSYARAGYKYMGGWCKCCA
51              KKYYWRKTTCRWKGYYKRMRGRCACGACGCYGGYARTCYWWGGYMRMKYTCCATWMTYMR
                ** **** :.  * ** *       .: . *.  . :. ** .* *  *.  . * .

40              RTGAKKTGTTCYRWRWCKAGTRKKMWATAATCAAGCCGMYYYMWGSARTGYTYARYYYCC
51              GGAGKGYKKYACGTRTTKGKCATGCTCWMRGMGGAYMTMYYYMWRSCGCRTCCCRYYYTA
                  ..*   . .   *  *.   .   .     ...   ****** *.      .**** .

40              YKRYRYSWRRRYCGGCYYMYGTRYWAWYRGGRCCGYAKTAAMSRMKWTAARRRYYTRMKC
51              YKRYRYSWRRRTYRAMYYMCKGATTRWTGARGMAAYTKCGTMCRCTTACGRRRYTCRMGA
                ***********   . ***       *  .   ..*:* .:*.* . :..****  **  .

40              RYKYYMRYGRKWMKRCMYMYMRKTRWTWTTCKRYKAMCAYACYGYYRRTKKRGRCKKGTS
51              AYKYYARYKATACKRGMCMYMRKCAACAGAYKRYKWMMRTGACCYYRRKKKGAGYKGCAS
                 **** **  . ** * *****      : **** *   ..  ****.** . *   :*

40              KRYKYKGRYMMMAKRKWSKYACCCGSWCGRSKTGTATCKAWYRYRTKSCMGGYRRYRCAC
51              KRYKCGKRYACCGKGKAGTYGYMGACTMKRSKCRYRWSGMATGYRKGCACRKYRRYRYRY
                ****   **   .* * ..*.   ..   ***     .     **. ..    *****

40              KYCGYRGAGAYYSTCYYCKGWGWKYYKYRYYATMTAGAGRTACKCCYRKYMCYWKWTGYK
51              KTYRYRKRKMTYSYYYYSTKTRTGTTKCGCCRKAWTKCKAYRYKYYYRKYMSTTTTCSCK
                *   **     **  **..       *     .  : .     *  *****.  .  . *

40              AWCKCWATTKKGGWAKCGRATGWCTGRAATTRCWYRGCCAGYKCAKACACCWMYRRTKCA
51              RWYKYWTYKKKARARKYKRCCRWYYRRRRKWRYAYRKYTGRCKTGKCYWYYTCCAAWKTG
                 * * *: .**.   *  *.  *   *  . *  **   .  * .*.          * .

40              RYRKGWRMYAYCKKYKRMYCKKRKGTATAKRAWCRKAWGTYRYMRGCKWGWYARGGKYGK
```

```
51      RYRGAWRMYRYTKKYKRMYAKKGKTACCMKAGTSGKRWRAYRYMARMKAKWYRGKKKYRK
        *** .**** * *******.** * :.  * . . * * :****   *  **    ** *

40      AGGTKTTCTGRYKKKSAWRKKCKARRWTGGTAAACTYCRCTMTCKKAKGWYKRCCMWCRR
51      RKRAKCAAGCRYTKKGGWRGTMKCGGWARAYMRRYYCMGTKAAYKKWKKTYKRYMMAYAA
           :* :.  **.**..** . *.  *: .          . : ** *  ***  *

40      WKWYMAAGGKACKYRKMKKAKGCRGTCRWRCCRCTGGTYYKWWRKKKKKKTGKYRMCAMR
51      WTTTMTGTAKGTKYRKMKKTGAMRAATGTAAYGYATAYYYGWWRKKKKKKCKTCGMATCA
        *.  *:. .*. *******: . *.:    .   : . ** *********  .  *.:

40      MYYMRWTAGTKGKTAGAKYYMYYSRRTTGKAKKYGYMRCKMCGYKYRRYRKSMTTTCAMK
51      MYYAGWYGKWKKKWRRTKYYMYYSRRWYRKWTKYRYMAMTMTKYGCGRCGGGCYYWYWCT
        ***  * .  * *   :*********   * .** **  .*  *   *   .       .

40      AWARKAACGTGAYCTCTYYKYYKCCCRKGMARYRGTGYWMSYYMSTKRWARYMWYTGTTT
51      RWMGKCMYRWAGYSYYWCTKYTKTAMRKKMTRCGRGRYWCSYYMGCKRWRGYAATATCGC
         *  *.    ..*.     ** * . ** *:*     ** ****. ***  *   :

40      YRRACTATGAKAKKMKCATRAGTWKYKSAATTRCCGTCCACTCTCGYGTGKGTSYKTACC
51      YGGRYYGCAGKWKKCGAGAGGAYWKTKGCGGWRMYAKTGMMYTAAKCAAATKYSYKYRYT
        *     . ..* **  ..: .. ** *...  *  ..      :.  .:..  ***

40      ATWCWCATTCTMWACMCWWSYTMKTCYRMRTWGCCCYMKMWACKWTWKMKKTKAAAAGGA
51      CATYTYGYKYYMWWMMMWWSTCMKYSYRAACATTTACMKATWYGWWWKCKKWKCGGRKTT
        .:    . .  **  * ***  ** .**       . **     * ** ** *...   :

40      YKARGMYRTATRMRRCKTRCWKTCCMSKYGMSWCTRYRAAGTAAAWCCCRTTCKKRKSKG
51      YKMARCCAWRKRMRRTKAATWKWYMCSKYRASWYCAYRGTAWGGGAYTAAACAKKRTCTS
        **        .**** *:  **    ***  **   **.:. ...   . : .***....

40      CGAARTRTGKRKMTAACTGMRWYYSCGGCRTCMACATATAKKYYTCCCCCGYGYWWGTYK
51      YKCTRARYRGRKCYCGYCRMRWYTSGKKMAYYMGMMWGCGKKYYCTATMGAYKYWWRYYT
          .:*:*   **  ..   **** *       *.   . .****  .   .* ***  *.

40      YRAYAGMGTKKTYKYRCKYAMMYKRKTYCKKAAYKMYCCTCCKYYWRYTTRRCYYCCWMR
51      YRRYCAATYGKKYKCAYKYTMMYKRTYYMKGWRYKACSYWMYKCYWRYCYGGYCTTTACR
        ** *..    *.**   **:*****. * *   **  .    * ****           *

40      AKWSAKRYYYKKRCWRGYYCAYMMCATKTYSRRKRKKMMAGAAKCYGKGGRSYWKY
51      GGWSRKACTYKKAYWRRYCYWCAAYTAKWYSRRKAKGCMRRMRKMTAKRKRSCAKY
        . ** *   ***  ** *       ::* ***** *  *    *  .*  **  **
```

The pairwise alignments of two accessions 42 and 43

```
43                 RRRMRRKRCRTGGRWRKYRGYWYWKMCWMCGRGKKRKWYRWWMKRTYWKWKGCCYRYYYW
42                 RRRMRGKRMGYACRWRKYRAYWTATCYACYSRRKKRKWCAWWMKRAYWKWGKYTYRYYYW
                   ***** **    .******.**  .     .* *****  *****:****    ******

43                 SWWCYYWTCTGYKYRRYRRMGYKKRWRYWMYWYTCCWYKCTRYWKSYYKRGWKRWYYRWW
42                 SWWMYYAYMWTYKYRRTRRMACGKRWGCWMCWYKMTAYKSYRYWKSTYKARWKRWYYRWW
                   *** **     ***** ***.  ***  ** **.   **. ***** **  *********

43                 RRRKYRKMWRWKKYYRKYWRYMRCCTTYCRRTAKYTYRKRMMGMSCCCKTATYSRYYYRM
42                 RRRKYRKMWRWKKCCRTTAGYMRYYYWCARGYRKTYYRKRMMAMCMTYKGTAYSRYYYRM
                   *************  *.   ***     .*   *  ******.*.   * ::********

43                 WAAWRMRRKMCYRRMSKMYMYRKWCYARMRYYTMKMYYRKWRWWARAWMRRYSRWMMMGW
42                 WGGTRAARKMYYRGACKMYMYRKWYYTRMGYYGMKCCYRKWRWWWRRTCARYSRWMMMAA
                   *.. *  *** **  .******** *:** ** **  ******* *    ********.

43                 YRYRYYRRKTWKMCRGACAYKACKTCGKKYTKACKCTAKWYWAKRWRCRTGWRRMCWRYK
42                 CRCRYYRRKYWKCYGAGTGYKMYKYMRKKYKTWYGYKRKWYTRKRWRYRYAARRMYTRYK
                    * ****** **   .. .**  *   ***..    . ***  **** * . ***  ***

43                 TRMMRWYGAMMYYGYTTAKRGMCKRRMRYKARYYYYRKCCCKRYAGASMMKWYKRRKCAM
42                 WRMMRWCKRMMCTRYYKRKRRCTKRRMRYKWRTYYYRKYYYKRYTAGSMMKWYKRRKMCC
                    *****   **   *  . **   ******* * *****   ***:..********** .

43                 CYYGMARSKKGTWKWGRWWMYRARAKGYYGRCKYARYYGKKRMMKKRCKYTCWWYYGYCW
42                 YCTTCWRSKTCWWKWKRWWMYRGRWKRYYKGYKTWRYTKKKRAMKKRYKYYMWWYYRYYW
                         ***.  *** ******.* * **   *  **  *** **** **  **** * *

43                 KRGRRRKKYGTKTCARKAAGARKRRRRYTGYMMYAATTATKRMKRRGCRKTCCKGKTKSK
42                 TRRRRRKKYSKKCTRGKRRRRRKRGGGCGAYMMYRRYYWYKRMKRRRYRKKSMKRKWKSK
                   .* ******..*    *    ***     .****      ******  **.. * * ***

43                 KCAAKRCGACARKMATGAGRATTMTCRYRYRMKCRCMTMGKYRKKYKKYKYTMRRYKKCA
42                 KTGGKGTAWGRRKMMCAGRGTKKMWYGCRYGMKYGYMAMRKYRGKYKGYKYWMRRYKGMG
                   * ..*  .   ***  ..  :..*    ** **   *:* *** *** *** *****  .

43                 AYYGCRMRYKYYKKKKKRYKKSSCYGGWYWMKCGRSYGSYMMMKKYYRWYACRYKRAMYK
42                 CTYRAACAYKYYKKGKKRYKKSSYYRKWYWMKTARSYACYMMMKKYYRWYGYRYKRMMCK
                   . * .   ****** ******** *  ***** .***..***********. **** * *

43                 KRYGATYMGYKWARCMKYKAGMMKCYTKRSRKCMCAYGGRARYMTRAACCKATTAWTTAT
42                 KRYTGAYMRYKTRGYMKTKTKCMKMYKKRSRKYMYRYKRRWGTCCAMRYMKCCCGWCCCC
                   *** .:** **    ** *:  ** *.***** *  *  *          *.  .*  .

43                 RRWMYAYMKTTAAYRKKKTAAGWKGTRRKKRKKYCKKMYKCYCRRMRYRMKCTCKTCCCT
42                 GRWMCRYMKKWRRYRKKKCRWRWKRYRRKTRKKYTKKMYKYTMRRMRTGCKYCYGYMAYA
                    ***  ***.   *****    **  ***.**** *****   ****   *      . :

43                 RRCGGAKSRKCGGGKYKYGGCTAAKAGKRRWRCGGRTKMGRKKRYWCYCRKKYYWKYTKR
42                 RRSRRRGSRGYKAAKTKYRSSGGCKGKKRRWRYTKGYKMRRKKRYAYYYRKGYYWKYYKR
```

```
            **.    **   ..* ** .. ..*. *****     ** *****  * ** ***** **

43          YTSAKKTKKYATKACKKMKKRWMAGGKKGTMYRKYKKAAGCCYYSYWKKATGSRKARKKY
42          YYSMKKYKKCGAKMMKKMKKRWMWTKKKKKMYRKYKKMRRYYYYSYWKKWWRSRKRRKKY
            * * ** ** .:*  ********   ** .*******     *******   *** ****

43          YKKKYTRCYKKKTRYGTTKYWTTAYMCAACKCRRGWKYKKYRYYTRRYRMMYYATWKRKG
42          YKKKYYRTYKGKGAYRYYTCWYKMCCSRMYKYRRKATYKKYRYYWRRYRMMYYRYWKGGR
            ***** * ** *  *   . * .   .   * **  .******* ********  **

43          WRCTTKRYYYYYAGGTKAKCTKYYYRKYCTCYYKAAKYYWYSRSYWYAAYRRRWYMAMYG
42          WRACGTACYYYYWRRWGRKMYTYYYGKCAYMCYKRRGYYWYCRSYWYMWYRRAATMMMYR
            **.  .  ****      *  .*** * .   **   ****.*****  ***   * **

43          AKGWTTMRSCRWRYCWRWRYWCAGMRRYRYMARYGKYWKRTGTGAAKYWATAMYYKRCTT
42          WKRWYKMRSMGWRYYWRWRCWYRRMRRYRYMRGCRKYWKRKKYRMRKYWCWMMYYKRMGC
             * * .***  *** **** *   *******    *****.     ***.  *****

43          AARMRRTYTKGGGKAKGKKGGTCYTYCRYGYGATCGCTMYRCGAGCTTTAARRGKYRRKR
42          CWRMRGCYKKRSAKRGRKKACAATCCGGYRYAGGYAYYCYRYRRRYKKYGMRGRKYGGKR
            . ***  *.* ..*   **. :.     * *..  .   **     .. . *  **  **

43          KKRYYCMTWTYKKAAAAKGWYCTRRYMATRCYGWKYRMTGAYTRRKYKMKKWRTKKYAAG
42          GTGYYYCCWKYKKMRRWKKWYACRGTCCYGTTAAKYRMYAGCWAGKYKMKKWGAKKYCGT
             . **   *.***    * **. *   .    . **** ..    ******* :***..

43          AAACGMYYYAASRRGAYSYYRGAGTGTTASWYRAATYRYRKMMWYYRRMKYCYYKYKMYK
42          TGRMSMYYYRWSGARRCCCCGRWRGCWCTSWYRWMYTATAKMMWCCRRMKYYTTGTKMYK
            :.  .****  *     .          :****       ****  *****     ****

43          GAMKTKTYGYYKMRGAYYGKSCRSMGYSYKKKKKSCCRWRRRKRMMRYMKCCTKKGCCWR
42          KCMKCKCYRYYKCGCCYCAKSSRSMSYSTKGKKKCMYGAGRRKRMMRTCKYYKKKKMYWR
             .** * * ***   .* .**.***.** * ***.     *******  *  .**   **

43          MCKWKWKCGCAKYYYTGGWYKMGYTKYGKKRKYSSCKYTKYKKTGTYGYSRYTGCAKKMY
42          MYTWKWKYRYMKYYYKKRWYKMAYYKYRKKRGYSSMKYATYTKYRCYSYSRYYRMCKKMY
            * .****    ****.  ****.* ** *** *** **:.*.*   *.****   .****

43          WTCAARKACTRRRSAACKGRYARMYMKCACAKAAYYYAYWTMCRKRYCKRMYGRYCRMRA
42          WWMWMRKRMWRRRSGGAKRGCCRMYMKMTTTKRRYYYWYWGCYGGRCTKRMYRRYYRCGM
            *    **   ****...*   .***** : :*  *** **     *  **** ** *

43          KYWTRGAKMMYTYTKGYMKKTTGAWGYYRMGGMKRGWKKRYRYYYYMWYWKKKCTGWMYG
42          GYWYRRRKCMYWCWKKYMKKYYRWWKYYRCRRMKRKWGKRYRYYYCAATWTTKTCAWMYK
             ** *  * **   * ****    * ***   *** * *******    *..*  .***

43          GATTYYYTRYYKMKYAMYYYTYYMRKYKKMRGWMKKRKRTATTMRCWYKYMMKYYTMYRY
42          TGAAYYYGACYKMKYRMYYYYYTMRKYKKMGTTMKKRKRYMYCMRYWYKYMMKYTYAYRY
             .::***   ***** **** * *******   ******    ** ********   ***

43          MYTCCARKRYWKMYYYWGKWKRATTKKKRKTWMGGRKWRYRKKGYRYKARMRAYTYKKCC
42          MYWMMRRKRYAKCYYYTAKWKAMWYKKKRGCWMSRRKWRYRKKSYRTKWGARMYCCTKYY
            **    **** * *** .***    ****  **. ********.** *   * *  .*
```

```
43          TKGCGWGGYGCKKRGGMGWRSKWMGATMYCCYRTTYRYRKKRCTARRRGGRRCYCCYYYK
42          YKKYRWSRCRTKKAKAAAWRSKWCRGGCCTTYRAKYRYRKKRTCGRRRRKGRGCYTYYYK
             *   *.    **  . .*****  .     **:.*******  .***   *    ****

43          RRWYRKAGKCRCTYGTGACKRMAARWRMCRRAKYWGYYRYMCGTRCAWKACTCWYRRCAY
42          RRWYRKRRKYRYYTAYRMMKRMWRRWRMTAATKYWKYYRYMYRYRYWTGTAYYWYRRGRY
            ******  * *   .    ***  ****   :*** *****   *    :.  ****  *

43          TGKKTAKYKTCGCRCAGTTAMMGATKYCTAYSGTAAAAKRWKYTWCCGYKRAAAYCCAAR
42          YRKTWRKTKYMRYRMMRYYRMMKWYKYYGMYSCGCTCRGRWKYCATTAYKRMGWYYYRRR
              *.  * *    *      **   **   **  .:.  ****    .*** . *    *

43          YSYYAYKAGGAYYRKYKKCYYMMKRYCTTTKGAYYYASRYKYGKMYRGYKYRMCRWAYAR
42          YSYYMTTMRKRYYRKYKGTYCMMKRYYYYWKSRYCCGGRYKYRKMYRKYKYRMSRWRYWR
            ****  .    ****** * *****    *. *  ..**** **** *****.** * *

43          RYKKRCTCACSGCKRTWWTKMTATAKAATRKTCTTGCWRYMMTKMRRWGCGCGWYMCGRW
42          RYKKGYYYWSGCTKRYAWCKMWWKWGCTYRKYYYYKYWRYMCKKMRRWKMRTAWTAGRRW
            ****     ..  **  * **  .  .: **      **** .*****    .*    **

43          KCWMKAARCMMKKKMWMRTKMRGKGWRGKGAATKYTKYRMRKCRMRMRRWYKRKCGMATY
42          KYWMKRRRYMMGKKMWMRYGMRRKRARRKRMWKKYWKYAMGKYRMRMRRWYKRGAAAMYC
            * ***  * ** ******  ** *  * *   .** ** * * ********** ..

43          TTKAGYYKYKTWKKKYKWRMKYYWMMWWARRYAGMKTRTGKTRYWYMTCKCRRRMYCGAA
42          CYKTKYYKYKKWKKKYKWRMKTYWMMWWTRRYCTAKKRYKKKRYWYMWYKMRRRMYYKRR
              *: *****.********** ******:***.  *.*  *.*****  * *****

43          KARKKYYYYRRKMKWGKRCRAKRCMYCAKWAKCGTKRKGWRGGWRSYGKYKRKKTTGAYY
42          KGRKKCYCYRRKMKTRGGGRGKRYMYTMKWCKYTGKRKKWGKRWRGTRKYKRKGYYRRTT
            *.*** * ******     *.** **  **.*   *** *   **.  *****

43          YCTKARKAYRGKTYGKTMKKYKRAAAAWRYTYKYKTCACACYCAMMMRTYYCRYCTGYWG
42          YYYKCGTTCGKKYYKKCCTKYKRRRRRWRYKYKYKCGGYCMYSRCAMRYYYMRCYKRYWK
            *  *. .:   * * *  .****    ***.****  . . *.   ** ** *  . **

43          TGYRAKAWKATYMYWRTKRYTWMCRMYSYMTKCTAKMTYMYGYWRMSRCKKCTAWKCGRM
42          YRTRMKTWKRYCMCWRKKRYAWMYRMYSYMYKYYRKCGYMCKYTRCGAAKKYYMWKYRRM
               * *:**   * **.***:** ****** *   *  **  * * . .**   **  **

43          TAGAATAYYRWYWYTYKWGCGATYTAYMYKSMRAAYRKWCTCAGYKKGTRYKRKGTTMAT
42          KRSMCYRTYRWYACYCKAAGRRKYKRYMYKSMRRMYGGAYYYRKYTGKYRYKRKRKYCRY
            . . .   ****    * .   .*. *******  *        *.   ***** .

43          TGKAWWCMMYATSTKYMKMGGKMCCKCYMRYYTAATYYAGACCYYCYRGRMGMRAYYYRY
42          YKKWWWMAMYCKCKKYMKMKKKMYMKMCMRYYKMMKYYRRCYYYYYCRRRMKMRRYYYRY
              * **  **....*****  **  *  ****.  .**  .  **  * ** ** *****

43          TMMKYCGGACSKAWRYRGGWWACRCYKMKWWMKAAATKYMRWRRKATTTKYGTKKKCGRW
42          KMMKCMSRRYSKMWRYRRAWWGYRYTTATWWMKGMRYKYMRWRRKRWWYKYACKKKMRRW
            .***  .   ** **** .**. *  . .****.   ********    **. ***  **
```

```
43                 MRGGSSMKAKRAATTAMYWGCCACKRGGGAWCCTRKGCGTKAKMMGKYKCYYKKTCRRKY
42                 CRRRSSMKMKRRMYYRMYWAMYGYTGAATGWMYKGKKAKAKRKCCRKYGMYYKGKYRRTY
                    *  **** **     ***.  . . .. .*  . * . :* *   **  *** . **.*

43                 GCRYCYKMYGAKGWRGRYKKRKTAYYAGKYMRRRCYTRYTRTCMRKKKYKTTYRRRRSWR
42                 KYGYYYKMYRRKRAGRRCKKRKKRYYRKKYMRRRMCWRYKRYMMRGKKYKYKYRRRRSTR
                      * ****  *    * ****. **  ******   **.*  ** **** .****** *

43                 YARTKKYKYTWYMGGYRYCAGYWMTGCRAWYGTKMKRKKATCCKTYTRMYYWKKKATSYR
42                 YTRWKKYGYKTYMRRYGCYRKTTCYRMRMWYRYKMKRKKWWYSKYCWRMYYWKKKRWSYG
                   *:* *** *. **  *           * **  ******   .*   ********  **

43                 CKKRKKGYKYRC
42                 MKKRKKKYKYRY
                    ***** ****
```

The pairwise alignments of two accessions 42 and 44

```
42              AMMYYCCAWYRYWGCYWKKYSYKYKTMSYAYWKCRRMRMYKRYKMMKRMWTRRWWKYMRK
44              MMAYYTTGWYRYWTMYWKKCSCGTKWMSYMYWKYRRMRMYKRYTAATGATYGRATKCAAG
                 * **  .*****  **** *   * *** *** *********.  .     *  *

42              GGGWYRYKKSRKYRKKSRRRRMYWKYYWWWYRYSKWWCMMKYKYKYYRRMKYKSYMRYKK
44              RARTYGTKKSGTTGKKGGAGRMYTKYYWAWYRYSKTTYCCTCKCKCTRRCTYKSYMRYKK
                 .  *  *** .  **.   *** **** ******     . * *  ** .*********

42              RWYTKRYYKRMRCARYYYYRRRRTRSTYRYWYRCRRRYYYYYGYGMMKRYYYYMYKYGRM
44              RWCAKAYTKAMRMRRYYYYRRARKRGWYRYWYRYRRRYYCCYRYRCMKRYYYYMYKYKAC
                ** :* * * **  ******* *.*. ****** *****  * *  ***********

42              RYKWGYMMWRMGYRKMYWRCGYYRKYKCTKYKRYRWRYWRKWYKKRGWRTYYRYKRYSSG
44              RYKWRYMMWRMRCRKCTWRAKCCRKTTSWKYKRYATGCAAKWYGKRRAGKYYRYKRYSST
                **** ******  **  **.   ** .. *****      *** **   .*********

42              YYWWRYCAYYYWYWRKMMRCWRMKYMYTTYMYRAKATTGRRRMCRGMYMRWKKWRYRWKC
44              YYTWATGGCTCWYWRKMMRMWRCKYMYCGYMYRWKTCCRRRAMTRKMYMRWKKWRYRTKT
                ** *   .   ******** ** ****  **** *:   ** * * *********** *

42              YGCTTYYKRWWWYKWRMRRRRWWMYYTRCKRMKRRKYKRCMRWWWWRRMYMMCGYTYWYC
44              CTAKYYYKRWWWYGTRMRRRGAWMYYWRMKGAGRRKYKRMCAWWWWGGMYMMTTCKYWTY
                  .. ********  *****  **** * *   ******   ****  ****   .**

42              MRGWYGYYWAACCKRCYGGTWKRWTMGGWGTRRRMSYMRRYYMYMGCGATTRKRCKKRRR
44              MRRWYSYYATMYTKRMYKRCAKRWGARRWKCGRRMSYMARYYMYMATARWYGGRAGGRRG
                ** **.** :   ** *    ***    *   ****** ******. .     *.  **

42              RKYRRYYRKYMYWYYGGAAACRYKAMSRWYCCCCMCCWYYGKMCRARMYYAKMYRYRYRW
44              AKYGRYYGKYMYWYCTTTGRMRCKRMSAWTTYATMATTYTATMSGTGCTCGGMYRCRYRW
                 ** *** ******   :.  * * ** *   . *.  * ..*. :    . *** ****

42              MMWWWKMKRYCWKWKKKWMTTGKYMKYGSRMKYCGCWTAGRRAYYKYYRRKRRKTWGYKM
44              MMWWTKAKRYYWKWKKKWCGCKKTCKYASRMKYTATWYWKGRCTYGCTRAKRRKKTKYKC
                **** * *** *******    *  **.***** . *    *. *   * ****.  **

42              GTKKWKYRRRWKYRYKRKAYGGCCGGGGYMSKYYRRAYWTGGYYTAKSRKCYMRKYKYCT
44              ACKTWKCAAGWKYRYKRKMYKRYTTARKYCSKYTARWTTCAAYYKRKSRTMYAATYKYGY
                . *.**    ******** *     .  * ***  *    ..**. ***. *  .***

42              YCCYRMYGYSYKMRCGCMCGCCKTARMRRRMYYCRCCTYGGCMKGYYWYRKTYRYMRKRG
44              CMYYGCCKYSTKCRYRSAYRTAKAGRMGRRCCCTRTTCTRRMMKRYYAYAKCYRTCGKRK
                   *    ** * *  .    .*:.** **    *       ** ** * * **   **

42              TAKRRTKYKYYRKWYYWTYRKRCTCMGKKRKYYGKYRGRGGTYRMKSRCTYRAYYRWYTG
44              CGKRRWKYKYYRKWYYWYYRKATGTCKKKRKYYKKYRCGTTAYRMKSRAATAWCTGWYAK
                 .*** *********** ***      ****** ***    :******.:      **:

42              MAYYYGYYKCRMGRTKTRRRKKYKMKMYYRKYKMMYWKAATKRYWGGYYWKRRKMGRTKY
44              MGTCCKYCKYRAKRAKCGRRKKYKCKMYYRGCKMCYWKWTGKGYTRRYYWKRRGCCAWKC
```

```
      *.    * * *  *:*  ****** *****  ** *** : * *   ******     *

42    YMARRTYRMRYGRRCAAMAGTGTTARTRYRYRWYYGCTYKSRKTYTCKRMMWYYWMAAWY
44    YCRGGCYRMRCAGGMWRMWSYKYWRRWGYRYRWYYRMCCKSRKCYKYTRMMWYYWMCGAY
      *     **** .     * .     *  *******    **** *. .********.. *

42    KMMYRWYWMRYYRAACWWMGSKGKRWMRKYWWYYKTAYKKAYTKKYRYYYKRYKKYYMWG
44    KMMCAAYWMRYYRTTAWWMAGTKGRWCRKYWWYYKWCYKKWTWKGYRYYYKRCTTCCCWA
      ***   *******::.***...  ** ******** .***   * ******* ..   *.

42    ATRGAMKRTGWKMYWYYCMKYTGAAWYCRYTKWSWYRYKKWWKRKYMTCTRRKWRKCRCC
44    RGRAWMKRAATKAYWYYYMKTGACTTYSRCYKWSWYRYKKTTKRKYMYYYRRKTAKTGYA
        *. ***:. * **** **  ..: *.*  *********  *****   ***  *   .

42    ACCTGCCCGTWRWKWRKYWYRAKKRSYKKWKRKRRMKKCYYYKTCWKKKRRKYWWKYART
44    RYYYKYMMKWWRWKWRKYTCRGKKRGTGKWKRKRGCGKYYYTKYYWKKKRRKYAAGTWGW
                ********  *.***.  ******   * ** *  ********

42    CCCKYWYRYMRRCMYRRRRKWRSCRRWRKTGRTTGCCRWYRRYMGKRYTCGRYRYKYCRK
44    SMYKYWCGCAGAYCCRRRRTWRCSAGTRKWRGKCCAMRWYRRYMRKRCKATRYRYKYYGG
      .  ***         ****.**..   **   .  . ******* ** .. ******

42    CCTGTYCYKYGRWGKMKMKAATAKRTTRKKYMYRMRRKYKRSYKWRYYRKKGKYYAGWTY
44    AMYAAYTYKYRRTKTMKCKGCCMTAYWRTTYMYGCGAKYKRSYKTACYRTKKKYCGRACY
      .  .:* *** *  .** *.. .   *..***    *******   **.* ** .   *

42    YRARYMKAKMKMYRWKYRRWRCGMKMKRYWWMRCAKKCATTRAYGAKRGRMRKMTYTCCM
44    YRRRYMKWKMKMYRWKTRRWAYKCKMKRYWTARYRTGTCCARWCRWTAARMAKMYTCYTC
      ** **** ******** ***    ******  *  .  . :*    . .** **

42    YYRCKKKWYWYRKWKYKKRRAKYKMRYYRWYYSCYKMWRCYYKRYYYMMRWYRRWWRYKK
44    YYRYKKGACACRKWKYKKGGRKYGCRYYRWYTGSCTCWRYTYKRYYYMMRWYRRWWRCKK
      *** **     *******   **  ****** .. . **  **************** **

42    KKCSYRYRRRTYGATRMRKKYYGYYMTKCAAYMRKYKYKCMKMYYYRMKTTACYTTRWKW
44    GKYSYRYRRRACKRYGMGKKYYRYCMCKSRRCAGKCKCKTMKCYYTAMKAYMSYKARWKW
       * *******:     * **** * * *.     * * * ** **  **:  .*.:****

42    MCAGKWRKYMACWTTARATTYTYAKAKRKCSYCATTRAGGKGYWACWKKARYKMYKKYGW
44    MTRRKAGTCCGTWACRAGYKYKCMKGKGKTGYYRYYGCKATACTGTAKKCRYKCCTKYKW
      *   *  .  . *:   . .*.  *.* * .*     . ...  .  **.***  .** *

42    WYKKWCRSKYRYMRYYYRSKTYMRKCKKYCYRKWRKKWKRYRYWYKRMYMKTKKKRYYWK
44    WYKKAARSGTGCMGYYYRSKKCMRKTKKCYYRKTRKKTGRYRYACKRMCCKCKGKRYYWK
      **** .**    * ******. *** **  *** ***  ****  ***  * * ******

42    SMYYRCKRKGTCMKGCCCKGKRCMRRKRRYSTTARTKTKKKTGACRGGGGTRTGMCSWMR
44    GMYYAYKAKKWYMKTATAKRGAYAGAGGGYSKYRACGAGKGGRGTGRRRKYAWRMMSTMR
      .***  * *   ** . .*          **.     : *   .          * * **

42    YACYRWYCGAGTTKWYYYRKWGATTTYRMYRRAKKKRAAKYSTWKKMMGATTTRRRYKYA
44    YRYYRWYMKWAKYKWYTCATAKRYCYYRCTGGMKKKRGGKYSKWTKMMKCCCGRRRYKCW
      *  ****   .. ***   .      **     ****..***.*.***  .   *****
```

```
42      GRMYYRTKGTRYKKMATTTWCTKAAYKYRRRYWWAATYRWKRWGMGYYKTGTRGKGARYC
44      RAATTRWTKYATGGCTGWCTTCGGTCKCAARYWACCYYRTGAWKCRYTKWACAAKRRRYT
             * .       :       .: *   *** .. **   *   * * .  .*  **

42      RTYYRYCAKWSKTAYWSRRYRRRKMWCCRYRKYAYMKRCTRRRRWSGYKRTCAATTKKYR
44      GYYTRYYGKWSKGTYWCGGCGRGGMTATRYGKTWYMKGTCGRRRWSCYKRWTTMCCTGYG
          * ** .**** :**.    *  * . ** *  ***    ***** ***  :   . *

42      YMCTWYTGRRYWWGATTRTRCTARWATCTTKRYYGGKGGCYMGTCKRATRKYRTRACGWY
44      YMTCTTARRRYWWAGCAAGRTACRWRWYYKKAYYRAKRRYYMKYMGGRKGGTRKAWMRWY
        **    : *****.. :  * :.**    .* ** .*   **      .   *.    **

42      YRKCKYYYRKMWYWGYWYRSYMYYRYKWARMMKTRWWRWRMKGGCKKMWWWYKYYMRYYK
44      YRKSGYYYRKMWYWAYWYRCCCTTGYKTMRACKGRWWRWRMKKKYKKMWWWYKYYMRYYK
        ***. *********.****.     **  *  * ********   ***************

42      RRWRTCGKKKKMWYRMRGRYYRKRCRKYWGKSYWCYWRRYYMMYKCRGKRKKMYMAKTSK
44      RRWRGYKKKKKAWYRARKRYCRKRMRKYAKTCYTTYTRRYYMMYKYRRKRKKMYMCKWSK
        ****   **** *** * ** *** ***  ..*  * ******** * *******.* **

42      KWRWMWTWRRRKTTTTWYSYRYKWYRKSRKRKYRGCYKRRYRKKKRRRYKMGYWTCCTRT
44      KWAACAWWRRRKYYYGTYSTRCGTYRKSRKRKYRKYYKRRYRGKKAAGYGMKYWYGTARC
        **     *****     ** *   **********  ****** **   * * **   :*

42      YYSKRCKYAWKKYRMRSGYYKMWYYKKYYGRTAARYKYMRYKKSRATKKYKYKRYRYYKK
44      YYSKAYKTMTGKYRMRSRYYKCAYYKKTCTRATWRYKYMACKGGGMKKKYKYKRYRYCKK
        ****  *    ****** ***  ****   *:: *****  * .  .********** **

42      RRRYWYWKTYMTRKGAGGRCYYKRRYMYTWRCKRGYKMKYWKSKKRYWCKCTRKGYYKYK
44      AARYWYTGCYMYRKKMKRAYYYKARTAYKTAYKRSYKMKYAKSKKRCWGKGGGKKYYKYG
          ****   ** **      *** *  *.   **.***** ***** * *   * ****

42      RYRMKKKWRRRKKYKATGWKWWYMGKKYSRKMTTYYYWYKWMKKRYRWMKWSSMRRKWMS
44      RYGMTKTTAARKTYKRWKWKAWTAKKKYGRKMCAYYYWCKWMKKRYRWCKWCCMRRKWMS
        ** *.*.   **.**   ** *   ***.*** :**** ********* **..*******

42      STTRRWYMRYKACKAARKYMKRYKRGYTRYYKRYYRRWYCTYCMYYKTKKCGYKYWMKTR
44      SCWARTYMATKTTKRRRKCCKRYKGKCARYYKGCYGGWYMWYMMYYKWTKGTTKYTCGGR
        *   * **  *: *  **  ****   :****  *  **  * **** .*   **    *

42      KYYKTKRKYRRCGCGTCRYYWWKYWYMKKYRYYTCKKYMRKKKRYCAYYATKWAYMKRRM
44      KYYKYGRKYRRYATRCTRTYWAKYWTMKGYRYYYYKTYMGTKKRYMCCCRCKACCCGRRM
        ****  ***** .    * ** *** ** ****  *.** .**** .    * .   ***

42      CKRWRKKRWSYKTTCYWWWSYKRMYRWRYTTCYGMTSAGYRYKWKWYRRYMSYKSMRKAG
44      MKRWRKKRWCTGCCTYWWWSCKRMYRWGCWWSYSAYSCACRYKWKWCAGYMSYKCCRKCK
         ********.     ***** ******    .*.  *.. ******   *****. **.

42      RRYRGWAWMYYYGAGTGCTRMKRKWCRCTTSAAMCCTTMKAYYWKTKRYMYMWMWKWYGK
44      RRYRKWRWMYYYCWAAAYCRMTRKTYGYYGGRWCYTCCMKWYYWKKKAYMCCTCATWYRK
        **** * *****  .:.  **.**      .       ** ****.* **     .** *
```

```
42          KKKTTAWYKKCRYWRYYWYSKSRMYRYRMYYAMYKCRAGCRYYTCCWYRTCKRAWYYKGY
44          KKKYYWWYKKTAYWRCTWYSKGAMYRYRMYYMMCTYRTTARYYCTTWYRCYKGWAYYKKY
            ***   ****  ***  ****. ******** * . *: .***   ***  *   *** *

42          KRTGTCCGATYWCCCGYSKAASKRKSKKSMRKKKSYYRKRRGGYKSMRMRYKTKYRSMMY
44          KGCACYAAGGYWATTACGKGGSKRKGGKSMRKKKSYYGKRRRRYKSMRARYKWKYRCAAY
            *  .  ... **.  . .*..****. ********** ***  ***** *** ***.  *

42          KYWKKWMKMKYYRKWACCGTRKWKMRYKGKKRRKYKWYGACKMMMYYKKRYWCRRRGMWW
44          KYAKKWCKMKYYRKWWMYSYGTWTAACKKKKGAKYKWYATTKAMATYKKACTYGGRKCAA
            ** *** ********   .  .*.   * **  *****.: * *  ***      *

42          KSAGGRYYYMRMYYYGGGGRARSWKYYKMKWSGGYRRYKKRGCRKYRYKYMRCAYCRMWK
44          GCRRRRYYYMGACTCAAAARRRSWKYYKMKACTKYRRYKKRKYRKYRYKCCAACCTGMWK
             .   *****     ....* ********* .  *******  ******   ..   ***

42          YKKRWKRMSYWYYMKGCKKYKKYWWKSAKKYYKMCSWRWYYRKRWYKYRMYKKRSCCYRW
44          CGKAWKRMSYWYYMTRYTKYKKYTTKSCKGYYKAYCAAWYYRKRWYKYRMTKKASMMCGA
              * **********.  .*****  **.* ***  .  ************ ** *

42          TGAARYRSYWKRGTYKYYYMKMMGYWRRKMKGYGKKWRAGGGRRGCCYYYAMGTYKRKYT
44          KKRWGYRSYWGGCWYKCTTMKAMATARGKMKKYAKKTGMKRKRRKMMYCTWMRWYKRKYY
            .    *****    **   ** *.  * *** *.**      **   *   *  *****

42          ATYMKKRKKKWYKRMRRYTCAMWMTMMWYRRRRGATRGWYRKWRYKKRRKWKKGRYYKRY
44          RWYMKKGKKKWYKRMRRYWMRATCKMMWTRGGGKGWGCWYRKWRYKKRRKWGGRRCCGRY
              **** ***********      .*** *    .   *************   *   **

42          KMKGYYKAYYKKKMRKRTYCRKRKYYMKYTYRWRRKYYAYGMYKYRMYWKRRKKKKKRKC
44          KMKCYCKWYYKKKMGGGCTTRKGKYYMKYWCRARRKCTGYKAYGYRCCAGRRKKKKKRKM
            *** * * ******      ** ******  * ***  .*  * **    *********

42          TYGTGARRKWYKGYRYYYYRKKWCCYMMKTKTYYRRGRKYYWAGGGYAYMTWWYYAKRYK
44          YYAAATRRKWYKKYGCCTTGKKWMYYMCKKKCYYRGRRTTYWGATACTYMWWACCMKRYK
             *.:.:****** *      ***  ** *.* ***  *. **.. . :** *    ****

42          YYKWYYRMKKYKKRSWCKARYTGTKRRWWYMYRWRRKKYRSKYYMSKYGKMYKRARWKMS
44          YYKWYYRMKKYTTAGTMKCRYYRYKRGWATMTGTAGKKTACKYCMSKCTKMYKRWGWKMS
            ***********.. .  *.**   ** *  *     **  .** ***  *****  ****

42          TSRAKWKYCYKWAKRRKKCATRMMYMYKYYMYTGCGMGAATKACRMKYRMRYYRRAGYGC
44          ACRMKWKTMTKTWKRRKKTTCRMMYMYKCYCCAATAMCGGCKWSRMKYRMRYYRRGKTTT
            :.* ***   *  ***** : ******* *  :. .* .. * .***********.

42          YYACGCRGAAGMCRMMYYYKMYRWACGTKYRKWGRYYAAGMYRYGRSKYYYYARYAKRMG
44          YTWMRTRRMRKMYRMMYYYKMYRATTTCKYAKWRGCYWRRMYRYAGSGYYYCCGCGKGCK
            *     *    * ********** :   ** **   *   ****. * *** .  .*

42          YTGWRRRYWMCGKGKMGKGASATKKKKMKKMKGRWYTMGRMRRKYTKRMYKRGYKYKWMY
44          YKAWRRGYWCTRGKKMSKCRCTGKKKKCTKMKKRWYYAKRMRGTYAKAAYKRRYKYKWCY
            *..*** **     **.* .:  **** .*** ***   *** .*:*  *** ***** *

42          GCMYAKMRRRMMWMKMWMTGRSYMRRYWKTMTKWKWKKKKGGKGRRRRYYKRKRRRSMCM
```

```
44          RYAYGKCRRRMMWMKATCAARSYARGTAKYCATWKAGKKKARKRARRRYCKRGGARSMYA
               *.* ********   :.*** *   *  :.**  ***. *  **** **   ***

42          TTYKKMYYRAYKRTTRRMRKRYYGRCTYYKAMKKMKMYCWGMMARYYRRRKRGYKKRYRY
44          CYYKKAYCGCYKRWWRRAGGACCRGYWYYKCMKKMKAYYWKMMWAYYGRRKRKYTKRYRY
              *** *  .***  **          ***.***** * * **  ** **** *.*****

42          YYYRRKTMRKKKKKRWAMYKGWARYRMYYRKWKKKKYTRYYTWKRKKRRKCARRWMRYGW
44          YYYRRKYARKGKKKRWMMYKATGGTAMYYRKTTKKKCCRCCYWKRKKRGKMRRGAMRYKW
            ******  ** ***** ***. .   ***** .***  *   ****** *  *  *** *

42          RRKKYYYCRGKRYKKRRRRRYRRRYRGGCYCYTCCACAKTTRRWMRRTKTSKYKRRWARM
44          RRKKYYTMRKKRCKKRRRRRTAGRCGTRYCYYGYMCYWTKYARAMGGKTWSKTTRAAGRM
            ******  * ** *******   *       *   .  ..  * *  .. ** .*  .**

42          RKKTACGKKTTTYAWGCYAYRRRRWAWRRWYRACGKRRRMWYCCTYRMYRKRYMRRWCCY
44          RKGAGYRKKGACTGACTCWCAGRRWRWRRWYRGAAKAAGMWYTTCYRCYGTGYMRRTYYY
            ** :.  ** :  .        *** ******...*   ***   ** * . ****   *

42          YKGKCGCRYMRW
44          YKRKTRYRYMAT
            ** *   ***
```

The pairwise alignments of two accessions 42 and 45

```
42              MWRYRYWSYRSYSMKWRYAMSWWKKCTYRRGKTTTKYRKRYRKKYRMRRWRRWMMKYYKS
45              MTAYRYWSYRSYSMGWRYTMGTWKKACYARKGGACKTATRYRKKYRMAAWRRWCMTYYKS
                *  *********** ***:*. ***. * *   : *  .********  **** *.****

42              YYYMRYMKTAYRWKYRCGYYYCRCAGAGGSRYSRWMWMYKYYMMRYRSAATARMKYYYWW
45              YYYMRYAKCRYRWKCATRYTCYAMCKRRRSRYSRTMWCYKYYCAAYRSTRCCGMKCCTTW
                ****** *  ****    *     .    ***** ** ****   ***:  . **    *

42              RYYRYMYYWRRKYWRWWYKYMYGGYGTTKRWMMMYWKRYWTYCWYRKYKMYWKYWKYWKR
45              RYYRYMYYWRRKYWRWAYKYCYTSYACCKRWAMAYWKRYWWYTTCAKYKMYWKYWKYWKR
                **************** *** * .*.  *** * ****** *    **************

42              RMRMGRYMYRKYRKMRYWYYRMKYRMYCRYYMWRKYMWMKTRKKYMYYWYMMMMYRYYYY
45              RMRMRATATRKYRKMRYTYYRMKYACTTRYYMWRKYMWMKWRKKYMYYWYMMMMYRYYYY
                ****     ******** ******    ************ *******************

42              YYRWRYKWYRRYKMYYYRRYRSYKKRMYYMYKMCYRYWRYWWWMKWSRRKRWMRRWKRWC
45              CYAARYKWYRRYTAYCYRRTGGTKKRMYYMYKMYYRYWRYTATMKWSRRKGACAATGRWA
                 *  ********. * ***  . ********** ******   *******       **.

42              RRMKRMYSRRRYSAGAYAYYMKYGAWWMCTKTGWRYRYYRWWMRMKRYKMMKRGKSKRWR
45              RGAGRMYSRRGYSTAGYTYYMKCRTAACYKKCRWRYRYTRWWMRAKRYTAATRTKSKRWR
                *   ****** **:..*:****  :    .*  ***** ***** ***.  .* ******

42              MRTTARRTKRRYKTKTWMRGWRMRSMMKTKCCCCKKATWRCCYRMGGMRMRKRKKKRMYR
45              MGKKMARCKRRYKYGCTCASWRMRSMAGYKYYMTKKRYTRMYCGMKKMRAGGAKKKRMYR
                * ..  * *****      .******   *    **   *    *  **    *******

42              TWCMRKYRMWKGCTKRCMRYYKRTYTTCCWYRMYWWGKACKRYKRYYYYYRRKYWMKMKC
45              AATCRKCRMWKRYYKAMMGYYKGCYACTAATGATAWKTRMKRYKRYYYYYRRKCWMKMKY
                :   ** ****   *  * ***  *:  .      * .  ************* *****

42              AWMRKYKRCMAGYYRMKMKKKRKMYYTWKYKKMRYRRMYWYRYYSCWKMRKGCGTYYYKT
45              WTMGKTKAYMCKYYRMKAKKKAKMCCYWTYTKCRYRRMYWYRYTGAWTCRKRYTCYYYKK
                  * * *  *. ***** *** **   *.*.* ********** ..*. **    ****.

42              MAGCCAMRWYRRWMWRRWKKSYKRARRKCATSGATRKGRTKYMCCCCAATARATRYMRKY
45              MMRYMRMRWYRRWAAARTKKSYKARRAKYTGSKRYGGAGWKCMYTMSRRKRRGCAYMRKC
                *     *******   * *****  * * : *     .  * *   .  . *.  ****

42              KKYKMKCYGYYWRRYMMKKGGKWYTKWMKYMRYMMMGGCMYWWTTARARCMKKRRRATGA
45              KKCKMKYTTCCTAATMMGKRCKWYATTAGCMAYMMMRRMMCWWACGAGRTMKKRRRRYAW
                ** ***         ** *  ***:.    * ****   * **: . .* ******  .

42              KKYYRKCYSRYMRWRWRYYWKWRTTRKRTAAWMRYWRAGTGTKTMWYTGKWRRWYYSYMT
45              KKYYGKTYGGYARWRWRYTTKTRWYRKAYTTACRCAGWSYKYKAAAYYKGARRWYCSTAY
                **** * *. * ******  * *  **  ::  *    .   *:  *    **** *

42              TGYACWRYRKMARCGGTTCTACCYYYYMMWYYRWMKWTGRCMTYGCGCMAAYKCWRKKYM
45              CRYCTAGYRKMCAMRKCAMCWTSCYYYMMWYTRWAKWAARTMCTKYRYMRRTTTWRKKTC
```

```
          *.   ****.     :    . ******* ** **:.* *      *   . ****

42        AYWKWCMWKRRMRTRYRWMCRWRYWYYWAAGYKKKYKYAKYYKGKYYMWYWKMYKKWKYW
45        TTTTTAAWKGGCGCRYRWMYRWRTTYYWWGAYKKGTKYGKYCTKTCCCWYWKCCTKWKYW
        : . . **     ***** ***  *** ..***  **.** . .   ****  .*****

42        KYTKYWCKCRWYYRSRTCTYRKWRKKMRKYRWWTWYMYKKCCWAYATKRGMSMARTKRYW
45        KCYKTWAGYRTYYRSGAGYCRTWRKKCRKYRTACTYATKKTGAWCWYKRKCCCTACKRYW
        *  * *.  * **** :   *.**** ****    *  **       **  . :  ****

42        TKYKYCGGCAGCYCGGATGTRKYRKKRYAYTRWWWWWMRGWWKKKGAGTWCRRYYKCYRR
45        KKTGCASRYCRYYMKKRYSCRGCRKKRYTYKRWWWWWMRRAWKKTRWSYTMRAYTKYYRR
        .*   ..  .  *     . *  *****:*.********  ***.  .   * * * ***

42        RWKAYARCAYTRYRKYRSMYYMMARMYYWRKRTWYYCYKKRRMMYMYMKKSTYKYTTKMR
45        GWKGTGGMCCCRTGKTACCYCMCWRMYCTAKRWWCTMTKKRRMMYMYMKKCCTKYKWKMR
         **. .  .  *  *  . * *  ***   ** *    ************.  **. ***

42        YYRKAGCKRYYTWRAGAACCRMMYWYYYWYYKYGKYWAKRRAKKCTACYRMSYMYYYRGC
45        YYRKGCTKRYYGAARRMRAYRMMYWYYYAYYKCCGTWWKGGGKGYYRYYRMCCCTYTGRM
        ****.  ****       . ******** ***    * *  .*     ***.   *

42        CMKWKYMKRRRWRMKSMRACKMKKMMKRWYWRMAKRKKGAYYGRRGRGGYRYMRWKKCKW
45        AMKWKYMKRRRWRMKSMRRYKCKKMMKRWYWRARKRKKKRCYKRRTRRTYRYMRWKKSKW
        .*****************  * **********  ****   * ** *  ********.**

42        RKYWKSCWKRKTWGKYMMKRKKMYCYTAKSKKWKMKRRRKTTTTRKTTCGRYCRKMRWKK
45        RKYATCTTGRKKTKKYMMKRKKMYYCYRKSKKWKMKRRRKYWYYRKAGTTGTMRKMRTKK
        *** ..   **.  **********    ************    **:      **** **

42        RKKKCYKMRRYRKYRRRYKRYRYWGRGWWKRGGAYMMRKKMCMMKRMRKYWRMAGYKYGR
45        RKKKYYKARRYRGYAAGYGRYRYWRAKTAKRRRMYMMRKKMYCCTGCRTYWRAWKTGYAR
        **** ** **** *   * *****     **   *******   .  *.***     *.*

42        GWYRYYGASKWMRCTWGRKRWWWKCCWKMTCCYWYMRKKGAGYKRYYRGYCTAKRTAGGA
45        RWTRTYSRSKWMGACARRKRWWWKMYWKAWAYYWYMAKKKGRYKRYYRKTGKWKACRART
         * * *. **** .   *******  **  . **** ** . ******   . *   . :

42        GAGRYYRATTKYYTATRRYRMMSRYYRCGGGWKYMWKTYRWCGTYGAMRMYYMTYRGCWY
45        ATTRYTGRKYKYYWMYGGCRMMSAYYRATTAWKYMWKKTGAYRKTACMRCTYMYYRRTAT
        .: **   . ***      **** ***.  .******.     . ..**  ** **

42        WSKTWRYTTWGCGYYMYKRTYMKRCTRRRRWSGRKMYKTKYMKTTMRYKMRWRWKWYGAT
45        WSKCTGTKAATARYTCCKGKTAGGTCGRRRWSRGKCCKKKCATYKMRYGCAARWTWYAGC
        ***    .:  . *   * .       *****  *  *.*  . .***    **.**..

42        RGCRGKKWARCSRYKYWWRWWRRTAYCAYGCYRMTYKRKCTMYYYSRGTACTGRAWKRTA
45        RTTGAGKWWAMCRYKCWWRWWARKGCYRYTTYRACYKRKYYCYYYSRRATMYSRMWKRCT
        *   . **   .*** ***** *..   *  **  ****   ***** ::  .* *** :

42        AGWKKCGRTKKTRRTKKAKGTTACAKKKSYYKKKRRTYKKKRKRKYGRKYTYKSSYRKRY
45        CKWGGYRRYKKGRRKKKRKRKWRTTKKGCYYKKGGRYCKKKRKRKYKAKTCYTCGYRKRY
        . *    * ** **.** * .   :** .****  *  ********  *  *...*****
```

```
42             CWGTYARYYYYWWMKCTGKGAGMCMGKRRKKKYCYMRYTWTCAGYYYRRKWYTKMYARKT
45             GTAWTMRYYYTTACKYYKKATTATMKGRRKKKYYYCRYAACTGRCYCRRKWYKKMYWRKC
                 .   ****    *   *.:   *  ****** * **:   .  * *****.*** **

42             RTGTYYRKWYTKYRMYRRKMKKYYAGGKKKKKWGCKKYKWKSYYKKAGTTRACTWGWKKY
45             GKKCTTGKWCKKYRMCGGKMTKTYRRKKTKKKWRYKGTKWGCTYKKCACCARYKAKWKKY
                .     ** .****   **.* *   *.****  *  ** . ***..     .  ****

42             YRYSYSSRMYKRMYYWRMYKRKSWYYKRYRTATKYRRRRWYMYKKTRKRKYKTGYMATYW
45             YRCCCSGAATKRMYYWRCCTRKCWYYKRYRYGCKYRGAGAYATKKYRKGKYKYAYMGGYW
               ** . *.   *******   .**.******* . ***     *  ** ** *** .**. **

42             YCCACCCGYSYRRSRSKKKMRKKGWRSKYKRMCYSAYKRKRRMYGKSMRYRARYKYRKSG
45             YTSRATTAYGTGASRGKKKMRKKAWRSKYKGCYCGWYKGKRRATKKSMRTGGRYKYRKCT
               * . .  .*.    **.*******.******     . ** ***    ****   .******.

42             KYYRTMKWYKAGSGAYRRRYKYCRTKYRKCCCCTAKYGKMKKKKKRKRRMRRRYRSYWWK
45             KYCRCMKWYKWRCKMYRGRYKYMGCTYRKYYMYWRKYRKMKKKKKRKRRCGARYRSYTTK
               ** * *****    .  ** ****    .***      ** ***********    *****  *

42             RYKKGYRKYYYKATCYGRYMRKKTTAARRMKMRYKKRRWMMYYKKRRYRRYTKATTRGKR
45             ACKTKYRKYYCKWGAYKACCRKKKYWRRRMKMRYKKRRWCCCYKKRRYRRYGTCCAGAKA
                 *. ***** *   .*     ***.    ************    ********* .. : .*

42             YKAKKYRMTKAMTTYACKKKKKWYTCGYWKKWYYYKRRKKRKRRRYYTKMAWKAWKGRRC
45             YKTGKCRMKKRMYCTTGKKKGTACWYRCTKKWYYYKARKKAKGAATTYKMTAKRWKSRRY
               **: * **.* *    : *** .       ******* *** *       **: * **.**

42             MKRMKGGTKKGKGTGGKRAYTGCGMRYYYRYRKTYMYMTCAGTTKWYRYTRCKTTCTYYYW
45             CGGAKCRAKGKTAKRRTAWYCTYKMRYYYRYRKYYMYAYYWAKYGWYGYYGYKCCTYYYYW
                    *   :*   ...   .   *     ******** ***     ..   ** *    *     ****

42             RMRYMMGCTRTGYSCYYRTSRCRAAMRRRYRKRRGKWWGGMYGKWYYRRCWYYMYGKATG
45             GMGYMMKYWRYKYSSTYRWSRYRGRCRRRYRGARKTTTAAMYTKWCCAATATYMTKKCYC
                * ***    *  **. ** ** *.  *****   *  .  ..** **        **   *.

42             TKKYRKYRMMYYWYYTYKYRSRMWYRYTCAAMGRCYSKKYKRMSKWWRWWMSWKRYWKKA
45             AKKTAKTGCMYYWYTWYKYGCGMWYRYCACGMAGTYSGKYGRMGKTTGAWMSWKGYWKKR
               :**  *   *****  *** . *****  ...*.  ** ** **.*    ***** ****

42             RAATKACKCKKMKGKRMACKCTYTKCYMCGCKKRMYKRKGGRKKWGTCACGMYKAKRMMK
45             GMTGKCTKYKKCKKGRMTYKSWTCKYYAYCSKKAAYKRGARAGGWRYYGTCMYKGKRMMT
                 : *. * ** *   **: *.     * *    .**  *** .      *     .  ***.****.

42             KMKKMWMYMRSMYRTWRKSYGRWKKYRMRWATKKKMKRRKYYGYCARKACYMRYRKMKRK
45             KMKKATMYCRSMCRGWGKGTRRAKKYRMGWRYKKKMKRRKYYRCMRGKGTYMRYRGAKRK
               ****   ** *** * * *.   * ***** *  **********       *. *****  ***

42             KYKKKYYYYYKTWGRYRYGYCKRCRRTKRRRGCYKRKKCTRTWARRSKRKKYRGKRRRKK
45             KYKKKYYYYYGKASRCRTRCYGAYRRYKAGRRYCKRKKMKAKAWRRSKGTTTGAGAAGKK
               ********** . .* *       ** *  *   **** . .  **** ..  .     **
```

```
42          MKYRRMWRRKWRYRTKRRRSKYYYAYKMYRAWYYTTACCGKYRKKRYKMRKKRWCYKRRR
45          MKTGRMWRRGWRYRYKAAGSKYYYMYKMCRWWYYKKGTTRKYRKTGYKMRKKATMYKRRR
            **  ***** **** *   ***** *** * ***...   ****. ******   *****

42          MKKYKRRTCKRRYYRRRMRKWMWYRKAKKRRRTRKWYMTWMYYKRYRRGWCKRMYYRRYY
45          CKKCKGGWTKRRYYRRRMRKWMWYGTWKKGRAYRKWYAWWATYKRCGRKTGKGMYTRGCY
             ** *    ***************.  ** *  ****  *  ***  *   * ** *  *

42          RSKKYTCTWKKRRKRYKCKTSKYMKCRRWRKYYRYTYYRRRATAYCARRMRYKKRKRRRS
45          RSKKYYTCAKKRRKRYKMKWSGTMGTRRAGKTYRYAYCARARWWCYRGAMRYGKAKRGRS
            *****    ******** * *  *  **  * ***:*  *        *** * ** **

42          YWYRKKKWYTGAAMWYMRRRCGRWWRYKCCCCRYRWKAMCCTTRYWCKKWYKKWYRRRAG
45          YWYRKGKWYGATTMWYMRRRYKGWWRYKMYYYRTGAKTCYTGWGCWGKKWYKKWCGARGS
            ***** ***  .::*******   *****    *   *:       * *******   *..

42          YYRKYWYYKKRKSYKRKWYKRKRKKKKKKKCYMYKGWKKYYWRWWTKYACYWYRYMRRMM
45          CCGGYTTCKKRKGTKRKWCKRKRKKKGGGKYYMYKKWKKYYWGWTCTTRYYWCGCAGACM
                *   ****. **** *******   * **** ****** *   .   **       *

42          KMYYRRRMRTKAWWRKRRWRRKTGCCYRYMRKCKARKMRGKRRRYCYYYTKWRRMCKYWR
45          KMCYRRRMRATCWWRKAGTRRKCCAMYRYMGTYKRRKMRRKRRRYMYCCCKTRRMMKTAA
            ** ******:..****   ***  . ****  . * **** ***** *   * *** *

42          KRRWRWMKTTTCGCKWWSGYCWGWMRKRYYCYRYYRSKMRYKKYRRKKYMSYGSMKGCRT
45          KRRWRAMKCCGTAYKWWSKTMWKWMRKRYYYTGCCRSKARYKKCAGKKYMSYRCCGKYGW
            ***** **    . ****   * *******     *** ****   ****** .

42          KYKRYRWYKWWGWMRMMYRKKKRYWMSYMGMYATKWKRYRRWMWKWYRYKKKKRYATKCT
45          KYKRYRATKWWCAMRMCTATKKRYWMSYMKMYGCKWKAYRRTCATWYAYKKKKRCGCGAC
            ******  ***  ***   .********* **. *** ***   .** ****** .  .

42          AYYRCYTRYRWMMWWYSSWKKMCMAAAKKGTCKKWKRKKTMTYMKYYRTTGMKYGCCKAK
45          WTYRYCKATAWMMWWYSSTKKCYCWTGKKRKYKKWKRKKAMKTCKYYRYYKMKYKYYKWK
              **  .   ******** **    :.** . *******:*.  ****   ***   * *

42          YACSRCTGAGCKYKYRKAAKTWYTWRKMGKKWTKYRRKRWKRYKRKRRRWRRWGYMTRRY
45          CWMGRYAATRTKCKYRKGGKKWYWWGKMAKTWYKCAAKGWKRYKRKRRRTRRWKTCYART
               .* :.:  * ****..*.** * **.*.* *   * ********** ***     *

42          YGRGCKWTARKKYSRKMRYYKYCYYCMYGGRRMYSYGCYMRTGGGKKYGCAGRRRKKGYG
45          CRGKYKAKRATTCSRTAAYYKYGYYSCCKKRRMYSTRMYCGGTACKKTRYCTGRRKKKYR
                 * .  .. **.  **** **.    *****   *    . **   .  **** *

42          AACCMKGTAGCTKWRAATGKKRMKKKYYRKWYYMCGARRYWMRKYYYRKYYTKAAARKGC
45          RMTAMKRCCTYYKAARCAAKKRCKKKYYRKWYYAYKMRRTTMAKCYYRKYYGKMWWRKRT
               .**  .   *   .:.*** **********    **  * * ****** *   **

42          RYTRTTTTKTYRMKSTRKYYYCCARCAGATMCTAAYKGRKRYMRGWTATMTYRTRCAGGT
45          GCGRACAKKKYRMKSGGKTCTTARGYRRGWCYWMRYKKRKRYMRKWATCAGYGARTGAAC
               *: :.*.*****  *     .     .      ** ****** *::   * :* ...

42          YYWRRMKSKYKKAYYKYATGATKWTCAKYSRSWMMRSSRYGYRAMCYYKRWTKRWSARGA
```

```
45                  YYWRRCKSKYKKMYYKCCGTCCKWCTGKYSRSWMMRCGAYSTAMMYYTKAAKKRTSMGRT
                    ***** ****** *** .  . **  .*********.. *.   * * *  .** *   :

42                  YKRGKMKYRRMMRYTGAATYGYRYYRYMRRGKGMARRWYYYYKKKWMKTCRKYKRMRKCC
45                  YKRCKMKYRRCMRYKRTTGTKCRYYRCAAGTGTCGGGAYYTYKTKTCGGSRKTGRCRKYM
                    *** ****** ***. ::    ****        .   ** **.*    .**  * **

42                  YKYWWYMYWKMYCKKYKRYCKWGCTAYCTSMYGMYSMRKRYMKWKYKYSRRTAGGAYKKR
45                  YKCWAYMYWKMTAGKYKRYYKAKMKWYSYSMYKMTGMGGGCAGWKYKYSRRCRRRGCKKA
                    ** * ****** . ***** *   . *. *** * .*      ********    . **

42                  KWRAKYKTAGYMRGAYYRKWRYGYYWMSKRCGRRYWRWCAKCTYRAGGGKRRMKGRKTRA
45                  GWGMKTGCWAYMRAWYYAGTACSYYAMSKRTARRCWRWGRKGGYGRRSKGRGMKRRKCRT
                     *  *    .***. **     .** **** .** ***  *  *   .  * ** ** *:

42                  TKCGMTYKKKYWYRGKGGYMGCKWWKKGKKGYATYKCKYKAAGCCYWYSKYAAYSKKKWK
45                  GGARAWTTKKYTCAKKTTTCKYKWWKKKKKKCGCYTYKYKTGRMGYWYSKTRCYCKKGWK
                      .    .***    *      ***** **  . *. ***:.   *****  .*.** **

42                  MCGGATSWRWYRYRSKTCTRWWYYARKYRMYSWRGAATGAYRWSYWKKYYYCMKMGWWRR
45                  AYCAGGSWRWYRYRSKYYWGAWYYGRKYRMTSWRKRWKKMYRASYWKKCTTMMKMAAARG
                       .. **********     ***.***** ***   .  ** *****    ***.  *

42                  KYYYKKGTGYCCWRYTGKKKWRGGYGRRWRWGCCYMTRKWAGARWATTCATKKWMRRKRY
45                  KTTTTKRYKCATWGCYKKKKTGKRTKRGARAKMMYMKRKAGCRGTCCGTGKKKWMRRKRT
                    *   .*    . *    ***      *  *    **.** .    .   ..********

42                  MWMMWYYRRRKWYRKR
45                  MWMMWTYRGGKWYRKG
                    ***** **  *****
```

The pairwise alignments of two accessions 42 and 46

```
42              RYWGGYRRYGRRKTTTWWKRYYYRMCYWCKRRRYRRAAYYWRRWMMKKYMRYRWKYYMRC
46              RYWRKYRGYTRGKCGCWWKRTCCRAYTTGKRRRYRRWRCTWRRWCMTKYMRYRWKTCMAY
                ***  ** * * *   ****   *     *******    **** *.********  *

42              RYYRSMYYYYRRWCGGMRMKYKYYWKRYRGRYMYYMGRCCTCKWRYRYRYKWYRYSYWKR
46              RTYASMYYYYRRWMKAMRMTCKYYWKGCAKGYMTYMRRMYCMKWRYRTATKWCAYCYWKR
                * * *********  .***. *****     ** ** *    *****   **  *.****

42              RKYRWWWYYRKWYSRGMRCYTMMCYRKCYKMMRARARYRYRWKSWWSYSCYMAKRKRYKM
46              RKTRWWWYYRKTYSRAMRTTWACYCRKYCKMMRWRRAYRYGATGAWSYSSYMRGRKRYTA
                ** ******** ***.**       **  **** *  ***   .. ****.**  ****.

42              MKYRKYMWKMWRMYSWYKSRKKYMYYTRKRMCWKMYYWRMRMYMGCYTYYRYWKYRRCTT
46              ATYRKCAWGMWRMYSTYKSGTGYMYYWRKRATWKMYYWRMRATMRYCYTCRYWKYRRTCC
                 .***  * ****** ***  .  **** ***  *********  *      *******

42              YMKMRRMRRRMKYRYWYRRRRWMKYRRMMRYWWWMCCCMATCRYTGMRAACRTYYSCKWC
46              YMKMGRMAAGAGYRYWYRRRRAMKYRRMMAYWWWMTGACWYYRYCTMRMWTGKTCCYTAT
                **** **     ********* ******* *****  .    **  **    .  . .

42              KMYWKMWRCRYRYYYRSGKMCMGYRGTGRKKRAMYYKRGTGYRAMKRRWKKYKYMKYRMY
46              KMYWKMWRYRYRYYYRSKKMAMRCRACARKKRWMYYKRRYRYGGMKRRWKKCKYMKYACY
                ******** ******** **.*  *. .**** *****   * .******* *****  *

42              WRGGTTCYAYRRYYGTRWWKSYSYYYYRYYKMWMRKMRYRYRRMYACKKWYYSCTYKMWR
46              WRAKWYTTGTRRYYAGGWWKGYSYYYYGYYKMWCAGCRYRTGRMYRYKTWYTSSKYTCWR
                **.     . ****.   ***.****** *****    ***  ***  *.** *..*. **

42              WYYYYKGMMRWMMWRMMRWRYKKAASYCRTTATGTRTRKKRCSARKRKYYMYMTYRCAAR
46              WYYYYKKMMRWCMWRMMRWRCKKGGSYTAWATCTWRYGKKRASGGKRGYYACMACRSRRR
                ****** **** ******** **..**   ::   *  ***.*. ** **  *: *.  *

42              RKCARYMRYKGKMKMRTCMRRYMMKYYATWYYRYKGRMAGGWTTARARYTRRRYRYWMCR
46              RKTGRYMRCKKKMKCRWYMAATMMKYYCGTCCAYKKRMWKATACGAGRTYRRRYAYWCYG
                ** .**** * *** *  *   *****.     ** **  . : . .*  **** **

42              CAWKKTACRTKWTTGCGKRYMWMRWMGMYYYWYWGYARTMWAGRKRRWYYTCATMGRMCK
46              TRWKTWCYRWGTYWKTKGAYCAMRWMKMCTCACAKYRRYAAGRRKRRWYCKYGYMRRMYK
                  **. . *          *  **** *       * *   . ****** . . * ** *

42              RRMGTRRKMCWYYRGWGRRMMRYAYYWMWRMRYRKYRRYKAWRATAAGAYRKGCWMSKKC
46              RRMTWRAKMTWYYARTTARMMGCCTTWMWRMRYRKCRRYTRWRWGGGAGYRKKMWMGTGY
                ***  * ** ***     ***  .  ********* ***. **  ....***  **..

42              RWWWKYWWYYKRYKKKYWTYKRYYCAKMSTAYWKYTTTWWCKTRKYGTYKACKMMMGGGG
46              RWWWKYWWYYKRYKKGYWWYKGCTYGKCGYTYWKYWWKAAYKYRKYKGYKGTKMMMKTKK
                *************** ** **    .* . :****  .   * ***  **. ****

42              ATRWWWWWMWYWKKKYKKSWCRYRYRRYRRRKYRKKGYRSKCYGAGMSYKYYWRKRACGT
46              WCRWWWWWMTYWKKKYTKGTMRYRYRRYRRGKTGKGKTACKMCAMRMSCKYCTAKRRMKW
```

```
                           ******* ******.*. ********* *  *    .*  .  ** **   **

42                         SRKWYMWRTMYMYKRKYRTAWSYMRYYSRRKCGGGKYCTMWRRKRMRGKYYYWRRYYYKR
46                         CAKWCCWRCMYMCGRKYRCRTGYMRYYSRRKTRRRKYAGCARRKRMRRKYYYARRYYTKA
                           . **  ** ***  ****   .*********    **.   ****** **** **** *

42                         RYMRKATKKRRYCAGYAWYMKWTTTTTRRMCAASYRKGAWRSYKKKKRWKRKWWSYYKCC
46                         AYMGTGWKKRRYMGTCTACCKWYCAYKRRMMGWSYRKKRWRCTKKKKRTKGKWWSYYKMT
                            ** .. ***** .  :   **  : .*** . ****  **. ***** * *******

42                         CTAWMYWCTTAKKWAKRYYWKRYKAACAGWYYRRYMKGATGSYSMRKKYKKRARYRGYAT
46                         TAGWMYWACCGKKTGKRYYWGRYKGWTTCACCARYMKAGWASYCCRKKYKKRCRYRKCRK
                            :.****.  .** .***** ***.  :     ****.. .**. *******.***   .

42                         SCWYMYYYRMWYMKKKAAYKWKYARGCCCCTKRYWYYWWGAKKRRKTWMWSKRRKKRRTR
46                         CYATMYYYRMACCKKKCCCKWKTGGATYYYYKRYWYYWWCTKKARGWTCASTRAKKRRCR
                           .   ******   ***.. *** . .    ******** :** *     *.* **** *

42                         RYCKRRSMMKTCCYMGMRMRKRCAKKYRWMKRRRRYRCYYRWYRMWWWGTRRKYMCKRKK
46                         RYYKRRSMAKYAYYMTMRAGGAYCKKYGAMKARRRYGAYYRWTGAATWRKARTYMMKRKK
                           ** ***** * . ** **     .***  ** **** .****     * . *.** ****

42                         RMCGAYYYYRTATWRRYYYRRKWKRKRYKKMYKWKYKRYKCMTCMGRMMKYKMYYAKKMR
46                         RAYRRCCCTGCCCWRRYYYRRKTTRKRYKTMYKTKTKATTYMYMCKRMAKCKMCTCKKMR
                           *          . ********* .*****.*** * *  . *    ** * **  .****

42                         RGYMTCAGYCMWYKKMGKKRYCMKWKWKKRTRAAYRYMKYMCSKYKGRKYRTARKKRYYK
46                         RKTMATRKYYMTYKKCCKKRYYAKWKWKTRCACTTRTCKYCMGKYGTRKCGGGRKKRYYK
                           *  *:   * * ***  ****  *****.*  .: *  **  .**  **   .*******

42                         KYKKYRAYGAKYMAYKRRKKCMTWTWYKKKKYRRRWKCRYWCYYGTMKYYRRYYRTCKRY
46                         GYKKCRCTATGCMWYKRRKKTMGWYWYKKTKCAAGWKAGYTYCTKKCKYTARYYRGTKRY
                            *** *. .:  * ****** * * ****.*    **. *     . **  ****  ***

42                         YYRARTRRWWCKKYKMRYCMYGMKKKKSKKWCKYKKAGKGATTKACACWSCTYCTRKYYK
46                         TYGGRCGRWAMKKCKAAYSCCKMTKKKGKKWYGTKKTAGTRYWKCTRYWSTWYTCRKYYK
                            * .*  **  ** *  *.   *.***.***   **:.     *.   **  *  *****

42                         WYMAGASRMYYRMYYYMYKRYYAYGAYWYTYKGARGTCYRRRRAWKKYYWYKYYRKRKYT
46                         WYCRRRGAMYYRMYYYMCTRYYCYRWYWCCYKTGGRYAYRGAGWAKKYYTTKYCRKGKYY
                           **     . ********* .***.*  **  ** .   .**     ****  ** ** **

42                         YWCCCCCGYSYRRSYKRRSGCRKKSKKSTCTRKRAKWRWSYYYYRCCCMYYRKRKYYRTC
46                         YWMSATTAYGTGASYKRRCTYRKKGKKSYYWRKRMKWRWSYYYTGYYMAYTAKRKYYRWA
                           ** ..  .*.   *****.  ***.***   *** ********      *  ****** .

42                         RCTKYRAKGCTYKRYCMTYRGTMWSKRYTATYGYYKKRMAYRKKRCTRRWSYTGYKKMKR
46                         ATYKYRRKKTYYKATYMKTRCAMWSKGTKCCCRYCGKGCGTRKKGTCRRWSTYSYKKMKG
                              *** *   **   *. * :****   ..   *  *  . ***   ****  .*****

42                         CTRGCKYKSMMYYWTRKTCYGATRCGTWYGRYRYTRRWMRYTCGGCCKRGRYAMKKGMGG
46                         TCGTTKYKSMMYTTGRKWYTAGCRYRYWTTGCRYYRAWMRYATTCATGAKAYRMKGAARK
                                *******   **    .. *   *    ** * ****:   .     * ** .
```

```
42          KRKRMWYCGCCGRKSAMYYYSRWGTCRMYWYRYMKKMRYMKYKKYAAYTCKTKKKTKKKS
46          KRKRAWYTATYARKSGCYYYSRWACTGCYWCAYMTKCGCMKYKKYRCCWSKCKKGKKKKS
            **** ** .  .***. ******.    **  **.*   ****** .  .* ** .****

42          WRWYYRKRTTTARYYRGYSWGAATGAAACYRWSYWKGATTKYYWKYKKYYYMKYYWWKRR
46          WRWYYRKRAGAMGYCARTSWKRWKKMWRYYRWSYWGKRKYKYCWKYKKCTTMKYTAWKRG
            ********: :  *    **   .     ******   . ** *****   ***  ***

42          KMYYKKWYYRYKWRYRRWGCCYRYYTMGRCCYMGGGCRKKKKMWKRMRRKRYGAYTCAKM
46          KMTTTKWYCGCKWRYRRAKMMYRCTCMRRTTYMRRRYRKKKKMWKRMRRKRYKWTWMRGM
            **  .***   ******    **   * *  **    ***************       *

42          WMMKKYWYGYRRRWYRKRMYKTTYWYKWAYWCRKAYYYRSWYMKRYRTTWWYWMYKKYYW
46          WMMKKCWTKYRGGWYRKRCCTWYYWYKWGTWYRGGYYYRSWCMKRYRYYWWCAATKKTYW
            ***** *  **  *****  .  *****. * * .****** *****  **    ** **

42          TYRAWRKRKMAYTGYSMKTKMMRYWKGYYKYMGGACARRWYAYYRSYMYYAWMKRMRWKG
46          WYRRARKRGCGCAAYCCKCKMMRYWKATTGCMRRMSRRRWYRYYRCCCTTMWMKRMRWKR
             **  ***  . :.*. * *******.    *   . **** ***.     ********

42          TYKGKKWYWWWYKKYMRYKRYWYWRMMRKYWSYYGTRGWKRRGTAWTAAKRKKYWGKSYA
46          ACKKKKWYTTTTKKYMRYKRTWYWRAMRKCACYYKKRTWKAGKYGTCCGKRGKYAKTCYR
            : * ****    ******** **** ***  .** .* **    .  ..** **  ..*

42          WYMMCCKCGKMYKTTTYCKMTWYRRRRKAGRRRWRKTWYKRKKGYRRYRKKYRRRRYKMR
46          TYMMTTGTAKMYKKCACYKMKAYRRRRKMRRRRTRKKTYKRKKKYRRYRGKYRAAGYGMG
             ***    .****. :  **. ******  *** **. ***** ***** ***   * *

42          YRGYYWTCCTRRTTTGYGRGWWKRKMGGAKYRMMRRMRMMTTYMWMYKYRWRYYKYKWYA
46          CGKYYWCGTARRYYWTTRARWWKRKMRRMKYRMMGGMRMMKYYCWMTTYRWRYTGCKWTM
               ***   :**        ******   *****  ****. * ** .*****   **

42          TAGCAGGKYSTRGYACRKKRWWWKMWWYCTCKYSKRRRMRKYAWMRYRKSYRKRRRRRAG
46          ACTYGAKKYSCGKCTARGKRWWWKMWWYACTKYSKARRMRKYRWMRYRKGYAKRRRARTT
            :.  .. ***    :.* **********.  **** ****** ******.* **** *:

42          MRYYRKKGYRRYYKYRWKGMYGAYYRWCGTRTMAMRRKMTTYAYYRSGRRTCGYKWAMYY
46          MRYYGKKRYRRYYKYRWKKMYRRYYRWYAKRKMGAGRGCKYYWTYGSRRRATKYGWMMYY
            **** ** ********** **  **** ..*.*.  *  . *  * * **:  * * ***

42          KYMRKKSRKKWYYKYSRRTAYRTYKRRYWRKYGYWYGCRKKKRWKYWYKRYRRYYMKWYR
46          KTMGKGGGKGWYYKYSRRCGYRYCKARYWRKCRTTCAMRKKKRAGYTYKRYRRYYMKAYR
            * * * . * ******** .**  * *****     . *****  * ********** **

42          TWKYMSKTRKRKYWCGAYKKCTKYYKYKKRYRRTGCRKKKKRTGYGKKGAAAKRYYMRCK
46          KATYMSKAAKRKCWYRMYKKGGKYYKYKGRYGRWATRTKKKACCYKKKKRWRKRYYMAYK
            . .****: *** *   ***  ****** ** * . *.***   * **    *****  *

42          KARTCTKAYKYRRCMMYRKKYRYGTYCKGRGGRYRYYRYRCWYCYMYMGKKYWYTRCRYY
46          KTGATCKWYKCAAYCAYRKKYAYACCYGRGRRAYRYYRYRYAYYYMYMKKKYWYGRTGYY
            *: :  * **      ***** *.         ******* * **** ***** *  **
```

```
42          YATYYYWGKYSKYRCKKSMGRRRYRRKWRWGGRYKKRWYYCTKRRMYRYRRRYWWYYAGR
46          TWYYYYWKKYSKYRYTKSMKRRRYRRTTRTAAATGGGWYTYWKRRMYRCAARYWATYGKR
               **** ****** .*** ******. * ..     **   ******   ***  *. *

42          MKWCTTMRAYYYYYRMWMKMKTYYYKRYCCWMKKRRMTTMRRWMSSRYGYKKTMRKMGTW
46          MTWSYYCAGCTCYTRMWMKMKCYYYKRCYYWMKKRRMKYMRRWMSSAYSYKKWCAKCSAT
            *.*.    .   * ******* *****   *******. ******* *.***   * .:

42          MMKRRKYRYMMMAKKRRRYMYYGYRYRWRRWYYTKKYWMKTKRKKRGYRRYYKWWKYSKM
46          MMKGRKYRCMMMGKKRRRYMTTKCRYRWGGWYYWTKYTMGGKRKKRRYRRCTKWAKYSKM
            *** **** ***.*******    ****  *** .** *  ***** ***  ** *****

42          YKYKTCMWKRYWRRKWKKKMKKMKYYKKRKGTATGRRKRRKKGCRWKRRWYSRYWWMKKC
46          TGYKKYAWKRYWRRKWGGKCKKMKTTGKRKAAGWARGGGRKKCYGTKRRWYSRTTACKKT
              **.  *********  * ****   ***.:. .*   ***    *******    **

42          YYTYSKRKYRRTKTCYGCKRRTCYYYRKWYKMYGYGAGYYRYYYYRKKCCYCYKTKMRMR
46          YYGCSKRKYRRWKWYYRTKRRAAYYCRKWYKMYRCRMKCYRYYTTGKKATYYCKKKMRMR
            **  ******* *  *  ***:.** *******      ****   **. *  *.*****

42          YYYRGAYRMKGYYYRKCKWYAYWATGRRMWWYRYTRWCRYWRKTARTCGRYYRAYAAWYW
46          TYYRKGCGMTKYTYRGYKWYGTACCARRMWWYRYCRWYRYTAKATGGYRGYCAGTTMWYW
             *** .  *. * **  ***.  . .******** ** **  *::     *  . : ***

42          KKKRSYKRKWYRCKRMRCYYYWWKATGGKKAKTYAKAGRWWKMARTCTKGCYRYMGMKMY
46          KKKRGTKRKWCAYKRCRYYYTWWKGCAAKKMKYCRGGRGWWKMTGAGGKRYCGCAKCKCC
            ****. ****   ** * ** ***. ..** *    .  ****: :  *        *

42          YRRRMRTKSKGGCGRRWKKRTGCCYRMKRKCAACWMRYKWRMRARKAKWMGYYSWYYYTR
46          YRRRMRATSKTAAAAGTKKACCAMYRMKRKMRGTAMRYKWRMRGRKMKWMKYYSTYYYKR
            ******:.** ...   **   . ******  .  ********.** *** *** ***.*

42          YRKYRKKRYRMCAGAACAKKTRRWKTYGRWKGKKYRRKRRGYMRKKCMACYRRMMRCRKR
46          CRKCAKKRCRMGCRRMYMKKYARATWYRGWKKKTTGAGARKYMRKKACGTTGAACRYAKR
             **  *** ** .     **  * . *  ** *.     * *****. .      *  **

42          KMGWRYRRRGKTTCTSKYKRWYYYYWAYRYYYRRYRRYTYMRWYKMMRTGRCGCATKKYW
46          KCKWRYRAARKGGTASKYKAAYYYYAMYRYYYRGYRRYKYMATTKMCGGTRYRMWAKKYW
            *  ****   *   :****  ****  ****** ****.**   **    *    :****

42          RWGCAGRGYMTTAGCKWRWTGAYYRAGAGCTWYMRRRRKWYYGTTYYRRYWKTARCTCMY
46          GWRTCTRKCMCACTYGARWCCGYYRCRWKYYWTCGGRRKWYYRYYTYRRYWKYRAYKYMY
             *  . *  * :.    **  .***.     *    ******    ******    . **

42          TCGAGCYYAMKRYRKATYTRMKSRYYRYMMYYYTWKKKKCMGTMMWCKRRRYYRKMYYRK
46          CYKRAYYYRAKRYRKGAYKRMKSRYTAYMMTCCATKKKKYMKCMCAGKRRRYYRKMYYRK
                . **  *****.:*.******  ***   : **** *  *   *************

42          RKMWTGKMMYKMYRRWYYWKRGGAGMWKYWYAYYATAYRRYKCRWKYTCGKWMKATCKKK
46          RKMWKKTACCKAYGRWYYWKRRRMKCWKYWYRYCWYWYGRYKYGWKYWYRKWMKMCTKKK
            ****. .   * * *******     ***** *    * ***  ***   ****   ***

42          RTGAKKRYKKYGRMRRYTRYRSYTKKWRYWKSCKTYRRYMWKGAMKMYRRWMYKKKKRCA
```

```
46          RCTCKKRCTTTRRCGAYKRYRSYYKKTAYTTSGKKYRAYMWKRCMKMYRRWCCKKKKRYR
            *  .*** ..  *   *.***** **  * .* *.** **** .*******  *****

42          TKKRYYKAKCTYTKYRCAKKKTRMWMWTGKKWYKKKGRKAKRMAYRYGRSKKKYTTAKGR
46          KTGRYYKTKMYCYKYRYRKKKARMTMWYAKKWYKKKRRKWKGMWYRYAGSGKKYGAGKKG
            .. ****:*    ***  ***:** ** .******* ** * * ***. * *** :.*

42          MKGYYRCTWRKRWRYWKKRTGTCCTAKKAKKGYRRAGCAATMRRRKGTKKCAAAGMKMAK
46          CKKYYRMKWRKGTGYWGKGCRYSTYGKKMKTSYRRRCARTGMRRAKRYKKTTTTAMTMRK
             * *** .***   ** *    .  .** *..***  . : *** *  ** :::.*.* *

42          YRRRKYMCTRMRYKKWMYAKCGYWMMMKYWMMWMYRSMMRRGWKKYRMKYRMKWKYMWWK
46          YRRGTCCYAAARYKGWMYGKYTYWCMMKCWMATCYRSMARGKAKKYRCKCAATWGYATAG
            *** .   :  *** ***.*  ** *** **   **** *   **** *   .* *

42          GRKRTYWYWMTRKKRTRGRKWKMWYRKKYYKRRRTRAYMTGWMYRRWCYYYAKAAYYGGG
46          ARKRYTTTACGAKKGYRKGKWKMWYGTTCCKGRAYRRCAARAATGRTGTCCMGWMTYAAA
            .***        **  *  ****** ..  * *  *   :     *          *...

42          RSMWKMCTGRWGYRRKRYCKTWSKYAACRYYKRWMRATAYRTRKMWRYTAYKKRWKRMKS
46          RSMWKMGCAGWKYRRKRYMKWWSGTMGYRYYKRWCARWTCGCAKMWRYYRCGKAWKRMKS
            ******  . * ****** * **   . ******    :    *****    * ******

42          YWY
46          YWY
            ***
```

The pairwise alignments of two accessions 42 and 47

```
42              YYWKYYMYWKYMYRRACWKGKKWRYRYSWKKCRWKYYYRMCYTKRRRYRMYYRYYKMMRR
47              YYWKYYMYWKYMYRRCYWKKTTTRYRYGTKKAAWKYYYRAYTWTRRRYRCYYRYYKMMAA
                ***************. ** .. ****. **. ******    .***** ********

42              YMYRWYAYWKYRYGGCCCKMMAYGTRYKAYRMKYRRRMRRWYYRRRGWWRYWRYRYWTYY
47              TATRTTMYWKYRYRKYYYKMMTYTCGYKMYRMKYRAACRRWYYRRRTWWRYWRYRTWCYY
                   *   ******     ***:*   ** ******   ******** ******** * **

42              YRKGYRWWYYRWWRYSRMCYRYYYYSRRKRMRYMRYRRWKKKKKKCKWRWMKYTKRCRSR
47              YRKKTRWWYYRTTRYSRMYCRYYYYSAAKRCAYMRYRRWKKKKKGTKWRWCKYAKRTASR
                ***  ******  *****  ******  **  ************  **** **:**  **

42              MWMRRRWRYRYSTTYYKYWWWMRSRRCCYTKKSRKMYKMRKRYRYMKCRYMMRYMWMWKY
47              MWMRGRWRYRYSGYYYKTTATMGGAAYYYCKKSRKMCKMGTRYGYCKTRYMMATAWMAKC
                **** *******  ***    * .    * ****** ** .** * * ****   ** *

42              RRMYRYWCYTWMYTRKMRCCMYYGRYMKYKWKYRRYKSCTKYYKMKRMKMMKKMYRCAYK
47              RRMYRYAATWWMYWRKARYTMYYTRCAKYKAKTRRYKGTYGYYKMKRMTAATKMYGYTYK
                ****** .  *** ** *  *** *  *** * ****.   *******.  .***  :**

42              SYWRKRMKRMYRYRRRYWYRWKYYYWRKYRRKKRYKGYYATTWKMKKRKYKKKRSWCRTG
47              SYWAGGAGAMYRCRRGCAYRWKYYYWRGCRRTKRYKKYYTGYWKMKKAKYKTTAGTMRAK
                ***      *** **   *********  **.**** **:  ***** ***.. .  *:

42              RYKYRRRWYYRKGYRSKYYMWAMARATAATKMTTYWRRWKMSSKWKYYYKRWRRKKRMMY
47              RYKYRRGWTTGKKTACKYCMWRCWRRCCGCKMWYCTARWKMSCKWGCTYKRWRRKKRMMY
                ****** *   *   .** **   *  .. **     *****.**   ************

42              KTMYMKMMRMTGYKYRWMRYYRRACRWRRWYYRGGAAKKKRTMWYYWKMYRWRYACGTYK
47              KCMYMKMMRMGKCKYRTMRYYRRWTRWRRWYYRRRMRKKKRKMATTAKMYRAGYTTTCYT
                * ********   *** ******  ********    ****.*    ****  *:   *.

42              YSWSKMRYRMYYYSCKMWRYKYYYKGMMRWTAGYRWWYKAAASYARKRRRWTAAGRYSRK
47              CSWCGCRYRMYYTSSTCWRYKYYYKKMMRWYRRYRATCKWGGSYMGKRRRWKMRKRYSGK
                 **.  ****** *.. ******** ****   **   *  ..**  *****.   *** *

42              MTYYMGKKCAAMYRRGAYMYYKGGMAMMMRRWMACYYTRKAGGATTTMRAAYWKMTRRRY
47              MWYCMKKGSRRMYRRATYCCCKTTMMCCMAAWMCGYYARKTTTWCGKMARMCWKMYRRRY
                * * * * .  ****.:*   *  *   *  **. **:**:     .*    *** ****

42              RCWYYTKYYRKAWRYMRWGAAGKGKRYCAATWMYKAKYYWYKTTKSKYKMYYYCCKTCYM
47              GYWYYGKYYGKCWRYMGWSMCATAKRCYMWYWCCTWKYYWYKYYGCGTKMYYYGTKKYCM
                  *** *** *.**** *. ....**     *  . ******   .  *****  *.  *

42              RKKKMYRWWMYKYKRRYRYAYYCKRKAKMKMMKYRYYWSKYKYMWMRRYMRMWSKRYYGK
47              RTKKCYRTAATKYKRRYRYRYCSKRKGTCKMMKYRYYWSKTGCMAMRRYMRMWSKGCYCK
                *.** **    ******** * .***.. ***********   * **********  * *

42              RKTMRCTRMRRWSACYKMYKYMYWTRYKGGATYGTTTRYYARWYRYYAMRYMYTWMKWRK
47              GKYMGTCRMRRWSWYTTMCKYMTWGRYKKAGCCRACGRYCCRWYAYYGMRYMCGWMTWAK
```

```
         * *   ******   .* *** * ***  ..   :  ** .*** **.****  **.* *

42       YKRMKRRRAYRCGCRRKSYYYSRKYWTKKKKMWKKYGAKKGKMKKKRRGKYKMMRYKRKR
47       YGRAKRRGGYATATRRKSYYYSRKYWGGKKKCWGGYRRKKKKMKKKRRRKYKCMGTKGGG
         * * *** .*  . ************  *** *  *  ** ******* *** *  *

42       RYKKKYKATCYWYYSGWRKCWYKTGRGYWWMKRWRGMYYKRRKKAKMRKYATTRRWKYAR
47       RCKKKYKMGTYTYCGAARKYWYKCCRTTTACKGAGKAYYKRRKKMKCRKYTAWRRWKYWR
         * *****   * * .. ** ***  *     *     ******* * ***:: ***** *

42       YYYRKKWYKKMRMRYYRWYKMKKYYKWAGGYMKRAYYATCWTWTTRRMKKRRWRWSYWRM
47       YTTGGKWCKKMRMRYYRTCKMTKYTKWRRKYMKRCCCRCMTCWYKRAAKKRRWRACTWRM
         *    ** *********  **.** **   ****.       * .*  ****** . ***

42       GTTTCYKATTWWWSYKCYYGTGKCWRYWKMRKWCTRYYRKYTMYTTMKKRYRYRRKYSGY
47       KCCGTYKRKYAWWSCKYYCAARKMWRTWGMRTAAGRYYRGTGCCWYAKKRYRCAGKYSKY
              ** .  *** * * .: * ** * **. . ****        *****   *** *

42       SMRMSYKRTAARYRGMMWYWMYYYGGTWKAYRMYKWKYRGCKRYWAKMTATCTTMYWWRR
47       CCRMSYKRKGGRYRRMMATWMYYYAAKAKWYAMCTWKTGAYKRYWRKMAGAAYYMYWWAR
         . ******...*** **  *****... * * * .**  . **** **:.:.  **** *

42       YMWMWKYYTAGRKKRWWYRYMRGCYSYKYGTKYYYKRKMWKRRWRYWKKTKCKKCKASKR
47       YMTCATYCKRKAKKGWAYRYAGRYYSYGCTCKYYTKRKAWKRGTGYWGKGKATKMKRCKR
         **   .* .   ** * ***    ***    *** *** ***   ** * *..* * .**

42       RKKKMGGKCKMKYKRKRMYKTMMRTKYKYKMYAKTYRRYMMCMRSMGCRRRWKKYTGAAY
47       AKKKMRRGYKMKTKRTAAYKAACRCKYKYGMYGKCYRRYMMYCRSMRYRGRAKKYCKRRY
          ****    *** **.  **:  * **** **.* ******  ***  * * ***    *

42       MMKTWKWRTRCGKCYACMWRWMGRGWTYRTATRYRWYYRYMAGTAYWRYWYYRMKWYACC
47       MCTKWKARWRTKGGYGMCAGTARRRAKYRYRYRYRWYCACARKWCYWRYWYYAMKWYWTS
         * ..** * *    *.       *  .**   *****       .******* ****  .

42       YYYMMRYGWRWMKGYMAAWRKRAAGRCGRYCKKKMRGGATGYRWRRKYKCKYKYYMWMYW
47       YYCCARYRTRWMKRTMRRWRKGTGARYAATTTTKMRKAGATYRARRKYKAKYKYYCWMYW
         **   **  ****  *  *** :..* .   ..*** ..: ** *****.***** ****

42       YYMRCKRTWKAYKRSAYYKTGCMTKRMRRKRRYKTTTRMKRKGRGGRSMYYRYCMWRKRY
47       YYMAYKAYWKWYKAGGTYKKAYCKGGAGAGGGYGYWYRMKAGAGRRASMYYRYMMWRKRY
         ***  *  ** ** .. **..  .        *    ***  .    ****** ******

42       YWRYAYYWRMKGKWKMKSMMGRRRYGRKRKKCTAYCTGRRSRRKYRRRYGAWYYRWGGTC
47       YWRYRTTTRMKKKWKMKSMMKRRRYKRGATKYACYYWTRRSRRKCAARYRMWTYRWKRAT
         ****    *** ******** **** *  .* :.*   ******   **  * ***  :

42       YKYAKYMYRWKMGAGAYYYYWWRSGYMMYRYYAMYYKKYRKSRKRMYKSRYMWKWYKKYS
47       CGCTKTMYAWKCKWRGCTCYWWGSRYMMCRYYGMYYKGTRKSRKRMCGGGCAWKTYKKYS
            :* ** **    .   *** * *** ***.****  *******  .   ** *****

42       RRYRYKKKRYYWKTATGGRRYYAKKTAKYKKTRYRYMTCKYWYKMSKAKRYWMWKCTKYK
47       RRYRCKKKGYTTGKGKAKRRYYRKKCWTYKGYACAYCYYGYAYKMSKWKRCWMWKGGKYG
         **** *** *   .... **** **  .**     *    * ***** ** ****  **
```

```
42          YRKCYGRKSGKWRRKKTRKKRYKYYRATGYGCCWTYMTKRAKRKMWYKYYYKYRAYWMKK
47          YGKYYRRKSRTTAAKKCRKKRYTYTARATYKYMWYTACKRRKRKMWYKYYYKYGCCWMKK
            * * * *** .   ** *****.*   : *   *    ** ************ . ****

42          KKTAYWMRSSRYGYTKWYRAKKRKTCWRKYRRSRRCYMMRMRYMYKYYGGYRYYWRRYYG
47          KKATYWMRSSAYSTWKAYGCKKRKGYWRKYRRSRGYYMMRCRYATGTYRKCRYYWGGYYA
            **::****** *.  * * .****  ********  **** **    *   ****  **.

42          YYKKKKYWKTCKKKRGYRRYKWWYAWYKMYMGGATCKKGRYRKWGTYWWYCYRSYMYYKW
47          CYTKKKYTGGSKKKRRYRRCKWAYGAYGMTMKRGGMKGKRYRKWKKYWWCMYRCCCTTKW
             *.****   .**** *** ** *. * * *  .  *  ***** .***  **.    **

42          KYRRWKRMKSMAGYKGKKMWWWWYYMMKRSYCYYKCTYRKKGRYKWWYRKRRKGGRAKCR
47          KYRRWKRMGSMGAYKKKKMWTTTTYMMKRSTGCCKMGCRKKKRYKWWYRKRRKTRRRGAR
            ******** **..** ****    ******    *   *** ***********  *  .*

42          KWKSYACYYACWKKYKRKKMYYYKRKAACRMKWTAKKRRKKRKKSRKRKYRKYRGRTRRR
47          KATCCRTCYRYTKKYKRKKMYCYKRGWWTAMKAYMKKRRKKRKKSRKRKYRGYRKRCAAG
            *  ..    *   ********* ***     **   **************** ** *

42          KMWGRWWYRGGAKYRKSKWRYKYKWKAGKGATTACRACWYCTGWKMKCRCWSYRSYSRYM
47          GMWRAATYGRRMKTRTSKWRYGTKWKWRKTRYWCTARYWYYKKWKMKMATWCCRSYGAYM
             **    *    * *.*****  ***  *    .    ** . ****   *. ***. **

42          YRRMYYGMYKRSTAACWYYKRYWTKWRTTYKRRRRAWKKYKYRWKRKGYMTWCCCGYSYR
47          YRRMYYRMCTRCATGTWYYKRYTCKWRYYYKRGAGWAKKYKYRAKGKRYCYWATTAYGTG
            ****** * .*.::. ******  ***  ***     ****** * * *  *.  .*.

42          RRKSYKRRGKTRKTAATKKSKKACYRRSMTCTMKGKWRKWSYTKKYYYRKRRMYYRRKKC
47          ARKSYKRRKKYRKCCCCKKGKKCACAGSMYYWMKTKAGKWSYCKKYYYGKRRAYTRAKKT
             ******* * ** .. **.**..   **   ** *  **** ***** *** * * **

42          SMYYRYKRACYYTTTGYTGTYWYKKRYGKKAYKGTRRYKTRYAYRKKKYWKMCTGKKTTT
47          SMYYRYKRCYYCYAARYYKYYWYKKRCKKKCTTKGGRYKKGYGYRKKKYWKMTCKKKYWC
            ********. *  :: *   ******  **. .   ***. *.*********   **

42          GAKRYKKYGRMRRYRKYWYKWRYYKKRGTTYRKTTATYTYMGCGCRRKARRYMKMYRWKY
47          TCKRCTTTRRCGAYRKYWYKTACYGTRKWYYRKWYMYYCYCRGCGRRKWRRYMKMYRWKT
             .** ..  *   *******   * .*   ***    * *     *** **********

42          RRWMRWKCCRATATCRYYKKYATTYYKACACTGTRMKTYKKYKTKKRWYRGWWATAGCYK
47          RRWMRWKTTRGCCCMRYYTGYMYAYYKWYGTCTGRMKYYKKYKYKKRACRKWWGGRAAYK
            *******  *. .  ***. *  :***  .    *** ***** ***  * **.  ..**

42          KKYKWKRKYAWCTMKAWAACCWKRYKGRYYYRYRWTKRKYKMRRRYWYKWMMMYYKRGWY
47          KKYKWKRKTRTYCCKWWRRTTWKRYKKGYYYRYRWGGGTYTARGAYWYKWAMATYKGKTY
            ********      * *    *****  *******   .*. *  ***** *  **   *

42          KKRMYYYTRSMKMYKMYRRKRYKKKWSKYCARKMRTYCTKKRWKMRARTMWYYKKRWKRK
47          KGGMYTYKRSMKMYKCYRRKRYKKKWSGTYRRKMRCYTWKKRWKCAGGKMWYCGKAWKRK
            *  ** *.******* ***********    **** *  *****  . .***  * ****
```

```
42          GSYWYTYGRYWAGRKCYYMGYYKWWRRWYYRRRGATGRKRRKKYYRKWYYYWMTRMTYWK
47          KSYWYKTKRYWCTGKMCCMRYYKAWARWYYRGRRCAAAKRRKKYYRKWYYCWMYRMKYWG
             ****.  ***.  *   * *** * ***** * .:. ************ ** **.**

42          RKGRGKKAYARKTTTTGGRGGCTKRKMRMKSYYWGGWMRTKKGGKRGRTMMWSAAYCRCA
47          AKRRKKTTTRRKCGCATSGKKYKKRKMRMKSTCAKRWMRWKKKRKRRGYMMWSGCCYRSR
             * * *.:  **   : .    .********     *** **  **   ****..  *.

42          RWRRKKYYKKMYYRKKRRYMYRRRYYWKRKRMGATRGARKYAYTTCRRYMWYAGGMRRRW
47          GWRRKKYYKKMYYRKGRRYAYGGRYYWKRKRCARGRAWAKYGYKCTRRYMWYGRRMRRRW
             ************** *** *  ******** .  *.  **.*.  ******.  *****

42          GYRYKKCRYWGKCCCTRKMSGRWGKWRRRAYRKSYKRKKWKKRKKKCYMYKGARRWKKKR
47          KYRYKKMRYWAKATTCRKMSRGATKWGARGCRKGTKRKKWKKRKGGYYMYKKTGAWKKKR
             ***** ***.*.   ****    **  *. **. *********   **** :  *****

42          RKMRGGWWKRTCKTGGWYWYWRRMMKMYYRRRMRTKKWCWRKGCGRRWRKKWRKTGCCYT
47          RKCGRRWWKRGYKATAWYWYWRRMMKMCYRRRMRWTKWYWRKRMRAGTRKKWRKCCAMYK
            **    ****  *: .*********** ****** .** ***      ******  . *.

42          TARTRRKYYKYRKKKWRRMRRSMMRKCCCGCYRAACMRRGTKKKRKRMYKRGGYMRKMMM
47          KMGKRRKCCKYRKTKWRRMRRSMARKAYYKYCGWWSMRRRYKKKRKGMYKRAAYCRKMMM
            .  .***  ****.********* **.        .***  ***** ****..* *****

42          WMKYYGRRRKRKKWKYKRYYYKYRRKMKRMRKKRWMYRKRYKMYYRMMKCKKKKYYCTRW
47          WMKCYKGRGKRTKWKYKRYYYKYRRKAKRMRKGATMYGKATTMYYRMAKTKKKKCYYYAW
            *** *  * **.************** *****   ** *  .***** * **** *   *

42          YYAKTMKARRYKYGYGCAYRWMRYRMWARKKCYKYTYYSYYYKKTGRYKRGYRGRRKKRG
47          YYRKKMKMRRYKYRCKMRCRAMRYRAWCRKKTCKCWYYSYYYKKAKRYKRACAARRKKRR
            ** *.** *****      * **** *.***  *  ********: ****.  .*****

42          CYRMKKATRKWMRRSGKYTYGKYRTGRKKKTYRKRWRRMKKWKYRYRRKRKYSKYAMYTC
47          YCGAKKMYAKAMGGSKKYCYTTYAGARKKKCTRKGWRRCGKWKYRYAAKRKYSKYRMYYM
                **   * *  * ** * .*  .****  ** ***  ******  ******* **

42          TATWYAAYRRKMYYKRYKRYKRWMKGRGCMMYARKYKAKWRKKCAGCKKKYKGMCTWWYR
47          CCCAYTTCRRKCYYTGYKRYKATMTARRYMMYMGKYKRKWRKGYWRYKKGTKAAYGWWYR
             .  *:: *** **. *****  *..*  ***  *** ****     **  *.   ****

42          WKYYYRYRMYKSCTACWWSGAATGAAKRYYYRSYWKTGGTKYKYYYMKYYWKRKKAYYKK
47          WKCTTAYRMTKSTWWYWTSKRWKKMRKRYYYRSYWGYKKYKYKCTTMKYTAKGKGWTTTK
            **    *** **    * *   .   *********     ***   ***  * *    .*

42          RRYCCRYGAAWRRRWRGCCYYYMRKCRCKMARYWMMRKRRKGTCATKWYKTCCGAAYRRR
47          RRCATGCCRWWRRRWRKMMYCTMRGTGTKMGGYWAMRKRRKKWMRWKWTGYYMTGGYRGG
            ** .      ******   *  **    **. ** ******     **      ..**

42          RRRMRYATKWYMYRYMRKRYRWMYSSWYMKKKKCKWKWMKKKKYMYKYCGGRYYWYRKGK
47          RRRMRCGCGATMYRCMRKRYRWMYSSTYAKKGGYKWKWMTKKKTCYKYMRKRCCWYRKKK
            ***** .    *** *********** * **   *****.***  ***   *  **** *

42          WMTACYGAGKYWAYACRRKAYTKWWYKKKKRWKRYKRKRCYYRGYMKGRYRRCYYRKYYA
```

```
47        WCKGAYTTKGCWMCCMRRKCCWKWWYKKTKGWKRYKRKRYCTRKYCKKGYARYYYRKTYR
          * ...* :   *  . ***.  ******.* ********   * * *  * * **** *

42        RWKCRKKYKYYGYYSRRRMYKMGYMRYRYWRAAYKRWYYARWAMRSTYRRCGCWKRWRWW
47        RAKYRKKYKYYKYYSRGGMYKMKYMRYRYWRMTTTRWTYRGATMRSCYGGTKYWKRWRWW
          * * ******* ****  **** ******** : .** *   :*** *     *******

42        WKCGGYCMKRRSKYGMMRTASYATRKKKMYGCKRTRRKGRKAGMRYYRKTTKYRRYRYKR
47        WKAATTTAKRRSKTACMRGGGYWCAKKKMYAMGRWRRKRAKWKMRYYGKKYKYRRYRYKA
          **..    ***** . ** ..*   *****.  * ***  *  **** *. ********

42        TATYYRMTYKYYRWYMMWKMMRKMYYR
47        YMYYYRMKYKYYRWYMMWKMMRKCTYR
             ****.***************  **
```

The pairwise alignments of two accessions 42 and 48

```
42                  YWKWCRRYMKKYMYGTGTRCRYRAYTGSCKWRYRCGYRYKWYRYSKWYRWWWYYRTWYYG
48                  YWKWMRRYMKKYMYRYSKRMRTRRYYRSMKWRYRTTTATKTTRYCKTTRWWWYYRYTCYA
                    **** *********  ..* * * *  * *****     *  **.*  *******   *.

42                  CMYCTTYSMKARRKMRMKCMRRRYCACTCCYRGYRRYMMMYRWKYYYYMYYRMMKKWRWR
48                  MMCTCCYSATGAAGAGAGTCRARYARMYYYCAKYRRYMMMYRWKYYYYMYCAAAKKWRWA
                     *    **  ..         * **.        *****************    *****

42                  MGWRCYRRYYKKWKMMWRKMYYMYTRRRWMSARYKSYRYWYYYWGTRRWCCCATWYKRTG
48                  CATGTTRRYYKKWKMMWRTAYYMCWRRRTMGRAYKSYRTWTCCWTGARAMMTWWAYKACT
                     .    ************. ***  *** *.  ***** *   *   *       **

42                  RKCSRRKWYMRYMRYRKAGCATRWKCWMAKKYRMRKRYMKCYRMKMYYKMATTCKMKYRR
48                  RKYSAAKWYCAYMRYRKTCTCGATGYTMTKGYRAGTRYMKMYRMKMCYKCRWWMKMKYRA
                    ** *  ***  ******:  .      *:* **  .**** ***** **     *****

42                  RWYYRRYKKMMKYGCWWYWWMWRTMRTTKYKSKYKYRYGGRKYRRCRSWGKKRWKYKRRY
48                  ATYYRRYKTAATYKYATCWWMWGYCGACKYKSKYKCAYRRRKYRRMAGTRKKAWKTTAAT
                      ******.  .*     ****    : *******  *  *****  .  ** ** .

42                  RRYRWYMTKCTWRMYRWSYMWMYYRTRKMYTRRMRWRCYRYWTTMSMYWKGRYKTCMTKA
48                  RRYRWYMGKYWWRMYRWSYMWMTYRWRKAYARRMRWRTYRYWCCMGMYWKAGCKYTCYTT
                    ******* *  *********** ** ** *:****** ****  *.****.  *    .:

42                  TAMYYYYRYMMKGTTAGTAWRCGKRGCCKRRWYRYYMRRCRKRYCYYMSGYWTRKAKRYT
48                  GCCCTCYRYMMKTCWRRYRWGYAKRRSYKRRWYRYYCRASGTRYSCCASTYWCRKRKRYC
                     .    ******       *  .** . ******** * . .**.   * ** ** ***

42                  RWWWWWMKWRTKYKKKSWTRKRYYRRRYYRKYSAAAKYYYMMGRYTAATKAMTTGCTYWR
48                  RWWWWWMKWRCKYKTKGTWRKRYYRRGTTGKTCRWMKYCTCMKGYCCGCKRMKARYCCTA
                    ********** ***.*.  *******    * .   **   *  * .. * *.:

42                  KRRKYRWRWKYYYKRWYRMMYRTMYGKKYRMRYYYYSRRRYGCRYCTMWARMRKYYYKWK
48                  KRRKCATGWGCTYKRWYRMMYRCMYRKKYRMRYYYYSRRRTTTRYAGCAGRMRKYYYKAK
                    ****    *   ********** ** **************   **.   .******** *

42                  GYWRSWRKKKRRRYRTGGCKWGRCKCTTGARRMCWKRRWSYTTCYWWKRKWWSYYYYKKA
48                  KTWRSWGTKKRRRYAWKRSKTTGYKAAWRWRRMMWKARWCTCGTYWWKRTWWSCCCCKKW
                      **** .******    .*    *.:   *** ** **.    *****.***    **

42                  GYRCTTAKCCYYKYAGWTKRKYYYYKWRYKYYMYRRKYMTSRWKAYKKRARYRKMMWYKK
48                  KTRMYYRKMYYYKYGTTGGRGTCTTKTRYKYYMCAGGYMASRWKCCKKRGRYRKMMATKK
                      *    *  ****.    *     * ******    **:****. ***.******  **

42                  WWMYYYWKKRYKATGMMSKTGGGGKRRTYRCYYMWMWAAWCYCSRKGMKRRKYYYYRACW
48                  WWMYYYAKKRCTWYRCMSKCRKKKKAGGCAAYCCTCATRWYCYCAKAMKGRKYYCCRGTA
                    ****** *** .    ***     *     .*     : *   . *.** ****  *.

42                  MRYYKAKRKKAAWYMCGYKKMYYAGWYYYKGRYRGGCCRGKRRRGCYRMKCKCKTKRWMR
48                  MRYYKCKRKKCCACCYTCKKMYYMRTYYYKKRYRARYMAAKAGRRYCGAKYTTKCKAAMG
```

```
        *****.****..      *****   **** ***.    .*  *     * . * *  *

42      RGRSSYKYRRKRTTTARKTCGKKKYYRYSAWKRWRRKMGRYRKRRKRMKYYYKYATYWYC
48      GRGSSYTTGAGGCACTRKATAKKKYTRTSGTKGWRRKCKGYGKAAKRMKYYYKYMKCAYG
           ***.      : :**: .**** * *. * ****   * *  ********* .  *

42      MTYMTMCCAKYTGTYYRACRYKRKYYYMRWYCYYWYRRYSKRYAWYKYRRYKGTTGRTKY
48      CCCAWCMYWKYKRWYYRRYGYKRKYYYMATCAYYWYRRYCKRCGTTTCRRYKRYWRGGKY
                 **.  ***   ********   .*******.** .  . ****      **

42      KCAYKRKKRYKKYYMGRCRMRRTRYRSYKWRYCAKKYRYYGTCYCYKRGRCAKWYYMRYR
48      KTTYKRKKRCTTTYMRRTRCGAKGYRSYKTACYRTKYRYCRWYYYYKRSRYWTATYMRYR
        * :****** .. ** * *   . *****     .****    * ***.*  .  *****

42      WYGRRWTMKMYRKKRGCRKRWYMGYGKRTKKRGARYYYGMYKKYKKKWYMWACKKGSWYK
48      WTARRWYCKCCRKKRKYGKRACATYKKRKTGRRWAYYYSCYKKYKKTACMWGTKKKSWYT
        * .***  *  ****   **    * **.. *   ***. ******.  **. ** ***.

42      KKMYWRRWYRTMSYMYYKWCTRACARWWRWKRMKCKSMRKTCAKMRTCCMKRWMRYRKYG
48      KKCYWRRWYRKMCCCTTKWYKRRYWRWWRWKRMKTKSMRKWYRKMRWYTMKRWMRYRKYK
        ** *******.*.    ** .*   ********* *****   ***   **********

42      KRKKMYMKGYYGSKWYCKGWARRKRCKWMKKYWYSCCYWYMMKRKKMYWYRCCTTCAYCR
48      KGKKAYMKKYYKSKWCTGATGRRKRMKWMKKYWYCTAYTYMMKRKKMYACGTYAATTTTA
        * ** *** ** ***   . .**** ********. .* *********     :: :

42      MKWTAKCCYKRRRKKKRKWKRKRKKKTYRWKKTYMYAGWYGKKRMAYYGAGMKKYRATTT
48      MKACCKYYTGRRRKKKRKTKRKRKGKAYRWKGKYMYMKWYRKKRAGCCRMKCKKYRGAAC
        **  .*    ******** ***** *:**** .***  ** *** .      ****.::

42      GRACRGCYGGYMWRYGRYSGAAYKKYCTTGRMTGGCYCTTTAWKRRGRYWMCGCCMRTKM
48      TRWYGATCKRTMWRYAGYSAGGCGKYYGARRMYRRYYYCWCGWKRRKGYWCTAYAARCKA
         *   .     ****. **...  **  : **    *    .****  ** . .  * *

42      KGGKYRRCGATTKKGAKKMKRKRMKKWYRRWMKMGRRKKRKRMYKKYKKTKKWCGMYMKM
48      KKSKYAGMCTGKKKAGKKCTRKRMKKWTRRWMKMCRGTKRKAAYKKYKKKKGWYSMYCKM
        * .**    : .**..** .******* ****** * .***  ******.* * .** **

42      MKWMKMWMAYMTGYRSMYRKYMTRWYRCAACAGAARMRKKRKGKKMRYYYTWYYTGAKWY
48      MKWMKATMWYCAAYRSMYGKYCCRATGGCWYGKRRRCGKGRKKKKMRYYYGWYCGTCKWY
        *****  * * :.***** **  *    .  .   *  * ** ******* **   .***

42      TCAKRKKWKTCMRRWMSSRYYTRKWYYKRKRYYTTYAYMRRRKRYKRGYRYYWWKRGKWY
48      CTGKRKKWGCYMRRWMSSATTAAKAYTKGTGYYKCYTYMRRRKRYKGKCRYYWWKGKKWY
          .*****   *******   : * * * . **. *:*********   ******  ***

42      YYYYRKMKKKYGGAWGGKRGYRATAYYYWWMYWKMYKYYGMRAAAKTCKAWRKKKYRKYY
48      YYYYRKMTKKYRTTTTAKRRYRGAGCCYWAMYWKMTKYYKCRWTGKYYKWWRKGGYGKCC
        *******.***  :  .** **.:.  ** ***** ***  * :.*  * ***  * *

42      KKTYAKRRGYKKKMMRYKTGYKYAACRCRKKTYRKRYKRYYYYWWMKTASMYKTCAYRRK
48      KKACMKRRRYKGKCAGTKGACKYMWYGTGKGWCRKRYKRYYYTTACKATCAYGKATTRRK
        **:  *** ** *    * . **      *   *********   *::. * ..: ***
```

```
42      CGMCRCTYGKTCYKCCYKMYRYYRKWYYGYYYSTKKYGTGYYRCRTTGATKKKTWCAYAC
48      YKMTRACCAKAAYKTYCGAYRTTGKWYCAYCYSKKKYRYRYYRYAACRWKTTKCTAMTRY
          * *.  .*:.**     **   *** .* **.***   ***  :   ...*  .

42      AGGKKKGYYRYRRYRMSAMGCKKKKRYCYWKWKRRMYMGYKYKKRWYWKSRYASYYYRKG
48      RRKKKGKTYRCRRYRASCATMKKKGRYYCWKWKRAMTCKYKYKKRWTWKSRCGGYCYAKK
           **   ** **** *.   *** **  ***** *   ******* **** ..* * *

42      KARMKWGWYKKMYRRWRWKYRYKRKRRCYKMYYYYGRRAYYRYYKRARTRCKYSRTGGYY
48      KCGMKWKWYKTMCAAWGWKYRYKRKRRYCKCYYYTRARWYYRTYKGGRCGYKCSRWRSYY
        *. *** ***.*   * **********  * ***   * *** ** .*   * **  .**

42      KYYYRMCYGYSYKKYWWMGWKCRYGRKYRRYWCCRKKRMRKKRMMRAKWYMKMYYKRGWY
48      KYYYGCMCKYSYKKTTACKWKGRYKGKYRRYWTMGTTGAAKKGAAARGWYAKATYKRKTY
        ****     *****     ** **  ******   ..   **      ** *  ***  *

42      YYMKAYYRSMWKYKKGGWSKYRRKRYKKKYRKKSRYYYRYRKATCAYGRMWRYWYRYKKR
48      YYMKMTCRSMAKYKKRKACKYRRKRYKKGTRKKCGTYCACRKRWMWCSGMWRYWYRCGKA
        ****   *** ****   .*********  ***.  *   **     .*******  *

42      WKRKSYWYRYRRRYTKCGYMYYTCYCKMRKKSKAMCACCYRWMTAKYKKKSYRTTKRSRY
48      WKGKSYWYRYRRATKKAATAYTYSYSTCRGKSKCMYMYTYRWMKGKCKKGSYAYYGRSRT
        ** *********  .*..  *  .*.. * ***.*    ****..* ** **    ***

42      YTYGYMKWRRYYYKYKYMYYRRRKWKYGKKWRKMAYARYKYKWTWMTTGRARGWMTKMRR
48      CWYKCMGAGRYYCKCKYMCTAAAKAKYAKKWRKMGCRAYKCKWCTCAYAARGAWCYKMAA
          *  *   *** * ***     * **.******.   ** **   : .   .*  **

42      RYWGRKYCTRWRRRYKKWKMKRTTAGKCRKYTCCRRRCMMRRKGGRAMGYYYRYRKYMYK
48      GYWAGKCMKRWRGATKKWKMKRCKTAKTRKYYTTGAAYMAARGRRARMRYYYRYRKYMYK
         **. *  .***   *******  .:.* ***       *  *     * ***********

42      WTGWYRYYKTGYTRCAMRTKCKYWYKSGMRRAARYKRRKWKCTRYKKYACMTGGCTWKRR
48      AYRWYRYTTYRYYRYWCGKKYKYWYTSRARRRRGYKRRTTKYYRYKKYRYMKRTTCWKRR
           **** .  * *    .* ****.*  **   ****. *  *****  *.    ****

42      AKYRRCCCRWRGTCYKTYAMKAMYKRWMRAYYYTYCTGTCYYMTAYATGGRKSKKGMWKY
48      CKCAAYMYRWRRATCGYCTAKMMYTAWCAGYTCYCYYKYMYTMKGYWGRAGKSGKKAWKY
        .*      *** :     : * **. *  .*         * *..*   . ** *  ***

42      WKSYKGCTKRACKGATWKMKYYWYWSKSRMYYMYYRMYKRSTWYYKRAGACCYKYRRRRW
48      WTGCKAMAKARYKRGCWKMKYYWYASKGAMYYMYYRMCTRCAWYYKRMRRYYTTYRGAGA
        *.. *. :*   * . ******** **. ******** .*.:*****      .**

42      KKYAWKRGYWTCCCCCGYSKRKSYKRRSKRKSKKMYYRGSKAKKKKWSYSKYRSRYYRKR
48      KKYMAKGRYWCTAATTAYGKRKSYKRRCKRKGKKATTAKSKMKKKKWSYSKCAGAYYGKR
        ***  *  **  ..  .*.********.***.**     ** *********  . ** **

42      RCCCCMWRYYRKSMKYYRYKYRSMAKGATGTAMMMRKMMWRYKYRYWRYKKGRGWAYYTW
48      RYYMMAWRTYAKSMKYYRYKYRCARKKCYRWWMMMRTMCWRTTYRYWRTGKRRRWMTYWA
        *     ** * ***********.  * .    ****.* ** .*****  * * *  *
```

```
42          AAACSRRWTRKRWWWKMYTTMWWKCRRCAYMATMKKYSMCTYTRKTKRGAGARRKRYKTT
48          GTMYSRRAYRKRWWWKAYYKMWWKTRRTTTCWYMKKTSMTGYCAKKKACGARRRKATKCY
            .:  ***  ******* * .**** ** :    *** **  *  *.*  .. ***  *

42          MGRYYRKYSYATTKAGGYMARYRGGWYGACAMYYYRWYKRMAARKMYRYRCSGYYGGKWA
48          ARRYTGKTCCRKYKCAAYMRRYRTAWTRMYRMCYYRWYKRMWGRKCTRYGSSRYYAKGWG
              **  * .  . *...** *** .*     * ******** .**  ** .* **.  *.

42          KATCTCTYYKRYSRWAKMKSRYMKWYYKYRTCAYRKRRYWRKYYWKYMRARKRMKWKKKK
48          KTGAKYKYYKRTSRWWKMGGGCAKWYYKYRYTGYRKARYWRKYTTGYMRRRKGAKTTKKG
            *: .. .**** *** ** .   *******  .*** ******   *** **  * .**

42          WRYYYMKWKCTKAMSTKKRAGTATKRYWWRKKCTCAASYRMKKKWRRKRKARKKYKYRYM
48          TRYYCMKAKYATWMSYKKARKYWKKRCWWRKKGGTMRSYGMKKTTAAKRKWGKKTTYRYM
             *** ** * :. ** **     .** *****     ** ***.   ***  ** .****

42          KMATGYMWGWKKGMKRKTRYYRTKYKRTCTWSKGGCARRKMWKRKYCCTRRRWSCYKKCR
48          KMGATTCWKWKKKMKRKYATTRYKYKRCYCWSKKAMMGGGATKGKTTTCRRRWSYTTKSG
            **.:   * *** ****    * ****   *** .       * *    *****  .*.

42          KGKKKYRACWRWYRMYGATYGMAKWAAKSCGARYKYRACSRARATYGKYRTRAAMYRWYW
48          KKKKKYRRYWRWYRMTAGCCTMTKATTTGMAWGCGYATASRRRGGYKGTRCRTTATAWYA
            * *****  ****** ..   *:* ::.. .    * :.** *. *   * *::   **

42          YYSRGATTTYMKKGCKYRKYYKRCCGCTKYWYSYAKKKTYKKMKSWRWYRMYKSRGKYRM
48          YYSRKGCWWYMTKRYTYRKYYKGGTTTAKYWYSYRKKGKYKKAKSWRWYRMYKSRRKYRM
            **** .   **.*  .******     :****** ** .*** ************ ****

42          YACGCRWKRWCCAYRWSYWKYYTKKKMYGRYWWCRGRKYMRKMYRYCTRRTTKARKCRCM
48          TGTAAAWKRAYYRYRWSYWGYCCKKKMYKGTATMRRGKTMRKCCGCAYRRKYKWRGTGTM
             . .. ***    ****** *  *****      *  * ***    . **. * *    *

42          YRCWKKRRYKWKRMRKRKYKAGTCAMWMGKWKYYRWYRKYWRYMRMMRRKWGGYKRKKCM
48          YRYWKKGRYKWKRMRKRKTTGAAAGATCKKWKTYRWYRKYWRTMRCCGGKAKKTKRKGYM
            ** *** *********** ...:..    *** ********* **    *    ***  *

42          RMKKYYWWRRWARGATGYMRRKRRMKKKYYRKTKMRYWMKRAYRMRGYRYAKYRKTYRKC
48          RMKTYYAWARWRGRCAATCGAKRRCKKKYYRKYKARYWMKARYGMRKTRYRKYRKKTRKT
            ***.** * **   .:.    *** ******* * *****  * **  ** ****. **

42          RWKYKYRRRYWWYAAAMRYYYKYKCATMKAYKARRYYCTGKYYRGAKRRKGMMYKRRTYY
48          GWKYKYRRACAWYRMMMRTCCGYKYRCMKCCKGRRYCACAKYYRRWKRRKAMMYKGGYYY
             *******   **   **    **   **. *.*** . .****  ****.****   **

42          WRTGGATRGAYCCCCKYKWYMKYWRGCCRYYGRRYKRRWKYRYYMTARAYRRKWRKCCYR
48          WRCKARGRAWYTTMTKYKWYMKYAGKTARYYKRRYKRRWKYRYYMGCRGYRRKWGGYYTG
            **  .  *. *    ********    .*** ************* .*.*****

42          WKTWRKTATWRTGMKCGRWGGYKRMGYKKRSYRKKWMRKTMKCYGWYARWKARMWKKKRT
48          AKGTAKWRKTGGKAKYRGATKYKRMKYKKRGTRKKWMRKGCGYYRAYRRAKGAMWKKKGC
             *   *  .     *      **** ****. *******    *  * * *. *****

42          RRKMRWWTKYCRTCTKCGGYWYRYMYWYWMKMYMRTKKRWRSKGGRRWKKTGCCRTTARR
```

```
48          RRKCGTTCTTYGAGGKTACYWCGCAYWYWMKMCARATKRWRCKKRAGTKKCCAMRKKMAR
            ***     .   :  * . **    *******  *:.****.*     **  . *..  *

42          RKKMRGTKWRSMTKKKTMTRCYRMRKRYAMKMSYRKKYRMSRYKRWMRKAYSWRMAWCKC
48          GKKARKYKTRSMYKKGKMYGYYRAGGAYTMKMSYRKKYRMSAYKRWCRKMTGTGMTWYGY
             ** *  * *** ** .*   **    *:************ **** **   .  *:*

42          TYKYYYYKRYKCCATCTGACKRYKRYYYYRTATKMRMRSRRYKRMRKKRWMYKKRKYKYY
48          YCKYTYYKRYTYAMWYYKRMKRYKRYYYTGCCCKMRARSRRCKRMRKKGAMYKKGKTKYT
              ** *****. .       ********   . *** **** ******  **** * **

42          KMYYRMKMKKKKKYKGTWCMKKTAACGCYRYMWTAKAYRKYCRYRWYTGSMAKSRCCTGW
48          TMYYRAKAGKKKKCKRKWYCKKWGGYRTCAYCAYRGRYRKYMRYRWYKRSAMKSRTYCRT
            .**** *  **** * .*  ** ..     *      **** *****. *  ***

42          TYTMYYMMYMRWMWTMMAACWRGGAKYRAYRRYTAKMRRYGGGTGGGYRYRKGCSKTWKY
48          YCWMYYMMTARWMWCCMRRYWRAAGKCARYRRYCTKMRRCARTCAASTRYRKATGTYAKY
               *****  ****  *   **...*   **** :**** .   ... ****. ..  **

42          WWYYTYKKGCKCCCWGKKTRTYKGKYYMYRWKWMYKKYWYWKAKGWKKMYKRSWYMRYRK
48          WWYYWYKGKTKYMYWKKKYRKYKKKYYCYRAKWCCTKYWYWKWGAWGKMCKRSWCMRYRK
            **** **   *   * ** *.** *** ** **  .******  .* ** **** *****

42          MRYTYRYRWWMYKYKRYRAWAKRSMTKRYTTTWTTCKRMRKMYW
48          CGCGTAYRTAATKYKRYRRARKRCCCKRYCCKTCGYKRMRKMYA
                  **    ******   **.  ***  .    *******
```

The pairwise alignments of two accessions 42 and 49

```
42          KYKSYMRYKMYRWKYKRYRYYKRTSMRYYYRRRCMCGGRMYYYYRMYYRMKWRWTAGRMK
49          TYKSYMRYGACRWKCKAYRTCKAYSCRYYYRRAMMTKARMYYYYRMYCAAKWRWKMRACG
            .*******   *** * **  *  * ******  *  .*********   ****.

42          YRYKWKKGRRYMMWRWRWYYRRYMSTYYRGKKYRYKMKKYYWKWWASMKYKYKRGCCKMK
49          TRYKWKTRGATMMWRWRACYRRYMGATTGRKKCGYTATKYYWKAWCSMKYKYKRRSYKMK
             *****.    ******  *****.:    **  *. .***** *.******** . ***

42          ARYKRSWTRWYMCGCYTYYRMTMYWKRYKCRWWGRYYYGRRYRKWKYYYKCYRMRMKWWM
49          RGCKAGTKGWYMYRYCYTCRMKCYWKRYKYGWWKRYYYKRRYAKWKYYYKTYRMGAGWWM
               * . . ***       **. ******  ** **** *** ******* ***   ***

42          YWRYTGAMRMYRYYMGMKRYGGRKMYRRYASYGTTGYYWKCGAMWMYYRTRKMYMYYRYR
49          YTRCCAGMAMYRYYMKMKRCRRGKMYGRYWSYRACKCYTKAKRMWMYYRWRKATMYYRYR
            * *  ..* ****** ***    *** ** ** :   * *.  ****** **  ******

42          RMRWYMWWWMWYMMWRCRYMWYMKWRRKRMRRWKAKMGCRWCSYYWKRWWYRWACYCCAC
49          RMRWYMWWWMAYMMWRYRYAAYMKWRRKRCARWKTKCKMRWYCYCATRWWCATCYYYTGT
            ********** ***** **  ********  ***:*   ** .*  .***   . *  .

42          MMRRRKRYMKWMMACTYYRGYWMWWYKSRRRCYRYKCRYWSGKAGMMMKCACCWGSKYRW
49          MARAGTRYMKAMMMMYCYRKYWMWWYKSGRRYYRYKYRYWSTTGAMMMKYWYYWRSKYRW
            * *  .**** **    ** ******** ** **** **** ...****    * *****

42          TKATKYMRRRTWMRGYYYWRAMYTAARTKRWYRYRGTYYWKSRYCTATGYMMWYWYMYRK
49          WKGAKYCGGRCTAGRCTCARWMYWRGRWKRWYRYRRCYYWKSRYTGCCRCMMWYWYCTGG
             *.:**   *         * **  .* *******  *******  .   ******

42          RYRMWMAMAAYKWRYGYMGGARATGRATTYRTAAGYRYRKWWKRKKYYKTCCGAMARRRW
49          RYRAWMTCGGTTWRYKYMAATAWCKRMKWYRGGGAYRYRKWWKRGGYTKYYYKMMCRRRW
            *** **: .. .*** **..:    * . ** ...*********  * *     *.****

42          RCGWYRRYGKRYWCYGATAYRRYKRTTATYKWAYWCKMKKRKKKRKRYKKYYMRSRYGKT
49          RYRWYRRYKKRYWTCKTCTYGRYTGACGAYKWMYWMKMKKRKKKRKRCTTTYMRSRYSGC
            *  ***** ****   : :* **. : .:*** ** *********** .. ******.

42          WKYYACAAYRRKKKCGYRKTYYARYKKMMRKKAAARRGCACAYMKMYKYRRWRYKMYMRK
49          TKCYGYRCCGRGTKGRYRKKYYWRYKGACAKKGTGRRKYGACYMKMCKTRRWAYKCCCRK
             * *.  .  * .*  ***.** ***    **.:.**  ...**** * *** **   **

42          KRRKRTAKKRRRMSMTYKYRRKKKKKKRTAWWYYKWTMYKMCCYASARKYMRYRAMWYAG
49          KRRKRKMKTRRGMGMYYKYRRKKKKKKRCGTACCKWYCYKAMATRGCAKTCGYRMMWTCT
            *****. *.** *.* ************ .    **  **  .  .. *   ** ** .

42          RGTCARYRYMYYWRAYRYKYRYMRWSRWKRRCGKKKKYYRKKYAAGKMRGRYYKKRRGRG
49          GSKYWGCRYMYYAARYRYKYRTCGWSRAKRRTTGKKKYYRKTYMMKKAAKRYYKTRRRGS
             ..    *****   ******   *** ***   *******.*   *   ****.**  .

42          CKRMKSYRTYWWMYYYKWYKWKKSKGCGRGKRRYYWATCKMYYRTGACYMWRRYMAYRRY
49          YKRMKSTAKCAWMTCCGWYKWKKCKKYAGAKRRYYARATKMYYRCRCTYMWGAYAWYRGY
```

```
                 *****  .  **    ******.*  . .*****  : *****  . ***  *  ** *

42              YWRRMRCACCKCAATMYKYYWYKGMYYYYKYGYKSKMYYRSRKYMRKKRKYRWYKWYKKR
49              YWRRCRSGYMKYMWYCCTTYWYGRMYCYTKYRYGCKMYYRSRKCMRKKRKYRTTGACTGA
                **** *..  *      . ***  ** * ** * .******** ********     .

42              ARYRYRKMSMTKGMYRYYYKYCKRYKYYMCACTYYYKGTCKYWACTATKWSGMSRRGKYK
49              TRTGCRKMCCCKACCRYYYKYYKRTGCYAGCYYYYYKAAYKYAMYAGCKWSKMCGGRKYK
                :*   ***.  *.  ****** **   *  .  ****.: **   :. *** *.   ***

42              ACKRCCTKAMKKTGTKKGRCKYMGATYAACMYKRRKRGRWRTRYGCRYRCCAMWYYWACW
49              RYKGYTCKMMGKCRYKKRRYKYMAGCCWWACYKGRKRRRWRCGCSSAYAYYGATTYWGAA
                  *    * * *   ** * ***..    . ** *** ***   .. *   .   **..

42              MKRKRRRYWATYCRKKMRYRRMYYYAYMWKMRKRGMYRGWMWCYKSYAKRRKWMCSYYMY
49              MKRGRRGTTGYYYRKGCRTGRMYYTMYMWTCRKRKMYRTWMWACKSYCKRRKWAGSYCMC
                *** **   . * **  *  ****  ***. *** *** ***. ***.*****  ** *

42              RCWCAAWAGRYMRKYKYKMMYMRRKYYYKAGRWYAGRYYCRKCMWRKYMAWMYRTGKTCA
49              RYASRRWTKRYMRKCTCKMMYMAAKYYYKMTRWYGRGCYYAKYMARTCCCWMTGYRKWSC
                *  .  *: ***** . *****  *****  ***.   *  * * *.  .**    * ..

42              AKYRTRKAKYYRYRWRRATRYTGCKRRKYYRWMRCGCYYKWTKKKKKYKGMYYGKKKYRW
49              RKYRKRKCKYYGTRWRGCWRYYSYKRRKCCRAMRTATYYKWCKKKKKYKKMYYRKKKCRW
                 ***.**.***  *** . ** . ****  * ** . **** ******* *** *** **

42              RRACYKGRRRGCYRMKKCKATCCTCTRAWMRRYRSRKSRKMTGRKKKTWYAATRWAKRWR
49              RRMMCGAAGRRYCGAKTTKCGTYWMYAMAMGGYRSGKSRTMAAATKKGWTRWAGWRKGWR
                **    .  *     *. *.         *  *** ***.*:. .** *   : * * **

42              MWRMWKRYRRRRKAMYKYYAGAMTYRRCAWATAATKYYYRYKRWYRRRKRKKKWKKKMRM
49              MTRCWTRYRAAGTRMYKYYWKCCCCRRSRWGCGGKKYYYGYKRWYAAAKAKKKWKKKMRA
                * * *.***   . *****  .   **. *. ...**** *****   * *********

42              RRKRRRKRCGGCYTGGKRCRYRYRAYRTCTYMGKYYKYWGAYAGYRYGTKGKYYWTMMMM
49              GAGGGRKRMKRYYKRRTAMRYRYRMYRWYWYMKKYYKYWTTYGAYGYRYKRKYYWYMMMM
                     ***    *.  .  ***** **   ** ****** :*..* *  * **** ****

42              CTGKGCCRYSAAATGTGCMRRYRKRTCAGKKWACCAAYGCTCGYRRRYCCAGWYKCMTTC
49              GAAKRATRYSWMMYTCRMMRRYRKRWTWAKTTWMYRWTRYKYRCAARYYYRRATTAMGGT
                 :.* . ***        *******   .*.         .     **      ..*

42              KRGMKMRACGGGTCTCAAYRMGWWRKGYRWAMRTKKKYTKKTYKKKKACRACCCWRWKRG
49              KRKAGMAWMRRRWTYYRGTGMKWWRKATGATAAWKKGTCKKKYKKKGCTARYTTWAWKGR
                **   *           .  * ****.   :   **    **.****  .      * **

42              KMRYMSKSRYMYYMYYYMYKRKSACWTTYRKKYKKRTRTGYARMTWCCGYSYRRSYKRRK
49              KMRYCSKGAYMYYMYYCMCTRKCTMWYYYRKKYKKGYRYAYGGAGTTTAYGTGASYKRRK
                **** **. ******* * .**.: *  *******  * .*.      .*.   ******

42              KSKKSGKKWRGKWSKYRKRRRMWYRSMYYRYKMMTTYYTTAYWRRRSYMYYYGYMKSRAW
49              KGKKSKKKWRKKWSKYGKRRGAWTASMYYRYKACCGYYCWCYWGGRCCCTTYKYMKSRWW
                *.*** **** ***** ***  *  *******    **  .**  *.    * ***** *
```

```
42          WRWRMSMGKMWKKMKRWMRKKSKKTMKMKCCCYMRCGKWYKTTTTGCWRKYWKYYYWYYM
49          WRWRMSMKKMWKKMKRWMRKGSKKKMKAKGTTYMRAKKWCKKKCATTWRKYAKYCYTYYM
            ******* ************ ***.** *   ***. ** *.. :  **** ** * ***

42          MYARMRYWYKCKRRRRKTTGGGKKMWKYRCYKKKKRGTRRRKMGYWCRAYCSKGRWWYKY
49          MYRAMRYAYKTGGRRRKYYKTKKKMTKYRYYKGKKRKCAAGGMRYWYRCYTSKRATAYGC
            **  *** **   ****     *** *** ** ***      * ** *.* **    *

42          KYRATRKWRRARTTRSRWAGMYKRYMGMRYSKYTYRRRCTYGGCGKGCWTWKRRYWKGKR
49          KYRTYAKARRMAWYGCGWMCMYKRYMAAGYSGYCYRRRMYYATATKATAGWKRGCAKRKG
            ***:  * **     . *  ******.  ** * ****  *. . *.   ***   * *

42          GCSKKKTAATKKGGTMKRWMACGKRMSRRKRRMYKWRYKRKKKMYYAKKMRRKMMKMWMM
49          CMCKKKYRWYTKAACMKRWMRTRTRACGGTRAAYKWRYKRGKKMYYGKKCRRGMMKATCM
              .***    .*.. *****   .* .  .*  ******* *****.** ** ***   *

42          AYMRRSWKSMTRKWGTCKKKRGRYWRYAKCGCCAYKWAMRRMCSYWSYWRKMKGCCWWST
49          MYARGSAKCCAGTWKWSKGKRAATARYCKMTTGMYKACCRRMMSYWCTWRGMKAGTWWSK
             * * * *. : .*  .* **.   **.*     ** . *** ***. ** **.  ***.

42          YTTKATGTMWKCTATTKMMKAYKYWRKYMTGAYWKYRKYMMGCYSMRKMYRKMMYSWYCK
49          CYAKTCTCMWKACGWCTMMKRCKYTRGTAYSWCTKCAKYMMRMYCCRGAYRKMMYSATYK
              :*:   ***. .  .***  ** *    .   *  ****  *. *  *******   *

42          MYYYGCWMKKRKKYRKRYWYYMYGCKKGGGKRAGCCYMKWMWSKRRTKKKMRKMKYYYRG
49          MYYYAAACKKRKKTGKRYWYTACTTKKKKKKACASYYMKTCASTRAKKKKMRKMKYYYRT
            ****..  *****  *****     **   * ... ***   *.* .************

42          TAYRKWWMMYKKKRRWRMRRWMKKRWMMMRKSRSRMYRCYGCAGMKARMRYMYKYGYRYR
49          GCCGKWWMMYKKKRRWRMRRWMKKAACMMAGSRSGMTRYYRATTMKCRCRYATGTRCRYG
             .  ********************   **  *** * * * .: **.* **      **

42          YRYRRACKRWYYKTTAKKKTYYTRKYYKMGYYYYYWYAWMYMKKCMRRGYWYRYWMKMKK
49          CRCGRTAKGWYYKYWRKTKYCYKRKYYGCRYYYTYWYRWMTMKKYMRRKTWYRCWMKAKK
             *  *:.* ****   *.*  *.****   *** *** ** *** ***  *** *** **

42          MGCKWWAARMTKYMKTGYKKWRYWCGCAKRYAAYYKTARRRWWAGCKKYYKKKTYRRTWK
49          MKYKWWWWRCGKTCKYCYKKTRYWTAYMKRYRMYCKWMARRWAWRYKKYYKKTKCAAAWK
            *  ***  *  *  *  *** *** .  ***  * *   ***    ******..   :**

42          WKRRYKRKRKKMSTYKYRYARYYGRGCYYWYYSRKMRKYKYYSRSGYTKYYKYAARKKSR
49          WKRRYKRKRKKCSACKTRYMATCRGKYTYAYCSRTAAKYKYYSGSRYYKYYKTTWRKKSR
            *********** *: * **         * * **.  ****** * * **** : *****

42          WAKKMTKSRKKKWKCKKYKRYYKKKRARYWRYKYGYWKYMRTRKWYMKKYKWKRYWKTMS
49          WWKKMKGGGKKGWKYKKYKRCCKKKARRYWRYKCATTKYMRWRKATATKYKTKAYAKKMS
            * ***. . ** ** *****  ***  ****** .  **** **   .*** * * *.**

42          RKKCGTRRYWWRWKAKKKCTYKYRKRKKKYYMYMKKAKGKWATYMKAAAKYKKYMKYRKY
49          RKKTAGRRCWWRWKRKKTGGYGYGKRKTKYTCYMKKRKKKWWYTAKRWRKYTKYMKYRKY
            *** . ** ***** **.  * * ***.**  **** * **    *   **.********
```

```
42                  YKRCKYKYKKKKKCKTKGGGYCMSWRWYCGWYTKYRKYMYKRWTGYGAKRSWWCTGAAYR
49                  YKGYKYKTKKKKGAKKGRKRTMASWRWYAKWYKKYRKYMTKRWCATATKRSTAYKKMRYR
                    **  *** **** .*.       *****. **.****** *** . .:***   .   **

42                  WSYWWWRYKYYYKYMYWKRKYKYKYRYKYKWRCGGRRYYTAMTKRYMWKKTRGKKYKRMR
49                  WSYWWWRYKCTTKYMTAKRKYKYKCGCKYKTGTKKRRCTCRMCKRYMAKKCGRKKYKRMR
                    *********   ***  *******   ***     **    * **** **   *******

42                  YAYTCAKMWYYRRRMKKRYGCTMKMYYKRRYYCRRRWWKYRYGKRGRTWKRKKCRTTTWC
49                  YMYWMRKMWTYRGGMKKAYSGAMKAYYKRRYYSRRRWTTYRYKKAARKWKRKTMGKKCWA
                    * *   *** **  *** *. :** *******.**** .*** * .*.****.  .. *.

42                  KKYWMCRARYGRCKYRRAYWYRMRSYKRKKWCKYKCYYAKRRMWGKKKGKYYWWKYYYYR
49                  KKYWAYAMGYRRYKCGAGYWYRAAGTGRKKWYKYGYYYRKRAMWTKKKRKYYWWKTYYCG
                    ****     * * *   .****  .  **** **  ** ** ** *** ****** **

42                  YMKRRMMKMYYRRRRTKGAKWGKRRWYRWYWRKKMAYRYMWASWAGYGKMRGWYYKWTCR
49                  CAKRRMMKMCYRRRRATRCKWSKAGTYRWYWATKMWCGYCWMSWWKTKGARAWTYKACMR
                      ******* *****:. .**.*   ***** .**   * * **      *.* **   *

42                  YRRWGRWWWWKMTCAARRRYMRRRRYKTKGCTARKRRMRYYRTTRYKYRMYRGGWYYYKM
49                  YRGARRWWWWKAYMMWARRYAARRRYKAKAGGTRKARMRYTGKYRYKYGMYRTAWYYYKM
                    **   ******      ***  *****:*.  :** ****  . **** *** .******

42                  RGCYKYYRWKTGTTTMARKGMYYGCGRYYKYWWKYTMWKWMRGRRYRKWCAGYMWYRKKK
49                  RTAYKYYRWKCAWYKMMRKKCTYAGTRYYKYWAKCCMWKTCGKRRYRKWYRRYAATGTKK
                    * .******* .  .* **   *.  ****** *  ***    ******   *    .**

42                  YKRRTTCCCCKKGCMMWMYYKTRGWYYYMYYRSMWKYKGYKKYCYRRKKYYRMRYRKATA
49                  CKGAYYMYSYKKRYAMWATYKCRKTYYYMTYRSMWKYKKYKGTTYRRKKYYRCACATRWW
                     *      . **   **  ** *  **** ******** **   ********    .

42                  GYRMRYKYKKRYSYWYWKRWRRKWKKRRYYKKKRRAYWKMYGKKYKCRMRKRYKKWKYKA
49                  KCGCRYKCGGAYSYWYWKRWAATAGGRRCCKKGRRRTTKMCRKKYKYRMGGGCKKAKYKT
                        ***    *********  .   **  ** **   **  **** **    ** ***:

42                  WKWKRWYSWYRKAMCYKKYMMKTYKRKWWCKTRTTCRGWKYRKMYARYYYRWYYYKYYSK
49                  TKAKRWYSWYRKRATYKKYCAKGCKRKWTYKGRACARATKCRKAYWRYTTGWYYYGCYSK
                     * *********   ****  *  ****  * *: .*. * ** * **   ****  ***

42                  GKKYYYRWRRTKKKYKYWTRRKGGKYYYKAYYTKRSMTKMTYRYRRMRKRRYAAKMKKYK
49                  CKKTYYRWGGCTKKYKYWKARKRKKYCCKMCCGTRSMYKMYCGYRRAGGARYCGKMKKYK
                     ** ****   .******. **  **  *    .*** **   ***    **..******

42                  MYTTCWCKGMMRCKCTRYRRRYRRRTGWYKATTGKAKRKYYYYRTATYRRKMYKWTAMRK
49                  MYCKTWYKKMMRYGYCAYAGGYGRRCAWYTGCCTKWKRKCCCTGYCCYRRKMCKAGWMRK
                    ** .  * * ***     *   * ** .**..   * ***      .***** *   ***

42                  RWMYKRKYRYYKMKKKYRYRYRWKYKRKYAATTTARWWRKYRGKRATYYKRKRWWWWWMW
49                  RWMYKGKTAYTTMKKKTRCATAWTYKRKYTGCWWWRWAAKYRTKRTYCYKRKRWWWWWMW
                    ***** *  * .**** *    *.*****:.    **  *** **:  ************

42                  WWKKYKKKRSWYRYKGYRRRWGYSYRTSKGYRSKYYGMWSRCTGRATKMYYRKRKRWRWY
```

```
49        AWKKYTKTAGTYRYKKYRRGWKTSTGGSKKTACKYCKMWSGTWAACAKMYCAKRKRWRWY
           ****.*. . **** *** *  *   **   .**  ***   . .:***  ********

42        YYCYKKRYRMMYTMMKKMKYKYRMRYYRRACYCYCARMMTRYYYKMYRMYWYGGCC
49        CTMTKKRYRMMYCMMKKMKYKYRMRYYRRWMYATSRRMMYGYYYKMYRMTWYAKYT
              ******** ****************  *. . ***  ******** **.
```

The pairwise alignments of two accessions 42 and 50

```
42              YRMRMYYRRYCATRRTTYMMWRTWRKKRMMKRGAKRWRYMYYMCRYAYYYKKMYYYYYMY
50              YAMACYYRRYYWARGWCYMMWRGWRGKRMMKRRRKRWRYMYYMMRYGYYYKKMYYYYYMY
                * *  *****  :*   ***** ** ******  ********* **.*************

42              YRMMGCTYKKWRKMMYMRYRRCKRMGMSYCRYKSYWYRRYWYYYWYWYRRRMWKMTAYKK
50              CAAARTWYKKWRKMMYMRCRRMKRMRMGTTRYKSYWYRRYTTCCWYWYRRRAAKCYWTKK
                       *********** ** *** *.  **********    *******  *    **

42              WRASYYMWWGKKWYRRCGYRSWKKYRWMWKRRYRMKTAWMMRWWRRMRRRMKRYWRRRYM
50              TRWGYYMWWRKKWYGRATCRGTKKYAWMWKRRYRMKWRWMARWWGRMRRGAGRYWRRATA
                 * .***** **** *.  *. *** **********  ** *** ****   *****

42              YCCTRKWTYWKGRYYSMKYYYRYWRWRSKRCTYAMYWWWRWSYRKMMAWMYRRMRMYYRS
50              TYYKRKTKYWKARYYCMKTYCACWRAACKRTATCAYWWWRWGCAKMMWWMYRRMRMYTRS
                   .** .***.***.** *   **  .** : . ******.  *** ********* **

42              RRYYKSRACRMTKYYMRYKKWRCRATARRYWSWRYRKYCYYYWYYRTWTTYSRYSWRKRM
50              RRCCKSRRYGCATYYMRYKKWRMRTCGRRYTCWRYRKCAYYTWYYRCWAAYSRYSWAGRM
                **  ***    :.********* *: .*** .***** .** **** *::******  **

42              YKMKGMYMMKRRRRWYKMMKKSKRKYYMGYKMRRWKRRKRYACYWKRRKRKKMYKYKMKR
50              YKMKRMCMMKGGRRWYTAATKSTRKYCMAYKCARWKAGTRYCMCWGRRGRKKMYKYKMKR
                **** * ***  ****.  .**.*** *.**  ***  .**.  * ** ***********

42              RKKYGMKRRAKRKMMGYKKRKRKRAYCRAAKYMKRRAWYWRRKWTGAAYWYRMYWWMKYY
50              RKKYKCKRRCKRKMMACKKRKRKRGCMGRCKYMKRRMTYARRGTAATTYWYRATTACKCT
                ****  ***.*****. *******.    .******  * **  :.::****     *

42              MYMMKYARYRRKKKWRRKTTCYRRWYKYYYRWYYMAYYKKKYTWWYAAGRKKTRYYYYKC
50              AYCAKYWRYRRKKKWRRKKAATRRWYKYTTGWYYMCCYKKKTAWTCGRKRKTKRYYTYKY
                 *  ** ***********.:. ******   ****. **** :*  .  **..*** **

42              KKKRRRYYYKRCMKRRRKRRYYKKRGGRTYRGYRTATAGWRTCKTTRYSRGRRARRYTAA
50              KKKRAATTYKAYMGGGAGGGYYKKGRRACYRRYRATATAWGCTKYCGYSRRRRRGRYGTG
                ****    **  *       ****     ** **::::.*   *   *** **  ** :.

42              RKWMRRYKKCWSKYGYWWYYYTRGRWYRRAYYYCYTGRWWKWYRRYGACTAYYCRYKRKK
50              RTTMRRYKKYWSKCKYWATTTYRKAWTGAGCTCYYWTRWWKWYRRYRRYYGYCYRYKGKK
                *. ****** ***  **     *  *   .    *  *********    .*  *** **

42              KCCACGYYKYWWKRYSWWKWRATAAYKRKKYKRSYMMRYRSYWYKYRKYTMMMRKWKACA
50              GTAGMKTYKCWTTRYSWWGTRCATGCKGTTTTAGYMCRYRSYTCTYRKYCMMCRKWKGAC
                  ..   ** * .*****  *.::. * .. . .** *****  .**** ** ****...

42              YMKWKKKRMKRWRYMKMYRKRRYAKATTYKYWWYGYRKRKMCYKKKKKWYKKKYKKMRKA
50              YMKWKKTGCKRWRYCKCCRKRRYRTMCACKYWWYTCAGRKMTYKKKKGACKKKYKKCGKC
                ******.  ***** *  ***** .  : *****    *** *****   ******  *.

42              SYMYYKARKRWWRWSMYKCATKMWYYYRYKRRWRKYMRYKGKYKRKWKRRMRKYKWYCGG
50              CCCTTKWRKRWWAASMYKTCGKMWYYYRYKRRWRKTAGYKKKCKRKWKAGMRKYTWYTCA
```

```
                .    * *****  **** . **************   ** * *****  ****.**  .

42              YACMKYMMCKKKMYCKTCSKKWRMKWAWMWRRKAWRGMMRTWGKYRYKKCCYYYATYYRG
50              CRACKYMMYKKKMYYKWYSKKWAMGWGACARRKWAGRAMGWTKKYRYGKYMCYYTCYYAK
                  . **** ***** *  **** * *.   ***     *    **** *   **: **

42              WWCKYRCGWKYRTTAGCCTRRKRKMRRYGYYMKCTGRGYRKWRRSMTKMGWAMRCYRRMR
50              WWYTYRTRWKYRKKMTTTARRKGKMRRYRCCMKYGRAKYRKWARSMYKMKWMMRYYRRAG
                ** .**  ****..    :*** *****   **     **** *** ** * ** ***

42              KRYRKKKKYRYKRTGMCKGCTKRKRRYYKRKAGTATKAYYYRRKTYKKMKRYTKWRWMYK
50              GAYRKKKKYRYKRCAMYGAYCKGKRRYYKRTCTGTWGTYYYRRKAYKKAKRYYKWRWMYK
                  *********** .*  .  * *******..  :  :******:*** *** *******

42              GWRKYRYYKMYYRKMKKRKKYKKMRYCTACYCTYYSKCAGGYKMCGARCRGCCKKTGMYR
50              ATGKTAYTTMYYRGAKKRKKCKGCRYMYRYYACCTSKSWKRYTCTKWRYRRTTKKYKMYR
                .  *  * .****  ***** *  **    *.   **.   *.    * *   **  ***

42              RTTYWTYKKKSYRRRKGATRRMTYYGGYAWAACGCTYKMKYYRMRRKGKYKCAWCMKKWR
50              RYYYWKCKKKSYRRRKKRYGGMKYYKRCMAWGTATKCKMKCYAMAAKKKYKYGWYMKKWR
                *  **. *********     *.**      . . . *** * *  * *** .* *****

42              KWKKWKCWMTTCMYWKYYRWCRWRRRKYMKYMTAATYKKYYAKKKKKKSWRWYRMWKYWT
50              TWKKWKYWMWCTMYWKYYGWYRWRGRGYMTYMCCGYYKKYCRKKKGKKSWRWYAMTKCWY
                .***** **   ****** * *** * **.** .. ****  *** ******* * * *

42              RYRMYKSYKSCCYRRWTGARSYWKGYWRKYMYMRYWRRKMCCYKKRYKRWRRRWYYTKKR
50              GYRMCKSYKSMMCGAAKKMRSYWGKYWRKTMYMRTARGKMYTTKKGCKRWRRRWCTCKKR
                 *** *****      .  ****  **** ****  * **   **  *******   ***

42              RKMTRRYKYYYWMMRYKRYYGMMYWYWYYRWYRMKKYRKMRYAKARRSRRYKRKKACTRR
50              RKMKGRYKTCTWAMRYKRTCKMMYWYWTYRWYRMKKYAKMRYGKGGGCGGYKGKKGTCRR
                ***. ***   * *****   ****** ********* ****.*.  .  ** **.  **

42              RWSRKKTAACTKTWMGMYTRCRWGCKACWRYYYMRMYYRAMRRKRRYRCGCRRKCCTMYY
50              RWSGKKKRTTAKKWMKCCGGARWRYGWYWRYCTMRMYYAGAGRGRRYRYRMRRKSYYCYY
                *** **. : :*.**     .**     ***  ***** .  * ****   ***.   **

42              SRGTCMRAGMYKKYRYMYWRYKCKRKMYRYRKMWRKKWYRYMYRWWMYRKKWKYRYRAKW
50              SRACTCRTACCTKTRYMYWRTKAGRKMCGYRKAWRKKWYRYMYRTAATRGKTKTRYRGKA
                **.   *:.  .* ****** *. ***  *** ***********    * * * ***.*

42              WRSMGKRYMWRWRCAWAYKYMRMGKMWKYAARYYYMMKWKKGRWMYRRWRWGKRRSTKRK
50              TRCCTKRYATATRMTTTTGCMRMRKMWTTRMRTTYAMKAKGRGWMYGRWRWKKRRSYKRK
                 *.  ***    * : :   *** ***.   *  * ** *   *** **** **** ***

42              YYMYYRKRRKKYRMRYYRKKYRRYKMYKYRYMACASKMRRMGYKMYCRCKKYRTYMYKSY
50              YYMYYRKRAKKTGMRYTGKKYRRYKMYKYRYAWMRSKCGRMAYKCTTGTTKTGKYMYKSY
                ******** **  ***  *************    **  **.**     .*  .******

42              CYWTATCCTAYYKYGCTTRSMKSRYMKWYYKYMRYGYKKKYKRRYWRYCYYYWTAGYMRK
50              TYWYTGAYKMYYKTRYYCRGMGGGCAGWYYKYMRCKCGKKYKARYAGYMCTYTCTAYMRK
                 ** : . . ***     *.* .    *******    **** **  *   *  :.****
```

```
42      KYYKRYYKTWRYKGWKAAAMSRKKTWKKCTYKRYWWYYKYKGSCRMKKKKWRRRKKKGKT
50      KYYKRTYKGTRYKRAKTWWMSRGKYWKKGGYKRYWWYYKYKRSMGMKKKTTAARKKTKTY
        ***** **  ***  *:  *** * ***  *********** *  ****.   ***. .

42      TYRYRYMWYRGGMWWWKYMMTAKKRAAWYGAMRWMWAWYMKTGCGKRRWYTYKRMYAMRT
50      KCAYRYMATAKKCWAWKTACACKKRTTWYRGCRWMWCWYMKYRYKKRRWYGTGRMYGMRA
        .  ****      * **   :.***::** . ****.****    *****   ***.**:

42      YYYYMTRKYGRAMAAWRMYRCRWMRYKAATAAGGRKTGKRTKRYYYWKKTCTYRKWAYMT
50      YYYTAYRKTRRTMRRWRMCATRWMRYKRMGGGASGKCAKGKGAYYCWKKATCYRKACCCY
        ***   **  *:*  ***   ******   .... * .* .  ** ***:  *** .

42      TRRMCSYRSYMKATGCWWSYYYWRYCGAAKCCMCMYYTYYAGRYWRTGTKYMSACSMRYK
50      KRRMMSYRCTMKRKATWWSCTYWRTYRRMKTMCYAYCWYTCARYWRYAYKYMSMSCCRYK
        .*** ***. ** .. ***  ***     *     *  * ..**** . **** .. ***

42      TACARYRKMMWYWWMYYYKCAKKWCCTKRYWMATGKWKRTTTYMKWMWGKKKKKRRKGAC
50      YRMRRYRKMMATWWMYYYKYGKGTYYYKGTAMGCRKWKRWWYYMGTCATGKKKKRGKTGT
            ******  ******* .*     *   *.  ****   **      ***** * .

42      WMRYYKWKKKKMAYYWGKWTMACGKKRMRYRWRKRRRYRGGCYRMGTWGGRKKRWMRRAT
50      AMRYYKWGKKKMWYYTRKTKCRTAKKRMRCRWRKAGRCGKRYCRMKGTCKRKKAAMGGWK
         ****** **** **  * .   .***** ****  *      **     ***  *   .

42      TSKRKKRKMRKKKYTCYGGGARGRWRKMWKRYRRRKMYYMTYRGTRYWWYYMKCTWKRKY
50      WSKRTGATMRKKKYCTTTRKGRRGWRKCWKRYRAAKMYYCWYRRYGYWWYYMTMATKGKY
         ***.  .******      .*  *** *****  ****  **   ******. : * **

42      AWWTYWKRRYYWCGKRRYWYRYYWRKRTCTKRYRWKKTTTRKMMKCGRWGYRRAYWRYSY
50      GAACYAKRRYYWSKKRRYWYRYCWRKRWYGKRTGAKKYAGGKCAKYRGWKYARGYWRYGT
        .   * ******. ******** ****   **   ** :  *  *   * * *.****.

42      RTCACRKWGRRKYKYGKTKKCYMKWKKRRKWWKYRTKYWYRYMWYYRRRMTKAKCGSKRR
50      RKYWMRKWKARTYKCRKYKKYYMKWKKRRKAAGTRGKYWCGCAWCYRRRMGKCKYRCKAG
        *.   ***  *.**  * ** *********    * ***    * ***** *.*  .*

42      WKKKTGCYRYRYMYWYYYCRWTWGAYAKRGCRYGRYYYRGCAGAACMKYYWCSTRARRYM
50      TKKKCCAYGTRYMYATCCMGWYWKMTWKRSYGCKRCCTRKYMRRMTMKYYAYCGARGATC
         ***  .*  ****      * *    **.    *   *       ****  .

42      RWSKKYAGKYYRKTRRYWRGYRYKYRKRRKCGAKYYWRCMYRMKSKYWYYWYMRYKKMYT
50      GWSKKYCTKYYRKYGAYWAKYRYKYRKRRKSRWKYYWRSMCRMKSKCATYWYMRYKKMTC
         *****. *****   **  **********.  *****.* *****   *********

42      MMTCKRRRYYAKMYYRRKKMMKRYRRYGCYYWRGGMYKTCGTKKGMWGAKYKKWGGYKAG
50      MCAGKRRRYYTKMYYRAKKMMKGYRGYKYYYWRRRCYTGGRAKKKAWATGTKKWRCYKWR
        * : ******:***** ***** ** *  ****   *.   :**  *.:  ***  **

42      KTKKKACACGASWCCRRWKRGKMRRYSKKKSRYMYYMYYYKWYYKKYYGYKAYTYRRRRA
50      KWKKGCTRYAGGWTTAAWKGKGMRRYSKKKGAYMYYMYYCKWYYKKYYRTTGTCYRGAGW
        * ** .   ...*    **   ********. ******* ********  ..  **
```

```
42      WKKYKKRGGAACRKYCCGYSYRRRSYKRRKRKKKMYYRACGSMRGRGKWRKWKYKRKRRG
50      AKKYKKRRRWRYGKYTTACGTGARSYKRRKRKKKATTACATSARARKKWRKWKYKGKRRR
         ******      **  . .   ***********    .. * *.* ******** ***

42      GGYKGRKSMYTGYYRYKYRGTGTYAWRSMCYKRYTMRYSYKYMRYRWKRRWRYWMCKMKK
50      AAYKKAKSMYWAYYRYKYRKYSKTGTGCMYYKRYAMGYSYGYCRYRWKRGTGCAMAKMKK
        ..**  **** .*******  .. .  .* ****:* *** * ******     *.****

42      YKGCRSKKKKMKAWCKMKKCAATKCCTMKTRRCTGKRMYKRTKKKKWMYGYARKRRTMMW
50      YKKYRCKKKKMTRWTKMKGSMMWKYYWAKYRGACAKAAYKRYKKKGWMYKYGRKRGAMMT
        **  *.*****. * *** .   *    * * . .*  *** *** *** *.*** :**

42      YMRRRKWWRWMMMKKWGKKKKCSKRMMRYYYWYRWMYKKRYWKRKWRSKMRRWMRYGYRK
50      YMRRGKWWRAAMCKTWKKGKKTGKRAMGYYYWYRWMYKKRYWKRKWRCKMRRWMAYSTAK
        **** ****  * *.* * ** .** * *******************.****** *.  *

42      RYYYWMMRKRCCRRTTTKAAGCCAGMRMYMYYYYRYRWTGKRCCRWYYYMYKKKYWATSK
50      AYYYAMMGTGMMGAKCYGTGTTATTMRCYMYYYCGCRWATKGTTGWYYYMYTKKYATACK
         *** ** .     .   :.  .: ** *****   **: *    ******.*** ::.*

42      WMKTKKKYRRKYYWMYWMYKTCYMWMTTTCGATCGCRKYRKKRYKWKYYRMGAKGYCKKR
50      TCGKKKKYRRKCTWMYWMYKYYYMWMAYYTTTYYATGGCRKKRYKWKYYRMRCKAYYTTA
           .*******  *******  ****:    :  .    ************ .*.* ..

42      KKYRRYRRRATYYRKTWRRSKGYRSKYRMWGKMYKRWKMRRWKWYCYKRMMYTMYYMKYK
50      KGYRRYRRGRKTTGKCTAGGKKTACKCRMWKKMYKRWKMRRWKWTMTKRMMYCMYYMKYK
        * ******  .   *    .*   .* *** *************   ***** *******

42      MRYYRRCRYSYTKMMYYYYRKGMRRYYRYYKWMMWWWMMCKKKCWKWWKKWYYMCGYSYM
50      MRYYRGTRYGYYKMAYYYYRKKMRRTYRCYKWMMWWTMAAKGGYAKWWKKWYTCMRYSYM
        *****  **.* ** ****** *** ** ******* * .*    *******    ****

42      KRKWRYWYCYGRCGTKKSRKYASRRYKTKYKRKKWKWKYYMKYKMYRRWRWKKRCWTTTM
50      KRKWRYWTTYRGMKWKKSRKCRGRRTTWKCKAKKWKTKYYMKCKMCAAWGWKKGMTGCCC
        *******  *     *****  .** . * * **** ***** **   * ***

42      TMAYTTTYGKCAYYYGCRYRRYYRKYYRRWRKKAKCYTYCCYMYSYKYKKWKRKYRRTWK
50      YCGYAAAYRKYWYYTRYRYARYYRKTYRRAATTGTGTYCMYYMYSYGCKKWKRKYRRKAK
          .*:::* *  **   ** ***** ***  ....      *****  *********. *

42      CGRKKKKTRRRKRMMMYYKRGWYKGCTRSWYKTMGWASKKRKRYCKYWRKYMYYRWKRYR
50      TAGTKKKKGARKRAMATYKRKTYKKYKRSWYKCCAAGCKKRKRYMGTTRKCMYCRWGACA
         . .***.  *** *  ***  **  .*****  . ..******    ** ** **

42      KRRMRYKKKKRKWKMSYWY
50      TGAARYKKGKAKWKMSYWY
        .   **** * ********
```

The pairwise alignments of two accessions 42 and 51

```
42          MKYRRRMRWYYRRYRRWRSWRYRYCGYWCYCYRYCCYTKWYRWWWYYRTWYRYYSRMRMM
51          MKYAAACRWYYRRYRRWRSWRYRYTTTTTCAYRYGTYKKWTRWWWYYRYTYRYYSRMRMM
            ***    *****************      .***  *.** *******  **********

42          RKRWMYYYYRRRKWKMRYMRYRSKYRATRWRWKKTYRKKTTYYYMYKAYGRYRYWSYSYY
51          RTGACTTCTRRRKWKCAYMRYRSKYGRCAWRWKKWYRKKGACYYMYKRCARYRYWGYSYC
            *.       ******  ********    ***** **** : *****  .*****.***

42          RWYMKRRYWYRATMWKMCAYMCATGRASRYKKWKYYWKRYWMKCKKYYKARYWRYSKKMW
51          RWYMKRRYTYRRYAAKCYWYMMMCTGWGGYTTWKYYWKRYAMTYKKTCKGACAACCKTCA
            ******** **    *   **      . *..******** *. **  *.     .*.

42          GRSTAYRYWMMRWRMKRYWYRRRRTTRMMYYMKRYYGTRKRRYWYYWSYGYRMMTWRMKY
51          KRSAGYRYWMMGWGAGRYWYRRRRYWRMMYCAKGTYAWGKRRYWYYWGTTYRMCAWRCKY
             **:.****** *   ********  ****  *  *.  ********.  *** :** **

42          TKYTCRRAMRRRYRMMKRAWRYRYYYYYRKYTAACCRWRCYYYYRYYMYYAGTWKYRGYR
51          AKCYMRATARRRYAMMKAWWRYRYYYYYRKYWTCTYRWRMYYTCAYYATYWCKWKYRKYR
            :*   * : **** ***  ************ :.  *** **   **  *  .**** **

42          RMYYYWWKRYKSRYYMWYRKMGKRRMRMMRWKTYMCGRMKRYRRWKRWRRKWKKYRYYRK
51          GMYCYTWKGCKSRYYMWYRKASKRRMRMMRWKWYMTKRMKRCRRWGRAAATAGGYRYYRK
             ** * **  ********** .********** **  **** *** *   .   ******

42          YYKCACYGCKYYYYKYKWYKRKYKKKMRWTKMKYKKKYTRYMRRTTTACCGRCRWYSWRK
51          CCKYMYYAAKTTCYKYKWYKRKTKTKMRTGKMKYKKKTGRYMRGYWACMTAGYGTYGARK
              *   *..*   ********* *.***  *******  ****   :.  .    *. **

42          WYRYYYKYYMYYAKMMGGSKRCGRRKWRRCYKMGGTYCRCRRKYARRGGATYYRKWYYMS
51          WYRYYYKYYAYYRKCAKKSKRTCRRKWRRYYKCAACTTAYRRKYTGRRRWCTTGKWYYMS
            ********* ** *    ***  ****** ** ..     ****: *       ******

42          KYYSKKYTYYRKKKWGYYKTATCTYCWAGGYMYRKRKACYKWYMKMTKARRKRWRWSYYY
51          KYYSKKYAYYRTTKTKYTKKMKYYCMWRRKYMYRKRKCAYKACCGCYGWRAKRWRWCTYY
            *******:***..*  * *. .    *   *******..**        * *****. **

42          RKKWWSYYAGKKRWCTTAWKYYWRKMYMRYCCATRYWKSGYSMAMRYKRYSCMYYYWKRW
51          RKKWWSCYRKKKRAMYYRWKYYTRKACARYTACGRYWKSKYCCGMRYKRYSYMYYYAKRA
            ****** *  ***     **** **   ** .. ***** *. .******* **** **

42          WKKMKTTGCRYWAATMGKGTYCCTAWKRMKTYGYCTAMKWMWSKTCRYKKRYMYRYMRCG
51          TKKMKAGCTGTATGCARKACCAMKCWKRMKWYACGCRCKTCASTCSAYKKGCMYRYMGYR
             ****:      :.   *.  . ..***** *.     *   *. . ***  *****

42          SYKWYYYCCRAYKYGGWMWRKRRMCGRKMRSRSATKKKWKKKYKRWMTCCTMCCTRRKRM
51          SYGWCYCYYGGTGYATWAWRKRGMMRGTMGCGCTGKKTWGKKYKRWMYYYWATTGRGKAA
            ** * *    .  *. * **** *   .* . .: **.* *******        * *

42          KYKTMMRYKMYKYMRMMKMMWYMYMRRWKMKKWKKKRRRKMRYKYYKKYYWRRRSMATTK
51          KYKWACRYKMYKYCRMMTMATYMYAGRAKCKTWKGKRRRKMRYKCCKKCCWRARSMMYCK
```

```
                    ***   ******* ***.*  ***  * * *.** *********  **  ** ***   *

42                  RCMCAGMMRWRRKMRKRCTYKKKMSKKRAYKMYKRAAAAYWCYWYGMMMMWSKYYGAYRK
51                  GYMYWRMMRWRRKAGGAYWYKKKCGKKGGTTMYKRTTTTYWTCTCRMMMMWSKYYTGYGK
                      *   *******      **** .** . .****::::**     ********* .* *

42                  YYRMYWGAYKGCACKTATRYWYKRYYYYRRKMYKYMRKMYKRYYKRKKKKYRYKKKYRGM
51                  YTGATATTYTTTTYKGGCRYWYKRYYYYRRKACKYMRTMYKGTTTRKKKKCRYKTKCAAM
                    *      :*.  : * . ************  ****.***    .***** ***.*  .*

42                  KKRRMRGMRWKSMRKTKMWKTGYCRRWRRYYMYKYRYRYMRCKWKKCYAMCKWKWRRKKM
51                  GKRRMRRMAAKSMRKKKMWKCACTRRWRRTCATKCRYRYMRMKWKKTCGCYKWKWRRKKM
                     ***** *  *****.****  .  *****    * ****** ****   .  *********

42                  MYYRKCYSKKWRWTAMWKYRRRKRCRWMMWKKRKCGCCRYRKKTRRRKYRKWWGRWWAKY
51                  CYYGKTCSKKWAAYMCAGCARRKRTGTAMTKKRKYAYMRYRGKCAAGGYRKWWRAATRKY
                     ** *  ****         ****    * ****  .  *** *     *****     **

42                  RGGAWWKYRYRYMYATYYYCCKWYRTKAACYGCMKYYKAYTGYYRRRYMRRWSRAYYYRK
51                  RRRMWWKYRTRYMYGCCTCMTKWTGYKRMTYAMMKYYGRYYRYYRGRTCGRWSRGYYYRK
                    *   ***** ****.       **   *   *. ****  *  *** *   ****.*****

42                  YRMTARGRKYRKKRGRGCKYYWKYRRRYWYWMYYYKKKKCGAACARRAGTRRKYYRGRYK
51                  TRMYRARRKYRKTRRGSYKYYWKYRGACATWMTCCGKKKYAGGTMRRRRKRRKYYRKRYK
                     **    *****.*  . *******     **    ***  ...  **  .****** ***

42                  KTGAAMMKMRKRRYYWRYMRKMYKKRYMCTYWYKWYKWKCRKMYWKTYMRWRCRYRYMAT
51                  GCRGGMMKAGKGGYYWRYCRGCCTKRYMSYYWYKWYKAGYRKMYWKYCMRAATRYRYMGA
                       ..***  *  ***** *    .****. *******   ******  **   *****.:

42                  TYRGRKKWYWTYRKRAYKMYTGWSMTCRYATATAAGYYYTRWKRWCTYAYKYMKMRGKMG
51                  GTAKRKKTTTAYRKRRYKMCAATCCCYRYWYRWRWKYYYGAWKGTYACGTGCMKMRKTAK
                       ***   :**** *** :. .   **       ***  **    : .   **** .

42                  TTYKYWWKKKKTCAKKKGKKMWYRCATYRWYKSGYRGRTYRMKRAYRGAAMYRWKYYMRY
51                  YCYKYTWKKKKKYMKKGKKKAWYGTRCCRWYKSKYRRAYYRMKRGCRKRWMYRWKTYMRT
                      *** *****.  **  ** **     ***** **   *****. *   ***** ***

42                  YWWRYKKYGTRTRRWRGCTCYAMYKARKRKYYYKKMWKRMRRMTKWYKGGRRRRGATRGW
51                  YAWGTKKCAYRKRRWRTACATWMCGTRKGKTCTKKMWKRMRRCYKWTGAARGGGKGWGCW
                    * *  **  . *.****  . .  *  :** *   *********  **   ..*    .   *

42                  YRMKCKYWRRYKRCCRYTATRYCRYWWKAKMTRTTYKKRMRRRRSYKWYYCACYAWKKYR
51                  YRMKMKYWRRYKRYMRCKGYRYYGYWWKWKMKRYWCTTGCGARRSYKTCYYRYCRATKYR
                    **** ********  * .. **  **** **.*    ..    *****  *      .***

42                  CTTYMKKTTGAATAYKWRYRWMKTTRTRWGMKMRCRRRMYKYKKRAKRCKYRRCMAMKMC
51                  YCAYMKKCCCTMWWYKWRYRWMTWCRKRWKCKMRYGRRMYTCTTGTGGYGYRRYCRCKMY
                      :****   :   ********.  *.**  ***  ****. .. :    ***    **

42                  YKKWMKWYWKKKYKRYKAAAKMGRYYMCWMMGGCWYRYMRWRRWYCTTRKYRYMYYWKMK
51                  YKKWMKACTKKKYKGYKGCGKKCKAYYCTTAMKKMWYGYARARRWYSCGRKYRYMYYWKMK
                    ******   ***** **...*   **    *    ** * * ****.  ***********
```

```
42          KRWAARMWRCTCYRYKRRKYYKMRYKYRKWTTTKKYWWYKTYRKMWCAYMYKRKYGRYAK
51          TRWGTAMWRAGYYRYKGAGTTKAAYKYRKWCACKKYWWYGAYGKMWACYMYKRGYTRYMK
            .**.: ***.  ****     *  ****** : ****** :* ***..***** * ** *

42          GKKKYYRMKKKRSWCTRARYKKKRRYWRYRSKYRSKYGGYGMGYKKMYRAACGRMKWYYK
51          KKKKYYRMKKTAGTMWRTRYKKKRGTWRTGGKTACGCATYAMRYKKMYRRRMKRAKATYT
             *********. .   *:******  **  .*  .  . *.* ******    * *  *.

42          CTATRRKKRMMYMMKKMYKRMKYRRYYMRYYRRGCSRGRKKMMKTWKWAACGTRMKKCRK
51          YWWKRRKKRMMYMMKKMYKRMKYRAYYMRYYRRTTCRTRKKMMKKAKWWTTTCRMGKYGK
               .******************** ********  .* ******. ** :   ** *  *

42          YYGYCARRYYWCATKAAKKKYGAYYSGKAGKKWRRRKRRRGCYRMWKKCRRWMRRSGYKK
51          YYRCMRRRYYWTGYKCCKKKCRCYYCRKGKKKWRRRKAGRRYCRMWKKMRAAMGGSKYTK
            **    ***** . *..***  .**. *. *******  *   ***** *  *  * *.*

42          RRGCAKMRKKKTMYKTTGYRKRWRRMWKRTYRYRCRRRYKYCGMYYRRYRRWYYYRKRYK
51          RAKTTGMRKKKWCYKCAATRKGWRRCWTRYYRYGGAAGYTCTRMYYGGYRRWTYYRTGYK
            *    : *****  ** :. ** *** *.* ***     *.   ***  **** ***. **

42          RTYYWRWTTMAWCWCKGWSKGAAGSRRKRTYYMCTYCYKKGTKYYYKKSKYKMYYCMYTC
51          RCYYAATAWCWWTATGAWSKKMGKCGGKGWYYMTCCYTTKKKKYYYKKSKTKMTCACCGA
            * **   :   *    .***  . .  *  ***     .* .******** **  .   .

42          RGRKWYYGRYTTYYMRWCTAWRYYSRRMARCKRKKRRYYRKRRMYYYKGRRSKTGTCYRR
51          RKRKTYYRAYYCCTCAAYYGTATTGGRMRAYKAKKAATTRKAAACCYKRGASKCAKMYRG
            * ** **  *         .    . **   * **    **     **   ** .. **

42          YMGAYYTRCKSGGTAKGKSMRRYAYYRKKTYGCRRKKCYCKYRWGGCMRTCYKYAKMRMG
51          YMKTYYWGYKSRRATKRTSMRRYTCYRTKYYRMRRKKTTTKCRWRRYCRATCGCTKMAMR
            ** :**   **  ::* .*****: **.* *  ****   * **    *:    :** *

42          YYYWACRSGYMMYKAASKYYKMYMRAKYKSMKSRKWKKYMRYKTATTKRRYWRYYKRRYY
51          YYTATTGSRYMMYKMTCKYYKMTMRMKYKGMGGGKWKKYMRCKCTCGKARYWGYTKRRYY
            **  :  * ***** :.***** ** ***.* . ******* * :  * *** * *****

42          RRYMKYWRYMKYWKRMSRKKRWWKKKCTYAAKYYKYSYTTRYRKKTRKTYKKKGYMYMWY
51          RGTATYTRYMKYAKRMSRKKRWWKKTGGYMRKYYKYSYAAGYRKKCRKWYKKKKYMYMTC
            *   .* ***** ************.  *  *******:: **** ** **** ****

42          KAAMYMWWKKKRMRRKCAYYRCCYGYWMMMWWWMKMRYKATTWRMKTKYMKYTYCTTGGA
51          GGGMYCAWKKKRMRRGYGTYRTYCTTWMMMWWTAKMRYKTAAWGMTYKTCKYYYTGKTCC
             ..**  ********  . **     ******  *****:::* *. *  ** *  .  .

42          YYSYMMKWRCRTCGAAGTKSRTYASYYGKYKYCRYKKWGYWCTYKKYYKMYRRWRWKRYK
51          YYSYMMKWRARAARCCTAKSRACGGYYKGCKYYGTKKWKYWSKYKKYYKMCAAWGWKRYK
            *********.*:. .. :***: ..**   **   *** **..*******   * *****

42          RKKKYTTSYGRRYYGRGCKYWRKKYSRKKYKYYYSKKSGMWCYKYTTKKWGTGSYAGYTK
51          RKKKYKYSYRRATCRGKYKTAATTCSRTKYKYYYSGKSKAWYYGTCCKKWAAACYWRCAK
            *****. ** *       *   .. **.******* **  * *    ***.:..*   :*
```

```
42      KACRAWCCTWWYMKKWSKYMYYYYYMYWYYKCCRGKAKKACCYRYRRAWKKYKRKYRYWC
51      KCTARWYTCAWYMKKWSKYMYYYYCMCWYYKYYRKKCKKRYYTRYGAWAKKYKGGYRYTA
        *.   *    ************** * ****  * *.**    **    ****  *** .

42      CCGSYRRRSYKRRSKCKKKSKKKRGKKWSYYRKRRCCCMGGYRKSMGCGARKYYMTRWRM
51      TTAGTGARSYKRRCKYKKKGTGKRRKKWSYYGKRRYYMARRTAKSMKTTGGKYYAKGWRM
          ..   ******.* ***.. ** ****** ***        ***   . *** . ***

42      WGRRRYWYMRTMTGYYMRYRYRRGWRAWMYGRRMYGRKAAYRAYYRCRAKTTGAKYRRYC
51      WKGGRYWTMRGCGAYYARYRYGRKWRWWAYKRRMYRAKTWCAWYYRYAWKWYRRGYRRTS
        *   *** **   .** **** * ** * * ****  *:    ***   *     *** .

42      WKMRYKYMYWTKATRYYKMYWYKWATGYYYMMCMTATMWKMMTMYYGKRMKYMWKYKYWC
51      WKMRYKYMYWYKRKGYYKMYWYKWGGAYYCMMTACTCMWKMAYCTYRKRCKCMTKYKYWT
        ********** * . *********. .** **   : ****    * ** * * *****

42      AGRKKKRRKGACMMMYRRYKKRYKKRMYYYSRSMWKYRMRCWGCSCKYRRKRYKKCKTKY
51      GAGTKKGAKRTTAMATRRYKKRYKGGMYTYSRSMWKYGMRMATMCTKYRRKRYKKMKAGT
        .. .**  * :  *  ********  ** ******** **    . ********* *:

42      WCRKYKRYGRWKKMRRMWKYKKRWKRGKSYWYKMAMKYYYWYRYRWMYKKYRAGRKKWGK
51      WYRKYTATTRWKKCAGMWKYGKAWKGRKSYWYKMMMKYYYWYRYRWMYGKYRWRRKKWRK
        * ***.    ****   **** * **  ******* ************* ***  **** *

42      MRRMSSMTGYCAYRKCTARWMKRKYRCCRMMAYAGYCMKRRRKRYGYYRYKWYTGMRRWY
51      MRRMSSMGAYTCTAKTCTAACGRKYRMMRMMGYTCYYMKRRRKRYACYGCKWCATAGGWY
        ******* .* .  *  :    ****  ***.*: * ********. *  ** :    **

42      YCMKKKWMKTCYKTCGKRGRYGTYKSYWYKMYYKKAYRKTCKAKRYRMYYKMCWRAMYKK
51      YYMTKKTCGGSYKAYAKRRRYRYCKSYWYKMTYGGWYRKYYTWKRYRMYYTCAWRWMYKK
        * *.**    .**: .** **   ******* *   ***  . *******. .** ****

42      KGMTYRRWTTMWRYKAGTSYTKRMYCRKCAAGCCRKMMRRATGYACTKTACYGAAGKRGY
51      KKMYYRRWWKAAACKRRYSYWKRMYYGKSRRAYMGKCMAARARYTGGTYMSYAGGCKGRT
        * * **** .    *   ** ****  *.  .   * *   : *:  .  .*... *

42      KARKGCYWMYRTRCSWYRWRRRYKTSRTCGYYYCWMYYCGWYRYKAMKWCCKKMCWYKYY
51      GRATRYCWMTGCGTGTTRWRGRYGACGAMAYYTTWMYYSKWYRYKRAKWYYKTAYWYKCT
           .   **     .  *** ** :. : .**  ****. *****  **  *.  ***

42      AAWYWRSYRYKWTRMMKKRKWACYYGKARWKTKKKTGWWKYKYYRYMRRMYRWRMWRSRR
51      TGTYWRGTRCGACRMMKKRKTMYYYAKGAWKAKKKCTAAGTKYCGCARRMCRWRMWRCAG
        :. ***. *    *******   **.*. **:***      **    *** ******.

42      WKKYR
51      TKKYG
         ***
```

The pairwise alignments of two accessions 43 and 44

```
43                  KRYYRRKWWYWKGWYGTGARMYCTWCYRGYRGWRGARRKKRKWTACGCMRRKYYKYWWRR
44                  KRYYRRKWAYWKRACRGAGAMYYCTTYRRYRRWRRRRRGKRKWCWMRTMRRKYTGYWWRR
                    ******** ***     .. **    ** ** **  ** ****     *****  *****

43                  RMRYRATTRKGTTYRWRYRRWRYWMYYWMWYTTYAATYTKCCYTKMTAYKRYWWWCKGGR
44                  RMRYRRYKRKAAAYRTRYRRWRYWATYWMWYYCYWWYYYGTTYWKMKCYKRYWAAYGARR
                    *****  .**.::** ********  *****  *   *    * **..*****    . *

43                  RRYCYRRAYKRYYCYTGRWRMKYRYAYCGCACAKSTYKMRRWYKMKSARWWTCRYYKYYR
44                  RRYTYARGYTRYYYTCAGWRMTTGTTCGATGTWKSGYKMRRWYKMKSWRWWYMGYYKYYR
                    *** * *.*.***   . ***.   :  . .  ** *********** ***   ******

43                  YMWYYMAWKKYYRRARGMWRWRRSWYAYYWYRWKYYWYAMRRRKYRCMWWRYMYMSKKYM
44                  YMWTTMGWKTYYRRTRKMWRWRRCWTTTYWYRWKYYWYGAAAGKYRMMWWRYMYMSKKYM
                    ***  *.**.****:* ******.* : **********.    *** *************

43                  YYCRYWRWWRYKYKYWTSMACYRYYCYCTTRYRKKMKRMMWKKRWKKKCRWYGRYWSSMY
44                  YYARYWRWWRYKYKYAYSMCAYRYYYYMYWRYRKKMKRMMWKTRWKKKYRWYKRYWSSMY
                    **.************  **..**** *   ************.***** *** *******

43                  KRRWKGMARYKRKTACRKKYAWSMKWYMYSKRTTCATYYYYKYYYYTYCYRYCYYTSWWA
44                  KRRWKKMWRYKGKYRYRKKCGWSMKWYMYSKAGAGRKYYCYTCCTCCCYYRYTCCASWWW
                    ***** * *** *   *** .**********  :  .** *.       ***   :***

43                  RWKKMYYGGAKTAGATRYKKRWKYAAGGAKYGKAATAAAYAAKYWACGYKYRYTAGYGST
44                  RTKKMYCTAGKCGKCYGCKKRWKYTGRTTKYCGRWWMWMYGRGCAWTRYKYRYYWKCRSC
                    * ****  ..* . .   ******:.  :**        *.       *****     *

43                  RMKYRRMYYTTRYWRYSKAWAWKWRATWKRTAKYGATWKAKKKRKCYKYKGAGAKRYRGC
44                  RMKYRRMYYCAGYWGCSKGWWWKWRTAWKGWGKTKTWWKTKKKGKTTKYKKRKRKRYGRT
                    ********* : **  **.* ****::**  .*  : **:*** *  ***    ***

43                  ACYGYMKRRGGGRGMMYGRYYYMYRYGCMRRKKTRYRCGRKACTGTKKRYSYKKARKYRR
44                  TATTYMKRGAARRKAMYKRTCTMYRYKYMRRKKKGCATARKTTYAKKKRTGTKKMRKYRR
                    :.  **** .. *  ** *   ****  *****.    .**:  ..*** . ** *****

43                  KKWYMMYAAYKKMYSKRYKRRKGYKGKKATAYKRKACGRWYKMTKYGRYKRWRYRYWKYA
44                  KKAYMMYWGYKGATSTRYKRRKTYKKKTGAGYKRKMTAAACKMKKYAGYKRWRYRYWKYC
                    ** **** .**   *.****** ** *..:.****  .   **.**. ***********.

43                  YKKRWKKTAMTKKCCCCRYACTGYKKYGMKWYKKMRWRWRWRRWMMRYAYRRMWCYTYKG
44                  YKKRWKKKMMGKKATGGGTRMYSYTKYKMKWYGKARWRTRWRRWMMRTGCRRMTYYWCKK
                    *******. * **.        .*.** **** * *** ******** . ***  *  *

43                  AYRKMRWKKGMYRYWTRRGCYATWRRYKMRRRRGRWRYYRTYTKATTGTCRAYCSGTKKS
44                  WYRTMRWKKRMTRYWCRRRYCRCAGRYKMRRRRARWRYYRWCYKTYYRYAARYMSKYKTC
                     **.***** * *** **       ********.******   *:    .  * *  *..

43                  RKKKCKKMGKKYYYYKYSYWTGTYGTAGGARRKCCGRAAYYKCATYKGRGYYKKKKRTYY
44                  AKKKTKKMKKKYYYYKYSYWYKWCAYRRRRRRKYYRRWWYYKTRKYKKRRYYKKKKRYYC
```

```
                 *** *** ***********    .     ***   *  ***  .** * ******* *

43               KTTGGYYWCKRRGYRYRYCTAAMWYKYGTCTKRMYYYMYKKYWYRKYYMTAKYTYYRSGC
44               KAAARYCWMKRRSYRYGYYYRMMWYKYRATCGRCYYCMCKKYWYRKTYMKRKYWTYRGKY
                 *::. * * ***.*** *    ***** :   * ** * ******* **. **  **.

43               YTYMWARTYCATTAWYMYKSRRYRGTYTTCCSWAYYTKRKTKGMRYKRKATKKATGKKYA
44               CYCMWRRYYTRCGRWCCYKSRRYRRCTYYTYSWRCYGTGGCKKMRYKRKCCKKCGKKKTR
                    ** * *     *  *******       **  * .   * ******. **.  **

43               YAGYRTTGKACKYMRRYYYCRMWWKYMKMKRRRMCCCRYGAGACTAGAAYTGTTRKWAGK
44               YMKTRYYKKTTKTMAGCYCTRCWAKYMKMKGRGCTMMRYRRRMYCCRGRYWRWYRKWCTG
                 *   *   *: * *   *  * * ****** *     **      . . *    ***.

43               YYMCAAGCWTTCTGWWGCWYMKCRKGAMWYAYRCKTTMRRWGYYKMKWWGWYMKKKRYAG
44               CTCMWGRYWWCTCKWWRYTCCKMGKRRMWYGYRYKCCAARWRYTTATWWKWTMKKKGYTK
                      .  *     **     *  *  ***.** *    ** * . .** * ****  *:

43               MCMKRMKWRRKKAGKKRGYRKMYSKRTRAKRYMCCTYYYYYKKYKKAGTCKKYKRYGKKK
44               CYMKRMKTGRKKRAKKRRYRKMYSKRYARKGTCTTCYYYYYKKYKKGRWYKKYKRYRKTK
                   *****  *** .*** ********   *      **********.   ****** *.*

43               RGYYAKCCATMKYYWAATYRSYWYKYRGWYGMTCYKYWMRRWRYRYWRCKSGGWMYYKRY
44               RCCTRKASWWMKYYWRTCYRSYWYKYRAATKMAMCKCWMRRWRYRYWRAGGRSWCYYKRY
                 *    *..  ***** : *********.   *:  * ***********. . .* *****

43               YKYWYKRYYRYWGYCWSKKWYKRGRRRWWWYKRCMWYCMYYRWRWAWRAYRKYRYMCRTY
44               YKYAYKGTYGYATCYTSKKWYGGTARRWTATKAYMWYYMYYRWATWTRCYRKYRYAAAKY
                 *** **   * *     *****    ***   *  *** *****    *.****** . .*

43               RYSYKKRGWRRRRRCAAGCGTWKTKRMYYMWRKKTGYRYGRCGYKMARRCWMCKYYYYRS
44               RYSYKKRKWRRRRRYRRRYRYAKYKRMYYMWRKKYTYRYKRYAYKMCRRTWCYKYYYYRS
                 ******* ******        * **********  *** * .***.** *  *******

43               MKRAARYYRTRRMCCYCCCCYGMKKKRGTGSGKKTYGKKKSGKKRKWKGCMYTYYKKMKY
44               MKRMMRYYRCAAATTYYYYMYKMKKKRAGAGRKKKYKTKTCKTKRKWKTYMTAYYKKMGC
                 ***  ****      *    *  *****. .. **.* .*.. .*****  * :*****

43               GRKKRWWSCCSAKSCMTAGKKGGCGWRCKKRYKKKTGTMRMWTAGCRYGTYYTRRYMRTA
44               KGKKRWWSYTSRKSYMCTKKKAATAWRTKKRYKKKWRKMAATKTTAGCSGYYKRRCMGWR
                   ******  * ** * : **.. .** *******  .*   .: .  . **.** *

43               MGASCCRMRTCKATGWKKYMGTTRYMKRACYYRKMRRKTGKKKGYRTGGKCWTYRATRCK
44               CRTGYTGCGAYKCWKWKKYMACARYMKRRMYYAGMRRKKKKTKRCGAAKGGAKYARWGTK
                   :.     : *.  *****. :*****  **  ****. *.*   :.    .*     *

43               KATTGMCRRKKKYMRKKYTAATAKYRRRYMKKYYGMAAYTKKKAKTAGYYCKGARYKGYY
44               KCCKCCAGGGTGYMRKKYWCCATKYAGRYMKKYYKCMMCWKKKRKACRCCYKRRRCKKYY
                 *. .  .   . ****** ..::**  *******      *** *:.     * * * **

43               RYYYWMCTRKKTGKWTKKWGGAKWRRSWMTTTAYYTGSKMKRYKCWAMRYKRYMYKRWYK
44               RYYYWMMKRTKCKKWYTKACARKWRGCWMYYKRYYYKSKMKRYKMARAGYKRYMYKRWYK
                 ****** .*.*  ** .*  . *** .**  . **  *******     ***********
```

```
43    ARWKKRTRGGTRCCSGRKYTCKKKMKWYRASTYYAAARMRWMTMTKGMCYGGGGRKYMYK
44    RRWKKGYGRAYRYSCTRKYYYKKKMKWYRMSCYYMWGGMRWMYMKKAMTYAAAARKYMYK
       ****    . * .. ***  ******** * **   . **** *.*.* *....******

43    CWCRGCYYGYGYWTKWKKWMRKRMMTKCAGTATWMAKTTKRYCGYTRKMATRAKTGCKKY
44    YATARTCTRTRCWAGWKKWMGKRCMYKYWACTCWMWGGYKGCMRYAATATCRMKKRAKTT
                  *: ***** ** * *   . : **    *     *: . : * *. .*.

43    YKKGYCGAMMAAKACMRWMKGMYCARYWYWTYYRMMRMKRYWMAMMWWARWWYGCCAMTK
44    YKKAYGTMCMGGKGTMATMKKMCTGGYACWCCYRACRMKRYWCWMMWWTATACAYACAKK
      ***.*    *..*. *  ** *   . *  *  **  ******  ****:     . .. .*

43    GYTKMTTKRYMRTAKWRCYRMRWRKMARYMKCGRKTGTMKWKKKGAAAKCCCYMYYRSRR
44    RCYKCWKKATCGKRKWRYCAAGWRKMRRYMKMKRKCRYAKWKKKKMWRKTMYCCCCACRR
         *  .*     . ***      **** ****  **     *****     *        .**

43    KCKRKKKMMKWYYATTRYAAGATGRMTSRGKYKKGGGWACKTYGKRRWRMYKTGRMGMTR
44    KYGGTKKAMKATCRYKRTTTTRYKRMCSRRKTKTRATWTTGKYKKRRWRMYKWKRMRCKA
      *    .** **      .* ::     ** ** * *. . *:  .* ********  **   .

43    RCAKTRYWKRRYMRKTYYWRKSYCCGCTKMRRYKKKGYTYGWSYCARGAYKKWKKKYKAR
44    RAGKYRYWGARYMRGAYYWRKSYYYKMWKMRRYKGGKTWYRWSCTTGTRTKKWKKKYKGR
      *..* ***  **** :*******      ******      * **   :     ********.*

43    KKTSSGMARYYGTGMKKTTTSYSTMKYRYYRKWRRYKMYRYRKWSMKWKTGGTMYMGCGG
44    KKKSSKMWGCTAWSMKKAACSYCYMKYRYYRKWRRYKMYRYRKWSMKTKYTAYACCKTAT
      **.** *     . .***:: **. ********************** *   .      .

43    MKGKRSKSMKWWKRYMRCYTKWKMWKGYKKRRRRTTAKRTRMRGYKTKKKRATSWRYRRC
44    MKAKRSKSMKWWGGTCRMYKKWKMWKRYKKRRRAAWGKGWRMRKYKYKKKRWKSWRYRRY
      **.*********     * *.****** ****** : .*   *** ** **** .******

43    ARCTYKYRMMYRMRYTMYYCYWMRKWAKRMKRKKGTTCTACCRGGGYYWMKGTRKTYRKK
44    WRYAYKYRCMYRMRYKMYYMTTCRKWMKGMKRKKACKYKGYGGRKRYYWMGKKGKKYRKK
       * :**** ******.***     *** * *****. . ..       ****  . *.****

43    KYCTCTGRGAKCKYKTCWYYRGACKRMRSYYRMYGAYKYKKKMSKAGGTGGAYRCKCTWY
44    KYSCTCRAAGTTKYKCTWYYRKWYKGMGSTYRMYAGCGYKTKMGKGKCYCRRYRSGAGWY
      **.      ... ***  ****    * * * ****..  **.**.*.      **. . **

43    RGKGYMAAGKKASTTAMTKYSWKAYGYRRKYMMYWYRTKMTWRMCWKKYGYYRYRGSTCM
44    RKKKYARWKTKCGYKRCCTTSWKMYRYRAKTMMYWYRAKMGWRMYTKKYKYYRYGSSKYM
      * * *    .*.. .    . *** * ** * ******:** ***   *** ****  .*. *

43    GWYRKKYGGYKSRKKKAGYKKKSWMMRGCGGCGRKYKRYYAKYMKKRKKYYYYMWYWKAS
44    TWYRTKTRKYKSRKKKGAYKKKSWMMRTYRRTAAKYKRYYRKYMKKRKKYYYYMWYAKGG
       ***.*   *******..*********     . ****** ************** *..

43    CWWTCAAMCAWCYYKKYYYTKKRKMMYYYYRKYGRKKYKRCYYMMMYYYYKYKGKAATA
44    TWWYTCWAYWWTYYKKYYYYKKRKMMYYYCRKYKRKKYKRYYYMMMYYYCYKYKKKWCGC
       **   .    * ******* ********* *** ****** ******** **** *  . .
```

```
43      RKKKRRKRYYYWMMWGCGGKAGKKKRWMWRYRGYGRTTYYYMRKTGYKCRCGGYRRRYRW
44      RKKKRRTRYYYTMMWRSRRKTTKKKRWMWRYRSYAACAYYYMAKWKYKYATRRYGRRYRW
        ******.**** *** .  *: **********.*.  :**** *  **     * *****

43      MGKGMYRTGRCRKKRTAAKKTAGYRWKRRRMGRWRCKKMKYAAYKKWCKKYGRCCRTRGG
44      MKKKMYRGCRYRKKRCGGKKYRRYRWKRRRCARWRYKKMKYTRYKKWTKKYKRMARKRRR
        * * ***  * **** ..**   ******* .*** *****: **** *** * .*.*

43      GAWKKKRRYYGMRWRYYRYRGAGCTKYKKRTGAYRRRYWAYRWCMYGWKMWYKAKAGKRK
44      KWWKKKGRCYRMRWRTTRYGAWAYWKYKKRYRMYGRRYWCYRWYMYKWKMWYKRKWKKRG
          **** * * ****  ** . .  *****   * ****.*** ** ****** *  **

43      KYTCCCCGYYCRWGKCYKGTGKTTCGCKKKMYMKWWR
44      KYCTMAYTTCGRTRGYYKRGTKKWYRYKKKMCCKWWR
        **   .     *    **   *.    ****  ****
```

The pairwise alignments of two accessions 43 and 45

```
43                  YTCGRRAAYYRWWCKRTYYRWWWMRMSWMRRYYYCCKYYRRYWKGTTAACGYYCYRRARM
45                  YKTRRRWWYYRWWTKRKTYGWWWMRMSWMRRYYCYYKCCAAYWKRCGGGMSTCMYRRRRM
                    *.  **  ***** **. * *************   *    ***   .. .   *** **

43                  MARCKKGGTYYYTRMKYYGWAWKMRRRYKKRMMAYYMGWGAYAMTRRYRKYRWYRAYKGS
45                  MRRTKKRKYYYYWRMKYYAWRAKAAAGYKKRMCRYTARWRTYRMWRRYRKYRWYRTYKTC
                    * * **   *** *****.*  *    *****  *   * :* * **********:** .

43                  WRYRRKSRYWGYYSWYMGKATWWRWKMRRMRKKGRYGCAGRRYAMKAGTGTRKCMYYCAG
45                  WRYRRKCRYWRCYSWTMRGTCWAATGMRRMRKKRRYRTGTRRYTMKWKAAWRKYMYYYRA
                    ******.***  *** *  : *    ******* **  . ***:**  :. ** ***  .

43                  KAMWKRYWMYKWGCGMRYWMKSYYKWKMSRWMKYTGGKGSKKRMYYMMRMSYMKYRMRKG
45                  KCMTTGYAATKWRAKCRYWMKSYYKWKMSRWMKYGAAGTSKKRMYCCMRMSYMKYRMAGA
                    *.* . *   ** .  ******************..   ******  **********  .

43                  SMKYRTAGCCTGKGRYKYRTTCKYRMCWRYRGTGMGYMCYYRRKRYKRYWYRCTCGGMKK
45                  SMKYGYGCTTAAGRRYKYRAYYKYRMYWRTGTAAMSYMYYYRRKRYKAYWYRAGMKTMKK
                    ****  .   :.  *****:  **** **   :.*.** ******** ****.    ***

43                  YYSYYKGCKYKKYRTGSRKRAKRGTYYKYWYSYACTRKTKRMRATACWKYKKRYRYKRYY
45                  YYSYYGKYKYKKYRAASGGRMKRKWYYKYACCCGTARKKKRAGGWGTWKYKKRTRYKRYY
                    *****   ******:.*  * **  ****  . . :**.**  . . ****** ******

43                  CGKRSCACWKWTTKGCKRYMKTYAKSWMTCRTKCGKRKMYWKKAACARRMTTAAAAAAAA
45                  TKKRSYRYWKWCCKKMTRYMKWYCKSWMGSRYGYAKATATWKKWWTWARMKWRRRRWMRR
                      ***   ***  *  .**** *.**** .*   .* .  ***     **.

43                  YMCYTTTGCYKKAAAKKWSGKYRGAACMMCCCCARTWMMWWGWKYYKYGCMTKGGARKCC
45                  YCMTCWYSTYKKRRWKKWSRKYRRRMTACTMMMRGKWMMWWTWKYCKYRYCKKATTRKYY
                    *      . ***   **** ***            .***** *** **   .*. :**

43                  RWYMWYWKKRWKCGAAKMWYAMRWWRGARAYKRMGKWMMGYSTKKKKYYRYYWCKCYTKT
45                  RWYMWYWKKRWKYRGCKMTTWMRWWGRRRMYKRMRKWMMRYSKKKKKYYRYYWYTTCCTW
                    ************  ..**   ****   * **** **** **.********** .   .

43                  CTGCYRYMRSKYMYKTAKWRWGRYYKMGGMKGRYYYRKKRMKKKCGSYWCTWWYMYYYRM
45                  YWKYYRYMRSKYMYKYWKAGAAGCYKMRRMKKRYYYRKTRMKKKSRSYWSYWWTMYYYRM
                        ***********  *   .  ***  ** ******.*****. ***. ** ******

43                  ATYYKYKMKCTKRKYKMGKMMKKKAARTYRRGGWYYMMKTKYRGTAKKKYYMATGARYYW
45                  GAYYKYKMKYYKRKYKMKKMMKKKGCRYCARRRWYYMMKWKYRRYRKKKYCCRCATRYYT
                    .:*******  ****** ******..*   *  ****** ***   ****    .:***

43                  MMWKKRRKRWMWRYRYGYKRKCKWWSSKSYAWRYKMTAYGKRKMYSRKYRKKMKKGAATA
45                  MMWKKRRKRWMWRYRYSYKRGAGWWSSGSCRTGTTMCTYKGGKAYSRKYRKKMKKSRRYR
                    ****************.*** . **** *     .* :*   * ***********.

43                  ACKYYRYTGTMTTTCKCRYYYYKKRYMRKMSKACGRMYRMRKAGWKTTATAMYTCCCCRK
45                  RMKCCGCAAGMKCATKYGCYYYKKRCMGKMSKGAARMYRMAKCKWKWYTARMYCYTYTRK
```

```
              *    :. *. : *   ****** * ****...***** *. **  :: **     **

43          KYRTRKYKYGTTTYGCRATYCCTWKCCCTGRKTTTWGRTKRKCWMYYGRTTWAMYTKYRY
45          KYRKAGYKYRYYWYKTGMYYYYYWKSAMWRGTWWKARAYTAKYWMYYRRAGWCMTKKYRC
            ***.  ***    *     *   **..    .  .    . * **** *: *.* .***

43          KKWTTKKSCGCTRRRKAAAKKWYTKCRMAYYKGYTGAGCRYRYGRAGGRGTCCGYKTARR
45          KKTYYKKSMKYYRRRKRWMGKWYYKYRMWYYKACGATATGTAYRRMKKRKKMTKYGAMRR
            **   ***    ****    *** * ** ***.  .:.    * *   * .   * : **

43          YTRYMRKSYYMCAGAASRSMCRCAYYRACAYCTCYYRSKKTGGCRGRCMSKKKGKKCRTR
45          YYRYMRKSYYAYWRGGSRSMTRTGYYRRYRTMWYYYRSKGCTAMRKRYCCTKKKKKYRWG
            * ********    ..**** * .***       *****   . * *  ..** ** *

43          GTYCWKRWKKYAWCCATGYYGCTKKYKRYKSWMKCTAMRRRKAKWMTAWGYSKRKKMATG
45          RCYMWKGWKGYTASATWKYTKYWKKYKRYKSWMKGCCMRRGKWKWCCGWRCSKRKKMRYR
              * ** ** *: ..:  *    *********** .*** * **  .*  ******

43          KWRRKCGATRYYKKTCGYRKKKRYSYRYMYYKKKKYKKRTTGAKCTTGGRYYYRWRMRWR
45          KWRGKMATARYYGKCTAYGKKTACGYRYMYYKKKKYKKRCYRMKACCRRRTYYGWRMRWG
            *** * .::*** *  .* **.  .**************    *.    * ** *****

43          YYGYKATCCYGKTAASKKTKAAATYCYYYMCRTTRGWKGCRRKYYACYTGMYGKYCMRKK
45          YYATKTYYTCKGCCCGKKYKWCWYCYYCTCTAGARRTGRYRRKYYCYCGRAYRKYMMRKK
            **. *:       ...** * .    *      :*     *****.     * ** ****

43          YKGTAKRRRYKRWRRRWYYKYKYKTTKKYASWRYRTKRAGGKCWGMWYRMRYTMYYYMKT
45          YTAGGKRRRYKRWGRRWYCKYKYGYYKKYWCTRYRAKARRSKATAMWYRMRYKMYYTCKC
            *.. .******** **** ****   *** . ***:*   .*. .*******.***  *

43          WWTTATMTRAWKMKRKKMTCCCCRTRARYYWYMTGKKATRGWKGYKTRAGCYKKYGGCRY
45          AAGWCYCYRMWKMKRKKMAYTGTRWRGGYYWCCYRKKWAGRWKKTTWGWRTYKKYRRMRY
                .   * ********:    * *. ***    ** :  **  .     ****   **

43          MRKRYAYTGKTWRMKKYWGYYMYMMKYWAKRWRRCCGKAAGGGKTKMGYRYYRRKYRRRW
45          MRKRYRYKKKYARMKKYWKYYCCAAKYWMTRARRMTAKWGRTKKYKMAYRYYRRKCGRRA
            ***** *. *  ****** **    *** .* **  .* .   * **.*******  **

43          MMYAYWRWYCKRYMKKTYAKKCCCCTRWYKCCKYCTKYYRRKYKAYRATKYAAAWYMYKR
45          MMYMYTGACAKRYMKKCYGKKATMTAAACKSMKYYYKYYRRKYGMYGMGKYTCTWYAYGG
            *** *    .****** *.**.   :   *. **  *******  *   **:.:** *

43          TCTGTRKYKWYRYYRMTAYRYYYCGRWKAYGTTKMRRKTRYRCSRYMYKSMCRKRRYKYT
45          YAYACRKYKWYRTYRMKRYRCYYYARWKRCTYAGAGATKRYRTSRYMYKSMYRKAACKYG
             . . ******* ***. ** ** .***    :    ..*** ******** **   **

43          WRYKRKAWKRCRYWAKYKYCGTTKTTGTRMMRYMYKTTTYTYTKRRYRMCGYYYYKKKYY
45          WRYKRKGTTRYRYARKYGYMRCAKAYAYRAMRYMYKYCGCYYKKRAYGMTAYYYYKTKCC
            ******. .* **  ** *   :*: . * ******     *.** * * .*****.*

43          CYRKTSKKRRCYKAKYATSYWYYRTRGYRYRGWYGMYKRYTWGTTMRWGKRYWTTRYRTA
45          SYRKCSKKRRACKRKYTCSYWYYRKRRYRYAAATKMYKGTYWKCGMRWAGRYWACRCRCT
            .*** *****. * **: ******.* *** .   ***   *   ***. ***: * * :
```

```
43      GGRRMAYRMYWRYRRCTRMYYSYTAYYCCTCTACCRAAGGYWKWMMKGCKRYWKRRRKYR
45      RSRRMWTGATARYRGYCACYYSYKRYYTMAMCWTSRCWRRYWKWMMKKMKAYWKRRRKYR
         .***      ***     ****. **   :   .*.   *******  * *********

43      TAKYRKKTTYKKSGCKCKMCCCTMKWRCTAMYRSTYYATGWATMAYAKMGTYKYRARSCG
45      AGKYRKKGCTTKSSYKMKMYYMYCKWGGKWAYRSCYYCCTWGWCCTWKMSKYKYGCRCYR
        :.*****   .**. * **     **  .  *** **.  *.   . **..*** .*.

43      KTCGYTGTAYWRKCYMYKKRKGCYRYRYYGYYMMYRWYYYGKCTAGCTKMWKTGTYYMMW
45      KKTAYACAGCWRKYYMYKKRKKYCRYRYYRYYMMCRWYYYKGMCCTYYKCWKKKCTCCMW
        *. .*: :. *** *******   ***** **** *****    .   * **.     **

43      YRYWGARGCGWATCKKWRYRRTTMRYARRWWGAYKMKAWYRKWYMWWKYMKWKMAKMRRY
45      YATTRGRCTRARKYKKWRYRGCCAAYWGRWWRGTTATMWYRKWYMWWKYMKWTMRKMRGC
        *    .*     . ******     *  *** . . . **************.* ***

43      GYAKGGAGKCTKGTGMMKARYTCKWYGMYKKGGYGWYKKKMCTCRKYKYGMKMGTAAMRT
45      AYRKKAWAKTCKKCKMCKRRYYYKWYKMYKGRRYRWYKKKMYWGRKYKTACKMKWRWAAA
        .* * . .*  *   * * **  *** ***   * ******   **** . **      :

43      RRTTYYTKYKYRWMYTCCTRRMYYYKAKTYWWSYYKTTCCGGGMRWSCAYRRYCAAKKWK
45      GAYKYYYKYKYRWMYCATARRMCCYKWKKYWWSYYKWWTYRAACGWSYGCGGTAGRKKWK
           .** ******** . :***  ** *.*******     ..  ** .    .. ****

43      TKKYKARTRSSGMKTTTRSYKTTYRKKKYKMGYAGGKRWACCTMYMYGTAGMAKRSMKWC
45      YKKYKGGARCGKMKAACRSYKCKYRKKTYKMCYWAAKATRYYCACCYKKRRCWKRSMKWY
         ****. :*.. **:: **** .****.*** * ..*         * .    ******

43      RTKKWWTTTMYKKKGRKYYYMTWCATTCYRRRWMKGRYSTKKYYWYRKWKRRCYYRAKRK
45      GWTKWACCWMYKKKRRKYYYMCWYWWYMYRRRTMKKRYSCKKYCWYRKWKRRYYYATKRK
           .**   ***** ****** *     **** ** *** *** ******** ** :***

43      MGTYKMKRMKRKTWKGTWTYMRWRKWRRYRMRKCTMGKGAKYMYRKKACAWCTAKSMTKK
45      MKCYKMKRMKRKKWKSWWACMRWRKWARYRMRKYKMKKKCTCMYRKKCGCWYGMKSMYKK
        *  *********.**. *: ****** ****** .* * .. *****. .*   *** **

43      AACCGRRGCWCAGGWWKRYKYRRMRYYRMKTKCGGATTKARKYRMKSRCWTGKCGGTRKC
45      GGTTARGTAWYWRSTWKRYKYRRMRYYGCKYKSKTGKAKGRGYRMKSRYWKRKTAAAGGA
        ..  .*  .*   . ************  * *.  ..:*.* ****** *. * ..:  .

43      CCCWTTARKCWTKKWGTMCRCGKRRCKRYYRACMRWRKCYKGAAGYCTCKKGRAWCRYCR
45      YMYWCCTGGYAKKKWKWMYRTAKRATKRYYRRYCRWRKMYGRCMKYYYYKKKRMWYGCTG
           *  :    .***  * * .**  *****   **** *  .  *   ** * *

43      TGWRYCYRAGGARWCCGMKMTCKKYARKYTCAAAKKKKYGMYKKKGYYRYMRRKKKGYCY
45      AAWRYGYRWSRGRWTAKMKMCMKKYGAKYATTGCKKKKYACCKKKRYYRCMRRKKKKYMY
        :.*** **  . .** . ***  ***. **: :..*****.  *** *** ****** * *

43      KKWKRKKYYYWKAYYKRKKSRMARKRRKKMKMMYRYYYYKGKTTCCYGASYWKTAGARKK
45      KKTKRKKYYYATTYYKRKKSRAMRKRRKKCKCCTAYYYYKKKKATTCAWSYWKYRRRRKK
        ** ******* .:********  ******  *    ***** *.:   . ****    ***
```

```
43                KYTCGYKKYKYKTWRCYGCTAKYWARRATGYKYACRYRRKRWTTKAWKKRRGGACTGYKY
45                KYYMRYGKYGTGKAASYTYYGKYWTRRRKSCKYWYGCRRKGWKCKMWKKRGRRRMYSYKY
                  **   * **   .  .*   .***:** .. **    *** *. * ****      .***

43                RGTGARKCCCCGRWMMRSTRRTYTCMYKMRWKYMTYGAWRYCKASMMKYRACMGRRKMTY
45                RKKTRRKTYYTTRWMMGCCGRKYYSMYTMRWKCMCCAMAGYYKGSMMKCRRYCRARGMYY
                  * .  **     **** .  *.* .**.**** *  .   * *.**** *     * * *

43                YRRRGRCYRCCC
45                YRRRARMYRYYY
                  ****.* **
```

The pairwise alignments of two accessions 43 and 46

```
43          SRRWRAAYRRSWRWKSRSCMYMYYYRKKKYRWYMRCCCYCAYMKYRGYRARRRYWKRMGC
46          SARWRRWYRRCAGATGRGYCYMYTYRKKKYRWYMRATTYSMYMKYRRYRCRRRYWKRMAT
            * ***  ***.   ..*.  *** ***********.  *. ***** **.********.

43          YWRYYMYRKRARKYYGGWMYSRYWMYRKKTWRYRKYACWCKARRWYMWRWKSKRYYYWMY
46          YWGYYMYRKRWRGYTRRWCYSRYWMYRKKATAYRKYMMAMKWARWYMWRWKSKRYYYWMY
            ** ******* * *   * **********:  ****    *  *****************

43          MWMYCRWYKRWGTYKKRYYWYRCTYCRCKRMKCYKSYKKCGYSRWWTRKKGMYYYGTRYW
46          AAATYAWYKRWRWCTKRYYAYRTATMAAKRMKYYKSYKKMRYSRWWWRKKAMYYYTKRYW
                  *****   .**** ** :   .**** ******  ***** ***.**** .***

43          WMRSMSKMYKMYWKRGCYYRRSGWYMTWMAYWMKRYWKKWGWYGTCACMATYWRKYYYYM
46          WMRCMSKMYKMYWKRCTYYRRSAACMAWMGYWMKRYWKKWRATKYTMYARYYARGYYYYM
            ***.***********  *****.  *:**.**********           * * *****

43          WYKCKTYGTAGCGCCWCYYYGCKMMYYYGCCRYRYYMWRMKKTGYRTYSKRYATCGKYKY
46          WYKYKKYACGKSATATTYYYATKMMYYYRYTGYRYYMTRMKKYRYRYYSKRYWCTRKYKY
            *** *.*. . .. .  ***. ******    ***** ****  ** *****    ****

43          MMGCATCKCGAGCRKKYYYYWKKKKWRYRYTGYRKRMKYGTCGSCGTYGGGAGAYYYCAA
46          MMRTTATKYKRAYRKTCYYYTKGKGWRYRYCSYRKRMKYKKMRSYTYYKAARRRYYYMGC
            **  :: *   . **. *** * * ***** .******* .  *   * ..   *** ..

43          GAYMTMRAAAYYYKRSMCRYTRRMCCACCYGRYGRAWMYRKRYWKMYGKTYYKKSKAGAY
46          SMYMYCAGTMYYYKRSMTGYCAAATTWYYYKRYAGRWMYRKRYWKMYKTKYYKTCKGRGT
            . **   .: *******  *         * **.  *********** ..***..*. .

43          YRRAGAGCTKKTAKWKYKYWTYGTTTSAYYTTRMRAGRSYWTRRSMRTMCAAMYYGRWYR
46          YARCRWKYYKKKRKWKYKYWKYKKKYGTYYKKAAGRRRSYWYRASMRCMYMMMYYRRWYG
            * *.     **. *******.* .. .:**..     **** * *** *   *** ***

43          CWMATYCCGYRYTCGMMRWRYMYYRRTYTYGKKACWRRRRRKGWWKRYYYTRRWTKCMYY
46          YWARGCTTKYRTKYKMMRWRYMYYARYYYYRKKWYWRRRRRKRAWKRYYYKRRWYKYMYY
             *       ** .  ********* * * * **  *******  ******.*** * ***

43          RTCCARWTRGTYGKWARYKKKGGYRKYKRWYWGKTYRWKRAYKWMMAARCRMGGRYMYYC
46          RGYYCRWKRRYYKKWMRYKKKRRYRKYKRWYWKKCYRWKRTTKWMMWRRYRMATRYMYTY
            *   .**.*  * ** *****  ********* * *****: ****  * **. ****

43          YKYKYRMRWYGKMCMMYYGYYWKRTRKTKAKRRRGWYRRRKWTGATTTMYSKYMKMYYRR
46          YGYKYRMRWTRKMGMMCTRYYWKRWGKYKRKRRRRTCRRRTAARRYWWMYSKYMKMYYRR
            * *******  ** **   *****  * * ****   ***. :     ************

43          RKRYTTWRYMRGTGYWYRAYTYYSSYYKKKKAYKGYRGGARGCACGKKYKTRKAAATCTC
46          RKRYCWWRCCRKWRYWYRMCCTYSSYYKKKGTTKRYRTTCGATTYKKKYKKRKRWRYTGG
            ****  **  *   ****    ******** : * **  . . :  ****.**

43          TMMCRKKTTRYKKKRTYYRATKRYYKRYKYKKMSMKMRCRYRKKKTAYARWSRACTTCWW
46          AMMYRKGGGGYKKKRKYYRMKKRYYKRYKCKKASMKMRTGYRKKKCRCCGWSRGTAAYWW
```

```
                    :** **    *****.*** .******** ** *****  *****    . ***. :: **

43                  GTYKWYRKKRGYGYWACAKKKCTKKKYWGKYKRAAAARWYRYMTWRGKYMRRWMYYYKMR
46                  RYYKWYRKKRRTKCWTMRKKKYGKKKYWKKYKRMRRWRWYRYMKWGKKYMRRTAYYYKMR
                      ********     *:  ***  ***** ****     ******.*  *****  ******

43                  SYCKYKTKCWYCRKMAGGAAGRAAGGKSCTTAYGYMWRRWWYSRKYSWKYKWYMMGTWRY
46                  GTYKYKKGYAYYRKMRKTTGRGRWKRKSYYKRYCYMWRRWWYSRTTSWKYKWYMMRYWAY
                    .  ***.   * ***   :.      **  . * **********. *********  * *

43                  YYRWKKYMGYAGTGRKCTRWRWRWSKMKYYRRGYYKKAGASGTKKRWGKGGGKCTCYTYR
46                  CCGWGKYMTYGTWARKMYRWRWRWSKMKYYAAKYTKKWKMSRKKKGTRKRAKGYWMTYCR
                       * *** *.  .**  ************   * **   * .**   * .        *

43                  KRRATTCGYTAGMMCYWTTTWATTATACRRYGMAGRRKWCKKYTYAGYWKRYKSWWWKYM
46                  KRRCACTAYYWKAAMYWACGWRKYGWRYGGCRCGRRRKWYKKYYYWRYWKRYKSWATGYM
                    ***.:  .*      **:  * . .        . **** *** *  ********   **

43                  GRKCYAYYYKGGGCCYKSYWKRCRKKRYRKKYMTCCTGGRKYYKYKWYTYYGSCKAWYAG
46                  TRKTCGYYYKKKRYYYKSYWGRYRKKRCAKKYMYAMWKKRKYYGTGWYYYCAGTKMWCCC
                     **  .****     ***** * ****  **** .     ****   ** * .. * * .

43                  RCAAMRRGGYRYRRKYMCAKRRCARTYTRGTMRYYRMYARYGACTCKRKYWTCGYAGTWC
46                  RARMARRKSYGCRRKYMMMKRGYRGYTYGRAMRYYRMCMRTKWTAYKRKYTYYTYWRCWY
                    *.   ** .*  *****  **          :******  *     : ****    *   *

43                  GAYGCMRTYKRMTMMAKGWTWRCYKKWRYRRMRWGAGCRYKMKKWAWWWYKMKGYAYGMK
46                  RMYRYCGCCKRMKCMCKRAKAAYTKKWRYRGAAWRGSYRTTATKWMWWWYTMKKYWYTMK
                      *      ***. *.*  .    ******   * .. * . .** ****.** * * **

43                  RMMRKKMYRAKTYTAYRRRYYRMKRRCGKWATKGARRYYRMRCGYKAKKYKYAATSWRYG
46                  RMMRKKMYRRKWYCMYRRRYYRCKRRMCKWRKKRWRRYYRARYKYKTTKYKCTWKSWRYR
                    ********* * *  ******* ***   ** .*  ***** *  **:.*** : .****

43                  RCKKCWGWMWKRYMYKRYYYMTRWMYKRKKKYRRGGGYYWYYKKSGKRYWKKKATKKKRR
46                  RYGKATAWMWKRYMYKGYCTCARWMYKRKKKCRRRRRYYWTYKKSKTGYWKKGGKKKKRR
                    *  *. .********* *   :********* **    *** ****  . **** ..*****

43                  WSTYKRTKRYRKMMRYCRCAMYWCTWGTKRRYYYYYKKKKKTYWYKTYWKKYCRYYAYKK
46                  WSCTTGCKRYRKMMRYTGTMMYWMKWRYKRAYYYYYKKKKKYYWYKYYWTKYTGYCRYKK
                    **   .  *********     *** .*  ** ********** **** **.**  *  ***

43                  YYWAATASGGKRSYWYAKRWMAAAGRYKWMRSTGTTACYWSRCSRGRGGMGCGRMYRMWW
46                  YYWRTCTCAKTRSYWYRKRWMTGTARYKWMRSYRYYRYYWCRYGGRGRSMSYKGCTGAAT
                    *** : :.. .***** ****:.:.*******      **.* .     .*.

43                  AGATCKYAGAMYRCKCRYGGAYKKKMRYRRMKKYWWYYMYMMKKKGGYTKYCCYRCYKWM
46                  WAGCTKYRKRATGTKSRYKRRYKKKMRYRRMKGCATYYCCAAKKKKRYKGTMTCGGYKWM
                     ..  **       *.**   **********     **     ***  *.       ****

43                  KRYRRRTATYCGACTCGTRYKRKMRRKRRKCTAYKKMYKRYYYRKTYATRWYTTRRRYMY
46                  KRYRRGGCCCTATTYMAWGCKRKMRRKRRKMKRYKKATTRYCYRKKCCGAACKKRRRYMY
                    *****   .   .:   .   ********** . ***  .** ***. .    ..******
```

```
43  ARKKRYYMRRMGMTAAKYGARRATCYWKKTTAWMKCKRGRYMKTATCCTMATMRGGWRGY
46  RGKKRYYMRRAACCGTKYKWRRMKSYWKKYKMWMKMTRKRYMKATASTGMGAMRARARRC
      ******** .  .:**  ** ..**** . *** .* ****:::.  *.:**.  *

43  AYCTRKMAWKKKYKGGRAAMRYKKRWCACGCAGATAATTRYYRKWMKWYYRWKWRMMKYT
46  MCYAATAMWKKKYKRAACCMRYKKRTARTATMRWYWWGKRYYGKWMKAYYGTKWRACKTA
       : .  ****** . ..****** .  .        .*** **** **  ***  * :

43  CCARWMKKYYYMTKRACGAARSYYYYMTRRKKKGRTGCCCKMRRYWSYCGAAKGRSTAAR
46  MMRRWMKKYYYCKKRCTATRRSYYYYMWARKKKTAATSYYKMRRYWSYYKRRKCRSYGGR
       ******** .**. .: *******  ****  : .  ********    * ** ..*

43  RYYRWYRKRWRMGTCRWRCMKAGYGTMTGAGYRRKYYRRTGGGAWKKWKGTMWMRWRYCR
46  AYYRWYRKRWRCAATRWRYMKGKYKYACSCCYRRKYYRRKRRKWWKKWKKKMTARWRTYG
     ********** .: *** **. *    .. ********.    ***** .*  ***

43  CKYCKRTCTKYWWSMRRKCWKSWAAATCKGYKCTAAKSSKGMKKRSYMMTAGAKKTYYRK
46  YKYYGGCMWKYWWSMRRKMWKSWRCGCTKKYKTCGTKSSKTCKKRSYMMCTCTKKCYYRK
     **      ********* **** ..  * **  .:****  ******* : :** ****

43  MKCRYKYCYRMMMKYYKKMRKGYAATMYMYTAGGMKKKKRSSMKWKARYMRCYTYKWKMC
46  ATYRYKYAYRMCAKCCTKMAKRYRMYACCYCGARMTKKKRSSMKWKWGTCRMYKYTWKMY
     . ****.***  *  .** * *      * .. *.**********    * *.*.***

43  GGYKRKKCRWYAKWYWKKRRWKGAWRKMSWGMWRTACATTKKWMMGYKKKKTGKMGATGG
46  RKYKRKKYRWYTKWYWKKRRWKRCWRGACWKMTRCGTGYYKKWMMRYKKKKKKKCAGACR
      ***** ***:********** .**  .* * * . .  ***** *****. * ..:

43  ACYRYAWMRYTYTTTWKKRTGCGAYKGCTARYYYTKYRWGCAWKGAKYSCTGKYYKRTGC
46  GYYRYRWMRYKCWYYTTKRYATTTYKKMCRGCYYKKYRTRTGTGRCTYSYWRKTTKRKRY
    . *** ****.     .** .  :**      **.***   .   ..**   *  **.

43  CWCACTKKYGWYGRTKATMRKKKYMCKKYCAAATAGTTYRYTYRRYRWWYWKWKKTTTAY
46  TAYTTCKTCSAYRAYKWCMGGTKYMMKKYYTCCATACCYRYWTGRYRWWYWKWKKCAWWY
       :  *. . *   *  *  .*** *** :..::.  ***   *********** :  *

43  KKMYKKRRYCTGKYAATYYCKTCAKYTCMACAKRRTTKMKKYRKKRKKTYWWSCGCGSCS
46  KGMYKKRRTACCKCGGCYYSGCTRTYYMAGMGKRRYYKMKKYRKGRKKCCWWSYAAKGYS
    * ****** .  * .. **.    .*   . .***  ******* ***  *** .. . *

43  MRYMGKMKRYSRYYKKAACYTGTCCTAYTGTYACAAYYMKRCYMRAKCKGRMYKATGWKK
46  MRYMKKAKRYSRYYKKRRMYCRYSYYGYAAGYRMWWYYCKRTCMGGKYKARMYKMWKWKK
    **** * *********   *   .  .*:. *    ** **  * .* *.****   ***

43  MCYKYGCRAYWWMKRYKYASYRYMYWKRRRWKYKAGACKCCRYRRRRAGAAKCRKKYKTC
46  MYYKYKTRCYWWMKRYKCGGCRYMYWKRRRWKYKRATTGYYRYARRRGAGRTYRKKYKYY
    * ***  *.******** .. ************* .:    ** ***... . *****

43  TYMMWYRSYYGTCMRRRMCCTCAGGGGRRSKCGYMYYYWYYYTCTARMWYRMTKRRWYRW
46  WCMMWYRSYYACTMRRRMYMKMRAAAAGRCKTAYMCCTACCYGYYRGMWYRMGGRRWYRW
      ********.  *****  .  .... *.* .**      *     *****  ******
```

```
43          RKKYKYRGYKYMGYCGGGYRWKYTAMACATGYMGRYRKRYWTMTWRKKWARGRAGKTTGA
46          RKKYKYRKYKYMRYYRKRYRWKYKRCWYWKAYMRRYGGRYTCMCWRKKAMGKRRATYYKR
            ******* **** *    *****.     ..** **  **  * ****    *  ..

43          GAWKKAWMYRMRMAGGGRWAYMKTTGGMYRKYTYKAAARYKYMYCGCAGGKMYKTRRMRY
46          AGWKKWWACAAGCMRAARWRYMKYYKKMYRKYCYKMWRRYKCCCYKYWRSKMYKKRRMGT
            ..*** *        ..** ***    ***** **   ***        .****.***

43          YATRMKTTCCGGGAKGGKRTMSRATARGARSRKGWMGRGWRAKTRRYWKCGKCAGRCMYM
46          TRAGCKCWYYKKKRKKAGGWMSRMAMRRCRSRGKWMKRRARWKYRRYWKYRGMWARYMYC
              :  *        * .   *** : * .***  ** *  * * *****     .* **

43          RKCGTY
46          RGYKYY
            *    *
```

The pairwise alignments of two accessions 43 and 47

```
43      RRRMKRYRYGRATYRRMKKTTRTTKRYYYRYWWRTRRYYYKRMWWRRYYTAGMSKWRYSR
47      AARMKRYRYSRWAYRRMKKCARWWKRYYTGYWWRKRRYYYKRMWWRRYYYGRMSKWRYSG
          *******.* :****** :*  ****  ****.************** . *******

43      RYWRMTYRKKMYRKYMRRKRMYSKKSKCWMRACGRWKSMTCYYCYYWRYRRSRYWWYRRG
47      RYWRMWYRKKMYRKYMAGKRMYSKKSKTWMGCYARWKSMYYTCAYYWRYRRCRYWTYRRK
        ***** **********  ********* ** . .*****    .*******.*** ***

43      YYKKMWMWYKMRMRYWGGWYYCCRKWKRYWGRMYRRMYRWYKKCKRYWKYKMRMYAYMYY
47      CYKKMWMWYKMGMGCAKAACYTTGKWTRYWKRMYRRMYGATKKMKRYTGTKCGCTMCMYY
         ********** *    .  *   **.*** *******   ** ***   *      ***

43      TCKRWYWTRYKRYAGWGRYTKWYYMWMRRYYRRSKYWKYMKCMWYMRYRRRWKSKYCYSR
47      YYKRWYTGRYKATWTWTRYWKAYYMWMRRYYRRSKYWKYMKTMWYMRYRRRWKSKCYYSR
          ****  ***    * ** * ******************* *************  ***

43      WWRWKSKWKATATATYRRRMMYMYRWGGWKRMRYYYRWYYYKYKYKTKKKATSWRYRRCK
47      WWRWKSKWKTCGCMKYRRRMMYMYRWKKWKRARYYYRWCCYKYKYGYKKKWKSWRYRRYK
        *********: .  .***********  *** ******  *****  *** .****** *

43      YWGWMYRMRYMYCMRYYTWWTTATMTRAWSYTKKYMKRKKMTCCKTYGCGATGCKCYYWR
47      YTRWCYRMRYMYTCRYYCAAGWCYCYRMWSYWKKYMKRKKMATGKKTAAACWCYGTYYWA
        *  * *******  ***     .   * *** *********:  *. ....     ***

43      YMYRKKAKCSYYKWRKKKKCYTYRGAYMKRGKKKYYYYMWYAKRMWCGSTWWGCWYYYRK
47      TMYRKKWKMSTYKWRKKKKAYACARTYMKRKGKKYYYYMWYMKRMWSRSKATKTWYYYRK
         ***** * * ********.*:   :****  ********* ****. *.    ******

43      GYKKMMYYYKYRYMRYYMKYYATWRGKMKKRKRRRTRGKKRGGTYKMKYRKGYKMYYYWM
47      RYKKMMYYYKCRTMRYYMKYYMKTGRKMKKRKRRRCAKKTRRRKYKAKYRKKYKCYYYTM
         ********* * ******** .   *********   *.*  .** **** ** *** *

43      MWGYACKKKWMWRYRYAKGYRCKMKMYYRTRYRRYYGKRRKRYMYMRKWAYGAAATGKYM
47      MWTYGTKKKWMWRYRYMKSYRYKMKMYYRWRYRRYYRKRRKRYMCMRKWWTKRWRYSKYM
        ** *. ********** *.** ******* ****** ******* ****       .***

43      GCYMKGYYYRYTGAMWYGGCCKKWRYRTMWGAYKMKWYARCCATMKMMGRYYKAWWRRMA
47      RYCCKACTTACKAGMWYCAYSKKWRYRKMWRGYKMKWTGATTCKMKMMKRYYKGWWRRMW
            *.     ...*** . .******.** .***** .   ..**** ****.*****

43      AKWRMTYMKRSAMRTGWKGKMARWWYGRKYKMMKKAKKRAAASYMMMAKCKACYGKRYKK
47      RKWRMWYMKRSTARKAWGRKMWRAATRRKYKMMKKMKKRRWRSCMMCTGYKGAYKKAYKK
         **** *****: *..*  ** *    ******** ***   * ** :  *..* * ***

43      RYYRRGKGWCTAYGMATMYKKRCTCWYYRRKRMRSYMGYRMGWKYKYYCATGKTKMYARM
47      RYYGGKKKTMCRCRMWYCYKKRMCTWYYRGKRMRSYAKYRMRWKYKYCTRWKKYKMYRRM
        ***   *       *   ****   **** ******  *** *****     * *** **

43      CKKRGGATCKYAATYKATTTTARRAYCMCTTSYKRYRWKMTWYYYYYMRKKGMRYRWGWS
47      SKKRRCGKMKYRWKCTTGCYKRRRCYMCYGCSYKATAWKMYWCCYYYMAKKKMRYRWRAS
```

```
            .***  .. **  . .:   . **.*     ***   *** *  **** ** *****  *

43          YCGAAKMYRRASKYTYRRWKRRAKRWRARMYYAGKYRKMKYYTTTRRGGGAYYWWKGRCM
47          YYKRRKMYRRRSKYWYRAWKRRTKRWRGRMYYRKKYRKMKYYKKGRRRRKWYYWWKKRTM
            *    ***** *** ** ****:****.****  ********.. **    ***** * *

43          WRAARMKYCKKMYKRYTRRCTKYKCAYYKMWCKRSMRCAYYRKTRRMCCKSKYKKTTGTK
47          WRGGRMKYTGKMCGAYCRRMYKYKTGYYKCWYKRSMRYGYYRKCAAATTKSKYKKCARWK
            **..****  **   * **  *** .*** * ***** .****      ****** :  *

43          KGCGTASAWRRRYMRCCRKKSKKGKKCRYYWKWWYRKYKWYTGAARATCKRKKSRKGMKM
47          KRTRYGGCWRARYMGYTRKTCKKKKKYRYYWKWWYGKYTWYYRGCGCYYKRKKSRKKAKM
            *    ...** ***   **..** ** ******** **.**  .. .  *******  **

43          KRAKYYYYRYYYMYRKKCCYAGCKSYWTAGKAKRKRGYYRKKYTCRGYKKKKWYTYRYYW
47          KRCKYYYYRTCTMYRKTTTYGCTKSYWYRRKRGRKRKYYRKKYKARRYKKKKWYYYGYYW
            **.******   ****.  *.  ****   *  *** ******..* ******* * ***

43          YSRRTKGTKKKYGRYRGTKKKCTAWYMYCGGTYRMGRGRKKYWYRGCMTAGGAWARGWAY
47          YSRRKKSYKGKYARYRKWKKKYYRWYMYYRSYYGCRRKRKKYWYRKYMKRARMWCACWGC
            ****.*. * **.***  ***   ****  . *   * *******  *. .  *.  *.

43          YAATRYRARYTYMYKSKRKYRCYRKMRKYMRKKYTWRWAKAYRRTTGKWWKCYTCCCGAG
47          YCGKRYRRRTCYMYKSKRKYRMYGGAGTYMRKKYATRARKCTRRYYKKWWKTCCYMYSRA
            *...*** *  ********** *    .******: *  *. **   ****      . .

43          RGKCCGTMCKKYRTKKRYWWSTCACGCGCGSCKSYAKKMMYKKRGKMKYSRRYGTMWRKT
47          RKKTAAAMMKTYGKKKRYWWSCMRGRYAAKGYKSYRKKMMYKGGKKMGYSRAYTCAWRKY
            * * ..:* *.* .*******      .. . *** ******   ** *** *   ***

43          TAKRACKYMMYMTTAACAAYYYTTYWTKKCRMYKWKKYMYKTKKYWARKMRYMYYRKTCY
47          YWKRRMKYMMYMKATGTTTYYYKGCWGKKYRMYKWKKYMYGCKKYWTRKMRYMYYRKCYY
              **  ******.::. ::***.  * ** **********  ****:**********  *

43          ACTKYKGAAAAKAWYMYYAKKCKKKGTKRRYYAAAKTGGATTTRGKRYGRKGTTGMMMRS
47          WYAKCKKMWRMKTWCCCYTKKYKKKKKKRGTTRRWGAKTGKAGRAGGYARKAKARMMCRS
              :* *     *:*   *:** *** .**       :  ..: *.  *.**..: ** **

43          GWMTMYCGRYARYTKRGRYWRRKRYWKRRYMRYGTYKGTKCTRTAYRMKYYGRSWRRKWK
47          KWMAMTTAGTRRYWKRRAYARRKRYWKARYMRYKYYKRCKYCGCGCAAKTCAGSWAGKAK
             **:*  .   ** **  * ******* *****  **  *    .   *  . **  * *

43          RYMCWGRTTWMYYTATMGMYYRYYRWRMTGKKRWRRKAGCGKKCWKYRCCYKRCRRGGRT
47          AYMYWRRAGWCCTYCCAKCCYRCYRAAMYRKKRWRRKRRYRGTTWKYRGYYKRYRGRKRK
             ** * *: *    .     ** **  *  *******     . ****  *** *   *.

43          RYRYKAAYKRYTGGARSMTCGYCKTTTCKRRYRRYRMRKTAGKYYTCRWKKTTACTTRWK
47          GTAYKGGTKRYCTATGSMCTKCYKACAATGGYRRYRCGTYTCKYYKYGWKKCARTAAAWK
               **.. ***  .: **     *: :..  *****  . : ***.  *** :  :: **

43          WRGCKWYKKRMYACTGCAKKKKKKKRRKYAYAGARYYRKKARRMKAKCYKKYWWGYYYGY
47          WRTYKWYKKRACGTCTAGKKGKKKKRRKYWTRKRRYTRKKWRRMKGGYTKKYWWKYYYKY
            **  ******  .   ..** ********     ** *** ****.   ***** *** *
```

```
43            KKMKRYRRRKRRTKGTTKTAYRRKRRYYMMYATGTTYGYGGATAGCRRKKRYMKRRKYKK
47            KKMTRTRRRKRRKKSKWKYMYRRKRGYYMMYMYRWYCAYKRWWGATAAKKRYMKRRKYKK
              ***.* ******.*.. *  ***** *****      .*    ..   ************

43            CYYKYTTRRWYKRKRRRRKRYGMRRRWRMMKYRYMKKCCRWYWKYWAMKMTTKRWKWRTT
47            MYYKYYKRAACKRKRRRRGRYKMGRRWRMMKYRYCKKYYRWYAKYWWAGCYWKRWKWRGG
               **** .*   ******* ** * ********** **  *** ***      ******

43            RMTARYRKKKKMKYMMYSRTKKKYYRGYYWKCYRYYTKYKGWRGCARYKRYGYYRRKKRY
47            RMKWRCGKKKKMKYMMYSAKKKKYYRKYYWKYYRYYYTYKRAGTAMRCKRYRCYRRKKRY
              **. *  *********** .****** **** **** .**     . * ***  *******

43            AYWGKSKWMMKKKRRAYKRYYAMRRRWCYYYRRYRRWKGACYWYAAYCSKSTTCTAMKYG
47            CYWKKSKWMMKKKRGGCKRYYTMRRRWYYYYRRYRRWKATTYWYGGTYGKSKYYWRMKCS
              .** ********** . ****:***** **********.: ***..  .**.    ** .

43            GAYSYYRKWKMCAATMAGYYCARYWYMYGRKKRWKMYKATKGGKTACTGYAKYGRKAWKA
47            RRYSYYRKWKMTCGKMCAYYGGRYWYMCRRKKGWKMYKMKKRRTARMYSYRKYKRKRTKR
                *********  ...*..** .*****  *** ***** .*  .:   .* ** **  *

43            RWKCCCCGRKAWRWMMRSRRMGRYSYCYKYKYYRYRWKMRWKGKCGTGYGGGAGRRCRAA
47            AWKTYTTTRTRTRWMMGCRRMRRYSYYYKCTYYRYATTMRWKRKTAGTYRRKRARRTRRR
               **     *.  **** .*** **** ** .****  .**** * .  *    .** *

43            CKKYWRRRYRKRYARYYMTTKKKAYAGCGKRWRRYGWWYSKMKKYYYRWMRWCYRRYRGA
47            YKKYWRRRYRTACRRYTACCKKKRYGTMAKRTRRYKWWYSKMGKYYYRWMGWMYAAYRKW
               *********.    **    *** *.  .** *** ****** ******* * *  **

43            GTAKCGKRWKMWYCYKTMGATKKMGRYGMAGKTRTCSKYCKKRGTCTRYWWSKYKKYTAY
47            KKCKSKKRTGMWYTTTGMRRKKKMKRYRCGRKYRKMCKYYKGGTCAARYWWSKYKKTWMY
               ..*. **  ***   . *  .*** **   . * *. .** *     .:*********   *

43            KYKKKKKKKYKTKSSMMSWKYYRWCYMMYGTKYRYKRMGKTYMKCYRSKKMYWWKRYMRY
47            KYKKKKKKKYKKKSSMCSWKYYRWYYMMYRCKYRCTRMKKYCMKYCRSKKMYWWKGTCRY
              ***********.**** ******* ****  *** .** *  **  *********   **

43            WTKRKGRCRWKGAMYYKTTAGCKKTYYKGKKRYKKCYRRWMMRKRRTAGTTCYCAAATGY
47            WWKRKRGAAWTAGMYYTCCCCGKKKTTKKKKRYTGSYRRWMMGTRRYRRYCMYTTCCAKY
              * ***  . *...***.  .  **.  * ****. .****** .**      * :..: *

43            TWYRRYTKRKKGAMGMAAYTKKYYGAYWKKYYWCKKKRTYRWGTKAACTKMWKMRGRAMM
47            WWTGRYKKRKKRRMKCMMCWKKYYRRCAKKYYWMKKKRYTRATGTTRYCKMWKMRKRMMM
               *  **.****  *      ****    ***** ****  *    .:   ****** * **

43            TAKKGAMRTGWRGRKMKKKARMYKKKRWGAAAAYGGTYKWMKMYRYWCKGRMMRMKYWMK
47            YWKKTTMRYAARRATAKKKWRMYKKKGTKWMRRCAAWYKWMKCYRYTMKKRACRMKYWMK
                ** :** . *  . *** ******        .. ***** ***  * *  *******

43            CYAGKYKMWGKTKAWAARYRMMRYMYKATWACRYKRYRYYYYYKKTMKGTKCKKKARYKR
47            TCCTKYKCWKKCTWWRRRYRMMRYMYKRYWRYACKGTRYYYYYKKGAKAAKYKGKRGCKR
                . *** * * . *  **********  *     *  ********  *.:* * *   **
```

```
43      KRKKKKWAYWAATRSYWYGWKYRRYRGWYMRMYKKWTTRKWYRRYWKRRYYYRRRGGGCA
47      KAKKKKTGYWRTCRSYWYTATYRRYAAATMRMYKKWYKRKWYGRYWKRRCTYGRGRSSYW
        * **** .** : *****  .**** .  ******* .**** ******  * *  ..

43      GAYRWMRRMYYK
47      RRCRACRRMYYK
           *  ******
```

The pairwise alignments of two accessions 43 and 48

```
43              GWCGKCRMCWMRMKCCGSYKYYYKYYRRATMAYYRRGYMRMKYKYYWYWKMKATARRWWK
48              KTYRGMRMYWMRMKYYRCYKYYYKTYRRRYMRTYAAKYMAMTTKYYWYTGAKGCTRRWWG
                      ** *****   .****** ***  *  *   ** *. *****   *. :****

43              CKRYYYRWWWYWRYYWRRWWKRYWWWWWRWMRKGMSRRKYGCCYMKYRRRMRKRRGYRKR
48              SKRYYTGWWAYWRTCARRWWKRTWAWWWRAAGTKMGAGGCRYYYMKYRRRMRKRRAYRKR
                .****  ** ***   ****** * ****   . *.       ************.****

43              STYMKRRAGKWYSMARYSSKCYTMYRTCYRAWRYYYRKCTYRKYRKGRYWRWKSRYRYSR
48              GAYMKRRRKKWCCATRTCSKTYYMYRWTYRRWRYYYRKAAYRKYGGTRYAATGCRYAYSR
                .:*****  ** . :* .** * ***  ** *******.:****   **    .** ***

43              WYYWYYRRYWKYGKWYGGRCGAGATCGYKCMYYSRKRMWCGAWKRCCGAAKCRYRRYKWW
48              WYCTYYRRYWKYKKTYKKRSAWRWCGTTTYMYYSRKRMWAATWKGTSAWWGTRYRGYKWW
                **  ******** * *  *..       . *********..:**  ..    *** ****

43              KMAAAGMMSYGWMRWRRMMYRKMCAYCARWKKTTTKYRYWKSKRWYTCYYMRYKRMMYRK
48              KMTGRKCMSTRWMRTGRMMYRKMTWTTTATGKAAKKYRYWKSKRWYWYCYMRCKRMMYRK
                **:.   **  ***  *******    :   *::.***********   *** *******

43              KCAAKYYYKGRYCTKAYCRTTTGYKTMKKRMRRKKYCAAATARWRWKYTAWAYRRYYYAA
48              KARGKTTYKKRYTCKCCAGKAYKYKWMKKRMRRKKYTTCCATATGTKYWRTRYGRYYYRM
                *. .*  ** **  *. . .:  ** ********** :..::    **    * ****

43              YRYYYKKTSYYYSRYYYGKGAYKWAWGGCYKWGKCKKRCTKWMYRGKWRYRMRKMRGGAY
48              YGYTYKKCGYYYSRYYYKKAGCKWCTKAYCKWRKTKKRAAKWMYRKKWRCAAGKMRRKCY
                * * *** .******** *.. **.  .  ** * ***.:***** ***    ***  .*

43              RRKYYYKYKKYKSMKRKYRRKRRKKWKMYKGRRMRYYAKGGWGGWKARRSRGYRSKWRYC
48              RAKCYYKCKGCKSMGRKYRRKRRKKTKMYGKRRMGTTRKRKATAWKRRRSRRCRSKWGCT
                * * *** *  *** ********** ***  ***    *    .** ****  ****

43              GCKCTGWTKKGTYRMWRKRYKKCCKCAGYRCWMRKGTYYYKRYRSYSMRGKYRRKKKKMA
48              AAGMKKWKKKKWYRCARKRYKKYAGMWATAMAARKKYYYYKRYGSYSMGRKYRRKKKKCG
                ..  . *.**  **  ****** .   .     **  ****** ****  ******** .

43              GTYRYWSKCRWGCKGWYRGKATKRKCKKKKYMMKRWYMRYCTGMCCKKRRYYTTKYAYRW
48              KACRYWSKYGWTYKKWYRKKGKKRKYKKKKYMMKRWYMRTTAACTAKKRRYYKYKYMCRW
                 : *****  *  * *** *..*** *************  :.  .******. **  **

43              AYTGTWGCKYWWRATTGKGARKMWYAWRYRCTMRRWWYKMKGCWGWYMMMWYWMYYMMTK
48              WTYRYWSYKTWWRWKCAKRRRKMWYRWRYRACAARWWTTATATWKWYMMCTTWMYYCCWT
                     *. * *** . .*  ***** ****.   *** . .. * ****   ****   .

43              RRMRRGGCRYKMTKMYWKKWMKMMRYMTAKACCKAGMMKKTYGWRGYTTRMYRMRKKRTA
48              AACRGRATRYKMCKMYWKKWMKACRYMYWKMMMKGCMMGKWYRARRCCCAAYRMRKKRAG
                   *  . **** *********  ***  *   *. ** * *  *      *******:.

43              MRYKKRWACGAKCCMYAAKGKKKGMYRYWKKRGMKYCARYWGKCAYRYGYKYMTRRYMAG
48              MRYKKRWRTATKYYCYGGKTKKKTMYRYWKKRKMKYMRRYWKKSRYRYRCKYCKRATCTA
```

```
                    *******  .:*   *..* *** ******** ***  *** *. ***  ** .*   :.

43                  RRGGWKWKCARMMKAGARRTYRKYWKWYKKMWCCCMRRRKRKWMMRRGYYATAKTAMYKK
48                  RGATAKWKSWAMCKGCGRGKCRKYAKWYGGATYTTAGRRTRKWMMRRKYCCCGKKWATKK
                    * .  ***.  * *. .* . *** ***         **.******* * . .*.   **

43                  TAYYGKMMKRYWKRYRRKMWCAKYWGMARRRYRYCCTYYKYRTAAGGYWMKRYKGCKWGT
48                  CGTCTKMMKRYWKRCRRKMWYMKYWRMWGRRYRCGTWYYKYRWCWARYWMKRYKKMKWRY
                     .   ********* *****  *** *  ****    ***** . . *******  **

43                  RYMYSRRRYKYRRTTGKYRRWKRRRYYRRAGAYAKMAGGKMMKATWRTATYRSTYYYYWW
48                  RYMYSRGAYKYRRCCRKYRRWGRAGTTAAGAGTRTMTKKKMMKGCWGKWGYRSCYYYYWW
                    ******  *****   ***** *      ...  .*:  ****. * .  *** ******

43                  KMGCCTKRKYMCGYYGGYRKCCYGSMKKRKYMWYWKRMKKGCGYGYCATCGGTTCCWCYY
48                  KMKYMKKGTCMTACTRKCRKYYCASMKKRGYMWYWKRMKGRSRYKYTGCSTTACTMTTYY
                    **   .* . * .     **   .***** *********  . * * . .  :     **

43                  YYGCYMRYKKYMYMYYYYTRYKGRMMKKYRYSRRGYTRMYMMGKYYAYGKCYCTRYKRYR
48                  YYRYYMRYKKYMYMYYYYWRYKKRCAKKYRYSRRKYCAMYMMAKYYTCTKYCTYRYKRYR
                    **  ************* *** *  ******** *  ****.***:  *    ******

43                  RRYYYYWTAWGCGGKKKTWYMCTWRYRYYSYRKMYYRRRYRYRGCCGCGWTAKMRGRRYY
48                  RGYYYYTKMWRSRRKKKYWCMTAWRYRYYSYRKMYYRRRCRYRRMTRYKWWRKCGRRRYY
                    * **** . * .  *** * * :*************** ***       *  *   ****

43                  YRAGCWGYKKKTYKKATGASWRYACRCAWWMAYRMRYYYYRYYTWAWMTGRTGAWKKYMC
48                  YRWSTWKYKKKKYKKWKKMSWRYMYATRTWMMYRMRYYYCRYYCATACYRRWRWWKKYMA
                    ** . * ****.*** .  ****      ** ******* ***  :    *   *****.

43                  CTKRKMKTAGGCYTYYWKKYGKYRKKKYYRCYRRKRMKYGTCGCKMGKYYKGGGAMTKRY
48                  MCKRKMKCWAKYCWYYWKGTRKYRKKKTYRYYRRKRMKYKKMRTKMTKCYKRRSMCYKRY
                      *****  .    ****   ****** ** ******** .   ** * **  .   ***

43                  MRTYGMYYYKRKGTRSMKRRRYYRTRRMCCYKYRKKKKRGTGSRKMYARCKKKSKKKKRG
48                  MRCYRCYYYKRKAKRSMKRGRYYRCAAATTYKYRKKKKRAGAGAKMYRRYKKTCTKTKRR
                    ** *  ******..***** ****      *********. .. *** * **...*.**

43                  GCYWKTWKCATAWYYAWKKCACRWYGYYWKSSMKKRKGWRKKMCAKRMCSRWYCAKKMYK
48                  ATYWKKWKTCAGWTYGWKKYMMRWYACYWKSSMKKRKSWRTKCTGKRMGSRWYTGKKMYK
                    . ***.** .:.* *.***   ***. **********.**.*  .*** **** .*****

43                  TACMKRKYYSKMKKGRGKYRRCCAMKCYRYKCAGCMYYYYKKGCRYWRGKKRGKYMWGAK
48                  YRYMKRKYYSKMKKKRKKYAAATWCKYYRYKMTASMYYYYKGRMGCAGAKKRKKYMWAGK
                       *********** * **   .   * **** :..******       .*** ****..*

43                  YRGRYKCCGYARYGYCKAMRRMKKYWWRYYYMYRYKSCRYRRCCRKTRRYRCGWKYYTKT
48                  YRKRYKTMATCGCTYYKTMRRMKGCATRTCTMYRYTSTRYRRMYRKYAGCATATKCCYKY
                    ** ***   . .    * *:*****     *    ****.* ****  **      . *   *

43                  AKGKYATTWMMYKYWRWYMKRYGKWRKKSKYGRKYRWYRRRYKGATCRRYMMGATYRGCG
48                  MKKKCRGAAMMYKYTGACMKRYKKWRKKSKYRRKYAACRRRYKSTKYGRYMMRRYYRRMR
                     * *   : *****    **** ******** ***   *****.:.  ****   **
```

```
43          AYYRYMRKKKTYWWSSKKSGWCYKMARYMTARKKYYYWGRKRRKGAATAACKYTGTKYMG
48          WYYRYMRKKKCYWWSSKKSTATCKMMRYMCTGKKTYYWTRKARKSRRYRRMKYAAGKYMR
             ********* ********    ** *** : ** *** ** **.      **:. ***

43          RCARAYCMTTTGATCAKYYYTYMWRYARWMYKKATGTGRCKGKKMYYYRKTKTGAKKKWY
48          ATCAWTYMWYARTWTTKYYYGCAWRCRRAMYKKCAKWKRSKAKKMYYYRGKKGAGKKKWY
              .    *  : :  :****   **  * ****.:   *.*.******* .* ..*****

43          RKYWGYKCCCMRKYYAKGAWYWMYATRKMAGSRYYSCWRWMYRRKRRMKRWRCKKMYYKY
48          RKYWKYKSYYMRKYYRKKGTTAMYGGRKCCASAYYSMTRWAYRGKRRCKRWRYKKMYYKY
            **** **.  ***** * .   **. ** ..* ***  ** ** *** **** *******

43          RKKRCWWGRTGGGATWKWKMGGTKGSYKCKKMRRYKKKTTYRGKGRMRWKSYKYKKKYKS
48          RKKRYWWRRKRRKWYWKWKMARYKASYKYKKMRRYKGKWWYRKKAACGTKCYKYKGKYKS
            **** ** *.     *****.  *.*** ******* *  ** *.    *.**** ****

43          SMACCYCGKGRSYSWMMMKKTTYRYRWKYMKYRMGGKKRCMTMYMAMAKRGAKRSKSMYW
48          SMRTATTRKKRSYSWCCMKKACYRYRWTYMKYRMARKTAYACACCWMWKRKRKRSKSMYW
            **  .   * *****  ***: *****.******. *.        * **  ********

43          WWKAKWKWATTMWYKAGWKRKCGGWGKTGGCRAGYTWYGGTKKSRKKRWWCCAGYKKMKK
48          WWGWKWKAGCWMWYKWKWKRKYKRWKKYRKAAGTYKATAAWKKSRKKRWWYYWTYKKMKK
            **  *** .  ****  ****   * *   . . *.  .. *********    ******

43          RYKYYYSYWAAYKKRCTAACTCRKKKMTWWYRKYTCGTRGYYKYTAAYAKKYKAGAAAMT
48          RYKYYYSYWTTYKKAGCTWMKYRKKGMKWWYAKYKMAKRRCTKYATCCTKGTKMRRMRMY
            *********::***   :  . *** *.*** **. ..*   **::. :*  *     *

43          KYWMYYYCTCATKRRGWKRGYRYYKTRGCTAYMYCGTYRMGYCTGAGYKRRTGCKKYCAA
48          KCWMYCTAYTGCKRRKWTRKYRYYKCRKYYRYACTCCTGCAYMYRGRTGGRAKYKKYYWW
            * ***  .  . *** *.* ***** *    *        .*   .    *:  ***

43          YRYKYYRRYMTATGCTATAAGYWRSMYCARYTRYRRMMYMYKSRRYRMRWYKWTRMRYKR
48          YRYKTYRRYMKRGKYCTYCRACTRGMYTGRYKRYRGAMYMYKSRRYRMRAYTTCGARYKR
            **** *****.     : . .  *.** .**.***  ************ *.    ****

43          KRCCAGKTYCWGAKWAATWCRYTKGTCCKKTTGCCGTRYRYRTRYYYCGYTKKKYYKGKR
48          KRATCKKGYTTTRKWGTAWTRCCGKCTTKTACATAAAATGTGKRYYYTAYAGKKYYKRKR
            **. . * *    **.::* *       *.: . ..:     .**** .*: ***** **

43          TGAKRWYWRRYSKRYYKRWKWRGKGTTTAGAKKGKRWKYRRYMYAGGATGATKYTATKKA
48          YRMKGWCWGRYSKGYYKRWKWAKKKYKWWKRKKKKGTGYRRYMCWKRWKRRKKTCCAKKG
               * * * **** *******  *  .    ** *   *****     .  .*  .:**.

43          RGTYMAKWKKRYRMCGGARTMYKWRKRRYKRYYYYYKKKKYTCWTYSKMKRYYYTYWYAA
48          GACCCGKWKKRYRAATATAYMYKWRTAACKGYYYYYKKKKYYSWCYSTCKRCCYACTTMG
             .   .******* . .:  *****.   * ********** .* **. **  *:    .

43          KKKTASKRSYWYKKYRRYYRWYMKKWTTRSGCRWTYATKGASRRGGKAYMRMRRRARMKM
48          KKKKRSKRSYWYKKYRRYYAATMKKWYKRSAYGWKCCWGKRGRGRSKGCCRMRRGCACTM
            ***. **************   **** .**.  *. .    .*  .*.  **** .  .*
```

```
43                  RYTGRRKYRRRRMRYTTWCYRCCTWKYYACRSMRMTCWYGRMKKMKKYYYTYRGGGAATK
48                  RTCARRKYRRRGAGYKKAACRYYYWKYYRTASMRACYWYRRCKKMKGCYTKYRRKATGYK
                    *  .*******   *.. . *   ****   ***   ** * ****  * .**  .:. *

43                  KYRYAAAWKCGCYYYKKRAKCRGYATYRGKMWRMKYYRCTTWYRMKKKKKATCGCYTRAA
48                  KYRYRWMAKTATYYYKTRWKMGRCMYYRKKMWRMKYYRYYKWYRMTKKGKRYMRYYKGGG
                    ****    *  . ****.* *      ** *********  .****.** *     *. ..

43                  GRCKRGGKKKKKKMMRYAAAGGTWARWRYMCTGWACKKRKYYKRYYCCRCCTCAAAMRMM
48                  TRTKARKKKKKKKMMRYTTGTKYTGRTGCCYKRAMMKKAKYYKRYYMYRTTCAGCGCGCA
                     * *   **********::.    .*     .    ** *******  *   ....

43                  KKYKRKWYAGCWGKYKYKMCYRYCGTTTARGKTRKKKTTAGRWWYSKTSKYWKTCSYYRR
48                  KKYGGKWYRKYWKKYKYKMYYRYSKGGATRAKYRKKKYKRKRWWYSKCSKYWKCTGTCRR
                    ***  ***   * ****** ***.   ::*.* **** .  ****** *****  .  **

43                  RKKYMMTWRCYY
48                  RKKYMMYWAYCC
                    ****** *
```

The pairwise alignments of two accessions 43 and 49

```
43          YARRTYYWRWYRRWRRRYRWTYYYWYRSAKTGACRWWGRTYRWWWMRYRWYRGRRRYKYY
49          YWRRCYYWRWYRRWRRRYGWKTYYWYRGRKAKRMRWWKRKYGWWWMRYRWTRAAGATKTY
            * ** ************* *. *****. *:   *** *.* ******** *.    * *

43          RKWMMWCYRWKKASYMRRMRMKWYRYMRMKARRYYWRAMTCAWWMRYRTRKKGGSKCYSY
49          RKWMMWTYRWKKMSYMRRMRMKWYRYMRMKGRRYYTATMGATWWMRCRYAKGKSSKMYSY
            ****** ***** *****************.****  :* .:**** *  *  .** ***

43          KRYWAAYTMRMRMYYKWYRKYKRWRRWGTTTGYGWYYAWMYRKWTRTMYMYYYRKCRWYY
49          KRYWWRYYMRMGCTTKWYRKYKRWGRWRAAARTAACYMWATAKWCGGMYMYCCRGMRWYY
            ****  * ***    ********* ** :::  .  * *   **   ****  *  ****

43          RRWYWKYYWYRRRWYMKYKYYTRKYRWYRMKYYYKCMMWKKRKYWRRKMRMYKWMWYRYS
49          RRWYWKYCTYRRGATMKYKCYCRKYRWYRMKCYYKYCAWKKRKYARRGMRMTKWMAYRYS
            *******  ***   **** * ********* ***   ****** ** *** *** ****

43          YSKAYACYRAYYRKYRKWRWKCYWRRYCAGKWMKKMCTRRWMKSKWKRMTMCYWRMSYKK
49          YSKRTCTTRCYYRKYRKTRWKAYWRRYYRRKWMKKMAARRWMKSKWKRMAMYYWRMSYKK
            ***  .  *.******* ***.*****   ******.:***********:* ********

43          SGKKATTTYCYWRTKKKYKYRGGKKGKYYTAAASKGCATAAKRRACYKCTWCGKRKYYRR
49          SRKKRWKGYACTGCKKKYKYRTAKGSKCCKRWRSKAMMGGTGGRRYYKYYAYAKRKYYRR
            * **  . *.    ******* .* .*  .   **.   .:  *  **    .*******

43          TATAKRYYRGRGTCGCKMGTGCYYTKMRRYKATACCRTTKYYYKYYYYWCRSMRRTCCAA
49          YMKMKRYYRKRKKMRYKMKKAYCYKKMRRYKGYCYTGAYKYYCKYYYCWTRSMRGKGAGG
              . ***** * .   ** ..  *.******. .   : *** **** * **** . ...

43          YKYKGKWMYRACYYMYGARCKSKKGTKRKYWKTYAATMGKKWKRKYCKAWKWTMACARYM
49          YKYKKKWMYRWTYYMYKRRYTCKKKGKRTYWKKCRMWCRKKWKGGYTKRWKWYMWTRRYM
            **** *****  ****  * ..**  **.***.      ****  * * *** *   ***

43          MAGCCKYYRKYKMTGTRTYGWRGKRKMTKMRYKRWWTAYAAAAKRMRYKGCKRKKKKGGC
49          MMRMMGYYRKYKMGAYRCTRWGRKATACGMRYKRWTARYTCGGKRCRYKRYTGGKKKRKY
            *     ******* . *   *  * .   ****** : *:...** ***  .  ***

43          TMGAYRYWGKYRTRMGTATTATKKRAACTYWMMKKYTYGGMKRKRKGCAYRYKKSMMCRC
49          ACRTYRYWKKYRCRMACTACGAKKRGGTAYWMMKKYYYKKCKRKGKKTGYRYKGSCMMGT
            :  :**** *** **. :: .:***.. :******* *   *** *  .**** * *

43          GCKRRYKRYYGKMKGTTMRWSRAWRARGRWWYWRKRTKRMMWACAKKSCGMTTYGAWCAK
49          TAKRRYKRYYRKCGAGCMRWSRRWRGRRRWWYWRKGKTRAMWTMRKKSMACCACAMATGK
             .******** *  .  ***** **.* ******* ..* **:  *** .  : .   .*

43          YTKRYCKRYRTYAGGYKKRTTWRGKRYYRGWRRRKRMYRYYWKMCYTKAYKYYTTKYYAY
49          YCKACAKGYRKYGTAYKKRWYWRKKATYRKWGRRKGMYGYYWKMTYGKMYKYTCCKTCWY
            * *  .* **.*. .****  ** *  ** * *** ** ***** * * ***   *   *

43          AGKKYTKRRRMYMWYYMKGWMKGRKGMCYRKYSGGAYCYGRGKYARYKARKTWRGYRWKK
49          WKKKYYKRRRCYMWYYAKRWMKKGKRCTYGKYSATGYTTRGKKCRRYKGGKKWRKYRWKK
```

```
              *** **** ***** * ***  *   * ***. .*     *  ***. *.** *****

43          RMTTRRRGTGKKKYMRRCCKKYTGGTYTAGRYACYRRYWKKRKCTCACAAAGTYAMWYMY
49          RCWWGGRRWRTKKYMRRTMKKYKTACYWWARYGYTGRYWKKRKYCGGYCWRKKYCAAYMY
            *     *   .******  ***. . *  .**.   *******   . .   .*.  ***

43          TGTRAYYRGTKWYKCAYKYCAAKKRWMMRWYRMYYTGYGSMRKRRYRKYMWYWKRMKWCG
49          YRKRCCYGKYKWYKTRCTCAGGKKRWMMRTTGMTYCACASMRGARTAKYMWYWKRMKWSR
              .*. *   ****   . ...*******   * * . .***  *  ***********.

43          RYKKWTMWYYYKYYKMRYMYYYKRYKKRMWGRKKKTGYRKRRGGMATACYKYMMYKTARK
49          RYTKWWATYYYKYYKMGYMYYYKRYKKRMWKGKKKCAYRKRRRRMGCCYYKYMMYKGCRK
            **.**   ******** *************  *** .*****  *. . ******* .**

43          KKYYYWKYKWKKKKGRWMTKGYKCTGGKTTYKYKRAAARTCKWYYRGWYMTKKGGYGGCG
49          KKYYYTKTGWKKKKKRWMCKSYKTCRKKGYYKYKRMRRRCTKWYYRAAYCKTKAATARYR
            ***** *  ***** *** *.**    *  *****   *  *****. * ..*.. .

43          YRMKWKYRRYKYKMYRKKCKKYAAKKTTAYYTGCASKYRATKGKWMMWYYYRKCTAMYYY
49          YRMGWKYAGCGYKMYGKKMKKYRWKKYKRYYKKMCSKYGRCKRKWMMWCCYGKYYRAYYY
            *** ***    **** ** ***  ** . **.  .***   * *****  * *    ***

43          KYKGCCKKYKYRYYGAARMAGTKKCGGRWRACTCAAAMCTGGTYMYKSRRRRYYCAYKKR
49          KYKKYAKKYKYRYYCGCRMTCCGTTKRRWRGYKYMWMCMKAKCTAYKSRRRAYYYCYKTG
            ***  .******** ..**:   .   ***. .      ..    ****** ** .**.

43          KGMRYKRKATARCRSKKAAMGTTGKWKYTKATTCGTCACRTRCRWYGYWSSMKKRRWKAC
49          KKMRYKRKCCTRYACKKMRCRYYKKWGCCKCAATRCTTGRYAYRWYRYWSSMKKRRWKGA
            * ******. :*  .**       **   *.::    : *   *** ***********..

43          ARKSWKWRTARAAYKTACMAKKYMMMKYSYRMKMKKKKRYYWYCYYRKRKCYRYYKKKRM
49          CRKCWTTRCWRGRYKYRYMGKKYMMMKYSYRMKMKKKKRYYWYYTCRKGTYYRYYKKKRM
            .**.*. *  *. **   *.***********************   ** . *********

43          YKKWGCKATAAKYCCGKRYAGTKTYGACTCYTTYKAYKYTYRRRYAAAWWWRRRCGRKMY
49          YKKWKMKGCTGKCTTTKRYGCCGKYRRYGYTCKTKCYKYGYRRGYMRGWAWRARYARKAY
            ****  *. :.*    ***.   .*       . *.*** *** *  .* ** * .** *

43          RYRGYKKRMRRRGKKKYYKKRCRMYYKKKCTKYYYGMKYKKRYWWSSTAGGRSKAYAGRY
49          RYRRYKKGMRRRRGKKYYKKRYRMYYKKKSYKCCCRMKCKKAYWWSSYRKRRSGRCRRGC
            *** *** ****  ******* *******. *    ** ** *****    **

43          CCMRYMTAYKRKMKAYCGYKTTKKRYRYYRTGTKYMACAKRYATKWKTSRRYKGGRMYKA
49          AYMRYMYWYGGKCKCYTAYGYKKKRYRYYRWRKTYMTTTKRCMGKWGKSRRCKCARMYKC
            . ****  *  * *.* .*  .********  ..**: :**   ** .*** * .****.

43          TGWGMAKKCMMMYYKTGAMKKKKTYWRMCRKWWKTYGYRRKTKYTKSMAAAGGKYMCCYG
49          AKWKARKKMACCYYKGAGMKKKKKYWRAYRKAAKYYTYRRKKKCYKSMMWRTTKCCYYCR
            : *   **    *** ..*****.***  **  * * ****.*  ***     *

43          TMKYRKRAKRKCKWKMYTAATKYWKRYKRRMGRYYAACKTGGGTTTACMYMSRRTCKMRS
49          KCGYGTRWKRGYKWKMYYRWYKYWKRYKRRMRGYTTCTGCKKKWCACYMYMSRGYYKCRS
            .  * .* **  *****    ********** * :.        :. *****   * **
```

```
43          RKKWMRKGGTYCGAKRMATRKRYYKKGTCYKYKSKYCTCYMRRYKYGYKYKKKKKKYRGA
49          RKKTMRKKKWYYRWKRMWWRKRYYKGTCMYKYKSKYYYMYMRRYKTKYKYKKKKKKYRKM
            *** ***   *   ***  ******    *******   ******  ***********

43          YWATCGCYRGCMCYYMGKSYMMTGTKKYRYRKWYKKKKRYMYRYRWSMRKKTWKCMYMGR
49          CTTATTAYGRMMACCCAKSYMMAACKKYRYRKWYKKKTRYMYRYRWSMATKYWKYMYMKR
              ::  .*   *.   .*****:. ************.********** .* ** *** *

43          MKKRSKKSMKWWKRMAWKAAGGCTTSRYKGRKYYYMAKYKKMRGCTYTRRRWMGSYCGAA
49          MKGRSKKSMKWWKGMRWKWGCTTACSRYKKRKYYYMRKYGGMRRYKYCRRRWMKSYYKRR
            ** ********** * ** .   : **** ****** **  **  .* ***** **

43          KGMYRRKYYCRWYRRWGKRWKRGRTCMARWRRRACGRKMYAGAKYKKYMRKYCCAAARRG
49          KKMYRRKCCYRWYRAARKRTGGRRWYMTRWRRGGYRRKMYRKRKYKKCMAKCTTGCCRAR
            * *****   ****   **    *  *:**** .  ****   **** * *   ...*

43          GGATAMWWYYGWKWRAAGAGMRSYKTCTWKMYKKKKAGRRGGKKWWRGRWRWWMMRYSWY
49          RKWCGMWWYYKATTRRRRWRMRGTKATCWKMYKKKKGCRGRRKKWWGKRWRWWMMATCTY
                .*****  . *     **. *:  ********. *   ****  *******  . *

43          CGKARWKWCGTGMKYKYYGATAAWMYRRRYYYAKRRRGRRRYYRCRRYRRGTTGGYMRRW
49          YRKRRWTATAGTMKTKCTARYRMWMYRRGYYYMKRRRARRRYYAYGGYRRRAARKYMGGT
              * **.  .  ** *  .    ***** *** ****.*****    *** ::  **

43          TKAMGARRKRYCATYGGRRYYYRGMYCRTGTTRWKGKWATSYRMKAGCWYMRATGGCGCM
49          GTCCKGRRGRYYRYYRARRYYYRKAYYGWRWYRWKKKWWCSYRMGCAYTCCRCWATATYC
             ..  .** **   * .******  *      *** **  ****  ..    *. . .

43          KKYKSGKYRKACRRWWGGYKMKWMKKRGMKYRMGRRWRRYYKTKYRCRYRYAKYKKCAKK
49          KKYKCRKYRGTTGRWWAKTTATTMKKGTMKYGMKAAWRRYYKWGTRYRYAYRKYKKYRTK
            ****. *** :  ***.  . . ***  *** *   ******   * ** * ****  .*

43          YRRMYYYYRMTCMKRKRRMRMTCKRGTTATCGKKTYYKKWARRKYRRKRYRRTGYWYMMY
49          YRRMYYYCGCATMKRKRRMGMKYKRSKWTATKKKCYYKKTGGRKYRRKGYRRGACAYMMY
            *******   : ******* *. **.. ::  ** **** . ****** *** .  ****

43          AYKRYMKRRYKKRYYKYTCCCRRRTMYWRRYMKRTKTGYYYACRGTRGYCKACTRAGSKM
49          GYKRYMKRRYKKGYYKYGTMYRRRYMYWGRYMKGGKYKYYYWSAACRKTYTGTWACKSKM
            .*********** ****    *** *** ****  *  *** . . *   ..   . ***

43          MRYWYRCYCRRYMYKCCTYRKKWRCARYRRYYYKCRTTAKAGWTCKMMKYRSCTYYKSKW
49          MRYWYRMYYARYMYKTYWYRKKTAYGRYARTTYKYRKKMKGKTCTKMMKYRSMYYYKSKW
            ****** *  *****   ****   .** *  ** *.. *.    *******  ******

43          RMKKKYRKRMTKRYMYYYYKKKRRRYMAMRGYAKGGTCWATKRMMRAWKYYYKTGKTKYW
49          RMKKKYRKRMKKAYMCTYYKGKRRRYMMMRRYRKRKCYWGYKRMARRWGCYYKYATKKYW
            **********.* **  *** ****** ** * *    *. *** * *  *** ...***

43          KSWRYGRRCKTCCYMWRYTMYYKYCCCCGRWGTCYKRCCGTGCKRMKKGGTCKGAGATAW
49          KSWRYRRRYKATAYMWRYGMYYKYYYYYKRWKATTGRTTTATAKGMKKARGGKAGAMYTA
            ***** ** *: .***** *****     ** :   *   : .* ***.   *...  :
```

```
43      YGYYWKTKWSWGRKYRTTGCYYYCSKKKSRWMRRRKKMRYYRACGYKKKKKCYSYWYGKR
49      CAYYWKAKASWKRKYRGCRAYTCYCKKKSRTCGRRKKMRYYRCTKYKKKKKYYSYWTKKR
         .****:* ** ****   .*   .*****   *********.  ****** ****  **

43      KKKRWAKRGYCMRKCKYYYYWCKRRKYRYCCARYRMRYSKKACGCAYYYYYKKAGRYGKA
49      KKKRWWKRKYYMRKMKYYYYWYKRRKYRYYYRRYRAACSKKRYRTGYYYYYKKCRAYKKR
        ***** ** * *** ****** *******   ***   ***    .*******.  * *

43      YWRYMWATTYGWRRRKKCYRYWYRRYYYAKYAKYARARSYWYKYRYYWRYRWYKMGYTWM
49      YWGYMWGCCYRAAGRTKTTRYWYRRYCYRGYTKYGARRSYWYKYRYCWRYAATKMKYYWM
        ** ***.  *    *.*  ******* *  *:**.  ********* ***   ** * **

43      RSGWRRYWKTRYAYRWKRMCYYKKARAAATA
49      RSKWRRYWKGRYRYRWKGCSYYKKTRWTTGG
        ** ****** ** ****  .****:* :: .
```

The pairwise alignments of two accessions 43 and 50

```
43                GGGYYRMYCWWYWRWCGCMMRYKYRMYSKYYRMYYYRRTGTMYRRCGMYGKMYYYWGRWW
50                RRRYYRMYYWWYWRWTAMMAAYKYRMCSKYYRMYYYRRCTCMYRRMKCYTKMYYYWRRWW
                     ***** ****** . *  ***** ***********   ****   * ****** ***

43                KRRWWWRMWMRRYCCYYKRRKWRYMYYWRWMYWMYYMYRYRSKWWMYRAARWKSMRCYKK
50                KRGWWWRMWMRRYYYCYKRRKWRYMYYWRWMYWMYYMYRYRSKWWMYRRMRWKSMAAYKK
                  ** **********   ********************************  ***** .***

43                MMRYWYSYRGCTAYTTCRKWGYMYMYCKRMYWYRKGTAAARYRYWKSYKMCCRWYYMWSA
50                MMRYWYSYRRYGGYCCYAKWKYMCMYYTACYWYRGKWWWWGYGTAKSYKMTARWYYMWSW
                  *********   .*    ** ** ** .  ****       *   ***** .*******

43                KRRKYRARMKGYMWKRYYRRYWKRYYRKGATYWMGRMYRKYKRWMRKKAGKWRWKMRMWW
50                KRRKYRRRMKRYMWKRYYRRYWKATCRKTCCCAMRRMYRKTGRWMRKGTCKWRTGARMWW
                  ****** *** ************   ** .   * *****  ***** : ***   ****

43                RYRWKRRMRMKRYAYWRCSMKRMRYCTKCKYYWGWYRYYYKKTCGKRMTYWWSRCAASGY
50                RYRWKRRMRMKRYWYWRGCMKRMRYAATYKYYWAWYRCTYKKYYKKRCYYWWSATRCCRC
                  ************* *** .******.:. ****.***  ***   **  ****   ..

43                CAYKKMKYYKYSRKTMCSAWRKYRRYAACYKMYMRRKKKYYKTCAGRRTTSRKRYCTRTM
50                MCCKKMKYYTYSRKCMYSRWRKYARYGGAYKMYMRRKKKCYKKYGAAAACSRGRCYWGWM
                   . ******.**** * * **** **...********** **. ..  : ** *     *

43                YKRAGWKYMYGATKKKKKYRTWGMRYMYKRAKTGGYRKRKKTTMRKSMAAAKCGCTYRRK
50                YKRCKWKYAYRRWKGKKKYRKWRARYMYKRGKYKKYRGAKKCWARKSMMWRKTTTCYRRK
                  ***. *** *   * *****.*  ******.*   **  **   ****   *    ****

43                TKRKKKTKKMKYYWKRYKRRMYKKTGGMKSRYRMRRMWRKMRSCKGGRKCTKGRRWRGTM
50                KGGTKKYKKAKYYWKRYKRRMTKGYTKMKSRYRMRAMTGKCRSYTRTGGMKKKRRWRKWM
                  .  .** ** *********** *    ******** *  * ** .     .* ****  *

43                YCGRYAKCGTTRKCATRRYWCYRKYTKWKKKWMTTCKMMTAKKMTRKGRKWYGRGKKRMM
50                TTAGTRKYRCGRKSMWRRYWYYRKYWKWKKKWMYYMKMMYWKKMKRGKGKAYRRRKKAAM
                    .   *    **.  **** **** *******   ***  ***.*   * * * **  *

43                YKCTCYGGYKAMKRWKKTRWTAAAAKMGTRYYATGMKYYRYWSWYRGAACMMMGKKYRAA
50                YKTCSTRRYKWMKRWKKKRTAWMRRKCAARYYGCAMKYYRYWSWYRRRMTACMKKKYRRR
                  **  .   ** ******.* :    * .:***. .***********      * ****

43                CYWMMWWGARTCYGAYGRAGYMRRYGGTAKRCTRASKTTGRKKGCKRYYRWMGYKKTKWM
50                YYWMMWWKTRCYCSCYRRMRCCRRYKACGKRTWGWSKGCCRTKKYKRTTGWMKYKKYKWA
                   ****** :*   ..* *    *** . .**    **   *.*  **   ** *** **

43                TGYKKSRKARKMRMYMRRWAYKWKYYMCCRGRYKKKKWKYTKKRYAAKKYRYYRYYYMKG
50                YCYKKSRKGRKMRMYMRRWWYKWKYYMAARARYKKGKWKYYKTRYRMKKYRYTRYYYMKR
                    ******.********** *******..*.**** **** *.**  ***** ******

43                YKKKRRYRWMGAKAWYWGYKRARRRRYRKWRKMKKRRKGMRWRWRKMKYCCKGKKWRKYC
50                YKKKRRYRWMKRKRTTAKYKRWRARRYRKWRKMKKRRKRCRWRWRKMKYYATKKKWRKYM
```

```
        **********  *     *** * **************  ********* .. ******

43      RRRRRKWKYCMRYYYRRYWMMGMRKKYCAWYMYTTSGTKWGRRYMYWRWARYGACRCATY
50      GGRRRKWKYACRYYCRRYWMMKCGKKYYMWYACGYCKCKWKRRYMCTRWGRYTGTATGKY
          *******. *** ******   ***  **    .  ** ****  **.** .   ..*

43      KSRRCYRARCKMAAARTTGKWAMGTKTKRYWYWRMKYWYYRAYKTGGWYRMKKKGCTTAA
50      KSRRSYGGGMTMCGRRYYKKWGMAAKAKRYWYWRMKYWTYRRTKWRKWYRAKGKKYCYWT
        ****.* .  .*.. *   **.*.:*:*********** **  *   *** * *     :

43      WKYTCGCTRRKCTKKKKATKTRKSYCWMMAAKRKGYRKYKRCTYKRYYRMYSACKGWTAG
50      AKTYMRYKRGGAYKKKKRYKKRKSCTWMMGTKRKTYRKYKRTCYKRYYRMYSRYKRAYRA
         *     .* . ****  *.***  ***.:*** ******  *********  *    .

43      TYYYYKKWRTKYWRRKRRYYKYWGTSYWYYRRRYCGRRYYTTMRSGTTKTTRRYWKGYKG
50      CYYYYKKAGYKYWRRKRRYCKCTACSYWYYRRRYYKRGCTYKMRSKWKGWYRRYWKACKA
         ******   *********  *  . *********  *    .***  .   *****. *.

43      AYWRATARKYYKYYSWKRCGTMCCKYWKRMKCGYWKCAAYYWRWYYYYKKWKMRYMYYYC
50      GTWRCYRRKYYKYYSWKRTACCTAKYWKRMKSRYWTSCTYYWRWYYYYKKWKMRYMYYYY
        . **.  *********** .   .*******. **...:********************

43      KRYATYKMTRMKKRGSRKKRYKTMYKYMMKYRYMWKYRYMYKRRYRRKYYYYWKGYWCKG
50      KRTWKYKMYGMKKRKCAKKRYKCMYKYMMKYRYMWKYRYMYKRRYRRTYYYYWKRYWYKK
        **  .***  ****  . ***** ************************.****** ** *

43      WMKWRYRYGYKRWYYWSSMKRWKGACAWRKRMSWYKRYKWYGTACKKWSKRATKYKYRMK
50      WMKWRYRYSYKRWYYWSSMKRWKRGACWRKRMSWYKRYKWCRYRYKKWSKRMYKYKYRMK
        ********.************** ...*************     ******  *******

43      RKYYKKKYYCYYKCKGMKCYRYMRKYGKCYKRYKWRYYKYKAGGCCMAAAKWYYRATMWC
50      AKYYKKTCCTTCGMKKCKYYRYMRKYRTYYKRYKAGYCKYKRKRTSMMRRKWYYRTGMWY
         *****.       *  * ******* . *****  * ***    .*   *****: **

43      KKRATMTYRMYYKMRSKYYRGKMGGAAGRKKYSYYRWWYSAYSYGGCMMKYKCRYTMRKR
50      KKRTCAYYRMYYKMRGKYYRAKMTTTGRGKTYSYYRWWYSCTSYKRMMMKYKSRYKMGGG
        ***:   ********.****.**  :.  *.*********. **   *****.**.*

43      YYKTRMRYYMKGYYGYKKYWGYRRCCRKWKKTACAKACCRRRYRRTAGTMMYKRRRKKKR
50      CYKYRMRYYMKKYYKYKKYWTYRRMYRKWKKCRYRKMYMRRGYRRCCCCMMYKRRRKKKG
         ** ******* ** ***** ***  *****    *   ** *** .  **********

43      CGCYAKWCKATGKRAYRTWRYRRYMATTKYGKRAKYKRYKSMMTGCGKWYRYYKRKKMYY
50      MKYYGKWYKGCAKRWYRWWRYRRYMGCAKYKTAGKYKRYKSMMCKYKKWYRYYKRKTMYY
           *.** *. .** ** *******. :** . .*********    *********.***

43      MWSKRWWKWYKMKWYRKKRRAGTWKKARWKKYKYRYRYMYYGTKYYYKKTKGGGGYWWTT
50      MWSKRWWKWYKMKWCGKGRRGAAWKKTRWKKYKYRYRYMYYRWKYYYKKYKRAKKTWWKK
        **************  * **..:***:**************  ****** * .   **..

43      YWMYYRWKMMARYKWTWYGAGAGGTWKKGKTCYTYCMYYYATGAGKKTMKWGCKMARTKT
50      YWMYYRWKMMRRYKTYTYKWKCRAATKKKKWMTYCGMYYTCCKTATKWAKAKYKMCGAKC
        ********** ***   *   . .: ** *      *** .  :..*  *   **. :*
```

```
43          RYMKWGWKKWKCRGACAKMYKACCCGGACTGYRTYMWYRWRKRWMMRRMWYRMRWYCYRY
50          RCCKTTTKKWKYGAGTGKMYKMYSSRRRMYSYRKTMWYGWRKRWMMRRMTYRCGTTTTGC
            *  *   ****  .. .****  ..     .**. *** ********** **

43          KGKWMWKKKKRKMWYYKRGRYYTKCCCKYYWRYGCKKKYMYWRRRGKYRRRCYRRYKKGC
50          KKKWMWKKKKRKMWYYKRKRYYKKYYYKYYWRYKMKKKYMYWRRRKKYRRRMYRRYKKSY
            * **************** ***.*   ******  ********** ***** ******.

43          KAMKYWYRCAGGYYMYCGCMRRWYMGYRKCCTMRRWKKYYRKRTYWKKRRRYRRWKRMMR
50          KCMKYWYRAGATYYMYTAAMGAWYMKYRKTAGMRRWKKYCRKRKYWKKRRRYRAWKRMAR
            *.******... **** ..*  *** *** . ******* ***.********* **** *

43          KWCKTKYWKRRRATSWRYRCYKKRKYYRMRTYGMWTYGCCCARKWSYKRKMCRKAGRRGY
50          KWTKYTYWKRRRWKSWRYRYYKGAKYYRMRKCTAAAYATYTWRTWSYKRKMGRKRRAGRY
            ** * .****** .***** **  ******.    :*.    *.******* **     *

43          YWKYKKYRWKTGRKYYRKYRKKYCRYMRRYKKTTGWKSKKKKCRKKRYKGMYGGGGYKWK
50          YWKYKKYGWKKKRKYYRKCRKKYYRYMRRYKKWWRTKCKKKGMGKKRYKAMYARAAYKWK
            ******* **. ****** **** ********    *.***   *****.**. ..****

43          ASWMMKKKYYYRKGMYRWSMYCAKRWKKTMYMKTRSKKWWTTGKKWKRKKYWKGGRRYSW
50          RCTMMKKKYYYRTKMYRWSMYTRKRWKKYMYMKKRSKKWWKKKKKWKRKKYWKKAGAYSW
             . *********. *******  ***** ****.******.. ********** .  ***

43          WRKYKYYRRYRMRSKGWKTYYWKRMGRWRWWTKYRYMYRASMYYRKKRWRRRRRCGKRRR
50          WRKYKYYRRYAAGSTKWKKCCWKAMRRWATWKKYRYMYGMSMYCRKKRTGARRRYRTARR
            **********   *. **.  ** * **  *.******  *** ****   ***  . **

43          WRYKGYCCAATYRKRGTCGCCKKMGRYKAKWYYYYYRSMCCRRAGYYRRRMKYYKSKMWR
50          WRYKKTTSMRKYRKRKKMRYYGKMKRYKGKWYYYYYRSMATRGTTYYRRRMKYYTSKMWR
            ****   .  .**** .     ** ***.**********. * : *********.*****

43          WKRMGKTKKSKKGYKCWCRRAKKSWKYWMKWKYCAAAGYWCKYMMYYMGTMCCCKRRYYG
50          WKRMKKKKKSKKKTKYTAGGMKKSWKCWMKWKYYWRMKYWAKYMMYYMKCCTAAKRRYYR
            **** *.*****  *  .   ***** ******     **.*******    ..*****

43          MATCTYRKRMCMWTMYWWCYMRGCACKWRYRATCACTTACGTRMWWGGKYKMKKKAGMKY
50          MGWYCYRKRMTMWYMYWWYCCRATGYKWRYRTCTGTWYCYRGRMWWKRKYKMKKKTKMKY
            *.   ***** ** ****   *. . *****:  .   .   ****  *******: ***

43          MKTGYMGMKKRRMCTAMGMAMYRRKYYGRRYKKRYRTRRRWYYKRKGMRKKKKRMRTGKK
50          MKWAYMKAKKRRMTCMMAMGMYRRKTTKRRYKGGCGWRRRWYYKRKRMGGTKKRMRCRKK
            ** .**  *****   *.*.*****   ****     ********* *  .*****  **

43          YAAATARYWYRYYYKKGKYKKGGATRGTGTKRYYCYKARYWKTYKYSYMGRCA
50          YTCCATRYWTGYYYKKKKYKKKTRKRSWSYKRCYAYKRRCWKYTTYSYMARTT
            *:..::***  ***** ****   .*. . ** *.** * **  .****.* :
```

The pairwise alignments of two accessions 43 and 51

```
43                  RYRYRYWRRYYGWCKWRRMKKGTMYKGRTYTCGTCTMKRYYSRYSTRMYYWRRWYWYAKK
51                  RYRYRYWRGYYKTYKWRRMKKTYMYKSGGCAYAAYYMKRYYSRYGWRMYYTRGATWYRKK
                    ******** **   *******  ***.   : .:  ********. **** *   ** **

43                  RWRTRYRRSGRYWRWKSAWYSYRRMRWYYRRRTYWKWYWWRYRKMYYKRWMYWMRRYGKC
51                  RWRYGYRRCKRYWRWKSMTYSYRRMRWYYRRAKYWKWYWWATRKMYYKRAATWMGGYRKM
                    ***  ***. *******  ************ .*******  *******   **  * *

43                  SMWYYYMCCRGKRWYRRRYWRMYRKCRWKKYYRKTCTGTRKTTATRRKYYYYYMMYTKKA
51                  SMWYYYMTTRRKRWYRAGTARMYRKTRWKKYTGKYSWKCRKYWWKRRKYYCCTMCYAKKR
                    *******  * *****    ***** *****  * .   **   .*****   * *:**

43                  RWKSTTARRRMRKSRMYYYWRWWYRWRWWRRMRRMSRYRYYWGKRSSKMYRYWMYWRTCY
51                  RWKSCCGRRRMRKSRMYYYWRWWTRWRWWRRMRRMSRYRYYTKTGCCTMYRYAMYWRWST
                    ****  .**************** *****************  . ...**** **** .

43                  SYYKCRKSKYRRYKRTWSRYYKYKTAYRTYYGKGRKKKGKKWRRRRWYRWKKMRRKKYMR
51                  SYYKSRKSKYRRYKRCWGRYYKYKKMYRKTTTKKRKGKATKWRRRRWYRWKKAGGTKYMR
                    ****.********** *.******. **.   * ** *..************   .****

43                  RAKKKKYCAAATAYWMKYRYYWWYWGRKAAGTYKTMYSTKGRYGKYWYRKWKRRYRYYKY
51                  RWKKKKYYTCCATYWMGYGYTATTTRRKWRKKYKAMYSYKRRYKKYAYRKWKRRCRYYKY
                    * ***** :..::*** * *      **   .**:*** * ** ** ******* *****

43                  MWKYGKRRRRMRMMRCRYAKRRWKGTATKKKRRSWRYGRCYYWMMWYRMRMYAYRYGYWM
51                  MWKTKKRRRRMAMMAYRYRKRRWGKWMYKKKRRSWRYKRYYYWCMWYRMAMYCYGYRTTC
                    ***  ****** **  ** ****     ********* * *** ***** **.* *

43                  WWMKTACRWSARWKRKMKRKKKTYCGRRWYYWYAYKTKKRKAYRCGKKGYRRKKKKYYRY
51                  WWMGYRYRWGCAWKRKMKRKKKCTTTRGWYYWYTYKKKKRKWTGYKKKKYRRKKKKYYRY
                    ***    **.. **********    * *****:**.****     ** ***********

43                  RKYRMYRKRYMRYRCTYKMKAGTRMKKYWKYYRYYYMYRKKKYKKKRYRRGAGKYTKARK
51                  RKYRMYRKRYMGTATGYKMKTAYRMKKYWKYYRTCTMYRKKKYKKTRYRRRTAKCYKMRK
                    ***********     ****:. **********   *********.**** :.*  * **

43                  RYRRKYRRRMMYGKKYYMKRRYYRTRKKSKKCGCYRCCCCCCMRYYRKARYRYWGAGARY
51                  RYRRKCGRRMMYSKGYYMKRRCCGYRKKSKKMTTYRATTYGTMGCYRKWRYRYWATKWGY
                    *****  *****.* ******    ******   **.     *  *** *****.:   *

43                  KYMKKRRTCAKRKKYRWWSSCAYAKMYYKKAKRACTCWRGKKMKRRRGAATKKAAAMGYG
51                  KCMKKRRCTGGGKKYRWWSSMCCMKMYYTKTKACMCTWARKKMKARRSRRYKKMGTMRYK
                    * *****  .  ******** .  ****.*:* .   *  **** **.   **  .:* *

43                  RRTRYMWCKKRYYYYCTYWTGRTRTKRYAGCMYKMYKATGWKKYYMYYYKTGTAMKKTWW
51                  GAWATATYKKRYYYYYGYWGAAARCGRCRRMMYKMYKCWKWKKYYMYYYKGAYGMKKKAT
                            *******  ** . :*  *    ******.  ********** . .***.

43                  WRRMKYGTCCKMYKTRRCYKRCAYYKYKKGRSMCKCGAWKKYCCKTTRTMKKRSWRRMGR
51                  ARRMKYKKMTKMYKCRRTYKGYGYYKYKKARSMAGTRGAKKYMYKAWRCMKKRSWRRMKG
```

```
                  *****  .  **** ** **   .******.***.    . ***   *: * *********

43                KKCAAAWKRRKKYKWKKMKWYYTCYKRKRWKSMKRWKWRKYKTMGRAWYKTACWSKRKTW
51                TKTCGTWKRRKKYKWKKCKWYYKACKRKRWKSMKRWKWRTYKKMKRCWCKYRYWSKRKCT
                  .* ..:*********** ****.. *************.**.* *.* *   *****

43                CATYMMKYSYRMYWRKMKKSKYYYYWKYYKCYRYTCGGYYKKYYKKKWRKKKRKYGYAYY
51                SRYYMMKYSYRMYWRKMKKSTYYYYWTYCKYYRYYYRKYYKKYYKKKAGGKKRKYRCMYY
                  .   ****************.*****.* * ***     *********    *****    **

43                GKKRMCTTGTYGGRGSKTKSRKKKKKRRKKMKRRYCKKMMYRKWKTAGKASRRKRKYMRY
51                KKKRMACGAAYKAGASKCKSRTTKKKRRKKMKRGYTKKMMYRKWKYRRKGSRRKRKYMRY
                   ****.   .:* . .** ***..********** * *********    *.**********

43                YKKKKKTCRKATKYWYTMTCAKTATRYRYTTGKYKYTCAGYTTRYTRMKKKWKYCRKWWR
51                TKKKKKYTRKMYTYWCYCYTGKYRKRYRYCCKKYKYYYMKCCYGYWAATKKWKCMGKAWA
                   *****  **   .**      .*  .****   ****         *    .****   * *

43                KYYWYWYRSKWKRTCTARWGWKTYRCYYMYRMYRKKYRWRRRKRCGKYRYYYYKRCRMYY
51                KYYWTTTGSKWKAYYYWATKTKKYRYYYMYGMYRKKYGTGARKKRYRKYRYYYYKATRMYY
                  ****    ****          *.** **** ******    ***  ********  ****

43                KCCAYCACRTCKYRMRKKKYKRAAAKWYYRKKYTYRMWGCYYYKMGKRGTCTKYRMKYAY
51                KTYMCTRYGYTKYRMRTGKYGRMRRKWYYRTKYYYRMWKYCYYKMKKRTWYGKYRMKYRY
                  *          *****. ** *   *****.** ****   **** **    ****** *

43                RARGWWYSKMKYKYRTSYRRWMYYYYKWTTTWYRMRARYMKYTGKKKSKYKYAWKSMKAT
51                RWGRWWYSKMTTKYRCGTRRAMYCCCTTGAGWYRMRRRTCKYYKKKKSKYKYMWKSMKRC
                  *   ******. ***  . ** **    .  : ***** *   **  ******** *****

43                CCYMCYGRCCTKKKKWKMYKYAGKMSRKCAYRSYCGYKGKRRWRWKTRMRMGMGRWRYKR
51                YTCCYCRRYYWKGKKWKMYKTRRKMSRKYGYRSYYRYGKKRRWRWKWGMRMRCKAARYKR
                         *   * *******   *****  .****  *  *******  ***     ****

43                RYWKKRCCKCAGCGWKKGTYYRYMYRYMKYYTKKYYTYTRKWRTKRKMYYYRGRMAAMAT
51                RYWTKAYAGMWAYRWKGKYYYRYMYRYMKYYKKKYYYCCRKWRYKRKMYYYRTRMGGMTA
                  ***.*   .   .  **   ************.****    **** ******** **..*::

43                ATKYRGMYASKAGGTKYRWKYRYCKRYRGYYGAKYYCRTCGCARYMAKKWKYCWKKKWMK
51                TCKYRTCYCGKCRAAKYGWKCGCYKGYRRYCKTTYYYGKYRYWGCCGKKWKYYWKKKWMK
                  : ***  *..*. .:** **    * ** *  :.**   .       .***** *******

43                MMRYMMKKKMTCKKTTAAMYACTKKYMRAAKKGATRRYKKYRGTTTRYMKMYYRYWCRYG
51                ACRYMMKKKMGSKKGKWMMYWMYKKTMRGMKKARWRRYKKYRKYYCRYMKMYYRYWMRYK
                    ******** .** .  **    ** **. **.  *******    ********** **

43                KYRAACMKKYRYWMMWWRYKTCCYAYYMRTASYYYYTTRYKGSCKMMKRYKRCKMYRYKY
51                KYRRMTMKKYRYWMMWWRYKCYTCCYCCRKRSYYYYCWRYKRSYKMMKRYKRMKMYRYKY
                  ***   **************     .*  *. *****   *** * ******** *******

43                RKRRWKKGRYTRYYCYKCSSRKKKMMKWTASYARWYMYRGTWGKKKYYYRYMCYYYKYRK
51                RKRRWGGRRYYATTYYKYSSRKKKMMKWKWSYRGWYMYRAKWAKKKYCCRYMGYCYKCGK
                  *****   **      ** **********. **  *****..*.****  *** * **  *
```

```
43          KYKCTKYMRRYKKYTYKYYAAGYTKKKKKRYKSSKRSYCTGKKYRYRKCGTAKKRYMWYR
51          KYKMWKYMRRYKKTAYKYCTMKTYKKGKKRYKSSKRSYYYAKKYRTAKYAGGKTRYMWYR
            ***  ******** :*** :    ** ***********  .****  * . .*.******

43          YMRYGRTYGTCMRMKRSRKMYCRYMTKWAKTWTKMCKYGGRMWRGCMSKKKYYRRCTTTG
51          YMRYKAYCKCYMRMKRSRKMYYGTCWTARKKWYKMYKYKKRMWRSYMSKKKYYRGSCGAA
            ****       **********     .  *.* ** **  ****. ******** .  :.

43          KWYRATWYRAKAKCCCTATCGCRAKRGTTKGYKKTTKKKMGTARGACCKRRKYRYKRKKA
51          GTCGGCWYRTTGKMYYYWYMRYRRKRAAATRCKKACKKKMKYRRATTTTRAKYRYKAKKG
                . ***:..*         * **.::.  **: ****   *.:  .* ***** **.

43          TYRYYKKKSMYYKRYGYYRYWWWYKGCWRAAKRWRYKKKKAAYCRYRMMYRKWYRCTTYK
51          GYRYCKKKSMYYKRYRCYAYWWWYKKYWGRGKRWRYKKKKTTTMRYRMMYGGWYRMCGYT
             *** **********  * ******  *  .*********::  ******  ***   *.

43          RRYRRYYYYYKKKWARYAYKYKYYYYWATSYWYMRRWYMKSYRSKWRRYWWGTRMYRMYW
51          AACRGTTCTCTKKAGGCRYKYKYYYCTTCSYWYMRAATMKSYRSTWRRYWTAWGCTGATA
               *      .** .   *******  : ******   ******.***** .

43          RKKRYKMYYRYYMKTGYRRAYMWKCGCMYWWGGCWYMKTGARYYYRMYYCKCAWRTTTMA
51          RKTRYKMYYRYYMGCTCRRWYMWKYTYMYWWKRYTCCKCAMRCYTRCYYSKMRWRCYCAT
            **.**********    ** ****   ****      * . * * * **.*  **    :

43          RGRCRWWWTGYKMKKWRRWYMKWWKMKKCRMTTRCGTKKKCTAYKYTYYCAKCKKWYRYY
51          AAGTRAWWYKYKMKKTRRWYMKWWKMKGTRMCWAYRYKKKYYRYGYCCCYRKAKKWYRYY
             .  * **  ***** ***********  **      ***   * *     *.*******

43          KRYRTMGCKTCTGGAWWACAYAKMRRKTRYRYMRKYRKWKGCYKKKMATTARGAGARTTG
51          KRYRKMATKCAAARMWARTGYGGAGATKRYRYMRKYGGAKKMCKKTMMYGWGKGKRRYYK
            ****.*. * .:.  *   .*.    ..********   *   **.*      .  *

43          KRGGCWKCCCCAGKMKCGTCRGACAMKKKAGARRAYTKYCWGYMARKRWRKKKRWMMYRS
51          KRAATWKAYMYRAKMKTAAYGAGTGMKKKGCGRRMYKKYTTRCAGGKGWRKKKRWMMCRC
            **.. **.    .*** .:  .. .****. .** *.**     . * ********* *.

43          TRRRMRRRGYCTTRCGGGAGTYYRWKGWKMMMYTCGGKRRRYYWYWCAMAKRRRYYYRWY
51          CGRRMGRAKYYCGAYRKTMAWYYATKRTKMMMYKTAAKRRRYYWYWYTCTKRRRYYYRWY
              *** *  *         . **  *  *****. ..********* : :**********

43          TTCAYYYYATYKMKRMRRYRMYCGAAKGMYGTTRRKRCCKYRRWKMKRRRWRMKYGKYYK
51          KWYMYYYYRAYKMKGMRRYRMYYKRRKKMYRAWRRKRTTKYRRWKMKRRRWRMGYKKYYK
            .   **** :**** *******    * ** : ****  ************** * ****

43          TKRTGGGAKWWKWRTKAMRYWKKTCTYYMGTGMRMSMKKRTWKRMKMRGTAWMYYYYMRY
51          AKRKRRKWKWWKWRKTMMRYWKKYTCTYAKWRMGCSMKKRAWKRMKMGAYRWMYYYYMGY
            :**.    ******.. ******    *    *  *****:****** .  ******* *

43          MYYYKRYYMRKYKMRKKYRYSKKTKGYKGYRYMMYRYMYTARRRMYYYWAACWGYCTKKY
51          MYYYKRYYMRKYKMRKKYRYSKKCKKYKRYRYMMYRYMYGCRRRCYYYWMRYWATTCKKY
            *********************** * ** ********** .*** ****   *.   ***
```

```
43                  WMRKMWRYRGY
51                  WMRKMWRYRSY
                    *********.*
```

The pairwise alignments of two accessions 44 and 45

```
44                  KMYKSYYYYRRRYMYTCMMYWWRRWRCYWYYMRRGCKRYSRWCKARSWWGAYRRWYGMRY
45                  KMYKSTCYYRRAYCYGAMMYWWRRWRYYTCYMRRASKRYSAWYKRRSWWRRYRRWYRMRT
                    *****  **** * * .********* *  ****..**** * * ****  ***** **

44                  RKCKKCGGYMGAMYYWMYYWCAKTRYMGCYGKRMKMKCYRSRKYCCKKMMKYMKKWYYKT
45                  RTYKKGRKYCRMMYYWMYYAGMGCGYASMYAKRMKAKTYRGRKYYYKKCAKYMGKWCYKG
                    *. **   *   *******      * . *.**** * **.***  **  *** ** **

44                  TCAARKKYRKYRYRSYRYRYRCGGCRYYARRCMRGACCYYRWYYMRRYCYYAAGYWSYYR
45                  CYGGRKKYGGTACASCACRYRYKRMRCYMRRYCAACTTTTAWCCMARYSYYWWKYWSYYR
                      ..****      *   ***    * * **   ..     *  * **.**   ******

44                  CSYKWRKYWYYRKTATAAWRCYKRTKCKWYYYWWYSMWTGCGGMKMRKKYYMRYYRMWKR
45                  YGYTWRKYWYYRTAWCGGTRAYKGKGYKWYCCATYSMWCATAAMKMAKKYYMRYTAMWKR
                     .*.********.:  .. *.** .  ***    **** . ..*** *******  ****

44                  KCYMGSKMYRRRKWYMRGCARYWRMKTRMMYMWRRKAAYTTRYRWKYRWKWGTCRYKYRK
45                  KYYMKCKMYRRRKWYMRRTRRYWRAKWRMMYMWAGGWWCYCGCRWKYRWKWAWMATGCAK
                    * ** .***********   **** * ******          ********.       *

44                  KYWRYRCMGGMRKMKTWMMYWRRKRRKRYRRWWYYYYKWCYRKWYRRYRYYYYAATAKYR
45                  KYWRYGGMKRMRKMKYWMMYWRRKRRKRYARWWYYYYKWTYRKWYRRYRYYYYTRCCKYR
                    *****  *  ***** ************* ********* *************:  .***

44                  YRKYRYSRRTTKAKRYYGYYCTYGWAYYRYYYRKRRCRMCGTWMRWYRYWKWYRMRYWWR
45                  YRKYRTSRRCYTRKRYYTYYAATRWRCYRCYYRKRATRCMKAWMRACRYWKWYRMRYWWR
                    ***** ***  . **** **.:  *  ** *****  *   :***  *************

44                  TYYYYKRYSAWSWYKYYRRWKSYMMMYWKRSATAYYWYRRYKMWKKKSRYKTTCKRWRYR
45                  CTYYYKGCCMWSWYKCCAAWKSTAMMYWKRSWYGCTTYRGYKMWKKGSRYKYYYKRWRYR
                      ****  . *****    ***  *******  .   ** ****** ****   ******

44                  KMMYYYRTTAGGTGYKYYKWYAKRGTAYGKYKKKKYRYKRKYCKKYRRRRMMRTGGRRKW
45                  KMMYYTAAGCSRYKYKYYKAYCTATATCAKYKKKKYRYKRKYTKKYRRRRAMRCAARRKW
                    *****  : ..   ***** *..  :: .************* ******* **  ..****

44                  CYYMYKYAKCYKSYWYKMMRGYYYRSSWWKCCYCKTKYMAMRYGYKYYKRKKYTWGCYKK
45                  GTCAYKTRKYYKSYWYKMMAKYYYRSSWWKYMTYKGKYMRCGYRCKYCKRKKYWTKYYKK
                        **  * *********  *********    * ***   *  ** *****    ***

44                  SRYCRYRMKGSRGRMSKKCTRRKKRRKKYSRKRGGRKWWYWMRATSRYKAGCTTGGGWAC
45                  SRYARCAMGKSRRRMSKKYKGRKKRRKKYSRGRKRGKWWYTMAMYCRYKCRACCRRKWGM
                    ***.*  *  ** *****  . ********** *   **** *   .***. .     *.

44                  GWWKMYWTRWRRSYCGKRWRKTMWRKTKGRYARRKRKYCGCGSRRMMYMWSYKGKSRRWM
45                  RWWKMYWCRTRRSYTTKRWRKCMWRKYKTGTWGGKGKYAATTGGRMMYMWSYKKKSRRWM
                     ****** * ****  ***** **** *      * **..  . ********* ******

44                  MKKMWWYYKKGRCCCARSKGRRRCTKTCWKKCCCAYATMKSYKMWCKRMRKKKYYRWSKK
45                  MKKMWWYYKKRGYYYGRSKAARGYGKKAWKKSMYGYMYMKSYKMWAKRMRKKKYYRASKK
                    **********     .***. *   *..***.  .*  *******.********** ***
```

```
44          YYRGRYYWKMKAKKTKAWSACGTKSYMYKYWSKRMYYKCAMYKTTYRYKRKYKKMCWYKA
45          YYRRRYYWKMKRKKWGGASMGKWKSTATKYWSKRMYYKMCMYKKWTRYKRKYKKMYTCKM
            *** ******* **  . *    **   ********** .***.  *********   *

44          KSYTYKRGATCKACWYAYKWCTRATKMRRKKYGWCYKACTTGCYYWKGATKRRTSCCTTS
45          KSYYYGARWGAKTGWYRYKATCGTYTAGRKKYKAYYKRTYYKMCCAKAGKKRRCGTTYAG
            *** *     .*: ** **    : .  ****   **         *...*** .   :.

44          CYGWKKRWMMMRRRKRRYATTGMYTMCKRRKTWKRYMMRKKYRMSRYATMCKGATTCCYR
45          SYRAKKRWMMMGRRKAATRYYRAYYMYKRGKWAKRYAMAKKYRMCRYGWCMKATCCYYCR
            .*  ******* ***        * * ** *  *** * *****.**.   *.:     *

44          YKKWYYMAGRGYKGRCYKGGYRRGYACKSKYRRWKKKYYYYRYRGACWYRRCYYKKRKRW
45          YTTWYYCGAAAYKKGTTKRKYRRAYGYKSGYRRWKKKYYYYRTRATAWYRGTTYKKRKRW
            *..*** .. .**    *  ***.*. ** ************ *.:.***   *******

44          CACYAKRKKAYTRTTYWYCRMYKYKKKYRRYYYWMKKYYKYYYSYYKKGKKCKKGGTYGY
45          YGTCCKRKKGYAGYYYWYYRCYTYKTKYGRYCTAMKKYYKYYYSCTKGKKKYKGKKCCKC
             .  .****.*:   *** * *.**.** **   **********  *  ** *

44          ARTRYCWTTCTYYKRKMAKWYYWCYYRYYKRTCGKWYCAAARAATCGTTRYYGCAATKKY
45          RACRYATKGTWYYKRKAWTWYYWACCATTKRCTAKWYTTGCRMMYMRAARYYAATTCKKY
               **. .   *****  .****.     **  .*** :..*     ::***..:: ***

44          KMAAACCYTTYYRMRWYYSTKYWKGTARGWMYWRRTYWYKRWKCGKKKSRWAYKWWYCRM
45          KMRRRYYYACYCAMRATYSWKYWKTCGRKWMYTARYTWYKGTKMAKKKSRWCYKWWYYRC
            **     *: *  **  ** ****  .* ***  *  ***  * .******.***** *

44          MYRRTTTYRCYKYKTCGKKKKYYKRYKKCWMYRYRKYGWKMYYRKTRYYSGYCYYMSRKY
45          ACGRACAYRYYKYKAAATKKKYTKRYKKTTAYATAKYRWKMYYRKARYYGKCYTYMSRKY
               *: :** ****:...**** *****   *   ** *******:***.    ******

44          MYTCWSRYCTRKGGYYCCKAGSSATARCGATKYKMYRWWYCYMSGARYYYYWTKKYTCAT
45          ATYYWSRYMWRKRRCTYYGRSCSRWRRYSWWGYKMCRWWCMYMCRGGYYYYWGKKYCMRK
                ****  **        ..*   * .   *** ***  **. . ***** ***   .

44          TKRWYATCTRGGGCAAGCRWGKRKYAWYRMMCCYYGRWRRGGCYYTARKYKWRCACKSGK
45          YKRWYMYMYRRRRYCCTAGTTKRKCMWYRMMTTCCKAWRGARMYCCGRKYTWRYGTKSKK
             ****    *    .. .   ***  *****      ** .  *  .***.** . ** *

44          MYKRYYYMYRRKKKRYWAMKTYACAYYKKRKYKMKRRAGAMMRGAKCGWTSYWRGTRRMR
45          MYKRYYYMYGAKKKRYWWMKCCGARYYKGRKYKMKRGTAGMMRKRKMRWWSYWRACRRAR
            *********  ****** **  .. *** ******* :..***  *  * ****. ** *

44          RMKKMRKRCAKMMYKMAGTGKSYGAYKRKTTKKKMCRTKRCGKMCTSYKKKKCMKYCCWK
45          RCKKMRKRYRKMMYKMTTATKSYAGYKRKAAKKKATGATGTKKMYYGTTKKKTMKYYYWK
            * ******  ******: : ***..****::***   :.   **  . .*** ***  **

44          YRCGWKRKKWTRWYRWMYRTYWKKKKRRRTRMRGSTAAKKGYKTWYWTYMKRWGTKKYRR
45          YRTAWKRTKWKRWYRTCYRWYWKGKKRRAAAMRKSYWMTKAYKYTYAWYCKGAAAKKYRR
            ** .***.**.****  ** *** **** : ** *   .*.**  *  * *  .:*****
```

```
44                RKMMYMMYWGCYYKARKKKCGTAWTKYCYKKCAAGRKRTRGYGCTKYKRYKKGYCRYYRW
45                RKMMYMMYWKAYYKRRKKKYRATWKKYYTKTYRMRRKRKRTYAAKKYKGYKKRYYGTCAT
                  ********* .*** ****  ::*.**  *.    ***.* *...*** *** *

44                RGRKTGKKKKKTTYWYKYYKWKMYYKWRRTTKYYKYWMKKKRRRRYMYMMWKAGKYWWSK
45                RAGKYKKKKKKKKYWYKYYKWKATTKWRRWKKYYKYTMKKKRGRRYMYMMWKWRKYWWSK
                  *. *  *****..*********   **** .***** ***** *********  ******

44                KGKKRKYKYKKSGRRYYYRRRKKYCRKRKKYAMRKGTYRYRRRYKKSWMAKRKGARRYWC
45                KKKKRKYKYKKSKRRYYYRRRKKYMRKRKKYGARKAACGYRRRCKGSWMTKRKAGGGCAY
                  * ********** *********** ******. **.:  **** * ***:***..

44                CGAKMWMRYKTGWCMGWKYYYARRAMSYRYYRACKGYKGAKWYYWKYYTTMWWWKRKGTA
45                MRRKAWCAYKWKWYCRWKYYYRRRTCCYRYYGRYKSYKATKWYTAKYYAKCTTAKRKRKM
                     * *  **  *   ***** **: .****   *.**.:***  ***:.    *** .

44                KCGKRMKYRYMYYYRKRRKRMKRTCAKTSGRRWTCKRYRYTMTTCCCYKRKYGKMRWKYC
45                GYKKRMKTRYMYYYRKRRKRMKRKSRKKSAGGTCTKRYRYKMCKYSYYKRKYTKMGWTTM
                     **** **************..  *.*.     *****.* . . ***** ** *.

44                MKRWRRYTRRWKRYYMACGMRKMKWGKYYYRYKSMYKWRWMGYWRAKCCCKKRRRSYYYW
45                CTGTRGCYAGWKRYYMRSKMRKMGWRKYYYRYKSMYKWRTMRCWRWGTSMKKRRRSYYYA
                   .  *     ****** .****  * ************* *  **   .*********

44                MYRMMRYRYMKCMRACTRKKKTKMRYYWRYYGKKRTRRCGYRWMGSKTMMWCCTKWRYRK
45                CYRMMRYGTMKTAGMAGRGKKKKMRCTAATTRKKGWGGMRYRWMACKWMMAYYKKWRYRK
                   ******  **    . * **.***       **      ****..* **   .******

44                KKRRKKYYYYKRKKWCKTGKKTGYYSGYRWKKYKCTTGTTKWRRGGKRGGRMYYGKCGCC
45                KKRRKKYYYYKRKKWYKWKTKYKYYSTTRWKGCGMAKKWYKWAGRKGRAARMCYRKMRTT
                  *************** *  .*  ***  ***    :.   **     *..** * *

44                CWTAKRYRYMKYYSKSKKWYACTAGKKKKYSYWYRRKCGWYYMYRRRMRAMYMYKWTWCY
45                TTKMKRYRYMKYYSKSKKWYWYYTKKKKKYSYWYRRKYRWYYMYRRRMRCMYMYKAWAYC
                    . ****************   : ************  **********.*****

44                RYMWYRYTMYKWRRRKCWAYKRWKYTMWYKMATCTTAGMMRWWGKCCCKMYYGTYGWKYK
45                ACMWYRYCMCKWRRGKMWMYKRWKTCATYKMCAGAATRCCRWWTGMMGKCYYKYYKWKYK
                    ***** * **** * * *****    ***.: :::   ***     * **  * ****

44                RYYGYMGTKRRYKYKAKCCRYYRKRWMYKRYYRKKGATKYMCKAGGAMRYMRKYWGKCRA
45                RYYKYMKYGARYKYKMGYYGYYRKRWMYKGYYRKKKRYKYMSKGTTWMRYMRKTTATAAC
                  *** **    *****     ********* *****   ***.*.   ******  ... .

44                KTAKYGYKWYRKYGKMKRKYYKCGCAKAMKRYCWYRTATSYKWYCKRKCYYWYRGYRKKY
45                KYRKTRCKWYRKCKKMKRTCCKATTTKWMKRYAWYRYWYGCKWYYKGKYCCACGRYRKKY
                  *  *   *****  ****.  *.  :* ****.***   . *** * *       *****

44                RCYAYMMRRKKKGRMYYKWMYYKKYMMMKRRKKYKYRYKWGMGWRKKKYYAKCRYRKMRR
45                RYYWYMMRRKKKKRMYYKWMYYKKYMMMKRAKKYGYRYKWAMCWGKKKYYRKARYRKMAG
                  * * ******** ***************** *** *****.* * ***** *.*****

44                GWKRMYYKRRKRSKYCCAMKRYRYMCKKCAGGATKWRWWMCARYRAYRYGKRSMKRTYCA
```

```
45              RWKRMYYKRRKRSKYYYRMKRYRTAATKTGARRKKAGTTMTGRYRGCACATRSMKRCYTG
                 **************   *****  ..* ..  .*    * .***.   ..***** * .

44              GRRMGTKKCKYKWKRYGCWMRRMTTGRAYYYKSAWKYRKTGYRGAGRKAATGKRRMKRKM
45              AARCKYGGYKYKWGRYAMWMRRMAGTRGYYYKSRWTYGKKKYRKRKGKWWYKKRRMKRKM
                . *      **** **. *****:  *.***** *.* *. **    *    ********

44              MMKYMKGTRYRYSKRTTTWKWAYRKRRMWRWTTAWMMKKWMKYKWYYYWCCTYYRTRYTW
45              MMKYCTKYRYGTSKAAGCWGTMYRKRRMWRWYWWWMMKKWMKYKWYTTWMYYYYRKRCAW
                **** .  **  ** :  *   *********   ************  *   ***.*  :*

44              YRKRAYRSRRYRKGKRTYCGCAMGACRMYYWYMAKYKRRRATKYRYSKWYYWYRKKKAGR
45              YRKRRYRSRRYRKKGRKYMRTTAAGARMYYWYMWKYKRRRRWKCGYSKWTCWYRKKKRRR
                **** ********  *.*   : ...******* ******  *  ****  ******  *

44              RTKCKTCRARYYSRWRYYYRYRKTTTKKTYCGYWKCCTKCCAKWCMYYMCWTYRWKGMRW
45              RKKMGCMRWRYYSRWRYYYRYRKCAAKKKYYRYWKMSWKGTTKWYMYYAYACYRWKKMRA
                *.*    * *************  ::**.*  *** . *  :** ***    **** **

44              YCTWKKRYYCGGGYYRYWRCWKYYKYTRTGCYRRGMKAKYYMYRKYRMYKKCMYCRKKKM
45              TTAAKKRYYAAAAYYRYWRMAKYYKYCGAATYAAKMTMKYTMCAKTRCYKKMMYARKKKA
                  : *****....******  *****  :. *   *. ** *  * * *** **.****

44              TWRAKWRKWCYWYRGRCCRRAMKMYKSYRKYKSWCRYYCARMWWAMKRWMKTTTKCKYTA
45              YTRGKAAKWYYWYRRAMTRRCMKMYKSYRKYKSWYRYYTGRMWWMAKRWMKCAAGAKYYR
                  *.*  ** ****    **.*************  *** .****  *****  ::  .**

44              YKRGWRRYYWYYCATGAWRCAAYGRCTRRWRRYCCTRRMRMRYGCTAYRRCYKYRSKAMY
45              TKRKWRRYTATTTATRWRYGCCCAMGRRTARTYAGRGCGCRYCYYRYRAMYKYRCGGCT
                 ** ****     ::  **  ..     **   *  . *    **    **   ****. .

44              YWCCGKKYRKYATTRWWAMWMCKKYAAKKGKKAYMWCTRYRAWYYSTATTSCGWYYKYKA
45              CAATAKKYRKYTAARWWRCTMYKKCGCKKAGTRYMWTGRYRMWCTGWWCGSTTTYYKYGR
                  . .******:::***   * **  ..**. . ***  *** *  .    *   ****

44              ARKRMATTCYKGRGMCRGKTKKCTTYWYTCWAAYMYKRYRKKKRYYTARYWTCCCTTYRM
45              GRKRMMWWYYKTGAMMRKKCKTTKYCWCYYWWRTMYKRYRKKKRYYYRRTAATAAAWCAC
                .****    **  .* * *. .  *   *   ************  *  : ..:

44              TMRKMKGRGTYGYRKWGRKKGRKMTTGMMGTYRRYMMYRKWAGGCKKGTWWGATKWKRAA
45              AMGTMKRGRWYRYRKARRKKKRKCGATMMRYYRRTAMYRKWRCAMKKTATTAGCKTGRTC
                :* .**    * ***  *** **  : **  ***  *****  . ** :  .. *  *:.

44              ATCKMRSWYYAAGCRRRKRRYMRMYWTRGAKMGCGCTRACYCYWKGKKTWYRAATKRRMK
45              RGAKMRSWTTMRATGRRGRRYCGMTAGARRKMKYRYWRWTCTYAKKKTAWYRGMCKRRMK
                  .*****    .  ** ***  *      **     *    * * *.:***.  *****

44              ARKRCRYMRYGKKYYMRWMYAKWYYKYTGTCGRCYYTRYMKRYRRKACYWGYAGMSWTYG
45              WRKRSRTMRYRKKYYMRTAYWGWYYKYYKCTARYYYYRYMKRYRRKMYTWRYMAMSWCTR
                 ***.* *** ******  *  *****    .* ** *********   * * .***

44              MKYYKARYRMMTTKRRRYKTACACTRYRKKKKSRRYYYKKKTRKKKRYTKKRCTMWMCKA
45              MTYTTRGYRMMAYTRRRYKWWYWTYRYRKKKKGGGYYYKKKWRKKKRYKKKGYYMWMYKM
```

```
        *.* .  ****: .*****      *******.  ****** ******.**   *** *

44      SRAYRKMYKSRRRYRCKWGCGCYRYKRAGKGTGYYKRMMKWAAKSMWWYKKRKGGTGYKK
45      GGRYRKMYGSRAATGYKWAMKYYRYKRTKKACATCTRMMKTCCKSMWWYKKGKKSYKYKK
        .  ***** **     **.   *****: *. .  .**** ..******** * .  ***

44      GCACTKAARRAKRCCRWRKYRCCARGRTTCCTCAMRWMTGRTTAYYKRKYRMYRMKYMMG
45      KMMYWKWTRRMKRMSRWRKYRYYRRKRAAGAATWMRWMYRRWWRYYKRKYRMYRAKYMMK
             * :** ** .******   * *:: .:  ****  *   ********** ****

44      GKTKGTCATCAKKCKYKKYYRYMRRWCWMRRRCATCACGCYRKYCWTKMTRKCCCCWYYK
45      KKCKKWTMKSRKKMKYTKYYRYCRRWMACAGATWWTTTTYTGKYTAGKAGRGTTATWYYK
         * *    .. ** **.***** ***          :     **    *  *   . ****

44      KRYKMRWKACCGGCARGYYMWMKTGYAAGGKRGRCARWRRRGATYRRMRKRKWKSKYGWY
45      KRYKMRAKRYTKTMGGATYMWMKWSTTGKKKRTGTGAWRRGSGAYRRMRKRKAKSKYKWY
        ****** *      . . ***** . :.  **   . *** ..:******** **** **

44      YKKMYMSKCTSRKKKWTTTRAKRRRYWYRYYGTYKYMRRYRYTCYRCKAYKKMGAYAYCK
45      YKKMYMSKTKSRKKGWYWYRWTRRRYWYRYYKYYKYARRYRYYMYGTGRYGKMRRYRCMK
        ******** .**** *   * .*********  *** *****  *    * **  *   *

44      KGKATAAWKTAGAGGCYWTTAWKYYYYMYCCGWKKSMMATWSYRAMYYYRRKKKYCKKYY
45      KKKTCTTWKCTAGRRMYAAACWKTYYYMCTTATKKSMMRWWGCGMMYYYRRKKKCMKKCC
        * *: ::** :..   * ::.** ****  .  *****  *.   *********  **

44      RCAGTRGAGYYKKAMKKKCGKKAWYYYGTAAAYYRKYGTRKYTTRSKWRKYRAATGKKKK
45      GTTRCRRWSYYKKRMKKKTRKKRWYYTKWTCCCYRKYKGRKYWGRSKWRKYARGCRKKKK
          :  *  .**** ****  ** ***   :.. ****  ***  *******  .  ****

44      KYRYGKYYRKKKKWKARKTGRKKYYSYWYSKKRKYCRYKMMRWYKCYTWKKCKGYRKGWW
45      KYRYCGTTRGKKKWKMRKYRRKKYYSYWYSKKRKYGRYKMMRAYKAYKWKKMKKYRGKTW
        ****    * ***** **  *************** ****** **.*.*** * **   *

44      GYRKGKYRRKKRRKTKYAARWKRWCGTKKKKRMTRYKYYSKGCATYGRYKKYKMYYKKSR
45      KTAKAKYRRKKRRKYKCMRRWKRWMTCKKKKRMCGCKYYSKTAGAYAGYKKTKMYYKKSR
           *.********* *   *****   ******   ***** ..:*. *** ********

44      KMTKRYSYSKYSYKKKYWRRRYGKMMKKCAYAYSKGSKMCGKKRKKKYKGTYYYAYRTAT
45      KMKKRYSYSKYSYKGKYTRRRYTKMMKKYRYRYSGKSKMYCKKRKKKTKKACCCTTGCMW
        **.*********** ** **** *****  * **  ***  ****** * :   :

44      CYKYKWKYYYYMRCTMRYSKGACGKRYKSAKYYWCAGCTMWMYGKGGGTWKYKCGARWTT
45      MYKTKWKYYYYMRYWMRYSKRGTTKRYKSRTYYWYMRYYMWCYKKRRRYWKYKYSGRWGG
         ** *********  ***** .  ***** .***     ** * *    **** ..**

44      CTGTTTYCKRYKKRKWMTWRRYWMYRCKTGGYKYGMRYYKRGYKYWMWYYAAKATRWSGM
45      TWRWWKYYKRYKKRKWMKWGGCWMYRMKGTKYKYSMRYYKRRCKYWMWYYGMKRCATGAA
             .* *********.*   **** *   ***.******  *******. *    ..

44      GWKKWRRRAAC
45      AWKKTRRGGWT
        .*** ** .
```

The pairwise alignments of two accessions 44 and 46

```
44          RYGAYGATGYWRSWYWKSYYMYRWKSMRRYKACGWYRMSARKYYYKMMKYKMCTGGMKMY
46          RYTRCRRWSYARSWYWKSTYMYGATGMRRYKGAATYRAGGRKYYYKMMKYKMMWRSMKMY
            **      .* ******* ***  ..*****... ** ..************   .****

44          SMMYWRRRRWKWAMMWRYYYYYGYRRWWKKYYYYTCYRAKCAKAYRRRKTYYRWRYMMWW
46          SMMYWRRRRWKWRMMWRYYYYYRYRRWWKGYYYYWTYRWKYWGRTRARKWYYRWRYMMWW
            ************ ********* ****** ****  ** *     * ** **********

44          WYRRCRCYYMRRGATRCMYMRMWYGYCCCMYYTYMRKMYRRWYYRYMWKTYRYKRKKYRS
46          WYRRGATYYMRRAGCRYACAAAAYKCTTTMYYAYMRKMYRRWYYRYMWKYYRYTRKKYRS
            ****   *****.. *       *     ***:**************** ***.******

44          AKRCKWWMRYWKKGKKWYRYRYAACMYYYYWKRSYRYYWWWRTKKRYRRTSRYRKYYWAR
46          RKRAKWWMRYWKKAKKWYRYRYWRYMYYYTAGRSYRCCATAAWKKRYRRASRYGKYYWWR
             **.*********.********   ****   ****       ******:*** **** *

44          GKYYMKCRYWWRRWYYTATKRYYRWYYKRYRCMGSWKYKGMMTWYWARRYRRRKWYGRKY
46          RKYYMKYRYWWRRWYYWWWKRYYRWYYKRTGYCRSWGYGSAAYTCWRRRYRRRKWYTRTY
             ***** *********   **********     ** * .     * ********* *.*

44          MWYMTWTWKWKRMRTYYWCKTYYGYKWWSYWWRWYRKYMYYWKWYYYYYKWGGYKYRKCY
46          MWYMYWGATATRMRCYCWTTKYYKYKWWSYWWRWYRKYMYYWKAYYYYYTWRRTKYRKMY
            **** *  . .*** * *  ..** ******************* *****.*   **** *

44          TAKKKSMMYSRRRRYSRMKYCAAWYSYKYKRRTACRRYKRYCWRSSWMYYKKYRYRWYMY
46          WWTKKSMMYSRRRRYSRMKYAGGWYSYGYKARAGAGRTTRTYWRSSWCYYKTYGTRWYMY
              .*****************...**** ** *:.. * .*  ***** ***.*  *****

44          YKYKGCRKRYKRRYRYMYWKWRRRYMRWMTKTTYKKKMWYKRKRMRYMMWGKKKTKKAKW
46          YKYKRARKRTTARYRYMYWGTGRGYMRWMCGACYKKKAWYKRKGCGYMMAKKKKKKTRGT
            **** .*** . *******   * *****  :  **** *****   ***  ***.*.

44          KRYYRYGTYKYMRWGCGKMRRRKTTYRRKGATASRTTGARRYKKRYKAGYAMRKYMKRYW
46          TATYRYRCYKYMRTCAAKCAGATWYYRRKACCWSRYGRWRGYKGRYKTCCCMRKYMKGYW
            .  ***  *****  ..*    .  ****..  **    * ** ***:  .****** **

44          MKYMTMYRAWTYYKRACKKWRCRRYRTRRRKKSYKKMYWKRKKMWWYCYGSWKRKYKTKT
46          MTCCGMYRRTKYYTRRMKKWRYRRYRKGGRKKSYKKMYWKRKKMWWYTCASWKRKCKYKA
            *.   ***  .**.*  **** ****.  ******************  .***** * *:

44          TKGMRCKKMWGTYATWRYSTYKKKRRARCTYWYYCKRGYMACKCMGRRGTKKCGKGRGRT
46          KGKMRMKKMTKCCCCWRYSCCKKKAGGRTYYWYYMKRAYCTYTYARGRTAKKTKKKRKRC
            .  ** ***    . ****  ***  .*  **** **.* : .    * :**  * * *

44          WKARMMKKRKRRMKRRWATTGMYMRTWCGTTKTKWMKRYKATGTCCCTAYYYYCSKKKRR
46          WKGRMCGGRKRRMKRRWRYYRMYMRCTTKWYKKKWMGRYKRCRYSTYYGYYYYSCKKKRR
            **.**   *********    ****      *.*** ***    .   .****..*****

44          RYYWATKYSYWTMMKKCTTKGTGYGGAGWKYKYSYWCRATTYRRKKYRRRARRWWYMYRY
46          RYYWRWKYSYTYMMKKYKKKKKRYARTTWKYKYSYWMATAWYRRTKYRRRCRRAACMYRY
            ****  ****  **** ..* . *. : ********  :: ***.*****.**   ****
```

```
44    KMWYRYCARRRTMTGYGCTTTTKTATGRCRGCTRWYRRYWAYTGCYKRRYKGTTTKYKYY
46    KMATGYACRRGCMGTCATWCCGKCCCTAARKYWRWYRRYARYCAAYKRRYKTCWKKYKYY
      **   *..**  *   .     * .   .*   ******  * ..******   .*****

44    KRRCCGTSTATWRGGWGKWKGCYYWYTTCTRWYYMGTKRKKKRYRYKRWRCYWMMKYYRT
46    KRRMTACSATGARRATKKWKKAYYTTACMCRWCYMKYKRKKKRYRYKRWRYYTMMKYTAG
      ***  . *::  * .  *** .**  :   ** **  ************  * ****

44    AGYKKYYRKMWCGAKKMKYRYKRKTYWYKKYRRRRMMRRRRWTMKKYAYGKKSYWYKMYR
46    CKYKKYYRTMAMRMKKMKYRYKRKKYWYKKYRRRRAAARRAWKMKKTRYKKGSYWYKMYA
. ******.*    **********.**********   ** *.***  * * *******

44    YMYYYYRYMKYSKRRKAAWKWYRKMKMKYYRKKKRYSYYKRYRKKMRRKSRKSKRAKRRK
46    YMYYYYRYMKYSGARKRRAKWYRKMKCKYYRKKGRYSYYKRYRKKMRRKSRKSKGMKRRK
      ************  **   ******* ****** ********************  ****

44    TMRYTAGACWWYWAMRKRYAKKRYSGTTGRGYYKTATKYACARMKRYKKYRWRWYRYCGT
46    KMGCACTRYWWYTRMRKRYMKKRYSRWARRRYTKYRCTTRSRRATGCKKYRWRWYRYMRY
      .*  :.   ***  ***** *****  : * * *   .  . * .  **********

44    GCCRSGKKGWYMCCYRYRTTTMMTGRYYRAKKGTTTRWYTKYRMKTTYTYWAWKYMWMYK
46    AATAGAKKKWTCYYCACGWKCCCKRRYYRRKKAWAARWYKKYRMTCWTCYTCTKTCTMCK
      ..  ..** *         .   . **** **. ::***.****.    * . *   * *

44    YRCCAGCCYYRKGCWRYKTAYATCCAKKKYYMYAMYYCTTMGMKYRRGTYSCGTGYRYKW
46    YGTYGATYYYRGRYWRYKCTCWCAATKKTCYMYWCCYTYCCRMKYRGTGYSTTCATACGW
      *   ..  ***   **** :   ..:**. ***   *     ****   **   .    *

44    AYMARTTGCWRRGKWAGGKCKRGKMRAGGWYYKMYKRYTKGKRYYCTGARYRYGRYAKRR
46    RYMRACCAAWRRTKWWKKKTKRATMRGAAWYTKMYKRCYKRKRYYACAGAYRYRGCRKRR
       **    ..*** **   * **..**...*******   * ****. .. ***    ***

44    RWSRKRKMYRYTRKKKATTWKYMYTTGMMYKKRMKAYRWTATWRKTTAYYYYWTGCCGRK
46    RWSRTRKMCRYCRKGKCGAWKYMCGATMMYKKAMKWYRWWRCWRKCAGCYYYWCRYYRAG
      ****.*** ** ** *. :****  : ***** ** ***   *** :. ****

44    YTWGARTRKYSWWYYYYTRGYTRRGKYSRYYMRMRRKYMKATTKMMRRMKGCMRKTKWKK
46    CAWTRRWRKTSWWYYYYKRTYGGGKKYSRYYMRMRRKYMTRYWKMMRRMKKAMRKKKWKK
       :*  * ** *******.* *    **************.   ******* .***.****

44    KWRWRTGCYKRRWKRRKRYYRYMRKATKYRRYYKRRYRKYKRYCCKYKTGRYKYWWTKKC
46    KWRWRATGCKAGWTRRKRYYRYMRGRYKYRRYYKRRYRKTKRYTYGCKKSRYKYWWKKKT
      *****:   *  *.**********   ************ ***    *..******.**

44    GCKRWWTTAWWYYYCCCMYWKMMGKMRGCSWSYRMMWYTKCTYSCWTRKKRCACAWYGAC
46    KGGGWAWACAAYYYATAMYWGMMSKMRTGSWGCGMMWYGKYYYGYTGRKKGMMTWTCRMY
          *  :.  ***. .*** **.***  **.  **** *  *.   ***

44    WYRTWRYKTMKGGYMKYKKRTAYKMYRYTGKRRGYTKWMRKRGSGKWYRMWKRRKCWTAK
46    WYRKWRYGWAKRRYMKYKKRAGYKMYRYWTKRRSCWKWMRKRRGKKWYRMWKRRGTACCK
      ***.***   *  *******:.******  ***.  ****** .  *********    .*
```

```
44              YYRTMRYATTAKKSYRRWWMKGKKRRRWKYYYAGKRRYCAGCMKRYRRKTMYYWGRRWMW
46              CYRYMRYCCCCKKSCRRATAGKKTRRRWKCCTRSKRRYAGCAMKRYRRKGCTYWKGAWMW
                 ** ***.  .*** **     *.*****    .****.. .*******   **   ***

44              YMGYGGGARKRYRYKRYRWMRSRRKKAYGKRYKRKKKSRRKKKYKCAKTWATAKYKKYKR
46              YMKCRSRRGKRYGYKRCGWMRSRRKKWYKKRYKRKKKGGRKKKCKYRKWWTAGKYKKCGR
                **   .   *** ***  ******** * ********. **** *  * *::.****  *

44              YKGKRMWMSTYKYRMYAKGARRYRWATCYRYRRRKKMKTTAKMKGATGGASSKYCMMKRK
46              YKRKRMWMSCYKYRMYRGRRRRYRWWYYYRYRRRKTMKYKMGMKAGACRGSSKYTCAKRK
                ** ****** ******    *****   *******.** .  **..:  .****   ***

44              SYYMYRKYMRYKKKYRGWWRRKRCTAMKKAYCYSGYKWAWTCAGMRRKWYKRWRAGRMCR
46              CTYMYRKYMRYKKKYARAWRRKRTGGMKKCCYYSKYTATTCMRCARRGTCGRARWRRMMR
                . *************   *****  .***.  ** *. :      **    * *  ** *

44              YKATCRRYYKYWKYRRWKYMYYYYRKKMRYSKKKGMGRKKYYWMMCTMWYGGGTWKYKYR
46              YKMWMRAYYKYWKYRAWKYMCCCTRKKMRYSKKKKCAGKKYYWMMYYMWYRRRYWKYGCG
                **   * ******** ****    **********  . *******  ***    ***

44              YKRWRKGYKWMWYWMYCTGAMKTYKMMYKMRYYKYYKKGYYWMWCCYRRWKWWKKRRAKK
46              CKRWRKKYGWMWYWMYMKKGCKCYKMMYKMRYYKYYKKRCYWMWSTYRRWKWWKKRRMKK
                 ***** * ******* . . * ***************  ****. *********** **

44              YWRWMAYGGTGCTMGAARMMGMRRYTTYKTTGTRRCTKYYRWMMMYCTWKGYYWRMYRYY
46              YWRWMCTRAYKSYMCTGRMCTMRACCGYKCCAARGGAGTCRWCCMTYCWKAYYWRCYRYY
                *****.  .  . * :.**  **    **  .:*  :   **  *   **.**** ****

44              GKKGCTRWYRYRYMGYWCCCCKTGYKCCTTKRTSMRRAYAYRTGMKGAAYMRKRRYKYTG
46              RKKRMYRWYRYRYMCYWTTTTKWRYKTTGCGAASMARMYRYRKKMKTCWYCAKGGCGYKK
                 **   ******** **    *  **      :** * * **. ** . *  *    *.

44              MRCCRRGGYCTTYTCKRYRYRYRKKYKTRKKKCTYTRATGGMKYMYKKYRKAGAKTTYMC
46              MGYTGGKKYTCCYCYKRYRYRYRKKYKARKKKYKYKRRKRRMKYMYKKYRKTTGKYYYMY
                *       *   *  ************:**** .*.* .  **********: .*  **

44              YYRYYKMKMWWKRRTTKYTYAKKCGATTAYKAYRYWYRTTWKTGWKKRRTRGKKGCKKYR
46              TYRYYKMKMWWGRRKYKYCYTTKGRRAGCYGRYRCWYRWWWTKSWKKAGKRRKKSTKKYR
                 ********** **. ** *:.*   : .*  ** ***  *...***  .* **. ****

44              TTGWCGCARKYYGTYACMMRKKRWRKKCKKTYWMKYYCMGKKKKMGGTCSTMYRTKKKMY
46              CAAWMRMRRKYYAAYTSMMRKKRWRKGYKKKTWMKYCYCKKKKKMKKWMCKMYRCKKKMY
                 :.*    ****.:*:.*********  **. ****    *****    ..*** *****

44              WRKRMWRGKRWRKKKCCTCGMKWTRWMKKRYSKKTMKKCGCGSRKMYMWKMWSYKRKSWR
46              WRKRMTRRGRWGKKKTTCMRCKWYRWATKRYSKKWMKKAATTSRKMYMWKMWSYKRKSWR
                ***** *  ** ***      ** ** .****** ***..  ******************

44              WMKYSKCMWWYYRKKTGMGGGCGRSKGTAYMKSACGGYACMWKRMRKKYACYRSKTTSGY
46              WMKYSTTMWAYYGKGAAMKRRYKRSGTGCYMKSRMKRYGTATKRMRKKCTYYRSKGKSRY
                *****. ** ** * :.*     **   .****    *.   ****** : **** .* *

44              YRTYYYWKMYARWCGKKKYRRRYGSYKKRWRSMYKKYKYYWRASKRMYRRGKYKWKRYRK
```

```
46          YRWYYYWKMYGAAYAKKKYRRRYKSYKKRWRSMYKKYKYYWRCSKRMYRRKKYKWKRYRK
            ** *******.   .******** ******************.******* *********

44          YWKGMYYYGCKKCAGAYRKRRMCTSRARYGMKMRGWSRYKMYKTGSMYSYAGYYRMAKYY
46          YWKRCTCCAYKKATTGYRKRRMYWSRGRYRATARSWSRYKMTGYKSMYSYRSYYRMRKYY
            ***     . **.: .******  **.**  . *.******    ***** .**** ***

44          KYKYYRMTGKYKAYYMRSRKGGKAYARMMYRKRRATTKWYWRGRWACAKRKMKCCCATGT
46          KYKCCRMWKTYKGYYMRSRGAAKGYGRMCYRKRRRKYKWYWRARACYMKRKMGAMYWYSY
            ***  **  .**.****** ..*.*.** ***** . *****.* .  **** .    .

44          KATCTRYTGMRKKKWRRWMTGCKCYKRGYKKKKGYCYYGGGGYCRYGYRYRKKKKWRMAA
46          GGYSYRYAACRKKTWRATCCAMTTYKRRTKKGKRTYYYKRRRCYGTSTGTRKTKKWRMMM
             . . **:. ***.**    . . ***  ** *   **        .   **.*****

44          KYRTRKCSYWYRKRRKRYRKAYARYYYCKMKMRWRWRKKYYKKKRYKYRKWWRKYYRKKY
46          KYRWGKMSYWYRKAGKRYGGGYRRYYYYKMKMGARWRKKYYKKKRYKYRKTWRKTTRTGY
            ***  * ******  ***  .* **** ****  **************** ***  *. *

44          YYWYRWYRKKRRYRTTATKTGTRGGAGKRKKKRKATYYTSTGCCKARYKGRRYKKYKYYK
46          YYWYRWYRKKRRYRYKRYKAACRATTAKRKKKRKGATCKCAAYTKRRYTRRRYGKTKYYK
            ************** .  *:. *. :.*******.:  ..:.  * **. *** * ****

44          SMRKMWYCYAGYRYKYGCTCGRYMCCRTTKKKRYWAMRYKYYKARKKTKWMRYCAYTWCR
46          SMRKMWYYYRKCGTKYAGATTRYMMMGYAKKKRYWWMRYKYYKGAKKKTWMRYMGTKTTA
            ******* *     **. :  ***    :****** *******. **..**** . .

44          TTYKRRGAAGMRRKRWSYWRGTYARAGMRKRKYTTYRRWKMRATRCRKAYKMMYWMRMKR
46          CYYKRRAWGAMRRKRWSYWRACYGRTTMRKRKYCCYRRWKMGTAAYRKRCKMMYWMRMKR
              ****. ..**********. *.*: ******  ****** ::  **  **********

44          RSYMAKKRKRRRKKAKKKYKKWYYYKWKRMYKKMYYYWRWCCGRKSKMKKRRMRYWSKMM
46          RSYMRKKRKRRRKKMKKKTKKWYYYKWKRMYKKMYYYWRWMYRRKSKMKKRRMRYWSKMM
            **** ********* *** *********************   *****************

44          GYMRKKKRWKTCARRGMRKKKYAACATARYWRCKYWYGYKWYKKYKTTRKKKAYYKMGGC
46          KYARKKKRWKAATRRKMRKKKYGRTTCTRYWRMKYTYAYKWYKGYKAKRGKKMYYKMAAT
             * *******:.:** ******.  : :**** ** *.***** **:.* ** ****..

44          CKKKKRMYRMCRKAWMYGRKKYKKKKMWKRYWWRMYRMYRYKYKWMYCRRRRMYKKRKCT
46          TKKKKRAYRMYRKGWMYTRKKCKKKKMWKRYWAAMYRMYRYKYKWMYYRRRRMYKKRKMK
             ***** *** **.*** *** **********  ************* ********** .

44          CSYRKGARCCTRRYGRKRSWKAYKYYKWKKRRRWTWAMKMSYYYKRWKYMMMYRYRGYYS
46          YGTRKRTATMWRRCARKRSWKCYKYYKWGKRAGTYWRMKMSYYTKRWKYMMMYRYRKYYS
             . ** :    ** .******.****** **    * ****** ************ ***

44          GGGKRYACYTTCAKKKKYWMSMRKWGCKWMMGTTARKKTCTKWYCTAMWRKYYRRRGTRY
46          ARAKAYMYYWKAGKKKKYWMSMRKWAGKWMMSYYRRKKYTCKWYMWGCWAKYYRRRRART
            . .* *  * ...************. ****.   ***   ***  . * ****** :*

44          YRCCRAKWRRSRRYRKRRARCCCKRCMYMKYRYRRRRRKKKYYKWYKRGCTRRYRYRMCY
46          YRYYRMKWRASRRYRKRRMGTSAKRGMCAGYRYRRRRRKKKTCKWYKRATYAAYRYRMMY
```

```
              **  * *** ********   ..** *   ***********  *****.    ***** *

44            TRYYKKSYKYGCGGYMTTRWTGTTKKWMYYRRTCYKYRYYRYYYKMRMKKWYYYRKSGKY
46            CRYYKKSYKYRMRRYMWKRAYRYYTTTCCCRRAMYKYRYYRYYYKMRMGKWYYYRKSRKY
               *********    ** .*     ..    **: ************** ******** **

44            WRWACRGCATAKACCYAKRKKKGMRKRSYWRMKMRYYMKWRYKARKMYYYWKKAAATATT
46            WATRYRRYRYWKGTTCGKRKKKRCGKRSYWRMKMRYTMKWRYKRRKMYYYAKKMMRWRAC
              *    *     *.   .*****   *********** ****** ****** **     :

44            ARTCGRGAYRTMKMKYCCKWRYRGKKKRKYYYKKWRCWWRKMYKYWKRSGMKRATWRYMR
46            TAATAGARYRCMKMKYYYKWRYRKKKKRKYYYKKWRMWWRKMYKYWGGGSMGRGAARYMR
              : : . . ** *****  ***** ************ *********  ..* *.: ****

44            MSRYMRRKKTYTTTGGKWKWYRYYKYRRKYRCKTSYRRKKKKRRRRWWCYYYRWYRTRWR
46            MSRYMRRGKWCACCARKWTWYRYYKYRGKYRTGASYRRKKKKRAAGTTYYYYRWYRWGWR
              ******* *  :  . **.******** ***  :*********      *******  **

44            WRYAKWRACYAKRKAAYWYMRRRMYKKKKYRMCGAGRGKKKYYKKYCCWKRRAYRRWGYK
46            WRYRKWRGTCCKGKRMYWYMRRRMYKGKKYRAARWARRKKKYYKGYSAAKRGGTGATSYT
              *** ***.  .* *  ********** **** .  .* ****** *.. ** .    .*.

44            RRYWATTARGYYTRKKKKCRCAYRYMYYMYKYTCRRYMYYKMSMKYMYRRYKCYYGGAGT
46            ARYWRCYRGATYWRKKKKYRMWYRYMYYMYKCAYRRYMYYGMSMKYMYRAYKATYATTRW
               ***     . * ***** *  ********* : ****** ******** **. *. :

44            TCRKKWRWWRSMRKRCCGCRKKKYKTCWGTYKWRKCKCATRTTWTRAKGAMKTGATRYWK
46            YMRKGWRWWRSMRKRYMTYRKKKYKYYWSWYKWRKYKTTWRWKWKGMKCGMKYATCRYWK
                ** **********    ******  *. ***** * : * .*.  * .** .: ****

44            RYRRATGGSGACAYYKWMYKWKKKRRRKMKMKKWYMYWKKYKYYKSKTWMWKKYCCYYKK
46            RYRRMYSSSKRYMYYKWMYGAKKKRRRKMKCKKWYMYWKKYKYYKSKCWMWKKYYYYYKK
              ****  ..*    ******  ********* **************** ******  ****

44            KRKRRTCTYGGCCMTTKCAAWMYRYWYKKSRTKGYKKKKYKYRYKKACKCTMYYYKSCRK
46            TRKRGYYWCKAAYMYKKSWRWMYRYWYKKGRKKRCKKTKYKYRYKKCTKYWATCTKSYRK
              .***      .. * .*.  *********.*.*  **.********. *      ** **

44            YYKYWMKKKRRWRRKMYMWKWWSRTAGKKYKYKACTRRAATGYRKKYKSYKCRYRRRYKA
46            YYKYTMGKKRRWRRKMYMWKWWSGCRKKKYKYKWYWGRMRKKYRKKYKSYKYGYRRRCTC
              **** * ****************    ******    *  . *********  **** ..

44            GAGCTYYKWKGMKRWYTCGRYCKYYYKRWGAAKTCTGYKYRKRCRGGCTRYCYYGTGCCA
46            RWKYYYYKWKKMKRTCWYKRYAKYYYKRWSMRGAMGKCKYRKRMGKAMARYYYCTCTAAT
                   ***** ***     **.*******.   :    *****   . :** *    ..:

44            RGMACACGYYRMMKYSCKKYWKATGTTAMWWYRWKKWYRWRAGCAAKYMRTCTRRTGMKK
46            RAMRYRYKYYRMMKYSYKKYWKRWKKWCCTWTRWKKWYRWRTRTGTKYMRAAGGGCSMKK
              *.*     ******** *****   . .  * *********:  .:****:.    .***

44            KKGGGGWYKR
46            KKRRRCWYKR
              **    ****
```

The pairwise alignments of two accessions 44 and 47

```
44      YMWYMAYMATCRTAWRRKMAKRRCYMMRYRYKMMWRRRMRWYYGRYMYWKARWRWWMSRM
47      YMWYCWYMRATRGRWAGKMGKRRYCMMRYAYKMMWRRRMRWYCKATAYWKRRWRWWMSRM
        ****  ** : *  *  **.***  **** ************     *** *********

44      YCTTRYMYYSRGRCRRKWYKKYSSRWGTKCRYWYYRYKGTCMYMRMRYKYRTYKCWRYRY
47      YTCYAYMYYCRSRYRRKWYKKYSSRWRWKMRYWYYRYKAAAMYMRMAYKYRKYKMWRYRY
        *    ****.*.* ************  * ********.:.***** ****.** *****

44      KSYYRSYYRRRYMRYRYGTYRMRSAAYRYWKSTRYYRMMRYRSKYRRRYYMRYSRYYYMY
47      KSYYRSYYRRRYMRYRCTCYRMRSGWCAYWKSYRCCRMMRYRSKYRRRYYMRYSRYYYAY
        ****************   *****.   **** *  ********************** *

44      YWRYRWWYYRYSRRKRYMTGMYYRYYRMKMMRWCYTGTGGYCRKGWYYKYWWKYRKWKYG
47      YWRYRWWYYRYSRRKRYMWRMYYRYYRMKMMATMCKRWKRYYRKRACYKYWWKYRKWKYR
        ******************  ***********    .    * **   ************

44      YKKYGCYWMGCGKYRRKYKMTYMMKMSMWRRMMRWRYRWWKYYYYKYRYYYKKWKYRRTG
47      YKKYATYWMKYAKYGATYKMKCMMKMSMWRRMMRWRYRWWKYYYYKYRYYYKKWKCRRAA
        ****. ***  .**  .***. ********************************* **:.

44      YWYRKWRCRKKYWYGRMYSRKYAWRSAYAKYKYAYYRRYTCTRRYYRWTKWYYYWWGRRY
47      TTYRTWATRKTYWYARMYSRKYWWRSGYTKYKYRYYRRYCYCGGYCATWKWYCCATTAAY
          **.*  **.***.******* ***.*:**** *****     *    ***       *

44      WSCYYYARRWYYRWYGYMGYWKWYKRMYWYYWMRTTRKYYRYMKRRYRWKMKRYYWKRWY
47      TCYYYYWRRWYYRWYTYMRYWKWYKRMYTTYWMRCARTCYRYMKRRYRWKMGRYTAGRWY
         . *** ******** ** *********  **** :*. ************ **   ***

44      RRYTYKYYWYYRYKSWTMRKKYYCGGKMWTRYGYCTWYKWTCMYASKRWKYGACTSYYGR
47      RRYYYKYYWYYRYKSWAMRKKCCYARKATYRTAYTCWYKWYSMYRSKRWKYRWMASYYRR
        *** ************:****   . *   *.*  **** .** ******   :*** *

44      CKATKKYYTMWWWRCAKRWKMKRYKYGGCRYRKRRAMKRWRGTCATCATCKWYKTYAYWG
47      MKRYKKYYKCTTARYRKRWGMKRTTYRKMRYRKRRGATGARKKSRGYWWTKWYKCYTYWT
         *  ****.    *  *** *** .*   ******. .  * ..      **** *:**

44      GYYKMYRKKYWRRKRRRWWMMKYTTYCYSSYKGTAWMGKWYKKRKGYRMMKKKTYYAKYK
47      AYYKATGKKYWRRKRRRWWMMKCKWCYYSSYKRGGTCRGTCKKRKRYRMMTKGACCTKYK
        .***   *************** .   *****  .      **** ****.* :  :***

44      YWYYRRCYWKMYYYYRKKACWYRTYYMRRWSKKRYYYWMWYKRKMWKKYKYRYKRWKRYT
47      YWYYRAYCWKMCCCTRKKTTTCRCYYMRRWSKKRYYYWMWYKRKAWKKYGCGCKRWKRYK
        *****   ***    ***:   * ******************** ****    ******.

44      GYKRWCWCAYCGGGGRYKWRKRCYKKTKMRYYKYYYKKGTAYKWMAACYRRWKTAWKKRR
47      RCKRWMATTTYARAARYKWRKRMYKKAKMRYYKYYYKKRYCCTWMWWYYRRWKYRWKKRR
          ***   :  . ..******* ***:***********  . .**   *****  *****
```

```
44          MAGYKKYWMTGCTCTGGTGMRKKWMYYKRKRMMRKMWYKCGAACTGTYCARRYRRTYRYY
47          CGRYKKYWMYKSYYYTKAAMRTTTCCYKRKRCMRKMWCTMAGGTCTGTTCRGCAGYYRYY
             . ******  .     :.**..   ***** ***** . ...      .*     ****

44          YKACGMRWKKKWAYYYRKKMGTGCCCATRWYRYWRRKRRKRMRYYKRRRWRMRKRYMRKK
47          YKRSKCGTKKGWMYYYRKKMTYKYYAGKGWTRYWRRKGGKRMRYYKRRRWRMRKGTARKK
            ** .    ** * *******     ... * ******  ***************   ***

44          TRKWWMKYMRAYWGYGGTRGKTRYRAGKMMKTGMMKWWRYRKTGKKKARRKYYYKYRKGK
47          CRKTTMKYCRCYARTRRWRRKWGYRTTKMMKWKMMKWWRYRKCKKTKRRRKYYYKYRKAG
             **  *** *.*      * *  **: ****  *********  *.* **********.

44          KASRWRWKMGGTGKKKGKYKRRMYSCTATYRKARMYRCAGCMRWMTGRYKKCARKRKYGG
47          KRSRWRWKMKSYKKTKRKYKRRMYSMWWYYRGRRMYRYWRYMRWMYRRYKKYRRKRKYRK
            * ******* .  *.* ********    **  ****    ****  ****  *****

44          KTKKYKKGKKYWYKRMRRCCCWMRRRKYAKRGYRRKCYGSRWATTTCWCCTGYRATWYKK
47          KKKKYKKRKKYTYTRMRRTAAACAGATTGGRTYRRKTYKSRWGKAAMTMAKTYGGGAYKK
            *.***** *** *.**** ..     . . * **** * ***..::   .. * .  ***

44          RYYMRKSYAGMWKKCYTMWMKTKTGYGKTKTTTGRARKWKGMRRRYRRYWRRYSKMWYKY
47          RYYMRKSYCTMWKKYYKMWMTGKWSCTKKKKWGAGGAKWKKMRRRYRRYWRRYSKMTYKY
            ********. **** *.***. * .  *.*.  . . *** *************** ***

44          GMRMSMYGTTKWYWGTGKYWWGTYWYKKYYMKYMYKKRRKKMRRKCWCTMYYRRMSYKRR
47          AMRMGCCRYYKWTWACSKYWWAYYWYKKYYMKCACGKRRKKMRRGMTTCMYYRRMSYKRR
            .***.     ** *. .****. *********    ********     ***********

44          WKCKRGAMYMTATKYCRWMRKRGATRSMKRKGAGRTTTMKAYKKRKYTTKWGTGGMMKTR
47          WGGKRKMMYMYMYKYARWMRKRRRKRSMKRKTTCRKKYCGTYKKRKCYYKWRAARCMKWR
            *  **  ***   **.******  .****** : *..   :*****   ** :.  ** *

44          KCWMRRRRRRATCYKRAMGRKMYRYMYRSYGARRRRWRYTAKTAKYKYCTAWKGMRCTYR
47          TMWMRRRRRRGAYYGGCCRRTMTRYCYRSYKRGGAGWRYWWKYRKYKYMYRWKRAGYWYR
            . ********.: *  .  *.* ** ****      ***  *  ****   **     **

44          RRWYGYYYRWRTGRRGMWTYKRYYRYCCGRYTYRAAAYMYKWYRARRRKKMYYWKKRRYM
47          RRWYRTTTRWRGARRKMWGYKACTGTTYRRYYYRGTRYMYKTTAGGRRGKCTCAKKRRYM
            ****    *** .** ** **        ** **.: ****   . ** *    ******

44          GYWTYKKKMACATYRRKCKWRCTKMWYMTGGWYRYCSYCGGRCYYKYMAAGTGTCARKRY
47          KYTACKKKMGTGCYRRGYKWRYYGATCCWRAWYRYYSYTTKAYCYKYCRRRYSKYRAKRY
             * : ****. . ***  ***         .**** **      ***     ..   ***

44          RKKYYRKYRYMYKMKYRKAGAYCKYYMKKGRWYGKCRARSKYYCGKCAKTCTATCYYSKM
47          RKKYTGKYRYMYTMKYRKTTGCTKYYMKKKRWYRKMRMRCGTCYRKGGKWMYWYYYYSKM
            ****  ******.*****: .  ****** *** * * *.     * .*      *****

44          KWWARRRCYRYWARYKWMYMTAYATRRWKGWCTKKTRRKTKYKRKKKRRKKYRWCGCAKK
47          TATGRRRYCRYWMRYKWATMACCGARRWTSWYYKKKAAKWKYKRKKKRRKGYRWMRMRKK
            .  .***  *** ****  *:. .:***..*  **.  * ********** ***    **

44          YYTGCKKRRRTWRYRKKRRTRWKYYMKYMCGRKYRTKCKARYYYMYWRKTTRYWYYRKKY
```

```
47    YYCRYKKRGGGWRYRKKRRWRWKYYMKYMYTRKYRWGMKWRYYYMYWRKKYRYWYYRKKY
      **   ***   ******** *********  ****   * *********. *********

44    YTCGGKKKKYSKWCMRAYKYYKKRSRRKGKTMRGAWSYWRGTMRKRYRRCKMKRRWMATA
47    YWATAKKKKYSKWAMRGYKYYKKRSRRKRKKMRKRWSYWRACMGKRTGGTTMGRRWMCAG
      * . .********.**.*********** *.**  *****. * **    .* ****.:.

44    YKCRGMYKKRRSYTRKTTAYKRKKYRKYKKMTTTKYKKYKWYKWKMYYYWGRMRYRATAT
47    CKTGACCKKRRSYYRKWYMYKRKKYRKYKKMCGWKYKKTKWYKWKMYYYWKRMRYRGCGW
       *  .  ****** **   ************   **** *********** *****. .

44    RYWRKKKKSRRRYKAWRTAARGTTKKMRKTMKWGAARRRKGATKCYCKWYYGGKKYSYWC
47    RYWRKKKKSRRRYKWWRYWMRTACKKMRKCMKWRWRRRRTRGWKYCYTATCTCKGYSYWM
      ************** **   * : ***** ***   ***. . *   .     * ****

44    TRATTYRRKAKYRRACARRMYKYRYKKMYYRMMMWYRCAKRRCGMCGYKWRGWYWYKRWY
47    AATAWYRRKRKYRRMYCRRMYKYRYKKMYYRMMMATGSCKRGAAMSRCKWRKWYWYKRWY
      : :: **** ****  .*****************   ..** ..*.  *** ********

44    RRYWYKWWATCTTAAAMRRYGKMAWCAAYYKKKWSCRKYKKRKKRRTAYYKRKYYMMMKT
47    RRYAYKWWMWSWWWMRAARYRKCGWYCRYYKKTWSYRKYKKAKKRRYMYYKRKYYMMMKC
      *** ****  .       ** * .* . ****.** ***** ****  ***********

44    RRRYRYYAKAYYKKRKYWRRMKYKWMMKKWMMYRKWTKYYMRKKKGATGTAYYYRKWYRR
47    GARYRTYRKMYYKKRKYWRRMKCKWMMKKWMMYRKWCGTTCAKKKRWKAYRTYYRKWYRR
        *** * * ************ *************      ***  ..   ********

44    KATRSRRYRKRGRCYKRMMRRKKYKRYWWCYKYSTMKRAKWYRKKKYRYMRKKMYKRKRR
47    KMWRGAATRKRCATYGGMMRRKGYGRYWWYYKYSCCGGMKWYRKKKYRYCRKKMYKRTGR
      *  *.   ***   *   ***** * **** ****     ********** *******. *

44    YKYSYYYRCTGSYYKARKMCCGTKKKRMCYYYKGKYRYKKKKKKWRMGGKRYRKMKRRYK
47    YKYSYYYRYASSYYKCRKMYYAAKKKRMMYYYKSKYRYGKKKKKWRMKKGRYRKMKRRYK
      ******** :.****.***  .:***** ****.**** ********   **********

44    KYSYWYRKKRKKWYRYKRKTYMMRWRKACTKKGCKYYMYRRRYRWYRKKRRYRYYKYKYA
47    KYSYWYRKKRKKWYRYKRGCYMMRWRKCAWKKAMKYYMYRRRYRWYRKKRRYRYYKTKYM
      ******************  *******.. **. ********************** **

44    WKKYKYYSKRYTKMRGCGCCYGCTKKCYGKMYYMYRSMRKMWYYRRYYSRYKYYMYMCAR
47    WKKYKYYSKRYYKMRKYKYYYRMYKKMTRKMYYMYRSMRKMTYYRRYYSRYKYYCYMYRR
      *********** ***     *   **   ************ ************ **  *

44    YSKKKKMRKWRKYSTYKKRWYYYYRYYKKMRRGKKKMATCRKMKRKWKKMKYMKKKRRWA
47    CSKKKKMRKWRKYSYYKKRWYYYYRYYTGAGRRKKKMCGYRGCKRTWKKMKYMKKKRRWR
       ************* ************.   * ****.  *  **.*************

44    TTGMYMMAGYWKRYRRYKWCWMYYKKMYRKCAGYYGAGMRRCYRMKMRWSRYATCTSACS
47    YYRMYMMWKYWKRYRRYKWYTMYYKKMYRKATTYYRTAMRRAYRATARWSRYMYYKSGYS
         ****  **********  *********.: ** :.***.** . *****   .*. *

44    TKYYKYKRRAWYRKRYCMMKAYGKGATTKRWYCCRKRTAKRKKGYWYRYRAACARYGAGG
47    YKYYKYKRRMWYRKRCMMCKGYKGKRKYKAWYYYGTACCKRKKKCWTGTGGRTMGCKGAT
```

```
         ******** *****  * *.*    . * **   .  .****  *    .      ..

44      AGRYYRWKKCMYRRRTMYYKYKYTCGRAWYRWGGGCCKAGTCRRRMRMYRYMMRYKKYYT
47      TTRTTRAKKTMYRRRYMYYKYKCWMKRRWYRWRRRMTGWRWYRRRMRMYRYMMRYKKYTY
        : *  * ** ***** ******    * ****          ****************

44      SAAKKGTCGRAWTKWYYKRMKRYRRKGWMMYRYMKYKYRMKKCTWYYKGGCTTGGAGCYT
47      CGTKGACAARWAYKWYYKRMKRYRRKKWAMYRYMKTTYRMKKTCWYYKATYGYSSTTTYK
        ..:* . ..*   ************* * ****** .*****  ****.    ..:  *.

44      KATRKKKAMTRYKRKKYMRKRATAYKGGRYKKRRRYKKYRKMYKSRRRYRWYRYKGGKRM
47      GWYRKKGGAYRYKAKKYMRKRTAGYKTARYKKRRRYKKYRKMYGSRRRYRWYRYKKRKRM
           *** .  *** *******::.** .*************** ***********  ***

44      MSYKYKSAYKYKKWWKTRKKRMGRRYGGRWKKTRWRMRYMKRGATKKMMKKACSRMMRSW
47      MCCKYKSMYKYKKWWKYGKKRMRRRYKRRWKKYRWRAGTAKRKWKKKAMKKTYSRMMRSW
        *. **** ********  **** ***  **** ***    **  .** ***: *******

44      SYKRKSRYRKMYWYKYKYGRAGRWCCMRSKTCAGWKKTAYMKSMRSMYAYGCAYKRKMRY
47      SYKRKSAYRKMYWYTYKYAGMAGTTTMRSKAAGAWKKGCYMKSMRSCTTTTGTYKRKMRY
        ****** *******.***.  .    ****:...*** .*******  :   :*******

44      RRSKKYYRKTYRKYYWKMYTKKRRRYKSRATAYAKSKMYKRYKKYYWRSKGRMYRKKYKM
47      RRSKKYYRKWYRTYYWKMCGKKRRRYKSGMWWYWGSKMYKRYKKYYWRSKKRMYRKKYKM
        ********* **.*****  ********    *  *************** *********

44      YYWYKKKRRRKMMYMKKYRKKTKYCCKKWCTMCWCKYKKRKRTKGCMKRKKYYKRYKYKW
47      YCWYKKKRRRKMMYMKKYRKKKKYTTKKWGAMYWYKYKTRKRKKAAMKGKKYYKAYKYKW
        * *******************.**  *** :* * ***.***.*..** ***** *****

44      CCMRKGKKWYMYYKKKRWKYCTSWRKRCGYYKYRCCTMKKRRRTRRMWKRYKMYWWSRTK
47      YMMRKKKKWYMYYKKKRWKYYWSWRKRYAYYKYAATCMKKGRRKRRMWKRYKMYWWSRYK
          *** **************  ***** .**** .  *** **.************** *

44      YTGKKYKYYKCCGYRTGGTYRRKWCATTWMYWWYMKRMSYKYRRRCKYYYMAKGKRWKRG
47      YWTKKYKYYKAAAYRYKKYCGRKAYTYGAATWWYMKRMSYKYRRRAKYYYAMKTKRWKRK
        *  *******...**      **  :     **************.****  * *****

44      MRWCYWRYYCGGGKCMRYRYWRCKACAYKYMYYKTGCYMKKKKSKMYTGTKWGRMYARKK
47      MRAYTARYYAAAAKYCGYRYWRMGTYMYKYMYYKKKAYMKKKKSKCCATCKWARMYGRKK
        **    ***....*   *****  :  *******. .********  :  **.***.***

44      KKKWRWAWYRMYRKKYWACRWRRKYTAWGYTMYKCTCSYRKYYGKSWKKRMYYWRKRRKC
47      KKKWRWGWYRMYRKKYWMYRWRRKTCGWRCCMYGMKYSYRKYCRTSWKKRMYYWRKRRKY
        ******.**********  *****  .*   **  . *****  .**************

44      YKKKKSWRYYKKRWCRYGYKWSYYKRKAMKKATGCTKYRYKRKYWYYYRRRRMAGRRRCG
47      CKKKKSWRYYKKRWTGYKYKWSYYKRKGMKKRKKMWKYRYKRKYWYYYRRRRAWRRRRGA
         *************  * *********.*** .   ****************   *** .

44      AGCMYYMRYRKGCCRGTKYRSYWYMMYYYYYRKYRKKAAAKRMKKKYMKKYRKRKKKYKS
47      RRTCTCAGYAKATYRCCKYRSYWYMMYYYYYRKYRKGRWRKRMKKKYMKKYRKRGKKYKS
                * *.  *  *******************    ************** *****
```

```
44      AYTKRRGRTKSRKKSKTGKRYWRMRCWRRKYRYKAWYWWSYYKRWKRYMMMKYRMRYMAA
47      RTCKRRRAAKSRKKSKYRKRYWRMGMTRRKYRYKMWTAWCYCKRWKRYMMMKYRMRYMRW
           ***  :*******  ******   ******* *  *.* ****************

44      RYWWYYKRKKRRYGTGRRACWSRYRKKAATGKYYYYRRYRMRRRRWRYWYYMKKKRKWYY
47      RYWWYYGRKKRRYACRRRTYWSRYRKKRRYSKYYYYRRTAAGRRRWRYWYYMKKKRKWYY
        ****** ******.  **: *******   .*******    ******************

44      RYKRKRWKTYWYKTGKRYRKYMRCMRTCARRKKKYYRYYYSAKCKKMSTCGMRRKKWKKW
47      RYGRTATKYYWYKWKKGYAKYMGYARATRGATKKYYRYYYSGKMKKMSGTRMRRKKWKGW
        ** *.  * ****  * * ***   *:    .*********.* ****   ******* *

44      CRYWRKRRRKRYRYRGKAWRYYCMMWRYKYKKKAKYKKKAYKRTTRYATCTMCCYWKKKC
47      YRYWRKRRRKRYRYGKKRWRYCMAATGCKYKKKGKCKKKTTGRAGGTRWYKCTAYWKKKY
         *************  * ***       *****.* ***:  *:      .  .*****

44      CYWTWGWYYYMWASWSYRMWYARTMWYKMKKYYYRTAKGWYKTTGTGYTAGGTKYMKYWK
47      TYTGAKAYYYMWWSWGCGMWYGRYCACGCKKYYYRWRKAWYKWYRKKCCMRRKKYMKYWK
         *     ***** **.  ***.*      ******  *.***    .      .*******

44      YYKYKYRCYYKKRKTTWMRMTCRKRTMKTRCTCGYMRKSKTCAAAAYMYKRGYKYAGKKR
47      YYKYKYRYYCKKRKKWWMRMAARKGYCKGRAATKYMRKSTYYTTWRYMYKRRYKYMTKKA
        ******* * ****. ****:.**   * *.:  *****.  ::  ***** ***  **

44      YTYWTRYTYRMRYAKKCRYGYRKRKCAWRKYRYGTGYKYWMKYKMKYWWAGGCKYAYTKW
47      YCCAAGCGCACGCRKKYGTAYRKRKGGWRKYGCACKYGCWMKYGMKYWWGCAAKTWYKKW
        *   :         **   .***** .****  .  *  **** *****. ..*  *.**

44      KTTAMKMMRKTRSWAYYKRAKACYTRGMRRKAGYYKCGGGKATTGAKMWKGKCKYWWKKK
47      KCKRMKMMRKKRSWMYYKRGGTTCCGTMRRTTTYYKYTATTRYWRRKMWKTKYKYWWKKK
        * . ******.*** ****. :     ***.: ***  . .     **** * *******

44      RRSCYKRYKRYYKSKTAAKGRYRYYKKRRYGYYAMMTYMACACKKYYYMRMMKKKYYSTK
47      RRSYYKRYKRYYKGKCWTKRRCATTKKRRYTYYMMCCCMRYRYGKYYYARMMKGKTYSKK
        *** *********.*  :* *    ***** ** *   *     **** **** * **.*

44      YYWKYTYYKRRRYWYYYKKKSRWKRYYRKRKYYYKKMGAWKCCYYR
47      YYWKCCYYKRRRTWTTCKKKSRWKRYYRKRKYYYKKAACAKYYYYR
        ****  ****** *   ******************* .. *  ***
```

The pairwise alignments of two accessions 44 and 48

```
44                    RTRRGKMMYCCAGCYSRRYTRMGMKKKYCCMMCRAWWKWKACTCCYMWYYGYYRSGAMCG
48                    GKRRKKMMYYYWKYYSRRYYRMRAKKKYYYCCYRMWATATRMYYYYMWYYACCRSAWMGA
                       .** ****     ***** **  ****     * * . .     *****.  **. * .

44                    WKGGGTAMYRTGGMKMYSMYCTMYWRKRKTRGMMWRYKYRWWKKARYYYYYKYRGKYMGK
48                    WKKRKWMMYRWRSMKMYSMCAAMYARKRGGAAMMWRYKYRWWKGGGYYYYYKYRAKYCRK
                      **     ***  .****** .:** ***   .*********** . ********.**  *

44                    AKWRRRRYWYWRRKWYYAMKYCCRYYRWCMMKYGWRYKGCYSWYKYYRRGSAYMRYGRTG
48                    RKWRRGRYWYWRRKWYYWMKYYYRYYRWTMMKYKARYKRYYSWYKYYRRTCGYMRYRAWR
                       **** *********** ***  ***** ****  ***  ********* ..****

44                    MRAARWAWKSRYMRKMYRRATAGWRRRYYYAWGRRYRRCGTRMRYGYYYYWKWMCYYSWR
48                    MRGWATRWKSRYMRKMYRRGGTTTRRRYYYWWRRRYRRYAWRMGYRCTTYWKWMYCCSWR
                      **.    ************. :  ****** * ***** . ** *    *****   ***

44                    RKWRYRWYKTYKKWMRAYAYMRRKYSSYCCTRYMYYWKYYRGYKYWKRRYYWWYYYRGGA
48                    RKWRYRWYKAYGKAAGCCGYMRGGTSSYMTWRYMYYWKYYRRYGTAGRRCCATYTYRRCG
                      *********:* *   . .***   ***   ********** *    **    * **  .

44                    CCAATKYYWCRGKYMAACKRYYYYKSRYYRCRYWWCRCYMRMWMKYMKAGMKRMMWTRRK
48                    TTWWYKYYWYRRKYMTGTKRCYYYKSRTYRYRYWWGATYMRMWMGYAKCKMKRAMWWAGG
                           **** * ***:. ** ****** ** ****   ****** * *. *** **

44                    RKKRRWYYCRCWKTYYGGYKGRKYRWYWWKYGKKYYKRMTYKKAMCMCGMRMKYRCYMWY
48                    RKKRATCTYGMWKACCRKYKTAKCAWYWWKCKKKYYKRMAYKKGMTCATCGCKYAMCMWT
                      ****       **:    **  *  *****  *******:***.*  .    **   **

44                    YSYYRGWAWKWTAAKMYWRWGTCKKYRTGTYASWYKKWYYYKWRSGTCCCAGGRWYYYAA
48                    YSYYRATGWKWCGTKMYWRWAAAKKYGAAYYRSWCKKTYCCTWRSRWYYYGARRWYYYWR
                      *****. .*** .:******.:.*** :. * ** ** *  .***     .. *****

44                    ARWRKKYCKCKKYKKRKKRKMTAMRYRKMRKRRGYKMYKYKCGKCKMKYGGGGRYGGKKY
48                    WRWRKKYYKMKKYKKRKKRKAWWMRYRKCRKRRRTGMYGYKMRGYKMGTKRRRRYKSKKT
                       ****** * **********   ***** ****   ** **    **       ** .**

44                    KKKTMRCKYWRKKSYWYYYKRKKTGYYRKYYRYKKCRTKYMWKMRRKMWKKRKKYMRYWR
48                    TGKCCGTKCWRGKSYWYYYKRKKGAYCGGYYRYKKYGKKYMWKMRRKCWGKRKKYARYWR
                      . *    * ** *********** .*   ******  .********* * ***** ****

44                    KKCYATYRYYMYRKKRRYRTTATYYKKYKKMRWWTAGKKKRYYYSTTGKRYYYRYRKGGR
48                    KTYYRWCATCMYRKKRRYRYKRYYYKKYGKMRWWGMRKKKRYYYSYCRKRYYYRYGKAAR
                      *. *      ********* .  ***** *****   ********   ******* *..*

44                    MKKCTKMYYSMRKMRRYYYRYYRGYKYCSAGAKCGKKKWWYRMRKYYYYTRAWYAKRRGA
48                    MKKYKKMYYSMRKMRRYYYRYCARTKYTSGAWGTKKKKWWYRMRKCCTCGAGAYCKRGRR
                      *** .****************    ** *..    **********      . *.**
```

```
44          GRRAAMYWGTGKWCATKKWYYMYSYWGATYYRRGCAKYCATRKRYMGYKYRYKKRCGTCW
48          RRRGGCYWAATKTMTYTKATCMYSYWRRWYTAGRMWKYYWYRTRYMKYKYRYKKRTTCTA
             **.. **.: *  : .*   *****   *      **   *.*** ********

44          TSMKWRTATATGMRCYKTYRYRYTGKKRGACYYWYRARTCGYAGTTCCCYKMGCAAAYYS
48          CCMKWRCRCCCTMRTYKKCGTRCCTKKGKRMTYWYGCRCGAYRRYCTTYYKCCMWCWYYS
             .****   .  ** **.   *   **     *** .*  .*       **    . ***

44          MGRRCYMMWGWKYYKKYCGYMGRKRKKKYKKRKCCCGKYRKMYMYRMKKAGAKKATTCKT
48          CRRRYYMMWKWKYYKKYTACCKRKGKKKYKKGKAYYRGYRKMYCYGMKKTTGKKMKKAKA
              ** **** ******* .   ** ****** *.    ***** * ***: .** ...*:

44          RWYGKCRAMTKGGTYGMKRYMRRKYMRYWWMYTYRRWCCATRGTCARKKTTAAKSWYWKK
48          ATTRKMRMCYKATGCKMKRYMRRKYMRYWTATAYRGWATTCGAYMWAKTCWGGKSWYAKK
                * *   *.    *************   :** *. :  .    *.  ..**** **

44          KKKRKKRRRKYRWCGCARWYKRKYWYGMMMRKKRYRYWMGGYAAWKWYTWKKYYYTGCYK
48          KKKRKKRRRKYRWMRMRRWYKRTTWYRMMMRKKRYRTWMSAYWTAGWYWWGKYYYCTTYK
            *************    *****. ** ********* **..* :  ** * ****   **

44          YMMYTCKSTGRRMYRRYRKKAGCGAACWTAMACGCYAGGGRWKWYRKRRRMKRYRTRKTW
48          CMMYCMGCKKRRMYRAYRKKRRYATTATATCGSATYWKRKRWKWYRKRRRMKRYRYRKYT
             ***   .. ***** ****   .::. :: ... *    *************** **

44          TRCAMCTYTAKSYTATMCARAYMRWYYKGCKRYYYAGAKRYWYRRKCTATMYWYGMAYMR
48          KRYTMMKYCCKSYYRWMYGRWYMRWYYKATKACCTGSWGRYTYRRKACTKCTWYKMRYMG
            .* :* .* .***   * .* *******. *    ..  ** ****. :.  ** * **

44          YKKYRMKRSRRKKTTCCYKRYRKKKSRRWAYRGMTMWYKKRYKKRMWMSKYRKMYKRRYR
48          YKKYRMTRSRRKKWCTAYKRYRKKKGGRWRTAKCWAWYKKRYKKRCTMSKYRKMYKAATG
            ******.******   .********. **       *********  *********

44          WYRYRRTKYWYMCRCYKMKKKTYKKGARRKMKRWRCTATYRMKSMRTRYYRYMRWWYKTT
48          WYRYRRKKYAYMAAYYKMKKKKYKKKGRRKCGRWRMWWYYRMKSMRCAYYGYMRWWYKYG
            ******.** **.  ******.*** .***  ***    *******  ** *******

44          KKGAKTATTAYKRKKYMMTWCRRGKKYTCRGTKRYYMRGCTAGCYCAYAGKRTTGYCCYY
48          KKAGGACCGCYKRKKYMMCTYARKKKYKTRAWKRYYMRAAGRTTTAGCGSKRYWAYYTYY
            **.. :.  .********    * ***. *. ******..     .. ..**  .*  **

44          CGCKKKTRMRYAMKKYYKMWMKYMKTMYMYMTWRRTYKWKGKSYKKATMGYWRKCAAGKR
48          YSYKGKKAMRTRMKKYYKMWMTCCKWCCAYCYWRRCYKWKKKSTKKMYAKYWRKTCMATA
             . * *. **  *********. *    *  *** **** ** **    **** . ..

44          YKRMYRKYMRYKYKYRWKRRKRGTRMMKKMYYYSSMRKWWGKMKKKTKKSSMCACKRRYY
48          CGRMYRKYMRYKCKYRAKRRKRRWAMMKKMYYTGSCATATCKAGKKYGKSSMYRYKRRYY
              ********** *** *****   ******* .*  .   *  **  ****   *****

44          KKWKYYYMYYYYRMRRWYSKKKRYKSGKRKYYWMCTMWYKRGGTKWKYRWMCCATCTRYK
48          KKWKYYYMCCCTRCGRWYSKKKRYKSRKRKYYWMYYMWYKRAACKWKYRWMYAMWYYGYK
            ********    *  *********** *******  *****.. *******  .     **

44          KRMRKWMYRRCGRCYTKMKWMCAGMRYYTYYKKYAKWMCAATKRRRRKKKWSWKKKWYTG
```

```
48               KRMRKWMYRRYKGYYKKMKWMTTKMRYYKYYKKCCTWMSMMWKRRRRKKKWSWKKKWYCA
                 **********    *.***** : ****.**** ..**.   *************** .

44               CRWMYAKCGAYKMGGTKKAKRRRMMYCYKKRKYKYYKCGWKKYTKKKYKRGYTTATTKCA
48               MRWMYMKYKRYKMATYKKMKRRRMMYYYGKRKYKYYKYRWKKYKKKTYKRTYYYRYKKSW
                  **** *   ***.  ** ******* * ********  ****.**.*** *    .*.

44               AKRRKKKRYWYKKRGMWYRRKCTAKTKYWRWYKYACGYKKTMYYYKSGWRCRKKYRKKMT
48               RGRRKKKRYWYKKARCWYRRKTYRKYKYWGWYKYWYRTKKWATCTKSRWRYRGKYRKKAW
                   ***********   *****   * *** ****    **      ** ** * *****

44               TKKKRRRRWRRMYMWKKMYWWSKCACTKYKSKKSRGYTAAKYWTAGCTWYYTMRMSYRKC
48               KKKKGGRRWAGMYMWKKMYWWSKTTTCKYKCKKSRKCYRMKCAKCAYCTCYCMRMSYRKY
                 .***  ***  ************ :  ***.****     *   ...    * *******

44               RWWYSKYMRGTKYGTRKKKWWYTRRKRYYYKKWYYYGYKCCTGKARYKKTCMGATAARTA
48               GWWYSKYMRRYGYRYRKKKWWYWRRKRYYYKKWYYYKYKGTAAKWRYKKWMCKTCTTRCG
                  ********   *  ******* ************ **  :.* ****    : ::* .

44               CAKRYYYYRKWYYYYWSCGCYCMRYSCWMMWYKYAWRRTRYMWYKRYYYRKYKGTTCGTCA
48               YGKGYYYYRKAYYYYTSYSMYTMRYSSWMMWYKYGWRGYRYMWCKRYYYRKYKSYCYRYYT
                  .* ***** **** *  . * ****.*******.**  **** *********.      :

44               YWKKGTYKSYRCRTGRCYTARSKKYRKKCRTAGTTRATGKKKRRMRTAWRYYKKRWRYMM
48               YWGKKWYKSYRYRKARMCWGACKGYRKTYRCWKYCRCYTKKKRRMRCRARYYTGAARYMM
                 ** *  ***** *..*   . .* ***. *     *.  *******   ***.   ****

44               YMRTATGTYKTWKMCWAYKKKKKYRYKRKYWKYYYKYRRRRCAACGCGRRKYYMRYWRKK
48               YMRKCGAGYKKWKMTTTYKKKKKYRYKRKYWKYYYKYRRRRYWTYRMARRKTCAGYTRKG
                 ***.. . **.***  :**********************  :   .***    * **

44               AYKRSYWYKMKYYYYRYMSKRTGKWWKRYRWKKAKKWYMRCWCYKRKRKYKSKTAARRRK
48               RYKRSYWYKMKYCYYRYMSKRKRKWWKGTGAKKWKKATMAYATYKRKRKYKSKCGGRRRK
                  *********** ********. ****    ** **  *    ********** ..****

44               MRRYRKYGSRGKSKKRKMYRKKYYSRGGMRKGRWWYWARCGKKSRYKWRYYYYMYCCGRT
48               MRRYRKYKSRRKSKKRKMYRKKYYSRTAMGKRRWWYTRRYAKKCRYKWRYYYYMYMMKGA
                 ******* ** ************** .* * ****  * .**.**********    :

44               KRYRMWKCTYAAYYYYSGKWMRYAGCTYCGCYRSRRYKRAGGAKGYWRTTTRKMKRTYKR
48               KRYRMWKTCCGRYYYYSAKWAGCCRAKYTATYRSRRTKRCAKRKKYWRGYCRKAKRCYKR
                 *******   . *****.**   . ..* . ***** **..  * ***   ** ** ***

44               RWKGRAKATRMMYMKRRSMKKRKKKYRRRKKKKKKYKKWYKCWKKMYYYWRRRGMGGRRY
48               RWKAARKTARMMYMKRRSMKKRKKKTAAGKKKKKKTKKWYKMWKKMYYYWRGGCMRKRAC
                 ***.  *::***************    ****** ***** *********   *  *

44               AYRTTKYRKMYKKKMKWACMYMCTCATYRKRGKTYYMRMWMRKWMARRWKYRMGGRYYGR
48               GYRCCKYGKMYKKKMKAWMMTCTCTGGYRKAKTACYARCWMRTWMRARWKYGCRKRYYRR
                 .**  ** ********   *     . ***  .: * * ***.**  ****    *** *

44               ATAKKRTGKTATCCTAKAYMKRTTYARYGYYRKRKRTKATCAKYCWATATWRRYKYCWGT
48               RATKKRWRKYTYYTCGKGYMKRKCYGRYKYYRKRKRWKRKSRKYYWGWGCWGGCGCMTRC
```

```
       ::***  * :    .*.****. *.** ******* * .. ** *. . *

44     TYCMRMMKMKAYYGTMYTRKYGRKGRGKKKKCCGGGKRCAGATWYCGCTYKKYTRYGKYM
48     CYYARAMTMKGCCACAYCGKYRRKKRKKKKGYAAAAKRMWRWWAYYSTWYKKYARYRKYM
        *  * *.**.  .  *  ** ** * ***  ....**      * .  ****:** ***

44     KKGKYMAMWMYTGKRKCKKMKRYWATRMYRYKWYWYRRYGCTCSYSKYRRGASWKKWKCA
48     KKKKYMWMWMYWTKRKYKTMKRYWWWRMYRYKWYWYRRYTMKYSYSGYRRRRSWKKWKAC
       ** *** ****  *** *.*****  *************  . *** ***  ******..

44     TGTRATTRCYYKYYRYRRWWKWYTTCMRTYMRSYKYRMRRTYMRKWWYKKKKYWYGAGRG
48     CAYRWAWAYYYKYYRYAGTWKWYYCYMRYYARSYKYRMRRKYMRKWWCKGKTTWYRGAAA
        . * :   *******   ****   ** * *********.****** * *. ** .. .

44     YYKRRKKWYRRYRMWSYRRKRRGYRWYRTGKRYCWRMAYKKKRKRWWYKKKKKYWYMRRT
48     YYKRGKKWYRRYRMWSYRRKGGAYGWYRWTKRYMWRCRYKKKRKRWWYGKKKKYWYMRRY
       **** ***************  .* ***  *** **  ********** **********

44     CGGAYYKKMRKYYYYYSYYWKKWWKMYTGCRRKRWATWWRRYKKRWYGKKRYYKRKKMRY
48     YRAGYYGKMRKYYYYYSTTAKKWWKMYCATGRKRTTWTWRRYKKRWYAKKRYYKRKKARY
         ..** **********   ******* .  *** :  *********.********* **

44     RKGKKRACCYGGSRCRAYTMMRSYKRSRRWSWTWYKKCTTYTCKYMTGRKRRRSGRKYMA
48     RKKKKRGAGYKRGGYRRYYMMRSYKRSRRWGTKWYKTAWYCYYKYMAARTRRRSKRKYMR
       ** ***.. *  .  * * ***********. .***..     ***:.*.**** ****

44     WKKYWYRTKKRAAAMRKKYYRSKKGKSYYRTYRKGGAKYYWKKKKYRRRYGSRKRSWYMY
48     WKKYAYRKTGAGTTMRKKCYRSKKRKSYYRWYRKACTKCTTKKKKYRRRYKSRKRSWTMY
       **** **..  .::**** ***** ***** ***. :*   ********* ****** **

44     KTYYWRRMYKCKRKYKYKYWKRSYKTWYYYWYRGGAGRWRYRYYKRWRKARTYYRRRKMM
48     KKYYWRRMYKGTRKYKYTYWKASYKCTYYYWYGRSMKRWGCATTKRWRKMGAYYAAGKMM
       *.******** .*****.*** ***  *****  .  **     *****  :**   ***

44     GCKYYGARMMKRKKYGCRMSKGKATRYMTCYYYRWRRWKYKMKSRWKWWTRYSMRATGGC
48     AMKYCRRRMMKGKKYSYRASKRKRWRYMCMCTTGWRRWKYKAKSRWKWWYAYSMRRYKKY
       . **   **** ***. * ** *  ***      ******* *******  ****

44     YKYKYTKKKKMSMWRRCRYTACKYCAGWTAAATAMAYRWTRYTYTYCKRARYRAYRRATR
48     YKYKTCKKKKMCAWRAYATYRYKTATAAACGTKGMTYRWKRYYTWCMTAWRYRMTRRGAG
       ****  *****. **       * .:. :..:..*:***.**     .  ***  **.:

44     TTMMRYYRTAAGAGKWYRYRWRKKYRTCGCCTTTMYYYRMMYYCKGWRRYMMWRTGRYYR
48     KCCCRYYRWRWRWKKWYACRWRGKYRYYAYYWWWMCCTGAAYYMKRWRRYMMTRWRRCCR
       .   ****      ***  *** ***  .     *      ** * ****** *  *  *

44     TSTRRYCSGCGRYWATGTAYAGTCCCARYKYRRKWYYYSKRRYWYRYRKKKKRWYKYMYK
48     WGCRRCMSATTACTGYSKWCMKKYYYRAYKYRRKWYTTSKRRYWYRYRKKGKRAYKYMYK
        . **  *.     . ..    .     ********  ************ ** ******

44     RYRRKRRYKYRWMYYRRMYGKKMKRYRYMMYGGAMRMRSACAWMRKRGATKKRYRRSMKG
48     RYRGKRRYKTGTMYYRRMYRKKAKRTATAAYATTCGMASRYWWMRKRRRKKKRYRRSMKT
       *** *****   ******* ** **     *. :  * *   *****  .*********
```

```
44      AGRYYMTKAYKTCWGTTKWYSWRKGRGYTRCARGCYYWYYRKMRRRYYARYTTYYKGGGT
48      TCAYYCCGGYKYYWSWYKWTGTGKRRRYYRYWRAATTACYRKMRRRYYMAYKGYYKAATC
        :  **   .**  *.  ** .  * * * *  *..    *********  *. ***..

44      YWRYRKYKWYKKCWGYGYMRGSRKARWSRYATCTCGSMYSYYSAAKYYYAKYKKCYMYSR
48      YWRYRGYKWYKKAWTYKYMARSRKWRWSRYMYYKASSMYSYYSRRKYYTCKYKKGTATCR
        ***** ******.* * **  *** *****   ...*******  *** .****    .*

44      AYWWYMYCRRKKYWMYYCSAKTATTRRTGCTGAGKRKMGYWGGGTRRKYRACGARYGTYT
48      TCWWYMCGRRTKYWCCTYGRKYRKYGRYAYCTCTGRKMKYAKCTCRRKYRGAAWAYKYCW
        : ****  **.***    . *  .  * .   .  *** *     *****...  *

44      YYRYWGCAAYGKWMYKRKRRGRKKYKYKMMMKYKRRGTRYRATAGKKMKYWTGCTWAGCG
48      TTGYWRTCGCTGAMYKGKRRKRKKYKYKMMMKYGRRKAACAWWMKKKMKCWYAGWAGKAT
           **  ..    *** *** ************ ** :       **** * .   . .

44      CWMMKKKMKKWYYCMRYYRAGYWYRRKAYRSRRYRKRGKMTMGRAKYYWKRRRWKKKWYA
48      MWMMKKKMKKWYYMCAYYRGACWYRRKMYRSRRYRKRRKACMARWKYYWKRRRWKKKTTW
         ************   ***.. ***** ********* *  *.* ************

44      AYKWYMARKRYAGAMYKRYYSYYYRKSYKYCGMMWRKKTRTWTYRKKSKMMRKAYYKRRT
48      CCKTTATRKRYRRMCYKRYYSYYYRKSCKYTAMMWRTKAAYTYYRKKSKMMRKRYYKRGA
        .  *   :****    ************ ** .****.*:    ********** **** :

44      AYKYKRYYKCYRYMKYCGMTRGTRTKGKGCGGKKKCTWRKYCYMWMMGTYKKYTYRMKWW
48      GYKYKRYYKMYATATTARMCGTCACTAKRAATKKKAGWRKYSYMWMARYYKKTWYRAKWW
        .******** *   . . *      ..* .. ***. ****.****   ***  ** ***

44      TKYYKKGGAGTATCYGRKYTARACCTCAMCRRGGRCRCKSKTKYSWYACYRGMMYTRRAC
48      YKCTGTRSRKYWKYYRRKYYCGRTTAACMYAARRAYRMKSKKKYSWYRTTRKMMCCGGMT
         *   . .    . * *** .    :..*       * ***.*****   * **

44      GYMKYKRYCCTCRYWRCYKRKCCGMRMRGKWSRKKRKYRYKMRMCAKWYYYGYRSSYMRM
48      AYMKYKGTTACTGCAATYKRKMMRMRMGKKWSRTGRKYRYKMRMGMKWYTYKYRSSYCGM
        .*****   .       ****   ***  ****. *********  *** * *****  *

44      GARRWKCYWRRKKRCYCATKTGTRYYKGTGKRRSYYYWMMYKRYKKKMYRYYKRATKMKY
48      KRRRWKYCTRGKKRTCYGCKYTARYYGRWRGRRSYYCWMMTKRYKKKMYRYYKRCAKMKY
          ****   * ***   . *  :***     ***** *** *************.:****

44      YMYCCKWRYRKKKRYKKYKKWRRKRGKKYKSWK
48      YMYYYKWRYRKKKRTKKYKKWRRGGKKKYKSWK
        ***  ********* ********   *******
```

The pairwise alignments of two accessions 44 and 49

```
44          ATKMMYYGCAKMYCYKGCRMRYCYRMMYRTMWWRRWYYYMWRYYYYCGGRKYMMYYKYYR
49          RYTMMYYATWKMCTCTRMRMRTAYRMMYRAMWWRRWYYCMWRTYYYMRRRKYMMYCKYYR
              .****.  **   .  *** .******:******** *** ***   ****** ****

44          YRYKYRRRYMRWTTSCRGKSYKYKRWWWWMRYWKKKRMRGYKWYRYMMTYYYYMRYWMKW
49          YATGCARRYMATKCSYGAKGTKYKRWWWWMRYWKKKRMRRYKWYRYAMWCCTTMRYWMKW
            *     ****  . *  .*. ****************** ****** *     *******

44          MYSMYWRRCMMYRYAKKKYYYYYRCTRYWYAWRRWYYRYYKKRYRWYMKYYWYRWYYTCK
49          MYSMYWRRTMMYRYCKKKYYYYYRTARYWYWWRRWYYRYYKKRYRWYMKYCWYRWTCCGK
            ******** *****.********* :**** ******************* ****    *

44          WSWKYKYYRRSYMRYGYSKGYYMWYRCGTASACWAWKSYMWGGCAWRGRWKKGYKYWYRY
49          WSWKYGYYRRSYMRYKCGTRYYMWYRYKYTSMYAWWKSYMWRATGTRAAAGKRYKTWYGY
            ***** *********  .. ******   :*    ****** . . *.   * ** ** *

44          YMAYYYRKMMWKYWWRKMMYTMWWRYRYKSRMRYYYMYSYWRKYRWWMTTYSRRWTTYCG
49          TMRYTYAGAAWKYWWRKCMYYMWWRYGYKSRMRYYYAYSYWRKYRWWMCGYSRRWCCCTK
             * * *    ******* ** ***** ********* ***********  *****

44          AYYMAYRRYYKRTCMYRYTYYWKRYGCYYYYCRRRTRMSWRRWMSRRKWYGCYWRYKMYC
49          RYYMWYRRTTTRCAMYRCGTYWKRYTTTTYYTRRRWRMSWRRWMSRRKWCRYYWRYKMYY
             *** ***  .* .***   *****    ** *** *************   *******

44          TRKWAYMYYWWMGTYMKSMRWYRYKGGYYMYYYWYATCAKKACRRKGCYWKRSRKRMMRK
49          WRKWMYMYYWWMRYCMKSMRWYATTRKYYMYYYWYRCYWKKGAGRKASTAGRSRKRMMRG
             *** *******   *******  .  ********    **.. **..   ********

44          KKYYWYCTTTRKWYRGKCMWGGYGCAYRYMWYMGCWMMKARYMWKTAKWKYAYTRGCRYG
49          KKYYWYYCAARKWYRKGYMARRYTTCCRYMWYMAMWMMGTAYCTGYRKTKYWTCRTMRYA
            ******  ::*****   *   *  . ******. *** : *     * **   *  **.

44          CCRSGTYGWRKYRSTKKRGTTTKGCAYCAYRTTTMRMTYRCCAKKKTRYKACGYWWWMYK
49          ATAGACYKWATYRSKKKRKKYWKKAGCTGTGAGCCGCGYRMYWKKGARYKGYAYWTTMCK
            .  .. * * .***.*** .  * ..  .  :      **   ** :***. .**  * *

44          KAAMCCCCYRWKYYKRKKRTYRMYKTRTGGGRRARRSGKGCTRCYKYRRGYMTAAWRCCW
49          KCTCGTTTYRAGYYKGGKRGYRMCGKAWRAARARRASTKTYWATYKYGARTCKRGTATYW
            *.:     **  ***  ** ***  .   ..*  * * *     ***     . .    *

44          YSWTRKKMRWGKRSYYTGRYAARWYYYTAAAYRRYATKRRKKYCGTRKKWGKRMYCKRWK
49          YSWWRKKMRTKKRSCYARGYGGAWYYYKWRWYGRYTCKRAKKYTKYRKKWRKRCCAKRWK
            *** *****  *** *:  *.. ****.   * **: ** ***   **** **  .****

44          KMKMRKMRRWTYGAAGCKAYYWCKYTWCCYMRCKSKGCTCAAMYKRCYKYKKKRYYAYRY
49          KMKCRKMRRWYTATCTYGMYYWTGYYTYAYMGTKSKAAYYWRMYKRSYKYKKKRYYGTRY
            *** ******  .:.    ***  *   .**  ***..    ****.*********. **
```

```
44      RMTRYKYYMTGKKWWRRKYWMKYMKYRYTCTKWWKCGKWKCGYRYTGACTARKKWAYSWM
49      ACAGCKYYMYKKKTWGRKYAMTYMGYRTCAGGWWKARGWKATTACCRCTCGATKACYCTM
          :  ****  ** * *** *.** **  .  ***.  **.      .  . .* .*. *

44      WYYARYRYMYKYRMAKMGCGWCCKCYCACATACRWWYYKKRTYGYYKMARRAWTCYTSCT
49      WYYCACGYMYTYRMRKMRYRATTGTCTGTCGGARWWYYKKRKYACCKMGAGGACACCCSG
        ***.   ***.*** **          . . ..********.*.  **.  .  .  .. 

44      ARRRRCGMCGGKYSYAKKWYYYRKYGAAKYMWRSYWCTRATTKYCGYRRKWYYRRMKGKM
49      CRRRRTACAACTTCTTKKACYYRKYARMKYMWRSYWMAATAWKCMKYRRKWYYRRMKKKM
        .**** . .. . . :**  *****.  ******** : :: *   *********** **

44      YYRMATMRYYYWRTRATTTATRYCYKWGYTMAACKRATYRYYRKAKMYYKCRRSMCKSRA
49      YYRMCCMRYYCWRYGWYYCCCAYTYKASYCATCAKRCAYRYYRKMKCYYKTRRGAATSRM
        ****. **** **      .  * ** .*  :..**.:****** * *** **. ..** 

44      WKYGRTCYCGGTKKTKCAYTTYKTTCGRWRWGGAGKKRRRMKMCRKYRKGRYKKYRKYMG
49      TGCKRCYTAKKYKKYKATCAYYGYYMKRWRWCRWRKKRRRMKMYRKYRKRRYGKYRKYMR
            *   .   ** *.: : *     ****    ******** ***** ** ******

44      AGRKKKRKKKRYMKWYRRKWRCGYGMMWYRYKYMMTAGTGTGKWSYRRYRATAKKAMGKK
49      MKRKGKGKGGRYMKTYRRKWAYRTAAMWYRYKYMMKTACKKRKWSCRRYRGGTKGTAAGK
          ** * *  **** *****    . *********.:.  . *** ****. :* : . *

44      KKWCKRMRTMTAYKRYKKACWRRYRYYKKYRMYSRRRYRWYRYKKKRRKYKTRAAAWKKK
49      KKWYKRMRWAAGYKRYKKWYWGATRYYKKYRMYSRAATGWYRYKKKRRKYKYRMMWAKKK
        *** ****  :.******  *   ***********    ************ *    ***

44      GKTYKYTTKKGKKRRMAARWRYRTATYYRAMRWKCKRYMGCRAYWYRRYKAKKKRKYMMW
49      RKYYGYAGKKKKGRRMMRRWRYRKGATTAGCGTKYGGCAKYRMYWYRRYKMKKKRTYMMW
         * * *: ** * ***  *****..:   .   *       * ******* ****.****

44      TCCGKTKKYKKAKGKYCRCYKYTAAMWAGRRWCCKMRRRKYRGCCYAYCAGYRRKYTCCC
49      KMMKKCKKYKKRTKKYAASYTYKGRCARKRRATAKCAGATCRATTYRCTRATGGGYYTTA
        .   * ***** . **. .*.*..     **  .*    . *.  *    .    *   .

44      CRWYYKGKTYMRKSKYGMWKKCGAYMWMKYMKCGGCGYYTKGTTYWRRAGTYRRRRKTRK
49      TRWYYGRKKTMRKGTYRCWKKYTGYMWMTCCGGAATCYYKKSKWTWRRGSAYRRRGKKRK
         ****  *. ***..*  ***  .****.    ..  **.*..  ***..:**** *.**

44      RYKKKMKKAAGYWAKMYRKWRYKSRYMRRKWWYGWYYKRKKWYRRTYAMWSKYRRKKWKY
49      RYKKKMKKTRTYWRKMYRKWRYKSRYMRRKWWYRWYYKGKKWYRRAYGMWSKYRRKKWKC
        ********:  ** ******************* **** ******:*.*********** 

44      YRKRCYKKWRRWSWRYYKRWWYKRKYKYWYCTRMRRYYKTAYGCKYRKRYKKYYKGCTRC
49      YAGGYYGKWRRWSWRYYKRWWYKRKYKTWYYYRMRRYYTARCTTGYRTRYKKYTKRTCAT
        *    * ******************** **  ******.:     **.***** *     

44      RMWWMYYKRKMRKMTWTKCKTRTAYRYYAATCYMKMWKACKAGYYYRSSKMWYAAKARAC
49      AMATCCYKRKMRKMCWATYKKRYMYRYCWCYYYCKMWKRYKWRYYYRSSKMWTGTKTRTT
         *    ******** *:. *.*  ***  .  * ****  *  ********* .:*:*: 

44      RWMKRYYYKKRSYYRYWRMARCCMYMRYKRYKGRTTGYRKCGATTRTCTGRWTMMTCYMC
```

```
49          RWMKRYYYKKRSYYRYWRMMRYYMCMGTKRCKRRCCTYRKYRWYCGWYWTRWWMMWYYMA
            ******************* *  * *  ** * *   ***          ** **  **.

44          GGCWTTWKKKRAKYYYKYMKKKKKKYKYWKCMRMGACGCCCKKWYYWGCGTGYMGWATAA
49          RCMAGYWKKKRMKYYYKYMKKKKKTYKTWKYMRMRWMKMTAKKWTTWATACAYMSAGCGW
                  ***** ************.** ** ***      .***  *. . .**. . .

44          GGMCKKYYRKKKYYYMKYMYKCGRRYKRRRYWAWTMSGMSGMMKKGKRRGRKRKYMRTTG
49          KKMYTKYYRKKKYYTCKYMYKAARGCKRRRYWWWWMSKMSRAMKTKKRRKRKRKYCAWWR
              * .*********  *****..*  ****** * ** **  **. *** *****

44          KSTKWRWWAGAYRYKRSMKKGAGRRMKYGTCCKKKWYKTRYWMRRYRTATCWRTYGTTAT
49          TSYGWRWWCAGCACKRSMKKKWCARCGYKAGTKKKWYKWRYTCGGYRCTCGWRYCAWGTA
            .*  ****...   ******    *  * :  ****** **    ** :  **  .  ::

44          AKRCYRKYYMAYKSYMYAATKKKWKGCTGKRYGGCKRMTCWTTCTTAWYYKKAYRYYKRW
49          MKRYYRTYCCMYKSYMYWWYKKKWKKTATKRYAATKRMKYTGGTYCRTYYKKRCATTKRT
             ** **.*   ******   *****  : ***.. ***.         ****     **

44          ARKMRTKKWRYRTWTAGTRKKWRMMKGATCYCMSCGCYCYWKRTACYWRWWYKKYCRGKK
49          CGTCRCKKWGYRYWATRYRKKWRCCKRTWTYSASTTACYYWKGCTMCWGWWYGKYMRAKK
            . . * *** ** *::  *****  * :  *. *  .  ***  :  * *** ** *.**

44          SRWKWWYCYRKRTYYKAKKKWAAYRKKTKKYKRCCMYRRRRKMMWKRTYYSKAKKKRCRM
49          SRWKWWYYYRKRCYYKMKKKWCWTRKKKKKYKRATMYRRRRKMMWKRKYYSKMGKKRTAM
            ******* **** *** ****.  ***.*****. ************.****  ***  *

44          YKSSMTYYAYRRRRYYKYKYRTWKWWWMYYRCCTYCMRYWYCKSKRKYYWYMMCAMTTWK
49          YGSSMACCTYRRRRYYKTKYRKWKWTTCCTRYSWYYMRYTCMKSKRKYYWYMMYMMAGWK
            * ***:  :******** ***.***     * . * ***   ***********  *: **

44          TYKGGTRWKYKKRWKMRRYTAGYGGKRWKWMKCGWKKTMRYYYYKKGGTAMYWTKACTRW
49          AYKAACAWKYKGRWKMRRCGTKTRTKRWKWAKMKAKKWMRYYYYKKRKYCAYWKKRSYRT
            :**..  **** ******  :    ***** *   ** ********   . **.* . *

44          GCSMWKWRKMRRAKYWRMYYRYRYATCRWGGTKGCWGYTCTCRYRGSRCGRGGYWSCCYG
49          RYGMWKARKMRRGTCTRMYYRYRYGCARWTAYKKATTYATCYRTRKSRMSAAAYWSYAYA
              .*** *****..  ********. .** . * .  *:   * * ** . ..*** .*.

44          CWYYKAGSTTKYYCATMTCRWYKYYYMYTMKYGYYYSYAYWATTTTTKWYWRKCTAGGGC
49          TATTGRSGGYKYYYWKMYYRWYKCYYMYGMKCAYYYSYRYWRWYYKYKWYWRKYYWAAAT
                  ..  ***  .*  **** **** ** .***** **    . ******   ...

44          TKKRKTWTGTWCCRMRATRYATCYYYYYRKKKWCAAARGACAAYKWWKRRWTGWARKMKR
49          GKKRKYAYSYAYSGCGRCRCMCMYYTTYRKKKTYCGGARGTCTYKWWKRRTWKTWRKMKR
             ****   .   .     *    **  *****  ...  . .:*******     *****

44          WKWRYMKCRKTTYSYRKGMKCRGRMTRSTTTGTSYKRKSAWYRRRKWYSKRMWKKKYMKK
49          WKWRYAKYRKCCYSYRKAMKMGRRMKRSGGCAKSYKRKSWWYRRRKTYSKRMWKKKYMKG
            ***** * **  *****.**   **.**   ..****** ****** ************

44          KMARSRKYMKKGRYYMKSYRCMRKYYRWSKACCGKGYYRRYYWKMCGGCTWGTKKYCKWS
49          KMGRSRKYMKKRRYYMKSYRMMRKCYRTSKMMARKAYYRRYYWKMYRKMWAAKKKYTGAS
```

```
                    **.******** ******** *** ** **  . *.*********      ..***   *

44                  GRWMYKYKKYYYWTGYRSKRMYWKKRRKRKCKKMMMYRYYYMSWCKCWATCGKAGYACYA
49                  KRWMYKYKKTYYWCTYRSKRMYWKKRRKRKYKKMMMYRYYYMSAYGMWWWTTKMKYMYYM
                     ******** ***  *************** ************    *    *  *  *

44                  KYRKYWGRRKMKYWRRRYYKWMMYKKWWMKYKWYYRTWGKCYYRMGYWYRAWRRKRRGTA
49                  KCGGYWKRRTMKYWRRRYCKWMMYKKWWMKYKWYYGYWRKTYYRCRCWYRMWRRKRRCYW
                    *   ** **.******** ***************   * * ***   *** ******

44                  AYRMKYMGWRRMAKRCAKRYYWRRRKRTYRKKRTYKRYGKMYKRKYSTTCYYYRKKSYMK
49                  CYRMKCAAWRRARKRYRGRYYWRRRKGYCGKKRWTKRYRKCYKRKYSAYYYYYRGKSYMK
                    .****  .***  **   ********    ***  *** * ******:  **** *****

44                  YMKGYCCARYKCCKKCCATYKGACRYYMYRKYYKYYGAAWMRRYKMWSKRCCARAGRKYT
49                  YMKRTTAGRYKMMKKAATAYKRRMRYYMYRKYYKYCAGRWMRRYKMWSKRYACRCARGTC
                    ***   ..***  **..::**   *********** ..  *********** ..*..*

44                  CGGCCGWRGTYAGRKMKRRTRRWKSRKMKKRRTSKRKKKKKYKTKKKWYKCCRAGKMYYY
49                  YTATYAWRACYGARKMKRACRRWKSRKMGKRRCSKRKKKKKYKCKKKWYKYTRWAKMYYY
                      .  .**. *..*****  ******** *** ********** ******  * .*****

44                  WRRTGTRYRSKYRRYWKYKKKSRMRGYYRGRSKCKKWGKKWYRTWWYYMYKRMAARYYAC
49                  WRRGAWRYRSKYRRYWKYKKKSRMRCYCRSRSTMKGWKKKWYRYWWYYMYKGMWGGYYGT
                    *** . ******************* * *.**. * * ***** ******* * . **.

44                  KATYARYKKKYWAGWCWGRTTAKWWYYYYWYSYAWRMMWYYWGCCRTMMKYKKMGGACCG
49                  KMWCWRYKKKYWMAWYWTRWACTAAYYYTAYSTMWGMMWCYWKSYRYMMGCGKMRRGYTA
                    *    ******* .* * * :..  ***  **  * *** ** . * **   **  .  .

44                  WAYYGAYKTTMRKCKYYWKYKMYRMYKRTYYTYRRKRMTRYMYTKATRRWKRGRMRKKKK
49                  WRYYSWYKWKAGKYGYYWKYKMYRMYKGCYCWYRGKRMCGTMCCKRYRRWKRKRMRGKKK
                    * **. ** .  *  ************  *  ** ***   *  *  ***** *** ***

44                  KCYRCWMRYRYWRCWAKCYYWRKKYYMKKYYKKRMCCGGCKAWAKCWMYAARTATRKKKY
49                  GYCRYACRYRYWRMTMGMYCWRKKYYMKKYCKKRMMTATTKGWTGATMYRGGCRKRKKKC
                       *   ******     * ********** ****  .  *.*: . **  .   .****

44                  RKKRWAAMKRMYRRYYKGYWATTYWYRRAAARRWMMYACTKCTCSYWSRKGYKMSWKWYY
49                  GKKRWGWMKRMYRRYYKRCWMKKYWYRRRWRRRTMCYRMYKMKYGTASRKRYKMSWKWYY
                     ****. **********  * ..*****   ** * *   * . .  *** *********

44                  WAKKRTGTWRYKYRWKKYTKYYRRRTKMMMYMGMMKKKMYMWCCKCYKKSGRRRKKKWKG
49                  TMKKRGRCWRYKYRWKTCKKYYRRRWKMMMYMKMMKKKMYMTTAKYYKKSKRGGKKKWKK
                      ***   ********. .****** ****** ********  .* **** *  *****

44                  KYKYRKKCAAGYKTGAGGTYCTCTKCAAKWMYRYCKWYKRSRYYRWRRRKGYKKKCKYWW
49                  KYKYRKTYRMRYKYKRRTAYAGYKKSWRKWMYRYMKWYKAGGYCATRRRKACKKKATYWW
                    ******.    **     :*.  .*.  ****** **** . *   ****. ***..***

44                  KYYGKKKARKGGSKRKYYKYYRKMKKKRRRRYMYMKYWWSAKYTAGGRYKYKYKRRRKKC
49                  KYYKKKKMRKRRSGRKYYKYYRKMKKKGGRRYMYMKYWWSTKYYRKRRYKYKYKGRRKKA
                    *** *** **  * *************  ***********:**    ******* ****.
```

```
44    GTKAKCRYKRACAAMYGRMKGTYRRRCYKGYKKKYGGGGRYKKYRCKKKKAAYGRCWRYK
49    RWKMTTGTTARYGCMYKRMKAACRRRACKRCGKKYKRRRRTGTYRSKKKKTWCRGYWRYK
        * .   .   ..** ***.: ***. *   ***    *  .**.****:     ****

44    YSYWYTYKARCKRMKTCYRYYYYKTGTTAKYMGWKMRWRKACGWKKKKYYMKYRGKGKYR
49    CSCAYCYKCAMKRMKCGYRYYYYKWRWYWKYMRAKMRWRKGTRWKKKKCCCKYGRTKKYR
       *  * **.  ****  *******     ***  ******.  *****   **  . ***

44    RKYRKKTGTTAATRWRCCKCKAKKKKYYSKARYAKAATGKCACGGTGYKKYMKTAKKMYY
49    RKYRKKACCCWMYRWGTTKYKMKKTKYYSKRRYRTRTCAKYRYRRWRYKKYCKGCKKMYY
      ******:      **   * * **.***** ** . : .*       **** * .*****

44    WMYRSRKMKKRRYYRYKYYSWACYRTCAGATRYRGTRRYCSTAYMWTKTARGMTWKMGWK
49    WMYRSRKMKKRRYYRYKYYSWTAYRGYGKGYRYRKWGGCYSKRYATYGGMAKCYWKMKWK
      *********************:.**  . . ***      *. *          *** **

44    YRAYYRYGKKKCCTYCYRKYYGATAYCRSMKTYARCGTGCSRMKYTRRYYMYYRKRKRWA
49    YRTYCGYRKKKYYCYTYRKYYAGGRCTGSMKATRAASARTSGMGTCRRYYAYYRKRKRAG
      **:*  * ***   * *****..     ***:   ..:  * *   **** ******* .

44    RTTTCACGAYGCATTTMAMRRWCKWGKRMAYAKKKSKYKKRYKMYCYKYYKCRRMYRCCG
49    AYWKSRYRRTRYGGCCAWMRRWTKWKKRATCTKKKSKYKKRYKMYMYKYYKYGGMYRAYA
         ..       .     **** ** ** : :************* *****   ***. .

44    KYKKKKKYRYKYRRYYKYRRRRMRRAKAWKKSYWYMKYKYYYRYMSKKCCTCWMKCRGYK
49    KYKKKKKYAYKYRRYYKYRRRRMRAGKMWKGSYWYMKYKYYYRYMSKKTACYWMKYGRYK
      ******** *************** .* ** ***************** .  ***   **

44    YRRKRKKGYYWYAAGCKRYYRWKAGCAMMKKKSKRASRWRYYARRMRTACGRKKSRYCAW
49    YRRKRKKRCCWYTCATKRYYAWKGAACMCKKKSKRWGRWRYYMAGMGYWTARKGSRYMMW
      *******   **:.. **** **....* ****** .*****   *    .** ***  *

44    AGYKRYRYTKKYKKYCYRRKKKKYYYWKMYMYKRRMKKAGAKKTYTTTKGRRWYYWGKCR
49    GACGRYRTYKGTKKYATAGKKKTYYYTKMYMYKRGMKKTTGKKGYYYYKKGATTTTATAA
      ..  ***  *  ***.   ***.*** ******* ***: .** *   *       ...

44    ARCAAAAKKYYAKMYRKWTRTMYRKWCGCSWAKKKAKRKAKCKRRRKCGCARYKTYACMM
49    CRYGGCGTGCTRGMCRKWCRCMYRKTYKMSWWKKKRKRKWGTKRGRKAAAGRYKYYCAMM
      .* .....     * *** * ****    ** *** ***   ** **....*** *..**

44    RRKKRKRYRMYAAGGWKWRYKRYYTGRYKKYMYMRYRKYTTT
49    RRKKRKRTGMYTTAAWKWATKRYYCKRYKGYMYMRYRKYGCC
      *******  **::..***  ****  *** *********
```

The pairwise alignments of two accessions 44 and 50

```
44                  KYMRYYAMYMGKYRRWKYMKGRKYKMMKYGKCCWMMGRRKGTYGRMWRCCCAYRRTKGGM
50                  KYMRYYTMYMRKYGRWKCCTARKYTMMKYRKSTWMMRRRKRWYTRMWRTAGWYRRYKKRM
                    ******:*** ** ***  ..***.**** *. *** ***  * **** .  *** *  *

44                  RWMKTATMMMYWRRRRWTTTMMSGKRYYYYYYRRRRWWKYYYYKYRKGMMTWRYWKRKYR
50                  RWMKYWYMMMYWRRRRWYYWMACTKRYYYYYYRGARWWKYYYYKYRGSAAYTRYWKRKYR
                    ****   **********   * . *********  *********** .    ********

44                  KYYYYWKRRTRWWMSYMYRRKTSRRYCGKTRMKMMYSRWKKYYGRWMMWYYAGYRSWMKY
50                  KYTTYWKRGKRWWMSYMYRRKWSRRTYRTKRMKMMYSRTKKYCARWMMWYCGAYRSWMKY
                    **  **** .*********** ***   ..******** *** .****** ..*******

44                  YYKRKYRYRSAYRWKWYKYRRGTCWRMWYWMRKKRMTTYYYYRCAKMRRKWYRRCYMASY
50                  YYKRKYACAGWCAWKAYKYRRAAAWRMACWMRKTACWYYYYYGYGGMRRKWYRRTYMRGY
                    ******   .   ** *****.:.***  ****.    ****  . ******** ** .*

44                  WCCTWKRWCYRWYRWMYYRSKRAYWYMYWWRKYYRMYAMYWYWWKRWYYKMGAKSRYYYY
50                  WYYWWKRWMYRWYGTAYYRSTAGYTYMYWWRKYYRMYWMYWYWWKRWTTKMRMKCRCTTC
                    *   **** ****   ****. .* ************ *********  **  *.*

44                  CGTCYYRRYKYYWKAYWKCTWRYYKSCGMYYRRRGGYKWKSGRYRYRYYYRGKWYYRGRK
50                  TRAMTTRRYKYYWKGYWKMYWRYYKSMSMYYRRRKRCKWKSTRYRYRYYYRRTAYYRKAK
                      :   ********.***  ****** .******   **** ********* . ***  *

44                  RYYKYYKRCTTAWYRWYWKYRRRRYWCCRRKRKKMAKYMYRKKYACCTTGCCGKKMRYKY
50                  RYYKYYKRMWKMWYRWYAGYRRRRYWMMRRKRKKMWKYMYRKKTRTAAYRMAKKKAGYKY
                    ********  . *****  *******  ******* *******   .:   . **  ***

44                  KKMWRAGCGCARYKWGSYWRYGMYTRCKAKWSRYKYMRRRTSYCGKRKKKYKMCRTKCKA
50                  KKMWRWKSAMGRYKTKSYWRYRMYGGAGGKWSRCKTMRRRASYTRKRKKKYKAYGATTKG
                    *****  .. .***  ***** **  . .**** * ****:**  *******   :. *.

44                  GCKKCKYKKTKWYKRKTSYKMYYYWRRAWWYRKGSKYKKKSRRAAYSYKKMYYYKMGCAW
50                  CYKKYKTKKWKWYKRKYSYTMYYYWGGWTTYRKASKYKKKSGRCRYSYKKMYCCKMSAGW
                      ** * ** ****** **.*****     ***.******* *. *******  **...*

44                  YYMTGGMMYCGCTCCKYKTACTYACRKRKKMKRKRRGCKKYYATCTTGCGYWCTGGMRKA
50                  YYCKRKMCYMSYAAMKYKWRYACGARKRKKMKRKRRTYGKYYTAAYCKAAYWMAAKCRKR
                    ** .  * * . :. ***   : ..***********   ***::.   ..** :.  **

44                  TTYTYTKGTTCCAAKCTKTKRMARGGAMMRCWKYWKTAACMRWMSYKYGCAMGKGTTRYW
50                  AWCACYKRCWYYGRGMGKGGACWRRRRMMRSWKYAKYTRYCAAACYKYRMGAAKKCYAYW
                    :  :  *     .    *     *   ***.*** * :      .***  . .*    **

44                  TSYCGRTGYKKRGTATKWKCTGYATYMRRATYRYGATCWYWWYYMRMRKKWAGGMKYKTT
50                  KSCYKGWRTKKRRGRWKWGYKRTTCYMRRRKYRYAGGYAYWWCTARARKKWGRRMKYKGK
                    .*       ***    **  .  : ****  .***..   ***   * ****.  **** .
```

```
44    AKRMKKAGRARRYYKGWAKKGKYSYWTRATTATKYRKGYYYTRWWMKWRTRWCYRRWWAM
50    CKRMKKRRRCGGYYKKTTKKKKYSYWWAWAWMWKYRGKYYYKRWWMKWRCRAMYRGAARA
      .*****  *.  ***  :** *****   :   ***  ***.******* *  **

44    RKKYKWCARWKWKYKRYTRWYMKKMYRYMRYKKYRWKRRKMMKRYYYSSYKKCKMYMSAA
50    AKKYKWYWAAKWKYGRYCRTYMKKMYRYMRYKKYRWKRRKMMKAYYYSSCKKMGMYMSRW
       *****    **** ** * *********************** ***** **  ****

44    KSSMKTTYYAKRRYYKYKRWRYMRKRGTKYRKYYWMYMMYKKYWKYKRWKKCCWMYMACK
50    GSSMKGACCTKRRYYKYKRWRYARKRTCKYRKYYWMYMMYKKCWKYKAAKKTTWMYMRYK
       **** :  :************ ***  ************** ****  **  ****  *

44    CGKKWMRYYYYKKKYAAAKWCAATYRRAWKKAWKRRKMKYTACCYWMTKKKKSRYYGKKY
50    YKKKWMRYYYYKKKYWCTTWSMMWYRRGAKGRWKRRKMKYAGAATWMAKKGKSRYYSGKY
        ************* .:.*.   ***. *  ********:... **:** *****. **

44    CCKRMCACTKKRYTRYSWYRKYYCARYKKYKKMGWKRKTWKKCSCGKKWGYYKRYWWKKK
50    YGKRMYMYKTKRYWGYSAYRKYYMWRYKKYKKMKWKRKGWGKMCMKKKWKTTKRYTWKGG
        ***   ..***  ** *****  ******** **** * *  . ***   *** **

44    YYKYRRTKKKRMRWRWKRKAYTKTYYSRYTCYYRGAKTCYRYSWWKGYKMYYMWYKYKYR
50    TTKYRRKKKKAMRWRWKRKWYGKGYYSRYYYTTGARKYAYRYSWWKRYKMYYMWYKYKYR
        ****.*** ******** * * *****     . * .******* *************

44    YKKYTAWCCGYWKRYMMKKYRMRYMWWYKCWYRYARGYYKYRKRRWSRYRRKRGCRAMWG
50    YKKYGMWYTRYWKRYMMKKYRMRYMWWYTYWYRYRARYYKYRKRRWSRYRGKRRYARCTA
      ****  *   ******************. ****   ************* **     .

44    GGCGGYRWTYRYKWRYYYRRKKWYYRKCACTYARRWYKYRYRWYMRRCGAYKYKRTRYMC
50    ARTARTRWAYGYKWRYYYRRKKWYYRKYGYYCCRRWCGYGYRTTMRRYSCYTYKGYAYMY
      .  .  **:* **************** .   .***  * **  ***  ..*.**   **

44    MRKKYKCYMRSKCCGTTCKRYYGTYRTRYRYRRRWSACCKKTTTWRARMKKYCYRKMMYR
50    ARKKYKAYMGSTATAAYYKRYYKKYRKRYRYRRRWSTTTKKGAYWACAMKGTYYRKAMYR
       *****.** *.. .:  **** .**.*********:  ** : * . **   *** ***

44    KWWGCTTTKAKAWAATCMCRGRKRYRYMMYKAGMKMYYCCTTRYKAARRCTCGKMRKKCM
50    KWWSYCYWTTTGAWMKMMYRRRKATRYMMTKRAMKMCCYTCCGYKTGRRMYTKKMRKKMC
      ***.    .:..   . * * **  **** * .***       **:.**    *****

44    GGKYRYWRCKYYMRKKMYMKKKYRKAKAMKRMYRKRKKRMYRMYRYYRRYMYMRKGKKSW
50    ARKYRYWRMTTYMRKGCYMKKKYRKRKGAKGMYRKRKKGACACYRYYRRYMYMRKRKKSW
      . ****** . ****  ******** *. * *******     ************ ****

44    GKWMYYMWWRYKRRWTGWGRKKWYKMKWKKKTYMARRRWRRKGMMYKKMYMKRKYYTYRR
50    KKWMYYMWWRYKRGTYKWKRKKWYKMKWKKKATATRRRWARKKMMYTKMYMKRKYYGYRG
       ************    * ************:  :**** ** ***.********* **

44    RKCSGRTCWKKKYKKCAAGRKRKYGKTTKRYTKWYKTACACYRYYRWATRYKYYKKKRKT
50    GKYCRRWYWKKKYKTYRMRRKRKYKTYKKATYKWYKYRMRGYGTCATCGRYKYYKKKRKW
       * . *  ******.    ***** . .*   ****     *     . **********

44    TYYKYYRKGMKKATAKRRRRRYRRYMMCTKYWWSYKMCAGAKYKYYMKSYYYGKRCTGKT
```

```
50      KYYKYYRKTMKKRKRKRGGRRYRRYMATCKYWWSYKMMWATKYKYYMKSYYYTTAGGAKA
        .******* *** . **  *******   ********  .:*********** .   .*:

44      CRMSYRRRCYAWWYSRRWRRKRAKWGRSKWKRWYRKKRYGKCGYAAARRKMKRMMWSYKR
50      TGMSYRRRYCMWWYSRRWRRKRWKARRSKWKRWYRKKRYCKSTYRRWAAKMKRMMWSYKR
          ******   *********** *  ************* *. *     ***********

44      YKATTGRYRRRWAWYAYKKKCTYYAYAMRAARRKYMGWKYYMKSRYYGMRYYRYKKYRKK
50      YKCWYSGYRRRWGWYRYKKTACYYRTGCRRGRRKYMKTGYYMKSRYYTMRCYRYKKCRKK
        **.  . *****.** ***.. **  . * .*****   ******** ** ***** ***

44      KRYKKSYKCCTTMYKKKYYWYRRMYKCKRKKWKKARYGGYRYYYTKRMRRWWMWMKRKKW
50      KRYKKSYKYAWCMYKKKYYWYRRMYKGTRKKWTKWRYAAYRYYYAKRMRAATAAAKRKKW
        ******** .  ************** .****.* **..*****:****      *****

44      RKKGYYMMMYGRRMMMYWRKYKRMWKRWTRTTYSYKYCKSRWKYCTARAAATGGYYYKKW
50      RKKRYCMMMYCARMMMYWRKYKRAWKGACRYACSYKYMKSRWKYTCTRWWRYKKYYYKKW
        *** * ****  *********** **   * : **** ******  :*      ******

44      KRGRTYKRKCTAGKMKWKCMWWKKARRYKTYTKWYMTTKWRYWRRKGRKTKYYTGTKAKY
50      KRRAYYKRKYCCRKMKWTMCATGKCRRYKGCYGWYMWYKWRYWRRGARKYKYYCACGGKY
        **   ****  . ****.     *.****    ***  ******* .** *** .  .**

44      YYMAATRRYMKRKKKMGRKMWYYWRKKKRRWYMYKKMMCKYACKWRYRKKRKRYYYKYKK
50      YYMTTARRYMKRKGGMRRKMAYTWRKGKRRWYMYKKMMYKYRTKWRYRKKGKRTYCKYKT
        ***:::*******  * *** * *** *********** **  ******* ** * ***.

44      MKYWMKKKKRWRYRAYMRWRKCYKKKYYRKKGYMMKKGGTCYGATKYYGRGYMAMRCKMT
50      MKYWMKKKKRWRYRRYCGTATMYKKKYYRTKKYMMKGAACYYRRKKYYTGRYMGCRAGCC
        ************** *    . *******.* **** ..  *  .***   **. *.

44      CCCCCWWCYYYTYMRKRKYKKYKKMWMKCGGCYKWRGARTCYMRKRCKKSKYMYWKRKMK
50      TTTATWAYYYYAYMRKRKYKKYKKMWMKGAATYKWRAGGYTYMGGGYKKSKYMYWKRKMK
           . *  ***:**************** .. ****..   **    *************

44      KRRRKWKYMMYRYYYMSRWRYWRRMGRWRYTKKKYKWMMKWMKMYRGKYMMYKWYYYRGG
50      KRRRKWKYMMYRYYTMSRWRYWRRMTAAGTWKKKYKWMMKWMKMYRRKTCMYKWYYYRRR
        ************** **********      *************** *  ********

44      WYRRARKRRRATRMRRRWRMRKKTRYYYGARKRYRRRWKKWYKRCCACTKCTRRYKKRGR
50      WYRRMRKRRRGAAMRRRWAARKGCATYYAMRKRYRRRWKKWYKRATGTCKAARRYKTGRR
        **** *****.: *****  **    **. **************. .  *.:****.  *

44      KKSTYTYYYRKSKYCKMWMTRYKTATTCTTMKRTAAACCRRYYWRGCCRSGRKWRYAAGG
50      TKSACGYYYRKSKYMKAWMCRYKYRGCAYYATGACGTYTRRYYWRAATAGAGTTGCRMRS
        .**:  ******** * ** ***    .   . :..:  ******..  .. .      .

44      ATTAGWRGYGAYRYKCYTCCRKRRYWYTCYRTYYYTTTYKYRKMYTACYYRGRRCGYKYA
50      GWWMATARTRGTRYGYYATYRKRRYWCYTCRGYYYCCKYGYRGCCGGTYYRKGRTTYKYR
        .   .     . **  *:  ******    * ***  .* **    . ***  *  ***

44      RKCTAGWRMSMYYGAMATGKATGKKWYYYTYMYKWKYRRKKMRRYRGMWWMYKRYYCTGG
50      AGACGCWRMSMYYATMCAKKRWKGKAYYCCCACGWKYRRKKMRRYGRAATMYKRCYMYRR
```

```
              . . *******.:*.: *    * **      ***********     **** *

44          RKTYRYTKRSMKGRRCWKKWGKKRKWMRRYYYRRRRYYGTATSTAAMRGCTTYYCSKTGC
50          RKGCACCKRSMKTARYTKKWRKKRKWMRRYYYRRRRYYAGTACGTGMRRMYWCYMCTAKG
            **     *****  *  *** *****************. ::. :.**     * ..:

44          CAYMYKKKKSAAATARYYKRWYKYYYRMRGYKKYYKKGRCMKCCGYKWKKKKYRYKYWKY
50          GGYMYKKKKSTGTGMRYYKRWYKYYTAMRRYKKYYKKKGYMKTTAYKWGKKKYRYKYWKY
             .********:.:  **********  ** *******   **  .*** ***********

44          YRRRRMAAGYYMRKYYKRSYWYKMMYYYRKYWTWCKRYRWTAGWKTCCKKWRTGKRKKKK
50          CAGAAAWWRTCARKTYKRSYWYKMMYYYRKYTYTYKRYRWCCRAKGMYKKWGWAKRKGKK
                        ** ****************    *****  .  *   ***  .*** **

44          KKRGYRKMRRYWKRKKKYYRMKKKRRSRRYYWRATSRYCWYYCGRKWMRRRRMKAGWRRA
50          GKRKYRKMGAYWKRKKKYYRMKKKRRSRRYYTRMYSRYAWTYYKRKWMARRRMKMKAGRR
             ** ****  ********************* *  ***.* *  **** *****    *

44          MYRRMGTWCRYRCYTYKGARYMAKTGWYKCMKWYRRKKMYYWMYRKYRMGCGSWYYWSAC
50          MYRAMRYWYRYRMCWYKKGRYMGGCKACKAMKWYRRKKCTYWMYRKYRMKACCWCYWSMT
            *** *  * ***   ** .***.     *.********  ********* . .* ***

44          RKKSYAMTWGRTYKRYKKTSKRKMYKRRYRWYRYCKTCKCSKYMRKRKKKKKKWRRAGRT
50          RKKGYMMWWRRKYGRYKKACGRKMYKRRYRWYRYTTCYKTSKTMRGRKKKTGKWRRRRGY
            ***.* * * *.* ****:. ************* .  * ** ** ****. ****

44          YCYYACYCGGYGKKYAYGGRYKKCYWKKKGARKYKYYYMWYWMWTACYWRYKACAWKWRG
50          CYYYRYYTRRCRTKYMCKTRYKKYYWKKGRTGGCTYYYMTYWMWYWYYWRCKRYGTKWAA
              **  *     .**    **** ****  :   .**** ****   *** *  . ** .

44          RYTGTMYAGTAWYYCKYKSYRGRKRSRKYAKYARKYWKMMTYYTGSRAGGRYWSSYKTYK
50          RYYRKMYCRATWYYYKYKSYRKRKRSRGYTKYCGKYWTCCYYYASSRWRRRTWSCYKKCG
            **  .**. ::*** ****** ***** *:**. ***.   **:.**   * **.**.

44          AYYMYKMRWGKWYKWMYMWMRTYWSRKGAWAAMYRCKKYKKRRKKKGCYGARRCMMYKGY
50          GYYMYKMRACTWYKWMYMACGCYASRKTGWMMMYRYKKYKGAGKKKKYYSRRRYMMYTTC
            .*******  .*******    * *** .*  *** ****   ***  *. ** ***.

44          AMKYKKKRKKKKRMAGCKAMYKRWYRWWCWKRKKKKKKGKTCCTRRRYRCATKYRRYKRY
50          CCKYKKKRKKKKACTATKWMYTRWYRWWAWKRKKKKKKRGYYYWRRRYRTCCKYRAYKRY
            . **********  :. * **.******.*********      *****  . *** ****

44          MMRKRYRKYTWWWKYKMKKMSMYKK
50          MMRKRYRKYGAWWKYKMKKMSMYKK
            *********  **************
```

The pairwise alignments of two accessions 44 and 51

```
44              KMKMKWWRYRRGGWRCTKWKRCKRGCYWRRWSRYMWSAGAAAMAGCYCRTKRWWYCYWKC
51              KMKCKWWRTRGKKWRYWKWKRYTGKTYWRRAGRYMWGGACGGMGAGCTRYKRWWYYYWKT
                *** **** *   **   **** .   **** .****......*..   * ***** *** 

44              AAKCMRRKMTYYYRYYYKRCAMWCYYKSKYRYMMWMMWWKYRRMRCYMRRWWMKYSRRKR
51              WCKAMRRKMWCCTRTCYGGMTMTYTYKSTYRYMCWMMWWGCRRCRYYMRGTTAKYSRRKR
                 .*.*****    *  *   :*   ***.**** *****  ** * ***    *******

44              MRKKYRWYAYWCWTYACARYSYARYYYWRKYRYRYWCGYRYRYMRRYWYWWRRRYYKYYR
51              ARKKYRWYGCWYWWCMYTGCCTTRTYYWRKYAYRYATRYRYRYMRRYWYTWRRRYYGYYG
                 *******. * *    :  . :* ****** ***   *********** ****** ** 

44              WCYRMCWWYYYRWSYYKSKWKKCRKMGGRRCAKRKYMRYMRWYMRRKRRTCWKYRYYCTY
51              TYCRMYWWYYTGWSYYKSKWKGYGGMRKRAYMKRKYMRYMRWCMGATGRYSWKYRYCAAC
                   ** ****  *********    *  *   ********** *  . * .***** .: 

44              YTYYRRRYCMRMWSKSYMAAWRYKWSSKWYWRGCSRKGMMTWTRMSAKRWMRMYSRRKWR
51              CKYYRRRYMMRMWSKSYMGGTRYKWSSKWYWRSSGAGSAAYTWRMSCKRWMRMYSRRKWR
                 .****** *********.. ***********...  .     ***.*************

44              AYWMACYGGYRKYTRKRYYWWMYTWGKYKKYYRYYYYYYRKKYYWKTRKMYRKCRMKAAR
51              MYWMWTYKKCAKYWRKAYYWWMYATRKYKTYYRCCTTCTRKKYYWKYAKMYRGMRMKRMR
                 ***  *    ** ** ******:  ***.***      *******  ****  ***  *

44              RKKYKMMKMSMWRRGTYKYRYRWYAYYYMRMYKCRMKGCCGYRYYMKYRWRWRTMRWKGM
51              RKKYKMMKMSMWRGAYYKYRYRWYMCYYMRMYKTRMKATYAYRYYMGYRWRWRCMRWKKM
                ************* .  ********  ******* ***.  .***** ****** **** *

44              RRRYGMGGGKWRMYRRYWRYYTWKRKATMRKKYMYWKKKYAGRMYAMKMKMYGKYRKYKK
51              RRRYSCARRKWRMYRRYWRCYCWKAKGAMRTKYMCTGKKYRRGACRMKAKMYKTCRKYKK
                ****. .  ********** * ** *.:**.***   ***      ** *** . *****

44              KWRRMYRRYKWCYGMYKCTCRKYTTTSWKKRYYWYRCCACTMWWRMKYKRYWACWRKCKM
51              KAARMYRRYKTYYKMYKMKYRGYCCCSWKKRYYACGTYRYCMWWRAKYGRYWRMWRKYKM
                *  *******  * *** . * *   *******        **** ** ***  *** **

44              CGCTAKATKGRGAAYYKYCCYYMRKYTGYRMRYKYYMYMATCTACGKYWWKWMRMCGGGK
51              AAGCRKCGKARATGYYKYYMYCCGGCKRCACRYKYYMYMTGYCRARKCWWKWMRCTAACK
                ..   *. *.*.:.****  *     .    ********:    . * ******  .. *

44              YRYMYYYWYGWWYKTCKYAMMTRKMATAYYYYKRCYKYMYKMACKKKKKSRYYTKRWCGA
51              YRYMYYYWYRWTYKWYKYRMCGRKCRKTYYYYKRTYKYMTKMTMKKKGKSRYYYKRWYKW
                ********* * **  ** *  **  .:****** **** **: *** ***** ***

44              YKMMYYRTTAKKGMYKYKKGYYKTGGCKCGCKYKKKYMRCKKYRYKKRKYKYYYKYRRRR
51              YKMMYTAAGCGKKMYKYKKKYYTKRRYTTAAKYKKKYMGYKTCATKKRKYKYYYKYRRRR
                *****  : . * ****** **..   . ..*******  *.   ***************
```

```
44          GGMWRYKKTTYSYWYKKYKYRYYYYKKYRWKWKWKMRTKYACCRKKRYYKRKMRRTKKRK
51          RRCTRYKKCGYSYWYKKYTTAYYYYKKYRWKAKWKMRGKCGTTRKKRYYKRKMRRYKKRK
                ****  ********.  ********** ***** * .  ************ ****

44          SKYYRMKKRRYYSRMRKWMRYSRYKATAWYYKKKKKGGGGRWYYKTSGAKKMRRMGYYWK
51          SKYYRMKKRRYYGAMGKTMRYSRYKWYMWYYKKKKKKRRRRWYYKGGTMKKCGRMRCCAT
            ************. * * *******   ********    ***** .  **  **    .

44          WRKWATARRYKSYCGAKRMKCGCYYRKACRYKKKKKYMKWKRCGYKWKKTSCCCKCTWRY
51          WRKWRYRRRYKSCTAGKRMKACYYCGGRYRYKKKGKYMKWKRAAYKWKKKCYYMKTATRT
            ****   *****  ..****.  *     ***** *******..*****..   * : *

44          YKKRKYRKKKYYYKYRWYKKRYRTATAATKARWKRYRYKYRYRTAGWGKYMKKKMWSMKY
51          YKKRKYRKKGTTCTYRWYKKRYRCTCGCYKWRWKRYGCKCRYRYRAWAKYAKKKMWSMTY
            *********    .********* : .. * *****  * ***  .*.** *******.*

44          YYYRYKKYCYYRMYKKWYKMKKATGCYRRAKYAYTTKKCCWKRYRRRRGTGMWMKYMRAA
51          CCYRYKKYYYCRMYKKWYKMKKRWKTCGGWKYGCCCTKTTWKRYRRRRKYKATMKYCRCT
              ****** * ***********        **.   .*  ********     *** *.:

44          WKAGRMYWCCKRYRMYRRGARGYCRYRRSMKCCKCWGTKKWKRGCKWRRYYGYMRGRKMK
51          WTMKRCTWTGKRYRAYRRRGRRTGRYRRSMKTAKTACAKKWKRSAGAGACYTYCRTGKMK
            *.  *  *  **** *** .*   ******* .*   :*****..     * * *  ***

44          GTSARMRRGYGGKYYMYMWRGGRWYRTGMATRYWMTRCYYKYRRYKKRYYKKMYRMMKYR
51          AACTGCARRCTTKYYMYCAGKKRWCRYRMWYRYWMKRMCYKCRRYKTACTGGMYRMMKYR
            .:.:   *    *****     ** *  *  ****.*  ** ****.     ********

44          RKMYGMRMKRYRMMKRRRGKCRKKTRKSYCMRKYKKKMSTYRKMYKRRYRWKWMYKKTYK
51          RKCTKMRATRYRMMTRRRAKMRKKWRKGYAMRKYKKKCGWYRKMYKRRYRWKAMYKKYYK
            **   ** .*****.***.* *** **.*.******* . ************ **** **

44          KKKYKRKKKRKYWMMYKYYKKRCCGAYKTCGYKARKYYWGKYRKGGTGKCYYRKWCTACR
51          KKKYKGKKKGGYTMMYKYYKKRMTRGYKYTRTKCGTTTTAKYAKAAARKYCYRKWYACYR
            ***** ***  * *********   .**    *. .   .** *..: *  **** :. *

44          WTRTTRCGTAGCWKYKKKKKRKKCCTKRRTGKKYRCRYKYYTCGYKKYRKMYKYWKMYCY
51          TCGYWAMSYRKMWKYKKKKKRGKYYWKRRCKKKYRYRYKYYYTRCKKYRKCYKYWKMYMC
                   .    ********* *   ***  **** *****    ***** *******

44          YKYCMGCYKMCGCTGCGWYYRKYTTTRYYYYMYYYKRKYKYRYMCYMKRGTAKKATGYWY
51          YKCAACYYKMYATCRMRAYYRKYWYYRYYYYMYYYKRKYKYRYMMYMKAAAGTKRKKYWY
            ** .   *** .      *****   ****************** *** .:..* . ***

44          SYWMRAAKSKRGAKMKCGTMARRGATGGMKYAYKMMYMRRSYMKRKMKTKKKKKYKKYKK
51          SYWMRGCKSKRAWKMKSACMMRGRTCACMKYRCKCCCMRRSYMKRKMGGKKKKKTTKTGG
            *****..****. ***.. * *  : . ***  *   **********  ***** .*

44          KWYKKMYYYWRGAYRATAKKSKRYWKKKKRRKKTATCAKAYMSWYWYRRYGCMYTYGRYR
51          KWYKKMYYYARACYRGCGKKSGRYWKKKKRRKKYRAMRGGYCGWYTYARYAAACAYRRYR
            ********* *..**. .*** ***********  :   .* .** * **..  :* ***

44          SGYAGYYMWACGYRYRARMYRRCCARKMGRWRRRKMYGWCGCCRRKWKYKKCCCYCTYAA
```

```
51          SATGKYTCTTTKTGTRWGCCARMYWGTCKGAGRRKMYATYRYYAATAKCKKTTTYMWYGT
            *. . *   :     *     *    .     *****.       . * **   *  *.:

44          CRKYYYWRKMGYARSTRKYKRCYYKYRWRGWKAKRRMYSKRWRYRYMYWTTCCTCARYKK
51          MGGTCTWRTMAYGRGCRKYKAYCYKYGTRRWKRKRRMYSKRWRYRYMYWAAGAATWRYKK
                  **.*.*.*. ****   ***  * ** ****************:: .:  ****

44          KYTRKGKYTWCRRGKKYKGYWYYAKRRWMRRRKGCKAYWATTWYYYWKRYKKKSYAYMKK
51          KYKRTRTYCTTGATGKYKKYWYYGKRRAMRGAKSMKMYWRKWWYYYWGRYKKKSYGCMKG
            **.*. .*       *** ****.*** **  *. * ** . ***** *******. **

44          YGGTYYTWCAARRCRCKGYRMRKRAGYTRYTKYKMWYWKKKMWATGTTMCYKRKYTRTSA
51          YKSKYYKWTGGRGYASKKYRMRKRMTCARYWKYKMWYWKKTAWTYRYYMYYKRKYKRCGC
            * ..**.* ..*   .* ******   :** *********. *:    * *****.* ..

44          RMMRKMRKWKTRYTRRCATAYRYKMKMMYYYRSSKMWYAWRRGAKKCYAWMGRYYYRRSY
51          RCCGKMGKWKWRCCAGTGCMYRYKMKMMYTYRSSKMWCGTARAGKKTCGWMTRYYYRRSY
            *   ** *** *     .  ********* ******* .  *..**  .** ********

44          YMKRYMCGTGRYYRYTRMCMYTCKKYRWTCGTTSKGKWWRYRKKKRKYKKKWYKKRYSWK
51          YMKGTMTKCAGCYRYYRMTMYWMKKYRWATTCGSKKKWWRYRKKKRKYKKKAYKKRYSWK
            ***  *   .  *** ** **  *****:    ** *************** ********

44          MKYTMYRYMRYKKTKKKYYYWRRKAARWWWMMKRYAYSSMRTCAGKKRMSTKSMYGTYYY
51          MKTWMCAYMRYKKCGGKYYYWRRKCTGTTWMMKRYRTGSCAYYMRGKRMSCGSMYKACCC
            **  *  ******   ********.:   ******  .*       ****  *** :

44          AKYKGWYKYRTWKWMRRYMRRWYKKKYYWMMMWMYKKWKYKRWMKRKYGKRYWMYCRWKR
51          TKYKRTYKYRWWKWMRRYMRRWYKKKYYWMMMWCYTGWKYKAAMGGKYRKRYWMYTAWKG
            :***  **** ********************** *. ****  *  ** ******  **

44          MTMCRACGGKYKMRYYKYYKAKKYSYAKAAYMRYWKAAWTCTRMYAWKGYMYRYWCRCKT
51          CWMMGGYRRKYGMRYYKYYKTTKYSYRKMRYMRYWKRMAYYCRMYRWKKYMYRYWYGYTA
              *  .   ** ********:.**** *  ******      *** ** ******   .:

44          ARSRWSMMKKWKYCGKCGTYKRYMMYSRYKYYWKYYTGGYKGMGSTGRSWWYGGKRYWRM
51          WGSRWSMMKKWKCAAKYRAYKRYMMYSRYKYYWKTYCARYGRMRSYKRGAWYKRKACAGC
              ********** ..*  :*************** * . *  * *  *. **  *

44          RTRACYTKMRACAYMKAGAYWWCAKRMRKYYMKYRMWSRYMKRKKCWYYKRRKKRRMRMW
51          GCRGYCCKMRRYRYMTMCGTTWGGGRMRKYTMKYRMWSRYMKRKGGWYYKRGKKRRMRMW
              *.   ***   **.  .  * . ***** *************  ***** ********

44          SYRRKKKRMWYYTCSYMGWYRKMGWSWRKRKWCACYAKRYARWKKKYYWYMRRYRARMYK
51          SYRGKKKRMWYCKYSYAKWYRKMRWSWRKRKWYGTCCKRYCATKKKYYWYMRRCAGRMYK
            *** ******* . **  ***** ******** .  .***.  **********  .****

44          KGRMRYKKYYSKKKTTKRRWAGTWTARRKRYWYRYGYYYARYATYRRYRYAKKKYYARYK
51          KKRMRYKKCCGKKKWCKRRWRRYWWWRRKRYWYRYAYYYRRYWYYRRYRYRKKKYCRRYK
            * ******  .***  ****   *  *********.*** **  ****** ****  ***

44          RWTKWYRWWYYYGCCMCCGWCAACTWMKMWYYYYWSWTRRMWKCRKYYYRGTAKAACGWG
51          RWGGWYRAAYYYATAMYYRWYTTTAWMKMWYYTYWGTGRRCAKARKCCCAAWGKRWTKWR
```

```
            **  ***  ***. .*   * :: :******* **.  **  *.**    . .*    *

44          RWRKTTGTKCARTARYMYAWAYYKGYKMYWWRSRGKRTRRTTYACYSKRYCKKMRRWMKG
51          GAAGWYRKKYRRATGCMYTWMYYKRYKMYWWRSATKRYGRWYYRMYSKRYMTGAGAACGT
                   .*  *::  **:* *** ********  **  *  *  ***** .

44          TKYCKKKRKARWKKWCWMGCKGWMKYKYWKKRRKRYCTGYKRKRAKTRKTAKKCRYWYKR
51          AKYTKKKRKCRWKKWYWMTTGRWMKYKYWTKRAGRYAGKYKGKRMKYRKCWTKYGTWYKR
            :** *****.***** **    *******.**  **.  ** ** * **  .*   ****

44          KTCAGMKMYMYYMYRRRATRMMKCKYRGTYKYKCWYWAAAGRKYKKWKWWWTRYSYRYYG
51          KATGTMKCYMYYMYRGARYRMMKYKYRRCTGYKYTYWMRRRRKTKKWKWWACRYSTRYYT
            *: . ** *******    **** ***    **  **    ** ******  *** ***

44          YTTKKKWGCKAYRMGGTTKYMYKYYAAYSTAGRTYRYMGCCGKSKKWKKWYYKKYTRATY
51          TKYKKKWRMKCTRATTACGYMYKYYGGTCKMKRYYRYMKMYAKSKKWKKATCGKYAATAY
             . ****  *. *   :  ******.. ..  * ****   .*******    **: ::*

44          RRKKYKRMYRYKKRCRKMKWRAAWYGCTYRRYYMRGYMGKKGAAYWCWYGCYMKKKWTYK
51          RRKGYTRMYRYKKGYGKMKWRRWAYKTACGRTYCGAYMAKKSTCYWYTTKAYMKKKWWYK
            *** *.*******   *****   *  :  * *  .**.**.:.**    .****** **

44          WKKYKKYYWRRRKMMYCMKMYKRWKRKYYYRRRKKKRGWGKAARKCAAGYCCGCWMKTTK
51          WKKYKKYYWRRRKMMYYMKMYGGWKRKTYYAGGKTTGRWKKRGRKYRMRYYYAYTMKCGK
            **************** ****  **** **   *..  * * .**    *  .  **  *

44          RYKYKRYWYKSWTAGTRYYRWTRYKKAYYKACCTMYYYWRKRKYYKYYRCAKMKATAKTA
51          ATKYTRCWYKCWKTTGGTCATKRYKKMYYKCTTWATCTWRGRKYYKYYRYRKMKTCGKAG
              **.* ***.*.:       .**** ***.       ** ********  ***: .*:.

44          TRRWTYYWMGMYWWSRGKAYTRWAGGAGGKKYKYAAKSARRTGYTTAKRTRYCYGTACAC
51          GRRAYTYWMKMYWWSGAKCCCGTGRAGKRKGYKYRGKSWRRYRYCCGKRAGTYYKCTARY
             **   *** ***** .*.    . ..  * *** .** **  *  .**:   *  :.

44          GGMRKTWYASWGARRMRRKKMWGKKWCKMYYYYGRKTYMRTCMYTGCTKTGYYSRAGAYY
51          KSMRKWWCMSWKTRRARAKKATRGKWAKMYYYYRGKWYMGCYCTYTTAKAAYYSRTTCYY
             .*** *  ** :** * **    **.******  * **        :*:.****: .**

44          AYRKRAWTTCYYMKRRRRRKYMMMKYRSYRTTTWAGYACGAKKWKWYYWYYKWMMKKWMK
51          GYRKRTWGCYYCAKRRRRRKCCMMGYRSYAWWKWMKYMYRMKTWKWYTACTKWMMKKWMK
            .****:*   *  *******  ** ****   .*  *    *.****    *********

44          KATKWYGAYCMWRKYYYRYRYRGACCAWYWRKKASRRYRKGKRMRRRWRKKRYRYRRRKK
51          KTAKWYRRCMCWAKYYYRYRYRRTTTRTCWRKKCSRRYRKRKRMRRRWRKGRYRYARRKK
            *::***     * ********* :     ****.****** ********* **** ****

44          RCKKCTACKCTRGMYRYSYYYRYYAMARRWRRRKKKGRWKYKKGYYGRRKKMKRMMSYKK
51          RMKKTYRTKAARRCYRYSYYYRYYWMGRRWRRRKKKRRWKYKGCCTKRRKKMKRMMSYKK
            * **    *.:*  ********** *.********* *****     *************

44          RKWYRYSRMWWWKGTRAGACWAGKCTRRRKWKKACGYTRSMYYKRMRYYRWYKYKYYRKR
51          RKWYRYSRMWWAKTCRGRGMWWRKMWAGGKWKKRMKYYRSMYYKRCGCYRTYKYKYYRKR
            *********** *  *. . *  *     ****   * *******   ** *********
```

```
44              KKMKYSGMKKRWMYRKCKYKYCYYRYKWKK
51              KKMKYSRMKKGTMYRKTGCGCYYYRYKWKG
                ****** ***  ****      *******
```

The pairwise alignments of two accessions 45 and 46

```
45                WGCWKWYGGTYYRWWAKYYYWRKRRMARMKMWWRTYYRCRRRYGWWYKWYWMRRMYSKRK
46                TTYAKWCRAAYYRWWRGTCYWRKRRMGRMKMTWRKTYRSRRRYATWYKWYTAARMYSKGK
                      **  .:*****    *******.**** **. **.****. *****   ***** *

45                RWCWKWCCYRWWATKRSSYWRGYWARMMMRRRCTRRRMRCMMYYRSYRYMRYTYGCYYGT
46                RWTWKWMMTRAARKKRSSYWGRYWWACMMRRRTWRRRMRMAMYYRSYRCMRCAYSYYYTG
                  ** ***   *   .******  **   *****  *****  ******* ** :*. **

45                YMYKYWKWRWWKWRYWRYYKRAYYYYKWMMYRTMRMYRKWGGWRRKRGKWYRSGCKRKWW
46                YMYKYWKWRWWKWRYWRYYKRCYYYYKWMMYRGMRMYRKTTAAGGKRRTWTGCTAGAKWW
                  *********************.********** ******  .   ** .*  . .  ***

45                WYRMYKRMRWRKYRWMYGRWTGYMRRMYCCWCTTGTAYYRYAYKKMKGGKKGRRRYYGYK
46                WYRMYKRMRWRKCRWMYRATYRYCAGAYYTTTCCACCYYRYCTKKMKTAGKARRRYYAYK
                  ************ ****     *    *      . .****. **** . *.*****.**

45                AYWWKYYMAKYMKCYRMRYRRYRKCCATRWGYKKKYSRYRAYWTRTKKRWCTMYYGWMMR
46                WCWWTYYCCKYMKMTRMRYRRYRKTTRKGTKYKKKYSRYRGYWCRKTKRWTGMYYRTMMR
                    **.** .****  *********   .   *********.** *..***  ***  ***

45                KKWKKMRRRYWKYMYWYKWTYMRYCYRCRMYKKGTRYRKYYSMKMWWYSYYKGGCKMYWS
46                KKTGKMGRRYWKYMCWTKTYYMRYTTRARMYKKAARYRKYYSMKMWWYSYTKTRTKMYWS
                  **  ** ******* * *  ****  *.*****.:*************** *   *****

45                YYYYMKYWKKRMYWCMYRRWWTRRYRRYRYTYMKKKYTYYARWKRRRYMYYKAMKKYYRR
46                YYYYCKYWKKRMYWMMYRRWWGRAYRRYRYYYMTKKYYYYWRWGRRRYMYTTMMKKYYRR
                  **** ********* ****** * ****** **.*** ** ** ****** . *******

45                MWYGSYKGYWWRRWYMMKACGRTAWKYRWYCYRKYRYKGRKRSKRYMWGMWKYTYTRYRK
46                MWCRCTKSYWWARTYMMKRMRRWTWKTRWYMCRKYRCKTRKRSKRYMWRCATCCYYRCRK
                  **  . *.*** * ****   * :** ***  **** * *********   .  * * **

45                MGGCRWRYRCTMSCRCGKKYYRAAGKRRCYMGTCTYMRAAAGTCKRRRYMKCYRYYGRCM
46                MRAGGAGTGAKMSMRYKKKYYRTCCKRRYYMKCTGYMRRWRAGTTRRRYMKMYRYYKRYA
                  * .      ..** *  *****:. *** **    ***   .  .****** **** *

45                RYYRARYRKKKAYKYRTRMKYMYGCYGCRYGKYRRTRYKRGWCMKRMKYRKKSWMYYWCW
46                RCCRMRYRKKGCYTCGKRMKYMCAMTRTRYRGCRACRYKRATMCKRMKYRKGSWMYYATA
                  *  * ***** .*.  .*****  .    **   *  ****.   ******* *****

45                AKAAYRRSKCCAKCGTYGGYAACWGTGRWYKRMTYATKYCSMMKKYYYYRRKMCCAYKYY
46                GKWWYGRSKTTGTMTAYRKYWGTTRKKRTYKGCKTGCGCTSMMKKYYYYRRKMSMWYKYY
                  .*  * ***  ..  :*  * .   . * **  . .    *************.  ****

45                MYTGYWTYYKCARWRGCCCAWRMYSKRTSRRKKKTYKKRWSYRYRKKKRMMKKCWRWMRR
46                MYWKCWYYYTTGGARAAGAWWRCYGKRKSRRKKKGTKKRWSYRYRKKKRAMKKAWRWMRR
                  **   * **. .  *.. . ** *.**.******  ************* ***.******
```

```
45          KYKRMRRYKKGYKRSYRYMKRKKYRCRRKGKMRYRATAKACKYYYTWYMMYKYKWKYRMK
46          KYKRMRRYKKRYKRSYRYCGRKGCGMRRKKKMRYRTYWTMYKYCYYWYMMYKYKWKTGMK
            ********** *******  **    *** *****:  .  ** * **********  **

45          MMWKKYWRSGYTYKYKKRKRMYRYTCTKWYKKMKSYWKRKKTAWKRRYRWRCKKYRYKKR
46          MMWTTCWRSTCCYKYKKRKGMYRYYYWKWYKKMKSYWKRKKYMWKRACATRTKKYRYKKR
            ***.. ***   ******* ****   **************  ***    * ********

45          KKYKKKKRKRYRYRKYMRWKYKKKRKMKYWRRRKYRKYKKGCGAGYRCCMWKRKYCKGAT
46          KTYKKKKRKRYRYRTTCRWKYKKKRGMKYWRRRKYRKYKGTYSGATGMMMWKRKYMKAGA
            *.************.  ******** *************   ...    ****** *..:

45          GGAWRRCTYRWSYRKARRCCARGKCRKAAMMKKRRMYWWKGKKYMMRKAKYYKKTGRGKK
46          CRGARRTGYGAGTGGRRRSMWAKTMRKRRAMKKRRMYWWKKKKYMMAKRKYYKKATRKKK
              . **  *  .    **.    . **   ********** ***** * *****: * **

45          YKMYSRTRWWRRKKYWRMRGCKWYWWKKRRRSMKKKWYRKYRKCTTKMMKKKRWRKMRRR
46          CKMYGGCRTAGRTKYWRMRKAKWYWWKKRRRSMKKKWTRKYRKYYWKMMKKKRWRKMRRR
             ***.  *   *.****** .**************** *****   **************

45          GCMSYRRARKYKMMCAKRWWRYTAKARMKYKKYKGAKYMWKMACSRRACTTACACCKRKM
46          KTASYRRRRKYKMMYWKRWWRCWRKWRMKYKKCGRWGCATKMGTGRRGYAYTTRYYKRKM
               **** ******  *****   * ******        **. .**. : :    ****

45          RMKYGGAKRKMWKKKKRKRSWYTKYKMATRYKKTWRRMRRYYYYCATTWMRYGYYRKTTG
46          RMKYKKWKAKMWKKKKRKRSWYWKYKMRYRYKKWTRRMRRCYYYTRGAWMRYRCYGGKKK
            ****   * ************* ****  ****  ***** ***   :****  *  ..

45          YMYRGACTTKTYYTYRKMWGCAGATMWWWWYCCCWRYACGYYRYYKCTATYYWRSYKRWG
46          YMYRAGTKWKKYYYYRKMWKSRKGKMWWWWYYYMWRYGAAYYRCTKAGGATYWRSYKRWA
            ****.. . *.** ***** .  ..******   ***...***  *. .: ********.

45          YRKYRMYRKMRYMRMRMRKAKKYKYYSTTKKKWKKKKMYKRTGCGRKKKKKYMTRRYSAM
46          YRKYGCYGKMRYMRMRMRKCKKCKYYSCGKKKAKKKKMYKRGASRRKKKKKYMYRRYSRM
            ****  * ***********.** ****  *** ******** .. ******** **** *

45          TYMMCCKKRRRAYRKKYKYYTGYWKRYKKCKTCKKWKCYCYYYMMTTTRMMRKRMYAYRR
46          WYMMYMKGRRRTYRKKYKYYCACWKRYKKSKWYKKTKYCAYYYMMCCGRMMRKRMYRYRR
             ***  * ***:******** .  ******.*  ** *  .*****   ******** ***

45          AKMYYKWRYYKKMMRYYKRRRYSWKYRKRKKGRYRCYACARRRRRRYRYYYRYCCRTRYS
46          TKCCCKWRYYKKMMRYYKRRRYSWKCAKRKKTGTRTYMTCRRGGGAYRYTTRYYTGYRYS
            :*   ********************  ****   * *  .**    ***  **    ***

45          KKKRKTMMSRKRAAATYWKKYWGWWKMKMRTRAYRKYKGWRYRRWKRRWRRKYRYYKRKM
46          KKKRKCAMSRKRMMMYYWKKYWCTWKMKMRGRCYRKYKSARYRRWKRRWGRKCGTYKRKC
            *****  *****    ******  ****** *.*****. ********* **   ****

45          RWMMYWMCTYYYRYKYCCTTYMYYWKAYKRRKWRSYGKKATYYYAGTYYSRRTKRYYKAK
46          RWMMYTMSAYYCGYKYYYYWYMYYWKWYKRRKWRSYKKKWGYYYTTCYYSRRKKRYYKWK
            ***** *.:**  ***    ****** ********* **  ***:  *****.***** *

45          MMYWKTTAKYCAKKKAGWYRRWWKGYRGCRKKRYMRYKGYYRKSMRKCMTKKWTKSYWYK
```

```
46      MMYWTACTKYAGKKKRTWYRRWWKRYRSYRGKGCCRYTRYYRKSMRKMMGKKWKKSYWYK
        ****.: :**..***  ******* **. * *   **. ******** * ***.******

45      ATAKYMKTKCYKSKRKKWYKKAAKRYKRKRYYKYWRRRKKKGSRKYKCTTCYWYTRWYYA
46      TAGKYMKYGMCKSKRKTWYKKWWKRYGRKRYYKYWRRRKKGRSRKCKMKWYYWYYAWYYR
        ::.****    *****.****  *** *************  *** * .  ***  ***

45      TTGTMKKYYKRKRRMYRRKRYKMWRRCYKKKCGWSKRYYKKKYMGYYRKRRYRRRGKYYY
46      KYAWMKKYYKRKRRMYRRKRYKMWRAYYKKKATWSKRYCKKKYMKYYRKRRYRRRKKCCC
        . . *********************  ****. ***** ***** ********** *

45      YRYKYWYYYGRCGCAAKYCKKKYSCKKMYKKKGMRYRRGYRWTAGATKYGRKRYMYKYWK
46      YRYKYWYYYAAYRGTRKYYKKKYSYKKMYKKGRMRYRRKYRACCCGKKCARKRYMYKYWK
        *********.    : ** ***** ******  ***** **  . ..* .**********

45      AKMMTSMMSMYKCGYTYKCGYYCTRMRWYRSYTYRYAKKKKAGGTYWKSYYRYRYRRCCG
46      WKMMYSMMSCYKYRTCYKATCYYYRMRWCRSYYTRYRKKKKRRKYYWGSYYGCGCAGSYT
         *** **** **    **.  *  **** ***  ** ****    ** ***      .

45      CCRKKRKWCACGYKRWKYWARWMYRYRSMKMMRYYYARGMWMMWRGKRYRYRYRRGWYRW
46      TARKKRTWYRYTYKRWKYWGRWAYRYRSMKMMRYYYTRAMAMMWRRKRYRYRYRRKWYRW
         .****.*    *******.** *************:*.* **** ********* ****

45      RWKMGYCKRMGTTRAWYWSWKRKGYKYYKMKKSKRKMYYYRRKAAARKCYKKRYYRRYAA
46      RWKMKYTKRAKKWRRWYWSWKRKRYGTCKAKKSKRKMYYYARKWGCGKYYKKRYYRRYWR
        **** * **  . * ******** *   * ********** ** .. * *********

45      RCRAGYKYKMWWYYRYKWAMMWGKKKYRWRCMTRWARAATKRTTYYTYTKTATYMKMKWW
46      RYGWRYKYGATTCYRYKWRMMWKKKKYRWRMMWRTWARWWTGWCTYCCGKYRKYCKAKWW
        *    ***     ***** *** ******* * *      .    *   *  .* * ***

45      KKRMYRRRKKWWSYACAYKGYTAAAGYRRKKYMMWKATKKYSRAWYWWAGCKGSYWSKRT
46      GKRMYRRRKKWWSYTMRYKRCYRRWAYRRKKYMMWKTAKGTSRRWYWWTAGKKSYWSKGC
         *************:  **      .**********::*  ** ****:. * *****

45      TCCRYKYKKYRRMGYKYKKRKKAGAMKKWYMAGAKYKYWYYWKMKKRMYYRYRYKAYYYT
46      YYSGYKCKKYRRMKYKYKGRKKWCTMKKAYMRRGKYKYWTYWKMGKRMYYRYRYKGCCYW
          . ** ****** **** ***  :*** **  .***** **** **********.  *

45      KGRYTKGKKMYAKKKKYYCYMYYKSKRRTAAAKKAMKWYWRCKMYYSRTMRWYRKYRMYM
46      KKRTWKRTKMYRKKKKYYYYMCCKGKRRATTGKKWMKWYWATGMYYSRYMRWYRKYAMYM
        * *  * .*** ****** **  *.***:::.** *****   ***** ******* ***

45      YYRTCTTTRCCMYYYKAKAGCTAAAKTWWKRKTCGGCTRTTTMYRKSRKMGGRYYKMCKY
46      YYRWYWWAATAAYYTKGGCRYCGGTGCWTKAKWTRAYKAAAAMYRKSRKCRRRYYKMYGY
        ***    :  . ** *. .   ..:  * * *   . . :::*******   *****  *

45      WATAYKYRKRTYKKYSTYKMKMGGGAAKYCKYKKKKMRWKKWKMTATGTYRYYYYMYAKM
46      TRYRYGYRKRYYKKYSWCGMKAAAAGRKYYKYKKKKMRWKKWKAKTATKYRYYYYMYRKM
            * **** *****   ** .... ** ************* .:: .******** **

45      MKYYMKMYSRKYRGYKKMRKMKRRRYKGCRKMYKWRMKRYMGWTATYRRRYMYWKWKKWK
46      MTYYMKMYSRKYRKYKKMRKCTRRRYKAMRKMYKARMKRCCTWCTWYRRRYMYWKWKKWK
```

```
        *.*********** ****** .*****. ***** ****   * : **************

45      KYKWYMYRYRAGYWRYRKKCCRTTGKWRTTTYRKGATAGYTGRMYGSRGKYATYGYRTYR
46      KYKWYMCGCACTYWRYRKKYTRGGAKWGYYCCRGKWGGACAAGCYRSRKKCWACRYRWYR
        ******    . *******  *  .**     *    .. :.  * ** *  :  ** **

45      KGYKCARMWRMKAGATKKKWMRGGTKWCYYKYKKKYAYRKWWKKYGTYRATAACYKATKR
46      GRYKYGRMWRMKRRRAKKKWMRRRWKWAYYKYKKKYMYRKWWKKYRCYRMGGCYYKMKKR
          ** .******   :******   **.******** ********  **  .. ** .**

45      RYKKRWWSSSYCYMRWRKWKYTCCCKSKKMYKRRKWKKYSKGWWYTGMYGWGTKATKYYK
46      RYKKRWWSSSTYYMRWGKWKYYYYMKSKKMYKRRTWKGYSKRWWYACACTTKCKMYKYYK
        **********  **** ****    *********.** *** ***:       *  ****

45      KKKKWRKRRWWKRYAWMKSYKYWYYKKRYKYRYRKKKMRKYMYKKTGYRYWGYKRWSRYR
46      KKKKWRKRRWWGRTGWMKSYTYWTTKKRYKYRYRKKKMRGCMTKKYRYRYWRYKRWSRYR
        *********** * .*****.**  **************  * **  **** ********

45      YRYYRRRKKKMKKKKMGRAAWKCCGARRYRYYWYRRYRTRMYSKGWCRRMMRSKSKYRYR
46      YRYYRRRKKKMKKTKMKRGTAGTTTGRRYRYYWYRAYRKRMYSKSWTARMMRSKSKYRYR
        *************.** *.:     .********* **.*****.*  ************

45      RRCKKTGGTGCKRRGRRYKMYYRAYYSGKKWWRKYYRYRRKGSKKATTTGRARWKATYWT
46      RRAKKASTAAYKGGRAACGCYYRRYYSKKKWWRKYYRYRRTKSKKWYYYKRMRWKRGYWG
        **.**:. :. *        *** *** ************. ***     * ***  **

45      AKKCYCRYWKKYYAKKAATWRWMAMYYKMAYYMGYRGCRRKYKAAGAATWKCWWYWRRKK
46      CTKTTTRYWKKYYGKKCCCARWMWMYYKCRCYMAYRRMRRKYKWWSMWYWKTWWYWRRKG
        ..*   *******.**..  *** ****   **.**  *****  .   ** *******

45      YTRMYSWKYMCCARWYWRYAGAYYKYWAGRRRAKKMWMWCYYYWKRASRRYYKRRKMKWY
46      YKGCYSWKYATTTRWYWAYRKRYYKYTWAAAAMKGMWMWMYYYWKRMGARYYKRRKMKWY
        *.  *****   :**** *   ****  .    * **** ****** . ***********

45      RKKRRKWKKMKKCYYKRRKRRRKWMYMRAWGRRYRYMSWGWKKRYYAKWYYGRKRGKRWS
46      RGKRRKWKKMKKMCYKRAKGGRKWAYMRRWRRRTGTCCTCAKGRYCMKWYYRRKRKKRWS
        * **********  *** *  *** *** * **    .   * **  **** *** ****

45      GCCTCSMYYRRRKKKWKKYRKKAKWKKKRYACRYRGYRRKKASARAYKRYWYKRKKCCYC
46      AMAAYSMYYRRRKKKWKKYRKKRKWKKKACMTRYRTTARKKWGRGWTKRYTCGRTKTYYY
        . .: ***************** *****    ***   *** .    ***   *.*  *

45      ATATYYRYRARYKRYTTARGTKKWAGTTCRRWKGAACRMMMKAACMKCCKMKRCCKKYWY
46      GCGGYYRYRGRYKRYAAGRRCKGWWRWWYRRWKRRMYRMMMKGTAMKATKAGRYYKKCWY
        . . *****.*****::.*  * *     ****    *****.:.**. *  *  ** **

45      MMYYRCMRKRKRRYMWKRRMTATMYYRKMWKGRYKYMRYAYTYRKWKWSKKGGARMMKYG
46      MMYYGSARKRKRRYMWTRRMWRAMYYRKMWKKRYKYMRYRYKCGKWKWSKGRSWRMMKYA
        **** . *********.***  :******** ******* *.  ******  . *****.

45      KGTGYKYWYKKGGYRCKTYMRMYYRKACMAGRRRYACGRSYAGTCMTATTRCKRRYYYMK
46      KKCKYKYWYKKATCATGKYMGMYYRKGAMRKARRCCYAGSYWSYYMCCACGYGRRYYYMK
        *   *******.     .** *****..*   ** . . ** .  * .:    *******
```

```
45              KYCGTGGCCGRKKKCCATCYTKKSCYRCKKAACCRYYAGRRYRKRYKYMRKKSRSYWRRR
46              KYYRYRRTYTRKKGYYCWSYWKKSTTATKKRMYYRYCRRRRYRTRYKCCATKSRSYWRRR
                **        ***   . .* ***    **    **   ****.***   .*********

45              YKRMKYCWWMTGCYTYWRMRKWMMRMWTYRRYMYMCSKYMYWYMYTRWRGCKMYAGYYKY
46              YKRMKYMWWMATGYYYWRMRKWMMRMWKCRGYMTCTSKYMYWYMYARWRSMGMYWKYYKY
                ****** ***:  * ************. * **   *********:***.  **  ****

45              KMRGCYKYTMWWMRRYYKARYYRMMYTGTYWSKTTGTKSKCYMKKKYYWRWRMTCTACAY
46              KMRKYYKYKMWWMRRYYKRRYYRMMCYKYYWSKAAACGSKYCMKKKYYWRWRMWYYRYRY
                ***  ***.********* ******    ****::.  **  ***********      *

45              WWRAGKWYSKKYYKKRYYMRKYWARRATSKKWKKKTCTAKSRCMKWYKMKKKMKYYKYWY
46              TTAWRKWYSKKCCKKRYYARKYWGRRRKSKKWKKKYMWGKSRMMKTYGMKKKMKYTKYWY
                     ******  ***** ****.** .*******   .*** ** * ******* ****

45              RYYYGMATMYYYKKKRWKKAYGRWRKYRRKKKRKRYMSKMRKKYGCATAYYATGYTRKWG
46              RTYYRAGCMYYYGKKRWGKWYRRWRKYRRKGKRKRYMSKMRKKYRYRYWTTRWKYKRKWT
                * **  . **** **** * * ******** *************          *.***

45              GGARKRMKKRWYYKMSYRMKMYSGMKYKRYKKYKYTYYKRKWYRRWGGCACYYWCMMYWA
46              AAGAKRMKKRWTTGMSYRMKMYCRMKYKRYKKYTCCYYKRKWYRRWKSYCYYYWMMMYWT
                ... *******   ********. *********.  ********** . . *** ****:

45              ACKWKWKKTMKRYKKKRCCAAKKCYWYYYMYGCCMYWMCAATKKKWKYYKKTAARYRKKY
46              TTTWKWKKYMKRYKKKRYYRRKKYTACCYMYRYSCYTMATCAKKGWKYYKKGTGRYRKKY
                : .***** ********    **     ***  . * *.:.:** ****** :.******

45              RGRYYRMMRYASRYCRYYGTRMRRYKKYWWYKMRKTGATGGWKKKYMYYGKKKTKRYWGA
46              ARAYYRMMRYMGGCMGCYTCRMRRYGKYATTKMRKKRGKKKWKGKYMYYKKKKYKRYATC
                   ******* .     *  ***** **   ****. ..  ** ***** *** ***  .

45              YYWRMCTGTMRMRAYKYTAMATYAATCCYYYRRCRRRYRMYKTCRRKMKMWRCTKKWYKW
46              YYWRMYCACMRMRRYKCYGATGCTWCAAYYYRRYRRRYRCCKKAGRKMKMWRACKKTYKW
                *****  . **** **  . :  :  ..***** *****  *.. *******. ** ***

45              RKSYKYMGMYCKSRRYKWRMKRYYGYRYRYYYYWYRSACCCMKYRWKRWKMRRRKKWWGY
46              RGGTGYMRMYTKSRRYKWRMKGTCRYRYRCCTYWYRSRYMYMKYRWKRWKMRRRKKWWRY
                * .  ** ** **********    ****   *****    ***************** *

45              KYKMRWRRKRYMMMYYYRAKRMYRYYYRKYKCTGAKMKSRYYRRYRWKGAKMKKKRGYMS
46              KYKMRWAAKRYMMCYYYRWKRMYRYYYRKYKYYRWKMKSRYYRRYRWKSRKMKKKRKYMS
                ******  ***** **** ************    *************. ****** ***

45              WKWAATGYRRAYRRGMSKRRKRYKRYGYKKAYRMGTKTKKTYYTTGCCARKRRRKYCCYC
46              WKWRRYRCRRMYRRKMSKRRKRYKRYTYKGCTRMRKKKKKATCKAAYTRRKGRRKYTTYY
                ***     ** *** *********** ** . ** .*.**:  .:.   ** ****  *

45              YTGGGAMTTWTAAGCYKRKKRWTTAGAGCTYYTCGCGYRRRRKACRSMRKWWYKYKYKKY
46              YYKTARMYCTAGGAAYKRKKRWYWCRWKYYTYGTRARYRRRRKRYRSMRKWWYKCKYKKY
                *   . *   :....*******  .      *   . ******  ********* *****
```

```
45          SWRKMMWYRSKKWKKAARYMKAACGAGMKGRKTKKKYYARYSKKTKKRKWWYKCAWTAAA
46          SWRKMMWYRSKKWGKGRRYMKCCAARAMGAATWKKKTTGRYSKKAKTRKWWYKYWWCMTT
            ************* *. ****.... .* . . ***  .*****:*.******  *  ::

45          YAGCGWKWYRARGATTCYGGWKCGRRRGRRYAGTKCYKMRYYKWRWKAYSYSKGYGAKYS
46          CTRYRWKWCARRRRYYYYCAWKMSRRRRRRYGSYKTYKMRYYKWRWKGYSYSKRYRMKCS
             :   ***   *     * .** .*** ***.. * ***********.***** *  * *

45          YMTTYKRRAGCWKGAACWGYTCGMCKKTMYKTACCAGRRSRKYRRAKWWTTCGSYYC
46          YMYYYKRRRRTWKKGTGASCWYRAYGTCMYKWRYMMRRRSRKYRRMKWWYGTASYCT
            **  ****   ** .:  .       . ***      ******** ***   .**
```

The pairwise alignments of two accessions 45 and 47

```
45                  GCYRKRWYKWGYCMWAWMMKRYYYKMYYRYRRMMTKYRYRCYYWWCYGRRMSGMMYRCYR
47                  KMCAKRWYKWTCTMARWMMKRYYYKCYYRYRRMMKKYRYRTYYWWMYRRRMCKCMYRMCR
                        ******   *  ********* ********.***** **** * ***.  ***  *

45                  MRCYCRMYWRYGKMYYCKYMGKYWKKMYMMRWKRRWKRYKRYAYYRYYYKYYYYKRRKWR
47                  MAYCYGMYWRYRKMYYYKCMKGYWKKMYMMRWTAAWKGYKRYRYYGYYYKYYYYKRATAR
                    *     ***** **** * *  **********.  ** **** ** ********** . *

45                  YMRAYRSWMKKWRRYYYRYWWRYWYMSYTWYWWWWYYWYRWKYYWRKMYKYRYRRACCRT
47                  YMGMYRSWMKKWRRYYYRYWWRYWYMSYCWYWWWWYYWYRWKYYWRGMYKYRYRRWTTRW
                    **  ************************ ***************** ********   *

45                  RYRYWYRRYKKYWCGYCYYTTARRYMYYYCGMYKSCSKMYKMKYKSKRMRYWYMYRKRRW
47                  RYRYWYRRYKKYAYTYYYYYWRARCMYYYYKCYKGTGGMTKMKYKSKRMRYWYMYRKRRW
                    ************   * **    * ****   **. . * ********************

45                  WARAAMKMMRYMKYWKKAYWYWYWRWKRKYMKYGMYWKRWWKYRRYRGCKYYTRMKMKYR
47                  WRRTWMKMMRYMKYWKKWYWCWYWRWKRKYMKYKMYWKRWWTYRRYRTAGCYGGCKMKTR
                    * *: ************ ** ************ *******.***** .  *   *** *

45                  KYKTYWWWTKYCKYYKKRCRYKGGTCYRKYYKYWMKKYSRRYRKYYSYYMKKRRYWMWKM
47                  KYKYYWAAKGYTKYYKKRTRYKAKATYRKYYKYWMKKYSRRYRKYCCYCMKKARYWMAGA
                    *** **  . * ****** ***. : ******************* .* *** ****

45                  MRYRWYSRSMKMKWMRKRCTRTMCKCAGYMRMMYSWWYWYKYMRYMYKRKKKRWKACGKR
47                  MRYRWYSRSMKMGTAAKRMWACCAKTGAYMRACYSWACAYKYMRYMYKRKGKRWKTTAKR
                    ************    **     .* ..***  ***   *********** ****: .**

45                  YSKGAWYWYYRMKKYWRTYYYYWRYRYKSAYYRKAGRKRYCKKRRCMTRRKKWCTYTAGS
47                  YSKKRWYWYYRCTKYWRYYYYYWRYRYTGMYYRKRKRKGTTGKRRYMKGRKKWSYYYCKG
                    ***  ****** .**** *********.. ****  **    *** *. ****. *  . .

45                  MARYKRGRYWTKGARSAGTRAYTAYYTWTKTCGKRYYKKYMRKYRMRSYWRRYRGCAKRM
47                  CGRYKATAYWYKATGGRRKRMTWGYCATAKYSSKRYYKKCCATYRMRSYWRRYRTTCKRM
                     .***   ** *.:  .  .*   .* : :* ..******   .***********  .***

45                  YYKKWWMWMYRYRKGTAWMMMGRARMWMKSMCCGGYGMCARWRKYGTKMRAMGCKRMMRR
47                  TYKKWWMWATRYRKRAWWMMMRRCRAAMGSMTAARYKMGWRWRKYKKKMRRMKYTRMMRR
                     *******  **** : **** *.*  * **  .. * *  ***** .*** *  .*****

45                  YYRYYRMMYWYKRCTRKGKSRGGTGGATCWTTYTTYTTRWRASRYGRACAKYWKSMRKKY
47                  YYRYYRMMCWCKRAWRKAGSATRAAAGAMWYWYWCTCGRWRRCACRRRYRGYWKSMRKKY
                    ******** * **. **. *   :...: *  *     *** .   *    *********

45                  MRKKWYRYWKKMKRWRKKRYRYKKRKKYAKKYTKKKKYRYKCTTRKKKRKMMYWRKRGGW
47                  MRKKWYRYWKKMKATAKKRYRYKKRKTCGKKYKGKKKYRYKYCGRKKKRKMMYWRKRKAW
                    *************   **********. .***. *******   ************* .*
```

```
45      KWRYRMWYRKYKWRRKYRRKRKKKAAKYYSKGRRGAWKTTKRMMRRKKSKKYYTKKKYMK
47      KWRYAMWYRKYKARRKYRRKRGKKRRKYYSKKRRRWWKKYKRMMRRKKSKKYYKKKKCMK
        **** ******* ******** **  ***** **  **. *************.*** **

45      YRYRYKKYWRWYKMKWYKKRRRSMKRKYRCTTMKRKKKSTTCATCYMTAMRKYMMRKSKG
47      YRYRYKKYWAWYKMKWYKKRRRSMKRKCRYYWMKRKKKSWWMWKAYMYRMRKYMMRKSKK
        ********* ***************** *   *******    ..**  **********

45      WKKWYGCGSAGTTRYKKKKMKYYKYMWKYRYKYRKTTAGAWYYYYYKKRWWKMTAACYCT
47      WKKACRYSSRTCAGCKKKKMKYTKTATGYRYKCGGGYTCGTYYYYYKKRWWGMATGTCYG
        ***    .*   :  ******* *    ****     : . ********** *::.

45      YKGWRRTTKRYKRYGTTKYYKSRMKRKAGYGTYKRMKATGKYKWTKRRKRKCTYCKRWWY
47      YKRWRRCKKGYGRCTCCKYYKSRMKRKTAYRKTKRMTRWKKYKAAKRRKGKYCYYKRWWY
        ** *** .* * *    **********:.* . ***.   *** :**** *  * *****

45      RMKRKSGMAAYTKKTKRWGGGRYKKYYTKYKKWAGCYRKRWYWKRTYRKRGRYRMKWRRY
47      RMKRKSRMGTCCKKGKRWRRKRYKKYCCKYKKWMRMYRKRWTWKGYYRKRRRCGCKWRRY
        ****** *.:  ** ***   *****  *****   ***** **  **** *   *****

45      YYRYGTTKKWKMKKTKKKWYKSYWYKKYMGRKKKKTRGYWKAARCTTKGGRRYYWRRRRK
47      YYRYACWKKWGMKKGKKKWYKSYWYKKYMKRKKKKKRKYTKWWRYYYKKSRRYYWRRRRK
        ****.  *** *** ************** *****.* * *  *   * .**********

45      KSYRRKKYWAAAGTYYMKYWWKCKRKRKRMRCCTKKWRYKTSYKAYCGYYKKKCTRMAYY
47      KSYGRKKYWCGTCYCYCKYWWKYKRKRKRMAMTAKKWRYKGCYKCCYKTCGKKYKACCYY
        *** *****..:   * ***** *******   :****** .**.      ** .  .**

45      RKWRKTRRRYKYYYRYRYWYYYYWKACKKKRCAYRYYRRKKKYCSRYMGRWKWRRYAYWR
47      RKWRKYRRRYKYYYRYRYWYYYYWKMTKKKAYRYRCYRRKKKYMSRCMKRWKWRRYMYWR
        ***** *******************  ***   ** ******* ** * ******* ***

45      GAAAKKRMCTRCYYRYRYRSYKYRMKYYKWYGKMCRTTAARYKRYYRTCAGCAKCGWCKR
47      RRWRKKRMMCAYYYRYRYRSYKYRMKYYKWYRKMTACCGCRYKRYYRGTCRYGKMATMTR
            ****    ******************* **    ..*******  .  .* .  .*

45      KSYWKYYSWGGRTMYYKYRRYYGTMKRYWRMWRWYRKRCKMTMKTYWRKKKYTKCYMGAC
47      KSYWKYYSWTTGCMYYTYRRYTRAMKRYAAATGWYRKRTKMWMKGCWRKKKYWKYYMRGT
        *********    ***.****  :****     ***** ** **  ****** * ** .

45      AYGATMGMKCSRRYKYWRKYRKYRRSWSKRSCCTCKRWWWWKAKGRSYCKYKGYMKMRWA
47      CYAMWMKMKTGAATKYWRKYRKYRRSWSKRSTGKAGRWWWWKRKKGGYYKYKTYMKMRWC
        .*.  * ** .   *****************  ..****** *  .* *** ******.

45      CCCKGCCAAKYYYRRMRYMYKYCTGAGYRGAMKKRYYMKRYRWKKCTGAMGTRCKKKRYY
47      TMTKATYTGKYYYRRMRYMYKYYYRWTCGSRMKKRYYMKRYRWKKTASRMAYRYKKKRYY
           *.  :.*************       . **************:.  *. * ******

45      YMSWRYMMYSRRKRYKSMKRYYKARKTMMAKRRYWYSMKYKKRWWSYKRRYKYKKWKYKY
47      YASWRCMMYSRRKRYKSMKRYYKCRKYMMMKRRYWYSMKYKTRWWSCKRRTKYKKWKYKY
        * *** *****************.** ** ***********.**** *** *********

45      GGGGGKCGRKYSSRRWKKSYWYMTGRYGKTATKYRYYKYYKWKRRTTWCGWMKSYYKRKW
```

```
47          SKRSRKYKGKYSSRRWKKSYWYMACACTKYMYKYRYYKYYKWKRRWYWYKWMKSYYKRKW
            .  . *   **************:    *   *************  *  **********

45          RYKKKYRYRKKKKKAGRKARYMCYKKTGRRYWYKYKWSKRYYRYYTAACRKKKMMKKACG
47          RYKKKYRYRKKKKKGARKRGYMMYKKYRRRYWYKYKWSKRYYRYYCTGYRKKKMMKTWMK
            **************..**  ** ***  ***************** :. *******.

45          MRWAAAACRYRTYRYRAKKYKATRRKSKKYKGCAARAYTSGCRKTKCCCAKKGGRRYYTG
47          MRARWTWTRYRCYRYRRKKYKCCRRKSGKCGRAWGAWYGGTTRKKKYYYRKKCARATCCR
            **   :  *** **** ****. **** *   . .  * .  **.*    ** .*

45          ASTKKRKYRRSGGCYTRWKSYWRKKRYYYYRMMKTWRKWYMTKYMRCKTYYTMACYRRYC
47          GSKTKRKYRRSKKSTYGTKSYWRKKRYYYYRMAKKARKWYCAKTARMTCCTCCGYYRRYG
            .*..*******  .    ************** *. **** :*  * .     . ****

45          WRRKRWKWGKSKKWRRYYWRSRRKRYSWCTTTASYMRWYWRYRYYRYYRRKMWMYWTKYM
47          ARAKRWKWRKSKKWRRYYWRSRRKRYSWTCCCRCYARWYWAYRYYRYYAAKMWMTWCKYM
             * ***** *******************     .* **** *******  **** * ***

45          GMYSRRAARGYACYRYWRYRRKMKRKWYRGARKWKKMKKYYRYMKGYKWMYMRAWMMRYR
47          KCCGARMRARTGYYRTAAYRRKMKRKWYRCGRKWKKMKKYYAYMKKCKWACMRRWMMRYR
               . *     . **   *********** .********** ***  **  ** ******

45          YYYYWYYRWTKTTKKKYKSMYYYRKRKRRRYKKATYKWKATGTKAYRAKRRKKKTGCTTK
47          YYYYWYYRACGYWKKKYKSMYYYRKRKRRRYKKGACKWKMWKKKWYRMTRRKGKCAMCCK
            ********     ********************.: ***   .* ** .*** *  .   *

45          YMKKRYCGKGRKKYMMRKYYYSKKGWYKSKYKMYRGRKKMSRTMRKCGYSTYMMTYRRSY
47          YMKKACMRGKRKKYMMRKCYYSKKRWYKSKYKMYRARGKMSRCMRKMRYSKYMMCYRRSY
            ****      ******** ***** **********.* **** ***  **.*** *****

45          KKTYRRRKKGMMYTYYWKYAYKCKKKTGKYAMKKRYYYMMSYWMRKRTMYAYRRKKKMYY
47          KKKYRARKKKMMYYYCWKYGYKSKKKWRKYGCKKRYYYMMSYAMRKRKMYRYRRKKKMYY
            **.** *** *** * ***.**.***  **. ********** ****.** *********

45          KWTCTTYKKMRYMRYYMRSWRKTKWKYKMAYMYRYYRWTYAKRYKYKAKMTTAAKRRSMM
47          KWCTGGYKKMRYMRYYMRSWRKCKTKYKCCYMTRCYRWKYRKRYKYKWKMCAGMKRRSMM
            **    **************** * *** .** * ***.* ****** ** :. ******

45          MYKTMWCYCTRMRWYMKSRKKKAYWRRRCGGCRKACKRTGRAKRKWKAAKTATGRWRMMY
47          MYKCCAMTYYRMRWCCKSRKKKRYWGGRYSKYRKWYKRGRAWKRKWKGGGARWRGWRMMY
            ***       ****  ****** **  * .  **  **    *****.. :    *****

45          WYWRMAWRMYTYGRGAARRGYWWYRWYRGACARMWYKKKKSKRKYRATRATCCAAKKKSR
47          WYWRMWWRMTACAGTWRRRKYAWYRWCRRGTGRAWTKKKKSKRKYRWYGGATTGGKKTGG
            ***** *** : .    ** * **** * . .* * **********   .:  ..**..

45          YYKRMWKATATRYCTARYKKWKRWKKAGTTACAGMMMWMYGGGYKGRWKWCRYRMWYYKW
47          TYKRMAKRCGKRYTAGRCKKAKRWKKTAAARARAMMMWMCCTRYKKRWKWSRYRMWCYGT
             **** *  ..** :.* ** *****:.:: . .*****    ** ****.***** *

45          YCATMMGYYYCTCACKKRRYTAAGAWMTKSKMYYRMKRYKYKAKKGYRRKWYWSYMGTRA
47          TTTCMMAYYYSAYRAKKRRYKCCACWMCKSKMYYRMKGCKYKRGGKYRRKWYWSYMAWRR
```

```
          : **.***.:  .*****.....** *********  ***    **********. *

45          TTKGYMKYYWYRKRWGGCYYWKWRRRYYWMAYGKWMKRYYKKKKKGYYKMKYTGRKYKWR
47          CYGKYMKYYWYRKRWKSYYYWKWRRRCYWCRYRKWMKRYYKKKKKKYYKMKYWKRKYKWR
                ***********  . ******** **  * ************ ******  ******

45          YKKTAMRYKKYRRYRMMKRWYYASRYCRYRRKKWYWWYKRKRKTKKKKKKKYMKYYKKKK
47          YKKYMMRYKKYAAYRMMKRWYYMGGCMGCRRKGWYATTKRKRKAKKKGGKKYAKYYKGKG
            ***  ******  ********* .     *** **   *****:***  *** **** *

45          YWCGAKARYYWRAYMTMRMRYAAGCWRKYYWKKYRRRRRRYCKMMYYRRKRKKYYYYRRK
47          YAMTCKRRYYWRWYMCMRMRYCCCYWRKYYWKTYRRAGAACMKMMYYGRTRKKYYYYRRK
            *   .* ***** ** *****..  *******.***      ***** *.**********

45          MGYYMYYAWRMAWRMCYTTSRRKKKKKKKRYRYYWSGYRYMKKKRKMRRWKGTCGWMRRS
47          MRCYMYYRARMWWGCYYKKSRRKKKKKKKRYRCYWSSYRYMKKKRKARRWKTYTRWMRRS
            *  ****  ** *   *..************* ***.********* ****    *****

45          RYSYTTKKKYSKKKYRYWYRRRYKYYRYKKYRKMCKKRKMRYYRAWKWYMTKKKYKYMGK
47          RYCTCYKKKYSKKKYRYWYRRRYKYCACGGCGKCYKKRKMRYYRRWKWYMKKKKYKYCTK
            **.   ******************        *  ********* *****.******  *

45          KWYRKMMYMYCRWRGTTSATKRYYKKKRKKKRAYYYTRKRWRYKKGGKASYWKRKKKWKK
47          KWYRKMCYMYYGWRSKWSRATRYYKKKRKKTGGYYYAGTRWRCKGKRKRSYWKRKKKWKK
            ****** ***  **.. * :.*********. .***: .*** *   * ***********

45          RRWRRKTGCYYYMYKMMYMKMSRRKYRYRRSKMRKKMRKRMAGAYYKYKTYGGTYTMYAR
47          RRWRRKCTTYYYMYKMMYMKMSRRKYRYRRSKCGKKMRKRCCATYYKYKYYAAATYMYCR
            ******   ***********************  ****** ..:***** *..:  **.*

45          YYRRRYWYMMWKKYWMTWYCKTATKYWWKCAGYKCATWRYRKKTRKKWYKKYRGTYRKYY
47          CYRRRCWYCCWKKYWMYWTYKKCWKCTWKGGKTKYMKWRYRKGWRKKTYKKYRRYYRGCY
             **** **  ****** *  *.. *  ** .  *  .*****  *** *****  **  *

45          KYCARGRMRKYYKKWMRGGWYYKKKKYRYYRTWWKKYARWAMRMYYRYKGRYKYTCCGRK
47          KTYRGRRMRKYYKKWMRRRWYYGKKKYRYYRKWWKKCMRTCMRMYYRYKTRYKYCTTARK
            *     ***********  *** ********.****  * .******** ****   .**

45          YAKYKYYMYMMYATGGARRGKKKYKWYAAKKKYSRKRYMYKYRMYYRTRCCGMAYRTTAA
47          YRKYKYYMYMMYWWRKRRRKKKKYTACRWGKGYSRGRYMYKYRMYYRAATARMRYRKYRG
            * **********     ** ****.     * *** ***********:  . * **.  .

45          RAMCMKKKTKMYRCRKYKAMKATAWSYWYKYRKRYKYSCCGYKYACATKWYYKYRWKKAT
47          RCCYMKKKYGMYRYATYKWMKRYRWCYATGYRKRYKYSYMKCGCMYMYKWYYKYRWKKRA
            *.  ****  ***  .** **   *.*   ********          ********** :

45          YRYCGTGRRYAGMYTYMWGTCTKTGRCYAKKCTWWWWKGYRYYRMCAWWYKRWKCGCGRR
47          YRYTTGTRRYGKMYWYCWACTYKKRGYYWKKYKWWWWGRYRCTAMMRWWYKRWKMSSKRR
            ***    ***. ** * *.   *.   * ** .****  **   *  ******* .. **

45          RRRYKYRYKKYKWYKKGYRKMYWYCCSYCGMAGTYRRTCRYRCCTAYMRCAKTCGTCKMC
47          RRRYKYRYKGTTWYKKRYGTCCAYTTSYSSMWKWCRRKTRCRYTATYMRMMKYMSWYGMT
            *********  .**** * .   *  **..*    **. * *  ::***  *  .   *
```

```
45          YKAATWMGWTCSKRRKACAAYRKYYCRYRKYRCATTAWRCAKRTKMSKYYCRWKGTRYWR
47          TKGTAWMRWCTSGRRKTAMGYRKYYTRYRKYRYRAAGTRMRKRCKMSKYYYATKRKRYWR
             *.::** *  * ***:. .***** ******  ::. *  ** ******   * .****

45          WYKYWAAKKTAGKMYYYRYRYRRKKKTTGKKRYRMRWMMWYKYAYMYTGTYRYCCARYKR
47          WYKYWGWGTAWRGMYYYRYRYRRKKKKWRKKGTRMRWMMWYKYWYMYYKYYRYYTTRCKR
            *****.  .:   *************.  **  ********** ***   ***  :* **

45          RKWRSTMYCTYKMYYYYYSRRYYKKWRYWWCKWWKYTTYKRRMTAGKAYCTKYYSGCARK
47          RKWGCKAYSYYKAYYYYYSRACTKKWRYWWMKWWKYYGYKGGCKTSKGYTCKYYSATRRK
            *** .. *. ** *******   ******* *****  **   .:.*.*  ****.  **

45          RRKATTCMYWWYTMMRRMRKYKRKRCAGATTTKYMKYYKMYYSWGYWKWMYKYAGCCKKK
47          RRGRCGTMYATYKAAARMRKYKRKRMWKTWGAKYMKYYKMYYSWKYWKWMYKCTTGAKKK
            **     **  *.   *********   :  :************ ******* :  .***

45          CKWRKYWKTRYTCGCTMKKKRKRMCAKKRYATATSYRKKKYRCAYRWRRKYYMMKKMYWS
47          YKWRTCWKYRYCYATCMGTGRKAAYGKKRYMKTASYGKKKYRTWYRWRRKYYMMKKMYWS
             ***. ** **  .  * . **   .**** .::** *****  ****************

45          YMCKMRMKYKWWYYKTATTKKMKKTKRRSWCTCYKMRYGMRTCYYWMRYWATYMYGRKMY
47          YMYKMRMKYKWWYYKGCCCTKMKKKKRRSWYYMYKMRYCMRWMYYWMRYWTYTCYKRKMY
            ** ************ .  .****.*****   ***** **  *******:   * ****

45          KKKMRRATKTGWMMWKKACGTWKYKYACGYGCKAWAARKMRYMRMRMRMKRAAATGTKKK
47          KGKAGRRKKYKWMAWKKTMRCWKYKYGAAYRMKRTTGRKMRYMRMRMRMKRRWRYSGKKK
            * *  * .*  ** ***:   *****...*  *  :.**************    . ***

45          RYYTGTCRKCKRYYMRRYKGYMWWYRYKRMWAMMYWKWRWCTTYYYWTCRKKRWKRRRMY
47          GTCAACGRKYKRYYARRYKRYATTCRYKRMWGCACTTWRWSYCYYYWKARKKRWKRRRMY
               :.  ** **** **** *    ******.    .***.  ****..***********

45          RRRWWWKRKYRRKWKATWKYCGAKRCCCCCAGCYKRYSWSKRTTCRKYRGMYYKKRKKMK
47          RRRWWWKRGYRRKWKWAAGTSKRKRTTMYTTAGYKRYSWSKGCYYGKYRRMYYKKRKKMK
            ******** ****** :   .  **     :. ********     *** **********

45          KGACRKKKWKKC
47          KKGTAKKKWKKY
            * .  ******
```

The pairwise alignments of two accessions 45 and 48

```
48                  YRRYKRWMYKMMMYRYKWWRRYWSGCTYYYKYYTTYWRYRYRAAAGKMKYYRCWWYRWRY
45                  YRRYKRWMYKMMMYRYKWWRRTASAAAYYYKYYCWYWRYGYRWWRATMKYYRTWWYRWRY
                    *********************  *..:******  **** **   ..***** *******

48                  YKSRYYYMRYYRWRWKYRYYGGRYRYRRWMKRMRRKRSWWRCKKYMKRYYYYYYRMGYKM
45                  YKSRCTTARYYGAAAKYRCYSARYRYRRWMKRMRRKGGWWRTKKTAKRYYYYYYRMAYKM
                    ****    ***    *** *..************** .*** **  **********.***

48                  KGRRYKYAATTTAGSRRMMMWKKYRYYKRMYYYYKWWYRYKRYWMWRGYKYMRRWCWWRY
45                  KTRRYKYGMCKKCAGAAMMMAKGYRYYKRMYYYYKAWYRYKRYWMWRAYKCMRAWYAAAC
                    * *****.  .....  *** * ************ ***********.** ** *

48                  YMKYMRRMRWWKKWKRRMCAYMYTTWWWGYYYTYCRRCRKRTAMKRRKYRTRYMRMMWYY
45                  YMKYMRRMRWWKKWKRRMYTYMYCAWWWAYYYCCTGAMRKAWGMKRRKYRYRYCGCAWYY
                    ****************** :*** :***.***      **  .******* **    ***

48                  ARKWCYYRRWRWYGMYMRWCGWRYSYSRWYKGGYYYRGCTYGRRMRRYYYWMMMYYMRYY
45                  TGKATCCAAWRWYKMYMRWTAWRCGCSRWYKARYYYARYYCSRRMRRYYYWMMMYYMRYY
                    : *      **** ***** .** . *****. ***     .******************

48                  YRKYRYYRRYWWGGYMRYYRARCCSKGKYRYKRRGWRRKYKWYRYGWTRKYATRYWKKSK
45                  YRKYRYYRRYWWAAYMRYYRMGYYSKRKYRYKGRTWRRKYKWYRYSTWRKYTGAYWKKSK
                    ************..******    ** ***** * **********.  ***:  ******

48                  TYKSRCTCGRYYKRGCATTACTKCRYRMMRYMTWYMYMYKWRRRRMCAGGTTYWKYRYYY
45                  CYKSRTCAARYYKRRYGGKTAYGMGYGMMRYMAWYMYMTKWRRRRMACATACYWKYRYYC
                     ****  ..*****  . .:.    * *****:***** *******... : *******

48                  KKKYSKWTRSYRKAAMKYYATATKRYYSWMYYYKYSKGWRYRTKAKKYKRWASKKWKKKY
45                  KGKYSKTGRSYRKRRMKYYTCGKTRYYSWMYYYKYSKKWRYRKKRKKYKRWWSKKWKKGY
                    * ****  *****  ****: ...************* ****.* ****** ****** *

48                  RKKYKRYKMMKMGAARKGAGTRAAYYTSYKKAKAWCKRKYCTGCCTTYWATCRKKSYRRK
45                  RKKTTACKMAKMAGGRKRWSYRGGTYKSYKKTTGTMKRKYYYRMTAAYWMWMRKGSYRRK
                    *** .  ** **...**  . *.. *.****:..  ****     ::**   ** *****

48                  RKKWKRMMYKRYKGYGAYYTRYKRRRYKYTAKYKKRYAKTRKKMKKKKRYCYWYRWYKRW
45                  RKKWKGMMYGRYKACTTYYGRCKRRRYGYCGKYKKAYTKAGKKMKGKKRYYYWYRAYKRT
                    ***** *** ***.  :** * ***** * .**** *:*: **** **** **** ***

48                  GTARRRWGRKKATYMRYSRGSMRGGYMKWKGKMRRTGAYKRRYYKTTWYYKTRRMYGAKR
45                  TGCRRRWKRKKWAYCRCSRRSAAAAYMKWKTKMRRGTTCKARYYKYKATYKYRRMYKRTR
                      .**** ***  :* * ** *  ..***** ****  : * **** .  ** ****  .*

48                  RWKMCMKKWRWGTGTCRTMYKMRRYRRRCACCGACTKMYMTYKRRSRYRTGCGRRAGRYY
45                  RWKMMCKKTGWKAAWYRYMYKMRACRAATWSMRRMYKMYMKYKRRSRYGCCGTRRTTRYY
                    ****  **  * :.  * *****  *    .     ****.*******     **: ***
```

```
48          RTRYWWACRAARAAGKRWSKWKGWWCGATKKKCGCRMRYYYTKYMRMYKRKYRWAAYYAA
45          RYAYWWWYGGWRWMRGRWSKWKKTWGTRYKKKTAAAMRYYYKTYMRMCKRKYRWMGYYRM
            *  ***   . *    ******  *    *** .. *****..**** ****** .**

48          RWMAKSYYSRSATGCWMTWSYKYCCATACYYRKKYKGGAKRMARMYKGKSYRYKMKRMWY
45          RWMRKSYYSRSGGCTTMYWSYKYYTGKGSYYRKKYKCCTKRMMRMCKKKSYRYKMKRAWY
            *** *******.    * *****  ....*******  :*** ** * ********* **

48          KKKRRYKGMTKYWYYRSMGAAKATGRKKKKKRYWYYYRRTCKYRKKRKYKRYRRKWKKRW
45          KKKRRYKRMWKYWCTRSMTGCGTAARKKKKKRYWYCCGAYYKYRKKRKYKRYRRKAKKRA
            ******* * ***  *** .. ::.**********      ************** ***

48          KKWKWRGYKRKTKTYYYMKGRATCMGRYKACARKWAMSYKRKRKKWRYKRRKKAAAKYRY
45          KKWKWRAYKGKCKKYYYMKRACCTMKRYKRSMRKWWAGYKRKRKKWRYKRRKKTCTKYRY
            ******.** * *.*****  .  * *** . ***  .***************:.:****

48          TGTKTAAMYYYRWKRRYAYWGRGYKCGTCKKTCGCGCKSKYTTYYYYRYYKKWYKMKYYA
45          ARAGYMTMYYYRAKRRYRYWKRRYKTAAAKKGYKYRYKSKCCAYYYCACYKKWYKMKYYW
            : :   :***** **** ** * ** .:.**      ***  :***   **********

48          AGTKMKYRKWKAKSRSKKKKGTRKRKMKRSYRCCAYSWWMKWSYYCKRYKKWKATCTKYK
45          WRYKMTYRKWKMKSRSKKKKRYAKRKMKRSYAMMRYSWWMKWSYYMKRYKKWKMYYYKCK
               **.***** ********   ********    ********** *******    * *

48          KWMRRMMWRKWMWKKKTKCYYKWWYSTRWRKWAGCAYMWRRWWMRWYGMATYMYMRRYKW
45          KWMRRMMWRKWCATKGKKYTYKWWYSYGWRKWTTTRYMWRRWWMGWCCMGAYAYMRGTKW
            ***********  .* .*  ******  ****:   ******** *  *.:* ***  **

48          WACRWKSMRCACKWKSKYYKYMRAKSWRYKCCKWTYWYRKKMKRRKMMRGKRKCGAYYYG
45          WMYRWKSMRACTKAKSKYYKYMRGKSWRYTYTKWCYWYRKKMKRRKMMRKKRKATTYYYK
            *  ******.. * *********.*****.  ** ************** ***. :***

48          ACKKKTRYRTWRRMTGYYRCAGGKRKYYTRKCTWKTCRGTTMKMRWMRWKGGGKWKRAGK
45          GSKKKCRYRCWRRMYSTTAYWRSKRTCCAGGTGWKGSRKGCAKCRWMRAKAAAKWKRTRK
            ..*** *** **** .      .**.  :    ** .*    * **** *...****: *

48          KWWSKKRYKRWKYRKKRWWGCKRRKRYYTRYYRKCAAGKYKMYGRRMMYYYTWYYAASKR
45          KWWSKKRYKRWKYRKKRWWRYKRRKRYYGRYYRKYRMRKYKMYKRRMMYYYYWYYGRSKR
            *******************  ******* *****    ***** ******* ***. ***

48          KKWYCACCTRKKYMRGRCWTKYRWKCAAARKRYYWYAACTWTAGKKWRAYGCWYKKYYYC
45          KKWYTGATARKKYMRARTTYKYRWKACGGGKRYYWYCGAGWYRKKKWRWCKMWYKKYYYM
            **** .. :******.*   *****.... ******... *   ****    *******

48          YYKYSMMGWKMKYYYYYKYWMMYKRRRYRMKWRKKTCKAYYKAAKMRKRKTGYAKGRYCT
45          TYKYSMMRWKMKTYYYYKYWMMYKRRRYRMKWRKKYMKTYYKWWGMRKRKKKYGKTRYAA
             ****** **** **********************  *:***   *****. *.* **.:

48          CMYWGRYYAGRYARMYMYCCGTWKKMKGCAAYKRYTTTWWKRYRRRKKWRKKGCMRYTWW
45          TMCTKRYYRRRYRRMYMYYYRAWKKMKRYGCCKRYYYYAAKRYRRRKKWRKKTAMRCWWW
             *   ***  ** *****   :*****  .. ***     ************ .**  **

48          WRWRKKGMRWKWRAYYYYRKCTTRTKWMYRKRMTTRCCYATYTTGCWARKRMRKYRKYMS
```

```
45          WRWRKGKMRWKWRCYYYCGGYKYGYKWMYGKGMYYRTYYRYYYYKMWCAGRMRKYGGCMS
            *****  ******.***    .   **** * *  *  *  *    *.  *****   **

48          RRYASCWMKGKCGMKGCTRARMRGCCGKMKKYRMWKAWTCTRRMRACMMGGRKKRWATYK
45          RRYGGYTCKRKTRCTTMAGTRMRATYKKMGKYRMWKMWYYKGAAATAAATRRKKRWRYYK
            ***..   * *   .  : :***.   ** ****** *  .    :.    *****  **

48          RRKSYYGCGSMKMRRYRMCTCRYRRYYRKYWKKWRKYYYKKYKKKGYKRYYCRTTYMMYK
45          AGKSYYTTKSAKMRACACYYMRYRRYYRKYWKKWRGYYYKKYKGKTCKRYTYRKYYMMYK
              ****   * ***       ************** ******* *  ***  *. *****

48          RRKYKTCKMKYGYRKCYGGCARKMKRKKKWYYMSYKKRYSKAAMYRMRKKACTAKYRRRK
45          RRKYKKTTMKCTYRKTCTRTWRKMKRKTKWYYMSTKKRYSKRRMYRMRKKCACGGYRRRK
            *****. .**  ***      ******.****** ******  *******.. . *****

48          RRKGTRYWYKWYRYSYRKCACYYMYSKYRKYMMKRYGKRWKKRCCYWWSKYMAYYRWRYK
45          RAGAGRYWYTAYACCYGGYWTYYMYSKYRKYMMKRCKKRWKKRMYYWWSKYMGYYRWRYK
            *  .****. *  .*      **************  ******  *******.*******

48          YMYYKKKACGGAMWKTYYKKTCYMGRRYGRRRRYYRKRKKYAGMRYYCKYTYKYRWKKAY
45          TMYYKKKRTTCRMWKWCYKKCTYCKRRYKRRRRYYAGGKKCGRMRYYMKYGYKYRAKKMY
             ******     ***  ***  *  *** ******   ** . **** ** **** ** *

48          WRARSRRYYRYGYAKKKKKKRYYYMMWRAYRMYMMCWSGRYYRWKRAYWKYRRMCGYMGY
45          WRGRSRRTTACRTGKKKKKKRYYYMMWRCYRMYMMAWSKRYYRWKRGCWKYRRMMRYMKY
            **.****      .**************.******.** *******. ******  ** *

48          GYKYKKGTGYWGRKRKGTCAGKMCTGYGGCAARKRKKGKCMTRKYYYMMSATCTWKGKTA
45          KYKYKKTCAYWAAKRKRGMGKKMYWRYKATGTRKRKKRKMMCAKYCCCCSGGTWWKKKYW
             *****  .**. ***   . **   * . .:***** * *  **    *.   ** *

48          YRMRKKKWGKYRYKRGTKRGKCWWYKKKRYKRYYSKYYYMKATCKRAKSKKCKAATYWMY
45          YRMRKKGWKKYRYKRRYKRKKYWWYKKKRYKAYYSKYYYMKWCAGRWKSKKYKRRWYWMY
            ****** * ******  ** * ********* *********  . * **** *   ****

48          SKRKCAGRYYCGGCAGCCRKYSRRRWKCGYGKKKYWWMRKRKYKRKRRRYKYWMRMWYRK
45          GKRKMGTRYYASRYCRYTGKYSAGGAKTTCRKKKYWACGKRKYKRKRRRYKYWMRMWYRK
            .*** . ***..  .    ***    *    *****   *********************

48          RTTARCYMRYYMWKGTCKRYWRKMYRKRKKGYMTAYMYYKYTYMTGYKGKTTKRAGKYYA
45          RCAGGTYMRYTCAKRKTKRYWRKMYRKRKKKYMYRCCCCKYGYMCTYKKKKKKGRKGYYW
            * :.  ****   * . ************* **      ** **  ** *..*    **

48          KRKWTYGKGAKYYYRRRMMCTRTTGKWAMRTMAKWYKKKYKMWKYRKRRKKTWKRTCGAK
45          KRKWYYKTKWGYYYRRRMCYYRKYRKWTMGYCMKWYKKKYKMWKCGKRRKKKTGAYYRRK
            **** * .   *******   *.  **:*    ***********  *****.       *

48          RYYCKYRRMKYACCCMKKKMYRRYKWTYKKMYWKTKAATRYRWMWKKYWYTWGWTCTYWW
45          RYYYKYRRMKCRYYYMKKKCYRRYKWKYKKMYWKKKTRCRYRWMWTKYWYKWRTWAKCWW
            *** ******     **** ******.*******.*:  ******.****.*   .. **

48          RCGYGRCRMSKRRYKYKKMYYYRRMKKSRRKSRKRKRAYCKACTCGCKTWAYYRTCGYGA
45          RMKYRRYRMSKRRYKYKKMYYYRRMKKSRRKSRKGKGRYMKGYGTATGYWCYYRYYKCAW
```

```
          *  * * ************************** *  * *.   .   *.***    .

48        CYRRYRCTKTKRWATYKYKKWRYAACTAWYRSYTACCTYMWKKKAARMKRARRTCGCCCC
45        YYRRYGAWKYKRWGCYKYKKWRYRRYYRWYRSYKRMTCYMWKKKGRGMKRWARGYRYGSY
           **** . * ***. ********     *****.    ******.  ***  *      .

48        AKYTTCTKYKRKRMKRYATRKRWRSRYTMWYTRAKKYAAMMCKMRAYRGMMKKGCMMSTT
45        WKYCATCKYKATGCKRTRARKRWRSRYKAWTAAGKKYTRMMYKMRCTARCCTGRMCACKY
           ** :  ***  .  **  :********. * : .***: ** ***.      .     ..

48        TTTTGCWKMRMRWKWAGYKACYKRCRYRYATRCKCRMSKRSRTGYGAYGASKWWWRWWKY
45        AAAYATWKMRMRWKWGAYTWYYKGTRYRYWKRTKYRMGKRSRYSYTMCTRSKWWARWWKY
          ::: . *********..*.  **  **** .* * **.**** .*      **** *****

48        TACGKYRYKRCKCTSTKGWKMKMYCACCGTRKSCTARYRRRGAAYRYSRWAKKWGYWMMM
45        YMMRKYRYTRYKYYSKKAAKMKMYMRSYKWRKSYYMAYRRRKRWYRYSRWRKKWKYWCMM
              ****.* *  *.*. *****   .    ***    ****   ****** *** ** **

48        RRMRMAMKGKYYWRGKGTTRKKTKAGCCCRMYRRAAATMCKRRWYYKYAARRGAGKYRRK
45        RRMRMGCKTKYYWRRKKWYGGKKKGRYSYRMYRRGGGWCYKRRWYYKCGGGGRGKKYRRK
          *****. * ***** *     *.*.  . *****...   *******  ..    . *****

48        KYYYYKYAGTRRRWMKKMGYGCRKGRMWRTYRRRKYRRYKWAACGCRGYGAYGRKKKKCA
45        KYYCTKCMRKRRRWMKKCRCRGGKKRMWRKYRRRKYRRYKWMMMKMARYRWTRGKGKKAC
          ***  *    .*******       * ****.***********        *     * **..

48        TYRWKRYMRYYYCRCGAAKRRRYRCACARRRRRYYKKMRKKTKMMYKRRWKMYKYYYACCA
45        AYRWKRYMRYYYAGAAGRKRRRYGYMYMRRRRRYYKKMRKKCKMMYKRRWKATKYCCWYYG
          :***********. ... *****      ************ *********  **      .

48        GTWMCWKRSMKYYYRMATKRRMRWTCCWRYKGKKYRCKKWKWWSMMRKMMKRRKRYWYYR
45        RAWAYWKRCAKYYYRMRYKRRMRWCTTWRYKKKKYRYKKWKWWSMMRKAMKRRKRYWYYR
           :*  ***. ******  ******   **** **** *********** ***********

48        KKTTTKYKKACKYWMYWKKWRMRYKKTTACKRCYKYKRTTKMWMYRRGKKWWTCAATSWK
45        KKWYYKCKKGGKYTMYWKKARMRYKKWYRYKRYTKYTRGGGMACCRAKKKWWGYTRYSWK
          **    * **. ** ***** ******    **  **.*   *   *  ****  :  ***

48        AKWRMGCYGMCCYCRGTKYCYYYKTCGYWGGGGYYRARGRTACMRMCCTCTATYYKGYKW
45        GKWRARMYKMYYYYRRYKYAYCYKKYRYWAAKTCCGRGRRAGAMGMAMKTCCCYYKAYKW
          .***    * *  * *  **.* **.  **..         *:..* *. .   . ***.***

48        GTSYKAMYKRKKYRYYYRRKKYRYCCSRRGCATYRCCMMRWRKGGACACATGMGKARMSY
45        KYSYKMMYKRKKYRYYYRRKKYRYYTSRRKYMWCGTGMCRAGKRRGTTTCGKMAGRRMSY
            *** *****************  ***        * *  *   . : .  *.  ****

48        WYKKCTAKYSYWKSWKRGCRGRRYAAGKYKRKKYWYKWRCWKRKAAGYRKGYYMMKKGTA
45        WYKKYKGKYSYWKSWKRRTGRRRTRWRKCKRKKYWYKWRYWKRKGCKYRKKYYMMKKAGR
          ****  ..**********     **     * ********** ****.. *** ******.

48        YRKRCRYYRYRYYYRYMWKGKRYKGCCKKAAYRYYTRSRKKRMKKACGKKCTAKKYYSKM
45        TGKRYRYYGTRYTYRYCTTRKRYKKSMGKWRYRYYARSRKKRMKKRYSKKYYRGTYYSTA
            ** ***  ** ***   . ****  .  *  ****:*********   .**     .***.
```

```
48                KCCCKKKWRRMYKKYYKKYYRCGRKYRWGKKWGRKRWYSYYAKKAKKKYKTCYKRGKKRK
45                KTATKKKWRRMYKKCTGGYTGATGKYRWKKTTARKRWTGTCRGKMKKKYKYYYKGRTKGK
                  * . **********    *  .  **** *. .**** .    * *****  **  .* *

48                RWKKTAARKKKACCARCCCCTYRMRRWWAYWMRARKRWMSKCKKKMYYKKRYSYYYGGTT
45                RWKKWMRRKKKMMYWRYTTYKYGMRRWWGYWCATRKRWMSKYKKKMYYKKRYSYYYKACC
                  ****   ****    *    .* *****.**  :******* ************** .

48                TYYYKTGWMYRYWKYTSMGKKKKKKYKYRRYWRMMYKKYKKKYRGKYRYMMYKKWRMYKT
45                YYYYKGAACCGYWKCGGCKKKKKKTYKYRRYWRMMYTKYKKKYRRKYRYCCYKKWRMYGA
                   **** .    ***  .  *****.***********.******* ****  ******* :

48                TGKKKKWMYKARSKYYKTYRYRRMWYMYRTTRGKRYGWYTGTTAWKMYTGCYKKMYCCGC
45                GTKKKKWMYKTRSKYYKWYRYRRMWYMYRYKRRKRYSTTATAATWKCTYRYYKKMYSYRY
                    ********:****** *********** .* ***.  : :::**     *****.

48                KKYKMRRKKYYRMGYGRYWGYKSMKKMYMYKTGKSRRCGCARKMYMYRKAYKRYYRKKRM
45                KKYKMRRTKYYRMKCARYTACKSMKKMYMTGCRKSRRAATGRKMYMYAKGYKRYYRKKRM
                  *******.*****  .** . ********    ****.. .****** *.**********

48                RTYKKTRKRKGGRGSKGRCKYYRKRAWRYMMMKKTRKTAGCYRYYKCTMKYYYRRWRKWY
45                RYYKKYRKRKRRRASKKRTTYYRKRMWRYMMMKKKRKYRRYYRYCGTAMKYYYRRWRKWY
                  * *** ****  *.** * .***** ********.**    ***   :************

48                YYYYRWCAYCWYRRKCRYYSWRYYKKMYKRWKCATATWRRWKYRMRKRYSKMYRWAACKM
45                YYCTGAAGCTWTRRKSGYYSTRYTGKMYKGWKATCGATGGWKYRMRGGYSKCYRWRRYKM
                  **    ..  * ***. *** **  **** **.: .:   ******  *** ***   **

48                WSTAWKKKGKKYKCRYWWKYMYRWWYRKAGKCGYWRYKMKTTCWYMCYTTWYYRRMKRYM
45                AGCGWKKKRGKYKMRYWWKYMYRWWYRKMKKMCYWRYKCKWWYWYMYYYKWYYRRMKRYM
                   . .****  *** **************  *  ***** *   *** * .**********

48                CCCWKMKKKWTTYMARKGTTAMRSMWYRYYYKMAYYCAKRMAYWKRRRKKKMWYWTGKRY
45                GMTWKCKKKWYYYARRKRKWRMRGMWTAYCYKMMYYYRKRMTYWKRRRKKKMWYWYRKRY
                     ** ****  *  ** .  **.**  * *** **  ***:*************  ***

48                KYRYRAWYYMYRRWYYMYKYKYRYYKYYKRWGYCRYKKWRRYTTRKKCRKRGCCAKYRKR
45                KYRYRRWCTMYRRWCCATKYKCRYYKYYKRWRYTGYKGWRRYGKAKKYRKGKTAWKYRKG
                  ***** *  *****    *** ********* *  ** **** . ** **   . ****

48                MAYGGM
45                CGTATM
                   . . *
```

The pairwise alignments of two accessions 45 and 49

```
45                AYYWYRWKYYYWRKYRGCTRYYMKWWRYRYKGGMKCKWMYGYKYYYRYGYYTAMWKRWGY
49                TYYWYRWKYYYWRKYRTTGRYYMTWWRTATKRKMKTKTMYRYKYYYRYSYYCRMWKRWSY
                  :***************   ****.***   *  ** * ** *******.**  *****.*

45                RYRKYMYYWCMYKWRYWRYKMRMYWKKWMMYYRWMYKRRMYYWYKWYTRATMYSRRRRKA
49                RYGGYMYYWTMYKWRYWRYKMRMYWKKWMMYYRWMYKRRMYYWYKWCKGWWCTGRGRRKR
                  **  ***** ************************************ .     .* *** 

45                MYCCYCYWWCRSSYCTAWCYKCYWTTGMWWRMMYRKWYRRKKMKATCTYRRRWWRYRYRR
49                MYMMYYYWWTRSSYTWTAAYTTTTCYAATARMMYRGWYRRKKMKGGYYYRRRWWRYRYRR
                  **  * *** ****  : .*.     .   ***** ********.   ************

45                RGYKYYRMGYRRYCATTRYKWGGKKTAGYAYMYRWYYKGTRRTKKWGAYWYMMRWKRRWY
49                RRYKYYRMAYRGYTRGWGYKTKKKKWMACGYMYRWYYKTCRRCGGTSWYWYMMRWGRRWY
                  * ******.** *     **   **  . .********  **    . ******* ****

45                WRYMYMYYYKYYYYKYYSYRKWYRMRYWRWSWMKMCYWKKRMYKMKYWMRRYYYKGYGCG
49                WRCMYMYYYKYYYYKYYSYATAYRMRYWRWSWMKMYCATKRMYKMKYWMRRYYYKTTCTA
                  ** **************** . *************   .****************    .

45                KYTRKWWCTKCRMKCAKSTAYGAYMWYRMYKRRWGYRYYYRKYRRYMMRRATYYYKTYKK
49                KYKRKWWYCKTRMKYRKSCCTTGTAWYRMYKRRWRCRYYYRKYRRYMMRRTCTCYKKTKK
                  **.****  * ***  ** .  .  *********  **************:   **. **

45                KYGCTGMKKYMRYYTYRYKMSYKSACYRWYRRSMTRKYRCYCCKWGGKMYYWKACSKCAC
49                KYAMWKMKKYMRYYYYRYKMSYKSMYYRWYGASMARKYAYYYYGARKKMYYWKRYSKYGT
                  **.   ******** *********  ****  **:***  *      ******  ** .

45                AYTWMWKYTRGAARRKKKCTWRRKMSAWCRYRYMKWYMKWYAAAKRYYRRYCTWRRYSRY
49                CCCWCWKCCRAGRRRGKKYYWRRKMSRTYRCRYMKWYMKWYRWRKAYYRRCTWWRRYSRY
                  .  * **  *.. ** **  ******   * **********   * ****   *******

45                AGGCATCGKKCAGRTMWRKMCAAKRYWWYATGGMCGWCCCTTYRRRMKKSWGTYYRGCAT
49                RRRTCKTTKGYGKGYMWRKMTTRTRTWWYGAATAMATMGTYCTARRMKKSWRKYYRAMRY
                      ..  *  .   ***** : .* ***.:.   .        ******* .***.

45                CAGCAAAAAKAKTCTTRRMGYRWYTYCRAATGACSKAAKKYRYKKSRCTRMYKKCKWRRC
49                TRRYGMGGMTCKCTCARRMKCGAYCYYRRGYTGTSKCWKKYRYKTGGYARMCTGYKWGGM
                      . .. ..*   :***    * * * .  . **. ******..  :** .  **

45                CYRKWMYATACGWCGGRKKRAWYKCRKCATMWMAMMWMYCCKKRYYGAYRWYRKCAAAGT
49                MYRKWACGCGMRTYACRKKRRWYKTRKAGKMWMGMMWMYYMKKRYYSGYRWYRKMMMRKK
                   ****  . .    . **** *** **...***.*****  *****..******     .

45                WYYKWRYYRRKRMWWWWMKKCYMRRKKCTYCWYWSTWTAKKCGRKYGGTYRYKRGMGKRC
49                TYYGTGYYAGGGMAWWTCKKSYMRRKGYWTYWYWSAACCKKAAGKYATCYRYKGKMRKGT
                   **   **    * **  **.*****     ****:  .**.. **.  ****  * *
```

```
45    AKATRWAGKTAACRMYKWYSYCGAMYRTCKTTAMKSRMKWKMKKKKRTRKWKRRSWYMRW
49    TKMYRWRKKKTTTRMYGWYSYAATMYRKTKWYRMKSRMKWKMKKKKRKGTTKRRSWYMRW
      :*  **  *.:: *** ****..:***. *   **************. . *********

45    RYWAAMRYTYWTMRYAGYYYMMTTYYGGTAMYRKGYRSCGRKGKKKKSYYCKGYRKMWCT
49    RYWRRMRCKYWYMAYWRCCYCCYYYYAAATMYRKATACYRRKKGKKKSYTYKKYRKMWSW
      ***  ** .** * *    *    **..::****.  .  **  *****  * *****.

45    YAWTKMWWCYTRKYYCTGGAKACGTWYYKYSYYYYYTKAAKTWGKYKMRKYSKYYYYRRK
49    YRACGMWWYTWAGCTYGRRWKTMRCWYYKYSYYYCYYKGGKWAKKYKMRKYCKYYYYRRG
      *    ***            *:   ********* * *..*   *******.*******

45    MAGTGKKTYMYWTYAATRMWRMSRRKKKKKRKKWSGGTYRYKKKKRRGKWMRRSRYKKYK
49    MTTYRTKACMYTYYMMYRMWGCSRRKKKKKRKKWSAKWYRYKKKKRGSKWMRRSRYKKYK
      *:   .*: **  *   ***  *************.  ********  .************

45    KRYMRKKYMGMKKTTCCKKYRYKCKYMCGGKKYRGKAKMRYYRAWCYMKTKYKKWYRKYG
49    GRYMRKKYCRCGKKKYYKGCGCGMKYCYAKKKCGSGWKMATTARWYYMKKKYKKWYRKTK
       *******    *..  *      **  . **  .  **     * ***.********

45    RWYMKMATGWRGTTRKKGGYTGAGGTWYWRWKKMYKASYWGTKRKKWKKWWAGRYRRKMM
49    GWYMKMWCTWRSKWRKKRKYYKRRTAAYARWKKATKRSYWCKKRKKWKKWWWRRCRRKMM
       *****   **.. ***  *     : * ****  * *** .*********  * *****

45    RMACCGAKRYTKGGGRYYRRWYKKATRRAMYKKRYACYYKTRRCRRWYCRYYYKKKKWRY
49    RATTTTGGRYYTRKTACYRRWTKKTCRRMMCKKRYGGYYKCGGYARAYAGYYYKKTGWRY
      * :   . ** .     **** **: ** * ****. ***     * *. *****. ***

45    YRWGYRRYRMRGSKWAGGWYKRWYKKATYTAKKKCYCKKAAKATTWKYKKYMYRTWKYRY
49    YRTKYRRYRMRRSKWCAAAYKRWYKKRGYGCKKKTYYKKCCKRYWAKYKTYMYRKAKYRY
      **  ******* ***... *******  * .*** * **..*    ***.****. ****

45    MYCWRKKKKGRRGMYKYWSSSKKWRRYGWYSWGMYRYMWYWRYYYRMYYRRYKMWMKKYK
49    AYGAAKGKKRRRKACKYWSSSKKWRRYAWYSWTMCRYAWYWRYYCRMYYRRYKMWMKGYK
       *   * ** **   ************.**** * ** ****** ************ **

45    MMYCTCYRWAGGKCAWKRYYYKCTAYYRKYWKMKRRSMSMYKATCMMYCYKYSRATYRKK
49    MCYYCYYRAGAAKYGAKRYYYKMKWYYRKYWKMKRRCCSMCKTATMCCMTKCSRRYCRKK
      * *   ** ...* . ****** . ***********. ** *:: *    * **   ***

45    AYWGCTRRAKCKWKRKRCTWWYRYARMMYKMYYYRGRKYGYTAKTYKRYGAAYMWYRRYK
49    RCAKTAARWTTKWKAKRTGWWYRYCRCMYKMYYYRKRGCATATKYCGRYRWMYMACRRTK
           : * . *** **  *****.* ******** *  . ::*   **    **  ** *

45    RYRWYYRKYYWMKMCGATTKSACTKRKYATGKYKYYSKYKTTKKKKMYKRSGYYGGGRKY
49    RYGAYYGKYYWMKAYKTGCKSMMYTRKCRYRKCKYYSKYKCCKKKKMYKRSRTCATAGKY
      **  ** ******   :  **   .**    * *******  *********   . . **

45    MYSRMKAMMKARRSKYRYTCYKARWMKTCTYWKRYKSTCCKYACCRRKKKGKKKRYYYYM
49    MYSRMKRMMKCRRCTYRYKYCTRRWMKCMAYWKRYKGCAGKYCTTGGGKKTKKKRYYYYM
      ****** ***.**..***.  . ****  :******. . **.     ** *********

45    MSYCCTKKMAYRRKKYYKWKKMRMRYYKRRRRTWRAARRKCRRRMGCRSGKCGWKWYSRG
```

```
49    MSYTTAGKMRYRRKKYYKWKKMRMRYYKRRRRGWATGRGKYAGGCKYRSRKYAWKWYSRK
      ***  : ** ********************** * :.* *       ** * .******

45    RCKMMKMKKYATATCKAYWKCYKYYKRRWTMKYKGSRWRAKRKYKSRMSYKKKRMKKKYR
49    GYKMMKMKKYMCCATKWTTKYYKYYKRRWCMKYKRSRWRGKRKYKSRMSYKTKRMTKKYG
        ********  .: *   * ******** **** ****.***********.***.***

45    KKRRRKKCTGKTRWYRAGMYKSMTKKYKYKKYKKKRRYKKATKMWWSKYGTYWGYSYKKR
49    KKRRRKKAATKKRWTRMRMYKSAKKKYKYKKYKKKRAYGKRYKMWWSKYACYTSYSYKKR
      *******.: *.** *  **** .************ * *  *******. * .******

45    AYKKWKYGCKTRTKKTKKRRRWKKKKKTMRGYWYCMWYMKKKKTATKAGYRYYKKYKKKW
49    GYKKWKYAYKCRCKKAKKARRWKKKKKWMRRYWYTCWYMKKKKYMYGMKYRYYKKYKGKW
      .******. * * **:** ******** ** ***  *******      ******** **

45    KKMWKCRTCKAGGWKAWMKSYKWAACTTGKKYRYKRKKKRCYCKKRRYTYKKWSKRYWYR
49    KKMWKYRWYTRRSWKGWMKSYKWGCAAATKKYRYKRKKKRSYAKKRRYYYKKWSKRYTYR
      ***** *  .  .**.*******...:: ***********.*.***** ******** **

45    RKKMKKRWYKAKGRRYRRSRRYMRYGCGGYRATCKMTTYKKAKSYWYTTAKGYRWYMKTG
49    RKKMKKRWTKTGRRRYRASRGCCRYCTRRYRCCTKMCCYKKMKSYWYKAGTTYRWTAKYR
      ******** *:  **** **   **    **.  **  *** *****.:.. ***  *

45    AKSKRKKAKATACKYKRTYKRRRKKKRKTGCKCGYWWRRATTAGRKKYKKKRRMYRRKRY
49    MKSKRKKMKMWGMKYKRYCTRRRKKKRKYRYKTTYWWRRWGYRTAKKYKKKRRMYRRKRY
       ****** *  . ****  .********   *  *****      ***************

45    CYMYYTCGCWSKRAKMRYYRRKYYRRKCCRRKGMKAATYYSKGWRKWYYKYYYGYRYKMR
49    YYMCYCTATWSKRCKMRYCRRKYYRRKYMARKAMKGGGYYSKRWRKWYYKCCCSYRYKMR
       ** *  . ****.**** ********   **.**.. **** *******   .******

45    YWWKKCRCKKKMKRMATARRYMRYYMYTARWCYCCAYKKKKKSKYRKKRYKYCGWARGGT
49    YWWKKYAYKKKMKRMCGMRGYMRYYCCYCRTYTGATYKKKKKSKYRKKRYKYMAWCAAAC
      *****   *******.  * *****   .*    .:**************** .*. ..

45    AAAAYRYMYKYYWSMRKKYRKWWTMMYKKMCYYTSYRGAATKKAAAGRTKKGCKKRKRYY
49    RGTTCATMYKYCWSMRKKTRKWWYMMYKKMYYYCSYRRGWYKKCTGAGAKKKYKKRKRYY
       .::   **** ****** **** ****** ** *** .  **.:.. :**  *******

45    RYYWMYRMWMMWWYYKRMWMMYWWKKRYRCGGRMTTAKYKAGYMKKKATWKGRRMYRRRR
49    RYYWMCACWMATTCYKRMWCACTWKKGYRGKRRMYYRKYKGCYMKKKMKWKARRMYRRRR
      *****   **    *****    *** **   **   ***. ***** .**.********

45    KSCRYGKYYRRKYMMWMYKKCRWYWWKSSGRRYYYWMMRKYYRCMRYYMKKKGRKTKACG
49    KSMGTRGYYRRKYMMTMYKKYRTCTTTCSKRRYYYWCCGKYYRYMGYCMGGGRRKWKWYK
      **     ******** **** *    ..* ******   **** * * *    ** *

45    AMRRSKCAKKTKYTKSMRYRRGYRYYSKRTCCWYTMKGCMSYYGKGTRGTCWGWYMKKYR
49    GMAGGKYTKKCKTAKSMRYRRKYRYYSKGYTYAYCAKTAMSYYAKRKACWTTAWYMKGYR
      .*  .* :** * :******* ******     *  * .****.* .     .**** **

45    KYKTGTGCKKRYCGRKTTRWMMYWMTAKACTYYYRKYYYYMYTCYMKYGKYKKKMKKGYT
49    KYKGAARTKTRYYRRKWWRWMMYAMCWKGTAYYYRKYYYYMYATTMKYRKTKKKMKKACA
```

```
                    *** .:  *.**  **  ***** *  *. :***********:  *** * ******. :

45                  YKRTYTTATYYWYKKWKYWRTWKATTTKKYKRKKKRYKKRKKKTCTGAGCRYRYKKRMKY
49                  CKRGYACTCYYWTKKWKYTGKTTRYYKKKYKRKGKRYKTAKKKWYWTWTTRYRYKTRMKY
                     ** *: : *** *****  . .   .****** ****. ***      *****.****

45                  KRYMKAKYWRRAAWKRWRYRMKWYMWKRKYKWGTKRTYRWSRRKRKAAMMRRMACYRRKK
49                  GRYMTGKYWRRGTWKRWRYRMKWYATGRKYKAKAKRKYGAGRRKRKRRACRRMMTYRRKK
                     ***..*****.:***********   ****  :**.*  .*****     ***  *****

45                  KKCRRMMRRKKKMYKGKYWRMRKMRMKYKMTTATKWYWWKRRSMWMTTTTCMYTRKRKCT
49                  KKAGRMMRRKKKMTKRTCTRMRKMRMKYKACATGKWYWWKRRSMWMCCCGYMTARKRKYY
                    **. ********* * .   **********  :: ************     * :****

45                  TKMKRKMRYRRTMKMRKYKMTATGWCMTMKRRYYKRKMTWYRRKKKCYYTMCYCGKCTMK
49                  WKMKAKMRYGGAMKMRKYKMWMYRWYMAMKARYYKRKMCWYRRGGKYTCCMAYYKKTYMK
                     *** ****   :********    * *:** ******* ****  *    *.*  *  **

45                  KRWMRKAAYAWTRGGGGRAYYGMCYRSRKKTGKRWSYMTWTYWRRRRRKYKKYRATMRRC
49                  KRWMRKRRYGWCGRKAARMYYRCYYRSGKKYRKRWSYMGACYWRGRRRKYKKYRMKMRRY
                    ******  *.*    ..* **   *** **  ******   *** ********* .***

45                  CKKKWYMKKKCKSKTTMWWKAAGRRMYYRYKKGTATYKYRKKGKKRKYYMSKMMYYYRYW
49                  YKKKWTCKKKMKSKKYMTAGMGKRRMYYRYKKKAGCYKYRKKRTKRKYYMSKMMYYYRYW
                     ****  *** ***. *     . ********* :. ****** .***************

45                  RRKKKGYYWRKMYKYRMRYSRWWRRWYKRKMYYRMYYKKTAGWYGRATTTTAKWKMYRCW
49                  RRKKKRYYWRGMYKYRMRYSRAWRRWYKRKMYYRMYTKKYRRWYAGGAGYWRKWKMYRYW
                    ***** **** ********** ************** **    **. .:    ****** *

45                  RAKMRKYATYATATTKAKKYKKYKCTGGGATYYKYKKKMAAWKKKWYKWTGCYCKRKMKYR
49                  RWKMGGYMCCRYRCCKGKKTKKCGYGCAACGYTKYKKKMWWWKKKWYKWCTTYMKRKMKYR
                    * **  *         *.** **     ... * ******  *******   * *******

45                  YMKYKKKKKKSRWRRAYRKKTKYYAKYYYRRRYRYYWYCMMWAGWKKRYKRRRWRRSYWY
49                  YAKYKKKKKKSRAARMYRKKCKYTRKYCYRRRYGCCWYTCCWRRWKKRYKRRRWRRSYWY
                    * **********   * **** **  ** *****   **    *  ****************

45                  MYKGYKKKRTTTTGAAAGTAYKKRYRRWRCAKYAKYYCARTMGAYAWKKTCGCRYYKCAA
49                  MYKKYKKKGYAGAAMGGTATYKKRYRRWRYCKYGGCTTGGKMRRYCWKKYTSYGYYKYTG
                    *** ****   : :. .. ::********* .**.     . .*  *.***   .   *** :.

45                  RRYKKCCTYYWAYRSKWWKYSTRWYYWYGYKWKGGKWWKCRYKKKRCCAAKYYYMMMYKT
49                  RRYKKYAGCYWMYRSTWWKYSKRWYYWYRYKWKRKKTTKYRYGKKRYYRRKYTYMCMYGW
                    *****  .   ** ***.*****.****** ****   *  * ** ***     ** ** **

45                  GKGCGTYKKMKWKKYYAKMKRRRRTYWKYGKYTCGMYWKGATCYRMMRKYCAGRRYKYYM
49                  KKKTRKYKKAKWKKYYCKMKRRAAKYWKCRKYYATMYWKKRYAYRAAGKYTCTRRYKYYM
                     *    .*** ******.*****   .***   ** . ****    .**    ** . *******

45                  KKRKRKRKTKTGTSTYGKKKKMKYWYTKKWTGYCGAKWYSWRRTMRMRKCAYYCACGGCK
49                  KKRGGGRKWKKTCCWCKKKGKMKYWYYKGTGRYMTCKWYGWRRYAAAGKYRYTTCTAAYG
                    ***    ** *.   .    ** ****** *     *   .***.***      *   *   . ..
```

```
45      GYKYYYRRTARRCYYKYYRMRGKYWYRKASKKGKYMKGYYACYTGKYRKYRKKWTCRSMK
49      KYKYYYRRCGGRYYYKYYGMRRKYTYRKTSKKRKYMKKYYMYYATTYRKYRKKWAMRSMK
         ******* . * ***** ** ** ***:*** **** **  *: .********: ****

45      KKCGRYAAYRWAKWAYKWTWGWYRTGACYRWKYKCAAKYRYKRKSRKCCTGATKKCSMKK
49      GGTRRCMWYRWMKWRYKWGAATYGYAGAYRWKYKTTGKYRYKRKSRKYYYRWCKKMSMGK
            *   *** ** ***  .  *  ...****** :.**********      ** ** *

45      YRYRWKMKKCGACGYYYSWTYRRKATAGSYSMGKSMRYYKRMGAKRTCCMYYRRRCKAYT
49      CRYRWKMKKYKRYKYYYCTACRRKCCTKSYSMRKSMRYYKRMKMKRKGMCYYRRGYGGTY
         ********     ***. : ***. : **** *********  **.   ****   .

45      KGRYYYYTYATARSRKYCGSGCTGTTCYWKKYRTTGTKTWGTYCKYRYRWRTMYKYMGTW
49      KKRYYYYKYCCTRSRKYYKCATAKYWSYWKKYGWATAKGWTWYMGYRYRWGKMYKYMKCW
        * *****.*. :*****  .. :   .*****  : :* *  *  ***** .*****  *

45      YRWTMWKYMYKAWYRWYTKRRWMMRTGMRYSCCTRGGAGKMMGGYKYRYKKYYKKRCRMK
49      YRWYAWKYMTKCWYRWYGKRRWMMRKKMATSYTAGRAMKKACAAYKCRYKKYYKKGYGCK
        ***  **** *.***** *******. *  *  :  .  *  ..** ********    *

45      KKYYSRKMKYYKRTKYMKRKACMTTWTRYKKKRKKYRRRKKYSRAAGCMRKATCAKKKSW
49      KKYYSRKCKYTKAYKYMKRKRAMYCTAAYKKKRKTYRRRKKYSAGGTAMRKWWTGKKKSW
        ******* ** *  ****** .*   : ******.******** .. .***   .*****

45      KWGAKWKCRYSMMKRKKKKRKKRAYSYYKTAKKKTAYRGKTRRMYYKMKCGYKGCTWYRK
49      TWTCKWKMRYSMMKRKKKKRKKRGYSYYKCCKKKYRYRKTCRGCYYKMKTATGASYWYRK
        .* .*** ***************.***** .***  ** . *  ***** .  .. ****

45      SGRYAGGWGTYRTAGCTAYRRYACCKRYKRKRCMYWCYASYTRRGRRRYGRCKMYYACMA
49      SAACTAKWKACAKWSAAGTRRYGMYKAYKRKRYCCAMCGSYYRRKRRGCKRYKMYTGTMM
        *.  :. * :  . ..:. ***.  * *****      .** ** **   * *** . *

45      TCAMTYCRYKYRGRTATGGTATRWRYYSWSK
49      CMMMWYTRYGYGRRAGGAAYRARWRYYSWSG
           * * ** *  *:. ..  :********
```

The pairwise alignments of two accessions 45 and 50

```
45              YKRTTCYYKWCCRWWRMKRSSWTWWCYYACGTATMCYRWYYYYYYAWRMRKYCRKYRYRC
50              CKRYYTYTKWMMRWARMKRSSWYWWYYYTATYWYCMYRWYYYYYYCWRARKYMRKYRYRG
                 **   * **  ** ******* ** **:.      *********.** *** ******

45              TYWWGYWWYGWWYKMCGGWKWAKYYYYRMMRRYYMKMWWRYRMKRMYYRMMKYRRAMKRY
50              AYWWKYWWTAWWYGCTTRTGWGKYYYYRMMRRYYMTMWWRTAMKRMYYRAAGCRRRMKRY
                :*** *** .***       *.*************.****  *******    ** ****

45              KKYSRYRKSWYWWRMMYGGYWRCKMYKYMYRWRCRWWWYRRRWKGAKKRYYKCGKMYWKK
50              KKYSRYRKSWYWWRMMYKRTWRYKMYKYMYRWRYRWWWYRRRWKRRKKRYYKYTGMYWKK
                *****************   ** ********** **********  ******   *****

45              RWMMYYGWYRKRRTKKKWYWRYYYYYKKYYSYRKWYMWRWAGCCTRYYRRWGYTYTAYTG
50              RWMMYYRACRTAACGTKTCTRYYYYYKTCTCTRTAYMWRWWTYYYRYYRRWRCGYCGYGT
                ******   *.    .*   *******. .  *. *****     ******   * .*

45              AYTACYKMKYGAGTWRRGSMMYKCKMKRYRRMGCTYRKMKYYRYRYYRYKGGTKCMYYKS
50              GTCTTYKMKYAWRWWGASGMMYKTKMKRYGGMRTWCRGMKYYRYRCYGCKTKCKSMTYKS
                .  : *****.   *  ..**** *****  *    * ******* *  *   *.* ***

45              KRMKWAYWKSKKYYTRMMMRMTAKMYKKRYKYSYMYCWMYWRYKWYMRYYMYCAMRYWKR
50              TACTWGTAGSKKYYGRMMMRMWMKMTTKRYGYSYMYMWMYWRYGWYMRYTCYAGMGTAKR
                .  .*.   ***** ******  ** .*** ***** ****** *****  *..*   **

45              WYMGGRMWSKYGWYYACTTAATTMMMCYYRSWTRKKYYYYKKYKWYMTYWYARMWRMYYT
50              WYCRRRMWSKCRWYYGAGGTGAYMMCYYCAGWCRKKYYYYKKYKACMKYWYRRMWRCYTK
                **   *****  ***..  :.: **  *  .* ***********  *.*** **** * .

45              KTSRRKKKTYKKRRYYWSYRYMKTCTGCRMRRKRRSRYYKTGTCCKGTATKRYTRRMKKT
50              TKSRRKKKGTKGRRCYWSYRYMGWYKSYGARRKARSRYYKGSAYMKRYMYKRYCRRMKKW
                ..******  * ** *******    ..  *** ****** .:   *    *** *****

45              TMMKKYRYRRKKRATKWKWKKWYRKYRMKYYYKKWRSKKYYKAGGAKKYRWWRKRKYKKS
50              YCCGGCGCRRKKARGKWGWKKWYRKYRMKYYYKKWRSKKYYKCATTKTYRWTGTGKYGKS
                        ****   ** ************************.. :*.***  . ** **

45              YWKKTRRRMRYYYKKKWYRKYKYYSKCTCAGGTTKYKYKKKCRTKTWTYKKWGTTYYRRR
50              YWKKCRRRMRYYYKKKWYRKYKYYSKACTGRCCCTYKYTKKMACKYTWYKKWRYWYYRRR
                **** *********************.   .   .***.**   *   ****   *****

45              ACRWMRKKYYACYYKKGTAMMYRCWACAWMKMSRYMMKKKKATKKYRYKRYRKYMKTKYR
50              RYRWMRKKYTTGTTGKRWCMMYRTWRYGWMKMSRYMMGTKKTCKKYRYKRYGKYMKCGYR
                  ******* :    *  .**** *  .********* .**: ******** ****  **

45              ARAMRGCATAMWGGRYYYTMYRMRMYYWRYMYWARGAGKSYRMRRACCAKTACYACAGRR
50              MRRMAKYCGMMAKRRYYCYMYRMRMYYWRYCCWWGTCAKSYRARRTTAGKYGTYRYRARG
                 * *   .  *   ***  ***********  *   ..**** **: ..* . *   .*
```

```
45          WRKYRYGKYKTTTTCTAASKMRKYKKMCTRRYKCCCTWSRRKWRRYRGWWRYRKKKYYGR
50          ARKCATRKYGCGYWMYTWCKMRKYKKMMYRRCGMMTYWSRRKWRRYRTWWRYAGKKYYAG
             **    **       : .********  **      ********** ****  ****.

45          TRWRRRWSTAAMWRYKWYGAKRRRYRKKGWCRMAATMCKGKRYWRMWRMRKTRKMMRMKT
50          YRWRRRWSGTGMWRYKWTKTGARRYRKKATMRCWWYMMKRKRCAAMTRMRKAAGMMRMKA
             ******* :.******  :  ******.  *    * * **   * ****:  *****:

45          TTGACAGTYRKYMYRRKRSSYWKKMAAKRRCMYKMKYRAMRWRSYKKYWSYRRKRKRGTY
50          YGRTYCAYYRKCMYRRKRSSTTGKMGCKRRTACKMKYRTCGTRSYKGCASYRRTRKGKWC
               : .. *** ********   **..***   *****:   ****   ****.**

45          WYRYRMMYYYMYAKGMRYGYWRMRGGYTGGWRWKRYKKYKYWKMYYKKSYKMRTKSMKSK
50          TTACRMMYYYMTTGKMGCKYWRMRARTATAWRWKRYKGCKTTTMTCKKSYKMRAKSMKSK
                ******* :  *   *****.  : .*******  *  .*  *******:******

45          KKYYCAKWGRRRYKYKKGYYKAAARRKWKYYRCYRMRGWSTCGTTKRWKGGRYWKYYYRM
50          KGYYAGGARRRRYKYKTACTGGRRRRKWKCYAYYRMRAWCCMKYWGRWKRRRYWKYYYRM
            * **..   *******..   .  ***** *  ****.*.      ***  *********

45          KKAAWARYWWYGYYYKYMYKKGYCARAGYKKYWAGSKKWWWWYWTRRAATTGRGYSGMAY
50          KKCCWWRYWWCRTTYKYMYKKCCTTAGRCKKCWGRSKKWWWWYWKRRWWYARRAYSTMRT
            **..* ****    *******   : .  ** *. *********.**   : *.** *

45          YWYWRYRYYYRRYMWMYRYMYYRKYMWMYKRWRKMTARRMGYGTAGCTTKYWWYYKMKKK
50          YWYWRYRYYYRRYMWMYRCMYYRGCMTMTKRWRKMCCRRMRYRWWTTYKKYWWYYKMKKG
            ****************** ****  * * ****** .*** *      .**********

45          RRKWKRRSKWMRYWMRARYYYMGGRYAYCCYKRATKMAATGTGGKCGGACCKTSYYGCKM
50          RGTTKRASKWMRYWMRRRYYCCSRRYRYYYYTGCGKCGCACWRAKYARGTMKKCTYATKA
            * . ** ********* ***  . ** *  *. . * ..:   .* . .  *.. *. *

45          RRKTCKWAWGGTWATWWRKGACGTWMTYKYACGKYKARWKCGYMRYMRKMRRRKSKWKMW
50          GRKCTTWGWKTCWTCTWAGKWMRYWCCYKYGAAKYKGRWKARYMRYMRKMRRGKSKWKMW
             **  .*.*   *:  *       *  ***...***.***. ********** *******

45          WKATGRKYMMKYTATCKTCGGTGYMWKYRYYYKTTRKRWYKRWRYAAMTCKKTAKATGKK
50          WKTAARKYMMKYYCATKWYTRWRTATKYRTTCKCYAKRWYKRWGYGWMAMKTACTRWKKK
            **::.******* .: *         ***   *   ******* *. *: *.:..   **

45          TKRRRYYKMYRMKYKGKMYTKYKRRKWYRTGKYGYRKATTRGAGAGRYRRMYCYWKTKKK
50          WKRRGTTGMYRMKYKKKMYYKYKRRKWYRGTGCAYRKTGARKGAGARCRRMYTYWKCGKK
             ***    ******* *** *********    .***: :* ....* **** ***  **

45          YYKRRKKRRWRKYTGRYKRMRWMMYAGGYYTMYYYMKRYWKGYKYKKRYYSRRRYYYRMM
50          YYKRRTGRRWRKYKKGTKRMRWMMYRRRYYCMYTCMKRYWKCYKYKKRYYSRRACTCRCA
            *****. ******.   ********   ** **  ****** ***********    *

45          YYYKKWYRTRWYYWAYCYKWYYYKKKTMKRYKKCCAAKKGYGMMWMKKRGKKKKYYKYMR
50          TCCKKWYRKAAYYWRYYYKWYYYKKGCMKRYGKYYRRKKSYRCMWMKKGRKKGKYYKYMR
               *****.  *** * ********  **** *    **.*  *****  ** *******

45          TYMYWRYRRYKWKWWYRKKRKKKKKMMYRWKKMYYKKKTYWGKYCYRMKKYRYSWWYYKY
```

```
50  CYMYWRYRRYGWKATTRGTRKKKKGMCTAAKKMYYGKKGYATKYATRMKKYRYSWWYYKY
     ********* **   * .***** *    ***** ** *  **. **************

45  RYYACKKYRMGRGTKRKKGTACKWTTMKMAMMYTGYRKYRRWKYKWKKKWRKWYMWMYWK
50  GYCGTKKYGMRRAYGRKKRWRYKTCKAKMCCMYATYRKYRRWKYKWGKKTRTAYCAACWK
     * . *** * *.  ***    *  . **. **: *********** ** *. *    **

45  KRSRYCKKRKGAKYYRYTRKMYKKKCCCYKRWKMKKAKKRKMRYYYKAYKAKSKGCGRKR
50  KRSRYTKKRKCWKYYRYGRKMYKKKATAYKRWKMKKWKKRKMGYYYKWCKWKSTKYRRKR
    ***** ****  ***** *******. .******** ***** ****  * **.   ***

45  YKKYYKKRKMMYRRTWYTKTGKKTTCTWCGMAGTTYRCGAGTMRMYKSTYARCTGRARSY
50  YKKYYKKRKMCYGAGWYKKYRTTYCSYWTAMRRKKCRGRGSYMRMYKCCYGGYCRGGRSY
    ********** *   **.*  ..  . * .*  .. *  .. *****. *.     .***

45  TKRRYTATAWGTTGCGCAKYCAATRGYYRWRCTACWTGTWRMYAYRYYKKWYRKMYYYWK
50  ATRATYRWGAKCAAAKYGKCMMCKGSTYRWRMYRYWYRYWRMTMYAYYKKWYRKMCTYWK
    :.*     .   :..  .*   .. . ****    *   ***  * *********  ***

45  YTACYCAGKYWMRTGGYYWYYAKKKKYRKRRRYKKYKWKKKYRRRAKKKKWACCCKKKMC
50  CAGYYYGAKYWCRYAACCWCYCKGKKYRKRGRYKKYKWKKKYRRRWKKKKTGTTTKKKMS
     :. * ..*** * ..  * *.* ****** ************** **** .   ****.

45  RAKYRTAMKKAYYKRKKKAKKKRRKKYCKCARKYMKKKKKRRMKYWRWRKKKWRYKWYWT
50  RRKYRACMKKCYYKRKKKWKKTARKKYMTTCRTTMGKKKKRRMKYWRWRKKGWRYKWYAK
    * ***:.***.******* **. **** . .*. * *************** ****** .

45  TGTMKARRRRRMYYRRKKKTTGAKKKRWKYRYRYRRKWYWYKKKKRRSMKWMRKRKKMRY
50  CCGMKRRRRRRMYYRRKKKGCCRKKKRAKCRCGTRRKTTTCKKKGRRSMKWMRKRKKMRY
       ** *************    **** * *   ***    *** ***************

45  KMMRKYKCMMYYMYCYRYKMAGMYWRYRTYYMYRMRKAGKKRKAAYTKTYRRRYWWWTGC
50  KMMRKYKGMMCCCTMYRYKMCTMTARCGCCYMYRMRGGAKKRKTWYAKKYRRRYWWWYRY
    ******* **     *****. *  *    ****** ..****: *:*.********

45  KRYAKRSYKMWRRMYSRWRYRKKCARCYSKRYRRKMKYAMWRTKKKWMRYYKYYMRRYKA
50  KRYRKRSYKMWRRMYSRWRYRGGYRGTYSKRYRRKMGCTMWRWKKKAMRYYKYYCRRYKR
    *** *****************      *********  :*** *** ******* ****

45  GGCAATARKCTAYTCCTYYSKKACYMMAASTKMMRMYYRYKKYYCKMKKAKYRKYMKMMY
50  KRTTTCGRKYYTTCTAGCTSKKGTTCCGCCWKMMRMYYRYKGYTYKCKKRKYRKYMKMMY
       :: .**  :   .   ***.    ... ********** *  * ** **********

45  GYWATWRRKYRKMKYSRCYCKYGGMYKYWWYSYKCRGWYRGKRKKMRKKAGKTTMWKTYY
50  TYAWYTRRKYRKMKYSRTTTKYAAMYKYWWYSTKAAAWYGRKRKKMGGKRKKWKMWKCYY
     *    ***********   **..******** *. .**  *****  *  * .*** **

45  RCKYRRKYKTAACYYKYKKKKRWKKYGTCRTYYRYRYKRYYRKTGTYRYRKMYRCWWSMM
50  RYKYRRKCGCTGAYYKYGKKGRWKKYRYYRYYTATGYKRYYRKCCGCAYAKMYRMWWSMA
    * *****   :..**** ** *****   * *    *******     * **** ****

45  KKKYKKYGCRCMYKRKGMRRWWKKSMWYMKTATKYAYRYMKTAAKWKWYRGRTYCKMWKK
50  KKKYKKYAMGTMCKRKKARRWWKKSCWYMKYMYKYGYRYMKYGRGWKWCGRRWTYGMWKK
```

```
        *******.   * ***  ******* ****   **.***** .  ***   *    ****

45      RWMKSYKKMKRYKKRKRRRYSWTMKYKCYYKKRYWGWRYKKRRWSKRYRYRRRKKMKKKK
50      RWMKSTKTMKRYKKRKRRRYSWWMKYKSYYKKRYWATAYKKRRWSKRYRYARRGKMKKKK
        ***** *.************** ****.*******.  ************ ** ******

45      CKAGRYAYMRRGTYCRGYKSGCTGCCTKYCCCYGTYRGTSRYRMMGCYWRRKRKYKRKMA
50      GKGTRYMYMRRTCYMRRYKSSTCTATAGYYYTCACYRRYGGYRMCKYYWRRKRKYKRKMR
         *. ** ****  * * ***.   . : *    . **  . ***   ************

45      WMMRTYRRKATYMYRRGRKKACCYACGSCCCTCRCYCKKMYKKMAGRWCGGRYKKRAYRG
50      WMMRKCRGGRCYMCGGCGKKTAMYTGTGATAYYGAYAKKMCTGMWRATYACRYKKRMYGA
        ****. *    **     **:. *:  .. .   .*.*** . *     . ***** * .

45      TTKYRTRYGAKKKWMYGGGCTKKRYYKWKTAGTWTMRRYRMKYYKAKSMWMKWYRYCKYG
50      WWGTRCGCKGKKKWMCCTRTAKKRYYKWKGGTGWYMRRYRMTCTGMTCMTCKWYRYMGYK
            *    .*****     :******** .  * ******.    ..*  *****  *

45      CGYYRWYKCTMTYRSYAYKTMKYYSWYYKKKRWGWCCTAKMYYMRSRRYMRYYRKMYKKK
50      SACYRWYTTYMKYRSYWYKGMKYYCWYTKKGRWKWYGCGKCCYARSGGCCRYYRKMYKKK
        .. ****.  *.**** ** ****.** ** ** *   .*  * **    **********

45      KACYKSYWYKKKYKWKCKKRKYWTTRRRKRWKYYRYYRRYKYYWKKRRRWRKRYRCTYRG
50      KRMCKSYWYKKKYKWKMKKRKYWYARRRKRWKYYRYYRRYGCYWKKRRRWRKRYATAYRK
        *   ************ ****** :***************  ************  :**

45      YYRKKCCRRKMKRAYGMKWRWYYYYYRYKYWKKKTKKRYYGTCAKCYKMGTKRYYGAAYK
50      YCRKKTMAGKMKRCYRCKWRAYYYYYRYKYWKKKCKKGTCACGTKYYKMCYKRYYSRRYK
        * ***    ****.*  *** ************* **   .  :* ***  ****.  **

45      RRYKRMWMMYWKRGCCGKKYTYRGKRMRKAACKMKWKYYRRRTCKSKRKKAYRRKYMMKT
50      RRYKRMWMMYWGGAATKKKYWYRKKRMRKTTTKAKWKYYRRRYAKSTAKKCYRRKYMMKA
        ***********  ..  *** ** *****:: * ******** .**. **.********:

45      RKGRCGCCCRKSSKRYTTYKYRGMKRKKKMKKWYTRRSAATTTCAKKKKKYYKAYKYYYS
50      GTKGMTTTTRKSSKATCYCKYRRMKRKKKMKKATYAGGGTGGYYGGKKKKYTKRYKCYYS
         .       *****     *** *********     ..:    . ***** * ** ***

45      KKTCGRTTKKKTTGCTMYRKRYKRMRKYSKTMMYRRSRAARGGCTTKGKYRWRCSKYKAW
50      KKCGARCCKKKCGRAAMYGKRYKRMRKYSGKMMYGGSGMGRACAWKKKKYRWGYGKYKMW
        **  .*  ***   .:** ********** .***  *  .*. . .* ****  .*** *

45      YKMMKMMRKRMRRKWRYYKKMMRYYYAGGKMR
50      YKMMKMCRKRMRRKWRYYKKMMRYYYWSRKMR
        ****** ******************* . ***
```

The pairwise alignments of two accessions 45 and 51

```
45      AAARYGRKYYYKYRRGYRRGGYAMYKKRMYYYWKYYWYMRYWTYGCAGCTATYKAACYMW
51      CGGRYRRKYYYKYRRACRGRRTCMYKKRMYCYWKYYWYCRCWYYATRRTYRYYGWMYYMA
        ...** *********. *    .******* ******* * * *.       *    **

45      KRGYRYYRYCYYTCGWYWWRMYKYWYAYKKWMTASKYYRCRGYRWWRYWYKWYRYGCYKY
51      GRKYRTYAYTYYCTTWTTWRMYKTWYRYKKWMCGSKYYRTRAYRWWRYWTKWYRYRYYGC
         * ** * * **   *  ***** ** ***** .***** *.******* *****  *

45      ACRGYCRMYGAYKGCTYTMYKMKYKSTARRRKRWTTGWCTWYRWSRGYYMKRYSTRYRMY
51      RTRKYMAMYKRCKCYACGMYKMKYKSWMRAAKRWWWRTAAWYRWSRKTYMKRCCYGYGMT
          * *  **   *  :  ********  *  ***    .:******  **** .  * *

45      STKGKWRSKKKTWYYKYMCMRYMRRWRKMYKRYWKTTCYYMKRWYRYYWMTRYMKTWCWW
51      GKGSKWGCTGKCWTTGCMTMRYMRRWAKMYKRYWKACAYYMKRWYRYTAAWRYMKYAYWW
        .. .** .. * *    * ******* ********: .*********    ****   **

45      YRACACTGKWRYRYRRWYYRKWRRRMKRRKYYRRRRTCYTTKKRRSWACAASGYRTRTAM
51      YRRYMYYRKWRYRYRRWYYRKWRRRMKRRKTCRRRRGTYYYKKGRSWWTTGSKCAYGKMM
        **      **********************  ****  *  ** ***  :.*     . *

45      ATWMMMGRYGGMMRKCAYAKKYRRCKCWMYWYYRYCYWKMMRKKKGYYYYYYRYYCYKRY
51      TAWMMMRRYKKMMRKTGYRKKYGGYKYWMYAYYRYYYWKMMRKKKSTTTTCTRYTYTKGT
        ::**** **  **** .* ***   * *** **** *********.      **   *

45      WAAMWWYTYKKWWYKYMRYGWRWWCTAKCGTRKRSSRCTKWRRYKKMCYKKKSYWYAAGT
51      ATWMWWYYYKTATTKYMRYKTAWWGCGKMRKAKRSSRMWKWRRYKKMTYKGKSYWYWRKK
         : **** **.   *****   **  .*  . *****  ******** ** *****   .

45      TGKRYMKGAGGKKGAAYKRKYWRRRRKKGTGSYRYKKYRAAGWRYRKWKCKRRRWKRKGG
51      YKGAYMKKCATKKTWWYKRKYWRRRAKKRKRSYRCKKYRGTCWRYGKWKMKRRRWKAKRR
            *** .. **   ********* ** . *** ****.: *** *** ****** *

45      CRYAMWRYYGRCYYYAWTGCSKRCWYKRYYRRAAKKMKYSKWRYYYYYYRRYYWWRYWYW
51      YRYCAWRCCAAYYYYTTAAYSKRTWYKRCCARRRKKMKTSKWRYYYYYYRRYYWWRYWYW
         **. **  .  ***: :. *** ****   *  **** *********************

45      KYCKKRCYKKSRYTKRKTTGAYWKWGGRWCGGTRKRWWRTYKCAMYWRRWMKRMAKMWMY
51      KYYKKGYYKKGGTKKRKWYRRTWKWKRATYACKRKRWWRCTKMRMCWGAAAKRMGKMWMC
        ** **  ***.  .***     ***     . .******  *  * *    ***.****

45      GGRKYAKYTRGCAMTRCTYYCYTTKRRKMAKWKKSKMGKKYAWYWYCWTSKYYWTTAKYY
51      TRRKYGTYAGTTTMGGGCYYSYWYGRRKMCTWTTCKMKKKCRAYWYTAYCKYYWYGWKYY
          ***..*:   :*    **.*   ****..*...** **   ***   .****   ***

45      KWYGWYYKYKCMRAYYMMYYRYRRAKKARRRGMKSKKKRYGCAARYTKKGGRCMCKKWRK
51      KWYKWTTKYKTMRGYYMMYYRCAGRKKMARRKMKSKKKGCAATRAYKKKARRMMTKTWRK
        *** *  *** **.*******    **  ** ******  ..:  *.**. * * *.***
```

```
45          YRMRSYTWKTRWYCCKKYRMMKGAGCAGWKYKTYKMYGKWRYWARKKRKAGSKKWRRAAY
51          YRMRSYCWKYGTYTAKKYRMAKRCRMCAWKYKACKACRTAGYATRKKRKGRSKKWRGTWY
            ****** **   * .***** * .  ..****: *   .  * :*****. ***** : *

45          TYSKWAGCCGMTYYRWKYWRYYYWYYYRRYKKRMTAWRKTGAAKKGGTMKGTYKYSRRYK
51          KTGKWGAYMTMYYYRWKYWRYYYWYYYRRYKKRMCCWRKCAGCKKRACMKKYYGYSRRYK
            . .**..   * ********************** .*** ...** . **  * ******

45          CYRYRKRWSWCCTCWKWRTWYYYKTAWMGWKKKGKGCCACAYYKYYMMMRWAGCCAAYRY
51          TYRYRKRWSTTGKAWKWRKWYYYKYWAAKWGGKRKSYYWYGYYKYYMMMRWMRYYWRYRY
             ********   ..****.*****     *  * *.    .**********      ***

45          MMYGTTMRYMYKKKRYYKRKYYRWKTGMKKRRYTCTGKRYYGASYSWRRKRYKKRYYKRM
51          MMYKYYMATMYKKKRYYKRKYYRWKYRMKKRRTATKRGRYYKWSYSWRRKRYKKRYYKRM
            ***   *  ****************  ***** : .  ***  *****************

45          GRCTAAKKRYYGTCKKGCKMYKYRTKRMMMYWWCKTRRTCCTYRSYWYYKCYWWRMYRRR
51          ARYCMMKKGTCACGKKKYKMYKYGYKRMCACTAYKWRRKYTCYRSTWYYKAYWWRMYRRR
            .*    **   .  **  *****  ***      * **.   *** ****.*********

45          WRSYRRKAYMYYYYWWCCTKTCCGRRCCCCCSYYRKRSKRYSYKRYYMYRKKMRGKRRSA
51          WRSYRRKGYMYYYYWATAYKATYRRRTTMYTSYYRKRSKRCSYKATCMYRKKMRKKAGGG
            *******.*******  . *:   **     ********* ***   ******* *  ..

45          RKTCCKAAYYKSMMYRYTKTKGWWRGTYYRAGAYRYRYKYWKCGRAGTKKYKRWKKASYG
51          GKAGTKCGYYKSMCYRYYGYGRWWRRKCTGGRRTRCGYKYWKARRGSYKGTTRWKKGSYK
             *:  *..***** ***     *** .   .   *  *****. *.. *  .****.**

45          MYAKGCYGGTRRAWCRTTGAMKKTMYGGMAKCRWRYACSYYKYRGCRKKGKTYRAGAGRY
51          MCCTKTYARCRRGAYGCAAGAGTYCCKKMRGTRWRCTASYCKYRKTRKGAKATRGAGARC
            *  ..  *.  **.    :..  .     *   *** :.** ***  ** .*: *....*

45          RAKRKKGTTYKTKKYRKYKRYRWCATCKACAAKCYAYGYGTKRTMRWACGYYCACTYCYY
51          RRKRKKRKYYKCTKYRKYKRYRWAGATKRTGGGYCRYATAAKRACRWRMRTYSRYWYTYY
            * **** . ** .**********..: *  ..    *. .:**: **    *.   * **

45          YYTMYRCACKTRYYSRKWKATTYYMKKYCYRYARRYGYKKTYYWGMRMMAAGCYMRWKKR
51          YYCMYRYRYTKRTTGGKAKMWKYYMKKYACAYGRRCKYKKAYYWRCGAMTCCAYMRTKKR
            ** ***   ..*  . * *  .******.  *.**  ***:***    *:. .*** ***

45          ATGGKTSRKKAWMKYKWYGGAGTYGACTARGWCRRMYRRWKSWTAARMSWGTACYRWTTT
51          TCACGYCRTGCAMKYGATAKTKATARYARGATMRRCYGRWKSWAGTRMSWRYMTYRWCWW
            : .   .*. . ***   . : : .  :  .  ** * *****:.:****    ***

45          MMKWRMWRGSAMTTCAWYRKYKKRKTYGTCRMSYRRRMTTWTKTYKWYMYRRRMKSWMRT
51          AATAAATGKSGMKWYMWYRKCKKRKCCKCMRMSYRRRCCCTAKAYKWYMYARRMTSWCGC
              .      *.*.   **** ****     *******    :*:****** ***.**

45          TAKMRYYKKWKMMWYRRKKAGRYMKKTKKRYSYKYMKKYRTCMMKMRTYRKRKMAAAMKR
51          CCKCRYCGKWKMMWYRGKKRKRYMKKCKKRYSYKYMKKYRAMMMKMRYYRKRKMGMGMKR
             .* **  ******** **  ***** *************: ***** ******. .***

45          RGCCSYKMASRCCMRWCRSYGGGKRKYCCKCKKGRRRCACYKRYWKCTGTYGWYRYTRRM
```

```
51      RKATGYKMMGAYYMRWYRSYKRKKRKCYTKTKTCRARYRYYKRYWKMWRWCKWYRYYRRM
        * . .*** .   *** ***   ***   * *. * *   ******      **** ***

45      CGRCKMCRARTGRWRYAARYGRWARWTWWWRYKGKSKRKMRYCMSYYYRAGGKRRYKCRS
51      YRGMTCMGTRCARWRTGWGCKRWRGAAWAAGYKKKSKRKMRYGACYYYRRKRKRRCGYRS
            .   :* .*** .    **   :*   ** ********  .****   ***   **

45      RKGCKAAWRRKRRMRRKTTAYRMYYRRRWKYSRGCWMAARWWMKRMWMMTMWSMAYRGMR
51      RTAAKGWTARKRRMRGKCCWYRMYCGAGTGCSRKMWMMWRTWMKRAWMMKMWSACCGKAG
        *...*.   ****** *   ****       **  **  * **** ***.***  .

45      MRRYSYYMRYGRYRYYMMYTMRMWSCCCCWKYWRKACAYRYKKYKWKSMRKKMRKRYKKM
51      CARYGYYMRYKRYRYYMMCYMRMWSYMYYWKYWAKGTGYRYKKYKWKSMRKKMRKRYKKM
          **.***** *******  *****    **** *. .**********************

45      RWYYWYYGKGMMYGWRWWAATTWRYCCAAKKYGAKKYGCYYMKGYTAGKTCAGKKWKKTA
51      AAYYWYYRTRCCCKWRWTMCWGTRYYYRRKKTAGTGCAYCYMKACCCRKWYRKKGWKKAG
          ***** .     ***  .   **    ** ...  .  ***.  . *    * ***:.

45      TAACYWYMRGKKKMKYRYRKKKATRTTRMKKKYMYYKKKKTKRTTWYMCKYAKKCAYCAG
51      GTGYYWCAGKKGKMKYRCRGKKRYRCYRCKKKYAYYKTKKYKRWWTTMYKYRKKTGCTCC
         :. **    * ***** * **  *  * **** ***.** **    * ** ** .  .

45      TGWTYYWKYRRRRRYRYKRGRTKKYKKRKKKRGKGYGRYRYCCKCARKYRYKTKKKKKRM
51      CRTGYYWKYRRRRRYRYKGRAYKKYKKRKKGGKKRTRGYRYYYKTCRKTATTKKKGGGRM
            **************    ********   *    ***  * .**   ..**   **

45      ATARKWKKWRMWKKKWRRMMRYYSCYAWTRRKKYTMMMRRKAKYKKYKCCKTAGCCGKMW
51      WCGRKWKKWRMWKKKWRRMMRCCGYYGAGRRKKTWCMMRRKRKYKKYKYYKCGRYMAKAW
          .******************  . *.  ****   ***** ******  * .   .* *

45      YCYGKKRRSMKWMRWCYYTAARKRKCTTKTCACCRKKKMYGCTTAMTAMMTTWSKKRWRR
51      YYCKKKRRSMKWMRTMCTARTRKRKYYWTYYMMAGKKKCYKYCGGAYRMMYGAGKKRWRR
        *   **********    : :****   .    . *** *    .   **   .******

45      KWTATAAKMKYTMWWYRRRKCAKWWYYKKKTCATCKKRKWKKRRSWAGCAMTRYKTWMRY
51      KWYRWRWKMKYYMWWTRRRKAGKWWYYKKKYMMYSKGRTTKKRRSWGTTGMCRYGWTMRY
        **     **** *** ****..********    .* *. ******.  .* **   ***

45      WCYTTGWMRYMYARTKKAKKRYWRRYAATAGCAWTKRGTACCKKACGTWYKYACGAGCKK
51      WTCYYRTMRYMYMGKKKMKKRYWRAYGMYGASGWCGAACMYYKKTAACWYKYGAARRMKK
        *      *****  .** ****** *.  ....*   .    **:.. ****...   **

45      ARAWKRYCMRMRKSKSWKMWWYYKWYSGACRKKKYTATCKKGCCACYYCAGCKCTRYKRW
51      GAGAKRYYMRMRKSKSWKMWWTTKWYSRRMRKKKYCCATKKKMTGAYYMRSMKTARCTRW
        . . *** *************  ****   ***** .: **   ..**  . * :* .**

45      RYKYTKACAGCKAKRYSRMSCMRGYAKKKYRWTKRRCGRKTRYTGTCRWAYKMKRWKTMK
51      RYKYCKGSCTYKRKRYSRMSMMRRYCTKKYRAAKAAYTRKYRYATWSGTRTGMKRWKGMK
        **** *...  * ******* ** *..**** :*    ** **:  .     ***** **

45      KKKYKKKTKYKWWKYRMWARYKRKAWYKRKRKKMTYYYMYKAKWMRAWMMRAGSCTYKKK
51      GKKYKKKYKYKWWKYRMWMAYKRKRWYKRKRKKMYCTYMYKWKWMRRWMMRCKGYCTKKK
```

```
             ****** **********  **** *********   **** **** ****. .   ***

45          YTYRTKWKAGMYRRRKKKKWKKKCTRCAYKTCGCKRGGGTKGGTAGGMRRYKKRKCKWWS
51          YGYRAKWKGACTARRKKKKWKTGTGRYRCKYMRMKGTRRCKATACKRMRRYGKRKMKWWS
            * **:***..   ********.   *   *    *     *. :.  **** *** ****

45          YMKKYKKYAMYGGAGTKKKRRKKCYWYWMTTTYMAAGCCAKYAYRYCKKKKYKCKWYRYK
51          TAKKYKKYRMCRCWRAKKKRRGKTYWYWCCCGYMGCAYTTKYGYRYYKKKKYTMKWCGTK
              ****** *     :***** * ****    **...  :**.*** *****. **   *

45          RMWKKAWKKKKRYKRKKCRRTKCCYWKKKTGRRRYCYKYKWSRYGRRGKKKMKKRKYYSM
51          RMWKKGWKKGKRYKRKKMRAWKYSYWKKKCARRRYSYKYKWSRYARRKTKKMKTGKYYSM
            *****.*** ******* *  * .***** .****.********.** .****. *****

45          KKGTTTCGKKRKRYYCTYRSGWCYKRRSYKAKYCYRRGKAAKMMWWKRYKSKYKKCCTCK
51          KKKCCYAKTKRKRYYTCYRSRWTYKRRCTKMGYTYRGRKGCKMMWWKGYKGKYKKYYWYK
            **    . .******  *** * ****. *  * **  *..****** **.****    *

45          YKKRYMMTWMRRKKWKKAWYKKMMRYYMKRRRWRKYRTTGCYYYMYKMYATKYGATGSKK
51          YKKRYMMYWMRRKKWKKMAYKKMMRYYAKRRRWRKCRCCTTYYYMYKMYCCTYKGCACGK
            ******* *********  ******** ******* *    ********. .* . .. *

45          KSRCKKKRKYKMKYYYMWKRYYWMRSKKKWWYKGWRYRKYCGYYRKRRGRWKKYCAMRGG
51          KSRSKTKRKCKMKYYYMWKRYYWMRSKKKTWYKTWRYRKYYRYTRKRRKRWKGCYGMGRR
            ***.*.*** ******************* *** ******  * **** ***   .*

45          KTMRCCYYKYYMKKKKACCTYYYRGKKYMRKYYYRRKMWGCTMGYRMWRMGYTTSRRKKK
51          KYMRTAYYKYCCKKKKMTAGCTYRKKKYMRKYYYRRKMAATGMAYRMWGCKYKKSRRKKK
            * ** .****  ****  .   ** ************* .  *.****   *..******

45          KKWSYRYYWMKKRKRKRSYWKWKKKAKYMWKATTAGCGKKRKTTMRYYRWKWKYYTKYKK
51          KKWSYRYYWMKKRKRKRSCAKWKKKTKYMWGWYYMRARKKRKCAMATTAWKWKYYYKYKK
            ******************  *****:****      . **** :*    ****** ****

45          KWYRKMGTYKKRARWRASTYYCGRGYYKKKWYYRCMAKKKAGKKKKKRYRWRTMMYKYKY
51          KWYRKMKYYKKAGAWRTCCTYYRAKYYKTKWYYRMMRKGKRSKKKKKACATRYMMYKYKY
            ******  *** . **:.  *    ***.***** * * * .*****    * *******

45          RYCTTYAKKKAKYRKMMMYTWWKKWYYKAYKYSRAWAYCKKYGGMYTTGTYRYGMKKKRT
51          GCYKWYCKKKRKYRKMMMYAATTTACYKWYKYSRGAGTYKKYRRMYAGACYRYKMGKKAA
               . *.*** ********:  ..  ** *****. .  ***  **: . *** * ** :

45          TTMRKWRRARRAKYWAKKKTRRYYKCKYMRWKWYK
51          AAMGGARGMRGRGYTGKKKWRGTYKTKYMRWKWYK
            ::*   *  *   *.*** *  ** *********
```

The pairwise alignments of two accessions 46 and 47

```
46          RYMKRWYWYYSKWYWYRYMMRKMWRYMYWMRWKYYTRMMYYYYRKYKYRAYWGWWRKRWW
47          RYMKRWYWYYSKWYWYRYMMAKMWRYMYWMRWKYYWGCMYYYYRKYKYRTTWRWWRKRWW
            ******************** **************   ***********: * *******

46          WWKWWRMYRMYKKAAGGRCRRYRWRMRYKMRTGKRYARKRWYSMGCGRRKKWRYMTCKYY
47          WWKAWRMYRMYKKWGKTGTRRYRWRMRYKMAARKGCCAKRWYSMKYAAAKKWRYCYYGYY
            *** ********* .    *********** : *  . ******  .  *****    **

46          YSRRCYYRRWRKKACYGKKYRKWKMYCTRKTTWTYRYYMRRSKSMKTWYKRRMGGYYYRR
47          YSRRMCYRRWRTGTMYRKKYAKWKCTTARKKWWKYRYYMRRSKSMKGWYKRRMTTYYYRR
            ****  *****. : * *** ***   :**. *.************ ******  *****

46          YMYMRWMMRRMMYYYRYKRWRRRYWRRWMYYYWKMKMGTRWRWKRMRKAYGWYKWYGYRM
47          YMYMRWMMRRMMYYYRYKRWRRRYWRRWMYYYWKMKMAYGTRWKRMRKWYTWYKWYRYRM
            *************************************.   ******* * ***** ***

46          MKGMKWYTGYWWRWWYYYYKMMKYMTCKRATYYRRRRKRKRYYWYKMYMRYRWYRYYYRM
47          MTRMTACACCWTATAYYYYKMMKYMYYKGRWTYRRRAGAKRYYAYKMYMRYRWYRYYYRM
            *. *.  :  *    **********  *    ****   **** ****************

46          RAYRWKKKYYRWRRYYRKMYTRYWWYRYRYRMRMKRWKRYRRWKWSKRRKWRKYSRWWYW
47          GTYGWKKKYYRWRRYYRKMYYRYWWYRCAYRMRMKRTKRYRATKWSKRRKWRKYSRWTYW
             :* **************** ******  ******* ****  ************** **

46          WSMRYTCMKYGKYYWMGATGTKYYRTTYSYRRYRYWKYTKSAMRRKWTYAAACRKTYKRA
47          WSMRYYYMKYRKYYWMRMWKKKYYRWAYSYRRYRYWKCYKSTMRRKWACGCGYRKYYKRT
            *****  *** *****    .**** :**********  **:*****: ... ** ***:

46          MWYTKKMRYYKMYKRWYKSYRWWRKYWMRYYMYYRYMTTAGMRYARRAASYWWMYYYYYY
47          MWYWKKMRYYKMYKRWYKSYRWWRGYWMRYYMYYRYMCATRMRYTRRTRSYWWMYYYYYY
            *** ******************** ************ :: ***:**: ***********

46          RKWKKRMRYMYKKWAYCKKMYYRCTCTGTGCKMKRGKCTKSRKTKYWKCRWKMMRMYGRS
47          RKWKKRMRYMYKKWGYTKKMYYRYWMCRWRTKAKRRTYWKCRGCKYWGARWKMMRMYARS
            **************.* ******        * ** .  *.*  *** .********.**

46          KYMWRGYKRRRRYRRYKKKRYYGAWTWKKKRRSAYARRRYGTRWYRYGRRMYTYRYRKKT
47          KTCTAAYKRRRRYRRCKKKRYYATACWKKKRRSRYTRRRYTCRWYRYRRRMYYYRYRKKC
            *    .********* ******.:  ******* *:****  ***** **** ******

46          KKYTGRRKCCTKWKRACGTRKCGKKTTKMYACGAMSRKGYTCCAYMRRYRKKYSYYTRYY
47          KGYARRRKTTCGWKRMYRYRKYTKKWYKMYMAAGMSRKKYYYYWYMRRYRKKYSCCKRYY
            * *: ***    ***    **  **  *** ...**** *    **********  .***

46          RRWKYKSYMYKKRYMGKWRGRYYKATRMGGAAYTYSYYKYYWATTCSYRWWRMYYKRYRA
47          RRWKYKSYMYKKRYMTKWRRRYTKWYRMKAMRCCTSYYKYYWWWKYSYRWWRMYYKRTAW
            *************** *** ** *  ** .     *******  . ***********
```

```
46  KAMGYRAYMKAAAGTRYKWKYTTWCSWGRYRSWGAAYYMMYYWAACYKKARTGRYYRRGT
47  KCCKYGRCMKMRRKCRYTWTTCGAASWKRYGCAATWYYMMYYARWYCKKWRCAGCCRRKW
    *.  *   **     **.*.    .** ** . .: ******     ** * .   **

46  AYAMYYKRGTYTGKRMYYMSYWRTKWMGKRSWYRRMYYCATRTRYTWYRYGGKKKMMKYK
47  RYWMYYKGTGYACKRMCYCSYWRKKWMSKRSWYGAACCYTGRGRYGTTGYRRTKKMMKYK
     * ****   *: *** * ****.***.*****       : * **    *  .*******

46  GRMAYMRWRKYKTTKRMRAKYMKYKKYKRKCTYRYYYRKGRSCRSMGTTMYYWMYKTTCW
47  RRMGYMRWRKYTCAKRMRRKYMTTKGYKRKTAYRYYYRKKRSYRSMSKWMYYWMYKWCAW
     **.*******. :**** ***. * **** :******* ** ***.. *******  .*

46  KCWGGKWKGGTGRYRRKMKGWRKKRMKKTRKMKMMYYGRWMKMTYYAKKRKYKTRKTWRK
47  KTWAKKWKKSYKAYRRKCKKWRKKRMKGYRKMKCMYYARWMKMCYYMKKRKYKKRKYTRK
    * *. *** .   **** * *******  **** ***.***** ** ******.**  **

46  YRRSYYKWTTGKKGYRWYGTKCKRYYKWKCAKYYYTGAYMKYKKYKWKRKMYMRKWRYKM
47  YRRSYTGWGCTKKRYRWYAATAKRYYKWKTMKYYYKRTYCTYKKTKWKRKMYMRKWRYTM
    *****  *   ** ****.:..*******  ****. :* .*** *************.*

46  TYCYWYYYKKYRYWSYRYYWRKTTKRKKGRTGTYKWRYRYKKAYYRKYKRRKKYKAYKCY
47  CYTYWYYYKKYRYWSYRYYWRKCYGRGKRGYSKYKWRYRYKKWYYRGYKRGKKYKMYKYY
     * *******************   * *   ..********* *** *** **** ** *

46  RKKKKMRAYGYYYRRKRYYKYRKYARTRGTTTKKYKYMKCCKYWKATCRARWKYSRMYYC
47  AGKGKCRGTKYYYRRKRYYKYRKYRRKRKCCGGKYKYMKTTKYWKTATGTRWKYSRMYCY
      * * *.  ************** *.*     ******  ****::  :********

46  RKKKKKYRGATRGAKKMRYWWYYWYYMYRWTWKKKRSYKRWYKWMKKMYKYCRWYRKRRK
47  RKKKKKYRARGRAWKKMRYWWYYWYYMYRWKWKKKRSYKRWYKWMGTMYKYARWYRKRRK
    ********.  *. ****************.************** .****.********

46  KKKTKKSMKYCTAKMMKRCKRMGKRKKKYGRWRYCRMKKYWRYRKYWRMYRWWSYKKGSK
47  KKKYKKSMKYACWKMMKRYKRMKKRKKKYKRWRYYRAKTYWRYRKYWRMYRWWSYKKRSK
    *** ******.  ***** *** ****** **** * *.****************** **

46  KKYRKKYRWAAGACKTARRYWCRYYKKKKRRYRMYKTYYMGMGKMTKMYATGGAWWSMRW
47  KKYRKKYRWTTAGMKWMRRYWMRYYKKTKRACRMYKKYTCACAKMAKMYWWRKRWWSMRW
    *********::.. *  **** *****.**  ****.*  . .**:***     ******

46  RKSCKKKMRYWRYAKKTCYSYWYTKYYYRSMSWYRKMGGKYRRWYWRMKRKKMKKYRYRT
47  RKSMKGGMRYWRYWKKCTCSYWYCKYCCRSMSWYRKMKKKYRRWYWRMKRGKMKGYRYRY
    *** *  ****** **   **** **  *********  *********** *** ****

46  GMWRYRYMRYYRYSKRWTSWKKKTRATGGRYYYKKMYKWYMCWCWGTSRWYKKRTATYMR
47  AMWRTAYMRYYRYGTGAYSWKKKKAWKSRRTTCKKMYKWYMYWYWTCSRWYKKRYMYYMG
    .***  *******..   *****.  .. *   ******** * *  *******   **

46  YCCRMAYKYYCMKRTCTYRYGAYWTYRSYYRRRWRYSTWYKMWMMYGWWWAAACCCRKYY
47  TGTAARYKYYMMKAYTWTGTTGYWGCRGCCGRRWRYSYWYKMTAMTSWWWTCTTGYRKYY
          **** **        .**  *.   ****** ****  * .***:.:   ****

46  KSSRKYWYRKYYKTARYKRWYMKRRWRWRKRYKCKYYKKKMKRRYRKRYAKKMYRYRKAK
```

```
47    KSSRKYWYRTYYKAWRYKRWYMKRRAAARKGCKTKYYKKGMKRRYRKRYMKKMYRYRKRK
      *********.***: **********   **  * ***** ********* ******** *

46    KRKYRKKKKWRRKWKRMYSKRKCSKRRWMGYKRATMYKKTAKTTWYRRYYTAATTTRCSK
47    KRKYRKKKKWRRKWKRMYSKRKMSKRRWMRYKRWWMYKKYGKYYWYRRYYWTTCGGGAGK
      ********************** ****** ***  **** .*  ****** ::    ..*

46    RSYKKMRWKKGTACGAYTMKYSRYSKYAWRWWRMRKWSMRRWYWKMWRRWKYKKRYSYWW
47    RCTKKMRWKKRWRYRWYCMKYSRYSKYGWRWWRMRKWSMRRWYWKMWRRWKYKKRYSYWW
      *. *******      * *********.********************************

46    RGAATTTAAKRWKMMAGGKRWSYRRKTYMKRYWWWKYKKTTKRYMCKGRYYGYYKWRYYA
47    RACCGYYRWKRWKMMGAAGRASYRRKYYMKRYWWWKYKKCCKRYASKKRYYKYTKWRCYC
      *...     ******... * ***** ************  *** .* *** * *** *.

46    KRSRRKYYRKRYYWYKKRWKRYTKSAKWKAARRAKKYRRRKWMMMWKKRCARRKYRRTMY
47    KRSRRKYYRKRYYWYKGRWKRYYKSWKWKMMRRGKKYRRRKTMMMWKKRYRARKYGRWCY
      **************** ***** ** ***  **.******* *******   *** *  *

46    RYWRYGCAYYRRYRRWRYCYYMKSYRSSYGMYRYYYRYRMKARCWMWKKYCWWMKCKKGY
47    RYWRYATMYYGRYRRWAYTYCMKSYRGGYRMYRYYYRYRMGTRSAATKKYYTWMKAKKRC
      *****.  ** ***** * * *****..* ********** :*.   ***  ***.**

46    KKRMRRKTYMRSMYKTMYKGRMRMMWKMYYRTCASYTTKKYRRAGCRYKWSYRTKKRRTM
47    GKRCGRKKCMRSMYKAMYKTRMRMMWTMYYRAGRGCYYKKYRGMATRYKWSYAYKKRACA
       **  **. ******:*** ******.****:  .   ****  . ******  ***

46    MTCKKKMYYKYRYKRKWWYKRYYYRYKCRYRKMKAYWAMRKGAKKKKYSMRYMRKKKYRK
47    AKTKKKMYYKYAYKRKWWYKRYYYRYKYRYRKMKMYWGARKSMKKKKYSMRYMRKTGYRK
       . ******** *************** ****** **. **. ************. ***

46    YTYTCARSYYKRWMKSSARMKRMTRYRKKYRRKYKYKYGYKKRYRRYYRKMRMRKRRKRR
47    YKYAMMRSYYKRWMKSSMRMKRMGRYRKKYRRKYKYKYTYGKRYRRYYRGMRMRKRRKRR
      *.*:  *********** ***** ************** * ******** **********

46    KKMYCKMRKCYSKRYGCYKRYYKRYYYYKRKRKGAATTMRARMGKWRYRKTGTSRRRKRY
47    KKCYGKMRKTCCKRYCACKRYYKRYYYYKRKRKATCWAMRCRMRKWRYRKAAGSRRRKRY
      ** * ****  .*** . ***************.:. :**.** ******:. *******

46    KRYKRRWYGRKKKKYRRRKYYYKRRGYWYMYYYMKYRYWYRRRKYKRRWYRMYMYYYATR
47    KRYKRRWYARKKKKYRRRKYYYKRGRYACAYYYMKYRYWYRRRKYKRRWYRMYMYYYTWA
      ********.***************  *   ***************************:

46    WKKKSRRKMRKRKKRRWYRRYKCYKRAYTKYMYKRWYYYRRRWKKKMYWRKMRWRRKWWR
47    WKTKSRRKMRKRKKRRWYRRYKTCGAGCYKYMYKRWYYYRRRWKKKMYWRKAGARRKWWR
      **.*******************     . **********************   ******

46    MKWAAMRYYRATKSGAKYKSGTCRRKSYRSCMWRYYTGAGTSWTKATRRRRRKRYSYAMY
47    MKWMGMRYYRCKKSAGKTKSKYYARKSYRSYMTACYCKMACSWCGTGRGRRRKRYSCGMY
      *** .*****..**..* **    ****** *   *   . **  : * ******* .**

46    RYYKYKWKCGCTWKWTWGCRKMTKYWKRTRRRWYMYRYAWRYYYYKYYKKKYYRWKYRKK
47    RYYKYGWTAATAWKWGWRYRKMGKYWKRKRRRWYMYRYMAACYYYKYYKKKYYRWKYRKK
```

```
                 ***** *... :*** *  *** *****.*********    *****************

46               KYWMAAGTTAWKKGKKYYRYYMKMYKRKKKMKWYMWYWCKTGRYRYKMKKRMYTRGCRKK
47               KYWMTRRGWRWKKAKKYYRYYMKMTKRKKKMKWYMTYWMTGTRYRYKMKKRMYCRRTRTK
                 ****:     ***.********** ********** **  .  *********** *  *.*

46               RYRKWRRYYYYRYCRRKKYKCTRCMTRKKKMKKWKWWKKRYSKYYMWMTKKKYASYWKWR
47               RYRKTAACCYYRYARRKKYKAARTAYRKKTMTKWKWWKKRYSKYCMTMGKKKYMSYWKWR
                 ****     ****.******.:*    ***.*.*********** * * **** ******

46               RKRRKYWRWATYKMRKMRKYKSYCYKKMRKRSGACRKYYKKMYKKSYKWMRWYRRKWYYK
47               RKRRKYWRWTGYKMGKMRKYKCTYYKKMRKRSRMMRKYYKKMYKKSYKWMRWYRRKWYYK
                 *********: *** ******.  ********   ***********************

46               KWMWRKWYRRKKYWTKKKYYKKRWCATCRWSKYMTRKRRSMRWATAAYYKYRWYRKRKRK
47               TWMWRKWCRAKKYAYKKKYYKKRWYTCTRWSKYMKRKRRSMRTWWRRYYKYRWYRKRKRK
                 .****** * ***  ********* :  ******.*******     *************

46               YYYRYRAGCWRRAWKGKKSYRRYYSCTMACKKKTACRYGYYCWRKYKKSKYRKKRYMKRR
47               YYYRYRCRTAGAGATKKKSYRACYSTAMGTKKKKMTRYTYYYWRKYGKSKYRKGRYMKRG
                 ******.     . . *****  ** :*. ***.  ** ** **** ****** *****

46               KWMYRRWKYYMRTYYKKYYSYCYYGKRACTWKYYKYRRTGACAYYAKTRSYGAMYRKWCG
47               GWMYRRWKTYMGACYKKYYSTYYYKTRRYKWKYYGCRRARRYGYYRKCRSYATMYRKWAT
                  ******* ** : ******  ** .*  .****  **:    .** * ***.:*****.

46               TTKKRYYARYTCAWAKRKYGCYCCRKRKRKKKRKMRRKGWYMYSMRKWWKYMKRYKRKYC
47               CGTGGYTGGTWACWRGRKCAMYYARKRKRKKKRKMRRKAWYMYSAGKWWKYMTRYTRKYT
                   .  * .   ..*  ** . * .*************.*****  ******.**.***

46               TCGGCWWKWKKMRMKKARKRRKKMRYWYSKKAKKRYYRMRYKRMKSWKKWKYKRKYRKWR
47               WSRRYWWKWKKMRMKKRRKRRKKMRYWYSKKMKKRYYRMRCKRMKSWKKWKYTRKCRKWR
                  .   *********** ************** ******** ***********.** ****

46               KKKYYYRRKKWTKYSSRMYRRMGYGRMKSYKRKYYKAYMRMSCTTASKAKSRYRRKKKRT
47               KKKYYYRRKGWCKYSSRMYRRMAYKRMKSYKRKYYKMYCGASTCCCSKMTSRYRRKKKRK
                 ********* * **********.* *********** *   *   .** .*********.

46               ATYYKRAGKRYMRGSAAYTKRKKYRRGGRAGYYRRMKRRWKRASYYYMYKGYCYRYACTT
47               RYYYKRWRKRYMRKGMMTWGRKKYRRRRAMRCCRGAKRRWKGMSYYCAYKRTTYRYGYYW
                   ****  *****  .     ******       *  *****  ***  **   ***.

46               RMKYCYYMYAKTYKKRWMMKYRWWYRGAWCRTGTYKKYTGATRKKRAWRKMRMWYWWYRA
47               RMKYYYYMYGGCYKKRWMMKYRAAYRRWWMRCTYYKKCARGCGKKGRWRGMGAAYWWTAG
                 **** ****.  **********  **  * *   *** : .  **  ** *   ***  .

46               WGGAGASWKKYYRATYRYWAYKRRYRGCKYKKKKAMWWMYKMCYCCAKYWRRKRYRKMYR
47               TTRMTWSWKKYYRTKYRYWGYTGRYRRAKYKGKKRMWWMYKMGCYTGKYTAAKRCRGMYR
                       *******:.****.*. *** .*** ** *******     .**   ** * ***

46               MWKMKYKRYCCTCKMMRYWKRWRCYSWRYKWKKRKYWMKKMKMKGTMKKWYRRYYMWYKK
47               MWGMTYKRYTGKAKMMRYAKRWRTYSWRTTWKGRKYWMKKMKMKKKMKKWYRRYYMWYKK
                 ** *.****   ..***** **** **** .** *********** .*************
```

```
46          YKMRGTATRYAYCTMKMYRTAYAYYSKMAAWKCRWKYYYWKYWKKRRGGCTYWRMYYYRW
47          YKMRCCGCGCTCAAMKATGWWYWYYSKMRWWKAGAGCTYWKYWGKGGATTGYWRMYYYRW
            ****  .   : .:**     * *****  **.     ***** *  .   *********

46          KTGATKKYSMRKRKRYRRMRRKWKKKYKRYRYYCAKYTGKKKATRGYYYKSYWWMMKRYM
47          KWKRKTKYSMRKRKRYRRCRRKWKGKYKRYRYYTMKYWKKKKMAGKYYTKSYWWMMKACM
            *   ..************ ***** ********  **  *** :  ** ********  *

46          RKRKKRYRKMWMRMWMRKYRMSYRCYMRYYMKTRRKWWGYYYRKRRKKRTTYTCRKMKSK
47          RKRKKRCRKMWAAAAMGTYRMSYRYYMRYYMGYRRKWWRCYYRKRRKKRGYTAYRKMKSG
            ****** ****    * .****** ******  *****  *********   : *****

46          RYYRRRKTKKGKMRKAKRMKMYYCCCRGRYYSRRKYKKYKKRRYKCKTCYRKKRKRKWYW
47          RCYRRRKYKKAKMRKMKAMKMYYTTYRKRYYSRRKYKKYKKRRYKYKCTYRKKRKRTWYW
            * ***** **.**** * *****   * ***************** *  *******.***

46          YWMKKKRYKWMKYMTKYKTWSRRMYWYYKWRRYKKCKKTATYRWKYYCRCYYRKRKKYMK
47          YWMKGKRYKTMKYACKYGYWSRRMYWYYKWRRYKGYKKACKCRWKYYYGYYYRKRKKCMK
            **** **** ***  **  ***************  **:.. *****   ******* **

46          KMKMMKTTKKMYKRAMYMRKMRKSSMKSKKKKRYTCTAWWRKKKTMYRKMRRKKRRKYWK
47          KMKMMKCWKKMYKRCMTMRKMRKSSMKSKKKKRYATWRWWRKKTKMYRKMRRKKRRKYWK
            ******  ******.* *****************:   *****..***************

46          KKKKKYMRRGCMKSMRYRKKKMKWKKWTACKRMRKYRKKWRCRMMKKKKKAKKRRKKYRR
47          KKKKKYMRRKTMKSMRYRTKKMKWKKWYTYKRCAGCAGKWRTRMMKKKKKRTKRRKKYRR
            *********  *******.******** : **      *** ******** .********

46          KGARYKYCYRMRYYKKRYKRKAMRKKRRKYTYKYYTAGRRRKWKKKYWMMKKKRCTGGKK
47          KAWRYKYMTRMRYYGKRYKRKRMRKKRRKYYTKYYYRKRRRKWKKKYWMCKTKRMCAKKK
            *. ****  ***** ****** ********  ***   *********** *.**  . **

46          YMRKYKSYWWMYRYRRYRAGRRYYKGRSKSMYCCTKYKAAYTKKKYRKKKKKSMRTKKAC
47          YMRGYKSYWWMYRYRRYRTARRYYKSRSKSMYYSWGYTTGCCKKKYRKTTKKSMRGKTCY
            *** **************:.*****.****** .  *.:.  ******..***** *..

46          SMYRYYWKYYSAYRGAACRTTCRWTGATKRGKKWKKCTYTCTGATGTKTMYCCKTAGCRY
47          SMYRYYWKYYGRYRKRRYRWYYRWYARCKGRKKWKGMYCGMCAGATYTWMYAYKCGAGRC
            **********. **    *   ** .  *  ****       ..: .  **. *  .. *

46          RRMYYMRRSWRCYWKYTRWYGAACGAKWWKYMKMRWYRMRWTGKKRKRWWSARMTTMAGR
47          RRMYYMRRSWRYYWKYKRWYRGCMCCKWWKYMGMRWCACGACKKKRGRWWSRRMWGMTAR
            *********** ****.*** ..  .****** ***       *** **** **  *:.*

46          MYSSTTAMAMRMYRWWGKKRRRYTGRRY
47          MYSSKKRAWCRMYRTTAKGRRAYYRRRY
            ****..    ****  .* ** *  ***
```

The pairwise alignments of two accessions 46 and 48

```
46            YMRYRYYMKYWAMYWKYRMCTWYSKWMRYRYWRMKRYKTMKRRSKWRWRKMKMYYYYKCC
48            YMRYRYYMKYWGMYWKTACAKWYSKAAGYRYWRMKRYKYAGAASKWRWRKMKMYYYYKTA
              ***********.****   ..****   **********     *************** .

46            GTYRWRRKWKMYYRRYKTCTYKCKGWMWYRRKWYCAARYYYYWRYYYKYYYYRRRRGKMW
48            AAYGARRKWKAYYRRYKYYKYKTGRWMWYRGGWYGRTAYCYYWRYYYTYYYYRRRRKKMW
              .:*  ***** ******  .**   *****  **  : * *******.******** ***

46            RKWYYKRSMYYYYKWRRYMKWRYRRYKMTRRYMMRRMWRMCCYYWKYMYRSYYWKRKYWY
48            RKAYYKRSMYYYYKWRRYMTTATRRYKMYGRYMMRRMWRMYYTYWKTMYASYYWKGTTAC
              ** ****************.   *****  **********   *** ** ***** .

46            YYRWYRRTYYYCTYRMRYMRWYWYYRRMWKSRRYMKWWRRYCRRTARGKYKKWYKWWYRW
48            TTRWYRGKCYYAYYRMAYMRWYWYYRRMTKCAACMKWWRRYYAGAWRRKYGGTTKWWYRW
                **** . **. *** *********** *.   *******   : * **    ******

46            YYRRWWYWRSYWWYGKRWGMYRRCATCCKYWWSSGWWTKYYKKYYTWMRWYKMRYCCGGK
48            YYRRWWYWRSYWWYRKRWAMYRAMTAYTKYWWSSRWWAKYYKKYTCWMRWYKMRYTTTTK
              ************** ***.***  ::  ****** **:******  *********    *

46            KYYGGTMYKRKSWKKSGRYTYACYYMRMWYMKGWWRMRSYYRWWWYYYKRRMMCMWYWMW
48            KYCTKAMYKRKSWKKSRAYWCTYTYMRMWYMKKWWRCGGCTATWATCYKGRMMTMAYAMW
              **   :**********  *  :  ******** ***  .    *   ** *** * * **

46            YRMSRRYCGAYKWYKWYMRWKSWAKTWAARRMTRMKYYMYRMRYYSKMRKYMYKYYWKRS
48            CAMSRRCYKRYKWYKWYMRWKSWCKWWWRRRMKGCKYYMYRMRYYSKMRKYMYKYYWKRS
                ****    *************.* *  ***.  *************************

46            WRWWYMKAKWMCYWRKRWRKYYSRRWCTGYMMWKYRKYYKRRRKKRSYMRMYRRRYTGRA
48            WRWWYMGTKWMMYWRGAARKYYSRRAYYKYMMWKCGGYYKGRRKKGSYMGMYRRGTARAM
              ****** :*** ***   *******    *****   *** **** *** ****  :

46            TKTRMRRWKWKRCGYYRMYKMWRYMKYMRKMGYKRRYYRRRWCGAKKRTMMKYRKYRTGC
48            AKKGARRWKAKGYKTCGAYKMWRYMKYCRKMACGRRTCRRRWYKRGKRCMMKYRKCRKKT
              :*.  **** *       ********* ***.  **  ****    ** ****** *.

46            TTTAYKKRYYGARKKGWWRRSMYCTYKYKWRYYYYWRWSGCYCRYRKYYTCKWRKMRRCM
48            CGAGYKKRYTKRRKGKWWRRSMYTATKTKWRYYYYWRWSTTYTATGKYYWMKWRGMRRYM
                :.*****   **  ******* : * ***********  *    ***  *** *** *

46            GWWMSYRYMYAKCARRYTKRWRRYYKRRRKKYKKMMRRYYYWKCCKRWTCGSWRKMRKYW
48            RWWMSYRYMYMKYCGRYYKRWGATYKGGGKKYKKMMRRYYYATYYKRWCATSWRGMRKYW
               ********* * . ** ***   **   ************ .  *** . *** *****

46            MMKMKATKMKKKAGTWYTMRYKKYYMWKKYRYYRACTKCYRYKYRKRKKKTMKKYYKTGA
48            MMKMKTCKMGKKRKYWYKCRCGKYYMWKKYRYYRTTAGYCGCKYRGRKKKWCGKYYKACC
              *****: ** **   **. *  ************: :     *** ****   ****: .
```

```
46    CKKKKTYYCRWKYYYWKYWRRRKRSYYWRYMSKYSMRKTCARRMMATRKRMKAKRRKKKK
48    YTKKTGYYAGAGCTYWKYWARRGRSYYWRTMSKTGMRKWYWRRCCTCRKRCGRKRRKKKK
       .**. **.     ***** ** ****** *** .***   **  : ***   *******

46    KYCGTKWYTTTRMWRYWKTMRTRYCSYAGAYKMKCYCGTRCYRYRRTRKWRRYYYRWYYR
48    KTYRYGWYCCCRMWRYWTKMRAGYYSYRKMYKMKMYYSYRYCGYRRCAKTAACCCRWYYR
      *     **   ******..**: * **   **** * . *   ***  *      *****

46    YYKKARAGCGGKYWRYRRKKYRATTCRKYKKCRYRYAMRYYYKRYKRYYTKYKCAMMMKK
48    YYKKTRTKYRCKYWRYRRKKYRRWWMRKYKGMRYRYWMRYYYKRYKRYYAKYKAGMMMKK
      ****:*:    ***********    ****  **** ************:***..*****

46    SWKKTRRKYYTAGRWRCACYYYKMYKKKTCCKYWMAGATGARKYKKYKYKKRCGCSWYWR
48    SWKKKRRTYYYRKRWRTGYYYYKMYKKKAYMKTACMATGCCRKYTKYKYKKRAASSWYAR
      ****.**.**   *** . *********:  *    .:  .***.*******...*** *

46    CGGARRYRRTRYKKKRTRSKSMYKKKKTKRSYRMKKYMAMYRYYWGGCKKKKKMSYTRRW
48    YRRWAATGRKRYKKKRGRSKSMYKKKKWKRSYRMKKYMRMYRYYWRTTKKKKKMSYYRRW
              *.****** ********** ********** ******   ******** ***

46    GCYWWRRYRKYKKWGGSYWWKTRKYYSMYRSYRRKAGYMTRAKWYWKWWKKKKAGYMGYT
48    ATYWWRRYATTKKWRKSYWWKWRKYYSMYRGCRRKRKCMARGKWYWKWWKKKKRKCCTCY
      . ****** . ***  ***** ********. ***   *:*.***********

46    KKRKYGKKYYYGCRYRYYWKRSWRWYYCAYTKRSKMKGYGKYYWYKKWWMYGTCAAKAAW
48    KKRKCRKKYYYRYRYRYYWKACWRWYYMCYWKRSKMKAYKKYYWYKKWWMYSYYMRGRGW
      ****  *****  ******* .***** .* ******.* ***********.      .*

46    KYGGRWKKYRMYRTYYYWYKTYWRRMMYTRYRKMAYMKRRKWRCARRKYKACMWYWMTAT
48    KYKKRWKKYRMYRKYYYWYKCYWRRCMYKRYRKCCTMKRRKARYRARKYKTTMTYWCYGC
      **  *********.****** **** **.**** . ***** *   ****: * **  .

46    YTKSYWYYWRRCGCKKKYRSMSMWYKWKRMCTMWYWRMKKKCWWCGCRYRYYSTYYYYYC
48    CCTSYWYYWRRYSYKKKYRSMSATTKWKAMYYMWYWRMKKKYWTYRMAYRYYSCYCYYYY
        .******** . ********   *** *  ********* *     ***** * ***

46    YKTCAACKSWMRKWYYKKKGATATGYSRAYWAGCCGSKRWYKRGTRMTCCACCSRMKWGM
48    YKYTCGTTSWMRKTTYKKKKWWTGCYSRRYTMAMYRSKRWYKRKCRMWTYCYYSRMKWKA
      **  .. .*****  ****   :  *** *  .   *******  **   .  *****

46    KYYAYYYTYRRWKWMYKYCWWKWWWKKYRRKRKYTAWWMYTCAATRYCGWMKRRKYGRCG
48    KYYWYCCKCGRWKWMYKYGWWKWWWKKYRRKRKYWCWWMYKSMMWRYTTTCGARKYKRTT
      *** *  .  ******** *************** .****..   **      *** *

46    RYYRRCTTCCSYMTRWCRKKMYTCAGGGTKTTTRYTRKMKYWKTYKAATTYYSYTYKYRY
48    AYCGAMCCTASYMCRWYRKKAYCMTTAKGTCWWRYWRGMKYTKKYKMRWYYYSYAYGCGT
       *       .*** ** *** *  : .  .   ** * *** *.**    ****:*

46    RARKMYSCYWYYWYYYGRGCGTYACRWYYRMKYMTTMKRARKCGAKWCCYYYKWSYYWRW
48    AGRKMYSYTATTTTCYKRRMRACMYAAYYRMKYAACMKRWRGSTCTTAMYYYKWSYTTRW
       .*****        * *   :     ****** : *** * . .. . *******  **

46    WRAGYRGAAMCGATGGGKATKRWYWWARKGAGWKMTRYSMKTRMYTRYCTWSKAYCRRWK
```

```
48              WRTAYRKRRMGTGCTTAKGCKRTCTTGRKTTAWKMKRYSMKGRMYKRTYCWSKCCARRWK
                **:.**   *  .   .*. **     .** :.***.***** ***.*    ***. .****

46              ACARYAYWCYRYCTGARAATMAAMGTRWWWKRMCGGGGRGKKTRKKRYGWRRWSAMCRYA
48              TACGYMTWAYRTYYTTARWWMMGCCYRAWWKRMYAARRGAKKKKRKKRCRTGATGGCMRYR
                :.. *  *.**     :    * .   * ***** ..   .**.****       ..  **

46              ACTCCWSMTTRRKWMYRKMSWAMYYCYRYRWRARYYRKYTGRGKRKWKCKYSMYAKAKWC
48              MMYYYWSMWGGRKAMYRGMSWGCCTYYRYRAGTRYYRKCCARTGAKWGAKYSMYWKMKTY
                     ***   ** *** ***.    ****  :*****  .*    ** .***** * *

46              SGCAYYRWKRKKKYGTGGKRKWRRYKRWKKCAWKMYRMRRTKMYTCWYASYKYCYCAAYY
48              CRTRYTRWKRKKGYTGAKKRGARRYKRWGTARWKMYRMGRCKMYYTACTSYKYSTYTCYY
                .   * ****** *   . **  ****** .. ****** * ***     :****.  :.**

46              YKKYRRCKGRMRYYYKKRARYYATAMWYYYTTMGKRKWWKSKYKGMRYYRCRMKCCCGGS
48              YGTCAAMKKRARYYTKTACRYYGCGMWYTYCCMAKRKWWKSKYKACRYYGTGMGATTTAS
                * .     * * *** *. .***. .*** *  *.**********. ***    * .    .*

46              YWRRWKYRWMKMRWRKYKSYMKMRKRSRMRKYCKTWYKKATGGAWYYRKKKRWRYWRYWK
48              YWRRWKCATMKMGARKYGCTMKAGKRSRMRKYTTKWYKKTWSRMTTYRKKKAAAYWRYTK
                ******   ***  *** . **  ******** ..****: .     *****   **** *

46              WMTRRWWRKKRWKRYSRRKYRARKTTTTYYYCRSKYYKKKGGGTRYGMWSRKMKTRSMRC
48              WAKRRAWRKKAWKRCSRRKYRMGKGAYCYYCGGGKYYKKKRAAWRYKCWSRKMKCRSMRT
                * .** **** *** ******  * :  **   .****** .. **  ****** ****

46              YRYRKGCKYYCAGGYRRTKYRMTAYACGWAGATKKGMYYYKKYRYRWYKARYYYRYKWKG
48              YRTGKTTKYYACAAYRRYKYAAYWCGTAACRWKKKTMYYYKKYAYRWYKRRYYYRYGWKA
                **  *  ***....*** **     . . .  .** ******* ***** ****** **.

46              GTARYYKRRRKMKKRKKKKRRRRKWMMYSKRKRKYYSYYKCAMSSARKRMTYYRSSMYRK
48              ACGRYYTGGRKMKKRKKKKRRRRKWMMTGGRKRKYYSYYKTMMSSMRKRMGYYRSSCTRK
                . .***.  ***************** . **********  *** **** *****  **

46              KYRRYKYYKMMRRRCKKWGATGTYRKCGARKYKRKKRYRGRRYKRRRCCCGMWKMGYKCY
48              KTAGYGYYKMMRAGMGGAARGRYYGKGSTRKYKRKKRYAKGAYKRRRAYYKMWGMAYKTY
                *   * ******       .     * * .:*********    *****.    ** *.** *

46              KRYTTGGCATCYYMRGYKTACKRKRKMRKKCYMRYRRYRWMMKRGAGRMRWKRKKWWYKY
48              KRYWWKKACCYYYMRKYKCWYKRKRKCAKKYYMRYRATGTMMKRRWRAMRWKRKGAWYKY
                ***     ..  **** **    *****  ** *****    ****    ******  ****

46              TGKRMWKKRARYRRRWSWTKRKTKYRYRYYRRYKRKMTWMYKWTKKMYKWKCCRTRRKKK
48              KKKRMWKKRGRYRRRWSWGKRKWKYRTRYCGRTKRKMCWMYKWYKTMYKWKAYRKRRKKK
                . *******.******** *** *** **  * **** ***** *.*****. *.*****

46              MRMKRWGSSMTRTMYYKYWKRKRGCKKRKKSWYRCRKMRRKKACKSRMYKKWKWYKKGYR
48              MRMKAWRSSMYAWMYYKYTKRKGCTKTRKKSWYRYRKMRRKKRYKSRMTGGWKWYKKAYR
                **** * ***   ***** ***   *.******* *******  ****   ******.**

46              YRYYWKKYYYYGCYKYKYRKWATGKTGRCKMYMRRKKWRYTGAYKWRGMYYYYSKMATCA
48              YRYYWKKYYYTAYYKYGYRKWRWRKYRRMKMYMRRKKWRYCKTYTWGRMCTYYGKCWYTT
```

```
            ********** . *** ****   *  * ***********  :*.*  *  **.*    :

46          AGMTRYWSKRKCRKRKTYKWATTRAKWGYWYRYKYYRWSKTCCYRGCWRYKWRYRRTKYY
48          WRCCRYWSKRKMRGAGGYKWRYWRMKWSYWYRYKYYRWSKKYSYRRMWRCKWRYRAAKYY
                ******* *    ***   * **.************. .**  ** ***** :***

46          YRRRYKRKWKYKRYKGKKWYYKTRKKKYSRARYYWAMGGCCAKKYMKCWTYSRMCTTRTK
48          YRRRYKRKAKYKRYKKKKATCKKRKKKYSRRRYYWGAAAYGGKKYMKTTCYSRMYACRCK
            ******** ****** **   *.******* ****. ..  .*****   **** : * *

46          KYTKYKKKGATRGAKYTGGKYMKMTRWYYYRYWRYKTGGKARAKRKTGTYRYRSYKKRWY
48          KYKGCKKKARGRAWKTCACGYMKMCRWYYYRYWATKWRSKMAGKRKCCGCAYASYKKRWY
            **.  ***.  *. *  .  **** ********  *  .*  .***     * *******

46          KMMWMKKKMYKYRWMYRGTKRYKKKKGWYMYGCAMKKRYKYRYRMGGATTTKSCCGCTRW
48          KCCWMKKKMYKYRWMYRAWKRYKKKKKWYMYAATCKKRYKCGTRMTRMCAAKSTTATKRW
            *  **************. ******* ****..: *****   **    ::**  . .**

46          RYYRSRRKWRYRMYWWWSKYAKSKYRKATKKRKRMRCWWYWKRYTTYTKYRMKYYMAKKK
48          RYYRSRRKWRYRMYAWWSKYCKSKYRKGYKKGKRARATWYWKRYACYYKYRAKCTCGTKG
            ************** *****.******. ** ** *. ******: * *** *   ..*

46          WWSMYCRMKGWKKKCGMGGYKWKKYKKKCAKGSRGGYKYWYMRMMYGYMWRGYKRRRSRK
48          WWSMCGRMKKWKKKAAMRKYKWKKYKKKAWKTCGRRYKYWYMRMMYAYCTRAYKRRRSRK
            ****  *** ****..*  *********. *  .   **********.*  *.********

46          KRYKRYYKKASRYYRRAKRRRWYWWGTYGCRWYRRMYKTYRYKKKKRYYMRYSRYKYGKK
48          KRYKRYYKKWSGYCRAGKRRRWYWWTCCCTRWYRRMYKYYRYKKKKRYYMRYSRYKYRKK
            ********* * * * .********     ******** ****************** **

46          RYMRGSMCTGCKYKKKKATYMRKYRCRKKYSYYGYYCYYTRARKYKSYKCYKKARYMRRW
48          RYMGKGCYARYKYKKKKRKYMRKYRYGKKYSCCRYYTYCCGTRKYKSYGYYKKMRYMRRW
            ***  .  :  ****** .******  ****   ** *   :******  *** ******

46          TRKGWMRYRYKYKKCYATGKWKSYWKGWMMMRKRYMRKTRKYKKRCMCMWMYKRMMWMRR
48          YRKRWMRYRYKYKGYYWYKKWKSYWKTWMMMRGAYMRGCRKYTKRMMACTMCGRMATCRR
             ** *********  *   ******* *****  ***  ***.** *.  *  **   **

46          KYMMKYYRWWTCRYRYKRRKGYGTCCTGACTYTRMKKKRRYKTKWKRMRKCRKWKMYYYY
48          KYMMKYYRWWGARYRYKRRKKCRCTYYTGTCTARMKKKRRYKYKWKRMRKYRTWKMYYYT
            ********** .********        .   :********* ******* *.******

46          YYSRRAWWYSKTTMMYKKRYYCGYGKKKKKSRRYYYGMRYYTRRMRRKKWRWTTYKKMSY
48          YYSRRWWWYSKGKMMYKKRYCMTCTKKKKTSRRYYYAARYYKRRMRRKTTGTYYYKKMSY
            ***** ***** .*******     ****.******. ***.******.     ******

46          YMMYRSMMMCMGTGGCRRKRYSKGATYYAKWKWWKKMGGRCKCTYWWWYRMRRRMRRRCR
48          YMAYRSMMMTCACKKMRRKRYSKAGCYTTGWKWWKKMAARMKTCYWWWYRMRRRCRRRTR
            ** ******  .    *******.. * : *******..* *  ********** *** *

46          YRTKGKYYRWRKKYMWWRWKYCTKRKKRGAYWKKKKKRAAAYYKKKSKGKYWTKRAYYYR
48          YRWKRKYYRWRTKYMWWRWKYYYKRKKGAGTWKKKKKRRWMYYKKKSKRKYWKKRGYYYR
            ** * ******.*********  **** .. *******   ******* ***.**.****
```

```
46      KYKRMKYWYMRYYTWYRRRWAAARKCYRKKKKMKKRMRYRYTCRKKKMGYRKKKKRWYTA
48      KYKGMKCTTMRYCAWYRRRWGGGAKTTAKKKGMKKRMRYRYGYRKKTMKYGTKKKRWYKM
        *** **   *** :******... *   *** *********  ***.* * .******.

46      KYGKWRRKKYMKAKRKSKRRRWMYKYYYRSYYMRRYRYMKRSYKKMMGTACGATKMKYRM
48      KYRKWRRKKYMKRKRKSKRRRWCYKYYYRSYYMRRYRYMKRCTKKMMRWRYRWCKMKYRM
        ** ********* ********* ******************. ****       ******

46      WSRKKRWWAMTRKWYTCCKKKRAKRKCYMRRCSKKKYKKYRRYKWKWWKKMMKRACTGWR
48      WSRKKRWWMMYGKWYCAYKKKRMKRKYYMRRMSKKKTKTYRGYKWKTWKKMMKRMSWRWR
        ******** *  *** . **** *** **** **** *.** **** ******* .  **

46      RYMKYSKKRRRKKRYMKKRYRYMMKKKYYKSKWKKYKRKRWKRKKRKKYGTKMKMSGRKY
48      RYMKTGGKRRRKKRYMKKRCGCMMKKKYYKSKWKKYTRKGWKRKGRKKCTYKMKAGKRKY
        **** . ************   **************.** **** ***   *** . ***

46      KKKYYYKKKKMRSTCTTASKSRKYMRWGYRGKRYRKKRKRYRKYYAKRKYKKYRRRKKYK
48      KKKYYYKGKKCGSYYYYMSTSRKYMRTKYRKKRYRKKRKRYRKYCCKRKYKKYRRRKKYG
        ******* **  *     *.******  ** ************* .*************

46      KTCYGYYYWKYRYAKYKYRRWYRMKMYYYTTGGWKCCRCTRKRRACRRKMRKRKRRRWYK
48      KCYYKYYYWKYRYTKYGYRRWYGMKMYYYGAAAWKGAGTARKRRWAARKCGKRKRRRWYK
        *  * ********:** ***** ****** :..** .  :**** . **  *********

46      CYKRAYKKKYTAWYMMMYCRRTRYWMYKWKKMRKMCTTRRKYWRSWKKMKAGMGKRYWYW
48      TCGAGCKKKYYWWYMMMYMRRYRYWMYKWKKMRKATCARAKCAGGTKKMKGTMRKRYWYW
            . ****  ****** ** ************   :* *   . ****. * ******

46      WYRRTRRKCARYYKKGAAKYYKWRRMYWGACAYYKWKYKRKKGRWAGTYKYCYKRRCYYR
48      WYRRARRKYWRYYKKRRRKYYKWAACTWKWYRTTKWKYKRKKRGWGACYKYTYKAAYYYR
        ****:***  *****   *****    *      ********  *.. *** **   ***

46      YMKKMWKYARYRGRMYMRGCRRSKKSKKKRYYWRKKATGKRKRCACKYWKKYRKWTTMSM
48      YMKKCTKYGRYRSRMYMRKSRRSKKSKKKRYCWRKKMGTKRKRYRYKYWKKYRKWWKMSM
        ****  **.***.***** .*********** ****   ****   ********* .***

46      RYRKKAAGGKWKTTKGWAKGYTRKMKRKRGYRKTAKRYYKYSGAGCKATCGGKYASATGG
48      RYRKKMMKKKWKGYKKWWKRYWRGAKRKGRCGKCGKRYYKYSACRTGGGTRAKYWSRYKK
        *****    ***  * * * * *  ***    * .*******..   .   .** *

46      RYYWYRKYWAKRYMAYKRRGTRRAATAAMYYYYSYKKGSYKAACKRKYRWMMRCGYYKRR
48      RTCTCGGYWWKRTMTCTGGKCGRTRYWRCYYYYCYKKTSYGCTGKGKYRWMMGAKYYKRR
        *      ** ** *: .     *:     ****.*** ** .: * ****** . *****

46      WRGAYYMMKGGCCWAYRSYTMYKYRGKRYWRATKCMTRRAYSRRARYYYTWYMRWCYSRY
48      TRATYYMMKAKAAARCRSCWMYKTRCGRYWRTYKAMKRAWYSRRGRYYYKTCCGTYYSGY
         *.:*****. ..   **  *** *  ****: *.*.*  ****.****.      ** *

46      MMYRMRT
48      MMYRMRY
        ******
```

The pairwise alignments of two accessions 46 and 49

```
49              WRWCSMRKMYWKRYWRSYKSRRYMWCRTKCAWSCCKTYWKKYWWMRMCYRRCTMYKRYYM
46              WRWMSMRKMYWKRYWRSYKSRRYMWMRYKMTWSYAKKYWKKYWWMRMMYRRMKMYKRYYM
                *** ********************* * * :** .*.********** *** .*******

49              WKYWMRAYKARSMYRAAYYWKRYYRWMMWSRYKGASKYKKRYAKWMWRTKWRYSRGYYRK
46              AKYTAGWYGRAGMYRWWYYWKRYYRWMMWCRYKARSKYKKRYRTWMWRCGWRYSRTYYRK
                 **    *   .***  ************.***. ******* .****  ***** ****

49              WKRGTTAYRYMKKKMKMSYWWMMRTGWCMAYRWYYYYYSKYWRMMRTACWYKRSYYMMRY
46              WKRAGCCYRYMKKKMKMSYWWMMRACAAMTYRWYYYYYSKYWRMMRYWYACKGSYYMMGC
                ***.  .*****************:  .*:****************     * *****

49              WRYWWYYAGGRRRKWYRMKYGGYRRYYCRWAWAYRYMYRKYWKWRWWKCYMKCYSYYSRK
46              AAYTTYYTARRRRKWYRMKYKAYRRTTSGATACYRYMYRKYAKWRAWKMYMTMYSYYSRK
                  *  **:. ********** .***  .  : .******** *** ** **. *******

49              TTCWATKSTYKRRMMYYARGTYGYRWYKMKTKTRKYWYKWWYYRYKYRYRTKWRWWMWKW
46              CCTAMWKSKYKRRMMYYRRRATRYRWTKAGAKARKYWYKWWYYRYKYRYRCKWRWWMWKW
                      **.******** *  :  *** *  :*:***************** *********

49              YRKRCYYTTAMKYYWRWRRRSWRYWRWYYRRRSKYYYYYGWRRWYYKRYAKMYKMKWYRK
46              YRKRMYYCWCMKYYWRWRRASWRYWRWYYRRRSKTCCYYRWRRWYYKRCRKMYKMKWYRK
                **** **  .********* **************   ** ********  **********

49              RRKYYMWRYKRRYWRWYMRYYYYMKRYMYGCKYRKYYWRMYCTGCWWYRMMKTKYAKKMK
46              RRGTCMWRYKRRYWRWYMRYYYYMKRYMYKYKYRKYYWRMYMWRAWAYRMMKYKYRKKCK
                **   ************************  **********   .* ***** ** ** *

49              KRKYTCRKRWAWMRKKKKKRYAMKCYKKKKSYWKKSWYRYRKKRKKMMRRKKKAYKKKYC
46              GRKYYMRKRAGWMRKKKKKRCCCKYCKKKKSYWKKSWYRYRKTAGKMMGGGKKMYKKKYY
                 ***  *** .********* . *  ****************.  ***   ** *****

49              YYYRKAKMRYRRYCKYAMKKKRKWYMRYYYMTYRWMKMRRRKRRKMYYARGWRYYYAGYY
46              YYYRKWKMGYRRYTKYRMKKKRKWYMRYYYMKYRWMKMRRRKRRKMYCTAAWRYYYTAYY
                ***** ** **** ** **************.*************** : .*****:.**

49              YTTACWATCYYMWKTCCKATCYGYAGRGGMYYKYWKYRYTTTRYAMKAKWMYCAGGGYYR
46              YAATGATATYYCAKKTYKWYYYAYRKRRKMYCGYWKYRCCYWRYRMGRKWMYYRRATCTR
                *:::  :: **  *.  *   *.*  *  **  *****    ** *  ****   .   *

49              WMKAKYRYYWYWGGSSGKYRKKYSKYMCRMYYYKKKGYATTASYKYAAMYYKACTATKWW
46              WMKRKCRYYWYTARGCKKYRTKYSKYMGAMYYYKKTKYRYYWSCTYRRMYYGWMYWYKWW
                *** * ***** . .. ***.******  ******. *    * .*  ***      ***

49              KKKYRYGTATRYYRKGYTAAAAGGTATRMTKRRRMKRWRYMWRWWKYRRKRKKYAKRRKR
46              KKKYRYCACCRYYRKRCYRRWRACWRCRMWKRRRMKRWRYMWRWWKYRRKRKKYTKRRKR
                ****** :. *****       .    ** ************************:*****

49              MRKWYWMMRKSKKKRTGRWTATKGRYKKYKMKSRYMRRKKRYWYYRTYTAKCWRKGGRRY
```

```
46          AGKWYWMMRKSKKKRCKRWYRYTKRYKKYGMKSRYCRRKTRYTYYGWYACKYWRKTTRRY
              *************  **   . ***** ***** ***.** **  *:.* ***  ***

49          RYKKKKGCTGGATKGCYYRRKYMCYKYKWATKYCTRKKKKYSYYYRYKRKGARYRYYKKW
46          RYKKKKATYAARWKAGYYRRKYMYTTYKTRCKTMWGKKKKYSYCYRYKRKACRYRYYKKW
            ******.  ..  *. *******  .**   *    ******* ******..********

49          KWARGWSGTKMTTKWTYYYRYSRKRKGRRRAAKKKCTGRCKYAYRKRATGKMRMWRACTC
46          KWRGRASRKGMYYKWKYYYRYSRKRKRRRRTGGKKGCRGTKYWYRKRRYRKMRMARTAAA
            **    * . *  **.********** ***:. **     ** ****   **** *:.:.

49          YRKKMKKYRWSKCMKKKRKRYKTGYRMYRTMMKKTCRAGRKTRYSMKSMRKGMSWRYKYK
46          YRKKMKKYRWSKYMKKKRKRYGCAYRMYRWMMKGWYRTRRKKATGMKSMRKKMSWRYTYK
            ************ ********  .***** ***   *: **.  .****** *****.**

49          MWTCYGTYGAMYKKGTTGRYKRRSKWYRRKYCKMCTYYCGGKRMGGGMMRYKRWGSYGYK
46          MWKYYRYYRRCYKKAAYRRYKARGTTYRRKYTKMTKYYYCRKRMKSRMMRYKRWASTKYK
            **. *  *   ***.:  *** *.. ***** ** .**   *** . *******.*  **

49          RYYKAATGMRYGKWCGRWYWYYMKWYRKTAKKRRYRRYRYCWYKACKRRWSMRYRGMRRK
46          RYYKRRKKMRYKKTTKGWYWYYMKWTRKYRKKRRTRGYRCYATKTAKRRWSMRYRKMRRK
            ****  . *** *    ******** **  **** * **    *:.********* ****

49          WYYTYRYYMKKYRMWYYKMRKTASWRRYWCTAYRGAWCCAMACTCTKKCYYRGYRRRYRY
46          WYYCYRYYMKKYRAWYYKMRKYMSWRRYWYCGCRSMWATTAGYWSKGGYYYRRYRRRCGT
            *** ********* *******  ******  . *. *. : .  ..   *** ****

49          KAGYRKYRMRYRYKRYYRYKRKGYWAGMCKKKRKYRYRYRKYKKRKMRMKMYTAWKKMKK
46          KGAYRKCRMRYRYKRYYRYKRKRYWMRCMKKKRKYRYRYRKYKKRKMRMKMYGCWKGAKK
            *..*** *************** **    *********************** .**  **

49          TGSATCRKRYTKRYMRWKMRRKAAKRGGCGWWYKYRMGYGCTYGKYRKRCTKYYRCAYRT
46          CACTWYRKRYYKRYMGAGCRGKWWKRTAMCWWYKYRMTCATCYTGYRKRGCKYYRYMYRW
             ..:  **** ****    * *  ** .  *******  .  *  ****  ****  **

49          WYYWMRTTYRTTGTTRGARRRYSYMMYKRKMCTKWKYWRKMRKTRKKYYKRKRAMKYWRM
46          TCTTMRAGTGKCAACRTCRGRYSYMMYKRKCMWKAKYWRKCAGWRKKYYKRKGRAKYWRM
                **:   . .: * .* **********   * *****    ********   *****

49          KKMSMRRYRKRYKKRYKAARARYCWKWATGWMYYRMYGATYYYMKKASKKRYMRCCCATT
46          KKMSMRRYATGTKKRYKWMRRGCMWKWRKKWMCYRMYKWACYYMKKWGGKRTMRYYYWKC
            ******** .  *****  *    *** . ** ****  : ***** . ** **    .

49          ACTAKKYSYWYYRMRKTRKWYRRWYWKKRMTGKRTCTCKMMMKKKRTCRMRTYRWTCTKM
46          CTYTGKYSYWYCAAGKKGKWYGGTTAKKRMYRKRYSYYKCAMKKKRATAMAYYRWCACKC
            .  : ******    *. ***     ****  ** .  *  *****:  *  *** . *

49          CRWMRRWWWRTCYAGYARRKMWYAAGRYKWMKSYMSATYRRMYAKRYYKYCTYTKYRKKA
46          MRWMRRWWWRYMTRKCTRRKMWYMRKRYKAMGCYMSWACGRMYCGAYYGYSCCWKYRKKW
             *********      :******   *** * .*** :  ***.  ** *.   *****

49          CCYKTKRRKRKYTKRKAAYTKRWRKTMYYYKKTMRTRKYRMRRSGKRYYTYKKGCSSWTK
46          TYYKYKRRKRKYCKRKGTTCKRWRKKMYYYKKYMRGATYRMRRSKKRYYACKKTGSSWWG
```

```
          ** ******* ***.:  *****.****** **  .****** ****: **  ***

49      MGGTTYYMMYKYKKKYYYKRRRYKKMMAMYRKKYWMWMKKRCTKWWYMRYYAGYTTYKKT
46      MSRGCYYMMYKYKTKYYYKRRRYKKMMRMYRKKYWMWMKKRYCKTTTMRYYGRYYKYTKC
        *.   ********.************* *************  *   ****. * .*.*

49      CACTRAARKKKKTTKKCGAWGAMYRRWKRSATKKRKMWRKKRYKYMAGSYCTRAKRKAGT
46      TGMWGGTRKKKKKWKKGRRARMMYRRWKGSGCKKRKMWRKGRCKYMRTCYMWRGKRKWSY
         .   .:*****. **      ****** *. ******** * ***  .*  *.*** .

49      CCAAGRWYKCWWSTCYCMRSKWYRKYGTYTGTTTATKKRKWYKKTKTKKCKRRRRKRKKY
46      YTTCARWYKTWWSKYYYMRSKWCRKYRCYAACKWRYGKRTWYGKATWKKYKRRRRKRKKY
          :..**** ***. * ***** ***  *:. .    **.** *:. ** **********

49      RTARKKKKWKCYCMWCMKWGWTCTAYWYWMYYMKTRGCYMKKSMSYRTTCCYMWYCYGYM
46      RWWGGKKKWKYYAMTAMKWRWYYCTCWYWMTCMKCATYYCKKSMSYRWYYSYMWYMYAYM
        *    ***** *.* .*** *   : ****  **    * *******   .**** *.**

49      KKWYYRCGAKWRWRYWKYKACKWAMRKKAKKKKTTTWWKYAAGTATRKWGATGGAGRMRM
46      KKWYYRMRMKWRWRYWKYKRTKWRCRKGRKKTKAACWWKYGTACGARKWAGACRGRRMRM
        ******   **********  **  **  **.*:: ****.:. .:***..:  . ****

49      KMYYRCYYARTGAKKTKKRCGACYCCYYKKYAMRAKYKYAGWTKAAGGRWTKTWTRRTMK
46      KMYYATCTGRWAGKKYKKRMAMMCMMYYKKYRMRRKYKYTATAKMMKRRWWKKWWRRCMK
        ****    .* ..** *** .     ***** ** ****:. :*    ** *.* ** **

49      KKMMKYRYWCATTKCTYSRYYKMMKKKWCKKYYWRKYMKTMRYYTKYSYYGGYSKKMGKK
46      KTMMKYRYWYWKYTYYCSRYCKMMKKGWSKKYYWRKCMTKCRYYYKYSCTRKYSKKMKGK
        *.*******  . .    *** ***** *.******* *.. *** ***    *****  *

49      YKKWRTTTYKRKYRTGYYRRMRRCYWWKKRRRGYTGCYARMWYKYWKYCRCGAKRWSAKY
46      YKKWRWCYCKRKTGCRCCRRMRRTYWWKKGRRRCYRYYGAAWYKYWKCMRTRGKRWSTKY
        *****    ***      ***** ***** **     *.  ******  *  .****:**

49      YKKGCWYRTWKKCAKRRRRYKKKKWWYYYKKWYCWKKKKRRCAGCKYYYYMARKTTTWTK
46      YKKTYWYGYWKKYWKRRRRYKKKKWWYYYKKWCMWKKKKRRMGRYKYYYYMTRKCWGWKK
        ***  **  ***  ******************  ******* .  ******:**   *.*

49      RKYRKRKYATRRYKWWSMYYKKKWKYRYKKAKKKRRKYCTTKKRMYGCYCKYWGGYKWRW
46      RKYRKRKYTKRRCGWWCMYYKGKWKYRYKKGKKKRRKYSAWKKRMYTACTKYWRKYKWRW
        ********:.**  **.**** ********.*******.: ***** .  ***  *****

49      RRGRKYKRKMMRWRWSWKAYMRYYKTRYSYYRYMYRYAARKCKGKMKSYGGGYTACKYSK
46      RRKRKYKRKMMRWRWSWKCYMRYYKGRYSYTRYMYRYGGRKSKKKAKSYRRRYWRYKTSK
        ** ***************.****** **** ******..**.* * ***   *   * **

49      YRYYYACGGCSKRCAKYRYYAKWYWMYYCTCARGYTYKKKKAACGGKWKKKCAYRWKASK
46      YRYYYTSACTCKRYMKYRCYRKWYWMYYAAYCRKYYTKKKKWRYKKKWKKKTTYRWKCSK
        *****:..  .**  *** * *******.: .* *  ****     ***** :****.**

49      YKRRGGTKMGYRRRSRWGAKYAYWMYRWYYRRCCGACWKKYGTGGCCGKRRAMWSKMRKK
46      YKRRATYKMKYRRRSRWKMKYRYWMYRWTCRRATTRTWKKYRKATTATGRGTCWSKARKK
        ****.  ** *******  ** ******  **.    **** ..  .  * : *** ***
```

```
49  RKYRWRKRRYYKAAYAATTARWKCATTKGRYRYRMKKMRYYYMRMKWRYRKYKKKKATYY
46  RKYGAGGRRYYKGGCCWACMGWKTTGCGAGYRYRAKKMAYYYMRAKWRYRKYKKKKTAYT
    ***    *****.. . :   ** :   . **** *** ***** ***********::*

49  MYKKYYYYRYYWYSWRTYRKRYRATCRGMYYWSKAMRKYKYYWWWRWKRKGCSYWYGYTG
46  MYKKTYYYRYYWYSWRYCRKRYRRYSRKMYYWSKWCRKYKYYWWWRWKRKRYSYWYTYCK
    **** ***********  *****  .* ******  **************  **** *

49  GWRMYTRRMWMYWYSWYGTKKKKKKRYRRSMWRRKMKKKYSWWRYYKSRKGKRKWWKTRM
46  KWRMYYRRAACYWYSWYKYKKKKKKGTGRSMWRRKMKKKYSWWRYCKSRKAKRGWWKGRA
     **** **   ******  ******   ***************** ****.** *** *

49  YKRMKGRRTKYGAYWAYTKCYRKKKGTTCCKKRYGTWTTGMKGGKRTRRWMYYKRKKKYG
46  YKRMKKRRCKYKMYWRYYKYYRKKKTAGGTKKRYRKWCAAAKAAKRCRRWMYYKRKKKYK
    ***** ** **  ** * * ***** :   **** .* :. *..** ************

49  AGWRKMRKKKRRRYYWMAWYSRKKYYMWKRRRKWSMKKRMYYCATACAKRRKGGMMYYRT
46  RRWRKMRKKKRRRYYWACWYSRKKYYMWKRRRGWGAKGRMYYTRCRYMKAAGKRMMYYRK
      ************** .************** *. * ****      *     *****.

49  YKMKRKWRWSKKRWAKKMKWKKYRAYKYKTKYKACATAWKRTKKMKKKGRYGYKGYKKCK
46  YKCKRKWRWSKKRWCKKMKAKKTRRYKYKKKYKRAGCWWGRCKKMKKTTGTKCGRTKKMK
    ** ***********.**** ** * ****.*** ..  * * *****.        ** *

49  RCCYSMKKKKKYYGCCGTTYCKYYCYYWKMTAWCGKKYYYRKKYCTRTYYYSGKMYKTTY
46  RMYTSMKKKKKYYTYGKYKYMKYYMYYWKMCGTYKKKTCCRTKYYYAWYYYSKKATGYGY
    *   *********     .* *** ***** .   **   *.**    **** *     *

49  GTRGRKKMYWRYRYKMYYRMRRCGMYRCGAWCTRKMAGTTYSKGRWGWCGKCCRGGKGKM
46  AARKRKKMYWRYRYKMYYRMGGTAMYRTAWWMKAKMRRAAYSKRRARTTRGATAKKTAKA
    .:* ****************   .*** . * . **  ::*** *      .    ..*

49  KKAGGYRKYTATAMRYMYYKMRMYMMYRRYYCAYYTMYKRYKWMKMKWRRKSRKRKRKMY
46  KKRRRYRGCKTATAACMYTTMRMYMMTAGTCAGYYCMYKRYKWMKMKWRRKSRKRKRKMY
    **   **   .:::  ** .******     ..** ************************

49  WKSWCRGKKKRRKMKYRYCTGCGTKWGRKKKMTKKGYRRRYMWYMYACGKKKMRRGKKMY
46  WKSWSRKKKKRRKMKYRYYKRYRYKWKRKKKMWKKKYRRRYAWYMYWMRKKKMRRKKKMY
    ****.* *********** .    ** ***** ** ***** ****   ****** ****

49  YTTSTRYMMYWSKRRKKKKWGWGYYTRYMGMKKYTAGSRCAAATAYKRYYWKYRSSRMYK
46  YCCCGATCMYWSKRRKKKKWRAAYYKRYMRMKKYYRKCRYTCCATYKRYYWKYRSSRMYK
    *  .    ************  .**.*** ****   .* :..::***************

49  KCGAYKTASKGRTYTMMMWRMRYCYKYWRYRRRKYGKCKKRRWKKAGTYYKMKKRYRKRW
46  KYRMYKGRSKRACTCMMMWRMRYYCKYWAYRRRKYAGYKKRRWKKWCCTYKMKKGYGKRW
    *   **  **     ********  *** ******.  *******    ***** * ***

49  SRKKTRWMRKRWKRKKWYYYYKYKRSRKMKMYRMKMGRRKYAAACARRKMRRYMRKYKCR
46  SRKKCRTARKRWTRKKWCYYYKYKRSRKMKMYRMKCRRRGYMWRYGRRKMRRYMRKYKYR
    **** *  ****.**** *****************  ** *    .************ *
```

```
49      TGCAGRAYYSRYYTRRKTACYKRRKWYGRRTTYTMTKTWGTWCTTWMKYMCGYRMRWKMK
46      CRTRKGGYYSRYTGAGGGRYYKRRKWYARRYKYYACKKWKKWYGCWMKYMTRCACGAKMK
              .*****        *******.** .*   *.* .*   *****       ***

49      TCAGKATYRCYGRTRYYKAKTCYYYWAATGKMMYMYGRSACYKYSKYTYKYYMCMRKWKY
46      GTWRGTYYRYTTGWRYYKRTKMYYYWRRYRGAAYMYARSGYYKYSTCYYKYYMYMRKWKY
             : **     **** .. ****       ***.**. ****.  ***** ******

49      KYWWYKKMRYAKKGCSYYYSRKKYWRYRYMSWRRSKMWRMRKAYWMRRWMKRCTKMRMKR
46      KYWTTTKARYWKKRYSTYYSRKKYWRYRYMSWRRSKMWRMRKTYWMRRWMKRSWKMRMKR
        ***  .* ** **  * *************************:*********. ******

49      RTTAATATKCRTRRWKYYKKGRCCKKSMYRYTAGWGWAAKYMRTTCMMKRWYCRGSKKSY
46      RAWRRCRCGMRYRAWTCCKKRGMMKKSMYATYRKARWRRKYMGCATCMKRWYTRKSGKSY
        *:        * * *.  **    *****       *  ***  :  ***** * * ***

49      RYTCKKKCTKSKMMTCGAYACRMYMWYCYYGYCGSRWKMKYRGYYYYKKRCWKGKYRAMC
46      RYCTKGKTAKSKMMGTTRYTTAMCAAYYYCKCTRSRWKMGYRRYYYYKGAAAKKKYRRMY
        **  * * :*****    *:  *   * *     ***** ** *****  . * *** *

49      KRYMYYKYYRRKCYSKSAYWWYYYRWRKMRYYMYGCCTMKTRTTCCRCG
46      KATMYYKYYRGKGYSKSCYAATYYRWRGMRYYAYKYMKCGCRCATTRTA
        *  ******* * ****.*   ***** **** *    .  * :  * .
```

The pairwise alignments of two accessions 46 and 50

```
46    YWKRKMRWGRKYRRYCTGARGCKYMYMKWYKWYGCCRRRYRYKGGRTKYRRWKRSKYRTY
50    YWKRKMRWKRGYRRCYWRWRTMKYMYMKACGAYAYGRRAYRCKSARGKYRRWKRSKYRWY
      ******** * ***     *  ******    *.  ** ** *..* *********** *

46    RMTSYRYKACRRYMRYYMRCKWRGCYRRKWKMRAARMYRYRMSYWWMYYRYMTRKCCTYY
50    RMWCYRCTRMRGYARYYMRYGWRKYYRRKWKMRTGRMYRYRMSYWWMYYRYMYRKYYWYY
      ** .** .  * * *****  **  ********:.***************** **   **

46    YYYKMRYYRRGACMRTWGATYKYRRKWYTGCMGATGCYMKYRRMWAKGKKSRRYWKWMKA
50    YYYKMRYYRRTGGMRGWRGYTKYRGGWYCTMMAMAATYMKYGGATWGRKKSRRTWKWMKT
      ********** . ** * .  ***  **   *. :. ****       ***** *****:

46    CCCKYTGGAKRKRYRRRMRRRMMMYMYKYTYAACKAMWYYWYRYMGKYSMRKRKRRYYTG
50    YTATYACTRTGGGCRRGMGGAMMMYCYKYACGGYKWMWYTWYRTMRTYSMRKRKRRYYCR
        ..*:   .    ** *   **** ***: .. * *** *** * .***********

46    CCKYSRRGATYRCRMRWYYCYAGYYRGYYWWRMWWWWRWYKKYYMRYRRTATTKMYYRWM
50    YTKYSRRAGATRTRMRWYYYYWAYYRRTYWWRMWWWWRAYKKYYMRYRRYWCKGMYTAWM
        *****..: * ****** * .***  ********** **********   . **  **

46    RRYWCYKGCRRYRKYCYMMYRYTYYWRKYMTGAGRTYMRWMMRWRYYYRYWKSRMKRYWT
50    RGTAAYKTTRRYAKYMYMMYRYGYYWRKYMARTCRKYMRWMMRWRYYYRYTKCRMKRYWY
      *   .**  *** ** ****** *******: : *.************** *.******

46    TKMMWRYYRSYYYYYRWWKSWKYSKYAYKYRKKMKACYMKKCGTKSYWYKAYAMTAYYMS
50    KKMMWRYYRSYYYYTRWWKSWKYSTYRTGCRKGMKWMYMKKYKYTSYWYKTCGCKWTTMS
      .************* *********.*    ** **  ****   .*****: . .   **

46    WRMYYCTKYWKRRWKWWRSYKWYWRKYTSWMKAYYMYKGKAAMGAAYYYKWMYMWWKKMK
50    TRMYYGCGYWKRGTKWWRSYGAYWRGYCSWMKRYYMYKRGGGASRRYCTKWMYMWTGGCK
       ****   ****  ******  *** * **** *****  .. .  *  ******    *

46    GGYMKMWKKYTGCYTYSRKYMYSYRKWWSRAYRKWWKKYKKRRYRRMTYCTWKKAGCRYK
50    KTYMGMWKKYGKSYGCGRKYMYCYRKWWGARTGGWWKKYKKRRYRRMGCTAWKKMATRYK
        ** *****  .*  .*****.*****.     *************   :*** . ***

46    YKTWYKKRYYYRYKKCGGGRTWCRRYRKKMYRYKKTAKRRRRKMMGRKKRKMYRKRAWAR
50    YKWWYKKRYYYRYKKYRRKRWWMGGYRTKMYGYTKCRKARRRKMMKGKKRKMYRKGTWMR
      ** ************    * *   **.*** *.*  * ******  ******** :* *

46    MRMGTRYRSSYRYYYRYYGWWGKKRYGGRKTRCYRRRKTCTTRCTKKYYGKMRKGMRTRW
50    MRMRGGYRSSYRYYYRYYKATAKGRYKKRGKGYTAGAKYYKYRGAKKYYTGMRKRCRWGW
      ***   ************   .* **  *  .      *  . * :****  ***  *  *

46    RKRAYYYAMYKRRKTMWKKRKYGRTCRCMKCYWRRKWMKKAYCCRWAKTRMKMMRKYWWT
50    RKRRYYYMMYKRAKYCWKKRGYRGWYRTCGAYWRRKWMKKCYTYGARKCRMKMMRKYATC
      *** *** **** *  **** *    *   .*********.*     * ********
```

```
46              ARKKMRGTRRYMSCYTKRKTASAMKWYRKKKRWRGRWKMRGSAWWKKRRKTACAYRKKKY
50              TRKKAGRYRRYMCAYKKRGCTSGMKWYRKTKAAACRATMGACGWAKKRRKYRMRYRKKKY
                :***    ****..*.**  :*.******.*    * .* ...* *****    ******

46              YKRWTACATRRYRWSMRKRRSMRSAYYYCMRKRKMKYYKKKYWSWKWKRKKKYWRKATYK
50              YKRWKWYWAARYRWSMRKRRSMRGRYYYTMRKRKMKYYKKKYWSWKWKRKKKCWRKRYYG
                ****.   : *************. *** *********************** ***  *

46              YYCGTKARKMRRKWCYYRCAKRKYKKMGMKYRYKRKKRRRKMRRWKRYSWWRRYWSAARK
50              YYTTAKGRKMRRKWYYYGGCKRKCKKARMKYRYKRKKRRRKMRRTKRYSWWRRYWSWWRK
                **  :*.******* **  .*** **  **************** ***********  **

46              KKKRAYWRKYRRKKKKWYMGMAACCMMKKWTAKMKWKWTACKRKYWRKMKKAKRAYRCRR
50              KKKRRYWRKYRRKKKKWYMRMCCGTMMKKTCGKMKWKTYTYKRKYWGKMKKGKRRYRYRR
                **** ************** *..  ****  .*****  : ***** ****.** ** **

46              KMWMSYMKYWACRCCRGKRRKKMRYAMATTRTGTKAKRRRRKYKSKMMRYRKWKRWCGTG
50              KMWMSYMKYATYGAYGKKRRKKMRYTARAAGCAAGRKGGGRKYKSKMMRYGKAKRWAKWR
                ********* :  .   ********:  ::  .:  *   ********** * ***.

46              YSWRKRMKKKYYACGMCRMWCMYCCGRYGCRCAGKRYKGKYRKKKKTGMMWKYYKRYMYK
50              YSWRGRMKKKYYWTKMYRMWYMYTATRYRMRMTAKRYGTKYGKKKKARCMWKYYKRYMYG
                **** *******   * *** **.  **  * :.***  ** ****:  **********

46              WKKKCRWKYYGRKYARKKSYRMYRWKAGGYWMYRMRKGKRKRRKSYRSKMYRGCRRKRYR
50              WKKKMGAGCTRGKYRRKKSYRMYRWKRKRYWMYRMRKTKRKRRGSYRSTMYGTARRKRYG
                ****        ** ***********   ******** ***** ****.**  .*****

46              RMYTWYKYYCKKWKMTTRWKKAYWWWYRRRRRRRRWYARRWSRYWRRYYRTKGKKKYRKR
50              RCTYWYKYTMKKWKMCCRWKKRYWAWYRGRRRRRRWYMRRTSRYAARCYGAGAKKKYRKR
                *   ****  *****  **** ** *** ******** ** ***  * * : .*******

46              WKKKYCKYWMWKKRKYRKKGYKYKCTYCKGYKMRYRYYYKKKWKSYWWMMMRWSTCAMRK
50              WKKKYAKYWMWKKGKYRKKKYKYTTWTTKACKMRYRYYYKKKTKSYWWMMCAWSGSMMRK
                *****.******* ***** ***.    *. *********** *******  ** . ***

46              YKGKMGRYATRKMYTCGTYRRRYRKRYYWWWYYRTYKGWYYRKMTCYRRRKKKKWMRRWK
50              YKKTMRATWYGTMYAARCTRRRYRKRYYWWAYYRCYKKTCYRKMYAYRRRKKKKWMRGWK
                ** .*      .**:.   *********** *** **   **** .*********** **

46              MYYKYYSRKAAGCYWWYSYMMMAGCRYYKRKRKWYYWMRYMCKKKWRWYAKKGAMAKGAT
50              MYCKYYSRTCWTTCWWYSYMMMGTARYYKRGRTWYYWMRYMTKKKWRWYMKKTGMCKTRK
                ** *****..    ********. .***** *.******** ******* ** .*.*  .

46              CKYCCSWTMYKCYYRATGCYCWCGKCSGAGRRYYWRYYWKRKKKYGCMRRWKMTGGKCCC
50              AKYTTSACMYGAYYRMAAACYTYRKTCCGKGGCCTRYYWKRKKGYRAMRGTKMWKAKYMM
                .**  *  ** .***:..      * . .      ******** * .**  **  .*

46              YACCYYSRCTKRGGTGCKKRRYYKRWYCAAARYMTCRYKKRKKRGTGSMYRRYYRWKGRM
50              CTYYYYSGMYGRKTCKAGKRRYYKRWYMRRGGTCCAGYKKRKKRKCRCMYRRTYATTRRM
                 :  ***    *    . *********   .    . *******   .**** *  . **

46              MKKRYGKRYMAACGRKRYGRKKKYRRRKYKCTGGRRRMWYYYYKYRTGTKAYMKWYRMMY
```

```
50      MKGRYKKRYMRRYKRKRYARKKKYRRRKYKAAACARAMWYTTCKCRCACGGYMKWYRMMY
        ** ** ****    ****.***********.:.  * ***   * * .  .*********

46      YYWWKWKTCKYRMRRKRKRKKWYRKRKYKWYKWKMMYYWKSKWKWCGSKMKYYRATTRAR
50      YYWWKWKAGKCGMRRKRKRKKWYRKRKCKWYKWKMMYYAKCKWKATRCKMKYYRCKKGMR
        *******: *  *************** ********** *.***   .******...  *

46      YSYKWMYSKKYYYKRCSCCAAMKCWCCTRYTYSWATGGYWYYKYYWAGCMYGRYMYKRYR
50      YGYKAMYSKKYYYKGTGAGWWMGYTTTCACCTSTRYKKCTYYKTCTRKTMTRRTACTGYG
        *.** *********  ..   *          *       ***      *  *   . *

46      RGATATYKKAWMYYAAAYRMKCGRMYYMMYYRTGTWTTRWMYRRMSYWRGSARAYKYYWC
50      RKTYWWYKKGWMYYRWMYRCTYRRMCCCMYYRYRYTGYRWMYRRMSYWRSSMRRTTTCTT
        * :   ***.****   ** .  **   ****      ***********.* *  .

46      RRCYCATRYMRYCCAYYTAKYMMCMRRYGAACCKATKMKRSYTTSARKYYTAKWRMGMKT
50      GGYCYWGAYMRYTMWCCCCKYMMYMRRCRWRYMKRYKMKRSYWYSCRKYYWGGWRMRAKY
                ****      .**** ***      *  ****** *.**** . ***  *

46      CRKYRMYKCAGAMGTGYSRRMMRMMMYRRWCWCGTGKYGCTKARYYKGTAKCYWRRMYYK
50      MGTCGAYKTTTTACAAYGGRMMRMAAYRRWYWAACKKYKSWKWRYYKAAGGTYWRRMYTK
          .   ** : :  :.*. *****  **** *..  ** . * ****.:.  ****** *

46      WYYSRTRYWCCYTWARRWKWRRKYCCCKKSSYWKWKYKKAMYRRRKWKWWSYKKKKKGYT
50      ATYSRGATTTSYYTGRRWGWRRKYYYYKKSSYWGWKYKKWMYRRRKWKWWSYGTTKKAYG
          ***     .*  .*** *****   ****** ***** ************ ..**.*

46      YTKKTGYRTCAWGRYYGCKTWWSMKSRAGTTACSRKRGYYYGRRKRKATKYYWYKKRWRY
50      CYKKWKCGWYWARRYYRYKKTACMKSRWRGAGASRTRRCYCRARKRKGCKYYWYKKRWRY
          **         ***  *.  .****   :..**.*  *   ****. ***********

46      GTKKTKWAGRKRKAKAAMYYTWKAGYRKMKKKYTWRTAGAYKRRYWRYMKRRYKRYKKMK
50      SYKKYKWTTRKRKMKRRCYTCWKGKYRGCGKKYKTRKMAWYKARYWRYMKRAYKRYKKMK
        . ** **: **** *   *  **. **   ***. *. . ** ******** ********

46      RYRYWKKCKYRKKRWYKKKRCGGMYRKTGKSWYWGYWMYKYYRAAYYKRKMKKYKKMKGT
50      RYRYWGTYTYGTKRWYKKKRTRAMYRKCAKSWYWATWMYKYYRTGYYKRKMTKYKKMKRW
        ***** . .* .********  .**** .*****.  *******:.******.******

46      ACGAYTMCASRWKYRMWKKGYAWRWYYKMKSWYRMMRWRRRAYYARRTTKCKTWYKKTRA
50      RYRWYCCMGGATKYRMWKKRTGWATCCKMKSTTGACGTGGAGCCGAACCGTKCWYKKKRT
            *   ..  *******  .*    ****          .  .      * ****.*:

46      GRRWSCKGYKYRCCYYRGKKYAGKKKWKRTRKRMRRKMGTGGYCYWMKKRCCACTGMRGK
50      KRRWSGKSYKYRTAYCRKKKYGTKKTWKGGRGRMRRKAKWRSCTYTMKKRGARTCACAAK
         **** *.**** .* * ***. **.**  * *****    .  * **** .   .  .*

46      KKRRTACKKMKRCTYCGTKKWRCYSTYYGCKKRRYKMYRTTYYKYRYRKKMYKRGMWRCA
50      KKRRYWYKKMKGYCCYRYKKWRTTCCCTAMTKGRYKMYRYYYYKYRYRKKMCKRKMWRSM
        ****   ****       ****  .   . .* ******  ********** ** ***.

46      ATYRYMKTTARYCGTWTYYGWTGCYSYRRCKMYRKSGTTCAAGKMKYWTMKWWYMYRCTA
50      MWYRYCGGGGRYAAGWGYCKWAAMYSYARTKMCRKCAWWYRMRGMKYTAMKWWTCYATCG
```

```
                  ***    .**.. * *  *:. *** * ** **..       *** :****  *   .

46                CTCRYMRYGYMRMRYYRAYRRYRRYKRAGCGSCRRWMKMKAYYCYAAYSRMYRKKKKKKR
50                TAMGTMRYKYARMRYYRTYRGTGAYKGMATTGGRRWAKMGWCYTCTTYSRMYRKKKKKKA
                   :   *** * ******:**    **  .  . *** **   *  ::************

46                WWWYGMKARCYRWRKYWKGRAACGTGTKRSYKMMWMKMYKRTACCKMYRKKRKMKKYMYK
50                ATTTCMKGRAYRWRKYWKAARCYRCCGTASYGMMWMGMYKRCTTTKMYRKGRKMKKYMYK
                       **.*.********.  .     . ** **** ****  :  ***** *********

46                KYRMWRKKAGTWRAGCYWKWRCAYWRYRKYAMYRSWWKRKRTAYAAWRYGYSMYKKKTYG
50                KYRMWRKKGRGWRGATYTGTRTCYWRYRKYRMYRSWWKRKRKRCRRWRYRYCCCKKTCYR
                  ********.  **.. *   * .******* **********.    *** *.  **. *

46                CGMYWMKYMKMMWWSYRWRYRRKYYAYYKRYWGMRYYKRRRKRRYCTKKKYKYAYKRKYT
50                MRMYWCKCMGCCWWSYRWRYRRKYYGYYGGYWRMRYYGRGRKAGTTYKKKTKYGTKRKYY
                    *** * *   *************.**  ** **** * **     *** **. ****

46                AGRWKKWYKYWMARRKWYKKARKYYKCCGCYRCGGRRYRMRYRYRRWAGRSTAKAAYTKM
50                RKRWKKTYKTACMRRKAYTKCRKCTKTAAGYRYRRRRYRMRYGTAAAGSRSKWTWRCYKM
                    **** **    *** *.*.**  * .. **   *******     ..**. .    **

46                RKWAYRMKKYMSMRKMYRYYWKAKYKKWRWKKRKKTTGMRRSKKKAWYRYMGGGTTGKGC
50                RKWRYRMKKYMSMRTMYRYCAKWGYKKWRWKKRKKKKKMRRSKKKTWYRYMRRRYWSKRM
                  *** **********.****  *  ***********.. *******:*****     .*

46                RRKGKCYKWWWKMRMKMMKRACTGWMRRMRYWYSKWYKKKKYKYRMKKRYRGCAWKKTAC
50                RRKCKTTKWWWKMRMKMMKRMSWRWMRRCGTAYSKWYKKKKYKYRMKKRYGRTWTKKYCT
                  *** *  ************* .  ****    *****************      ** .

46                CKSTCKWWKYKKYKKKRKKTSSRMCRTACSKRKMKSYKKYKYKKCCAGAGMRMCKSYYCC
50                GKSYYKWWKCKKYKKKRKKCSSRMMGYGTGKRKMKSYKKYKYGKYATKTRCRAMKSCCYT
                   **  **** ********* ****   . .************ * .: :  *  **

46                TTRSKKRYKKKKWAKCKKKKWMMMYYKRRRSRRYWKKKRYYWKKKAGRARRRYGRMYKYW
50                CCGSKKRYKKGKWTKAKKKKWMMMYYKRRRSRRYWKKKRYYTGKKWRRWRRRYKRMYKYA
                     ******* **:*.*************************  **  * **** *****

46                KRYKKKCRYGRKKWRRRKMYRKGRTAAYCKWYCGRKGKMARKSMWYKKCRRWCYGCGTMR
50                KRYKKKYRYRAKKAGRRKCYRKKRKCWYYKWYMRGKCKMTRKSMTTKKYRRWGYRYKYMR
                  ****** **  **  *** *** *.. * ***   * **:****  ** *** *    **

46                RKKWYKGYRWKWYRGRKKTAGGRYRKRTTYYKWKYYYYKRKWKMYMRRMKKYWYWRMRYW
50                RKKWYKKYRWGWYRKGKKYWSKRYRGRKYYYKWKYYYYKRKWKMYMRRMKKYWYWGMRYW
                  ****** *** ***  **  . *** *. *************************  ****

46                YYYTWYRYWSKRYKYMRRAKWSYYRKKYKAWYKYRRKAKWKRYKYYYRWYMRTYKKKYRY
50                YYYCAYRYWSKRYKYMRRMKWSYYRKKYKMTCKYRRKMKWKRYTYYYRTTCACCGKKYRY
                  ***  ************* *********    ***** *****.****       *****

46                KRRYKWYYARKKYKTRYRCWTGRTKKKRMYRRCRGYGKMYRYYRMCYARTTWTARACKCG
50                KARYKATCRRGKYTWRYGYWWRRCKKKAMYRRTRKCRTMYRYYGAYYGGAGWAGGWTKYR
                  * ***    * **. **  *  * *** **** *   .*****   *. : *:.   *
```

```
46                  CCATMACRGTTATYAACGACGKATTRTWTTTKKYWRCKGKCCARMGTATWYSSCTMWYYM
50                  YYWYMCGGKYKMATGCMCCMTGGGCRWTCKYKKYWRMGRGYMMAAAARGWYSSMCAACCM
                        *.    . : ..  .   .  *   . *****         .:  ****      *

46                  CRMMRWRYWRAMGYY
50                  YRMMRWRYWRGCRCT
                     *********.
```

The pairwise alignments of two accessions 46 and 51

```
46                  AKYYSKKYWYMRATMYSYRYRTMWATCMYYWATACYYRWWGKTGYSYWWYWRYYMYMYSR
51                  GGYYSKKCWTAGTACTGYRYRWMWGCTMYYWGCTYTYRWWTGKSYGTATYWRYYMYCYSR
                    . ***** *   ::  .**** **.  ****. :  ****  ..*.   ******* ***

46                  RGGGWWATTGKYGGCGWRRYYYWKTRWYYYWYRRYMKSWKTAYWMKKWMWCGCCGGYCRK
51                  GRRRWWGGCTKYKKMRWGGYTYWKYGTTYYWYRRYMKSWGWRYWMKKTAWTATMATTTRK
                        **.   **    *  * ***    ***********   *****  * .  .   **

46                  KTTYYWRMRWYMRGKSAMKWKWMWRYWYKYYRRRYRSTTYRYKRKTRYYRMRCWAYCRKR
51                  GGACYWRMRTCAGKKSRMKWKWMWRYWYGYYRRGYRCKWYGYTRKWRCYRMRYTGTYAGA
                      : *****     ** *********** **** **.. * *.** * ****  .

46                  YTYCCMYMRYCCCYRYWRTTYTRKYWGWAGWWRWYKRWRKTCMCRYYSRKMAGTYRRRKW
51                  YKYTYMYMRYYYTYRYWRYAYGRKYWRWMRTTRWTKRWRKYYMYRYYSRKMGAYYRRRKW
                    *.*  *****   ***** :* **** *    ** *****  * *******.. ******

46                  KRAGCYKKRRYYYYSKKYWWRMTGYMKWYRYMKRATYYYYRYRMKYRMGARYRYYTGRYW
51                  KACAGYKKRRYYYYGKTTWWRMWTYMTACGYMKGRWTYYYAYRMKYRMKTAYRYYAAGTA
                    * .. *********.*. ****  **.   ***    *** ******* : ****:.

46                  RMMKKAYKYRMYRCYWYTTWRKMYKRWWSMSRRGYCCWKSYCYYMRKYRRGWSYRMYKRK
51                  RMMKKWYKYAMYRAYWYWAWRKMYKRWWSMSRRTYMTAGGTYCCCRKCARRASCRMYKRK
                    ***** *** ***.*** :************** *    .     **  *  * ******

46                  YMKRRMKYYWAYTTCYYWMMTYMSWRTYRTCYWRMRTGKWYCWSSYYCCYCGRKKKGAGR
51                  YMKRRMKYYWGYGAYYYWMMCYMCWRATAWAYWRMRKRKWYTWSSYYTTCTAGKKKRRRG
                    **********.* : ***** **.**:   .*****. *** *****    . ***

46                  AKTYMKKKRRWYMRMMRKYGYKRRSRGRAGTYKYWKYYKKSYRRRYGTTYYRKRYRKKKR
51                  RKCYMKKKRRWYMGMMRKYKYKRRSRKRTAAYKYWKYYKKSYRRRYTCACCAKRYRTGKR
                     * ********** ***** ****** *:.:***************  :   ****. **

46                  KRCKRYKKWRWKKRRRMYRAKKMACARARWRMMACYRYMCYRCKKTTAYRRWKSYKSYKR
51                  KRYKRYKKAGWKKRRRMYRRKKMTTTRWRWRMMGTYRYMYYRGKKYCGCRRWKSTGCTKR
                    ** *****  ********* ***: :* *****. **** ** **  . *****  . **

46                  YKMKTRRRYYMSRWWCMMAYRACACWYYAKARTYWRACKGTAAAYRMATAGCTYCRGYST
51                  YKMKCRRRYYAGATAYACMYRWYMYWYYGKCRCCARWMKTCCWGCRMMCGAGAYTRAYSW
                    **** ***** .       **    ***.*.*   *  *  . . **  .. :* *.**

46                  CKMRTGTAYCARGGKGGTTRRWCMKKKRYWSKYRCKRYYAMRMMRWRYRAMGYYRAGYGT
51                  TKMAGACTCACGKKKRTCGAAWAATKKRCTGTCATKAYYCCRAAATACAGCRCTRRACKW
                     **  . : ..   *      *. .***  ..   * **. *       .    * .

46                  SYWYKWRYCACMSYMGWRMKYCWYRMKATTYYRYRYRYYGGYSRGKKRAKWRYYYSTCYS
51                  SYWYKWRCGCTMSYMAWRMKTTTCRMKGWKYCGCGYRYYTTCSGRKKRMKWRYYYGCAYS
                    *******  . ****.****    ***. .*    ****   *  *** ******. .**
```

```
46          WMKAYRAATGYYMKKTRMRMRYCCCRRMKYATATGRYTYSARYRYKMYSTTAWWKSRAYR
51          WMKGYRMTGCTYMKKKAMRMGCTTTRGAKYMYMYRRYGCGGRYRYKMYSGCRWWKGARTG
            ***.** :   ****. ***     *  **     **  ..********   ***.

46          WWWTRMRRKCCKYRRYKWARMYMYWWWWKYRMSKKKRYYKWKTYYYYYKKRWRKGCMMKK
51          WWWYRMRRKYMKYRRYKTWRMYMYWWWWKYRMSKKGRYYKWKWYYYYYGKRWRKAAMMKK
            *** *****  ******  **************** ****** ***** *****..****

46          AAYKWRRYRRMKYYYGRYWSKTTCCTYRKRKKWATTYRKWYRKYKAWYRTCARRKCTCTA
51          GTCKWRRYRGMTYYYKRYWSKCYTACYRGAGTWRYWYRKWYRKTKMTCRATCRRKYGTGR
            .: ****** *.*** *****   . **   .*   ******* *   *: .***

46          AGTTYYYYRKRYTGTRARKTTTYRTTAKGKYRGRYKKMKYCGTKCGTMWWRYCRYSTYYG
51          GTCCTYYYRKRYYAGAMRKGCCYRCGGGKKYRRRYKGMKYYRYKAACCAWRYTGTCCCTA
            .    ******* .   **   **  .  *** *** ***   *..   ***   .   .

46          GWKKMRTRYRKRWTCMYRGYWRYRKKRWYYYKCGRKKRWAKKYWSRMYMKWYKRYRKKKW
51          RTTKMRWRYRKRTCAMYRRYWRYRGKRWYYYKMARKKRWWKKYWSRMYAKWYKRYRKKKW
              .*** *****  .*** ***** ******* .***** ******** ***********

46          AGCKYYYRTGCKRRMRRCYYRKKKKMMWKMKCKRMRRMYRRSYKKKKKRAYWWRRYYATT
51          TCYKYYYGCAAKRRMRRYYYGKKKKCMTKMKSGRMRRMYRRSYKKKKKRGYWWRRYYWYY
            :  ****  ..****** ** **** * ***. ****************.*******

46          AGMRGRKWMKKYYRKKMKMKRYKRSRYRKKKWKTTKKRYTRWRRKKKRKYSTCRRWWWRY
51          MRARKRKWMKKYYRTKMKMKRYKRSRYRKKKWKGYGKRYWAWRRKKKRKYSYYRRWWWRY
               * *********.******************   ***  **********  *******

46          RYKSSWYKRAAAKKRKRYWWKKTKKYYGCMWKYYSRCAMYYKYRYYGTKRSCAMKKAGRK
51          ATKSSWYKRGGGGKRKRYWWKKWKKCCCTAWKYYSRMTMYYKYRYYRKKRSMMMKKRKAK
              *******... ********* **     ****** :******** .***  ***   *

46          YRKRKATYYWAYKKRWKAAKKRKTCAKRGYKSRMMYRYRKKMWKTRRMRRWGRYYKKMRR
51          TRKRKGCYYWTYKKRWKMRKKGKKMTKGRYKSRCMYGTGKKMWKCRRMRRWARYYKKMRR
             ****. ***:******  ** *. :*  **** **   ***** ******.********

46          RSKKWYRYMWKGWRYAGAWTTKWWSRWTTCCGYSWACTGWMWYMYSKKKRYYRKKRMTCC
51          RSKKWCRYMWTKWRYMACWCCKWWSRWWKYYKYSWMGAATMWYMTGKKKRYYRKKRMWAY
            ***** ****. *** ..*  ****** .   ***  :. **** .*********** .

46          KSKWKWKYKKGAYYRYRKYYRKMKRKKTGYRRGMAKKYRRMRSYRYAACWYKYKMRMKCT
51          GSKWKAKYTKRCYYRYGKYYRKCKRKKCKCAARMTKKCGAMACYRYCCATYKYKMRMKYC
             **** **.* .**** ***** ****      *:**   * .***... ********

46          TAAKSRYRRCMYRKYMMKMKYKGCKKRRYKGRRRYKMMTRYKRWCRKKRKTCTKMKKWGY
51          CCMTSRYRRACYGKYMMKMKYKSYKKRRYKRGGGYTMMCRYKRAMRKGRGCTYKCKKWRC
             . .*****. * *********. ******    *.** ****  ** *    * ***

46          KYKYYMWKKYGCGRRKTAMMYRCYYKGMWKKKKMYCRWKYYCGRKYRKRSYRGRYWRMSR
51          GYKYYMWKTYRYRRRKAWMMCAMCYKRMWKKKKMYMGAGCTTKGKYRKRSYRRGTAGMSR
             *******.*   ***: **    ** ********         ********     ***

46          WKKMRKRRGMMARKRRYRRRMKRKKATCGRYYWRCSCCRYYYWYKYGRTGSYYGAYTKYR
```

```
51              WKKMRKRRTCCWRKGRYRRRMKRKKGGTKRCYTGTGAGRYTCTYKYKACACCCKTYKKTG
                ********    ** **********.   * *   .. **   ***   ..   :*.*

46              MWMRCYSYYMMYYRWYKKRCYGRMKGYRMYGMYYRGYSKMRCMAGTMRYSKYWKRKATCR
51              CAMGAYSYYMMYYRWYTTGTCRRMKRYRAYSMYYRRYSKMGTAWRCCGYSKYAKRKTATR
                  * .***********..    *** ** *.**** ****        **** ***:: *

46              AYRRYKKYARYRWYMKRRKKKMKMRYYWYGGMKKCAKMRYRRKKMRKAKKWRKKCGKKK
51              GYRRYKTYWRYRTYMKRAKKKKMKMRYYWCAAMKKYRKARYRRKKMRKMKKWRKKTAKKK
                .*****.* *** **** *********** ..***  * ********* ****** .***

46              SKWMGYGMYKKKYYRYYYYTKKTKKMMGTACGARTWCKAMTYRMWSWRWYCRYRKMKYSR
51              SKWMATKMYKKGYYRYCYCGGTWKKMMRWRYRWRCTSKWAAYRMWSWRWYMGYRKAGYSR
                ****.  **** **** *   . ****      *  .*  :*********  ***  ***

46              SYCRMYYYWWRRKCATGCTRRMKWKKGCARAYMYKTMTCYCTTKTTGTRYCTYRRWYATT
51              SYTRMYYYWWRRKMRKSMAARMKWKKKTGGWCAYKWCYYTAGYKWWRWRYMCTRAATRWW
                ** **********  .. : ******  .    **     .  *    **   *

46              RRMMKRWWYRTWRYKYYRKWWRRRRWGSMYRKSWRYYRYWRYYTRYWYAGKYKKACMACG
51              RRMMKGAAYGWAGYKCYRGWAARGAARCMTAKCWGYYRYWGYYGATTYGTKYKKGTMGTA
                *****   *    ** ** *  *    .*  *.* ***** **    *. ****. *. .

46              WAKKKYYYKAYKYYKTWYKRYYYYKTCKKKAAKKAGKKKRKACMMRKKRKGKYRYYCRKS
51              ACTKKYYYKRYKYYGAWYGRYYYYKYYKKKCGKKWRKKKRKGSMMRGKRTRGYRYYARTG
                 ..****** **** :** ******  ***..**  *****..*** **.  ****.*..

46              YYCMRSSRMYRSSTCGYAYTGYKYYMMRYRCCYYCRYSKRYGAKRRYRGRRKTGCTTRRC
51              CCYMRSSRMYRSSYYRYGYYSYKYYMMRYRMYYYTRYSKRCRCKRGTAKGAKYKYKYRRG
                   **********   *.* .*********  ** *****  .**      *   . **

46              TKKYYMRRTAAAGYKRCKMKRKRKKKKRCAKRATRGATMMMRWRRRYYARRTTKAKKYRT
51              AKKYYMRRWGCTTYKRTKMKRKRKKKKGMRKGTYGCGAMMMRWGRACCGAACCGGGKYRY
                :******* ..: *** **********   * :   .:***** *   .     . ***

46              AKYYCKKGCKYYRRYYWAAKMTWMYTTCMRCWRAKKKMWKMRRRRKYKKAGTRKYGKWRC
51              GTYYGKKKTKYCGRTTATCKMYWMYACAMRTWRRKKKMWKMRRGRKYKKRRYAKYRGWRY
                ..** **  **  *   :.** ***: .** ** ********* *****    **  **

46              GRCSKMRTYAASTTKMGMYGRKKRKKGCKRGMYTKMMMYRRGTRYMRKKYRSWKRCAAGK
51              AGTSKMACTTGCYGGMKCYKRKKRKKRYGRKCYGTAAATRRRYRYMRKKYGSWKRYRMRG
                .  ***   :..   *  * ******   *  * .    **  ******* ****

46              MMWTGCRKMAWYYRKYRYRKATYYRMWAAYKTTGKAYARSKWSYAGCYKYKKWYACYYGR
51              MMWCATRKMWAYYAKYRYRGMKYYRMWMRYTCCAKTYMRSKWSYCRYCGYKTAYMACYKG
                *** . ***  ** *****  .*****  *.  .*:* ******.    **. * . *

46              RYYWCCCWKRKKKRKYKWTRMRTCKYYTWCRGKRYYRYYGGKARSYWGGTRKMRMTGGAR
51              GCCTMMMWKRKGKRKYKWARMGWMGYYKWMRKGRYYRYYAAKGACYWSAYAGMRMYRCTR
                       ****  ******:**    **.* *  ******..*. .**..   ***   :*

46              SYRSMWRYAGCCGSMMATRRKRYRAGYKYAAKKYTWWYKKGTMRMYKMKYWWYRRMKRTC
51              SYRSMTACGAYMTGAMTGRRKRYRRRYKYTTKKYCWWYKKKYMRMYKMKYWWYRRMKRYM
```

```
                    *****   ..   . *: ******  ***::*** *****  ***************

46                  TAGRWWRYYYKYYRMTWKKYRWRWKYKYYRKTGCKMYKKYRRKKGKCYGYYRWMRYYKRY
51                  YRRRTAAYYYKYCAMGWGKYRWRWKYTYYRKCAATMTKKYRRKGRKYYKYYRWMRYYKRY
                       *   *****  * * ********.****  ...* ******  * * ***********

46                  KRYMKRYKKRCCKKKYYSKYRMYWRKMWYYWMGATGGRKKYKYYKCGSWTGYTTRWGRYC
51                  TRYMKRYKKRTYKKKTCSKYRMYWRKMWYYWMRWKSARKKYKYYKAASWWRCYARWKRCT
                    .*********  ***  **************   ...********..**    :** *

46                  GGYRRYRYTRYAKRRSKSYATKCAAYTKYGRKKKMYRYYRYTYRYYKKYYKWMMCCKYAC
51                  AAYRRYRYKRYRKRRSKSYTYTSTGCCKYARKKKMYRYYAYYYRYYKKYYKWMMTTKCGT
                    ..******.** *******: ..:.  **.********* * ***********  * .

46                  CMRRKYMCCTRKTATGYKCRMGMWMRRKYKYGWWGCWYYRRGTTKYRKYYRKTKWKRKMR
51                  TMRRKYMTGGRKGCGAYKTGMKMWMGRKYKYTWWCYAYYRRKCYKYRKCYAKYKWGAKCG
                     ******   ** . .**  * *** ***** **   ****   **** * * **  *

46                  RKMYYYMRCYYSRKRWWYSYMMKKKKKTCACRYYYGKKYRKRGCKGTSRRYYGATAYYWM
51                  GKMYYYARYTTGGKRWWYSYMMKKKKKYYMMRYCCTKKTRKRTTTKGSRRYYRTCRCYWM
                     ***** *    .*************    **   ** ***  .  ***** :   ***

46                  KRAYKYGMKKMTAGWKYRYMSRRKGRWRGCGRCAWTMRAKMGRKYMRRAKKKRYCYYTYR
51                  KRRYGYKMKKMYWAWGYGCMSRRKRRWGRAARYRAAMGRKMKRKYARRTKKKRYTYYCYA
                    ** * * ****  .* *  ***** **  ..*   :*  ** *** **:***** ** *

46                  MKMRRWRKKCKKWMRSWWRKMYAGGRKGWWSRYKAGTCKKYYKRWTACACKRYSWSAKTY
51                  MGCGRWGKKYKKWMRSWTRKMCWKSAGRATGRYKMRCYKKYYKRWKWYCYKRYSWSTKCY
                    *   ** ** ******* ***     .     .***    *******.  . ******:* *

46                  RSMRSKWAYGRWKMYKYTRYRGTTCACRKRMKKMRKYYKYMCYRKRKRKRKKYRRRKKYW
51                  RSMRSKATYRRWKMYKYWRTAKKWYMYRKRCKKARKYYKYMYYRKRKRKRKKTGGGKKYW
                    ****** :* ******* *    .     *** ** ******* **********    ****

46                  YKYRYKRRKYWYRMMYYYRTWRKKRRAKKMRKRWKRRRRYCYKRAYKWYYYRYRKKKMRK
51                  YKYRYKRRKYWYRMMYYYRYWRKKRRWKKMRKRWKRRRRYTCGAGCKWYYYRYRKKKMRK
                    ****************** ****** *************     . **************

46                  YATKYWGTTASKWKWCSKKAWRRKRTTSYCCKMKCAAKYSRMYRKKYKKYRRKYYMRRKK
51                  YGCKYWACCCCKWKATSKKMWRRKRKKSYYYKMKTTTKYSRMYRGKTKKYRRKYYMGGKG
                    *. ***.  ..***  *** *****..**  *** ::******* * *********  *

46                  RWWYMCYRWKKCGWAAKRSYYKYCKGMYYCAWTACCKMYRCRYKWKMKKKMMKRTTMKKS
51                  ATTTMMYRWGKYRAGRKASYYKCMKKMYCTGWYWYYKMYRTRYKWKMKKKMMKRKKMKGS
                        * *** *   . * ****  * **  .*    **** ************..** *

46                  WRTYWRYRKTACRSWWCKYKRKKYRYKAARKRMAYWKARYSMYKKYKYTYYMAGMGKYMR
51                  WRKYWAYRKCGYRSWWTGTKRKKYRYKTTRKRAMCATGRYCCYTKTKYKYTCMACAKYMR
                    **.** *** . ****   ********::***    ..**. *.* **.*   . .****

46                  MYKYGWWSYW
51                  MYGYAWWSYW
                    ** *.*****
```

The pairwise alignments of two accessions 47 and 48

```
47              RRGGKWYTMWKKCARWKRRKAGWYMYKCRYYRARWCYRYRRATYYRYWKMYKRYKMYSRT
48              RRRAKWYACATGYRGWKRRKCATCMYKYGYCRTGTYYRYRRGCYYRYWKMYKRCKMYSGY
                ** .***:  .    *****..  ***  * *:   *****. ********** ****

47              YYGCWAMKYRKTTAGRKRWYYYYKRYWMWRYWKYWKGCTAYARMWYYGRWYKYGTCCMYW
48              YYKYATMKYRKYWWRRGAAYYYYKRYWMWRYWKYWKATWMTRRMWYYRAWTKYAAATAYW
                **   :*****    *   *****************.     *****  * **.:.  **

47              WMKRAYRKTMCAARRYRASTRKWRYSRRYYMRYMRWKRRCCWWCGCRWYKKRYSMYRMYR
48              WMTGCYRGYCGRTARYRMSWRGWAYSRRYYMRYMATGRRYYWTYRMAWYKKRYSCCRMYR
                **. .**     : *** * * * **********   **  *     *******  ****

47              WRRMYRYSYYYRRYYRRWMRYYYMGGCYYRGRWKYRRCACYMRARASSCGMMMRKRMMMT
48              WRRMYRYSYYYRRCYRRWMRYYYMTRMYCRRRWKYRRYWTYMRWARSSTTMMMRKRMMMC
                ************* **********   * * ******   ***   **  *********

47              GYRCKWYYRYYMRCWWRRSRKWYKWYYKWMKSYWYKRWRRRWRWTRRYMYKRKYYWYAMR
48              RYRMKWYYRYYMAAWWRRSRKWYKWTTKWAKSYWYKRWRRRWRAWARYAYKRKYYWYGMA
                 ** ******** .***********  ** *************   ** ********.*

47              RRYYRRYMKKWWYRKYRWWTYARWYRRYWGGSYYYYYWTRMKYMYWYRRKMYMGRYMKRW
48              RRYCRRYMKKWWYRKYRWWWYRRWYRRYTKTCYYYYYWWRMKYMYWYRAKMYMSRYMKRW
                *** *************** * ******   .****** ********* ****.******

47              RRYRRGWKYYGKKTACCMKKRKKRYYYYSWRRAYYYKYYRYWYWKYRTGTGGRYKKWKTY
48              RATAGKWKYYAGKWWAYMKKRKKRYYYYSWRARYYYKYYRYWYWKYRKAYSTGYKKTKYY
                *     ****. *  . **************  **************.. .  *** * *

47              KKYATAKRMYTKMKKMKMRYMKYACYYKRGKYKKTRMYKRSYKRCACCATTYWTTRMGWK
48              KKYMAMKRMYYKMKKMKMRYCKYRYYYKRKKYKKKRMYKRSYKRYWSYMAAYWWARMTWK
                *** : **** ********* **  **** ****.*********  .  ::** :** **

47              KRYYKKYYAKRRKYGCAKSYWKKMKRYYWAMRYYRRRCYKYYSKWYYKYMKAATTWYWTA
48              KRYYKKCYGKARKYRYWKSYWGKMKRYYAGMRYYRRRSYKYYSKWYYKYMKGTGKWYWYR
                ****** *.* ***   **** ****** .*******.*************.: .***

47              TYYCYRYRTTKRAYGWYMRWRRMTYKYKMYGTRYMYYSMRWYYCCYRKWYWKMYSYRRMR
48              KCTAYRYRYYKRCCKTYARWAAAWYKYKMYRYRYMYYSMRWYYTTYRKWYWKMYGCRRMR
                .  .****  **.   * **    ******  ***********  *********. ****

47              RRRYCYARYYSYTTKCCYKTGARSKKMRGSTRMYWKRKRKTRTRRKKYAARSWYGRYSYY
48              RRRYYYGGCYSYAGKYMYKACGACKGCGKSKGACWKRKRKYACRRKKYRGRSWYARYSYY
                **** *.  ***: *  **: . .*    *.   ******   ***** .****.*****

47              RYRKCYGKRCATRCTWYKGKYRYKKYWRKKGWYYYCRRRKKKMRYKTGYMKRMKCCRRRY
48              RYRGYTRKRYGGGTKWYKKKYRYKKYWRKKKWYYYMGAGKKTMGYTCTYMKRMGMTRRRY
                ***    ** .   .*** *********** ****    **.* *.  *****   ****
```

```
47                  YRRYTTWRKYRSKYKKAKKRKKMMWGYTKWMRKYKMTYRKWKRRMKKYRWKMSRKWKRYW
48                  YRRYWYWRKYRSKYKKRKKRKKMMWRCCKWMRKYKMKYRKWKGRMKKYRWKMSRTWKRTW
                    ****  ********** ********   ********.***** ***********.*** *

47                  WRYKGYKGGAGKRRKYRKYRSYYYKYRKKRYSKWRYRRWAMMRKRKKMTMMYYKRGTYAG
48                  WRYKRCKRSMKKRRKYRKYRSYYYKYRGKAYSKWRYRRWRMMRKRKKMKMMYYKRRWYRR
                    ****  * .  **************** * ********* ********.******  *

47                  KMMMMMWKWRYKKKMRRGMKMWYYRRMRGRTTTGTKMRYKCWGGRYYYRCWMSKGRRGRY
48                  KMMMCAWKWRYKKKMRRKMKCTYYRRMRKRGAYACTMRYKMWKARYYYRYWMGKKRRKRY
                    ****  *********** **  ****** * : . .**** * .***** **.* ** **

47                  YYKYRRKCWTAKKYWWACTCMMMTTACYYGCRWRMMMRKYTGTCGYYMWAWYYRSRKTYR
48                  YYKYRRKTWKTKKCTWGGWYCMMCAGAYYRYRARMMMRKYYSYYRYYMWTTYYRSRKYYR
                    ******* *.:**  *.    ** :..**  * ******* .   ****: ****** **

47                  TRYYGRGRWRKRRRYYYKTYKKKKKRRKCSWYTCGMYYKMKRGKMRWYMMATYRRYWRMY
48                  YRTYKRKAAAKRRAYYYKYYKKKKKRRKMSWYWMRCYYKMKRAKMRWYMMTWCRRYWRMY
                     * * *    *** **** ********* ***    ******.*******:  *******

47                  KMYYYYSKRYSYKSMAMRYYWYTTKKMKCKRYKYRWKRYRKKKYRYRMKSYSYWYWRTGG
48                  KMYYYYSTGTGYKSMMCRYYWYCAKKMTTKRYKYRWKRYRKKKCGTAMKSYSYWYWRCTT
                    *******.  .****  ***** :***. **************    **********

47                  WGRMKYYKKKYMRKSYMKSRKKMRMRKWKRMRYRYKRWYYKKKRRWRWKMSMKWKYKKYW
48                  AAAAKYYKKKYMRKSYMKSRKKMRAGKWGRMRYRYKRWYYKKKRRWRWKMSMKWTTGGTW
                     .  ********************  ** *************************.    *

47                  MYYKRWWRYAKMCTGMYKKYKRYYWRKKKWWKARSWKMKRTRKACCKYRRKAAWTRCTGW
48                  MYCKRWWRYTKAMCKCTTTTTRTYWRGKKWWKTRSWKMGRYGTTATKYRRKCCTGRYKKW
                    ** ******:*      .. .* *** *****:***** *  .:. *****..  * . *

47                  KRRSRRAWTCRRKYCCYKYKYKMKCGGRWKRRYRRMRKKKYARGYWYWKCKRYKTRYTTW
48                  KRRSRRTTCTRRKYYACTYKYKMKYRRRWKRRYRRMRKKTCGGKYWYWKMKRYKKRYACA
                    ******:   **** . .******   ************. .  ***** ****.**:

47                  GMYRATGKRRGKYMMRYKMMYSYYSARYYKWKTGYKSWRMRYRKWRGTATTRKYYAGGGT
48                  RAYRRGAKRGKKYMMRYKMCTSYYSMRYYKWKKRYKSWRMRYRKWRKAGAARKYYWARAA
                      **  .**  ********  **** ******. ************ :.::**** . .:

47                  KKRYYCAAMMYYYRYYKCGMRYSMKMRTRKKWKMTYKYKKRKRRGYKYYYMWTRKAKYKY
48                  KKRYCYTRMMYYYRYYGYRMRYSMKMRKRKGWKMWYKYKKRKRGKYKCCTCAGRTTKYKY
                    ****  : ********   ********.** *** ********  **      *.:****

47                  WWWSCGRYRGTMMYRRKKRGCKYMRKKRYKKWKYYSRCKKYYMARAGKYGCRRKGRYYRG
48                  WAWSTKGCGKWMCYRRKKRRMKCMRKKRYKGWKYYSRYKKYCMWRRRTTRYGAKKRYYRR
                    * **       * ******  * ******* ****** *** * *  .     * ****

47                  GYRMKTCKKKGRMKGRYKRWMMMWWRKRYCCTRMWYRCYCYYYGTYRKWMYRSRTRSMKT
48                  KYRMKKYKKKCGMKKRYKRTAAMWWRKRTYMKAMWYRYYMYYTRWYRKWMYGSRWRSMTK
                     ****. ***  ** ****   ******   . **** * **   ****** ** ***..

47                  RYSMRKKRMRSKMAMWWYYRYYYRWTAYTRKKRYRYYRKKRRKSYKKRYKWYMYWRSRCA
```

```
48      RYSMRKKRMRSKMWMWWYYRYYYRWYGYYRKKRYRYTRGKRRKSYKKRYKWYAYWRSATT
        ************* *********** .* ******* * ************* ****  :

47      CMRWGKTKRKKKKWRSYWKGRCKWTCTRRKRYYGKWKCGKKKCCAKKKCAAWRRGAMRMY
48      TMRWKKCKRKKKKWRSYWKKRTKWCYGGRKAYYRKWKYSKKKMYRKKGTGCTRRRRMRMY
         *** * ************ * **    ** ** *** .***   **  .. **  ****

47      YKKKKTTYKWRKRGWTTTTRGRAKRYKRYYTKRGCKRSKCYKRWYWYKCCTARWWKWWKY
48      YKKKKAYCKWRKRRACCCCRAGWKRYKRYYYKRATKRSKYYKGWCWCTTTWTRWWKWWKY
        *****:  *****      *.  ******* **. **** ** * * .   :********

47      KRYRRMRYMRKWKKMWYRKRSKKWKRYRGYGYYMKTCGKTARMYCGMWKKWSRYKYKKWY
48      KRYRRMRYMAKWKKMWYRKRSKKWTRYRSYRYYMGGTRKCCAMYAAMWKKWGRYKYGKWY
        ********* **************.***.* ***    * . **..*****.**** ***

47      KYTMRTTAKWAKRKKSWYRKWCTTTKRWYGGYCWYRWKKWKYKRRRGYMKKKTCRRCCGA
48      KTKMRYYRKWGKRKKSWYRKATCKYKRWTAATTWYRWKKWKYKRRRRCMKKKGTRGGMKW
        * .**   **.*********   . ***  ..  *************  ****  *

47      CGCCCSKKKYTYRRKRWKYWKWKYTKYWRYGCRRKMKRYRYRGCYGYSRMYTTCATYKYC
48      TRTTTSKKKYCYGRKAAKYWKTGCCKYWRCRYRRKMKRYRYRAYTKTGRMYCCMWWYKYT
             ***** * **  ****    ****   **********.    .***     ***

47      GSWWKYYKRSRKKRCRRAKCYYYYCRGAGTKKYYRMRRKYKYRKRKRKYMKMRMAYSWMK
48      RSWWKYYKRSRKKRTRATKMYYYYYGTTTCKKYYGMRRKYKYAKRKRKYMKCGCRYSWMK
         ************* * :* ****   :  **** ******* ********    *****

47      MYSMWKMKKWMKYCWRRKRYTKRYYKWRRMMTTKRCCATCKMYCRYSYRKMYWYRTKWMS
48      MYSMWKMKKWMGCATRRKRYAGRYCKWRRMAKKGRYAMWYKMYMRYSYRKMYWYRYKWMS
        ***********  . *****: ** ***** .. * .   *** *********** ****

47      WYKYRYKTRAWTRRYYWYWYSKYYAGKATWWYWKYRKGGTGTKRMRKYCCTTAKWKTRAC
48      WYKYRYKGAGAARRYYWYWYCGCCCAKGAAWYWTYGKCAAACKRMRKYTTGGMTTKCRCY
        *******  . :********.   ..*.: ***.* * .:. ******     . * *.

47      CGRCRRWRYRRGSKRTCCAYYKYCKKGYAKGRCRWRTKTKSKMKWRKKGKKKKRYKKYYK
48      YRGYGGTRYRRKSKRWMYWYYKYTKKRYTKKRARARKKYKSKAKWRTKKKKKKRYKKYYK
               **** ***    **** ** *:* *.* *.* *** ***.* ***********

47      KYYCKKKKRWYKRRRKRRYYKRRWSCRYTGAGGRKMRYRRWTMKRRYKMYRRCGKAMCGY
48      KYYTKKKKGTCKAGRKRATTKRRWSMRYACCAKRKMRYRRWKAKRRYGCTGAYKKWMGRY
        *** ****   *  ***   ***** **: .. ********. ****       * *  *

47      TCTMCCYKKKWRSKYMRRWRACRKCMTRRKWRKYTRKMAKRRYRKMACYWWKKKKCKKSR
48      GAGAAAYKKKTAGKYMGRWRWYGKTMYRRKWRKYYRKMRKRGYRKMGAYWWGKKKSKKSR
         .  ..****  .*** ***   * * ******* *** ** ****..*** ***.****

47      YRRKKYKKKKMRWYRYSMYRYTAKRKKYRKKRTCGKATRAMSGGGCWAGSKRKYYATYYY
48      YRRKKYKKKKMATYRYSMYRYWWKGTGYRKKRATAKGARRASRKRYTGASKRKCYGCYYY
        ***********  ********  * . *****: .*.:*  *     ..**** *. ***

47      KCYRMRRKKKKRRKKMWYYYMYMKWRYRWKYKRYTKYRYYKKYKTYYSYMRYKKYSYTKY
48      KYYRCARGKKKGGGKMWYYYMYMKWRYRWKYKRYCKYRYYKKYKAYYSTMRYKKYSYAKY
```

```
                 * **   * ***    ****************** *********:*** ********:**

47               KKWMMKWSGCCMKRKYAGKKRYRWYYRMYKRYYYYYRWKWYRCYKWCMWKRKMRKKRKKK
48               KGWMMKWSAYAMKRKYTCGKRYRWYYRMYKRYYYYYRWKWYRTYKAYCWKRKMRKKRKKT
                 * ******. .*****:  ********************** **   ***********.

47               MRKYRYYTAWYRTKYKRYKYKTTRKGCYKRKKMYGACAWYRRKYRWKCTCCRYMRWACKY
48               MRKTRYYYGWYGKKYKRYKYGYAGKKYYKRKKMYKWYRWYRRKYRWKTKYTRYMRWRYKY
                 *** *** .** .*******  : *  *******    ********* .  *****  **

47               KYMYYKKRRKRCGCGRTTKGKKYRRKWSTYWWKKMMKKYRYYWKMRYRRCCTKCGKYRAT
48               KYMYYKGRRKRMSYAGYYGKKKYRRKWSCYWWGGAMKKYRYYWKMRYRRYYYKYAKCRGC
                 ****** ****  . .      ******** ***   *************   * .* *.

47               AKAWSMRKSRWRYWCTKKMYYRKKRMRRMAAMRMSMYKKRYYMRKKYWYTWAWAAYYYRW
48               GKCASMRKSRAGTAMCKKMYYRKKGCAGCWMMRMGMYKKRYYMRKGYWYCAGTTGYYYRT
                 .*. ******      ********       ***.********** ***   . :.****

47               RCRMWMGKMRRYCCCYRWYWRMYMMTKWYYRKMMMMKWWKRMKYKYYGKWKYYYSYRYTG
48               GMGAWMKTMRRYYMTYRWYWRMTMMYKWYYRKMMMMTWWKRMKYKYYKKWKYYYSYRYCA
                     ** .****   ******* ** **********.********** ********** .

47               KKKKYRYKYYRWSRKRRKYYYRGCTGCTMWKRKWRRARWGCSYKKWRWRKRRRKKRKKYY
48               KKKKYRYKYYRWSRKRRGYYYRAAYRYWMWKRKWRRWRTRMSYKKWAWRKRRRKKRKKYY
                 **************** ****..     ******** *    ***** ************

47               KKRRKKYKKTKKYYCAAWWYRMYYMMRRWWSMAYMTTKYGACKKYKYKTRMYRYKKWYYY
48               KKRRKKYKKKKKCTYGGWWYRMYYMMRRWWSAGYMCCKYKGAKKYKYKGRMYRYKKWCCT
                 *********.**    ..*************  .**   **  ..****** ********

47               MRYRMKYAAAYYMRMRKYMWKAGWRMYRRKKWMRRGKTYYRKYYWGRRAKRMYYKWYWGA
48               CRYRMKYWRWYYMRMRTCCTKRRWACTRRKKWMRRAKKYTGTTCTRGRRKAMYYKWYWKR
                  ******    ******.    *  *    ********.*.*    .      * * *******

47               TRYGMYRYKKKKYRRWCRYYYYKKKMRYRYYKKWTCYRKKRWTKYAMYAYRYKRRRKWKK
48               KRYRMCGTKKKKYRRWAGYYYYKKKMRYRYYKKWYTYRKKRWWKYGMYWYRYKRRRKWKK
                 .** *    ********. ***************   ****** **.** ***********

47               RYRTWMRRKRACKKMMMRKRYRRKKKYKYCKCCYWKMAAWMYKTRRKWKKKYGGGYYWCK
48               RYRWWCGRKGTSKKMMMRKRYRRKKGYKYYKYYYWKCRGTMYKWRRKWKKKCRATYYWMK
                 *** *  ** :.************ *** *  ***   . *** *******   . *** *

47               KATKYKKARRRTCCCRYYTYRYKWKKRKRRYKRWWWYKWWRKRKYYRRRKKRYYRRATCK
48               KCCKYKKRRRRYYYMRYYYYRCGWKKRKRRYKRWWWYKWWRKRKYYRRRKKRYYRRWWMK
                 *. **** ***     *** **  *******************************    *

47               YRMYRWYKYYRKKRSKARCCRKYYYKRMKWMRKMYKWWKTCCYMYWYYKYRYRCTACYRK
48               YRMYRWYKYYRKKRSKRAMYRKCYYKRMKWMRKMYKWWKCTYTMYACCKYRYRAGTTYRK
                 ***************     ** ***************     **    *****. : ***

47               RYRMRKRYYYYYTKYYYARRTWAAGRWRYSYRRWRAYYRARWKYKARYTYAWTKSKYGAAT
48               AYRMGKRYYYYYKKYYYWRGYTTWKAAATGYARWGWYYRGGTKYKRRYKCTACKSKYSRRY
                  *** ******.**** *    :       .* **  ***.  *** **. :  ****.
```

```
47          KWYYCGMKKKRRKRYKRKKKYKWMWGAAKKAWCKYYYYYTMKYKAWYYYGACCTSRRYRY
48          KWYYMRMKKKRRKRYKRKKKYKWMTSRRKKCTTKTYYYYKMTTKMWYYYKRATASRRYRY
            ****  ****************** .  **.  * ****.*. * ****  . :******

47          RKAMRKYRKKKWMAYRWTRWYKYRYRSMWRKKRKYRRRWWRRYKYKKMGAYRYWRYATCA
48          RKRAAKTRKKKWMTYRWCRTYKYRYRSMTRTKRKYRRRWARRYTYKKMKTYRYWRYMATW
            **   * ******:*** * ******** *.******** ***.**** :****** :

47          YRYKKKTCCWMKGTYWKYSMRSGKYYKTCAGCKRRMRWYTAGWTWKSYWRCACYRRSKWK
48          YRYKKGWTTWMKAYYWGTSMRGCGYYKCMTRTKRRMRTCCTATYTKSYAAGCYYARSKWT
            *****    ***. **  ***.  ***  :  *****   :.   ***   . * ****.

47          KMYAKGCARTSAGYGYRYYKGKYKRYRTCYKYMYAGAAAWRRYWWYRMYSKMRWMKCMYM
48          GMYMKTTTAGSGCCACRYYKAKYKRYRKYYKYCCMRRMRWRRYTTTRMYSKMRWMTYCTM
             ** *  :  *.  . ****.******. ***       ****   *********.   *

47          CGYTYKSKKKWKTRRYWRWMTYKKRGKCYRKYKSKWYAACCMYYYKRYKKKMKKTATRKK
48          ACYWYKSKKKWKWRRYWRWAAYKKGATTYRKYKSKWYCTYGMYYYKRYKKKMKKCRARKK
            . * ******** ****** :*** .. *********.:  *************  :***

47          TRAYKYYWYYYWKKYRRWRGAGYKRRAKGWRKWSWMMYRMRKKSYRRRKAWSKMRGRTGT
48          CATYGYYWYYYWGKTRGWRAMTYKRRMKRWRKWSWMMYRMRKKSYRRRKRWSKMRKRKAC
              :* ******* * * **.  **** * ******************** ***** *..

47          YACKGSMKKRMKKYKMYRKKTYWMYMYGYWYKWRKKRKKYW
48          YRYKRGMKKRMKKYKMYRKKKYWMYMYRYWYKWRKKRKKCW
            *  * .**************.****** *********** *
```

The pairwise alignments of two accessions 47 and 49

```
47              RTYYYWKMKRWTWMYRYRRKKKWYTWYRGTCYMAWAKWRARYRWYWKYRYRRRRMWTYWY
49              RAYYYWKMKRWKWMYATAGGKKWYCWYRKCTYAWTWTWRRRYRWYWKCAYRRRRMTACWY
                *:*********.***     **** ***   *    .** *******  ****** : **

47              MWRRYKRRSCWRYMKSWRRYYARWKGYWCAMKWMMKRWWKRYRRKYMWYYWRYGRWCGKW
49              MWRRYKRRSTTRYMKSWARYCTGTTKYWMCAKWMMKRWWKRYRRKYMWYYARYKGATTGT
                *********  ****** ** :  . ** . ******************* **

47              YYRKYYWYAKYYYYYYCARYRRASTRKWRCCARYYMRYRKKRWKYRRKTRRAYMYYYRYA
49              YYRKYYWCRKYYYYYYYRGYRGMSWRGWAYYWRYYMRYRKKRWKYRRKYRRTTATYYRYW
                *******  *******   **  * * *    **************** **:   ****

47              CYYKKKYRKCRYWRCCRKKRSGWGRWYKWYYKMYYSKMMKSYWRYKRMWKRKRMWYRCRK
49              YCYKKTCRKYRYWGYYRKKRSRWRRWYKWYYKMYYSKMMKSCAGCKRMWTAKRMWYRYRK
                  ***. ** ***   ***** * *****************    ****. ****** **

47              RWRYRRYMKGMMWRWRYYMRSYYGCGRMYRKYWKRMMMYYWGRYYRMCTGGAAAYRKYMY
49              RWRTARYCTRMMWRWRYYMRSYYSYRRMYRKYWKRMCCYYWSRYYRMTCTAGGGYRKYMY
                ***  ** . *************.  **********  ***.*****   ....******

47              KRYYRRSMWAYKMKWRYYRMRWRRWYWRYMMWRYMAGWMYRMYWKYMYYWTCCWWKKGRK
49              KRYYRRSMWCYKMKWRYYAMRARRWYTRYMMWRYMGSAMYRMYWKCMYYWAATWWKKRGG
                *********.******** ** **** ********.. ******* ****:. ****

47              MYYRYMYKKRRWMMKYMMKYYYMRRRYRYRYYKYYWYRYKRRKKYGWMYYRKCGKWRMRY
49              CYYRYMYKKGRWMMKYMMKYYYCRARYRYAYYKYYWYRYKRRKKTKTCYYRKTAKWRMRY
                 ******** ************ * **** **************    **** .******

47              YWRWCKKMMKYMKAWYRCGYGTTRKKRYRRYWMTKYACCYRKKCSMKSRMYTKMYYRKSK
49              YWRWMKGAMKYMGCTYRMKCAKCRKKRYRGYTCKKCRYYYAKKGSMKSRMTYKMYYRKSK
                **** *  **** . **   .. ****** *  .*    * ** ******  ********

47              RSRKYYTGTATKWKYKKYAKKYSYTWAMMMGRKKMWACTAGARGTGAYRMMTYCKAGTMM
49              RSATYYYAGTAKTTCKKCRKKYCYYWMMMMKRKKMAGTGRAGGAKCMYRCAWYYKMCCMM
                ** .** . ::* . **  ***.* * *** **** .   .. ..  **   * *   **

47              MMWACYRWWKWRMYMMTCSYKYYYWWKAKGRYCYAYSRWYCWRRRYWYWTKRTMGMRGRR
49              MMWRYYRWWKWRMYMMYTGTKYYYWWKMKTGCYCMYSRWYYTRGATTTTCKRCCTCRKRR
                ***  *********** .  ******* *      *****  *       **    * **

47              RKRKTMWSWWWSWMKRKWYKTYMKYKKRYYRKMMSKKTTKGTTTAWRRYCGYKARWKMYG
49              AKRGWCAGAATSWATAGWYKWYMKYKKRYTRKMMSKGGYGTAAGGWRRYTSYKRRWTCTK
                 **    .   ** .  *** ******** ******     :: .**** .** **.

47              AGACYKSKMRTRKKMMKYYKKYTGTARCKRRRYRRRWRMGKYCWYWKGRCRCRSKYRRYY
49              MRRYYKSKMRKRKKMMKYYKKYCRYCATKARRYRRRWRASKYYWYWKRGYRYRSKYRRYY
                    ******.***********   .  * ******** .** ****   * ********
```

```
47          MMYKRKRKGRCRYRKKYYGSMKRKKRYAKKKWGYAATGGCARYKRKRTCTRYWWYYRRRS
49          MMYTRGRTKGSRYRKKYYKSMKGKKGTRGKKWRYGWWRASWRYKRKRKSKRYWWYYRRRS
            ***.* *.  .******* *** **    *** *.   .. ******...**********

47          YRKRYYRKRYGYYCRKKKCRTKCKRMYRWTYKYAYRMMKSARCGYYYGKWMWMRRRRKYK
49          YRKGTCGKRYATYYRKKKGRYGMKRMYRWAYKYGYRMMKSRAMRYYYRTAMWMRRRRKYG
            ***    ***. * **** *   ******:***.******    *** . *********

47          WWKYMYTKKTWRKRYRCRYRMKKKRCKYGYYKKAATRYARWRYSTCRTWRMYYWYYRARW
49          WWKYMYGKKYWRKRYRAAYRMKKKGYKYKYYKKMRKRYWRWRYSYYGYWRMTYWYYRGGT
            ****** ** ******. ******  ** ****  .** *****    *** *****.

47          YYYRRYYYKGKKKWMRRKKKTCYKAYYKMKMRRWKYTRWRMYMYYYWKYSCKMWSCRACR
49          YYYRRYYYKKKKKWMRRKKKWYYGWYYKMKMRRWKYWRWRMYMYYYWTYSYKMWSYRRYR
            ********* **********  *  *********** **********.** **** *  *

47          YYKKKMKRYKYRWKMARYRKMSYSYWYKWKRYKKRYKCSYYMATCSRMRKYRYKRRMRKW
49          YYKKKMKRYTYRWKMTRYRKMSYSYWYKWTGTKKRYKASYYMRAGSRMRKYRYKRRMRKW
            *********.*****:*************.  *****.**** : ***************

47          RMRYKYRYRYWYKKWKTKKRRYWRWYRRCYTTCTYRKYKYYYKTYSKYKKWYMYYKRWRA
49          RMRYKYRYRYWYKKWKWKKRRYWRWYRRTYKGGCYRGYKYYYKYYSKYKKWYMYYKRWRW
            **************** *********** *.   ** ****** ***************

47          TYKMKKYYKYRTKYKYYTKYYWYMKTGCYYKYRRWRTGTYKRRTKMKCGGCTTGKAWSWA
49          YCTCKKYYKYAWKYKYTAKYYWYMTWRMYYKYRRWRCTYYKRRCTMKYRKMWCCTCWSWM
              . ******  **** :******.   ********   **** .**       ..***

47          MMMYMRKKKWWRRKWSSKRYRMTYMRARRMKKKRRMKTTKCKMYRYKKRMYMGCYWYSRR
49          CMMYMRKKGTTAAKWSSKRYGMGTCRRRAMKKKRAMKYGKAKMYRYKKRMYMRMYWYSRR
             *******     ******* *   * * ***** **  *.***********  ******

47          WRKKRKKAYKMCYTCGKWYWRYRSMWKKKKRRRMYRYKRKRYKKSYRRKKGRAYGKYCKK
49          WRKKRKKTYKMYYATAKWCWRYRCMWKKKKRRRMYRYKRKRYKKSYRRKKAAGYRKCYKK
            *******:*** *: .** ****.**************************. .* *  **

47          KASRWMRYRKRYWYRMYACTRWMYKKWKYRAMGMWRRRMKKKKWSRKTYYCCAYRRKKCW
49          KWSRWMRYRKRYWYRMYRYWRWAYKKWKCGMMACWRRRMKKKKWSRKYYYSYRTGAKKYA
            * ***************   ** *****   *. ************* **.      **

47          MKKYGKWRKKKTAATRYGYKARMMYYWYRYRSTYYAWRYWTGKGKAGTKRRRMRKCKWRY
49          CKKYKKWRKKKYRWYRYRYGRRMMYCAYRYRSACCCWRYWYTKRKRRAKAGRMRKYKWRT
             *** ******    ** *  ****  *****:  .****  * *  :*  **** ***

47          SSRCRTRKCGTYCCACGKYKYKRCRRYYRRKSSKGCKWRAKRRYKWMKRMYGKTWAGYRG
49          GGRSRWRKMRYYATTTRKYKYKRTAAYYRRKSSKKATWRGKRRYKWCKGCYKKCACTYRK
            ..*.* **   *. :  ******   ******** ..**.****** *  * *  . **

47          YKYYGAYYRCYCKKRKRRRYYKRRWWYRKMWRYRWGCGAKRAYRKRYCAKKAKKCRKWMR
49          YKYYRMYTRTYTKKGKAGRYYKRRWWYRKMWRTGWRTCCKRGCGTRYSWKKWKKTRKTCG
            ****  * * * ** *  **************  *   .**.  .**. ** ** **

47          RGKYYRKCYRRYSMYGKYWGKTRMKYRYKMYKRRRYKYYCAAGKRWSGAACGKMMRKAYY
```

```
49      GKKYYRKYYRRYSMYSKYWKKKRMKCGTKMYKRRRYKYYYRMRKRWSTTRTTKMMRKWYT
          ***** *******.*** *.***   ***********    **** :   ***** *

47      KMKYRYYYKKWRYRYSRYTTWMRKRRWKKKMYMKKKYRRKKKKKKRKTARMRKYWYWKAW
49      KMKYRYCTKKWRYRYSRYAAWMRKRRWKKKMYMKKKYRRKKKKKKRKYRRMRKYWYWKGW
        ******  **********::***************************  *********.*

47      KRRRRKWKKAKYRYRKKRMAAWKKKRRRRCWTGGMWAYWRYYMKYYTRKMMRCRRRYTTA
49      KRRRRKWKKGKYACRKKRMGTWKKKRRRRATATTCTWYWRYYMKYYYRKACRYRGATCGG
        *********.**  *****.:********. :     ********* **  * *     .

47      RSYAYGMRYWYMGGGGATATTCTYKRMRRYCCKKKWYYGGRMRKRWMKTRYWRYYYRYWY
49      ACYCTKCRYAYMKTAAMWWYYAYCKRMRRYMYGTKWYYAKRMGKRACTYRTWRYYYRYWY
         .*.   ** **  ..     .  ******   .****. ** **  . * *********

47      RYYKYTMRARKTGGTGTGCRYYYAYKYKSKATTCWMTMYMRYKMAAKKRRKYWMYKRGGG
49      RYCKYYMRGAGCKRGAARTRYYCTTKTKSKRWWYWCYMYCGYKMTGKKRRKYWAYKRASA
        ** ** **.      .:  *** : * ***    *  **  ***:.******* ***...

47      RCCKAATTMKKAYTYAAYKKRYRRKYYKWMYRWYKKRYGTGCRRYRSMKKAGYYYRKYKR
49      GYAKMRYYMKKMYCTGCYKKRYRATTYKWMYRWYKKRYRCCMGGYRSMKKTRCYYRKYKR
          .*    *** *  ..****** . ************      ******:  *******

47      RKKRCGGYWRRKYYRRYKYKMGYCGYYYRYKCWAGCYWMSRKYKKKWMMKMWKRAYTGAY
49      RKKRGCAYWRRKYYRRYKYGMATYAYYYRYGYACKMTTMSRKYKKKWMMKMWKRTYKSWY
        ****  .************ *.  .*****   .    ****************:*.. *

47      GGYWYGKRYYYWKYWMYRCARTCGWKWMRKKMSYRYKKAGAKMRKKRYKWYKKRYKTYKK
49      AKYWYKKRYYYWGTWMYRYGGAGAWKAMRKKMSYRYKKTACKMRKKRYKWYKKRYKAYKK
        . *** ******  **** . : .** ***********:..***************:***

47      YKRRRRKMYKKRWYKRWKTRYRKTYMYTYKARWYKKMRKRMWMMKTGGGKYYKYTTGCYC
49      YKRRRRKCYKKRWYKRWKKRYRTKYMYYYKRRWYKKMRKRMWAATAAAAKTTKYAGRSYT
        ******* **********.***..*** ** ***********  .:...*  **:  .*

47      YYGARKWCRGTAYKRSWGYYKATRRRKWKGWRWMMRGAWYWTYAYAYRMWACMCWGGTGK
49      TYRWAKWYRKKMYKRSWKYYKRYRRRKWKRARWMMRRWWYAWYMYMYRMWRMMGWSRYRK
         *   ** * . ***** ***  ******  *****  **  * * ****  * *.   *

47      RRRWCACAYSMRWKMRKYKWKAKKWTTSYWYKKYTKWRKKAAAYYGYRMRKKRKKKKRKR
49      RRGTTCTRYCMRWKMRGTKWKRKKTCCSYWYKKYYKWRKKGMGYYKYRMRKKRKKKKRKA
        **   .  *.******  *** **   ******* *****. .** *************

47      WMMWTMAYCGCGRTYYKRKYKWKCTCTKWTYKYARRMGGSRYKRKKRSCYGGWYKRCTWA
49      TMMAKMMTAAAARYCYKGKYKWKTGTKKWAYKTMRRCKKSRYKRTKRSTYARWYKRSYWR
         ** .*  ....*  ** *****   .**:**  **   *****.*** *. ****. *

47      TTGAWYRKRYRMAGYGSWKMKWKRRWKYYYKKYRKYKRMRRWWKRMRYYYRSRYYYKYRR
49      YWKRTYRKRYRMMKTKSWKMKWKRRWKYCTGGTRKYKRARRWWKRAACYYRSRYTYTYRR
             *******    ************     ***** ******   ****** *.***

47      YKRWWKWWWKYRRSKYATGYRYRRKKYKRKWYKRRTKTCKKGKKRRSRRMAKKRYKYKYK
49      YGGTTKWWWKYRRSKYMYKYRYRRKGYKAGWYKRRWKGYKKKKKRRSRRMRKKRCKYKYK
```

```
     *    ***********   ****** **  ***** *  ** ******** *** *****

47   KKKMRRWRKGGAGYKRWKKKYTCKYMWYKTAKKGTCCYGCRKKKKTKMKMKKRYKRMWWR
49   KKKMRRWRKRRRRCGRWKKKYYMKYMWYKKWKKAYYYTRYRKKKKYKMGMKKRYKRMWWR
     *********      ******  ******. **.      ***** ** ***********

47   RYMGCWCYKTGTYCYWRYYKWRSRTTKTKMKYTACRSKYMWMWWYYRYYYRWRKAKRKRK
49   RYMKTTAYTYAACACWRYYKWRSRWWKYKMKYAGTRSKYCWMWWYYRYYYRWRKMKGKRK
     ***   .*. .: . *********  * ****:. **** ************** * ***

47   KRGKYARKRRSKRYKKKCKRKWWKYMYRSCMKKKGMRTKRMKRSRCKACGTTCTMKGWAY
49   TATKYWAKRRSKRYKKKYGATWWKTMYRSTMKKKKMRKKAMKRSRSKGTAGWYCMKKWMY
     .  **  **********   .*** **** **** **.* *****.*. .    ** * *

47   KKYGWTTTRWMMKYRRMRKMRACKAWCSRWKCATKRYYKKKCTGGTKSRKGMRKYMYRYM
49   TKYRAGKCRWMMKYRRMRKMRGAKMATSRWKTTCKRYTKKKACCKGKSRKKAGGTMYRYA
     .**   . *************..*   **** : *** ***.    ****     ****

47   RKYRKKRTWGWRKGTASYAYMKYKKKKKKKKKWYCYCWYKRKKKYYCKKYGTYGYCYWSA
49   RKYRKKRWWKARKKWWSYRCMKYKKKKKKKKKWYMYYWYKRKKKYYYKKYRACKCYYWSW
     ******* *  **   **  ************** * ********* *** :    ***

47   KYYKKYRRYYYRRARRKKKKRKTYYWYCTRKGKRRKSKMKYYKYRYYRRRMWKRWRRKTM
49   KYYKKYGRYYYRGGRRKTKKRKYYYWYTAAKTKRRKSKMKYYTYRYYRRRMWKRWRRKYM
     ****** ***** .***.**** **** : * **********.*************** *

47   SYKGKWRYKYSKKKYGCAKKKKRKRKKKYKGKKKYTKWYWWSAAGAMYTCAAGKYKKYKY
49   SYKKKWRYKTCKKKTRYWKKKKRKRKKKYKKKGKYGKWYWWSRTKWMYAYGGRKYKKYKY
     *** ***** .***    ************ * ** ****** :  **: .. *******

47   KTYMMYRKYTKWTWRRRWAAKYYGAGKKMRMRKKMWRWWRMYSRRYSKTTCWTCKYRMYS
49   KKYMMYRKYKKWKTARRWWGKYYRWRKKAAAGKTCTGWWRMYSRRYSKKCYWYTKYRMYS
     *.*******.**.  *** .***   **    *.   ***********.  *  ******

47   WGWKRSYWSRKKRTKMRAAYCYYYYWKTYRKRYGKYKYRSKRYKRKKYRMRARYKYYRRR
49   WTWKRSYWSRKKRKKMRGTYMYYYYWKYYRKAYKKYKYRSKRYKRKKCGMRMACKTTRRR
     * ***********.***.:* ****** *** * *************  **   *  ***

47   MMYSWMMYWMWKKMWCWYCKKRYYYTRYRRKMKMYKYWYRKWWYKYRYKRGCGGYMYYAA
49   MMYSWMATWMWKKMWSWTTKTRYYYYRYRRKMKMYKYWYRKWWYKYRYKRRARAYACTGG
     ******  *******.*  *.**** ************************ . .*   ..

47   MRYYSYCYYCAACWAMYRTACRKYRYWTKYYYYRKMARYRYRWTGKMRGACAKMRWYYRK
49   MRYYSTATCYMWYWCMYRCTGRKYRYWYKYYYYRKMCAYRYRWAKKMAAGTRKCRWYYRK
     ***** .      *.*** : ****** ********. *****: ** ..  * ******

47   MYTMRRRRTAYYYKSYMYRSRTRKARKKYKYCGYRSYWYKWACGKRRKKKYGMRWWWRGR
49   MYYMRRRRCGYYYKSYACRSRKRKMAKKYKYTCCRSYWYKWWYTTRRKKKYKMRWTAGSG
     ** ***** .******  ***.**  *****   *******   .****** ***   .

47   GYYRRGKYYAKAMRYGKYKKKMYKKKKKTYYKYKYAKRMYRMYMAKKMRYMKKKRCYGGM
49   SYYGAKGCYTGGMRCAKYKKKMCKKKKKACYGCKYRKRMYGCTCGKKMRYCKKKRYYATM
     .**     *: .** .****** *****: *  ** ****    .***** **** *. *
```

```
47          RMYRSGYMRYYKRMKYKYTKWYKRKGGTSWWYKKAGKRYWTMRYYRYYYSKKYMGMCKKY
49          RMYRSRYMRYYKRMKYKYGKWYKRKARKGWATKKMCGRYAYMRYYRYYYSGKYMRCYTKC
            ***** ************ ******. ..*  **   **  ********* ***   .*

47          YWGACGCYRRRSYRRYYKRKKRSKYMKKRKKMMWCKWMRKYKYKRKYWKRRMMMKMRGAG
49          TTRWMKMYRRRSYRRYYGRKKRSKYMKKRKGAAWMKWMRKYKCKRKYTKRRMMMKMRRMR
                   ********** ************   * ******* **** *********

47          RKWRSRSSKKKWKCMMMRYKGKTKKRRKKYYWRYYKYYKKYRRKRCGCKRCTCTGSKWYK
49          AKWRSRSSKTKWKTMAMRYKRKKKKRRKKYYWRYYKYYKKYRRKRSKYKRYCYYSSKWYG
             ********.*** * **** *.**********************.  **    .****

47          KYRRRWCMYRRKRKKMMYYKRYMMM
49          GYRRRWSMTRRKRKKMMYYKRYMMC
             *****.* ***************
```

The pairwise alignments of two accessions 47 and 50

```
47                RTWRKRYRRWYRYWKCTYMWCCCTAYKWMKYRKKKKYRYGGGWMGAMYMRRRWMMRRKRM
50                RCWRKAYRRWYRYWKYKCMWMYYCWYGTMKYRKKKKYRYSARWMTWMYMRAAWMMRRTAC
                  * *** ********* . **     *  ***********.. **  ****  *****.

47                RWRRYMYMWMRYWWKYYMKYYRYKKRRRRSRWMYKKRMTYWRRMWRYTGRWYYYYRYKYR
50                RWGGYMYMWMRYWWKYYMKYYRYKKRGRRGRTAYTKRCWYWARMWRYGTRWYYYYRYKYR
                  **  ********************** **.*  *.**  ** *****  ***********

47                RWRRYWKYKWRYAGMKRWRWYYRYYYRRRMWRKRTYAARSRKWMTWYWWYYYYKMYMRCK
50                RWRGTAKYKWRYCRMKRWRWYYRYYYRRRMWRKRCCMRRSRKAMGAYWWYYYYGMYMRGK
                  ***   ******. ********************    **** *  ******* **** *

47                RWSYAKYYWRYYMYKKYYMRYWYWKMWWKKYWRYYYMKGAKGKAYKMWMYWRRMYRRKMK
50                RWGYRGYYWRYTATKKYYMRYWYWKMWWKKYWRYYYMTRMKTKRTKMWMYWGGAYRRKMK
                  **.*  *****   ***********************.  * *  ******   ******

47                RRWAWYRWYGRKCMRRYRASRKWRWYSRRWKWWRGWMKRWWRKYCCTYYYRKYYKYYYYY
50                RRWGAYRWYKAKAMGRYGMSRGWAWYSRRWKWWRKWMKRWWRKYAYWYYYRKYYKYYYYY
                  ***. ****  *.* **  ** * ********** *********.  *************

47                RRRYGCKSWRTRYWKMGYRYYRRARYTYYYRRWRYYYYRYGKSWYKWMKRWGARYRTTRY
50                RRRYRYTSWRKAYWKMAYRYYRRCRYYCCTRRWRYYYYRYRKSACGAMKRWKMRYRYWRY
                  ****  .***. ****.******.**    ********** **    ****  ***  **

47                RARYYKRWCAAKRWMKMTRYWYTYRGWYTYWKRRAMYCMRRRYYSGRYKRRWSMAAMKRW
50                RCAYYKRWARRKRWMKMGATTCYYRKATGYWKRGGMYYMRRRYYSRRYKRRWSMGGAKRW
                  *. *****.  ******      **    **** .** ******* ********.. ***

47                KWRMSRKRTRRRMKKYWYWKWYKWKATYKATASKKKKWGKRYRKWTACWWRRKRKACKAM
50                KWRMSRKRCRRRAKGYWTTTWYGATGACKGGMSKKKKWKTRYRKWWRYAWAAGAKCAGGM
                  ******** *** * **  .**  ..: *.  ****** .*****    *    *.. .*

47                YKCGGATRRRYKMRKKWRKMKYKKYWCCCRRRYYKYYKYYYMKYWWRYYYYMMYRKGRKR
50                YTMATCCRRRYKMRKKWRKMKYKKYWTAGGRRYYKYYKYYYMKYWWRYYYYMMYAGKGKR
                  *. . . ******************* .  ************************    **

47                KTCCKYCRRRWKRWKYYSRKKYAGCKKAGKKGYKKRMKKMMKRWYYWWKTGTAYYAWMYR
50                KAMMKYSRRRWKRWKYYSRKKYWRAKKGAKKAYTKRMTKMMKRWYYWWTCRKCYYMWMYR
                  *:  **.***************  .**..**.*.***.**********.  ..** ****

47                SRKKCRKKYRYKAGACRKGMRKGRYKRRKSYYKKKYAYRKWWKMRYWKRKKMCTMGRWRY
50                SRKTAAKKYRYKMRWYRKKMRKSRYKRRKSYTKKKTRCRKWTGMGYWKRKKMMYMTRWRT
                  ***.. ******    ** ***.******** ***   ***  * *******  * ***

47                SSWCGMYWYYKRCGYRKMKYKYTAATTKYACSMKMYYRRMRRMRMSKYKRGTATRTGWSK
50                SSWYRMCWYYKGYRYRKCKYKYYCGKCKTGTSMKMYYRGCAGARMGKTKRAGTARWAACT
                  ***  * ****   *** **** ... * . *******     **.* **. ::* . ..
```

```
47              YKTTAKWMRYYWRRAGMWYYGMMMMKYRMCRYWWYSKWKMYYRWRKKMMRRKGKCTCRRK
50              YKCGGKWMRYYWRRCCMTTYCMMMMKYRMYRYWWYSKTKMYYRWRKKMMRRKKKSCARRK
                **  .*********. *  * ******** ******* ************** *. .***

47              RKWMWCCYWYWWGAAKKMYRMWWYKKWYTTRRSKRKKRRYSWWYRWWRATGYYRSMKMKK
50              RKTATTMCWYWTSRRKKAYRMWACTKATCCRRSKRKKRRYSWWYRWTRRCCCYRSMKMKK
                **      *** .  ** ****  .*    **************** *    ********

47              YYRKRYYRKATTMRRKKYKKAGTAYYGCRYWRYRYWWYWRASMRKKWTRAKKKKSYWKYM
50              YYRKRYYRKMWKMRRKTYKKCRATYYTYRYWRYRYTACARCCMRGKWCRRKKKKSYWKYM
                *********  .****.***. ::**  *******    *..** ** * **********

47              KWYYRKYYTKKYYYKYWKRRKKSRRKKKYAKKKRWTCAGAYAKYKMCTCKWYRRKRKGKR
50              KWYYRKYYGKKYYYKYTKRGKKSRRKKKYGKKKRWWTCKRYGKCKMTKTGWYRRKRKKKR
                ******** ******* ** *********.*****   . *.* ** .  ******* **

47              YRKKKRKKMRSYGKWYKKRGCTWWTWKMYGTWKKCATMTAWMKKWMMWSKCGCKCRYKYK
50              YRKKTRKKMRSYRKWYKKRKSYWWKWKMYAAWKKATCMGRWMKKWMMWSTMSYKYRYTYK
                ****.******* ****** . **.****.:***.: *  *********. . * **.**

47              RRKYRYYRMYKRYYYYYRTAYMKMWKRRKMRKKATATRKKRMRRWSYWRWKRYKAGATTC
50              RRKYRYYRMYKRYYYYYRYWCMKCWKRRKMRKKWYGCRKKRMRRWSYWRWKRYKRRCCWA
                ******************   ** *********  . *****************   . .

47              MWYKMCRKRRRKRYMYKKKRRKARWGMYMRWKKCACYKKYATAMYYGAKRRYRYYWRKYC
50              MWYKMMGKRRRKRYAYKKKRRKMATKMYCGTKKYMYYKKYRKRMYYAGKRRYRYYWRKYT
                *****  ******* *******    **   **   **** . ***..***********

47              GTMRYKYYRSKYKKRKKGMMTWCYYYKMKKRKGKRKRKWYTGSRKAKKWWYKKKYGRRKY
50              KYMRYKYYRSKYKKRKGSAAYTTCYYKMKKRKKKRKRKWYWTSRTTKKWWYKGKYRRRTY
                  ************** .      ******** *******  **.:****** ** **.*

47              KYYAACKRATCKRYSKWYRRMYKRWKMRGRKARMKKRKMMRKMGGKRRYRRMKKYMTYYT
50              KYYWMMTRGYYKRYGTTYRRMYKRWKMRKRKRRMKKRKMMRKMRRKRRYRRMKKYAAYYK
                ***   .*.  ***.. *********** ** ***********  ********** :**.

47              KTTCGYRKYKYYYCACSKYCCYYYWTYYKKYYKYKKYYWYWKKRYKRRAKKMKKKTSWMM
50              KGATCYRGTTTCYYWYSKYTGYYYWCTYKKYYKYKKYYWYWKKAYKRRMKKMKKTCSWMM
                * :  **  .  *   ***  ****  **************** **** *****. ****

47              YRKKYRRRKWSKGTARKAMRYCMAMKKRWKMKCKKYRRAYKRWMMYWRYYYMKRAKKYRR
50              YRKKYRRRKWSKRWCGKWCRYYMGMKKRWGMTMKKYGAMYKRWMMYWRYYYMKRWKKYRR
                ************   .*  ** *.***** *. ***   *************** *****

47              WWRYMKWKKMRCAGTKWKKGGWCWRMWKMCMKYYRWMYRRTGRYGKRGTAKCRMRGWYWM
50              WWRYMKWKKMRTGRYKATKRRAYWRMWKCGMKYYRWMYRRAARYKKRAAGGTRMRRWCTM
                *********** .  * .*    *****  **********:.** **.:.  *** *  *

47              RRRYRRMMWGYMWYGKATCAMGRWRKMKWWSMYKYKKYTCTRRRWWYRKWYYARRWWWSW
50              RRRYRRMMTRYMWYRGCCTCMKAAAKMKWWSMYKYKKYWTCGRRWWYRKTYYCRRWWWSW
                ********  ****  .  .*    *************    ******* **.*******

47              WKKAYYRKRRSTCCYRYTGKMMKRKWRYWYRRYYKWRKKKSRRMAAARCKKMKKSACGGR
```

```
50      WKGGTYRTRRSAMTYRYWKKCMGGTWRYWCGACYKWGKKKSRRMGMRATKGMKKSWYRRR
        ** . **.***:  ***  * *  .****    *** *******.    * ****    *

47      WKSKRRWYRRRRKKKKMGRMKRGKRRRKMCMKWWKKRRRMKRRYYMRTTMTTGACCTRWK
50      WGGKRRWYRAAAKKKKMTGMKRKKRRRKMYMKWWKKRAAMKRRTYMRCGMGCCGAYAAWK
        * .******   *****  *** ****** *******  **** ***  *   .. : **

47      MKYCYKKKTMRRRYKCCARKRKYRKRRGGKWMYRKMMYRWSGWAGYSKYTAYMKYKKKKA
50      MKYTYKKKKMRRRYKATGRKRKYRKRAACKWMYRKMCTAASAWRRYSKYGGYMKCKKKGM
        *** ****.******. .******** . *******    *.*  **** .*** ***

47      KGKYTKGKWYWRYRWRYYKKCYYKYWASYYKKRRCMWKKKGKKMWYRMMYTMGRYMKRKR
50      KKKCATTKWYWRYRWRYYKKTYYKYWMSYYKKRATMTKGKKKKCTTGAMYYMRGYAKAKR
        * * :. ************* ***** ******  * * * **     ** *  * * **

47      YRMYRSKGYRYTYYCMYWCACGKRMMYYKATAKRKGAKCKRYTYMRYYRRYYSKKWYYKW
50      YRMYRSKRYRYKYTMMYWAGTAKRMCYYKRYMKAKARKTKRYGYMRYYRAYYSKKWYYKA
        ******* ***.*  ***.. .*** ***   * *. * *** ****** *********

47      YMYYGKKTTYCGTYRRMWKYYYCGYKTKKKMKTRRKKKAYYKMYYKMKKKMRWKYMMYRS
50      YMCCKKKWYYYRCYRRAAKYYYAAYKYKKKCKCRRTKKMYYKMYYKMKKKMRWKYMMYGS
        **   **  *   ***  ****..** *** * **.** ******************* *

47      YWRAKAMYYYKKRYSMTYGYTWRYYWYYAKKMYTGYRYWKMCYRKMKYSYWYWKYKKKSW
50      YWRRTCMYYYKKRYSMATRYGWRYYWYYCKKMYYSYRYWKATYRKMKYSYWYWKYKKGSW
        *** ..**********:  * *******.**** .*****  *************** **

47      KRYMSMRGYMRMRKKWRYKKRAYWYRMAYYTCKMRRMTWTCARYRYKRRWYYRRRAKKWW
50      KRYMSMRKYMRAGKKWRTKKRGYWYGCTCCCTKMRRCYTCYGGYRYKRRWYYRRRGKKWW
        ******* ***  **** ***.***  :    ****     . ************.****

47      RKRKKSYKRTTCCTRRMYRWYKYYRMKSAAKYYYMWMKWYYRMYMYWKKKRRYRMGYRKY
50      RKRKKSYKGCWYYKRGACAWYKYYRMKSRMKYYYMWMKWYYRMYMYTKTKRRYRMRCRKY
        ********     .*    *********  ****************  *.******  ***

47      ATTGYYYWMRRRAAAYRYKWKAMRSYAYAAKGAMMSRYWYTACCWMKSAGGATATMGCRY
50      RYWKYYYWMRRRWWWYGYKAKRMRSYRYWMKAGMMGGCWCYMTTWAKSCATTCGWMTARY
            ********   * ** * **** *  *..**.  *     * **.. : . * .**

47      KCCWMWRGYTATTYKACTYWYGYYMYTCTTMYMTTKTYKAGGCYYRMYSKRWYYKSAGAG
50      KSYWAWRAYATAGYKMTCTATAYCCYWMWAMCMKYGWCTWRKYYYRMYSKRWYYKSTCGA
        *. * **.*::: **      .*  *   :* *.    .    *************: ..

47      GTYYYRRKKRKKTKRRRTWMSKMKYYMWWTRRCWTKKKSYKCKTMKRWRKTTCGAATWKY
50      KWTTCRRKKGKGWKGGGCAMSKMKYYMWACAAYTGKGKSYKYKYMKRWRKGCAACGCWKY
             **** *  *     *********       * **** * ******  .... ***

47      RSKKKMWKRKKYKRYCGKKWYRMRYRWWSMTCACKKYKKYRYAKKGAKWRGGKYKWWYYM
50      RSKGKMWKRKKYKGYMKKGWYRMRYRWWSAWYTYKKYKGYACWKKTCTARKRKYKWWYYM
        *** ********* *  * **********   : **** *   ** .. *  ********

47      RYSYYYMRMRKKMWWTWYSYRKKYWKKKKRKACTARKMYYYYARTGTKRYAGMRKRKKRY
50      RYGYTYMRMATGCTWCWTSYRKKYWKKKKRKCMGGRKAYYYYGGYACKAYWRMRGRKKRY
```

```
                **.* **** .   * * *************.  .** ****.  . * *  ** *****

47              KRKYRKYRTACAYMMSMWMCKRWKRYRMYSKRRKRYRYKKKWRRKRMKMYACAYWKKWYY
50              KRKYRKYRYRMRYMMSMWMMKRWGRYRMCCKRRKRYRYGKKWRRKRMKMYRYRYWKKWYY
                ********    ******* *** **** .******** ***********   *******

47              RYYKMRYCWYYWYRMKTYYATATMWKYMRWGYSGGGYKRCKGAGMRKKRSRYYRTAMKKK
50              RYCKMRYMACYWYRMKYYYMYRYCTGYMRWKCSTRRTKAMKACAMRTKASRYCRWMMKKK
                ** ****   ****** **       ****  *    *  *...**.* *** *  ****

47              YYRKYYWCYTYMYKWYWTRRMYKKKYYRRKYYCCAAGKKKTCYKMKKYRYKKKKWRYKRY
50              CYRKTCTYTWCMYKWYWKRRMCKKKYYRRTYYYYRMRKKKCYYKMKKYRYKKKKWRYKRY
                 ***       ******.*** *******.**     ***  ******************

47              RTCKWKRRWKRKMYMMGKCCGKRYRRKKKKYYWKCTWGMKKYRRKKYCYKRMYKKYKKKR
50              RYYKWKRRWKGKMYMMKGAMKKRYRRKKKKYYWKYAWRATKYRRKKCMYKRMYKKYKKKR
                *  ******* *****  .  ************* :*  .******  ************

47              RCRTWWWYWYRYRYYKRYKKMKCTYYRYCTTTRKWKTCARRYTGRKKTRYCSYKYKYKMR
50              RYRAAWWCAYRYRYYKRYKKMKAAYCGCTCCWRKWKCYGRRYYRRKKYRYTSYKYKYKMR
                * *: **  *************.:*       ****  .***  *** ** *********

47              YYTKRRKKKSCMWRRRKKKWAWAGKKRYKYKKTTGKKKWYRRRKKGWKRYYRWKMRYRKA
50              YYCTARKKKSYMWRRRKKKWGACTGKRYTYKKWCKKKGWYRRRKKKWKRYYRWKMRYRKW
                ** . ***** *********. .  ***.***   ** ******* *************

47              KKMWYRKRYKYKKWRGTAMYKKWKRYCWKKKKWKYKYRKYWKRRCAMYKTARWKKWYKYA
50              KKMWTRKRYKCKKWRKYMMYKKWKRYAWKKKKWKYKYRKYWKGRYRMYKWRAAKKWYKYG
                **** ***** ****   ********.*************** *  ***    ******.

47              GTKWWRACMKKYKTGAAKASRRWMKCAAGKKYWRKRYYRYYSM
50              AYKWWRGTMKKYKKRRTKMSRAACKTGTRKTCWRKRYYRYYSM
                . ****. *****.  :* **   * .: *. ***********
```

The pairwise alignments of two accessions 47 and 51

```
47              YYMYKRYSYRMYYRRYRRGGGCYMYYCTRCCRSASRASTRKWRWGTYRRCAKYWMTYRKY
51              YYMYKRYSYRMYYRRYGAKKATYMYYYAGTYGGWSGMGAAGTAWACYRGYWKYWMYYRKY
                ****************    . **** :    . *  .:    *. **   **** ****

47              SRYSWTYKKWRYRSMKSAYYWYWWMKCAAKRYRWKWCWRRKYRRCWAGWTYMYYWKRAAS
51              SRCCWWTKKWGYRSMKSGYCACAWMKTGCKRYRWKAGWRRKYRRYWMRAWTATYWKGCTG
                ** .*  *** ******.*    *** ..******  ******* *       *** .:.

47              GYGRWWKAYTWRTTYTGGYWWMKRGCCRKMRSSYCWMRWMMAYRRYMTRWMKYMKRWWYR
51              KTRATWKTTWWRWAYKSAYATMTAATTRKMRSSYTAMRWMMTCGGYMKRWMKYMKRWWYR
                     **:  ** :*...*  *. .  *******  *****:   **.************

47              RMKYRKMCCWRGGRWYYRKYYWYYYYRGMRCYTRWRRYWYYRRRYYKWYWRGRCCARKWT
51              RMGCRKMGYARKKRWYYRKTCWTTCTGAMAYYWGWRRYWYYRRRTTGWYWRKATYRGTWK
                **  ***   *  ******  *     .*  *  **********   ****      .*.

47              ATCYRMRRMYTRRKWKMCACMRWTYCWGYRKKWRAMMKYYYMKRYRRYKKKWRGMRYRWY
51              GCTTRMGRMYWRRKWKMYMYMRWYCMWRYRKKWRTMMKYYYMKRYRRYKKKWRRMRYRWY
                .   ** *** ******   ***   * ******:****************** ******

47              CMRMYGARYWRYRYMYRGRRYMRGGRWYWSRYRRWWWSRKCCRKYYWGAYRARYYYKYTA
51              YMRMYRCRYWRYRCMYRTRRYMRKARTYWSATAGWWWSRKYMGKYYWRMTRRAYYYKYGT
                 **** .****** *** ***** .* ***    ******   ****   *  ***** :

47              YRYWTGRMRYYWKKKCTYMRYYYCCKRWYCYWRRYYRAWSYTYRMKYYGTRMRSYWYKYY
51              YGTAWRRMRYYWGKKMCYMRYYYMTKRWYYYWAAYCRWWSYKCGMKYYRYRMRSYAYKYT
                *     ****** **  ******  **** **  * * ***.  ****  ***** ***

47              ATATGKARSYCGYCMRYKKRWYWCYKARYKRMKRRYWYTGMKYWKRKRTTYTKMWKRARG
51              MYRYTTGACYTKYYMRYKKRWYAMYKGGYKRMKRRYWYKKCKYWKRKRWWCKKMATGGAA
                     .. .*  * ********  **. **********.  *******   .** . . .

47              RYRAGGRTTTCAKGKMRKWRRKMKYYYGTTKRWMMKCCCRMKYKTARMRYWKKYCGCAAY
51              RYRGKRAACWTMKRKMRKWRRKMKYCYRAGKRWACKTAGGMKYKCGGCGYWKKTGATTGY
                ***.   :    * *********** * : ***  * .  ****  .   ****  . :.*

47              WWSRYRKSKYMMYTTATRYGTYRKRYKKRRRTGCRRKKKKKKRYRRRWWGYYKKKKYSMW
51              WWSRYRGCTTMMYCCGGACRWYAGGYKKRRGCAARRKKKKKKRYRRRWWRYYKKKKCSMW
                ****** .. ***  .     *   *****  ..*************** ****** ***

47              KKKTCTAGRKRRYRMYYKMWKGGYWKRYKKTKYKRKRRKKKWYRKRYWWWTARWMKKWKY
51              KKKGTYMRAKRRYRMYYKMWKATYWKRYKKCKYKRKRGKKKWYRKRYWWAWRAACKKWKY
                ***      ************. ******* ****** ***********     *****

47              ATRMGMKKATWRCKKMYYYYWCRTMMCTSRCAYRRKKYKKYWRKRYRYYYKYWMGACTRR
51              GYGCACKKWWWRMKKMYYYYWYRCMMTGSRMCYGAGKYKTCWRKRYRYYYKCAMAGTGRR
                .   . **  ** ******** * **  ** .*   ***. **********  *..  **
```

```
47      YKKKMRRWKCATGCRCMMWYMWAAAMKYGGYGRKWMYAGRTACCRYKRKMGYYYKYYGAM
51      YKKKMRRWKMRKSMRAMAAYAAGWWMKYRKYKRKWMYMARYRYYGYKRKMSYYYKYCRTM
        *********  .. *.*  *  .  ***  * ***** .*     *****.*****  :*

47      MYRMMKGCTYKGWMWCWYMRSRRSMGRWRKMRYYSRKYWSYKAKYGCYKWWKRMKKGRTM
51      MTRMMKRTGYKKWMWAWYMAGRGCMKAAAGMRTTGGKYWSCGWKTRYYKWWKRMKKCGWM
        * ****   ** ***.*** .* .*     **  . ****   *   *********   *

47      RATCAMTWWAAKTRRYRRYKKTGTYYRYYYTACYRYWRRGYGKKMKYRSKYGCGCMAYRY
51      RWKYRMCATMWKWGRYRRYKTCAWCYRYYCGCTYRYWRRRCKKKMKYRCGYRMAYMWYRY
        * .  *     *  ******. .  ****  . ******   ******. *  . * ***

47      KGGTTTWYKKMGRKGYRRTKRRWKWCCGTAYWYYRKWWKMRSWGAATTRYTAMRRYYYKK
51      GAAGCCWYGKMRGKKCARCKRRWKWMTAAGYWYYRKWWKMRSWRCRWAAYYRCGRYCYKK
         ..   ** **  *   * ******  .:.************* .  : *    ** ***

47      KAWRYRRRKRRGRAYWYYAKKRRMKKSYRKKARRMYRWYKYKYRRYRKKTTYYYWAGMWM
51      KGWRYRRAKRRRGCYWCYGKGRRMKKSYRKTGRRMYRWYKYKYARYRKKYYYYYAGRCWM
        *.***** ***  .** *.* *********..*********** *****  *** .  **

47      YMYKTGCKKYWRKYRRYYRKKKYRWKSYYYYRGYYWGATARYCAYWRWMYYRARWRTMRY
51      YMYKCATKKYWRKYRRYYAKKKYRWKSTTYYGAYYWKGGCRYYRYWRWMYYRGGTRKMRY
        **** . *********** ********  ** .*** . .**  ********.  *.***

47      MKKKKRRKYYRKCTCKARYYRKKSRRRKGGCRRKKKKRWKMMTYSKRKKMMSWKWRYRKR
51      MKKKKRRKYYRKYYMKRAYYRKKSRGRKTATGRKTKKRWKMMYYGKRKKMMGWKARYGKR
        ************   *  ******* ** .  **.******* *.******.** ** **

47      RKMRSCWKYKAKWCWRYYRMYRWYWYRAKKYMYSMKKKYKKKKAKRMYACATGKRYKCYK
51      RTMRSYWKYKTKWMWGCYAMTGTTWYRRKTCMYSMKKKYKKKTRKRMYTGGCAKRYKMYK
        *.*** ****:** *  * *    *** *. ***********. ****: . .**** **

47      KYTTKKCKKSYKYKRMCRKKYYATYRKKKGCAYKKRYMRYYGRKWYKKWYYKYMTACATC
51      KYWYKKYKKSYKYKRMMRKKCYGGYAGKKRYWYKKRYMRYYKRKWYKKWYYKYMGTGCAT
        **  ** ********* *** *. *  **   ********* ************ : .:

47      TGCCMTCYWYKYRRRKYCRWTCTCTAAKYMRWMATMYYSKTRYWYRGGMRYYRRKYWTMY
51      YKMTMAYYWYKYRRRKYYRWWTAMARRKYMRWMMCMCCGKGATTYGCCMRYYRRKYWWMY
            *: ********** **  : :  ******  *  .*    *   ********* **

47      YMRRRYSYAKKKRRSMSYSKYSYYKRKRKRYKRMSAAAKWKYYYCAAATYKSMKYKKYWA
51      YMRRRYSYGKKKARSMSYSKYGYYKRKRGRYKRMSRCRKAKYYYYGGGACKGMGYGKYWT
        ********.*** ********.****** ****** . * **** ...: *.* * ***:

47      TGGMRGTCTAYGGATTGRYRWRTAKRKTATAAYMRARRMRRYKRRKKYRYMYRGGCKRRR
51      CACMRKYYWWYRRGGCTAYRWGCGGAKYRWRWYMRCRGMRRYKRRKKYRYMYRRAAKRRG
         . **     *  .    ***  .  *     ***.* *************** ..***

47      RWSMKYYRYRRRMWGKRRAKKSYKRRKRKYRKWRYWKKKRRKTRMYKKYWYYGKYYMKRW
51      RASMKYYRYRRRMWSKRRCKKSYGAAKRKTRKWRYWKKKRRGWRCCTKYWCYKKYYMTRW
        * ************.***.****   *** ***********  *  .*** * ****.**

47      WSTCKKKYKKKKKWYARSYKYASYYWKMGWMYRRYKRWYSKYYYYCWYWKYRYCYRRRKY
```

```
51          WSWYKKKYKKKKKWYRRGYKTGSYYWTCAWMYRRTKRWYSKYYYCTWYWKYRYTCGRRKY
            **  *********** *.** .****. .***** *********  *******   ****

47          CKKTRYSYRYRTKYCGYKYKGRCTARGKTMKYYKRTWRRRKKKGTCKKTYKRYYAAYYGW
51          YKKKRYSYRYRYKYTRYKCKKRTYWRRKYMKYYKGWWRRRKGKAWYKKCYKRYYRRYTKT
             **.******* **  ** * *   * * *****  ***** *.  ** *****  *

47          AKGKYKKRRMKRGKRKRYYTKWSYYRKWKKRYMYKSYKRCCSTRKCTRGRMKARWKWRRK
51          WKKKCKGRRMGAKKRKATTWKWSYYRKWKGRYMTGCTKRTTCGRKTCGKGMKWAWKWRRK
             * * * ***   ***    ********* ***  . **  . **     **  ******

47          WWTTRRRMYRKRYYMYYRWTSAMGRWGWKKYYRRKKGMRMKRWRYMKRKYRKKKWRMSWY
51          WAGGRGRMYRKRYYMYYRAYCRMAGWRAKKYYRRKKKMRMKRWRYATAGYRKGKWRMSWY
            *   * ************  . *. *  ******** ******** .  *** *******

47          SYYATKYGTAGGKKKKMWYYYMYMWRYRWKKYYKYSMYYRYSRKWCCTKRAWMYKARMAC
51          SYYGCKYRWWRRKKKKMWYYYMYAARYRWGKYYKCSMYYRYSRGWAGCKRRWMYKMRMWM
            ***. **     ***********  **** **** ******** *.  ** **** **

47          TKMWAKRTTMMAAKSSASRKRKTKWMYACRYSYYWTTKKRTTYKKYTKMSWRRCYYKCAW
51          YKCTCKRCYCCGGKSCTCRKRGAKWAYMARYSTYAGWKKAAAYKKYYKMSWRRTCYKYTW
             *  .**    ..**.:.*** :** * .*** *   ** ::**** ******  ** :*

47          GAYYTKSKRGKMWRTTTCAATCSGTRRYKKKMMCKRYYKCRRYRKCGRYAATKATYACKS
51          AGYYWKSKRKKMWACGGMRTAMSKCGRYKKKMMYTRYYKYRRYRKYRRYCGCGCATGTKS
            ..** **** ***      :: *   ******* .**** *****  **..  .: . **

47          MKKSRYKAGCYSKGMGMTRYCYKGKRWYKYTATTRRKMWKMRKRACTRRYCTTYRTMRAG
51          MKKSRYKTATYSKKARCYGCAYKAKRTYTYCGCARRKMWKCRKRGTGRRYTACYRCAGRR
            *******:. ***       .**.** *.* . :****** ***.  *** : **

47          TWYTYKKGCTTMMKYYRMKKYWTAKSYSWWYYCGKSARYRMRRRWYYYYCRGAKKRYSRK
51          YWYGTGKCYCCMMKYYRMKKYTCRKCYSWWYYMRGSWRYRMRRRWYYYYYRTTKTRYSRG
             **   *    **********   *.******   * ************ * :*.****

47          KKSRYKRKRMRKRRMMYSKMSKKMRRMACWYRKMTGRRKRYKRYYRRMKMYARKYKYKYR
51          KKCRYKRKRMRKRRMMYSKMGKKAARCGMWYRTMCKRRKRYTRYYRRMKMYRRKYKYKYR
            **.*****************.**  * . ***.*  *****.********* ********

47          YKYAAMAARYKKAKGAATMKKACMKGGTYKKWYMCKRRTGCYKYMRYRAYGGKYRMYCTG
51          YKYGGMRMRYTKRGSRRYMKKMYMGAAACKKWYMMKRRASMCKYMGCACYTTKYRMCGCK
            ***..*  **.* .   ***  * ..: ***** ***:.  ***   .*  ****

47          MCYYYWWYGKKKYSAGCARWRYRWYKRYRSMKGRKKTCGTAGMKYRKRRWKRYMKKMYYY
51          CYYYYWACATKKTCWRYTRTRTAWYKRYRSMKSRKKKTRCGRMKYRKRRWKRYMTKMYYY
              ****  ..** .   :* *  *********.***.   . ************.*****

47          YKRYKWWACCYWWKKYYRRKYKYRKYSKKWMYTTCAYYWRKKWRKKYYKYKYYYWMWYWK
51          YKRYKAATTYCTWGKYCARKYKYRKYSKKWMYKWYMYYWRKKARKKYYKYKYYYWMWYWK
            *****  :    * **  **************.   ****** *****************

47          KYRRKKYKRRKKMWRKWWSWMMYRMRSYRRRKWSSKRKYYYRRMRGRMKGKKRWKKYKKM
51          KYRRKKYKRRGTMWRKWWSWMMYRCGSTGGGKWSSKRKYYYRRMRRRMKRKKRWKKCKKM
```

```
        ********** .************  *    ************** *** ****** ***

47      YRKYYRWGMYKKKYWYATCTWRTGATAYWKGTRRRRRYWWYKKYKKRKYKGMMTWAGYKA
51      YRKYYRWAMYKKKYWYRCSWWRYRRCTYWGKYRRRGRYAWYKKYKKRKCGSAAYTRKYKW
        *******.********  . **    :**   *** ** *********  .      **

47      KTWKRMMWKMSKKWWKSRGAKKWMMKKATTAGMYKACGRYYRKYWYYYRRCGTCKRKYWY
51      KGTKRMMWKMSKKWWTSRATKKWCCGKWYYMRAYKRAARYYRKYWYYYRRSKYYKRKYWY
        *  ************.**.:***   *      ** ..************.   ******

47      RYSKWRKMKRRKRRKKMMYYGAKRMMKKWMMMKTMTGAYKWMYYKKRRKWKRWGGWTYYK
51      RYGTTRKMKRRKARKKMMYYCWKRMCKKWMMMKWCYRRTGWMYYKKGRKWKRARRAYYTT
        **.. ******* *******  *** *******       ****** *****     * .

47      WRKKWWKKKYRKATATRRSKYKRKRYRYKRKRKWKRSRRMKYKYMKSRWMYWRRRRMWKY
51      WRGKWWKKGYRTTCGYRRSKCGRKRYRYKRKRKWKRSRRMKYKYMKSRWMYWRRRRAAKY
        ** ***** **.: . ****  **********************************  **

47      YYTCMKKKKKCRRAKYYYKMMRWYYRRSYWRRMYYKMYYYKKYSSKYMRYYKKKRYKYRY
51      YYWYMKKKKKYRRGKYYYKMMRWYYRRSYWRRMYYKMYYYTKTGSKYMAYYKKKRYKYRT
        **  ****** **.**************************.* .**** **********

47      WKAKYWRYRKKYKSYWYKWATGTRRGKACTGKRYKTYKYMRGSYYMKYRMRRRMRKWRYK
51      WKGGCTRYRGGYKSYWYKATAACRRAKRYYKKRYKCCKCAGKSYYMKTRMRRRMRKWRYK
        **.   ***  ******* ::. **.*    ****  *    ***** ************

47      KRWYMYKWMMWSWKKKGKYYKKYKYRMKRYYYYYRWYKMWMWKMRKKRKKCMRKKKKKMW
51      KRWYMYKWMMWSWKKKRGYYKKYKYRMKRYYYYYRAYGAWAAKMRTGRKKMMRKKKGKAW
        ****************  ***************** *  *  ***. *** ***** * *

47      YKMYYTRRRKRRRKMTYKCRYRKRRWRYRMRWKGKMYYKYKKYRMTRGKAGMRKYRKYYW
51      YKMCCWRRGKRRRKMKYKYRYRKRRWGYRMRWGKKMYYKYKKYRCYGRKCTMRKYRKTCT
        ***   ** ******.** ******* *****  **********    *. ******

47      YKMTMGMMKRKYRRYRTWTARYYYCAAGKTGCWTCAAKKMYKKWKYRWKAKYRYTKGYWR
51      CKMKMKCMKRKYRRYGYAYWRYYYYRMRKKKSACATGKKMYKKWKYRWKGKYRYYKRYWR
         **.*  ********     ****    *. .  .:.************.**** * ***

47      YKRYRTYCTTTMRKRWKKKMYMCCKYRRKKKKTGCKRRMKKCWYWKKYRRKRMYCYWSRC
51      YKRYRACYAAGMRKRWKKKMYMYAKYRRKKKKYRYGRRMTKYWYTKKYRRKRMCAYTCGY
        *****:  :: *********** .********    ***.* ** ******** .* .

47      KKRMAKYATTCKYKKKTARR
51      KKRMGGCCCYYKYKKKCTRR
        ****.  .   ***** :**
```

The pairwise alignments of two accessions 48 and 49

```
48          RMMKCWRWWRYKYKYYRRYWWYWRWYKRRWYKYCKYYMRSYYYRWYGYRYRYYYYTRYYA
49          RMMGYWRAAACKYKYYRRYWWYWRWYKRRWYKCYKYYMRSYYYRWYRYRYRYYYYWRYYW
            ***  **    *********************  ************ ******** ***

48          YMRYCYGKYYRWYRMKRMCYYSKRYRRWRRKSYARAWWGCKYRRYWKCYMTRGKCMKYWK
49          YMRYTCKTTYRWYRMGRMTYYSKRYRRWGRGSYRACAARTGYRRYWTMTMCATTYMGYWK
            ****   . ****** ** ********* * **  .     *****.  *   . * ***

48          RMMYMKWWYYRYGGKCRAGCYCYRYWYMCCWRKRYYMYKMKYMTWYYRMYMRRKWTRRWR
49          RMMYMKWWYYRYRKKYRTATTTYRYWTCYTWRKAYYMYKMKYMGWYYRMYMRRKWCGATA
            ************  * *:.   ****    *** ********* ***********

48          MKTTRYKGRSWWKMKYWRKYKRRWRMRRCWMWMYKYKRGAYRRSYYYKYRKYYCYACTCY
49          MTWWRYKARSWWKMKYTRKYKRRWRMRRTTAWMYKYKGAGYRRSYYYKYRKYYACTTCTY
            *.  ***.******** ***********   ****** ..*************. :   *

48          RWAYWKRRKYWWKSRGYCCYYKSRKKRCKYKTYRMRTYWRWWKKKMMCCRGWWTRYWYRM
49          RWWYWKRRKYWWKCRTYATYYKSRKKRYKYKKYRMRYYWRWWKKKMMTYGRWWAGCWYRC
            ** **********.* *. ******** ***.**** **********    **:  ***

48          WRYYRRMRKAGCYRYYWYRRKMKRYYYYWAYYYKSKKAYRYRKYRRTYRRRWKSYATTRY
49          WRYYRRMRKRATYRYYWYRRKMKRYYYYWWYYYKSKKMCAYRKYRRKYRRRWKSYTCCRY
            ********* . ***************** *******   ******.********:  **

48          YYYWWRSMYRMKKWRYKACCRRWYACYRSMMYYYRTTGCMRRYRWMYRYYRKYWAKCGMG
49          YYYWTGGMYRMKKARYTGATRRWYRAYRSAMYYYRCGTTMRRYRWCYRYYRKTTMKTTCR
            ****  .****** **... **** .*** *****    ****** ******   *

48          WYYRKKYAYWKKKYATGKGYYMKKYYRRKRACYMKKYRYKACGCYGCKMYKKRKMWYYWC
49          ACYRKKTTYWKKKCRWKKKYYMKKYTRRKAGTYMGKYRYKRMATCTYKMYTKRKMWYYWY
              **** :*****    * ****** *** . ** *****  .    ***.********

48          YRYRRRKCGTAKWWRMYKGSRRGKKCWMYGCCRKKYRTMKKRKYYTMWYMYRWTTRRRYM
49          YRYRRRKTACGGWWRMTKRSRRKKGMWMYRYYGKGTRCAKKRKYYAMWCMYAAYGRRRYM
            ******* . . **** * *** *  ***    *  *  ******:** **    *****

48          WYRYKWMYRYWMKYKRKKKTKKKKRRYYRYGRYWYKSMKKKMYKTAMRYMKRKMYCGCTM
49          WYRYKWMCGCWMKYKRKKKKKGGKGRYYRYRRYWYKSMKKKMYKWCMRYMKRKMCYKMWA
            *******   *********.*  * ***** ************* .********

48          YGRMTGTGCAYYKRKMYYRKKRRWRRGRYCTSYGTWKKMGGAGGKWRYKRKKRCMWKKGG
49          CRRMGAARTMYYKRKMYYRKKGRWAATGYYKGCACWKGMRRWKKTWRYKRKKRTMWKKAT
              ** .:   *********** **    * .. . ** *     .******** ****.

48          MYTRYWYGKRWWGMWWYACRYAAGARGGWWWTYAMSKAKYKMTMWRRRCTRKYKKKYKRR
49          CCCRYWYKKRWWKMWWYGARYRMRMGRKWWWCCGMSKGKYKMCMWRRRTGRKYKKKCGAR
               **** **** ****..**       ***  .***.**** *****  ******   *
```

```
48      KTWYRWYCTRMRYKKRMYKYKKTGRKAWYYYMTWRARWKKKYMRWYTRRRKKKYTAGGKG
49      KYWYRACTGRMRYKKRMYKYKKYKRKMWYYYMYWRGAWKGKYMRWTYAGAKKKYAGRKTR
        * ***    *************  ** ***** **. ** *****     ****:.   .

48      TYYWRKTKKARKKYKYKATAAKGMYSMYKYMTYRYMKYKCMRYYARRKKYKKKKAGAGTT
49      YYYWRKKKKGGKKYTCKTCGTTRACSMYGCCYYRYMKYKYMRYTMRRKKYKKKKMATRYY
         *****.**. ***. *: .:.   ***    ******* ***  ********* .:

48      CAKCCWKKKKKKRRRYKKYKCRYGKGWKMRTYRYRKGSYWYKKYKYKGYASYWKSWYRRR
49      YRKTAWKKKGTKRGRYKKTKYRYRKAAKMRWYRYRKKSYWYKKYKYKRYMSYWKCTYRRR
          * .**** .** **** * ** *. *** ***** ********** * ****. ****

48      RRWRRYMYAAKKKYMRKTMWRMYYSKYYRRAGCMKRRKWMWRAKYKRATGMGKASRKGKK
49      RRWRRYATTWKKKCMRKWAWRMYYSKYYRGGRYMKRGKWCTGTKYKRWYRCATGGRKAKK
        ******  : *** ***  ********** .  *** **   :****    ....**.**

48      RWCWWKYYMYRKWTRSRKMYKKKCYGKRCCYSKKRRRRKKKRMYRRYYKYTTTRYGGYCC
49      RTAWWKYYMYRKWWRSAGMYKKKYYKKAMMYSKKRRGGKKKRMYRRYCKCYCARCAACYM
        * .********** **  ***** * *   ******  ********* *   :* ..

48      KRRSMAGCTWGKYTKRWMKSMRKKYYWYGAAMRRRTSWRYTAYTCTTAKWWYKAAAKRCT
49      KAGGARRYCWKKYKKRWMKSMRKKYYWYKWTAAGACSWRYAGCGTCCCKTAYKMGGKGMC
        *  .     * **.**************  :     ****:.     .*  ** ..*

48      YKGAYKRATYYYTYKMKYRYKKRTTKRRMTGYYRKTRWKWWMWYRWMKKGYGKCYKRWWS
49      CTTGYKRGCYYYGYKMKYRYKKGACGARMYSYYRKKRWKWWMWTRWCKKRTRKYYKRWWS
         . .***. *** ********* :   ** .****.******* ** **   * ******

48      KKRYWKKRWKRYMKRYKAAGTYRYMRYYCAAGTTKYGTRYWYRMKRAKRGRTKYYRWRAM
49      KKRYWKKGWTRYMKRYKWRKKTATMRYYYRMRCAKYAGRYWYRMKAGKRKRKKYYRWRCC
        ******* *.*******   .   ****     :**. ******* .** *.******.

48      CAWTWRWKKRYMMYRRWRYGAMYKAYYKAKSSTYKKKTCYMRRYRMKWCRKTMRYYGTGY
49      TTTCARWKKRTCMYRRWATKRMYKMYYKMKSSKYKGKCTYCRRYAMKASGKKMRYYRYRY
         :   *****  *****    *** *** ***.** *  * *** ** . *.****   *

48      YKYTYRKKAWTKRYWMSYKWRRMKMKRYKRKRYMMRYKRGCYGWCYMYMMMSKKAATACC
49      YKYWYRKKGACKRYWAGYKAGRMKCTRYKRKRYMMRYKGRYYRWAYMYMMMSKKCGCCTY
        *** ****.  **** .**  *** .************   * *.*********.. .

48      WACRCWKCAMCGRTSYRAATYWKMKAYCCWKYRWYGCGCYTMMCAYKKKAAMMKWWYTMY
49      AGTRYWKYMMMRRCSYRGRYYWKCKGYYTAGCRWYRTRAYYCCTGTKKTTRMMKWWYGMY
         . * **  *  * ***.  *** *.*     ***   .*    . **.: ****** **

48      SCYKCAWYACTYYTKKKSKKKAYKKYCCYMMRASGGTCGWRWKSKAKKYYYKRWSYKRKY
49      SYYKMCTYGAAYYCKKKSKTKGYKKYYYYMCGGGTTYAAWRWKSKGKKYYYGAAGTKRKY
        * ** . *..:** *****.*.****  **  ..   ..******.*****   . ****

48      YRKYKRKYRYTYRYMKKKSYRRKYRKAKWKRRWKYKRTGCCKCTAAAKYRKKKRYKKMRK
49      YRKYTRKYRYGYRCCTKGSYRRKYRKWKWKRGWGYKRGRTYKTACCMKYRKKGGTKKMRK
        ****.***** **  .* ******** **** * ***    * :.. *****   *****

48      GRMCRYRKRRCATGGGYRRYAGGRWKTWTYGYGTRAAWYMWWRKYRRYSRATCACYCAGS
```

```
49      RGCMRYAKRRARYTTTYRRYCAARWKYWACKYSGRRRWYMWWRKTRGCSRRYYWYCYCAS
            ** ***.     ****...*** *:  *. *  ******* *  **       ..*

48      MMYMSYGRRSRTRYRYWWYKWRYYRKMKKCGAAKCRMYWRKMYARMKCCKMCCGGYKMKM
49      MMYMSYSRRGGKRYRYAWCKWRYYRKMKKTCGWKMRMYWRKACCAMKTMKMTTACYKMKM
        ******.**. .**** * **********  . * ******  . **  **  . *****

48      RYCGYSKMGATWYRMRATKKKTCTGGGGYYWRMKRRRTKKCKKTTWTMWYWTRYYRRGYM
49      RYYAYGKMRWWWYRMRMKKTKASYKTTRYYWRMKRRRYKKYKKCCTWMWYWYRYYGARTC
        ** .*.**   ***** .*.*:.     ********* ** **    **** ***

48      TRYYRKYMRGTMRKYKKTGACKRKGSYYRWKRYKSWKARKCRKKKGYRKGTCYTYCWSAC
49      KRYYRKYMRRYARKYKKWRMAKRKASYYRWKRYKSWKGGKTRKKKRCRKKATYGYYWSRM
        .********   *****   .***.************. * ****  ** : * * **

48      TMMKYKRRRRYGCGTSRTAGRTYGRKRCTTGYWCCTCTRCRWKKWYRTGCAACGTRGAKC
49      YMMKYKRRRRYKTAGCRAGKRYYRRGAMWWKYWTYCYGRYRWTKWYRYRYGGSKKAAGKS
         **********  . .*:. * * *      **     * **.****   ... . ..*.

48      KKKKMYYRKAKATCATGAARWMRYKKMACYCWMWSYTAAGGAGKKGSRRYKWMSKASTCA
49      KKKGCYYRKRKWCYCAACGAAMRYKKMTACYWMWSYGGCARWRGKKSRRYKWMSKGCCTW
        ***  **** *   .:...  ******:.  ***** ...    * *********..

48      RYRKYMRYYCTKRYKRSMKYRAGWRYAACTYWTSWSWRKCAWKKGTTYYRMYKTYGAAYW
49      GYRKYMRYYTAKRYKRSMKYRRRTRYCGAGTWGSWSWRKYWWKKSYYYYRMYKCYRWRYW
         ******** :**********   **...  * ******  ***.  ****** *   **

48      KSWKRKWMKATKYKKYMYWMMKYGCRRRAGAAMKWRKYKKKYYRYKRRWKYKRRYCARRR
49      KSWKRKWMKCCKYGKYMYWMMKYAYRRRMKRRMKWRKYKKKTCGYKRRWKYKGRYTCRRR
        *********. ** *********. ***    *********   ******** ** .***

48      RMRYATTGCTKRKYKYKYRTCWRRKMMYTKRRWKKMRRWYCTKYATAGTKRRWKKYRMRR
49      RMRYRWYRTCKAKYKYKYRATTAGKMMYYKRRWKKMRRWYTGGCGCTKWKRRWKKYRMRR
        ****      * *******:    **** ***********    . :  ***********

48      TAATWRYTAKGCCATTYRMTCKRSKTCTWMTGGGYRWTMYKTTKKKGGAKGACMMKRRYY
49      CTGYWRYYMKKTYGCGYRMKYKRSKWYWACYRRRTRWYMYKYYKKKKKWKRGTCCKRACY
         :. ***  *   .  ***. ****          ** ***  ***   * .   **  *

48      YWRWKKMTYMKKSYKMRYYCKKAYMKRCAMMRRKYYWRYRTGTRKKMCKYRRYGYWKRGW
49      YWRAKKMKYMKKSYKMRYYYKKGYMKRMWMMRRKYYWRYRCTYAKKMTGYRRYRYWGRRT
        *** ***.*********** **.****  ***********    ***  **** ** *

48      CRCACAKRYATGAKKRRYRKKWWKRWRKKKKWRKMTKKCYKMYRRMYYAYKCRWRWRCCA
49      YRYGACKRYMWKWKKRRYRKKWWKRWRKKKKWRKCCKKYYGCCRRMYYRCKTAARARMYR
         * ...***    *********************  ** *   *****  *   * *

48      AKWGYARTMTMYKYWKKTKCTYAMWYYWRYKTGYSWTKRYKYGKMWTRMYKYRTTWCMRM
49      MKWKYCAKMCCYKYWKKKKMGCCATCTWRYKWSYSWYKRYKYRKMWGRMYKYAGGWMMRA
         ** *. .*  ******.*   .    **** .*** ***** *** *****   * **

48      CKRCRRWWAMSYSWRKKRRGCATWRRYMTATGTKACTYGCYYYYYYKKTWGAYYYYKRMC
49      MKRMRRWWRCGYSWRKGRRCTTKWRRYACCCKCGWYAYAAYYTYYYKKCTKWYYYYTAMM
```

```
                     ** ****  .***** **  :.****  .      :*..** *****    ****. *

48                  CYYRKYTKYKMYKRKKYYYCTYKYRTTCWRRCRATTYGARCWRYSTYGGMYKRWRRRYGC
49                  MYYRGYCTYKMYKRKKYYYYWYKYRGGTWGGAGRWWCRWGTTRYGYYRAMYKRWRRRYRY
                     *** * .***********  ****   *  .          **. * .*********

48                  AAGCAAMYRWMRKRKYWRKRKTYSYYYAAAAWWTKKWKYKRWKKATCTKTRYKACRCCTR
49                  CWAYGGMTAWMRKRKYWRKRKWTGTCCRRWRWWKKKWKYKRWKTCKSYKKRYKGYRYYKG
                    . . ..*  ************  .       **.*********.... *.***. *  .

48                  MMWGGCYGCRWTCYMKMRKYYWMKWTCRYMYYGTTKAYYYGTTGYTYKYYKYKKMGAYRT
49                  CMTRRTYRMRWYMYMKMRKYYWMKTKYRYMYYAKKKGYYYACCACTYYKYKKMCGCRK
                     *    *  **  *********** . *****...*.***.  . : .******* . *.

48                  YTGKKCCTYWTTAKKYKKYRAGRRYWYYRYKTMYYYRGMKKRRAWKYAYWYKYKYKKKKY
49                  CGKKKAAAYWGCGTKYKKYRGARGYWYYRYKYMYYYRKMKKRRMAKYRYTYKYKYKGKKY
                       **..:**  ..******..* ******* ***** *****  ** * ****** ***

48                  YYKRYMTAYGYKKMKYKMKYRWKRGGCRAWRKSRKRSSKGWWKKKKTGTARCKAGGGRRY
49                  YYKRYMWMYKYKKMGCKMTYRWKGSKSGRTGKSRKRSSKRWWKKKKCRYCATKWRKRGRY
                    ******  * ****  **.**** . .    ******** ******   .  *     **

48                  MKKRKSYYYRYWKRSYWSYKRYWKTYKRMKWKRYTRRMWCKCTKKYGYAKWWYSRKKKGA
49                  MKKGTSYYYRYWKRSYWSYKRYWTKTKRMKWTRTCGRMTTKTAKKTACRKWWYSRKKKRW
                    *** .******************.. *****.*   **  * :**  .  *********

48                  KKYYMWRRWWYWRRSWRMTTTSAWWYKYKKWWGKKAYRRTAGCCSKWRYKKACCGKMKKY
49                  KKYYMAGGATYWRRSWRCAAASTWWYKYKKWWTKGMYRACRRMMGKWRYKKRYTTKMKKY
                    *****     ******* :::*:********* *  **      .******    *****

48                  YACARKKTGMKRGKYYYMMSMKKSYACKRKYWYKKYYMWTARRSKKCATCWYMRKKGRYW
49                  YGACRKKACMGRKKYYYMMSMKKSYWYKRKYWYTKYYMWAGRRSKKTTCMWYMRKKARYW
                    *...***: * * ************  ******.*****:.***** :  ******.***

48                  RKKYYWKKKSKKRKYMYWYRKYYYYKKSKWYTATTMACCAKRCARRTRGKYKKKKYRYRR
49                  AKKYYWKKKSKKGKYMYWTGKCYYYKKSKWTYRYYCWMMRGASRGGKGRKYKKKKYRYRR
                     *********** *****  * ********            .   .  ***********

48                  KKTRKKYMRMSRYTYWMSCWKGKKRRYKKKYYRKYMGKRSMKKRMTAYGCCRKYWYYYRK
49                  KKGRKGYCGMSRYYYWMSYWKCKKRRYKKKCCRKYMKKRSMGKRMAGYATTRGYWYYYRG
                    ** ** *  **** **** ** ********  **** **** ***:.*.  * ******

48                  KYRKKRRWKYYRYKMKCCYTGGRCCKKKWARGYKRKATGCMTWYMGKGTAATCCKAKCGT
49                  KYRKKRRWTYYRYKMKYAYKAAGYYKKKTWRRYKGGCCRMMKWYMRKRGGGGYYKWGTRY
                    ********.******* .*...   ***  * **  .   *.*** *  ..   *

48                  GTYKKKKRKWRMTKKRGGCCGTKMWKKAYWSYYYTTYYRWTCWYYRRKYYWYSKKMKRYY
49                  KCYKKKKRKWRMKKKRSKYYRCKMAGKCCACCYYYKYYRWCTWYYRRTYYWYSKKMKRYY
                      **********.***.      **  *.  . ** .****  *****.************

48                  MRKKCCACYGRRYCACKRKWKRRTWMSCCYCAKMCGMRYKWWKCYYCKASGTTWWYRKAK
49                  MRKKTYGTYRGGCYWYKAGWKRRWWMSMACYGKMYRAGYKWWKMYYYKRSRYWWTYRGMK
                    ****  . *       *  **** ***  .  .**    ***** ** * *   * **  *
```

```
48          GGKRYWYWYYYATTCAMMYAGTKTRSYCGTKRYKRRTCTKGTKTSKYTTCTKKKCCGKSG
49          RKKRYWCACYYMYWTRMMYCKKKKRSCGAAKRTKRRCTGKTCGCSKTCCACKKKMYKKSK
              ****   **     ***. .*.**  .:** ***   *    **   . ***   **

48          YRKYYMWYYRSRAGRGRCWAGGCYYYTACCKGKSMMRTRRGRKYYYWRCWTWKTKKARYK
49          YRTYYMWYYRSRMRRAGMWMRRYCCYWRYYKKKSMMGYRRARKYYYWRGTGWKAKKGRYK
            **.*********  *.  *      *    * ****  **.*******   **:**.***

48          WRYRRMYYRYYYRRYKKGTRRRTKTKRAAATKSGACTTRRWTYRMRMTWRKRRKYRMKMA
49          WRYRRMYYRYYYGGYGKRKRRRKTKKRWMMYKSRWYAARRWKYRMRMKWRKRRKYRMKMM
            ************  * * .***...**    **   ::***.*****.***********

48          MWTKYGSYGGAAAAKKWRYAAAKMRKWKKWYRMKRKRKACTAARRYKRMWYTKGKTRWMY
49          MWKKYKGTAKWMWTKKWRTGTTKMGTWKKWYRMKRKRKCTCTTRRYKRMWYKKKKYRWMC
            **.** . .    :**** .::** .************.  ::********.* * ***

48          MRTTKCCYKKRYWAGRTWYRKSRTKYGKKTKKKYMKTRYKMYMMACTKYTKKRMMRKKWK
49          ARWYKGMYKKRCAWKGWWYRKSRYKYKKKKKGKYMKGRYKMYMMMMYKYKKKRMMRKKWK
             *  *  ****      ****** ** **.* **** *******   **.**********

48          YGYYSRYYYRKCKKKMKMWKRRKYKWWKKKRYYCCKYRRMKKTAKW
49          YKYYSRYTTGKYKTKMKMWKRRKYKWWKKKRYYTYKYRRMKKCGKW
            * *****   * *.*******************  ******* .**
```

The pairwise alignments of two accessions 48 and 50

```
48          WWKRYYGGGYYRKYRKRAYRKRKYYYRRCRSRCKKRSRRRYKTAGAAACSKKTKYMKTAK
50          WWGRYYKRKYYRKYRKRRYRKRKYYYRRTRSRTKKRSRRRYKKMKTGTTSKTWGTMKAGK
            ** ***   ******** ********** *** *********.  :.: **.   **:.*

48          YWKKKSWWRRWAARGKMKYYKAYAYWCRKRYTYKSRYYYMGKWMYRWRWKYAYAGYRRKM
50          YWKKKSWWRRWWWRTKMKYYKWYRTWTRKRYWYKSRTCCCAKWMYRWRAKYCYWRYRRKM
            ***********  * ****** *  * **** ****    .******* **.*  *****

48          YMMRMRWYATWYYYRYYYKWYCKYCWYWKRRRMSYWWRWRKYCRRSMTARRRRWRRKWWK
50          YMCGMRWYCYAYYYRYYYKWTYTTYWYWKRRRMSYWWRWRKYTRRCMYRRRRRWRRKWWK
            **  ****.  *********  .  ***************** **.*  ***********

48          MGRYRAGYYTCCWYYKYACGTGKKRMYYYYRYYYCAYWKAKWKWKGRCWWARMYMRYRWY
50          MRAYRWRYCWYYWYCTTCYRWRKGAMYYYYRYYYYRYTKRKWKWKCRYWWGRMYMRYRWY
            *  **  *    ** . .    *  *********  * * ***** * **.*********

48          YYYMRYYYYCRRGTYYSRYWMYWTSMRRRYRKYYMYRYRRCRMRWWMRKMKARKATTWRY
50          YYYMRYYYTYRRKCYYSRYWMCAYSMRRRYRKYYMYGYRRMGARWWMRKMKGGTRYYWRY
            ********  **  *******   ************ ***   ********. .   ***

48          CMMRMRYRYWSYKYKYYRRRRMMYKTYWRYYKRRMRTKCRSRRRYKRMCGGYYWRSWYMM
50          YCMRMRYRYWGCKYKYYRRRRMMYKYYWRYYKRRMRCKYASRRRYTACTATTYTGGWYMM
              ********. ************* ********** *  *****.   .  *  .****

48          WKYYKMYMYYYRMRRMKKRSWKRGKGGATWWMCCGGTKYCAAYKRRYKRWWRGRYRGRKY
50          WKYYKMYMYYYRMRRAKKRSWKRAKKTRKAAMAARRWKYYGRYKRRYKATAGRACARRKY
            *************** *******.*   .  *..   ** . ******         ***

48          KKMMCYGYYCKRMYYKKKYCYWGKYAWYYKTYKRRRKWMRKGAATMYKAKCKRSYKRYMK
50          KKMMMYRCTYKACTYKKKCYCTTKTTWYYKYYKRRRTWMRKACGCMYKTKYKRSYKRYMK
            **** *    *   ****     * :**** *****.****... ***:* *********

48          AAKMRYSACMYYRYYYCKYRYSWWKRCWRYRYWRWYKAKMRKAWAKYWKKRYRKKKKKRK
50          RRKARYSMYMYYRYYYGKYRYSWWKRYWRYRYWRWYKTKARGGWWKYWKKRYRKKKGKRG
              * ***  ******* ********* **********:* * .* *********** **

48          YRKYRYKRTTGRGCCSKKKCKKYRMRMKRKKSYKYRRRYTYTARSRCWKKKRRCGGAYMY
50          YGKYRYTRYKKGKYASKGGYKKYRMRMKRKKSYKYRRRYAYYWRSRMWKKKRRYRRRYMY
            * ****.* .    .**   *******************:*  *** ******    ***

48          MMRRWRMRYYWWKYGKKARYRTMKWYYKYGRKYYYTYWRTGTRRRKKKRKKTGTSAMKWR
50          MMRRWRMRYYWWKYRKKGRYRAMKWYYKYKRKYYYCYWRCRKRRRKTKRKKWRWCWMKWR
            ************** **.***:******* ***** ***  .****.****   . ****

48          WYTRRRRTTGTGYCYRWMMMYKWRRRWSRWYTGACATWRYRRKAGAGAMGCGMRAYMTYY
50          WYKRRRGKWRWAYYYRWMMMYKARRRWSRWTCAGACAWRYRRKGRGTGCRMRMRRYMWYT
            **.*** .   .* ******** *******  ....:******. . .    ** ** *
```

```
48          KYYRRMTRYKYRKRTWWMWRAAGGCGRAWMAKWGAAARCRRGAGKRYRYYMRAYKAGKRY
50          TTCRRMGRYKTGGACAWAWRWMRAYARRWMTKWTTGGASRGARRGRYRYYMRGCKRRKRY
            .  *** ***      * **   . .* **:** :.. .* .   *******. *  ***

48          RGATCYYSRACYSWRWMWSMRRKWWRMYKKYMYMMKRGKYKCRTKYKMYRRMWMCMYRKM
50          GKTGAYYSRGMYSWRWMWSCRRKWWRMYKKYMYMMKRKKYGTRGKYKMYRRMWMYMYRKM
              : .****. ******** ***************** **  * ********** *****

48          WKAYCTTYKWCMKYKKKWRKWTRGYWKSMKKKMKMKRYKSRYRYKRYKTMRYRMKRKKRK
50          WKGYMAAYKWAMKYKKKWGKWKRKYWKSMKKKMKMKRYKSRTGCKRYKYMRCGMKRKKRK
            **.* ::***.******* **.* *****************   **** **  *******

48          WKMYWARYKRKKRAAYKMKKYKMAKMRMKKSKMKTRMYWWKCCKYWKYKKKMTTWAYRYK
50          WKMYAMRYKRKKRWWYKCGKYKMRKMRMTKGTMKCRMYWWKYTKTTTTKKKCYCTWTGTK
            ****  *******  **  **** ****.*..** ******  *  . ***        *

48          KRYRYKYRCYGKKKTTKYRKYKYYKGKWKCRYRYYKRYKRKAGGTKCWAAGYKYRKKRKR
50          KRYRYKYRYCTKKKWAKCRKTKCYKAKWKYRYRYTKGYKRKCRAAKTTGTRCKTRKKRKR
            ********   *** :* ** * **.*** **** * ****. .:*  .:  * ******

48          KWKAKYKKWRATKKYKKKTTYKRWKKCTCKTRTTKYKKKRYCCRMMRWYWKWAWYRRYCK
50          KWKRKYKKWGGWGKYKTKAGTKRWKKSYAKKRKKKYKKKRCTTGCMRTCWKWWWYRRTYK
            *** ***** .  ***.*:  *****. .*.*..******     **  *** ****  *

48          WMKKYRYKMYYKYMMMKYWAYMSKKYKAKKKKYRGGYTKKKYKKYAYTRKRTRWYYYYGC
50          WMKKCRYKMYYKYMMMKYWMCCSTKYKCKKKKYGCAYWKKKYKKYWYCRKRYGTCYCYKY
            **** **************   *.***.*****  .* ******* * ***    * *

48          KTCKWARGKMATCTYAYWRKMGACATTYTKRYKRRYRKKMYRKRKCTTTTTAMKWRKYRA
50          KYYKTTRRKMGGGYYGYWRKMRGACAGYGKRYKRRYRKKMYRKRKYYWGGAGMKWRKYRR
            *  * :* **.   *.***** ...: * ****************     :.*******

48          YTTGMGKYYYWMYRAWAGTAKWKCTAKYYACRYWKMKRYCRKTYAWYYKSWMYYTGMWYW
50          CYCKAAKYYYWMYRWWRKKMKWKYWWTYYRYGTTKMKRYMRKKCCTCTKSWMYTCAMWYW
                 .******** *  . ***   .**     ***** **. .   *****  .****

48          YYSYMGAYWGTARKKACWKYWCATSRYWSMKKRRTKAMMKCKKCGRYWYKSCTGTTGTGA
50          YYSYCKCYTAAGRKKCTAGCWTGGGRYWSMKGRGCTTMMKTKGTTGTWYGSYACACCAAG
            ****  .* .:.***.    * . .****** *  .:*** *     ** * : :  :..

48          WRKYRYKYTTKGARRAYAAKCAATRMRTGKSGTACACRYGACAYKRYTMYTMTKRAASCW
50          AGGYGCKYAKKRGRRTYGTTYGRYRMRYSKSRATTTGRCRMYRYGRCCMYWMCKRTTCTW
               *  **:.* .**:*.:. .  *** .** :: : *     * *  ** * **::. *

48          KGTAYYTWYYMKRGWMYWYRYYYRRAMTKWWRKRYKTTKMWRCTRYRMYWMMRKRKGTGK
50          KTWCCCKWYYCKGRWCCWCGYYYRRRMWKWWRKRYKCCKMWRTGGCRATWMMRGRKACAK
            *  .  .*** *  *  *  ***** * ********  ****    *  **** **. .*

48          KYKKRTWKYWYMYMWARCACKKYKSMTKYYKSYKRKKCGACGTTAGTYMKGMYKWAWKYW
50          KYKKGYWKTWYCYMTRGMGYKKYKSMCKYYKSYKRKKTRGMKYWCACTAGRAYKWGTTYW
            ****  ** ** **    . ****** **********  .    ..      ***. .**

48          WWKRYKGKGCKGKKKSTRYWYRKKMKRMRYWKRRYACCRCWCYYWTYRMARMWKKYYKMR
```

```
50          WWKRYGRKKSKRGTGSYGYWTRKKMKGCRYWKRRYGTTRYWAYCWWCGCRRMWKKTYKMR
            *****  * .*  . *  ** *****  *******.  * *.* *     ***** ****

48          CYGRWMTKTATTCTMKRRYRWGRAKYTKKKRMYRYWKRYMKWWRKYWYRKCWYRYTRWRK
50          MCRRWMKKKGGGYAMTGRYRARATKYCKKKRMYRYWKRYMTWWRGYWYRKTWCGTYRWRT
               ***.*..   :*. ***   :** *************.*** ***** *    ***.

48          YYWRRYYYCYCMWKRYYMRYYYRATARCCYRYWARCKGTYRKRYRGRCCTYWAMMRKGSR
50          YYWRRYYYTYTMWKRYYMRYYYRRKRGAYYRYWGAYGRGYRGGYRKGYTCCWRAMRGRSR
            ******** * ************ .  . ****.     **  **      *  **  **

48          KYKYYYKKKRKGCGCGWYWCWYKRYKMYTGASGCARRYYKYYRKKRYWYKRRKSMYKYRK
50          GYTYYYKKKRKRYKMTWYWYACKRCGMYKAGSSTGRRYYKTYRKKRYWYKRAKSMYKYRK
             *.********     ***   **  **...*. .***** ********** ********

48          YKRRKKRYRWRKCWYKRKAASWKRKMAWWKRSKKGTTTAGGGYYWRRKRYSTYKYTTTCT
50          YKRRKKRYRWRTGWYKRKCCSWKRKMTWTGGCKKSYCARRRRYYWRRGATGGYKYCYWMY
            ***********. *****..******:*   .**.  :    *****   . ***

48          AASSMMWKMMKYKKKYYWMMRCGKRYRWRKACKKKKYKRTGTTKYWYMRYSTTKYKKRRY
50          TWCSMMWKMMKYKKKYYWMMRTTKRYRWRKWYKKKKYKRYSKWKYTYMRYSCCGTKKRRY
            : .******************  *******  ******* .. ** *****    *****

48          YRCKKRWKKYMRTRYRGGKATGTMMKYRAYYKYYWSSCGKKKRRTYRYMRSRRYKKMRYY
50          YRMKKATKKYMGCGYGRKKRYRYMMTYRWYYKYCASSAAKKKRRKYRYMRCGRYKKMRYY
            ** **  ****   *   *    **.** ****  **..*****.*****. ********

48          CGKYRKGAGTTAMAYYMCKKKKYAYMMRTRWKMMMSKRWKMRMYYRTTWKMYWWMKWYRK
50          YKKYRKRTAWKMMRYYAMGKKKYGYMMRCAWKMMMSKRWKMRMYYRYKWKMYWWMKWYRK
              **** :. . * **   ****.****  **************** .************

48          RCKYWKKRRYKRKKRARMWYRKKMCKTWRYWMKKKYCCYGCCYKKKYGTAKCTYTTGACT
50          RMKCTGKRRYKRGTAGAMWYRKKMYKCARCAMGGKYYYYKGTYKKKYAAGGTKYCGRCAA
            * *   ****** . . ******* *  *  *  **  *   *****.:.  .*   ..:

48          KYAYAYKRKKWTGAKYWGYGKWTYYRCYAKRMCRMKKMMKTKYKWKTRCAAYKRYYKATA
50          GYGTRCTRKKWYRWKYARTRTAWTYGYYGGRMYRCKKMMKKKYKTGYAYRGYKRYYKCCG
             *.   .****   **    .   *  *. ** * *****.***      .******. .

48          TMYWKKKKTKRYARRRRWRKKMRYCYKYKMMYKRRRKKMRRWYKAAGCKMYWWKRRWYYY
50          CMYWKKKKKKRYMRRRRWRKKMRYTYKYKMMYKRRRKGMRRWYGRGAYKCTAWKRRWYYY
             *******.*** *********** ************ *****  .. *   ********

48          RMRRWRYKKGKKCRYATWWRRRKSKKCAGYTGYCMRRYYKWYYRKTMYCACYKYKKMMYW
50          RMRRWRYKKKGGYAYRYWWRRRKSKKYWRYATCMAGAYCKWYYRKYMYYMYYKYGGMMYW
            *********     *  *********   *:      * ****** **   ***  ****

48          CRKKYYWYMSYKCWKMRYYKKRKRRRYKYRYYRMKRRYCGGWKYWYGYKYRYWRKKKKKY
50          MRKKYYWYMSYKYTGMRYYKKRKRRRYGYRYYRMKRRYTTRTKYTYKYKYRYWRKGKKKY
             ***********   ************ **********    ** * ******** ****

48          YYYYRTTCCYKKKMCYMKKKRAWRKRSRWKKRTKRKKMKSYYSYWRSYWSKRYKWKKACM
50          CYYYRYWYTYKTKCTYMKKTGRTGKRSGWKGRYKRKKMKSYYSYWRSYWSKRYKWTKRMM
```

```
                     ****    **.*  ****.    *** ** * *********************.*   *

48                  KRMKWRMGWMMGWMWKCAWMKKKATWWYSRTSSYGAKKYMWRWMATSKYYYKWAKKSMRK
50                  KGMKWRMKAMMRAMWKMGWMTKGGCWWYSRKGGCRWKKYMWRWCGASKYYTKWMKKSMRK
                    * *****  **  *** .**.* . *****...   ******* .:**** ** ******

48                  AGYYKSYRAAMYMRSSWRYAATGKWWYWYRRAKRCGAAGCCTKKYYYRAYRYKRRMKKRR
50                  CAYYKSTGRMMYMRSSWRYRRYRKWWYWYRRCKRSRGGAATCKKYYYRMYRYKRRMKKRR
                    ..****    *********    ********.**. ....  ****** ***********

48                  KTCTWKTMGRWMWKKWGAYKRGWWSKKWKRGCCWKKYKAKRKRYRKRTACAAGKYKYRYY
50                  KATGWKKARRWAWKKAKWYKRRWWSKKTGGTTMWKKYKMKRKRYAKRYRYRMRKTKYRYY
                    *:  **.  ** ***   *** *****      ***** ***** **      * *****

48                  RRTGGRMRKSKKMSCRYTYMKMKYYKKKRRGKMKYYYKRSMKATGCRKRYWYYKYRKRKR
50                  RRGKRRMRKCKTCGMRYYYMKMKYYKKKRRRKMGYCTGRSMGRWKSRKRYWYCKYRKRTR
                    **   ****.*. . ** ************ ** *   ***    .****** *****.*

48                  YAKKKMCCYYRYKKKRTRCYYKTTGWGKKWMYRAKGCCKATKKRKSYWYKYYKKSYWRAK
50                  YCKKKCMMTYRYGKKRCATYYKCGKWKKKWMYRGKRYAKWWKKRKSYWYKYYKKSYWRWK
                    *.***    *** ***   ***   * ******.*  .*  ***************** *

48                  TWWRATTCWMRWRYYKYKKKYRMGKWTGTWCTTCAKKKRKKRYKKKYMKKKYRKYTRKKM
50                  GWWRMCATWMRWRYYKYKGKYRMKKWGACTAKYYWKKKRTKRYKKKYMKKGYRKYAAGKM
                     ***  : ********** **** **  .  ..   ****.********** ****:  **

48                  KYKKAYYSYTGAGKKMACRRCWRKRWGGAACKYRMYRYTACYRYYYRKKKWGKCCKWYKR
50                  KYKTGYYSCARTCKTMTTGGAAGKRWRRWRYKYGACAYWRYYRCYYRKKKWKKMMKWYTR
                    ***..*** : : *.*:   .  ***     **    *   ** ******* *  ***.*

48                  KKTMKYKKRKRKGKYYKTAKKYYYMSGCCTMWWTKYRMRKRKCAKYKAATARRRWYRGTR
50                  KKGMKYKKRKRKCKYYKKMKKYCCCSTAAACAAAKYRMRKRKTGGTGRRGRGGGACARYR
                    ** ********* ****. ***   * ..:   :******** .               *

48                  SYWKKWYCKKRYKWRKYCTCKKKRCGTKSKKKYYWYRYSAKKGYYRYCKSKWKYYKKKYY
50                  SYWKKTTYKGRYKWRGYYYTKKKRTTATCTGKYYWYRYGGKKKCYRYMTGTTTCCKKKYY
                    *****   * ***** *   ****  :... *******..**  *** ... .  *****

48                  RKWKYKRYMYSYGATRMYYYRMRCCRWYRRYYRGYWAKYYYKYRKWKKATGKMYYMKART
50                  RKWKYKAYMCSYAGCRMYYYRMRTARWYRRYYRAYTWKYYYKYRKWKKRWKKMYYMKWGY
                    ****** ** **.. ******** .********.*  ***********   ******

48                  RRKCGKMKMKRTKKYRYKRKKKKAKRKKAWMWWSRYYYAKCKYRKKKYR
50                  RRKMAKATMKRKKTYRYKAKKKKRKRKKGWMWWSRYYTTTTKYRKKGYR
                    *** .* .***.*.**** **** ****.******** :. ***** **
```

The pairwise alignments of two accessions 48 and 51

```
48                  TTWCKKYMKYWGKTATYWKCWRRYYKRRYYYMKWCRWYKCKYRKYWCTWWCAKYRRYKRW
51                  AWWYKGYMKYTAGCGAYWKTWRRYYKRRYCCCKWYGWYKMGCRKYTYWWWATKYRRYKRW
                    : * * **** .  .:*** *********   **  ***   ***   **.:********

48                  MYKMRYWRYMRKYRRSAGGYYYKYRKYMYYRKGYKKGWYYYRYYRRMRYYRYWCARMMYK
51                  MYKMGYWRYMRKYRRSWKKYYYKYRKYMYTRKKYKKATYYYRYYRRMRYYRYWTGRMMYG
                    **** ***********   ********** ** ***. *************** .****

48                  YMYMRYYKCGGWATRWYRKRRWYTYYSKWRYMRKWYRACYGYKSRKKCTTTYRYRRYKRC
51                  CMYMRYCTTCAWWYGTYRGRGTYWCYCTTRYMRKWYARMYRYKSRGKTCWWYRYRRYKRT
                     ***** .  .*    ** *  *  *.. *******   * **** *    ********

48                  YAYKRKRKYYMRYTGYAKWKWYMYYYYCRRRMYYKMKRYRYYRGYACCCGGYCYRYRRGG
51                  CRYKRKRKYYMRYWKCWGWKWYMTTCTMGRAMYYKMKRYRYYRTYWYMTATTTYRYGRAT
                      ***********     *****      * ************ *    .   *** *.

48                  YTRWRKYAAAARYYRRWSRTAGKCWAAKYWRKRMSYWWGCMARCTRRMRWWWKMRRKTYM
51                  YGRWRKYGCGGRYYRRWSRAGTTYWMCKYWAKRMGTATRYCCRTGRRMRWWWKMRRKWYC
                    * *****....********:. . * .*** ***.      .*  ************ *

48                  GRRKWAAYGCAKCRMYMRYWRWYCTCTTKYMKWRGKCYWKSAYYSTCYCKKWRRRRRRRK
51                  ARRKWTGTTGTKARMYMRYWRWYMWGYYKYMKWRCGYYWKSMYYSYMYYGKWRARRRRRK
                    .****:.   :*.**********     ******   **** ***  *  *** ******

48                  YWKMRMRRCRYKYRYYYRWMKCYCWYCTGKRCTWCCKCGCKRWYASRWYRCCRMMKCRMY
51                  YWKMRMRRMRYKTRYYYRWMKYYAWYTATKGMATTTKMRMKRWYGGAWYRYYGMMKGRMY
                    ******** *** ******** *.** : *  :   *   ****.. ***   *** ***

48                  SCCCATKAMYRTYACCAKWGRYAGCACKRKYKRMAMYKYSMYGRWKKYYTAAYRGAKYCT
51                  SMGTCKKGMCAWCWTYCKWKAYGKTTTGRKTTGMMAYKCGMYRRWKGYYYMCTAAGKTTY
                    *   ..*.*       .**  *.  :  **  .*  ** .** *** **  .  ..*

48                  TCYYSCGKKTGKGTRSYKRYKTYWRRKKYMSRYYYRYYKYSKWGMRTRCKKACRMWKKMW
51                  ATYCCYRKTWKKRYRSYKRYKCTARRKTYMSRYYYRYYKYSKARMRARMKKTYRMWKKAW
                    : * .  *.  *  *******   ***.**************  **:* **: ***** *

48                  AWKTGRWKYKWRYKKKKCCKCTKMRGRKRRYRKKRCKYSYRRKRKWKRKRKYTCYARGAC
51                  WWGCKRWKYKWRYKKKKTAGTAKMATRKGGYRKKRMTYSYRRKAKWKRKRKYYMYRRARY
                     *   ************ .  :**  **  ***** .****** ********  * *.

48                  KRYKYRAAKKRYTYWWKKRYRYRWWCYRWGMKYRYGASRRYSMCKCGYKMKYYMRGKARG
51                  GGYKYRWRKKRYKYWWKKRYRYRWWYYRWKMKYRYAGSRGYSMMGATCKMTYYMRRKMAK
                      ****  ****.************ *** ***** ..** ***  .  **.**** *

48                  RWATYWMKRYWKMGMKKYWWKRRKAMKYRKRKGKTACKYYYYMMKKMYYRMAKRKKGCKK
51                  RWWCYWMKRYWKMRCTKYWWKARKGMKYRKRKCKKMYTYCYYMMKKMYYRMWKRKKSYKG
                    **  *********  .***** ** .******* *.  .* *********** ****. *
```

```
48              YATCAARKRRSGAKWYARYAYTRYMYKATCRKKKKYMCKYRKRYAGRYCAKCKWKKWMKC
51              TRYTTGRKRRSATKWYRRYMCKAYMYKWCARKGKKYATGYRKRYGTRYACKGTTKKAMGY
                    :.*****.:*** **  . ****  .** ***   *****. **..* . ** *

48              SYKCYMMWKKRTRARWWTWWYWRMTKGSRRMGGKTKTKGKWYRRRTCKKRYKKRWWAYGA
51              GYTACCMWKKRGGTRTWKWWYWGMWKKSRRMKKKWGWKKKWYRRRWSKKRYKKRTWRCSR
                .*..  *****  :* *.**** * * ****  *   * ****** .******* *  .

48              RMRYYRYWRKKKMRMCACCCGKYCGMRMKKWWTCYCCGGGKWRMYYMKKMGCCGYRACYR
51              GMRYYGYWRKGKMRMYGTATKKYYRMRATKWWYMYAATSKKWRMTCCGKMKYYKCGTYYR
                 **** **** **** . .  **  ** .***  *.. . ****    **      : **

48              CTCTYKTYWRRYYYYTGTKTWTTRWWCYYGGKAKMAMYMATARMMYCYYKRMYYMYKRKK
51              AAMYCTYYWRRYYYYGAKGCTAWATAYYCRTKRKACACCWAWRMMYMYYKRMYYMYKRKK
                .:   . ******** ..   :     *   * * .    : **** *************

48              KYWYRCYWMYGKRTTCAKAGRKCTCGAAYYRRKKYRTMRMRYKYSYTYGTKRMKRMASKK
51              KTWCGTYACYKGGKWYMTWRRKSKTTGGYTRRKKYRGMRMRYKCSYYYKYKRMKRMMSKK
                * *   *  *   .   .  **..  ..* ****** ****** ** *  ****** ***

48              KKKKRRGKTKMYRKRSMRKKRTKKYWYYAAKCKRKCYTGTRTYWKKGMYYYWKKYMCCCY
51              KKKKRRRKAGMYGKRSMRKKRYKKYWYYMMGYKRKTTCCCAAYWKKKMYYYWKKYCAYAY
                ****** *: ** ******** ******    ***      :**** ******** . .*

48              RMWMKYRYRYRCCCYWARYGYKTCCKYKKYMKWRYACYKKAKRKKRKTCCKTKAGARGCT
51              RMWAKCRYGCAYYYYWWRYRCGAAAKYKKYMKWRYGAYKGWKRKKRKYYYGCGGTGAAMY
                *** * **      ** **   :..**********..**  ******      . . .

48              TGSRRYKRATRYRKRTCCTRGGAWKMGCRKKKTRSRGGCRYYMMCRCRCKRYKKKTCAKM
51              WSSRRYKGGCRTGGAKYMWRAARWKCRSGGGGARSARKYRYYMMYRMGTGACKKTKYMKM
                 .***** . *    .   *.. **  .    :**    ***** *      **..  **

48              TRKKYYSRWMYKTGTKMRKWYRKRRKYWKRKRKACTAAGKAAMWYKKRTMCKKRKKKSRK
51              GGKKTTGGWMYKYRKKCRKWYRKRRKYWKGKRKCTCTTRKGTMWYKKGCMYKKRKKKSRK
                  **  . ****  .* ************ ***.  :: *.:*****  * *********

48              YKTAAKKKCRWKKYKKGTYTCKWYKYRRMMYRMCRKKWYKWYSCMRRWKYKYKCMYRYKT
51              YKYRTKKKMATTKYKKKCYWYKWYKTRRMMYGCYRKKWYKWYSYMRRWKYKYKMMYGYKY
                **  :***   .****  *  **** *****   ********* ********* ** **

48              ATCKKWWWRKYMYWTAGCCRYYGMKRMACYMARRWRGKWYYRWYKYYGRYWYSWWCTCMW
51              MYSKGWWWRGCMYWYRRYMAYYKMKGMCSYCWRRWRSKWTCRWYKYYRGYWYSWWTCTMW
                  .* ****  ***      ** ** *..*  ****.**  ******  ******   **

48              KWRAMRYAGAYRRCCYATTTYMCRWMRRRWCMWSTWYRYMSRCYMRRTYKATGTAKSYKK
51              KWRMMRCWRGYGGYYCGYWYCAYRAAAGRWYMWSWWYRYMSRMYMRRCYKRYAAGKSCKK
                *** **   .*     .      *    ** *** ******* **** **  .:.** **

48              AYAWRRMYRWRWYMCYYMMSAGKGTKATYCTKRKYYYMYKRKRYMRGYRRWKKRKYMRGM
51              TYWAGRCYRWRWCMYYYMMSTAKRKKGKYTCTRKYYYMYTRKRYMRKYRRWKKRKCAGKM
                :*   * ***** * *****:.* .*..*  .*******.****** ********    *

48              RGMYRYYKSSKCRYRMGRSRRYWAMRYYTKYGTKYTYKRKKKRAWRYKRCTMWGCYRATK
```

```
51      GRMYGYYKSSKYRYGCKRSRRYATMRYYKKYCGKYWYKAKKKRMWRYKRTCATAAYRWKG
          ** ****** **   ***** :****.**  ** ** **** *****    ..** .

48      KWRRYYRTYSTRWWRTMTYWTYYRMCGYRMRYRRKSGKKYKKRYKACTKKSYRRRGAAYA
51      KTRGCYAWYSCRWTGKACCAGYYRMMRYRMRYRRKSSKKYKKAYKRSWKGSTGGGKRWYR
        * *  *  ** **  .     ****  *********.***** ** . * *       *

48      GTYSRAGRCWMWRRKKRWCMMRRKKMRMRYYWRKGTTKYSKYRMGWYYKYKKGTRRRRKR
51      KKYSRMRRMWCAAAKGGAGMMRRGKMRMRYYWRKTACKYSKTAMKWYYKYKGAGRRRGKR
         .***  * *    *    **** ********** : ****  * ****** . *** **

48      KMKGACKATACCGWMTTWRYRYMKWRAKKKYYYSWRRCCCYCTGTKKWYWYRMGGCKGRR
51      KCGRTTKWAWMYTWMWYWRYRYMKWRGKKGYYYSWRRMYAYGARAKKWYWYRMKKTKKGA
        *   : * :    **  *********.** *******  .* : :********   *

48      KATKKYYYKSYRCYTTACRYRYKRRWRRCYKRKRWTCKKMRWMKKRRTAWYKYKRAGWWS
51      TRYKKYYYKSYGYYWACMRYRYKRRARRYTKRKRAGSKKARWMKKRRAWTTTYKRTRWWS
        .  ********  * :. ******* **  ****  .** *******:   .***: ***

48      KKKYRYRWKRGTKAGKRRKRYKTCCRRKTACAAGKYKRKKYKKWKAWYKWRCATRRTTRK
51      KKKYGYRTKRAGTMRKRRKRYKGAMRRKCGYRMRKYKRKKYKGWKRWYKWGTRCGGWGRG
        **** ** **. .  ******* . *** .    ******** ** ****        *

48      CKWRWKWRKYYKSYKTKRYKKAKACKYRMMSGWKWAWYRGYYYKGYKKRYKMKTYYKKYS
51      TGARWKWRKYTGCTKKKRYKGRKWSKCGCMGRWKWMWYRKYYYKKYKKRYKMKKTYKKYG
           *******  . *.****  * .*   *. *** *** **** ********. ****.

48      YYYAKTGWKYKCKYCRGWKKKYRKRMYKKKGWMYKYYRKRKKSKYYYGTCGAGRYRRMWR
51      CCYGKGAAKYKYGYYGAWTKKYRGRMYKKKRAMYKYCGGRKKSKYYTKYYARKRYRRMWR
          *.* . ***  *  .*.**** ******  ****   *******    .  *******

48      YAAYYMYRRKTGMWYKYKKKWRKKYYGYYRYYYSMKRYTCKSRKYAGTMRYCGACYKRWK
51      CGTTYMYRRKYKMWYGYKKKTRKKYYSYYRCYYSMKRYYYKSRKTRRYMRCMARMYKRWK
         .: ******  *** **** *****.*** *******  ****    **  .  *****

48      KMWKKKGKWARYKRKRCYSYWYKYYYYARMKYWYRKWKKRWMYWWMAAAARRYKYWYYRY
51      KMWKKTKKAGRYKRGGYYSYWYGYCTCRRMKYTYRKWKTRWMYWWMMRGCGRYKYWYYAT
        *****. * .****   ***** *    **** *****.*******  .. *******

48      RYKCMKRKKTCAKYYRCKKYKRRATYGTKCMKRYRYRYYTMKKYSKKMKCRTTGAKKYRR
51      GYTTMKRKGYYWKYYRMKKYKRGRYCKAGGMKRYRYRYYAMKKYSKTMKYRCCCTKKYGG
         *. ****    **** *****     :  *********:******.** *   :***

48      RRMYRRKKYRGGYKKKKRSMYWRRKYRYWKRMKRRYTYKYCYKKTSYYRTTTTYRYKYKM
51      ARMYRRGKYRRRYKKKKGGAYARRKYRYWKRMGRRYKYKYSYKKWSCCACCAYYRYKYKM
         ***** ***  ***** . * ********** ***.***.*** *     : *******

48      YAKKYWSRWYTCAGCCKKKKRTWRKKKKRRKSYYSYWRYYWSKRRYKWKWKKKMKTARMM
51      YMKKYWSRWYCTGRTYKGKKRYWRKKKKAGTSYYSYWRYYWSKRRYKWKWKKKMKCGRMM
        * ********  .   * *** ******  .************************ .***

48      WGKAACATKKWWYSRCWYMACTGRRWCTWWMKKKWWGTTKWKKKYYYKRCRRRRMRYTCW
51      WKKTGMRAKKWWYSRYWYMMYCTARWAKWWMTKKWWRGCKWKKKYYYKRYRRRRMRYATT
```

```
        * *:.  :******* ***     **..***.****   ********** *******:

48      KMMYRWTKAGCCGTAKMRRYGCGWMWKRRAYYKRYRMMKRRWRYKGTATRGWARKYCYCY
51      KMMYRWKKGAYMTWRKMRRYTGCWMWKRRMYYKAYRMCGAAWRYKKYGCARWWRKCTTAC
        ******.*..     *****   ****** *** ***    ****  .   * **   .

48      TGKSCACRRKWYYRTMYYKYKMMKRYYYWYMKKMRKKTTACRRKKAAWTCACMMGKKRWK
51      CAKSYWYRRKWYYRKMYYKYKMMKRYYYWYMKKMRKKWYRYRAKKRMAYYTAAATKKRWK
         .**   *******.**********************    * **     :.   *****

48      RRYMYGCYRTMYYYRTGRRYRTCTCTATYRRKKWRKKYWKATGKKYCYYWMYYMRRRYAG
51      RGYMTKYCRCMYYYRYRRGTGWYYMKMKYRRKKWRKKYWKRWKKKYYYYWMYYMRRRYTT
        * **    * *****  *       . .************   *** ***********:

48      KMMKKYYRYKKKCCACKKAKKMWWMKYRKKKYRKRTKYYYKKYKRKYYWKGTGTATGARK
51      KMMKKCYRYKKKTAMYKKRKKMWWMKYRKKKYRKRYKYYYKKYKRKYYWKRCTCRARGRK
        ***** ****** .  ** **************** **************     : .**

48      YYYWKRYKWKRKMWCTKKRRYRKKWACRWWAAMRKMKWRYSGGTKKYTYYACTYRRAGCW
51      YYYWKRCTWGRKMTYGKKRRYRKKWGTRATMWMRKMKTGYSAACKKYKYYGAGTGRMKMW
        ****** .* ***   *********. *    *****  **.. ***.**..   *   *

48      GYRMYGYYKMKRYKYWTYAMWYYRKSWKKKWKMGMRRGWTKKMKGCCGGYKMKMRTATGC
51      KYRMYKYYKMKRYKTTKCCATCTRKSWKGKTKMKMRRRWYKGMKRTTAKYKMKCGAGGRM
         **** ********  . .    ***** * ** *** * * **   . ****  :.

48      YKMRKKMGCACACCGAKKRMWMAGMWGCGGRYYRYRKYMCCYKKTAKRAKRKRGGCGYKY
51      YTMRKKMKARYGTTARKKRCTMTAMAATAARYYRYRKYMYSYKKWGKGCKRKRAAYKYKY
        *.***** .  .  . ***  *:.* . ..********* .*** .* .****..  ***

48      RKKWKRYRKKRKRKMAKKMRRRRYKKRKRKRYYKKRSSMGTTRWKKWRAAYKKKWSWKRM
51      RKKWKRYRKKRKGKCWKGMRRRRYKKRKRKAYYTKRSSMKYCGWKKWRGRCKKGWSWKRM
        ************ *  * ************ **.*****    *****.  ** ******

48      GACCYWWKRSKYWRRYKASMMKKYMKYGTCTTRYKMYWMMRRYRMKWRKYKRYKRR
51      RTGACWTGGCKYAAAYKRSMMKKYMKTSKYWWGCKMYWMMRRYRMKWRKYKRYKRR
         : . *   .**   ** ******** ..     **********************
```

The pairwise alignments of two accessions 49 and 50

```
49                  YYSKWKRWYKYYGKYTTWMRYYMKYYRRMMKKSRKYKYMWRYYTYRKMYYGMWYKRYKYK
50                  YYSKAGRWYKYYRKCYYWMRYYMKYYRRMMKKSRTYKYMWRYYGCRKMYYAMWYKRTTCK
                    ****  ****** *   *****************.********  *****.***** . *

49                  SYGRRRWWAMKMMYMKRMKYYWCARYYWRYYYYWRYWRYRWRSRRYKTWYWWYCYYTGYY
50                  SYRRRRWWWMKMMYMTACKYYWYRRYTWRYYCYWRYWRYRWRSRRYGYWYWWYMYYCCYY
                    ** ***** ******.  ****  ** **** **************  ***** **  **

49                  MWYMARRRWTRMKMRCYYYKRRWMWRWWRMRRGTYYTAMKRCCYGMTARTCRYYYRKGAS
50                  MAYMTGARTKGMGCAMYYYTRRWMWRWWRMRGRWYTWTMKRTTYTMYWAYMRYYYRKTWS
                    * **:  * . *    ***.***********   *  :***  * *     ******  *

49                  KRKYMTRKSYRYTMKWMCCRRMKRYKYYWKMRCWMRAKCYYKGYKYYGWYRKYKSRYRMY
50                  KRKYMAAKSYRCYMKWMTARRMKRCKYYWKMRTAMGTGTYYTKYKYTRWYRKYKSRYGCY
                    *****: ****  **** .***** *******  * :  **. ***  *********  *

49                  YRYWAMYRRWYMRYYWRKKYKTTTCSKWRRKRYRKWRYTTRMWWSYCTTWYCTRMYTCWY
50                  YGTAGAYRRWYMRYYWRGKYGYCCTSKWRRKATRKWRYYWRMWWSYYYWACTYRMYAYTY
                    *   . *********** **     ******  *****  ******       ***:  *

49                  YYKMTAGCRWWKYRWYYWWMYRMRRRAAKYRYGRYWAYKYWWRYMRKYWWWMRMWYSRYY
50                  YYKMGGATAAAGTRWYCTWMYRCRARTCKYRYTRYWTYKYWWAYMRKYWWWMRMWYSRYY
                    ****  ..      ***  **** * *:.**** ***:***** *****************

49                  YYWMTMGGYYWKAWRYRKRKYWYGYCCCMRYYRMKRAYRMGGTRMMWRKGKYMKCWTARK
50                  YYWMYMRKYYAKRWRYRKRKYWYAYTTTMRYYRMTRGTRMSRYRAMWRKRKYMKMAYRGT
                    **** *  ** * **********.*   ******.*. **.  * **** ****     .

49                  RMYWRTYRRKKKTCWYKYRRYKTKRRWYWRKKGRTARKKCRCGGCTMSMMMYTMYRARAY
50                  GMYWRKYRRKGKYYWYGCAGYKCTRATYWRKKAGYMGGKYGTSRYYMSMMMYWMYGGAGC
                     ****.**** *  **    ** .*  *****.     *   .   ****** ** . .

49                  AYYWMKGMKYKKMRKRAAKRGTAKCKMKKMKSRRYCWCWAKRKKKWKYYKRWWKYYRYRK
50                  GCYWMKKMKYKKMRKRWWKRAAGGTKCTKMKSRACAWTTTKRKKKWKYYKGWWGYYRYRK
                    . **** *********  **.:.  * .*****  .*  :********** ** ******

49                  RTTKGGKYMRRKKKGAGGCSTTASAYRKKKKKYYRKTMKYKKACYKYAWWYSTCTYYYYT
50                  RYWGKRKYMRRKKKKCTATCYCTSGYRKKGGTYYRKYMKYKKRYYKCRWWYSWYGYYYYC
                    *     ******** . . .  :*.****  .**** *****  **  ****   ****

49                  WKACAKKYKSGMTYWYRRWRKTKCKRWGTTYMWKWKYRSYYKYKKRMKAATYWWMRYKAT
50                  ATTYRTKYKSKMCYWYRRWRKKKTGAWKYWYMWKWKYRSYYKCKKAAKTTGYWWMRYGRK
                     .:  .**** * ********.*   *   ************ **  *:: ******  .

49                  TGYCCTRWRSKWYRRKYKYTGKWCGGMWSTKYYRCAAYKKRMMAKYMMRYYRYRYKKKAM
50                  CAYTAGRARGTTYRRKYKYCKKWYRRMTSAKYYRMTTYKKGMMTKYMMRYTRYRYKGKRM
                     .* . * *.. *******  **   * *:**** ::*** **:****** ***** * *
```

```
49          YWMGTGRYYKRKYRCRRKKCYKSRYYYMKYRKKYRYRYKYRRKKMMYRMKMKRKRRRTKY
50          YWMSYKRYYKRKYRTRRKKTCGSRYYYMKYGGKYRYRYGYRRKKMAYRMGMKRKRRRCKY
            ***.  ******** ****   ********  ****** ****** *** ******* **

49          RKKYWMMKYAATKMTMMKAYTKARYSMKKYMRRKKMYYYRRKRYYMCRKYMWRMGRGYYW
50          RKKYWMMGYWMKKAKMMGMYCKWRYSMKKYMGGGKAYCYRRKATTCYGKYMWACKRKYYW
            ******* *  .* .**  * * ********   * * ****      ****   * ***

49          YYSWWRKKKATGMKTYYYGYKWYRKYSYGAATYKKTCTKKWYYYCYYKTTCAMKKRWRMK
50          YYSWWGKKKGGAMKKYYYKYTWYRKYSYKGTCYKKYYWKKWYYYYYYKCGAGCKKRWRMK
            ***** ***. .**.*** *.******* .: ***   ****** ***  .. *******

49          YYYYCYKYRYCYKYWCTACWCMYKWRWRRRGTYCKYYKKRMWSMRYYWKKYKKYRCAMTG
50          YYYYAYKTRCTYKYWTCRMTYAYKWAAARRSKCMKYYKKRMWSMRYYWKKYKKYRYGACA
            ****.** *  ****       ***   **..  ********************* .  .

49          YRYMKRYKWSKKKTRRMKKMKRYYKSMKAKGSAMTKWKKRKYWYYGWKYYRRKRKKTGTK
50          TRYMKRYKWSKKKKRGMKGMKRYYKSMKRKRSRCCKWKKRKYWTYKWKYYRRKRKKKKK
             ************.* ** ********* * *   ******** * **********. .*

49          CYRWKKSCGYAYKWWKRMRYRYRRYWYCTAAYKRWMCTAMSYSWRRRKKGRKKYKKYRYR
50          YYRWKKSMKYRYKWWKRMRYRYRRYWYMCMRYKRAMTWTMSCCARRRKKTGKGYGKYRYR
             ******  * ****************    *** *  :** . *****  * * *****

49          KKMKKRRKKKARYGTKMKKRWGKKKYMYYYKMYYRKKRCGCKRYYSSWMMKKYKKGYRKM
50          KKMKKRRGKKWRYSKKMKKAAAKKKYMYYYKACCRKKRMSYKRYYSSWMATKYKKRYGKM
            ******* ** **..****  .*********   **** . ******** .**** * **

49          MRKYTMYWKWMKCWMTTAYCTKYKYRMKATAYRKWCTRRKKKYYYRRKKYKYYRKWKRYM
50          MRKCACTWKWMKMTCCAWYTCKYKYRMKTCWYRKWAWGRKKKYYYRRKKTGCTAKWKRYM
            *** :  *****    : *  *******:  ****.  ***********     ******

49          MMRSTRYWCRYMGATAYTCYRAYGGCKGGWYRKCTGRKGKMKTKWKCKKWWKKMKRWTKT
50          MMGGYACTMAYMAGYWYCAYRRCKRTKKTWYRTMKTRKKKMKKKAGAKKWWKKMKRWKKK
            ** .      **..  * .**     *  ***. . ** ***.*  .**********.*.

49          KMTYRYKKKRYRYKKYKMRKAGAYKMMRWTMYWTGCRAGKRRAYRYTTYRWKWYCGRRRR
50          KAKTAYKKKRYRYKKYKMRTWRWYKMMRWWMYWWKYRGTKRRGTRTKGYRWKWTTARRRR
            * .  **************.   ****** ***   *. ***. * . *****  .****

49          YCAYWGAACGWGCTGGYRKYYRCTRRKWYRRMYGTAMCMGTAGRYKAATGYWYRGTKYGC
50          YYRYWRWRYRWRMYRRCGKCYRYKRRKWCAGMYRCCAYAACGAAYKRRYRYWYRKYKYTM
            *  **     *       * ** .****   **  .   . .. **    ****  **

49          CWMYRMGTGRCTKWGYTYAMGASGWSTMARTRKYRATKAACACTWRWRYYYKCYYWYCKM
50          AWMYRMRWRRYWKWKYWYGAAGSSWSCARGYRKYRRWKWMYMYKWRWRYYYKTYYWYTKM
            .*****   *  ** * *. ..*.**     ****  *     .******** **** **

49          RRTKRRYWRWRRRYSYKYYRKYMYKTGCCTCKYMSWRRWATWKTCYYCMYYKGGCKRTCK
50          RRGKGRYWRWRRRCSYKYYRKYMYTCTAYAYKYMSWRRWMYWKCYCTYMYYGRKYKRCTK
            ** * ******** **********.  . : ********  **     ***    **  *

49          KKTKYWKMKKYGAYYKYGRYYMTCYMRYTKKRTYKKYGMMKGSKKTGYWMYKSCGKYTKM
```

```
50          KGKKYWKAKKYKTYYKYKRYYMWTYMRYKKKRCYKKTRMMGTSKKYKYWMYKSTTKYKKM
            * .**** *** :**** ****  ****.*** ***  **  ***  ******  **.**

49          RKYMKRWKKTKGAMARYRRAGYRSKKMSTTYKMKCRKKRWWSYRMGYRYYRKYKRKTWYK
50          RKYMKRWKKGKRMMWRYRRCTTRSKKMGYCYKMKAAKKRWWSYGMTTRYYRKYKAKCWYK
            ********* *  * ****.  *****.  ****. ******* *  ******* * ***

49          KTTRWTGCTTTGAYMKTMWYYRYKAATTYRYWRMKRRKWRSYKRCKYGACGAYRYMGKCA
50          GCCRACATYYKRGYMKKMWCCRTKCGCKYRTTAMKRRKWRSYKGGKCKTTATYRYMRKYR
               *  .   . .***.**  * *.. .**   **********  *  : .:**** *

49          WTGGRGRYRYRSKKMRWYGWYYGKKKMMYRKACTTMKYKAATTAARAKGWGKKYGYCYSY
50          WYKRGRGCRCRSKKMRWYKWYCCKTGCCCRKGMGCATCKGTAAGGRRKRWTKKYKYMYCC
            *       * ******** **  *.    **.    . *.:::..* * * *** * *.

49          RYRKKYKYYKRKKYWRMKRCKMRRRKRYRKKRYKRYCWKMKYWMYKACCCKRMYKMYRRK
50          RYRKKYKYYKRKKYWRMKRYKMRRATGTRKGAYKRYMWKMKYWMYKTYYAKRMYKMYRRK
            ******************* **** .  **  **** *********:  .**********

49          RKMWKRSAYKKYYKRYRRKKYGGGYWYYRMRGWRCRSYWWWYYRKWWKMRYMAATMMRKK
50          RKMWKRGTYKKYYKRYRRKKYATKYWYYRMRATGMRSYWWWYTRKWWKMRYMGTCMMRKK
            ******.:*************.  *******.   ******* *********.: *****

49          MCRCMKKKYGCCCTAKWKKRYRYSKRKWYWKGYWKKKWYSYWKKKKMTKKYRMKMKMRKR
50          CYRAMKKTYKAYMAWKWKKRYRYSKAKWYWTAYWKKKTYSYWKKKKMKKGYRMKMKAGGA
              *.***.* .  : ********** ****..***** *********.* ******

49          MCYYKTRMRYYKYRYYRARMYRRRYCYRYYKYRKRMRYSYWKYRYKRKAYKKKKRKYRYR
50          MTYCGWGMRYYKYRYYRGAAYRRRYYYRYYKYRKRMRYSYWKYRYKRKRYGKKKRKYRYR
            * *    **********.  ***** ********************** * *********

49          YYRKGYYKKRKKRYRWKGTCKKMKKRRYKYCCTRAAACCCKRYMRKKKKRWRYKYRKMGG
50          YYRKAYYKKRKKATGWKRYYKGAKKRRYKCYMYRRWRYMYKRYMRKKKKRWRYKYRKMTK
            ****.*******   **   *  ******    *      ******************

49          RWWKYKMWRYRWKCMTRYRYCKKKWYMRRYCYCAGRYMTKGCSKMWYSRKSKCKRTWAKC
50          RWWKYKMWRYRWKTCAAYRYAKKKTCMRGYACTCRRYMCKRYSKMWCCAKSKYKRCWTKT
            *************  : ***.***  ** *.  . *** *  **** . *** ** *:*

49          TKYMYATAKYYYMSKRKYKRMMWRMRKKKKRCRARAMKGGACKWSYWKRKMKKKRYRYKR
50          AKYMYRKRKYYCCCKGGYKRCAAGMRKKKKRYRRRMMKRRGTKWSYWKRKAKKKRYRYKR
            :**** . ***  .*  ***    ******* * * **  . ******** *********

49          KTASAMMMKKKYKYYRMYRRYMKTGCTAKKRKWYMRYYYRTGRYKKRSMRRKRWRTYSWT
50          KWWGGCMMKKKYKYYRMYRRYMKYSMWRKKRKTTCRYYYRYRGYKKRSMRRKRWRCTGWW
            *  .. ***************** .   ****   *****   ************  .*

49          TKYMAWTKKRMYATYTYYYCCMYRYRYRKTKKKKACTGYRYGYCKMYYRYYYTTRAYYYY
50          YKYMGTCKGRMTWYTWYYYTAATRYRYRKAKKKKRSCCYRYATTKMYYRTYYCYAGYYYY
             ***.  * **     *** .  ******:**** .  ***.  ***** **   .****

49          ARKAMKYAYGGWWRCGTRRYRRYMMYRRYYTAMYYKTKYWYWCRMMRYWRKKMYYWWRSR
50          WATTAGYRCRRWWRMKWRRTRAYMMYRRYYCGMYYTKKCTYWARAMRYWRKKMYYWWRSR
```

```
            .:  *    ***   ** * ******** .***..*  **.* ***************

49        KKRRRSRGCRCKCRCRYCTKWKTKWRKYYWRWKGKWRKWKKRAWRWMGKGWKMKWWYMKS
50        GKRRRSRAMRGGSRYRYACKWKKTWAGCTWRWGRKWRGWKKRGAATMCKCWKMKWWYMKS
           ******. *  .* **. ***..*    ***  *** ****.   * * **********

49        WYRYRKSMYRRTGKKKATTYWMYCTAYKKKYKKKKKWWRRRRGRTTTKGKRKKKKWYKWK
50        WYRYRKCMYRRYRKKTGCYYWACYKWYKKKYKKKKKAWRRRRRRCGGKRKRKKKKWYKWT
          ******.****  **..  **   . ********** ***** *   * **********.

49        YRWKMRRRWYYYMRYRRMKSWRMYRCKKRWMYYKKACACKRRMKWYKKKAMKRKCAAATA
50        TAWKARRRWYYYMRYRRMKGWRMYRTKKRWMYYKKTTGAKRRMKWYKKKWMKRTTTCCAT
            ** **************.***** *********: ..********** ***. :..::

49        YRYYKWYRSSMYGRYWKKTRAAKRTTMRYYRYKYRRRYYRWRYRGTRRMYKKKRRYYKKY
50        YRYYKWYRSSMYKRTWKKAATGKAYYMRYYGCTCAGRTTGWRYRRYRRMYKKKRRYYKKY
          ************ * ***: :.*   ****  .   *   ****  **************

49        KMCAYYSRRYGCTTKSYKRWSRWRKMRWMKKRRCGKKYSGRKKKYYYYKYRSYKMRRKMK
50        KMSMYYSRRYRMYYKSYKRWSRWRKMRTAKKGRTRKKYSRRKKKYYYYKYRSYKMGGKMK
          **. ******    *************  ** *  **** ***************  ***

49        TCRRRGAYYKKRWSMMKKKYRYSRCCGGRRKTCKKKACRYKYRATTACKYAYYKRYYRMK
50        CARRRTGYYKKRWSMMKKKYRYSRTTKRRRKKYKKKGYRYKYRGCCCTKYRYYKRYYRMK
           .*** .*****************    ***. ***. *****.  . ** *********

49        MRWACTCYRYSKKMKKRWKMKKKKKMYYWYWSRMYRMRKWKWKRKGGTCYGMKSMRCKKK
50        MAATAAAYRYSKKMKKRWKMKKKKKMYYWYWSGACAMRKWKWKRKAACYYRMKSMGYGKK
          *  :.:.*************************    *********..  * ****   **

49        GMKWRYRTKYKYYKYYSYYRRYGYGKAATKKKGMGRRYKYKCCKKYKRKKRRKYMRSYWY
50        TMKWRYGYKTTTCKYYSYYRAYRTTGTCGKKKRCRRRYKYKAATKYKRKKRRKYMRSYWY
           *****  * .  ******* *    :. ***   ******...****************

49        YTGTCMRRMYKWRRWGYKYGTYRKKRMRTKYSWWRMKYRARKYRYMRRKGTYYAATAYAM
50        YWRYTCGRMYKARRWRYTYKYTAGKRMRKKYSWWRMKCRRRKYRYMRRKRYYYRRYRYMM
          *      **** *** *.*     ****.******** * *********  **    * *

49        KWMCYYKYRKKKWWCCCGKRYYGWMWK
50        KWMYYYKYRKKKWWYYYRGRYYRWMWK
          *** **********     *** ****
```

The pairwise alignments of two accessions 49 and 51

```
49                YRYYKKYWMRTYYYTWRYRWWKKGGCWKYYWRYRKCRYWCGGWMASRKRAWRRGYYYYYY
51                YRTYKGCWMAAYTTCWRYRWWKKRRYTKYYWRYRKYRTWTKKWCWGAKRWWRRAYTTTCT
                  ** **  ** :*   ********    ******** * *   *   .** ***.*

49                MCCCKYWAMYMGYRWYTSKYRWRWMKATTWYKSYGYGGAMMRYWMWMMCCTAWKWWKWRY
51                AATTKYWRMYMSYRWYYSKYRWRWMGWGCACKCTKYRKGMMRYWMWMMAMKGWKWWTWRY
                   .  *** ***.**** ********      *.  *  .*********. ..****.***

49                MKMYWRAWGATRRRAWWRKMYKMYKRKRYKKSWMYYWRMMYRYKARWWYYYMYKYWKWGM
51                MGMYWRWWAGCARGGWWRKMYKMYKRKRYKKSWMCYWRMCYAYKWRWWYTCMYTYWKAKM
                  * **** *..  * .******************* **** * ** ****  **.***  *

49                KSMRCGRCKRWYKKWCTGKYKSRKYYYWKRYWAGATMKYMRYKWYKMKKTYYGYWGRGYR
51                KSMRMRAMGRWYKKWMYRKYKSRKYYYWKGTATTCCMTYMRYKWYKMKKCYYRCWRRAYR
                  ****     ******   ***********   : . *.*********** **  * *.**

49                WKCYRRWWCATWYKAMWKYKYSRRKRTKRRKWGRYCYRKWYKWWCAYYRRAYYMWRRTYR
51                WKYYRRATTGCWYKTMWKYKYSRRGRCKRRKWTRYYYRKWYKWWMRYYRRWYYMWRRYTR
                  ** ***   . ***:********* * ***** ** ********  **** ******  *

49                AKKYKYYMMYKWKMKRCYYYSRYGCKYRKRMKMMKKYYMYTYMWTRCRWRKYYRYRKYKW
51                RTKYKYYMMTKWKMKRTYYYSRYKYKYATGMKMMKKYYMTWYMWGGTRWRKYYRYRKYKW
                   .******* ****** ******  ** . *********  ***   *************

49                YYRYYWTYMRRTTCTTTTGCYCKTWSWMMKKYKKAKKSKWGGCMARRYRYGRCGCYRKKM
51                YYRYYWYCMRGACACAGCAYCTKWWSWMMKKCGGGKKGKWRRYCCRRYRYKGTKTTAKKM
                  ******  ** : . :  .   * *******   .**.**    .*****       ***

49                KKKWRWYKKKRMKRKMGKKKRYYKRRKYSKKYKYMRKRKMKKMGRRKMRWKRMYKGYCGY
51                KKKWRWCKKKRMKGKMKTKKRYYKAAKYSKKCKYMRGRKMKKMRARKMRWKGMYKRTYTY
                  ****** ****** ** .******  ***** **** ******  ****** ***    *

49                RRKSWTMYKKGTKKYMAKRYAYMMRYWKMRKMTMKRRRYYWRKYWWYYKRKYKKKRRRGS
51                RRKSWYMYKKTCKKYCCGRYGCCMRYWKMRKAYMKRRRYYWAKYWWYYKRKTKKKRRRRS
                  ***** ****  ***  . **.  ********  ******** ********* ****** *

49                RKKARMYRRYTGTKKTWKYYCYYKWCKRWAKMWMKWGYYWKKCCTAAKKKKKWWKKYTMS
51                RKKTRMYRRYCKYKKWWKYTGYCTWYKRWCTMWMKWKTTAKKTGGCRKKKGKWWKKYKMS
                  ***:******   ** ***  * .* ***..*****    **   . *** ******.**

49                KWRRMWACYRYKKYWMRRYAAACKWSKGKKKKKWWCWRRKKRKKKKRYRKKGMKKKTWKY
51                KWARMATYYRCKKYWARRYGWGTGWSKRKKKKKWTYWRRKKRKKKKRYRKKKMKKKCWTY
                  ** ** : ** **** ***. .  *** ******  *************** **** *.*

49                WKKGRRWYYYYACCMRKYRKMRYGRYRYAKMYRRSRWKMYYKSKYYCCKRMKWSKKWKMG
51                WKKKRRWYYYCGTTMRKTGKMRYRRCAYWGMYRRSRAKMYYKSGYTATKRMKWSKKWKMK
                  *** ****** .  ***  **** *  *  ****** ****** * . ***********
```

```
49     AYYRYKRRRGMYYRYKYKYTKRRYSKYWGRYYRYGCKKRAGSGAGKYYRKYRKMMYKRKG
51     GYYRYKRGGRCYYRYKYKCYKRRYSKYWKRYTAYKTKKRWACTWRKKYYRKYRKCMYKRKT
       .******    *******  ******** **  *  *** ..   ******** *****

49     KKYGCYKMKGSYYGGRKRYKYAMGRKRWRYRRYKYRAMYWYKMWYGACSAYWKYYAKYRY
51     KKYRMYGAKKCYYAAAKRYKYGMARKRWRYRRYKYGMMYWYKMWCACAGRCAKYYMKYRY
       ***  *  * .**.. *****.*.***********  ******* ....   *** ****

49     YMRYRMRGRAAYSKYKSKYSGRAKKASKYKRAKYMYYKYYMSYYMCAKGRRKGYTARKYW
51     YMRYRMRAGCGYSKYKSKCCRARKGRSKYKRMKYMYYKYCCSYYCYMGRARKACCGRKCA
       *******. ..******* .   *  ***** *******  ***      **.  .**

49     TRGCGKKKSKYKKYMRCTRKKACKATATKKKKRCWKKWYMYRKMWKRYCTKMKKKAATYM
51     YGRMRKKKSKYKKCCRYWRKKGTKTATCGKKGGMWKKATMYRKCTKRYMWKMKKKWRYYM
            ********  *  ***. *:::  **   ***  ****  ***  *****   **

49     YRRRYYRMKKRKKYYKKKKYRRAMRRKYMRWYRRTKTTCCYWKYYGYRKTMWKYYYMRRY
51     YRRRYYRMKKRKKYYKKGKYRGRMRRKTMRWCRGCGYWMTYWKTYAYRTKMWKYYYMRAY
       ***************** ***  **** *** *       *** *.**..******** *

49     GYCRRTTATGSYKKYYWGACTRYCAWYTRRSKCTRKWCTTRKKYSMKKKAKKAGCRKGWT
51     RTYARKAGWRGTKKYYWRMSGRYYRWYARRSKMYRKWGYYRKKCSMKTGWKKTTARGAWG
           *.:.  . *****  . **  **:****  ***   *** ***.  **: .* .*

49     GWASYGYSMGKYYKWRRKKYRKRYMMRRWYKAACTGGKMAMWCTRRTGRATRSKYKASCT
51     AWGGCACSAAKYYKWRRKKYRKRYMMGRWYKTCGWKTKMGCWTGAAAKGMWGSGYKWSSA
       .*.. . * .**************** ****:.    **. *    :     * ** *.:

49     CKYRWYRYKKYKYAWWMCKWRCAYKWWKAAGTATRWRKMMYYTSKGKAAYYKYAKMRKAW
51     MKYRWYRYKKYKCRWWMTKWGATCKWWKRWRYRWRWRKMAYYYSKKKWTYYKYCKMRTGW
        ***********  *** **  .: ****      ***** ** ** * :****.***..*

49     YRATCTKKWRRYYRAGRACCYTCGKYAGYYTTRRYYYAGWWAAMYRKRRAATCYRRMMYR
51     TRTKYWKGWRRYYRRKRGTSYATTKYGTCTAGGRYTYGRATMWMYRKRRWWCYTGGMMYR
        *:.  * ******  *. .*:  **.   :  ** *.     ******       ****

49     AKMWWAGTRRRKKGGYKMWCYYGYRTYRWKKAGYCMMYMYWYYYSSKWYRRKKMTYYGCA
51     GKCTWGTYAGAKGRKCGCTTCCRTGACAWKGGAYYAAYMYWYYYSGTTYRRKKCKYYRTW
       .*  *.     *             :  ** ..*   ********.. ***** .**

49     CGTYKGCGTTTGCAAAWYYSYGKRYWKKRGYARWKMRYWCRWRYRCMAAACKTKRWWMRG
51     MRKYKAAKYCCATGGWAYYSYKKRYWKKRKYRAWKMRYTYGWGCRYMGTWTKAKGAACAT
         .**..    . ..  **** ******* *  *****   *  * *.:  *:*

49     CGRTAKCWCYRRAGCTATACKMGMWYRYAKWYKGSYRWRWRGYAGTTCACGKAAAGKKKR
51     GCRYRKMTATAGWRMWGCWYKMKMWYRYRGWYKKSYRWRWRKYTACYYMMKGGWCRKGKR
         *  *  .       .   ** *****  *** ******* *:.       . . * **

49     YRRRKKMKMKKMKRKRRKKMMRKWWMMRKSKRKMKRYSMYKYRRWKYKKWCCAAYYMAYT
51     YRRRKKMKMKKMKRKRRKKAAGKWWMMRKSKRKCTRYSMYKYRRWKYKTTAYRGYYMWCC
       *******************   *********** .*************. .  .***

49     RYAYMWGRMCAWYYCGTAKYTYYWRRGKMRYATYTMGRKAGATKRWKKYYSYYYRKKYRY
```

```
51          GCRYMWAACYGWYYAAAWKYKYYWRRAGARYCKCYMKGGCACKKRWKKYYSYCYRKGYRY
               ***.   .***..: **.*****.  **..  *   ....********* *** ***

49          YYYYKKKRKMMRRKKYYRYKKRRYKWKMWWTAAKATARMYTRYRCGRCGKSRTMYMKYKS
51          YYYYKKKRKCMRRKKTYRYKKRRYKWKMWWGGGKRKMRMCKRYRYRRYSKCGAMYMKYKS
            ********* ***** ************** ..* . ** .***  * .*. :*******

49          WRWCTCGGTAAYMRYAYRWYYRMRMYKKKAGYRYRYRMTCKYYTTMGMMKKKRMAGCCGR
51          WGTTATCACCCYMRYMYRWYYRMRMYKKKWKCRYRYRMYYKYYAAMKMMKKKRMGAYMTA
            *   :  . ..**** *************   ******  ***::* *******..

49          GRYGTGCYTTMRRTWGAGTTWSRWRMRKKRWRKKATYCKRWRRYRYCKKKMYWYMRRTMK
51          KRYCCATYCACGRWWKRRYYWSRARMRKKRAGGGRWYMKRWRGTGYYGKKMYWYMRRGAK
             **  . * :  * *     *** ******      * ****   *  *********  *

49          KMRRRYYWYYKKYYRKYYMYRYRYGAWKRRKRWKKGRYRKWKKYYWMKCAARRTAAKYSC
51          KMRRRYYACYKKYYRKYYCCGTATTTWKRRTRTKKKRYRKWGKYTWMGMGRRRGGGKYCT
            *******  *********       :****.* ** ***** ** **  . ** ..**.

49          CTSMRTMKYYRSTKKYKYCKWWYSAYYMWMYYGYYWWYKKTTAYCYWYRRWTKKYKRYRS
51          YYSMRCMKYYRSWKGYKCYKWWYSMYCAAACYTYYWWYKTACGCYYWYRRWGTKCKRYRS
              *** ****** * **  ***** *     * ******.: .  ****** .* *****

49          YYKWYYRKKTTTAGYTRRYWWAYAKKYSKRCTCACTSGACMCCYRMKKRMMRMKTTGSCA
51          YYKAYYRKKKYYMTCGRRYAAWYGGGYSKRACGTYKSSCTMTTYRMTKRMMRMKACAGTC
            *** *****.      ***   *.  ****.  : .*.. *  ***.*******: .. .

49          GMKKKKKMKAKKRKYKYRRYGRKCMRATWRRWYRYAYRRWKKMYSMMMYRYKKRWYMYYR
51          TMKKKKKMKRKKRKYKYRRYKRGYCRRYWRRWYRCGYRRWKKMYSMMMYRYKKGWYMYYR
             ******** ********** *   *  ****** .***************** ******

49          MKKKMRKRRKSKMKSRYRWKRCAGRKMYYWTYYWKRKKYYKKARMKYKKRTYYCYCCCKW
51          MKKKMRKRRKSKCTSRYGWKATGTRKMYYWKYYTKRTKYYKKWRMKYKKRWTYYYATAKW
            ************ .*** **  . ******.** **.***** *******  * *. .**

49          RWRRRRKYKRKKWSCMRYYWKKYKRKYRRYYRYMSKKKKKKSMKRYMYKKYRYYSMKAAK
51          AAAARAKYKRKKWSYMRYYWKKYKRKYRRYYRYMSKGKKKKSMKRYMTKKTRYYSMKMRK
                * ******** ********************* ********** ** ******  *

49          MMRWKRKKYYASYKRWSRMYKAYYWRKRKKKMKMTYRWKKRKSKACKWAMSWCAAATAYR
51          MMRWGRKKYYRSYKRWSRMYKMYYWAGRKKKMKMKYRWKKRKSKTTKWRCCWTTCCATYR
            **** ***** ********** ***  *******.*********: **  .* :..::**

49          YYWYRSSKARYWKKWYKRTYTKMGMYRYYRYKRRYRKRGCTCMYKKGYYKYWKTCCMYYK
51          YYWYRSSTMRYWKKWCKACTCKCKMTRYYRYKRRYGKRRMYMMYKKRYYKTAKATTCCCT
            *******. ****** *    *  * ********* **    **** ***  *:     .

49          MMCMRYGAKKRAGKWRRRRYMRKRACWCKYYKKYYKYKYMKKMTCGKRRRRGKKRYMRYY
51          MMYARYRRKKRRKKWRRRRYMRKRGMAYKYYTKYYKYTYMKGMCAKKGGRRRKKRYMRYY
            **  **  ***  ***********.   ***.*****.*** * . *  ** ********

49          YWYWKKRKYKTTRKCKKGKAYKKRKSYWYYTMRRMKWRMWWSWKKAGACRRSMKAGATKK
51          YAYWKKRKYKGARKYKKRGRYKKRKSYWYYYCGRMGARAAWSWKKTCGYGRSMKRRRKKT
```

```
            * ******** :** **   **********   **  *  *****: .  ****   .*.

49          RYSWRYYMGKKSRKKWAMMRYYKYKWYKKTCKKKKYMYWKKRYRSYWCWSMWKAYKYKAT
51          RTGAAYYMRKKSRKKWGMMRYYKYKWYKKWYKKKKYMYWKKRYRSYWTACMWKGYKCKWY
            * .  *** *******.************  ***************   .***.** *

49          TAGMRYRRKSKKCTMWRKGSRYKMGCSYRKCTWWSTCYCMCMYRSKWYRGKKKTKKRKTM
51          YMRARYRRKSKKYGATGKKGRYKMKMGCGKYYWWSKYYYMMMYRSKWYRKKKKYKKRKAC
                ********     * .****  .  *  ***. * * ******** *** ****:

49          AYRYMKYGCKRWCRGRGGRYRYKMKMRWCATGCMSRTMKYYYTCCATWYRYRKGTCYYYK
51          MYRYCKYATKAWYRTRRRRYRYKMKCGAMRKSMMSRKMKYYYCGTGCTYRYRKKCTCCYK
             *** **. * * * *  *******     .. ***.*****   .  *****     **

49          RKKRRKKRMYRWRKTYMYYYKKMYRRMKKRYYKKSSAKYYMMKYKKYKRKKMMRRKWRTK
51          RKKRRKKRMCAARKWYMYYYKKACRRMKKRYYKKSSWTYYMMKTKKYKRKKMMRRKWRWK
            *********   ** *******  ************ .***** ************** *

49          TGKRMKKRRYCTKMMYRMKYRKWRKATKKKYWWCKKRKYCKYGWKCRMGCGRYYYYYSAM
51          WRKGMKKRGYTCKMAYRMKYRKWRKGGKKGYWWTKKRKYAKYKTTYRMRYTGTCYYYCMC
              * **** *  ** **********. ** *** *****.**  . **       ***.

49          GRRKMYCYTRRRRKKTKYRYKYYRYYYYYWKAWRRRRRCKYMYRKGARKRTACTYYRRYY
51          TGRGAYYCYARRRKKYKYRYKTYRYYYTTWKRWGGGGRTGYMYRTARRKRATTCTYRRYY
              *  *    ***** ***** *****  ** *    *  ****.. ***::   *****

49          KKWKSKGKTKWYSRMRRWKKKCKKATGYYCCRYRKYAKKYYRYYRMKMACCYYRGCCKKK
51          KKWKSGTGYKWYSRMRRWGKKYKKRKRCYTYAYRKYRKKYYRYYRMKMTAMYYRRYMKKK
            *****    ********* ** **  .  *   **** ***********:. ***   ***

49          RWKMKKGGTAYKRMYYYWYKKYKRMYRMTATKWKARKYKKRKGMWRCWYYKYTKYMWYSY
51          RWKMKKKAARYKRMYYYWYKGCGRMYRMAGGKWKMRKYGKRKKMWRYWCYKYCKYMWYSY
            ****** .: **********   *****:. *** *** *** *** * *** *******

49          GTKYRMRYRMTWYRYYRRKRKKATCYKAKRKYYWYKKRGATMKRYGGGYYKYCKCKKKRY
51          RCKYRCRYRMWWYATTRRKRTGTATYKCKRKYYTCKKRAGCAKRYATRYCGYMGYKKKRY
              *** **** **    ****. :: **.*****  ***..  ***.  *  *   *****

49          AGCRRRYMMKYRWYRRYWKKAAAYCRAYKKTYWMSYGGTKRWRTAKS
51          GTTRRRYMMKYRAYRRCWKKTWTCYGCTKGWYWMSTSKWKGAGKGTS
            .  ********* *** ***: :   . *  ****  .  *   ...*
```

The pairwise alignments of two accessions 50 and 51

```
50              SRRRRRYYMYYRTTWMKWKYRYKRWGYMRKKWYMRYYKYKSSYRYKRKRYYWKWYMYYMM
51              SRRRRRYYMYYRGGWMKAKYRYKGWKYMRKKWYMRYCGTKSSYRYKRKRCYTKWYMYYMM
                ************  *** ***** * **********   ********** * ********

50              YRTTRRCGGYTYYYRRYYRKRRWWWRKAAWRYRMYKYWARCRCKYSYYYKRRRWWCAYKR
51              YGCARRTATYGYYYRRYYRKRRWTWRKGMWRCRMYKYWRRTAYKYSYYYKRRRWWYCCKR
                *  :** . * ************ ***. ** ****** *   ************ . **

50              RYWYWKTWRWRCAYMGMMYWWRWKWWCMAYYAWYKYRWRRRCKRKRWRTYYYYKSKYAYK
51              RYWYWKAWRWRAGYMRMMTATRWKWWYMTTCRWYKYRWRRRSKRGGWGCTTCTKSKYTCK
                ******:****..** **   ***** *:   *********.**  *      ****: *

50              CKYRYWCCAWRYRYWYMRCCGCTCKWMKRRRKMYSRARTYYRKMYGRRWYMWRYYYWRYR
51              YKTRYWMYWWRYRYWYMRAMKYWYKWMGARAKAYSRGAAYYRKMYRRRWYMWRYYYWRYR
                 * ***   *********.     ***  * * ***. :****** **************

50              KKKYCYYYYWRYYWKRKMKYTCWKGMWKYRYGYRYMMGMKTTCRKWYRYMMRCTARKRYY
51              KKKCYYYYYWRYYWKRTMKYWAWKRAWKYRYTYRYMMKMGWYYRGWYRYMCGYCMRKRYY
                ***  ***********.*** .**  ***** ***** *    * *****     *****

50              KWTTTYYMKKRYRSKTYRMWKRRYYKYKYRKYKKYRYSKKYKMRYYKACYRCAKWKMKYR
51              KWWWKYYMKKRYRSKKYRMWKRRYYKYKYRKYKKYRYSKKCKMRYYKGMYRYWKWGMKYR
                **  .**********.************************ ******. **  ** ****

50              KYSWGYRRRKWRTTTKKGRRYKRYWAKRYRRYRMRRYRRKMWRRYKMKKMYAKRKTTGYM
51              KYSWTYRRRKWRCWGKKKGRCKRYWTKRYRRYRCGATGAKMWRRYKMKKMCMKGKACKYA
                **** *******   **  * ****:*******      ***********  * *:  *

50              WRRKYSYMKKMTAYRMRRRYRRTRRKMYMYYMRTTACKRYKYKAATTKYSSYKKGTTKMY
51              WRRTYSYMKKMGRYRMRRRYRGCRAKMYMYYMRWYRYKGTKYKCGGYKYSSTKKRYYKMY
                ***.*******  ********  * ********    *  ***..  **** **   ***

50              CKYGCYYWGKYRRYTYGCARGTYRKRTSYTYYKCACCTCCTACKMKYMRYSWWKRMKWKY
51              TGCATTYACKYRRYYYTTCRTWYRKGCCCKYYKYGTYYYTACTKMGYMRYSWWKRMKWKC
                   .  *  ***** *  .*  ***  . .*** .     :. ** *************

50              GRRKKRKKKTCAYRCAAYRYYRKAYMSSGTMGRGGGYAYKTYYRRKRKYKKKMRYYYKKK
51              SGAKKRKKKYYMYRYGTTRYYRKRYMSSAWCARRAAYWTTGTYRRKRKYKKKCGYYYKKT
                .  ******   ** .: ***** ****.  .* ..*  .  **********  *****.

50              KCYCKYGYKKKGKKKYWKMYRYWYYWTTAYRYKRRYYRKRYKKYRARKYWKKRCTYTWGK
51              KMCMKYAYKKKRGKKYWKMYRYWYYTWWGCGYKRRYYRKRYKKCARRKYAKKRTCTCAKT
                *   **.****  ************   .  ************   *** ***      .

50              KKWRRRYMRAYKYYRKKAWGCTAGKCRYRYKKAAYYTRGYGCCTWCCGTGYCTYKKWRKY
51              GKWRRRYCRMCGYYRKKTTATGMRKTACAYKKRRTYYRAYATAAAMTCCRTYCCKKWGKC
                 ****** *   *****: .    *    ***   * *.*. .:          *** *

50              YKACCCTYCTYWTTYTTCKMYKKRKRRMKYYKRTMYMKKYWRKKRRWKRRKYRMRKYGAK
```

```
51      CTMMYYYCMGYWCCCCAYKMYKKRKRRMKYYKGYMYCKKYWRKKRAAKRRKYRMRGYKCT
         .        **    : **************  ** ********  ******** * ..

50      YYAGKKWKKKWYMRKSKCKGSMACKKMYKAYYWKMWRTATCCCKYRYSKKWYGYTMYSWY
51      TYMRKKWKGKWYMRKSKYKKSMTTKTMYKRYYWKMWRKGWYYMKYRYSKKWYAYAMYSWY
         *  **** ******** * **: *.*** *******..    *********.*:*****

50      YKKKYKKYTRKKMKRRRWKATRAKMRRSRTGKWTRYYKYRKKTYRRKKKWYSMMWKTKCK
51      YKKKYKKYWGKKMKRRRWKCGRGGMRRSRWAKWKRYYKYRKKYTRRKKGWYSMMWKCKMK
        ********  *********. *. ***** .**.********  **** ******* * *

50      KWRRYRKKYRRWRKSRTCMASRYYTWAKRSMCYMYRGKRTRKRKWKKKRMYYYRAKKKKM
51      KWRGYRKKYRRWRKSRAYCGSRYYKWMKRSMYYMYRKKRARGRKWKKKRMYYYRMKKKKM
        *** ************:  .****.* **** **** **:* ************ *****

50      KRYKKWYYYKKKKMMRGTKMKRWMAWGKGATATCRRKYSKKYRYARRCTGGYWMKKYRRK
51      KRYKGWYYYKKKKMARRKKMKRWCGTRKKGCGGSRRKTGKKYRYGGGAGRRYWMKKYRRG
        **** ********* * .***** .  * . . .*** .*****.  .   ********

50      RYKKYKTCYRAKKCGKKRWWKKKMYRKKRYYKYKKKKWRYKKKKKAYMYRRYRMKKYCAG
51      RYKKYKATCRMKKMRKKRWWKGKMYRKKRYYKYKKKKWRYKKKKKTYMYRRYRMKKYYWA
        ******:  * **  ****** ***********************:***********  .

50      KRMKYKMMRRRYYYKMYKRRAGYRKWMCARMYRYKRCTKMRYKKKWYKMAKKSRKYRWRM
51      KRMKYKAMRRRYYYKMYKRRMKYRKWMATRMYRYGGTGKMRYKKKWYKMRGKSRKYRWRM
        ****** *************  *****.:*****    ***********  *********

50      RKWWGWMYMMKYRGACYYWAMKWYWGYKRYRKKKTRKGYRMRRMCACSYKWRWKYKKYRR
51      RKWWKWMYMMKYGACACTWRMKWYWAYGRYRKKKCGKAYRMRRMYMYSYKWRWKYKKYRR
        **** ******* ...  * *****.* ******  *.******   *************

50      KCAGMMYYRWRWKKKKYKRGGARYKAGCCRYMKYYRCKTWRKYKRMRWKYKKYYCYYWTK
51      KYWKMMYYRWRWKKKKYKGKATGCKWKTMGYMKYYGYKKWRKYKRMRWKYKKYTTTYWKK
        *   **************  .:  *     *****  *.**************   **.*

50      YKSKTAWRYMYTKMRYYRWKYKRKGTTGRMSTKKSTYMWKGKKKRMKKKKKMKMWYKWSY
51      YTSKWMAACCCYKMRYYRWKYKRKACAARMSYKKSWYCAKRTKKGMKKKKKMKCWCTWSY
        *.**        ************. :.*** *** *  * .** ******** * .***

50      KCATCYWKRGYTARYYYYYRWKYWKMKRYYRKYWKYSYTRCRGKAKKKRKRKRGYCCKKK
51      KYWWMYWKRKTYGRCYCYYRWKYWKMKRYYRKYWKYCYKRYRTKWKKKRKRKRTYSMKGT
        *    ****   .* * *******************.*.* * * ******** *. * .

50      MMWKGWYCTYTCGRRYRRGCAKRKCCAYCCTTKKGRKKTYYRYKKRGKYRKWKSMYYMYY
51      MCWKTWCTCYWMRRRCGRSACKRKTTRCGTGCTKTRKKWYYRYKKARKYRKWKSMYYMYY
        * ** *   *   **  *...***        .* *** ******  *************

50      YYGYKSWAAYYRSRRRWKSYKKRKMKMSYWYKKYTACKMMKRYKARYTYYKKKMYMMKKM
51      YYKYKSWTTYCRSRRRWKSYKKRGMKMSYWYKKYCCMKMMTRYKGAYGYYKKKMYMMKKM
        ** ****::* ************ ********** . ***.***. * ************

50      RMKCKKKWKAYMMKYAYKWMSYYMRRMYRKYRKKYKWRYYRMKKMYWRYRWSRGAYYMRK
51      RMGMKKKWKTYMMKYGYKACGCYARAMYRKTRKKYGWRYYRMKKMYWRCATSRKMYYMRK
```

```
                **  *****:*****.**   . * * **** **** ************  **   *****

50              MYRMWYYRYWYYRMTACATRKMMYKWMMAYKKYKRYYRWYKKRYYMCYYMYAARMKRMCR
51              AYRMWYYRYWYYRMKCYTGRKMMYKAMMRYKKYKGYYRWYKKRYYCYYYMYGCRMKGCYR
                 *************.. : ****** ** ***** **********  ****..***   *

50              RRWYRYKSYWKRMYRMWKWKSGGAYCKKRYYRCTRTMWKKRRWKRYYRKYYYKRRYSCTG
51              RRAYRYKCTWKRMYRMWKWKSRRMCYKKRYYRGYACAWKKRRWKRYYRKYYYKRRYSMYR
                ** ****. ************     ******     *******************

50              CCRYMMKCKKKYRYMKKYKYGMMKTCAWKKMKYYYKRMYRYCKKKKKWWWSYKRRKRYSK
51              MMRYMMKMKKKTAYMKKYKYKMMKKYMWKKMKCYTKRMYGCYKKKKKWWWSYKRRKRYSK
                  ***** ***  ******* ***.  ***** * ****   ******************

50              RYMYAKYWYMMWATYKWKKKCGRYWMYTWCKTTGMGKWMMYYRWRKKYMKKAKYWTGKWK
51              RYMCRKYWYMMWGCYKWKTKTTGTTCTAAGKAAKARKWMMYYRWRKKYMKKRKYWWRKWK
                ***  *******. ****.*       :  *::   *************** ***  ***

50              RYMRWKRRKWKRYKRYRKYYKRRKCYTAMAKRWKWRYSSARMMYYSKMWSYKYRWYRGRS
51              RYMRWKRRKWKRYKACAGYTKRRKSCWWCTKRWKWRYSGCAMMYYGKMWSYKYRWYRKAC
                **************    * ****.    :********.. ****.***********  .

50              YYYRKYAKKKTKWYYKKWKTWRWRRARWYKAKRWKTCRWGTSMCYRSYAKKGYRKYYYYT
51              YYYRKYGKTKKTWYYKKWKWWRWRGRAWYKWKRWKATRWAYSMGYRSYMKKKYGKYYCYK
                ******.*.*..******* ****   *** ****: **. ** **** ** * *** *.

50              CKKKYKKWMWWMRYWRKMRYYGRRMWWMWGKGRYYKWYTKKYKYYYKKTRKGAARGKYKY
51              AKTKYKKWMWWMATWRKMRYYKRRMWWMWKKKRYYKWYKKTYKYYYKKKRKTMRRRKYKY
                .*.*********  ******* ******* * ******.*.*******.**   * ****

50              KRWWWYRYMWRRKWTKTMRKGTRSTYKKYYWYRRKKKKWCAYYGKAYKKTAYYKYYMCAY
51              KRAWWYRYMWRRKWKKWMRKRKRSCYKKCYWCRRKKKKWYCYTAGTYKKYRYYKYYMACY
                ** ***********.* *** .** *** ** ******* .* . :***  ******..*

50              CCRRYYWYYWRSKKRMKTMRCRKCKCTTMRRTWAWMWMTKYMWRMYRWRYMYMMSMMWRK
51              TGRRYYWTCTASKKRMKKMRGRKYKSKYMRRGTRTMWMWKCATRMYGTRYMYMMSMAWRK
                  *****    ******.** ** *.. ***    *** *   ***  ******** ***

50              MKRYKGMKKRGYYWTAGYGYRKRWKRKYYRKRKKWYMKMKMYRSYKRRRMKKWWMKRYGA
51              MKAYKKMKKRTYYWCTTYKYRKRWGRKYYRKRKKWYMKMKMYRSYKRRRMKKWWMKRYRR
                ** ** **** *** : * ***** *********************************

50              CMGKAGRCYKGYRRYWMKKYWWCCGAAKYRKRSRSYMKYRKKRKRMRKGTKMKKYKRWKY
51              SMRKRKRMYKRCRRYWMKKYWWYTARGKYRKRSRSYMKYRKKRKRMRKACKMKKYKRWKC
                .* *  * **  **********  . .*********************. *********

50              KKRYYYWMWYTYKWWKYMMCRYKYTTKCGTATCYRGTRYSYSRRCMGKMKWMMCAGMKGK
51              GKACTYWMWYCYKWWKYMMSRYKYAKKYRGTYTYRAWGYSYSRRYMRKMKWMMMMTMKSK
                 *   ***** ********.****:.*   :  **.  ****** * ******   **.*

50              YRYYCGMMSRKRKKWKRKKRTGMYYYKWRKYKYWYRMKWMKYKWMMRRKAAKKSRWKWRR
51              YRYYMRMMSRKRKKWKRKKRWRCYYYKTGTYKYWYRMKWMKYKWMMRRKGGKKSRTKWRR
                ****  **************   ****  .*******************..**** ****
```

```
50                    KKYKARAGWRRMTCATKKWKKWWYSRYGCKKYMYGGTWKWTWYYRYRKKYKGGWTKKMKK
51                    KKYKGRTRAAAAYYMCKKTKKWWYSRCRAKTCMYTSYATTWTYYRYRKKYKAAAAKKMKK
                      ****.*:         ** *******  .*. ** .  .   *********.. :*****

50                    AYKYKATRTMWKRWMRWWKRMCMWKKACCKTKCWKGGTYRTGCCTRYYWCKTKKYYRRMM
51                    RYGYKRWRKMWKRWMRAAKRMYMWKKWMYKAGGWGAAATGATTYWRYYWYKKKKYYRRMM
                       * **  *.*******  *** ****   *:  * ..:  :    **** *.********

50                    ATGAAGKRYMYYYYMKRRYYRAMTAARMRMKMYAGTAYRRKKYRYYYMKRRRKRYTTCMC
51                    RCSRGRKRYMYYYYMKRRYYRGCGGGAMRMKMYRRYRYRRKGYRYYYMKRRRGRYKYTAY
                        . . ***************.  .. ******    **** ********** **.

50                    GRRKKYRKYYWCGKRYGRRKYAWGMRYRRAATTWYRTYSMWR
51                    ARRKKCRKYYWYKKRYKGGKYTWRMRYRRCGGWWYRKTGCTA
                      .**** *****  ***   **:* *****..  ***. .
```
